# Hybrid Models and Biological Model Reduction with PyDSTool

**DOI:** 10.1371/journal.pcbi.1002628

**Published:** 2012-08-09

**Authors:** Robert Clewley

**Affiliations:** Neuroscience Institute, Georgia State University, Atlanta, Georgia, United States of America; University of California, San Diego, United States of America

## Abstract

The PyDSTool software environment is designed to develop, simulate, and analyze dynamical systems models, particularly for biological applications. Unlike the engineering application focus and graphical specification environments of most general purpose simulation tools, PyDSTool provides a programmatic environment well suited to exploratory data- and hypothesis-driven biological modeling problems. In this work, we show how the environment facilitates the application of hybrid dynamical modeling to the reverse engineering of complex biophysical dynamics; in this case, of an excitable membrane. The example demonstrates how the software provides novel tools that support the inference and validation of mechanistic hypotheses and the inclusion of data constraints in both quantitative and qualitative ways. The biophysical application is broadly relevant to models in the biosciences. The open source and platform-independent PyDSTool package is freely available under the BSD license from http://sourceforge.net/projects/pydstool/. The hosting service provides links to documentation and online forums for user support.

This is a *PLoS Computational Biology* Software Article.

## Introduction

There are now many simulation and visualization software packages available for individual application domains across the biosciences, and several general purpose packages for analyzing dynamical systems. However, less attention has been paid to the tools needed to develop models for complex phenomena through inference and reverse engineering of natural systems [1]–[3]. The unique scientific focus of the PyDSTool software package [4] (current version provided in Supplemental Text S4) is providing integrated simulation, informatic, and diagnostic tools to support a forward-looking modeling methodology for the biosciences. This fills a niche in the biosciences where mathematical tools such as dimension reduction, qualitative dynamical systems theory and bifurcation analysis can be better integrated into modeling workflows. To enhance this support, PyDSTool is open source, and is extensible and inter-operable with existing application-specific tools rather than competing with them. As such, PyDSTool fills a niche that is between large-scale simulation tools, capable of efficiently handling thousands of variables (e.g., Neuron [5], NEST [6], VCell [7] or Bio-SPICE [8]), graphically interactive environments that are better suited for relatively small numbers of variables (e.g., XPPAUT [9], DsTool [10] and CONTENT [11]), and lightweight scripting in Matlab [12] or SciPy [13]. This niche is detailed below by considering some unmet challenges to model development.

Model development in the biosciences requires tools such as simulation, visualization, data analysis, diagnostics and validation, sensitivity analysis, and parameter optimization [14]. There is no rigorous methodology for combining these tools effectively, making development an esoteric and often *ad hoc* process that blends intuition and exploration [15]. In addressing this shortcoming, systematic and computer-assisted approaches to model development have received attention in the software engineering and business management literature [16], [17], but they typically focus on graphically-designed workflows for integrating large quantities of data analysis with large scale simulation, and on management of sharing, collaboration, and provenance [18]–[20]. Previous work has suggested that a computer-assisted approach to identifying models using qualitative representations can bridge the scale from microscopic to macroscopic models and can guide users to develop heterogeneous and multi-level representations that assist in comprehending complex mechanisms [21]–[31]. However, an integrated and general-purpose software environment has not yet emerged in response to this need. In response, PyDSTool has been developed as a platform for prototyping model development principles gleaned from other fields.

At a more technical level, PyDSTool provides a range of general purpose simulation and analysis tools with similar functionality to existing packages. A detailed feature comparison is provided on the website [32] and is summarized here. A major difference from other packages is the programmatic and interactive aspects of the PyDSTool library within the Python environment. For instance, PyDSTool provides high-level compositional model-building tools involving symbolic expressions and modular component templates. This helps users construct large models efficiently and in a mathematically natural fashion compared to graphical approaches or *de novo* coding directly in C, Fortran, Java, Python, etc. The rich representation of model structure also facilitates the sophisticated manipulation of models both interactively and algorithmically. The symbolic tools provided allow Jacobian functions to be defined automatically for smooth dynamical systems, making their analysis more efficient. While computer algebra systems such as Maple [33] and Mathematica [34] provide more advanced symbolic tools, they do not provide fast numerical integration and bifurcation tools, and they are not intended for intensive numerical exploration of high-dimensional, nonlinear dynamical systems.

PyDSTool exhibits superior performance in numerical integration over other high-level environments because it automatically generates low-level C code from user model descriptions. The low-level code is linked dynamically with C or Fortran solvers and reloaded as a dynamic linked library for transparent user access within Python. Comparable high-level programmatic environments such as Matlab or Python-based packages such as SciPy, Brian [35], or PySCeS [36], rely on the less efficient high-level implementation of model codes, the results of which are accessed via an expensive ‘call-back’ interface from low-level integrators. In addition, Matlab is neither free nor open source, and does not provide convenient model description and analysis tools for working with dynamical systems models.

This article describes an example of how the PyDSTool environment supports flexible and extensible workflows to be built over a class hierarchy designed for development and analysis of dynamical models in scientific applications, as well as for inter-operability between packages and algorithms. Example workflows suited to this environment include: (1) model creation specified by code that later adaptively modifies the model structure or parameters (e.g., when interfaced with optimization algorithms); (2) adaptive batches of simulations and analyses that post-process simulation results to determine which simulations to perform next; and (3) exploration of model properties and live prototyping of model development code. Further examples are discussed throughout the text.

### Using hybrid systems for reduction

To illustrate some of the unique benefits of PyDSTool to the modeler, we will discuss an example that combines elements of the workflows (1) and (3) above using reduction techniques and hybrid dynamical systems (see [37] and references therein). Hybrid dynamical systems are defined later, but loosely speaking they are made up of smooth vector fields (such as those defined by differential equations) that are punctuated by discrete changes. This makes them especially useful for modeling systems that display modularity in functional state or inherent structure. We will discuss a measurement of modularity in Results.

In contrast to dimension reduction methods that aim to increase simulation efficiency (e.g., see [38]), reduction to hybrid system models has been demonstrated as a tool for *inferring* the key causal mechanisms underlying a high-dimensional or otherwise complex phenomenon, and for *validating* the resulting hypotheses with a dynamical system of lower dimension or greater simplicity (e.g., [39]–[42]). In lieu of a more developed qualitative theory of nonlinear dynamics for hybrid systems, explicitly simulated representations of the reduced dynamics are needed to ensure that the hypotheses are both logically self-consistent and consistent with experimental data. (E.g., see [43] for an example of exploring this issue in biomechanical modeling.) In addition, algorithms have recently been developed that assist with the inference of mechanistic relationships in ordinary differential equation (ODE) models, and also with the systematic and semi-automated construction of the resulting reduced descriptions in terms of hybrid systems [44]–[46].

A simple example of reducing an ODE in this way is the replacement of a smooth sigmoidal function having a steep slope with a piecewise-constant or piecewise-linear step function in contexts that are not sensitive to the details of the smooth slope (see [47]). This reduction can be understood in terms of functional modularity because, in this situation, the strong nonlinearity of the function ensures effective decoupling between the input and output when the input is sub-threshold. The validation of the reduction in an explicit context tests the hypothesis that the details of the function's transition were not mechanistically relevant. The integrate-and-fire neuron described in Supplementary Text S1 uses a similar replacement of smooth spiking dynamics with a discrete, instantaneous reset.

Multiple time scale systems such as the Van der Pol oscillator or chemical reaction systems with explicit small parameters are classic examples of systems that can be reduced to hybrid systems [48]–[50]. The models are studied as singularly perturbed systems, from which a quasi-steady state approximation and similar techniques obtain a ‘fast-slow’ reduction. This reduction is generally in the form of a set of differential-algebraic equations (DAEs) with domain consistency conditions, and can be seen as a piecewise-local dimension reduction of the model. Thus, it can be simulated and numerically analyzed using a hybrid model formalism [44].

We demonstrate hybrid model reduction in a scenario involving the space-clamped Hodgkin-Huxley (HH) formalism for neural action potentials (AP) [51]. This is a common biophysical model based on the first-order kinetics of ion transport across a cell membrane, and reflects an equation structure inherently similar to many models in systems biology (two recent examples that use PyDSTool can be found in [52], [53]). Although the HH model has been analyzed extensively by mathematical and numerical means, PyDSTool provides a novel opportunity to algorithmically derive, specify, analyze, and validate a reduced and explicit description of an AP, from which we claim that superior insight into its biophysical mechanism is possible.

## Design and Implementation

The PyDSTool package is a library-based environment written primarily in Python, utilizing the numpy [54], SciPy [13], and matplotlib [55] packages. A few optional dependencies are a C and Fortran compiler and the SWIG interfacing software (http://swig.org), which are only necessary to run simulations at their fastest or to run the bundled AUTO continuation software [56]. The avoidance of non-Python external dependencies simplifies the installation process on any operating system, which is described in full via the ‘Getting Started’ link from http://pydstool.sourceforge.net.

### Core classes

PyDSTool is unique in providing a variety of high-level data types (‘classes’) and library functions that closely mimic mathematical counterparts in dynamical systems theory and provide intuitive functionality. For example, domains and numeric intervals are represented by the Interval class, for which membership, intersection, endpoint testing, etc., are simple operations. Similarly, numerical arrays are extended to become Pointsets, incorporating several features: named fields instead of indices for accessing variables, an associated independent variable that may parameterize the data, and metadata labels that can be indexed and cross-referenced. Pointsets are further abstracted to Trajectories (parameterized, smooth curves), which further add a transparent layer of domain checking and interpolation that allows numerically computed data to be treated as continuous, when appropriate. Among others, such classes provide intuitive abstractions that allow users to more efficiently express their mathematical ideas in new algorithms, or to naturally specify complex meta-model constraints.

At the lowest level, PyDSTool supports simulations of ordinary differential equations (ODEs), differential-algebraic equations (DAEs), and discrete mappings [57]. Few comparable dynamical systems packages support DAEs, which are useful in hybrid modeling, and rarely support hybrid dynamics beyond simple case-based ‘switch’ or ‘if’ statements or Heaviside functions. PyDSTool users can specify dynamics using evolution equations or explicit functions of time or state. The range of possible formalisms for specifying dynamics is supported by the Generator abstract class, which creates Trajectory objects on demand. There are several ODE solver implementations supported: the adaptive time step solvers Dopri and Radau (an implicit solver that is well-suited to stiff systems and also supports DAEs) [58], a 4th-order Runge-Kutta fixed time-step method, and a wrapping of VODE (via SciPy) [59]. All solvers support *arbitrary-precision* event detection with a simple Event class, which is crucial for defining hybrid systems and is missing from many application-specific simulators. PyDSTool is modular and can be extended to support other solvers.

Bundled toolboxes provide special functionality such as phase plane analysis, model reduction, optimization, data analysis, and templates and interfaces for application-specific modeling and third-party software. For instance, users who install the PySCeS [36] or SloppyCell [60] systems biology packages have the option to create models by exporting from those packages (e.g., based on SBML definitions [61]). Equally, with the NineML Python API installed [62], [63], many forms of neural models can be imported directly. Alternatively, models can be prepared directly in PyDSTool using modular constructors and symbolic expressions, or by writing raw text definitions. An export option to the ADOL-C periodic solver in Matlab is also provided [64].

### Hybrid model implementation

We take a practical approach to implementing hybrid systems (sometimes known as composite models in other fields) in PyDSTool that is most applicable to biophysical models, where smooth dynamics are primary and are punctuated by finite numbers of discrete events. This differs from the majority of existing simulation platforms, which typically focus on physical models for engineering applications with many parallel discrete event processes mixed with smooth dynamics. Hybrid models in PyDSTool can be built from sub-models that mix discrete mappings, ODEs, DAEs, preset trajectories, or any other embedded code that can produce a Trajectory object.

There are many formalisms for hybrid systems, but the 6-tuple of Simić et al. is adequate for our purposes [65]: 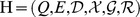. We do not take a formal approach here, and it is sufficient to describe these elements informally and direct the reader to the reference for details. 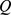 is a finite set of discrete states of the system, which we will refer to as regimes. The regime transition graph is given by nodes from 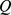 and edges from 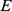. In each regime, a sub-model is defined from a corresponding 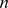-dimensional vector field from the set 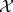 over a domain from the set 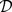. Transition events for the edges in 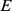 are indicated by ‘guards’ from the set 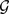, which are 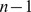 dimensional sub-manifolds in each domain. We will define these guards by zero-crossing functions on those domains. Finally, there is an optional resetting map associated with each edge, taken from the set 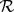, which discretely changes the state variables on a regime transition. Consistent with some formalisms, we allow a subset (often just one) of the 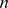 state variables (known as ‘indicator’ variables) to be discrete and therefore constant during each regime.

PyDSTool uses three essential code elements to define a hybrid model: (1) a hierarchy of component sub-models, (2) a mixture of zero-crossing events and global self-consistency conditions, and (3) transition rules between the sub-models that are applied on occurrence of an associated event or condition failure. During simulation of a sub-model, a terminal event may occur that stops the trajectory generation (see the Tutorial in Supplemental Text S1). In such a scenario, the transition rules for the stopped sub-model are applied to the final state to choose the next sub-model. The final state may also be mapped to a new value before becoming the initial condition for the next sub-model.

A user may define a hybrid model with only one sub-model, such that a terminal event or maximum elapsed time defined for that sub-model's regime will be associated with a transition back to the same sub-model, typically after a discrete change to the state is applied. An example of this is using a simple threshold-crossing event to signal an action potential (AP) in an integrate-and-fire neuron model [66], after which a discrete change is applied to reset the membrane potential. (This model's implementation is described further in Supplemental Texts S1 and S2.)

**Figure 1:**
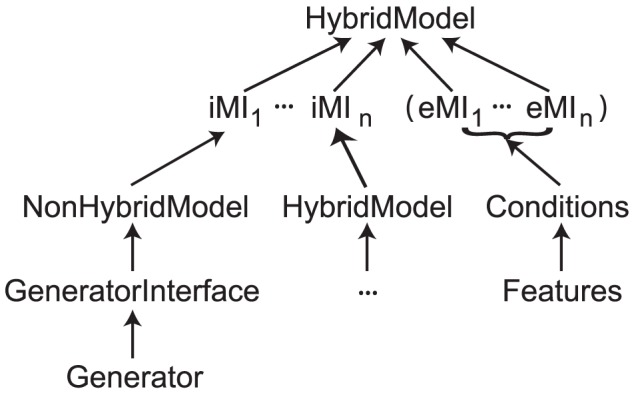
Class containment diagram for a hybrid model in PyDSTool. 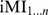
 represent ‘internal’ model interface objects that wrap 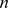 sub-models (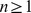). If global consistency conditions are applied to these sub-models, then 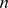 ‘external’ model interface objects, 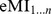, may also be provided. Each iMI may either contain a non-hybrid or another hybrid model object (an example is shown). Non-hybrid models combine with a GeneratorInterface to make a thin wrapper for Generator objects, ensuring API-compatibility with hybrid model objects and other MIs and thus promoting interchangeability. Conditions in each eMI specify a target combination of truth or falsity of one or more constituent features. The features measure properties of the corresponding iMI and compare them to those in some external data such as a user-imposed logical template or experimental data.

**Figure 2:**
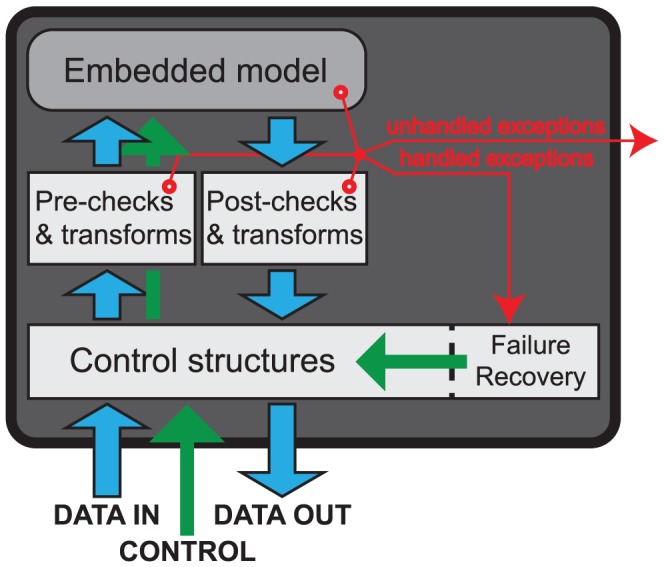
Structure of a ModelInterface (MI) class. An MI wraps a hybrid or non-hybrid model, providing users an option to add pre- and post-simulation code to validate input and output, or otherwise filter or transform the data flow. The control structure also permits failure recovery in the model to be added.

Figure 1 summarizes the implementation structure of hybrid models as a hierarchy of HybridModel objects: other HybridModels may reside at nodes while NonHybridModels (that are non-hierarchical and can only wrap Generators) reside at the leaves of the tree. In between sub-models and their parent are ModelInterface (MI) objects. MIs are generic wrappers that filter, transform or otherwise post-process the output of a sub-model (Figure 2). For the simplest hybrid models (see Text S1 for an example), MIs are neutral and no external conditions are necessary, but their high level of generality allows validation of hybrid model self-consistency conditions (see Results) and facilitates qualitative model optimization algorithms (see Future Directions). MIs do these tasks by encoding data- and hypothesis-driven constraints. Additionally, they can make optimization more robust using internal failure recovery. For example, recovery code can safeguard attempts by a standard optimization routine to test parameter values in the model that may be inconsistent with its definition. For instance, such code can catch a problem and return special values of the objective function rather than an error condition.

## Results

### Scaffolding concepts and implementation in PyDSTool

In this sub-section, we introduce a broad conceptual framework for the established analytical approach of piecewise-reduced models, and focus on the validation of the analysis by implementing the result as a hybrid systems reduction within PyDSTool.

**Figure 3:**
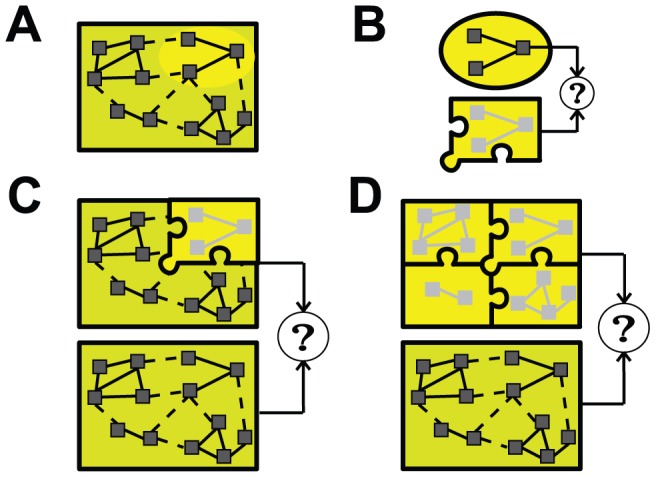
Schematic of model reduction methodology with hybrid systems using spatial decomposition. **A**) The complex model involves many inter-dependent state variables (black boxes), depicted in a connectivity graph. Analysis of a particular model behavior indicates that some inter-dependencies are effectively weak (dashed lines), suggesting a functional decomposition into sub-model components. One such sub-model is highlighted by the yellow oval. **B**) The internal dynamics of the sub-model is analyzed in the context of known input and output conditions, and a reduced model of the dynamics is derived that closely mimics the original sub-model. The puzzle piece indicates that the reduced model is derived under certain explicit constraints and assumptions that relate to the broader context of the original model. **C**) Further testing of the reduced component involves embedding it back into the full system as a surrogate for the original sub-model, possibly fine-tuning reduced model parameters to maximize the overall model output similarity under various conditions. If successful, the reduced component represents an abstracted description of the mechanism of that part of the model under these conditions. **D**) This process can be repeated for other sub-models, building a global hypothesis of the whole mechanism.

**Figure 4:**
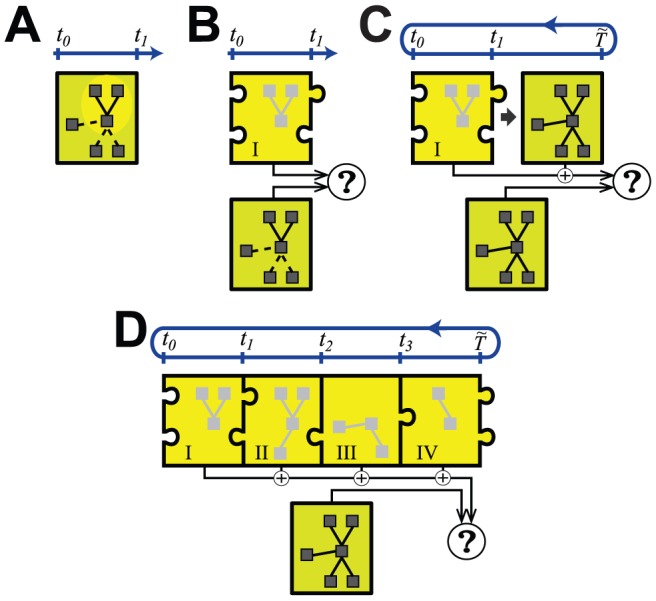
Schematic of the temporal aspects of model reduction with hybrid systems. This example assumes a model with hub-like connectivity, exhibiting multiple scale dynamics, and a periodic behavior (period 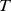), but a similar process can be described for non-periodic dynamics. State variables are shown by boxes and their inter-coupling by lines. **A**) Dominant scale analysis identifies Regime I over some time window 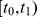 (indicated on the blue time axis) within which a subset of the variables (yellow oval) are the most influential on the system's output; the other connections are effectively weak (dashed lines). **B**) The internal dynamics of the resulting sub-model for the regime (yellow puzzle piece) is analyzed in the context of known input and output conditions alongside the full model under equivalent conditions, and the parameters and contextual conditions for the reduction are tuned to maximize the accuracy of this representation over 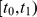. **C**) The consistency of the sub-model with the full dynamics beyond 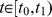 is tested for the generation of accurate cyclic behavior over a period 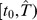 for 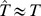, allowing for further refinement. **D**) The process in A–C is repeated for other regimes, creating four consecutive sub-models in this example. These should form a self-consistent cycle of entry and exit conditions (indicated by matching puzzle pieces) such that from the composition emerges a periodic behavior closely matching that of the full model.

Modularity of systems (more accurately, ‘near decomposability’) can be described as the occurrence of clusters of state variables that are highly inter-coupled but sparsely or weakly coupled to external variables [2], [67], [68]. Figure 3 schematically describes a spatial or *structural* form of the conceptual reduction framework used here, in which each inferred module can be analyzed, reduced and tested separately, and then further tuned in the context of the whole system. Following similar steps, Figure 4 summarizes the approach for a model that exhibits modular *functional* patterns that change over time. The modules form the basis of each sub-model of a hybrid model reduction. Reduction approaches are complicated when the effective decoupling between groups of variables is time- or state-dependent, as in the singularly perturbed systems mentioned earlier, and in the main example described below.

In its focus on supporting the exploration and understanding of emergent dynamics across scales, PyDSTool permits embedding of data playback, simplified model components, or even analytical tools as surrogates inside MI objects, thereby creating a hybrid model of heterogeneous component types. For instance, an embedded optimization tool could interface with regular model components to infer a low-dimensional characterization (e.g. a functional curve fit or a reduced-order model) of the dynamics of some part of a larger process [40], [41] (Figures 3 and 4). This exploratory approach can be described as ‘scaffolding,’ and permits users to focus on developing specific parts of a model in a contextually appropriate fashion. Some components may be temporary placeholders while others are refined, e.g. fitted to experimental data, such that the eventual goal may be to unify the model as an entirely ODE-based (non-hybrid) model. Similarly, scaffolding can allow model order reductions to be targeted to specific temporal or state sub-domains, increasing the degree of reduction possible. A more advanced example is replacing a dynamic variable with a surrogate time series that plays back experimental or simulated data (known as an ‘external input’ variable in PyDSTool) and ignoring feedback to it from other coupled variables.

The scaffolding idea has not been exploited in computational software previously, but is conceptually related to using code stubs and testing with surrogate data in software engineering, and to the mathematical ‘buffering’ principle for tackling complex models of biochemical systems [69].

### Example: The Hodgkin-Huxley action potential

We present a hybrid system implementation of the reduced Hodgkin-Huxley (HH) action potential (AP) derived from a ‘dominant scale’ analysis [70]. Previously, rigorous mathematical approaches to this system have yielded asymptotically-valid DAEs with only implicit dynamics for some slow variables, whereas we derive a fully explicit reduced-order model of the dynamics throughout an AP [46], [71]. Details of the ODEs for this system and their analysis can be found in these references, and for reasons of space we briefly summarize the mathematical setup. Coding details of this hybrid model specification can be found in Supplemental Texts S1 and S3.

There are four state variables in the HH model: 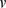 for the membrane potential and three ionic channel gating variables, two for the fast sodium (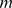, 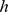) and one for delayed-rectifier potassium (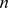). These are given by differential equations for their first order kinetics, and are only coupled with the equation for 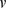 in a hub-like graph with non-symmetric coupling rules. The validity of a reduced regime determined by dominant scale analysis is determined by the truth of the defining assumptions. These include controls on the relative time scales of the variables (each available in explicit algebraic form) and the relative scales of dominance calculated as the quantities 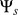 for 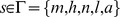, which includes the passive input terms for the leak conductance and an applied current 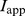 (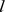 and 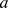 are dummy variables that are 0 or 1 depending on their inclusion in a regime). The 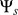 are essentially sensitivities of the quasi-static resting potential of 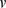 with respect to changes in each other variable, and are available in explicit algebraic form. The ‘active’ set of variables in a regime can now be defined as 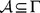 such that 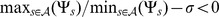 for some user-defined scale tolerance 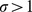. A terminal event will indicate that a variable has left 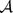 during the regime when the above inequality becomes an equality (a zero-crossing), with a similar event for variables joining 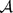. Post-processing of trajectories is required to determine exactly *which* variable left or joined 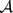 (see Supplemental Text S1).

Dominant scale analysis of a periodic orbit indicated four reduced regimes that capture the essential dynamics of qualitatively distinct parts of the AP cycle relative to 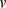 [70]. The sub-models for the regimes do not need to be decomposed further into sub-models, and so are implemented by NonHybridModels containing single Generator objects. Each sub-model contains 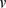 and a different set of active inputs requiring ODEs for zero or more truly dynamic state variables, resulting in a DAE. The simplified equations and their consistency conditions form the scaffolding for this sub-model. There is no coupling to 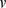 from the variables not in the active set, but we must track any changes they make because they are ‘slaved’ to 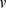. The validation method requires that we simulate the non-active ‘shadow variables’ in order to correctly determine when the sub-model's assumptions become inconsistent with the original model [44]. For this situation, PyDSTool naturally supports sub-models having differing dimension by allowing dummy variables to be included in a sub-model. Thus all sub-models must formally include all state variables in one form or other.

**Figure 5:**
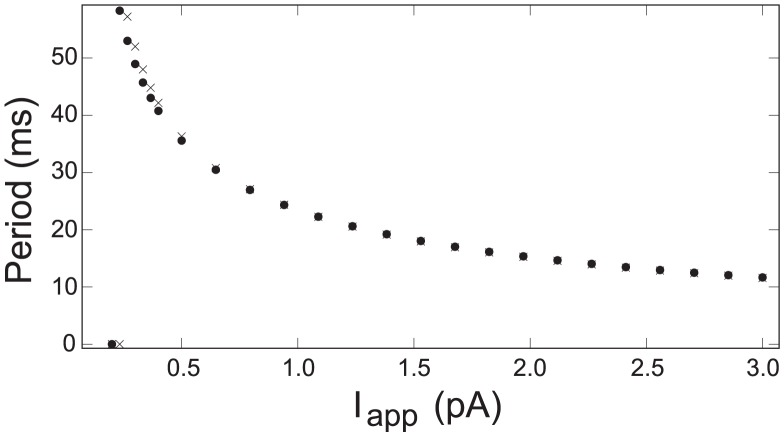
AP firing period comparison. The graph compares the period of regular AP firing in the original Hodgkin-Huxley model (‘x’ markers) and the hybrid reduction (‘o’ markers) as a function of applied current 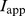. Zero period indicates no APs (steady state).

**Figure 6:**
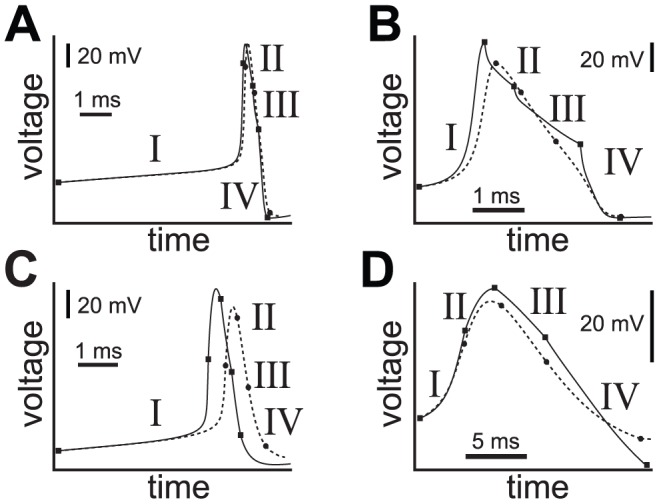
Voltage trace fits for various hybrid AP models. Voltage traces versus time summarize the qualitative fit of four smooth biophysical models of APs (solid lines) with their hybrid counterparts (dashed lines). The four sub-model regimes of the AP are indicated with Roman numerals, with onsets indicated by solid square or circular markers. **A**) The fast interneuron model studied here. **B**) Original Hodgkin-Huxley parameters. **C**) A Wang-Buzsáki form of interneuron. **D**) A heart interneuron model with a larger set of sub-threshold and AP ionic currents. *Adapted from Figure 7 of Clewley* [*46*] *, in which full model and analysis details can be found*.

Taken together as a hybrid model, the piecewise-local reductions form a formal hypothesis about the key mechanistic relationships between variables during an AP. The inferred mechanistic description is formalized as a sequence of domain and transition rules called a ‘template’ (e.g., see Table 1 of [46]). Such clear and specific insights give an advantage for hybrid modeling over other forms of model reduction. For parameters corresponding to a fast inhibitory interneuron, the trajectories computed with this hybrid model are almost indistinguishable from that of the full model over a wide range of applied currents. For instance, the periods of oscillation match closely (Figure 5) as 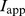 is increased, except around the onset of AP oscillations from a steady state (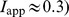. Figure 6 summarizes the similarity of APs in a more sophisticated dominant scale analysis that determined hybrid models from four HH-type neurons all matching the same template, thereby validating the proposed mechanism [46]. Greater mismatches between reduced and original model trajectories indicate weakening self-consistency conditions in the reductions and provide a diagnostic focus for refinement of the mechanisms at work.

## Availability and Future Directions

The software and its source code are publicly available, anonymously and for free, under the BSD license: http://sourceforge.net/projects/pydstool/. The download is accompanied by a test suite, documentation of the API, and a link to online documentation at http://pydstool.sourceforge.net. Sourceforge provides user forums for feedback about software use, bug reporting, etc. Interfaces to specialized modeling and simulation packages will continue to be developed in collaboration with interested users.

This work reinforces the idea that a reduction should take place in the *context* of a particular phenomenon that the model captures, and that constraints from that context can be imposed as part of a computational reduction process. The hybrid model reduction methodology has determined an *explicit* mechanistic model for the sodium and potassium dynamics of the action potential process using a sequence of low-dimensional approximations with no *a priori* assumptions or formal asymptotic limits. This decompositional approach is intended to facilitate a more sophisticated investigation of underlying mechanisms in complex dynamics than the comparatively naive approaches of brute-force parameter sweeps and large-scale simulation. It also expected to lead to more effective methods of *designing* complex dynamics in continuous dynamical systems.

The design of PyDSTool facilitates qualitative and multi-objective optimization techniques, which are increasingly recognized as important aspects of biological modeling [3], [72], [73]. Work in progress extends the use of PyDSTool classes to develop the concept of scaffolding further into model optimization and analysis applications [74].

## Supporting Information

Text S1 Tutorial for Hybrid Model Implementation in PyDSTool.
(PDF)

Text S2 Syntax-highlighted code for the demonstration script IF_squarespike_model.py.
(PDF)

Text S3 Syntax-highlighted code for the demonstration script HH_DSSRTtest.py.
(PDF)

Text S4 Complete source code for the PyDSTool package (version 0.88.120504). Includes API documentation and help files linking to web pages. This file is identical to the current public release on Sourceforge.net.
(ZIP)

## References

[1] Beard DA, Bassingthwaighte JB, Greene AS (2005) Computational modeling of physiological systems. Physiol Genomics 23: 1–3. 16179418 10.1152/physiolgenomics.00117.2005

[2] Csete ME, Doyle JC (2002) Reverse engineering of biological complexity. Science 295: 1664–1669. 11872830 10.1126/science.1069981

[3] Guckenheimer J (1998) Numerical computation in the information age. Computing Research News 10.

[4] Clewley RH, Sherwood WE, LaMar MD, Guckenheimer JM (2007) PyDSTool, a software environment for dynamical systems modeling. Available: http://pydstool.sourceforge.net. Accessed 10 June 2012.

[5] Carnevale NT, Hines ML (2006) The NEURON Book. Cambridge University Press.

[6] Neural simulation technology (NEST). Available: http://www.nest-initiative.org. Accessed 10 June 2012.

[7] Schaff J, Loew LM (1999) The virtual cell. In: Biocomputing: Proceedings of the 1999 Pacific Symposium. Altman RB, et al., editors. Singapore: World Scientific. 228–239. 10.1142/9789814447300_0023 10380200

[8] Bio-SPICE simulation program. Available: http://biospice.sourceforge.net. Accessed 10 June 2012.

[9] Ermentrout GB (2002) Simulating, Analyzing, and Animating Dynamical Systems: A Guide to XPPAUT for Researchers and Students. Philadelphia: SIAM.

[10] Back A, Guckenheimer J, Myers M, Wicklin F, Worfolk P (1992) DsTool: Computer assisted exploration of dynamical systems. Notices Amer Math Soc 39: 303–309.

[11] Kuznetsov YA (1998) CONTENT – integrated environment for analysis of dynamical systems. tutorial. Technical Report UPMA-98-224, Ecole Normale Superieure de Lyon.

[12] The MathWorks. MATLAB and Simulink for technical computing. Available: http://www.mathworks.com. Accessed 10 June 2012.

[13] Jones E, Oliphant T, Peterson P, et al. (2001) SciPy: Open source scientific tools for Python. Available: http://www.scipy.org/. Accessed 10 June 2012.

[14] Commission on Physical Sciences, Mathematics, and Applications (1991) Mathematical Foundations of High-Performance Computing and Communications. National Academy Press.

[15] National Research Council of the National Academies (2005) Mathematics and 21st Century Biology. National Academy Press.

[16] Muhanna WA, Pick RA (1994) Meta-modeling concepts and tools for model management: A systems approach. Manage Sci 40: 1093–1123.

[17] The Eclipse Foundation. Eclipse modeling framework project. Available: http://www.eclipse.org/modeling/emf/. Accessed 10 June 2012.

[18] Davidson S, Cohen-Boulakia S, Eyal A, Ludäscher B, McPhillips T, et al (2007) Provenance in scientific workow systems. IEEE Data Eng Bull 30: 44–50.

[19] Görlach K, Sonntag M, Karastoyanova D, Leymann F, Reiter M (2011) Conventional workow technology for scientific simulation. In: Guide to e-science. Yang X, Wang L, Jie W, editors. Springer-Verlag.

[20] The myGrid team. Taverna: open source and domain independent workow management system. Available: http://www.taverna.org.uk. Accessed 10 June 2012.

[21] Abelson H (1990) The bifurcation interpreter: a step towards the automatic analysis of dynamical systems. Computers Math Applic 20: 13–35.

[22] Bradley E, Easley M, Stolle R (2001) Reasoning about nonlinear system identification. Artif Intell 133: 139–188.

[23] Bratko I, Suc D (2003) Learning qualitative models. AI Mag 24: 107–119.

[24] Coiera E (1992) The qualitative representation of physical systems. Know Eng Rev 7: 55–77.

[25] Chou IC, Voit E (2009) Recent developments in parameter estimation and structure identification in biochemical and genomic systems. Math Biosci 219: 57–83. 19327372 10.1016/j.mbs.2009.03.002 PMC2693292

[26] Fishwick PA, Narayanan NH, Sticklen J, Bonarini A (1994) A multimodel approach to reasoning and simulation. IEEE Trans Sys Man Cyber 24: 1433–1449.

[27] Gomez-Cabrero D, Compte A, Tegner J (2011) Workow for generating competing hypotheses from models with parameter uncertainty. Interface Focus 1: 438–449. 22670212 10.1098/rsfs.2011.0015 PMC3262450

[28] Lee WW, Kuipers B (1993) A qualitative method to construct phase portraits. In: National Conference on Artificial Intelligence (AAAI-93). Menlo Park, Calif.: American Association for Artificial Intelligence. 614–619.

[29] Li J, Kevrekidis PG, Gear CW, Kevrekidis IG (2007) Deciding the nature of the coarse equation through microscopic simulations: The baby-bathwater scheme. SIAM Rev 49: 469–487.

[30] Talbi EG (2009) Metaheuristics: From Design to Implementation. New York: Wiley.

[31] Yip K (1987) Extracting qualitative dynamics from numerical experiments. In: National Conference on Artificial Intelligence (AAAI-87). Menlo Park, Calif.: American Association for Artificial Intelligence. 665–670.

[32] Clewley R (2010) PyDSTool Project Overview. Available: http://www.ni.gsu.edu/~rclewley/PyDSTool/ProjectOverview.html. Accessed 10 June 2012.

[33] Cornhill JM, Testud P (2001) An Introduction to Maple V. Berlin: Springer.

[34] Wolfram Research. Mathematica. Available: http://www.wolfram.com/mathematica/. Accessed 10 June 2012.

[35] Goodman DFM, Brette R (2009) The Brian simulator. Front Neurosci 3: 192–197. 20011141 10.3389/neuro.01.026.2009 PMC2751620

[36] Olivier BG, Rohwer JM, Hofmeyr JHS (2004) Modelling cellular systems with PySCeS. Bioinformatics 21: 560–561. 15454409 10.1093/bioinformatics/bti046

[37] Carloni L, DiBenedetto M, Pinto A, Sangiovanni-Vincentelli A (2004) Modeling techniques, programming languages, and design toolsets for hybrid systems. Technical report, IST-2001-38314 WPHS, Columbus Project.

[38] Rathinam M, Petzold LR (2003) A new look at proper orthogonal decomposition. SIAM J Numer Anal 41: 1893–1925.

[39] Bose A, Manor Y, Nadim F (2001) Bistable oscillations arising from synaptic depression. SIAM J Appl Math 62: 706–727.

[40] Clewley R (2011) Inferring and quantifying the role of an intrinsic current in a mechanism for a half-center bursting oscillation: A dominant scale and hybrid dynamical systems analysis. J Biol Phys 37: 285–306. 22654178 10.1007/s10867-011-9220-1 PMC3101323

[41] Ermentrout GB, Kopell N (1998) Fine structure of neural spiking and synchronization in the presence of conduction delays. Proc Nat Acad Sci U S A 95: 1259–1264. 10.1073/pnas.95.3.1259 PMC18738 9448319

[42] Spardy L, Markin SN, Shevtsova NA, Prilutsky BI, Rybak IA, et al (2011) A dynamical systems analysis of afferent control in a neuromechanical model of locomotion: I. rhythm generation. J Neural Eng 8: 065003. 22058274 10.1088/1741-2560/8/6/065003 PMC3422643

[43] Edwards D (2010) Neuromechanical simulation. Front Behav Neurosci 4: pii 40. 10.3389/fnbeh.2010.00040 PMC2914529 20700384

[44] Clewley R, Rotstein HG, Kopell N (2005) A computational tool for the reduction of nonlinear ODE systems possessing multiple scales. Multiscale Model Simul 4: 732–759.

[45] Clewley R, Soto-Treviño C, Nadim F (2009) Dominant ionic mechanisms explored in the transition between spiking and bursting using local low-dimensional reductions of a biophysically realistic model neuron. J Comput Neurosci 26: 75–90. 18594958 10.1007/s10827-008-0099-1 PMC2710314

[46] Clewley R (2010) Encoding the fine-structured mechanism of action potential dynamics with qualitative motifs. J Comput Neurosci 30: 391–408. 20717840 10.1007/s10827-010-0267-y

[47] Lincoln P, Tiwari A (2004) Symbolic systems biology: Hybrid modeling and analysis of biological networks. In: Hybrid Systems: Computation and Control HSCC. Alur R, Pappas G, editors. Springer. 660–672.

[48] Deuhard P, Heroth J (1996) Dynamic dimension reduction in ODE models. In: Scientific Computing in Chemical Engineering. Keil F, Mackens W, Voß H, Werther J, editors. Springer-Verlag. 29–43.

[49] Jones C (1994) Geometric singular perturbation theory. In: Dynamical systems. Montecatini Terme. Arnold L, editor. Dynamical systems.Montecatini Terme, Berlin: Springer-Verlag, Lecture notes in mathematics. 44–118.

[50] Maas U, Pope SB (1992) Simplifying chemical kinetics: Intrinsic low dimensional manifolds in composition space. Combust Flame 88: 239–264.

[51] Hodgkin AL, Huxley AF (1952) Currents carried by sodium and potassium ions through the membrane of the giant axon of Loligo. J Physiol 117: 500–544. 14946713 10.1113/jphysiol.1952.sp004717 PMC1392213

[52] Hong T, Xing J, Li L, Tyson JJ (2011) A mathematical model for the reciprocal differentiation of T helper 17 cells and induced regulatory T cells. PLoS Comput Biol 7: e1002122. 21829337 10.1371/journal.pcbi.1002122 PMC3145653

[53] Kidd PB, Wingreen NS (2010) Modeling the role of covalent enzyme modification in Escherichia coli nitrogen metabolism. Phys Biol 7: 16006. 10.1088/1478-3975/55/1/016006 PMC3894576 20057006

[54] Oliphant TE (2006) Guide to NumPy. Provo, UT. Available: http://www.tramy.us/. Accessed 10 June 2012.

[55] Hunter JD (2007) Matplotlib: A 2D graphics environment. Comput Sci Eng 9: 90–95.

[56] Doedel E, Keller HB, Kernevez JP (1991) Auto. Int J Bifurc Chaos 1: 493.

[57] Guckenheimer J, Holmes P (1983) Nonlinear Oscillations, Dynamical Systems, and Bifurcations of Vector Fields. Applied Mathematical Sciences. New York: Springer-Verlag.

[58] Hairer E, Nørsett SP, Wanner G (1993) Solving ordinary differential equations I: Nonstiff Problems. Berlin: Springer-Verlag.

[59] Hindmarsh AC, Serban R (2008) User documentation for CVODE v2.6.0. Technical Report UCRLSM- 208108, LLNL.

[60] Myers CR, Gutenkunst RN, Sethna JP (2007) Python unleashed on systems biology. Comput Sci Eng 9: 34–37.

[61] Hucka M, Finney A, Sauro HM, Bolouri H, Doyle JC, et al (2003) The systems biology markup language (SBML): A medium for representation and exchange of biochemical network models. Bioinformatics 9: 524–531. 10.1093/bioinformatics/btg015 12611808

[62] INCF Multiscale Modeling Task Force (2011) NineML. Available: http://software.incf.org/software/nineml. Accessed 10 June 2012.

[63] INCF Multiscale Modeling Task Force (2011) Python API for NineML. Available: http://phobos.incf.ki.se/. Accessed 10 June 2012.

[64] Guckenheimer J, Meloon B (2000) Computing periodic orbits and their bifurcations with automatic differentiation. SIAM J Sci Stat Comp 22: 951–985.

[65] Simic SN, Johansson KH, Lygeros J, Sastry S (2005) Towards a geometric theory of hybrid systems. Dynam Contin Discrete Impuls Systems, Series B 12: 649–687.

[66] Lapique L (1907) Recherches quantitatives sur l'excitation électriques des nerfs traitée comme une polarization. J Physiol Pathol Gen 9: 620–635.

[67] Levin M (1998) Combinatorial Engineering of Decomposable Systems. New York: Springer.

[68] Dill DL, Lincoln P (2003) Evolution as design engineer. In: Computational Methods in Systems Biology, First International Workshop, CMSB 2003. Priami C, editor. Springer. 202–206.

[69] Voit EO, Ferreira AEN (1998) Buffering in models of integrated biochemical systems. J Theor Biol 191: 429–438.

[70] Clewley R (2004) Dominant-scale analysis for the automatic reduction of high-dimensional ODE systems. In: ICCS 2004 Proceedings. New England Complex Systems Institute. Minai A, Bar-Yam Y, editors. Boston, MA. Available: http://necsi.org/events/iccs/openconf/author/papers/f237.pdf. Accessed 10 June 2012.

[71] Suckley R, Biktashev V (2003) The asymptotic structure of the Hodgkin-Huxley equations. Int J Bifurc Chaos 13: 3805–3826.

[72] Daniels J, Anderson EW, Nonato LG, Silva CT (2010) Interactive vector field feature identification. IEEE Trans Vis Comput Graph 16: 1560–8. 20975198 10.1109/TVCG.2010.170

[73] Druckmann S, Banitt Y, Gidon A, Schurmann F, Markram H, et al (2007) A novel multiple objective optimization framework for constraining conductance-based neuron models a novel multiple objective optimization framework for constraining conductance-based neuron models by experimental data. Front Neurosci 1: 7–18. 18982116 10.3389/neuro.01.1.1.001.2007 PMC2570085

[74] Clewley R, Dobric M (2010) A qualitative optimization technique for biophysical neuron models with many parameters. BMC Neurosci (Suppl 1) P39.

