# Supplementary material for: Hybrid Models and Biological Model Reduction with PyDSTool
Source: PLoS Comput Biol. 2012 Aug 9;8(8):e1002628. doi: 10.1371/journal.pcbi.1002628 (PMC3415397; doi:10.1371/journal.pcbi.1002628)
Supplement: Text S1 — Tutorial for Hybrid Model Implementation in PyDSTool. (PDF) [file pcbi.1002628.s001.pdf]

Tutorial for Hybrid Model Implementation in PyDSTool

1 Notes about Python code

In this tutorial, fixed-width font will indicate valid Python code, with the syntax coloring shown in Table 1.

In Python, dictionaries are hash-table-like data types that map 'keys' to values, and PyDSTool generally uses string-typed keys to set arguments that are passed to class initializers and certain class methods. The `args` class is provided in PyDSTool to simplify the syntax for arguments and similar structures that would otherwise use dictionaries. This class is akin to a simple 'struct' in C but is sub-classed from a Python dictionary, allowing it to be used conveniently in multiple ways. Thus, a declaration such as `pars = {'Iapp': 1.3, 'gl': 0.1}` can be equivalently written `pars = args(Iapp=1.3, gl=0.1)`, such that in both cases a value of a parameter can be recovered using `pars['gl']`, but in the second case is also available as `pars.gl`. We will use both formats in the code provided here for illustration.

Full PyDSTool code for the two examples discussed here are provided in the IF_squarespike_model.py and HH_DSSRTtest.py scripts in the PyDSTool/tests/ directory of the package, and provided as Supplemental Text S2 and S3 with syntax highlighting, for convenience. Further details of the specification syntax and options, as well as more introductory tutorials for using PyDSTool, are given in the documentation at `http://pydstool.sf.net`. For simplicity, we specify all model equations here directly using text 'strings', although symbolic and modular tools are available but not discussed here (see the online documentation and demonstration scripts provided with the download). The ultimate syntactic composition of sub-models into a hybrid model is the same regardless of these specification choices.

2 Introductory example with IF neuron

The leaky integrate-and-fire (IF) single compartment neuron model uses a simple linear leaky term when the membrane potential v is below a threshold [1]. This can be defined using a current balance differential equation

$$\dot{v} = f_1(v) = \left(I_{\text{app}} - g_l(v - v_l)\right)/C, \tag{1}$$

which adds an applied current parameter I_{app} (in pA) to the term defining relaxation of v towards the leak reversal potential parameter v_l, at a rate governed by the conductance parameter g_l (in nS). For simplicity, we will assume the membrane capacitance $C = 1$ pF and ignore it below. The user must take care of the units for their model quantities in PyDSTool.

Table 1: Syntax coloring for formatted Python code

`class`	Python keyword
`None`	object or function built in to Python, or imported from numpy or matplotlib libraries
`embed`	PyDSTool function
`Model`	PyDSTool class or module
`pts`	any other Python identifier
`"string"`	Python string
`# text`	Python comment

For the sake of demonstrating a non-trivial and more interesting example, we will add an explicit square pulse to represent the neuron's action potential (AP) 'spike'. Under some circumstances it can be helpful or even necessary to provide such a coarse representation of the spike. An example is the 'scaffolding' framework described in the main article, which could involve embedding such an implementation of IF in place of a more biophysically realistic neuron model in a larger model simulation that requires all components to generate explicit voltage spikes (rather than spike-time event signals). In this tutorial, we provide a spike pulse that is pre-determined in shape and duration by an explicit function. (Note that for the standard IF model without an explicit spike, there are more efficient means to simulate networks of such neurons than with this approach.)

Figure 1 illustrates the conceptual relationships between the objects that constitute the IF hybrid model in PyDSTool, and how a call into the model object to compute a trajectory is processed.

2.1 Specifications of sub-models

The following PyDSTool code snippet defines several dictionary objects that specify smooth dynamics for the linear leaky integrator, sub-threshold part of the IF neuron's behavior. The specifications include the definition of a terminal event that will be used to signal the transition to the AP pulse.

```
event_args = {'name': 'threshold',
              'eventtol': 1e-3,
              'eventdelay': 1e-4,
              'term': True}
thresh_ev = makeZeroCrossEvent('v-threshval',
                     1, event_args,
                     varnames=['v'],
                     parnames=['threshval'])
lin_args = {'pars': {'Iapp': 1.3, 'gl': 0.1, 'vl': -67, 'C': 1,
                     'threshval': -65},
            'varspecs': {'excited': "0", 'v': "(Iapp - gl*(v-vl))/C"},
            'xdomain': {'v': [-120,50], 'excited': 0.},
            'ics': {'v': -80, 'excited': 0},
            'tdomain': [0, Inf],
            'algparams': {'init_step': 0.1},
```

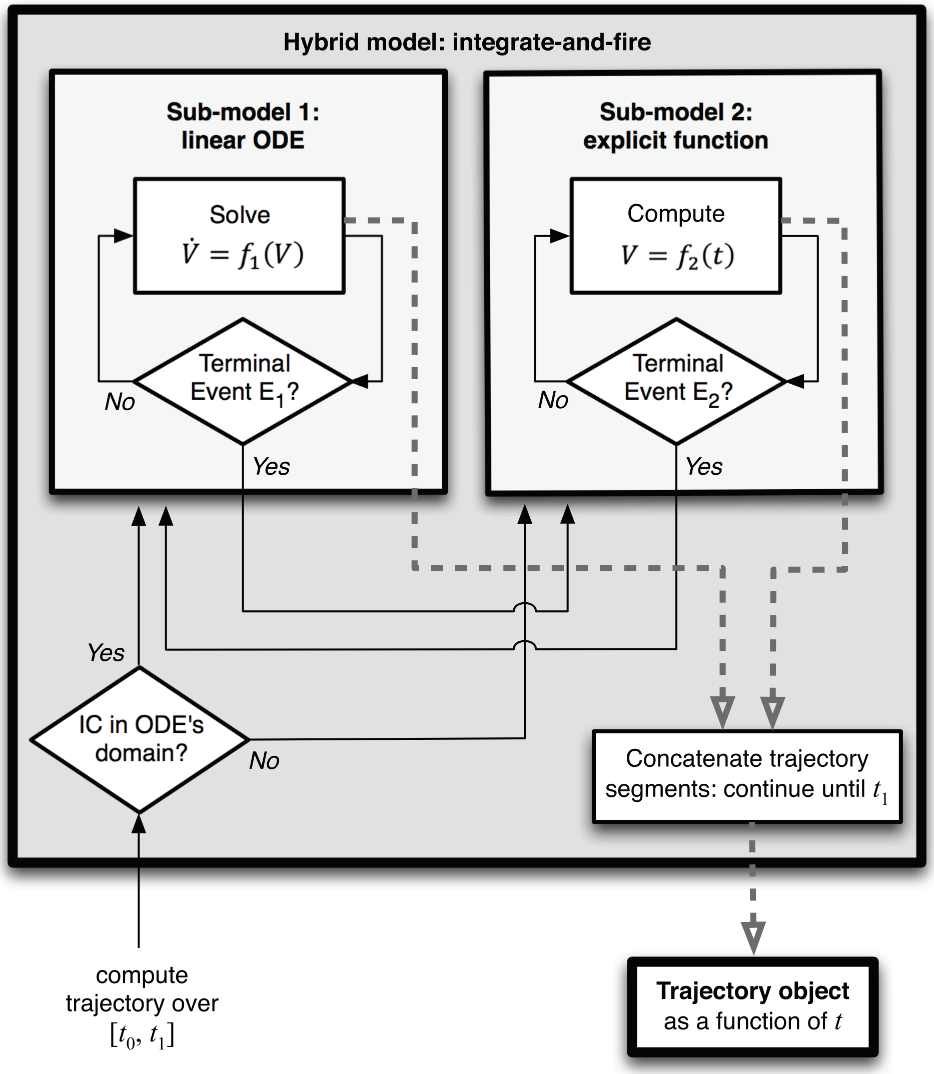

Figure 1: **Hybrid model representation of integrate-and-fire (IF) with an explicit action potential square-pulse spike.** Computing a trajectory curve over a given independent variable range $[t_0, t_1]$ and given a valid initial condition (IC). The domain of the IC is first determined, thus selecting which sub-model begins the trajectory computation. Trajectory segments are computed within a sub-model until the occurrence of a terminal event associated with that sub-model. In this IF model, sub-model 1 is the linear ODE given by Eq. (1), and its terminal event E_1 is defined by $v \geq$ threshval. Sub-model 2 is the explicit function of time that defines the square-shape pulse of the spike as well as a brief refractory period. Its terminal event E_2 is defined by $t \geq$ splen (see main text).

```
            'events': thresh_ev,
            'name': 'linear'}
linear_model = embed(Generator.Vode_ODEsystem(lin_args),
             name='linear', tdata=[0,200])
```

A terminal zero-crossing event named 'threshold' is defined, which is triggered only when v increases through the parameter 'threshval'. (See Appendix A.2 for more details about events.) The second argument to the function makeZeroCrossEvent is the direction code, 1 specifying the increasing direction, -1 for decreasing, and 0 for either. The event will be resolved to an accuracy in time given by the option 'eventtol', and will not be active for the first 'eventdelay' time units from the local initial time of this sub-model when it is initialized. The `lin_args` dictionary specifies several important items for this dynamical system. The 'varspecs' define the right-hand sides of ODEs for each variable of the system. The state indicator variable 'excited' is a dummy variable that remains constant, and is set to the value 0 by the initial conditions key 'ics'. Thus, this segment of the final hybrid system is uniquely characterized by 'excited' being zero. The state variables' domains are set by the key 'xdomain' and are used to ensure that 'excited' cannot be legally changed from zero in this system, while the voltage is allowed to span a range that is reasonable for this model. The time domain set by 'tdomain' sets the legal range of times for this sub-model. Lastly, the call to 'embed' trivially wraps the resulting Generator into a NonHybridModel object for API compatibility (see Figure 1 of main article text).

The other part of the IF neuron's dynamics is a discontinuous, instantaneous reset of the membrane potential to a preset level once it increases through the voltage threshold. The pulse shape is programmed using an explicit function type of Generator, $v = f_2(t)$. It involves an 'if' statement that sets the voltage v to 48 mV for 0.75 ms then to the reset value -97 mV for a further 0.75 ms, mimicking a refractory period. This segment of the hybrid dynamics ends when the preset duration of 1.5 ms elapses (no associated event is necessary). Thus, the use of time in this specification is relative to the beginning of its instantiation during simulation.

```
spike_args = {'tdomain': [0.0, 1.5],
              'varspecs': {'excited': "1", 'v': "if(t<splen,48,-97)"},
              'ics': {'v': 48, 'excited': 1},
              'pars': {'splen': 0.75},
              'xdomain': {'v': [-98, 51], 'excited': 1.},
              'name': 'spike'}
spike_model =  embed(Generator.ExplicitFnGen(spike_args), name='spike')
```

2.2 Composition of sub-models into a hybrid model

The next code snippet declares the internal model interfaces that wrap each sub-model, and define the transition rules between them. A temporary information object is built for each segment of the hybrid model, which associates the model interface with its exit transition rules that are given as a pair $(\langle trans\_event\_name\rangle, \langle target\_submodel\_name\rangle)$.

```
all_names = ('linear', 'spike')
linear_MI = intModelInterface(linear_model)
linear_info = makeModelInfoEntry(linear_MI, all_names,
              [('threshold', 'spike')])
```

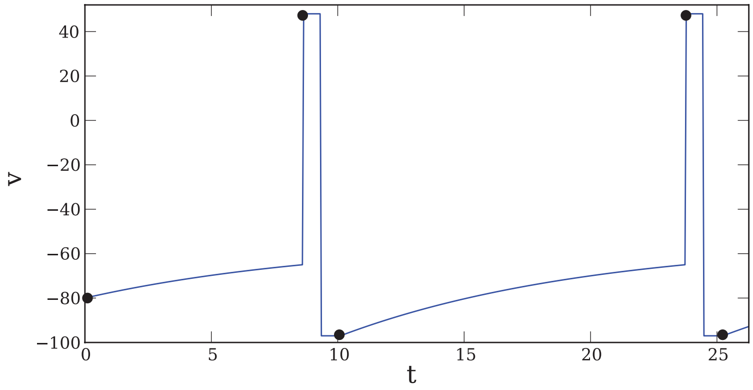

Figure 2: A portion of the voltage trace of the integrate-and-fire neuron model with square-pulse spike. Hybrid state transitions are marked with circles.

```
spike_MI = intModelInterface(spike_model)
spike_info = makeModelInfoEntry(spike_MI, all_names, [('time', 'linear')])
```

The transition rule specification 'linear_info' indicates that the linear leak sub-model can undergo a 'threshold' terminal event that should transition the hybrid model to the spike sub-model. Similarly, 'spike_info' indicates that the time expiration of the spike sub-model should transition the hybrid model back to the linear leak sub-model. Finally, the information objects are composed in a dictionary and used as an argument to the initializer of a HybridModel object:

```
modelInfoDict = makeModelInfo([linear_trans, spike_trans])
mod_args = {'name': 'IF_model', 'modelInfo': modelInfoDict}
IFmodel = Model.HybridModel(mod_args)
```

The ICs and domains for the 'excited' indicator variable in each segment's sub-model ensure that its value is discretely set when the sub-model is initialized during trajectory computation (see Section 2.4). (If other discrete changes were needed to the state variables at transition times between sub-models, the specifications can include state mappings.)

2.3 Working with hybrid trajectories

We compute a 60 ms-long trajectory named 'test' from an IC that puts the system in the sub-threshold state:

```
IFmodel.compute(trajname='test', tdata=[0, 60],
                ics={'v': -80, 'excited': 0})
```

Hybrid trajectories are stored inside the Model object they are associated with. They can be accessed much the same way as regular trajectory objects when treated as continuous curves. For instance, they can be sampled into a Pointset:

```
pts = IFmodel.sample('test', dt=0.05)
```

where the 'dt' option selects the time mesh for sampling, independently of what time mesh was selected by the ODE solver for the linear leak segments (thus, the new mesh may involve interpolation). This data was plotted using the pyplot library (http://matplotlib.sf.net) using the command `plt.plot(pts['t'], pts['v'])` in Figure 2, without the need for the user to remember array row indices associated with variable names. The trajectory can also be extracted using

```
traj = IFmodel.trajectories['test']
```

Hybrid trajectories can also be treated as discrete mappings, from one transition event to the next. To access a hybrid trajectory this way, we call it with the additional option `asmap=True`. In this call, the range of integers covers each time partition of the hybrid trajectory (noting that in Python indices begin at 0, and index ranges do not include the last value).

```
num_parts = len(traj.timePartitions)
eventvals = traj(range(0, num_parts+1), asmap=True)
```

In this case, `eventvals` is a Pointset containing time and state variables for each hybrid state transition. This data is used to provide the circles in Figure 2, using the command `plt.plot(eventvals['t'], eventvals['v'], 'ko')`. Indexing with integer 2, as in `eventvals[2]`, returns a Point that displays at the interactive prompt as

```
excited:  0.0
t:  10.1023372287
v:  -97.0
```

showing that a transition to the sub-threshold state $v = -97$ mV occurred at $t \approx 10.1$ ms. The Pointset can also be accessed with index 'slices'. Thus `eventvals[2:4]` returns a new Pointset with two consecutive entries, which displays

```
Pointset <no name> (parameterized)
Independent variable:
event:  [2 3]
Coordinates:
excited:  [ 0.  1.]
t:  [ 10.10233723  23.73542074]
v:  [-97.  48.]
Labels by index: Empty
```

2.4 Notes on determining the initial sub-model

PyDSTool requires that an initial condition (IC) for the state variables uniquely determines which sub-model should be used to begin determining a trajectory. This is done by the simulator, which evaluates the events and domain conditions associated with each sub-model at the IC. The IC should belong to the domains specified for the sub-model that will start the simulation. It is the user's obligation to place a set of self-consistent conditions on the IC in order for the hybrid solver to determine which sub-model to use at the starting time–the user may not explicitly name it.

Problems might arise if directionless threshold-crossing events do not partition the state space in a way that helps to determine in which sub-domain an initial condition lies, or if ICs lie exactly on

the event surface associated with one of the sub-models. One solution is an indicator variable, such as 'excited' in the above IF example. This is an artificial, discrete-valued state variable (usually an integer) that must lie in a singleton domain set associated with each state.

3 Example with the Hodgkin-Huxley action potential

We present a hybrid system implementation of the reduced Hodgkin-Huxley (HH) action potential (AP) based on a 'dominant scale' analysis [2, 3]. While the mathematical details are left to these references, we briefly describe the primary objects computed here. The dominant scales are measured for the 'inputs' to an ordinary differential equation (ODE) for an observable variable of interest. Here, that variable is the membrane potential, v. The inputs usually refer to variables appearing on the equation's right hand side (RHS), but parameters that play equivalent roles in the meaning of the terms on the RHS may also be categorized as inputs. this meaning depends on the form of ODE. In the current balance equation form of the spatially clamped HH model, these meanings are apparent from the summed terms corresponding to ionic or applied currents, where each has a variable or parameter governing its size:

$$C\frac{dv}{dt} = -\sum I_{\text{ionic}} + I_{\text{app}}, \tag{2}$$

Here, the somatic membrane capacitance is C, and the sum of ionic currents and an applied bias current that is possibly time-varying. Each ionic current takes the form $I_s = g_s s^{p_s} \tilde{s}^{q_s}(v - v_s)$ for channel reversal potential v_s, maximum conductance g_s, activation gating variable s and, if present, inactivation gating variable $\tilde{s}$, for some integer powers p_s and q_s (possibly 1). Similarly, the applied current is formally denoted aI_{app} for a formal variable $a \equiv 1$ for the constant applied current used here. The passive leak current is denoted $g_l l (v - v_l)$ with a formal activation $l \equiv 1$. This slightly non-standard notation makes later algorithmic definitions uniform.

Conductance-based inputs to the voltage equation belong to the index set $\Gamma_1 = \{m, n, l\}$ (h is an inactivation variable multiplying the activation variable m in the sodium current, and so h is not included in the set for the granularity of the present analysis), while the passive leak input belongs to $\Gamma_2 = \{a\}$ [4]. The instantaneous time scale of the voltage equation is given by

$$\tau_V = C / \sum_{s \in \Gamma_1} g_s s^{p_s} \tilde{s}^{q_s}. \tag{3}$$

The asymptotic target voltage is the membrane potential that would eventually be reached if all gating variables were held constant (i.e., the solution to $dv/dt = 0$ at time t for m, h, and n fixed according to their values on the given trajectory at time t). v_∞ is available explicitly for the models we consider here and plays the same role as s_∞ for a dynamic gating variable s:

$$v_\infty = \tau_v \left(\sum_{s \in \Gamma_1} g_s s^{p_s} \tilde{s}^{q_s} v_s + aI_{\text{app}} \right) / C. \tag{4}$$

Eq. (2) can now be rewritten in the first-order kinetic form used for the gating variables m, n, and h:

$$\frac{dv}{dt} = \left(v_\infty(m, n, h) - v \right) / \tau_v(m, n, h). \tag{5}$$

In this formalism, the reciprocal of the instantaneous time scale τ plays the role of a time-varying "eigenvalue" towards the time-varying "fixed point" value (the asymptotic target). If any one the values of variables $s = m, h, n$ is allowed to vary, the set of values $v_\infty(s)$ forms the respective nullcline in the (s, v) phase plane.

For the present purposes, the dominance (or influence strength) of an input s in one of the Γ sets is measured as a sensitivity:

$$\Psi_s = s \left| \frac{\partial v_\infty}{\partial s} \right| \tag{6}$$

As described in the main article text, dominant scale analysis indicated four reduced regimes that capture the essential dynamics of qualitatively distinct parts of the AP cycle, relative to the v variable that is the output visible to other neurons. The full code of the demonstration script shows how the dominant scale analysis was achieved, but this tutorial focuses on the encoding of the hybrid model associated with the derived regimes using Regime IV as an example (the other regimes are similar).

The PyDSTool toolbox 'dssrt' contains classes and functions for dominant scale analysis. The functions `define_psi_events` and `make_evs` are used to build regime transition event definitions from the specified changes in the regime template (see main text). In addition to the set of active inputs $\mathcal{A}$ in a regime, the analysis also defines the modulatory inputs $\mathcal{M}$ to the v equation as the inputs with dominance strengths at the 'scale below' the active inputs: using the scale tolerance threshold σ, modulatory inputs $s \in \mathcal{M}$ satisfy $\sigma \leq \Psi_x/\Psi_s < \sigma^2$ for the most dominant input $x \in \mathcal{A}$ at any time t. These are the inputs most likely to become active during the regime for any reason, and may be acting as quasi-static bifurcation parameters. The toolbox does all of the calculations of these input sets automatically, based on definitions of the target value, time scale, and Ψ quantities.

In Regime IV, the *domain rules* are given by the active and modulatory inputs for the sub-model are given by `acts4` and `mods4`, respectively:

```
acts4 = ['Iapp', 'leak', 'n']
mods4 = ['m']
psi_evs4 = make_evs(define_psi_events(acts4, mods4, 'v',
                    ignore_transitions=[('leave','Iapp'), ('leave', 'leak')]),
                    pars, event_tol, targetlang)
```

In the events declaration, `pars` is a dictionary mapping model parameter names to values (common to all regimes), and `event_tol` is the desired temporal accuracy of the event detection (e.g., 10^{-4} ms). The dominant scale analysis compresses certain trivial changes to the actives set $\mathcal{A}$ (for instance, the leaving of a passive current term in the ODE from the set, such as I_{app} or l in the above example) into the same set, by allowing some transitions to be ignored. To this end, the 'ignore_transitions' option avoids a proliferation of uninformative regimes when defining the *transition rules* for the regime. Lastly, the 'targetlang' argument is a string, `'c'` or `'python'`, set at the top of the script, which ensures the events are built for the needs of the C- or Python-based ODE solvers that are selected.

A convenient description of the ODEs for v in each regime's sub-model is via an 'auxiliary' function for the total ionic current to the cell, $\text{Itot}(v, m, n, h, l, a)$. When an input is present in a regime, its value will be provided to this function, otherwise the argument will be set to zero. The right-hand side for the v ODE in Regime IV is specified as

```
vfn_str4 = '(-Itot(v,0,0,n,1,1))/C'
```

where C is the membrane capacitance. In the dictionary of auxiliary functions declared to PyDSTool we include:

```
auxfns['Itot'] = (['v','m','h','n','l','a'],
                  'gna*m*m*m*h*(vv-vna)' + \
                  'gk*n*n*n*n*(vv-vk)' + \
                  'gl*l*(vv-vl)␣-␣a*Iapp')
```

This declaration consists of a pair: the six arguments to the function, which are local variables, and the string definition of the body. The state variables must be passed explicitly as they are not otherwise visible to the scope of this function, but static parameters such as g_{Na}, v_{Na}, g_K, v_K, g_l, v_l, I_{app} are globally visible.

We must declare a compatible internal ModelInterface class for the regime external ModelInterface that will verify the regime conditions:

```
class int_regime(intModelInterface):
    pass  # do nothing but declare name / sub-class

class regime4(extModelInterface):
    actives = acts4
    fast = ['n']    # not discussed here
    slow = []       # not discussed here
```

Inside an instance of this last class, the post-processing of a trajectory segment from Regime IV will be performed by a PyDSTool feature object, `reg4_feature` (an instance of the `regime_feature` sub-class defined below). Features are user-defined objects that build quantitative metrics of experimental or simulation data in a potentially hierarchical composition, enabling context-dependent detection of qualitative, emergent properties [5,6]. The type of feature we use here is qualitative, meaning that it only returns a Boolean to signal whether its detection criteria are met. To do this, we sub-class from `ql_feature_leaf` (we use leaf rather than node because there is no need for a hierarchical structure of features in this setup), and override its 'evaluate' method to provide the functionality. Static definitions used for feature evaluation are provided as declarations at the class level ('inputs', 'taus', 'infs', and 'psis_reg'), and provide the 'dssrt' toolbox with information about the system to analyze.

```
class regime_feature(ql_feature_leaf):
    inputs = {'v': args(gamma1=['m', 'n', 'leak'],
                        gamma2=['Iapp']),
              'm': args(gamma1=['v']),
              'n': args(gamma1=['v'])}
    taus = {'v': 'tau_v', 'm': 'tau_m', 'n': 'tau_n'}
    infs = {'v': 'inf_v', 'm': 'inf_m', 'n': 'inf_n'}
    psis_reg = { 'm': None, 'n': None,
                 'v': args(Iapp='tau_v*Iapp',
                           leak='tau_v*gl*abs(vl-inf_v)',
                           m='tau_v*gna*3*m*m*m*h*abs(vna-inf_v)',
                           n='tau_v*gk*4*n*n*n*n*abs(vk-inf_v)') }

    def evaluate(self, target):
        # code omitted
```

The 'evaluate' method extracts the trajectory segment from the target MI after the integration stopped at a terminal event. It then determines exactly what dominant scale change occurred and records audit information for the hybrid solver to choose the correct transition rule. It returns True if the regime's consistency conditions are met, otherwise it records the last time in the trajectory that was valid (so that the solver can roll-back the trajectory to that point before continuing) and returns False. Because the raw ODE integration can be made very fast using the C-based integrators, the efficiency of the hybrid model simulation will depend crucially on the speed at which this 'evaluate' method can operate. The 'dssrt' dominant scale toolbox functions are currently pure Python (but will later be optimized to use embedded C code for some loops) and perform complex calculations at every time point determined by the integrator. Thus the time step control of the integrator is the primary determinant of this post-processing step's speed.

```
reg4_feature = regime_feature('regime4', pars=args(actives=acts4, slow=[],
                    fast=['n'], debug=False))
reg4_condition = condition({reg4_feature: True})
reg4_eMI = regime4(conditions=reg4_condition,
                   compatibleInterfaces=['int_regime'])
```

We wish this regime feature to be present in the activity of the sub-model dynamics, so we set the condition on the feature to be True. (If we wanted the feature to be absent, we could set the condition to be False.)

We now declare all the regime names and the transition rule names that correspond to the results of consistency checks from external model interfaces (rather than terminal events):

```
all_names = ['regime1', 'regime2', 'regime3', 'regime4']
nonevent_reasons = ['join_m', 'join_n', 'leave_m', 'leave_n', 'join_leak',
                    'leave_leak', 'join_Iapp', 'leave_Iapp']
```

We build a sub-model into an internal ModelInterface `reg4_iMI` with code similar to that for the IF example, but including auxiliary variable definitions for the Ψ_s values that are used in post-processing. By providing these definitions in this way, they will be computed . The other sub-models for Regimes I–III are defined similarly, none of which require discrete state mappings.

```
aux_vars = args(
    psi_v_m='tau_v_fn(v,m,h,n)*gna*3*m*m*m*h*abs(vna-inf_v_fn(v,m,h,n))',
    psi_v_n='tau_v_fn(v,m,h,n)*gk*4*n*n*n*n*abs(vk-inf_v_fn(v,m,h,n))',
    psi_v_leak='tau_v_fn(v,m,h,n)*gl*abs(vl-inf_v_fn(v,m,h,n))',
    psi_v_Iapp='tau_v_fn(v,m,h,n)*Iapp'
    )

gen_reg4 = makeHHneuron('regime4', pars, ics, vfn_str, psi_evs, aux_vars)
model_reg4 = embed(gen_reg4)
reg4_iMI = int_regime(model_reg4)
```

where `ics` is any dictionary of initial conditions (ICs) for the sub-model. The `makeHHneuron` function creates a Generator object for the sub-model, which is then embedded in a NonHybridModel object and used as the basis for the regime's internal ModelInterface. The intermediate declarations storing `gen_reg4` and `model_reg4` provide direct access to these objects after they are placed into the ModelInterface, for possible later use.

Finally, we specify external model interface's global consistency conditions with the argument `globcon_list` below, as well as the transition rule back to Regime I, namely that n must leave $\mathcal{A}$:

```
all_info = []
# Info entries for regimes 1-3 omitted here
all_info.append(makeModelInfoEntry(reg4_iMI, all_names,
        [('leave_n', 'regime1')], nonevent_reasons=nonevent_reasons,
        globcon_list=[reg4_eMI]))
modelInfoDict = makeModelInfo(all_info)
hybrid_HH = HybridModel({'name': 'HH_hybrid', 'modelInfo': modelInfoDict})
```

The call to the constructor for the `HybridModel` class completes the creation of the model object 'hybrid_HH', which is now capable of being simulated, introspected, etc., in the ways already described.

A Appendix

A.1 Theoretical issues for hybrid dynamics

In this work, we have not considered theoretical issues such as the reachability of states (which we will assume is understood in advance based on the way the system is derived), formal verification of specified behaviors, or the stability analysis of non-smooth trajectories [7,8]. For instance, the current implementation is not guaranteed to accurately simulate physical impact models that exhibit chattering or Zeno behavior (an infinite number of transitions occurring within a finite time) [9], although it does accurately simulate the temporal synchronization of coupled hybrid-model oscillators that interact through discrete events. Zeno-like behaviors are not typical of the biological models considered here, and the operational approach of this work focuses on assumptions prevalent in biophysical models: e.g., smooth dynamics are treated as a more typical process than discrete event processes. As such, we take an alternative implementation approach to the majority of existing simulation platforms, which focus on physical models for engineering applications with many parallel discrete event processes mixed with continuous dynamics (see [10] and refs. therein).

A.2 Event detection in PyDSTool

Event detection in PyDSTool is inherent to the solvers, and is done transparently to the user. The implementations are built into the C code wrappers for Dopri and Radau, and supported for the VODE integrator's wrapper using Python code. For Dopri and Radau, we make use of the dense representation of the solution (a polynomial interpolation to the same high order as the solvers) in the neighborhood of two successive integration steps that were found to straddle the event surface. The polynomial's intersection with this surface is then determined using simple bisection to within the desired tolerance in time. This method ensures convergence and works quickly in practice. VODE event detection is achieved by incrementally reducing the step size near the zero crossing. All event points are automatically inserted in temporal order into the output array of trajectory points, as well as being separately recorded in Generator attributes and Pointset labels for easy reference.

References

[1] Lapique L (1907) Recherches quantitatives sur l'excitation électriques des nerfs traitée comme une polarization. J Physiol Pathol Gen 9: 620–635.

[2] Clewley R (2004) Dominant-scale analysis for the automatic reduction of high-dimensional ODE systems. In: Bar-Yam Y, editor, ICCS 2004 Proceedings. New England Complex Systems Institute.

[3] Clewley R (2010) Encoding the fine-structured mechanism of action potential dynamics with qualitative motifs. J Comput Neurosci 30: 391–408.

[4] Clewley R, Rotstein HG, Kopell N (2005) A computational tool for the reduction of nonlinear ODE systems possessing multiple scales. Multiscale Modeling and Simulation 4: 732–759.

[5] Clewley R, Dobric M (2010) A qualitative optimization technique for biophysical neuron models with many parameters. In: BMC Neuroscience. volume 11(Suppl 1).

[6] Druckmann S, Berger TK, Hill S, Schurmann F, Segev I (2008) Evaluating automated parameter constraining procedures of neuron models by experimental and surrogate data. Biological Cybernetics 99: 371-379.

[7] di Bernardo M, Budd C, Champneys A, Kowalczyk P (2007) Piecewise-smooth dynamical systems: Theory and Applications. Springer.

[8] Van Der Schaft A, Schumacher JM (2000) An Introduction to Hybrid Dynamical Systems. London: Springer.

[9] Simic SN, Johansson KH, Lygeros J, Sastry S (2005) Towards a geometric theory of hybrid systems. Dynamics of Continuous, Discrete and Impulsive Systems, Series B 12: 649–687.

[10] Carloni L, DiBenedetto M, Pinto A, Sangiovanni-Vincentelli A (2004) Modeling techniques, programming languages, and design toolsets for hybrid systems. Technical report, Technical Report IST-2001-38314 WPHS, Columbus Project.
