# Supplementary material for: Hybrid Models and Biological Model Reduction with PyDSTool
Source: PLoS Comput Biol. 2012 Aug 9;8(8):e1002628. doi: 10.1371/journal.pcbi.1002628 (PMC3415397; doi:10.1371/journal.pcbi.1002628)
Supplement: Text S2 — Syntax-highlighted code for the demonstration script IF_squarespike_model.py. (PDF) [file pcbi.1002628.s002.pdf]

Supplemental Text 2: Code for PyDSTool/tests/IF_squarespike_model.py

```
"""Library function to build Integrate and fire model with square-pulse
spike, as a hybrid system.

   Robert Clewley, March 2005.
"""

from PyDSTool import *
from time import clock

# --------------------------------------------------------------------

def makeLinearLeak(name, rhs, par_args, inputs, evtol=None):
    rhs_full = {'v': "(Iapp␣-␣gl*(v-vl))/C",
                'excited': "0"}
    if rhs is not None:
        assert isinstance(rhs, dict)
        rhs_full.update(rhs)
    # testaux demonstrates a simple auxiliary variable using the global
    # independent variable built-in function
    rhs_full['testaux'] = "globalindepvar(t)-50"
    for parname in ['threshval', 'vl', 'gl', 'Iapp', 'C']:
        assert parname in par_args, "Essential␣pars␣missing"
    if evtol is None:
        evtol = 1e-3
    DS_event_args = {'name': 'threshold',
               'eventtol': evtol,
               'eventdelay': evtol*0.1,
               'starttime': 0,
               'term': True
                }
    thresh_ev = Events.makeZeroCrossEvent('v␣-␣threshval', 1,
                                        DS_event_args,
                                        varnames=['v'],
                                        parnames=['threshval'])

    DS_args = {'pars': par_args,
               'varspecs': rhs_full,
               'auxvars': 'testaux',
               'xdomain': {'v': [-120,50], 'excited': 0.},
```

```
                'ics': {'v': -80, 'excited': 0},
                'tdomain': [0,Inf],
                'algparams': {'init_step': 0.1},
                'events': thresh_ev,
                'name': name}

    if inputs != {}:
        DS_args['inputs'] = inputs

    return embed(Generator.Vode_ODEsystem(DS_args), name=name, tdata=[0,200])


def makeSpike(name, par_args):
    # spike length parameter 'splen' must be contained within 'tdomain' in
    # order to get a fully-formed square-pulse `spike`
    DS_spike_args = {'tdomain': [0.0, 1.5],
            'varspecs': {'v': "if(t<splen,48,-97)", 'excited': "1",
                         'testaux': "globalindepvar(t)-50"},
            'auxvars': 'testaux',
            'ics': {'v': 48, 'excited': 1},
            'pars': {'splen': par_args['splen']},
            'xdomain': {'v': [-98, 51], 'excited': 1.},
            'name': name}
    return embed(Generator.ExplicitFnGen(DS_spike_args), name=name)


def makeIFneuron(name, par_args_linear, par_args_spike, rhs=None, inputs={},
                 icdict=None, evtol=None):
    allDSnames = ['linear', 'spike']

    # get models
    DS_linear = makeLinearLeak('linear', rhs, par_args_linear,
                               inputs, evtol=evtol)
    DS_spike = makeSpike('spike', par_args_spike)

    # make model interfaces
    DS_linear_MI = intModelInterface(DS_linear)
    DS_spike_MI = intModelInterface(DS_spike)

    DS_linear_info = makeModelInfoEntry(DS_linear_MI, allDSnames,
                                        [('threshold', 'spike')])
    DS_spike_info = makeModelInfoEntry(DS_spike_MI, allDSnames,
                                        [('time', 'linear')])
    modelInfoDict = makeModelInfo([DS_linear_info, DS_spike_info])

    # 'excited' is an indicator variable of the model, and is used to
    # ensure that the compute() method can determine which DS
    # to start the calculation with
    mod_args = {'name': name,
                'modelInfo': modelInfoDict}
    if icdict is not None:
        mod_args['ics'] = icdict.copy()
```

```
    IFmodel = Model.HybridModel(mod_args)
    return IFmodel


# --------------------------------------------------------------------

if __name__=='__main__':
    # need the __main__ to use above functions as imports for other
    # scripts without running this part
    print '--------␣IF␣model␣test␣1'

    par_args_linear = {'Iapp': 1.3, 'gl': 0.1, 'vl': -67,
                       'threshval': -65, 'C': 1}
    par_args_spike = {'splen': 0.75}

    IFmodel = makeIFneuron('IF_fit', par_args_linear, par_args_spike)
    icdict = {'v': -80, 'excited': 0}

    start = clock()
    print 'Computing␣trajectory...'
    IFmodel.compute(trajname='onespike',
                    tdata=[0, 60],
                    ics=icdict,
                    verboselevel=0)
    print '\n...␣finished␣in␣%.3f␣seconds.\n' % (clock()-start)

    IFmodel.set(pars={'Iapp': 1.0, 'threshval': -60})
    print 'Recomputing␣trajectory␣with␣new␣params...'
    IFmodel.compute(trajname='twospike',
                    tdata=[0, 60],
                    ics=icdict)


    print 'Preparing␣plot'
    plotData = IFmodel.sample('onespike', dt=0.05)
    plotData2 = IFmodel.sample('twospike', ['v', 'testaux'], 0.05)
    plt.ylabel('v,␣testaux')
    plt.xlabel('t')
    vline = plt.plot(plotData['t'], plotData['v'])
    vline2 = plt.plot(plotData2['t'], plotData2['v'])
    aline = plt.plot(plotData['t'], plotData['testaux'])

    print "\nLast␣point␣of␣hybrid␣trajectory:␣"
    print "IFmodel.getEndPoint('onespike')␣-->\n", \
          IFmodel.getEndPoint('onespike')

    print "\nFirst␣point␣of␣hybrid␣trajectory:␣"
    print "IFmodel.getEndPoint('onespike',␣0)␣-->\n", \
          IFmodel.getEndPoint('onespike', 0)

    print "Testing␣IF␣hybrid␣model␣as␣mapping␣..."
```

```
num_parts = len(IFmodel.getTrajTimePartitions('twospike'))
#eventvals = IFmodel('onespike', range(0, num_parts+1), asmap=True)
eventvals = IFmodel('twospike', range(0, num_parts+1), asmap=True)
for i in range(0,num_parts+1):
    print "(v, t) at event(%i) = (%.4f, %.4f)" % (i, eventvals(i)('v'),
                                              eventvals(i)('t'))
print "\nAlternative access to explicit event info using " + \
      "getTrajEvents(trajname) method:\n"
evs = IFmodel.getTrajEvents('twospike')
evtimes = IFmodel.getTrajEventTimes('onespike')
print evs
assert len(evs['threshold']) == 2, "Problem with hybrid events"
assert len(evtimes['threshold']) == 4, "Problem with hybrid events"
assert allclose(evtimes['threshold'][3], 54.009, 1e-3), \
         "Problem with hybrid events"
assert allclose(evs['threshold'][1]['v'], -60, 1e-3), \
         "Problem with hybrid events"

print "\nDepending on your platform and pylab configuration you may need"
print " to execute the plt.show() command to see the plots"
# plt.show()
```
