# Supplementary material for: Hybrid Models and Biological Model Reduction with PyDSTool
Source: PLoS Comput Biol. 2012 Aug 9;8(8):e1002628. doi: 10.1371/journal.pcbi.1002628 (PMC3415397; doi:10.1371/journal.pcbi.1002628)
Supplement: Text S3 — Syntax-highlighted code for the demonstration script HH_DSSRTtest.py. (PDF) [file pcbi.1002628.s003.pdf]

Supplemental Text 3: Code for PyDSTool/tests/HH_DSSRTtest.py

```
"""Reproduces dominant scale analysis results for Hodgkin-Huxley neuron model
from R. Clewley, Proc. ICCS 2004."""

from PyDSTool import *
from PyDSTool.Toolbox.dssrt import *
import sys

# ------------------------------------------------------------
targetlang = 'python'
# Works much faster if you use 'c', provided gcc and SWIG installed
# ------------------------------------------------------------

Itot_defn = (['v', 'm', 'h', 'n', 'l', 'a'],
              'gna*m*m*m*h*(v-vna)␣+␣gk*n*n*n*n*(v-vk)␣+␣' + \
              'l*gl*(v-vl)␣-␣a*Iapp')

def makeHHneuron(name, par_args, ic_args, vfn_str='(-Itot(v,m,h,n,1,1))/C',
                 evs=None, aux_vars=None):
    mfn_str = 'ma(v)*(1-m)-mb(v)*m'
    nfn_str = 'na(v)*(1-n)-nb(v)*n'
    hfn_str = 'ha(v)*(1-h)-hb(v)*h'

    auxdict = {
        'Itot': Itot_defn,
        'ma': (['v'], '0.32*(v+54)/(1-exp(-(v+54)/4))'),
        'mb': (['v'], '0.28*(v+27)/(exp((v+27)/5)-1)'),
        'ha': (['v'], '.128*exp(-(50+v)/18)'),
        'hb': (['v'], '4/(1+exp(-(v+27)/5))'),
        'na': (['v'], '.032*(v+52)/(1-exp(-(v+52)/5))'),
        'nb': (['v'], '.5*exp(-(57+v)/40)'),
        'tau_v_fn': (['v', 'm', 'h', 'n'],
                     'C/(gna*m*m*m*h␣+␣gk*n*n*n*n␣+␣gl)'),
        'inf_v_fn': (['v', 'm', 'h', 'n'],
                     'tau_v_fn(v,␣m,␣h,␣n)*(gna*m*m*m*h*vna␣+␣' + \
                     'gk*n*n*n*n*vk␣+␣gl*vl␣+␣Iapp)/C')}

    DSargs = {}
    DSargs['varspecs'] = {'v': vfn_str, 'm': mfn_str,
                          'h': hfn_str, 'n': nfn_str,
                          'tau_v': 'tau_v_fn(v,␣m,␣h,␣n)',
                          'inf_v': 'inf_v_fn(v,␣m,␣h,␣n)',
```

```
                            'tau_m': '1/(ma(v)+mb(v))',
                            'inf_m': 'ma(v)/(ma(v)+mb(v))',
                            'tau_n': '1/(na(v)+nb(v))',
                            'inf_n': 'na(v)/(na(v)+nb(v))',
                            'tau_h': '1/(ha(v)+hb(v))',
                            'inf_h': 'ha(v)/(ha(v)+hb(v))'}
    DSargs['auxvars'] = ['tau_v', 'inf_v', 'tau_m', 'inf_m', 'tau_n', 'inf_n',
                         'tau_h', 'inf_h']
    if aux_vars is not None:
        DSargs['varspecs'].update(aux_vars)
        DSargs['auxvars'].extend(aux_vars.keys())
    DSargs['pars'] = par_args
    DSargs['fnspecs'] = auxdict
    DSargs['xdomain'] = {'v': [-130, 70], 'm': [0,1], 'h': [0,1], 'n': [0,1]}
    if targetlang == 'python':
        # init_step must be small as VODE integrator will only record
        # at a fixed step. max_step is irrelevant for this integrator.
        DSargs['algparams'] = {'init_step': 0.01}
    else:
        # adaptive step
        DSargs['algparams'] = {'init_step': 0.1, 'max_step': 1}
    DSargs['checklevel'] = 0
    DSargs['ics'] = ic_args
    DSargs['name'] = name
    # These optional events are for convenience, e.g. to find period of
    # oscillation
    peak_ev = Events.makeZeroCrossEvent(vfn_str, -1,
                              {'name': 'peak_ev',
                               'eventtol': 1e-5,
                               'term': False}, ['v','m','n','h'], par_args.keys(),
                              fnspecs={'Itot': auxdict['Itot']},
                              targetlang=targetlang)
    trough_ev = Events.makeZeroCrossEvent(vfn_str, 1,
                              {'name': 'trough_ev',
                               'eventtol': 1e-5,
                               'term': False}, ['v','m','n','h'], par_args.keys(),
                              fnspecs={'Itot': auxdict['Itot']},
                              targetlang=targetlang)
    DSargs['events'] = [peak_ev, trough_ev]
    if evs is not None:
        DSargs['events'].extend(evs)
    if targetlang == 'python':
        return Generator.Vode_ODEsystem(DSargs)
    else:
        return Generator.Dopri_ODEsystem(DSargs)


# ----------------------------------------------------------------

pars = {'gna': 100, 'gk': 80, 'gl': 0.1,
        'vna': 50, 'vk': -100, 'vl': -67,
        'Iapp': 1.75, 'C': 1.0}
```

```
ics = {'v':-70.0, 'm': 0, 'h': 1, 'n': 0}

HH = makeHHneuron('HH_DSSRT', pars, ics)
HH.set(tdata=[0, 150])
traj = HH.compute('pre-test')

# ------------------------------------------------------------------
# ANALYSIS using DSSRT
# set proper IC from last minimum event
new_ics = HH.getEvents()['trough_ev'][-1]
# get a couple of periods so that epochs can be compared from cycle to cycle
HH.set(ics=new_ics, tdata=[0, 50])
traj = HH.compute('test')
pts = traj.sample()

# Start a new trajectory from the end of the previous one to ensure very close
# to a limit cycle, but don't use the continue option of compute so as to limit
# number of epochs needed to be computed later
HH.set(ics=pts[-1])
traj = HH.compute('test')
pts = traj.sample()

### DSSRT-related
Dargs = args()
Dargs.internal_vars = ['h']
Dargs.model = HH

# initial values
Dargs.inputs = {}
Dargs.taus = {}
Dargs.infs = {}
Dargs.psis = {}

for var in ics.keys():
    Dargs.taus[var] = 'tau_%s' % var
    Dargs.infs[var] = 'inf_%s' % var
    Dargs.psis[var] = None
    Dargs.inputs[var] = args(gamma1=['v'])

Dargs.inputs['v'] = args(gamma1=['m', 'n', 'leak'],
                         gamma2=['Iapp'])
Dargs.inputs['leak'] = None
Dargs.inputs['Iapp'] = None

Dargs.psis['v'] = args(
    m='tau_v*gna*3*m*m*m*h*abs(vna-inf_v)',
    n='tau_v*gk*4*n*n*n*n*abs(vk-inf_v)',
    leak='tau_v*gl*abs(vl-inf_v)',
    Iapp='tau_v*Iapp')


da = dssrt_assistant(Dargs)
```

```
da.focus_var='v'
da.traj=traj
da.calc_psis()
da.make_pointsets()
a = da.psi_pts
da.calc_rankings()

gamma = 3 # time scale threshold
sigma = 3 # dominance scale threshold
min_len = 10000
cycle_ixs = []
da.domscales['psi'].calc_epochs(sigma, gamma)
epochs = da.domscales['psi'].epochs
cycle_len, cycle_ixs = find_epoch_period(epochs)
epochs = da.domscales['psi'].epochs[cycle_ixs[0]:cycle_ixs[1]]

t0 = epochs[0].t0
t1 = epochs[-1].t1
ix0 = pts.find(t0) # guaranteed to be exact
ix1 = pts.find(t1) # guaranteed to be exact
ts = pts['t'][ix0:ix1+1]
cycle = pts[ix0:ix1+1]
plt.plot(ts, cycle['v'])
plt.plot(ts, cycle['inf_v'])
plt.title('v(t) and v_inf(t) for one approximate period')
print "Graph shows **approximate** period of tonic spiking =", t1-t0

for ep in epochs:
    ep.info()
    plot(ep.t0,ep.traj_pts[0]['v'],'k.')



# ---------------------------------------------------------------------------
# SYNTHESIS
# build model interfaces for each regime, according to regimes determined
# in ICCS 2004 conference proceedings paper.

pars.update({'dssrt_sigma': 3, 'dssrt_gamma': 3})

def make_evs(evdefs, pars, evtol, targetlang):
    extra_evs = []
    for evname, evargs in evdefs.items():
        all_pars = pars.keys()
        all_pars.extend(remain(evargs.pars, pars.keys()))
        extra_evs.append(makeZeroCrossEvent(evargs.defn, evargs.dirn,
                              {'name': evname,
                               'eventtol': evtol, 'term': True},
                              ['psi_v_Iapp', 'psi_v_m', 'psi_v_n', 'psi_v_leak',
                               'tau_v', 'tau_m', 'tau_n', 'tau_h'],
                              all_pars,
                              fnspecs={'Itot': Itot_defn},
```

```
                        targetlang=targetlang))
    return extra_evs

class iface_regime(extModelInterface):
    pass


class regime_feature(ql_feature_leaf):
    inputs = {'v': args(gamma1=['m', 'n', 'leak'],
                         gamma2=['Iapp']),
              'm': args(gamma1=['v']),
              'n': args(gamma1=['v'])}
    taus = {'v': 'tau_v', 'm': 'tau_m', 'n': 'tau_n'}
    infs = {'v': 'inf_v', 'm': 'inf_m', 'n': 'inf_n'}
    psis_reg = { 'm': None, 'n': None,
                 'v': args(Iapp='tau_v*Iapp',
                           leak='tau_v*gl*abs(vl-inf_v)',
                           m='tau_v*gna*3*m*m*m*h*abs(vna-inf_v)',
                           n='tau_v*gk*4*n*n*n*n*abs(vk-inf_v)') }
    da_regimes = {}   # fill these in later

    def evaluate(self, target):
        # Determine DSSRT-related info about next hybrid state to switch to,
        # and whether epoch conditions are met throughout the trajectory.
        #
        # Acquire underlying Generator from target model interface
        gen = target.model.registry.values()[0]
        ptsFS = target.test_traj.sample()

        # pick up or create DSSRT assistant object for this regime
        try:
            da_reg = self.da_regimes[gen.name]
        except KeyError:
            Dargs = args(model=gen, inputs=self.inputs,
                  taus=self.taus, infs=self.infs, psis=self.psis_reg)
            da_reg = dssrt_assistant(Dargs)  # SLOW, so re-use these
            self.da_regimes[gen.name] = da_reg
        else:
            # reset, otherwise epochs won't be recalculated
            da_reg.reset()

        da_reg.focus_var = 'v'
        ptsFS.mapNames(gen._FScompatibleNames)

        # refer to target's current trajectory in da
        # and down-sample at an appropriate rate to minimize the slow,
        # pure-python post-processing in the DSSRT toolbox
        if len(ptsFS) > 100:
            ds_fac = 6
        elif len(ptsFS) > 20:
            ds_fac = 2
        else:
```

```
    ds_fac = None
if ds_fac is not None:
    new_pts = ptsFS[::ds_fac]
    try:
        new_pts.append(ptsFS[[-1]])
    except:
        # length was a multiple of 5 so final point was already in
        pass
    da_reg.traj = pointset_to_traj(new_pts)
else:
    da_reg.traj = target.test_traj
da_reg.traj.mapNames(gen._FScompatibleNames)

gamma = gen.pars['dssrt_gamma']
sigma = gen.pars['dssrt_sigma']
da_reg.calc_psis()
da_reg.make_pointsets()
da_reg.calc_rankings()
da_reg.domscales['psi'].calc_epochs(sigma, gamma)

epochs = da_reg.domscales['psi'].epochs
epoch_reg = epochs[-1]

# don't bother using timescale criterion -- not relevant to
# this example anyway
criteria_types = ['psi_reg'] #, 'timescale']
# find one of these criteria from the terminal event
# that occurred
warns = gen.diagnostics.findWarnings(Generator.W_TERMEVENT)
if len(warns) > 0:
    term_ev = warns[-1]
    evname = term_ev[1][0]
    # the terminal event ftol tolerance must be small enough
    # to distinguish near-concurrent events
    ftol = 1e-2
    tran = var = crit = None
    try:
        tran, var = transition_psi(epoch_reg, ptsFS[-1], ftol)
    except PyDSTool_ValueError:
        ftol = 1e-4
        try:
            tran, var = transition_psi(epoch_reg, ptsFS[-1], ftol)
        except PyDSTool_ValueError:
            # no transition, but the flagged event may cause an error
            #pass
            raise
    else:
        crit = 'psi_reg'
    # var is the offending variable that breaks the regime
    if crit is not None:
        self.results.reasons = [tran+'_'+var]
    else:
```

```
                self.results.reasons = []

        acts = epoch_reg.actives
        ref_acts = self.pars.actives
        test1 = len(intersect(acts, ref_acts)) == len(ref_acts)
        test2 = len(intersect(epoch_reg.fast, self.pars.fast)) == \
            len(self.pars.fast)
        test3 = len(intersect(epoch_reg.slow, self.pars.slow)) == \
            len(self.pars.slow)

        # diagnostics
        if test1 and (not test3 or not test2):
            print "Time␣scale␣tests␣failed"
            print "Fast:", epoch_reg.fast, self.pars.fast
            print "Slow:", epoch_reg.slow, self.pars.slow
        return test1 and test2 and test3


aux_vars = args(
    psi_v_m=’tau_v_fn(v,␣m,␣h,␣n)*gna*3*m*m*m*h*abs(vna-inf_v_fn(v,␣m,␣h,␣n))’,
    psi_v_n=’tau_v_fn(v,␣m,␣h,␣n)*gk*4*n*n*n*n*abs(vk-inf_v_fn(v,␣m,␣h,␣n))’,
    psi_v_leak=’tau_v_fn(v,␣m,␣h,␣n)*gl*abs(vl-inf_v_fn(v,␣m,␣h,␣n))’,
    psi_v_Iapp=’tau_v_fn(v,␣m,␣h,␣n)*Iapp’)

##
## build hybrid model
all_model_names = [’regime1’, ’regime2’, ’regime3’, ’regime4’]
nonevent_reasons = [’join_m’, ’join_n’, ’leave_m’, ’leave_n’, ’join_leak’,
                    ’leave_leak’, ’join_Iapp’, ’leave_Iapp’, ’fast_join_ev’,
                    ’fast_leave_ev’, ’slow_join_ev’, ’slow_leave_ev’]

evtol = 1e-4
debug = False  # Use True for full traceback in case of problems

class int_regime(intModelInterface):
    pass


# regime 1: Iapp, leak
vfn_str1 = ’(-Itot(v,0,0,0,1,1))/C’
acts1 = [’Iapp’,’leak’]
mods1 = [’m’, ’n’]
psi_evs1 = make_evs(define_psi_events(acts1, mods1, ’v’,
                      ignore_transitions=[(’leave’,’Iapp’), (’leave’, ’leak’)]),
                    pars, evtol, targetlang)
# don’t make the tau_evs since we put them all in nonevent_reasons anyway,
# otherwise provide some in the ignore_transitions list
tau_evs1 = []
#tau_evs1 = make_evs(define_tau_events([], [], [’v’], ’v’),
#               pars, evtol, targetlang)
gen_reg1 = makeHHneuron(’regime1’, pars, ics, vfn_str1,
                        psi_evs1+tau_evs1, aux_vars)
```

```
model_reg1 = embed(gen_reg1)
reg1_iMI = int_regime(model_reg1)

class regime1(iface_regime):
    actives = acts1
    fast = []
    slow = []

reg1_feature = regime_feature('regime1', pars=args(actives=acts1,
                                               slow=[], fast=[],
                                               debug=debug))
reg1_condition = condition({reg1_feature: True})
reg1_eMI = regime1(conditions=reg1_condition,
                  compatibleInterfaces=['int_regime'])

# regime 2: Iapp, leak, m
vfn_str2 = '(-Itot(v,m,h,0,1,1))/C'
acts2 = ['Iapp', 'leak', 'm']
mods2 = ['n']
psi_evs2 = make_evs(define_psi_events(acts2, mods2, 'v',
                     ignore_transitions=[('leave','Iapp'), ('leave', 'leak')]),
                    pars, evtol, targetlang)
# don't make the tau_evs since we put them all in nonevent_reasons
tau_evs2 = []
#tau_evs2 = make_evs(define_tau_events([], ['m'], ['v'], 'v'),
#              pars, evtol, targetlang)
gen_reg2 = makeHHneuron('regime2', pars, ics, vfn_str2,
                       psi_evs2+tau_evs2, aux_vars)
model_reg2 = embed(gen_reg2)
reg2_iMI = int_regime(model_reg2)

class regime2(iface_regime):
    actives = acts2
    fast = ['m']
    slow = []

reg2_feature = regime_feature('regime2', pars=args(actives=acts2,
                                           slow=[], fast=['m'],
                                           debug=debug))
reg2_condition = condition({reg2_feature: True})
reg2_eMI = regime2(conditions=reg2_condition,
                  compatibleInterfaces=['int_regime'])

# regime 3: m, n
vfn_str3 = '-Itot(v,m,h,n,0,0)/C'
acts3 = ['m', 'n']
mods3 = ['leak', 'Iapp']
psi_evs3 = make_evs(define_psi_events(acts3, mods3, 'v',
                     ignore_transitions=[('join','Iapp'), ('join', 'leak')]),
                    pars, evtol, targetlang)
# don't make the tau_evs since we put them all in nonevent_reasons
tau_evs3 = []
```

```
#tau_evs3 = make_evs(define_tau_events(['n'], [], ['m', 'v'], 'v'),
#                pars, evtol, targetlang)
gen_reg3 = makeHHneuron('regime3', pars, ics, vfn_str3,
                        psi_evs3+tau_evs3, aux_vars)
model_reg3 = embed(gen_reg3)
reg3_iMI = int_regime(model_reg3)

class regime3(iface_regime):
    actives = acts3
    fast = []
    slow = ['n']

reg3_feature = regime_feature('regime3', pars=args(actives=acts3,
                                             slow=['n'], fast=[],
                                             debug=debug))
reg3_condition = condition({reg3_feature: True})
reg3_eMI = regime3(conditions=reg3_condition,
                  compatibleInterfaces=['int_regime'])

# regime 4: Iapp, leak, n
vfn_str4 = '(-Itot(v,0,0,n,1,1))/C'
acts4 = ['Iapp', 'leak', 'n']
mods4 = ['m']
psi_evs4 = make_evs(define_psi_events(acts4, mods4, 'v',
                     ignore_transitions=[('leave','Iapp'), ('leave', 'leak')]),
                    pars, evtol, targetlang)
# don't make the tau_evs since we put them all in nonevent_reasons
tau_evs4 = []
#tau_evs4 = make_evs(define_tau_events([], ['n'], ['v'], 'v'),
#                pars, evtol, targetlang)
gen_reg4 = makeHHneuron('regime4', pars, ics, vfn_str4,
                        psi_evs4+tau_evs4, aux_vars)
model_reg4 = embed(gen_reg4)
reg4_iMI = int_regime(model_reg4)

class regime4(iface_regime):
    actives = acts4
    fast = ['n']
    slow = []

reg4_feature = regime_feature('regime4', pars=args(actives=acts4,
                                             slow=[], fast=['n'],
                                             debug=debug))
reg4_condition = condition({reg4_feature: True})
reg4_eMI = regime4(conditions=reg4_condition,
                  compatibleInterfaces=['int_regime'])

## Combine regime sub-models into hybrid model
all_info = []
all_info.append(makeModelInfoEntry(reg1_iMI, all_model_names,
                    [('join_m', 'regime2')],
                nonevent_reasons=nonevent_reasons, globcon_list=[reg1_eMI]))
```

```
all_info.append(makeModelInfoEntry(reg2_iMI, all_model_names,
                        [('join_n', 'regime3')],
                nonevent_reasons=nonevent_reasons, globcon_list=[reg2_eMI]))
all_info.append(makeModelInfoEntry(reg3_iMI, all_model_names,
                        [('leave_m', 'regime4')],
                nonevent_reasons=nonevent_reasons, globcon_list=[reg3_eMI]))
all_info.append(makeModelInfoEntry(reg4_iMI, all_model_names,
                        [('leave_n', 'regime1')],
                nonevent_reasons=nonevent_reasons, globcon_list=[reg4_eMI]))
modelInfoDict = makeModelInfo(all_info)

hybrid_HH = HybridModel({'name': 'HH_hybrid', 'modelInfo': modelInfoDict})

## Compute test trajectory at original parameter values
hybrid_HH.compute(trajname='test', tdata=[0,40], ics=ics, verboselevel=2)
pts_hyb=hybrid_HH.sample('test')

HH.set(ics=filteredDict(pts_hyb[0],pts.coordnames))
HH.set(tdata=[0,40])
traj = HH.compute('orig')
pts_orig = traj.sample()

plt.figure()
plt.plot(pts_orig['t'],pts_orig['v'],'b')
plt.plot(pts_hyb['t'], pts_hyb['v'], 'g')
plt.title('Original (B) and hybrid (G) model voltage vs. t')

## Bifurcation-like diagram, to compare at different parameter values
print "\nComparing bifurcations of spiking onset"

bifpar = 'Iapp'
bifpar_range = concatenate((linspace(0.2, 0.4, 7), linspace(0.5, 3, 18)))

period_data_orig = []
period_data_hyb = []

def max_V(traj):
    # not used here
    return max(traj.getEvents('peak_ev')['v'])

def find_period(traj):
    """Find approximate period of oscillation using the last two
    peak event times in voltage"""
    try:
        evs = traj.getEventTimes('peak_ev')
        return evs[-1] - evs[-2]
    except:
        return 0

# Compute long runs (100 ms) to approximate a settled periodic orbit
print "\nOriginal model",
sys.stdout.flush()
```

```
for bpval in bifpar_range:
    print ".",
    sys.stdout.flush()
    HH.set(pars={bifpar: bpval}, tdata=[0,100])
    traj = HH.compute('orig_bif')
    period_data_orig.append(find_period(traj))

print "\nHybrid␣model",
sys.stdout.flush()
for i, bpval in enumerate(bifpar_range):
    print ".",
    sys.stdout.flush()
    hybrid_HH.set(pars={bifpar: bpval})
    hybrid_HH.compute('hyb_bif', tdata=[0,100], ics=ics, verboselevel=0,
                      force=True)
    period_data_hyb.append(find_period(hybrid_HH['hyb_bif']))

plt.figure()
plt.plot(bifpar_range, period_data_hyb, 'ko')
plt.plot(bifpar_range, period_data_orig, 'kx')
print "\nDepending␣on␣your␣platform␣and␣pylab␣configuration␣you␣may␣need"
print "␣to␣execute␣the␣plt.show()␣command␣to␣see␣the␣plots"
# plt.show()
```
