# Supplementary material for: Hybrid Models and Biological Model Reduction with PyDSTool
Source: PLoS Comput Biol. 2012 Aug 9;8(8):e1002628. doi: 10.1371/journal.pcbi.1002628 (PMC3415397; doi:10.1371/journal.pcbi.1002628)
Supplement: Text S4 — Complete source code for the PyDSTool package (version 0.88.120504). Includes API documentation and help files linking to web pages. This file is identical to the current public release on Sourceforge.net. (ZIP) [file pcbi.1002628.s004.zip › PyDSTool/FAQ.html]

PyDSTool -- Frequently Asked Questions


# Frequently Asked Questions

1. Questions about set-up and installation- Questions about using the libraries- Questions about documentation

---


## 1. Questions about set-up and installation

**1.1 : On unix systems, when I run a PyDSTool script involving a C-based ODE integrator, I get the error: "don't know how to
compile C/C++ code on platform 'posix' with 'gcc' compiler". How do I fix it?**

The most likely problem is that Python's compiler, accessed through the 'distutils' package,
is looking at an environment variable 'CC' that is set wrong.
Go to the Python installation directory's /lib/python2.4/config/Makefile. Grep that file for gcc and
see if the first line returned starts with CC = gcc ... e.g. something like this

$ cd *python\_installation\_dir*  
$ grep -n gcc /lib/python2.4/config/Makefile

If this entry is not set correctly you might have to rebuild Python with that entry changed by hand.

You should also see what your shell's CC is set to, e.g. using the 'set' command from the unix prompt.
There is an identically-named environment variable in the shell, which may not be the critical one to have set correctly.
The shell environment variable CC does **not** necessarily have to be set to 'gcc'.
If the makefile is correct for you, but the shell CC is not, you could try 'export CC=gcc' or 'set CC=gcc' before the python
session to see if that works. ... *and please let me know the outcome!*

**1.2 : Are there going to be installers available for the different platforms?**

For unix systems we are developing a shell script using autoconf that will collect and compile appropriate versions of the sources
for the underlying libraries and their dependencies. Windows installation from binaries is relatively simple in comparison,
but Windows users wishing to install using a comparable script will probably have to have a unix-like shell already
available (such as Cygwin or MinGW's MinSys). We are also investigating the use of a proper Python setup.py installer
for PyDSTool itself.

**1.3 : I want to use native 64 bit with my python installation,
but I get errors when I try to use Dopri, Radau, or AUTO. What do I
do?**

In lieu of an automated system for recognizing fully-working native 64
bit platforms in python, PyDSTool is currently hard-wired to make
32-bit compatible compilations of all C and Fortran code. To remove
that, simply do a search for the string '-m32' which appears in the
Dopri\_ODEsystem.py, Radau\_ODEsystem.py, and ContClass.py files. Remove
these strings leaving the other options and it should work. 64 bit
support is in development and feedback about your experience is
helpful in better supporting this in the future.

  

---

  


## 2. Questions about using the libraries

**2.1 : Why do I get an error about using the carat character (^) in my function specifications?**

There are problems in Python when trying to express powers in the form x^p, where x and p are floating point
numbers. The safest solution is to use the syntax pow(x,p) or x\*\*p instead. PyDStool provides a utility called
'convertPowers(my\_string, target)' which will automatically convert expressions involving powers (using any
mixture of ^, \*\*, or pow()), and return the converted string. The target argument must be a string: '^', '\*\*', or
'pow'.

**2.2 : Is it possible to write the entire RHS in C/C++ for the Dopri
or Radau integrators?**

Yes, but I only recommend doing it in a certain way. Python still
needs to see what variables and parameters you are declaring. I'd
suggest putting in at least a dummy RHS and full declarations for
variables and params as usual. Then, before you make the Generator
object, add the switch

DSargs.nobuild = True

to the arguments to the Generator initializer. The object you get back
won't be ready to integrate but, if your Gen is called "odesys" then
you'd do

odesys.makeLibSource()

and you can go edit the .c file, which might be called odesys\_vf.c in
the dopri853\_temp/ directory within your local working dir. Be sure to
follow the same argument list and return through the f\_ vector. If you
read an existing .c vector field file you'll get the idea. Obviously
you can add any other functions and library imports you want to that
file. Once you're finished editing you go back to the python prompt
and run

odesys.compileLib()

and now you can access the RHS function you build directly using
odesys.Rhs() and can call odesys.compute() etc. as usual.

## 3. Questions about documentation

**3.1 : Where is the wiki documentation?**

The wiki server was hosted at Cornell, and crashed in 2010. The data
was not recovered and the server was not restored. As the project is no longer
developed at Cornell, there will be a wait before a new wiki can be
established. In the meantime, a few of the primary wiki pages have
been mirrored at the main website for PyDSTool.

**3.2 : Where is the API documentation?**

Here.
