# Supplementary material for: Hybrid Models and Biological Model Reduction with PyDSTool
Source: PLoS Comput Biol. 2012 Aug 9;8(8):e1002628. doi: 10.1371/journal.pcbi.1002628 (PMC3415397; doi:10.1371/journal.pcbi.1002628)
Supplement: Text S4 — Complete source code for the PyDSTool package (version 0.88.120504). Includes API documentation and help files linking to web pages. This file is identical to the current public release on Sourceforge.net. (ZIP) [file pcbi.1002628.s004.zip › PyDSTool/html/class-tree.html]

xml version="1.0" encoding="ascii"?


Class Hierarchy


| Home | Trees | Indices | Help | | PyDSTool | | --- | |
| --- | --- | --- | --- | --- | --- |

|  |  |  |  |
| --- | --- | --- | --- |
|  | |  | | --- | | [hide private] | | [frames] | no frames] | |

**[ Module Hierarchy
| Class Hierarchy ]**  

# Class Hierarchy

- ***unreachable***
- ***unreachable***
- ***unreachable***
- **PyDSTool.fixedpickle.Pickler**
- **PyDSTool.Toolbox.optimizers.tests.test\_powell.Powell**:
  *The Powell function*
- **PyDSTool.Toolbox.optimizers.tests.test\_quadratic.Quadratic**:
  *A simple quadratic function*
- **PyDSTool.Toolbox.optimizers.tests.test\_rosenbrock.Rosenbrock**:
  *The Rosenbrock function*
- **PyDSTool.fixedpickle.Unpickler**
- **PyDSTool.fixedpickle.\_EmptyClass**
- **PyDSTool.integrator'.integrator**
  - **PyDSTool.Generator.Dopri\_ODEsystem'.dopri**:
    *Dopri 853 specialization of the basic integrator class.*
  - **PyDSTool.Generator.Radau\_ODEsystem'.radau**:
    *Radau 5 specialization of the basic integrator class.*
- **object**:
  *The most base type*
  - **PyDSTool.Toolbox.optimizers.criterion.information\_criteria.AICCriterion**:
    *The Akaike information criterion*
  - **PyDSTool.common.API\_class**:
    *Adapted from .*
  - **PyDSTool.Toolbox.optimizers.criterion.criteria.AbsoluteParametersCriterion**:
    *The absolute criterion stops the optimization when the relative
    error of the parameters is below a certain level (xtol)*
  - **PyDSTool.Toolbox.optimizers.criterion.criteria.AbsoluteValueCriterion**:
    *The absolute criterion stops the optimization when the absolute
    error of the value is below a certain level (ftol)*
  - **PyDSTool.Toolbox.optimizers.line\_search.adaptive\_last\_step\_modifier.AdaptiveLastStepModifier**:
    *Overrides the default step size and replaces it with a factor times
    the last one*
  - **PyDSTool.Toolbox.optimizers.criterion.composite\_criteria.AndComposition**:
    *Compose several criteria with an and rule*
  - **PyDSTool.Toolbox.optimizers.line\_search.backtracking\_search.BacktrackingSearch**:
    *The backtracking algorithm for enforcing Armijo rule*
  - **PyDSTool.common.BarycentricInterpolator**:
    *The interpolating polynomial for a set of points*
  - **exceptions.BaseException**:
    *Common base class for all exceptions*
    - **exceptions.Exception**:
      *Common base class for all non-exit exceptions.*
      - **PyDSTool.Toolbox.FSM.ExceptionFSM**:
        *This is the FSM Exception class.*
      - **PyDSTool.PyCont.misc.IterationError**
      - **PyDSTool.fixedpickle.PickleError**:
        *A common base class for the other pickling exceptions.*
        - **PyDSTool.fixedpickle.PicklingError**:
          *This exception is raised when an unpicklable object is passed to
          the dump() method.*
        - **PyDSTool.fixedpickle.UnpicklingError**:
          *This exception is raised when there is a problem unpickling an
          object, such as a security violation.*
      - **cPickle.PickleError**
        - **cPickle.PicklingError**
          - **cPickle.UnpickleableError**
        - **cPickle.UnpicklingError**
          - **cPickle.BadPickleGet**
      - **PyDSTool.errors.PyDSTool\_Error**
        - **PyDSTool.Toolbox.ParamEst.ConstraintFail**
        - **PyDSTool.Toolbox.ParamEst.Converged**
        - **PyDSTool.errors.PyDSTool\_AttributeError**
        - **PyDSTool.errors.PyDSTool\_BoundsError**
        - **PyDSTool.errors.PyDSTool\_ClearError**
        - **PyDSTool.errors.PyDSTool\_ContError**
        - **PyDSTool.errors.PyDSTool\_ExistError**
        - **PyDSTool.errors.PyDSTool\_InitError**
        - **PyDSTool.errors.PyDSTool\_KeyError**
        - **PyDSTool.errors.PyDSTool\_TypeError**
        - **PyDSTool.errors.PyDSTool\_UncertainValueError**
        - **PyDSTool.errors.PyDSTool\_UndefinedError**
        - **PyDSTool.errors.PyDSTool\_ValueError**
      - **PyDSTool.fixedpickle.\_Stop**
  - **PyDSTool.PyCont.BifPoint.BifPoint**
    - **PyDSTool.PyCont.BifPoint.BPoint**:
      *Special point that represents boundary of computational domain.*
    - **PyDSTool.PyCont.BifPoint.BTPoint**
    - **PyDSTool.PyCont.BifPoint.BranchPoint**:
      *Currently only works for EquilibriumCurve*
    - **PyDSTool.PyCont.BifPoint.CPPoint**
    - **PyDSTool.PyCont.BifPoint.DHPoint**
    - **PyDSTool.PyCont.BifPoint.FoldPoint**
    - **PyDSTool.PyCont.BifPoint.GHPoint**
    - **PyDSTool.PyCont.BifPoint.HopfPoint**
    - **PyDSTool.PyCont.BifPoint.LPCPoint**
    - **PyDSTool.PyCont.BifPoint.NSPoint**
    - **PyDSTool.PyCont.BifPoint.PDPoint**
    - **PyDSTool.PyCont.BifPoint.SPoint**:
      *Special point that represents user-selected free parameter values.*
    - **PyDSTool.PyCont.BifPoint.ZHPoint**
  - **PyDSTool.Toolbox.optimizers.step.conjugate\_gradient\_step.ConjugateGradientStep**:
    *The basic conjugate gradient step*
  - **PyDSTool.PyCont.Continuation.Continuation**:
    *Abstract continuation class*
    - **PyDSTool.PyCont.Continuation.EquilibriumCurve**:
      *Child of Continuation class that represents curves of
      equilibrium points.*
    - **PyDSTool.PyCont.Continuation.FixedPointCurve**
    - **PyDSTool.PyCont.Continuation.FoldCurve**:
      *Child of Continuation class that represents curves of limit
      points.*
    - **PyDSTool.PyCont.Continuation.HopfCurveOne**:
      *Child of Continuation class that represents curves of Hopf
      points.*
    - **PyDSTool.PyCont.Continuation.HopfCurveTwo**:
      *Child of Continuation class that represents curves of Hopf points.*
    - **PyDSTool.PyCont.Continuation.LimitCycleCurve**:
      *Wrapper for auto limit cycle computations.*
    - **PyDSTool.PyCont.Continuation.UserDefinedCurve**:
      *User defined curve.*
  - **PyDSTool.Toolbox.optimizers.line\_search.cubic\_interpolation.CubicInterpolationSearch**:
    *Line Search with the cubic interpolation when the gradient of the
    function is provided*
  - **PyDSTool.Toolbox.optimizers.step.quasi\_newton\_step.DFPNewtonStep**:
    *The Davidson-Fletcher-Powell Quasi-Newton step*
  - **PyDSTool.Toolbox.DSSRT\_tools.DSSRT\_info**
  - **PyDSTool.Toolbox.optimizers.line\_search.damped\_line\_search.DampedLineSearch**:
    *A damped line search, takes a point and a direction.*
  - **PyDSTool.common.Diagnostics**:
    *General purpose diagnostics manager.*
    - **PyDSTool.Variable'.VarDiagnostics**
  - **PyDSTool.common.DomainType**
  - **PyDSTool.ModelConstructor'.EvMapping**:
    *Event mapping class, for use by makeModelInfoEntry and, when
    instantiated, the Model class.*
  - **PyDSTool.Events.Event**:
    *Generic Event.*
    - **PyDSTool.Events.HighLevelEvent**:
      *Event defined using python function code.*
    - **PyDSTool.Events.LowLevelEvent**:
      *Event defined using externally-compiled and linked function code
      (i.e.*
      - **PyDSTool.Events.MatlabEvent**:
        *Event defined using MATLAB syntax for use with ADMC++*
  - **PyDSTool.Events.EventStruct**:
    *A data structure to store and interface with multiple events.*
  - **PyDSTool.Toolbox.event\_driven\_simulator.FIFOqueue\_uniquenode**:
    *Only one entry per node is allowed.*
  - **PyDSTool.Toolbox.FSM.FSM**:
    *This is a Finite State Machine (FSM).*
    - **PyDSTool.Toolbox.FSM.ObjFSM**:
      *A subclass of FSM where input\_symbol may be any kind of object,
      even an unhashable one.*
  - **PyDSTool.Toolbox.optimizers.line\_search.fibonacci\_section.FibonacciSectionSearch**:
    *Line Search with the Fibonacci section method, optimal section
    method*
  - **PyDSTool.Toolbox.optimizers.helpers.finite\_difference.FiniteDifferencesFunction**
    - **PyDSTool.Toolbox.optimizers.helpers.finite\_difference.CenteredFiniteDifferences**:
      *A function that will be able to computes its derivatives with a
      centered difference formula*
    - **PyDSTool.Toolbox.optimizers.helpers.finite\_difference.FiniteDifferencesCache**:
      *General class for recognition by ParamEst as a function with a
      non-explicit derivative.*
      - **PyDSTool.Toolbox.optimizers.helpers.finite\_difference.ForwardFiniteDifferencesCache**:
        *A function that will be able to computes its derivatives with a
        forward difference formula.*
        - **PyDSTool.Toolbox.ParamEst.residual\_fn\_context**
        - **PyDSTool.Toolbox.ParamEst.residual\_fn\_context\_1D**
    - **PyDSTool.Toolbox.optimizers.helpers.finite\_difference.ForwardFiniteDifferences**:
      *A function that will be able to computes its derivatives with a
      forward difference formula*
  - **PyDSTool.Toolbox.optimizers.line\_search.fixed\_last\_step\_modifier.FixedLastStepModifier**:
    *Overrides the default step size and replaces it with a factor times
    the last one*
  - **PyDSTool.FuncSpec'.FuncSpec**:
    *Functional specification of dynamics: abstract class.*
    - **PyDSTool.FuncSpec'.ExpFuncSpec**:
      *Explicit definition of vars defined.*
    - **PyDSTool.FuncSpec'.ImpFuncSpec**:
      *Assumes this will be set to equal zero when solving for vars
      defined.*
    - **PyDSTool.FuncSpec'.RHSfuncSpec**:
      *Right-hand side definition for vars defined.*
  - **PyDSTool.PyCont.TestFunc.Function**:
    *F: R^n --> R^m*
    - **PyDSTool.PyCont.TestFunc.AddTestFunction**:
      *Only works with testfuncs that don't have PreTestFunc and rely only
      on sysfunc.*
    - **PyDSTool.PyCont.TestFunc.DiscreteMap**:
      *Turns a function into a map composed with itself period times.*
    - **PyDSTool.PyCont.TestFunc.FixedPointMap**:
      *Turns a discrete map into a fixed point map.*
    - **PyDSTool.PyCont.TestFunc.TestFunc**:
      *You need to define the function yourself within an inherited class.*
      - **PyDSTool.PyCont.TestFunc.BT\_Fold**
      - **PyDSTool.PyCont.TestFunc.BT\_Hopf**
      - **PyDSTool.PyCont.TestFunc.BT\_Hopf\_One**
      - **PyDSTool.PyCont.TestFunc.B\_Check**:
        *There is an attempt here to be a little efficient.*
      - **PyDSTool.PyCont.TestFunc.BiAltMethod**
        - **PyDSTool.PyCont.TestFunc.Hopf\_Bor**
        - **PyDSTool.PyCont.TestFunc.Hopf\_Det**
        - **PyDSTool.PyCont.TestFunc.Hopf\_Double\_Bor\_One**
        - **PyDSTool.PyCont.TestFunc.NS\_Det**
      - **PyDSTool.PyCont.TestFunc.BorderMethod**:
        *Border method:*
        - **PyDSTool.PyCont.TestFunc.Branch\_Bor**
        - **PyDSTool.PyCont.TestFunc.Fold\_Bor**
        - **PyDSTool.PyCont.TestFunc.Hopf\_Bor**
        - **PyDSTool.PyCont.TestFunc.Hopf\_Double\_Bor\_One**
        - **PyDSTool.PyCont.TestFunc.Hopf\_Double\_Bor\_Two**
      - **PyDSTool.PyCont.TestFunc.Branch\_Det**
      - **PyDSTool.PyCont.TestFunc.CP\_Fold**
      - **PyDSTool.PyCont.TestFunc.DH\_Hopf**
      - **PyDSTool.PyCont.TestFunc.Fold\_Det**
      - **PyDSTool.PyCont.TestFunc.Fold\_Tan**
      - **PyDSTool.PyCont.TestFunc.GH\_Hopf**
      - **PyDSTool.PyCont.TestFunc.GH\_Hopf\_One**
      - **PyDSTool.PyCont.TestFunc.Hopf\_Eig**
      - **PyDSTool.PyCont.TestFunc.LPC\_Det**
      - **PyDSTool.PyCont.TestFunc.PD\_Det**
      - **PyDSTool.PyCont.TestFunc.ParTestFunc**
      - **PyDSTool.PyCont.TestFunc.UserDefinedTestFunc**
  - **PyDSTool.Generator.baseclasses.GenSpecHelper**:
    *Generator specification helper - abstract class.*
  - **PyDSTool.Generator.baseclasses.GenSpecInfoObj**
  - **PyDSTool.MProject.GenTransform**:
    *Generator Transformer class.*
  - **PyDSTool.Generator.baseclasses.Generator**:
    *Trajectory Generator abstract class.*
    - **PyDSTool.Generator.baseclasses.ctsGen**:
      *Abstract class for continuously-parameterized trajectory
      generators.*
      - **PyDSTool.Generator.DDEsystem.DDEsystem**:
        *Delay-differential equations.*
      - **PyDSTool.Generator.EmbeddedSysGen'.EmbeddedSysGen**:
        *Embedded dynamical system form specifying a trajectory.*
      - **PyDSTool.Generator.ExplicitFnGen'.ExplicitFnGen**:
        *Explicit functional form specifying a trajectory.*
      - **PyDSTool.Generator.ExtrapolateTable'.ExtrapolateTable**:
        *Data lookup table with piecewise linear or piecewise constant
        interpolation.*
      - **PyDSTool.Generator.ImplicitFnGen'.ImplicitFnGen**:
        *Implicitly defined functional-form trajectory generator.*
      - **PyDSTool.Generator.InterpolateTable'.InterpolateTable**:
        *Data lookup table with piecewise linear or piecewise constant
        interpolation.*
      - **PyDSTool.Generator.ODEsystem'.ODEsystem**:
        *Abstract class for ODE system solvers.*
        - **PyDSTool.Generator.ADMC\_ODEsystem'.ADMC\_ODEsystem**:
          *Wrapper for code generator for ADMC++32 and Matlab.*
        - **PyDSTool.Generator.Dopri\_ODEsystem'.Dopri\_ODEsystem**:
          *Wrapper for Dopri853 integrator.*
        - **PyDSTool.Generator.Euler\_ODEsystem'.Euler\_ODEsystem**:
          *Euler method.*
        - **PyDSTool.Generator.Radau\_ODEsystem'.Radau\_ODEsystem**:
          *Wrapper for Radau integrator (with support for
          differential-algebraic equations).*
        - **PyDSTool.Generator.Vode\_ODEsystem'.Vode\_ODEsystem**:
          *Wrapper for VODE, from SciPy.*
    - **PyDSTool.Generator.baseclasses.discGen**:
      *Abstract class for discretely-parameterized trajectory generators.*
      - **PyDSTool.Generator.LookupTable'.LookupTable**:
        *Lookup table trajectory with no interpolation.*
      - **PyDSTool.Generator.MapSystem'.MapSystem**:
        *Discrete dynamical systems, as maps (difference equations).*
  - **PyDSTool.ModelConstructor'.GeneratorConstructor**
  - **PyDSTool.Toolbox.optimizers.line\_search.golden\_section.GoldenSectionSearch**:
    *Line Search with the golden section method*
  - **PyDSTool.Toolbox.optimizers.step.goldfeld\_step.GoldfeldStep**:
    *The Goldfeld step*
  - **PyDSTool.Toolbox.optimizers.step.goldstein\_price\_step.GoldsteinPriceStep**:
    *The Goldstein-Price step*
  - **PyDSTool.Toolbox.optimizers.line\_search.goldstein\_rule.GoldsteinRule**:
    *The Goldstein rule for a inexact line search*
  - **PyDSTool.Toolbox.optimizers.criterion.criteria.GradientCriterion**:
    *The gradient criterion stops the optimization when the gradient at
    the current point is less that a given tolerance*
  - **PyDSTool.Toolbox.optimizers.step.gradient\_step.GradientStep**:
    *The simple gradient step*
  - **PyDSTool.Toolbox.optimizers.line\_search.hyperbolic\_line\_search.HyperbolicLineSearch**:
    *An inverse line search, takes a point, adds a step
    (1/(1+iterations)) and returns it*
  - **PyDSTool.scipy\_ode.IntegratorBase**
    - **PyDSTool.scipy\_ode.vode**
  - **PyDSTool.Interval'.Interval**:
    *Numeric Interval class.*
  - **PyDSTool.Toolbox.optimizers.criterion.criteria.IterationCriterion**:
    *A simple criterion that stops when the iteration limit is reached*
  - **PyDSTool.PyCont.Plotting.KeyEvent**:
    *Used in 'highlight' method of plot\_cycles.*
  - **PyDSTool.common.KroghInterpolator**:
    *The interpolating polynomial for a set of points*
  - **PyDSTool.Toolbox.optimizers.step.local\_brute\_force\_1dstep.LocalBruteForce1DStep**:
    *Local brute force search for 1D parameter (sub-)space, making no
    use of gradient information.*
  - **PyDSTool.MProject.MReg**:
    *Registry class for Model descriptors and instances in Model
    projects.*
  - **PyDSTool.Toolbox.optimizers.step.marquardt\_step.MarquardtStep**:
    *The simple gradient step*
  - **PyDSTool.Model.Model**:
    *General-purpose Hybrid and Non-Hybrid Model abstract class.*
    - **PyDSTool.Model.HybridModel**:
      *obsvars specifies the observable variables for this model, which
      must be present in all trajectory segments (regardless of which
      other variables are specified in those segments).*
    - **PyDSTool.Model.NonHybridModel**
  - **PyDSTool.ModelConstructor'.ModelConstructor**
  - **PyDSTool.MProject.ModelLibrary**:
    *Store a set of related candidate model types, and within each,
    represent various relevant "dimensions" along which the
    model can be augmented structurally.*
  - **PyDSTool.MProject.ModelManager**:
    *Model management and repository class.*
  - **PyDSTool.ModelSpec'.ModelSpec**:
    *Model specification abstract class.*
    - **PyDSTool.ModelSpec'.Component**:
      *Non-leaf node sub-class of ModelSpec abstract class.*
      - **PyDSTool.Toolbox.neuralcomp.compatODEComponent**
        - **PyDSTool.Toolbox.neuralcomp.compartment**
          - **PyDSTool.Toolbox.neuralcomp.dendr\_compartment**
          - **PyDSTool.Toolbox.neuralcomp.neurite\_compartment**
          - **PyDSTool.Toolbox.neuralcomp.soma**
        - **PyDSTool.Toolbox.neuralcomp.network**
        - **PyDSTool.Toolbox.neuralcomp.neuron**
        - **PyDSTool.Toolbox.neuralcomp.pnnetwork**
    - **PyDSTool.ModelSpec'.LeafComponent**:
      *Leaf node sub-class of ModelSpec abstract class.*
      - **PyDSTool.Toolbox.NineML.NineMLModel**
      - **PyDSTool.Toolbox.ModelHelper.SpiffyODEModel**
      - **PyDSTool.Toolbox.neuralcomp.compatODELeafComponent**
        - **PyDSTool.Toolbox.neuralcomp.channel**
          - **PyDSTool.Toolbox.neuralcomp.channel\_off**
          - **PyDSTool.Toolbox.neuralcomp.channel\_on**
        - **PyDSTool.Toolbox.neuralcomp.synapse**
          - **PyDSTool.Toolbox.neuralcomp.exc\_synapse**
          - **PyDSTool.Toolbox.neuralcomp.inh\_synapse**
      - **PyDSTool.Toolbox.makeSloppyModel.sloppyModel**
  - **PyDSTool.MProject.ModelTransform**:
    *Model Transformer class.*
  - **PyDSTool.Toolbox.optimizers.criterion.information\_criteria.ModifiedAICCriterion**:
    *The Akaike information criterion with several trials authorized*
  - **PyDSTool.Toolbox.optimizers.criterion.criteria.MonotonyCriterion**:
    *A simple criterion that stops when the values of the function
    starts to rise again*
  - **PyDSTool.Toolbox.optimizers.step.newton\_step.NewtonStep**:
    *The Newton step*
  - **PyDSTool.Toolbox.optimizers.optimizer.optimizer.Optimizer**:
    *The simple optimizer class This class lacks some intel that must be
    populated/implemented in the subclasses :*
    - **PyDSTool.Toolbox.optimizers.optimizer.standard\_optimizer.StandardOptimizer**:
      *A standard optimizer, takes a step and finds the best candidate
      Must give in self.optimalPoint the optimal point after optimization*
    - **PyDSTool.Toolbox.optimizers.optimizer.standard\_optimizer\_modifying.StandardOptimizerModifying**:
      *A standard optimizer, takes a step and finds the best candidate
      Must give in self.optimalPoint the optimal point after optimization
      After each iteration the resulting optimization point is modified
      by a call to a function*
  - **PyDSTool.Toolbox.optimizers.criterion.composite\_criteria.OrComposition**:
    *Compose several criteria with an or rule*
  - **PyDSTool.Variable'.OutputFn**:
    *One-dimensional function wrapper.*
  - **PyDSTool.Toolbox.optimizers.step.partial\_step.PartialStep**:
    *A partial step*
  - **PyDSTool.Points.Point**:
    *N-dimensional point class.*
    - **PyDSTool.Toolbox.phaseplane.Point2D**:
      *Convenience sub-class of PyDSTool.Point for 2D Euclidean points.*
    - **PyDSTool.Points.Pointset**:
      *1D parameterized or non-parameterized set of discrete points.*
  - **PyDSTool.Points.PointInfo**:
    *Structure for storing individual point labels and information
    dictionaries within a Pointset object.*
  - **PyDSTool.Toolbox.optimizers.helpers.quadratic.Quadratic**:
    *Defines a cost function with a quadratic cost*
    - **PyDSTool.Toolbox.optimizers.helpers.levenberg\_marquardt.LMQuadratic**:
      *Defines a cost function with a quadratic cost but the
      Levenberg-Marquardt approximation of the hessian*
  - **PyDSTool.Toolbox.optimizers.line\_search.quadratic\_interpolation.QuadraticInterpolationSearch**:
    *Line Search with the quadratic interpolation when the gradient of
    the function is provided*
  - **PyDSTool.Symbolic.QuantSpec**:
    *Specification for a symbolic numerical quantity.*
  - **PyDSTool.Symbolic.Quantity**:
    *Abstract class for symbolic numerical quantities.*
    - **PyDSTool.Symbolic.Fun**
    - **PyDSTool.Symbolic.Input**
    - **PyDSTool.Symbolic.Par**
    - **PyDSTool.Symbolic.Var**
  - **PyDSTool.Redirector.Redirector**
  - **PyDSTool.Toolbox.optimizers.criterion.criteria.RelativeParametersCriterion**:
    *The relative criterion stops the optimization when the relative
    error of the parameters is below a certain level (xtol)*
  - **PyDSTool.Toolbox.optimizers.criterion.criteria.RelativeValueCriterion**:
    *The relative criterion stops the optimization when the relative
    error of the value is below a certain level (ftol)*
  - **PyDSTool.Toolbox.optimizers.step.restart\_conjugate\_gradient.RestartNotOrthogonalConjugateGradientStep**:
    *A step decorator that deletes the direction key in the dictionary
    if the last gradients are not orthogonal enough so that the CG
    search can be restarted*
  - **PyDSTool.Toolbox.optimizers.step.restart\_conjugate\_gradient.RestartPeriodicallyConjugateGradientStep**:
    *A step decorator that periodically deletes the direction key in the
    dictionary so that the CG search can be restarted*
  - **PyDSTool.Toolbox.optimizers.line\_search.scaled\_line\_search.ScaledLineSearch**:
    *A simple line search, takes a point, adds a step and returns it
    Scales step according to given scales of the parameters and ignores
    \*magnitude\* of gradient.*
  - **PyDSTool.Toolbox.dssrt.Scorer**
    - **PyDSTool.Toolbox.dssrt.EpochSeqScorer**
  - **PyDSTool.Toolbox.optimizers.line\_search.simple\_line\_search.SimpleLineSearch**:
    *A simple line search, takes a point, adds a step and returns it*
  - **PyDSTool.Toolbox.optimizers.line\_search.strong\_wolfe\_powell\_rule.StrongWolfePowellRule**:
    *The strong Wolfe-Powell rule for a inexact line search*
  - **PyDSTool.PyCont.misc.Struct**
  - **PyDSTool.common.Struct**:
    *The args class is a more sophisticated type of Struct.*
  - **PyDSTool.Trajectory'.Trajectory**:
    *Parameterized and non-parameterized trajectory class.*
    - **PyDSTool.Trajectory'.HybridTrajectory**:
      *Hybrid, parameterized, trajectory class.*
  - **PyDSTool.common.Utility**:
    *Utility abstract class for manipulating and analyzing dynamical systems.*
    - **PyDSTool.PyCont.ContClass'.ContClass**:
      *Stores continuation curves for a specified model.*
    - **PyDSTool.Toolbox.ModelEst.ModelEst**:
      *General-purpose model estimation class.*
    - **PyDSTool.Toolbox.ParamEst.ParamEst**:
      *General-purpose parameter estimation class.*
      - **PyDSTool.Toolbox.ParamEst.BoundMin**:
        *Bounded minimization parameter and initial condition optimizer for
        one-dimensional DS trajectories.*
      - **PyDSTool.Toolbox.ParamEst.LMpest**:
        *Unconstrained least-squares parameter and initial condition
        optimizer for n-dimensional DS trajectories.*
  - **PyDSTool.Toolbox.dssrt.VarAlphabet**
  - **PyDSTool.Points.VarCaller**:
    *Wrapper for Variable type to call Pointset and return array type.*
  - **PyDSTool.Variable'.Variable**:
    *One-dimensional discrete and continuous real variable class.*
    - **PyDSTool.Variable'.HybridVariable**:
      *Mimics part of the API of a non-hybrid variable.*
  - **PyDSTool.common.Verbose**:
    *A class to handle reporting.*
  - **PyDSTool.Toolbox.optimizers.line\_search.wolfe\_powell\_rule.WolfePowellRule**:
    *The standard Wolfe-Powell rule for a inexact line search*
  - **PyDSTool.Symbolic.\_mathobj**
  - **PyDSTool.common.args**:
    *Mapping object class for building arguments for class
    initialization calls.*
    - **PyDSTool.ModelConstructor'.Descriptor**:
      *Abstract class for model and generator descriptors*
      - **PyDSTool.ModelConstructor'.GDescriptor**:
        *All-in-one descriptor class for single Generators, and information
        necessary to be able to build a Model object using a
        ModelConstructor call -- i.e.*
      - **PyDSTool.ModelConstructor'.MDescriptor**:
        *All-in-one descriptor class for hybrid model definitions and
        information necessary to be able to build a Model object using a
        ModelConstructor call.*
    - **PyDSTool.PyCont.Plotting.pargs**
  - **PyDSTool.parseUtils.auxfnDBclass**:
    *Auxiliary function database, for use by parsers.*
  - **PyDSTool.Generator.baseclasses.auxfn\_container**:
    *Auxiliary function interface for python user*
  - **PyDSTool.Toolbox.phaseplane.base\_n\_counter**:
    *Simple counter in base-n, using d digits.*
  - **PyDSTool.MProject.condition**:
    *Model context condition, made up of a boolean composition of wanted
    and unwanted features.*
  - **PyDSTool.Toolbox.event\_driven\_simulator.connection**
  - **PyDSTool.MProject.context**:
    *A collection of related model interfaces that apply to a model.*
  - **PyDSTool.Toolbox.FR.cool**
  - **PyDSTool.Toolbox.dataanalysis.data\_bins**:
    *Class for data binning.*
  - **PyDSTool.Toolbox.data\_analysis.data\_bins**:
    *Class for data binning.*
  - **dict**:
    *dict() -> new empty dictionary.*
    - **PyDSTool.common.DefaultDict**:
      *Dictionary with a default value for unknown keys.*
    - **PyDSTool.Generator.baseclasses.ixmap**
  - **PyDSTool.Toolbox.phaseplane.distance\_to\_pointset**:
    *First and second maximum and/or minimum distances of a point q
    to a set of points, returning a dictionary keyed by 'min' and
    'max' to dictionaries keyed by integers 1 and 2 (respectively).*
  - **PyDSTool.Toolbox.dssrt.domscales**
  - **PyDSTool.MProject.dsInterface**:
    *Generic and abstract interface class for dynamical systems.*
    - **PyDSTool.MProject.GeneratorInterface**:
      *Wrapper for Generator (for non-hybrid models) that shares similar
      API with ModelInterface for use in HybridModel objects.*
    - **PyDSTool.MProject.ModelInterface**:
      *Model constraints expressed as a uni-directional interface to another
      formal system model:
      - Made up of conditions imposed on the other system's test trajectory.*
      - **PyDSTool.MProject.extModelInterface**:
        *Interface from a trajectory of numerical data and test conditions
        providing external evaluation criteria for a model.*
      - **PyDSTool.MProject.intModelInterface**:
        *Interface providing internal evaluation criteria between models.*
  - **PyDSTool.Toolbox.dssrt.dssrt\_assistant**
  - **PyDSTool.Toolbox.phaseplane.dx\_scaled\_2D**:
    *Supports a delta x vector that automatically re-scales according to
    the known scalings of each of the vector's component directions.*
  - **PyDSTool.Toolbox.FR.edge**:
    *v -> u directed edge*
  - **PyDSTool.Toolbox.dssrt.epoch**
  - **PyDSTool.Toolbox.neuro\_data.estimate\_spiking**:
    *Estimate pattern of spiking in tonic or burst patterns.*
  - **PyDSTool.Generator.Euler\_ODEsystem'.euler\_solver**
  - **PyDSTool.MProject.feature**:
    *End users of concrete sub-classes provide (required) evaluate
    method and (optional) prepare, finish methods.*
    - **PyDSTool.MProject.feature\_leaf**:
      *Abstract super-class for feature leaf nodes.*
      - **PyDSTool.MProject.ql\_feature\_leaf**:
        *Qualitative feature (leaf node).*
        - **PyDSTool.MProject.always\_feature**:
          *Use this for a single vector field model that uses discrete event
          mappings.*
        - **PyDSTool.MProject.binary\_feature**:
          *Use this as a binary switch feature, toggled by a given variable
          name 'varname' that is supplied in the pars dict at initialization.*
        - **PyDSTool.Toolbox.neuro\_data.get\_spike\_data**:
          *Qualitative test for presence of spike in noisy data.*
        - **PyDSTool.Toolbox.neuro\_data.get\_spike\_model**:
          *Qualitative test for presence of spike in model trajectory data
          using events to identify spike times.*
        - **PyDSTool.Toolbox.phaseplane.zone\_leaf**:
          *Phase plane 'zone' for leaf of hierarchical qualitative feature
          abstract class.*
          - **PyDSTool.Toolbox.phaseplane.nullcline\_zone\_leaf**:
            *Parameters to apply: pars: xtol, refine (integer, default 0)
            optional pars: find\_exact\_center (unused)*
            - **PyDSTool.Toolbox.phaseplane.fixedpoint\_zone**
            - **PyDSTool.Toolbox.phaseplane.inflection\_zone\_leaf**:
              *A single inflection point zone.*
            - **PyDSTool.Toolbox.phaseplane.max\_curvature\_zone\_leaf**:
              *A single zone of locally maximal curvature*
            - **PyDSTool.Toolbox.phaseplane.min\_curvature\_zone**
      - **PyDSTool.MProject.qt\_feature\_leaf**:
        *Quantitative feature (leaf node).*
        - **PyDSTool.Toolbox.ParamEst.L2\_feature**
        - **PyDSTool.Toolbox.ParamEst.L2\_feature\_1D**:
          *Use with scalar optimizers such as BoundMin*
        - **PyDSTool.Model.boundary\_containment**
          - **PyDSTool.Model.boundary\_containment\_by\_event**
          - **PyDSTool.Model.boundary\_containment\_by\_postproc**
        - **PyDSTool.Toolbox.neuro\_data.geom\_feature**:
          *Measures the residual between two 1D parameterized geometric curves
          (given as Trajectory objects).*
        - **PyDSTool.Toolbox.neuro\_data.get\_burst\_active\_phase**
        - **PyDSTool.Toolbox.neuro\_data.get\_burst\_dc\_offset**
        - **PyDSTool.Toolbox.neuro\_data.get\_burst\_downsweep**
        - **PyDSTool.Toolbox.neuro\_data.get\_burst\_duration**
        - **PyDSTool.Toolbox.neuro\_data.get\_burst\_isi\_env**:
          *Requires tol and num\_samples parameters.*
        - **PyDSTool.Toolbox.neuro\_data.get\_burst\_num\_spikes**
        - **PyDSTool.Toolbox.neuro\_data.get\_burst\_passive\_extent**
        - **PyDSTool.Toolbox.neuro\_data.get\_burst\_peak\_env**:
          *Requires tol and num\_samples parameters.*
        - **PyDSTool.Toolbox.neuro\_data.get\_burst\_period\_info**
        - **PyDSTool.Toolbox.neuro\_data.get\_burst\_trough\_env**:
          *Requires tol and num\_samples parameters.*
        - **PyDSTool.Toolbox.neuro\_data.get\_burst\_upsweep**
    - **PyDSTool.MProject.feature\_node**:
      *Abstract super-class for feature regular nodes (supporting
      sub-features).*
      - **PyDSTool.MProject.ql\_feature\_node**:
        *Qualitative feature (regular node).*
        - **PyDSTool.Toolbox.neuro\_data.burst\_feature**:
          *Embed the following sub-features, if desired: get\_burst\_X, where X
          is a number of feature types defined in this module.*
        - **PyDSTool.Toolbox.neuro\_data.get\_burst\_spikes**:
          *Requires a get\_spike\_data and get\_spike\_model instance to be the
          only sub-features (supplied as a dict with keys 'is\_spike\_data' and
          'is\_spike\_model').*
        - **PyDSTool.Toolbox.phaseplane.zone\_node**:
          *Phase plane 'zone' node of hierarchical qualitative feature
          abstract class.*
          - **PyDSTool.Toolbox.phaseplane.nullcline\_zone\_node**
            - **PyDSTool.Toolbox.phaseplane.inflection\_zone\_node**:
              *Find all inflection point zones*
            - **PyDSTool.Toolbox.phaseplane.max\_curvature\_zone\_node**:
              *Find all zones with locally maximal curvature.*
      - **PyDSTool.MProject.qt\_feature\_node**:
        *Quantitative feature (regular node).*
        - **PyDSTool.Model.domain\_test**
        - **PyDSTool.Toolbox.neuro\_data.spike\_feature**:
          *pars keys: tol*
  - **PyDSTool.common.fit\_function**:
    *Abstract super-class for fitting explicit functions to 1D arrays of data
    using least squares.*
    - **PyDSTool.common.fit\_cubic**:
      *Fit a cubic function y=a\*x^3+b\*x^2+c\*x+d to the (x,y) array data.*
    - **PyDSTool.common.fit\_diff\_of\_exp**:
      *Fit a 'difference of two exponentials' function y =
      k\*a\*b\*(exp(-a\*x)-exp(-b\*x))/(b-a) to the (x,y) array data.*
    - **PyDSTool.common.fit\_exponential**:
      *Fit an exponential function y=a\*exp(b\*x) to the (x,y) array data.*
    - **PyDSTool.common.fit\_linear**:
      *Fit a linear function y=a\*x+b to the (x,y) array data.*
    - **PyDSTool.common.fit\_quadratic**:
      *Fit a quadratic function y=a\*x^2+b\*x+c to the (x,y) array data.*
    - **PyDSTool.common.fit\_quadratic\_at\_vertex**:
      *Fit a quadratic function y=a\*(x+h)\*\*2+k to the (x,y) array data,
      constrained to have a vertex at (h, k), leaving only the free
      parameter a for the curvature.*
  - **PyDSTool.Toolbox.phaseplane.fixedpoint\_nD**
    - **PyDSTool.Toolbox.phaseplane.fixedpoint\_2D**
  - **PyDSTool.Generator.baseclasses.genDBClass**:
    *This class keeps a record of which non-Python Generators have been
    created in a session.*
  - **int**:
    *int(x[, base]) -> integer*
    - **PyDSTool.Interval'.IntervalMembership**:
      *Numeric Interval membership type.*
  - **PyDSTool.common.interpclass**:
    *Abstract class for interpolators.*
    - **PyDSTool.common.PiecewisePolynomial**:
      *Piecewise polynomial curve specified by points and derivatives.*
    - **PyDSTool.common.interp0d**:
      *Design of this class based on SciPy's interp1d*
    - **PyDSTool.common.interp1d**
  - **PyDSTool.Toolbox.event\_driven\_simulator.map**
    - **PyDSTool.Toolbox.event\_driven\_simulator.map1D**
      - **PyDSTool.Toolbox.event\_driven\_simulator.composed\_map1D**
      - **PyDSTool.Toolbox.event\_driven\_simulator.delay\_map**
      - **PyDSTool.Toolbox.event\_driven\_simulator.identity\_map**
    - **PyDSTool.Toolbox.event\_driven\_simulator.map2D**
  - **PyDSTool.Toolbox.phaseplane.mesh\_patch\_2D**:
    *2D mesh patch generator (for 4 or 8 points of a fixed distance from
    a central point).*
  - **PyDSTool.common.metric**:
    *Abstract metric class for quantitatively comparing scalar or vector
    quantities.*
    - **PyDSTool.common.metric\_L2**:
      *Measures the standard "distance" between two 1D pointsets
      or arrays using the L-2 norm.*
    - **PyDSTool.common.metric\_L2\_1D**:
      *Measures the standard "distance" between two 1D pointsets
      or arrays using the L-2 norm.*
    - **PyDSTool.common.metric\_float**:
      *Simple metric between two real-valued floats.*
    - **PyDSTool.common.metric\_float\_1D**:
      *Simple metric between two real-valued floats.*
    - **PyDSTool.common.metric\_weighted\_L2**:
      *Measures the standard "distance" between two 1D pointsets
      or arrays using the L-2 norm, after weighting by weights attribute
      (must set weights after creation, e.g.*
    - **PyDSTool.common.metric\_weighted\_deadzone\_L2**:
      *Measures the standard "distance" between two 1D pointsets
      or arrays using the L-2 norm, after weighting by weights attribute.*
    - **PyDSTool.Toolbox.neuro\_data.spike\_metric**:
      *Measures the distance between spike time and height, using an
      inherent weighting of height suited to neural voltage signals (0.05
      of time distance).*
  - **PyDSTool.ModelSpec'.nameResolverClass**:
    *This class keeps a tab of how many times a local name has been used
    for a given specfication type ('var', 'par', 'input' or 'auxfn'),
    and renames it with an appropriate globalized name (hierarchical
    according to declared parent object, with possible numbered suffix
    for multiple name declarations).*
  - **PyDSTool.Toolbox.event\_driven\_simulator.node**
  - **PyDSTool.Toolbox.phaseplane.nullcline**:
    *Nullcline representation class in 2D (x, y) plane parameterizable
    by x variable only.*
  - **PyDSTool.scipy\_ode.ode**:
    *ode - a generic interface class to numeric integrators.*
  - **PyDSTool.parseUtils.parserObject**:
    *Alphanumeric symbol (pseudo-)parser for mathematical expressions.*
  - **PyDSTool.Toolbox.phaseplane.phaseplane**:
    *Working environment for 2D phase-plane analysis.*
  - **PyDSTool.Toolbox.phaseplane.plotter\_2D**:
    *Plotting manager for phase plane analysis.*
  - **PyDSTool.common.predicate**
    - **PyDSTool.Toolbox.dssrt.become\_most\_dominant**
    - **PyDSTool.Toolbox.dssrt.is\_active**
    - **PyDSTool.Toolbox.dssrt.is\_fast**
    - **PyDSTool.Toolbox.dssrt.is\_inactive**
    - **PyDSTool.Toolbox.dssrt.is\_modulatory**
    - **PyDSTool.Toolbox.dssrt.is\_most\_dominant**
    - **PyDSTool.Toolbox.dssrt.is\_order1**
    - **PyDSTool.Toolbox.dssrt.is\_slow**
    - **PyDSTool.Toolbox.dssrt.join\_actives**
    - **PyDSTool.Toolbox.dssrt.join\_fast**
    - **PyDSTool.Toolbox.dssrt.join\_slow**
    - **PyDSTool.Toolbox.dssrt.leave\_actives**
    - **PyDSTool.Toolbox.dssrt.leave\_fast**
    - **PyDSTool.Toolbox.dssrt.leave\_slow**
    - **PyDSTool.common.null\_predicate\_class**
  - **PyDSTool.common.predicate\_op**
    - **PyDSTool.common.and\_op**
    - **PyDSTool.common.not\_op**
    - **PyDSTool.common.or\_op**
  - **PyDSTool.ModelSpec'.regObject**:
    *Registry object container.*
  - **PyDSTool.Toolbox.dssrt.regime**
  - **PyDSTool.Toolbox.event\_driven\_simulator.simulator**:
    *Mapping-based, event-driven simulator for dynamical systems
    reductions.*
  - **PyDSTool.Toolbox.neuro\_data.spike\_envelope**:
    *Find an amplitude envelope over a smooth 1D signal that features
    roughly periodic spikes.*
  - **PyDSTool.parseUtils.symbolMapClass**:
    *Abstract class for hassle-free symbol re-mappings.*
  - **PyDSTool.ModelSpec'.typeCounter**
  - **PyDSTool.Toolbox.FR.vertex**
  - **PyDSTool.Toolbox.test\_protocols.virtual\_experiment**

| Home | Trees | Indices | Help | | PyDSTool | | --- | |
| --- | --- | --- | --- | --- | --- |

|  |  |
| --- | --- |
| Generated by Epydoc 3.0.1 on Fri May 4 15:24:01 2012 | http://epydoc.sourceforge.net |
