# Supplementary material for: Hybrid Models and Biological Model Reduction with PyDSTool
Source: PLoS Comput Biol. 2012 Aug 9;8(8):e1002628. doi: 10.1371/journal.pcbi.1002628 (PMC3415397; doi:10.1371/journal.pcbi.1002628)
Supplement: Text S4 — Complete source code for the PyDSTool package (version 0.88.120504). Includes API documentation and help files linking to web pages. This file is identical to the current public release on Sourceforge.net. (ZIP) [file pcbi.1002628.s004.zip › PyDSTool/html/cPickle-module.html]

xml version="1.0" encoding="ascii"?


cPickle


| Home | Trees | Indices | Help | | PyDSTool | | --- | |
| --- | --- | --- | --- | --- | --- |

|  |  |  |  |
| --- | --- | --- | --- |
| Module cPickle | |  | | --- | | [hide private] | | [frames] | no frames] | |

# Module cPickle

C implementation and optimization of the Python pickle module.

---

**Version:**
1.71


|  |  |  |  |
| --- | --- | --- | --- |
| |  |  | | --- | --- | | Classes | [hide private] | | |
|  | BadPickleGet |
|  | PickleError |
|  | PicklingError |
|  | UnpickleableError |
|  | UnpicklingError |


|  |  |  |  |
| --- | --- | --- | --- |
| |  |  | | --- | --- | | Functions | [hide private] | | |
|  | |  |  | | --- | --- | | Pickler(file, protocol=0)  Create a pickler. |  | |
|  | |  |  | | --- | --- | | Unpickler(file)  Create an unpickler. |  | |
|  | |  |  | | --- | --- | | dump(obj, file, protocol=0)  Write an object in pickle format to the given file. |  | |
|  | |  |  | | --- | --- | | dumps(obj, protocol=0)  Return a string containing an object in pickle format. |  | |
|  | |  |  | | --- | --- | | load(file)  Load a pickle from the given file |  | |
|  | |  |  | | --- | --- | | loads(string)  Load a pickle from the given string |  | |


|  |  |  |  |
| --- | --- | --- | --- |
| |  |  | | --- | --- | | Variables | [hide private] | | |
|  | HIGHEST\_PROTOCOL = `2` |
|  | compatible\_formats = `['1.0', '1.1', '1.2', '1.3', '2.0']` |
|  | format\_version = `'2.0'` |


|  |  |  |  |
| --- | --- | --- | --- |
| |  |  | | --- | --- | | Function Details | [hide private] | | |

|  |  |  |
| --- | --- | --- |
| |  |  | | --- | --- | | Pickler(file, protocol=0) |  |   Create a pickler.  This takes a file-like object for writing a pickle data stream. The optional proto argument tells the pickler to use the given protocol; supported protocols are 0, 1, 2. The default protocol is 0, to be backwards compatible. (Protocol 0 is the only protocol that can be written to a file opened in text mode and read back successfully. When using a protocol higher than 0, make sure the file is opened in binary mode, both when pickling and unpickling.)  Protocol 1 is more efficient than protocol 0; protocol 2 is more efficient than protocol 1.  Specifying a negative protocol version selects the highest protocol version supported. The higher the protocol used, the more recent the version of Python needed to read the pickle produced.  The file parameter must have a write() method that accepts a single string argument. It can thus be an open file object, a StringIO object, or any other custom object that meets this interface. |

|  |  |  |
| --- | --- | --- |
| |  |  | | --- | --- | | dump(obj, file, protocol=0) |  |   Write an object in pickle format to the given file.  See the Pickler docstring for the meaning of optional argument proto. |

|  |  |  |
| --- | --- | --- |
| |  |  | | --- | --- | | dumps(obj, protocol=0) |  |   Return a string containing an object in pickle format.  See the Pickler docstring for the meaning of optional argument proto. |

  


| Home | Trees | Indices | Help | | PyDSTool | | --- | |
| --- | --- | --- | --- | --- | --- |

|  |  |
| --- | --- |
| Generated by Epydoc 3.0.1 on Fri May 4 15:24:06 2012 | http://epydoc.sourceforge.net |
