# Supplementary material for: Hybrid Models and Biological Model Reduction with PyDSTool
Source: PLoS Comput Biol. 2012 Aug 9;8(8):e1002628. doi: 10.1371/journal.pcbi.1002628 (PMC3415397; doi:10.1371/journal.pcbi.1002628)
Supplement: Text S4 — Complete source code for the PyDSTool package (version 0.88.120504). Includes API documentation and help files linking to web pages. This file is identical to the current public release on Sourceforge.net. (ZIP) [file pcbi.1002628.s004.zip › PyDSTool/html/help.html]

xml version="1.0" encoding="ascii"?


Help


| Home | Trees | Indices | Help | | PyDSTool | | --- | |
| --- | --- | --- | --- | --- | --- |

|  |  |  |  |
| --- | --- | --- | --- |
|  | |  | | --- | | [hide private] | | [frames] | no frames] | |

# API Documentation

This document contains the API (Application Programming Interface)
documentation for PyDSTool. Documentation for the Python
objects defined by the project is divided into separate pages for each
package, module, and class. The API documentation also includes two
pages containing information about the project as a whole: a trees
page, and an index page.

## Object Documentation

Each **Package Documentation** page contains:

- A description of the package.
- A list of the modules and sub-packages contained by the
  package.
- A summary of the classes defined by the package.
- A summary of the functions defined by the package.
- A summary of the variables defined by the package.
- A detailed description of each function defined by the
  package.
- A detailed description of each variable defined by the
  package.

Each **Module Documentation** page contains:

- A description of the module.
- A summary of the classes defined by the module.
- A summary of the functions defined by the module.
- A summary of the variables defined by the module.
- A detailed description of each function defined by the
  module.
- A detailed description of each variable defined by the
  module.

Each **Class Documentation** page contains:

- A class inheritance diagram.
- A list of known subclasses.
- A description of the class.
- A summary of the methods defined by the class.
- A summary of the instance variables defined by the class.
- A summary of the class (static) variables defined by the
  class.
- A detailed description of each method defined by the
  class.
- A detailed description of each instance variable defined by the
  class.
- A detailed description of each class (static) variable defined
  by the class.

## Project Documentation

The **Trees** page contains the module and class hierarchies:

- The *module hierarchy* lists every package and module, with
  modules grouped into packages. At the top level, and within each
  package, modules and sub-packages are listed alphabetically.
- The *class hierarchy* lists every class, grouped by base
  class. If a class has more than one base class, then it will be
  listed under each base class. At the top level, and under each base
  class, classes are listed alphabetically.

The **Index** page contains indices of terms and
identifiers:

- The *term index* lists every term indexed by any object's
  documentation. For each term, the index provides links to each
  place where the term is indexed.
- The *identifier index* lists the (short) name of every package,
  module, class, method, function, variable, and parameter. For each
  identifier, the index provides a short description, and a link to
  its documentation.

## The Table of Contents

The table of contents occupies the two frames on the left side of
the window. The upper-left frame displays the *project
contents*, and the lower-left frame displays the *module
contents*:

|  |  |
| --- | --- |
| Project Contents  ---  ... | API Documentation Frame |
| Module Contents  ---    ... |

  

The **project contents frame** contains a list of all packages
and modules that are defined by the project. Clicking on an entry
will display its contents in the module contents frame. Clicking on a
special entry, labeled "Everything," will display the contents of
the entire project.

The **module contents frame** contains a list of every
submodule, class, type, exception, function, and variable defined by a
module or package. Clicking on an entry will display its
documentation in the API documentation frame. Clicking on the name of
the module, at the top of the frame, will display the documentation
for the module itself.

The "**frames**" and "**no frames**" buttons below the top
navigation bar can be used to control whether the table of contents is
displayed or not.

## The Navigation Bar

A navigation bar is located at the top and bottom of every page.
It indicates what type of page you are currently viewing, and allows
you to go to related pages. The following table describes the labels
on the navigation bar. Note that not some labels (such as
[Parent]) are not displayed on all pages.

| Label | Highlighted when... | Links to... |
| --- | --- | --- |
| **[Parent]** | *(never highlighted)* | the parent of the current package |
| **[Package]** | viewing a package | the package containing the current object |
| **[Module]** | viewing a module | the module containing the current object |
| **[Class]** | viewing a class | the class containing the current object |
| **[Trees]** | viewing the trees page | the trees page |
| **[Index]** | viewing the index page | the index page |
| **[Help]** | viewing the help page | the help page |

The "**show private**" and "**hide private**" buttons below
the top navigation bar can be used to control whether documentation
for private objects is displayed. Private objects are usually defined
as objects whose (short) names begin with a single underscore, but do
not end with an underscore. For example, "`_x`",
"`__pprint`", and "`epydoc.epytext._tokenize`"
are private objects; but "`re.sub`",
"`__init__`", and "`type_`" are not. However,
if a module defines the "`__all__`" variable, then its
contents are used to decide which objects are private.

A timestamp below the bottom navigation bar indicates when each
page was last updated.

| Home | Trees | Indices | Help | | PyDSTool | | --- | |
| --- | --- | --- | --- | --- | --- |

|  |  |
| --- | --- |
| Generated by Epydoc 3.0.1 on Fri May 4 15:24:01 2012 | http://epydoc.sourceforge.net |
