# Supplementary material for: Hybrid Models and Biological Model Reduction with PyDSTool
Source: PLoS Comput Biol. 2012 Aug 9;8(8):e1002628. doi: 10.1371/journal.pcbi.1002628 (PMC3415397; doi:10.1371/journal.pcbi.1002628)
Supplement: Text S4 — Complete source code for the PyDSTool package (version 0.88.120504). Includes API documentation and help files linking to web pages. This file is identical to the current public release on Sourceforge.net. (ZIP) [file pcbi.1002628.s004.zip › PyDSTool/html/identifier-index-_.html]

xml version="1.0" encoding="ascii"?


Identifier Index


| Home | Trees | Indices | Help | | PyDSTool | | --- | |
| --- | --- | --- | --- | --- | --- |

|  |  |  |  |
| --- | --- | --- | --- |
|  | |  | | --- | | [hide private] | | [frames] | no frames] | |

|  |  |
| --- | --- |
| Identifier Index | [ A B C D E F G H I J K L M N O P Q R S T U V W X Y Z \_ ] |

|  |  |  |  |  |  |  |  |  |  |  |  |  |  |  |  |  |  |  |  |  |  |  |  |  |  |  |  |  |  |  |  |  |  |  |  |  |  |  |  |  |  |  |  |  |  |  |  |  |  |  |  |  |  |  |  |  |  |  |  |  |  |  |  |  |  |  |  |  |  |  |  |  |  |  |  |  |  |  |  |  |  |  |  |  |  |  |  |  |  |  |  |  |  |  |  |  |  |  |  |  |  |  |  |  |  |  |  |  |  |  |  |  |  |  |  |  |  |  |  |  |  |  |  |  |  |  |  |  |  |  |  |  |  |  |  |  |  |  |  |  |  |  |  |  |  |  |  |  |  |  |  |  |  |  |  |  |  |  |  |  |  |  |  |  |  |  |  |  |  |  |  |  |  |  |  |  |  |  |  |  |  |  |  |  |  |  |  |  |  |  |  |  |  |  |  |  |  |  |  |  |  |  |  |  |  |  |  |  |  |  |  |  |  |  |  |  |  |  |  |  |  |  |  |  |  |  |  |  |  |  |  |  |  |  |  |  |  |  |  |  |  |  |  |  |  |  |  |  |  |  |  |  |  |  |  |  |  |  |  |  |  |  |  |  |  |  |  |  |  |  |  |  |  |  |  |  |  |  |  |  |  |  |  |  |  |  |  |  |  |  |  |  |  |  |  |  |  |  |  |  |  |  |  |  |  |  |  |  |  |  |  |  |  |  |  |  |  |  |  |  |  |  |  |  |  |  |  |  |  |  |  |  |  |  |  |  |  |  |  |  |  |  |  |  |  |  |  |  |  |  |  |  |  |  |  |  |  |  |  |  |  |  |  |  |  |  |  |  |  |  |  |  |  |  |  |  |  |  |  |  |  |  |  |  |  |  |  |  |  |  |  |  |  |  |  |  |  |  |  |  |  |  |  |  |  |  |  |  |  |  |  |  |  |  |  |  |  |  |  |  |  |  |  |  |  |  |  |  |  |  |  |  |  |  |  |  |  |  |  |  |  |  |  |  |  |  |  |  |  |  |  |  |  |  |  |  |  |  |  |  |  |  |  |  |  |  |  |  |  |  |  |  |  |  |  |  |  |  |  |  |  |  |  |  |  |  |  |  |  |  |  |  |  |  |  |  |  |  |  |  |  |  |  |  |  |  |  |  |  |  |  |  |  |  |  |  |  |  |  |  |  |  |  |  |  |  |  |  |  |  |  |  |  |  |  |  |  |  |  |  |  |  |  |  |  |  |  |  |  |  |  |  |  |  |  |  |  |  |  |  |  |  |  |  |  |  |  |  |  |  |  |  |  |  |  |  |  |  |  |  |  |  |  |  |  |  |  |  |  |  |  |  |  |  |  |  |  |  |  |  |  |  |  |  |  |  |  |  |  |  |  |  |  |  |  |  |  |  |  |  |  |  |  |  |  |  |  |  |  |  |  |  |  |  |  |  |  |  |  |  |  |  |  |  |  |  |  |  |  |  |  |  |  |  |  |  |  |  |  |  |  |  |  |  |  |  |  |  |  |  |  |  |  |  |  |  |  |  |  |  |  |  |  |  |  |  |  |  |  |  |  |  |  |  |  |  |  |  |  |  |  |  |  |  |  |  |  |  |  |  |  |  |  |  |  |  |  |  |  |  |  |  |  |  |  |  |  |  |  |  |  |  |  |  |  |  |  |  |  |  |  |  |  |  |  |  |  |  |  |  |  |  |  |  |  |  |  |  |  |  |  |  |  |  |  |  |  |  |  |  |  |  |  |  |  |  |  |  |  |  |  |  |  |  |  |  |  |  |  |  |  |  |  |  |  |  |  |  |  |  |  |  |  |  |  |  |  |  |  |  |  |  |  |  |  |  |  |  |  |  |  |  |  |  |  |  |  |  |  |  |  |  |  |  |  |  |  |  |  |  |  |  |  |  |  |  |  |  |  |  |  |  |  |  |  |  |  |  |  |  |  |  |  |  |  |  |  |  |  |  |  |  |  |  |  |  |  |  |  |  |  |  |  |  |  |  |  |  |  |  |  |  |  |  |  |  |  |  |  |  |  |  |  |  |  |  |  |  |  |  |  |  |  |  |  |  |  |  |  |  |  |  |  |  |  |  |  |  |  |  |  |  |  |  |  |  |  |  |  |  |  |  |  |  |  |  |  |  |  |  |  |  |  |  |  |  |  |  |  |  |  |  |  |  |  |  |  |  |  |  |  |  |  |  |  |  |  |  |  |  |  |  |  |  |  |  |  |  |  |  |  |  |  |  |  |  |  |  |  |  |  |  |  |  |  |  |  |  |  |  |  |  |  |  |  |  |  |  |  |  |  |  |  |  |  |  |  |  |  |  |  |  |  |  |  |  |  |  |  |  |  |  |  |  |  |  |  |  |  |  |  |  |  |  |  |  |  |  |  |  |  |  |  |  |  |  |  |  |  |  |  |  |  |  |  |  |  |  |  |  |  |  |  |  |  |  |  |  |  |  |  |  |  |  |  |  |  |  |  |  |  |  |  |  |  |  |  |  |  |  |  |  |  |  |  |  |  |  |  |  |  |  |  |  |  |  |  |  |  |  |  |  |  |  |  |  |  |  |  |  |  |  |  |  |  |  |  |  |  |  |  |  |  |  |  |  |  |  |  |  |  |  |  |  |  |  |  |  |  |  |  |  |  |  |  |  |  |  |  |  |  |  |  |  |  |  |  |  |  |  |  |  |  |  |  |  |  |  |  |  |  |  |  |  |  |  |  |  |  |  |  |  |  |  |  |  |  |  |  |  |  |  |  |  |  |  |  |  |  |  |  |  |  |  |  |  |  |  |  |  |  |  |  |  |  |  |  |  |  |  |  |  |  |  |  |  |  |  |  |  |  |  |  |  |  |  |  |  |  |  |  |  |  |  |  |  |  |  |  |  |  |  |  |  |  |  |  |  |  |  |  |  |  |  |  |  |  |  |  |  |  |  |  |  |  |  |  |  |  |  |  |  |  |  |  |  |  |  |  |  |  |  |  |  |  |  |  |  |  |  |  |  |  |  |  |  |  |  |  |  |  |  |  |  |  |  |  |  |  |  |  |  |  |  |  |  |  |  |  |  |  |  |  |  |  |  |  |  |  |  |  |  |  |  |  |  |  |  |  |  |  |  |  |  |  |  |  |  |  |  |  |  |  |  |  |  |  |  |  |  |  |  |  |  |  |  |  |  |  |  |  |  |  |  |  |  |  |  |  |  |  |  |  |  |  |  |  |  |  |  |  |  |  |  |  |  |  |  |  |  |  |  |  |  |  |  |  |  |  |  |  |  |  |  |  |  |  |  |  |  |  |  |  |  |  |  |  |  |  |  |  |  |  |  |  |  |  |  |  |  |  |  |  |  |  |  |  |  |  |  |  |  |  |  |  |  |  |  |  |  |  |  |  |  |  |  |  |  |  |  |  |  |  |  |  |  |  |  |  |  |  |  |  |  |  |  |  |  |  |  |  |  |  |  |  |  |  |  |  |  |  |  |  |  |  |  |  |  |  |  |  |  |  |  |  |  |  |  |  |  |  |  |  |  |  |  |  |  |  |  |  |  |  |  |  |  |  |  |  |  |  |  |  |  |  |  |  |  |  |  |  |  |  |  |  |  |  |  |  |  |  |  |  |  |  |  |  |  |  |  |  |  |  |  |  |  |  |  |  |  |  |  |  |  |  |  |  |  |  |  |  |  |  |  |  |  |  |  |  |
| --- | --- | --- | --- | --- | --- | --- | --- | --- | --- | --- | --- | --- | --- | --- | --- | --- | --- | --- | --- | --- | --- | --- | --- | --- | --- | --- | --- | --- | --- | --- | --- | --- | --- | --- | --- | --- | --- | --- | --- | --- | --- | --- | --- | --- | --- | --- | --- | --- | --- | --- | --- | --- | --- | --- | --- | --- | --- | --- | --- | --- | --- | --- | --- | --- | --- | --- | --- | --- | --- | --- | --- | --- | --- | --- | --- | --- | --- | --- | --- | --- | --- | --- | --- | --- | --- | --- | --- | --- | --- | --- | --- | --- | --- | --- | --- | --- | --- | --- | --- | --- | --- | --- | --- | --- | --- | --- | --- | --- | --- | --- | --- | --- | --- | --- | --- | --- | --- | --- | --- | --- | --- | --- | --- | --- | --- | --- | --- | --- | --- | --- | --- | --- | --- | --- | --- | --- | --- | --- | --- | --- | --- | --- | --- | --- | --- | --- | --- | --- | --- | --- | --- | --- | --- | --- | --- | --- | --- | --- | --- | --- | --- | --- | --- | --- | --- | --- | --- | --- | --- | --- | --- | --- | --- | --- | --- | --- | --- | --- | --- | --- | --- | --- | --- | --- | --- | --- | --- | --- | --- | --- | --- | --- | --- | --- | --- | --- | --- | --- | --- | --- | --- | --- | --- | --- | --- | --- | --- | --- | --- | --- | --- | --- | --- | --- | --- | --- | --- | --- | --- | --- | --- | --- | --- | --- | --- | --- | --- | --- | --- | --- | --- | --- | --- | --- | --- | --- | --- | --- | --- | --- | --- | --- | --- | --- | --- | --- | --- | --- | --- | --- | --- | --- | --- | --- | --- | --- | --- | --- | --- | --- | --- | --- | --- | --- | --- | --- | --- | --- | --- | --- | --- | --- | --- | --- | --- | --- | --- | --- | --- | --- | --- | --- | --- | --- | --- | --- | --- | --- | --- | --- | --- | --- | --- | --- | --- | --- | --- | --- | --- | --- | --- | --- | --- | --- | --- | --- | --- | --- | --- | --- | --- | --- | --- | --- | --- | --- | --- | --- | --- | --- | --- | --- | --- | --- | --- | --- | --- | --- | --- | --- | --- | --- | --- | --- | --- | --- | --- | --- | --- | --- | --- | --- | --- | --- | --- | --- | --- | --- | --- | --- | --- | --- | --- | --- | --- | --- | --- | --- | --- | --- | --- | --- | --- | --- | --- | --- | --- | --- | --- | --- | --- | --- | --- | --- | --- | --- | --- | --- | --- | --- | --- | --- | --- | --- | --- | --- | --- | --- | --- | --- | --- | --- | --- | --- | --- | --- | --- | --- | --- | --- | --- | --- | --- | --- | --- | --- | --- | --- | --- | --- | --- | --- | --- | --- | --- | --- | --- | --- | --- | --- | --- | --- | --- | --- | --- | --- | --- | --- | --- | --- | --- | --- | --- | --- | --- | --- | --- | --- | --- | --- | --- | --- | --- | --- | --- | --- | --- | --- | --- | --- | --- | --- | --- | --- | --- | --- | --- | --- | --- | --- | --- | --- | --- | --- | --- | --- | --- | --- | --- | --- | --- | --- | --- | --- | --- | --- | --- | --- | --- | --- | --- | --- | --- | --- | --- | --- | --- | --- | --- | --- | --- | --- | --- | --- | --- | --- | --- | --- | --- | --- | --- | --- | --- | --- | --- | --- | --- | --- | --- | --- | --- | --- | --- | --- | --- | --- | --- | --- | --- | --- | --- | --- | --- | --- | --- | --- | --- | --- | --- | --- | --- | --- | --- | --- | --- | --- | --- | --- | --- | --- | --- | --- | --- | --- | --- | --- | --- | --- | --- | --- | --- | --- | --- | --- | --- | --- | --- | --- | --- | --- | --- | --- | --- | --- | --- | --- | --- | --- | --- | --- | --- | --- | --- | --- | --- | --- | --- | --- | --- | --- | --- | --- | --- | --- | --- | --- | --- | --- | --- | --- | --- | --- | --- | --- | --- | --- | --- | --- | --- | --- | --- | --- | --- | --- | --- | --- | --- | --- | --- | --- | --- | --- | --- | --- | --- | --- | --- | --- | --- | --- | --- | --- | --- | --- | --- | --- | --- | --- | --- | --- | --- | --- | --- | --- | --- | --- | --- | --- | --- | --- | --- | --- | --- | --- | --- | --- | --- | --- | --- | --- | --- | --- | --- | --- | --- | --- | --- | --- | --- | --- | --- | --- | --- | --- | --- | --- | --- | --- | --- | --- | --- | --- | --- | --- | --- | --- | --- | --- | --- | --- | --- | --- | --- | --- | --- | --- | --- | --- | --- | --- | --- | --- | --- | --- | --- | --- | --- | --- | --- | --- | --- | --- | --- | --- | --- | --- | --- | --- | --- | --- | --- | --- | --- | --- | --- | --- | --- | --- | --- | --- | --- | --- | --- | --- | --- | --- | --- | --- | --- | --- | --- | --- | --- | --- | --- | --- | --- | --- | --- | --- | --- | --- | --- | --- | --- | --- | --- | --- | --- | --- | --- | --- | --- | --- | --- | --- | --- | --- | --- | --- | --- | --- | --- | --- | --- | --- | --- | --- | --- | --- | --- | --- | --- | --- | --- | --- | --- | --- | --- | --- | --- | --- | --- | --- | --- | --- | --- | --- | --- | --- | --- | --- | --- | --- | --- | --- | --- | --- | --- | --- | --- | --- | --- | --- | --- | --- | --- | --- | --- | --- | --- | --- | --- | --- | --- | --- | --- | --- | --- | --- | --- | --- | --- | --- | --- | --- | --- | --- | --- | --- | --- | --- | --- | --- | --- | --- | --- | --- | --- | --- | --- | --- | --- | --- | --- | --- | --- | --- | --- | --- | --- | --- | --- | --- | --- | --- | --- | --- | --- | --- | --- | --- | --- | --- | --- | --- | --- | --- | --- | --- | --- | --- | --- | --- | --- | --- | --- | --- | --- | --- | --- | --- | --- | --- | --- | --- | --- | --- | --- | --- | --- | --- | --- | --- | --- | --- | --- | --- | --- | --- | --- | --- | --- | --- | --- | --- | --- | --- | --- | --- | --- | --- | --- | --- | --- | --- | --- | --- | --- | --- | --- | --- | --- | --- | --- | --- | --- | --- | --- | --- | --- | --- | --- | --- | --- | --- | --- | --- | --- | --- | --- | --- | --- | --- | --- | --- | --- | --- | --- | --- | --- | --- | --- | --- | --- | --- | --- | --- | --- | --- | --- | --- | --- | --- | --- | --- | --- | --- | --- | --- | --- | --- | --- | --- | --- | --- | --- | --- | --- | --- | --- | --- | --- | --- | --- | --- | --- | --- | --- | --- | --- | --- | --- | --- | --- | --- | --- | --- | --- | --- | --- | --- | --- | --- | --- | --- | --- | --- | --- | --- | --- | --- | --- | --- | --- | --- | --- | --- | --- | --- | --- | --- | --- | --- | --- | --- | --- | --- | --- | --- | --- | --- | --- | --- | --- | --- | --- | --- | --- | --- | --- | --- | --- | --- | --- | --- | --- | --- | --- | --- | --- | --- | --- | --- | --- | --- | --- | --- | --- | --- | --- | --- | --- | --- | --- | --- | --- | --- | --- | --- | --- | --- | --- | --- | --- | --- | --- | --- | --- | --- | --- | --- | --- | --- | --- | --- | --- | --- | --- | --- | --- | --- | --- | --- | --- | --- | --- | --- | --- | --- | --- | --- | --- | --- | --- | --- | --- | --- | --- | --- | --- | --- | --- | --- | --- | --- | --- | --- | --- | --- | --- | --- | --- | --- | --- | --- | --- | --- | --- | --- | --- | --- | --- | --- | --- | --- | --- | --- | --- | --- | --- | --- | --- | --- | --- | --- | --- | --- | --- | --- | --- | --- | --- | --- | --- | --- | --- | --- | --- | --- | --- | --- | --- | --- | --- | --- | --- | --- | --- | --- | --- | --- | --- | --- | --- | --- | --- | --- | --- | --- | --- | --- | --- | --- | --- | --- | --- | --- | --- | --- | --- | --- | --- | --- | --- | --- | --- | --- | --- | --- | --- | --- | --- | --- | --- | --- | --- | --- | --- | --- | --- | --- | --- | --- | --- | --- | --- | --- | --- | --- | --- | --- | --- | --- | --- | --- | --- | --- | --- | --- | --- | --- | --- | --- | --- | --- | --- | --- | --- | --- | --- | --- | --- | --- | --- | --- | --- | --- | --- | --- | --- | --- | --- | --- | --- | --- | --- | --- | --- | --- | --- | --- | --- | --- | --- | --- | --- | --- | --- | --- | --- | --- | --- | --- | --- | --- | --- | --- | --- | --- | --- | --- | --- | --- | --- | --- | --- | --- | --- | --- | --- | --- | --- | --- | --- | --- | --- | --- | --- | --- | --- | --- | --- | --- | --- | --- | --- | --- | --- | --- | --- | --- | --- | --- | --- | --- | --- | --- | --- | --- | --- | --- | --- | --- | --- | --- | --- | --- | --- | --- | --- | --- | --- | --- | --- | --- | --- | --- | --- | --- | --- | --- | --- | --- | --- | --- | --- | --- | --- | --- | --- | --- | --- | --- | --- | --- | --- | --- | --- | --- | --- | --- | --- | --- | --- | --- | --- | --- | --- | --- | --- | --- | --- | --- | --- | --- | --- | --- | --- | --- | --- | --- | --- | --- | --- | --- | --- | --- | --- | --- | --- | --- | --- | --- | --- | --- | --- | --- | --- | --- | --- | --- | --- | --- | --- | --- | --- | --- | --- | --- | --- | --- | --- | --- | --- | --- | --- | --- | --- | --- | --- | --- | --- | --- | --- | --- | --- | --- | --- | --- | --- | --- | --- | --- | --- | --- | --- | --- | --- | --- | --- | --- | --- | --- | --- | --- | --- | --- | --- | --- | --- | --- | --- | --- | --- | --- | --- | --- | --- | --- | --- | --- | --- | --- | --- | --- | --- | --- | --- | --- | --- | --- | --- | --- | --- | --- | --- | --- | --- | --- | --- | --- | --- | --- | --- | --- | --- | --- | --- | --- | --- | --- | --- | --- | --- | --- | --- | --- | --- | --- | --- | --- | --- | --- | --- | --- | --- | --- | --- | --- | --- | --- | --- | --- | --- | --- | --- | --- | --- | --- | --- | --- | --- | --- | --- | --- | --- | --- | --- | --- | --- | --- | --- | --- | --- | --- | --- | --- | --- | --- | --- | --- | --- | --- | --- | --- | --- | --- | --- | --- | --- | --- | --- | --- | --- | --- | --- | --- | --- | --- | --- | --- | --- | --- | --- | --- | --- | --- | --- | --- | --- | --- | --- | --- | --- | --- | --- | --- | --- | --- | --- | --- | --- | --- | --- | --- | --- | --- | --- | --- | --- | --- | --- | --- | --- | --- | --- | --- | --- | --- | --- | --- | --- | --- | --- | --- | --- | --- | --- | --- | --- | --- | --- | --- | --- | --- | --- | --- | --- | --- | --- | --- | --- | --- | --- | --- | --- | --- | --- | --- | --- | --- | --- | --- | --- | --- | --- | --- | --- |
| \_ | |  |  |  | | --- | --- | --- | | \_1DimplicitSolveMethods  (in PyDSTool.Events) | \_\_lt\_\_()  (in Interval) | \_implicitSolveMethods  (in PyDSTool.Generator.Dopri\_ODEsystem') | | \_1DimplicitSolveMethods  (in PyDSTool.FuncSpec') | \_\_lt\_\_()  (in Point) | \_implicitSolveMethods  (in PyDSTool.Generator.EmbeddedSysGen') | | \_1DimplicitSolveMethods  (in PyDSTool.Generator.ADMC\_ODEsystem') | \_\_lt\_\_()  (in Pointset) | \_implicitSolveMethods  (in PyDSTool.Generator.Euler\_ODEsystem') | | \_1DimplicitSolveMethods  (in PyDSTool.Generator.DDEsystem) | \_\_lt\_\_()  (in Point2D) | \_implicitSolveMethods  (in PyDSTool.Generator.ExplicitFnGen') | | \_1DimplicitSolveMethods  (in PyDSTool.Generator.Dopri\_ODEsystem') | \_\_lt\_\_()  (in args) | \_implicitSolveMethods  (in PyDSTool.Generator.ExtrapolateTable') | | \_1DimplicitSolveMethods  (in PyDSTool.Generator.EmbeddedSysGen') | \_\_max\_grad()  (in mesh\_patch\_2D) | \_implicitSolveMethods  (in PyDSTool.Generator.ImplicitFnGen') | | \_1DimplicitSolveMethods  (in PyDSTool.Generator.Euler\_ODEsystem') | \_\_min\_grad()  (in mesh\_patch\_2D) | \_implicitSolveMethods  (in PyDSTool.Generator.InterpolateTable') | | \_1DimplicitSolveMethods  (in PyDSTool.Generator.ExplicitFnGen') | \_\_mul\_\_()  (in Interval) | \_implicitSolveMethods  (in PyDSTool.Generator.LookupTable') | | \_1DimplicitSolveMethods  (in PyDSTool.Generator.ExtrapolateTable') | \_\_mul\_\_()  (in Point) | \_implicitSolveMethods  (in PyDSTool.Generator.MapSystem') | | \_1DimplicitSolveMethods  (in PyDSTool.Generator.ImplicitFnGen') | \_\_mul\_\_()  (in Pointset) | \_implicitSolveMethods  (in PyDSTool.Generator.ODEsystem') | | \_1DimplicitSolveMethods  (in PyDSTool.Generator.InterpolateTable') | \_\_mul\_\_()  (in QuantSpec) | \_implicitSolveMethods  (in PyDSTool.Generator.Radau\_ODEsystem') | | \_1DimplicitSolveMethods  (in PyDSTool.Generator.LookupTable') | \_\_mul\_\_()  (in Quantity) | \_implicitSolveMethods  (in PyDSTool.Generator.Vode\_ODEsystem') | | \_1DimplicitSolveMethods  (in PyDSTool.Generator.MapSystem') | \_\_mul\_\_()  (in Point2D) | \_implicitSolveMethods  (in PyDSTool.Generator.baseclasses) | | \_1DimplicitSolveMethods  (in PyDSTool.Generator.ODEsystem') | \_\_ne\_\_()  (in Interval) | \_implicitSolveMethods  (in PyDSTool.Interval') | | \_1DimplicitSolveMethods  (in PyDSTool.Generator.Radau\_ODEsystem') | \_\_ne\_\_()  (in condition) | \_implicitSolveMethods  (in PyDSTool.Model) | | \_1DimplicitSolveMethods  (in PyDSTool.Generator.Vode\_ODEsystem') | \_\_ne\_\_()  (in feature) | \_implicitSolveMethods  (in PyDSTool.ModelSpec') | | \_1DimplicitSolveMethods  (in PyDSTool.Generator.baseclasses) | \_\_ne\_\_()  (in ModelSpec) | \_implicitSolveMethods  (in PyDSTool.Points) | | \_1DimplicitSolveMethods  (in PyDSTool.Interval') | \_\_ne\_\_()  (in Point) | \_implicitSolveMethods  (in PyDSTool.Symbolic) | | \_1DimplicitSolveMethods  (in PyDSTool.Model) | \_\_ne\_\_()  (in PointInfo) | \_implicitSolveMethods  (in PyDSTool.Trajectory') | | \_1DimplicitSolveMethods  (in PyDSTool.ModelSpec') | \_\_ne\_\_()  (in Pointset) | \_implicitSolveMethods  (in PyDSTool.Variable') | | \_1DimplicitSolveMethods  (in PyDSTool.Points) | \_\_ne\_\_()  (in QuantSpec) | \_implicitSolveMethods  (in PyDSTool) | | \_1DimplicitSolveMethods  (in PyDSTool.Symbolic) | \_\_ne\_\_()  (in Quantity) | \_implicitSolveMethods  (in PyDSTool.utils) | | \_1DimplicitSolveMethods  (in PyDSTool.Trajectory') | \_\_ne\_\_()  (in DomainType) | \_indentstr  (in PyDSTool.Events) | | \_1DimplicitSolveMethods  (in PyDSTool.Variable') | \_\_ne\_\_()  (in args) | \_indentstr  (in PyDSTool.FuncSpec') | | \_1DimplicitSolveMethods  (in PyDSTool) | \_\_neg\_\_()  (in Interval) | \_indentstr  (in PyDSTool.ModelSpec') | | \_1DimplicitSolveMethods  (in PyDSTool.utils) | \_\_neg\_\_()  (in Point) | \_indentstr  (in PyDSTool.Symbolic) | | \_\_abs\_\_()  (in Point) | \_\_neg\_\_()  (in QuantSpec) | \_indentstr  (in PyDSTool.Trajectory') | | \_\_abs\_\_()  (in Point2D) | \_\_neg\_\_()  (in Quantity) | \_indentstr  (in PyDSTool.parseUtils) | | \_\_add\_\_()  (in Interval) | \_\_neg\_\_()  (in Point2D) | \_infostr()  (in Event) | | \_\_add\_\_()  (in Point) | \_\_neq\_\_()  (in edge) | \_infostr()  (in FuncSpec) | | \_\_add\_\_()  (in Pointset) | \_\_neq\_\_()  (in vertex) | \_infostr()  (in Generator) | | \_\_add\_\_()  (in QuantSpec) | \_\_neq\_\_()  (in symbolMapClass) | \_infostr()  (in Interval) | | \_\_add\_\_()  (in Quantity) | \_\_or\_\_()  (in IntervalMembership) | \_infostr()  (in ModelManager) | | \_\_add\_\_()  (in Point2D) | \_\_pos\_\_()  (in Point) | \_infostr()  (in HybridModel) | | \_\_add\_\_()  (in args) | \_\_pos\_\_()  (in QuantSpec) | \_infostr()  (in Model) | | \_\_and\_\_()  (in IntervalMembership) | \_\_pos\_\_()  (in Quantity) | \_infostr()  (in NonHybridModel) | | \_\_call\_\_()  (in Event) | \_\_pow\_\_()  (in Point) | \_infostr()  (in ModelSpec) | | \_\_call\_\_()  (in EventStruct) | \_\_pow\_\_()  (in QuantSpec) | \_infostr()  (in Point) | | \_\_call\_\_()  (in FuncSpec) | \_\_pow\_\_()  (in Quantity) | \_infostr()  (in PointInfo) | | \_\_call\_\_()  (in GenSpecHelper) | \_\_pow\_\_()  (in Point2D) | \_infostr()  (in Pointset) | | \_\_call\_\_()  (in Interval) | \_\_processTokens()  (in FuncSpec) | \_infostr()  (in epoch) | | \_\_call\_\_()  (in feature) | \_\_radd\_\_()  (in Interval) | \_infostr()  (in Trajectory) | | \_\_call\_\_()  (in Model) | \_\_radd\_\_()  (in Pointset) | \_infostr()  (in Variable) | | \_\_call\_\_()  (in ModelSpec) | \_\_radd\_\_()  (in QuantSpec) | \_infostr()  (in args) | | \_\_call\_\_()  (in nameResolverClass) | \_\_radd\_\_()  (in Quantity) | \_init\_from\_MSpec()  (in dssrt\_assistant) | | \_\_call\_\_()  (in regObject) | \_\_rand\_\_()  (in IntervalMembership) | \_instances  (in PyDSTool.Toolbox.event\_driven\_simulator) | | \_\_call\_\_()  (in Point) | \_\_rdiv\_\_()  (in Interval) | \_instantiate()  (in Unpickler) | | \_\_call\_\_()  (in Pointset) | \_\_rdiv\_\_()  (in Point) | \_int\_types  (in PyDSTool.Events) | | \_\_call\_\_()  (in VarCaller) | \_\_rdiv\_\_()  (in Pointset) | \_int\_types  (in PyDSTool.FuncSpec') | | \_\_call\_\_()  (in KeyEvent) | \_\_rdiv\_\_()  (in QuantSpec) | \_int\_types  (in PyDSTool.Generator.ADMC\_ODEsystem') | | \_\_call\_\_()  (in Function) | \_\_rdiv\_\_()  (in Quantity) | \_int\_types  (in PyDSTool.Generator.DDEsystem) | | \_\_call\_\_()  (in Fun) | \_\_rdiv\_\_()  (in Point2D) | \_int\_types  (in PyDSTool.Generator.Dopri\_ODEsystem') | | \_\_call\_\_()  (in QuantSpec) | \_\_repr\_\_()  (in Event) | \_int\_types  (in PyDSTool.Generator.EmbeddedSysGen') | | \_\_call\_\_()  (in Quantity) | \_\_repr\_\_()  (in FuncSpec) | \_int\_types  (in PyDSTool.Generator.Euler\_ODEsystem') | | \_\_call\_\_()  (in \_mathobj) | \_\_repr\_\_()  (in Generator) | \_int\_types  (in PyDSTool.Generator.ExplicitFnGen') | | \_\_call\_\_()  (in cool) | \_\_repr\_\_()  (in auxfn\_container) | \_int\_types  (in PyDSTool.Generator.ExtrapolateTable') | | \_\_call\_\_()  (in data\_bins) | \_\_repr\_\_()  (in genDBClass) | \_int\_types  (in PyDSTool.Generator.ImplicitFnGen') | | \_\_call\_\_()  (in data\_bins) | \_\_repr\_\_()  (in ixmap) | \_int\_types  (in PyDSTool.Generator.InterpolateTable') | | \_\_call\_\_()  (in composed\_map1D) | \_\_repr\_\_()  (in ModelManager) | \_int\_types  (in PyDSTool.Generator.LookupTable') | | \_\_call\_\_()  (in delay\_map) | \_\_repr\_\_()  (in Model) | \_int\_types  (in PyDSTool.Generator.MapSystem') | | \_\_call\_\_()  (in identity\_map) | \_\_repr\_\_()  (in ModelConstructor) | \_int\_types  (in PyDSTool.Generator.ODEsystem') | | \_\_call\_\_()  (in spike\_metric) | \_\_repr\_\_()  (in nameResolverClass) | \_int\_types  (in PyDSTool.Generator.Radau\_ODEsystem') | | \_\_call\_\_()  (in AndComposition) | \_\_repr\_\_()  (in regObject) | \_int\_types  (in PyDSTool.Generator.Vode\_ODEsystem') | | \_\_call\_\_()  (in OrComposition) | \_\_repr\_\_()  (in Point) | \_int\_types  (in PyDSTool.Generator.baseclasses) | | \_\_call\_\_()  (in AbsoluteParametersCriterion) | \_\_repr\_\_()  (in PointInfo) | \_int\_types  (in PyDSTool.Interval') | | \_\_call\_\_()  (in AbsoluteValueCriterion) | \_\_repr\_\_()  (in Pointset) | \_int\_types  (in PyDSTool.Model) | | \_\_call\_\_()  (in GradientCriterion) | \_\_repr\_\_()  (in Continuation) | \_int\_types  (in PyDSTool.ModelConstructor') | | \_\_call\_\_()  (in IterationCriterion) | \_\_repr\_\_()  (in pargs) | \_int\_types  (in PyDSTool.ModelSpec') | | \_\_call\_\_()  (in MonotonyCriterion) | \_\_repr\_\_()  (in Struct) | \_int\_types  (in PyDSTool.Points) | | \_\_call\_\_()  (in RelativeParametersCriterion) | \_\_repr\_\_()  (in QuantSpec) | \_int\_types  (in PyDSTool.Symbolic) | | \_\_call\_\_()  (in RelativeValueCriterion) | \_\_repr\_\_()  (in Quantity) | \_int\_types  (in PyDSTool.Toolbox.data\_analysis) | | \_\_call\_\_()  (in AICCriterion) | \_\_repr\_\_()  (in \_mathobj) | \_int\_types  (in PyDSTool.Toolbox.dataanalysis) | | \_\_call\_\_()  (in ModifiedAICCriterion) | \_\_repr\_\_()  (in connection) | \_int\_types  (in PyDSTool.Toolbox.event\_driven\_simulator) | | \_\_call\_\_()  (in FiniteDifferencesCache) | \_\_repr\_\_()  (in node) | \_int\_types  (in PyDSTool.Trajectory') | | \_\_call\_\_()  (in ForwardFiniteDifferences) | \_\_repr\_\_()  (in Point2D) | \_int\_types  (in PyDSTool.Variable') | | \_\_call\_\_()  (in Quadratic) | \_\_repr\_\_()  (in mesh\_patch\_2D) | \_int\_types  (in PyDSTool.common) | | \_\_call\_\_()  (in AdaptiveLastStepModifier) | \_\_repr\_\_()  (in DomainType) | \_int\_types  (in PyDSTool.parseUtils) | | \_\_call\_\_()  (in BacktrackingSearch) | \_\_repr\_\_()  (in Struct) | \_int\_types  (in PyDSTool.utils) | | \_\_call\_\_()  (in CubicInterpolationSearch) | \_\_repr\_\_()  (in args) | \_join\_sublist()  (in PyDSTool.Symbolic) | | \_\_call\_\_()  (in DampedLineSearch) | \_\_repr\_\_()  (in PyDSTool\_Error) | \_keep\_alive()  (in PyDSTool.fixedpickle) | | \_\_call\_\_()  (in FibonacciSectionSearch) | \_\_repr\_\_()  (in auxfnDBclass) | \_Keller()  (in Continuation) | | \_\_call\_\_()  (in FixedLastStepModifier) | \_\_repr\_\_()  (in symbolMapClass) | \_keys  (in distance\_to\_pointset) | | \_\_call\_\_()  (in GoldenSectionSearch) | \_\_revision\_\_  (in PyDSTool) | \_kw\_process\_algparams()  (in Generator) | | \_\_call\_\_()  (in GoldsteinRule) | \_\_rmul\_\_()  (in Interval) | \_kw\_process\_allvars()  (in Generator) | | \_\_call\_\_()  (in HyperbolicLineSearch) | \_\_rmul\_\_()  (in Pointset) | \_kw\_process\_dispatch()  (in Generator) | | \_\_call\_\_()  (in QuadraticInterpolationSearch) | \_\_rmul\_\_()  (in QuantSpec) | \_kw\_process\_events()  (in Generator) | | \_\_call\_\_()  (in ScaledLineSearch) | \_\_rmul\_\_()  (in Quantity) | \_kw\_process\_fnspecs()  (in Generator) | | \_\_call\_\_()  (in SimpleLineSearch) | \_\_rmul\_\_()  (in Point2D) | \_kw\_process\_ics()  (in Generator) | | \_\_call\_\_()  (in StrongWolfePowellRule) | \_\_rmul\_\_()  (in dx\_scaled\_2D) | \_kw\_process\_ignorespecial()  (in Generator) | | \_\_call\_\_()  (in WolfePowellRule) | \_\_ror\_\_()  (in IntervalMembership) | \_kw\_process\_inputs()  (in Generator) | | \_\_call\_\_()  (in ConjugateGradientStep) | \_\_rpow\_\_()  (in QuantSpec) | \_kw\_process\_pars()  (in Generator) | | \_\_call\_\_()  (in GoldfeldStep) | \_\_rpow\_\_()  (in Quantity) | \_kw\_process\_pdomain()  (in Generator) | | \_\_call\_\_()  (in GoldsteinPriceStep) | \_\_rsub\_\_()  (in Interval) | \_kw\_process\_reuseterms()  (in Generator) | | \_\_call\_\_()  (in GradientStep) | \_\_rsub\_\_()  (in Point) | \_kw\_process\_system()  (in Generator) | | \_\_call\_\_()  (in LocalBruteForce1DStep) | \_\_rsub\_\_()  (in Pointset) | \_kw\_process\_target()  (in Generator) | | \_\_call\_\_()  (in MarquardtStep) | \_\_rsub\_\_()  (in QuantSpec) | \_kw\_process\_tdata()  (in Generator) | | \_\_call\_\_()  (in NewtonStep) | \_\_rsub\_\_()  (in Quantity) | \_kw\_process\_tdomain()  (in Generator) | | \_\_call\_\_()  (in PartialStep) | \_\_rsub\_\_()  (in Point2D) | \_kw\_process\_tstep()  (in Generator) | | \_\_call\_\_()  (in DFPNewtonStep) | \_\_session\_ext  (in PyDSTool) | \_kw\_process\_ttype()  (in Generator) | | \_\_call\_\_()  (in RestartNotOrthogonalConjugateGradientStep) | \_\_setitem\_\_()  (in EventStruct) | \_kw\_process\_varspecs()  (in Generator) | | \_\_call\_\_()  (in RestartPeriodicallyConjugateGradientStep) | \_\_setitem\_\_()  (in Interval) | \_kw\_process\_vfcodeinserts()  (in Generator) | | \_\_call\_\_()  (in Powell) | \_\_setitem\_\_()  (in MReg) | \_kw\_process\_xdomain()  (in Generator) | | \_\_call\_\_()  (in Quadratic) | \_\_setitem\_\_()  (in typeCounter) | \_kw\_process\_xtype()  (in Generator) | | \_\_call\_\_()  (in Rosenbrock) | \_\_setitem\_\_()  (in Point) | \_local\_init()  (in L2\_feature) | | \_\_call\_\_()  (in distance\_to\_pointset) | \_\_setitem\_\_()  (in PointInfo) | \_local\_init()  (in L2\_feature\_1D) | | \_\_call\_\_()  (in dx\_scaled\_2D) | \_\_setitem\_\_()  (in Pointset) | \_local\_init()  (in burst\_feature) | | \_\_call\_\_()  (in nullcline) | \_\_setitem\_\_()  (in Function) | \_local\_init()  (in geom\_feature) | | \_\_call\_\_()  (in HybridTrajectory) | \_\_setitem\_\_()  (in QuantSpec) | \_local\_init()  (in get\_burst\_active\_phase) | | \_\_call\_\_()  (in Trajectory) | \_\_setitem\_\_()  (in Quantity) | \_local\_init()  (in get\_burst\_dc\_offset) | | \_\_call\_\_()  (in HybridVariable) | \_\_setitem\_\_()  (in data\_bins) | \_local\_init()  (in get\_burst\_downsweep) | | \_\_call\_\_()  (in OutputFn) | \_\_setitem\_\_()  (in data\_bins) | \_local\_init()  (in get\_burst\_duration) | | \_\_call\_\_()  (in Variable) | \_\_setitem\_\_()  (in Scorer) | \_local\_init()  (in get\_burst\_isi\_env) | | \_\_call\_\_()  (in API\_class) | \_\_setitem\_\_()  (in Point2D) | \_local\_init()  (in get\_burst\_num\_spikes) | | \_\_call\_\_()  (in BarycentricInterpolator) | \_\_setitem\_\_()  (in fixedpoint\_nD) | \_local\_init()  (in get\_burst\_passive\_extent) | | \_\_call\_\_()  (in KroghInterpolator) | \_\_setitem\_\_()  (in args) | \_local\_init()  (in get\_burst\_peak\_env) | | \_\_call\_\_()  (in PiecewisePolynomial) | \_\_setitem\_\_()  (in symbolMapClass) | \_local\_init()  (in get\_burst\_period\_info) | | \_\_call\_\_()  (in interp0d) | \_\_setstate\_\_()  (in Event) | \_local\_init()  (in get\_burst\_spikes) | | \_\_call\_\_()  (in interp1d) | \_\_setstate\_\_()  (in MapSystem) | \_local\_init()  (in get\_burst\_trough\_env) | | \_\_call\_\_()  (in metric) | \_\_setstate\_\_()  (in ODEsystem) | \_local\_init()  (in get\_burst\_upsweep) | | \_\_call\_\_()  (in metric\_L2) | \_\_setstate\_\_()  (in Generator) | \_local\_init()  (in get\_spike\_data) | | \_\_call\_\_()  (in metric\_L2\_1D) | \_\_setstate\_\_()  (in Interval) | \_local\_init()  (in spike\_feature) | | \_\_call\_\_()  (in metric\_float) | \_\_setstate\_\_()  (in EvMapping) | \_local\_init()  (in zone\_node) | | \_\_call\_\_()  (in metric\_float\_1D) | \_\_setstate\_\_()  (in Point) | \_localfuncnames  (in PyDSTool.Symbolic) | | \_\_call\_\_()  (in metric\_weighted\_L2) | \_\_setstate\_\_()  (in Pointset) | \_lowlevel()  (in PyDSTool.Events) | | \_\_call\_\_()  (in metric\_weighted\_deadzone\_L2) | \_\_setstate\_\_()  (in dssrt\_assistant) | \_macroFor()  (in FuncSpec) | | \_\_call\_\_()  (in not\_op) | \_\_setstate\_\_()  (in Trajectory) | \_macroSum()  (in FuncSpec) | | \_\_call\_\_()  (in predicate) | \_\_setstate\_\_()  (in HybridVariable) | \_make\_polynomial()  (in PiecewisePolynomial) | | \_\_call\_\_()  (in predicate\_op) | \_\_setstate\_\_()  (in OutputFn) | \_make\_res\_float()  (in LMpest) | | \_\_call\_\_()  (in auxfnDBclass) | \_\_setstate\_\_()  (in Variable) | \_makeBoundsEvents()  (in Generator) | | \_\_call\_\_()  (in parserObject) | \_\_setstate\_\_()  (in PiecewisePolynomial) | \_makeDefaultVarNames()  (in Model) | | \_\_call\_\_()  (in symbolMapClass) | \_\_setstate\_\_()  (in interp0d) | \_makeFilterDict()  (in EventStruct) | | \_\_cmp\_\_()  (in EvMapping) | \_\_setstate\_\_()  (in interp1d) | \_makeUnique()  (in PyDSTool.Toolbox.ParamEst) | | \_\_cmp\_\_()  (in epoch) | \_\_str\_\_()  (in Interval) | \_map\_names\_to\_ixs()  (in Point) | | \_\_cmp\_\_()  (in args) | \_\_str\_\_()  (in IntervalMembership) | \_map\_to\_features()  (in context) | | \_\_combine()  (in QuantSpec) | \_\_str\_\_()  (in ModelLibrary) | \_mappings  (in PyDSTool.common) | | \_\_combine()  (in Quantity) | \_\_str\_\_()  (in condition) | \_mappings  (in PyDSTool.utils) | | \_\_contains\_\_()  (in EventStruct) | \_\_str\_\_()  (in feature\_leaf) | \_match\_indepvararray()  (in Pointset) | | \_\_contains\_\_()  (in GenSpecHelper) | \_\_str\_\_()  (in feature\_node) | \_mathobj  (in PyDSTool.Symbolic) | | \_\_contains\_\_()  (in auxfn\_container) | \_\_str\_\_()  (in Descriptor) | \_mesh5  (in mesh\_patch\_2D) | | \_\_contains\_\_()  (in Interval) | \_\_str\_\_()  (in ModelSpec) | \_mesh9c  (in mesh\_patch\_2D) | | \_\_contains\_\_()  (in MReg) | \_\_str\_\_()  (in regObject) | \_mesh9s  (in mesh\_patch\_2D) | | \_\_contains\_\_()  (in condition) | \_\_str\_\_()  (in Pointset) | \_metric\_utils  (in PyDSTool.Toolbox.data\_analysis) | | \_\_contains\_\_()  (in ModelSpec) | \_\_str\_\_()  (in ContClass) | \_metric\_utils  (in PyDSTool.Toolbox.dataanalysis) | | \_\_contains\_\_()  (in Point) | \_\_str\_\_()  (in Quantity) | \_MoorePenrose()  (in Continuation) | | \_\_contains\_\_()  (in PointInfo) | \_\_str\_\_()  (in \_mathobj) | \_Natural()  (in Continuation) | | \_\_contains\_\_()  (in Pointset) | \_\_str\_\_()  (in ExceptionFSM) | \_needKeys  (in EmbeddedSysGen) | | \_\_contains\_\_()  (in ContClass) | \_\_str\_\_()  (in data\_bins) | \_needKeys  (in ExplicitFnGen) | | \_\_contains\_\_()  (in QuantSpec) | \_\_str\_\_()  (in data\_bins) | \_needKeys  (in ImplicitFnGen) | | \_\_contains\_\_()  (in Quantity) | \_\_str\_\_()  (in epoch) | \_needKeys  (in MapSystem) | | \_\_contains\_\_()  (in Point2D) | \_\_str\_\_()  (in Point2D) | \_needKeys  (in ODEsystem) | | \_\_contains\_\_()  (in args) | \_\_str\_\_()  (in base\_n\_counter) | \_needKeys  (in Generator) | | \_\_contains\_\_()  (in symbolMapClass) | \_\_str\_\_()  (in Trajectory) | \_needKeys  (in Model) | | \_\_copy\_\_()  (in Event) | \_\_str\_\_()  (in HybridVariable) | \_needKeys  (in ModelEst) | | \_\_copy\_\_()  (in Generator) | \_\_str\_\_()  (in Variable) | \_newton\_step()  (in PyDSTool.Toolbox.phaseplane) | | \_\_copy\_\_()  (in Interval) | \_\_str\_\_()  (in PyDSTool\_Error) | \_nonterm()  (in PyDSTool.Events) | | \_\_copy\_\_()  (in Model) | \_\_sub\_\_()  (in Interval) | \_notactive()  (in PyDSTool.Events) | | \_\_copy\_\_()  (in ModelSpec) | \_\_sub\_\_()  (in Point) | \_notprecise()  (in PyDSTool.Events) | | \_\_copy\_\_()  (in Point) | \_\_sub\_\_()  (in Pointset) | \_notvarlinked()  (in PyDSTool.Events) | | \_\_copy\_\_()  (in Pointset) | \_\_sub\_\_()  (in QuantSpec) | \_num\_equivtype  (in PyDSTool.Events) | | \_\_copy\_\_()  (in ContClass) | \_\_sub\_\_()  (in Quantity) | \_num\_equivtype  (in PyDSTool.FuncSpec') | | \_\_copy\_\_()  (in Continuation) | \_\_sub\_\_()  (in Point2D) | \_num\_equivtype  (in PyDSTool.Generator.ADMC\_ODEsystem') | | \_\_copy\_\_()  (in QuantSpec) | \_\_symbolic\_ext  (in PyDSTool) | \_num\_equivtype  (in PyDSTool.Generator.DDEsystem) | | \_\_copy\_\_()  (in Quantity) | \_\_warningregistry\_\_  (in PyDSTool.Generator.Dopri\_ODEsystem') | \_num\_equivtype  (in PyDSTool.Generator.Dopri\_ODEsystem') | | \_\_copy\_\_()  (in Point2D) | \_active()  (in PyDSTool.Events) | \_num\_equivtype  (in PyDSTool.Generator.EmbeddedSysGen') | | \_\_copy\_\_()  (in nullcline) | \_addEvents()  (in Generator) | \_num\_equivtype  (in PyDSTool.Generator.Euler\_ODEsystem') | | \_\_copy\_\_()  (in Trajectory) | \_addTraj()  (in HybridModel) | \_num\_equivtype  (in PyDSTool.Generator.ExplicitFnGen') | | \_\_copy\_\_()  (in Variable) | \_adjust\_ix()  (in PyDSTool.Toolbox.data\_analysis) | \_num\_equivtype  (in PyDSTool.Generator.ExtrapolateTable') | | \_\_copy\_\_()  (in symbolMapClass) | \_adjust\_ix()  (in PyDSTool.Toolbox.dataanalysis) | \_num\_equivtype  (in PyDSTool.Generator.ImplicitFnGen') | | \_\_deepcopy\_\_()  (in Event) | \_all\_complex  (in PyDSTool.Events) | \_num\_equivtype  (in PyDSTool.Generator.InterpolateTable') | | \_\_deepcopy\_\_()  (in EventStruct) | \_all\_complex  (in PyDSTool.FuncSpec') | \_num\_equivtype  (in PyDSTool.Generator.LookupTable') | | \_\_deepcopy\_\_()  (in Generator) | \_all\_complex  (in PyDSTool.Generator.ADMC\_ODEsystem') | \_num\_equivtype  (in PyDSTool.Generator.MapSystem') | | \_\_deepcopy\_\_()  (in Model) | \_all\_complex  (in PyDSTool.Generator.DDEsystem) | \_num\_equivtype  (in PyDSTool.Generator.ODEsystem') | | \_\_deepcopy\_\_()  (in ModelSpec) | \_all\_complex  (in PyDSTool.Generator.Dopri\_ODEsystem') | \_num\_equivtype  (in PyDSTool.Generator.Radau\_ODEsystem') | | \_\_deepcopy\_\_()  (in ContClass) | \_all\_complex  (in PyDSTool.Generator.EmbeddedSysGen') | \_num\_equivtype  (in PyDSTool.Generator.Vode\_ODEsystem') | | \_\_deepcopy\_\_()  (in Continuation) | \_all\_complex  (in PyDSTool.Generator.Euler\_ODEsystem') | \_num\_equivtype  (in PyDSTool.Generator.baseclasses) | | \_\_deepcopy\_\_()  (in QuantSpec) | \_all\_complex  (in PyDSTool.Generator.ExplicitFnGen') | \_num\_equivtype  (in PyDSTool.Interval') | | \_\_deepcopy\_\_()  (in Quantity) | \_all\_complex  (in PyDSTool.Generator.ExtrapolateTable') | \_num\_equivtype  (in PyDSTool.Model) | | \_\_deepcopy\_\_()  (in Trajectory) | \_all\_complex  (in PyDSTool.Generator.ImplicitFnGen') | \_num\_equivtype  (in PyDSTool.ModelConstructor') | | \_\_deepcopy\_\_()  (in Variable) | \_all\_complex  (in PyDSTool.Generator.InterpolateTable') | \_num\_equivtype  (in PyDSTool.ModelSpec') | | \_\_del\_\_()  (in EventStruct) | \_all\_complex  (in PyDSTool.Generator.LookupTable') | \_num\_equivtype  (in PyDSTool.Points) | | \_\_del\_\_()  (in ADMC\_ODEsystem) | \_all\_complex  (in PyDSTool.Generator.MapSystem') | \_num\_equivtype  (in PyDSTool.Symbolic) | | \_\_del\_\_()  (in DDEsystem) | \_all\_complex  (in PyDSTool.Generator.ODEsystem') | \_num\_equivtype  (in PyDSTool.Toolbox.event\_driven\_simulator) | | \_\_del\_\_()  (in Dopri\_ODEsystem) | \_all\_complex  (in PyDSTool.Generator.Radau\_ODEsystem') | \_num\_equivtype  (in PyDSTool.Trajectory') | | \_\_del\_\_()  (in EmbeddedSysGen) | \_all\_complex  (in PyDSTool.Generator.Vode\_ODEsystem') | \_num\_equivtype  (in PyDSTool.Variable') | | \_\_del\_\_()  (in Euler\_ODEsystem) | \_all\_complex  (in PyDSTool.Generator.baseclasses) | \_num\_equivtype  (in PyDSTool.common) | | \_\_del\_\_()  (in ExplicitFnGen) | \_all\_complex  (in PyDSTool.Interval') | \_num\_equivtype  (in PyDSTool.parseUtils) | | \_\_del\_\_()  (in ExtrapolateTable) | \_all\_complex  (in PyDSTool.Model) | \_num\_equivtype  (in PyDSTool.utils) | | \_\_del\_\_()  (in ImplicitFnGen) | \_all\_complex  (in PyDSTool.ModelConstructor') | \_num\_inf  (in PyDSTool.Toolbox.phaseplane) | | \_\_del\_\_()  (in InterpolateTable) | \_all\_complex  (in PyDSTool.ModelSpec') | \_num\_maxmin  (in PyDSTool.Events) | | \_\_del\_\_()  (in LookupTable) | \_all\_complex  (in PyDSTool.Points) | \_num\_maxmin  (in PyDSTool.FuncSpec') | | \_\_del\_\_()  (in MapSystem) | \_all\_complex  (in PyDSTool.Symbolic) | \_num\_maxmin  (in PyDSTool.Generator.ADMC\_ODEsystem') | | \_\_del\_\_()  (in ODEsystem) | \_all\_complex  (in PyDSTool.Toolbox.event\_driven\_simulator) | \_num\_maxmin  (in PyDSTool.Generator.DDEsystem) | | \_\_del\_\_()  (in Radau\_ODEsystem) | \_all\_complex  (in PyDSTool.Trajectory') | \_num\_maxmin  (in PyDSTool.Generator.Dopri\_ODEsystem') | | \_\_del\_\_()  (in Vode\_ODEsystem) | \_all\_complex  (in PyDSTool.Variable') | \_num\_maxmin  (in PyDSTool.Generator.EmbeddedSysGen') | | \_\_del\_\_()  (in Generator) | \_all\_complex  (in PyDSTool.common) | \_num\_maxmin  (in PyDSTool.Generator.Euler\_ODEsystem') | | \_\_del\_\_()  (in ctsGen) | \_all\_complex  (in PyDSTool.parseUtils) | \_num\_maxmin  (in PyDSTool.Generator.ExplicitFnGen') | | \_\_del\_\_()  (in discGen) | \_all\_complex  (in PyDSTool.utils) | \_num\_maxmin  (in PyDSTool.Generator.ExtrapolateTable') | | \_\_del\_\_()  (in GeneratorConstructor) | \_all\_float  (in PyDSTool.Events) | \_num\_maxmin  (in PyDSTool.Generator.ImplicitFnGen') | | \_\_del\_\_()  (in Trajectory) | \_all\_float  (in PyDSTool.FuncSpec') | \_num\_maxmin  (in PyDSTool.Generator.InterpolateTable') | | \_\_del\_\_()  (in HybridVariable) | \_all\_float  (in PyDSTool.Generator.ADMC\_ODEsystem') | \_num\_maxmin  (in PyDSTool.Generator.LookupTable') | | \_\_del\_\_()  (in Variable) | \_all\_float  (in PyDSTool.Generator.DDEsystem) | \_num\_maxmin  (in PyDSTool.Generator.MapSystem') | | \_\_del\_\_()  (in integrator) | \_all\_float  (in PyDSTool.Generator.Dopri\_ODEsystem') | \_num\_maxmin  (in PyDSTool.Generator.ODEsystem') | | \_\_delitem\_\_()  (in EventStruct) | \_all\_float  (in PyDSTool.Generator.EmbeddedSysGen') | \_num\_maxmin  (in PyDSTool.Generator.Radau\_ODEsystem') | | \_\_delitem\_\_()  (in MReg) | \_all\_float  (in PyDSTool.Generator.Euler\_ODEsystem') | \_num\_maxmin  (in PyDSTool.Generator.Vode\_ODEsystem') | | \_\_delitem\_\_()  (in Model) | \_all\_float  (in PyDSTool.Generator.ExplicitFnGen') | \_num\_maxmin  (in PyDSTool.Generator.baseclasses) | | \_\_delitem\_\_()  (in ModelSpec) | \_all\_float  (in PyDSTool.Generator.ExtrapolateTable') | \_num\_maxmin  (in PyDSTool.Interval') | | \_\_delitem\_\_()  (in Point) | \_all\_float  (in PyDSTool.Generator.ImplicitFnGen') | \_num\_maxmin  (in PyDSTool.Model) | | \_\_delitem\_\_()  (in PointInfo) | \_all\_float  (in PyDSTool.Generator.InterpolateTable') | \_num\_maxmin  (in PyDSTool.ModelConstructor') | | \_\_delitem\_\_()  (in Pointset) | \_all\_float  (in PyDSTool.Generator.LookupTable') | \_num\_maxmin  (in PyDSTool.ModelSpec') | | \_\_delitem\_\_()  (in QuantSpec) | \_all\_float  (in PyDSTool.Generator.MapSystem') | \_num\_maxmin  (in PyDSTool.Points) | | \_\_delitem\_\_()  (in Quantity) | \_all\_float  (in PyDSTool.Generator.ODEsystem') | \_num\_maxmin  (in PyDSTool.Symbolic) | | \_\_delitem\_\_()  (in Point2D) | \_all\_float  (in PyDSTool.Generator.Radau\_ODEsystem') | \_num\_maxmin  (in PyDSTool.Toolbox.event\_driven\_simulator) | | \_\_delitem\_\_()  (in args) | \_all\_float  (in PyDSTool.Generator.Vode\_ODEsystem') | \_num\_maxmin  (in PyDSTool.Trajectory') | | \_\_delitem\_\_()  (in symbolMapClass) | \_all\_float  (in PyDSTool.Generator.baseclasses) | \_num\_maxmin  (in PyDSTool.Variable') | | \_\_div\_\_()  (in Interval) | \_all\_float  (in PyDSTool.Interval') | \_num\_maxmin  (in PyDSTool.common) | | \_\_div\_\_()  (in Point) | \_all\_float  (in PyDSTool.Model) | \_num\_maxmin  (in PyDSTool.parseUtils) | | \_\_div\_\_()  (in Pointset) | \_all\_float  (in PyDSTool.ModelConstructor') | \_num\_maxmin  (in PyDSTool.utils) | | \_\_div\_\_()  (in QuantSpec) | \_all\_float  (in PyDSTool.ModelSpec') | \_num\_name2equivtypes  (in PyDSTool.Events) | | \_\_div\_\_()  (in Quantity) | \_all\_float  (in PyDSTool.Points) | \_num\_name2equivtypes  (in PyDSTool.FuncSpec') | | \_\_div\_\_()  (in Point2D) | \_all\_float  (in PyDSTool.Symbolic) | \_num\_name2equivtypes  (in PyDSTool.Generator.ADMC\_ODEsystem') | | \_\_eq\_\_()  (in Interval) | \_all\_float  (in PyDSTool.Toolbox.event\_driven\_simulator) | \_num\_name2equivtypes  (in PyDSTool.Generator.DDEsystem) | | \_\_eq\_\_()  (in condition) | \_all\_float  (in PyDSTool.Trajectory') | \_num\_name2equivtypes  (in PyDSTool.Generator.Dopri\_ODEsystem') | | \_\_eq\_\_()  (in feature) | \_all\_float  (in PyDSTool.Variable') | \_num\_name2equivtypes  (in PyDSTool.Generator.EmbeddedSysGen') | | \_\_eq\_\_()  (in ModelSpec) | \_all\_float  (in PyDSTool.common) | \_num\_name2equivtypes  (in PyDSTool.Generator.Euler\_ODEsystem') | | \_\_eq\_\_()  (in regObject) | \_all\_float  (in PyDSTool.parseUtils) | \_num\_name2equivtypes  (in PyDSTool.Generator.ExplicitFnGen') | | \_\_eq\_\_()  (in Point) | \_all\_float  (in PyDSTool.utils) | \_num\_name2equivtypes  (in PyDSTool.Generator.ExtrapolateTable') | | \_\_eq\_\_()  (in PointInfo) | \_all\_int  (in PyDSTool.Events) | \_num\_name2equivtypes  (in PyDSTool.Generator.ImplicitFnGen') | | \_\_eq\_\_()  (in Pointset) | \_all\_int  (in PyDSTool.FuncSpec') | \_num\_name2equivtypes  (in PyDSTool.Generator.InterpolateTable') | | \_\_eq\_\_()  (in QuantSpec) | \_all\_int  (in PyDSTool.Generator.ADMC\_ODEsystem') | \_num\_name2equivtypes  (in PyDSTool.Generator.LookupTable') | | \_\_eq\_\_()  (in Quantity) | \_all\_int  (in PyDSTool.Generator.DDEsystem) | \_num\_name2equivtypes  (in PyDSTool.Generator.MapSystem') | | \_\_eq\_\_()  (in edge) | \_all\_int  (in PyDSTool.Generator.Dopri\_ODEsystem') | \_num\_name2equivtypes  (in PyDSTool.Generator.ODEsystem') | | \_\_eq\_\_()  (in vertex) | \_all\_int  (in PyDSTool.Generator.EmbeddedSysGen') | \_num\_name2equivtypes  (in PyDSTool.Generator.Radau\_ODEsystem') | | \_\_eq\_\_()  (in Point2D) | \_all\_int  (in PyDSTool.Generator.Euler\_ODEsystem') | \_num\_name2equivtypes  (in PyDSTool.Generator.Vode\_ODEsystem') | | \_\_eq\_\_()  (in DomainType) | \_all\_int  (in PyDSTool.Generator.ExplicitFnGen') | \_num\_name2equivtypes  (in PyDSTool.Generator.baseclasses) | | \_\_eq\_\_()  (in args) | \_all\_int  (in PyDSTool.Generator.ExtrapolateTable') | \_num\_name2equivtypes  (in PyDSTool.Interval') | | \_\_eq\_\_()  (in symbolMapClass) | \_all\_int  (in PyDSTool.Generator.ImplicitFnGen') | \_num\_name2equivtypes  (in PyDSTool.Model) | | \_\_ge\_\_()  (in Interval) | \_all\_int  (in PyDSTool.Generator.InterpolateTable') | \_num\_name2equivtypes  (in PyDSTool.ModelConstructor') | | \_\_ge\_\_()  (in Point) | \_all\_int  (in PyDSTool.Generator.LookupTable') | \_num\_name2equivtypes  (in PyDSTool.ModelSpec') | | \_\_ge\_\_()  (in Pointset) | \_all\_int  (in PyDSTool.Generator.MapSystem') | \_num\_name2equivtypes  (in PyDSTool.Points) | | \_\_ge\_\_()  (in Point2D) | \_all\_int  (in PyDSTool.Generator.ODEsystem') | \_num\_name2equivtypes  (in PyDSTool.Symbolic) | | \_\_ge\_\_()  (in args) | \_all\_int  (in PyDSTool.Generator.Radau\_ODEsystem') | \_num\_name2equivtypes  (in PyDSTool.Toolbox.event\_driven\_simulator) | | \_\_getitem\_\_()  (in EventStruct) | \_all\_int  (in PyDSTool.Generator.Vode\_ODEsystem') | \_num\_name2equivtypes  (in PyDSTool.Trajectory') | | \_\_getitem\_\_()  (in auxfn\_container) | \_all\_int  (in PyDSTool.Generator.baseclasses) | \_num\_name2equivtypes  (in PyDSTool.Variable') | | \_\_getitem\_\_()  (in ixmap) | \_all\_int  (in PyDSTool.Interval') | \_num\_name2equivtypes  (in PyDSTool.common) | | \_\_getitem\_\_()  (in Interval) | \_all\_int  (in PyDSTool.Model) | \_num\_name2equivtypes  (in PyDSTool.parseUtils) | | \_\_getitem\_\_()  (in MReg) | \_all\_int  (in PyDSTool.ModelConstructor') | \_num\_name2equivtypes  (in PyDSTool.utils) | | \_\_getitem\_\_()  (in ModelLibrary) | \_all\_int  (in PyDSTool.ModelSpec') | \_num\_name2type  (in PyDSTool.Events) | | \_\_getitem\_\_()  (in ModelManager) | \_all\_int  (in PyDSTool.Points) | \_num\_name2type  (in PyDSTool.FuncSpec') | | \_\_getitem\_\_()  (in condition) | \_all\_int  (in PyDSTool.Symbolic) | \_num\_name2type  (in PyDSTool.Generator.ADMC\_ODEsystem') | | \_\_getitem\_\_()  (in feature\_node) | \_all\_int  (in PyDSTool.Toolbox.event\_driven\_simulator) | \_num\_name2type  (in PyDSTool.Generator.DDEsystem) | | \_\_getitem\_\_()  (in Model) | \_all\_int  (in PyDSTool.Trajectory') | \_num\_name2type  (in PyDSTool.Generator.Dopri\_ODEsystem') | | \_\_getitem\_\_()  (in GDescriptor) | \_all\_int  (in PyDSTool.Variable') | \_num\_name2type  (in PyDSTool.Generator.EmbeddedSysGen') | | \_\_getitem\_\_()  (in ModelSpec) | \_all\_int  (in PyDSTool.common) | \_num\_name2type  (in PyDSTool.Generator.Euler\_ODEsystem') | | \_\_getitem\_\_()  (in typeCounter) | \_all\_int  (in PyDSTool.integrator') | \_num\_name2type  (in PyDSTool.Generator.ExplicitFnGen') | | \_\_getitem\_\_()  (in PointInfo) | \_all\_int  (in PyDSTool.parseUtils) | \_num\_name2type  (in PyDSTool.Generator.ExtrapolateTable') | | \_\_getitem\_\_()  (in Pointset) | \_all\_int  (in PyDSTool.utils) | \_num\_name2type  (in PyDSTool.Generator.ImplicitFnGen') | | \_\_getitem\_\_()  (in ContClass) | \_all\_numpy\_complex  (in PyDSTool.Events) | \_num\_name2type  (in PyDSTool.Generator.InterpolateTable') | | \_\_getitem\_\_()  (in pargs) | \_all\_numpy\_complex  (in PyDSTool.FuncSpec') | \_num\_name2type  (in PyDSTool.Generator.LookupTable') | | \_\_getitem\_\_()  (in Function) | \_all\_numpy\_complex  (in PyDSTool.Generator.ADMC\_ODEsystem') | \_num\_name2type  (in PyDSTool.Generator.MapSystem') | | \_\_getitem\_\_()  (in QuantSpec) | \_all\_numpy\_complex  (in PyDSTool.Generator.DDEsystem) | \_num\_name2type  (in PyDSTool.Generator.ODEsystem') | | \_\_getitem\_\_()  (in Quantity) | \_all\_numpy\_complex  (in PyDSTool.Generator.Dopri\_ODEsystem') | \_num\_name2type  (in PyDSTool.Generator.Radau\_ODEsystem') | | \_\_getitem\_\_()  (in data\_bins) | \_all\_numpy\_complex  (in PyDSTool.Generator.EmbeddedSysGen') | \_num\_name2type  (in PyDSTool.Generator.Vode\_ODEsystem') | | \_\_getitem\_\_()  (in data\_bins) | \_all\_numpy\_complex  (in PyDSTool.Generator.Euler\_ODEsystem') | \_num\_name2type  (in PyDSTool.Generator.baseclasses) | | \_\_getitem\_\_()  (in EpochSeqScorer) | \_all\_numpy\_complex  (in PyDSTool.Generator.ExplicitFnGen') | \_num\_name2type  (in PyDSTool.Interval') | | \_\_getitem\_\_()  (in Scorer) | \_all\_numpy\_complex  (in PyDSTool.Generator.ExtrapolateTable') | \_num\_name2type  (in PyDSTool.Model) | | \_\_getitem\_\_()  (in Point2D) | \_all\_numpy\_complex  (in PyDSTool.Generator.ImplicitFnGen') | \_num\_name2type  (in PyDSTool.ModelConstructor') | | \_\_getitem\_\_()  (in base\_n\_counter) | \_all\_numpy\_complex  (in PyDSTool.Generator.InterpolateTable') | \_num\_name2type  (in PyDSTool.ModelSpec') | | \_\_getitem\_\_()  (in fixedpoint\_nD) | \_all\_numpy\_complex  (in PyDSTool.Generator.LookupTable') | \_num\_name2type  (in PyDSTool.Points) | | \_\_getitem\_\_()  (in mesh\_patch\_2D) | \_all\_numpy\_complex  (in PyDSTool.Generator.MapSystem') | \_num\_name2type  (in PyDSTool.Symbolic) | | \_\_getitem\_\_()  (in DefaultDict) | \_all\_numpy\_complex  (in PyDSTool.Generator.ODEsystem') | \_num\_name2type  (in PyDSTool.Toolbox.event\_driven\_simulator) | | \_\_getitem\_\_()  (in args) | \_all\_numpy\_complex  (in PyDSTool.Generator.Radau\_ODEsystem') | \_num\_name2type  (in PyDSTool.Trajectory') | | \_\_getitem\_\_()  (in symbolMapClass) | \_all\_numpy\_complex  (in PyDSTool.Generator.Vode\_ODEsystem') | \_num\_name2type  (in PyDSTool.Variable') | | \_\_getstate\_\_()  (in Event) | \_all\_numpy\_complex  (in PyDSTool.Generator.baseclasses) | \_num\_name2type  (in PyDSTool.common) | | \_\_getstate\_\_()  (in MapSystem) | \_all\_numpy\_complex  (in PyDSTool.Interval') | \_num\_name2type  (in PyDSTool.parseUtils) | | \_\_getstate\_\_()  (in ODEsystem) | \_all\_numpy\_complex  (in PyDSTool.Model) | \_num\_name2type  (in PyDSTool.utils) | | \_\_getstate\_\_()  (in Generator) | \_all\_numpy\_complex  (in PyDSTool.ModelConstructor') | \_num\_type2name  (in PyDSTool.Events) | | \_\_getstate\_\_()  (in Interval) | \_all\_numpy\_complex  (in PyDSTool.ModelSpec') | \_num\_type2name  (in PyDSTool.FuncSpec') | | \_\_getstate\_\_()  (in EvMapping) | \_all\_numpy\_complex  (in PyDSTool.Points) | \_num\_type2name  (in PyDSTool.Generator.ADMC\_ODEsystem') | | \_\_getstate\_\_()  (in Point) | \_all\_numpy\_complex  (in PyDSTool.Symbolic) | \_num\_type2name  (in PyDSTool.Generator.DDEsystem) | | \_\_getstate\_\_()  (in Pointset) | \_all\_numpy\_complex  (in PyDSTool.Toolbox.event\_driven\_simulator) | \_num\_type2name  (in PyDSTool.Generator.Dopri\_ODEsystem') | | \_\_getstate\_\_()  (in dssrt\_assistant) | \_all\_numpy\_complex  (in PyDSTool.Trajectory') | \_num\_type2name  (in PyDSTool.Generator.EmbeddedSysGen') | | \_\_getstate\_\_()  (in Trajectory) | \_all\_numpy\_complex  (in PyDSTool.Variable') | \_num\_type2name  (in PyDSTool.Generator.Euler\_ODEsystem') | | \_\_getstate\_\_()  (in HybridVariable) | \_all\_numpy\_complex  (in PyDSTool.common) | \_num\_type2name  (in PyDSTool.Generator.ExplicitFnGen') | | \_\_getstate\_\_()  (in OutputFn) | \_all\_numpy\_complex  (in PyDSTool.parseUtils) | \_num\_type2name  (in PyDSTool.Generator.ExtrapolateTable') | | \_\_getstate\_\_()  (in Variable) | \_all\_numpy\_complex  (in PyDSTool.utils) | \_num\_type2name  (in PyDSTool.Generator.ImplicitFnGen') | | \_\_getstate\_\_()  (in PiecewisePolynomial) | \_all\_numpy\_float  (in PyDSTool.Events) | \_num\_type2name  (in PyDSTool.Generator.InterpolateTable') | | \_\_getstate\_\_()  (in interp0d) | \_all\_numpy\_float  (in PyDSTool.FuncSpec') | \_num\_type2name  (in PyDSTool.Generator.LookupTable') | | \_\_getstate\_\_()  (in interp1d) | \_all\_numpy\_float  (in PyDSTool.Generator.ADMC\_ODEsystem') | \_num\_type2name  (in PyDSTool.Generator.MapSystem') | | \_\_gt\_\_()  (in Interval) | \_all\_numpy\_float  (in PyDSTool.Generator.DDEsystem) | \_num\_type2name  (in PyDSTool.Generator.ODEsystem') | | \_\_gt\_\_()  (in Point) | \_all\_numpy\_float  (in PyDSTool.Generator.Dopri\_ODEsystem') | \_num\_type2name  (in PyDSTool.Generator.Radau\_ODEsystem') | | \_\_gt\_\_()  (in Pointset) | \_all\_numpy\_float  (in PyDSTool.Generator.EmbeddedSysGen') | \_num\_type2name  (in PyDSTool.Generator.Vode\_ODEsystem') | | \_\_gt\_\_()  (in Point2D) | \_all\_numpy\_float  (in PyDSTool.Generator.Euler\_ODEsystem') | \_num\_type2name  (in PyDSTool.Generator.baseclasses) | | \_\_gt\_\_()  (in args) | \_all\_numpy\_float  (in PyDSTool.Generator.ExplicitFnGen') | \_num\_type2name  (in PyDSTool.Interval') | | \_\_hash\_\_()  (in FuncSpec) | \_all\_numpy\_float  (in PyDSTool.Generator.ExtrapolateTable') | \_num\_type2name  (in PyDSTool.Model) | | \_\_hash\_\_()  (in feature) | \_all\_numpy\_float  (in PyDSTool.Generator.ImplicitFnGen') | \_num\_type2name  (in PyDSTool.ModelConstructor') | | \_\_hash\_\_()  (in Quantity) | \_all\_numpy\_float  (in PyDSTool.Generator.InterpolateTable') | \_num\_type2name  (in PyDSTool.ModelSpec') | | \_\_init\_\_()  (in Event) | \_all\_numpy\_float  (in PyDSTool.Generator.LookupTable') | \_num\_type2name  (in PyDSTool.Points) | | \_\_init\_\_()  (in EventStruct) | \_all\_numpy\_float  (in PyDSTool.Generator.MapSystem') | \_num\_type2name  (in PyDSTool.Symbolic) | | \_\_init\_\_()  (in HighLevelEvent) | \_all\_numpy\_float  (in PyDSTool.Generator.ODEsystem') | \_num\_type2name  (in PyDSTool.Toolbox.event\_driven\_simulator) | | \_\_init\_\_()  (in LowLevelEvent) | \_all\_numpy\_float  (in PyDSTool.Generator.Radau\_ODEsystem') | \_num\_type2name  (in PyDSTool.Trajectory') | | \_\_init\_\_()  (in MatlabEvent) | \_all\_numpy\_float  (in PyDSTool.Generator.Vode\_ODEsystem') | \_num\_type2name  (in PyDSTool.Variable') | | \_\_init\_\_()  (in ExpFuncSpec) | \_all\_numpy\_float  (in PyDSTool.Generator.baseclasses) | \_num\_type2name  (in PyDSTool.common) | | \_\_init\_\_()  (in FuncSpec) | \_all\_numpy\_float  (in PyDSTool.Interval') | \_num\_type2name  (in PyDSTool.parseUtils) | | \_\_init\_\_()  (in ImpFuncSpec) | \_all\_numpy\_float  (in PyDSTool.Model) | \_num\_type2name  (in PyDSTool.utils) | | \_\_init\_\_()  (in RHSfuncSpec) | \_all\_numpy\_float  (in PyDSTool.ModelConstructor') | \_num\_types  (in PyDSTool.Events) | | \_\_init\_\_()  (in ADMC\_ODEsystem) | \_all\_numpy\_float  (in PyDSTool.ModelSpec') | \_num\_types  (in PyDSTool.FuncSpec') | | \_\_init\_\_()  (in DDEsystem) | \_all\_numpy\_float  (in PyDSTool.Points) | \_num\_types  (in PyDSTool.Generator.ADMC\_ODEsystem') | | \_\_init\_\_()  (in Dopri\_ODEsystem) | \_all\_numpy\_float  (in PyDSTool.Symbolic) | \_num\_types  (in PyDSTool.Generator.DDEsystem) | | \_\_init\_\_()  (in dopri) | \_all\_numpy\_float  (in PyDSTool.Toolbox.event\_driven\_simulator) | \_num\_types  (in PyDSTool.Generator.Dopri\_ODEsystem') | | \_\_init\_\_()  (in EmbeddedSysGen) | \_all\_numpy\_float  (in PyDSTool.Trajectory') | \_num\_types  (in PyDSTool.Generator.EmbeddedSysGen') | | \_\_init\_\_()  (in Euler\_ODEsystem) | \_all\_numpy\_float  (in PyDSTool.Variable') | \_num\_types  (in PyDSTool.Generator.Euler\_ODEsystem') | | \_\_init\_\_()  (in euler\_solver) | \_all\_numpy\_float  (in PyDSTool.common) | \_num\_types  (in PyDSTool.Generator.ExplicitFnGen') | | \_\_init\_\_()  (in ExplicitFnGen) | \_all\_numpy\_float  (in PyDSTool.parseUtils) | \_num\_types  (in PyDSTool.Generator.ExtrapolateTable') | | \_\_init\_\_()  (in ExtrapolateTable) | \_all\_numpy\_float  (in PyDSTool.utils) | \_num\_types  (in PyDSTool.Generator.ImplicitFnGen') | | \_\_init\_\_()  (in ImplicitFnGen) | \_all\_numpy\_int  (in PyDSTool.Events) | \_num\_types  (in PyDSTool.Generator.InterpolateTable') | | \_\_init\_\_()  (in InterpolateTable) | \_all\_numpy\_int  (in PyDSTool.FuncSpec') | \_num\_types  (in PyDSTool.Generator.LookupTable') | | \_\_init\_\_()  (in LookupTable) | \_all\_numpy\_int  (in PyDSTool.Generator.ADMC\_ODEsystem') | \_num\_types  (in PyDSTool.Generator.MapSystem') | | \_\_init\_\_()  (in MapSystem) | \_all\_numpy\_int  (in PyDSTool.Generator.DDEsystem) | \_num\_types  (in PyDSTool.Generator.ODEsystem') | | \_\_init\_\_()  (in ODEsystem) | \_all\_numpy\_int  (in PyDSTool.Generator.Dopri\_ODEsystem') | \_num\_types  (in PyDSTool.Generator.Radau\_ODEsystem') | | \_\_init\_\_()  (in Radau\_ODEsystem) | \_all\_numpy\_int  (in PyDSTool.Generator.EmbeddedSysGen') | \_num\_types  (in PyDSTool.Generator.Vode\_ODEsystem') | | \_\_init\_\_()  (in radau) | \_all\_numpy\_int  (in PyDSTool.Generator.Euler\_ODEsystem') | \_num\_types  (in PyDSTool.Generator.baseclasses) | | \_\_init\_\_()  (in Vode\_ODEsystem) | \_all\_numpy\_int  (in PyDSTool.Generator.ExplicitFnGen') | \_num\_types  (in PyDSTool.Interval') | | \_\_init\_\_()  (in GenSpecHelper) | \_all\_numpy\_int  (in PyDSTool.Generator.ExtrapolateTable') | \_num\_types  (in PyDSTool.Model) | | \_\_init\_\_()  (in Generator) | \_all\_numpy\_int  (in PyDSTool.Generator.ImplicitFnGen') | \_num\_types  (in PyDSTool.ModelConstructor') | | \_\_init\_\_()  (in auxfn\_container) | \_all\_numpy\_int  (in PyDSTool.Generator.InterpolateTable') | \_num\_types  (in PyDSTool.ModelSpec') | | \_\_init\_\_()  (in genDBClass) | \_all\_numpy\_int  (in PyDSTool.Generator.LookupTable') | \_num\_types  (in PyDSTool.Points) | | \_\_init\_\_()  (in ixmap) | \_all\_numpy\_int  (in PyDSTool.Generator.MapSystem') | \_num\_types  (in PyDSTool.Symbolic) | | \_\_init\_\_()  (in Interval) | \_all\_numpy\_int  (in PyDSTool.Generator.ODEsystem') | \_num\_types  (in PyDSTool.Toolbox.NineML) | | \_\_init\_\_()  (in IntervalMembership) | \_all\_numpy\_int  (in PyDSTool.Generator.Radau\_ODEsystem') | \_num\_types  (in PyDSTool.Toolbox.PySCes\_SBML) | | \_\_init\_\_()  (in GenTransform) | \_all\_numpy\_int  (in PyDSTool.Generator.Vode\_ODEsystem') | \_num\_types  (in PyDSTool.Toolbox.data\_analysis) | | \_\_init\_\_()  (in GeneratorInterface) | \_all\_numpy\_int  (in PyDSTool.Generator.baseclasses) | \_num\_types  (in PyDSTool.Toolbox.dataanalysis) | | \_\_init\_\_()  (in MReg) | \_all\_numpy\_int  (in PyDSTool.Interval') | \_num\_types  (in PyDSTool.Toolbox.event\_driven\_simulator) | | \_\_init\_\_()  (in ModelInterface) | \_all\_numpy\_int  (in PyDSTool.Model) | \_num\_types  (in PyDSTool.Toolbox.phaseplane) | | \_\_init\_\_()  (in ModelLibrary) | \_all\_numpy\_int  (in PyDSTool.ModelConstructor') | \_num\_types  (in PyDSTool.Trajectory') | | \_\_init\_\_()  (in ModelManager) | \_all\_numpy\_int  (in PyDSTool.ModelSpec') | \_num\_types  (in PyDSTool.Variable') | | \_\_init\_\_()  (in ModelTransform) | \_all\_numpy\_int  (in PyDSTool.Points) | \_num\_types  (in PyDSTool.common) | | \_\_init\_\_()  (in condition) | \_all\_numpy\_int  (in PyDSTool.Symbolic) | \_num\_types  (in PyDSTool.parseUtils) | | \_\_init\_\_()  (in context) | \_all\_numpy\_int  (in PyDSTool.Toolbox.event\_driven\_simulator) | \_num\_types  (in PyDSTool.utils) | | \_\_init\_\_()  (in extModelInterface) | \_all\_numpy\_int  (in PyDSTool.Trajectory') | \_objects  (in PyDSTool.ModelSpec') | | \_\_init\_\_()  (in feature) | \_all\_numpy\_int  (in PyDSTool.Variable') | \_objects  (in PyDSTool.parseUtils) | | \_\_init\_\_()  (in feature\_node) | \_all\_numpy\_int  (in PyDSTool.common) | \_optionalKeys  (in EmbeddedSysGen) | | \_\_init\_\_()  (in intModelInterface) | \_all\_numpy\_int  (in PyDSTool.parseUtils) | \_optionalKeys  (in ExplicitFnGen) | | \_\_init\_\_()  (in HybridModel) | \_all\_numpy\_int  (in PyDSTool.utils) | \_optionalKeys  (in ImplicitFnGen) | | \_\_init\_\_()  (in Model) | \_analysis\_utils  (in PyDSTool.Toolbox.data\_analysis) | \_optionalKeys  (in MapSystem) | | \_\_init\_\_()  (in NonHybridModel) | \_analysis\_utils  (in PyDSTool.Toolbox.dataanalysis) | \_optionalKeys  (in ODEsystem) | | \_\_init\_\_()  (in boundary\_containment) | \_applyStateMap()  (in HybridModel) | \_optionalKeys  (in Generator) | | \_\_init\_\_()  (in boundary\_containment\_by\_event) | \_auxfn\_getindex()  (in Generator) | \_optionalKeys  (in Model) | | \_\_init\_\_()  (in domain\_test) | \_auxfn\_getindex()  (in Variable) | \_optionalKeys  (in ModelEst) | | \_\_init\_\_()  (in Descriptor) | \_auxfn\_globalindepvar()  (in Generator) | \_paraminfo  (in Dopri\_ODEsystem) | | \_\_init\_\_()  (in EvMapping) | \_auxfn\_globalindepvar()  (in Variable) | \_paraminfo  (in Radau\_ODEsystem) | | \_\_init\_\_()  (in GeneratorConstructor) | \_auxfn\_heav()  (in Generator) | \_parse\_func\_deriv()  (in PyDSTool.Symbolic) | | \_\_init\_\_()  (in ModelConstructor) | \_auxfn\_heav()  (in Variable) | \_parseReusedTermsPy()  (in FuncSpec) | | \_\_init\_\_()  (in ModelSpec) | \_auxfn\_if()  (in Generator) | \_PCA\_utils  (in PyDSTool.Toolbox.data\_analysis) | | \_\_init\_\_()  (in nameResolverClass) | \_auxfn\_if()  (in Variable) | \_PCA\_utils  (in PyDSTool.Toolbox.dataanalysis) | | \_\_init\_\_()  (in regObject) | \_auxfn\_initcond()  (in Generator) | \_pest\_classes  (in PyDSTool.Toolbox.ParamEst) | | \_\_init\_\_()  (in typeCounter) | \_auxfn\_initcond()  (in Variable) | \_pollInputs()  (in PyDSTool.Generator.baseclasses) | | \_\_init\_\_()  (in Point) | \_avoid\_math\_symbols  (in PyDSTool.Symbolic) | \_precise()  (in PyDSTool.Events) | | \_\_init\_\_()  (in PointInfo) | \_batch\_appends()  (in Pickler) | \_predicates  (in PyDSTool.Toolbox.dssrt) | | \_\_init\_\_()  (in Pointset) | \_batch\_setitems()  (in Pickler) | \_prepare()  (in nullcline\_zone\_leaf) | | \_\_init\_\_()  (in VarCaller) | \_BATCHSIZE  (in Pickler) | \_prepareAuxContents()  (in ADMC\_ODEsystem) | | \_\_init\_\_()  (in BPoint) | \_bialttoeig()  (in HopfCurveOne) | \_prepareCompute()  (in Model) | | \_\_init\_\_()  (in BTPoint) | \_check\_bounds()  (in interp0d) | \_prepareEventFuncStrings()  (in ADMC\_ODEsystem) | | \_\_init\_\_()  (in BifPoint) | \_check\_bounds()  (in interp1d) | \_prepareEventsFileContents()  (in ADMC\_ODEsystem) | | \_\_init\_\_()  (in BranchPoint) | \_check\_self\_ref()  (in QuantSpec) | \_prepareEventSpecs()  (in ADMC\_ODEsystem) | | \_\_init\_\_()  (in CPPoint) | \_checkForBifPoints()  (in Continuation) | \_prepareEventSpecs()  (in Dopri\_ODEsystem) | | \_\_init\_\_()  (in DHPoint) | \_checkKeys  (in Descriptor) | \_prepareEventSpecs()  (in Radau\_ODEsystem) | | \_\_init\_\_()  (in FoldPoint) | \_checkKeys  (in GDescriptor) | \_prepareGetFileContents()  (in ADMC\_ODEsystem) | | \_\_init\_\_()  (in GHPoint) | \_checkKeys  (in MDescriptor) | \_prepareICContents()  (in ADMC\_ODEsystem) | | \_\_init\_\_()  (in HopfPoint) | \_classes  (in PyDSTool.MProject) | \_prepareICs()  (in HybridModel) | | \_\_init\_\_()  (in LPCPoint) | \_classes  (in PyDSTool.ModelSpec') | \_prepareMatlabPDefines()  (in FuncSpec) | | \_\_init\_\_()  (in NSPoint) | \_classes  (in PyDSTool.PyCont.BifPoint) | \_prepareMatlabVDefines()  (in FuncSpec) | | \_\_init\_\_()  (in PDPoint) | \_classes  (in PyDSTool.PyCont.ContClass') | \_prepareModelContents()  (in ADMC\_ODEsystem) | | \_\_init\_\_()  (in SPoint) | \_classes  (in PyDSTool.PyCont.Continuation) | \_prepareParamContents()  (in ADMC\_ODEsystem) | | \_\_init\_\_()  (in ZHPoint) | \_classes  (in PyDSTool.PyCont.Plotting) | \_prepareSetFileContents()  (in ADMC\_ODEsystem) | | \_\_init\_\_()  (in ContClass) | \_classes  (in PyDSTool.PyCont.TestFunc) | \_prepareVfieldContents()  (in ADMC\_ODEsystem) | | \_\_init\_\_()  (in Continuation) | \_classes  (in PyDSTool.PyCont.misc) | \_prepareVfieldDefines()  (in ADMC\_ODEsystem) | | \_\_init\_\_()  (in EquilibriumCurve) | \_classes  (in PyDSTool.Symbolic) | \_present\_and\_sensitive()  (in PyDSTool.Toolbox.ParamEst) | | \_\_init\_\_()  (in FixedPointCurve) | \_classes  (in PyDSTool.Toolbox.NineML) | \_preTestFunc()  (in Continuation) | | \_\_init\_\_()  (in FoldCurve) | \_classes  (in PyDSTool.Toolbox.PySCes\_SBML) | \_preTestFunc()  (in FixedPointCurve) | | \_\_init\_\_()  (in HopfCurveOne) | \_classes  (in PyDSTool.Toolbox.dssrt) | \_pretty\_print\_label()  (in PyDSTool.Points) | | \_\_init\_\_()  (in HopfCurveTwo) | \_classes  (in PyDSTool.Toolbox.event\_driven\_simulator) | \_print\_values()  (in API\_class) | | \_\_init\_\_()  (in LimitCycleCurve) | \_classes  (in PyDSTool.Toolbox.phaseplane) | \_process\_expr()  (in dssrt\_assistant) | | \_\_init\_\_()  (in UserDefinedCurve) | \_classes  (in PyDSTool.common) | \_processIfMatlab()  (in FuncSpec) | | \_\_init\_\_()  (in KeyEvent) | \_classes  (in PyDSTool.parseUtils) | \_processReused()  (in PyDSTool.FuncSpec') | | \_\_init\_\_()  (in AddTestFunction) | \_classes  (in PyDSTool.utils) | \_processReusedC()  (in FuncSpec) | | \_\_init\_\_()  (in BT\_Fold) | \_classifcations  (in fixedpoint\_nD) | \_processReusedMatlab()  (in FuncSpec) | | \_\_init\_\_()  (in BT\_Hopf) | \_classify()  (in fixedpoint\_2D) | \_processReusedPy()  (in FuncSpec) | | \_\_init\_\_()  (in BT\_Hopf\_One) | \_classify()  (in fixedpoint\_nD) | \_processSpecialC()  (in FuncSpec) | | \_\_init\_\_()  (in B\_Check) | \_commandLineVerbose  (in Verbose) | \_propagate\_str\_eval()  (in PyDSTool.Symbolic) | | \_\_init\_\_()  (in BiAltMethod) | \_comp()  (in PyDSTool.Toolbox.dssrt) | \_pydstool\_path  (in PyDSTool.Generator.ADMC\_ODEsystem') | | \_\_init\_\_()  (in BorderMethod) | \_complex\_types  (in PyDSTool.Events) | \_pydstool\_path  (in PyDSTool.Generator.Dopri\_ODEsystem') | | \_\_init\_\_()  (in Branch\_Bor) | \_complex\_types  (in PyDSTool.FuncSpec') | \_pydstool\_path  (in PyDSTool.Generator.Radau\_ODEsystem') | | \_\_init\_\_()  (in Branch\_Det) | \_complex\_types  (in PyDSTool.Generator.ADMC\_ODEsystem') | \_pydstool\_path  (in PyDSTool.PyCont.ContClass') | | \_\_init\_\_()  (in CP\_Fold) | \_complex\_types  (in PyDSTool.Generator.DDEsystem) | \_pyDSToolTypes  (in PyDSTool) | | \_\_init\_\_()  (in DH\_Hopf) | \_complex\_types  (in PyDSTool.Generator.Dopri\_ODEsystem') | \_pytypefromtype  (in PyDSTool.Events) | | \_\_init\_\_()  (in DiscreteMap) | \_complex\_types  (in PyDSTool.Generator.EmbeddedSysGen') | \_pytypefromtype  (in PyDSTool.FuncSpec') | | \_\_init\_\_()  (in FixedPointMap) | \_complex\_types  (in PyDSTool.Generator.Euler\_ODEsystem') | \_pytypefromtype  (in PyDSTool.Generator.ADMC\_ODEsystem') | | \_\_init\_\_()  (in Fold\_Bor) | \_complex\_types  (in PyDSTool.Generator.ExplicitFnGen') | \_pytypefromtype  (in PyDSTool.Generator.DDEsystem) | | \_\_init\_\_()  (in Fold\_Det) | \_complex\_types  (in PyDSTool.Generator.ExtrapolateTable') | \_pytypefromtype  (in PyDSTool.Generator.Dopri\_ODEsystem') | | \_\_init\_\_()  (in Fold\_Tan) | \_complex\_types  (in PyDSTool.Generator.ImplicitFnGen') | \_pytypefromtype  (in PyDSTool.Generator.EmbeddedSysGen') | | \_\_init\_\_()  (in Function) | \_complex\_types  (in PyDSTool.Generator.InterpolateTable') | \_pytypefromtype  (in PyDSTool.Generator.Euler\_ODEsystem') | | \_\_init\_\_()  (in GH\_Hopf) | \_complex\_types  (in PyDSTool.Generator.LookupTable') | \_pytypefromtype  (in PyDSTool.Generator.ExplicitFnGen') | | \_\_init\_\_()  (in GH\_Hopf\_One) | \_complex\_types  (in PyDSTool.Generator.MapSystem') | \_pytypefromtype  (in PyDSTool.Generator.ExtrapolateTable') | | \_\_init\_\_()  (in Hopf\_Bor) | \_complex\_types  (in PyDSTool.Generator.ODEsystem') | \_pytypefromtype  (in PyDSTool.Generator.ImplicitFnGen') | | \_\_init\_\_()  (in Hopf\_Det) | \_complex\_types  (in PyDSTool.Generator.Radau\_ODEsystem') | \_pytypefromtype  (in PyDSTool.Generator.InterpolateTable') | | \_\_init\_\_()  (in Hopf\_Double\_Bor\_One) | \_complex\_types  (in PyDSTool.Generator.Vode\_ODEsystem') | \_pytypefromtype  (in PyDSTool.Generator.LookupTable') | | \_\_init\_\_()  (in Hopf\_Double\_Bor\_Two) | \_complex\_types  (in PyDSTool.Generator.baseclasses) | \_pytypefromtype  (in PyDSTool.Generator.MapSystem') | | \_\_init\_\_()  (in Hopf\_Eig) | \_complex\_types  (in PyDSTool.Interval') | \_pytypefromtype  (in PyDSTool.Generator.ODEsystem') | | \_\_init\_\_()  (in LPC\_Det) | \_complex\_types  (in PyDSTool.Model) | \_pytypefromtype  (in PyDSTool.Generator.Radau\_ODEsystem') | | \_\_init\_\_()  (in NS\_Det) | \_complex\_types  (in PyDSTool.ModelConstructor') | \_pytypefromtype  (in PyDSTool.Generator.Vode\_ODEsystem') | | \_\_init\_\_()  (in PD\_Det) | \_complex\_types  (in PyDSTool.ModelSpec') | \_pytypefromtype  (in PyDSTool.Generator.baseclasses) | | \_\_init\_\_()  (in ParTestFunc) | \_complex\_types  (in PyDSTool.Points) | \_pytypefromtype  (in PyDSTool.Interval') | | \_\_init\_\_()  (in TestFunc) | \_complex\_types  (in PyDSTool.Symbolic) | \_pytypefromtype  (in PyDSTool.Model) | | \_\_init\_\_()  (in UserDefinedTestFunc) | \_complex\_types  (in PyDSTool.Toolbox.event\_driven\_simulator) | \_pytypefromtype  (in PyDSTool.ModelConstructor') | | \_\_init\_\_()  (in Struct) | \_complex\_types  (in PyDSTool.Trajectory') | \_pytypefromtype  (in PyDSTool.ModelSpec') | | \_\_init\_\_()  (in Redirector) | \_complex\_types  (in PyDSTool.Variable') | \_pytypefromtype  (in PyDSTool.Points) | | \_\_init\_\_()  (in Fun) | \_complex\_types  (in PyDSTool.common) | \_pytypefromtype  (in PyDSTool.Symbolic) | | \_\_init\_\_()  (in QuantSpec) | \_complex\_types  (in PyDSTool.parseUtils) | \_pytypefromtype  (in PyDSTool.Toolbox.event\_driven\_simulator) | | \_\_init\_\_()  (in Quantity) | \_complex\_types  (in PyDSTool.utils) | \_pytypefromtype  (in PyDSTool.Trajectory') | | \_\_init\_\_()  (in \_mathobj) | \_compute()  (in Continuation) | \_pytypefromtype  (in PyDSTool.Variable') | | \_\_init\_\_()  (in DSSRT\_info) | \_compute()  (in LimitCycleCurve) | \_pytypefromtype  (in PyDSTool.common) | | \_\_init\_\_()  (in cool) | \_constants  (in PyDSTool.PyCont.ContClass') | \_pytypefromtype  (in PyDSTool.parseUtils) | | \_\_init\_\_()  (in edge) | \_constants  (in PyDSTool.PyCont.Continuation) | \_pytypefromtype  (in PyDSTool.utils) | | \_\_init\_\_()  (in vertex) | \_constants  (in PyDSTool.Symbolic) | \_querykeys  (in Generator) | | \_\_init\_\_()  (in ExceptionFSM) | \_constants  (in PyDSTool.common) | \_querykeys  (in dsInterface) | | \_\_init\_\_()  (in FSM) | \_constants  (in PyDSTool.parseUtils) | \_querykeys  (in Model) | | \_\_init\_\_()  (in ModelEst) | \_createEventTimes()  (in Trajectory) | \_real\_types  (in PyDSTool.Events) | | \_\_init\_\_()  (in BoundMin) | \_createTestFuncs()  (in Continuation) | \_real\_types  (in PyDSTool.FuncSpec') | | \_\_init\_\_()  (in ParamEst) | \_createTestFuncs()  (in EquilibriumCurve) | \_real\_types  (in PyDSTool.Generator.ADMC\_ODEsystem') | | \_\_init\_\_()  (in data\_bins) | \_createTestFuncs()  (in FixedPointCurve) | \_real\_types  (in PyDSTool.Generator.DDEsystem) | | \_\_init\_\_()  (in data\_bins) | \_createTestFuncs()  (in FoldCurve) | \_real\_types  (in PyDSTool.Generator.Dopri\_ODEsystem') | | \_\_init\_\_()  (in EpochSeqScorer) | \_createTestFuncs()  (in HopfCurveOne) | \_real\_types  (in PyDSTool.Generator.EmbeddedSysGen') | | \_\_init\_\_()  (in Scorer) | \_createTestFuncs()  (in HopfCurveTwo) | \_real\_types  (in PyDSTool.Generator.Euler\_ODEsystem') | | \_\_init\_\_()  (in VarAlphabet) | \_createTestFuncs()  (in UserDefinedCurve) | \_real\_types  (in PyDSTool.Generator.ExplicitFnGen') | | \_\_init\_\_()  (in domscales) | \_ctn\_functions  (in PyDSTool.Toolbox.ParamEst) | \_real\_types  (in PyDSTool.Generator.ExtrapolateTable') | | \_\_init\_\_()  (in dssrt\_assistant) | \_curveToPointset()  (in Continuation) | \_real\_types  (in PyDSTool.Generator.ImplicitFnGen') | | \_\_init\_\_()  (in epoch) | \_debug\_snapshot()  (in Euler\_ODEsystem) | \_real\_types  (in PyDSTool.Generator.InterpolateTable') | | \_\_init\_\_()  (in regime) | \_debug\_snapshot()  (in Vode\_ODEsystem) | \_real\_types  (in PyDSTool.Generator.LookupTable') | | \_\_init\_\_()  (in FIFOqueue\_uniquenode) | \_default\_pert()  (in PyDSTool.Toolbox.PRCtools) | \_real\_types  (in PyDSTool.Generator.MapSystem') | | \_\_init\_\_()  (in composed\_map1D) | \_defaults  (in Descriptor) | \_real\_types  (in PyDSTool.Generator.ODEsystem') | | \_\_init\_\_()  (in connection) | \_defaults  (in GDescriptor) | \_real\_types  (in PyDSTool.Generator.Radau\_ODEsystem') | | \_\_init\_\_()  (in delay\_map) | \_defaults  (in MDescriptor) | \_real\_types  (in PyDSTool.Generator.Vode\_ODEsystem') | | \_\_init\_\_()  (in node) | \_delTraj()  (in Model) | \_real\_types  (in PyDSTool.Generator.baseclasses) | | \_\_init\_\_()  (in simulator) | \_deprecated\_functions  (in PyDSTool.Toolbox.ParamEst) | \_real\_types  (in PyDSTool.Interval') | | \_\_init\_\_()  (in estimate\_spiking) | \_distance()  (in distance\_to\_pointset) | \_real\_types  (in PyDSTool.Model) | | \_\_init\_\_()  (in spike\_envelope) | \_do\_fit()  (in fit\_function) | \_real\_types  (in PyDSTool.ModelConstructor') | | \_\_init\_\_()  (in AndComposition) | \_doPreMacrosC()  (in FuncSpec) | \_real\_types  (in PyDSTool.ModelSpec') | | \_\_init\_\_()  (in OrComposition) | \_dummy\_userfunc()  (in PyDSTool.Generator.Euler\_ODEsystem') | \_real\_types  (in PyDSTool.Points) | | \_\_init\_\_()  (in AbsoluteParametersCriterion) | \_EmptyClass  (in PyDSTool.fixedpickle) | \_real\_types  (in PyDSTool.Symbolic) | | \_\_init\_\_()  (in AbsoluteValueCriterion) | \_ensure\_inputs()  (in Dopri\_ODEsystem) | \_real\_types  (in PyDSTool.Toolbox.event\_driven\_simulator) | | \_\_init\_\_()  (in GradientCriterion) | \_ensure\_inputs()  (in Radau\_ODEsystem) | \_real\_types  (in PyDSTool.Trajectory') | | \_\_init\_\_()  (in IterationCriterion) | \_ensure\_jac()  (in fixedpoint\_2D) | \_real\_types  (in PyDSTool.Variable') | | \_\_init\_\_()  (in MonotonyCriterion) | \_ensure\_jac()  (in fixedpoint\_nD) | \_real\_types  (in PyDSTool.common) | | \_\_init\_\_()  (in RelativeParametersCriterion) | \_ensure\_solver()  (in Dopri\_ODEsystem) | \_real\_types  (in PyDSTool.integrator') | | \_\_init\_\_()  (in RelativeValueCriterion) | \_ensure\_solver()  (in Radau\_ODEsystem) | \_real\_types  (in PyDSTool.parseUtils) | | \_\_init\_\_()  (in AICCriterion) | \_errors  (in PyDSTool.Toolbox.ParamEst) | \_real\_types  (in PyDSTool.utils) | | \_\_init\_\_()  (in ModifiedAICCriterion) | \_eval()  (in QuantSpec) | \_register()  (in Generator) | | \_\_init\_\_()  (in CenteredFiniteDifferences) | \_eval()  (in PyDSTool.Symbolic) | \_register()  (in LeafComponent) | | \_\_init\_\_()  (in FiniteDifferencesCache) | \_eval()  (in get\_burst\_spikes) | \_register()  (in ModelSpec) | | \_\_init\_\_()  (in ForwardFiniteDifferences) | \_features  (in PyDSTool.Toolbox.NineML) | \_res\_fn()  (in residual\_fn\_context) | | \_\_init\_\_()  (in Quadratic) | \_features  (in PyDSTool.Toolbox.PySCes\_SBML) | \_res\_fn()  (in residual\_fn\_context\_1D) | | \_\_init\_\_()  (in AdaptiveLastStepModifier) | \_features  (in PyDSTool.Toolbox.phaseplane) | \_res\_fn()  (in FiniteDifferencesCache) | | \_\_init\_\_()  (in BacktrackingSearch) | \_filter\_consecutive()  (in PyDSTool.Toolbox.phaseplane) | \_residual\_info()  (in condition) | | \_\_init\_\_()  (in CubicInterpolationSearch) | \_filter\_utils  (in PyDSTool.Toolbox.data\_analysis) | \_residual\_info()  (in feature\_leaf) | | \_\_init\_\_()  (in DampedLineSearch) | \_find\_idx()  (in binary\_feature) | \_residual\_info()  (in feature\_node) | | \_\_init\_\_()  (in FibonacciSectionSearch) | \_find\_idx()  (in condition) | \_resolve\_indepvar()  (in Pointset) | | \_\_init\_\_()  (in FixedLastStepModifier) | \_find\_idx()  (in feature) | \_s2  (in mesh\_patch\_2D) | | \_\_init\_\_()  (in GoldenSectionSearch) | \_find\_idx()  (in boundary\_containment\_by\_event) | \_sample\_array\_interior()  (in PyDSTool.Toolbox.phaseplane) | | \_\_init\_\_()  (in GoldsteinRule) | \_find\_idx()  (in boundary\_containment\_by\_postproc) | \_savePointInfo()  (in Continuation) | | \_\_init\_\_()  (in HyperbolicLineSearch) | \_find\_idx()  (in domain\_test) | \_savePointInfo()  (in LimitCycleCurve) | | \_\_init\_\_()  (in QuadraticInterpolationSearch) | \_find\_min\_pt()  (in PyDSTool.Toolbox.phaseplane) | \_scalar\_diff()  (in PyDSTool.common) | | \_\_init\_\_()  (in ScaledLineSearch) | \_findTrajInitiator()  (in HybridModel) | \_seq\_types  (in PyDSTool.Events) | | \_\_init\_\_()  (in SimpleLineSearch) | \_findTrajInitiator()  (in NonHybridModel) | \_seq\_types  (in PyDSTool.FuncSpec') | | \_\_init\_\_()  (in StrongWolfePowellRule) | \_fit\_utils  (in PyDSTool.Toolbox.data\_analysis) | \_seq\_types  (in PyDSTool.Generator.ADMC\_ODEsystem') | | \_\_init\_\_()  (in WolfePowellRule) | \_fit\_utils  (in PyDSTool.Toolbox.dataanalysis) | \_seq\_types  (in PyDSTool.Generator.DDEsystem) | | \_\_init\_\_()  (in Optimizer) | \_float\_types  (in PyDSTool.Events) | \_seq\_types  (in PyDSTool.Generator.Dopri\_ODEsystem') | | \_\_init\_\_()  (in StandardOptimizer) | \_float\_types  (in PyDSTool.FuncSpec') | \_seq\_types  (in PyDSTool.Generator.EmbeddedSysGen') | | \_\_init\_\_()  (in StandardOptimizerModifying) | \_float\_types  (in PyDSTool.Generator.ADMC\_ODEsystem') | \_seq\_types  (in PyDSTool.Generator.Euler\_ODEsystem') | | \_\_init\_\_()  (in ConjugateGradientStep) | \_float\_types  (in PyDSTool.Generator.DDEsystem) | \_seq\_types  (in PyDSTool.Generator.ExplicitFnGen') | | \_\_init\_\_()  (in GoldsteinPriceStep) | \_float\_types  (in PyDSTool.Generator.Dopri\_ODEsystem') | \_seq\_types  (in PyDSTool.Generator.ExtrapolateTable') | | \_\_init\_\_()  (in LocalBruteForce1DStep) | \_float\_types  (in PyDSTool.Generator.EmbeddedSysGen') | \_seq\_types  (in PyDSTool.Generator.ImplicitFnGen') | | \_\_init\_\_()  (in MarquardtStep) | \_float\_types  (in PyDSTool.Generator.Euler\_ODEsystem') | \_seq\_types  (in PyDSTool.Generator.InterpolateTable') | | \_\_init\_\_()  (in PartialStep) | \_float\_types  (in PyDSTool.Generator.ExplicitFnGen') | \_seq\_types  (in PyDSTool.Generator.LookupTable') | | \_\_init\_\_()  (in DFPNewtonStep) | \_float\_types  (in PyDSTool.Generator.ExtrapolateTable') | \_seq\_types  (in PyDSTool.Generator.MapSystem') | | \_\_init\_\_()  (in RestartNotOrthogonalConjugateGradientStep) | \_float\_types  (in PyDSTool.Generator.ImplicitFnGen') | \_seq\_types  (in PyDSTool.Generator.ODEsystem') | | \_\_init\_\_()  (in RestartPeriodicallyConjugateGradientStep) | \_float\_types  (in PyDSTool.Generator.InterpolateTable') | \_seq\_types  (in PyDSTool.Generator.Radau\_ODEsystem') | | \_\_init\_\_()  (in Rosenbrock) | \_float\_types  (in PyDSTool.Generator.LookupTable') | \_seq\_types  (in PyDSTool.Generator.Vode\_ODEsystem') | | \_\_init\_\_()  (in Point2D) | \_float\_types  (in PyDSTool.Generator.MapSystem') | \_seq\_types  (in PyDSTool.Generator.baseclasses) | | \_\_init\_\_()  (in base\_n\_counter) | \_float\_types  (in PyDSTool.Generator.ODEsystem') | \_seq\_types  (in PyDSTool.Interval') | | \_\_init\_\_()  (in distance\_to\_pointset) | \_float\_types  (in PyDSTool.Generator.Radau\_ODEsystem') | \_seq\_types  (in PyDSTool.Model) | | \_\_init\_\_()  (in dx\_scaled\_2D) | \_float\_types  (in PyDSTool.Generator.Vode\_ODEsystem') | \_seq\_types  (in PyDSTool.ModelConstructor') | | \_\_init\_\_()  (in fixedpoint\_2D) | \_float\_types  (in PyDSTool.Generator.baseclasses) | \_seq\_types  (in PyDSTool.ModelSpec') | | \_\_init\_\_()  (in fixedpoint\_nD) | \_float\_types  (in PyDSTool.Interval') | \_seq\_types  (in PyDSTool.Points) | | \_\_init\_\_()  (in mesh\_patch\_2D) | \_float\_types  (in PyDSTool.Model) | \_seq\_types  (in PyDSTool.Symbolic) | | \_\_init\_\_()  (in nullcline) | \_float\_types  (in PyDSTool.ModelConstructor') | \_seq\_types  (in PyDSTool.Toolbox.NineML) | | \_\_init\_\_()  (in phaseplane) | \_float\_types  (in PyDSTool.ModelSpec') | \_seq\_types  (in PyDSTool.Toolbox.ParamEst) | | \_\_init\_\_()  (in plotter\_2D) | \_float\_types  (in PyDSTool.Points) | \_seq\_types  (in PyDSTool.Toolbox.PySCes\_SBML) | | \_\_init\_\_()  (in HybridTrajectory) | \_float\_types  (in PyDSTool.Symbolic) | \_seq\_types  (in PyDSTool.Toolbox.data\_analysis) | | \_\_init\_\_()  (in Trajectory) | \_float\_types  (in PyDSTool.Toolbox.event\_driven\_simulator) | \_seq\_types  (in PyDSTool.Toolbox.dataanalysis) | | \_\_init\_\_()  (in HybridVariable) | \_float\_types  (in PyDSTool.Trajectory') | \_seq\_types  (in PyDSTool.Toolbox.event\_driven\_simulator) | | \_\_init\_\_()  (in OutputFn) | \_float\_types  (in PyDSTool.Variable') | \_seq\_types  (in PyDSTool.Toolbox.phaseplane) | | \_\_init\_\_()  (in Variable) | \_float\_types  (in PyDSTool.common) | \_seq\_types  (in PyDSTool.Trajectory') | | \_\_init\_\_()  (in BarycentricInterpolator) | \_float\_types  (in PyDSTool.parseUtils) | \_seq\_types  (in PyDSTool.Variable') | | \_\_init\_\_()  (in DefaultDict) | \_float\_types  (in PyDSTool.utils) | \_seq\_types  (in PyDSTool.common) | | \_\_init\_\_()  (in Diagnostics) | \_force\_coords\_to\_ixlist()  (in Point) | \_seq\_types  (in PyDSTool.parseUtils) | | \_\_init\_\_()  (in DomainType) | \_functions  (in PyDSTool.MProject) | \_seq\_types  (in PyDSTool.utils) | | \_\_init\_\_()  (in KroghInterpolator) | \_functions  (in PyDSTool.ModelSpec') | \_set\_for\_hybrid\_DS()  (in Generator) | | \_\_init\_\_()  (in PiecewisePolynomial) | \_functions  (in PyDSTool.PyCont.Plotting) | \_set\_for\_hybrid\_DS()  (in Model) | | \_\_init\_\_()  (in Struct) | \_functions  (in PyDSTool.PyCont.misc) | \_set\_mutex\_zone\_by\_width()  (in nullcline\_zone\_leaf) | | \_\_init\_\_()  (in Verbose) | \_functions  (in PyDSTool.Symbolic) | \_setkeys  (in dsInterface) | | \_\_init\_\_()  (in args) | \_functions  (in PyDSTool.Toolbox.NineML) | \_setkeys  (in Model) | | \_\_init\_\_()  (in fit\_function) | \_functions  (in PyDSTool.Toolbox.PySCes\_SBML) | \_setRanges()  (in Variable) | | \_\_init\_\_()  (in interp0d) | \_functions  (in PyDSTool.Toolbox.dssrt) | \_setup\_taus\_psis()  (in dssrt\_assistant) | | \_\_init\_\_()  (in interp1d) | \_functions  (in PyDSTool.Toolbox.event\_driven\_simulator) | \_specStrParse()  (in FuncSpec) | | \_\_init\_\_()  (in metric) | \_functions  (in PyDSTool.Toolbox.phaseplane) | \_stability  (in fixedpoint\_nD) | | \_\_init\_\_()  (in not\_op) | \_functions  (in PyDSTool.common) | \_Stop  (in PyDSTool.fixedpickle) | | \_\_init\_\_()  (in predicate) | \_functions  (in PyDSTool.parseUtils) | \_symbconsts  (in PyDSTool.parseUtils) | | \_\_init\_\_()  (in predicate\_op) | \_functions  (in PyDSTool.utils) | \_symbfuncs  (in PyDSTool.parseUtils) | | \_\_init\_\_()  (in PyDSTool\_Error) | \_genAuxFnC()  (in FuncSpec) | \_system()  (in Continuation) | | \_\_init\_\_()  (in PyDSTool\_UncertainValueError) | \_genAuxFnMatlab()  (in FuncSpec) | \_systemjac()  (in Continuation) | | \_\_init\_\_()  (in Pickler) | \_genAuxFnPy()  (in FuncSpec) | \_systemjac\_withpars()  (in Continuation) | | \_\_init\_\_()  (in Unpickler) | \_generate\_ixmaps()  (in Generator) | \_systemjacuser()  (in Continuation) | | \_\_init\_\_()  (in \_Stop) | \_generate\_subderivatives()  (in PyDSTool.Symbolic) | \_systemuser()  (in Continuation) | | \_\_init\_\_()  (in integrator) | \_generateParamInfo()  (in Model) | \_term()  (in PyDSTool.Events) | | \_\_init\_\_()  (in auxfnDBclass) | \_generators  (in PyDSTool.Toolbox.synthetic\_data) | \_test()  (in PyDSTool.fixedpickle) | | \_\_init\_\_()  (in parserObject) | \_generators  (in PyDSTool.Toolbox.syntheticdata) | \_trajname  (in ModelInterface) | | \_\_init\_\_()  (in symbolMapClass) | \_generic\_opt  (in PyDSTool.Toolbox.ParamEst) | \_tuplesize2code  (in PyDSTool.fixedpickle) | | \_\_init\_\_()  (in ode) | \_genFromMSpec()  (in ModelConstructor) | \_typefrompytype  (in PyDSTool.common) | | \_\_init\_\_()  (in vode) | \_genFromStrings()  (in ModelConstructor) | \_utils  (in PyDSTool.Toolbox.ParamEst) | | \_\_iter\_\_()  (in args) | \_genSpecC()  (in FuncSpec) | \_validate\_weights()  (in EpochSeqScorer) | | \_\_le\_\_()  (in Interval) | \_genSpecFnC()  (in FuncSpec) | \_validateParameters()  (in HybridModel) | | \_\_le\_\_()  (in Point) | \_genSpecFnMatlab()  (in FuncSpec) | \_validateRegistry()  (in HybridModel) | | \_\_le\_\_()  (in Pointset) | \_genSpecFnPy()  (in FuncSpec) | \_validateRegistry()  (in NonHybridModel) | | \_\_le\_\_()  (in Point2D) | \_genSpecMatlab()  (in FuncSpec) | \_validateVarNames()  (in Model) | | \_\_le\_\_()  (in args) | \_genSpecPy()  (in FuncSpec) | \_validKeys  (in EmbeddedSysGen) | | \_\_len\_\_()  (in Model) | \_get\_eigen()  (in fixedpoint\_nD) | \_validKeys  (in ExplicitFnGen) | | \_\_len\_\_()  (in Point) | \_get\_initiator\_cache()  (in ModelInterface) | \_validKeys  (in ImplicitFnGen) | | \_\_len\_\_()  (in PointInfo) | \_grow\_zone\_by\_radius()  (in nullcline\_zone\_leaf) | \_validKeys  (in MapSystem) | | \_\_len\_\_()  (in Pointset) | \_grow\_zone\_by\_width()  (in nullcline\_zone\_leaf) | \_validKeys  (in ODEsystem) | | \_\_len\_\_()  (in QuantSpec) | \_highlevel()  (in PyDSTool.Events) | \_validKeys  (in Descriptor) | | \_\_len\_\_()  (in Quantity) | \_hypercuboid\_utils  (in PyDSTool.Toolbox.synthetic\_data) | \_validKeys  (in GDescriptor) | | \_\_len\_\_()  (in Point2D) | \_hypercuboid\_utils  (in PyDSTool.Toolbox.syntheticdata) | \_validKeys  (in MDescriptor) | | \_\_len\_\_()  (in args) | \_implicitSolveMethods  (in PyDSTool.Events) | \_varlinked()  (in PyDSTool.Events) | | \_\_len\_\_()  (in symbolMapClass) | \_implicitSolveMethods  (in PyDSTool.FuncSpec') | \_verify\_type\_names  (in PyDSTool.common) | | \_\_LICENSE\_\_  (in PyDSTool) | \_implicitSolveMethods  (in PyDSTool.Generator.ADMC\_ODEsystem') | \_xinf\_1D()  (in PyDSTool.Toolbox.phaseplane) | | \_\_locate\_newton()  (in BranchPoint) | \_implicitSolveMethods  (in PyDSTool.Generator.DDEsystem) | \_xinf\_ND()  (in PyDSTool.Toolbox.phaseplane) | |

  
  

| Home | Trees | Indices | Help | | PyDSTool | | --- | |
| --- | --- | --- | --- | --- | --- |

|  |  |
| --- | --- |
| Generated by Epydoc 3.0.1 on Fri May 4 15:24:00 2012 | http://epydoc.sourceforge.net |
