# Supplementary material for: Hybrid Models and Biological Model Reduction with PyDSTool
Source: PLoS Comput Biol. 2012 Aug 9;8(8):e1002628. doi: 10.1371/journal.pcbi.1002628 (PMC3415397; doi:10.1371/journal.pcbi.1002628)
Supplement: Text S4 — Complete source code for the PyDSTool package (version 0.88.120504). Includes API documentation and help files linking to web pages. This file is identical to the current public release on Sourceforge.net. (ZIP) [file pcbi.1002628.s004.zip › PyDSTool/html/identifier-index-B.html]

xml version="1.0" encoding="ascii"?


Identifier Index


| Home | Trees | Indices | Help | | PyDSTool | | --- | |
| --- | --- | --- | --- | --- | --- |

|  |  |  |  |
| --- | --- | --- | --- |
|  | |  | | --- | | [hide private] | | [frames] | no frames] | |

|  |  |
| --- | --- |
| Identifier Index | [ A B C D E F G H I J K L M N O P Q R S T U V W X Y Z \_ ] |

|  |  |  |  |  |  |  |  |  |  |  |  |  |  |  |  |  |  |  |  |  |  |  |  |  |  |  |  |  |  |  |  |  |  |  |  |  |  |  |  |  |  |  |  |  |  |  |  |  |  |  |  |  |  |  |  |  |  |  |  |  |  |  |  |  |  |  |  |  |  |  |  |  |  |  |  |  |  |  |  |  |  |  |  |  |  |  |  |  |  |  |  |  |  |  |  |  |  |  |  |  |  |  |  |  |  |  |  |  |  |  |  |  |  |  |  |  |  |  |  |  |  |  |  |  |  |  |  |  |  |  |  |  |  |  |  |  |  |  |  |  |  |  |  |  |  |  |  |  |  |  |  |  |  |  |  |  |  |  |  |  |  |  |  |
| --- | --- | --- | --- | --- | --- | --- | --- | --- | --- | --- | --- | --- | --- | --- | --- | --- | --- | --- | --- | --- | --- | --- | --- | --- | --- | --- | --- | --- | --- | --- | --- | --- | --- | --- | --- | --- | --- | --- | --- | --- | --- | --- | --- | --- | --- | --- | --- | --- | --- | --- | --- | --- | --- | --- | --- | --- | --- | --- | --- | --- | --- | --- | --- | --- | --- | --- | --- | --- | --- | --- | --- | --- | --- | --- | --- | --- | --- | --- | --- | --- | --- | --- | --- | --- | --- | --- | --- | --- | --- | --- | --- | --- | --- | --- | --- | --- | --- | --- | --- | --- | --- | --- | --- | --- | --- | --- | --- | --- | --- | --- | --- | --- | --- | --- | --- | --- | --- | --- | --- | --- | --- | --- | --- | --- | --- | --- | --- | --- | --- | --- | --- | --- | --- | --- | --- | --- | --- | --- | --- | --- | --- | --- | --- | --- | --- | --- | --- | --- | --- | --- | --- | --- | --- | --- | --- | --- | --- | --- | --- | --- | --- | --- | --- |
| B | |  |  |  | | --- | --- | --- | | B\_Check  (in PyDSTool.PyCont.TestFunc) | bitwise\_and  (in PyDSTool.Toolbox.DSSRT\_tools) | bitwise\_xor  (in PyDSTool.Toolbox.fracdim) | | backtracking\_search  (in PyDSTool.Toolbox.optimizers.line\_search) | bitwise\_and  (in PyDSTool.Toolbox.InputProfile) | bitwise\_xor  (in PyDSTool.Toolbox.makeSloppyModel) | | BacktrackingSearch  (in PyDSTool.Toolbox.optimizers.line\_search.backtracking\_search) | bitwise\_and  (in PyDSTool.Toolbox.ModelHelper) | bitwise\_xor  (in PyDSTool.Toolbox.neuralcomp) | | backward()  (in Continuation) | bitwise\_and  (in PyDSTool.Toolbox.NineML) | bitwise\_xor  (in PyDSTool.Toolbox.phaseplane) | | BadPickleGet | bitwise\_and  (in PyDSTool.Toolbox.adjointPRC) | bitwise\_xor  (in PyDSTool.Toolbox.synthetic\_data) | | BarycentricInterpolator  (in PyDSTool.common) | bitwise\_and  (in PyDSTool.Toolbox.dataanalysis) | bitwise\_xor  (in PyDSTool.Toolbox.syntheticdata) | | barzilai\_borwein\_non\_monotone\_search  (in PyDSTool.Toolbox.optimizers.line\_search) | bitwise\_and  (in PyDSTool.Toolbox.fracdim) | bitwise\_xor  (in PyDSTool) | | barzilai\_borwein\_search  (in PyDSTool.Toolbox.optimizers.line\_search) | bitwise\_and  (in PyDSTool.Toolbox.makeSloppyModel) | bitwise\_xor  (in matplotlib.pylab) | | BarzilaiBorwein()  (in PyDSTool.Toolbox.optimizers.line\_search.barzilai\_borwein\_search) | bitwise\_and  (in PyDSTool.Toolbox.neuralcomp) | BorderMethod  (in PyDSTool.PyCont.TestFunc) | | BarzilaiBorwein\_nonmonotone()  (in PyDSTool.Toolbox.optimizers.line\_search.barzilai\_borwein\_non\_monotone\_search) | bitwise\_and  (in PyDSTool.Toolbox.phaseplane) | boundary\_containment  (in PyDSTool.Model) | | base\_n\_counter  (in PyDSTool.Toolbox.phaseplane) | bitwise\_and  (in PyDSTool.Toolbox.synthetic\_data) | boundary\_containment\_by\_event  (in PyDSTool.Model) | | baseclasses  (in PyDSTool.Generator) | bitwise\_and  (in PyDSTool.Toolbox.syntheticdata) | boundary\_containment\_by\_postproc  (in PyDSTool.Model) | | become\_most\_dominant  (in PyDSTool.Toolbox.dssrt) | bitwise\_and  (in PyDSTool) | BoundMin  (in PyDSTool.Toolbox.ParamEst) | | Beta()  (in PyDSTool.Toolbox.ActivationFuncs) | bitwise\_and  (in matplotlib.pylab) | BPoint  (in PyDSTool.PyCont.BifPoint) | | Betavariate  (in PyDSTool) | bitwise\_not  (in PyDSTool.PyCont.ContClass') | Branch\_Bor  (in PyDSTool.PyCont.TestFunc) | | Betavariate  (in PyDSTool.ModelSpec') | bitwise\_not  (in PyDSTool.Toolbox.ActivationFuncs) | Branch\_Det  (in PyDSTool.PyCont.TestFunc) | | Betavariate  (in PyDSTool.Symbolic) | bitwise\_not  (in PyDSTool.Toolbox.DSSRT\_tools) | BranchPoint  (in PyDSTool.PyCont.BifPoint) | | Betavariate  (in PyDSTool.Toolbox.ActivationFuncs) | bitwise\_not  (in PyDSTool.Toolbox.InputProfile) | BT\_Fold  (in PyDSTool.PyCont.TestFunc) | | Betavariate  (in PyDSTool.Toolbox.DSSRT\_tools) | bitwise\_not  (in PyDSTool.Toolbox.ModelHelper) | BT\_Hopf  (in PyDSTool.PyCont.TestFunc) | | Betavariate  (in PyDSTool.Toolbox.InputProfile) | bitwise\_not  (in PyDSTool.Toolbox.NineML) | BT\_Hopf\_One  (in PyDSTool.PyCont.TestFunc) | | Betavariate  (in PyDSTool.Toolbox.ModelHelper) | bitwise\_not  (in PyDSTool.Toolbox.adjointPRC) | BTPoint  (in PyDSTool.PyCont.BifPoint) | | Betavariate  (in PyDSTool.Toolbox.NineML) | bitwise\_not  (in PyDSTool.Toolbox.dataanalysis) | BUFSIZE  (in PyDSTool) | | Betavariate  (in PyDSTool.Toolbox.adjointPRC) | bitwise\_not  (in PyDSTool.Toolbox.fracdim) | BUFSIZE  (in PyDSTool.PyCont.ContClass') | | Betavariate  (in PyDSTool.Toolbox.dataanalysis) | bitwise\_not  (in PyDSTool.Toolbox.makeSloppyModel) | BUFSIZE  (in PyDSTool.Toolbox.ActivationFuncs) | | Betavariate  (in PyDSTool.Toolbox.fracdim) | bitwise\_not  (in PyDSTool.Toolbox.neuralcomp) | BUFSIZE  (in PyDSTool.Toolbox.DSSRT\_tools) | | Betavariate  (in PyDSTool.Toolbox.makeSloppyModel) | bitwise\_not  (in PyDSTool.Toolbox.phaseplane) | BUFSIZE  (in PyDSTool.Toolbox.InputProfile) | | Betavariate  (in PyDSTool.Toolbox.neuralcomp) | bitwise\_not  (in PyDSTool.Toolbox.synthetic\_data) | BUFSIZE  (in PyDSTool.Toolbox.ModelHelper) | | Betavariate  (in PyDSTool.Toolbox.phaseplane) | bitwise\_not  (in PyDSTool.Toolbox.syntheticdata) | BUFSIZE  (in PyDSTool.Toolbox.NineML) | | Betavariate  (in PyDSTool.Toolbox.synthetic\_data) | bitwise\_not  (in PyDSTool) | BUFSIZE  (in PyDSTool.Toolbox.adjointPRC) | | Betavariate  (in PyDSTool.Toolbox.syntheticdata) | bitwise\_not  (in matplotlib.pylab) | BUFSIZE  (in PyDSTool.Toolbox.dataanalysis) | | BiAltMethod  (in PyDSTool.PyCont.TestFunc) | bitwise\_or  (in PyDSTool.PyCont.ContClass') | BUFSIZE  (in PyDSTool.Toolbox.fracdim) | | bialtprod()  (in BiAltMethod) | bitwise\_or  (in PyDSTool.Toolbox.ActivationFuncs) | BUFSIZE  (in PyDSTool.Toolbox.makeSloppyModel) | | bialtprodeye()  (in BiAltMethod) | bitwise\_or  (in PyDSTool.Toolbox.DSSRT\_tools) | BUFSIZE  (in PyDSTool.Toolbox.neuralcomp) | | bialttoeig()  (in PyDSTool.PyCont.misc) | bitwise\_or  (in PyDSTool.Toolbox.InputProfile) | BUFSIZE  (in PyDSTool.Toolbox.phaseplane) | | bif\_curve\_colors  (in PyDSTool.PyCont.Continuation) | bitwise\_or  (in PyDSTool.Toolbox.ModelHelper) | BUFSIZE  (in PyDSTool.Toolbox.synthetic\_data) | | bif\_point\_colors  (in PyDSTool.PyCont.Continuation) | bitwise\_or  (in PyDSTool.Toolbox.NineML) | BUFSIZE  (in PyDSTool.Toolbox.syntheticdata) | | BifPoint  (in PyDSTool.PyCont) | bitwise\_or  (in PyDSTool.Toolbox.adjointPRC) | BUFSIZE  (in matplotlib.pylab) | | BifPoint  (in PyDSTool.PyCont.BifPoint) | bitwise\_or  (in PyDSTool.Toolbox.dataanalysis) | BUILD  (in PyDSTool.fixedpickle) | | bilinearform()  (in PyDSTool.PyCont.misc) | bitwise\_or  (in PyDSTool.Toolbox.fracdim) | build()  (in ModelManager) | | bin()  (in data\_bins) | bitwise\_or  (in PyDSTool.Toolbox.makeSloppyModel) | build\_vf()  (in phaseplane) | | bin()  (in data\_bins) | bitwise\_or  (in PyDSTool.Toolbox.neuralcomp) | builtin\_auxnames  (in PyDSTool.FuncSpec') | | binary\_feature  (in PyDSTool.MProject) | bitwise\_or  (in PyDSTool.Toolbox.phaseplane) | builtin\_auxnames  (in PyDSTool.ModelSpec') | | BINFLOAT  (in PyDSTool.fixedpickle) | bitwise\_or  (in PyDSTool.Toolbox.synthetic\_data) | builtin\_auxnames  (in PyDSTool.Symbolic) | | BINGET  (in PyDSTool.fixedpickle) | bitwise\_or  (in PyDSTool.Toolbox.syntheticdata) | builtin\_auxnames  (in PyDSTool.Trajectory') | | BININT  (in PyDSTool.fixedpickle) | bitwise\_or  (in PyDSTool) | builtin\_auxnames  (in PyDSTool.parseUtils) | | BININT1  (in PyDSTool.fixedpickle) | bitwise\_or  (in matplotlib.pylab) | builtinFnSigInfo  (in PyDSTool.FuncSpec') | | BININT2  (in PyDSTool.fixedpickle) | bitwise\_xor  (in PyDSTool.PyCont.ContClass') | builtinFnSigInfo  (in PyDSTool.ModelSpec') | | BINPERSID  (in PyDSTool.fixedpickle) | bitwise\_xor  (in PyDSTool.Toolbox.ActivationFuncs) | builtinFnSigInfo  (in PyDSTool.Symbolic) | | BINPUT  (in PyDSTool.fixedpickle) | bitwise\_xor  (in PyDSTool.Toolbox.DSSRT\_tools) | builtinFnSigInfo  (in PyDSTool.Trajectory') | | BINSTRING  (in PyDSTool.fixedpickle) | bitwise\_xor  (in PyDSTool.Toolbox.InputProfile) | builtinFnSigInfo  (in PyDSTool.parseUtils) | | BINUNICODE  (in PyDSTool.fixedpickle) | bitwise\_xor  (in PyDSTool.Toolbox.ModelHelper) | builtinlookup  (in PyDSTool.Symbolic) | | bisection()  (in PyDSTool.Toolbox.phaseplane) | bitwise\_xor  (in PyDSTool.Toolbox.NineML) | burst\_feature  (in PyDSTool.Toolbox.neuro\_data) | | bitwise\_and  (in PyDSTool.PyCont.ContClass') | bitwise\_xor  (in PyDSTool.Toolbox.adjointPRC) | bylabel()  (in Pointset) | | bitwise\_and  (in PyDSTool.Toolbox.ActivationFuncs) | bitwise\_xor  (in PyDSTool.Toolbox.dataanalysis) |  | |

  
  

| Home | Trees | Indices | Help | | PyDSTool | | --- | |
| --- | --- | --- | --- | --- | --- |

|  |  |
| --- | --- |
| Generated by Epydoc 3.0.1 on Fri May 4 15:23:56 2012 | http://epydoc.sourceforge.net |
