# Supplementary material for: Hybrid Models and Biological Model Reduction with PyDSTool
Source: PLoS Comput Biol. 2012 Aug 9;8(8):e1002628. doi: 10.1371/journal.pcbi.1002628 (PMC3415397; doi:10.1371/journal.pcbi.1002628)
Supplement: Text S4 — Complete source code for the PyDSTool package (version 0.88.120504). Includes API documentation and help files linking to web pages. This file is identical to the current public release on Sourceforge.net. (ZIP) [file pcbi.1002628.s004.zip › PyDSTool/html/identifier-index-C.html]

xml version="1.0" encoding="ascii"?


Identifier Index


| Home | Trees | Indices | Help | | PyDSTool | | --- | |
| --- | --- | --- | --- | --- | --- |

|  |  |  |  |
| --- | --- | --- | --- |
|  | |  | | --- | | [hide private] | | [frames] | no frames] | |

|  |  |
| --- | --- |
| Identifier Index | [ A B C D E F G H I J K L M N O P Q R S T U V W X Y Z \_ ] |

|  |  |  |  |  |  |  |  |  |  |  |  |  |  |  |  |  |  |  |  |  |  |  |  |  |  |  |  |  |  |  |  |  |  |  |  |  |  |  |  |  |  |  |  |  |  |  |  |  |  |  |  |  |  |  |  |  |  |  |  |  |  |  |  |  |  |  |  |  |  |  |  |  |  |  |  |  |  |  |  |  |  |  |  |  |  |  |  |  |  |  |  |  |  |  |  |  |  |  |  |  |  |  |  |  |  |  |  |  |  |  |  |  |  |  |  |  |  |  |  |  |  |  |  |  |  |  |  |  |  |  |  |  |  |  |  |  |  |  |  |  |  |  |  |  |  |  |  |  |  |  |  |  |  |  |  |  |  |  |  |  |  |  |  |  |  |  |  |  |  |  |  |  |  |  |  |  |  |  |  |  |  |  |  |  |  |  |  |  |  |  |  |  |  |  |  |  |  |  |  |  |  |  |  |  |  |  |  |  |  |  |  |  |  |  |  |  |  |  |  |  |  |  |  |  |  |  |  |  |  |  |  |  |  |  |  |  |  |  |  |  |  |  |  |  |  |  |  |  |  |  |  |  |  |  |  |  |  |  |  |  |  |  |  |  |  |  |  |  |  |  |  |  |  |  |  |  |  |  |  |  |  |  |  |  |  |  |  |  |  |  |  |  |  |  |  |  |  |  |  |  |  |  |  |  |  |  |  |  |  |  |  |  |  |  |  |  |  |  |  |  |  |  |  |  |  |  |  |  |  |  |  |  |  |  |  |  |  |  |  |  |  |  |  |  |  |  |  |  |  |  |  |  |  |  |  |  |  |  |  |  |  |  |  |  |  |  |  |  |  |  |  |  |  |  |  |  |  |  |  |  |  |  |  |  |  |  |  |  |  |  |  |  |  |  |  |  |  |  |  |  |  |  |  |  |  |  |  |  |  |  |  |  |  |  |  |  |  |  |  |  |  |  |  |  |  |  |  |  |  |  |  |  |  |  |  |  |  |  |  |  |  |  |  |  |  |  |  |  |  |  |  |  |  |  |  |  |  |  |  |  |  |  |  |  |  |  |
| --- | --- | --- | --- | --- | --- | --- | --- | --- | --- | --- | --- | --- | --- | --- | --- | --- | --- | --- | --- | --- | --- | --- | --- | --- | --- | --- | --- | --- | --- | --- | --- | --- | --- | --- | --- | --- | --- | --- | --- | --- | --- | --- | --- | --- | --- | --- | --- | --- | --- | --- | --- | --- | --- | --- | --- | --- | --- | --- | --- | --- | --- | --- | --- | --- | --- | --- | --- | --- | --- | --- | --- | --- | --- | --- | --- | --- | --- | --- | --- | --- | --- | --- | --- | --- | --- | --- | --- | --- | --- | --- | --- | --- | --- | --- | --- | --- | --- | --- | --- | --- | --- | --- | --- | --- | --- | --- | --- | --- | --- | --- | --- | --- | --- | --- | --- | --- | --- | --- | --- | --- | --- | --- | --- | --- | --- | --- | --- | --- | --- | --- | --- | --- | --- | --- | --- | --- | --- | --- | --- | --- | --- | --- | --- | --- | --- | --- | --- | --- | --- | --- | --- | --- | --- | --- | --- | --- | --- | --- | --- | --- | --- | --- | --- | --- | --- | --- | --- | --- | --- | --- | --- | --- | --- | --- | --- | --- | --- | --- | --- | --- | --- | --- | --- | --- | --- | --- | --- | --- | --- | --- | --- | --- | --- | --- | --- | --- | --- | --- | --- | --- | --- | --- | --- | --- | --- | --- | --- | --- | --- | --- | --- | --- | --- | --- | --- | --- | --- | --- | --- | --- | --- | --- | --- | --- | --- | --- | --- | --- | --- | --- | --- | --- | --- | --- | --- | --- | --- | --- | --- | --- | --- | --- | --- | --- | --- | --- | --- | --- | --- | --- | --- | --- | --- | --- | --- | --- | --- | --- | --- | --- | --- | --- | --- | --- | --- | --- | --- | --- | --- | --- | --- | --- | --- | --- | --- | --- | --- | --- | --- | --- | --- | --- | --- | --- | --- | --- | --- | --- | --- | --- | --- | --- | --- | --- | --- | --- | --- | --- | --- | --- | --- | --- | --- | --- | --- | --- | --- | --- | --- | --- | --- | --- | --- | --- | --- | --- | --- | --- | --- | --- | --- | --- | --- | --- | --- | --- | --- | --- | --- | --- | --- | --- | --- | --- | --- | --- | --- | --- | --- | --- | --- | --- | --- | --- | --- | --- | --- | --- | --- | --- | --- | --- | --- | --- | --- | --- | --- | --- | --- | --- | --- | --- | --- | --- | --- | --- | --- | --- | --- | --- | --- | --- | --- | --- | --- | --- | --- | --- | --- | --- | --- | --- | --- | --- | --- | --- | --- | --- | --- | --- | --- | --- | --- | --- | --- | --- | --- | --- | --- | --- | --- | --- | --- | --- | --- | --- | --- | --- | --- | --- | --- | --- | --- | --- | --- | --- | --- | --- | --- | --- | --- | --- | --- | --- | --- | --- | --- | --- | --- | --- | --- | --- | --- | --- | --- | --- | --- | --- | --- | --- | --- | --- | --- | --- | --- | --- | --- | --- | --- | --- | --- | --- | --- | --- | --- | --- | --- | --- | --- | --- | --- | --- | --- | --- | --- | --- |
| C | |  |  |  | | --- | --- | --- | | c  (in PyDSTool.Interval') | CLIP  (in PyDSTool.Toolbox.ActivationFuncs) | contained  (in PyDSTool.Toolbox.synthetic\_data) | | c  (in PyDSTool.Symbolic) | CLIP  (in PyDSTool.Toolbox.DSSRT\_tools) | contained  (in PyDSTool.Toolbox.syntheticdata) | | c\_  (in PyDSTool.PyCont.ContClass') | CLIP  (in PyDSTool.Toolbox.InputProfile) | contained  (in PyDSTool.Trajectory') | | c\_  (in PyDSTool.PyCont.Continuation) | CLIP  (in PyDSTool.Toolbox.ModelHelper) | contained  (in PyDSTool.Variable') | | c\_  (in PyDSTool.PyCont.misc) | CLIP  (in PyDSTool.Toolbox.NineML) | contains()  (in Event) | | c\_  (in PyDSTool.Toolbox.ActivationFuncs) | CLIP  (in PyDSTool.Toolbox.adjointPRC) | contains()  (in Generator) | | c\_  (in PyDSTool.Toolbox.DSSRT\_tools) | CLIP  (in PyDSTool.Toolbox.dataanalysis) | contains()  (in Interval) | | c\_  (in PyDSTool.Toolbox.InputProfile) | CLIP  (in PyDSTool.Toolbox.fracdim) | ContClass  (in PyDSTool.PyCont.ContClass') | | c\_  (in PyDSTool.Toolbox.ModelHelper) | CLIP  (in PyDSTool.Toolbox.makeSloppyModel) | ContClass'  (in PyDSTool.PyCont) | | c\_  (in PyDSTool.Toolbox.NineML) | CLIP  (in PyDSTool.Toolbox.neuralcomp) | context  (in PyDSTool.MProject) | | c\_  (in PyDSTool.Toolbox.adjointPRC) | CLIP  (in PyDSTool.Toolbox.phaseplane) | Continuation  (in PyDSTool.PyCont) | | c\_  (in PyDSTool.Toolbox.dataanalysis) | CLIP  (in PyDSTool.Toolbox.synthetic\_data) | Continuation  (in PyDSTool.PyCont.Continuation) | | c\_  (in PyDSTool.Toolbox.fracdim) | CLIP  (in PyDSTool.Toolbox.syntheticdata) | Continue()  (in dopri) | | c\_  (in PyDSTool.Toolbox.makeSloppyModel) | CLIP  (in matplotlib.pylab) | Continue()  (in radau) | | c\_  (in PyDSTool.Toolbox.neuralcomp) | CLOSE  (in PyDSTool.Toolbox.dssrt) | Continue()  (in integrator) | | c\_  (in PyDSTool.Toolbox.phaseplane) | closest\_perp\_distance\_between\_sample\_points()  (in PyDSTool.Toolbox.phaseplane) | Continuous  (in PyDSTool.FuncSpec') | | c\_  (in PyDSTool.Toolbox.synthetic\_data) | closest\_perp\_distance\_between\_splines()  (in PyDSTool.Toolbox.phaseplane) | Continuous  (in PyDSTool.Generator.ADMC\_ODEsystem') | | c\_  (in PyDSTool.Toolbox.syntheticdata) | closest\_perp\_distance\_on\_spline()  (in PyDSTool.Toolbox.phaseplane) | Continuous  (in PyDSTool.Generator.Dopri\_ODEsystem') | | c\_  (in PyDSTool) | collate\_results()  (in condition) | Continuous  (in PyDSTool.Generator.EmbeddedSysGen') | | c\_  (in matplotlib.pylab) | collect\_numbers()  (in PyDSTool.parseUtils) | Continuous  (in PyDSTool.Generator.Euler\_ODEsystem') | | calc\_epochs()  (in domscales) | collectReused()  (in PyDSTool.Events) | Continuous  (in PyDSTool.Generator.ExplicitFnGen') | | calc\_fast\_slow()  (in domscales) | colorbar\_doc  (in PyDSTool.PyCont.ContClass') | Continuous  (in PyDSTool.Generator.ExtrapolateTable') | | calc\_inf()  (in dssrt\_assistant) | colorbar\_doc  (in matplotlib.pylab) | Continuous  (in PyDSTool.Generator.ImplicitFnGen') | | calc\_Is()  (in dssrt\_assistant) | colormap()  (in PyDSTool.Toolbox.data\_analysis) | Continuous  (in PyDSTool.Generator.InterpolateTable') | | calc\_psi()  (in dssrt\_assistant) | colormap()  (in PyDSTool.Toolbox.dataanalysis) | Continuous  (in PyDSTool.Generator.LookupTable') | | calc\_psis()  (in dssrt\_assistant) | commit()  (in GenTransform) | Continuous  (in PyDSTool.Generator.MapSystem') | | calc\_rankings()  (in dssrt\_assistant) | commit()  (in ModelTransform) | Continuous  (in PyDSTool.Generator.ODEsystem') | | calc\_regimes()  (in domscales) | commit\_gentrans()  (in ModelTransform) | Continuous  (in PyDSTool.Generator.Radau\_ODEsystem') | | calc\_tau()  (in dssrt\_assistant) | commit\_trans()  (in ModelManager) | Continuous  (in PyDSTool.Generator.Vode\_ODEsystem') | | calc\_taus\_infs()  (in dssrt\_assistant) | common  (in PyDSTool) | Continuous  (in PyDSTool.Interval') | | cartesianProduct()  (in PyDSTool.utils) | comp\_seqs()  (in PyDSTool.Toolbox.dssrt) | Continuous  (in PyDSTool.ModelConstructor') | | cast  (in PyDSTool.PyCont.ContClass') | compare\_data\_from\_events()  (in PyDSTool.Toolbox.ParamEst) | Continuous  (in PyDSTool.ModelSpec') | | cast  (in PyDSTool.Toolbox.ActivationFuncs) | compare\_pert()  (in PyDSTool.Toolbox.PRCtools) | Continuous  (in PyDSTool.Symbolic) | | cast  (in PyDSTool.Toolbox.DSSRT\_tools) | compareBaseClass()  (in PyDSTool.common) | Continuous  (in PyDSTool.Toolbox.NineML) | | cast  (in PyDSTool.Toolbox.InputProfile) | compareClassAndBases()  (in PyDSTool.common) | Continuous  (in PyDSTool.Toolbox.dataanalysis) | | cast  (in PyDSTool.Toolbox.ModelHelper) | compareList()  (in PyDSTool.utils) | Continuous  (in PyDSTool.Toolbox.event\_driven\_simulator) | | cast  (in PyDSTool.Toolbox.NineML) | compareNumTypes()  (in PyDSTool.common) | Continuous  (in PyDSTool.Toolbox.phaseplane) | | cast  (in PyDSTool.Toolbox.adjointPRC) | comparePointCoords()  (in PyDSTool.Points) | Continuous  (in PyDSTool.Toolbox.synthetic\_data) | | cast  (in PyDSTool.Toolbox.dataanalysis) | compartment  (in PyDSTool.Toolbox.neuralcomp) | Continuous  (in PyDSTool.Toolbox.syntheticdata) | | cast  (in PyDSTool.Toolbox.fracdim) | compatGens  (in PyDSTool.Toolbox.neuralcomp) | Continuous  (in PyDSTool.Trajectory') | | cast  (in PyDSTool.Toolbox.makeSloppyModel) | compatible\_formats  (in PyDSTool.fixedpickle) | Continuous  (in PyDSTool.Variable') | | cast  (in PyDSTool.Toolbox.neuralcomp) | compatible\_formats  (in cPickle) | Continuous  (in PyDSTool.common) | | cast  (in PyDSTool.Toolbox.phaseplane) | compatibleContainers  (in Component) | Continuous  (in PyDSTool.parseUtils) | | cast  (in PyDSTool.Toolbox.synthetic\_data) | compatibleContainers  (in LeafComponent) | Continuous  (in PyDSTool.utils) | | cast  (in PyDSTool.Toolbox.syntheticdata) | compatibleContainers  (in ModelSpec) | Converged  (in PyDSTool.Toolbox.ParamEst) | | cast  (in PyDSTool) | compatibleContainers  (in channel) | convert\_power\_reserved\_keywords  (in PyDSTool.parseUtils) | | cast  (in matplotlib.pylab) | compatibleContainers  (in neuron) | convert\_ptlabel\_events()  (in PyDSTool.Trajectory') | | Ceil  (in PyDSTool) | compatibleContainers  (in soma) | convertComponent()  (in GenTransform) | | Ceil  (in PyDSTool.ModelSpec') | compatibleContainers  (in synapse) | convertPowers()  (in PyDSTool.parseUtils) | | ceil  (in PyDSTool.PyCont.ContClass') | compatibleGens  (in ModelSpec) | convertQuantity()  (in GenTransform) | | Ceil  (in PyDSTool.Symbolic) | compatibleGens  (in Quantity) | cool  (in PyDSTool.Toolbox.FR) | | ceil  (in PyDSTool.Symbolic) | compatibleGens  (in NineMLModel) | cool\_simple()  (in PyDSTool.Toolbox.FR) | | Ceil  (in PyDSTool.Toolbox.ActivationFuncs) | compatibleGens  (in sloppyModel) | copy()  (in args) | | Ceil  (in PyDSTool.Toolbox.DSSRT\_tools) | compatibleGens  (in compatODEComponent) | copy()  (in symbolMapClass) | | Ceil  (in PyDSTool.Toolbox.InputProfile) | compatibleGens  (in compatODELeafComponent) | copyright  (in PyDSTool.conf) | | Ceil  (in PyDSTool.Toolbox.ModelHelper) | compatibleSubcomponents  (in Component) | copysign  (in PyDSTool.PyCont.ContClass') | | Ceil  (in PyDSTool.Toolbox.NineML) | compatibleSubcomponents  (in LeafComponent) | copysign  (in PyDSTool.Toolbox.ActivationFuncs) | | ceil  (in PyDSTool.Toolbox.NineML) | compatibleSubcomponents  (in ModelSpec) | copysign  (in PyDSTool.Toolbox.DSSRT\_tools) | | Ceil  (in PyDSTool.Toolbox.adjointPRC) | compatibleSubcomponents  (in compartment) | copysign  (in PyDSTool.Toolbox.InputProfile) | | Ceil  (in PyDSTool.Toolbox.dataanalysis) | compatibleSubcomponents  (in network) | copysign  (in PyDSTool.Toolbox.ModelHelper) | | ceil  (in PyDSTool.Toolbox.dataanalysis) | compatibleSubcomponents  (in neuron) | copysign  (in PyDSTool.Toolbox.NineML) | | Ceil  (in PyDSTool.Toolbox.fracdim) | compatibleSubcomponents  (in pnnetwork) | copysign  (in PyDSTool.Toolbox.adjointPRC) | | Ceil  (in PyDSTool.Toolbox.makeSloppyModel) | compatODEComponent  (in PyDSTool.Toolbox.neuralcomp) | copysign  (in PyDSTool.Toolbox.dataanalysis) | | Ceil  (in PyDSTool.Toolbox.neuralcomp) | compatODELeafComponent  (in PyDSTool.Toolbox.neuralcomp) | copysign  (in PyDSTool.Toolbox.fracdim) | | Ceil  (in PyDSTool.Toolbox.phaseplane) | compile\_next\_state()  (in simulator) | copysign  (in PyDSTool.Toolbox.makeSloppyModel) | | ceil  (in PyDSTool.Toolbox.phaseplane) | compileAutoLib()  (in ContClass) | copysign  (in PyDSTool.Toolbox.neuralcomp) | | Ceil  (in PyDSTool.Toolbox.synthetic\_data) | compileFuncSpec()  (in Component) | copysign  (in PyDSTool.Toolbox.phaseplane) | | ceil  (in PyDSTool.Toolbox.synthetic\_data) | compileFuncSpec()  (in LeafComponent) | copysign  (in PyDSTool.Toolbox.synthetic\_data) | | Ceil  (in PyDSTool.Toolbox.syntheticdata) | compileFuncSpec()  (in ModelSpec) | copysign  (in PyDSTool.Toolbox.syntheticdata) | | ceil  (in PyDSTool.Toolbox.syntheticdata) | compileFuncSpec()  (in Quantity) | copysign  (in PyDSTool) | | ceil  (in matplotlib.pylab) | compileLib()  (in Dopri\_ODEsystem) | copysign  (in matplotlib.pylab) | | CenteredFiniteDifferences  (in PyDSTool.Toolbox.optimizers.helpers.finite\_difference) | compileLib()  (in Radau\_ODEsystem) | copyVarDict()  (in PyDSTool.common) | | CFGorder  (in PyDSTool.Toolbox.DSSRT\_tools) | Component  (in PyDSTool.ModelSpec') | corr\_dim()  (in PyDSTool.Toolbox.fracdim) | | change\_bgd()  (in KeyEvent) | composed\_map1D  (in PyDSTool.Toolbox.event\_driven\_simulator) | corr\_dim\_with\_progress()  (in PyDSTool.Toolbox.fracdim) | | change\_curr()  (in KeyEvent) | composite\_criteria  (in PyDSTool.Toolbox.optimizers.criterion) | Cos  (in PyDSTool) | | change\_fgd()  (in KeyEvent) | compute()  (in Dopri\_ODEsystem) | Cos  (in PyDSTool.ModelSpec') | | changeDomain()  (in GenTransform) | compute()  (in EmbeddedSysGen) | cos  (in PyDSTool.PyCont.ContClass') | | changeTargetGen()  (in GenTransform) | compute()  (in Euler\_ODEsystem) | Cos  (in PyDSTool.Symbolic) | | channel  (in PyDSTool.Toolbox.neuralcomp) | compute()  (in ExplicitFnGen) | cos  (in PyDSTool.Symbolic) | | channel\_off  (in PyDSTool.Toolbox.neuralcomp) | compute()  (in ExtrapolateTable) | Cos  (in PyDSTool.Toolbox.ActivationFuncs) | | channel\_on  (in PyDSTool.Toolbox.neuralcomp) | compute()  (in ImplicitFnGen) | Cos  (in PyDSTool.Toolbox.DSSRT\_tools) | | check()  (in genDBClass) | compute()  (in InterpolateTable) | Cos  (in PyDSTool.Toolbox.InputProfile) | | check\_arguments()  (in Optimizer) | compute()  (in LookupTable) | Cos  (in PyDSTool.Toolbox.ModelHelper) | | check\_bounds()  (in PyDSTool.Toolbox.phaseplane) | compute()  (in MapSystem) | Cos  (in PyDSTool.Toolbox.NineML) | | check\_damped\_gradient\_relative()  (in test\_Quadratic) | compute()  (in ODEsystem) | cos  (in PyDSTool.Toolbox.NineML) | | check\_opts()  (in PyDSTool.Toolbox.dssrt) | compute()  (in Radau\_ODEsystem) | Cos  (in PyDSTool.Toolbox.adjointPRC) | | check\_simple\_gradient()  (in test\_Quadratic) | compute()  (in Vode\_ODEsystem) | Cos  (in PyDSTool.Toolbox.dataanalysis) | | check\_simple\_gradient\_monotony()  (in test\_Rosenbrock) | compute()  (in HybridModel) | cos  (in PyDSTool.Toolbox.dataanalysis) | | check\_simple\_gradient\_relative()  (in test\_Rosenbrock) | compute()  (in NonHybridModel) | Cos  (in PyDSTool.Toolbox.fracdim) | | check\_simple\_marquardt()  (in test\_Quadratic) | compute\_traj()  (in intModelInterface) | Cos  (in PyDSTool.Toolbox.makeSloppyModel) | | check\_simple\_newton()  (in test\_Powell) | computeEigen()  (in ContClass) | Cos  (in PyDSTool.Toolbox.neuralcomp) | | check\_simple\_newton()  (in test\_Quadratic) | computeEigen()  (in Continuation) | Cos  (in PyDSTool.Toolbox.phaseplane) | | check\_simple\_newton\_relative()  (in test\_Rosenbrock) | computeFibonacci()  (in FibonacciSectionSearch) | cos  (in PyDSTool.Toolbox.phaseplane) | | check\_swp\_cwgradient\_relative()  (in test\_Quadratic) | concatStrDict()  (in PyDSTool.common) | Cos  (in PyDSTool.Toolbox.synthetic\_data) | | check\_swp\_dpgradient\_relative()  (in test\_Quadratic) | concavity()  (in nullcline) | cos  (in PyDSTool.Toolbox.synthetic\_data) | | check\_swp\_dypgradient\_relative()  (in test\_Quadratic) | concavity\_at\_sample\_points()  (in nullcline) | Cos  (in PyDSTool.Toolbox.syntheticdata) | | check\_swp\_frgradient\_relative()  (in test\_Quadratic) | condition  (in PyDSTool.MProject) | cos  (in PyDSTool.Toolbox.syntheticdata) | | check\_swp\_prpgradient\_relative()  (in test\_Quadratic) | conf  (in PyDSTool) | cos  (in matplotlib.pylab) | | check\_swpr\_dygradient()  (in test\_Rosenbrock) | conj  (in PyDSTool.PyCont.ContClass') | Cosh  (in PyDSTool) | | check\_wpr\_cwgradient()  (in test\_Rosenbrock) | conj  (in PyDSTool.Toolbox.ActivationFuncs) | Cosh  (in PyDSTool.ModelSpec') | | checkArgs()  (in Generator) | conj  (in PyDSTool.Toolbox.DSSRT\_tools) | cosh  (in PyDSTool.PyCont.ContClass') | | checkBasic()  (in integrator) | conj  (in PyDSTool.Toolbox.InputProfile) | Cosh  (in PyDSTool.Symbolic) | | checkbraces()  (in PyDSTool.Symbolic) | conj  (in PyDSTool.Toolbox.ModelHelper) | cosh  (in PyDSTool.Symbolic) | | checkEvents()  (in integrator) | conj  (in PyDSTool.Toolbox.NineML) | Cosh  (in PyDSTool.Toolbox.ActivationFuncs) | | checkExtInputs()  (in integrator) | conj  (in PyDSTool.Toolbox.adjointPRC) | Cosh  (in PyDSTool.Toolbox.DSSRT\_tools) | | CheckHopf()  (in PyDSTool.PyCont.misc) | conj  (in PyDSTool.Toolbox.dataanalysis) | Cosh  (in PyDSTool.Toolbox.InputProfile) | | checkInitialConditions()  (in MapSystem) | conj  (in PyDSTool.Toolbox.fracdim) | Cosh  (in PyDSTool.Toolbox.ModelHelper) | | checkInitialConditions()  (in ODEsystem) | conj  (in PyDSTool.Toolbox.makeSloppyModel) | Cosh  (in PyDSTool.Toolbox.NineML) | | checkInteg()  (in integrator) | conj  (in PyDSTool.Toolbox.neuralcomp) | cosh  (in PyDSTool.Toolbox.NineML) | | checkRunParams()  (in integrator) | conj  (in PyDSTool.Toolbox.phaseplane) | Cosh  (in PyDSTool.Toolbox.adjointPRC) | | Choice  (in PyDSTool) | conj  (in PyDSTool.Toolbox.synthetic\_data) | Cosh  (in PyDSTool.Toolbox.dataanalysis) | | Choice  (in PyDSTool.ModelSpec') | conj  (in PyDSTool.Toolbox.syntheticdata) | cosh  (in PyDSTool.Toolbox.dataanalysis) | | Choice  (in PyDSTool.Symbolic) | conj  (in PyDSTool) | Cosh  (in PyDSTool.Toolbox.fracdim) | | Choice  (in PyDSTool.Toolbox.ActivationFuncs) | conj  (in matplotlib.pylab) | Cosh  (in PyDSTool.Toolbox.makeSloppyModel) | | Choice  (in PyDSTool.Toolbox.DSSRT\_tools) | conjugate  (in PyDSTool.PyCont.ContClass') | Cosh  (in PyDSTool.Toolbox.neuralcomp) | | Choice  (in PyDSTool.Toolbox.InputProfile) | conjugate  (in PyDSTool.PyCont.misc) | Cosh  (in PyDSTool.Toolbox.phaseplane) | | Choice  (in PyDSTool.Toolbox.ModelHelper) | conjugate  (in PyDSTool.Toolbox.ActivationFuncs) | cosh  (in PyDSTool.Toolbox.phaseplane) | | Choice  (in PyDSTool.Toolbox.NineML) | conjugate  (in PyDSTool.Toolbox.DSSRT\_tools) | Cosh  (in PyDSTool.Toolbox.synthetic\_data) | | Choice  (in PyDSTool.Toolbox.adjointPRC) | conjugate  (in PyDSTool.Toolbox.InputProfile) | cosh  (in PyDSTool.Toolbox.synthetic\_data) | | Choice  (in PyDSTool.Toolbox.dataanalysis) | conjugate  (in PyDSTool.Toolbox.ModelHelper) | Cosh  (in PyDSTool.Toolbox.syntheticdata) | | Choice  (in PyDSTool.Toolbox.fracdim) | conjugate  (in PyDSTool.Toolbox.NineML) | cosh  (in PyDSTool.Toolbox.syntheticdata) | | Choice  (in PyDSTool.Toolbox.makeSloppyModel) | conjugate  (in PyDSTool.Toolbox.adjointPRC) | cosh  (in matplotlib.pylab) | | Choice  (in PyDSTool.Toolbox.neuralcomp) | conjugate  (in PyDSTool.Toolbox.dataanalysis) | count\_sep()  (in PyDSTool.parseUtils) | | Choice  (in PyDSTool.Toolbox.phaseplane) | conjugate  (in PyDSTool.Toolbox.fracdim) | CP\_Fold  (in PyDSTool.PyCont.TestFunc) | | Choice  (in PyDSTool.Toolbox.synthetic\_data) | conjugate  (in PyDSTool.Toolbox.makeSloppyModel) | cPickle | | Choice  (in PyDSTool.Toolbox.syntheticdata) | conjugate  (in PyDSTool.Toolbox.neuralcomp) | CPPoint  (in PyDSTool.PyCont.BifPoint) | | classmap  (in PyDSTool.fixedpickle) | conjugate  (in PyDSTool.Toolbox.phaseplane) | create\_test\_fn()  (in PyDSTool.Toolbox.phaseplane) | | className()  (in PyDSTool.common) | conjugate  (in PyDSTool.Toolbox.synthetic\_data) | create\_test\_fn\_with\_events()  (in PyDSTool.Toolbox.phaseplane) | | clean()  (in pargs) | conjugate  (in PyDSTool.Toolbox.syntheticdata) | createGenerators()  (in ModelConstructor) | | cleanLabels()  (in Continuation) | conjugate  (in PyDSTool) | createJac()  (in ModelConstructor) | | cleanupMemory()  (in ODEsystem) | conjugate  (in matplotlib.pylab) | createJacP()  (in ModelConstructor) | | cleanupMemory()  (in HybridModel) | conjugate\_gradient\_step  (in PyDSTool.Toolbox.optimizers.step) | createParameterization()  (in nullcline) | | cleanupMemory()  (in NonHybridModel) | ConjugateGradientStep  (in PyDSTool.Toolbox.optimizers.step.conjugate\_gradient\_step) | createQ()  (in Event) | | clear()  (in nameResolverClass) | connection  (in PyDSTool.Toolbox.event\_driven\_simulator) | createStdEvts()  (in ModelConstructor) | | clear()  (in pargs) | connectWithSynapse()  (in PyDSTool.Toolbox.neuralcomp) | criteria  (in PyDSTool.Toolbox.optimizers.criterion) | | clear()  (in data\_bins) | ConstraintFail  (in PyDSTool.Toolbox.ParamEst) | criterion  (in PyDSTool.Toolbox.optimizers) | | clear()  (in data\_bins) | cont\_args\_list  (in PyDSTool.PyCont.Continuation) | criterion()  (in PyDSTool.Toolbox.optimizers.criterion.facilities) | | clear()  (in args) | cont\_bif\_points  (in PyDSTool.PyCont.Continuation) | criterion\_\_all\_\_  (in PyDSTool.Toolbox.optimizers.criterion) | | clear()  (in auxfnDBclass) | contained  (in PyDSTool.Generator.ADMC\_ODEsystem') | crop()  (in nullcline) | | clear\_history()  (in distance\_to\_pointset) | contained  (in PyDSTool.Generator.Dopri\_ODEsystem') | crop\_2D()  (in PyDSTool.Toolbox.phaseplane) | | clear\_memo()  (in Pickler) | contained  (in PyDSTool.Generator.EmbeddedSysGen') | cross()  (in PyDSTool.Toolbox.mechmatlib) | | clearall()  (in genDBClass) | contained  (in PyDSTool.Generator.Euler\_ODEsystem') | ctn\_residual\_info()  (in PyDSTool.Toolbox.ParamEst) | | clearall()  (in nameResolverClass) | contained  (in PyDSTool.Generator.ExplicitFnGen') | ctsGen  (in PyDSTool.Generator.baseclasses) | | clearall()  (in pargs) | contained  (in PyDSTool.Generator.ExtrapolateTable') | cubic\_interpolation  (in PyDSTool.Toolbox.optimizers.line\_search) | | clearAll()  (in Diagnostics) | contained  (in PyDSTool.Generator.ImplicitFnGen') | CubicInterpolationSearch  (in PyDSTool.Toolbox.optimizers.line\_search.cubic\_interpolation) | | clearAll()  (in integrator) | contained  (in PyDSTool.Generator.InterpolateTable') | current\_defining\_args()  (in Model) | | clearall()  (in auxfnDBclass) | contained  (in PyDSTool.Generator.LookupTable') | curvature()  (in nullcline) | | clearErrors()  (in Diagnostics) | contained  (in PyDSTool.Generator.MapSystem') | curvature\_at\_sample\_points()  (in nullcline) | | clearEvents()  (in integrator) | contained  (in PyDSTool.Generator.ODEsystem') | curve\_args\_list  (in ContClass) | | clearExtInputs()  (in integrator) | contained  (in PyDSTool.Generator.Radau\_ODEsystem') | curve\_args\_list  (in PyDSTool.PyCont.ContClass') | | clearInteg()  (in integrator) | contained  (in PyDSTool.Generator.Vode\_ODEsystem') | curve\_list  (in ContClass) | | clearRunParams()  (in integrator) | contained  (in PyDSTool.Interval') | curve\_list  (in PyDSTool.PyCont.ContClass') | | clearWarnings()  (in Diagnostics) | contained  (in PyDSTool.Toolbox.NineML) | CWConjugateGradientStep()  (in PyDSTool.Toolbox.optimizers.step.conjugate\_gradient\_step) | | CLIP  (in PyDSTool) | contained  (in PyDSTool.Toolbox.dataanalysis) |  | | CLIP  (in PyDSTool.PyCont.ContClass') | contained  (in PyDSTool.Toolbox.phaseplane) |  | |

  
  

| Home | Trees | Indices | Help | | PyDSTool | | --- | |
| --- | --- | --- | --- | --- | --- |

|  |  |
| --- | --- |
| Generated by Epydoc 3.0.1 on Fri May 4 15:23:57 2012 | http://epydoc.sourceforge.net |
