# Supplementary material for: Hybrid Models and Biological Model Reduction with PyDSTool
Source: PLoS Comput Biol. 2012 Aug 9;8(8):e1002628. doi: 10.1371/journal.pcbi.1002628 (PMC3415397; doi:10.1371/journal.pcbi.1002628)
Supplement: Text S4 — Complete source code for the PyDSTool package (version 0.88.120504). Includes API documentation and help files linking to web pages. This file is identical to the current public release on Sourceforge.net. (ZIP) [file pcbi.1002628.s004.zip › PyDSTool/html/identifier-index-D.html]

xml version="1.0" encoding="ascii"?


Identifier Index


| Home | Trees | Indices | Help | | PyDSTool | | --- | |
| --- | --- | --- | --- | --- | --- |

|  |  |  |  |
| --- | --- | --- | --- |
|  | |  | | --- | | [hide private] | | [frames] | no frames] | |

|  |  |
| --- | --- |
| Identifier Index | [ A B C D E F G H I J K L M N O P Q R S T U V W X Y Z \_ ] |

|  |  |  |  |  |  |  |  |  |  |  |  |  |  |  |  |  |  |  |  |  |  |  |  |  |  |  |  |  |  |  |  |  |  |  |  |  |  |  |  |  |  |  |  |  |  |  |  |  |  |  |  |  |  |  |  |  |  |  |  |  |  |  |  |  |  |  |  |  |  |  |  |  |  |  |  |  |  |  |  |  |  |  |  |  |  |  |  |  |  |  |  |  |  |  |  |  |  |  |  |  |  |  |  |  |  |  |  |  |  |  |  |  |  |  |  |  |  |  |  |  |  |  |  |  |  |  |  |  |  |  |  |  |  |  |  |  |  |  |  |  |  |  |  |  |  |  |  |  |  |  |  |  |  |  |  |  |  |  |  |  |  |  |  |  |  |  |  |  |  |  |  |  |  |  |  |  |  |  |  |  |  |  |  |  |  |  |  |  |  |  |  |  |  |
| --- | --- | --- | --- | --- | --- | --- | --- | --- | --- | --- | --- | --- | --- | --- | --- | --- | --- | --- | --- | --- | --- | --- | --- | --- | --- | --- | --- | --- | --- | --- | --- | --- | --- | --- | --- | --- | --- | --- | --- | --- | --- | --- | --- | --- | --- | --- | --- | --- | --- | --- | --- | --- | --- | --- | --- | --- | --- | --- | --- | --- | --- | --- | --- | --- | --- | --- | --- | --- | --- | --- | --- | --- | --- | --- | --- | --- | --- | --- | --- | --- | --- | --- | --- | --- | --- | --- | --- | --- | --- | --- | --- | --- | --- | --- | --- | --- | --- | --- | --- | --- | --- | --- | --- | --- | --- | --- | --- | --- | --- | --- | --- | --- | --- | --- | --- | --- | --- | --- | --- | --- | --- | --- | --- | --- | --- | --- | --- | --- | --- | --- | --- | --- | --- | --- | --- | --- | --- | --- | --- | --- | --- | --- | --- | --- | --- | --- | --- | --- | --- | --- | --- | --- | --- | --- | --- | --- | --- | --- | --- | --- | --- | --- | --- | --- | --- | --- | --- | --- | --- | --- | --- | --- | --- | --- | --- | --- | --- | --- | --- | --- | --- | --- | --- | --- | --- | --- | --- | --- | --- | --- | --- | --- | --- |
| D | |  |  |  | | --- | --- | --- | | DAILY  (in PyDSTool.PyCont.ContClass') | derivative()  (in KroghInterpolator) | display()  (in simulator) | | DAILY  (in matplotlib.pylab) | derivative()  (in PiecewisePolynomial) | display\_raster()  (in simulator) | | damped\_line\_search  (in PyDSTool.Toolbox.optimizers.line\_search) | derivatives()  (in KroghInterpolator) | dist\_between\_datasets()  (in PyDSTool.Toolbox.data\_analysis) | | DampedLineSearch  (in PyDSTool.Toolbox.optimizers.line\_search.damped\_line\_search) | derivatives()  (in PiecewisePolynomial) | dist\_between\_datasets()  (in PyDSTool.Toolbox.dataanalysis) | | data\_analysis  (in PyDSTool.Toolbox) | derive\_angles()  (in mesh\_patch\_2D) | distance\_to\_pointset  (in PyDSTool.Toolbox.phaseplane) | | data\_bins  (in PyDSTool.Toolbox.data\_analysis) | Descriptor  (in PyDSTool.ModelConstructor') | distutil\_destination()  (in PyDSTool.Generator.Dopri\_ODEsystem') | | data\_bins  (in PyDSTool.Toolbox.dataanalysis) | determine\_if\_intersect()  (in PyDSTool.Toolbox.phaseplane) | distutil\_destination()  (in PyDSTool.Generator.Radau\_ODEsystem') | | dataanalysis  (in PyDSTool.Toolbox) | DFPNewtonStep  (in PyDSTool.Toolbox.optimizers.step.quasi\_newton\_step) | distutil\_destination()  (in PyDSTool.PyCont.ContClass') | | DConjugateGradientStep()  (in PyDSTool.Toolbox.optimizers.step.conjugate\_gradient\_step) | DH\_Hopf  (in PyDSTool.PyCont.TestFunc) | divide  (in PyDSTool.PyCont.ContClass') | | DDEsystem  (in PyDSTool.Generator) | DHPoint  (in PyDSTool.PyCont.BifPoint) | divide  (in PyDSTool.PyCont.Continuation) | | DDEsystem  (in PyDSTool.Generator.DDEsystem) | Diagnostics  (in PyDSTool.common) | divide  (in PyDSTool.PyCont.misc) | | decode\_long()  (in PyDSTool.fixedpickle) | DICT  (in PyDSTool.fixedpickle) | divide  (in PyDSTool.Toolbox.ActivationFuncs) | | DefaultDict  (in PyDSTool.common) | dict\_tol  (in PyDSTool.Toolbox.data\_analysis) | divide  (in PyDSTool.Toolbox.DSSRT\_tools) | | defaults  (in PyDSTool.Toolbox.optimizers) | dict\_tol  (in PyDSTool.Toolbox.dataanalysis) | divide  (in PyDSTool.Toolbox.InputProfile) | | defaultVars()  (in Model) | diff()  (in AddTestFunction) | divide  (in PyDSTool.Toolbox.ModelHelper) | | define\_psi\_events()  (in PyDSTool.Toolbox.dssrt) | diff()  (in DiscreteMap) | divide  (in PyDSTool.Toolbox.NineML) | | define\_tau\_events()  (in PyDSTool.Toolbox.dssrt) | diff()  (in FixedPointMap) | divide  (in PyDSTool.Toolbox.adjointPRC) | | deg2rad  (in PyDSTool.PyCont.ContClass') | diff()  (in Fold\_Bor) | divide  (in PyDSTool.Toolbox.dataanalysis) | | deg2rad  (in PyDSTool.Toolbox.ActivationFuncs) | diff()  (in Function) | divide  (in PyDSTool.Toolbox.fracdim) | | deg2rad  (in PyDSTool.Toolbox.DSSRT\_tools) | diff()  (in Hopf\_Bor) | divide  (in PyDSTool.Toolbox.makeSloppyModel) | | deg2rad  (in PyDSTool.Toolbox.InputProfile) | diff()  (in TestFunc) | divide  (in PyDSTool.Toolbox.neuralcomp) | | deg2rad  (in PyDSTool.Toolbox.ModelHelper) | Diff()  (in PyDSTool.Symbolic) | divide  (in PyDSTool.Toolbox.phaseplane) | | deg2rad  (in PyDSTool.Toolbox.NineML) | diff()  (in PyDSTool.common) | divide  (in PyDSTool.Toolbox.synthetic\_data) | | deg2rad  (in PyDSTool.Toolbox.adjointPRC) | diff2()  (in PyDSTool.common) | divide  (in PyDSTool.Toolbox.syntheticdata) | | deg2rad  (in PyDSTool.Toolbox.dataanalysis) | difference()  (in ModelSpec) | divide  (in PyDSTool) | | deg2rad  (in PyDSTool.Toolbox.fracdim) | difference()  (in QuantSpec) | divide  (in matplotlib.pylab) | | deg2rad  (in PyDSTool.Toolbox.makeSloppyModel) | difference()  (in Quantity) | do\_2Ddirn()  (in PyDSTool.Toolbox.ParamEst) | | deg2rad  (in PyDSTool.Toolbox.neuralcomp) | DiffStr()  (in PyDSTool.Symbolic) | do\_2Dstep()  (in PyDSTool.Toolbox.ParamEst) | | deg2rad  (in PyDSTool.Toolbox.phaseplane) | discGen  (in PyDSTool.Generator.baseclasses) | DO\_DEC  (in PyDSTool.parseUtils) | | deg2rad  (in PyDSTool.Toolbox.synthetic\_data) | disconnectSynapse()  (in PyDSTool.Toolbox.neuralcomp) | DO\_POW  (in PyDSTool.parseUtils) | | deg2rad  (in PyDSTool.Toolbox.syntheticdata) | Discrete  (in PyDSTool.FuncSpec') | do\_stats()  (in PyDSTool.Toolbox.fracdim) | | deg2rad  (in PyDSTool) | Discrete  (in PyDSTool.Generator.ADMC\_ODEsystem') | doadd()  (in PyDSTool.parseUtils) | | deg2rad  (in matplotlib.pylab) | Discrete  (in PyDSTool.Generator.Dopri\_ODEsystem') | dodiv()  (in PyDSTool.parseUtils) | | Degrees  (in PyDSTool) | Discrete  (in PyDSTool.Generator.EmbeddedSysGen') | dofun()  (in PyDSTool.Symbolic) | | Degrees  (in PyDSTool.ModelSpec') | Discrete  (in PyDSTool.Generator.Euler\_ODEsystem') | domain\_test  (in PyDSTool.Model) | | degrees  (in PyDSTool.PyCont.ContClass') | Discrete  (in PyDSTool.Generator.ExplicitFnGen') | DomainType  (in PyDSTool.common) | | Degrees  (in PyDSTool.Symbolic) | Discrete  (in PyDSTool.Generator.ExtrapolateTable') | domscales  (in PyDSTool.Toolbox.dssrt) | | Degrees  (in PyDSTool.Toolbox.ActivationFuncs) | Discrete  (in PyDSTool.Generator.ImplicitFnGen') | domul()  (in PyDSTool.parseUtils) | | Degrees  (in PyDSTool.Toolbox.DSSRT\_tools) | Discrete  (in PyDSTool.Generator.InterpolateTable') | doneg()  (in PyDSTool.parseUtils) | | Degrees  (in PyDSTool.Toolbox.InputProfile) | Discrete  (in PyDSTool.Generator.LookupTable') | doPCA()  (in PyDSTool.Toolbox.data\_analysis) | | Degrees  (in PyDSTool.Toolbox.ModelHelper) | Discrete  (in PyDSTool.Generator.MapSystem') | doPCA()  (in PyDSTool.Toolbox.dataanalysis) | | Degrees  (in PyDSTool.Toolbox.NineML) | Discrete  (in PyDSTool.Generator.ODEsystem') | dopower()  (in PyDSTool.parseUtils) | | degrees  (in PyDSTool.Toolbox.NineML) | Discrete  (in PyDSTool.Generator.Radau\_ODEsystem') | doPreMacros()  (in FuncSpec) | | Degrees  (in PyDSTool.Toolbox.adjointPRC) | Discrete  (in PyDSTool.Generator.Vode\_ODEsystem') | dopri  (in PyDSTool.Generator.Dopri\_ODEsystem') | | Degrees  (in PyDSTool.Toolbox.dataanalysis) | Discrete  (in PyDSTool.Interval') | Dopri\_ODEsystem  (in PyDSTool.Generator.Dopri\_ODEsystem') | | degrees  (in PyDSTool.Toolbox.dataanalysis) | Discrete  (in PyDSTool.ModelConstructor') | Dopri\_ODEsystem'  (in PyDSTool.Generator) | | Degrees  (in PyDSTool.Toolbox.fracdim) | Discrete  (in PyDSTool.ModelSpec') | dosub()  (in PyDSTool.parseUtils) | | Degrees  (in PyDSTool.Toolbox.makeSloppyModel) | Discrete  (in PyDSTool.Symbolic) | dot()  (in PyDSTool.Toolbox.mechmatlib) | | Degrees  (in PyDSTool.Toolbox.neuralcomp) | Discrete  (in PyDSTool.Toolbox.NineML) | dsInterface  (in PyDSTool.MProject) | | Degrees  (in PyDSTool.Toolbox.phaseplane) | Discrete  (in PyDSTool.Toolbox.dataanalysis) | dssrt  (in PyDSTool.Toolbox) | | degrees  (in PyDSTool.Toolbox.phaseplane) | Discrete  (in PyDSTool.Toolbox.event\_driven\_simulator) | dssrt\_assistant  (in PyDSTool.Toolbox.dssrt) | | Degrees  (in PyDSTool.Toolbox.synthetic\_data) | Discrete  (in PyDSTool.Toolbox.phaseplane) | DSSRT\_info  (in PyDSTool.Toolbox.DSSRT\_tools) | | degrees  (in PyDSTool.Toolbox.synthetic\_data) | Discrete  (in PyDSTool.Toolbox.synthetic\_data) | DSSRT\_tools  (in PyDSTool.Toolbox) | | Degrees  (in PyDSTool.Toolbox.syntheticdata) | Discrete  (in PyDSTool.Toolbox.syntheticdata) | dump()  (in Pickler) | | degrees  (in PyDSTool.Toolbox.syntheticdata) | Discrete  (in PyDSTool.Trajectory') | dump()  (in PyDSTool.fixedpickle) | | degrees  (in matplotlib.pylab) | Discrete  (in PyDSTool.Variable') | dump\_progress()  (in PyDSTool.Toolbox.data\_analysis) | | delay\_map  (in PyDSTool.Toolbox.event\_driven\_simulator) | Discrete  (in PyDSTool.common) | dump\_progress()  (in PyDSTool.Toolbox.dataanalysis) | | delConnxnTarget()  (in ModelSpec) | Discrete  (in PyDSTool.parseUtils) | dumps()  (in PyDSTool.fixedpickle) | | delCurve()  (in ContClass) | Discrete  (in PyDSTool.utils) | dumpTrajData()  (in DSSRT\_info) | | delete()  (in pargs) | DiscreteMap  (in PyDSTool.PyCont.TestFunc) | DUP  (in PyDSTool.fixedpickle) | | delete\_variables()  (in Trajectory) | dispatch  (in Pickler) | dvode  (in scipy.integrate.vode) | | deleteall()  (in pargs) | dispatch  (in Unpickler) | dx\_scaled\_2D  (in PyDSTool.Toolbox.phaseplane) | | deleteQ()  (in Event) | display()  (in ContClass) | DYConjugateGradientStep()  (in PyDSTool.Toolbox.optimizers.step.conjugate\_gradient\_step) | | dendr\_compartment  (in PyDSTool.Toolbox.neuralcomp) | display()  (in Continuation) |  | |

  
  

| Home | Trees | Indices | Help | | PyDSTool | | --- | |
| --- | --- | --- | --- | --- | --- |

|  |  |
| --- | --- |
| Generated by Epydoc 3.0.1 on Fri May 4 15:23:57 2012 | http://epydoc.sourceforge.net |
