# Supplementary material for: Hybrid Models and Biological Model Reduction with PyDSTool
Source: PLoS Comput Biol. 2012 Aug 9;8(8):e1002628. doi: 10.1371/journal.pcbi.1002628 (PMC3415397; doi:10.1371/journal.pcbi.1002628)
Supplement: Text S4 — Complete source code for the PyDSTool package (version 0.88.120504). Includes API documentation and help files linking to web pages. This file is identical to the current public release on Sourceforge.net. (ZIP) [file pcbi.1002628.s004.zip › PyDSTool/html/identifier-index-E.html]

xml version="1.0" encoding="ascii"?


Identifier Index


| Home | Trees | Indices | Help | | PyDSTool | | --- | |
| --- | --- | --- | --- | --- | --- |

|  |  |  |  |
| --- | --- | --- | --- |
|  | |  | | --- | | [hide private] | | [frames] | no frames] | |

|  |  |
| --- | --- |
| Identifier Index | [ A B C D E F G H I J K L M N O P Q R S T U V W X Y Z \_ ] |

|  |  |  |  |  |  |  |  |  |  |  |  |  |  |  |  |  |  |  |  |  |  |  |  |  |  |  |  |  |  |  |  |  |  |  |  |  |  |  |  |  |  |  |  |  |  |  |  |  |  |  |  |  |  |  |  |  |  |  |  |  |  |  |  |  |  |  |  |  |  |  |  |  |  |  |  |  |  |  |  |  |  |  |  |  |  |  |  |  |  |  |  |  |  |  |  |  |  |  |  |  |  |  |  |  |  |  |  |  |  |  |  |  |  |  |  |  |  |  |  |  |  |  |  |  |  |  |  |  |  |  |  |  |  |  |  |  |  |  |  |  |  |  |  |  |  |  |  |  |  |  |  |  |  |  |  |  |  |  |  |  |  |  |  |  |  |  |  |  |  |  |  |  |  |  |  |  |  |  |  |  |  |  |  |  |  |  |  |  |  |  |  |  |  |  |  |  |  |  |  |  |  |  |  |  |  |  |  |  |  |  |  |  |  |  |  |  |  |  |  |  |  |  |  |  |  |  |  |  |  |  |  |  |  |  |  |  |  |  |  |  |  |  |  |  |  |  |  |  |  |  |  |  |  |  |  |  |  |  |  |  |  |  |  |  |  |  |  |  |  |  |  |  |  |  |  |  |  |  |  |  |  |  |  |  |  |  |  |  |  |  |  |  |  |  |  |  |  |  |  |  |  |  |  |  |  |  |  |  |  |  |  |  |  |  |  |  |  |  |  |  |  |  |  |  |  |  |  |  |  |  |  |  |  |  |  |  |  |  |  |  |  |  |  |  |  |  |  |  |  |  |  |  |  |  |  |  |  |  |  |  |  |  |  |  |  |  |  |  |  |  |  |  |  |  |  |  |  |  |  |  |  |  |  |  |  |  |  |  |  |  |  |  |  |  |  |  |  |  |  |  |  |  |  |  |  |  |  |  |  |  |  |  |  |  |  |  |  |  |  |  |  |  |  |  |  |  |  |  |  |  |  |  |  |  |  |  |  |  |  |  |  |  |  |  |  |  |  |  |  |  |  |  |  |  |  |  |  |
| --- | --- | --- | --- | --- | --- | --- | --- | --- | --- | --- | --- | --- | --- | --- | --- | --- | --- | --- | --- | --- | --- | --- | --- | --- | --- | --- | --- | --- | --- | --- | --- | --- | --- | --- | --- | --- | --- | --- | --- | --- | --- | --- | --- | --- | --- | --- | --- | --- | --- | --- | --- | --- | --- | --- | --- | --- | --- | --- | --- | --- | --- | --- | --- | --- | --- | --- | --- | --- | --- | --- | --- | --- | --- | --- | --- | --- | --- | --- | --- | --- | --- | --- | --- | --- | --- | --- | --- | --- | --- | --- | --- | --- | --- | --- | --- | --- | --- | --- | --- | --- | --- | --- | --- | --- | --- | --- | --- | --- | --- | --- | --- | --- | --- | --- | --- | --- | --- | --- | --- | --- | --- | --- | --- | --- | --- | --- | --- | --- | --- | --- | --- | --- | --- | --- | --- | --- | --- | --- | --- | --- | --- | --- | --- | --- | --- | --- | --- | --- | --- | --- | --- | --- | --- | --- | --- | --- | --- | --- | --- | --- | --- | --- | --- | --- | --- | --- | --- | --- | --- | --- | --- | --- | --- | --- | --- | --- | --- | --- | --- | --- | --- | --- | --- | --- | --- | --- | --- | --- | --- | --- | --- | --- | --- | --- | --- | --- | --- | --- | --- | --- | --- | --- | --- | --- | --- | --- | --- | --- | --- | --- | --- | --- | --- | --- | --- | --- | --- | --- | --- | --- | --- | --- | --- | --- | --- | --- | --- | --- | --- | --- | --- | --- | --- | --- | --- | --- | --- | --- | --- | --- | --- | --- | --- | --- | --- | --- | --- | --- | --- | --- | --- | --- | --- | --- | --- | --- | --- | --- | --- | --- | --- | --- | --- | --- | --- | --- | --- | --- | --- | --- | --- | --- | --- | --- | --- | --- | --- | --- | --- | --- | --- | --- | --- | --- | --- | --- | --- | --- | --- | --- | --- | --- | --- | --- | --- | --- | --- | --- | --- | --- | --- | --- | --- | --- | --- | --- | --- | --- | --- | --- | --- | --- | --- | --- | --- | --- | --- | --- | --- | --- | --- | --- | --- | --- | --- | --- | --- | --- | --- | --- | --- | --- | --- | --- | --- | --- | --- | --- | --- | --- | --- | --- | --- | --- | --- | --- | --- | --- | --- | --- | --- | --- | --- | --- | --- | --- | --- | --- | --- | --- | --- | --- | --- | --- | --- | --- | --- | --- | --- | --- | --- | --- | --- | --- | --- | --- | --- | --- | --- | --- | --- | --- | --- | --- | --- | --- | --- | --- | --- | --- | --- | --- | --- | --- | --- | --- | --- | --- | --- | --- | --- | --- | --- | --- | --- | --- | --- | --- | --- | --- | --- | --- | --- | --- | --- | --- | --- | --- | --- | --- | --- | --- | --- | --- | --- | --- | --- | --- | --- | --- | --- | --- | --- | --- | --- | --- | --- | --- | --- | --- | --- | --- | --- | --- | --- | --- | --- | --- | --- | --- | --- | --- | --- | --- | --- | --- | --- |
| E | |  |  |  | | --- | --- | --- | | E  (in PyDSTool) | ERR\_DEFAULT  (in PyDSTool.Toolbox.syntheticdata) | evaluate()  (in feature\_node) | | e  (in PyDSTool.Generator.ADMC\_ODEsystem') | ERR\_DEFAULT  (in matplotlib.pylab) | evaluate()  (in boundary\_containment\_by\_event) | | e  (in PyDSTool.Generator.DDEsystem) | ERR\_DEFAULT2  (in PyDSTool) | evaluate()  (in boundary\_containment\_by\_postproc) | | e  (in PyDSTool.Generator.Dopri\_ODEsystem') | ERR\_DEFAULT2  (in PyDSTool.PyCont.ContClass') | evaluate()  (in domain\_test) | | e  (in PyDSTool.Generator.EmbeddedSysGen') | ERR\_DEFAULT2  (in PyDSTool.Toolbox.ActivationFuncs) | evaluate()  (in L2\_feature) | | e  (in PyDSTool.Generator.Euler\_ODEsystem') | ERR\_DEFAULT2  (in PyDSTool.Toolbox.DSSRT\_tools) | evaluate()  (in L2\_feature\_1D) | | e  (in PyDSTool.Generator.ExplicitFnGen') | ERR\_DEFAULT2  (in PyDSTool.Toolbox.InputProfile) | evaluate()  (in ParamEst) | | e  (in PyDSTool.Generator.ExtrapolateTable') | ERR\_DEFAULT2  (in PyDSTool.Toolbox.ModelHelper) | evaluate()  (in become\_most\_dominant) | | e  (in PyDSTool.Generator.ImplicitFnGen') | ERR\_DEFAULT2  (in PyDSTool.Toolbox.NineML) | evaluate()  (in is\_active) | | e  (in PyDSTool.Generator.InterpolateTable') | ERR\_DEFAULT2  (in PyDSTool.Toolbox.adjointPRC) | evaluate()  (in is\_fast) | | e  (in PyDSTool.Generator.LookupTable') | ERR\_DEFAULT2  (in PyDSTool.Toolbox.dataanalysis) | evaluate()  (in is\_inactive) | | e  (in PyDSTool.Generator.MapSystem') | ERR\_DEFAULT2  (in PyDSTool.Toolbox.fracdim) | evaluate()  (in is\_modulatory) | | e  (in PyDSTool.Generator.ODEsystem') | ERR\_DEFAULT2  (in PyDSTool.Toolbox.makeSloppyModel) | evaluate()  (in is\_most\_dominant) | | e  (in PyDSTool.Generator.Radau\_ODEsystem') | ERR\_DEFAULT2  (in PyDSTool.Toolbox.neuralcomp) | evaluate()  (in is\_order1) | | e  (in PyDSTool.Generator.Vode\_ODEsystem') | ERR\_DEFAULT2  (in PyDSTool.Toolbox.phaseplane) | evaluate()  (in is\_slow) | | e  (in PyDSTool.Generator.allimports) | ERR\_DEFAULT2  (in PyDSTool.Toolbox.synthetic\_data) | evaluate()  (in join\_actives) | | e  (in PyDSTool.Generator.baseclasses) | ERR\_DEFAULT2  (in PyDSTool.Toolbox.syntheticdata) | evaluate()  (in join\_fast) | | e  (in PyDSTool.Generator) | ERR\_DEFAULT2  (in matplotlib.pylab) | evaluate()  (in join\_slow) | | E  (in PyDSTool.ModelSpec') | ERR\_IGNORE  (in PyDSTool) | evaluate()  (in leave\_actives) | | e  (in PyDSTool.ModelSpec') | ERR\_IGNORE  (in PyDSTool.PyCont.ContClass') | evaluate()  (in leave\_fast) | | e  (in PyDSTool.PyCont.ContClass') | ERR\_IGNORE  (in PyDSTool.Toolbox.ActivationFuncs) | evaluate()  (in leave\_slow) | | E  (in PyDSTool.Symbolic) | ERR\_IGNORE  (in PyDSTool.Toolbox.DSSRT\_tools) | evaluate()  (in geom\_feature) | | e  (in PyDSTool.Symbolic) | ERR\_IGNORE  (in PyDSTool.Toolbox.InputProfile) | evaluate()  (in get\_burst\_active\_phase) | | E  (in PyDSTool.Toolbox.ActivationFuncs) | ERR\_IGNORE  (in PyDSTool.Toolbox.ModelHelper) | evaluate()  (in get\_burst\_dc\_offset) | | E  (in PyDSTool.Toolbox.DSSRT\_tools) | ERR\_IGNORE  (in PyDSTool.Toolbox.NineML) | evaluate()  (in get\_burst\_downsweep) | | E  (in PyDSTool.Toolbox.InputProfile) | ERR\_IGNORE  (in PyDSTool.Toolbox.adjointPRC) | evaluate()  (in get\_burst\_duration) | | E  (in PyDSTool.Toolbox.ModelHelper) | ERR\_IGNORE  (in PyDSTool.Toolbox.dataanalysis) | evaluate()  (in get\_burst\_isi\_env) | | E  (in PyDSTool.Toolbox.NineML) | ERR\_IGNORE  (in PyDSTool.Toolbox.fracdim) | evaluate()  (in get\_burst\_num\_spikes) | | e  (in PyDSTool.Toolbox.NineML) | ERR\_IGNORE  (in PyDSTool.Toolbox.makeSloppyModel) | evaluate()  (in get\_burst\_passive\_extent) | | E  (in PyDSTool.Toolbox.adjointPRC) | ERR\_IGNORE  (in PyDSTool.Toolbox.neuralcomp) | evaluate()  (in get\_burst\_peak\_env) | | E  (in PyDSTool.Toolbox.dataanalysis) | ERR\_IGNORE  (in PyDSTool.Toolbox.phaseplane) | evaluate()  (in get\_burst\_period\_info) | | e  (in PyDSTool.Toolbox.dataanalysis) | ERR\_IGNORE  (in PyDSTool.Toolbox.synthetic\_data) | evaluate()  (in get\_burst\_spikes) | | E  (in PyDSTool.Toolbox.fracdim) | ERR\_IGNORE  (in PyDSTool.Toolbox.syntheticdata) | evaluate()  (in get\_burst\_trough\_env) | | E  (in PyDSTool.Toolbox.makeSloppyModel) | ERR\_IGNORE  (in matplotlib.pylab) | evaluate()  (in get\_burst\_upsweep) | | E  (in PyDSTool.Toolbox.neuralcomp) | ERR\_LOG  (in PyDSTool) | evaluate()  (in get\_spike\_data) | | E  (in PyDSTool.Toolbox.phaseplane) | ERR\_LOG  (in PyDSTool.PyCont.ContClass') | evaluate()  (in get\_spike\_model) | | e  (in PyDSTool.Toolbox.phaseplane) | ERR\_LOG  (in PyDSTool.Toolbox.ActivationFuncs) | evaluate()  (in spike\_feature) | | E  (in PyDSTool.Toolbox.synthetic\_data) | ERR\_LOG  (in PyDSTool.Toolbox.DSSRT\_tools) | evaluate()  (in inflection\_zone\_leaf) | | e  (in PyDSTool.Toolbox.synthetic\_data) | ERR\_LOG  (in PyDSTool.Toolbox.InputProfile) | evaluate()  (in inflection\_zone\_node) | | E  (in PyDSTool.Toolbox.syntheticdata) | ERR\_LOG  (in PyDSTool.Toolbox.ModelHelper) | evaluate()  (in max\_curvature\_zone\_leaf) | | e  (in PyDSTool.Toolbox.syntheticdata) | ERR\_LOG  (in PyDSTool.Toolbox.NineML) | evaluate()  (in max\_curvature\_zone\_node) | | e  (in matplotlib.pylab) | ERR\_LOG  (in PyDSTool.Toolbox.adjointPRC) | evaluate()  (in zone\_leaf) | | E\_COMPUTFAIL  (in PyDSTool.Generator.ADMC\_ODEsystem') | ERR\_LOG  (in PyDSTool.Toolbox.dataanalysis) | evaluate()  (in and\_op) | | E\_COMPUTFAIL  (in PyDSTool.Generator.Dopri\_ODEsystem') | ERR\_LOG  (in PyDSTool.Toolbox.fracdim) | evaluate()  (in not\_op) | | E\_COMPUTFAIL  (in PyDSTool.Generator.EmbeddedSysGen') | ERR\_LOG  (in PyDSTool.Toolbox.makeSloppyModel) | evaluate()  (in null\_predicate\_class) | | E\_COMPUTFAIL  (in PyDSTool.Generator.Euler\_ODEsystem') | ERR\_LOG  (in PyDSTool.Toolbox.neuralcomp) | evaluate()  (in or\_op) | | E\_COMPUTFAIL  (in PyDSTool.Generator.ExplicitFnGen') | ERR\_LOG  (in PyDSTool.Toolbox.phaseplane) | evaluate()  (in predicate) | | E\_COMPUTFAIL  (in PyDSTool.Generator.ExtrapolateTable') | ERR\_LOG  (in PyDSTool.Toolbox.synthetic\_data) | evaluate()  (in predicate\_op) | | E\_COMPUTFAIL  (in PyDSTool.Generator.ImplicitFnGen') | ERR\_LOG  (in PyDSTool.Toolbox.syntheticdata) | evec1\_standard()  (in PyDSTool.PyCont.misc) | | E\_COMPUTFAIL  (in PyDSTool.Generator.InterpolateTable') | ERR\_LOG  (in matplotlib.pylab) | Event  (in PyDSTool.Events) | | E\_COMPUTFAIL  (in PyDSTool.Generator.LookupTable') | ERR\_PRINT  (in PyDSTool) | event\_driven\_simulator  (in PyDSTool.Toolbox) | | E\_COMPUTFAIL  (in PyDSTool.Generator.MapSystem') | ERR\_PRINT  (in PyDSTool.PyCont.ContClass') | Events  (in PyDSTool) | | E\_COMPUTFAIL  (in PyDSTool.Generator.ODEsystem') | ERR\_PRINT  (in PyDSTool.Toolbox.ActivationFuncs) | EventStruct  (in PyDSTool.Events) | | E\_COMPUTFAIL  (in PyDSTool.Generator.Radau\_ODEsystem') | ERR\_PRINT  (in PyDSTool.Toolbox.DSSRT\_tools) | EvMapping  (in PyDSTool.ModelConstructor') | | E\_COMPUTFAIL  (in PyDSTool.Generator.Vode\_ODEsystem') | ERR\_PRINT  (in PyDSTool.Toolbox.InputProfile) | exc\_synapse  (in PyDSTool.Toolbox.neuralcomp) | | E\_COMPUTFAIL  (in PyDSTool.Generator.messagecodes) | ERR\_PRINT  (in PyDSTool.Toolbox.ModelHelper) | ExceptionFSM  (in PyDSTool.Toolbox.FSM) | | E\_COMPUTFAIL  (in PyDSTool.Toolbox.NineML) | ERR\_PRINT  (in PyDSTool.Toolbox.NineML) | exclude\_patterns  (in PyDSTool.conf) | | E\_COMPUTFAIL  (in PyDSTool.Toolbox.dataanalysis) | ERR\_PRINT  (in PyDSTool.Toolbox.adjointPRC) | Exp  (in PyDSTool) | | E\_COMPUTFAIL  (in PyDSTool.Toolbox.phaseplane) | ERR\_PRINT  (in PyDSTool.Toolbox.dataanalysis) | Exp  (in PyDSTool.ModelSpec') | | E\_COMPUTFAIL  (in PyDSTool.Toolbox.synthetic\_data) | ERR\_PRINT  (in PyDSTool.Toolbox.fracdim) | exp  (in PyDSTool.PyCont.ContClass') | | E\_COMPUTFAIL  (in PyDSTool.Toolbox.syntheticdata) | ERR\_PRINT  (in PyDSTool.Toolbox.makeSloppyModel) | Exp  (in PyDSTool.Symbolic) | | E\_NONUNIQUETERM  (in PyDSTool.Generator.ADMC\_ODEsystem') | ERR\_PRINT  (in PyDSTool.Toolbox.neuralcomp) | exp  (in PyDSTool.Symbolic) | | E\_NONUNIQUETERM  (in PyDSTool.Generator.Dopri\_ODEsystem') | ERR\_PRINT  (in PyDSTool.Toolbox.phaseplane) | Exp  (in PyDSTool.Toolbox.ActivationFuncs) | | E\_NONUNIQUETERM  (in PyDSTool.Generator.EmbeddedSysGen') | ERR\_PRINT  (in PyDSTool.Toolbox.synthetic\_data) | Exp  (in PyDSTool.Toolbox.DSSRT\_tools) | | E\_NONUNIQUETERM  (in PyDSTool.Generator.Euler\_ODEsystem') | ERR\_PRINT  (in PyDSTool.Toolbox.syntheticdata) | Exp  (in PyDSTool.Toolbox.InputProfile) | | E\_NONUNIQUETERM  (in PyDSTool.Generator.ExplicitFnGen') | ERR\_PRINT  (in matplotlib.pylab) | Exp  (in PyDSTool.Toolbox.ModelHelper) | | E\_NONUNIQUETERM  (in PyDSTool.Generator.ExtrapolateTable') | ERR\_RAISE  (in PyDSTool) | Exp  (in PyDSTool.Toolbox.NineML) | | E\_NONUNIQUETERM  (in PyDSTool.Generator.ImplicitFnGen') | ERR\_RAISE  (in PyDSTool.PyCont.ContClass') | exp  (in PyDSTool.Toolbox.NineML) | | E\_NONUNIQUETERM  (in PyDSTool.Generator.InterpolateTable') | ERR\_RAISE  (in PyDSTool.Toolbox.ActivationFuncs) | Exp  (in PyDSTool.Toolbox.adjointPRC) | | E\_NONUNIQUETERM  (in PyDSTool.Generator.LookupTable') | ERR\_RAISE  (in PyDSTool.Toolbox.DSSRT\_tools) | Exp  (in PyDSTool.Toolbox.dataanalysis) | | E\_NONUNIQUETERM  (in PyDSTool.Generator.MapSystem') | ERR\_RAISE  (in PyDSTool.Toolbox.InputProfile) | exp  (in PyDSTool.Toolbox.dataanalysis) | | E\_NONUNIQUETERM  (in PyDSTool.Generator.ODEsystem') | ERR\_RAISE  (in PyDSTool.Toolbox.ModelHelper) | Exp  (in PyDSTool.Toolbox.fracdim) | | E\_NONUNIQUETERM  (in PyDSTool.Generator.Radau\_ODEsystem') | ERR\_RAISE  (in PyDSTool.Toolbox.NineML) | Exp  (in PyDSTool.Toolbox.makeSloppyModel) | | E\_NONUNIQUETERM  (in PyDSTool.Generator.Vode\_ODEsystem') | ERR\_RAISE  (in PyDSTool.Toolbox.adjointPRC) | Exp  (in PyDSTool.Toolbox.neuralcomp) | | E\_NONUNIQUETERM  (in PyDSTool.Generator.messagecodes) | ERR\_RAISE  (in PyDSTool.Toolbox.dataanalysis) | Exp  (in PyDSTool.Toolbox.phaseplane) | | E\_NONUNIQUETERM  (in PyDSTool.Toolbox.NineML) | ERR\_RAISE  (in PyDSTool.Toolbox.fracdim) | exp  (in PyDSTool.Toolbox.phaseplane) | | E\_NONUNIQUETERM  (in PyDSTool.Toolbox.dataanalysis) | ERR\_RAISE  (in PyDSTool.Toolbox.makeSloppyModel) | Exp  (in PyDSTool.Toolbox.synthetic\_data) | | E\_NONUNIQUETERM  (in PyDSTool.Toolbox.phaseplane) | ERR\_RAISE  (in PyDSTool.Toolbox.neuralcomp) | exp  (in PyDSTool.Toolbox.synthetic\_data) | | E\_NONUNIQUETERM  (in PyDSTool.Toolbox.synthetic\_data) | ERR\_RAISE  (in PyDSTool.Toolbox.phaseplane) | Exp  (in PyDSTool.Toolbox.syntheticdata) | | E\_NONUNIQUETERM  (in PyDSTool.Toolbox.syntheticdata) | ERR\_RAISE  (in PyDSTool.Toolbox.synthetic\_data) | exp  (in PyDSTool.Toolbox.syntheticdata) | | edge  (in PyDSTool.Toolbox.FR) | ERR\_RAISE  (in PyDSTool.Toolbox.syntheticdata) | exp  (in PyDSTool.common) | | editdist\_edits()  (in PyDSTool.Toolbox.dssrt) | ERR\_RAISE  (in matplotlib.pylab) | exp  (in matplotlib.pylab) | | eliminate\_group()  (in estimate\_spiking) | ERR\_WARN  (in PyDSTool) | exp2  (in PyDSTool.PyCont.ContClass') | | embed()  (in PyDSTool.ModelConstructor') | ERR\_WARN  (in PyDSTool.PyCont.ContClass') | exp2  (in PyDSTool.Toolbox.ActivationFuncs) | | EmbeddedSysGen  (in PyDSTool.Generator.EmbeddedSysGen') | ERR\_WARN  (in PyDSTool.Toolbox.ActivationFuncs) | exp2  (in PyDSTool.Toolbox.DSSRT\_tools) | | EmbeddedSysGen'  (in PyDSTool.Generator) | ERR\_WARN  (in PyDSTool.Toolbox.DSSRT\_tools) | exp2  (in PyDSTool.Toolbox.InputProfile) | | EMPTY\_DICT  (in PyDSTool.fixedpickle) | ERR\_WARN  (in PyDSTool.Toolbox.InputProfile) | exp2  (in PyDSTool.Toolbox.ModelHelper) | | EMPTY\_LIST  (in PyDSTool.fixedpickle) | ERR\_WARN  (in PyDSTool.Toolbox.ModelHelper) | exp2  (in PyDSTool.Toolbox.NineML) | | EMPTY\_TUPLE  (in PyDSTool.fixedpickle) | ERR\_WARN  (in PyDSTool.Toolbox.NineML) | exp2  (in PyDSTool.Toolbox.adjointPRC) | | encode\_long()  (in PyDSTool.fixedpickle) | ERR\_WARN  (in PyDSTool.Toolbox.adjointPRC) | exp2  (in PyDSTool.Toolbox.dataanalysis) | | ensure()  (in dssrt\_assistant) | ERR\_WARN  (in PyDSTool.Toolbox.dataanalysis) | exp2  (in PyDSTool.Toolbox.fracdim) | | ensure\_has\_test\_traj()  (in extModelInterface) | ERR\_WARN  (in PyDSTool.Toolbox.fracdim) | exp2  (in PyDSTool.Toolbox.makeSloppyModel) | | ensure\_has\_test\_traj()  (in intModelInterface) | ERR\_WARN  (in PyDSTool.Toolbox.makeSloppyModel) | exp2  (in PyDSTool.Toolbox.neuralcomp) | | ensurebare()  (in PyDSTool.parseUtils) | ERR\_WARN  (in PyDSTool.Toolbox.neuralcomp) | exp2  (in PyDSTool.Toolbox.phaseplane) | | ensuredecimalconst()  (in PyDSTool.parseUtils) | ERR\_WARN  (in PyDSTool.Toolbox.phaseplane) | exp2  (in PyDSTool.Toolbox.synthetic\_data) | | ensurefloat()  (in PyDSTool.common) | ERR\_WARN  (in PyDSTool.Toolbox.synthetic\_data) | exp2  (in PyDSTool.Toolbox.syntheticdata) | | ensureints()  (in PyDSTool.parseUtils) | ERR\_WARN  (in PyDSTool.Toolbox.syntheticdata) | exp2  (in PyDSTool) | | ensureparen()  (in PyDSTool.parseUtils) | ERR\_WARN  (in matplotlib.pylab) | exp2  (in matplotlib.pylab) | | ensureparen\_div()  (in PyDSTool.parseUtils) | errmessages  (in PyDSTool.Generator.ADMC\_ODEsystem') | ExpFuncSpec  (in PyDSTool.FuncSpec') | | ensureQlist()  (in PyDSTool.Symbolic) | errmessages  (in PyDSTool.Generator.Dopri\_ODEsystem') | ExplicitFnGen  (in PyDSTool.Generator.ExplicitFnGen') | | ensureStrArgDict()  (in PyDSTool.Symbolic) | errmessages  (in PyDSTool.Generator.EmbeddedSysGen') | ExplicitFnGen'  (in PyDSTool.Generator) | | epoch  (in PyDSTool.Toolbox.dssrt) | errmessages  (in PyDSTool.Generator.Euler\_ODEsystem') | expm1  (in PyDSTool.PyCont.ContClass') | | EpochSeqScorer  (in PyDSTool.Toolbox.dssrt) | errmessages  (in PyDSTool.Generator.ExplicitFnGen') | expm1  (in PyDSTool.Toolbox.ActivationFuncs) | | equal  (in PyDSTool.PyCont.ContClass') | errmessages  (in PyDSTool.Generator.ExtrapolateTable') | expm1  (in PyDSTool.Toolbox.DSSRT\_tools) | | equal  (in PyDSTool.Toolbox.ActivationFuncs) | errmessages  (in PyDSTool.Generator.ImplicitFnGen') | expm1  (in PyDSTool.Toolbox.InputProfile) | | equal  (in PyDSTool.Toolbox.DSSRT\_tools) | errmessages  (in PyDSTool.Generator.InterpolateTable') | expm1  (in PyDSTool.Toolbox.ModelHelper) | | equal  (in PyDSTool.Toolbox.InputProfile) | errmessages  (in PyDSTool.Generator.LookupTable') | expm1  (in PyDSTool.Toolbox.NineML) | | equal  (in PyDSTool.Toolbox.ModelHelper) | errmessages  (in PyDSTool.Generator.MapSystem') | expm1  (in PyDSTool.Toolbox.adjointPRC) | | equal  (in PyDSTool.Toolbox.NineML) | errmessages  (in PyDSTool.Generator.ODEsystem') | expm1  (in PyDSTool.Toolbox.dataanalysis) | | equal  (in PyDSTool.Toolbox.adjointPRC) | errmessages  (in PyDSTool.Generator.Radau\_ODEsystem') | expm1  (in PyDSTool.Toolbox.fracdim) | | equal  (in PyDSTool.Toolbox.dataanalysis) | errmessages  (in PyDSTool.Generator.Vode\_ODEsystem') | expm1  (in PyDSTool.Toolbox.makeSloppyModel) | | equal  (in PyDSTool.Toolbox.fracdim) | errmessages  (in PyDSTool.Generator.messagecodes) | expm1  (in PyDSTool.Toolbox.neuralcomp) | | equal  (in PyDSTool.Toolbox.makeSloppyModel) | errmessages  (in PyDSTool.Toolbox.NineML) | expm1  (in PyDSTool.Toolbox.phaseplane) | | equal  (in PyDSTool.Toolbox.neuralcomp) | errmessages  (in PyDSTool.Toolbox.dataanalysis) | expm1  (in PyDSTool.Toolbox.synthetic\_data) | | equal  (in PyDSTool.Toolbox.phaseplane) | errmessages  (in PyDSTool.Toolbox.phaseplane) | expm1  (in PyDSTool.Toolbox.syntheticdata) | | equal  (in PyDSTool.Toolbox.synthetic\_data) | errmessages  (in PyDSTool.Toolbox.synthetic\_data) | expm1  (in PyDSTool) | | equal  (in PyDSTool.Toolbox.syntheticdata) | errmessages  (in PyDSTool.Toolbox.syntheticdata) | expm1  (in matplotlib.pylab) | | equal  (in PyDSTool) | errorfields  (in PyDSTool.Generator.ADMC\_ODEsystem') | export\_pointset\_to\_CSV()  (in PyDSTool.Points) | | equal  (in matplotlib.pylab) | errorfields  (in PyDSTool.Generator.Dopri\_ODEsystem') | exportGeomview()  (in ContClass) | | equilibrium\_args\_list  (in PyDSTool.PyCont.Continuation) | errorfields  (in PyDSTool.Generator.EmbeddedSysGen') | exportGeomview()  (in Continuation) | | equilibrium\_bif\_points  (in PyDSTool.PyCont.Continuation) | errorfields  (in PyDSTool.Generator.Euler\_ODEsystem') | exportMatlab()  (in ContClass) | | EquilibriumCurve  (in PyDSTool.PyCont.Continuation) | errorfields  (in PyDSTool.Generator.ExplicitFnGen') | exportPointset()  (in PyDSTool.Points) | | ERR\_CALL  (in PyDSTool) | errorfields  (in PyDSTool.Generator.ExtrapolateTable') | Expovariate  (in PyDSTool) | | ERR\_CALL  (in PyDSTool.PyCont.ContClass') | errorfields  (in PyDSTool.Generator.ImplicitFnGen') | Expovariate  (in PyDSTool.ModelSpec') | | ERR\_CALL  (in PyDSTool.Toolbox.ActivationFuncs) | errorfields  (in PyDSTool.Generator.InterpolateTable') | Expovariate  (in PyDSTool.Symbolic) | | ERR\_CALL  (in PyDSTool.Toolbox.DSSRT\_tools) | errorfields  (in PyDSTool.Generator.LookupTable') | Expovariate  (in PyDSTool.Toolbox.ActivationFuncs) | | ERR\_CALL  (in PyDSTool.Toolbox.InputProfile) | errorfields  (in PyDSTool.Generator.MapSystem') | Expovariate  (in PyDSTool.Toolbox.DSSRT\_tools) | | ERR\_CALL  (in PyDSTool.Toolbox.ModelHelper) | errorfields  (in PyDSTool.Generator.ODEsystem') | Expovariate  (in PyDSTool.Toolbox.InputProfile) | | ERR\_CALL  (in PyDSTool.Toolbox.NineML) | errorfields  (in PyDSTool.Generator.Radau\_ODEsystem') | Expovariate  (in PyDSTool.Toolbox.ModelHelper) | | ERR\_CALL  (in PyDSTool.Toolbox.adjointPRC) | errorfields  (in PyDSTool.Generator.Vode\_ODEsystem') | Expovariate  (in PyDSTool.Toolbox.NineML) | | ERR\_CALL  (in PyDSTool.Toolbox.dataanalysis) | errorfields  (in PyDSTool.Generator.messagecodes) | Expovariate  (in PyDSTool.Toolbox.adjointPRC) | | ERR\_CALL  (in PyDSTool.Toolbox.fracdim) | errorfields  (in PyDSTool.Toolbox.NineML) | Expovariate  (in PyDSTool.Toolbox.dataanalysis) | | ERR\_CALL  (in PyDSTool.Toolbox.makeSloppyModel) | errorfields  (in PyDSTool.Toolbox.dataanalysis) | Expovariate  (in PyDSTool.Toolbox.fracdim) | | ERR\_CALL  (in PyDSTool.Toolbox.neuralcomp) | errorfields  (in PyDSTool.Toolbox.phaseplane) | Expovariate  (in PyDSTool.Toolbox.makeSloppyModel) | | ERR\_CALL  (in PyDSTool.Toolbox.phaseplane) | errorfields  (in PyDSTool.Toolbox.synthetic\_data) | Expovariate  (in PyDSTool.Toolbox.neuralcomp) | | ERR\_CALL  (in PyDSTool.Toolbox.synthetic\_data) | errorfields  (in PyDSTool.Toolbox.syntheticdata) | Expovariate  (in PyDSTool.Toolbox.phaseplane) | | ERR\_CALL  (in PyDSTool.Toolbox.syntheticdata) | errors  (in PyDSTool.Toolbox.optimizers.defaults) | Expovariate  (in PyDSTool.Toolbox.synthetic\_data) | | ERR\_CALL  (in matplotlib.pylab) | errors  (in PyDSTool) | Expovariate  (in PyDSTool.Toolbox.syntheticdata) | | ERR\_DEFAULT  (in PyDSTool) | estimate\_spiking  (in PyDSTool.Toolbox.neuro\_data) | expr2fun()  (in PyDSTool.Symbolic) | | ERR\_DEFAULT  (in PyDSTool.PyCont.ContClass') | Euler\_ODEsystem  (in PyDSTool.Generator.Euler\_ODEsystem') | EXT1  (in PyDSTool.fixedpickle) | | ERR\_DEFAULT  (in PyDSTool.Toolbox.ActivationFuncs) | Euler\_ODEsystem'  (in PyDSTool.Generator) | EXT2  (in PyDSTool.fixedpickle) | | ERR\_DEFAULT  (in PyDSTool.Toolbox.DSSRT\_tools) | euler\_solver  (in PyDSTool.Generator.Euler\_ODEsystem') | EXT4  (in PyDSTool.fixedpickle) | | ERR\_DEFAULT  (in PyDSTool.Toolbox.InputProfile) | eval()  (in QuantSpec) | extend()  (in PiecewisePolynomial) | | ERR\_DEFAULT  (in PyDSTool.Toolbox.ModelHelper) | eval()  (in Quantity) | extensions  (in PyDSTool.conf) | | ERR\_DEFAULT  (in PyDSTool.Toolbox.NineML) | eval()  (in mesh\_patch\_2D) | extent()  (in PyDSTool.common) | | ERR\_DEFAULT  (in PyDSTool.Toolbox.adjointPRC) | evalMultiRefToken()  (in PyDSTool.Symbolic) | extModelInterface  (in PyDSTool.MProject) | | ERR\_DEFAULT  (in PyDSTool.Toolbox.dataanalysis) | evaluate()  (in ModelInterface) | extract\_digraph()  (in PyDSTool.Toolbox.event\_driven\_simulator) | | ERR\_DEFAULT  (in PyDSTool.Toolbox.fracdim) | evaluate()  (in always\_feature) | extract\_from\_model()  (in PyDSTool.MProject) | | ERR\_DEFAULT  (in PyDSTool.Toolbox.makeSloppyModel) | evaluate()  (in binary\_feature) | extract\_history\_events()  (in simulator) | | ERR\_DEFAULT  (in PyDSTool.Toolbox.neuralcomp) | evaluate()  (in condition) | ExtrapolateTable  (in PyDSTool.Generator.ExtrapolateTable') | | ERR\_DEFAULT  (in PyDSTool.Toolbox.phaseplane) | evaluate()  (in context) | ExtrapolateTable'  (in PyDSTool.Generator) | | ERR\_DEFAULT  (in PyDSTool.Toolbox.synthetic\_data) | evaluate()  (in feature) |  | |

  
  

| Home | Trees | Indices | Help | | PyDSTool | | --- | |
| --- | --- | --- | --- | --- | --- |

|  |  |
| --- | --- |
| Generated by Epydoc 3.0.1 on Fri May 4 15:23:57 2012 | http://epydoc.sourceforge.net |
