# Supplementary material for: Hybrid Models and Biological Model Reduction with PyDSTool
Source: PLoS Comput Biol. 2012 Aug 9;8(8):e1002628. doi: 10.1371/journal.pcbi.1002628 (PMC3415397; doi:10.1371/journal.pcbi.1002628)
Supplement: Text S4 — Complete source code for the PyDSTool package (version 0.88.120504). Includes API documentation and help files linking to web pages. This file is identical to the current public release on Sourceforge.net. (ZIP) [file pcbi.1002628.s004.zip › PyDSTool/html/identifier-index-F.html]

xml version="1.0" encoding="ascii"?


Identifier Index


| Home | Trees | Indices | Help | | PyDSTool | | --- | |
| --- | --- | --- | --- | --- | --- |

|  |  |  |  |
| --- | --- | --- | --- |
|  | |  | | --- | | [hide private] | | [frames] | no frames] | |

|  |  |
| --- | --- |
| Identifier Index | [ A B C D E F G H I J K L M N O P Q R S T U V W X Y Z \_ ] |

|  |  |  |  |  |  |  |  |  |  |  |  |  |  |  |  |  |  |  |  |  |  |  |  |  |  |  |  |  |  |  |  |  |  |  |  |  |  |  |  |  |  |  |  |  |  |  |  |  |  |  |  |  |  |  |  |  |  |  |  |  |  |  |  |  |  |  |  |  |  |  |  |  |  |  |  |  |  |  |  |  |  |  |  |  |  |  |  |  |  |  |  |  |  |  |  |  |  |  |  |  |  |  |  |  |  |  |  |  |  |  |  |  |  |  |  |  |  |  |  |  |  |  |  |  |  |  |  |  |  |  |  |  |  |  |  |  |  |  |  |  |  |  |  |  |  |  |  |  |  |  |  |  |  |  |  |  |  |  |  |  |  |  |  |  |  |  |  |  |  |  |  |  |  |  |  |  |  |  |  |  |  |  |  |  |  |  |  |  |  |  |  |  |  |  |  |  |  |  |  |  |  |  |  |  |  |  |  |  |  |  |  |  |  |  |  |  |  |  |  |  |  |  |  |  |  |  |  |  |  |  |  |  |  |  |  |  |  |  |  |  |  |  |  |  |  |  |  |  |  |  |  |  |  |  |  |  |  |  |  |  |  |  |  |  |  |  |  |  |  |  |  |  |  |  |  |  |  |  |  |  |  |  |  |  |  |  |  |  |  |  |  |  |  |  |  |  |  |  |  |  |  |  |  |  |  |  |  |  |  |  |  |  |  |  |  |  |  |  |  |  |  |  |  |  |  |  |  |  |  |  |  |  |  |  |  |  |  |  |  |  |  |  |  |  |  |  |  |  |  |  |  |  |  |  |  |  |  |  |  |  |  |  |  |  |  |  |  |  |  |  |  |  |  |  |  |  |  |  |  |  |  |  |  |  |  |  |  |  |  |  |  |  |  |  |  |  |  |  |  |  |  |  |  |  |  |  |  |  |  |  |  |  |  |  |  |  |  |  |  |  |  |  |  |  |  |  |  |  |  |  |
| --- | --- | --- | --- | --- | --- | --- | --- | --- | --- | --- | --- | --- | --- | --- | --- | --- | --- | --- | --- | --- | --- | --- | --- | --- | --- | --- | --- | --- | --- | --- | --- | --- | --- | --- | --- | --- | --- | --- | --- | --- | --- | --- | --- | --- | --- | --- | --- | --- | --- | --- | --- | --- | --- | --- | --- | --- | --- | --- | --- | --- | --- | --- | --- | --- | --- | --- | --- | --- | --- | --- | --- | --- | --- | --- | --- | --- | --- | --- | --- | --- | --- | --- | --- | --- | --- | --- | --- | --- | --- | --- | --- | --- | --- | --- | --- | --- | --- | --- | --- | --- | --- | --- | --- | --- | --- | --- | --- | --- | --- | --- | --- | --- | --- | --- | --- | --- | --- | --- | --- | --- | --- | --- | --- | --- | --- | --- | --- | --- | --- | --- | --- | --- | --- | --- | --- | --- | --- | --- | --- | --- | --- | --- | --- | --- | --- | --- | --- | --- | --- | --- | --- | --- | --- | --- | --- | --- | --- | --- | --- | --- | --- | --- | --- | --- | --- | --- | --- | --- | --- | --- | --- | --- | --- | --- | --- | --- | --- | --- | --- | --- | --- | --- | --- | --- | --- | --- | --- | --- | --- | --- | --- | --- | --- | --- | --- | --- | --- | --- | --- | --- | --- | --- | --- | --- | --- | --- | --- | --- | --- | --- | --- | --- | --- | --- | --- | --- | --- | --- | --- | --- | --- | --- | --- | --- | --- | --- | --- | --- | --- | --- | --- | --- | --- | --- | --- | --- | --- | --- | --- | --- | --- | --- | --- | --- | --- | --- | --- | --- | --- | --- | --- | --- | --- | --- | --- | --- | --- | --- | --- | --- | --- | --- | --- | --- | --- | --- | --- | --- | --- | --- | --- | --- | --- | --- | --- | --- | --- | --- | --- | --- | --- | --- | --- | --- | --- | --- | --- | --- | --- | --- | --- | --- | --- | --- | --- | --- | --- | --- | --- | --- | --- | --- | --- | --- | --- | --- | --- | --- | --- | --- | --- | --- | --- | --- | --- | --- | --- | --- | --- | --- | --- | --- | --- | --- | --- | --- | --- | --- | --- | --- | --- | --- | --- | --- | --- | --- | --- | --- | --- | --- | --- | --- | --- | --- | --- | --- | --- | --- | --- | --- | --- | --- | --- | --- | --- | --- | --- | --- | --- | --- | --- | --- | --- | --- | --- | --- | --- | --- | --- | --- | --- | --- | --- | --- | --- | --- | --- | --- | --- | --- | --- | --- | --- | --- | --- | --- | --- | --- | --- | --- | --- | --- | --- | --- | --- | --- | --- | --- | --- | --- | --- | --- | --- | --- | --- | --- | --- | --- | --- | --- | --- | --- | --- | --- | --- | --- | --- | --- | --- | --- | --- | --- | --- | --- | --- | --- | --- | --- | --- | --- |
| F | |  |  |  | | --- | --- | --- | | f  (in PyDSTool.Symbolic) | fixed\_last\_step\_modifier  (in PyDSTool.Toolbox.optimizers.line\_search) | forceLibRefresh()  (in Dopri\_ODEsystem) | | f1()  (in PyDSTool.scipy\_ode) | FixedLastStepModifier  (in PyDSTool.Toolbox.optimizers.line\_search.fixed\_last\_step\_modifier) | forceLibRefresh()  (in Radau\_ODEsystem) | | f2()  (in PyDSTool.scipy\_ode) | fixedpickle  (in PyDSTool) | forceObsVars()  (in Model) | | Fabs  (in PyDSTool) | fixedpoint\_2D  (in PyDSTool.Toolbox.phaseplane) | format\_version  (in PyDSTool.fixedpickle) | | Fabs  (in PyDSTool.ModelSpec') | fixedpoint\_args\_list  (in PyDSTool.PyCont.Continuation) | format\_version  (in cPickle) | | fabs  (in PyDSTool.PyCont.ContClass') | fixedpoint\_bif\_points  (in PyDSTool.PyCont.Continuation) | forward()  (in Continuation) | | Fabs  (in PyDSTool.Symbolic) | fixedpoint\_nD  (in PyDSTool.Toolbox.phaseplane) | ForwardFiniteDifferences  (in PyDSTool.Toolbox.optimizers.helpers.finite\_difference) | | fabs  (in PyDSTool.Symbolic) | fixedpoint\_zone  (in PyDSTool.Toolbox.phaseplane) | ForwardFiniteDifferencesCache  (in PyDSTool.Toolbox.optimizers.helpers.finite\_difference) | | Fabs  (in PyDSTool.Toolbox.ActivationFuncs) | FixedPointCurve  (in PyDSTool.PyCont.Continuation) | FPE\_DIVIDEBYZERO  (in PyDSTool) | | Fabs  (in PyDSTool.Toolbox.DSSRT\_tools) | FixedPointMap  (in PyDSTool.PyCont.TestFunc) | FPE\_DIVIDEBYZERO  (in PyDSTool.PyCont.ContClass') | | Fabs  (in PyDSTool.Toolbox.InputProfile) | flattenSpec()  (in ModelSpec) | FPE\_DIVIDEBYZERO  (in PyDSTool.Toolbox.ActivationFuncs) | | Fabs  (in PyDSTool.Toolbox.ModelHelper) | FLOAT  (in PyDSTool.fixedpickle) | FPE\_DIVIDEBYZERO  (in PyDSTool.Toolbox.DSSRT\_tools) | | Fabs  (in PyDSTool.Toolbox.NineML) | FLOATING\_POINT\_SUPPORT  (in PyDSTool) | FPE\_DIVIDEBYZERO  (in PyDSTool.Toolbox.InputProfile) | | fabs  (in PyDSTool.Toolbox.NineML) | FLOATING\_POINT\_SUPPORT  (in PyDSTool.PyCont.ContClass') | FPE\_DIVIDEBYZERO  (in PyDSTool.Toolbox.ModelHelper) | | Fabs  (in PyDSTool.Toolbox.adjointPRC) | FLOATING\_POINT\_SUPPORT  (in PyDSTool.Toolbox.ActivationFuncs) | FPE\_DIVIDEBYZERO  (in PyDSTool.Toolbox.NineML) | | Fabs  (in PyDSTool.Toolbox.dataanalysis) | FLOATING\_POINT\_SUPPORT  (in PyDSTool.Toolbox.DSSRT\_tools) | FPE\_DIVIDEBYZERO  (in PyDSTool.Toolbox.adjointPRC) | | fabs  (in PyDSTool.Toolbox.dataanalysis) | FLOATING\_POINT\_SUPPORT  (in PyDSTool.Toolbox.InputProfile) | FPE\_DIVIDEBYZERO  (in PyDSTool.Toolbox.dataanalysis) | | Fabs  (in PyDSTool.Toolbox.fracdim) | FLOATING\_POINT\_SUPPORT  (in PyDSTool.Toolbox.ModelHelper) | FPE\_DIVIDEBYZERO  (in PyDSTool.Toolbox.fracdim) | | Fabs  (in PyDSTool.Toolbox.makeSloppyModel) | FLOATING\_POINT\_SUPPORT  (in PyDSTool.Toolbox.NineML) | FPE\_DIVIDEBYZERO  (in PyDSTool.Toolbox.makeSloppyModel) | | Fabs  (in PyDSTool.Toolbox.neuralcomp) | FLOATING\_POINT\_SUPPORT  (in PyDSTool.Toolbox.adjointPRC) | FPE\_DIVIDEBYZERO  (in PyDSTool.Toolbox.neuralcomp) | | Fabs  (in PyDSTool.Toolbox.phaseplane) | FLOATING\_POINT\_SUPPORT  (in PyDSTool.Toolbox.dataanalysis) | FPE\_DIVIDEBYZERO  (in PyDSTool.Toolbox.phaseplane) | | fabs  (in PyDSTool.Toolbox.phaseplane) | FLOATING\_POINT\_SUPPORT  (in PyDSTool.Toolbox.fracdim) | FPE\_DIVIDEBYZERO  (in PyDSTool.Toolbox.synthetic\_data) | | Fabs  (in PyDSTool.Toolbox.synthetic\_data) | FLOATING\_POINT\_SUPPORT  (in PyDSTool.Toolbox.makeSloppyModel) | FPE\_DIVIDEBYZERO  (in PyDSTool.Toolbox.syntheticdata) | | fabs  (in PyDSTool.Toolbox.synthetic\_data) | FLOATING\_POINT\_SUPPORT  (in PyDSTool.Toolbox.neuralcomp) | FPE\_DIVIDEBYZERO  (in matplotlib.pylab) | | Fabs  (in PyDSTool.Toolbox.syntheticdata) | FLOATING\_POINT\_SUPPORT  (in PyDSTool.Toolbox.phaseplane) | FPE\_INVALID  (in PyDSTool) | | fabs  (in PyDSTool.Toolbox.syntheticdata) | FLOATING\_POINT\_SUPPORT  (in PyDSTool.Toolbox.synthetic\_data) | FPE\_INVALID  (in PyDSTool.PyCont.ContClass') | | fabs  (in matplotlib.pylab) | FLOATING\_POINT\_SUPPORT  (in PyDSTool.Toolbox.syntheticdata) | FPE\_INVALID  (in PyDSTool.Toolbox.ActivationFuncs) | | facilities  (in PyDSTool.Toolbox.optimizers.criterion) | FLOATING\_POINT\_SUPPORT  (in matplotlib.pylab) | FPE\_INVALID  (in PyDSTool.Toolbox.DSSRT\_tools) | | failed  (in PyDSTool.matplotlib\_import) | Floor  (in PyDSTool) | FPE\_INVALID  (in PyDSTool.Toolbox.InputProfile) | | FAILED\_WITH\_UNIMPLEMENTED\_OR\_UNKNOWN\_REASON  (in PyDSTool.Toolbox.optimizers.defaults) | Floor  (in PyDSTool.ModelSpec') | FPE\_INVALID  (in PyDSTool.Toolbox.ModelHelper) | | FALSE  (in PyDSTool.fixedpickle) | floor  (in PyDSTool.PyCont.ContClass') | FPE\_INVALID  (in PyDSTool.Toolbox.NineML) | | False\_  (in PyDSTool) | Floor  (in PyDSTool.Symbolic) | FPE\_INVALID  (in PyDSTool.Toolbox.adjointPRC) | | False\_  (in PyDSTool.PyCont.ContClass') | floor  (in PyDSTool.Symbolic) | FPE\_INVALID  (in PyDSTool.Toolbox.dataanalysis) | | False\_  (in PyDSTool.Toolbox.ActivationFuncs) | Floor  (in PyDSTool.Toolbox.ActivationFuncs) | FPE\_INVALID  (in PyDSTool.Toolbox.fracdim) | | False\_  (in PyDSTool.Toolbox.DSSRT\_tools) | Floor  (in PyDSTool.Toolbox.DSSRT\_tools) | FPE\_INVALID  (in PyDSTool.Toolbox.makeSloppyModel) | | False\_  (in PyDSTool.Toolbox.InputProfile) | Floor  (in PyDSTool.Toolbox.InputProfile) | FPE\_INVALID  (in PyDSTool.Toolbox.neuralcomp) | | False\_  (in PyDSTool.Toolbox.ModelHelper) | Floor  (in PyDSTool.Toolbox.ModelHelper) | FPE\_INVALID  (in PyDSTool.Toolbox.phaseplane) | | False\_  (in PyDSTool.Toolbox.NineML) | Floor  (in PyDSTool.Toolbox.NineML) | FPE\_INVALID  (in PyDSTool.Toolbox.synthetic\_data) | | False\_  (in PyDSTool.Toolbox.adjointPRC) | floor  (in PyDSTool.Toolbox.NineML) | FPE\_INVALID  (in PyDSTool.Toolbox.syntheticdata) | | False\_  (in PyDSTool.Toolbox.dataanalysis) | Floor  (in PyDSTool.Toolbox.adjointPRC) | FPE\_INVALID  (in matplotlib.pylab) | | False\_  (in PyDSTool.Toolbox.fracdim) | Floor  (in PyDSTool.Toolbox.dataanalysis) | FPE\_OVERFLOW  (in PyDSTool) | | False\_  (in PyDSTool.Toolbox.makeSloppyModel) | floor  (in PyDSTool.Toolbox.dataanalysis) | FPE\_OVERFLOW  (in PyDSTool.PyCont.ContClass') | | False\_  (in PyDSTool.Toolbox.neuralcomp) | Floor  (in PyDSTool.Toolbox.fracdim) | FPE\_OVERFLOW  (in PyDSTool.Toolbox.ActivationFuncs) | | False\_  (in PyDSTool.Toolbox.phaseplane) | Floor  (in PyDSTool.Toolbox.makeSloppyModel) | FPE\_OVERFLOW  (in PyDSTool.Toolbox.DSSRT\_tools) | | False\_  (in PyDSTool.Toolbox.synthetic\_data) | Floor  (in PyDSTool.Toolbox.neuralcomp) | FPE\_OVERFLOW  (in PyDSTool.Toolbox.InputProfile) | | False\_  (in PyDSTool.Toolbox.syntheticdata) | Floor  (in PyDSTool.Toolbox.phaseplane) | FPE\_OVERFLOW  (in PyDSTool.Toolbox.ModelHelper) | | False\_  (in matplotlib.pylab) | floor  (in PyDSTool.Toolbox.phaseplane) | FPE\_OVERFLOW  (in PyDSTool.Toolbox.NineML) | | FAR  (in PyDSTool.Toolbox.dssrt) | Floor  (in PyDSTool.Toolbox.synthetic\_data) | FPE\_OVERFLOW  (in PyDSTool.Toolbox.adjointPRC) | | fast()  (in VarAlphabet) | floor  (in PyDSTool.Toolbox.synthetic\_data) | FPE\_OVERFLOW  (in PyDSTool.Toolbox.dataanalysis) | | feature  (in PyDSTool.MProject) | Floor  (in PyDSTool.Toolbox.syntheticdata) | FPE\_OVERFLOW  (in PyDSTool.Toolbox.fracdim) | | feature\_leaf  (in PyDSTool.MProject) | floor  (in PyDSTool.Toolbox.syntheticdata) | FPE\_OVERFLOW  (in PyDSTool.Toolbox.makeSloppyModel) | | feature\_node  (in PyDSTool.MProject) | floor  (in matplotlib.pylab) | FPE\_OVERFLOW  (in PyDSTool.Toolbox.neuralcomp) | | feval\_map\_const  (in PyDSTool.Symbolic) | floor\_divide  (in PyDSTool.PyCont.ContClass') | FPE\_OVERFLOW  (in PyDSTool.Toolbox.phaseplane) | | feval\_map\_symb  (in PyDSTool.Symbolic) | floor\_divide  (in PyDSTool.Toolbox.ActivationFuncs) | FPE\_OVERFLOW  (in PyDSTool.Toolbox.synthetic\_data) | | fibonacci\_section  (in PyDSTool.Toolbox.optimizers.line\_search) | floor\_divide  (in PyDSTool.Toolbox.DSSRT\_tools) | FPE\_OVERFLOW  (in PyDSTool.Toolbox.syntheticdata) | | FibonacciSectionSearch  (in PyDSTool.Toolbox.optimizers.line\_search.fibonacci\_section) | floor\_divide  (in PyDSTool.Toolbox.InputProfile) | FPE\_OVERFLOW  (in matplotlib.pylab) | | FIFOqueue\_uniquenode  (in PyDSTool.Toolbox.event\_driven\_simulator) | floor\_divide  (in PyDSTool.Toolbox.ModelHelper) | FPE\_UNDERFLOW  (in PyDSTool) | | filter\_by\_radius()  (in PyDSTool.Toolbox.fracdim) | floor\_divide  (in PyDSTool.Toolbox.NineML) | FPE\_UNDERFLOW  (in PyDSTool.PyCont.ContClass') | | filter\_close\_points()  (in PyDSTool.Toolbox.phaseplane) | floor\_divide  (in PyDSTool.Toolbox.adjointPRC) | FPE\_UNDERFLOW  (in PyDSTool.Toolbox.ActivationFuncs) | | filter\_feats()  (in PyDSTool.Toolbox.ParamEst) | floor\_divide  (in PyDSTool.Toolbox.dataanalysis) | FPE\_UNDERFLOW  (in PyDSTool.Toolbox.DSSRT\_tools) | | filter\_iface()  (in PyDSTool.Toolbox.ParamEst) | floor\_divide  (in PyDSTool.Toolbox.fracdim) | FPE\_UNDERFLOW  (in PyDSTool.Toolbox.InputProfile) | | filter\_NaN()  (in PyDSTool.Toolbox.phaseplane) | floor\_divide  (in PyDSTool.Toolbox.makeSloppyModel) | FPE\_UNDERFLOW  (in PyDSTool.Toolbox.ModelHelper) | | filter\_pars()  (in PyDSTool.Toolbox.ParamEst) | floor\_divide  (in PyDSTool.Toolbox.neuralcomp) | FPE\_UNDERFLOW  (in PyDSTool.Toolbox.NineML) | | filteredDict()  (in PyDSTool.common) | floor\_divide  (in PyDSTool.Toolbox.phaseplane) | FPE\_UNDERFLOW  (in PyDSTool.Toolbox.adjointPRC) | | filtfilt()  (in PyDSTool.Toolbox.data\_analysis) | floor\_divide  (in PyDSTool.Toolbox.synthetic\_data) | FPE\_UNDERFLOW  (in PyDSTool.Toolbox.dataanalysis) | | find()  (in Pointset) | floor\_divide  (in PyDSTool.Toolbox.syntheticdata) | FPE\_UNDERFLOW  (in PyDSTool.Toolbox.fracdim) | | find()  (in parserObject) | floor\_divide  (in PyDSTool) | FPE\_UNDERFLOW  (in PyDSTool.Toolbox.makeSloppyModel) | | find()  (in PyDSTool.utils) | floor\_divide  (in matplotlib.pylab) | FPE\_UNDERFLOW  (in PyDSTool.Toolbox.neuralcomp) | | find\_central\_point()  (in PyDSTool.Toolbox.data\_analysis) | FLOQ\_TOL  (in PyDSTool.PyCont.misc) | FPE\_UNDERFLOW  (in PyDSTool.Toolbox.phaseplane) | | find\_central\_point()  (in PyDSTool.Toolbox.dataanalysis) | flush()  (in Redirector) | FPE\_UNDERFLOW  (in PyDSTool.Toolbox.synthetic\_data) | | find\_class()  (in Unpickler) | fmax  (in PyDSTool.PyCont.ContClass') | FPE\_UNDERFLOW  (in PyDSTool.Toolbox.syntheticdata) | | find\_closest\_val()  (in PyDSTool.Toolbox.data\_analysis) | fmax  (in PyDSTool.Toolbox.ActivationFuncs) | FPE\_UNDERFLOW  (in matplotlib.pylab) | | find\_closest\_val()  (in PyDSTool.Toolbox.dataanalysis) | fmax  (in PyDSTool.Toolbox.DSSRT\_tools) | FR  (in PyDSTool.PyCont.ContClass') | | find\_cover()  (in PyDSTool.Toolbox.fracdim) | fmax  (in PyDSTool.Toolbox.InputProfile) | FR  (in PyDSTool.Toolbox) | | find\_diameter()  (in PyDSTool.Toolbox.data\_analysis) | fmax  (in PyDSTool.Toolbox.ModelHelper) | FR  (in matplotlib.pylab) | | find\_diameter()  (in PyDSTool.Toolbox.dataanalysis) | fmax  (in PyDSTool.Toolbox.NineML) | FR()  (in PyDSTool.Toolbox.FR) | | find\_ep\_ix()  (in PyDSTool.Toolbox.dssrt) | fmax  (in PyDSTool.Toolbox.adjointPRC) | fracdim  (in PyDSTool.Toolbox) | | find\_epoch\_period()  (in PyDSTool.Toolbox.dssrt) | fmax  (in PyDSTool.Toolbox.dataanalysis) | FRConjugateGradientStep()  (in PyDSTool.Toolbox.optimizers.step.conjugate\_gradient\_step) | | find\_fixedpoints()  (in PyDSTool.Toolbox.phaseplane) | fmax  (in PyDSTool.Toolbox.fracdim) | Frexp  (in PyDSTool) | | find\_from\_sorted()  (in PyDSTool.Toolbox.data\_analysis) | fmax  (in PyDSTool.Toolbox.makeSloppyModel) | Frexp  (in PyDSTool.ModelSpec') | | find\_from\_sorted()  (in PyDSTool.Toolbox.dataanalysis) | fmax  (in PyDSTool.Toolbox.neuralcomp) | frexp  (in PyDSTool.PyCont.ContClass') | | find\_from\_sorted()  (in PyDSTool.Toolbox.fracdim) | fmax  (in PyDSTool.Toolbox.phaseplane) | Frexp  (in PyDSTool.Symbolic) | | find\_integrator()  (in PyDSTool.scipy\_ode) | fmax  (in PyDSTool.Toolbox.synthetic\_data) | frexp  (in PyDSTool.Symbolic) | | find\_internal\_extrema()  (in PyDSTool.Toolbox.neuro\_data) | fmax  (in PyDSTool.Toolbox.syntheticdata) | Frexp  (in PyDSTool.Toolbox.ActivationFuncs) | | find\_knees()  (in PyDSTool.Toolbox.data\_analysis) | fmax  (in PyDSTool) | Frexp  (in PyDSTool.Toolbox.DSSRT\_tools) | | find\_knees()  (in PyDSTool.Toolbox.dataanalysis) | fmax  (in matplotlib.pylab) | Frexp  (in PyDSTool.Toolbox.InputProfile) | | find\_logs()  (in ParamEst) | fmin  (in PyDSTool.PyCont.ContClass') | Frexp  (in PyDSTool.Toolbox.ModelHelper) | | find\_nearby\_annulus()  (in PyDSTool.Toolbox.data\_analysis) | fmin  (in PyDSTool.Toolbox.ActivationFuncs) | Frexp  (in PyDSTool.Toolbox.NineML) | | find\_nearby\_annulus()  (in PyDSTool.Toolbox.dataanalysis) | fmin  (in PyDSTool.Toolbox.DSSRT\_tools) | frexp  (in PyDSTool.Toolbox.NineML) | | find\_nearby\_ball()  (in PyDSTool.Toolbox.data\_analysis) | fmin  (in PyDSTool.Toolbox.InputProfile) | Frexp  (in PyDSTool.Toolbox.adjointPRC) | | find\_nearby\_ball()  (in PyDSTool.Toolbox.dataanalysis) | fmin  (in PyDSTool.Toolbox.ModelHelper) | Frexp  (in PyDSTool.Toolbox.dataanalysis) | | find\_nearest\_sample\_points\_by\_angle()  (in PyDSTool.Toolbox.phaseplane) | fmin  (in PyDSTool.Toolbox.NineML) | frexp  (in PyDSTool.Toolbox.dataanalysis) | | find\_nhd()  (in PyDSTool.Toolbox.fracdim) | fmin  (in PyDSTool.Toolbox.adjointPRC) | Frexp  (in PyDSTool.Toolbox.fracdim) | | find\_nullclines()  (in PyDSTool.Toolbox.phaseplane) | fmin  (in PyDSTool.Toolbox.dataanalysis) | Frexp  (in PyDSTool.Toolbox.makeSloppyModel) | | find\_outliers()  (in PyDSTool.Toolbox.fracdim) | fmin  (in PyDSTool.Toolbox.fracdim) | Frexp  (in PyDSTool.Toolbox.neuralcomp) | | find\_period()  (in PyDSTool.Toolbox.phaseplane) | fmin  (in PyDSTool.Toolbox.makeSloppyModel) | Frexp  (in PyDSTool.Toolbox.phaseplane) | | find\_recurrences()  (in PyDSTool.Toolbox.data\_analysis) | fmin  (in PyDSTool.Toolbox.neuralcomp) | frexp  (in PyDSTool.Toolbox.phaseplane) | | find\_recurrences()  (in PyDSTool.Toolbox.dataanalysis) | fmin  (in PyDSTool.Toolbox.phaseplane) | Frexp  (in PyDSTool.Toolbox.synthetic\_data) | | find\_regime\_transition()  (in PyDSTool.Toolbox.dssrt) | fmin  (in PyDSTool.Toolbox.synthetic\_data) | frexp  (in PyDSTool.Toolbox.synthetic\_data) | | find\_saddle\_manifolds()  (in PyDSTool.Toolbox.phaseplane) | fmin  (in PyDSTool.Toolbox.syntheticdata) | Frexp  (in PyDSTool.Toolbox.syntheticdata) | | find\_spike\_ixs\_dir()  (in spike\_envelope) | fmin  (in PyDSTool) | frexp  (in PyDSTool.Toolbox.syntheticdata) | | find\_zero\_phase()  (in PyDSTool.Toolbox.adjointPRC) | fmin  (in matplotlib.pylab) | frexp  (in matplotlib.pylab) | | findApproxPeriod()  (in PyDSTool.Trajectory') | Fmod  (in PyDSTool) | fromkeys()  (in args) | | findClosestArray()  (in PyDSTool.utils) | Fmod  (in PyDSTool.ModelSpec') | fromLabel()  (in pargs) | | findClosestPointIndex()  (in PyDSTool.utils) | fmod  (in PyDSTool.PyCont.ContClass') | fromvector()  (in QuantSpec) | | findEndBrace()  (in PyDSTool.parseUtils) | Fmod  (in PyDSTool.Symbolic) | fromvector()  (in Quantity) | | findGenSubClasses()  (in PyDSTool.Generator) | fmod  (in PyDSTool.Symbolic) | FRPRPConjugateGradientStep()  (in PyDSTool.Toolbox.optimizers.step.conjugate\_gradient\_step) | | findMultiRefs()  (in PyDSTool.Symbolic) | Fmod  (in PyDSTool.Toolbox.ActivationFuncs) | FSM  (in PyDSTool.Toolbox) | | findNumTailPos()  (in PyDSTool.parseUtils) | Fmod  (in PyDSTool.Toolbox.DSSRT\_tools) | FSM  (in PyDSTool.Toolbox.FSM) | | findpreciseroot()  (in PyDSTool.Events) | Fmod  (in PyDSTool.Toolbox.InputProfile) | Fun  (in PyDSTool.Symbolic) | | findStaticVars()  (in GenTransform) | Fmod  (in PyDSTool.Toolbox.ModelHelper) | func()  (in AddTestFunction) | | findTrajInitiator()  (in PyDSTool.Model) | Fmod  (in PyDSTool.Toolbox.NineML) | func()  (in BT\_Fold) | | findWarnings()  (in Diagnostics) | fmod  (in PyDSTool.Toolbox.NineML) | func()  (in BT\_Hopf) | | findzero()  (in Function) | Fmod  (in PyDSTool.Toolbox.adjointPRC) | func()  (in BT\_Hopf\_One) | | findzero()  (in TestFunc) | Fmod  (in PyDSTool.Toolbox.dataanalysis) | func()  (in BorderMethod) | | finish()  (in feature) | fmod  (in PyDSTool.Toolbox.dataanalysis) | func()  (in Branch\_Bor) | | finish()  (in get\_spike\_data) | Fmod  (in PyDSTool.Toolbox.fracdim) | func()  (in Branch\_Det) | | finish()  (in get\_spike\_model) | Fmod  (in PyDSTool.Toolbox.makeSloppyModel) | func()  (in CP\_Fold) | | finish()  (in zone\_leaf) | Fmod  (in PyDSTool.Toolbox.neuralcomp) | func()  (in DH\_Hopf) | | finish()  (in zone\_node) | Fmod  (in PyDSTool.Toolbox.phaseplane) | func()  (in DiscreteMap) | | finite\_difference  (in PyDSTool.Toolbox.optimizers.helpers) | fmod  (in PyDSTool.Toolbox.phaseplane) | func()  (in FixedPointMap) | | FiniteDifferencesCache  (in PyDSTool.Toolbox.optimizers.helpers.finite\_difference) | Fmod  (in PyDSTool.Toolbox.synthetic\_data) | func()  (in Fold\_Bor) | | FiniteDifferencesFunction  (in PyDSTool.Toolbox.optimizers.helpers.finite\_difference) | fmod  (in PyDSTool.Toolbox.synthetic\_data) | func()  (in Fold\_Det) | | finitePRC()  (in PyDSTool.Toolbox.PRCtools) | Fmod  (in PyDSTool.Toolbox.syntheticdata) | func()  (in Fold\_Tan) | | firstlyapunov()  (in PyDSTool.PyCont.misc) | fmod  (in PyDSTool.Toolbox.syntheticdata) | func()  (in GH\_Hopf) | | fit()  (in ModelEst) | fmod  (in matplotlib.pylab) | func()  (in GH\_Hopf\_One) | | fit()  (in fit\_cubic) | fn()  (in fit\_cubic) | func()  (in Hopf\_Bor) | | fit()  (in fit\_diff\_of\_exp) | fn()  (in fit\_diff\_of\_exp) | func()  (in Hopf\_Det) | | fit()  (in fit\_exponential) | fn()  (in fit\_exponential) | func()  (in Hopf\_Double\_Bor\_One) | | fit()  (in fit\_function) | fn()  (in fit\_function) | func()  (in Hopf\_Double\_Bor\_Two) | | fit()  (in fit\_linear) | fn()  (in fit\_linear) | func()  (in Hopf\_Eig) | | fit()  (in fit\_quadratic) | fn()  (in fit\_quadratic) | func()  (in LPC\_Det) | | fit()  (in fit\_quadratic\_at\_vertex) | fn()  (in fit\_quadratic\_at\_vertex) | func()  (in NS\_Det) | | fit\_cubic  (in PyDSTool.common) | fname  (in PyDSTool.Symbolic) | func()  (in PD\_Det) | | fit\_diff\_of\_exp  (in PyDSTool.common) | fold\_args\_list  (in PyDSTool.PyCont.Continuation) | func()  (in ParTestFunc) | | fit\_exponential  (in PyDSTool.common) | fold\_bif\_points  (in PyDSTool.PyCont.Continuation) | func()  (in UserDefinedTestFunc) | | fit\_function  (in PyDSTool.common) | Fold\_Bor  (in PyDSTool.PyCont.TestFunc) | funcnames  (in PyDSTool.Symbolic) | | fit\_linear  (in PyDSTool.common) | Fold\_Det  (in PyDSTool.PyCont.TestFunc) | FuncSpec  (in PyDSTool.FuncSpec') | | fit\_quadratic  (in PyDSTool.common) | Fold\_Tan  (in PyDSTool.PyCont.TestFunc) | FuncSpec'  (in PyDSTool) | | fit\_quadratic\_at\_vertex  (in PyDSTool.common) | FoldCurve  (in PyDSTool.PyCont.Continuation) | funcstr  (in PyDSTool.Symbolic) | | fitline()  (in PyDSTool.Toolbox.data\_analysis) | FoldPoint  (in PyDSTool.PyCont.BifPoint) | Function  (in PyDSTool.PyCont.TestFunc) | | fitline()  (in PyDSTool.Toolbox.dataanalysis) | forceAutoLibRefresh()  (in ContClass) | FVAL\_IS\_ENOUGH  (in PyDSTool.Toolbox.optimizers.defaults) | | fix\_PRC()  (in PyDSTool.Toolbox.PRCtools) | forceIntVars()  (in Model) |  | |

  
  

| Home | Trees | Indices | Help | | PyDSTool | | --- | |
| --- | --- | --- | --- | --- | --- |

|  |  |
| --- | --- |
| Generated by Epydoc 3.0.1 on Fri May 4 15:23:57 2012 | http://epydoc.sourceforge.net |
