# Supplementary material for: Hybrid Models and Biological Model Reduction with PyDSTool
Source: PLoS Comput Biol. 2012 Aug 9;8(8):e1002628. doi: 10.1371/journal.pcbi.1002628 (PMC3415397; doi:10.1371/journal.pcbi.1002628)
Supplement: Text S4 — Complete source code for the PyDSTool package (version 0.88.120504). Includes API documentation and help files linking to web pages. This file is identical to the current public release on Sourceforge.net. (ZIP) [file pcbi.1002628.s004.zip › PyDSTool/html/identifier-index-G.html]

xml version="1.0" encoding="ascii"?


Identifier Index


| Home | Trees | Indices | Help | | PyDSTool | | --- | |
| --- | --- | --- | --- | --- | --- |

|  |  |  |  |
| --- | --- | --- | --- |
|  | |  | | --- | | [hide private] | | [frames] | no frames] | |

|  |  |
| --- | --- |
| Identifier Index | [ A B C D E F G H I J K L M N O P Q R S T U V W X Y Z \_ ] |

|  |  |  |  |  |  |  |  |  |  |  |  |  |  |  |  |  |  |  |  |  |  |  |  |  |  |  |  |  |  |  |  |  |  |  |  |  |  |  |  |  |  |  |  |  |  |  |  |  |  |  |  |  |  |  |  |  |  |  |  |  |  |  |  |  |  |  |  |  |  |  |  |  |  |  |  |  |  |  |  |  |  |  |  |  |  |  |  |  |  |  |  |  |  |  |  |  |  |  |  |  |  |  |  |  |  |  |  |  |  |  |  |  |  |  |  |  |  |  |  |  |  |  |  |  |  |  |  |  |  |  |  |  |  |  |  |  |  |  |  |  |  |  |  |  |  |  |  |  |  |  |  |  |  |  |  |  |  |  |  |  |  |  |  |  |  |  |  |  |  |  |  |  |  |  |  |  |  |  |  |  |  |  |  |  |  |  |  |  |  |  |  |  |  |  |  |  |  |  |  |  |  |  |  |  |  |  |  |  |  |  |  |  |  |  |  |  |  |  |  |  |  |  |  |  |  |  |  |  |  |  |  |  |  |  |  |  |  |  |  |  |  |  |  |  |  |  |  |  |  |  |
| --- | --- | --- | --- | --- | --- | --- | --- | --- | --- | --- | --- | --- | --- | --- | --- | --- | --- | --- | --- | --- | --- | --- | --- | --- | --- | --- | --- | --- | --- | --- | --- | --- | --- | --- | --- | --- | --- | --- | --- | --- | --- | --- | --- | --- | --- | --- | --- | --- | --- | --- | --- | --- | --- | --- | --- | --- | --- | --- | --- | --- | --- | --- | --- | --- | --- | --- | --- | --- | --- | --- | --- | --- | --- | --- | --- | --- | --- | --- | --- | --- | --- | --- | --- | --- | --- | --- | --- | --- | --- | --- | --- | --- | --- | --- | --- | --- | --- | --- | --- | --- | --- | --- | --- | --- | --- | --- | --- | --- | --- | --- | --- | --- | --- | --- | --- | --- | --- | --- | --- | --- | --- | --- | --- | --- | --- | --- | --- | --- | --- | --- | --- | --- | --- | --- | --- | --- | --- | --- | --- | --- | --- | --- | --- | --- | --- | --- | --- | --- | --- | --- | --- | --- | --- | --- | --- | --- | --- | --- | --- | --- | --- | --- | --- | --- | --- | --- | --- | --- | --- | --- | --- | --- | --- | --- | --- | --- | --- | --- | --- | --- | --- | --- | --- | --- | --- | --- | --- | --- | --- | --- | --- | --- | --- | --- | --- | --- | --- | --- | --- | --- | --- | --- | --- | --- | --- | --- | --- | --- | --- | --- | --- | --- | --- | --- | --- | --- | --- | --- | --- | --- | --- | --- | --- | --- | --- | --- | --- | --- | --- | --- | --- | --- | --- | --- | --- | --- | --- | --- | --- | --- | --- | --- | --- | --- | --- | --- | --- | --- | --- | --- |
| G | |  |  |  | | --- | --- | --- | | Gamma()  (in PyDSTool.Toolbox.ActivationFuncs) | get\_burst\_period\_info  (in PyDSTool.Toolbox.neuro\_data) | Getrandbits  (in PyDSTool.Toolbox.phaseplane) | | Gammavariate  (in PyDSTool) | get\_burst\_spikes  (in PyDSTool.Toolbox.neuro\_data) | Getrandbits  (in PyDSTool.Toolbox.synthetic\_data) | | Gammavariate  (in PyDSTool.ModelSpec') | get\_burst\_trough\_env  (in PyDSTool.Toolbox.neuro\_data) | Getrandbits  (in PyDSTool.Toolbox.syntheticdata) | | Gammavariate  (in PyDSTool.Symbolic) | get\_burst\_upsweep  (in PyDSTool.Toolbox.neuro\_data) | getSpecFromFile()  (in PyDSTool.FuncSpec') | | Gammavariate  (in PyDSTool.Toolbox.ActivationFuncs) | get\_cover\_radii()  (in PyDSTool.Toolbox.fracdim) | getSpecialPoint()  (in Continuation) | | Gammavariate  (in PyDSTool.Toolbox.DSSRT\_tools) | get\_desc()  (in ModelManager) | Getstate  (in PyDSTool) | | Gammavariate  (in PyDSTool.Toolbox.InputProfile) | get\_desc()  (in MDescriptor) | Getstate  (in PyDSTool.ModelSpec') | | Gammavariate  (in PyDSTool.Toolbox.ModelHelper) | get\_domscales\_point()  (in domscales) | Getstate  (in PyDSTool.Symbolic) | | Gammavariate  (in PyDSTool.Toolbox.NineML) | get\_evec()  (in PyDSTool.Toolbox.data\_analysis) | Getstate  (in PyDSTool.Toolbox.ActivationFuncs) | | Gammavariate  (in PyDSTool.Toolbox.adjointPRC) | get\_evec()  (in PyDSTool.Toolbox.dataanalysis) | Getstate  (in PyDSTool.Toolbox.DSSRT\_tools) | | Gammavariate  (in PyDSTool.Toolbox.dataanalysis) | get\_extension()  (in Unpickler) | Getstate  (in PyDSTool.Toolbox.InputProfile) | | Gammavariate  (in PyDSTool.Toolbox.fracdim) | get\_extrema()  (in PyDSTool.Toolbox.ParamEst) | Getstate  (in PyDSTool.Toolbox.ModelHelper) | | Gammavariate  (in PyDSTool.Toolbox.makeSloppyModel) | get\_extrema\_from\_events()  (in PyDSTool.Toolbox.ParamEst) | Getstate  (in PyDSTool.Toolbox.NineML) | | Gammavariate  (in PyDSTool.Toolbox.neuralcomp) | get\_filtered\_ixs()  (in PyDSTool.Toolbox.fracdim) | Getstate  (in PyDSTool.Toolbox.adjointPRC) | | Gammavariate  (in PyDSTool.Toolbox.phaseplane) | get\_infs()  (in PyDSTool.Toolbox.dssrt) | Getstate  (in PyDSTool.Toolbox.dataanalysis) | | Gammavariate  (in PyDSTool.Toolbox.synthetic\_data) | get\_linear\_regression\_residual()  (in PyDSTool.Toolbox.data\_analysis) | Getstate  (in PyDSTool.Toolbox.fracdim) | | Gammavariate  (in PyDSTool.Toolbox.syntheticdata) | get\_linear\_regression\_residual()  (in PyDSTool.Toolbox.dataanalysis) | Getstate  (in PyDSTool.Toolbox.makeSloppyModel) | | GAP  (in PyDSTool.Toolbox.dssrt) | get\_nineml\_model()  (in PyDSTool.Toolbox.NineML) | Getstate  (in PyDSTool.Toolbox.neuralcomp) | | Gauss  (in PyDSTool) | get\_opt()  (in PyDSTool.common) | Getstate  (in PyDSTool.Toolbox.phaseplane) | | Gauss  (in PyDSTool.ModelSpec') | get\_orthonormal()  (in PyDSTool.Toolbox.phaseplane) | Getstate  (in PyDSTool.Toolbox.synthetic\_data) | | Gauss  (in PyDSTool.Symbolic) | get\_perp()  (in PyDSTool.Toolbox.phaseplane) | Getstate  (in PyDSTool.Toolbox.syntheticdata) | | Gauss  (in PyDSTool.Toolbox.ActivationFuncs) | get\_PP()  (in PyDSTool.Toolbox.phaseplane) | getSuperClasses()  (in PyDSTool.common) | | Gauss  (in PyDSTool.Toolbox.DSSRT\_tools) | get\_pysces\_model()  (in PyDSTool.Toolbox.PySCes\_SBML) | getTermEvents()  (in EventStruct) | | Gauss  (in PyDSTool.Toolbox.InputProfile) | get\_regime\_model()  (in PyDSTool.Toolbox.NineML) | getTrajEvents()  (in Model) | | Gauss  (in PyDSTool.Toolbox.ModelHelper) | get\_residual()  (in PyDSTool.Toolbox.data\_analysis) | getTrajEventStruct()  (in Model) | | Gauss  (in PyDSTool.Toolbox.NineML) | get\_residual()  (in PyDSTool.Toolbox.dataanalysis) | getTrajEventTimes()  (in Model) | | Gauss  (in PyDSTool.Toolbox.adjointPRC) | get\_rotated()  (in PyDSTool.Toolbox.phaseplane) | getTrajModelName()  (in Model) | | Gauss  (in PyDSTool.Toolbox.dataanalysis) | get\_rV\_curves()  (in PyDSTool.Toolbox.fracdim) | getTrajTimeInterval()  (in Model) | | Gauss  (in PyDSTool.Toolbox.fracdim) | get\_slope\_info()  (in PyDSTool.Toolbox.ParamEst) | getTrajTimePartitions()  (in Model) | | Gauss  (in PyDSTool.Toolbox.makeSloppyModel) | get\_spike\_data  (in PyDSTool.Toolbox.neuro\_data) | getVW()  (in BorderMethod) | | Gauss  (in PyDSTool.Toolbox.neuralcomp) | get\_spike\_model  (in PyDSTool.Toolbox.neuro\_data) | getWarnings()  (in VarDiagnostics) | | Gauss  (in PyDSTool.Toolbox.phaseplane) | get\_symbol\_sequence()  (in PyDSTool.Toolbox.dssrt) | getWarnings()  (in Diagnostics) | | Gauss  (in PyDSTool.Toolbox.synthetic\_data) | get\_taus()  (in PyDSTool.Toolbox.dssrt) | GH\_Hopf  (in PyDSTool.PyCont.TestFunc) | | Gauss  (in PyDSTool.Toolbox.syntheticdata) | get\_test\_traj()  (in dsInterface) | GH\_Hopf\_One  (in PyDSTool.PyCont.TestFunc) | | GDescriptor  (in PyDSTool.ModelConstructor') | get\_test\_traj()  (in extModelInterface) | GHPoint  (in PyDSTool.PyCont.BifPoint) | | ge()  (in Verbose) | get\_test\_traj()  (in intModelInterface) | GLOBAL  (in PyDSTool.fixedpickle) | | genDB  (in PyDSTool.Generator.Dopri\_ODEsystem') | get\_transition()  (in FSM) | golden\_section  (in PyDSTool.Toolbox.optimizers.line\_search) | | genDB  (in PyDSTool.Generator.Radau\_ODEsystem') | get\_transition()  (in ObjFSM) | GoldenSectionSearch  (in PyDSTool.Toolbox.optimizers.line\_search.golden\_section) | | genDB  (in PyDSTool.Generator.baseclasses) | getActiveEvents()  (in EventStruct) | goldfeld\_step  (in PyDSTool.Toolbox.optimizers.step) | | genDB  (in PyDSTool.Toolbox.NineML) | getAllEvents()  (in EventStruct) | GoldfeldStep  (in PyDSTool.Toolbox.optimizers.step.goldfeld\_step) | | genDB  (in PyDSTool.Toolbox.dataanalysis) | getAuxVars()  (in PyDSTool.Model) | goldstein\_price\_step  (in PyDSTool.Toolbox.optimizers.step) | | genDB  (in PyDSTool.Toolbox.phaseplane) | getDataPoints()  (in HybridVariable) | goldstein\_rule  (in PyDSTool.Toolbox.optimizers.line\_search) | | genDB  (in PyDSTool.Toolbox.synthetic\_data) | getDataPoints()  (in Variable) | GoldsteinPriceStep  (in PyDSTool.Toolbox.optimizers.step.goldstein\_price\_step) | | genDB  (in PyDSTool.Toolbox.syntheticdata) | getdim()  (in PyDSTool.Symbolic) | GoldsteinRule  (in PyDSTool.Toolbox.optimizers.line\_search.goldstein\_rule) | | genDBClass  (in PyDSTool.Generator.baseclasses) | getDSAlgPars()  (in Model) | grad\_curvature()  (in nullcline) | | generate\_ball()  (in PyDSTool.Toolbox.synthetic\_data) | getDSEventActive()  (in Model) | grad\_curvature\_at\_sample\_points()  (in nullcline) | | generate\_ball()  (in PyDSTool.Toolbox.syntheticdata) | getDSEventTerm()  (in Model) | grad\_from\_psens()  (in PyDSTool.Toolbox.ParamEst) | | generate\_discspiral()  (in PyDSTool.Toolbox.synthetic\_data) | getEndPoint()  (in Model) | gradient()  (in CenteredFiniteDifferences) | | generate\_discspiral()  (in PyDSTool.Toolbox.syntheticdata) | getEndTime()  (in Model) | gradient()  (in ForwardFiniteDifferences) | | generate\_hypercube()  (in PyDSTool.Toolbox.synthetic\_data) | getErrors()  (in Diagnostics) | gradient()  (in ForwardFiniteDifferencesCache) | | generate\_hypercube()  (in PyDSTool.Toolbox.syntheticdata) | getEventMappings()  (in Model) | gradient()  (in Quadratic) | | generate\_spiral()  (in PyDSTool.Toolbox.synthetic\_data) | getEvents()  (in Generator) | gradient()  (in Powell) | | generate\_spiral()  (in PyDSTool.Toolbox.syntheticdata) | getEvents()  (in Trajectory) | gradient()  (in Quadratic) | | generate\_swirl()  (in PyDSTool.Toolbox.synthetic\_data) | getEventTimes()  (in Generator) | gradient()  (in Rosenbrock) | | generate\_swirl()  (in PyDSTool.Toolbox.syntheticdata) | getEventTimes()  (in Trajectory) | gradient\_step  (in PyDSTool.Toolbox.optimizers.step) | | generateAuxFns()  (in FuncSpec) | getFlowJac()  (in PyDSTool.PyCont.misc) | gradient\_total\_residual()  (in LMpest) | | generateSpec()  (in FuncSpec) | getFlowMaps()  (in PyDSTool.PyCont.misc) | GradientCriterion  (in PyDSTool.Toolbox.optimizers.criterion.criteria) | | Generator  (in PyDSTool) | getGenerator()  (in GeneratorConstructor) | GradientStep  (in PyDSTool.Toolbox.optimizers.step.gradient\_step) | | Generator  (in PyDSTool.Generator.baseclasses) | getGlobalName()  (in regObject) | greater  (in PyDSTool.PyCont.ContClass') | | GeneratorConstructor  (in PyDSTool.ModelConstructor') | getHighLevelEvents()  (in EventStruct) | greater  (in PyDSTool.Toolbox.NineML) | | GeneratorInterface  (in PyDSTool.MProject) | getIndices()  (in PointInfo) | greater  (in PyDSTool.Toolbox.dataanalysis) | | GenSpecHelper  (in PyDSTool.Generator.baseclasses) | getLabels()  (in PointInfo) | greater  (in PyDSTool.Toolbox.phaseplane) | | GenSpecInfoObj  (in PyDSTool.Generator.baseclasses) | getLeftEvecs()  (in PyDSTool.PyCont.misc) | greater  (in PyDSTool.Toolbox.synthetic\_data) | | GenTransform  (in PyDSTool.MProject) | getlftrtD()  (in PyDSTool.Symbolic) | greater  (in PyDSTool.Toolbox.syntheticdata) | | geom\_feature  (in PyDSTool.Toolbox.neuro\_data) | getLowLevelEvents()  (in EventStruct) | greater  (in PyDSTool.common) | | GET  (in PyDSTool.fixedpickle) | getModel()  (in ModelConstructor) | greater  (in PyDSTool.utils) | | get()  (in Generator) | getNonActiveEvents()  (in EventStruct) | greater  (in matplotlib.pylab) | | get()  (in Interval) | getNonPreciseEvents()  (in EventStruct) | greater\_equal  (in PyDSTool.PyCont.ContClass') | | get()  (in GeneratorInterface) | getNonTermEvents()  (in EventStruct) | greater\_equal  (in PyDSTool.Toolbox.ActivationFuncs) | | get()  (in ModelInterface) | getPreciseEvents()  (in EventStruct) | greater\_equal  (in PyDSTool.Toolbox.DSSRT\_tools) | | get()  (in Point) | Getrandbits  (in PyDSTool) | greater\_equal  (in PyDSTool.Toolbox.InputProfile) | | get()  (in pargs) | Getrandbits  (in PyDSTool.ModelSpec') | greater\_equal  (in PyDSTool.Toolbox.ModelHelper) | | get()  (in Point2D) | Getrandbits  (in PyDSTool.Symbolic) | greater\_equal  (in PyDSTool.Toolbox.NineML) | | get()  (in args) | Getrandbits  (in PyDSTool.Toolbox.ActivationFuncs) | greater\_equal  (in PyDSTool.Toolbox.adjointPRC) | | get()  (in Pickler) | Getrandbits  (in PyDSTool.Toolbox.DSSRT\_tools) | greater\_equal  (in PyDSTool.Toolbox.dataanalysis) | | get\_burst\_active\_phase  (in PyDSTool.Toolbox.neuro\_data) | Getrandbits  (in PyDSTool.Toolbox.InputProfile) | greater\_equal  (in PyDSTool.Toolbox.fracdim) | | get\_burst\_dc\_offset  (in PyDSTool.Toolbox.neuro\_data) | Getrandbits  (in PyDSTool.Toolbox.ModelHelper) | greater\_equal  (in PyDSTool.Toolbox.makeSloppyModel) | | get\_burst\_downsweep  (in PyDSTool.Toolbox.neuro\_data) | Getrandbits  (in PyDSTool.Toolbox.NineML) | greater\_equal  (in PyDSTool.Toolbox.neuralcomp) | | get\_burst\_duration  (in PyDSTool.Toolbox.neuro\_data) | Getrandbits  (in PyDSTool.Toolbox.adjointPRC) | greater\_equal  (in PyDSTool.Toolbox.phaseplane) | | get\_burst\_isi\_env  (in PyDSTool.Toolbox.neuro\_data) | Getrandbits  (in PyDSTool.Toolbox.dataanalysis) | greater\_equal  (in PyDSTool.Toolbox.synthetic\_data) | | get\_burst\_num\_spikes  (in PyDSTool.Toolbox.neuro\_data) | Getrandbits  (in PyDSTool.Toolbox.fracdim) | greater\_equal  (in PyDSTool.Toolbox.syntheticdata) | | get\_burst\_passive\_extent  (in PyDSTool.Toolbox.neuro\_data) | Getrandbits  (in PyDSTool.Toolbox.makeSloppyModel) | greater\_equal  (in PyDSTool) | | get\_burst\_peak\_env  (in PyDSTool.Toolbox.neuro\_data) | Getrandbits  (in PyDSTool.Toolbox.neuralcomp) | greater\_equal  (in matplotlib.pylab) | |

  
  

| Home | Trees | Indices | Help | | PyDSTool | | --- | |
| --- | --- | --- | --- | --- | --- |

|  |  |
| --- | --- |
| Generated by Epydoc 3.0.1 on Fri May 4 15:23:57 2012 | http://epydoc.sourceforge.net |
