# Supplementary material for: Hybrid Models and Biological Model Reduction with PyDSTool
Source: PLoS Comput Biol. 2012 Aug 9;8(8):e1002628. doi: 10.1371/journal.pcbi.1002628 (PMC3415397; doi:10.1371/journal.pcbi.1002628)
Supplement: Text S4 — Complete source code for the PyDSTool package (version 0.88.120504). Includes API documentation and help files linking to web pages. This file is identical to the current public release on Sourceforge.net. (ZIP) [file pcbi.1002628.s004.zip › PyDSTool/html/identifier-index-H.html]

xml version="1.0" encoding="ascii"?


Identifier Index


| Home | Trees | Indices | Help | | PyDSTool | | --- | |
| --- | --- | --- | --- | --- | --- |

|  |  |  |  |
| --- | --- | --- | --- |
|  | |  | | --- | | [hide private] | | [frames] | no frames] | |

|  |  |
| --- | --- |
| Identifier Index | [ A B C D E F G H I J K L M N O P Q R S T U V W X Y Z \_ ] |

|  |  |  |  |  |  |  |  |  |  |  |  |  |  |  |  |  |  |  |  |  |  |  |  |  |  |  |  |  |  |  |  |  |  |  |  |  |  |  |  |  |  |  |  |  |  |  |  |  |  |  |  |  |  |  |  |  |  |  |  |  |  |  |  |  |  |  |  |  |  |  |  |  |  |  |  |  |  |  |  |  |  |  |  |  |  |  |  |  |  |  |  |  |  |  |  |  |  |  |  |  |  |  |  |  |  |  |
| --- | --- | --- | --- | --- | --- | --- | --- | --- | --- | --- | --- | --- | --- | --- | --- | --- | --- | --- | --- | --- | --- | --- | --- | --- | --- | --- | --- | --- | --- | --- | --- | --- | --- | --- | --- | --- | --- | --- | --- | --- | --- | --- | --- | --- | --- | --- | --- | --- | --- | --- | --- | --- | --- | --- | --- | --- | --- | --- | --- | --- | --- | --- | --- | --- | --- | --- | --- | --- | --- | --- | --- | --- | --- | --- | --- | --- | --- | --- | --- | --- | --- | --- | --- | --- | --- | --- | --- | --- | --- | --- | --- | --- | --- | --- | --- | --- | --- | --- | --- | --- | --- | --- | --- | --- | --- | --- |
| H | |  |  |  | | --- | --- | --- | | has\_exact\_traj()  (in Model) | haveJacobian\_pars()  (in MapSystem) | HOURLY  (in PyDSTool.PyCont.ContClass') | | has\_key()  (in Point) | haveJacobian\_pars()  (in ODEsystem) | HOURLY  (in matplotlib.pylab) | | has\_key()  (in Point2D) | haveJacobian\_pars()  (in Generator) | html\_static\_path  (in PyDSTool.conf) | | has\_key()  (in args) | haveJacobian\_pars()  (in HybridModel) | html\_theme  (in PyDSTool.conf) | | has\_key()  (in symbolMapClass) | haveJacobian\_pars()  (in NonHybridModel) | htmlhelp\_basename  (in PyDSTool.conf) | | has\_test\_traj()  (in extModelInterface) | haveMass()  (in ODEsystem) | HybridModel  (in PyDSTool.Model) | | has\_test\_traj()  (in intModelInterface) | head()  (in PyDSTool.Toolbox.event\_driven\_simulator) | HybridTrajectory  (in PyDSTool.Trajectory') | | hasErrors()  (in Diagnostics) | helpers  (in PyDSTool.Toolbox.optimizers) | HybridVariable  (in PyDSTool.Variable') | | hasWarnings()  (in Diagnostics) | helpers\_\_all\_\_  (in PyDSTool.Toolbox.optimizers.helpers) | hyperbolic\_line\_search  (in PyDSTool.Toolbox.optimizers.line\_search) | | HAVE\_PSYCO  (in PyDSTool.Generator.DDEsystem) | hess()  (in PyDSTool.PyCont.misc) | HyperbolicLineSearch  (in PyDSTool.Toolbox.optimizers.line\_search.hyperbolic\_line\_search) | | HAVE\_PSYCO  (in PyDSTool.Generator.EmbeddedSysGen') | hess3()  (in PyDSTool.PyCont.misc) | Hypot  (in PyDSTool) | | HAVE\_PSYCO  (in PyDSTool.Generator.Euler\_ODEsystem') | hessian()  (in CenteredFiniteDifferences) | Hypot  (in PyDSTool.ModelSpec') | | HAVE\_PSYCO  (in PyDSTool.Generator.ExplicitFnGen') | hessian()  (in ForwardFiniteDifferences) | hypot  (in PyDSTool.PyCont.ContClass') | | HAVE\_PSYCO  (in PyDSTool.Generator.ImplicitFnGen') | hessian()  (in ForwardFiniteDifferencesCache) | Hypot  (in PyDSTool.Symbolic) | | HAVE\_PSYCO  (in PyDSTool.Generator.MapSystem') | hessian()  (in LMQuadratic) | hypot  (in PyDSTool.Symbolic) | | HAVE\_PSYCO  (in PyDSTool.Generator.ODEsystem') | hessian()  (in Quadratic) | Hypot  (in PyDSTool.Toolbox.ActivationFuncs) | | HAVE\_PSYCO  (in PyDSTool.Generator.Vode\_ODEsystem') | hessian()  (in Powell) | Hypot  (in PyDSTool.Toolbox.DSSRT\_tools) | | HAVE\_PSYCO  (in PyDSTool.Generator.baseclasses) | hessian()  (in Quadratic) | Hypot  (in PyDSTool.Toolbox.InputProfile) | | HAVE\_PSYCO  (in PyDSTool.Toolbox.NineML) | hessian()  (in Rosenbrock) | Hypot  (in PyDSTool.Toolbox.ModelHelper) | | HAVE\_PSYCO  (in PyDSTool.Toolbox.ParamEst) | Hessian\_total\_residual()  (in LMpest) | Hypot  (in PyDSTool.Toolbox.NineML) | | HAVE\_PSYCO  (in PyDSTool.Toolbox.dataanalysis) | hessianvect()  (in CenteredFiniteDifferences) | hypot  (in PyDSTool.Toolbox.NineML) | | HAVE\_PSYCO  (in PyDSTool.Toolbox.phaseplane) | hessianvect()  (in ForwardFiniteDifferences) | Hypot  (in PyDSTool.Toolbox.adjointPRC) | | HAVE\_PSYCO  (in PyDSTool.Toolbox.synthetic\_data) | HIGHEST\_PROTOCOL  (in PyDSTool.fixedpickle) | Hypot  (in PyDSTool.Toolbox.dataanalysis) | | HAVE\_PSYCO  (in PyDSTool.Toolbox.syntheticdata) | HIGHEST\_PROTOCOL  (in cPickle) | hypot  (in PyDSTool.Toolbox.dataanalysis) | | haveJacobian()  (in EmbeddedSysGen) | HighLevelEvent  (in PyDSTool.Events) | Hypot  (in PyDSTool.Toolbox.fracdim) | | haveJacobian()  (in ExplicitFnGen) | hopf\_args\_list  (in PyDSTool.PyCont.Continuation) | Hypot  (in PyDSTool.Toolbox.makeSloppyModel) | | haveJacobian()  (in ImplicitFnGen) | hopf\_bif\_points  (in PyDSTool.PyCont.Continuation) | Hypot  (in PyDSTool.Toolbox.neuralcomp) | | haveJacobian()  (in MapSystem) | Hopf\_Bor  (in PyDSTool.PyCont.TestFunc) | Hypot  (in PyDSTool.Toolbox.phaseplane) | | haveJacobian()  (in ODEsystem) | Hopf\_Det  (in PyDSTool.PyCont.TestFunc) | hypot  (in PyDSTool.Toolbox.phaseplane) | | haveJacobian()  (in Generator) | Hopf\_Double\_Bor\_One  (in PyDSTool.PyCont.TestFunc) | Hypot  (in PyDSTool.Toolbox.synthetic\_data) | | haveJacobian()  (in HybridModel) | Hopf\_Double\_Bor\_Two  (in PyDSTool.PyCont.TestFunc) | hypot  (in PyDSTool.Toolbox.synthetic\_data) | | haveJacobian()  (in NonHybridModel) | Hopf\_Eig  (in PyDSTool.PyCont.TestFunc) | Hypot  (in PyDSTool.Toolbox.syntheticdata) | | haveJacobian\_pars()  (in EmbeddedSysGen) | HopfCurveOne  (in PyDSTool.PyCont.Continuation) | hypot  (in PyDSTool.Toolbox.syntheticdata) | | haveJacobian\_pars()  (in ExplicitFnGen) | HopfCurveTwo  (in PyDSTool.PyCont.Continuation) | hypot  (in matplotlib.pylab) | | haveJacobian\_pars()  (in ImplicitFnGen) | HopfPoint  (in PyDSTool.PyCont.BifPoint) | HZConjugateGradientStep()  (in PyDSTool.Toolbox.optimizers.step.conjugate\_gradient\_step) | |

  
  

| Home | Trees | Indices | Help | | PyDSTool | | --- | |
| --- | --- | --- | --- | --- | --- |

|  |  |
| --- | --- |
| Generated by Epydoc 3.0.1 on Fri May 4 15:23:57 2012 | http://epydoc.sourceforge.net |
