# Supplementary material for: Hybrid Models and Biological Model Reduction with PyDSTool
Source: PLoS Comput Biol. 2012 Aug 9;8(8):e1002628. doi: 10.1371/journal.pcbi.1002628 (PMC3415397; doi:10.1371/journal.pcbi.1002628)
Supplement: Text S4 — Complete source code for the PyDSTool package (version 0.88.120504). Includes API documentation and help files linking to web pages. This file is identical to the current public release on Sourceforge.net. (ZIP) [file pcbi.1002628.s004.zip › PyDSTool/html/identifier-index-I.html]

xml version="1.0" encoding="ascii"?


Identifier Index


| Home | Trees | Indices | Help | | PyDSTool | | --- | |
| --- | --- | --- | --- | --- | --- |

|  |  |  |  |
| --- | --- | --- | --- |
|  | |  | | --- | | [hide private] | | [frames] | no frames] | |

|  |  |
| --- | --- |
| Identifier Index | [ A B C D E F G H I J K L M N O P Q R S T U V W X Y Z \_ ] |

|  |  |  |  |  |  |  |  |  |  |  |  |  |  |  |  |  |  |  |  |  |  |  |  |  |  |  |  |  |  |  |  |  |  |  |  |  |  |  |  |  |  |  |  |  |  |  |  |  |  |  |  |  |  |  |  |  |  |  |  |  |  |  |  |  |  |  |  |  |  |  |  |  |  |  |  |  |  |  |  |  |  |  |  |  |  |  |  |  |  |  |  |  |  |  |  |  |  |  |  |  |  |  |  |  |  |  |  |  |  |  |  |  |  |  |  |  |  |  |  |  |  |  |  |  |  |  |  |  |  |  |  |  |  |  |  |  |  |  |  |  |  |  |  |  |  |  |  |  |  |  |  |  |  |  |  |  |  |  |  |  |  |  |  |  |  |  |  |  |  |  |  |  |  |  |  |  |  |  |  |  |  |  |  |  |  |  |  |  |  |  |  |  |  |  |  |  |  |  |  |  |  |  |  |  |  |  |  |  |  |  |  |  |  |  |  |  |  |  |  |  |  |  |  |  |  |  |  |  |  |  |  |  |  |  |  |  |  |  |  |  |  |  |  |  |  |  |  |  |  |  |  |  |  |  |  |  |  |  |  |  |  |  |  |  |  |  |  |  |  |  |  |  |  |  |  |  |  |  |  |  |  |  |  |  |  |  |  |  |  |  |  |  |  |  |  |  |  |  |  |  |  |  |  |  |  |  |  |  |  |  |  |  |  |  |  |  |  |  |  |  |  |  |  |  |  |  |  |  |  |  |  |  |  |  |  |  |  |  |  |  |  |  |  |  |  |  |  |  |  |  |  |  |  |  |  |
| --- | --- | --- | --- | --- | --- | --- | --- | --- | --- | --- | --- | --- | --- | --- | --- | --- | --- | --- | --- | --- | --- | --- | --- | --- | --- | --- | --- | --- | --- | --- | --- | --- | --- | --- | --- | --- | --- | --- | --- | --- | --- | --- | --- | --- | --- | --- | --- | --- | --- | --- | --- | --- | --- | --- | --- | --- | --- | --- | --- | --- | --- | --- | --- | --- | --- | --- | --- | --- | --- | --- | --- | --- | --- | --- | --- | --- | --- | --- | --- | --- | --- | --- | --- | --- | --- | --- | --- | --- | --- | --- | --- | --- | --- | --- | --- | --- | --- | --- | --- | --- | --- | --- | --- | --- | --- | --- | --- | --- | --- | --- | --- | --- | --- | --- | --- | --- | --- | --- | --- | --- | --- | --- | --- | --- | --- | --- | --- | --- | --- | --- | --- | --- | --- | --- | --- | --- | --- | --- | --- | --- | --- | --- | --- | --- | --- | --- | --- | --- | --- | --- | --- | --- | --- | --- | --- | --- | --- | --- | --- | --- | --- | --- | --- | --- | --- | --- | --- | --- | --- | --- | --- | --- | --- | --- | --- | --- | --- | --- | --- | --- | --- | --- | --- | --- | --- | --- | --- | --- | --- | --- | --- | --- | --- | --- | --- | --- | --- | --- | --- | --- | --- | --- | --- | --- | --- | --- | --- | --- | --- | --- | --- | --- | --- | --- | --- | --- | --- | --- | --- | --- | --- | --- | --- | --- | --- | --- | --- | --- | --- | --- | --- | --- | --- | --- | --- | --- | --- | --- | --- | --- | --- | --- | --- | --- | --- | --- | --- | --- | --- | --- | --- | --- | --- | --- | --- | --- | --- | --- | --- | --- | --- | --- | --- | --- | --- | --- | --- | --- | --- | --- | --- | --- | --- | --- | --- | --- | --- | --- | --- | --- | --- | --- | --- | --- | --- | --- | --- | --- | --- | --- | --- | --- | --- | --- | --- | --- | --- | --- | --- | --- | --- | --- | --- | --- | --- | --- | --- | --- | --- | --- | --- | --- | --- | --- | --- | --- | --- | --- | --- | --- | --- | --- | --- | --- | --- | --- | --- | --- | --- | --- | --- | --- | --- | --- | --- | --- | --- | --- | --- | --- | --- | --- | --- | --- | --- | --- | --- | --- | --- | --- | --- | --- | --- | --- | --- |
| I | |  |  |  | | --- | --- | --- | | i  (in PyDSTool.Symbolic) | info()  (in FoldPoint) | isdiscrete()  (in PyDSTool.Variable') | | i  (in Verbose) | info()  (in GHPoint) | isEmpty()  (in Component) | | Id  (in PyDSTool.Toolbox.mechmatlib) | info()  (in HopfPoint) | isEmpty()  (in LeafComponent) | | identity\_map  (in PyDSTool.Toolbox.event\_driven\_simulator) | info()  (in LPCPoint) | isEmpty()  (in ModelSpec) | | idfn()  (in PyDSTool.common) | info()  (in NSPoint) | isfinite  (in PyDSTool.Generator.ADMC\_ODEsystem') | | idmap  (in PyDSTool.Toolbox.event\_driven\_simulator) | info()  (in PDPoint) | isfinite  (in PyDSTool.Generator.Dopri\_ODEsystem') | | ijtoind()  (in PyDSTool.PyCont.misc) | info()  (in ZHPoint) | isfinite  (in PyDSTool.Generator.EmbeddedSysGen') | | ImpFuncSpec  (in PyDSTool.FuncSpec') | info()  (in ContClass) | isfinite  (in PyDSTool.Generator.Euler\_ODEsystem') | | ImplicitFnGen  (in PyDSTool.Generator.ImplicitFnGen') | info()  (in Continuation) | isfinite  (in PyDSTool.Generator.ExplicitFnGen') | | ImplicitFnGen'  (in PyDSTool.Generator) | info()  (in LimitCycleCurve) | isfinite  (in PyDSTool.Generator.ExtrapolateTable') | | importPointset()  (in PyDSTool.Points) | info()  (in Quantity) | isfinite  (in PyDSTool.Generator.ImplicitFnGen') | | in\_degree()  (in PyDSTool.Toolbox.FR) | info()  (in epoch) | isfinite  (in PyDSTool.Generator.InterpolateTable') | | inc()  (in base\_n\_counter) | info()  (in Point2D) | isfinite  (in PyDSTool.Generator.LookupTable') | | increment()  (in data\_bins) | info()  (in HybridTrajectory) | isfinite  (in PyDSTool.Generator.MapSystem') | | increment()  (in data\_bins) | info()  (in Trajectory) | isfinite  (in PyDSTool.Generator.ODEsystem') | | indent()  (in PyDSTool.Toolbox.dssrt) | info()  (in HybridVariable) | isfinite  (in PyDSTool.Generator.Radau\_ODEsystem') | | index\_exp  (in PyDSTool.PyCont.ContClass') | info()  (in Variable) | isfinite  (in PyDSTool.Generator.Vode\_ODEsystem') | | index\_exp  (in PyDSTool.Toolbox.ActivationFuncs) | info()  (in Diagnostics) | isfinite  (in PyDSTool.Interval') | | index\_exp  (in PyDSTool.Toolbox.DSSRT\_tools) | info()  (in args) | isfinite  (in PyDSTool.ModelConstructor') | | index\_exp  (in PyDSTool.Toolbox.InputProfile) | info()  (in PyDSTool.utils) | isfinite  (in PyDSTool.ModelSpec') | | index\_exp  (in PyDSTool.Toolbox.ModelHelper) | information\_criteria  (in PyDSTool.Toolbox.optimizers.criterion) | isfinite  (in PyDSTool.PyCont.ContClass') | | index\_exp  (in PyDSTool.Toolbox.NineML) | infty  (in PyDSTool.PyCont.ContClass') | isfinite  (in PyDSTool.PyCont.Continuation) | | index\_exp  (in PyDSTool.Toolbox.adjointPRC) | infty  (in PyDSTool.Toolbox.ActivationFuncs) | isfinite  (in PyDSTool.PyCont.misc) | | index\_exp  (in PyDSTool.Toolbox.dataanalysis) | infty  (in PyDSTool.Toolbox.DSSRT\_tools) | isfinite  (in PyDSTool.Symbolic) | | index\_exp  (in PyDSTool.Toolbox.fracdim) | infty  (in PyDSTool.Toolbox.InputProfile) | isfinite  (in PyDSTool.Toolbox.NineML) | | index\_exp  (in PyDSTool.Toolbox.makeSloppyModel) | infty  (in PyDSTool.Toolbox.ModelHelper) | isfinite  (in PyDSTool.Toolbox.PySCes\_SBML) | | index\_exp  (in PyDSTool.Toolbox.neuralcomp) | infty  (in PyDSTool.Toolbox.NineML) | isfinite  (in PyDSTool.Toolbox.dataanalysis) | | index\_exp  (in PyDSTool.Toolbox.phaseplane) | infty  (in PyDSTool.Toolbox.adjointPRC) | isfinite  (in PyDSTool.Toolbox.event\_driven\_simulator) | | index\_exp  (in PyDSTool.Toolbox.synthetic\_data) | infty  (in PyDSTool.Toolbox.dataanalysis) | isfinite  (in PyDSTool.Toolbox.phaseplane) | | index\_exp  (in PyDSTool.Toolbox.syntheticdata) | infty  (in PyDSTool.Toolbox.fracdim) | isfinite  (in PyDSTool.Toolbox.synthetic\_data) | | index\_exp  (in PyDSTool) | infty  (in PyDSTool.Toolbox.makeSloppyModel) | isfinite  (in PyDSTool.Toolbox.syntheticdata) | | index\_exp  (in matplotlib.pylab) | infty  (in PyDSTool.Toolbox.neuralcomp) | isfinite  (in PyDSTool.Variable') | | indtoij()  (in PyDSTool.PyCont.misc) | infty  (in PyDSTool.Toolbox.phaseplane) | isfinite  (in PyDSTool.common) | | Inf  (in PyDSTool.Generator.ADMC\_ODEsystem') | infty  (in PyDSTool.Toolbox.synthetic\_data) | isfinite  (in PyDSTool.utils) | | Inf  (in PyDSTool.Generator.Dopri\_ODEsystem') | infty  (in PyDSTool.Toolbox.syntheticdata) | isfinite  (in matplotlib.pylab) | | Inf  (in PyDSTool.Generator.EmbeddedSysGen') | infty  (in PyDSTool) | isfinite()  (in Interval) | | Inf  (in PyDSTool.Generator.Euler\_ODEsystem') | infty  (in matplotlib.pylab) | isHierarchicalName()  (in PyDSTool.parseUtils) | | Inf  (in PyDSTool.Generator.ExplicitFnGen') | inh\_synapse  (in PyDSTool.Toolbox.neuralcomp) | isincreasing()  (in PyDSTool.common) | | Inf  (in PyDSTool.Generator.ExtrapolateTable') | initialize\_model()  (in intModelInterface) | isinf  (in PyDSTool.Interval') | | Inf  (in PyDSTool.Generator.ImplicitFnGen') | initializeDisplay()  (in PyDSTool.PyCont.Plotting) | isinf  (in PyDSTool.PyCont.ContClass') | | Inf  (in PyDSTool.Generator.InterpolateTable') | Input  (in PyDSTool.Symbolic) | isinf  (in PyDSTool.Toolbox.ActivationFuncs) | | Inf  (in PyDSTool.Generator.LookupTable') | InputProfile  (in PyDSTool.Toolbox) | isinf  (in PyDSTool.Toolbox.DSSRT\_tools) | | Inf  (in PyDSTool.Generator.MapSystem') | insert()  (in Pointset) | isinf  (in PyDSTool.Toolbox.InputProfile) | | Inf  (in PyDSTool.Generator.ODEsystem') | insertInOrder()  (in PyDSTool.common) | isinf  (in PyDSTool.Toolbox.ModelHelper) | | Inf  (in PyDSTool.Generator.Radau\_ODEsystem') | INST  (in PyDSTool.fixedpickle) | isinf  (in PyDSTool.Toolbox.NineML) | | Inf  (in PyDSTool.Generator.Vode\_ODEsystem') | INT  (in PyDSTool.fixedpickle) | isinf  (in PyDSTool.Toolbox.adjointPRC) | | Inf  (in PyDSTool.Interval') | integrate()  (in euler\_solver) | isinf  (in PyDSTool.Toolbox.dataanalysis) | | Inf  (in PyDSTool.ModelConstructor') | integrate()  (in ode) | isinf  (in PyDSTool.Toolbox.fracdim) | | Inf  (in PyDSTool.ModelSpec') | integrator  (in PyDSTool.integrator') | isinf  (in PyDSTool.Toolbox.makeSloppyModel) | | Inf  (in PyDSTool.PyCont.ContClass') | integrator'  (in PyDSTool) | isinf  (in PyDSTool.Toolbox.neuralcomp) | | inf  (in PyDSTool.PyCont.ContClass') | integrator\_classes  (in IntegratorBase) | isinf  (in PyDSTool.Toolbox.phaseplane) | | Inf  (in PyDSTool.PyCont.Continuation) | IntegratorBase  (in PyDSTool.scipy\_ode) | isinf  (in PyDSTool.Toolbox.synthetic\_data) | | Inf  (in PyDSTool.PyCont.misc) | interp0d  (in PyDSTool.common) | isinf  (in PyDSTool.Toolbox.syntheticdata) | | Inf  (in PyDSTool.Symbolic) | interp1d  (in PyDSTool.common) | isinf  (in PyDSTool.integrator') | | inf  (in PyDSTool.Toolbox.ActivationFuncs) | interp\_axis  (in interpclass) | isinf  (in PyDSTool) | | inf  (in PyDSTool.Toolbox.DSSRT\_tools) | interpclass  (in PyDSTool.common) | isinf  (in matplotlib.pylab) | | inf  (in PyDSTool.Toolbox.InputProfile) | InterpolateTable  (in PyDSTool.Generator.InterpolateTable') | isinputcts()  (in PyDSTool.Variable') | | inf  (in PyDSTool.Toolbox.ModelHelper) | InterpolateTable'  (in PyDSTool.Generator) | isinputdiscrete()  (in PyDSTool.Variable') | | Inf  (in PyDSTool.Toolbox.NineML) | intersect()  (in Interval) | isinstantiable()  (in GDescriptor) | | inf  (in PyDSTool.Toolbox.NineML) | intersect()  (in PyDSTool.common) | isinstantiable()  (in MDescriptor) | | inf  (in PyDSTool.Toolbox.adjointPRC) | intersect()  (in PyDSTool.utils) | isIntegerToken()  (in PyDSTool.parseUtils) | | Inf  (in PyDSTool.Toolbox.dataanalysis) | Interval  (in PyDSTool.Interval') | isinterval()  (in PyDSTool.Interval') | | inf  (in PyDSTool.Toolbox.dataanalysis) | Interval'  (in PyDSTool) | ismonotonic()  (in PyDSTool.common) | | Inf  (in PyDSTool.Toolbox.event\_driven\_simulator) | IntervalMembership  (in PyDSTool.Interval') | isMultiDef()  (in PyDSTool.Symbolic) | | inf  (in PyDSTool.Toolbox.fracdim) | intModelInterface  (in PyDSTool.MProject) | isMultiDefClash()  (in PyDSTool.Symbolic) | | inf  (in PyDSTool.Toolbox.makeSloppyModel) | inverse()  (in symbolMapClass) | isMultiRef()  (in PyDSTool.Symbolic) | | inf  (in PyDSTool.Toolbox.neuralcomp) | inverseMathNameMap  (in PyDSTool.ModelSpec') | isNameToken()  (in PyDSTool.parseUtils) | | Inf  (in PyDSTool.Toolbox.phaseplane) | inverseMathNameMap  (in PyDSTool.Symbolic) | isnan  (in PyDSTool.Generator.ADMC\_ODEsystem') | | inf  (in PyDSTool.Toolbox.phaseplane) | inverseMathNameMap  (in PyDSTool.Toolbox.NineML) | isnan  (in PyDSTool.Interval') | | Inf  (in PyDSTool.Toolbox.synthetic\_data) | inverseMathNameMap  (in PyDSTool.Toolbox.dataanalysis) | isnan  (in PyDSTool.PyCont.ContClass') | | inf  (in PyDSTool.Toolbox.synthetic\_data) | inverseMathNameMap  (in PyDSTool.Toolbox.phaseplane) | isnan  (in PyDSTool.PyCont.Continuation) | | Inf  (in PyDSTool.Toolbox.syntheticdata) | inverseMathNameMap  (in PyDSTool.Toolbox.synthetic\_data) | isnan  (in PyDSTool.Toolbox.NineML) | | inf  (in PyDSTool.Toolbox.syntheticdata) | inverseMathNameMap  (in PyDSTool.Toolbox.syntheticdata) | isnan  (in PyDSTool.Toolbox.dataanalysis) | | Inf  (in PyDSTool.Trajectory') | invert  (in PyDSTool.PyCont.ContClass') | isnan  (in PyDSTool.Toolbox.phaseplane) | | Inf  (in PyDSTool.Variable') | invert  (in PyDSTool.Toolbox.ActivationFuncs) | isnan  (in PyDSTool.Toolbox.synthetic\_data) | | Inf  (in PyDSTool.common) | invert  (in PyDSTool.Toolbox.DSSRT\_tools) | isnan  (in PyDSTool.Toolbox.syntheticdata) | | inf  (in PyDSTool) | invert  (in PyDSTool.Toolbox.InputProfile) | isnan  (in matplotlib.pylab) | | Inf  (in PyDSTool.integrator') | invert  (in PyDSTool.Toolbox.ModelHelper) | isnotzero()  (in PyDSTool.PyCont.misc) | | Inf  (in PyDSTool.utils) | invert  (in PyDSTool.Toolbox.NineML) | isNumericToken()  (in PyDSTool.parseUtils) | | Inf  (in matplotlib.pylab) | invert  (in PyDSTool.Toolbox.adjointPRC) | isoutputcts()  (in PyDSTool.Variable') | | inf  (in matplotlib.pylab) | invert  (in PyDSTool.Toolbox.dataanalysis) | isoutputdiscrete()  (in PyDSTool.Variable') | | inf\_norm()  (in PyDSTool.Toolbox.synthetic\_data) | invert  (in PyDSTool.Toolbox.fracdim) | isparameterized()  (in PyDSTool.Points) | | inf\_norm()  (in PyDSTool.Toolbox.syntheticdata) | invert  (in PyDSTool.Toolbox.makeSloppyModel) | issingleton()  (in PyDSTool.Interval') | | Infinity  (in PyDSTool) | invert  (in PyDSTool.Toolbox.neuralcomp) | isspecdefined()  (in QuantSpec) | | Infinity  (in PyDSTool.PyCont.ContClass') | invert  (in PyDSTool.Toolbox.phaseplane) | isspecdefined()  (in Quantity) | | Infinity  (in PyDSTool.Toolbox.ActivationFuncs) | invert  (in PyDSTool.Toolbox.synthetic\_data) | isToken()  (in PyDSTool.parseUtils) | | Infinity  (in PyDSTool.Toolbox.DSSRT\_tools) | invert  (in PyDSTool.Toolbox.syntheticdata) | isUniqueSeq()  (in PyDSTool.common) | | Infinity  (in PyDSTool.Toolbox.InputProfile) | invert  (in PyDSTool) | isvector()  (in QuantSpec) | | Infinity  (in PyDSTool.Toolbox.ModelHelper) | invert  (in matplotlib.pylab) | isvector()  (in Quantity) | | Infinity  (in PyDSTool.Toolbox.NineML) | invertMap()  (in PyDSTool.common) | isVectorClause()  (in PyDSTool.parseUtils) | | Infinity  (in PyDSTool.Toolbox.adjointPRC) | invwedge()  (in PyDSTool.PyCont.misc) | iszero()  (in PyDSTool.PyCont.misc) | | Infinity  (in PyDSTool.Toolbox.dataanalysis) | is\_active  (in PyDSTool.Toolbox.dssrt) | items()  (in auxfn\_container) | | Infinity  (in PyDSTool.Toolbox.fracdim) | IS\_ALL\_VARS\_FIXED  (in PyDSTool.Toolbox.optimizers.defaults) | items()  (in condition) | | Infinity  (in PyDSTool.Toolbox.makeSloppyModel) | is\_continuous\_valued()  (in Variable) | items()  (in Point) | | Infinity  (in PyDSTool.Toolbox.neuralcomp) | is\_counter\_clockwise()  (in PyDSTool.Toolbox.phaseplane) | items()  (in Point2D) | | Infinity  (in PyDSTool.Toolbox.phaseplane) | is\_discrete\_valued()  (in Variable) | items()  (in args) | | Infinity  (in PyDSTool.Toolbox.synthetic\_data) | is\_fast  (in PyDSTool.Toolbox.dssrt) | items()  (in symbolMapClass) | | Infinity  (in PyDSTool.Toolbox.syntheticdata) | is\_inactive  (in PyDSTool.Toolbox.dssrt) | iterate()  (in ParamEst) | | Infinity  (in matplotlib.pylab) | IS\_LINE\_SEARCH\_FAILED  (in PyDSTool.Toolbox.optimizers.defaults) | iterate()  (in Optimizer) | | inflection\_zone\_leaf  (in PyDSTool.Toolbox.phaseplane) | IS\_MAX\_CPU\_TIME\_REACHED  (in PyDSTool.Toolbox.optimizers.defaults) | iterate()  (in StandardOptimizer) | | inflection\_zone\_node  (in PyDSTool.Toolbox.phaseplane) | IS\_MAX\_FUN\_EVALS\_REACHED  (in PyDSTool.Toolbox.optimizers.defaults) | iterate()  (in StandardOptimizerModifying) | | info()  (in Event) | IS\_MAX\_ITER\_REACHED  (in PyDSTool.Toolbox.optimizers.defaults) | IterationCriterion  (in PyDSTool.Toolbox.optimizers.criterion.criteria) | | info()  (in EventStruct) | IS\_MAX\_TIME\_REACHED  (in PyDSTool.Toolbox.optimizers.defaults) | IterationError  (in PyDSTool.PyCont.misc) | | info()  (in FuncSpec) | is\_min\_bracket()  (in PyDSTool.Toolbox.phaseplane) | itercool()  (in PyDSTool.Toolbox.FR) | | info()  (in Generator) | is\_modulatory  (in PyDSTool.Toolbox.dssrt) | iteritems()  (in Point) | | info()  (in Interval) | is\_monotonic()  (in nullcline) | iteritems()  (in Point2D) | | info()  (in ModelManager) | is\_most\_dominant  (in PyDSTool.Toolbox.dssrt) | iteritems()  (in args) | | info()  (in feature) | IS\_NAN\_IN\_X  (in PyDSTool.Toolbox.optimizers.defaults) | iteritems()  (in symbolMapClass) | | info()  (in Model) | is\_order1  (in PyDSTool.Toolbox.dssrt) | iterkeys()  (in Point) | | info()  (in ModelSpec) | is\_slow  (in PyDSTool.Toolbox.dssrt) | iterkeys()  (in Point2D) | | info()  (in Point) | isComplete()  (in ModelSpec) | iterkeys()  (in args) | | info()  (in Pointset) | isCompound()  (in QuantSpec) | iterkeys()  (in symbolMapClass) | | info()  (in BPoint) | isCompound()  (in parserObject) | itervalues()  (in Point) | | info()  (in BTPoint) | iscontinuous()  (in PyDSTool.Variable') | itervalues()  (in Point2D) | | info()  (in BifPoint) | isDefined()  (in Component) | itervalues()  (in args) | | info()  (in BranchPoint) | isDefined()  (in ModelSpec) | itervalues()  (in symbolMapClass) | | info()  (in CPPoint) | isDefined()  (in QuantSpec) | ixmap  (in PyDSTool.Generator.baseclasses) | | info()  (in DHPoint) | isDefined()  (in Quantity) |  | |

  
  

| Home | Trees | Indices | Help | | PyDSTool | | --- | |
| --- | --- | --- | --- | --- | --- |

|  |  |
| --- | --- |
| Generated by Epydoc 3.0.1 on Fri May 4 15:23:57 2012 | http://epydoc.sourceforge.net |
