# Supplementary material for: Hybrid Models and Biological Model Reduction with PyDSTool
Source: PLoS Comput Biol. 2012 Aug 9;8(8):e1002628. doi: 10.1371/journal.pcbi.1002628 (PMC3415397; doi:10.1371/journal.pcbi.1002628)
Supplement: Text S4 — Complete source code for the PyDSTool package (version 0.88.120504). Includes API documentation and help files linking to web pages. This file is identical to the current public release on Sourceforge.net. (ZIP) [file pcbi.1002628.s004.zip › PyDSTool/html/identifier-index-L.html]

xml version="1.0" encoding="ascii"?


Identifier Index


| Home | Trees | Indices | Help | | PyDSTool | | --- | |
| --- | --- | --- | --- | --- | --- |

|  |  |  |  |
| --- | --- | --- | --- |
|  | |  | | --- | | [hide private] | | [frames] | no frames] | |

|  |  |
| --- | --- |
| Identifier Index | [ A B C D E F G H I J K L M N O P Q R S T U V W X Y Z \_ ] |

|  |  |  |  |  |  |  |  |  |  |  |  |  |  |  |  |  |  |  |  |  |  |  |  |  |  |  |  |  |  |  |  |  |  |  |  |  |  |  |  |  |  |  |  |  |  |  |  |  |  |  |  |  |  |  |  |  |  |  |  |  |  |  |  |  |  |  |  |  |  |  |  |  |  |  |  |  |  |  |  |  |  |  |  |  |  |  |  |  |  |  |  |  |  |  |  |  |  |  |  |  |  |  |  |  |  |  |  |  |  |  |  |  |  |  |  |  |  |  |  |  |  |  |  |  |  |  |  |  |  |  |  |  |  |  |  |  |  |  |  |  |  |  |  |  |  |  |  |  |  |  |  |  |  |  |  |  |  |  |  |  |  |  |  |  |  |  |  |  |  |  |  |  |  |  |  |  |  |  |  |  |  |  |  |  |  |  |  |  |  |  |  |  |  |  |  |  |  |  |  |  |  |  |  |  |  |  |  |  |  |  |  |  |  |  |  |  |  |  |  |  |  |  |  |  |  |  |  |  |  |  |  |  |  |  |  |  |  |  |  |  |  |  |  |  |  |  |  |  |  |  |  |  |  |  |  |  |  |  |  |  |  |  |  |  |  |  |  |  |  |  |  |  |  |  |  |  |  |  |  |  |  |  |  |  |  |  |  |  |  |  |  |  |  |  |  |  |  |  |  |  |  |  |  |  |  |  |  |  |  |  |  |  |  |  |  |  |  |  |  |  |  |  |  |  |  |  |  |  |  |  |  |  |  |  |  |  |  |  |  |  |  |  |  |  |  |  |  |  |  |  |  |  |  |  |  |  |  |  |  |  |  |  |  |  |  |  |  |  |  |  |  |  |  |  |  |  |  |  |  |  |  |  |
| --- | --- | --- | --- | --- | --- | --- | --- | --- | --- | --- | --- | --- | --- | --- | --- | --- | --- | --- | --- | --- | --- | --- | --- | --- | --- | --- | --- | --- | --- | --- | --- | --- | --- | --- | --- | --- | --- | --- | --- | --- | --- | --- | --- | --- | --- | --- | --- | --- | --- | --- | --- | --- | --- | --- | --- | --- | --- | --- | --- | --- | --- | --- | --- | --- | --- | --- | --- | --- | --- | --- | --- | --- | --- | --- | --- | --- | --- | --- | --- | --- | --- | --- | --- | --- | --- | --- | --- | --- | --- | --- | --- | --- | --- | --- | --- | --- | --- | --- | --- | --- | --- | --- | --- | --- | --- | --- | --- | --- | --- | --- | --- | --- | --- | --- | --- | --- | --- | --- | --- | --- | --- | --- | --- | --- | --- | --- | --- | --- | --- | --- | --- | --- | --- | --- | --- | --- | --- | --- | --- | --- | --- | --- | --- | --- | --- | --- | --- | --- | --- | --- | --- | --- | --- | --- | --- | --- | --- | --- | --- | --- | --- | --- | --- | --- | --- | --- | --- | --- | --- | --- | --- | --- | --- | --- | --- | --- | --- | --- | --- | --- | --- | --- | --- | --- | --- | --- | --- | --- | --- | --- | --- | --- | --- | --- | --- | --- | --- | --- | --- | --- | --- | --- | --- | --- | --- | --- | --- | --- | --- | --- | --- | --- | --- | --- | --- | --- | --- | --- | --- | --- | --- | --- | --- | --- | --- | --- | --- | --- | --- | --- | --- | --- | --- | --- | --- | --- | --- | --- | --- | --- | --- | --- | --- | --- | --- | --- | --- | --- | --- | --- | --- | --- | --- | --- | --- | --- | --- | --- | --- | --- | --- | --- | --- | --- | --- | --- | --- | --- | --- | --- | --- | --- | --- | --- | --- | --- | --- | --- | --- | --- | --- | --- | --- | --- | --- | --- | --- | --- | --- | --- | --- | --- | --- | --- | --- | --- | --- | --- | --- | --- | --- | --- | --- | --- | --- | --- | --- | --- | --- | --- | --- | --- | --- | --- | --- | --- | --- | --- | --- | --- | --- | --- | --- | --- | --- | --- | --- | --- | --- | --- | --- | --- | --- | --- | --- | --- | --- | --- | --- | --- | --- | --- | --- | --- | --- | --- | --- | --- | --- | --- | --- | --- | --- | --- | --- | --- | --- | --- | --- | --- | --- | --- | --- | --- | --- | --- | --- | --- | --- | --- | --- | --- | --- | --- | --- | --- | --- | --- | --- | --- | --- | --- |
| L | |  |  |  | | --- | --- | --- | | L2\_feature  (in PyDSTool.Toolbox.ParamEst) | load()  (in Unpickler) | log1p  (in PyDSTool.Toolbox.phaseplane) | | L2\_feature\_1D  (in PyDSTool.Toolbox.ParamEst) | load()  (in PyDSTool.fixedpickle) | log1p  (in PyDSTool.Toolbox.synthetic\_data) | | LargestInt32  (in PyDSTool.FuncSpec') | load\_append()  (in Unpickler) | log1p  (in PyDSTool.Toolbox.syntheticdata) | | LargestInt32  (in PyDSTool.Generator.ADMC\_ODEsystem') | load\_appends()  (in Unpickler) | log1p  (in PyDSTool) | | LargestInt32  (in PyDSTool.Generator.Dopri\_ODEsystem') | load\_binfloat()  (in Unpickler) | log1p  (in matplotlib.pylab) | | LargestInt32  (in PyDSTool.Generator.EmbeddedSysGen') | load\_binget()  (in Unpickler) | log\_distances()  (in PyDSTool.Toolbox.data\_analysis) | | LargestInt32  (in PyDSTool.Generator.Euler\_ODEsystem') | load\_binint()  (in Unpickler) | log\_distances()  (in PyDSTool.Toolbox.dataanalysis) | | LargestInt32  (in PyDSTool.Generator.ExplicitFnGen') | load\_binint1()  (in Unpickler) | log\_distances\_with\_D()  (in PyDSTool.Toolbox.data\_analysis) | | LargestInt32  (in PyDSTool.Generator.ExtrapolateTable') | load\_binint2()  (in Unpickler) | log\_distances\_with\_D()  (in PyDSTool.Toolbox.dataanalysis) | | LargestInt32  (in PyDSTool.Generator.ImplicitFnGen') | load\_binpersid()  (in Unpickler) | logaddexp  (in PyDSTool.PyCont.ContClass') | | LargestInt32  (in PyDSTool.Generator.InterpolateTable') | load\_binput()  (in Unpickler) | logaddexp  (in PyDSTool.Toolbox.ActivationFuncs) | | LargestInt32  (in PyDSTool.Generator.LookupTable') | load\_binstring()  (in Unpickler) | logaddexp  (in PyDSTool.Toolbox.DSSRT\_tools) | | LargestInt32  (in PyDSTool.Generator.MapSystem') | load\_binunicode()  (in Unpickler) | logaddexp  (in PyDSTool.Toolbox.InputProfile) | | LargestInt32  (in PyDSTool.Generator.ODEsystem') | load\_build()  (in Unpickler) | logaddexp  (in PyDSTool.Toolbox.ModelHelper) | | LargestInt32  (in PyDSTool.Generator.Radau\_ODEsystem') | load\_dict()  (in Unpickler) | logaddexp  (in PyDSTool.Toolbox.NineML) | | LargestInt32  (in PyDSTool.Generator.Vode\_ODEsystem') | load\_dup()  (in Unpickler) | logaddexp  (in PyDSTool.Toolbox.adjointPRC) | | LargestInt32  (in PyDSTool.Interval') | load\_empty\_dictionary()  (in Unpickler) | logaddexp  (in PyDSTool.Toolbox.dataanalysis) | | LargestInt32  (in PyDSTool.ModelConstructor') | load\_empty\_list()  (in Unpickler) | logaddexp  (in PyDSTool.Toolbox.fracdim) | | LargestInt32  (in PyDSTool.ModelSpec') | load\_empty\_tuple()  (in Unpickler) | logaddexp  (in PyDSTool.Toolbox.makeSloppyModel) | | LargestInt32  (in PyDSTool.Symbolic) | load\_eof()  (in Unpickler) | logaddexp  (in PyDSTool.Toolbox.neuralcomp) | | LargestInt32  (in PyDSTool.Toolbox.NineML) | load\_ext1()  (in Unpickler) | logaddexp  (in PyDSTool.Toolbox.phaseplane) | | LargestInt32  (in PyDSTool.Toolbox.dataanalysis) | load\_ext2()  (in Unpickler) | logaddexp  (in PyDSTool.Toolbox.synthetic\_data) | | LargestInt32  (in PyDSTool.Toolbox.event\_driven\_simulator) | load\_ext4()  (in Unpickler) | logaddexp  (in PyDSTool.Toolbox.syntheticdata) | | LargestInt32  (in PyDSTool.Toolbox.phaseplane) | load\_false()  (in Unpickler) | logaddexp  (in PyDSTool) | | LargestInt32  (in PyDSTool.Toolbox.synthetic\_data) | load\_float()  (in Unpickler) | logaddexp  (in matplotlib.pylab) | | LargestInt32  (in PyDSTool.Toolbox.syntheticdata) | load\_get()  (in Unpickler) | logaddexp2  (in PyDSTool.PyCont.ContClass') | | LargestInt32  (in PyDSTool.Trajectory') | load\_global()  (in Unpickler) | logaddexp2  (in PyDSTool.Toolbox.ActivationFuncs) | | LargestInt32  (in PyDSTool.Variable') | load\_inst()  (in Unpickler) | logaddexp2  (in PyDSTool.Toolbox.DSSRT\_tools) | | LargestInt32  (in PyDSTool.common) | load\_int()  (in Unpickler) | logaddexp2  (in PyDSTool.Toolbox.InputProfile) | | LargestInt32  (in PyDSTool.parseUtils) | load\_list()  (in Unpickler) | logaddexp2  (in PyDSTool.Toolbox.ModelHelper) | | LargestInt32  (in PyDSTool.utils) | load\_long()  (in Unpickler) | logaddexp2  (in PyDSTool.Toolbox.NineML) | | latex\_documents  (in PyDSTool.conf) | load\_long1()  (in Unpickler) | logaddexp2  (in PyDSTool.Toolbox.adjointPRC) | | Ldexp  (in PyDSTool) | load\_long4()  (in Unpickler) | logaddexp2  (in PyDSTool.Toolbox.dataanalysis) | | Ldexp  (in PyDSTool.ModelSpec') | load\_long\_binget()  (in Unpickler) | logaddexp2  (in PyDSTool.Toolbox.fracdim) | | ldexp  (in PyDSTool.PyCont.ContClass') | load\_long\_binput()  (in Unpickler) | logaddexp2  (in PyDSTool.Toolbox.makeSloppyModel) | | Ldexp  (in PyDSTool.Symbolic) | load\_mark()  (in Unpickler) | logaddexp2  (in PyDSTool.Toolbox.neuralcomp) | | ldexp  (in PyDSTool.Symbolic) | load\_newobj()  (in Unpickler) | logaddexp2  (in PyDSTool.Toolbox.phaseplane) | | Ldexp  (in PyDSTool.Toolbox.ActivationFuncs) | load\_none()  (in Unpickler) | logaddexp2  (in PyDSTool.Toolbox.synthetic\_data) | | Ldexp  (in PyDSTool.Toolbox.DSSRT\_tools) | load\_obj()  (in Unpickler) | logaddexp2  (in PyDSTool.Toolbox.syntheticdata) | | Ldexp  (in PyDSTool.Toolbox.InputProfile) | load\_persid()  (in Unpickler) | logaddexp2  (in PyDSTool) | | Ldexp  (in PyDSTool.Toolbox.ModelHelper) | load\_pop()  (in Unpickler) | logaddexp2  (in matplotlib.pylab) | | Ldexp  (in PyDSTool.Toolbox.NineML) | load\_pop\_mark()  (in Unpickler) | logical\_and  (in PyDSTool.PyCont.ContClass') | | ldexp  (in PyDSTool.Toolbox.NineML) | load\_proto()  (in Unpickler) | logical\_and  (in PyDSTool.Toolbox.ActivationFuncs) | | Ldexp  (in PyDSTool.Toolbox.adjointPRC) | load\_put()  (in Unpickler) | logical\_and  (in PyDSTool.Toolbox.DSSRT\_tools) | | Ldexp  (in PyDSTool.Toolbox.dataanalysis) | load\_reduce()  (in Unpickler) | logical\_and  (in PyDSTool.Toolbox.InputProfile) | | ldexp  (in PyDSTool.Toolbox.dataanalysis) | load\_setitem()  (in Unpickler) | logical\_and  (in PyDSTool.Toolbox.ModelHelper) | | Ldexp  (in PyDSTool.Toolbox.fracdim) | load\_setitems()  (in Unpickler) | logical\_and  (in PyDSTool.Toolbox.NineML) | | Ldexp  (in PyDSTool.Toolbox.makeSloppyModel) | load\_short\_binstring()  (in Unpickler) | logical\_and  (in PyDSTool.Toolbox.adjointPRC) | | Ldexp  (in PyDSTool.Toolbox.neuralcomp) | load\_stop()  (in Unpickler) | logical\_and  (in PyDSTool.Toolbox.dataanalysis) | | Ldexp  (in PyDSTool.Toolbox.phaseplane) | load\_string()  (in Unpickler) | logical\_and  (in PyDSTool.Toolbox.fracdim) | | ldexp  (in PyDSTool.Toolbox.phaseplane) | load\_true()  (in Unpickler) | logical\_and  (in PyDSTool.Toolbox.makeSloppyModel) | | Ldexp  (in PyDSTool.Toolbox.synthetic\_data) | load\_tuple()  (in Unpickler) | logical\_and  (in PyDSTool.Toolbox.neuralcomp) | | ldexp  (in PyDSTool.Toolbox.synthetic\_data) | load\_tuple1()  (in Unpickler) | logical\_and  (in PyDSTool.Toolbox.phaseplane) | | Ldexp  (in PyDSTool.Toolbox.syntheticdata) | load\_tuple2()  (in Unpickler) | logical\_and  (in PyDSTool.Toolbox.synthetic\_data) | | ldexp  (in PyDSTool.Toolbox.syntheticdata) | load\_tuple3()  (in Unpickler) | logical\_and  (in PyDSTool.Toolbox.syntheticdata) | | ldexp  (in matplotlib.pylab) | load\_unicode()  (in Unpickler) | logical\_and  (in PyDSTool) | | LeafComponent  (in PyDSTool.ModelSpec') | loadAutoMod()  (in ContClass) | logical\_and  (in matplotlib.pylab) | | leave\_actives  (in PyDSTool.Toolbox.dssrt) | loadObjects()  (in PyDSTool.utils) | logical\_not  (in PyDSTool.PyCont.ContClass') | | leave\_fast  (in PyDSTool.Toolbox.dssrt) | loads()  (in PyDSTool.fixedpickle) | logical\_not  (in PyDSTool.Toolbox.ActivationFuncs) | | leave\_slow  (in PyDSTool.Toolbox.dssrt) | loadSession()  (in PyDSTool) | logical\_not  (in PyDSTool.Toolbox.DSSRT\_tools) | | left\_shift  (in PyDSTool.PyCont.ContClass') | local\_brute\_force\_1dstep  (in PyDSTool.Toolbox.optimizers.step) | logical\_not  (in PyDSTool.Toolbox.InputProfile) | | left\_shift  (in PyDSTool.Toolbox.ActivationFuncs) | local\_fndef  (in PyDSTool.Symbolic) | logical\_not  (in PyDSTool.Toolbox.ModelHelper) | | left\_shift  (in PyDSTool.Toolbox.DSSRT\_tools) | LocalBruteForce1DStep  (in PyDSTool.Toolbox.optimizers.step.local\_brute\_force\_1dstep) | logical\_not  (in PyDSTool.Toolbox.NineML) | | left\_shift  (in PyDSTool.Toolbox.InputProfile) | locate()  (in BPoint) | logical\_not  (in PyDSTool.Toolbox.adjointPRC) | | left\_shift  (in PyDSTool.Toolbox.ModelHelper) | locate()  (in BifPoint) | logical\_not  (in PyDSTool.Toolbox.dataanalysis) | | left\_shift  (in PyDSTool.Toolbox.NineML) | locate()  (in BranchPoint) | logical\_not  (in PyDSTool.Toolbox.fracdim) | | left\_shift  (in PyDSTool.Toolbox.adjointPRC) | Log  (in PyDSTool) | logical\_not  (in PyDSTool.Toolbox.makeSloppyModel) | | left\_shift  (in PyDSTool.Toolbox.dataanalysis) | Log  (in PyDSTool.ModelSpec') | logical\_not  (in PyDSTool.Toolbox.neuralcomp) | | left\_shift  (in PyDSTool.Toolbox.fracdim) | log  (in PyDSTool.PyCont.ContClass') | logical\_not  (in PyDSTool.Toolbox.phaseplane) | | left\_shift  (in PyDSTool.Toolbox.makeSloppyModel) | Log  (in PyDSTool.Symbolic) | logical\_not  (in PyDSTool.Toolbox.synthetic\_data) | | left\_shift  (in PyDSTool.Toolbox.neuralcomp) | log  (in PyDSTool.Symbolic) | logical\_not  (in PyDSTool.Toolbox.syntheticdata) | | left\_shift  (in PyDSTool.Toolbox.phaseplane) | Log  (in PyDSTool.Toolbox.ActivationFuncs) | logical\_not  (in PyDSTool) | | left\_shift  (in PyDSTool.Toolbox.synthetic\_data) | Log  (in PyDSTool.Toolbox.DSSRT\_tools) | logical\_not  (in matplotlib.pylab) | | left\_shift  (in PyDSTool.Toolbox.syntheticdata) | Log  (in PyDSTool.Toolbox.InputProfile) | logical\_or  (in PyDSTool.PyCont.ContClass') | | left\_shift  (in PyDSTool) | Log  (in PyDSTool.Toolbox.ModelHelper) | logical\_or  (in PyDSTool.Toolbox.NineML) | | left\_shift  (in matplotlib.pylab) | Log  (in PyDSTool.Toolbox.NineML) | logical\_or  (in PyDSTool.Toolbox.dataanalysis) | | less  (in PyDSTool.PyCont.ContClass') | log  (in PyDSTool.Toolbox.NineML) | logical\_or  (in PyDSTool.Toolbox.phaseplane) | | less  (in PyDSTool.Toolbox.NineML) | Log  (in PyDSTool.Toolbox.adjointPRC) | logical\_or  (in PyDSTool.Toolbox.synthetic\_data) | | less  (in PyDSTool.Toolbox.dataanalysis) | Log  (in PyDSTool.Toolbox.dataanalysis) | logical\_or  (in PyDSTool.Toolbox.syntheticdata) | | less  (in PyDSTool.Toolbox.phaseplane) | log  (in PyDSTool.Toolbox.dataanalysis) | logical\_or  (in PyDSTool.common) | | less  (in PyDSTool.Toolbox.synthetic\_data) | Log  (in PyDSTool.Toolbox.fracdim) | logical\_or  (in matplotlib.pylab) | | less  (in PyDSTool.Toolbox.syntheticdata) | Log  (in PyDSTool.Toolbox.makeSloppyModel) | logical\_xor  (in PyDSTool.PyCont.ContClass') | | less  (in PyDSTool.common) | Log  (in PyDSTool.Toolbox.neuralcomp) | logical\_xor  (in PyDSTool.Toolbox.ActivationFuncs) | | less  (in PyDSTool.utils) | Log  (in PyDSTool.Toolbox.phaseplane) | logical\_xor  (in PyDSTool.Toolbox.DSSRT\_tools) | | less  (in matplotlib.pylab) | log  (in PyDSTool.Toolbox.phaseplane) | logical\_xor  (in PyDSTool.Toolbox.InputProfile) | | less\_equal  (in PyDSTool.Generator.Euler\_ODEsystem') | Log  (in PyDSTool.Toolbox.synthetic\_data) | logical\_xor  (in PyDSTool.Toolbox.ModelHelper) | | less\_equal  (in PyDSTool.Generator.Vode\_ODEsystem') | log  (in PyDSTool.Toolbox.synthetic\_data) | logical\_xor  (in PyDSTool.Toolbox.NineML) | | less\_equal  (in PyDSTool.PyCont.ContClass') | Log  (in PyDSTool.Toolbox.syntheticdata) | logical\_xor  (in PyDSTool.Toolbox.adjointPRC) | | less\_equal  (in PyDSTool.Toolbox.NineML) | log  (in PyDSTool.Toolbox.syntheticdata) | logical\_xor  (in PyDSTool.Toolbox.dataanalysis) | | less\_equal  (in PyDSTool.Toolbox.dataanalysis) | log  (in PyDSTool.common) | logical\_xor  (in PyDSTool.Toolbox.fracdim) | | less\_equal  (in PyDSTool.Toolbox.phaseplane) | log  (in matplotlib.pylab) | logical\_xor  (in PyDSTool.Toolbox.makeSloppyModel) | | less\_equal  (in PyDSTool.Toolbox.synthetic\_data) | Log10  (in PyDSTool) | logical\_xor  (in PyDSTool.Toolbox.neuralcomp) | | less\_equal  (in PyDSTool.Toolbox.syntheticdata) | Log10  (in PyDSTool.ModelSpec') | logical\_xor  (in PyDSTool.Toolbox.phaseplane) | | less\_equal  (in PyDSTool.common) | log10  (in PyDSTool.PyCont.ContClass') | logical\_xor  (in PyDSTool.Toolbox.synthetic\_data) | | less\_equal  (in matplotlib.pylab) | log10  (in PyDSTool.PyCont.Continuation) | logical\_xor  (in PyDSTool.Toolbox.syntheticdata) | | level  (in Verbose) | Log10  (in PyDSTool.Symbolic) | logical\_xor  (in PyDSTool) | | levels  (in Verbose) | log10  (in PyDSTool.Symbolic) | logical\_xor  (in matplotlib.pylab) | | levenberg\_marquardt  (in PyDSTool.Toolbox.optimizers.helpers) | Log10  (in PyDSTool.Toolbox.ActivationFuncs) | logistic\_spec  (in PyDSTool.Toolbox.model\_primitives) | | lfilter\_zi()  (in PyDSTool.Toolbox.data\_analysis) | Log10  (in PyDSTool.Toolbox.DSSRT\_tools) | Lognormvariate  (in PyDSTool) | | limitcycle\_args\_list  (in PyDSTool.PyCont.Continuation) | Log10  (in PyDSTool.Toolbox.InputProfile) | Lognormvariate  (in PyDSTool.ModelSpec') | | limitcycle\_bif\_points  (in PyDSTool.PyCont.Continuation) | Log10  (in PyDSTool.Toolbox.ModelHelper) | Lognormvariate  (in PyDSTool.Symbolic) | | LimitCycleCurve  (in PyDSTool.PyCont.Continuation) | Log10  (in PyDSTool.Toolbox.NineML) | Lognormvariate  (in PyDSTool.Toolbox.ActivationFuncs) | | line\_intersection()  (in PyDSTool.Toolbox.phaseplane) | log10  (in PyDSTool.Toolbox.NineML) | Lognormvariate  (in PyDSTool.Toolbox.DSSRT\_tools) | | line\_search  (in PyDSTool.Toolbox.optimizers) | Log10  (in PyDSTool.Toolbox.adjointPRC) | Lognormvariate  (in PyDSTool.Toolbox.InputProfile) | | line\_search\_\_all\_\_  (in PyDSTool.Toolbox.optimizers.line\_search) | Log10  (in PyDSTool.Toolbox.dataanalysis) | Lognormvariate  (in PyDSTool.Toolbox.ModelHelper) | | linear\_decay  (in PyDSTool.Toolbox.model\_primitives) | log10  (in PyDSTool.Toolbox.dataanalysis) | Lognormvariate  (in PyDSTool.Toolbox.NineML) | | linearInterp()  (in PyDSTool.common) | Log10  (in PyDSTool.Toolbox.fracdim) | Lognormvariate  (in PyDSTool.Toolbox.adjointPRC) | | LIST  (in PyDSTool.fixedpickle) | Log10  (in PyDSTool.Toolbox.makeSloppyModel) | Lognormvariate  (in PyDSTool.Toolbox.dataanalysis) | | listid()  (in PyDSTool.common) | Log10  (in PyDSTool.Toolbox.neuralcomp) | Lognormvariate  (in PyDSTool.Toolbox.fracdim) | | little\_endian  (in PyDSTool.PyCont.ContClass') | Log10  (in PyDSTool.Toolbox.phaseplane) | Lognormvariate  (in PyDSTool.Toolbox.makeSloppyModel) | | little\_endian  (in PyDSTool.Toolbox.ActivationFuncs) | log10  (in PyDSTool.Toolbox.phaseplane) | Lognormvariate  (in PyDSTool.Toolbox.neuralcomp) | | little\_endian  (in PyDSTool.Toolbox.DSSRT\_tools) | Log10  (in PyDSTool.Toolbox.synthetic\_data) | Lognormvariate  (in PyDSTool.Toolbox.phaseplane) | | little\_endian  (in PyDSTool.Toolbox.InputProfile) | log10  (in PyDSTool.Toolbox.synthetic\_data) | Lognormvariate  (in PyDSTool.Toolbox.synthetic\_data) | | little\_endian  (in PyDSTool.Toolbox.ModelHelper) | Log10  (in PyDSTool.Toolbox.syntheticdata) | Lognormvariate  (in PyDSTool.Toolbox.syntheticdata) | | little\_endian  (in PyDSTool.Toolbox.NineML) | log10  (in PyDSTool.Toolbox.syntheticdata) | LONG  (in PyDSTool.fixedpickle) | | little\_endian  (in PyDSTool.Toolbox.adjointPRC) | log10  (in matplotlib.pylab) | LONG1  (in PyDSTool.fixedpickle) | | little\_endian  (in PyDSTool.Toolbox.dataanalysis) | log1p  (in PyDSTool.PyCont.ContClass') | LONG4  (in PyDSTool.fixedpickle) | | little\_endian  (in PyDSTool.Toolbox.fracdim) | log1p  (in PyDSTool.Toolbox.ActivationFuncs) | LONG\_BINGET  (in PyDSTool.fixedpickle) | | little\_endian  (in PyDSTool.Toolbox.makeSloppyModel) | log1p  (in PyDSTool.Toolbox.DSSRT\_tools) | LONG\_BINPUT  (in PyDSTool.fixedpickle) | | little\_endian  (in PyDSTool.Toolbox.neuralcomp) | log1p  (in PyDSTool.Toolbox.InputProfile) | lookup\_index()  (in domscales) | | little\_endian  (in PyDSTool.Toolbox.phaseplane) | log1p  (in PyDSTool.Toolbox.ModelHelper) | lookup\_time()  (in domscales) | | little\_endian  (in PyDSTool.Toolbox.synthetic\_data) | log1p  (in PyDSTool.Toolbox.NineML) | LookupTable  (in PyDSTool.Generator.LookupTable') | | little\_endian  (in PyDSTool.Toolbox.syntheticdata) | log1p  (in PyDSTool.Toolbox.adjointPRC) | LookupTable'  (in PyDSTool.Generator) | | little\_endian  (in PyDSTool) | log1p  (in PyDSTool.Toolbox.dataanalysis) | LowLevelEvent  (in PyDSTool.Events) | | little\_endian  (in matplotlib.pylab) | log1p  (in PyDSTool.Toolbox.fracdim) | LPC\_Det  (in PyDSTool.PyCont.TestFunc) | | LMpest  (in PyDSTool.Toolbox.ParamEst) | log1p  (in PyDSTool.Toolbox.makeSloppyModel) | LPCPoint  (in PyDSTool.PyCont.BifPoint) | | LMQuadratic  (in PyDSTool.Toolbox.optimizers.helpers.levenberg\_marquardt) | log1p  (in PyDSTool.Toolbox.neuralcomp) |  | |

  
  

| Home | Trees | Indices | Help | | PyDSTool | | --- | |
| --- | --- | --- | --- | --- | --- |

|  |  |
| --- | --- |
| Generated by Epydoc 3.0.1 on Fri May 4 15:23:58 2012 | http://epydoc.sourceforge.net |
