# Supplementary material for: Hybrid Models and Biological Model Reduction with PyDSTool
Source: PLoS Comput Biol. 2012 Aug 9;8(8):e1002628. doi: 10.1371/journal.pcbi.1002628 (PMC3415397; doi:10.1371/journal.pcbi.1002628)
Supplement: Text S4 — Complete source code for the PyDSTool package (version 0.88.120504). Includes API documentation and help files linking to web pages. This file is identical to the current public release on Sourceforge.net. (ZIP) [file pcbi.1002628.s004.zip › PyDSTool/html/identifier-index-M.html]

xml version="1.0" encoding="ascii"?


Identifier Index


| Home | Trees | Indices | Help | | PyDSTool | | --- | |
| --- | --- | --- | --- | --- | --- |

|  |  |  |  |
| --- | --- | --- | --- |
|  | |  | | --- | | [hide private] | | [frames] | no frames] | |

|  |  |
| --- | --- |
| Identifier Index | [ A B C D E F G H I J K L M N O P Q R S T U V W X Y Z \_ ] |

|  |  |  |  |  |  |  |  |  |  |  |  |  |  |  |  |  |  |  |  |  |  |  |  |  |  |  |  |  |  |  |  |  |  |  |  |  |  |  |  |  |  |  |  |  |  |  |  |  |  |  |  |  |  |  |  |  |  |  |  |  |  |  |  |  |  |  |  |  |  |  |  |  |  |  |  |  |  |  |  |  |  |  |  |  |  |  |  |  |  |  |  |  |  |  |  |  |  |  |  |  |  |  |  |  |  |  |  |  |  |  |  |  |  |  |  |  |  |  |  |  |  |  |  |  |  |  |  |  |  |  |  |  |  |  |  |  |  |  |  |  |  |  |  |  |  |  |  |  |  |  |  |  |  |  |  |  |  |  |  |  |  |  |  |  |  |  |  |  |  |  |  |  |  |  |  |  |  |  |  |  |  |  |  |  |  |  |  |  |  |  |  |  |  |  |  |  |  |  |  |  |  |  |  |  |  |  |  |  |  |  |  |  |  |  |  |  |  |  |  |  |  |  |  |  |  |  |  |  |  |  |  |  |  |  |  |  |  |  |  |  |  |  |  |  |  |  |  |  |  |  |  |  |  |  |  |  |  |  |  |  |  |  |  |  |  |  |  |  |  |  |  |  |  |  |  |  |  |  |  |  |  |  |  |  |  |  |  |  |  |  |  |  |  |  |  |  |  |  |  |  |  |  |  |  |  |  |  |  |  |  |  |  |  |  |  |  |  |  |  |
| --- | --- | --- | --- | --- | --- | --- | --- | --- | --- | --- | --- | --- | --- | --- | --- | --- | --- | --- | --- | --- | --- | --- | --- | --- | --- | --- | --- | --- | --- | --- | --- | --- | --- | --- | --- | --- | --- | --- | --- | --- | --- | --- | --- | --- | --- | --- | --- | --- | --- | --- | --- | --- | --- | --- | --- | --- | --- | --- | --- | --- | --- | --- | --- | --- | --- | --- | --- | --- | --- | --- | --- | --- | --- | --- | --- | --- | --- | --- | --- | --- | --- | --- | --- | --- | --- | --- | --- | --- | --- | --- | --- | --- | --- | --- | --- | --- | --- | --- | --- | --- | --- | --- | --- | --- | --- | --- | --- | --- | --- | --- | --- | --- | --- | --- | --- | --- | --- | --- | --- | --- | --- | --- | --- | --- | --- | --- | --- | --- | --- | --- | --- | --- | --- | --- | --- | --- | --- | --- | --- | --- | --- | --- | --- | --- | --- | --- | --- | --- | --- | --- | --- | --- | --- | --- | --- | --- | --- | --- | --- | --- | --- | --- | --- | --- | --- | --- | --- | --- | --- | --- | --- | --- | --- | --- | --- | --- | --- | --- | --- | --- | --- | --- | --- | --- | --- | --- | --- | --- | --- | --- | --- | --- | --- | --- | --- | --- | --- | --- | --- | --- | --- | --- | --- | --- | --- | --- | --- | --- | --- | --- | --- | --- | --- | --- | --- | --- | --- | --- | --- | --- | --- | --- | --- | --- | --- | --- | --- | --- | --- | --- | --- | --- | --- | --- | --- | --- | --- | --- | --- | --- | --- | --- | --- | --- | --- | --- | --- | --- | --- | --- | --- | --- | --- | --- | --- | --- | --- | --- | --- | --- | --- | --- | --- | --- | --- | --- | --- | --- | --- | --- | --- | --- | --- | --- | --- | --- | --- | --- | --- | --- | --- | --- | --- | --- | --- | --- | --- | --- | --- | --- | --- | --- | --- | --- | --- | --- | --- | --- | --- | --- | --- | --- | --- | --- | --- | --- | --- | --- | --- | --- | --- | --- | --- | --- | --- | --- | --- | --- | --- |
| M | |  |  |  | | --- | --- | --- | | m  (in PyDSTool.Symbolic) | mathNameMap  (in PyDSTool.Toolbox.synthetic\_data) | Min  (in PyDSTool.Toolbox.synthetic\_data) | | Macheps  (in PyDSTool.common) | mathNameMap  (in PyDSTool.Toolbox.syntheticdata) | Min  (in PyDSTool.Toolbox.syntheticdata) | | make\_disp()  (in PyDSTool.Toolbox.mechmatlib) | MatlabEvent  (in PyDSTool.Events) | min\_curvature\_zone  (in PyDSTool.Toolbox.phaseplane) | | make\_distance\_to\_known\_line\_auxfn()  (in PyDSTool.Toolbox.phaseplane) | matplotlib\_import  (in PyDSTool) | MIN\_EXP  (in PyDSTool.Interval') | | make\_distance\_to\_line\_auxfn()  (in PyDSTool.Toolbox.phaseplane) | Max  (in PyDSTool) | min\_gradient\_dir()  (in mesh\_patch\_2D) | | make\_flow\_normal\_event()  (in PyDSTool.Toolbox.phaseplane) | Max  (in PyDSTool.ModelSpec') | min\_gradient\_mesh()  (in mesh\_patch\_2D) | | make\_Jac\_wrap()  (in PyDSTool.utils) | Max  (in PyDSTool.Symbolic) | minimum  (in PyDSTool.PyCont.ContClass') | | make\_mesh()  (in mesh\_patch\_2D) | Max  (in PyDSTool.Toolbox.ActivationFuncs) | minimum  (in PyDSTool.Toolbox.ActivationFuncs) | | make\_opt()  (in PyDSTool.Toolbox.ParamEst) | Max  (in PyDSTool.Toolbox.DSSRT\_tools) | minimum  (in PyDSTool.Toolbox.DSSRT\_tools) | | make\_pointsets()  (in dssrt\_assistant) | Max  (in PyDSTool.Toolbox.InputProfile) | minimum  (in PyDSTool.Toolbox.InputProfile) | | make\_poly\_interpolated\_curve()  (in PyDSTool.common) | Max  (in PyDSTool.Toolbox.ModelHelper) | minimum  (in PyDSTool.Toolbox.ModelHelper) | | make\_RHS\_wrap()  (in PyDSTool.utils) | Max  (in PyDSTool.Toolbox.NineML) | minimum  (in PyDSTool.Toolbox.NineML) | | make\_rot()  (in PyDSTool.Toolbox.mechmatlib) | Max  (in PyDSTool.Toolbox.adjointPRC) | minimum  (in PyDSTool.Toolbox.adjointPRC) | | make\_secant\_fig\_dict()  (in PyDSTool.Toolbox.fracdim) | Max  (in PyDSTool.Toolbox.dataanalysis) | minimum  (in PyDSTool.Toolbox.dataanalysis) | | make\_T()  (in PyDSTool.Toolbox.mechmatlib) | Max  (in PyDSTool.Toolbox.fracdim) | minimum  (in PyDSTool.Toolbox.fracdim) | | make\_varspecs()  (in PyDSTool.Toolbox.PySCes\_SBML) | Max  (in PyDSTool.Toolbox.makeSloppyModel) | minimum  (in PyDSTool.Toolbox.makeSloppyModel) | | make\_vec\_at\_A\_face\_B()  (in PyDSTool.Toolbox.phaseplane) | Max  (in PyDSTool.Toolbox.neuralcomp) | minimum  (in PyDSTool.Toolbox.neuralcomp) | | makeAdaptingSynapse()  (in PyDSTool.Toolbox.neuralcomp) | Max  (in PyDSTool.Toolbox.phaseplane) | minimum  (in PyDSTool.Toolbox.phaseplane) | | makeArrayIxMap()  (in PyDSTool.common) | Max  (in PyDSTool.Toolbox.synthetic\_data) | minimum  (in PyDSTool.Toolbox.synthetic\_data) | | makeAutoLib()  (in ContClass) | Max  (in PyDSTool.Toolbox.syntheticdata) | minimum  (in PyDSTool.Toolbox.syntheticdata) | | makeAutoLibSource()  (in ContClass) | max\_curvature\_zone\_leaf  (in PyDSTool.Toolbox.phaseplane) | minimum  (in PyDSTool) | | makeBiasChannel()  (in PyDSTool.Toolbox.neuralcomp) | max\_curvature\_zone\_node  (in PyDSTool.Toolbox.phaseplane) | minimum  (in matplotlib.pylab) | | makeCallFn()  (in EvMapping) | max\_euclidean\_distance()  (in PyDSTool.Toolbox.synthetic\_data) | mins()  (in PyDSTool.Toolbox.synthetic\_data) | | makeChannel\_halfact()  (in PyDSTool.Toolbox.neuralcomp) | max\_euclidean\_distance()  (in PyDSTool.Toolbox.syntheticdata) | mins()  (in PyDSTool.Toolbox.syntheticdata) | | makeChannel\_rates()  (in PyDSTool.Toolbox.neuralcomp) | max\_gradient\_dir()  (in mesh\_patch\_2D) | MINUTELY  (in PyDSTool.PyCont.ContClass') | | makeDataDict()  (in PyDSTool.utils) | max\_gradient\_mesh()  (in mesh\_patch\_2D) | MINUTELY  (in matplotlib.pylab) | | makeDefaultGraphics()  (in DSSRT\_info) | MAXDIMS  (in PyDSTool) | misc  (in PyDSTool.PyCont) | | makeDendrite()  (in PyDSTool.Toolbox.neuralcomp) | MAXDIMS  (in PyDSTool.PyCont.ContClass') | MISMATCH  (in PyDSTool.Toolbox.dssrt) | | makeDSSRTcfg()  (in DSSRT\_info) | MAXDIMS  (in PyDSTool.Toolbox.ActivationFuncs) | MO  (in PyDSTool.PyCont.ContClass') | | makeEvMapping()  (in PyDSTool.ModelConstructor') | MAXDIMS  (in PyDSTool.Toolbox.DSSRT\_tools) | MO  (in matplotlib.pylab) | | makeExtInputConductanceChannel()  (in PyDSTool.Toolbox.neuralcomp) | MAXDIMS  (in PyDSTool.Toolbox.InputProfile) | mod  (in PyDSTool.ModelSpec') | | makeExtInputCurrentChannel()  (in PyDSTool.Toolbox.neuralcomp) | MAXDIMS  (in PyDSTool.Toolbox.ModelHelper) | mod  (in PyDSTool.PyCont.ContClass') | | makeFun()  (in PyDSTool.Toolbox.neuralcomp) | MAXDIMS  (in PyDSTool.Toolbox.NineML) | mod  (in PyDSTool.PyCont.Continuation) | | makeImplicitFunc()  (in PyDSTool.utils) | MAXDIMS  (in PyDSTool.Toolbox.adjointPRC) | mod  (in PyDSTool.PyCont.misc) | | makeInputTable()  (in PyDSTool.Toolbox.InputProfile) | MAXDIMS  (in PyDSTool.Toolbox.dataanalysis) | mod  (in PyDSTool.Symbolic) | | makeIxMaps()  (in Point) | MAXDIMS  (in PyDSTool.Toolbox.fracdim) | mod  (in PyDSTool.Toolbox.NineML) | | makeIxMaps()  (in Pointset) | MAXDIMS  (in PyDSTool.Toolbox.makeSloppyModel) | mod  (in PyDSTool.Toolbox.dataanalysis) | | makeLib()  (in ADMC\_ODEsystem) | MAXDIMS  (in PyDSTool.Toolbox.neuralcomp) | mod  (in PyDSTool.Toolbox.phaseplane) | | makeLib()  (in Dopri\_ODEsystem) | MAXDIMS  (in PyDSTool.Toolbox.phaseplane) | mod  (in PyDSTool.Toolbox.synthetic\_data) | | makeLib()  (in Radau\_ODEsystem) | MAXDIMS  (in PyDSTool.Toolbox.synthetic\_data) | mod  (in PyDSTool.Toolbox.syntheticdata) | | makeLibSource()  (in ADMC\_ODEsystem) | MAXDIMS  (in PyDSTool.Toolbox.syntheticdata) | mod  (in matplotlib.pylab) | | makeLibSource()  (in Dopri\_ODEsystem) | MAXDIMS  (in matplotlib.pylab) | mod\_path()  (in PyDSTool.Toolbox.optimizers) | | makeLibSource()  (in Radau\_ODEsystem) | maximum  (in PyDSTool.PyCont.ContClass') | Model  (in PyDSTool) | | makeMfileFunction()  (in PyDSTool.utils) | maximum  (in PyDSTool.Toolbox.ActivationFuncs) | Model  (in PyDSTool.Model) | | makeModelInfo()  (in PyDSTool.ModelConstructor') | maximum  (in PyDSTool.Toolbox.DSSRT\_tools) | model\_primitives  (in PyDSTool.Toolbox) | | makeModelInfoEntry()  (in PyDSTool.ModelConstructor') | maximum  (in PyDSTool.Toolbox.InputProfile) | ModelConstructor  (in PyDSTool.ModelConstructor') | | makeMultilinearRegrFn()  (in PyDSTool.common) | maximum  (in PyDSTool.Toolbox.ModelHelper) | ModelConstructor'  (in PyDSTool) | | makeNeurite()  (in PyDSTool.Toolbox.neuralcomp) | maximum  (in PyDSTool.Toolbox.NineML) | ModelEst  (in PyDSTool.Toolbox) | | makeNeuronNetwork()  (in PyDSTool.Toolbox.neuralcomp) | maximum  (in PyDSTool.Toolbox.adjointPRC) | ModelEst  (in PyDSTool.Toolbox.ModelEst) | | makeNonParameterized()  (in PyDSTool.Points) | maximum  (in PyDSTool.Toolbox.dataanalysis) | ModelHelper  (in PyDSTool.Toolbox) | | makePar()  (in PyDSTool.Toolbox.neuralcomp) | maximum  (in PyDSTool.Toolbox.fracdim) | ModelInterface  (in PyDSTool.MProject) | | makeParList()  (in PyDSTool.parseUtils) | maximum  (in PyDSTool.Toolbox.makeSloppyModel) | ModelLibrary  (in PyDSTool.MProject) | | makePartialJac()  (in PyDSTool.FuncSpec') | maximum  (in PyDSTool.Toolbox.neuralcomp) | ModelManager  (in PyDSTool.MProject) | | makePointNeuron()  (in PyDSTool.Toolbox.neuralcomp) | maximum  (in PyDSTool.Toolbox.phaseplane) | ModelSpec  (in PyDSTool.ModelSpec') | | makePointNeuronNetwork()  (in PyDSTool.Toolbox.neuralcomp) | maximum  (in PyDSTool.Toolbox.synthetic\_data) | ModelSpec'  (in PyDSTool) | | makePowerSpec()  (in PyDSTool.Toolbox.neuralcomp) | maximum  (in PyDSTool.Toolbox.syntheticdata) | ModelTransform  (in PyDSTool.MProject) | | makePythonStateZeroCrossEvent()  (in PyDSTool.Events) | maximum  (in PyDSTool) | Modf  (in PyDSTool.ModelSpec') | | makeSeqUnique()  (in PyDSTool.common) | maximum  (in matplotlib.pylab) | Modf  (in PyDSTool) | | makeSloppyModel  (in PyDSTool.Toolbox) | maxs()  (in PyDSTool.Toolbox.synthetic\_data) | modf  (in PyDSTool.PyCont.ContClass') | | makeSloppyModel()  (in PyDSTool.Toolbox.makeSloppyModel) | maxs()  (in PyDSTool.Toolbox.syntheticdata) | Modf  (in PyDSTool.Symbolic) | | makeSoma()  (in PyDSTool.Toolbox.neuralcomp) | MDescriptor  (in PyDSTool.ModelConstructor') | modf  (in PyDSTool.Symbolic) | | makeSpiffyODEModel()  (in PyDSTool.Toolbox.ModelHelper) | mean()  (in data\_bins) | Modf  (in PyDSTool.Toolbox.ActivationFuncs) | | makeSpikeProfile()  (in PyDSTool.Toolbox.InputProfile) | mean()  (in data\_bins) | Modf  (in PyDSTool.Toolbox.DSSRT\_tools) | | makeStaticVar()  (in GenTransform) | mechmatlib  (in PyDSTool.Toolbox) | Modf  (in PyDSTool.Toolbox.InputProfile) | | makeSynapse()  (in PyDSTool.Toolbox.neuralcomp) | memoize()  (in Pickler) | Modf  (in PyDSTool.Toolbox.ModelHelper) | | makeSynapseChannel()  (in PyDSTool.Toolbox.neuralcomp) | mergePointsets()  (in PyDSTool.Points) | Modf  (in PyDSTool.Toolbox.NineML) | | makeUniqueFn()  (in PyDSTool.common) | mesh\_patch\_2D  (in PyDSTool.Toolbox.phaseplane) | modf  (in PyDSTool.Toolbox.NineML) | | makeZeroCrossEvent()  (in PyDSTool.Events) | messagecodes  (in PyDSTool.Generator) | Modf  (in PyDSTool.Toolbox.adjointPRC) | | man\_pages  (in PyDSTool.conf) | messages  (in vode) | Modf  (in PyDSTool.Toolbox.dataanalysis) | | map  (in PyDSTool.Toolbox.event\_driven\_simulator) | metric  (in PyDSTool.common) | modf  (in PyDSTool.Toolbox.dataanalysis) | | map1D  (in PyDSTool.Toolbox.event\_driven\_simulator) | metric\_float  (in PyDSTool.common) | Modf  (in PyDSTool.Toolbox.fracdim) | | map2D  (in PyDSTool.Toolbox.event\_driven\_simulator) | metric\_float\_1D  (in PyDSTool.common) | Modf  (in PyDSTool.Toolbox.makeSloppyModel) | | mapEvent()  (in ModelConstructor) | metric\_L2  (in PyDSTool.common) | Modf  (in PyDSTool.Toolbox.neuralcomp) | | mapIndices()  (in PointInfo) | metric\_L2\_1D  (in PyDSTool.common) | Modf  (in PyDSTool.Toolbox.phaseplane) | | mapNames()  (in Point) | metric\_weighted\_deadzone\_L2  (in PyDSTool.common) | modf  (in PyDSTool.Toolbox.phaseplane) | | mapNames()  (in PointInfo) | metric\_weighted\_L2  (in PyDSTool.common) | Modf  (in PyDSTool.Toolbox.synthetic\_data) | | mapNames()  (in QuantSpec) | mgrid  (in PyDSTool.PyCont.ContClass') | modf  (in PyDSTool.Toolbox.synthetic\_data) | | mapNames()  (in Quantity) | mgrid  (in PyDSTool.Toolbox.ActivationFuncs) | Modf  (in PyDSTool.Toolbox.syntheticdata) | | mapNames()  (in Point2D) | mgrid  (in PyDSTool.Toolbox.DSSRT\_tools) | modf  (in PyDSTool.Toolbox.syntheticdata) | | mapNames()  (in Trajectory) | mgrid  (in PyDSTool.Toolbox.InputProfile) | modf  (in matplotlib.pylab) | | mapNames()  (in PyDSTool.parseUtils) | mgrid  (in PyDSTool.Toolbox.ModelHelper) | ModifiedAICCriterion  (in PyDSTool.Toolbox.optimizers.criterion.information\_criteria) | | mapPowStr()  (in PyDSTool.parseUtils) | mgrid  (in PyDSTool.Toolbox.NineML) | modlookup  (in PyDSTool.Symbolic) | | MapSystem  (in PyDSTool.Generator.MapSystem') | mgrid  (in PyDSTool.Toolbox.adjointPRC) | monotone()  (in PyDSTool.PyCont.misc) | | MapSystem'  (in PyDSTool.Generator) | mgrid  (in PyDSTool.Toolbox.dataanalysis) | MonotonyCriterion  (in PyDSTool.Toolbox.optimizers.criterion.criteria) | | MARK  (in PyDSTool.fixedpickle) | mgrid  (in PyDSTool.Toolbox.fracdim) | MONTHLY  (in PyDSTool.PyCont.ContClass') | | marker()  (in Unpickler) | mgrid  (in PyDSTool.Toolbox.makeSloppyModel) | MONTHLY  (in matplotlib.pylab) | | marquardt\_step  (in PyDSTool.Toolbox.optimizers.step) | mgrid  (in PyDSTool.Toolbox.neuralcomp) | MProject  (in PyDSTool) | | MarquardtStep  (in PyDSTool.Toolbox.optimizers.step.marquardt\_step) | mgrid  (in PyDSTool.Toolbox.phaseplane) | MReg  (in PyDSTool.MProject) | | MassMatrix()  (in Radau\_ODEsystem) | mgrid  (in PyDSTool.Toolbox.synthetic\_data) | multiply  (in PyDSTool.PyCont.ContClass') | | MassMatrix()  (in GeneratorInterface) | mgrid  (in PyDSTool.Toolbox.syntheticdata) | multiply  (in PyDSTool.PyCont.misc) | | MassMatrix()  (in ModelInterface) | mgrid  (in PyDSTool) | multiply  (in PyDSTool.Toolbox.ActivationFuncs) | | MassMatrix()  (in HybridModel) | mgrid  (in matplotlib.pylab) | multiply  (in PyDSTool.Toolbox.DSSRT\_tools) | | MassMatrix()  (in NonHybridModel) | Min  (in PyDSTool) | multiply  (in PyDSTool.Toolbox.InputProfile) | | MassMatrix()  (in integrator) | Min  (in PyDSTool.ModelSpec') | multiply  (in PyDSTool.Toolbox.ModelHelper) | | master\_doc  (in PyDSTool.conf) | Min  (in PyDSTool.Symbolic) | multiply  (in PyDSTool.Toolbox.NineML) | | MATCH  (in PyDSTool.Toolbox.dssrt) | Min  (in PyDSTool.Toolbox.ActivationFuncs) | multiply  (in PyDSTool.Toolbox.adjointPRC) | | matchSubName()  (in PyDSTool.ModelSpec') | Min  (in PyDSTool.Toolbox.DSSRT\_tools) | multiply  (in PyDSTool.Toolbox.dataanalysis) | | math\_dir  (in PyDSTool.Symbolic) | Min  (in PyDSTool.Toolbox.InputProfile) | multiply  (in PyDSTool.Toolbox.fracdim) | | math\_globals  (in PyDSTool.Symbolic) | Min  (in PyDSTool.Toolbox.ModelHelper) | multiply  (in PyDSTool.Toolbox.makeSloppyModel) | | mathlookup  (in PyDSTool.Symbolic) | Min  (in PyDSTool.Toolbox.NineML) | multiply  (in PyDSTool.Toolbox.neuralcomp) | | mathNameMap  (in PyDSTool.ModelConstructor') | Min  (in PyDSTool.Toolbox.adjointPRC) | multiply  (in PyDSTool.Toolbox.phaseplane) | | mathNameMap  (in PyDSTool.ModelSpec') | Min  (in PyDSTool.Toolbox.dataanalysis) | multiply  (in PyDSTool.Toolbox.synthetic\_data) | | mathNameMap  (in PyDSTool.Symbolic) | Min  (in PyDSTool.Toolbox.fracdim) | multiply  (in PyDSTool.Toolbox.syntheticdata) | | mathNameMap  (in PyDSTool.Toolbox.NineML) | Min  (in PyDSTool.Toolbox.makeSloppyModel) | multiply  (in PyDSTool) | | mathNameMap  (in PyDSTool.Toolbox.dataanalysis) | Min  (in PyDSTool.Toolbox.neuralcomp) | multiply  (in matplotlib.pylab) | | mathNameMap  (in PyDSTool.Toolbox.phaseplane) | Min  (in PyDSTool.Toolbox.phaseplane) |  | |

  
  

| Home | Trees | Indices | Help | | PyDSTool | | --- | |
| --- | --- | --- | --- | --- | --- |

|  |  |
| --- | --- |
| Generated by Epydoc 3.0.1 on Fri May 4 15:23:58 2012 | http://epydoc.sourceforge.net |
