# Supplementary material for: Hybrid Models and Biological Model Reduction with PyDSTool
Source: PLoS Comput Biol. 2012 Aug 9;8(8):e1002628. doi: 10.1371/journal.pcbi.1002628 (PMC3415397; doi:10.1371/journal.pcbi.1002628)
Supplement: Text S4 — Complete source code for the PyDSTool package (version 0.88.120504). Includes API documentation and help files linking to web pages. This file is identical to the current public release on Sourceforge.net. (ZIP) [file pcbi.1002628.s004.zip › PyDSTool/html/identifier-index-N.html]

xml version="1.0" encoding="ascii"?


Identifier Index


| Home | Trees | Indices | Help | | PyDSTool | | --- | |
| --- | --- | --- | --- | --- | --- |

|  |  |  |  |
| --- | --- | --- | --- |
|  | |  | | --- | | [hide private] | | [frames] | no frames] | |

|  |  |
| --- | --- |
| Identifier Index | [ A B C D E F G H I J K L M N O P Q R S T U V W X Y Z \_ ] |

|  |  |  |  |  |  |  |  |  |  |  |  |  |  |  |  |  |  |  |  |  |  |  |  |  |  |  |  |  |  |  |  |  |  |  |  |  |  |  |  |  |  |  |  |  |  |  |  |  |  |  |  |  |  |  |  |  |  |  |  |  |  |  |  |  |  |  |  |  |  |  |  |  |  |  |  |  |  |  |  |  |  |  |  |  |  |  |  |  |  |  |  |  |  |  |  |  |  |  |  |  |  |  |  |  |  |  |  |  |  |  |  |  |  |  |  |  |  |  |  |  |  |  |  |  |  |  |  |  |  |  |  |  |  |  |  |  |  |  |  |  |  |  |  |  |  |  |  |  |  |  |  |  |  |  |  |  |  |  |  |  |  |  |  |  |  |  |  |  |  |  |  |  |  |  |  |  |  |  |  |  |  |  |  |  |  |  |  |  |  |  |  |  |  |  |  |  |  |  |  |  |  |  |  |  |  |  |  |  |  |  |  |  |  |  |  |  |  |  |  |  |  |  |  |  |  |  |  |  |  |  |  |  |  |  |  |  |  |  |  |  |  |  |  |  |  |  |  |  |  |  |  |  |  |  |  |  |  |  |  |  |  |  |  |  |  |  |  |  |  |  |  |  |  |  |  |  |  |  |  |  |  |  |  |  |  |  |  |  |  |  |  |  |  |  |  |  |  |  |  |  |  |  |  |  |  |  |  |  |  |  |  |  |  |  |  |  |  |  |  |  |  |  |  |  |  |  |  |  |  |  |  |  |  |  |  |  |  |
| --- | --- | --- | --- | --- | --- | --- | --- | --- | --- | --- | --- | --- | --- | --- | --- | --- | --- | --- | --- | --- | --- | --- | --- | --- | --- | --- | --- | --- | --- | --- | --- | --- | --- | --- | --- | --- | --- | --- | --- | --- | --- | --- | --- | --- | --- | --- | --- | --- | --- | --- | --- | --- | --- | --- | --- | --- | --- | --- | --- | --- | --- | --- | --- | --- | --- | --- | --- | --- | --- | --- | --- | --- | --- | --- | --- | --- | --- | --- | --- | --- | --- | --- | --- | --- | --- | --- | --- | --- | --- | --- | --- | --- | --- | --- | --- | --- | --- | --- | --- | --- | --- | --- | --- | --- | --- | --- | --- | --- | --- | --- | --- | --- | --- | --- | --- | --- | --- | --- | --- | --- | --- | --- | --- | --- | --- | --- | --- | --- | --- | --- | --- | --- | --- | --- | --- | --- | --- | --- | --- | --- | --- | --- | --- | --- | --- | --- | --- | --- | --- | --- | --- | --- | --- | --- | --- | --- | --- | --- | --- | --- | --- | --- | --- | --- | --- | --- | --- | --- | --- | --- | --- | --- | --- | --- | --- | --- | --- | --- | --- | --- | --- | --- | --- | --- | --- | --- | --- | --- | --- | --- | --- | --- | --- | --- | --- | --- | --- | --- | --- | --- | --- | --- | --- | --- | --- | --- | --- | --- | --- | --- | --- | --- | --- | --- | --- | --- | --- | --- | --- | --- | --- | --- | --- | --- | --- | --- | --- | --- | --- | --- | --- | --- | --- | --- | --- | --- | --- | --- | --- | --- | --- | --- | --- | --- | --- | --- | --- | --- | --- | --- | --- | --- | --- | --- | --- | --- | --- | --- | --- | --- | --- | --- | --- | --- | --- | --- | --- | --- | --- | --- | --- | --- | --- | --- | --- | --- | --- | --- | --- | --- | --- | --- | --- | --- | --- | --- | --- | --- | --- | --- | --- | --- | --- | --- | --- | --- | --- | --- | --- | --- | --- | --- | --- | --- | --- | --- | --- | --- | --- | --- | --- | --- | --- | --- | --- | --- | --- | --- | --- | --- | --- | --- | --- | --- | --- | --- | --- | --- | --- | --- | --- | --- | --- | --- | --- | --- | --- |
| N | |  |  |  | | --- | --- | --- | | n  (in PyDSTool.Interval') | NaN  (in PyDSTool.utils) | Normalvariate  (in PyDSTool.Toolbox.ActivationFuncs) | | n  (in PyDSTool.Symbolic) | NAN  (in matplotlib.pylab) | Normalvariate  (in PyDSTool.Toolbox.DSSRT\_tools) | | n  (in PyDSTool.Toolbox.ActivationFuncs) | NaN  (in matplotlib.pylab) | Normalvariate  (in PyDSTool.Toolbox.InputProfile) | | n  (in PyDSTool.Toolbox.DSSRT\_tools) | nan  (in matplotlib.pylab) | Normalvariate  (in PyDSTool.Toolbox.ModelHelper) | | n  (in PyDSTool.Toolbox.InputProfile) | nbytes  (in PyDSTool.PyCont.ContClass') | Normalvariate  (in PyDSTool.Toolbox.NineML) | | n  (in PyDSTool.Toolbox.ModelHelper) | nbytes  (in PyDSTool.Toolbox.ActivationFuncs) | Normalvariate  (in PyDSTool.Toolbox.adjointPRC) | | n  (in PyDSTool.Toolbox.NineML) | nbytes  (in PyDSTool.Toolbox.DSSRT\_tools) | Normalvariate  (in PyDSTool.Toolbox.dataanalysis) | | n  (in PyDSTool.Toolbox.adjointPRC) | nbytes  (in PyDSTool.Toolbox.InputProfile) | Normalvariate  (in PyDSTool.Toolbox.fracdim) | | n  (in PyDSTool.Toolbox.dataanalysis) | nbytes  (in PyDSTool.Toolbox.ModelHelper) | Normalvariate  (in PyDSTool.Toolbox.makeSloppyModel) | | n  (in PyDSTool.Toolbox.fracdim) | nbytes  (in PyDSTool.Toolbox.NineML) | Normalvariate  (in PyDSTool.Toolbox.neuralcomp) | | n  (in PyDSTool.Toolbox.makeSloppyModel) | nbytes  (in PyDSTool.Toolbox.adjointPRC) | Normalvariate  (in PyDSTool.Toolbox.phaseplane) | | n  (in PyDSTool.Toolbox.neuralcomp) | nbytes  (in PyDSTool.Toolbox.dataanalysis) | Normalvariate  (in PyDSTool.Toolbox.synthetic\_data) | | n  (in PyDSTool.Toolbox.phaseplane) | nbytes  (in PyDSTool.Toolbox.fracdim) | Normalvariate  (in PyDSTool.Toolbox.syntheticdata) | | n  (in PyDSTool.Toolbox.synthetic\_data) | nbytes  (in PyDSTool.Toolbox.makeSloppyModel) | not\_equal  (in PyDSTool.PyCont.ContClass') | | n  (in PyDSTool.Toolbox.syntheticdata) | nbytes  (in PyDSTool.Toolbox.neuralcomp) | not\_equal  (in PyDSTool.Toolbox.ActivationFuncs) | | n  (in PyDSTool) | nbytes  (in PyDSTool.Toolbox.phaseplane) | not\_equal  (in PyDSTool.Toolbox.DSSRT\_tools) | | n\_sigdigs\_str()  (in PyDSTool.common) | nbytes  (in PyDSTool.Toolbox.synthetic\_data) | not\_equal  (in PyDSTool.Toolbox.InputProfile) | | name  (in become\_most\_dominant) | nbytes  (in PyDSTool.Toolbox.syntheticdata) | not\_equal  (in PyDSTool.Toolbox.ModelHelper) | | name  (in is\_active) | nbytes  (in PyDSTool) | not\_equal  (in PyDSTool.Toolbox.NineML) | | name  (in is\_fast) | nbytes  (in matplotlib.pylab) | not\_equal  (in PyDSTool.Toolbox.adjointPRC) | | name  (in is\_inactive) | nearest\_2n\_indices()  (in PyDSTool.common) | not\_equal  (in PyDSTool.Toolbox.dataanalysis) | | name  (in is\_modulatory) | negate()  (in PyDSTool.PyCont.misc) | not\_equal  (in PyDSTool.Toolbox.fracdim) | | name  (in is\_most\_dominant) | negative  (in PyDSTool.PyCont.ContClass') | not\_equal  (in PyDSTool.Toolbox.makeSloppyModel) | | name  (in is\_order1) | negative  (in PyDSTool.Toolbox.ActivationFuncs) | not\_equal  (in PyDSTool.Toolbox.neuralcomp) | | name  (in is\_slow) | negative  (in PyDSTool.Toolbox.DSSRT\_tools) | not\_equal  (in PyDSTool.Toolbox.phaseplane) | | name  (in join\_actives) | negative  (in PyDSTool.Toolbox.InputProfile) | not\_equal  (in PyDSTool.Toolbox.synthetic\_data) | | name  (in join\_fast) | negative  (in PyDSTool.Toolbox.ModelHelper) | not\_equal  (in PyDSTool.Toolbox.syntheticdata) | | name  (in join\_slow) | negative  (in PyDSTool.Toolbox.NineML) | not\_equal  (in PyDSTool) | | name  (in leave\_actives) | negative  (in PyDSTool.Toolbox.adjointPRC) | not\_equal  (in matplotlib.pylab) | | name  (in leave\_fast) | negative  (in PyDSTool.Toolbox.dataanalysis) | not\_op  (in PyDSTool.common) | | name  (in leave\_slow) | negative  (in PyDSTool.Toolbox.fracdim) | notcontained  (in PyDSTool.Generator.ADMC\_ODEsystem') | | name  (in and\_op) | negative  (in PyDSTool.Toolbox.makeSloppyModel) | notcontained  (in PyDSTool.Generator.Dopri\_ODEsystem') | | name  (in not\_op) | negative  (in PyDSTool.Toolbox.neuralcomp) | notcontained  (in PyDSTool.Generator.EmbeddedSysGen') | | name  (in null\_predicate\_class) | negative  (in PyDSTool.Toolbox.phaseplane) | notcontained  (in PyDSTool.Generator.Euler\_ODEsystem') | | name  (in or\_op) | negative  (in PyDSTool.Toolbox.synthetic\_data) | notcontained  (in PyDSTool.Generator.ExplicitFnGen') | | name  (in predicate) | negative  (in PyDSTool.Toolbox.syntheticdata) | notcontained  (in PyDSTool.Generator.ExtrapolateTable') | | name\_chars\_RE  (in PyDSTool.FuncSpec') | negative  (in PyDSTool) | notcontained  (in PyDSTool.Generator.ImplicitFnGen') | | name\_chars\_RE  (in PyDSTool.ModelSpec') | negative  (in matplotlib.pylab) | notcontained  (in PyDSTool.Generator.InterpolateTable') | | name\_chars\_RE  (in PyDSTool.Symbolic) | network  (in PyDSTool.Toolbox.neuralcomp) | notcontained  (in PyDSTool.Generator.LookupTable') | | name\_chars\_RE  (in PyDSTool.Trajectory') | neuralcomp  (in PyDSTool.Toolbox) | notcontained  (in PyDSTool.Generator.MapSystem') | | name\_chars\_RE  (in PyDSTool.parseUtils) | neurite\_compartment  (in PyDSTool.Toolbox.neuralcomp) | notcontained  (in PyDSTool.Generator.ODEsystem') | | nameResolver  (in PyDSTool.ModelSpec') | neuro\_data  (in PyDSTool.Toolbox) | notcontained  (in PyDSTool.Generator.Radau\_ODEsystem') | | nameResolver  (in PyDSTool.Toolbox.NineML) | neuron  (in PyDSTool.Toolbox.neuralcomp) | notcontained  (in PyDSTool.Generator.Vode\_ODEsystem') | | nameResolver  (in PyDSTool.Toolbox.dataanalysis) | newaxis  (in PyDSTool.PyCont.ContClass') | notcontained  (in PyDSTool.Interval') | | nameResolver  (in PyDSTool.Toolbox.phaseplane) | newaxis  (in PyDSTool.Toolbox.ActivationFuncs) | notcontained  (in PyDSTool.Toolbox.NineML) | | nameResolver  (in PyDSTool.Toolbox.synthetic\_data) | newaxis  (in PyDSTool.Toolbox.DSSRT\_tools) | notcontained  (in PyDSTool.Toolbox.dataanalysis) | | nameResolver  (in PyDSTool.Toolbox.syntheticdata) | newaxis  (in PyDSTool.Toolbox.InputProfile) | notcontained  (in PyDSTool.Toolbox.phaseplane) | | nameResolverClass  (in PyDSTool.ModelSpec') | newaxis  (in PyDSTool.Toolbox.ModelHelper) | notcontained  (in PyDSTool.Toolbox.synthetic\_data) | | NAMESEP  (in PyDSTool.FuncSpec') | newaxis  (in PyDSTool.Toolbox.NineML) | notcontained  (in PyDSTool.Toolbox.syntheticdata) | | NAMESEP  (in PyDSTool.ModelConstructor') | newaxis  (in PyDSTool.Toolbox.adjointPRC) | notcontained  (in PyDSTool.Trajectory') | | NAMESEP  (in PyDSTool.ModelSpec') | newaxis  (in PyDSTool.Toolbox.data\_analysis) | notcontained  (in PyDSTool.Variable') | | NAMESEP  (in PyDSTool.Symbolic) | newaxis  (in PyDSTool.Toolbox.dataanalysis) | NS\_Det  (in PyDSTool.PyCont.TestFunc) | | NAMESEP  (in PyDSTool.Trajectory') | newaxis  (in PyDSTool.Toolbox.makeSloppyModel) | NSPoint  (in PyDSTool.PyCont.BifPoint) | | NAMESEP  (in PyDSTool.parseUtils) | newaxis  (in PyDSTool.Toolbox.neuralcomp) | null\_predicate  (in PyDSTool.FuncSpec') | | NaN  (in PyDSTool.Generator.ADMC\_ODEsystem') | newaxis  (in PyDSTool.Toolbox.phaseplane) | null\_predicate  (in PyDSTool.Generator.ADMC\_ODEsystem') | | NaN  (in PyDSTool.Generator.Dopri\_ODEsystem') | newaxis  (in PyDSTool.Toolbox.synthetic\_data) | null\_predicate  (in PyDSTool.Generator.Dopri\_ODEsystem') | | NaN  (in PyDSTool.Generator.EmbeddedSysGen') | newaxis  (in PyDSTool.Toolbox.syntheticdata) | null\_predicate  (in PyDSTool.Generator.EmbeddedSysGen') | | NaN  (in PyDSTool.Generator.Euler\_ODEsystem') | newaxis  (in PyDSTool) | null\_predicate  (in PyDSTool.Generator.Euler\_ODEsystem') | | NaN  (in PyDSTool.Generator.ExplicitFnGen') | newaxis  (in matplotlib.pylab) | null\_predicate  (in PyDSTool.Generator.ExplicitFnGen') | | NaN  (in PyDSTool.Generator.ExtrapolateTable') | newCurve()  (in ContClass) | null\_predicate  (in PyDSTool.Generator.ExtrapolateTable') | | NaN  (in PyDSTool.Generator.ImplicitFnGen') | NEWFALSE  (in PyDSTool.fixedpickle) | null\_predicate  (in PyDSTool.Generator.ImplicitFnGen') | | NaN  (in PyDSTool.Generator.InterpolateTable') | NEWOBJ  (in PyDSTool.fixedpickle) | null\_predicate  (in PyDSTool.Generator.InterpolateTable') | | NaN  (in PyDSTool.Generator.LookupTable') | newTempVars()  (in ImplicitFnGen) | null\_predicate  (in PyDSTool.Generator.LookupTable') | | NaN  (in PyDSTool.Generator.MapSystem') | newton\_step  (in PyDSTool.Toolbox.optimizers.step) | null\_predicate  (in PyDSTool.Generator.MapSystem') | | NaN  (in PyDSTool.Generator.ODEsystem') | NewtonStep  (in PyDSTool.Toolbox.optimizers.step.newton\_step) | null\_predicate  (in PyDSTool.Generator.ODEsystem') | | NaN  (in PyDSTool.Generator.Radau\_ODEsystem') | NEWTRUE  (in PyDSTool.fixedpickle) | null\_predicate  (in PyDSTool.Generator.Radau\_ODEsystem') | | NaN  (in PyDSTool.Generator.Vode\_ODEsystem') | nextafter  (in PyDSTool.PyCont.ContClass') | null\_predicate  (in PyDSTool.Generator.Vode\_ODEsystem') | | NaN  (in PyDSTool.Interval') | nextafter  (in PyDSTool.Toolbox.ActivationFuncs) | null\_predicate  (in PyDSTool.Interval') | | NaN  (in PyDSTool.ModelConstructor') | nextafter  (in PyDSTool.Toolbox.DSSRT\_tools) | null\_predicate  (in PyDSTool.ModelConstructor') | | NaN  (in PyDSTool.ModelSpec') | nextafter  (in PyDSTool.Toolbox.InputProfile) | null\_predicate  (in PyDSTool.ModelSpec') | | NAN  (in PyDSTool) | nextafter  (in PyDSTool.Toolbox.ModelHelper) | null\_predicate  (in PyDSTool.Symbolic) | | NAN  (in PyDSTool.PyCont.ContClass') | nextafter  (in PyDSTool.Toolbox.NineML) | null\_predicate  (in PyDSTool.Toolbox.NineML) | | NaN  (in PyDSTool.PyCont.ContClass') | nextafter  (in PyDSTool.Toolbox.adjointPRC) | null\_predicate  (in PyDSTool.Toolbox.dataanalysis) | | nan  (in PyDSTool.PyCont.ContClass') | nextafter  (in PyDSTool.Toolbox.dataanalysis) | null\_predicate  (in PyDSTool.Toolbox.event\_driven\_simulator) | | NaN  (in PyDSTool.PyCont.Continuation) | nextafter  (in PyDSTool.Toolbox.fracdim) | null\_predicate  (in PyDSTool.Toolbox.phaseplane) | | NaN  (in PyDSTool.PyCont.misc) | nextafter  (in PyDSTool.Toolbox.makeSloppyModel) | null\_predicate  (in PyDSTool.Toolbox.synthetic\_data) | | NaN  (in PyDSTool.Symbolic) | nextafter  (in PyDSTool.Toolbox.neuralcomp) | null\_predicate  (in PyDSTool.Toolbox.syntheticdata) | | NAN  (in PyDSTool.Toolbox.ActivationFuncs) | nextafter  (in PyDSTool.Toolbox.phaseplane) | null\_predicate  (in PyDSTool.Trajectory') | | nan  (in PyDSTool.Toolbox.ActivationFuncs) | nextafter  (in PyDSTool.Toolbox.synthetic\_data) | null\_predicate  (in PyDSTool.Variable') | | NAN  (in PyDSTool.Toolbox.DSSRT\_tools) | nextafter  (in PyDSTool.Toolbox.syntheticdata) | null\_predicate  (in PyDSTool.common) | | nan  (in PyDSTool.Toolbox.DSSRT\_tools) | nextafter  (in PyDSTool) | null\_predicate  (in PyDSTool.parseUtils) | | NAN  (in PyDSTool.Toolbox.InputProfile) | nextafter  (in matplotlib.pylab) | null\_predicate  (in PyDSTool.utils) | | nan  (in PyDSTool.Toolbox.InputProfile) | nhd()  (in PyDSTool.Toolbox.fracdim) | null\_predicate\_class  (in PyDSTool.common) | | NAN  (in PyDSTool.Toolbox.ModelHelper) | NineML  (in PyDSTool.Toolbox) | nullcline  (in PyDSTool.Toolbox.phaseplane) | | nan  (in PyDSTool.Toolbox.ModelHelper) | NineMLModel  (in PyDSTool.Toolbox.NineML) | nullcline\_zone\_leaf  (in PyDSTool.Toolbox.phaseplane) | | NAN  (in PyDSTool.Toolbox.NineML) | NINF  (in PyDSTool) | nullcline\_zone\_node  (in PyDSTool.Toolbox.phaseplane) | | NaN  (in PyDSTool.Toolbox.NineML) | NINF  (in PyDSTool.PyCont.ContClass') | num\_chars  (in PyDSTool.FuncSpec') | | nan  (in PyDSTool.Toolbox.NineML) | NINF  (in PyDSTool.Toolbox.ActivationFuncs) | num\_chars  (in PyDSTool.ModelSpec') | | NAN  (in PyDSTool.Toolbox.adjointPRC) | NINF  (in PyDSTool.Toolbox.DSSRT\_tools) | num\_chars  (in PyDSTool.Symbolic) | | nan  (in PyDSTool.Toolbox.adjointPRC) | NINF  (in PyDSTool.Toolbox.InputProfile) | num\_chars  (in PyDSTool.Trajectory') | | NAN  (in PyDSTool.Toolbox.dataanalysis) | NINF  (in PyDSTool.Toolbox.ModelHelper) | num\_chars  (in PyDSTool.parseUtils) | | NaN  (in PyDSTool.Toolbox.dataanalysis) | NINF  (in PyDSTool.Toolbox.NineML) | numeric\_to\_traj()  (in PyDSTool.Trajectory') | | nan  (in PyDSTool.Toolbox.dataanalysis) | NINF  (in PyDSTool.Toolbox.adjointPRC) | numeric\_to\_vars()  (in PyDSTool.Variable') | | NaN  (in PyDSTool.Toolbox.event\_driven\_simulator) | NINF  (in PyDSTool.Toolbox.dataanalysis) | numhess()  (in Function) | | NAN  (in PyDSTool.Toolbox.fracdim) | NINF  (in PyDSTool.Toolbox.fracdim) | NZERO  (in PyDSTool) | | nan  (in PyDSTool.Toolbox.fracdim) | NINF  (in PyDSTool.Toolbox.makeSloppyModel) | NZERO  (in PyDSTool.PyCont.ContClass') | | NAN  (in PyDSTool.Toolbox.makeSloppyModel) | NINF  (in PyDSTool.Toolbox.neuralcomp) | NZERO  (in PyDSTool.Toolbox.ActivationFuncs) | | nan  (in PyDSTool.Toolbox.makeSloppyModel) | NINF  (in PyDSTool.Toolbox.phaseplane) | NZERO  (in PyDSTool.Toolbox.DSSRT\_tools) | | NAN  (in PyDSTool.Toolbox.neuralcomp) | NINF  (in PyDSTool.Toolbox.synthetic\_data) | NZERO  (in PyDSTool.Toolbox.InputProfile) | | nan  (in PyDSTool.Toolbox.neuralcomp) | NINF  (in PyDSTool.Toolbox.syntheticdata) | NZERO  (in PyDSTool.Toolbox.ModelHelper) | | NAN  (in PyDSTool.Toolbox.phaseplane) | NINF  (in matplotlib.pylab) | NZERO  (in PyDSTool.Toolbox.NineML) | | NaN  (in PyDSTool.Toolbox.phaseplane) | node  (in PyDSTool.Toolbox.event\_driven\_simulator) | NZERO  (in PyDSTool.Toolbox.adjointPRC) | | nan  (in PyDSTool.Toolbox.phaseplane) | nodes\_order  (in PyDSTool.Toolbox.DSSRT\_tools) | NZERO  (in PyDSTool.Toolbox.dataanalysis) | | NAN  (in PyDSTool.Toolbox.synthetic\_data) | NONE  (in PyDSTool.fixedpickle) | NZERO  (in PyDSTool.Toolbox.fracdim) | | NaN  (in PyDSTool.Toolbox.synthetic\_data) | noneFn()  (in PyDSTool.common) | NZERO  (in PyDSTool.Toolbox.makeSloppyModel) | | nan  (in PyDSTool.Toolbox.synthetic\_data) | noneFn()  (in PyDSTool.scipy\_ode) | NZERO  (in PyDSTool.Toolbox.neuralcomp) | | NAN  (in PyDSTool.Toolbox.syntheticdata) | NonHybridModel  (in PyDSTool.Model) | NZERO  (in PyDSTool.Toolbox.phaseplane) | | NaN  (in PyDSTool.Toolbox.syntheticdata) | norm\_D\_sum()  (in PyDSTool.Toolbox.ParamEst) | NZERO  (in PyDSTool.Toolbox.synthetic\_data) | | nan  (in PyDSTool.Toolbox.syntheticdata) | normalized\_psis()  (in PyDSTool.Toolbox.dssrt) | NZERO  (in PyDSTool.Toolbox.syntheticdata) | | NaN  (in PyDSTool.Variable') | Normalvariate  (in PyDSTool.ModelSpec') | NZERO  (in matplotlib.pylab) | | NaN  (in PyDSTool.common) | Normalvariate  (in PyDSTool) |  | | nan  (in PyDSTool) | Normalvariate  (in PyDSTool.Symbolic) |  | |

  
  

| Home | Trees | Indices | Help | | PyDSTool | | --- | |
| --- | --- | --- | --- | --- | --- |

|  |  |
| --- | --- |
| Generated by Epydoc 3.0.1 on Fri May 4 15:23:58 2012 | http://epydoc.sourceforge.net |
