# Supplementary material for: Hybrid Models and Biological Model Reduction with PyDSTool
Source: PLoS Comput Biol. 2012 Aug 9;8(8):e1002628. doi: 10.1371/journal.pcbi.1002628 (PMC3415397; doi:10.1371/journal.pcbi.1002628)
Supplement: Text S4 — Complete source code for the PyDSTool package (version 0.88.120504). Includes API documentation and help files linking to web pages. This file is identical to the current public release on Sourceforge.net. (ZIP) [file pcbi.1002628.s004.zip › PyDSTool/html/identifier-index-O.html]

xml version="1.0" encoding="ascii"?


Identifier Index


| Home | Trees | Indices | Help | | PyDSTool | | --- | |
| --- | --- | --- | --- | --- | --- |

|  |  |  |  |
| --- | --- | --- | --- |
|  | |  | | --- | | [hide private] | | [frames] | no frames] | |

|  |  |
| --- | --- |
| Identifier Index | [ A B C D E F G H I J K L M N O P Q R S T U V W X Y Z \_ ] |

|  |  |  |  |  |  |  |  |  |  |  |  |  |  |  |  |  |  |  |  |  |  |  |  |  |  |  |  |  |  |  |  |  |  |  |  |  |  |  |  |  |  |  |  |  |  |  |  |  |  |  |  |  |  |  |  |  |  |  |  |  |  |  |  |  |  |  |  |
| --- | --- | --- | --- | --- | --- | --- | --- | --- | --- | --- | --- | --- | --- | --- | --- | --- | --- | --- | --- | --- | --- | --- | --- | --- | --- | --- | --- | --- | --- | --- | --- | --- | --- | --- | --- | --- | --- | --- | --- | --- | --- | --- | --- | --- | --- | --- | --- | --- | --- | --- | --- | --- | --- | --- | --- | --- | --- | --- | --- | --- | --- | --- | --- | --- | --- | --- | --- |
| O | |  |  |  | | --- | --- | --- | | OBJ  (in PyDSTool.fixedpickle) | one()  (in B\_Check) | ones\_like  (in matplotlib.pylab) | | object2str()  (in PyDSTool.common) | one\_period\_traj()  (in PyDSTool.Toolbox.PRCtools) | only\_dynamic()  (in PyDSTool.Toolbox.dssrt) | | ObjFSM  (in PyDSTool.Toolbox.FSM) | ONES  (in PyDSTool.FuncSpec') | open\_gentrans()  (in ModelTransform) | | ode  (in PyDSTool.scipy\_ode) | ONES  (in PyDSTool.ModelSpec') | open\_trans()  (in ModelManager) | | ODEsystem  (in PyDSTool.Generator.ODEsystem') | ONES  (in PyDSTool.Symbolic) | optimize()  (in Optimizer) | | ODEsystem'  (in PyDSTool.Generator) | ONES  (in PyDSTool.Trajectory') | optimizer  (in PyDSTool.Toolbox.optimizers) | | ogrid  (in PyDSTool.PyCont.ContClass') | ONES  (in PyDSTool.parseUtils) | optimizer  (in PyDSTool.Toolbox.optimizers.optimizer) | | ogrid  (in PyDSTool.Toolbox.ActivationFuncs) | ones\_like  (in PyDSTool.PyCont.ContClass') | Optimizer  (in PyDSTool.Toolbox.optimizers.optimizer.optimizer) | | ogrid  (in PyDSTool.Toolbox.DSSRT\_tools) | ones\_like  (in PyDSTool.Toolbox.ActivationFuncs) | optimizer\_\_all\_\_  (in PyDSTool.Toolbox.optimizers.optimizer) | | ogrid  (in PyDSTool.Toolbox.InputProfile) | ones\_like  (in PyDSTool.Toolbox.DSSRT\_tools) | optimizers  (in PyDSTool.Toolbox) | | ogrid  (in PyDSTool.Toolbox.ModelHelper) | ones\_like  (in PyDSTool.Toolbox.InputProfile) | or\_op  (in PyDSTool.common) | | ogrid  (in PyDSTool.Toolbox.NineML) | ones\_like  (in PyDSTool.Toolbox.ModelHelper) | OrComposition  (in PyDSTool.Toolbox.optimizers.criterion.composite\_criteria) | | ogrid  (in PyDSTool.Toolbox.adjointPRC) | ones\_like  (in PyDSTool.Toolbox.NineML) | orderEventData()  (in PyDSTool.utils) | | ogrid  (in PyDSTool.Toolbox.dataanalysis) | ones\_like  (in PyDSTool.Toolbox.adjointPRC) | organize\_feature\_sens()  (in PyDSTool.Toolbox.ParamEst) | | ogrid  (in PyDSTool.Toolbox.fracdim) | ones\_like  (in PyDSTool.Toolbox.dataanalysis) | other\_special\_points  (in PyDSTool.PyCont.Continuation) | | ogrid  (in PyDSTool.Toolbox.makeSloppyModel) | ones\_like  (in PyDSTool.Toolbox.fracdim) | out\_degree()  (in PyDSTool.Toolbox.FR) | | ogrid  (in PyDSTool.Toolbox.neuralcomp) | ones\_like  (in PyDSTool.Toolbox.makeSloppyModel) | out\_of\_seq()  (in PyDSTool.Toolbox.data\_analysis) | | ogrid  (in PyDSTool.Toolbox.phaseplane) | ones\_like  (in PyDSTool.Toolbox.neuralcomp) | out\_of\_seq()  (in PyDSTool.Toolbox.dataanalysis) | | ogrid  (in PyDSTool.Toolbox.synthetic\_data) | ones\_like  (in PyDSTool.Toolbox.phaseplane) | outputCFG()  (in DSSRT\_info) | | ogrid  (in PyDSTool.Toolbox.syntheticdata) | ones\_like  (in PyDSTool.Toolbox.synthetic\_data) | OutputFn  (in PyDSTool.Variable') | | ogrid  (in PyDSTool) | ones\_like  (in PyDSTool.Toolbox.syntheticdata) |  | | ogrid  (in matplotlib.pylab) | ones\_like  (in PyDSTool) |  | |

  
  

| Home | Trees | Indices | Help | | PyDSTool | | --- | |
| --- | --- | --- | --- | --- | --- |

|  |  |
| --- | --- |
| Generated by Epydoc 3.0.1 on Fri May 4 15:23:58 2012 | http://epydoc.sourceforge.net |
