# Supplementary material for: Hybrid Models and Biological Model Reduction with PyDSTool
Source: PLoS Comput Biol. 2012 Aug 9;8(8):e1002628. doi: 10.1371/journal.pcbi.1002628 (PMC3415397; doi:10.1371/journal.pcbi.1002628)
Supplement: Text S4 — Complete source code for the PyDSTool package (version 0.88.120504). Includes API documentation and help files linking to web pages. This file is identical to the current public release on Sourceforge.net. (ZIP) [file pcbi.1002628.s004.zip › PyDSTool/html/identifier-index-P.html]

xml version="1.0" encoding="ascii"?


Identifier Index


| Home | Trees | Indices | Help | | PyDSTool | | --- | |
| --- | --- | --- | --- | --- | --- |

|  |  |  |  |
| --- | --- | --- | --- |
|  | |  | | --- | | [hide private] | | [frames] | no frames] | |

|  |  |
| --- | --- |
| Identifier Index | [ A B C D E F G H I J K L M N O P Q R S T U V W X Y Z \_ ] |

|  |  |  |  |  |  |  |  |  |  |  |  |  |  |  |  |  |  |  |  |  |  |  |  |  |  |  |  |  |  |  |  |  |  |  |  |  |  |  |  |  |  |  |  |  |  |  |  |  |  |  |  |  |  |  |  |  |  |  |  |  |  |  |  |  |  |  |  |  |  |  |  |  |  |  |  |  |  |  |  |  |  |  |  |  |  |  |  |  |  |  |  |  |  |  |  |  |  |  |  |  |  |  |  |  |  |  |  |  |  |  |  |  |  |  |  |  |  |  |  |  |  |  |  |  |  |  |  |  |  |  |  |  |  |  |  |  |  |  |  |  |  |  |  |  |  |  |  |  |  |  |  |  |  |  |  |  |  |  |  |  |  |  |  |  |  |  |  |  |  |  |  |  |  |  |  |  |  |  |  |  |  |  |  |  |  |  |  |  |  |  |  |  |  |  |  |  |  |  |  |  |  |  |  |  |  |  |  |  |  |  |  |  |  |  |  |  |  |  |  |  |  |  |  |  |  |  |  |  |  |  |  |  |  |  |  |  |  |  |  |  |  |  |  |  |  |  |  |  |  |  |  |  |  |  |  |  |  |  |  |  |  |  |  |  |  |  |  |  |  |  |  |  |  |  |  |  |  |  |  |  |  |  |  |  |  |  |  |  |  |  |  |  |  |  |  |  |  |  |  |  |  |  |  |  |  |  |  |  |  |  |  |  |  |  |  |  |  |  |  |  |  |  |  |  |  |  |  |  |  |  |  |  |  |  |  |  |  |  |  |  |
| --- | --- | --- | --- | --- | --- | --- | --- | --- | --- | --- | --- | --- | --- | --- | --- | --- | --- | --- | --- | --- | --- | --- | --- | --- | --- | --- | --- | --- | --- | --- | --- | --- | --- | --- | --- | --- | --- | --- | --- | --- | --- | --- | --- | --- | --- | --- | --- | --- | --- | --- | --- | --- | --- | --- | --- | --- | --- | --- | --- | --- | --- | --- | --- | --- | --- | --- | --- | --- | --- | --- | --- | --- | --- | --- | --- | --- | --- | --- | --- | --- | --- | --- | --- | --- | --- | --- | --- | --- | --- | --- | --- | --- | --- | --- | --- | --- | --- | --- | --- | --- | --- | --- | --- | --- | --- | --- | --- | --- | --- | --- | --- | --- | --- | --- | --- | --- | --- | --- | --- | --- | --- | --- | --- | --- | --- | --- | --- | --- | --- | --- | --- | --- | --- | --- | --- | --- | --- | --- | --- | --- | --- | --- | --- | --- | --- | --- | --- | --- | --- | --- | --- | --- | --- | --- | --- | --- | --- | --- | --- | --- | --- | --- | --- | --- | --- | --- | --- | --- | --- | --- | --- | --- | --- | --- | --- | --- | --- | --- | --- | --- | --- | --- | --- | --- | --- | --- | --- | --- | --- | --- | --- | --- | --- | --- | --- | --- | --- | --- | --- | --- | --- | --- | --- | --- | --- | --- | --- | --- | --- | --- | --- | --- | --- | --- | --- | --- | --- | --- | --- | --- | --- | --- | --- | --- | --- | --- | --- | --- | --- | --- | --- | --- | --- | --- | --- | --- | --- | --- | --- | --- | --- | --- | --- | --- | --- | --- | --- | --- | --- | --- | --- | --- | --- | --- | --- | --- | --- | --- | --- | --- | --- | --- | --- | --- | --- | --- | --- | --- | --- | --- | --- | --- | --- | --- | --- | --- | --- | --- | --- | --- | --- | --- | --- | --- | --- | --- | --- | --- | --- | --- | --- | --- | --- | --- | --- | --- | --- | --- | --- | --- | --- | --- | --- | --- | --- | --- | --- | --- | --- | --- | --- | --- | --- | --- | --- | --- | --- | --- | --- | --- | --- | --- | --- | --- | --- | --- | --- | --- | --- | --- | --- | --- | --- | --- | --- | --- | --- | --- | --- | --- |
| P | |  |  |  | | --- | --- | --- | | pad()  (in PyDSTool.Toolbox.dssrt) | plot\_cycles()  (in LimitCycleCurve) | prepVarNames()  (in DSSRT\_info) | | padPointset()  (in PyDSTool.Points) | plot\_dim\_by\_thickness()  (in PyDSTool.Toolbox.fracdim) | process()  (in BPoint) | | Par  (in PyDSTool.Symbolic) | plot\_fps()  (in plotter\_2D) | process()  (in BTPoint) | | par\_sensitivity()  (in ParamEst) | plot\_hline()  (in plotter\_2D) | process()  (in BifPoint) | | ParamEst  (in PyDSTool.Toolbox) | plot\_line\_from\_points()  (in plotter\_2D) | process()  (in BranchPoint) | | ParamEst  (in PyDSTool.Toolbox.ParamEst) | plot\_markers()  (in PyDSTool.Toolbox.prep\_boxplot) | process()  (in CPPoint) | | parameters  (in PyDSTool.Toolbox.optimizers.defaults) | plot\_nullcline()  (in plotter\_2D) | process()  (in DHPoint) | | Paretovariate  (in PyDSTool.ModelSpec') | plot\_PCA\_residuals()  (in PyDSTool.Toolbox.data\_analysis) | process()  (in FoldPoint) | | Paretovariate  (in PyDSTool) | plot\_PCA\_residuals()  (in PyDSTool.Toolbox.dataanalysis) | process()  (in GHPoint) | | Paretovariate  (in PyDSTool.Symbolic) | plot\_PCA\_spectrum()  (in PyDSTool.Toolbox.data\_analysis) | process()  (in HopfPoint) | | Paretovariate  (in PyDSTool.Toolbox.ActivationFuncs) | plot\_PCA\_spectrum()  (in PyDSTool.Toolbox.dataanalysis) | process()  (in LPCPoint) | | Paretovariate  (in PyDSTool.Toolbox.DSSRT\_tools) | plot\_point()  (in plotter\_2D) | process()  (in NSPoint) | | Paretovariate  (in PyDSTool.Toolbox.InputProfile) | plot\_PP\_fps()  (in PyDSTool.Toolbox.phaseplane) | process()  (in PDPoint) | | Paretovariate  (in PyDSTool.Toolbox.ModelHelper) | plot\_PP\_vf()  (in PyDSTool.Toolbox.phaseplane) | process()  (in SPoint) | | Paretovariate  (in PyDSTool.Toolbox.NineML) | plot\_psis()  (in PyDSTool.Toolbox.dssrt) | process()  (in ZHPoint) | | Paretovariate  (in PyDSTool.Toolbox.adjointPRC) | plot\_radius\_distribution()  (in PyDSTool.Toolbox.fracdim) | process()  (in FSM) | | Paretovariate  (in PyDSTool.Toolbox.dataanalysis) | plot\_thickness\_by\_dim()  (in PyDSTool.Toolbox.fracdim) | process\_list()  (in FSM) | | Paretovariate  (in PyDSTool.Toolbox.fracdim) | plot\_vf()  (in plotter\_2D) | process\_raw\_residual()  (in PyDSTool.MProject) | | Paretovariate  (in PyDSTool.Toolbox.makeSloppyModel) | plot\_vline()  (in plotter\_2D) | processMacro()  (in PyDSTool.ModelSpec') | | Paretovariate  (in PyDSTool.Toolbox.neuralcomp) | plotNetworkGraph()  (in PyDSTool.Toolbox.DSSRT\_tools) | processMultiDef()  (in PyDSTool.Symbolic) | | Paretovariate  (in PyDSTool.Toolbox.phaseplane) | plotProfile()  (in PyDSTool.Toolbox.InputProfile) | processMultiRef()  (in PyDSTool.Symbolic) | | Paretovariate  (in PyDSTool.Toolbox.synthetic\_data) | plotter  (in PyDSTool.Toolbox.phaseplane) | processReused()  (in PyDSTool.ModelConstructor') | | Paretovariate  (in PyDSTool.Toolbox.syntheticdata) | plotter\_2D  (in PyDSTool.Toolbox.phaseplane) | processReusedC()  (in PyDSTool.Events) | | pargs  (in PyDSTool.PyCont.Plotting) | Plotting  (in PyDSTool.PyCont) | processReusedMatlab()  (in PyDSTool.Events) | | pars\_array\_to\_dict()  (in ParamEst) | pnnetwork  (in PyDSTool.Toolbox.neuralcomp) | processReusedPy()  (in PyDSTool.Events) | | pars\_dict\_to\_array()  (in ParamEst) | Point  (in PyDSTool.Points) | project  (in PyDSTool.conf) | | pars\_to\_ixs()  (in ParamEst) | Point2D  (in PyDSTool.Toolbox.phaseplane) | propagate\_verbosity()  (in feature\_node) | | parse()  (in parserObject) | point\_keys  (in PyDSTool.Points) | proper\_match()  (in PyDSTool.parseUtils) | | parseMatrixStrToDictStr()  (in PyDSTool.parseUtils) | PointInfo  (in PyDSTool.Points) | protected\_allnames  (in PyDSTool.FuncSpec') | | parserObject  (in PyDSTool.parseUtils) | Points  (in PyDSTool) | protected\_allnames  (in PyDSTool.ModelSpec') | | parseUtils  (in PyDSTool) | Pointset  (in PyDSTool.Points) | protected\_allnames  (in PyDSTool.Symbolic) | | ParTestFunc  (in PyDSTool.PyCont.TestFunc) | pointset\_to\_traj()  (in PyDSTool.Trajectory') | protected\_allnames  (in PyDSTool.Toolbox.NineML) | | partial\_step  (in PyDSTool.Toolbox.optimizers.step) | pointset\_to\_vars()  (in PyDSTool.Variable') | protected\_allnames  (in PyDSTool.Toolbox.dataanalysis) | | PartialStep  (in PyDSTool.Toolbox.optimizers.step.partial\_step) | pointsToPointset()  (in PyDSTool.Points) | protected\_allnames  (in PyDSTool.Toolbox.phaseplane) | | partition()  (in PyDSTool.PyCont.misc) | poll()  (in connection) | protected\_allnames  (in PyDSTool.Toolbox.synthetic\_data) | | partition\_range()  (in PyDSTool.Toolbox.dssrt) | poll()  (in node) | protected\_allnames  (in PyDSTool.Toolbox.syntheticdata) | | pca\_dim()  (in PyDSTool.Toolbox.data\_analysis) | pollHighLevelEvents()  (in EventStruct) | protected\_allnames  (in PyDSTool.Trajectory') | | pca\_dim()  (in PyDSTool.Toolbox.dataanalysis) | POP  (in PyDSTool.fixedpickle) | protected\_allnames  (in PyDSTool.parseUtils) | | PCANode  (in PyDSTool.Toolbox.dataanalysis) | pop()  (in FIFOqueue\_uniquenode) | protected\_auxnamesDB  (in PyDSTool.FuncSpec') | | PCANode()  (in PyDSTool.Toolbox.data\_analysis) | pop()  (in args) | protected\_auxnamesDB  (in PyDSTool.ModelSpec') | | PD\_Det  (in PyDSTool.PyCont.TestFunc) | POP\_MARK  (in PyDSTool.fixedpickle) | protected\_auxnamesDB  (in PyDSTool.Symbolic) | | PD\_E()  (in PyDSTool.Toolbox.fracdim) | popFromQ()  (in Event) | protected\_auxnamesDB  (in PyDSTool.Toolbox.NineML) | | PDPoint  (in PyDSTool.PyCont.BifPoint) | popitem()  (in args) | protected\_auxnamesDB  (in PyDSTool.Toolbox.dataanalysis) | | PERSID  (in PyDSTool.fixedpickle) | postprocess\_ref\_traj()  (in feature) | protected\_auxnamesDB  (in PyDSTool.Toolbox.phaseplane) | | persistent\_id()  (in Pickler) | postprocess\_ref\_traj()  (in L2\_feature) | protected\_auxnamesDB  (in PyDSTool.Toolbox.synthetic\_data) | | phaseplane  (in PyDSTool.Toolbox) | postprocess\_ref\_traj()  (in L2\_feature\_1D) | protected\_auxnamesDB  (in PyDSTool.Toolbox.syntheticdata) | | phaseplane  (in PyDSTool.Toolbox.phaseplane) | postprocess\_ref\_traj()  (in burst\_feature) | protected\_auxnamesDB  (in PyDSTool.Trajectory') | | pi  (in PyDSTool.Generator.ADMC\_ODEsystem') | postprocess\_ref\_traj()  (in get\_burst\_active\_phase) | protected\_auxnamesDB  (in PyDSTool.parseUtils) | | pi  (in PyDSTool.Generator.DDEsystem) | postprocess\_ref\_traj()  (in get\_burst\_dc\_offset) | protected\_macronames  (in PyDSTool.FuncSpec') | | pi  (in PyDSTool.Generator.Dopri\_ODEsystem') | postprocess\_ref\_traj()  (in get\_burst\_downsweep) | protected\_macronames  (in PyDSTool.ModelSpec') | | pi  (in PyDSTool.Generator.EmbeddedSysGen') | postprocess\_ref\_traj()  (in get\_burst\_duration) | protected\_macronames  (in PyDSTool.Symbolic) | | pi  (in PyDSTool.Generator.Euler\_ODEsystem') | postprocess\_ref\_traj()  (in get\_burst\_isi\_env) | protected\_macronames  (in PyDSTool.Trajectory') | | pi  (in PyDSTool.Generator.ExplicitFnGen') | postprocess\_ref\_traj()  (in get\_burst\_passive\_extent) | protected\_macronames  (in PyDSTool.parseUtils) | | pi  (in PyDSTool.Generator.ExtrapolateTable') | postprocess\_ref\_traj()  (in get\_burst\_peak\_env) | protected\_mathnames  (in PyDSTool.FuncSpec') | | pi  (in PyDSTool.Generator.ImplicitFnGen') | postprocess\_ref\_traj()  (in get\_burst\_spikes) | protected\_mathnames  (in PyDSTool.ModelSpec') | | pi  (in PyDSTool.Generator.InterpolateTable') | postprocess\_ref\_traj()  (in get\_burst\_trough\_env) | protected\_mathnames  (in PyDSTool.Symbolic) | | pi  (in PyDSTool.Generator.LookupTable') | postprocess\_ref\_traj()  (in get\_burst\_upsweep) | protected\_mathnames  (in PyDSTool.Trajectory') | | pi  (in PyDSTool.Generator.MapSystem') | postprocess\_test\_traj()  (in ModelInterface) | protected\_mathnames  (in PyDSTool.parseUtils) | | pi  (in PyDSTool.Generator.ODEsystem') | Pow  (in PyDSTool.ModelSpec') | protected\_randomnames  (in PyDSTool.FuncSpec') | | pi  (in PyDSTool.Generator.Radau\_ODEsystem') | Pow  (in PyDSTool) | protected\_randomnames  (in PyDSTool.ModelSpec') | | pi  (in PyDSTool.Generator.Vode\_ODEsystem') | Pow  (in PyDSTool.Symbolic) | protected\_randomnames  (in PyDSTool.Symbolic) | | pi  (in PyDSTool.Generator.allimports) | pow  (in PyDSTool.Symbolic) | protected\_randomnames  (in PyDSTool.Trajectory') | | pi  (in PyDSTool.Generator.baseclasses) | Pow  (in PyDSTool.Toolbox.ActivationFuncs) | protected\_randomnames  (in PyDSTool.parseUtils) | | pi  (in PyDSTool.Generator) | Pow  (in PyDSTool.Toolbox.DSSRT\_tools) | protected\_scipynames  (in PyDSTool.FuncSpec') | | Pi  (in PyDSTool.ModelSpec') | Pow  (in PyDSTool.Toolbox.InputProfile) | protected\_scipynames  (in PyDSTool.ModelSpec') | | pi  (in PyDSTool.ModelSpec') | Pow  (in PyDSTool.Toolbox.ModelHelper) | protected\_scipynames  (in PyDSTool.Symbolic) | | Pi  (in PyDSTool) | Pow  (in PyDSTool.Toolbox.NineML) | protected\_scipynames  (in PyDSTool.Trajectory') | | pi  (in PyDSTool.PyCont.ContClass') | Pow  (in PyDSTool.Toolbox.adjointPRC) | protected\_scipynames  (in PyDSTool.parseUtils) | | PI  (in PyDSTool.PyCont.misc) | Pow  (in PyDSTool.Toolbox.dataanalysis) | protected\_specialfns  (in PyDSTool.FuncSpec') | | Pi  (in PyDSTool.Symbolic) | Pow  (in PyDSTool.Toolbox.fracdim) | protected\_specialfns  (in PyDSTool.ModelSpec') | | pi  (in PyDSTool.Symbolic) | Pow  (in PyDSTool.Toolbox.makeSloppyModel) | protected\_specialfns  (in PyDSTool.Symbolic) | | Pi  (in PyDSTool.Toolbox.ActivationFuncs) | Pow  (in PyDSTool.Toolbox.neuralcomp) | protected\_specialfns  (in PyDSTool.Trajectory') | | Pi  (in PyDSTool.Toolbox.DSSRT\_tools) | Pow  (in PyDSTool.Toolbox.phaseplane) | protected\_specialfns  (in PyDSTool.parseUtils) | | Pi  (in PyDSTool.Toolbox.InputProfile) | Pow  (in PyDSTool.Toolbox.synthetic\_data) | PROTO  (in PyDSTool.fixedpickle) | | Pi  (in PyDSTool.Toolbox.ModelHelper) | Pow  (in PyDSTool.Toolbox.syntheticdata) | PRPConjugateGradientStep()  (in PyDSTool.Toolbox.optimizers.step.conjugate\_gradient\_step) | | Pi  (in PyDSTool.Toolbox.NineML) | POW\_STR  (in PyDSTool.parseUtils) | push()  (in FIFOqueue\_uniquenode) | | pi  (in PyDSTool.Toolbox.NineML) | Powell  (in PyDSTool.Toolbox.optimizers.tests.test\_powell) | PUT  (in PyDSTool.fixedpickle) | | Pi  (in PyDSTool.Toolbox.adjointPRC) | power  (in PyDSTool.Symbolic) | put()  (in Pickler) | | Pi  (in PyDSTool.Toolbox.dataanalysis) | power  (in PyDSTool.Toolbox.NineML) | PyCont  (in PyDSTool) | | pi  (in PyDSTool.Toolbox.dataanalysis) | power  (in PyDSTool.Toolbox.ParamEst) | PyDSTool | | Pi  (in PyDSTool.Toolbox.fracdim) | power  (in PyDSTool.Toolbox.dataanalysis) | PyDSTool\_AttributeError  (in PyDSTool.errors) | | Pi  (in PyDSTool.Toolbox.makeSloppyModel) | power  (in PyDSTool.Toolbox.phaseplane) | PyDSTool\_BoundsError  (in PyDSTool.errors) | | Pi  (in PyDSTool.Toolbox.neuralcomp) | power  (in PyDSTool.Toolbox.synthetic\_data) | PyDSTool\_ClearError  (in PyDSTool.errors) | | Pi  (in PyDSTool.Toolbox.phaseplane) | power  (in PyDSTool.Toolbox.syntheticdata) | PyDSTool\_ContError  (in PyDSTool.errors) | | pi  (in PyDSTool.Toolbox.phaseplane) | pp()  (in PyDSTool.Toolbox.ParamEst) | PyDSTool\_Error  (in PyDSTool.errors) | | Pi  (in PyDSTool.Toolbox.synthetic\_data) | PRCtools  (in PyDSTool.Toolbox) | PyDSTool\_ExistError  (in PyDSTool.errors) | | pi  (in PyDSTool.Toolbox.synthetic\_data) | precondition()  (in become\_most\_dominant) | PyDSTool\_InitError  (in PyDSTool.errors) | | Pi  (in PyDSTool.Toolbox.syntheticdata) | precondition()  (in join\_actives) | PyDSTool\_KeyError  (in PyDSTool.errors) | | pi  (in PyDSTool.Toolbox.syntheticdata) | precondition()  (in join\_fast) | PyDSTool\_TypeError  (in PyDSTool.errors) | | pi  (in matplotlib.pylab) | precondition()  (in join\_slow) | PyDSTool\_UncertainValueError  (in PyDSTool.errors) | | PickleError  (in PyDSTool.fixedpickle) | precondition()  (in leave\_actives) | PyDSTool\_UndefinedError  (in PyDSTool.errors) | | PickleError | precondition()  (in leave\_fast) | PyDSTool\_ValueError  (in PyDSTool.errors) | | Pickler  (in PyDSTool.fixedpickle) | precondition()  (in leave\_slow) | pygments\_style  (in PyDSTool.conf) | | PicklingError  (in PyDSTool.fixedpickle) | precondition()  (in not\_op) | pylab  (in matplotlib) | | PicklingError | precondition()  (in predicate) | PySCes\_SBML  (in PyDSTool.Toolbox) | | PiecewisePolynomial  (in PyDSTool.common) | precondition()  (in predicate\_op) | PyStringMap  (in PyDSTool.fixedpickle) | | PINF  (in PyDSTool) | predicate  (in PyDSTool.common) | PZERO  (in PyDSTool) | | PINF  (in PyDSTool.PyCont.ContClass') | predicate\_op  (in PyDSTool.common) | PZERO  (in PyDSTool.PyCont.ContClass') | | PINF  (in PyDSTool.Toolbox.ActivationFuncs) | prep\_boxplot  (in PyDSTool.Toolbox) | PZERO  (in PyDSTool.Toolbox.ActivationFuncs) | | PINF  (in PyDSTool.Toolbox.DSSRT\_tools) | prep\_boxplots()  (in PyDSTool.Toolbox.prep\_boxplot) | PZERO  (in PyDSTool.Toolbox.DSSRT\_tools) | | PINF  (in PyDSTool.Toolbox.InputProfile) | prep\_secant\_figure()  (in PyDSTool.Toolbox.fracdim) | PZERO  (in PyDSTool.Toolbox.InputProfile) | | PINF  (in PyDSTool.Toolbox.ModelHelper) | prepare()  (in feature) | PZERO  (in PyDSTool.Toolbox.ModelHelper) | | PINF  (in PyDSTool.Toolbox.NineML) | prepare()  (in burst\_feature) | PZERO  (in PyDSTool.Toolbox.NineML) | | PINF  (in PyDSTool.Toolbox.adjointPRC) | prepare\_conditions()  (in ModelInterface) | PZERO  (in PyDSTool.Toolbox.adjointPRC) | | PINF  (in PyDSTool.Toolbox.dataanalysis) | prepAuxFns()  (in DSSRT\_info) | PZERO  (in PyDSTool.Toolbox.dataanalysis) | | PINF  (in PyDSTool.Toolbox.fracdim) | prepCFG()  (in DSSRT\_info) | PZERO  (in PyDSTool.Toolbox.fracdim) | | PINF  (in PyDSTool.Toolbox.makeSloppyModel) | prepDEpars()  (in DSSRT\_info) | PZERO  (in PyDSTool.Toolbox.makeSloppyModel) | | PINF  (in PyDSTool.Toolbox.neuralcomp) | prepDirection()  (in ODEsystem) | PZERO  (in PyDSTool.Toolbox.neuralcomp) | | PINF  (in PyDSTool.Toolbox.phaseplane) | prepGraph()  (in DSSRT\_info) | PZERO  (in PyDSTool.Toolbox.phaseplane) | | PINF  (in PyDSTool.Toolbox.synthetic\_data) | prepInputs()  (in DSSRT\_info) | PZERO  (in PyDSTool.Toolbox.synthetic\_data) | | PINF  (in PyDSTool.Toolbox.syntheticdata) | prepJacobian()  (in PyDSTool.Symbolic) | PZERO  (in PyDSTool.Toolbox.syntheticdata) | | PINF  (in matplotlib.pylab) | preprocessFlatten()  (in ModelConstructor) | PZERO  (in matplotlib.pylab) | | plot()  (in PyDSTool.matplotlib\_import) | preprocessReuseTerms()  (in ModelConstructor) |  | |

  
  

| Home | Trees | Indices | Help | | PyDSTool | | --- | |
| --- | --- | --- | --- | --- | --- |

|  |  |
| --- | --- |
| Generated by Epydoc 3.0.1 on Fri May 4 15:23:58 2012 | http://epydoc.sourceforge.net |
