# Supplementary material for: Hybrid Models and Biological Model Reduction with PyDSTool
Source: PLoS Comput Biol. 2012 Aug 9;8(8):e1002628. doi: 10.1371/journal.pcbi.1002628 (PMC3415397; doi:10.1371/journal.pcbi.1002628)
Supplement: Text S4 — Complete source code for the PyDSTool package (version 0.88.120504). Includes API documentation and help files linking to web pages. This file is identical to the current public release on Sourceforge.net. (ZIP) [file pcbi.1002628.s004.zip › PyDSTool/html/identifier-index-R.html]

xml version="1.0" encoding="ascii"?


Identifier Index


| Home | Trees | Indices | Help | | PyDSTool | | --- | |
| --- | --- | --- | --- | --- | --- |

|  |  |  |  |
| --- | --- | --- | --- |
|  | |  | | --- | | [hide private] | | [frames] | no frames] | |

|  |  |
| --- | --- |
| Identifier Index | [ A B C D E F G H I J K L M N O P Q R S T U V W X Y Z \_ ] |

|  |  |  |  |  |  |  |  |  |  |  |  |  |  |  |  |  |  |  |  |  |  |  |  |  |  |  |  |  |  |  |  |  |  |  |  |  |  |  |  |  |  |  |  |  |  |  |  |  |  |  |  |  |  |  |  |  |  |  |  |  |  |  |  |  |  |  |  |  |  |  |  |  |  |  |  |  |  |  |  |  |  |  |  |  |  |  |  |  |  |  |  |  |  |  |  |  |  |  |  |  |  |  |  |  |  |  |  |  |  |  |  |  |  |  |  |  |  |  |  |  |  |  |  |  |  |  |  |  |  |  |  |  |  |  |  |  |  |  |  |  |  |  |  |  |  |  |  |  |  |  |  |  |  |  |  |  |  |  |  |  |  |  |  |  |  |  |  |  |  |  |  |  |  |  |  |  |  |  |  |  |  |  |  |  |  |  |  |  |  |  |  |  |  |  |  |  |  |  |  |  |  |  |  |  |  |  |  |  |  |  |  |  |  |  |  |  |  |  |  |  |  |  |  |  |  |  |  |  |  |  |  |  |  |  |  |  |  |  |  |  |  |  |  |  |  |  |  |  |  |  |  |  |  |  |  |  |  |  |  |  |  |  |  |  |  |  |  |  |  |  |  |  |  |  |  |  |  |  |  |  |  |  |  |  |  |  |  |  |  |  |  |  |  |  |  |  |  |  |  |  |  |  |  |  |  |  |  |  |  |  |  |  |  |  |  |  |  |  |  |  |  |  |  |  |  |  |  |  |  |  |  |
| --- | --- | --- | --- | --- | --- | --- | --- | --- | --- | --- | --- | --- | --- | --- | --- | --- | --- | --- | --- | --- | --- | --- | --- | --- | --- | --- | --- | --- | --- | --- | --- | --- | --- | --- | --- | --- | --- | --- | --- | --- | --- | --- | --- | --- | --- | --- | --- | --- | --- | --- | --- | --- | --- | --- | --- | --- | --- | --- | --- | --- | --- | --- | --- | --- | --- | --- | --- | --- | --- | --- | --- | --- | --- | --- | --- | --- | --- | --- | --- | --- | --- | --- | --- | --- | --- | --- | --- | --- | --- | --- | --- | --- | --- | --- | --- | --- | --- | --- | --- | --- | --- | --- | --- | --- | --- | --- | --- | --- | --- | --- | --- | --- | --- | --- | --- | --- | --- | --- | --- | --- | --- | --- | --- | --- | --- | --- | --- | --- | --- | --- | --- | --- | --- | --- | --- | --- | --- | --- | --- | --- | --- | --- | --- | --- | --- | --- | --- | --- | --- | --- | --- | --- | --- | --- | --- | --- | --- | --- | --- | --- | --- | --- | --- | --- | --- | --- | --- | --- | --- | --- | --- | --- | --- | --- | --- | --- | --- | --- | --- | --- | --- | --- | --- | --- | --- | --- | --- | --- | --- | --- | --- | --- | --- | --- | --- | --- | --- | --- | --- | --- | --- | --- | --- | --- | --- | --- | --- | --- | --- | --- | --- | --- | --- | --- | --- | --- | --- | --- | --- | --- | --- | --- | --- | --- | --- | --- | --- | --- | --- | --- | --- | --- | --- | --- | --- | --- | --- | --- | --- | --- | --- | --- | --- | --- | --- | --- | --- | --- | --- | --- | --- | --- | --- | --- | --- | --- | --- | --- | --- | --- | --- | --- | --- | --- | --- | --- | --- | --- | --- | --- | --- | --- | --- | --- | --- | --- | --- | --- | --- | --- | --- | --- | --- | --- | --- | --- | --- | --- | --- | --- | --- | --- | --- | --- | --- | --- | --- | --- | --- | --- | --- | --- | --- | --- | --- | --- | --- | --- | --- | --- | --- | --- | --- | --- | --- | --- | --- | --- | --- | --- | --- | --- | --- | --- | --- | --- | --- | --- | --- | --- | --- |
| R | |  |  |  | | --- | --- | --- | | r\_  (in PyDSTool.PyCont.ContClass') | Randrange  (in PyDSTool) | res\_fn\_lookup  (in PyDSTool.Toolbox.ParamEst) | | r\_  (in PyDSTool.PyCont.Continuation) | Randrange  (in PyDSTool.Symbolic) | rescatter()  (in PyDSTool.Toolbox.fracdim) | | r\_  (in PyDSTool.PyCont.misc) | Randrange  (in PyDSTool.Toolbox.ActivationFuncs) | reserved\_keywords  (in PyDSTool.parseUtils) | | r\_  (in PyDSTool.Toolbox.ActivationFuncs) | Randrange  (in PyDSTool.Toolbox.DSSRT\_tools) | reset()  (in Event) | | r\_  (in PyDSTool.Toolbox.DSSRT\_tools) | Randrange  (in PyDSTool.Toolbox.InputProfile) | reset()  (in Continuation) | | r\_  (in PyDSTool.Toolbox.InputProfile) | Randrange  (in PyDSTool.Toolbox.ModelHelper) | reset()  (in EquilibriumCurve) | | r\_  (in PyDSTool.Toolbox.ModelHelper) | Randrange  (in PyDSTool.Toolbox.NineML) | reset()  (in LimitCycleCurve) | | r\_  (in PyDSTool.Toolbox.NineML) | Randrange  (in PyDSTool.Toolbox.adjointPRC) | reset()  (in FSM) | | r\_  (in PyDSTool.Toolbox.adjointPRC) | Randrange  (in PyDSTool.Toolbox.dataanalysis) | reset()  (in dssrt\_assistant) | | r\_  (in PyDSTool.Toolbox.data\_analysis) | Randrange  (in PyDSTool.Toolbox.fracdim) | reset()  (in FIFOqueue\_uniquenode) | | r\_  (in PyDSTool.Toolbox.dataanalysis) | Randrange  (in PyDSTool.Toolbox.makeSloppyModel) | reset()  (in base\_n\_counter) | | r\_  (in PyDSTool.Toolbox.makeSloppyModel) | Randrange  (in PyDSTool.Toolbox.neuralcomp) | Reset()  (in integrator) | | r\_  (in PyDSTool.Toolbox.neuralcomp) | Randrange  (in PyDSTool.Toolbox.phaseplane) | reset()  (in IntegratorBase) | | r\_  (in PyDSTool.Toolbox.phaseplane) | Randrange  (in PyDSTool.Toolbox.synthetic\_data) | reset()  (in vode) | | r\_  (in PyDSTool.Toolbox.synthetic\_data) | Randrange  (in PyDSTool.Toolbox.syntheticdata) | reset\_log()  (in ParamEst) | | r\_  (in PyDSTool.Toolbox.syntheticdata) | rcParams  (in PyDSTool.PyCont.ContClass') | reset\_metric()  (in feature) | | r\_  (in PyDSTool) | rcParams  (in matplotlib.pylab) | reset\_weights()  (in context) | | r\_  (in matplotlib.pylab) | rcParamsDefault  (in PyDSTool.PyCont.ContClass') | resetEvents()  (in Generator) | | rad2deg  (in PyDSTool.PyCont.ContClass') | rcParamsDefault  (in matplotlib.pylab) | resetEventTimes()  (in Generator) | | rad2deg  (in PyDSTool.Toolbox.ActivationFuncs) | re\_number  (in PyDSTool.Interval') | resetEventTimes()  (in Model) | | rad2deg  (in PyDSTool.Toolbox.DSSRT\_tools) | readArgs()  (in PyDSTool.parseUtils) | resetEvtimes()  (in EventStruct) | | rad2deg  (in PyDSTool.Toolbox.InputProfile) | reciprocal  (in PyDSTool.PyCont.ContClass') | resetHighLevelEvents()  (in EventStruct) | | rad2deg  (in PyDSTool.Toolbox.ModelHelper) | reciprocal  (in PyDSTool.Toolbox.ActivationFuncs) | resetParArgs()  (in ParamEst) | | rad2deg  (in PyDSTool.Toolbox.NineML) | reciprocal  (in PyDSTool.Toolbox.DSSRT\_tools) | residual()  (in context) | | rad2deg  (in PyDSTool.Toolbox.adjointPRC) | reciprocal  (in PyDSTool.Toolbox.InputProfile) | residual()  (in FiniteDifferencesCache) | | rad2deg  (in PyDSTool.Toolbox.dataanalysis) | reciprocal  (in PyDSTool.Toolbox.ModelHelper) | residual()  (in FiniteDifferencesFunction) | | rad2deg  (in PyDSTool.Toolbox.fracdim) | reciprocal  (in PyDSTool.Toolbox.NineML) | residual\_fn\_context  (in PyDSTool.Toolbox.ParamEst) | | rad2deg  (in PyDSTool.Toolbox.makeSloppyModel) | reciprocal  (in PyDSTool.Toolbox.adjointPRC) | residual\_fn\_context\_1D  (in PyDSTool.Toolbox.ParamEst) | | rad2deg  (in PyDSTool.Toolbox.neuralcomp) | reciprocal  (in PyDSTool.Toolbox.dataanalysis) | resolve\_bin\_index()  (in data\_bins) | | rad2deg  (in PyDSTool.Toolbox.phaseplane) | reciprocal  (in PyDSTool.Toolbox.fracdim) | resolve\_bin\_index()  (in data\_bins) | | rad2deg  (in PyDSTool.Toolbox.synthetic\_data) | reciprocal  (in PyDSTool.Toolbox.makeSloppyModel) | resolveClashingAuxFnPars()  (in PyDSTool.FuncSpec') | | rad2deg  (in PyDSTool.Toolbox.syntheticdata) | reciprocal  (in PyDSTool.Toolbox.neuralcomp) | resolveMacroTargets()  (in PyDSTool.ModelSpec') | | rad2deg  (in PyDSTool) | reciprocal  (in PyDSTool.Toolbox.phaseplane) | resolveSpecTypeCombos()  (in PyDSTool.Symbolic) | | rad2deg  (in matplotlib.pylab) | reciprocal  (in PyDSTool.Toolbox.synthetic\_data) | restart()  (in PyDSTool) | | radau  (in PyDSTool.Generator.Radau\_ODEsystem') | reciprocal  (in PyDSTool.Toolbox.syntheticdata) | restart\_conjugate\_gradient  (in PyDSTool.Toolbox.optimizers.step) | | Radau\_ODEsystem  (in PyDSTool.Generator.Radau\_ODEsystem') | reciprocal  (in PyDSTool) | RestartNotOrthogonalConjugateGradientStep  (in PyDSTool.Toolbox.optimizers.step.restart\_conjugate\_gradient) | | Radau\_ODEsystem'  (in PyDSTool.Generator) | reciprocal  (in matplotlib.pylab) | RestartPeriodicallyConjugateGradientStep  (in PyDSTool.Toolbox.optimizers.step.restart\_conjugate\_gradient) | | Radians  (in PyDSTool.ModelSpec') | recordHistory()  (in Optimizer) | restrict\_opt()  (in PyDSTool.Toolbox.ParamEst) | | radians  (in PyDSTool.PyCont.ContClass') | recreate()  (in FuncSpec) | reverse()  (in Pointset) | | Radians  (in PyDSTool) | rectify()  (in PyDSTool.Toolbox.data\_analysis) | Rhs()  (in Dopri\_ODEsystem) | | Radians  (in PyDSTool.Symbolic) | recurrence\_times()  (in PyDSTool.Toolbox.data\_analysis) | Rhs()  (in Euler\_ODEsystem) | | Radians  (in PyDSTool.Toolbox.ActivationFuncs) | recurrence\_times()  (in PyDSTool.Toolbox.dataanalysis) | Rhs()  (in MapSystem) | | Radians  (in PyDSTool.Toolbox.DSSRT\_tools) | redefine()  (in QuantSpec) | Rhs()  (in ODEsystem) | | Radians  (in PyDSTool.Toolbox.InputProfile) | redefine()  (in Quantity) | Rhs()  (in Radau\_ODEsystem) | | Radians  (in PyDSTool.Toolbox.ModelHelper) | redefineQuantity()  (in GenTransform) | Rhs()  (in Vode\_ODEsystem) | | Radians  (in PyDSTool.Toolbox.NineML) | Redirector  (in PyDSTool) | Rhs()  (in GeneratorInterface) | | radians  (in PyDSTool.Toolbox.NineML) | Redirector  (in PyDSTool.Redirector) | Rhs()  (in ModelInterface) | | Radians  (in PyDSTool.Toolbox.adjointPRC) | REDUCE  (in PyDSTool.fixedpickle) | Rhs()  (in HybridModel) | | Radians  (in PyDSTool.Toolbox.dataanalysis) | reduced\_Vode\_system()  (in dssrt\_assistant) | Rhs()  (in NonHybridModel) | | radians  (in PyDSTool.Toolbox.dataanalysis) | refresh()  (in pargs) | Rhs()  (in integrator) | | Radians  (in PyDSTool.Toolbox.fracdim) | regime  (in PyDSTool.Toolbox.dssrt) | RHSfuncSpec  (in PyDSTool.FuncSpec') | | Radians  (in PyDSTool.Toolbox.makeSloppyModel) | register()  (in genDBClass) | right\_shift  (in PyDSTool.PyCont.ContClass') | | Radians  (in PyDSTool.Toolbox.neuralcomp) | regObject  (in PyDSTool.ModelSpec') | right\_shift  (in PyDSTool.Toolbox.ActivationFuncs) | | Radians  (in PyDSTool.Toolbox.phaseplane) | RelativeParametersCriterion  (in PyDSTool.Toolbox.optimizers.criterion.criteria) | right\_shift  (in PyDSTool.Toolbox.DSSRT\_tools) | | radians  (in PyDSTool.Toolbox.phaseplane) | RelativeValueCriterion  (in PyDSTool.Toolbox.optimizers.criterion.criteria) | right\_shift  (in PyDSTool.Toolbox.InputProfile) | | Radians  (in PyDSTool.Toolbox.synthetic\_data) | release  (in PyDSTool.conf) | right\_shift  (in PyDSTool.Toolbox.ModelHelper) | | radians  (in PyDSTool.Toolbox.synthetic\_data) | remain()  (in PyDSTool.common) | right\_shift  (in PyDSTool.Toolbox.NineML) | | Radians  (in PyDSTool.Toolbox.syntheticdata) | remain()  (in PyDSTool.utils) | right\_shift  (in PyDSTool.Toolbox.adjointPRC) | | radians  (in PyDSTool.Toolbox.syntheticdata) | remainder  (in PyDSTool.PyCont.ContClass') | right\_shift  (in PyDSTool.Toolbox.dataanalysis) | | radians  (in matplotlib.pylab) | remainder  (in PyDSTool.Toolbox.ActivationFuncs) | right\_shift  (in PyDSTool.Toolbox.fracdim) | | RAISE  (in PyDSTool.PyCont.ContClass') | remainder  (in PyDSTool.Toolbox.DSSRT\_tools) | right\_shift  (in PyDSTool.Toolbox.makeSloppyModel) | | RAISE  (in PyDSTool) | remainder  (in PyDSTool.Toolbox.InputProfile) | right\_shift  (in PyDSTool.Toolbox.neuralcomp) | | RAISE  (in PyDSTool.Toolbox.ActivationFuncs) | remainder  (in PyDSTool.Toolbox.ModelHelper) | right\_shift  (in PyDSTool.Toolbox.phaseplane) | | RAISE  (in PyDSTool.Toolbox.DSSRT\_tools) | remainder  (in PyDSTool.Toolbox.NineML) | right\_shift  (in PyDSTool.Toolbox.synthetic\_data) | | RAISE  (in PyDSTool.Toolbox.InputProfile) | remainder  (in PyDSTool.Toolbox.adjointPRC) | right\_shift  (in PyDSTool.Toolbox.syntheticdata) | | RAISE  (in PyDSTool.Toolbox.ModelHelper) | remainder  (in PyDSTool.Toolbox.dataanalysis) | right\_shift  (in PyDSTool) | | RAISE  (in PyDSTool.Toolbox.NineML) | remainder  (in PyDSTool.Toolbox.fracdim) | right\_shift  (in matplotlib.pylab) | | RAISE  (in PyDSTool.Toolbox.adjointPRC) | remainder  (in PyDSTool.Toolbox.makeSloppyModel) | rint  (in PyDSTool.PyCont.ContClass') | | RAISE  (in PyDSTool.Toolbox.dataanalysis) | remainder  (in PyDSTool.Toolbox.neuralcomp) | rint  (in PyDSTool.Toolbox.ActivationFuncs) | | RAISE  (in PyDSTool.Toolbox.fracdim) | remainder  (in PyDSTool.Toolbox.phaseplane) | rint  (in PyDSTool.Toolbox.DSSRT\_tools) | | RAISE  (in PyDSTool.Toolbox.makeSloppyModel) | remainder  (in PyDSTool.Toolbox.synthetic\_data) | rint  (in PyDSTool.Toolbox.InputProfile) | | RAISE  (in PyDSTool.Toolbox.neuralcomp) | remainder  (in PyDSTool.Toolbox.syntheticdata) | rint  (in PyDSTool.Toolbox.ModelHelper) | | RAISE  (in PyDSTool.Toolbox.phaseplane) | remainder  (in PyDSTool) | rint  (in PyDSTool.Toolbox.NineML) | | RAISE  (in PyDSTool.Toolbox.synthetic\_data) | remainder  (in matplotlib.pylab) | rint  (in PyDSTool.Toolbox.adjointPRC) | | RAISE  (in PyDSTool.Toolbox.syntheticdata) | remove()  (in GenTransform) | rint  (in PyDSTool.Toolbox.dataanalysis) | | RAISE  (in matplotlib.pylab) | remove()  (in ModelManager) | rint  (in PyDSTool.Toolbox.fracdim) | | Randint  (in PyDSTool.ModelSpec') | remove()  (in ModelTransform) | rint  (in PyDSTool.Toolbox.makeSloppyModel) | | Randint  (in PyDSTool) | remove()  (in ModelSpec) | rint  (in PyDSTool.Toolbox.neuralcomp) | | Randint  (in PyDSTool.Symbolic) | remove()  (in PointInfo) | rint  (in PyDSTool.Toolbox.phaseplane) | | Randint  (in PyDSTool.Toolbox.ActivationFuncs) | remove()  (in Pointset) | rint  (in PyDSTool.Toolbox.synthetic\_data) | | Randint  (in PyDSTool.Toolbox.DSSRT\_tools) | remove()  (in phaseplane) | rint  (in PyDSTool.Toolbox.syntheticdata) | | Randint  (in PyDSTool.Toolbox.InputProfile) | remove\_indices\_from\_range()  (in PyDSTool.parseUtils) | rint  (in PyDSTool) | | Randint  (in PyDSTool.Toolbox.ModelHelper) | removeAuxFn()  (in auxfnDBclass) | rint  (in matplotlib.pylab) | | Randint  (in PyDSTool.Toolbox.NineML) | removelabel()  (in Point) | rollback\_gentrans()  (in ModelTransform) | | Randint  (in PyDSTool.Toolbox.adjointPRC) | removelabel()  (in Pointset) | rollback\_trans()  (in ModelManager) | | Randint  (in PyDSTool.Toolbox.dataanalysis) | rename()  (in ModelSpec) | Rosenbrock  (in PyDSTool.Toolbox.optimizers.tests.test\_rosenbrock) | | Randint  (in PyDSTool.Toolbox.fracdim) | rename()  (in Pointset) | rotate\_phase()  (in PyDSTool.Toolbox.adjointPRC) | | Randint  (in PyDSTool.Toolbox.makeSloppyModel) | rename()  (in Quantity) | rout  (in PyDSTool.Generator.Dopri\_ODEsystem') | | Randint  (in PyDSTool.Toolbox.neuralcomp) | renameTraj()  (in Model) | rout  (in PyDSTool.Generator.Radau\_ODEsystem') | | Randint  (in PyDSTool.Toolbox.phaseplane) | renderForCode()  (in QuantSpec) | rout  (in PyDSTool.PyCont.ContClass') | | Randint  (in PyDSTool.Toolbox.synthetic\_data) | renderForCode()  (in Quantity) | rout  (in PyDSTool.Toolbox.NineML) | | Randint  (in PyDSTool.Toolbox.syntheticdata) | replaceCallsWithDummies()  (in PyDSTool.parseUtils) | rout  (in PyDSTool.Toolbox.ParamEst) | | Random  (in PyDSTool.ModelSpec') | replaceSep()  (in PyDSTool.parseUtils) | rout  (in PyDSTool.Toolbox.dataanalysis) | | Random  (in PyDSTool) | replaceSepInv()  (in PyDSTool.parseUtils) | rout  (in PyDSTool.Toolbox.phaseplane) | | Random  (in PyDSTool.Symbolic) | replaceSepList()  (in PyDSTool.parseUtils) | rout  (in PyDSTool.Toolbox.synthetic\_data) | | Random  (in PyDSTool.Toolbox.ActivationFuncs) | replaceSepListInv()  (in PyDSTool.parseUtils) | rout  (in PyDSTool.Toolbox.syntheticdata) | | Random  (in PyDSTool.Toolbox.DSSRT\_tools) | replaceSepQSpec()  (in PyDSTool.parseUtils) | rout  (in PyDSTool.utils) | | Random  (in PyDSTool.Toolbox.InputProfile) | replaceSepStr()  (in PyDSTool.parseUtils) | Run()  (in dopri) | | Random  (in PyDSTool.Toolbox.ModelHelper) | report()  (in Verbose) | Run()  (in radau) | | Random  (in PyDSTool.Toolbox.NineML) | report\_error()  (in Verbose) | run()  (in BoundMin) | | Random  (in PyDSTool.Toolbox.adjointPRC) | rerr  (in PyDSTool.Generator.Dopri\_ODEsystem') | run()  (in LMpest) | | Random  (in PyDSTool.Toolbox.dataanalysis) | rerr  (in PyDSTool.Generator.Radau\_ODEsystem') | run()  (in ParamEst) | | Random  (in PyDSTool.Toolbox.fracdim) | rerr  (in PyDSTool.PyCont.ContClass') | run()  (in simulator) | | Random  (in PyDSTool.Toolbox.makeSloppyModel) | rerr  (in PyDSTool.Toolbox.NineML) | Run()  (in integrator) | | Random  (in PyDSTool.Toolbox.neuralcomp) | rerr  (in PyDSTool.Toolbox.ParamEst) | run()  (in IntegratorBase) | | Random  (in PyDSTool.Toolbox.phaseplane) | rerr  (in PyDSTool.Toolbox.dataanalysis) | run()  (in vode) | | Random  (in PyDSTool.Toolbox.synthetic\_data) | rerr  (in PyDSTool.Toolbox.phaseplane) | run\_relax()  (in IntegratorBase) | | Random  (in PyDSTool.Toolbox.syntheticdata) | rerr  (in PyDSTool.Toolbox.synthetic\_data) | run\_relax()  (in vode) | | randomlookup  (in PyDSTool.Symbolic) | rerr  (in PyDSTool.Toolbox.syntheticdata) | runner  (in IntegratorBase) | | Randrange  (in PyDSTool.ModelSpec') | rerr  (in PyDSTool.utils) | runner  (in vode) | |

  
  

| Home | Trees | Indices | Help | | PyDSTool | | --- | |
| --- | --- | --- | --- | --- | --- |

|  |  |
| --- | --- |
| Generated by Epydoc 3.0.1 on Fri May 4 15:23:58 2012 | http://epydoc.sourceforge.net |
