# Supplementary material for: Hybrid Models and Biological Model Reduction with PyDSTool
Source: PLoS Comput Biol. 2012 Aug 9;8(8):e1002628. doi: 10.1371/journal.pcbi.1002628 (PMC3415397; doi:10.1371/journal.pcbi.1002628)
Supplement: Text S4 — Complete source code for the PyDSTool package (version 0.88.120504). Includes API documentation and help files linking to web pages. This file is identical to the current public release on Sourceforge.net. (ZIP) [file pcbi.1002628.s004.zip › PyDSTool/html/identifier-index-S.html]

xml version="1.0" encoding="ascii"?


Identifier Index


| Home | Trees | Indices | Help | | PyDSTool | | --- | |
| --- | --- | --- | --- | --- | --- |

|  |  |  |  |
| --- | --- | --- | --- |
|  | |  | | --- | | [hide private] | | [frames] | no frames] | |

|  |  |
| --- | --- |
| Identifier Index | [ A B C D E F G H I J K L M N O P Q R S T U V W X Y Z \_ ] |

|  |  |  |  |  |  |  |  |  |  |  |  |  |  |  |  |  |  |  |  |  |  |  |  |  |  |  |  |  |  |  |  |  |  |  |  |  |  |  |  |  |  |  |  |  |  |  |  |  |  |  |  |  |  |  |  |  |  |  |  |  |  |  |  |  |  |  |  |  |  |  |  |  |  |  |  |  |  |  |  |  |  |  |  |  |  |  |  |  |  |  |  |  |  |  |  |  |  |  |  |  |  |  |  |  |  |  |  |  |  |  |  |  |  |  |  |  |  |  |  |  |  |  |  |  |  |  |  |  |  |  |  |  |  |  |  |  |  |  |  |  |  |  |  |  |  |  |  |  |  |  |  |  |  |  |  |  |  |  |  |  |  |  |  |  |  |  |  |  |  |  |  |  |  |  |  |  |  |  |  |  |  |  |  |  |  |  |  |  |  |  |  |  |  |  |  |  |  |  |  |  |  |  |  |  |  |  |  |  |  |  |  |  |  |  |  |  |  |  |  |  |  |  |  |  |  |  |  |  |  |  |  |  |  |  |  |  |  |  |  |  |  |  |  |  |  |  |  |  |  |  |  |  |  |  |  |  |  |  |  |  |  |  |  |  |  |  |  |  |  |  |  |  |  |  |  |  |  |  |  |  |  |  |  |  |  |  |  |  |  |  |  |  |  |  |  |  |  |  |  |  |  |  |  |  |  |  |  |  |  |  |  |  |  |  |  |  |  |  |  |  |  |  |  |  |  |  |  |  |  |  |  |  |  |  |  |  |  |  |  |  |  |  |  |  |  |  |  |  |  |  |  |  |  |  |  |  |  |  |  |  |  |  |  |  |  |  |  |  |  |  |  |  |  |  |  |  |  |  |  |  |  |  |  |  |  |  |  |  |  |  |  |  |  |  |  |  |  |  |  |  |  |  |  |  |  |  |  |  |  |  |  |  |  |  |  |  |  |  |  |  |  |  |  |  |  |  |  |  |  |  |  |  |  |  |  |  |  |  |  |  |  |  |  |  |  |  |  |  |  |  |  |  |  |  |  |  |  |  |  |  |  |  |  |  |  |  |  |  |  |  |  |  |  |  |  |  |  |  |  |  |  |  |  |  |  |  |  |  |  |  |  |  |  |  |  |  |  |  |  |  |  |  |  |  |  |  |  |  |  |  |  |  |  |  |  |  |  |  |  |  |  |  |  |  |  |  |  |  |  |  |  |  |  |  |  |  |  |  |  |  |  |  |  |  |  |  |  |  |  |  |  |  |  |  |  |  |  |  |  |  |  |  |  |  |  |  |  |  |  |  |  |  |  |  |  |  |  |  |  |  |  |  |  |  |  |  |  |  |  |  |  |  |  |  |  |  |  |  |  |  |  |  |  |  |  |  |  |  |  |  |  |  |  |  |  |  |  |  |  |  |  |  |  |  |  |  |  |  |  |  |  |  |  |  |  |  |  |  |  |  |  |  |  |  |  |  |  |  |  |  |  |  |  |  |  |  |  |  |  |  |  |  |  |  |  |  |  |  |  |  |  |  |  |  |  |  |  |  |  |  |  |  |  |  |  |
| --- | --- | --- | --- | --- | --- | --- | --- | --- | --- | --- | --- | --- | --- | --- | --- | --- | --- | --- | --- | --- | --- | --- | --- | --- | --- | --- | --- | --- | --- | --- | --- | --- | --- | --- | --- | --- | --- | --- | --- | --- | --- | --- | --- | --- | --- | --- | --- | --- | --- | --- | --- | --- | --- | --- | --- | --- | --- | --- | --- | --- | --- | --- | --- | --- | --- | --- | --- | --- | --- | --- | --- | --- | --- | --- | --- | --- | --- | --- | --- | --- | --- | --- | --- | --- | --- | --- | --- | --- | --- | --- | --- | --- | --- | --- | --- | --- | --- | --- | --- | --- | --- | --- | --- | --- | --- | --- | --- | --- | --- | --- | --- | --- | --- | --- | --- | --- | --- | --- | --- | --- | --- | --- | --- | --- | --- | --- | --- | --- | --- | --- | --- | --- | --- | --- | --- | --- | --- | --- | --- | --- | --- | --- | --- | --- | --- | --- | --- | --- | --- | --- | --- | --- | --- | --- | --- | --- | --- | --- | --- | --- | --- | --- | --- | --- | --- | --- | --- | --- | --- | --- | --- | --- | --- | --- | --- | --- | --- | --- | --- | --- | --- | --- | --- | --- | --- | --- | --- | --- | --- | --- | --- | --- | --- | --- | --- | --- | --- | --- | --- | --- | --- | --- | --- | --- | --- | --- | --- | --- | --- | --- | --- | --- | --- | --- | --- | --- | --- | --- | --- | --- | --- | --- | --- | --- | --- | --- | --- | --- | --- | --- | --- | --- | --- | --- | --- | --- | --- | --- | --- | --- | --- | --- | --- | --- | --- | --- | --- | --- | --- | --- | --- | --- | --- | --- | --- | --- | --- | --- | --- | --- | --- | --- | --- | --- | --- | --- | --- | --- | --- | --- | --- | --- | --- | --- | --- | --- | --- | --- | --- | --- | --- | --- | --- | --- | --- | --- | --- | --- | --- | --- | --- | --- | --- | --- | --- | --- | --- | --- | --- | --- | --- | --- | --- | --- | --- | --- | --- | --- | --- | --- | --- | --- | --- | --- | --- | --- | --- | --- | --- | --- | --- | --- | --- | --- | --- | --- | --- | --- | --- | --- | --- | --- | --- | --- | --- | --- | --- | --- | --- | --- | --- | --- | --- | --- | --- | --- | --- | --- | --- | --- | --- | --- | --- | --- | --- | --- | --- | --- | --- | --- | --- | --- | --- | --- | --- | --- | --- | --- | --- | --- | --- | --- | --- | --- | --- | --- | --- | --- | --- | --- | --- | --- | --- | --- | --- | --- | --- | --- | --- | --- | --- | --- | --- | --- | --- | --- | --- | --- | --- | --- | --- | --- | --- | --- | --- | --- | --- | --- | --- | --- | --- | --- | --- | --- | --- | --- | --- | --- | --- | --- | --- | --- | --- | --- | --- | --- | --- | --- | --- | --- | --- | --- | --- | --- | --- | --- | --- | --- | --- | --- | --- | --- | --- | --- | --- | --- | --- | --- | --- | --- | --- | --- | --- | --- | --- | --- | --- | --- | --- | --- | --- | --- | --- | --- | --- | --- | --- | --- | --- | --- | --- | --- | --- | --- | --- | --- | --- | --- | --- | --- | --- | --- | --- | --- | --- | --- | --- | --- | --- | --- | --- | --- | --- | --- | --- | --- | --- | --- | --- | --- | --- | --- | --- | --- | --- | --- | --- | --- | --- | --- | --- | --- | --- | --- | --- | --- | --- | --- | --- | --- | --- | --- | --- | --- | --- | --- | --- | --- | --- | --- | --- | --- | --- | --- | --- | --- | --- | --- | --- | --- | --- | --- | --- | --- | --- | --- | --- | --- | --- | --- | --- | --- | --- | --- | --- | --- | --- | --- | --- | --- | --- | --- | --- | --- | --- | --- | --- | --- | --- | --- | --- | --- | --- | --- | --- | --- | --- | --- | --- | --- | --- | --- | --- | --- | --- | --- | --- | --- | --- | --- | --- | --- | --- | --- | --- | --- | --- | --- | --- | --- | --- | --- | --- | --- | --- | --- | --- | --- | --- | --- | --- | --- | --- | --- | --- | --- | --- | --- | --- | --- | --- | --- | --- | --- | --- | --- | --- | --- | --- | --- | --- | --- | --- | --- | --- | --- | --- | --- | --- | --- | --- | --- | --- | --- | --- | --- | --- | --- | --- | --- | --- | --- | --- | --- | --- | --- | --- | --- | --- | --- | --- | --- | --- | --- | --- | --- | --- | --- | --- | --- | --- | --- | --- | --- | --- | --- | --- | --- | --- | --- | --- | --- | --- | --- | --- |
| S | |  |  |  | | --- | --- | --- | | s  (in PyDSTool.parseUtils) | setFn()  (in ParamEst) | Sinh  (in PyDSTool.Toolbox.makeSloppyModel) | | s\_  (in PyDSTool.PyCont.ContClass') | setForcedAuxVars()  (in GeneratorConstructor) | Sinh  (in PyDSTool.Toolbox.neuralcomp) | | s\_  (in PyDSTool.Toolbox.ActivationFuncs) | setglobalt0()  (in EventStruct) | Sinh  (in PyDSTool.Toolbox.phaseplane) | | s\_  (in PyDSTool.Toolbox.DSSRT\_tools) | setICs()  (in Model) | sinh  (in PyDSTool.Toolbox.phaseplane) | | s\_  (in PyDSTool.Toolbox.InputProfile) | setIndepdomain()  (in Variable) | Sinh  (in PyDSTool.Toolbox.synthetic\_data) | | s\_  (in PyDSTool.Toolbox.ModelHelper) | setInteg()  (in integrator) | sinh  (in PyDSTool.Toolbox.synthetic\_data) | | s\_  (in PyDSTool.Toolbox.NineML) | setInternalVars()  (in ModelConstructor) | Sinh  (in PyDSTool.Toolbox.syntheticdata) | | s\_  (in PyDSTool.Toolbox.adjointPRC) | SETITEM  (in PyDSTool.fixedpickle) | sinh  (in PyDSTool.Toolbox.syntheticdata) | | s\_  (in PyDSTool.Toolbox.dataanalysis) | SETITEMS  (in PyDSTool.fixedpickle) | sinh  (in matplotlib.pylab) | | s\_  (in PyDSTool.Toolbox.fracdim) | setLabel()  (in pargs) | slope\_range()  (in PyDSTool.Toolbox.fracdim) | | s\_  (in PyDSTool.Toolbox.makeSloppyModel) | setLabels()  (in pargs) | sloppyModel  (in PyDSTool.Toolbox.makeSloppyModel) | | s\_  (in PyDSTool.Toolbox.neuralcomp) | setLegend()  (in pargs) | slow()  (in VarAlphabet) | | s\_  (in PyDSTool.Toolbox.phaseplane) | setLegends()  (in pargs) | SMALL\_DELTA\_F  (in PyDSTool.Toolbox.optimizers.defaults) | | s\_  (in PyDSTool.Toolbox.synthetic\_data) | setLevel()  (in Verbose) | SMALL\_DELTA\_F\_F  (in PyDSTool.Toolbox.optimizers.defaults) | | s\_  (in PyDSTool.Toolbox.syntheticdata) | setOptions()  (in GeneratorConstructor) | SMALL\_DELTA\_X  (in PyDSTool.Toolbox.optimizers.defaults) | | s\_  (in PyDSTool) | setOutput()  (in Variable) | SMALL\_DELTA\_X\_X  (in PyDSTool.Toolbox.optimizers.defaults) | | s\_  (in matplotlib.pylab) | setPars()  (in Model) | SMALL\_DF  (in PyDSTool.Toolbox.optimizers.defaults) | | SA  (in PyDSTool.PyCont.ContClass') | setPreciseFlag()  (in EventStruct) | smooth\_pts()  (in PyDSTool.common) | | SA  (in matplotlib.pylab) | setRadauParams()  (in radau) | solution\_measures  (in PyDSTool.PyCont.Continuation) | | Sample  (in PyDSTool.ModelSpec') | setReuseTerms()  (in GeneratorConstructor) | solution\_measures\_list  (in PyDSTool.PyCont.Continuation) | | Sample  (in PyDSTool) | setReuseTerms()  (in ModelConstructor) | SOLVED\_WITH\_UNIMPLEMENTED\_OR\_UNKNOWN\_REASON  (in PyDSTool.Toolbox.optimizers.defaults) | | Sample  (in PyDSTool.Symbolic) | setRunParams()  (in integrator) | solver\_lookup  (in PyDSTool.Toolbox.ParamEst) | | Sample  (in PyDSTool.Toolbox.ActivationFuncs) | setStartTime()  (in EventStruct) | soma  (in PyDSTool.Toolbox.neuralcomp) | | Sample  (in PyDSTool.Toolbox.DSSRT\_tools) | Setstate  (in PyDSTool.ModelSpec') | sortByIndex()  (in PointInfo) | | Sample  (in PyDSTool.Toolbox.InputProfile) | Setstate  (in PyDSTool) | sortByLabel()  (in PointInfo) | | Sample  (in PyDSTool.Toolbox.ModelHelper) | Setstate  (in PyDSTool.Symbolic) | sorted\_by\_radius()  (in PyDSTool.Toolbox.fracdim) | | Sample  (in PyDSTool.Toolbox.NineML) | Setstate  (in PyDSTool.Toolbox.ActivationFuncs) | sorted\_by\_slope()  (in PyDSTool.Toolbox.fracdim) | | Sample  (in PyDSTool.Toolbox.adjointPRC) | Setstate  (in PyDSTool.Toolbox.DSSRT\_tools) | sortedDictItems()  (in PyDSTool.common) | | Sample  (in PyDSTool.Toolbox.dataanalysis) | Setstate  (in PyDSTool.Toolbox.InputProfile) | sortedDictKeys()  (in PyDSTool.common) | | Sample  (in PyDSTool.Toolbox.fracdim) | Setstate  (in PyDSTool.Toolbox.ModelHelper) | sortedDictLists()  (in PyDSTool.common) | | Sample  (in PyDSTool.Toolbox.makeSloppyModel) | Setstate  (in PyDSTool.Toolbox.NineML) | sortedDictValues()  (in PyDSTool.common) | | Sample  (in PyDSTool.Toolbox.neuralcomp) | Setstate  (in PyDSTool.Toolbox.adjointPRC) | sortedEventNames()  (in EventStruct) | | Sample  (in PyDSTool.Toolbox.phaseplane) | Setstate  (in PyDSTool.Toolbox.dataanalysis) | source\_suffix  (in PyDSTool.conf) | | Sample  (in PyDSTool.Toolbox.synthetic\_data) | Setstate  (in PyDSTool.Toolbox.fracdim) | spacing  (in PyDSTool.PyCont.ContClass') | | Sample  (in PyDSTool.Toolbox.syntheticdata) | Setstate  (in PyDSTool.Toolbox.makeSloppyModel) | spacing  (in PyDSTool.Toolbox.ActivationFuncs) | | sample()  (in Interval) | Setstate  (in PyDSTool.Toolbox.neuralcomp) | spacing  (in PyDSTool.Toolbox.DSSRT\_tools) | | sample()  (in Model) | Setstate  (in PyDSTool.Toolbox.phaseplane) | spacing  (in PyDSTool.Toolbox.InputProfile) | | sample()  (in HybridTrajectory) | Setstate  (in PyDSTool.Toolbox.synthetic\_data) | spacing  (in PyDSTool.Toolbox.ModelHelper) | | sample()  (in Trajectory) | Setstate  (in PyDSTool.Toolbox.syntheticdata) | spacing  (in PyDSTool.Toolbox.NineML) | | sample\_x\_domain()  (in nullcline) | setTermFlag()  (in EventStruct) | spacing  (in PyDSTool.Toolbox.adjointPRC) | | save()  (in Pickler) | setTol()  (in Pointset) | spacing  (in PyDSTool.Toolbox.dataanalysis) | | save\_bool()  (in Pickler) | setUnitBounds()  (in DSSRT\_info) | spacing  (in PyDSTool.Toolbox.fracdim) | | save\_dict()  (in Pickler) | setup()  (in plotter\_2D) | spacing  (in PyDSTool.Toolbox.makeSloppyModel) | | save\_empty\_tuple()  (in Pickler) | setup\_conditions()  (in ModelInterface) | spacing  (in PyDSTool.Toolbox.neuralcomp) | | save\_fig()  (in PyDSTool.matplotlib\_import) | setup\_grad()  (in mesh\_patch\_2D) | spacing  (in PyDSTool.Toolbox.phaseplane) | | save\_float()  (in Pickler) | setVfCodeInsertEnd()  (in GeneratorConstructor) | spacing  (in PyDSTool.Toolbox.synthetic\_data) | | save\_global()  (in Pickler) | setVfCodeInsertStart()  (in GeneratorConstructor) | spacing  (in PyDSTool.Toolbox.syntheticdata) | | save\_inst()  (in Pickler) | SHIFT\_DIVIDEBYZERO  (in PyDSTool.PyCont.ContClass') | spacing  (in PyDSTool) | | save\_int()  (in Pickler) | SHIFT\_DIVIDEBYZERO  (in PyDSTool) | spacing  (in matplotlib.pylab) | | save\_list()  (in Pickler) | SHIFT\_DIVIDEBYZERO  (in PyDSTool.Toolbox.ActivationFuncs) | specialfns  (in PyDSTool.parseUtils) | | save\_long()  (in Pickler) | SHIFT\_DIVIDEBYZERO  (in PyDSTool.Toolbox.DSSRT\_tools) | spectral\_gaps\_absolute()  (in PyDSTool.Toolbox.dssrt) | | save\_none()  (in Pickler) | SHIFT\_DIVIDEBYZERO  (in PyDSTool.Toolbox.InputProfile) | spectral\_gaps\_relative()  (in PyDSTool.Toolbox.dssrt) | | save\_pers()  (in Pickler) | SHIFT\_DIVIDEBYZERO  (in PyDSTool.Toolbox.ModelHelper) | specTypes  (in PyDSTool.ModelSpec') | | save\_reduce()  (in Pickler) | SHIFT\_DIVIDEBYZERO  (in PyDSTool.Toolbox.NineML) | specTypes  (in PyDSTool.Symbolic) | | save\_string()  (in Pickler) | SHIFT\_DIVIDEBYZERO  (in PyDSTool.Toolbox.adjointPRC) | specTypes  (in PyDSTool.Toolbox.NineML) | | save\_tuple()  (in Pickler) | SHIFT\_DIVIDEBYZERO  (in PyDSTool.Toolbox.dataanalysis) | specTypes  (in PyDSTool.Toolbox.dataanalysis) | | save\_unicode()  (in Pickler) | SHIFT\_DIVIDEBYZERO  (in PyDSTool.Toolbox.fracdim) | specTypes  (in PyDSTool.Toolbox.phaseplane) | | saveObjects()  (in PyDSTool.utils) | SHIFT\_DIVIDEBYZERO  (in PyDSTool.Toolbox.makeSloppyModel) | specTypes  (in PyDSTool.Toolbox.synthetic\_data) | | saveSession()  (in PyDSTool) | SHIFT\_DIVIDEBYZERO  (in PyDSTool.Toolbox.neuralcomp) | specTypes  (in PyDSTool.Toolbox.syntheticdata) | | ScalarType  (in PyDSTool.PyCont.ContClass') | SHIFT\_DIVIDEBYZERO  (in PyDSTool.Toolbox.phaseplane) | SpiffyODEModel  (in PyDSTool.Toolbox.ModelHelper) | | ScalarType  (in PyDSTool) | SHIFT\_DIVIDEBYZERO  (in PyDSTool.Toolbox.synthetic\_data) | spike\_envelope  (in PyDSTool.Toolbox.neuro\_data) | | ScalarType  (in PyDSTool.Toolbox.ActivationFuncs) | SHIFT\_DIVIDEBYZERO  (in PyDSTool.Toolbox.syntheticdata) | spike\_feature  (in PyDSTool.Toolbox.neuro\_data) | | ScalarType  (in PyDSTool.Toolbox.DSSRT\_tools) | SHIFT\_DIVIDEBYZERO  (in matplotlib.pylab) | spike\_metric  (in PyDSTool.Toolbox.neuro\_data) | | ScalarType  (in PyDSTool.Toolbox.InputProfile) | SHIFT\_INVALID  (in PyDSTool.PyCont.ContClass') | split()  (in PyDSTool.Toolbox.dssrt) | | ScalarType  (in PyDSTool.Toolbox.ModelHelper) | SHIFT\_INVALID  (in PyDSTool) | split\_pts()  (in PyDSTool.Toolbox.dssrt) | | ScalarType  (in PyDSTool.Toolbox.NineML) | SHIFT\_INVALID  (in PyDSTool.Toolbox.ActivationFuncs) | splitargs()  (in PyDSTool.parseUtils) | | ScalarType  (in PyDSTool.Toolbox.adjointPRC) | SHIFT\_INVALID  (in PyDSTool.Toolbox.DSSRT\_tools) | splitastLR()  (in PyDSTool.parseUtils) | | ScalarType  (in PyDSTool.Toolbox.dataanalysis) | SHIFT\_INVALID  (in PyDSTool.Toolbox.InputProfile) | SPoint  (in PyDSTool.PyCont.BifPoint) | | ScalarType  (in PyDSTool.Toolbox.fracdim) | SHIFT\_INVALID  (in PyDSTool.Toolbox.ModelHelper) | Sqrt  (in PyDSTool.ModelSpec') | | ScalarType  (in PyDSTool.Toolbox.makeSloppyModel) | SHIFT\_INVALID  (in PyDSTool.Toolbox.NineML) | sqrt  (in PyDSTool.PyCont.ContClass') | | ScalarType  (in PyDSTool.Toolbox.neuralcomp) | SHIFT\_INVALID  (in PyDSTool.Toolbox.adjointPRC) | sqrt  (in PyDSTool.PyCont.misc) | | ScalarType  (in PyDSTool.Toolbox.phaseplane) | SHIFT\_INVALID  (in PyDSTool.Toolbox.dataanalysis) | Sqrt  (in PyDSTool) | | ScalarType  (in PyDSTool.Toolbox.synthetic\_data) | SHIFT\_INVALID  (in PyDSTool.Toolbox.fracdim) | Sqrt  (in PyDSTool.Symbolic) | | ScalarType  (in PyDSTool.Toolbox.syntheticdata) | SHIFT\_INVALID  (in PyDSTool.Toolbox.makeSloppyModel) | sqrt  (in PyDSTool.Symbolic) | | ScalarType  (in matplotlib.pylab) | SHIFT\_INVALID  (in PyDSTool.Toolbox.neuralcomp) | Sqrt  (in PyDSTool.Toolbox.ActivationFuncs) | | scaled\_line\_search  (in PyDSTool.Toolbox.optimizers.line\_search) | SHIFT\_INVALID  (in PyDSTool.Toolbox.phaseplane) | Sqrt  (in PyDSTool.Toolbox.DSSRT\_tools) | | ScaledLineSearch  (in PyDSTool.Toolbox.optimizers.line\_search.scaled\_line\_search) | SHIFT\_INVALID  (in PyDSTool.Toolbox.synthetic\_data) | Sqrt  (in PyDSTool.Toolbox.InputProfile) | | scatter\_histo()  (in PyDSTool.Toolbox.fracdim) | SHIFT\_INVALID  (in PyDSTool.Toolbox.syntheticdata) | Sqrt  (in PyDSTool.Toolbox.ModelHelper) | | scatterplot\_slopes()  (in PyDSTool.Toolbox.fracdim) | SHIFT\_INVALID  (in matplotlib.pylab) | Sqrt  (in PyDSTool.Toolbox.NineML) | | scipy\_ode  (in PyDSTool) | SHIFT\_OVERFLOW  (in PyDSTool.PyCont.ContClass') | sqrt  (in PyDSTool.Toolbox.NineML) | | Scorer  (in PyDSTool.Toolbox.dssrt) | SHIFT\_OVERFLOW  (in PyDSTool) | Sqrt  (in PyDSTool.Toolbox.adjointPRC) | | scorer()  (in EpochSeqScorer) | SHIFT\_OVERFLOW  (in PyDSTool.Toolbox.ActivationFuncs) | Sqrt  (in PyDSTool.Toolbox.dataanalysis) | | sctypeDict  (in PyDSTool.PyCont.ContClass') | SHIFT\_OVERFLOW  (in PyDSTool.Toolbox.DSSRT\_tools) | sqrt  (in PyDSTool.Toolbox.dataanalysis) | | sctypeDict  (in PyDSTool.Toolbox.ActivationFuncs) | SHIFT\_OVERFLOW  (in PyDSTool.Toolbox.InputProfile) | Sqrt  (in PyDSTool.Toolbox.fracdim) | | sctypeDict  (in PyDSTool.Toolbox.DSSRT\_tools) | SHIFT\_OVERFLOW  (in PyDSTool.Toolbox.ModelHelper) | Sqrt  (in PyDSTool.Toolbox.makeSloppyModel) | | sctypeDict  (in PyDSTool.Toolbox.InputProfile) | SHIFT\_OVERFLOW  (in PyDSTool.Toolbox.NineML) | Sqrt  (in PyDSTool.Toolbox.neuralcomp) | | sctypeDict  (in PyDSTool.Toolbox.ModelHelper) | SHIFT\_OVERFLOW  (in PyDSTool.Toolbox.adjointPRC) | Sqrt  (in PyDSTool.Toolbox.phaseplane) | | sctypeDict  (in PyDSTool.Toolbox.NineML) | SHIFT\_OVERFLOW  (in PyDSTool.Toolbox.dataanalysis) | sqrt  (in PyDSTool.Toolbox.phaseplane) | | sctypeDict  (in PyDSTool.Toolbox.adjointPRC) | SHIFT\_OVERFLOW  (in PyDSTool.Toolbox.fracdim) | Sqrt  (in PyDSTool.Toolbox.synthetic\_data) | | sctypeDict  (in PyDSTool.Toolbox.dataanalysis) | SHIFT\_OVERFLOW  (in PyDSTool.Toolbox.makeSloppyModel) | sqrt  (in PyDSTool.Toolbox.synthetic\_data) | | sctypeDict  (in PyDSTool.Toolbox.fracdim) | SHIFT\_OVERFLOW  (in PyDSTool.Toolbox.neuralcomp) | Sqrt  (in PyDSTool.Toolbox.syntheticdata) | | sctypeDict  (in PyDSTool.Toolbox.makeSloppyModel) | SHIFT\_OVERFLOW  (in PyDSTool.Toolbox.phaseplane) | sqrt  (in PyDSTool.Toolbox.syntheticdata) | | sctypeDict  (in PyDSTool.Toolbox.neuralcomp) | SHIFT\_OVERFLOW  (in PyDSTool.Toolbox.synthetic\_data) | sqrt  (in matplotlib.pylab) | | sctypeDict  (in PyDSTool.Toolbox.phaseplane) | SHIFT\_OVERFLOW  (in PyDSTool.Toolbox.syntheticdata) | square  (in PyDSTool.PyCont.ContClass') | | sctypeDict  (in PyDSTool.Toolbox.synthetic\_data) | SHIFT\_OVERFLOW  (in matplotlib.pylab) | square  (in PyDSTool.Toolbox.ActivationFuncs) | | sctypeDict  (in PyDSTool.Toolbox.syntheticdata) | SHIFT\_UNDERFLOW  (in PyDSTool.PyCont.ContClass') | square  (in PyDSTool.Toolbox.DSSRT\_tools) | | sctypeDict  (in PyDSTool) | SHIFT\_UNDERFLOW  (in PyDSTool) | square  (in PyDSTool.Toolbox.InputProfile) | | sctypeDict  (in matplotlib.pylab) | SHIFT\_UNDERFLOW  (in PyDSTool.Toolbox.ActivationFuncs) | square  (in PyDSTool.Toolbox.ModelHelper) | | sctypeNA  (in PyDSTool.PyCont.ContClass') | SHIFT\_UNDERFLOW  (in PyDSTool.Toolbox.DSSRT\_tools) | square  (in PyDSTool.Toolbox.NineML) | | sctypeNA  (in PyDSTool.Toolbox.ActivationFuncs) | SHIFT\_UNDERFLOW  (in PyDSTool.Toolbox.InputProfile) | square  (in PyDSTool.Toolbox.adjointPRC) | | sctypeNA  (in PyDSTool.Toolbox.DSSRT\_tools) | SHIFT\_UNDERFLOW  (in PyDSTool.Toolbox.ModelHelper) | square  (in PyDSTool.Toolbox.dataanalysis) | | sctypeNA  (in PyDSTool.Toolbox.InputProfile) | SHIFT\_UNDERFLOW  (in PyDSTool.Toolbox.NineML) | square  (in PyDSTool.Toolbox.fracdim) | | sctypeNA  (in PyDSTool.Toolbox.ModelHelper) | SHIFT\_UNDERFLOW  (in PyDSTool.Toolbox.adjointPRC) | square  (in PyDSTool.Toolbox.makeSloppyModel) | | sctypeNA  (in PyDSTool.Toolbox.NineML) | SHIFT\_UNDERFLOW  (in PyDSTool.Toolbox.dataanalysis) | square  (in PyDSTool.Toolbox.neuralcomp) | | sctypeNA  (in PyDSTool.Toolbox.adjointPRC) | SHIFT\_UNDERFLOW  (in PyDSTool.Toolbox.fracdim) | square  (in PyDSTool.Toolbox.phaseplane) | | sctypeNA  (in PyDSTool.Toolbox.dataanalysis) | SHIFT\_UNDERFLOW  (in PyDSTool.Toolbox.makeSloppyModel) | square  (in PyDSTool.Toolbox.synthetic\_data) | | sctypeNA  (in PyDSTool.Toolbox.fracdim) | SHIFT\_UNDERFLOW  (in PyDSTool.Toolbox.neuralcomp) | square  (in PyDSTool.Toolbox.syntheticdata) | | sctypeNA  (in PyDSTool.Toolbox.makeSloppyModel) | SHIFT\_UNDERFLOW  (in PyDSTool.Toolbox.phaseplane) | square  (in PyDSTool) | | sctypeNA  (in PyDSTool.Toolbox.neuralcomp) | SHIFT\_UNDERFLOW  (in PyDSTool.Toolbox.synthetic\_data) | square  (in matplotlib.pylab) | | sctypeNA  (in PyDSTool.Toolbox.phaseplane) | SHIFT\_UNDERFLOW  (in PyDSTool.Toolbox.syntheticdata) | stab\_line\_styles  (in PyDSTool.PyCont.Continuation) | | sctypeNA  (in PyDSTool.Toolbox.synthetic\_data) | SHIFT\_UNDERFLOW  (in matplotlib.pylab) | standard\_optimizer  (in PyDSTool.Toolbox.optimizers.optimizer) | | sctypeNA  (in PyDSTool.Toolbox.syntheticdata) | SHORT\_BINSTRING  (in PyDSTool.fixedpickle) | standard\_optimizer\_modifying  (in PyDSTool.Toolbox.optimizers.optimizer) | | sctypeNA  (in PyDSTool) | show\_epochs()  (in PyDSTool.Toolbox.dssrt) | StandardOptimizer  (in PyDSTool.Toolbox.optimizers.optimizer.standard\_optimizer) | | sctypeNA  (in matplotlib.pylab) | show\_log\_record()  (in ParamEst) | StandardOptimizerModifying  (in PyDSTool.Toolbox.optimizers.optimizer.standard\_optimizer\_modifying) | | sctypes  (in PyDSTool.PyCont.ContClass') | show\_PPs()  (in PyDSTool.Toolbox.phaseplane) | start()  (in Redirector) | | sctypes  (in PyDSTool.Toolbox.ActivationFuncs) | show\_res\_info()  (in context) | std()  (in data\_bins) | | sctypes  (in PyDSTool.Toolbox.DSSRT\_tools) | showAuxFnSpec()  (in Generator) | std()  (in data\_bins) | | sctypes  (in PyDSTool.Toolbox.InputProfile) | showAuxFnSpec()  (in Model) | STDERR  (in PyDSTool.Redirector) | | sctypes  (in PyDSTool.Toolbox.ModelHelper) | showAuxSpec()  (in Generator) | STDOUT  (in PyDSTool.Redirector) | | sctypes  (in PyDSTool.Toolbox.NineML) | showAuxSpec()  (in Model) | step  (in PyDSTool.Toolbox.optimizers) | | sctypes  (in PyDSTool.Toolbox.adjointPRC) | showDef()  (in Model) | step()  (in IntegratorBase) | | sctypes  (in PyDSTool.Toolbox.dataanalysis) | showDSEventInfo()  (in Model) | step()  (in vode) | | sctypes  (in PyDSTool.Toolbox.fracdim) | showErrors()  (in Diagnostics) | step\_\_all\_\_  (in PyDSTool.Toolbox.optimizers.step) | | sctypes  (in PyDSTool.Toolbox.makeSloppyModel) | showEventSpec()  (in Generator) | STOP  (in PyDSTool.fixedpickle) | | sctypes  (in PyDSTool.Toolbox.neuralcomp) | showEventSpec()  (in Model) | stop()  (in Redirector) | | sctypes  (in PyDSTool.Toolbox.phaseplane) | showRegimes()  (in HybridTrajectory) | strIfSeq()  (in PyDSTool.Symbolic) | | sctypes  (in PyDSTool.Toolbox.synthetic\_data) | showSpec()  (in Generator) | STRING  (in PyDSTool.fixedpickle) | | sctypes  (in PyDSTool.Toolbox.syntheticdata) | showSpec()  (in Model) | string2ast()  (in PyDSTool.parseUtils) | | sctypes  (in PyDSTool) | showWarnings()  (in Diagnostics) | strip\_speed()  (in PyDSTool.Toolbox.dssrt) | | sctypes  (in matplotlib.pylab) | Shuffle  (in PyDSTool.ModelSpec') | strong\_wolfe\_powell\_rule  (in PyDSTool.Toolbox.optimizers.line\_search) | | search()  (in GDescriptor) | Shuffle  (in PyDSTool) | StrongWolfePowellRule  (in PyDSTool.Toolbox.optimizers.line\_search.strong\_wolfe\_powell\_rule) | | search()  (in ModelSpec) | Shuffle  (in PyDSTool.Symbolic) | Struct  (in PyDSTool.PyCont.misc) | | search\_both()  (in distance\_to\_pointset) | Shuffle  (in PyDSTool.Toolbox.ActivationFuncs) | Struct  (in PyDSTool.common) | | search\_max()  (in distance\_to\_pointset) | Shuffle  (in PyDSTool.Toolbox.DSSRT\_tools) | SU  (in PyDSTool.PyCont.ContClass') | | search\_min()  (in distance\_to\_pointset) | Shuffle  (in PyDSTool.Toolbox.InputProfile) | SU  (in matplotlib.pylab) | | searchForEvents()  (in Event) | Shuffle  (in PyDSTool.Toolbox.ModelHelper) | sub\_models()  (in Model) | | searchForNames()  (in Model) | Shuffle  (in PyDSTool.Toolbox.NineML) | subs()  (in PyDSTool.Symbolic) | | searchForVars()  (in Model) | Shuffle  (in PyDSTool.Toolbox.adjointPRC) | subtract  (in PyDSTool.PyCont.ContClass') | | searchModelSpec()  (in PyDSTool.ModelSpec') | Shuffle  (in PyDSTool.Toolbox.dataanalysis) | subtract  (in PyDSTool.PyCont.Continuation) | | second\_diff()  (in PyDSTool.Toolbox.data\_analysis) | Shuffle  (in PyDSTool.Toolbox.fracdim) | subtract  (in PyDSTool.PyCont.misc) | | second\_diff()  (in PyDSTool.Toolbox.dataanalysis) | Shuffle  (in PyDSTool.Toolbox.makeSloppyModel) | subtract  (in PyDSTool.Toolbox.ActivationFuncs) | | SECONDLY  (in PyDSTool.PyCont.ContClass') | Shuffle  (in PyDSTool.Toolbox.neuralcomp) | subtract  (in PyDSTool.Toolbox.DSSRT\_tools) | | SECONDLY  (in matplotlib.pylab) | Shuffle  (in PyDSTool.Toolbox.phaseplane) | subtract  (in PyDSTool.Toolbox.InputProfile) | | Seed  (in PyDSTool.ModelSpec') | Shuffle  (in PyDSTool.Toolbox.synthetic\_data) | subtract  (in PyDSTool.Toolbox.ModelHelper) | | Seed  (in PyDSTool) | Shuffle  (in PyDSTool.Toolbox.syntheticdata) | subtract  (in PyDSTool.Toolbox.NineML) | | Seed  (in PyDSTool.Symbolic) | Sigma()  (in PyDSTool.Toolbox.ActivationFuncs) | subtract  (in PyDSTool.Toolbox.adjointPRC) | | Seed  (in PyDSTool.Toolbox.ActivationFuncs) | Sigma2()  (in PyDSTool.Toolbox.ActivationFuncs) | subtract  (in PyDSTool.Toolbox.dataanalysis) | | Seed  (in PyDSTool.Toolbox.DSSRT\_tools) | SigmaNeg()  (in PyDSTool.Toolbox.ActivationFuncs) | subtract  (in PyDSTool.Toolbox.fracdim) | | Seed  (in PyDSTool.Toolbox.InputProfile) | SigmaV()  (in PyDSTool.Toolbox.ActivationFuncs) | subtract  (in PyDSTool.Toolbox.makeSloppyModel) | | Seed  (in PyDSTool.Toolbox.ModelHelper) | sign  (in PyDSTool.Generator.Euler\_ODEsystem') | subtract  (in PyDSTool.Toolbox.neuralcomp) | | Seed  (in PyDSTool.Toolbox.NineML) | sign  (in PyDSTool.Generator.Vode\_ODEsystem') | subtract  (in PyDSTool.Toolbox.phaseplane) | | Seed  (in PyDSTool.Toolbox.adjointPRC) | sign  (in PyDSTool.Interval') | subtract  (in PyDSTool.Toolbox.synthetic\_data) | | Seed  (in PyDSTool.Toolbox.dataanalysis) | sign  (in PyDSTool.PyCont.ContClass') | subtract  (in PyDSTool.Toolbox.syntheticdata) | | Seed  (in PyDSTool.Toolbox.fracdim) | sign  (in PyDSTool.PyCont.Continuation) | subtract  (in PyDSTool) | | Seed  (in PyDSTool.Toolbox.makeSloppyModel) | sign  (in PyDSTool.PyCont.misc) | subtract  (in matplotlib.pylab) | | Seed  (in PyDSTool.Toolbox.neuralcomp) | sign  (in PyDSTool.Toolbox.NineML) | success  (in IntegratorBase) | | Seed  (in PyDSTool.Toolbox.phaseplane) | sign  (in PyDSTool.Toolbox.ParamEst) | successful()  (in euler\_solver) | | Seed  (in PyDSTool.Toolbox.synthetic\_data) | sign  (in PyDSTool.Toolbox.PySCes\_SBML) | successful()  (in ode) | | Seed  (in PyDSTool.Toolbox.syntheticdata) | sign  (in PyDSTool.Toolbox.dataanalysis) | Sum  (in PyDSTool.ModelSpec') | | select\_pars\_for\_features()  (in PyDSTool.Toolbox.ParamEst) | sign  (in PyDSTool.Toolbox.phaseplane) | Sum  (in PyDSTool) | | seq\_str()  (in epoch) | sign  (in PyDSTool.Toolbox.synthetic\_data) | Sum  (in PyDSTool.Symbolic) | | sequences\_to\_eventlist()  (in PyDSTool.Toolbox.event\_driven\_simulator) | sign  (in PyDSTool.Toolbox.syntheticdata) | Sum  (in PyDSTool.Toolbox.ActivationFuncs) | | set()  (in EmbeddedSysGen) | sign  (in PyDSTool.common) | Sum  (in PyDSTool.Toolbox.DSSRT\_tools) | | set()  (in ExplicitFnGen) | sign  (in matplotlib.pylab) | Sum  (in PyDSTool.Toolbox.InputProfile) | | set()  (in ExtrapolateTable) | signbit  (in PyDSTool.PyCont.ContClass') | Sum  (in PyDSTool.Toolbox.ModelHelper) | | set()  (in ImplicitFnGen) | signbit  (in PyDSTool.Toolbox.ActivationFuncs) | Sum  (in PyDSTool.Toolbox.NineML) | | set()  (in InterpolateTable) | signbit  (in PyDSTool.Toolbox.DSSRT\_tools) | Sum  (in PyDSTool.Toolbox.adjointPRC) | | set()  (in MapSystem) | signbit  (in PyDSTool.Toolbox.InputProfile) | Sum  (in PyDSTool.Toolbox.dataanalysis) | | set()  (in ODEsystem) | signbit  (in PyDSTool.Toolbox.ModelHelper) | Sum  (in PyDSTool.Toolbox.fracdim) | | set()  (in Generator) | signbit  (in PyDSTool.Toolbox.NineML) | Sum  (in PyDSTool.Toolbox.makeSloppyModel) | | set()  (in Interval) | signbit  (in PyDSTool.Toolbox.adjointPRC) | Sum  (in PyDSTool.Toolbox.neuralcomp) | | set()  (in GeneratorInterface) | signbit  (in PyDSTool.Toolbox.dataanalysis) | Sum  (in PyDSTool.Toolbox.phaseplane) | | set()  (in ModelInterface) | signbit  (in PyDSTool.Toolbox.fracdim) | Sum  (in PyDSTool.Toolbox.synthetic\_data) | | set()  (in Model) | signbit  (in PyDSTool.Toolbox.makeSloppyModel) | Sum  (in PyDSTool.Toolbox.syntheticdata) | | set\_curr\_fig()  (in plotter\_2D) | signbit  (in PyDSTool.Toolbox.neuralcomp) | sup\_norm()  (in PyDSTool.Toolbox.synthetic\_data) | | set\_default\_transition()  (in FSM) | signbit  (in PyDSTool.Toolbox.phaseplane) | sup\_norm()  (in PyDSTool.Toolbox.syntheticdata) | | set\_f\_params()  (in euler\_solver) | signbit  (in PyDSTool.Toolbox.synthetic\_data) | supports\_run\_relax  (in IntegratorBase) | | set\_f\_params()  (in ode) | signbit  (in PyDSTool.Toolbox.syntheticdata) | supports\_run\_relax  (in vode) | | set\_initial\_value()  (in euler\_solver) | signbit  (in PyDSTool) | supports\_step  (in IntegratorBase) | | set\_initial\_value()  (in ode) | signbit  (in matplotlib.pylab) | supports\_step  (in vode) | | set\_integrator()  (in ode) | simple\_bisection()  (in PyDSTool.common) | swdist()  (in PyDSTool.Toolbox.dssrt) | | set\_jac\_params()  (in euler\_solver) | simple\_line\_search  (in PyDSTool.Toolbox.optimizers.line\_search) | sweep1D()  (in PyDSTool.Toolbox.ParamEst) | | set\_jac\_params()  (in ode) | SimpleLineSearch  (in PyDSTool.Toolbox.optimizers.line\_search.simple\_line\_search) | sym2name()  (in PyDSTool.parseUtils) | | set\_node\_state()  (in simulator) | simplify()  (in QuantSpec) | symb  (in PyDSTool.Symbolic) | | set\_ref\_traj()  (in condition) | simplify()  (in Quantity) | symbol\_map  (in PyDSTool.Toolbox.prep\_boxplot) | | set\_ref\_traj()  (in feature) | simplify()  (in PyDSTool.parseUtils) | Symbolic  (in PyDSTool) | | set\_ref\_traj()  (in feature\_leaf) | simplify\_str()  (in PyDSTool.parseUtils) | symbolMapClass  (in PyDSTool.parseUtils) | | set\_ref\_traj()  (in feature\_node) | simplifyMatrixRepr()  (in PyDSTool.common) | symbolMapDict  (in PyDSTool.Generator.ADMC\_ODEsystem') | | set\_single\_feat\_weights()  (in context) | simulator  (in PyDSTool.Toolbox.event\_driven\_simulator) | symbolMapDict  (in PyDSTool.Generator.Dopri\_ODEsystem') | | set\_test\_traj()  (in extModelInterface) | Sin  (in PyDSTool.ModelSpec') | symbolMapDict  (in PyDSTool.Generator.EmbeddedSysGen') | | set\_weights()  (in context) | sin  (in PyDSTool.PyCont.ContClass') | symbolMapDict  (in PyDSTool.Generator.Euler\_ODEsystem') | | set\_yi()  (in BarycentricInterpolator) | Sin  (in PyDSTool) | symbolMapDict  (in PyDSTool.Generator.ExplicitFnGen') | | setActiveFlag()  (in EventStruct) | Sin  (in PyDSTool.Symbolic) | symbolMapDict  (in PyDSTool.Generator.ExtrapolateTable') | | setAlgParams()  (in LMpest) | sin  (in PyDSTool.Symbolic) | symbolMapDict  (in PyDSTool.Generator.ImplicitFnGen') | | setAlgParams()  (in ParamEst) | Sin  (in PyDSTool.Toolbox.ActivationFuncs) | symbolMapDict  (in PyDSTool.Generator.InterpolateTable') | | setBisect()  (in EventStruct) | Sin  (in PyDSTool.Toolbox.DSSRT\_tools) | symbolMapDict  (in PyDSTool.Generator.LookupTable') | | setContParams()  (in integrator) | Sin  (in PyDSTool.Toolbox.InputProfile) | symbolMapDict  (in PyDSTool.Generator.MapSystem') | | setdata()  (in AddTestFunction) | Sin  (in PyDSTool.Toolbox.ModelHelper) | symbolMapDict  (in PyDSTool.Generator.Radau\_ODEsystem') | | setdata()  (in BorderMethod) | Sin  (in PyDSTool.Toolbox.NineML) | symbolMapDict  (in PyDSTool.Generator.Vode\_ODEsystem') | | setdata()  (in Branch\_Bor) | sin  (in PyDSTool.Toolbox.NineML) | symbolMapDict  (in PyDSTool.Toolbox.NineML) | | setdata()  (in Fold\_Bor) | Sin  (in PyDSTool.Toolbox.adjointPRC) | symbolMapDict  (in PyDSTool.Toolbox.dataanalysis) | | setdata()  (in Hopf\_Bor) | Sin  (in PyDSTool.Toolbox.dataanalysis) | symbolMapDict  (in PyDSTool.Toolbox.phaseplane) | | setdata()  (in Hopf\_Double\_Bor\_One) | sin  (in PyDSTool.Toolbox.dataanalysis) | symbolMapDict  (in PyDSTool.Toolbox.synthetic\_data) | | setdata()  (in Hopf\_Double\_Bor\_Two) | Sin  (in PyDSTool.Toolbox.fracdim) | symbolMapDict  (in PyDSTool.Toolbox.syntheticdata) | | setdata()  (in UserDefinedTestFunc) | Sin  (in PyDSTool.Toolbox.makeSloppyModel) | syms  (in PyDSTool.FuncSpec') | | setdefault()  (in args) | Sin  (in PyDSTool.Toolbox.neuralcomp) | syms  (in PyDSTool.ModelSpec') | | setDepdomain()  (in Variable) | Sin  (in PyDSTool.Toolbox.phaseplane) | syms  (in PyDSTool.Symbolic) | | setDomain()  (in Quantity) | sin  (in PyDSTool.Toolbox.phaseplane) | syms  (in PyDSTool.Trajectory') | | setDopriParams()  (in dopri) | Sin  (in PyDSTool.Toolbox.synthetic\_data) | syms  (in PyDSTool.parseUtils) | | setDSAlgPars()  (in Model) | sin  (in PyDSTool.Toolbox.synthetic\_data) | synapse  (in PyDSTool.Toolbox.neuralcomp) | | setDSEventActive()  (in Model) | Sin  (in PyDSTool.Toolbox.syntheticdata) | synthetic\_data  (in PyDSTool.Toolbox) | | setDSEventBisect()  (in Model) | sin  (in PyDSTool.Toolbox.syntheticdata) | syntheticdata  (in PyDSTool.Toolbox) | | setDSEventDelay()  (in Model) | sin  (in matplotlib.pylab) | Systemrandom  (in PyDSTool.ModelSpec') | | setDSEventDir()  (in Model) | Sinh  (in PyDSTool.ModelSpec') | Systemrandom  (in PyDSTool.Symbolic) | | setDSEventICs()  (in Model) | sinh  (in PyDSTool.PyCont.ContClass') | Systemrandom  (in PyDSTool) | | setDSEventInterval()  (in Model) | Sinh  (in PyDSTool) | Systemrandom  (in PyDSTool.Toolbox.ActivationFuncs) | | setDSEventPrecise()  (in Model) | Sinh  (in PyDSTool.Symbolic) | Systemrandom  (in PyDSTool.Toolbox.DSSRT\_tools) | | setDSEventStartTime()  (in Model) | sinh  (in PyDSTool.Symbolic) | Systemrandom  (in PyDSTool.Toolbox.InputProfile) | | setDSEventTerm()  (in Model) | Sinh  (in PyDSTool.Toolbox.ActivationFuncs) | Systemrandom  (in PyDSTool.Toolbox.ModelHelper) | | setDSEventTol()  (in Model) | Sinh  (in PyDSTool.Toolbox.DSSRT\_tools) | Systemrandom  (in PyDSTool.Toolbox.NineML) | | setEventDelay()  (in EventStruct) | Sinh  (in PyDSTool.Toolbox.InputProfile) | Systemrandom  (in PyDSTool.Toolbox.adjointPRC) | | setEventDir()  (in EventStruct) | Sinh  (in PyDSTool.Toolbox.ModelHelper) | Systemrandom  (in PyDSTool.Toolbox.dataanalysis) | | setEventICs()  (in EventStruct) | Sinh  (in PyDSTool.Toolbox.NineML) | Systemrandom  (in PyDSTool.Toolbox.fracdim) | | setEventICs()  (in Generator) | sinh  (in PyDSTool.Toolbox.NineML) | Systemrandom  (in PyDSTool.Toolbox.makeSloppyModel) | | setEventInterval()  (in EventStruct) | Sinh  (in PyDSTool.Toolbox.adjointPRC) | Systemrandom  (in PyDSTool.Toolbox.neuralcomp) | | setEvents()  (in integrator) | Sinh  (in PyDSTool.Toolbox.dataanalysis) | Systemrandom  (in PyDSTool.Toolbox.phaseplane) | | setEventTol()  (in EventStruct) | sinh  (in PyDSTool.Toolbox.dataanalysis) | Systemrandom  (in PyDSTool.Toolbox.synthetic\_data) | | setExtInputs()  (in integrator) | Sinh  (in PyDSTool.Toolbox.fracdim) | Systemrandom  (in PyDSTool.Toolbox.syntheticdata) | |

  
  

| Home | Trees | Indices | Help | | PyDSTool | | --- | |
| --- | --- | --- | --- | --- | --- |

|  |  |
| --- | --- |
| Generated by Epydoc 3.0.1 on Fri May 4 15:23:58 2012 | http://epydoc.sourceforge.net |
