# Supplementary material for: Hybrid Models and Biological Model Reduction with PyDSTool
Source: PLoS Comput Biol. 2012 Aug 9;8(8):e1002628. doi: 10.1371/journal.pcbi.1002628 (PMC3415397; doi:10.1371/journal.pcbi.1002628)
Supplement: Text S4 — Complete source code for the PyDSTool package (version 0.88.120504). Includes API documentation and help files linking to web pages. This file is identical to the current public release on Sourceforge.net. (ZIP) [file pcbi.1002628.s004.zip › PyDSTool/html/identifier-index-T.html]

xml version="1.0" encoding="ascii"?


Identifier Index


| Home | Trees | Indices | Help | | PyDSTool | | --- | |
| --- | --- | --- | --- | --- | --- |

|  |  |  |  |
| --- | --- | --- | --- |
|  | |  | | --- | | [hide private] | | [frames] | no frames] | |

|  |  |
| --- | --- |
| Identifier Index | [ A B C D E F G H I J K L M N O P Q R S T U V W X Y Z \_ ] |

|  |  |  |  |  |  |  |  |  |  |  |  |  |  |  |  |  |  |  |  |  |  |  |  |  |  |  |  |  |  |  |  |  |  |  |  |  |  |  |  |  |  |  |  |  |  |  |  |  |  |  |  |  |  |  |  |  |  |  |  |  |  |  |  |  |  |  |  |  |  |  |  |  |  |  |  |  |  |  |  |  |  |  |  |  |  |  |  |  |  |  |  |  |  |  |  |  |  |  |  |  |  |  |  |  |  |  |  |  |  |  |  |  |  |  |  |  |  |  |  |  |  |  |  |  |  |  |  |  |  |  |  |  |  |  |  |  |  |  |  |  |  |  |  |  |  |  |  |  |  |  |  |  |  |  |  |  |  |  |  |  |  |  |  |  |  |  |  |  |  |  |  |  |  |  |  |  |  |  |  |  |  |  |  |  |  |  |  |  |  |  |  |  |  |  |  |  |  |  |  |  |  |  |  |  |  |  |  |  |  |  |  |  |  |  |  |  |  |  |  |  |  |  |  |  |  |  |  |  |  |  |  |  |  |  |  |  |  |  |  |  |  |  |  |  |  |  |  |  |  |  |  |  |  |  |  |  |  |  |  |  |  |  |  |  |  |  |  |  |  |  |  |  |  |  |  |  |  |  |  |  |  |  |  |  |  |  |  |  |  |  |  |  |  |  |  |  |  |  |
| --- | --- | --- | --- | --- | --- | --- | --- | --- | --- | --- | --- | --- | --- | --- | --- | --- | --- | --- | --- | --- | --- | --- | --- | --- | --- | --- | --- | --- | --- | --- | --- | --- | --- | --- | --- | --- | --- | --- | --- | --- | --- | --- | --- | --- | --- | --- | --- | --- | --- | --- | --- | --- | --- | --- | --- | --- | --- | --- | --- | --- | --- | --- | --- | --- | --- | --- | --- | --- | --- | --- | --- | --- | --- | --- | --- | --- | --- | --- | --- | --- | --- | --- | --- | --- | --- | --- | --- | --- | --- | --- | --- | --- | --- | --- | --- | --- | --- | --- | --- | --- | --- | --- | --- | --- | --- | --- | --- | --- | --- | --- | --- | --- | --- | --- | --- | --- | --- | --- | --- | --- | --- | --- | --- | --- | --- | --- | --- | --- | --- | --- | --- | --- | --- | --- | --- | --- | --- | --- | --- | --- | --- | --- | --- | --- | --- | --- | --- | --- | --- | --- | --- | --- | --- | --- | --- | --- | --- | --- | --- | --- | --- | --- | --- | --- | --- | --- | --- | --- | --- | --- | --- | --- | --- | --- | --- | --- | --- | --- | --- | --- | --- | --- | --- | --- | --- | --- | --- | --- | --- | --- | --- | --- | --- | --- | --- | --- | --- | --- | --- | --- | --- | --- | --- | --- | --- | --- | --- | --- | --- | --- | --- | --- | --- | --- | --- | --- | --- | --- | --- | --- | --- | --- | --- | --- | --- | --- | --- | --- | --- | --- | --- | --- | --- | --- | --- | --- | --- | --- | --- | --- | --- | --- | --- | --- | --- | --- | --- | --- | --- | --- | --- | --- | --- | --- | --- | --- | --- | --- | --- | --- | --- | --- | --- | --- | --- | --- | --- | --- | --- | --- | --- | --- | --- | --- | --- | --- | --- | --- | --- | --- | --- | --- | --- | --- | --- | --- | --- | --- | --- | --- | --- | --- | --- | --- | --- | --- | --- | --- |
| T | |  |  |  | | --- | --- | --- | | t  (in PyDSTool.Toolbox.ActivationFuncs) | targetLangs  (in PyDSTool.utils) | True\_  (in PyDSTool.Toolbox.syntheticdata) | | t  (in PyDSTool.Toolbox.DSSRT\_tools) | tarray  (in PyDSTool.Toolbox.FR) | True\_  (in PyDSTool) | | t  (in PyDSTool.Toolbox.InputProfile) | teardown()  (in plotter\_2D) | True\_  (in matplotlib.pylab) | | t  (in PyDSTool.Toolbox.ModelHelper) | temp\_macro\_names()  (in PyDSTool.parseUtils) | true\_divide  (in PyDSTool.PyCont.ContClass') | | t  (in PyDSTool.Toolbox.NineML) | temp\_macro\_names\_inv()  (in PyDSTool.parseUtils) | true\_divide  (in PyDSTool.Toolbox.ActivationFuncs) | | t  (in PyDSTool.Toolbox.adjointPRC) | templates\_path  (in PyDSTool.conf) | true\_divide  (in PyDSTool.Toolbox.DSSRT\_tools) | | t  (in PyDSTool.Toolbox.dataanalysis) | TENS  (in PyDSTool.parseUtils) | true\_divide  (in PyDSTool.Toolbox.InputProfile) | | t  (in PyDSTool.Toolbox.fracdim) | test()  (in PyDSTool.Toolbox.optimizers) | true\_divide  (in PyDSTool.Toolbox.ModelHelper) | | t  (in PyDSTool.Toolbox.makeSloppyModel) | test1()  (in PyDSTool.scipy\_ode) | true\_divide  (in PyDSTool.Toolbox.NineML) | | t  (in PyDSTool.Toolbox.neuralcomp) | test2()  (in PyDSTool.scipy\_ode) | true\_divide  (in PyDSTool.Toolbox.adjointPRC) | | t  (in PyDSTool.Toolbox.phaseplane) | test\_powell  (in PyDSTool.Toolbox.optimizers.tests) | true\_divide  (in PyDSTool.Toolbox.dataanalysis) | | t  (in PyDSTool.Toolbox.synthetic\_data) | test\_Powell  (in PyDSTool.Toolbox.optimizers.tests.test\_powell) | true\_divide  (in PyDSTool.Toolbox.fracdim) | | t  (in PyDSTool.Toolbox.syntheticdata) | test\_protocols  (in PyDSTool.Toolbox) | true\_divide  (in PyDSTool.Toolbox.makeSloppyModel) | | t  (in PyDSTool) | test\_quadratic  (in PyDSTool.Toolbox.optimizers.tests) | true\_divide  (in PyDSTool.Toolbox.neuralcomp) | | tabulate\_epoch\_seqs()  (in PyDSTool.Toolbox.dssrt) | test\_Quadratic  (in PyDSTool.Toolbox.optimizers.tests.test\_quadratic) | true\_divide  (in PyDSTool.Toolbox.phaseplane) | | tail()  (in PyDSTool.Toolbox.event\_driven\_simulator) | test\_rosenbrock  (in PyDSTool.Toolbox.optimizers.tests) | true\_divide  (in PyDSTool.Toolbox.synthetic\_data) | | Tan  (in PyDSTool.ModelSpec') | test\_Rosenbrock  (in PyDSTool.Toolbox.optimizers.tests.test\_rosenbrock) | true\_divide  (in PyDSTool.Toolbox.syntheticdata) | | tan  (in PyDSTool.PyCont.ContClass') | testall()  (in PyDSTool.Toolbox.optimizers) | true\_divide  (in PyDSTool) | | Tan  (in PyDSTool.Symbolic) | testdomain()  (in Continuation) | true\_divide  (in matplotlib.pylab) | | tan  (in PyDSTool.Symbolic) | testdomaingrid()  (in Continuation) | trunc  (in PyDSTool.PyCont.ContClass') | | Tan  (in PyDSTool) | testdomaintangrid()  (in Continuation) | trunc  (in PyDSTool.Toolbox.ActivationFuncs) | | Tan  (in PyDSTool.Toolbox.ActivationFuncs) | TestFunc  (in PyDSTool.PyCont) | trunc  (in PyDSTool.Toolbox.DSSRT\_tools) | | Tan  (in PyDSTool.Toolbox.DSSRT\_tools) | TestFunc  (in PyDSTool.PyCont.TestFunc) | trunc  (in PyDSTool.Toolbox.InputProfile) | | Tan  (in PyDSTool.Toolbox.InputProfile) | testindij()  (in PyDSTool.PyCont.misc) | trunc  (in PyDSTool.Toolbox.ModelHelper) | | Tan  (in PyDSTool.Toolbox.ModelHelper) | tests  (in PyDSTool.Toolbox.optimizers) | trunc  (in PyDSTool.Toolbox.NineML) | | Tan  (in PyDSTool.Toolbox.NineML) | tgt\_vec()  (in nullcline) | trunc  (in PyDSTool.Toolbox.adjointPRC) | | tan  (in PyDSTool.Toolbox.NineML) | TH  (in PyDSTool.PyCont.ContClass') | trunc  (in PyDSTool.Toolbox.dataanalysis) | | Tan  (in PyDSTool.Toolbox.adjointPRC) | TH  (in matplotlib.pylab) | trunc  (in PyDSTool.Toolbox.fracdim) | | Tan  (in PyDSTool.Toolbox.dataanalysis) | theGenSpecHelper  (in PyDSTool.Generator.ADMC\_ODEsystem') | trunc  (in PyDSTool.Toolbox.makeSloppyModel) | | tan  (in PyDSTool.Toolbox.dataanalysis) | theGenSpecHelper  (in PyDSTool.Generator.Dopri\_ODEsystem') | trunc  (in PyDSTool.Toolbox.neuralcomp) | | Tan  (in PyDSTool.Toolbox.fracdim) | theGenSpecHelper  (in PyDSTool.Generator.EmbeddedSysGen') | trunc  (in PyDSTool.Toolbox.phaseplane) | | Tan  (in PyDSTool.Toolbox.makeSloppyModel) | theGenSpecHelper  (in PyDSTool.Generator.Euler\_ODEsystem') | trunc  (in PyDSTool.Toolbox.synthetic\_data) | | Tan  (in PyDSTool.Toolbox.neuralcomp) | theGenSpecHelper  (in PyDSTool.Generator.ExplicitFnGen') | trunc  (in PyDSTool.Toolbox.syntheticdata) | | Tan  (in PyDSTool.Toolbox.phaseplane) | theGenSpecHelper  (in PyDSTool.Generator.ExtrapolateTable') | trunc  (in PyDSTool) | | tan  (in PyDSTool.Toolbox.phaseplane) | theGenSpecHelper  (in PyDSTool.Generator.ImplicitFnGen') | trunc  (in matplotlib.pylab) | | Tan  (in PyDSTool.Toolbox.synthetic\_data) | theGenSpecHelper  (in PyDSTool.Generator.InterpolateTable') | truncate\_to\_idx()  (in Trajectory) | | tan  (in PyDSTool.Toolbox.synthetic\_data) | theGenSpecHelper  (in PyDSTool.Generator.LookupTable') | truncate\_to\_idx()  (in Variable) | | Tan  (in PyDSTool.Toolbox.syntheticdata) | theGenSpecHelper  (in PyDSTool.Generator.MapSystem') | truncate\_to\_indepvar()  (in Trajectory) | | tan  (in PyDSTool.Toolbox.syntheticdata) | theGenSpecHelper  (in PyDSTool.Generator.ODEsystem') | trysimple()  (in PyDSTool.parseUtils) | | tan  (in matplotlib.pylab) | theGenSpecHelper  (in PyDSTool.Generator.Radau\_ODEsystem') | TU  (in PyDSTool.PyCont.ContClass') | | Tanh  (in PyDSTool.ModelSpec') | theGenSpecHelper  (in PyDSTool.Generator.Vode\_ODEsystem') | TU  (in matplotlib.pylab) | | tanh  (in PyDSTool.PyCont.ContClass') | theGenSpecHelper  (in PyDSTool.Generator.baseclasses) | TUPLE  (in PyDSTool.fixedpickle) | | Tanh  (in PyDSTool.Symbolic) | theGenSpecHelper  (in PyDSTool.Toolbox.NineML) | TUPLE1  (in PyDSTool.fixedpickle) | | tanh  (in PyDSTool.Symbolic) | theGenSpecHelper  (in PyDSTool.Toolbox.dataanalysis) | TUPLE2  (in PyDSTool.fixedpickle) | | Tanh  (in PyDSTool) | theGenSpecHelper  (in PyDSTool.Toolbox.phaseplane) | TUPLE3  (in PyDSTool.fixedpickle) | | Tanh  (in PyDSTool.Toolbox.ActivationFuncs) | theGenSpecHelper  (in PyDSTool.Toolbox.synthetic\_data) | typecodes  (in PyDSTool.PyCont.ContClass') | | Tanh  (in PyDSTool.Toolbox.DSSRT\_tools) | theGenSpecHelper  (in PyDSTool.Toolbox.syntheticdata) | typecodes  (in PyDSTool.Toolbox.ActivationFuncs) | | Tanh  (in PyDSTool.Toolbox.InputProfile) | threshold  (in PyDSTool.Toolbox.model\_primitives) | typecodes  (in PyDSTool.Toolbox.DSSRT\_tools) | | Tanh  (in PyDSTool.Toolbox.ModelHelper) | timeseq()  (in PyDSTool.Toolbox.fracdim) | typecodes  (in PyDSTool.Toolbox.InputProfile) | | Tanh  (in PyDSTool.Toolbox.NineML) | timestamp()  (in PyDSTool.common) | typecodes  (in PyDSTool.Toolbox.ModelHelper) | | tanh  (in PyDSTool.Toolbox.NineML) | to\_arrays()  (in data\_bins) | typecodes  (in PyDSTool.Toolbox.NineML) | | Tanh  (in PyDSTool.Toolbox.adjointPRC) | to\_arrays()  (in data\_bins) | typecodes  (in PyDSTool.Toolbox.adjointPRC) | | Tanh  (in PyDSTool.Toolbox.dataanalysis) | to\_pointset()  (in data\_bins) | typecodes  (in PyDSTool.Toolbox.dataanalysis) | | tanh  (in PyDSTool.Toolbox.dataanalysis) | to\_pointset()  (in data\_bins) | typecodes  (in PyDSTool.Toolbox.fracdim) | | Tanh  (in PyDSTool.Toolbox.fracdim) | toarray()  (in Point) | typecodes  (in PyDSTool.Toolbox.makeSloppyModel) | | Tanh  (in PyDSTool.Toolbox.makeSloppyModel) | toarray()  (in Pointset) | typecodes  (in PyDSTool.Toolbox.neuralcomp) | | Tanh  (in PyDSTool.Toolbox.neuralcomp) | toarray()  (in Point2D) | typecodes  (in PyDSTool.Toolbox.phaseplane) | | Tanh  (in PyDSTool.Toolbox.phaseplane) | toarray()  (in fixedpoint\_2D) | typecodes  (in PyDSTool.Toolbox.synthetic\_data) | | tanh  (in PyDSTool.Toolbox.phaseplane) | toCircumflexSyntax()  (in PyDSTool.parseUtils) | typecodes  (in PyDSTool.Toolbox.syntheticdata) | | Tanh  (in PyDSTool.Toolbox.synthetic\_data) | tocoords()  (in PyDSTool.PyCont.misc) | typecodes  (in PyDSTool) | | tanh  (in PyDSTool.Toolbox.synthetic\_data) | todict()  (in Point) | typecodes  (in matplotlib.pylab) | | Tanh  (in PyDSTool.Toolbox.syntheticdata) | todict()  (in Pointset) | typeCounter  (in PyDSTool.ModelSpec') | | tanh  (in PyDSTool.Toolbox.syntheticdata) | todict()  (in PyDSTool.PyCont.misc) | typeDict  (in PyDSTool.PyCont.ContClass') | | tanh  (in matplotlib.pylab) | todict()  (in Point2D) | typeDict  (in PyDSTool.Toolbox.ActivationFuncs) | | tanh\_spec  (in PyDSTool.Toolbox.model\_primitives) | toDoubleStarSyntax()  (in PyDSTool.parseUtils) | typeDict  (in PyDSTool.Toolbox.DSSRT\_tools) | | targetLangs  (in PyDSTool.FuncSpec') | toggleAll()  (in pargs) | typeDict  (in PyDSTool.Toolbox.InputProfile) | | targetLangs  (in PyDSTool.Generator.ADMC\_ODEsystem') | toggleCurve()  (in pargs) | typeDict  (in PyDSTool.Toolbox.ModelHelper) | | targetLangs  (in PyDSTool.Generator.Dopri\_ODEsystem') | toggleCurves()  (in pargs) | typeDict  (in PyDSTool.Toolbox.NineML) | | targetLangs  (in PyDSTool.Generator.EmbeddedSysGen') | toggleCycle()  (in pargs) | typeDict  (in PyDSTool.Toolbox.adjointPRC) | | targetLangs  (in PyDSTool.Generator.Euler\_ODEsystem') | toggleCycles()  (in pargs) | typeDict  (in PyDSTool.Toolbox.dataanalysis) | | targetLangs  (in PyDSTool.Generator.ExplicitFnGen') | toggleLabel()  (in pargs) | typeDict  (in PyDSTool.Toolbox.fracdim) | | targetLangs  (in PyDSTool.Generator.ExtrapolateTable') | toggleLabels()  (in pargs) | typeDict  (in PyDSTool.Toolbox.makeSloppyModel) | | targetLangs  (in PyDSTool.Generator.ImplicitFnGen') | togglePoint()  (in pargs) | typeDict  (in PyDSTool.Toolbox.neuralcomp) | | targetLangs  (in PyDSTool.Generator.InterpolateTable') | togglePoints()  (in pargs) | typeDict  (in PyDSTool.Toolbox.phaseplane) | | targetLangs  (in PyDSTool.Generator.LookupTable') | tonumeric()  (in QuantSpec) | typeDict  (in PyDSTool.Toolbox.synthetic\_data) | | targetLangs  (in PyDSTool.Generator.MapSystem') | tonumeric()  (in Quantity) | typeDict  (in PyDSTool.Toolbox.syntheticdata) | | targetLangs  (in PyDSTool.Generator.ODEsystem') | Toolbox  (in PyDSTool) | typeDict  (in PyDSTool) | | targetLangs  (in PyDSTool.Generator.Radau\_ODEsystem') | toPoint()  (in Point2D) | typeDict  (in matplotlib.pylab) | | targetLangs  (in PyDSTool.Generator.Vode\_ODEsystem') | toPointset()  (in nullcline) | typeNA  (in PyDSTool.PyCont.ContClass') | | targetLangs  (in PyDSTool.Interval') | toPowSyntax()  (in PyDSTool.parseUtils) | typeNA  (in PyDSTool.Toolbox.ActivationFuncs) | | targetLangs  (in PyDSTool.ModelConstructor') | Trajectory  (in PyDSTool.Trajectory') | typeNA  (in PyDSTool.Toolbox.DSSRT\_tools) | | targetLangs  (in ModelSpec) | Trajectory'  (in PyDSTool) | typeNA  (in PyDSTool.Toolbox.InputProfile) | | targetLangs  (in PyDSTool.ModelSpec') | transition\_psi()  (in PyDSTool.Toolbox.dssrt) | typeNA  (in PyDSTool.Toolbox.ModelHelper) | | targetLangs  (in Quantity) | transition\_tau()  (in PyDSTool.Toolbox.dssrt) | typeNA  (in PyDSTool.Toolbox.NineML) | | targetLangs  (in PyDSTool.Symbolic) | trilinearform()  (in PyDSTool.PyCont.misc) | typeNA  (in PyDSTool.Toolbox.adjointPRC) | | targetLangs  (in NineMLModel) | TRUE  (in PyDSTool.fixedpickle) | typeNA  (in PyDSTool.Toolbox.dataanalysis) | | targetLangs  (in PyDSTool.Toolbox.NineML) | True\_  (in PyDSTool.PyCont.ContClass') | typeNA  (in PyDSTool.Toolbox.fracdim) | | targetLangs  (in PyDSTool.Toolbox.dataanalysis) | True\_  (in PyDSTool.Toolbox.ActivationFuncs) | typeNA  (in PyDSTool.Toolbox.makeSloppyModel) | | targetLangs  (in PyDSTool.Toolbox.event\_driven\_simulator) | True\_  (in PyDSTool.Toolbox.DSSRT\_tools) | typeNA  (in PyDSTool.Toolbox.neuralcomp) | | targetLangs  (in sloppyModel) | True\_  (in PyDSTool.Toolbox.InputProfile) | typeNA  (in PyDSTool.Toolbox.phaseplane) | | targetLangs  (in compatODEComponent) | True\_  (in PyDSTool.Toolbox.ModelHelper) | typeNA  (in PyDSTool.Toolbox.synthetic\_data) | | targetLangs  (in compatODELeafComponent) | True\_  (in PyDSTool.Toolbox.NineML) | typeNA  (in PyDSTool.Toolbox.syntheticdata) | | targetLangs  (in PyDSTool.Toolbox.phaseplane) | True\_  (in PyDSTool.Toolbox.adjointPRC) | typeNA  (in PyDSTool) | | targetLangs  (in PyDSTool.Toolbox.synthetic\_data) | True\_  (in PyDSTool.Toolbox.dataanalysis) | typeNA  (in matplotlib.pylab) | | targetLangs  (in PyDSTool.Toolbox.syntheticdata) | True\_  (in PyDSTool.Toolbox.fracdim) | typestr  (in Fun) | | targetLangs  (in PyDSTool.Trajectory') | True\_  (in PyDSTool.Toolbox.makeSloppyModel) | typestr  (in Input) | | targetLangs  (in PyDSTool.Variable') | True\_  (in PyDSTool.Toolbox.neuralcomp) | typestr  (in Par) | | targetLangs  (in PyDSTool.common) | True\_  (in PyDSTool.Toolbox.phaseplane) | typestr  (in Quantity) | | targetLangs  (in PyDSTool.parseUtils) | True\_  (in PyDSTool.Toolbox.synthetic\_data) | typestr  (in Var) | |

  
  

| Home | Trees | Indices | Help | | PyDSTool | | --- | |
| --- | --- | --- | --- | --- | --- |

|  |  |
| --- | --- |
| Generated by Epydoc 3.0.1 on Fri May 4 15:23:59 2012 | http://epydoc.sourceforge.net |
