# Supplementary material for: Hybrid Models and Biological Model Reduction with PyDSTool
Source: PLoS Comput Biol. 2012 Aug 9;8(8):e1002628. doi: 10.1371/journal.pcbi.1002628 (PMC3415397; doi:10.1371/journal.pcbi.1002628)
Supplement: Text S4 — Complete source code for the PyDSTool package (version 0.88.120504). Includes API documentation and help files linking to web pages. This file is identical to the current public release on Sourceforge.net. (ZIP) [file pcbi.1002628.s004.zip › PyDSTool/html/identifier-index-U.html]

xml version="1.0" encoding="ascii"?


Identifier Index


| Home | Trees | Indices | Help | | PyDSTool | | --- | |
| --- | --- | --- | --- | --- | --- |

|  |  |  |  |
| --- | --- | --- | --- |
|  | |  | | --- | | [hide private] | | [frames] | no frames] | |

|  |  |
| --- | --- |
| Identifier Index | [ A B C D E F G H I J K L M N O P Q R S T U V W X Y Z \_ ] |

|  |  |  |  |  |  |  |  |  |  |  |  |  |  |  |  |  |  |  |  |  |  |  |  |  |  |  |  |  |  |  |  |  |  |  |  |  |  |  |  |  |  |  |  |  |  |  |  |  |  |  |  |  |  |  |  |  |  |  |  |  |  |  |  |  |  |  |  |  |  |  |  |  |  |  |  |  |  |  |  |  |  |  |  |  |  |  |  |  |  |  |  |  |  |  |  |  |  |  |  |  |  |  |  |  |  |  |  |  |  |
| --- | --- | --- | --- | --- | --- | --- | --- | --- | --- | --- | --- | --- | --- | --- | --- | --- | --- | --- | --- | --- | --- | --- | --- | --- | --- | --- | --- | --- | --- | --- | --- | --- | --- | --- | --- | --- | --- | --- | --- | --- | --- | --- | --- | --- | --- | --- | --- | --- | --- | --- | --- | --- | --- | --- | --- | --- | --- | --- | --- | --- | --- | --- | --- | --- | --- | --- | --- | --- | --- | --- | --- | --- | --- | --- | --- | --- | --- | --- | --- | --- | --- | --- | --- | --- | --- | --- | --- | --- | --- | --- | --- | --- | --- | --- | --- | --- | --- | --- | --- | --- | --- | --- | --- | --- | --- | --- | --- | --- | --- |
| U | |  |  |  | | --- | --- | --- | | u  (in PyDSTool.Interval') | uncertain  (in PyDSTool.Generator.Euler\_ODEsystem') | Uniform  (in PyDSTool.Toolbox.synthetic\_data) | | UFUNC\_BUFSIZE\_DEFAULT  (in PyDSTool.PyCont.ContClass') | uncertain  (in PyDSTool.Generator.ExplicitFnGen') | Uniform  (in PyDSTool.Toolbox.syntheticdata) | | UFUNC\_BUFSIZE\_DEFAULT  (in PyDSTool.Toolbox.ActivationFuncs) | uncertain  (in PyDSTool.Generator.ExtrapolateTable') | Uniform  (in PyDSTool) | | UFUNC\_BUFSIZE\_DEFAULT  (in PyDSTool.Toolbox.DSSRT\_tools) | uncertain  (in PyDSTool.Generator.ImplicitFnGen') | union()  (in PyDSTool.utils) | | UFUNC\_BUFSIZE\_DEFAULT  (in PyDSTool.Toolbox.InputProfile) | uncertain  (in PyDSTool.Generator.InterpolateTable') | unique()  (in PyDSTool.PyCont.misc) | | UFUNC\_BUFSIZE\_DEFAULT  (in PyDSTool.Toolbox.ModelHelper) | uncertain  (in PyDSTool.Generator.LookupTable') | uniquePoints()  (in PyDSTool.common) | | UFUNC\_BUFSIZE\_DEFAULT  (in PyDSTool.Toolbox.NineML) | uncertain  (in PyDSTool.Generator.MapSystem') | UnpickleableError | | UFUNC\_BUFSIZE\_DEFAULT  (in PyDSTool.Toolbox.adjointPRC) | uncertain  (in PyDSTool.Generator.ODEsystem') | Unpickler  (in PyDSTool.fixedpickle) | | UFUNC\_BUFSIZE\_DEFAULT  (in PyDSTool.Toolbox.dataanalysis) | uncertain  (in PyDSTool.Generator.Radau\_ODEsystem') | UnpicklingError  (in PyDSTool.fixedpickle) | | UFUNC\_BUFSIZE\_DEFAULT  (in PyDSTool.Toolbox.fracdim) | uncertain  (in PyDSTool.Generator.Vode\_ODEsystem') | UnpicklingError | | UFUNC\_BUFSIZE\_DEFAULT  (in PyDSTool.Toolbox.makeSloppyModel) | uncertain  (in PyDSTool.Interval') | unregister()  (in genDBClass) | | UFUNC\_BUFSIZE\_DEFAULT  (in PyDSTool.Toolbox.neuralcomp) | uncertain  (in PyDSTool.Toolbox.NineML) | unresolved()  (in GenTransform) | | UFUNC\_BUFSIZE\_DEFAULT  (in PyDSTool.Toolbox.phaseplane) | uncertain  (in PyDSTool.Toolbox.dataanalysis) | unresolved()  (in ModelTransform) | | UFUNC\_BUFSIZE\_DEFAULT  (in PyDSTool.Toolbox.synthetic\_data) | uncertain  (in PyDSTool.Toolbox.phaseplane) | update()  (in Point) | | UFUNC\_BUFSIZE\_DEFAULT  (in PyDSTool.Toolbox.syntheticdata) | uncertain  (in PyDSTool.Toolbox.synthetic\_data) | update()  (in PointInfo) | | UFUNC\_BUFSIZE\_DEFAULT  (in PyDSTool) | uncertain  (in PyDSTool.Toolbox.syntheticdata) | update()  (in ContClass) | | UFUNC\_BUFSIZE\_DEFAULT  (in matplotlib.pylab) | uncertain  (in PyDSTool.Trajectory') | update()  (in Continuation) | | UFUNC\_PYVALS\_NAME  (in PyDSTool.PyCont.ContClass') | uncertain  (in PyDSTool.Variable') | update()  (in EquilibriumCurve) | | UFUNC\_PYVALS\_NAME  (in PyDSTool.Toolbox.ActivationFuncs) | underlyingMesh()  (in HybridTrajectory) | update()  (in FixedPointCurve) | | UFUNC\_PYVALS\_NAME  (in PyDSTool.Toolbox.DSSRT\_tools) | underlyingMesh()  (in Trajectory) | update()  (in FoldCurve) | | UFUNC\_PYVALS\_NAME  (in PyDSTool.Toolbox.InputProfile) | underlyingMesh()  (in HybridVariable) | update()  (in HopfCurveOne) | | UFUNC\_PYVALS\_NAME  (in PyDSTool.Toolbox.ModelHelper) | underlyingMesh()  (in Variable) | update()  (in HopfCurveTwo) | | UFUNC\_PYVALS\_NAME  (in PyDSTool.Toolbox.NineML) | UNICODE  (in PyDSTool.fixedpickle) | update()  (in LimitCycleCurve) | | UFUNC\_PYVALS\_NAME  (in PyDSTool.Toolbox.adjointPRC) | Uniform  (in PyDSTool.ModelSpec') | update()  (in UserDefinedCurve) | | UFUNC\_PYVALS\_NAME  (in PyDSTool.Toolbox.dataanalysis) | Uniform  (in PyDSTool.Symbolic) | update()  (in Point2D) | | UFUNC\_PYVALS\_NAME  (in PyDSTool.Toolbox.fracdim) | Uniform  (in PyDSTool.Toolbox.ActivationFuncs) | update()  (in mesh\_patch\_2D) | | UFUNC\_PYVALS\_NAME  (in PyDSTool.Toolbox.makeSloppyModel) | Uniform  (in PyDSTool.Toolbox.DSSRT\_tools) | update()  (in Diagnostics) | | UFUNC\_PYVALS\_NAME  (in PyDSTool.Toolbox.neuralcomp) | Uniform  (in PyDSTool.Toolbox.InputProfile) | update()  (in args) | | UFUNC\_PYVALS\_NAME  (in PyDSTool.Toolbox.phaseplane) | Uniform  (in PyDSTool.Toolbox.ModelHelper) | update()  (in symbolMapClass) | | UFUNC\_PYVALS\_NAME  (in PyDSTool.Toolbox.synthetic\_data) | Uniform  (in PyDSTool.Toolbox.NineML) | updatedata()  (in BorderMethod) | | UFUNC\_PYVALS\_NAME  (in PyDSTool.Toolbox.syntheticdata) | Uniform  (in PyDSTool.Toolbox.adjointPRC) | updatedata()  (in Hopf\_Double\_Bor\_One) | | UFUNC\_PYVALS\_NAME  (in PyDSTool) | Uniform  (in PyDSTool.Toolbox.dataanalysis) | userdefined\_args\_list  (in PyDSTool.PyCont.Continuation) | | UFUNC\_PYVALS\_NAME  (in matplotlib.pylab) | Uniform  (in PyDSTool.Toolbox.fracdim) | UserDefinedCurve  (in PyDSTool.PyCont.Continuation) | | uncertain  (in PyDSTool.Generator.ADMC\_ODEsystem') | Uniform  (in PyDSTool.Toolbox.makeSloppyModel) | UserDefinedTestFunc  (in PyDSTool.PyCont.TestFunc) | | uncertain  (in PyDSTool.Generator.Dopri\_ODEsystem') | Uniform  (in PyDSTool.Toolbox.neuralcomp) | Utility  (in PyDSTool.common) | | uncertain  (in PyDSTool.Generator.EmbeddedSysGen') | Uniform  (in PyDSTool.Toolbox.phaseplane) | utils  (in PyDSTool) | |

  
  

| Home | Trees | Indices | Help | | PyDSTool | | --- | |
| --- | --- | --- | --- | --- | --- |

|  |  |
| --- | --- |
| Generated by Epydoc 3.0.1 on Fri May 4 15:23:59 2012 | http://epydoc.sourceforge.net |
