# Supplementary material for: Hybrid Models and Biological Model Reduction with PyDSTool
Source: PLoS Comput Biol. 2012 Aug 9;8(8):e1002628. doi: 10.1371/journal.pcbi.1002628 (PMC3415397; doi:10.1371/journal.pcbi.1002628)
Supplement: Text S4 — Complete source code for the PyDSTool package (version 0.88.120504). Includes API documentation and help files linking to web pages. This file is identical to the current public release on Sourceforge.net. (ZIP) [file pcbi.1002628.s004.zip › PyDSTool/html/identifier-index-V.html]

xml version="1.0" encoding="ascii"?


Identifier Index


| Home | Trees | Indices | Help | | PyDSTool | | --- | |
| --- | --- | --- | --- | --- | --- |

|  |  |  |  |
| --- | --- | --- | --- |
|  | |  | | --- | | [hide private] | | [frames] | no frames] | |

|  |  |
| --- | --- |
| Identifier Index | [ A B C D E F G H I J K L M N O P Q R S T U V W X Y Z \_ ] |

|  |  |  |  |  |  |  |  |  |  |  |  |  |  |  |  |  |  |  |  |  |  |  |  |  |  |  |  |  |  |  |  |  |  |  |  |  |  |  |  |  |  |  |  |  |  |  |  |  |  |  |  |  |  |  |  |  |  |  |  |  |  |  |  |  |  |  |  |  |  |  |  |  |  |  |  |  |  |  |  |
| --- | --- | --- | --- | --- | --- | --- | --- | --- | --- | --- | --- | --- | --- | --- | --- | --- | --- | --- | --- | --- | --- | --- | --- | --- | --- | --- | --- | --- | --- | --- | --- | --- | --- | --- | --- | --- | --- | --- | --- | --- | --- | --- | --- | --- | --- | --- | --- | --- | --- | --- | --- | --- | --- | --- | --- | --- | --- | --- | --- | --- | --- | --- | --- | --- | --- | --- | --- | --- | --- | --- | --- | --- | --- | --- | --- | --- | --- | --- | --- |
| V | |  |  |  | | --- | --- | --- | | V  (in PyDSTool.Toolbox.neuralcomp) | values()  (in Point) | vertex  (in PyDSTool.Toolbox.FR) | | vald  (in Verbose) | values()  (in data\_bins) | VFAR  (in PyDSTool.Toolbox.dssrt) | | validate()  (in feature) | values()  (in data\_bins) | virtual\_experiment  (in PyDSTool.Toolbox.test\_protocols) | | validate()  (in Descriptor) | values()  (in Point2D) | vode  (in PyDSTool.scipy\_ode) | | validate()  (in GDescriptor) | values()  (in args) | vode  (in scipy.integrate) | | validate()  (in MDescriptor) | values()  (in symbolMapClass) | Vode\_ODEsystem  (in PyDSTool.Generator.Vode\_ODEsystem') | | validate()  (in ModelSpec) | Var  (in PyDSTool.Symbolic) | Vode\_ODEsystem'  (in PyDSTool.Generator) | | validate()  (in Quantity) | VarAlphabet  (in PyDSTool.Toolbox.dssrt) | voltage  (in PyDSTool.Toolbox.neuralcomp) | | validate()  (in simulator) | VarCaller  (in PyDSTool.Points) | Vonmisesvariate  (in PyDSTool.ModelSpec') | | validateDef()  (in FuncSpec) | VarDiagnostics  (in PyDSTool.Variable') | Vonmisesvariate  (in PyDSTool.Symbolic) | | validateDependencies()  (in FuncSpec) | Variable  (in PyDSTool.Variable') | Vonmisesvariate  (in PyDSTool.Toolbox.ActivationFuncs) | | validateEvents()  (in EventStruct) | Variable'  (in PyDSTool) | Vonmisesvariate  (in PyDSTool.Toolbox.DSSRT\_tools) | | validateICs()  (in ODEsystem) | VCLOSE  (in PyDSTool.Toolbox.dssrt) | Vonmisesvariate  (in PyDSTool.Toolbox.InputProfile) | | validateSpec()  (in DDEsystem) | ver  (in PyDSTool.matplotlib\_import) | Vonmisesvariate  (in PyDSTool.Toolbox.ModelHelper) | | validateSpec()  (in EmbeddedSysGen) | Verbose  (in PyDSTool.common) | Vonmisesvariate  (in PyDSTool.Toolbox.NineML) | | validateSpec()  (in ExplicitFnGen) | verify\_intbool()  (in PyDSTool.common) | Vonmisesvariate  (in PyDSTool.Toolbox.adjointPRC) | | validateSpec()  (in ExtrapolateTable) | verify\_nonneg()  (in PyDSTool.common) | Vonmisesvariate  (in PyDSTool.Toolbox.dataanalysis) | | validateSpec()  (in ImplicitFnGen) | verify\_pos()  (in PyDSTool.common) | Vonmisesvariate  (in PyDSTool.Toolbox.fracdim) | | validateSpec()  (in InterpolateTable) | verify\_values()  (in PyDSTool.common) | Vonmisesvariate  (in PyDSTool.Toolbox.makeSloppyModel) | | validateSpec()  (in LookupTable) | version  (in PyDSTool.conf) | Vonmisesvariate  (in PyDSTool.Toolbox.neuralcomp) | | validateSpec()  (in Generator) | verstr\_parts  (in PyDSTool.Toolbox.NineML) | Vonmisesvariate  (in PyDSTool.Toolbox.phaseplane) | | validateSpec()  (in ctsGen) | verstr\_parts  (in PyDSTool.Toolbox.dataanalysis) | Vonmisesvariate  (in PyDSTool.Toolbox.synthetic\_data) | | validateSpec()  (in discGen) | verstr\_parts  (in PyDSTool.Toolbox.phaseplane) | Vonmisesvariate  (in PyDSTool.Toolbox.syntheticdata) | | validateTransitionName()  (in PyDSTool.ModelConstructor') | verstr\_parts  (in PyDSTool.Toolbox.synthetic\_data) | Vonmisesvariate  (in PyDSTool) | | values()  (in auxfn\_container) | verstr\_parts  (in PyDSTool.Toolbox.syntheticdata) |  | | values()  (in condition) | verstr\_parts  (in PyDSTool) |  | |

  
  

| Home | Trees | Indices | Help | | PyDSTool | | --- | |
| --- | --- | --- | --- | --- | --- |

|  |  |
| --- | --- |
| Generated by Epydoc 3.0.1 on Fri May 4 15:23:59 2012 | http://epydoc.sourceforge.net |
