# Supplementary material for: Hybrid Models and Biological Model Reduction with PyDSTool
Source: PLoS Comput Biol. 2012 Aug 9;8(8):e1002628. doi: 10.1371/journal.pcbi.1002628 (PMC3415397; doi:10.1371/journal.pcbi.1002628)
Supplement: Text S4 — Complete source code for the PyDSTool package (version 0.88.120504). Includes API documentation and help files linking to web pages. This file is identical to the current public release on Sourceforge.net. (ZIP) [file pcbi.1002628.s004.zip › PyDSTool/html/identifier-index-W.html]

xml version="1.0" encoding="ascii"?


Identifier Index


| Home | Trees | Indices | Help | | PyDSTool | | --- | |
| --- | --- | --- | --- | --- | --- |

|  |  |  |  |
| --- | --- | --- | --- |
|  | |  | | --- | | [hide private] | | [frames] | no frames] | |

|  |  |
| --- | --- |
| Identifier Index | [ A B C D E F G H I J K L M N O P Q R S T U V W X Y Z \_ ] |

|  |  |  |  |  |  |  |  |  |  |  |  |  |  |  |  |  |  |  |  |  |  |  |  |  |  |  |  |  |  |  |  |  |  |  |  |  |  |  |  |  |  |  |  |  |  |  |  |  |  |  |  |  |  |  |  |  |  |  |  |  |  |  |  |  |  |  |  |  |  |  |  |  |  |  |  |  |  |  |  |  |  |  |  |  |  |  |  |  |  |  |  |  |  |  |  |  |  |  |  |  |  |  |  |  |  |  |  |  |  |  |  |  |  |  |  |  |  |  |  |  |  |  |  |  |  |  |  |  |  |  |  |  |  |  |  |  |  |  |  |  |  |  |  |  |  |  |  |  |  |  |  |  |  |  |  |  |  |  |  |  |  |  |  |  |  |  |  |  |  |  |  |  |  |  |  |  |  |  |  |  |  |  |  |  |  |  |  |  |  |  |  |  |  |  |  |  |  |  |  |  |  |  |  |  |  |  |  |  |  |  |  |  |  |  |  |  |  |
| --- | --- | --- | --- | --- | --- | --- | --- | --- | --- | --- | --- | --- | --- | --- | --- | --- | --- | --- | --- | --- | --- | --- | --- | --- | --- | --- | --- | --- | --- | --- | --- | --- | --- | --- | --- | --- | --- | --- | --- | --- | --- | --- | --- | --- | --- | --- | --- | --- | --- | --- | --- | --- | --- | --- | --- | --- | --- | --- | --- | --- | --- | --- | --- | --- | --- | --- | --- | --- | --- | --- | --- | --- | --- | --- | --- | --- | --- | --- | --- | --- | --- | --- | --- | --- | --- | --- | --- | --- | --- | --- | --- | --- | --- | --- | --- | --- | --- | --- | --- | --- | --- | --- | --- | --- | --- | --- | --- | --- | --- | --- | --- | --- | --- | --- | --- | --- | --- | --- | --- | --- | --- | --- | --- | --- | --- | --- | --- | --- | --- | --- | --- | --- | --- | --- | --- | --- | --- | --- | --- | --- | --- | --- | --- | --- | --- | --- | --- | --- | --- | --- | --- | --- | --- | --- | --- | --- | --- | --- | --- | --- | --- | --- | --- | --- | --- | --- | --- | --- | --- | --- | --- | --- | --- | --- | --- | --- | --- | --- | --- | --- | --- | --- | --- | --- | --- | --- | --- | --- | --- | --- | --- | --- | --- | --- | --- | --- | --- | --- | --- | --- | --- | --- | --- | --- | --- | --- | --- | --- | --- | --- | --- | --- | --- | --- | --- | --- | --- |
| W | |  |  |  | | --- | --- | --- | | W\_BISECTLIMIT  (in PyDSTool.Generator.ADMC\_ODEsystem') | W\_TERMEVENT  (in PyDSTool.Toolbox.dataanalysis) | warnmessages  (in PyDSTool.Generator.Radau\_ODEsystem') | | W\_BISECTLIMIT  (in PyDSTool.Generator.Dopri\_ODEsystem') | W\_TERMEVENT  (in PyDSTool.Toolbox.phaseplane) | warnmessages  (in PyDSTool.Generator.Vode\_ODEsystem') | | W\_BISECTLIMIT  (in PyDSTool.Generator.EmbeddedSysGen') | W\_TERMEVENT  (in PyDSTool.Toolbox.synthetic\_data) | warnmessages  (in PyDSTool.Generator.messagecodes) | | W\_BISECTLIMIT  (in PyDSTool.Generator.Euler\_ODEsystem') | W\_TERMEVENT  (in PyDSTool.Toolbox.syntheticdata) | warnmessages  (in PyDSTool.Toolbox.NineML) | | W\_BISECTLIMIT  (in PyDSTool.Generator.ExplicitFnGen') | W\_TERMSTATEBD  (in PyDSTool.Generator.ADMC\_ODEsystem') | warnmessages  (in PyDSTool.Toolbox.dataanalysis) | | W\_BISECTLIMIT  (in PyDSTool.Generator.ExtrapolateTable') | W\_TERMSTATEBD  (in PyDSTool.Generator.Dopri\_ODEsystem') | warnmessages  (in PyDSTool.Toolbox.phaseplane) | | W\_BISECTLIMIT  (in PyDSTool.Generator.ImplicitFnGen') | W\_TERMSTATEBD  (in PyDSTool.Generator.EmbeddedSysGen') | warnmessages  (in PyDSTool.Toolbox.synthetic\_data) | | W\_BISECTLIMIT  (in PyDSTool.Generator.InterpolateTable') | W\_TERMSTATEBD  (in PyDSTool.Generator.Euler\_ODEsystem') | warnmessages  (in PyDSTool.Toolbox.syntheticdata) | | W\_BISECTLIMIT  (in PyDSTool.Generator.LookupTable') | W\_TERMSTATEBD  (in PyDSTool.Generator.ExplicitFnGen') | WE  (in PyDSTool.PyCont.ContClass') | | W\_BISECTLIMIT  (in PyDSTool.Generator.MapSystem') | W\_TERMSTATEBD  (in PyDSTool.Generator.ExtrapolateTable') | WE  (in matplotlib.pylab) | | W\_BISECTLIMIT  (in PyDSTool.Generator.ODEsystem') | W\_TERMSTATEBD  (in PyDSTool.Generator.ImplicitFnGen') | wedge()  (in PyDSTool.PyCont.misc) | | W\_BISECTLIMIT  (in PyDSTool.Generator.Radau\_ODEsystem') | W\_TERMSTATEBD  (in PyDSTool.Generator.InterpolateTable') | WEEKLY  (in PyDSTool.PyCont.ContClass') | | W\_BISECTLIMIT  (in PyDSTool.Generator.Vode\_ODEsystem') | W\_TERMSTATEBD  (in PyDSTool.Generator.LookupTable') | WEEKLY  (in matplotlib.pylab) | | W\_BISECTLIMIT  (in PyDSTool.Generator.messagecodes) | W\_TERMSTATEBD  (in PyDSTool.Generator.MapSystem') | Weibullvariate  (in PyDSTool.ModelSpec') | | W\_BISECTLIMIT  (in PyDSTool.Toolbox.NineML) | W\_TERMSTATEBD  (in PyDSTool.Generator.ODEsystem') | Weibullvariate  (in PyDSTool.Symbolic) | | W\_BISECTLIMIT  (in PyDSTool.Toolbox.dataanalysis) | W\_TERMSTATEBD  (in PyDSTool.Generator.Radau\_ODEsystem') | Weibullvariate  (in PyDSTool.Toolbox.ActivationFuncs) | | W\_BISECTLIMIT  (in PyDSTool.Toolbox.phaseplane) | W\_TERMSTATEBD  (in PyDSTool.Generator.Vode\_ODEsystem') | Weibullvariate  (in PyDSTool.Toolbox.DSSRT\_tools) | | W\_BISECTLIMIT  (in PyDSTool.Toolbox.synthetic\_data) | W\_TERMSTATEBD  (in PyDSTool.Generator.messagecodes) | Weibullvariate  (in PyDSTool.Toolbox.InputProfile) | | W\_BISECTLIMIT  (in PyDSTool.Toolbox.syntheticdata) | W\_TERMSTATEBD  (in PyDSTool.Toolbox.NineML) | Weibullvariate  (in PyDSTool.Toolbox.ModelHelper) | | W\_NONTERMEVENT  (in PyDSTool.Generator.ADMC\_ODEsystem') | W\_TERMSTATEBD  (in PyDSTool.Toolbox.dataanalysis) | Weibullvariate  (in PyDSTool.Toolbox.NineML) | | W\_NONTERMEVENT  (in PyDSTool.Generator.Dopri\_ODEsystem') | W\_TERMSTATEBD  (in PyDSTool.Toolbox.phaseplane) | Weibullvariate  (in PyDSTool.Toolbox.adjointPRC) | | W\_NONTERMEVENT  (in PyDSTool.Generator.EmbeddedSysGen') | W\_TERMSTATEBD  (in PyDSTool.Toolbox.synthetic\_data) | Weibullvariate  (in PyDSTool.Toolbox.dataanalysis) | | W\_NONTERMEVENT  (in PyDSTool.Generator.Euler\_ODEsystem') | W\_TERMSTATEBD  (in PyDSTool.Toolbox.syntheticdata) | Weibullvariate  (in PyDSTool.Toolbox.fracdim) | | W\_NONTERMEVENT  (in PyDSTool.Generator.ExplicitFnGen') | W\_UNCERTVAL  (in PyDSTool.Generator.ADMC\_ODEsystem') | Weibullvariate  (in PyDSTool.Toolbox.makeSloppyModel) | | W\_NONTERMEVENT  (in PyDSTool.Generator.ExtrapolateTable') | W\_UNCERTVAL  (in PyDSTool.Generator.Dopri\_ODEsystem') | Weibullvariate  (in PyDSTool.Toolbox.neuralcomp) | | W\_NONTERMEVENT  (in PyDSTool.Generator.ImplicitFnGen') | W\_UNCERTVAL  (in PyDSTool.Generator.EmbeddedSysGen') | Weibullvariate  (in PyDSTool.Toolbox.phaseplane) | | W\_NONTERMEVENT  (in PyDSTool.Generator.InterpolateTable') | W\_UNCERTVAL  (in PyDSTool.Generator.Euler\_ODEsystem') | Weibullvariate  (in PyDSTool.Toolbox.synthetic\_data) | | W\_NONTERMEVENT  (in PyDSTool.Generator.LookupTable') | W\_UNCERTVAL  (in PyDSTool.Generator.ExplicitFnGen') | Weibullvariate  (in PyDSTool.Toolbox.syntheticdata) | | W\_NONTERMEVENT  (in PyDSTool.Generator.MapSystem') | W\_UNCERTVAL  (in PyDSTool.Generator.ExtrapolateTable') | Weibullvariate  (in PyDSTool) | | W\_NONTERMEVENT  (in PyDSTool.Generator.ODEsystem') | W\_UNCERTVAL  (in PyDSTool.Generator.ImplicitFnGen') | weighted\_par\_sensitivity()  (in ParamEst) | | W\_NONTERMEVENT  (in PyDSTool.Generator.Radau\_ODEsystem') | W\_UNCERTVAL  (in PyDSTool.Generator.InterpolateTable') | whichmodule()  (in PyDSTool.fixedpickle) | | W\_NONTERMEVENT  (in PyDSTool.Generator.Vode\_ODEsystem') | W\_UNCERTVAL  (in PyDSTool.Generator.LookupTable') | whiten()  (in PyDSTool.Toolbox.data\_analysis) | | W\_NONTERMEVENT  (in PyDSTool.Generator.messagecodes) | W\_UNCERTVAL  (in PyDSTool.Generator.MapSystem') | whiten()  (in PyDSTool.Toolbox.dataanalysis) | | W\_NONTERMEVENT  (in PyDSTool.Toolbox.NineML) | W\_UNCERTVAL  (in PyDSTool.Generator.ODEsystem') | who()  (in PyDSTool) | | W\_NONTERMEVENT  (in PyDSTool.Toolbox.dataanalysis) | W\_UNCERTVAL  (in PyDSTool.Generator.Radau\_ODEsystem') | whoQ()  (in PyDSTool.Symbolic) | | W\_NONTERMEVENT  (in PyDSTool.Toolbox.phaseplane) | W\_UNCERTVAL  (in PyDSTool.Generator.Vode\_ODEsystem') | Wichmannhill  (in PyDSTool.ModelSpec') | | W\_NONTERMEVENT  (in PyDSTool.Toolbox.synthetic\_data) | W\_UNCERTVAL  (in PyDSTool.Generator.messagecodes) | Wichmannhill  (in PyDSTool.Symbolic) | | W\_NONTERMEVENT  (in PyDSTool.Toolbox.syntheticdata) | W\_UNCERTVAL  (in PyDSTool.Toolbox.NineML) | Wichmannhill  (in PyDSTool.Toolbox.ActivationFuncs) | | W\_NONTERMSTATEBD  (in PyDSTool.Generator.ADMC\_ODEsystem') | W\_UNCERTVAL  (in PyDSTool.Toolbox.dataanalysis) | Wichmannhill  (in PyDSTool.Toolbox.DSSRT\_tools) | | W\_NONTERMSTATEBD  (in PyDSTool.Generator.Dopri\_ODEsystem') | W\_UNCERTVAL  (in PyDSTool.Toolbox.phaseplane) | Wichmannhill  (in PyDSTool.Toolbox.InputProfile) | | W\_NONTERMSTATEBD  (in PyDSTool.Generator.EmbeddedSysGen') | W\_UNCERTVAL  (in PyDSTool.Toolbox.synthetic\_data) | Wichmannhill  (in PyDSTool.Toolbox.ModelHelper) | | W\_NONTERMSTATEBD  (in PyDSTool.Generator.Euler\_ODEsystem') | W\_UNCERTVAL  (in PyDSTool.Toolbox.syntheticdata) | Wichmannhill  (in PyDSTool.Toolbox.NineML) | | W\_NONTERMSTATEBD  (in PyDSTool.Generator.ExplicitFnGen') | warnfields  (in PyDSTool.Generator.ADMC\_ODEsystem') | Wichmannhill  (in PyDSTool.Toolbox.adjointPRC) | | W\_NONTERMSTATEBD  (in PyDSTool.Generator.ExtrapolateTable') | warnfields  (in PyDSTool.Generator.Dopri\_ODEsystem') | Wichmannhill  (in PyDSTool.Toolbox.dataanalysis) | | W\_NONTERMSTATEBD  (in PyDSTool.Generator.ImplicitFnGen') | warnfields  (in PyDSTool.Generator.EmbeddedSysGen') | Wichmannhill  (in PyDSTool.Toolbox.fracdim) | | W\_NONTERMSTATEBD  (in PyDSTool.Generator.InterpolateTable') | warnfields  (in PyDSTool.Generator.Euler\_ODEsystem') | Wichmannhill  (in PyDSTool.Toolbox.makeSloppyModel) | | W\_NONTERMSTATEBD  (in PyDSTool.Generator.LookupTable') | warnfields  (in PyDSTool.Generator.ExplicitFnGen') | Wichmannhill  (in PyDSTool.Toolbox.neuralcomp) | | W\_NONTERMSTATEBD  (in PyDSTool.Generator.MapSystem') | warnfields  (in PyDSTool.Generator.ExtrapolateTable') | Wichmannhill  (in PyDSTool.Toolbox.phaseplane) | | W\_NONTERMSTATEBD  (in PyDSTool.Generator.ODEsystem') | warnfields  (in PyDSTool.Generator.ImplicitFnGen') | Wichmannhill  (in PyDSTool.Toolbox.synthetic\_data) | | W\_NONTERMSTATEBD  (in PyDSTool.Generator.Radau\_ODEsystem') | warnfields  (in PyDSTool.Generator.InterpolateTable') | Wichmannhill  (in PyDSTool.Toolbox.syntheticdata) | | W\_NONTERMSTATEBD  (in PyDSTool.Generator.Vode\_ODEsystem') | warnfields  (in PyDSTool.Generator.LookupTable') | Wichmannhill  (in PyDSTool) | | W\_NONTERMSTATEBD  (in PyDSTool.Generator.messagecodes) | warnfields  (in PyDSTool.Generator.MapSystem') | wolfe\_powell\_rule  (in PyDSTool.Toolbox.optimizers.line\_search) | | W\_NONTERMSTATEBD  (in PyDSTool.Toolbox.NineML) | warnfields  (in PyDSTool.Generator.ODEsystem') | WolfePowellRule  (in PyDSTool.Toolbox.optimizers.line\_search.wolfe\_powell\_rule) | | W\_NONTERMSTATEBD  (in PyDSTool.Toolbox.dataanalysis) | warnfields  (in PyDSTool.Generator.Radau\_ODEsystem') | WRAP  (in PyDSTool.PyCont.ContClass') | | W\_NONTERMSTATEBD  (in PyDSTool.Toolbox.phaseplane) | warnfields  (in PyDSTool.Generator.Vode\_ODEsystem') | WRAP  (in PyDSTool.Toolbox.ActivationFuncs) | | W\_NONTERMSTATEBD  (in PyDSTool.Toolbox.synthetic\_data) | warnfields  (in PyDSTool.Generator.messagecodes) | WRAP  (in PyDSTool.Toolbox.DSSRT\_tools) | | W\_NONTERMSTATEBD  (in PyDSTool.Toolbox.syntheticdata) | warnfields  (in PyDSTool.Toolbox.NineML) | WRAP  (in PyDSTool.Toolbox.InputProfile) | | W\_TERMEVENT  (in PyDSTool.Generator.ADMC\_ODEsystem') | warnfields  (in PyDSTool.Toolbox.dataanalysis) | WRAP  (in PyDSTool.Toolbox.ModelHelper) | | W\_TERMEVENT  (in PyDSTool.Generator.Dopri\_ODEsystem') | warnfields  (in PyDSTool.Toolbox.phaseplane) | WRAP  (in PyDSTool.Toolbox.NineML) | | W\_TERMEVENT  (in PyDSTool.Generator.EmbeddedSysGen') | warnfields  (in PyDSTool.Toolbox.synthetic\_data) | WRAP  (in PyDSTool.Toolbox.adjointPRC) | | W\_TERMEVENT  (in PyDSTool.Generator.Euler\_ODEsystem') | warnfields  (in PyDSTool.Toolbox.syntheticdata) | WRAP  (in PyDSTool.Toolbox.dataanalysis) | | W\_TERMEVENT  (in PyDSTool.Generator.ExplicitFnGen') | warnmessages  (in PyDSTool.Generator.ADMC\_ODEsystem') | WRAP  (in PyDSTool.Toolbox.fracdim) | | W\_TERMEVENT  (in PyDSTool.Generator.ExtrapolateTable') | warnmessages  (in PyDSTool.Generator.Dopri\_ODEsystem') | WRAP  (in PyDSTool.Toolbox.makeSloppyModel) | | W\_TERMEVENT  (in PyDSTool.Generator.ImplicitFnGen') | warnmessages  (in PyDSTool.Generator.EmbeddedSysGen') | WRAP  (in PyDSTool.Toolbox.neuralcomp) | | W\_TERMEVENT  (in PyDSTool.Generator.InterpolateTable') | warnmessages  (in PyDSTool.Generator.Euler\_ODEsystem') | WRAP  (in PyDSTool.Toolbox.phaseplane) | | W\_TERMEVENT  (in PyDSTool.Generator.LookupTable') | warnmessages  (in PyDSTool.Generator.ExplicitFnGen') | WRAP  (in PyDSTool.Toolbox.synthetic\_data) | | W\_TERMEVENT  (in PyDSTool.Generator.MapSystem') | warnmessages  (in PyDSTool.Generator.ExtrapolateTable') | WRAP  (in PyDSTool.Toolbox.syntheticdata) | | W\_TERMEVENT  (in PyDSTool.Generator.ODEsystem') | warnmessages  (in PyDSTool.Generator.ImplicitFnGen') | WRAP  (in PyDSTool) | | W\_TERMEVENT  (in PyDSTool.Generator.Radau\_ODEsystem') | warnmessages  (in PyDSTool.Generator.InterpolateTable') | WRAP  (in matplotlib.pylab) | | W\_TERMEVENT  (in PyDSTool.Generator.Vode\_ODEsystem') | warnmessages  (in PyDSTool.Generator.LookupTable') | wrap()  (in Verbose) | | W\_TERMEVENT  (in PyDSTool.Generator.messagecodes) | warnmessages  (in PyDSTool.Generator.MapSystem') | wrapArgInCall()  (in PyDSTool.parseUtils) | | W\_TERMEVENT  (in PyDSTool.Toolbox.NineML) | warnmessages  (in PyDSTool.Generator.ODEsystem') |  | |

  
  

| Home | Trees | Indices | Help | | PyDSTool | | --- | |
| --- | --- | --- | --- | --- | --- |

|  |  |
| --- | --- |
| Generated by Epydoc 3.0.1 on Fri May 4 15:23:59 2012 | http://epydoc.sourceforge.net |
