# Supplementary material for: Hybrid Models and Biological Model Reduction with PyDSTool
Source: PLoS Comput Biol. 2012 Aug 9;8(8):e1002628. doi: 10.1371/journal.pcbi.1002628 (PMC3415397; doi:10.1371/journal.pcbi.1002628)
Supplement: Text S4 — Complete source code for the PyDSTool package (version 0.88.120504). Includes API documentation and help files linking to web pages. This file is identical to the current public release on Sourceforge.net. (ZIP) [file pcbi.1002628.s004.zip › PyDSTool/html/identifier-index.html]

xml version="1.0" encoding="ascii"?


Identifier Index


| Home | Trees | Indices | Help | | PyDSTool | | --- | |
| --- | --- | --- | --- | --- | --- |

|  |  |  |  |
| --- | --- | --- | --- |
|  | |  | | --- | | [hide private] | | [frames] | no frames] | |

|  |  |
| --- | --- |
| Identifier Index | [ A B C D E F G H I J K L M N O P Q R S T U V W X Y Z \_ ] |

|  |  |  |  |  |  |  |  |  |  |  |  |  |  |  |  |  |  |  |  |  |  |  |  |  |  |  |  |  |  |  |  |  |  |  |  |  |  |  |  |  |  |  |  |  |  |  |  |  |  |  |  |  |  |  |  |  |  |  |  |  |  |  |  |  |  |  |  |  |  |  |  |  |  |  |  |  |  |  |  |  |  |  |  |  |  |  |  |  |  |  |  |  |  |  |  |  |  |  |  |  |  |  |  |  |  |  |  |  |  |  |  |  |  |  |  |  |  |  |  |  |  |  |  |  |  |  |  |  |  |  |  |  |  |  |  |  |  |  |  |  |  |  |  |  |  |  |  |  |  |  |  |  |  |  |  |  |  |  |  |  |  |  |  |  |  |  |  |  |  |  |  |  |  |  |  |  |  |  |  |  |  |  |  |  |  |  |  |  |  |  |  |  |  |  |  |  |  |  |  |  |  |  |  |  |  |  |  |  |  |  |  |  |  |  |  |  |  |  |  |  |  |  |  |  |  |  |  |  |  |  |  |  |  |  |  |  |  |  |  |  |  |  |  |  |  |  |  |  |  |  |  |  |  |  |  |  |  |  |  |  |  |  |  |  |  |  |  |  |  |  |  |  |  |  |  |  |  |  |  |  |  |  |  |  |  |  |  |  |  |  |  |  |  |  |  |  |  |  |  |  |  |  |  |  |  |  |  |  |  |  |  |  |  |  |  |  |  |  |  |  |  |  |  |  |  |  |  |  |  |  |  |  |  |  |  |  |  |  |  |  |  |  |  |
| --- | --- | --- | --- | --- | --- | --- | --- | --- | --- | --- | --- | --- | --- | --- | --- | --- | --- | --- | --- | --- | --- | --- | --- | --- | --- | --- | --- | --- | --- | --- | --- | --- | --- | --- | --- | --- | --- | --- | --- | --- | --- | --- | --- | --- | --- | --- | --- | --- | --- | --- | --- | --- | --- | --- | --- | --- | --- | --- | --- | --- | --- | --- | --- | --- | --- | --- | --- | --- | --- | --- | --- | --- | --- | --- | --- | --- | --- | --- | --- | --- | --- | --- | --- | --- | --- | --- | --- | --- | --- | --- | --- | --- | --- | --- | --- | --- | --- | --- | --- | --- | --- | --- | --- | --- | --- | --- | --- | --- | --- | --- | --- | --- | --- | --- | --- | --- | --- | --- | --- | --- | --- | --- | --- | --- | --- | --- | --- | --- | --- | --- | --- | --- | --- | --- | --- | --- | --- | --- | --- | --- | --- | --- | --- | --- | --- | --- | --- | --- | --- | --- | --- | --- | --- | --- | --- | --- | --- | --- | --- | --- | --- | --- | --- | --- | --- | --- | --- | --- | --- | --- | --- | --- | --- | --- | --- | --- | --- | --- | --- | --- | --- | --- | --- | --- | --- | --- | --- | --- | --- | --- | --- | --- | --- | --- | --- | --- | --- | --- | --- | --- | --- | --- | --- | --- | --- | --- | --- | --- | --- | --- | --- | --- | --- | --- | --- | --- | --- | --- | --- | --- | --- | --- | --- | --- | --- | --- | --- | --- | --- | --- | --- | --- | --- | --- | --- | --- | --- | --- | --- | --- | --- | --- | --- | --- | --- | --- | --- | --- | --- | --- | --- | --- | --- | --- | --- | --- | --- | --- | --- | --- | --- | --- | --- | --- | --- | --- | --- | --- | --- | --- | --- | --- | --- | --- | --- | --- | --- | --- | --- | --- | --- | --- | --- | --- | --- | --- | --- | --- | --- | --- | --- | --- | --- | --- | --- | --- | --- | --- | --- | --- | --- | --- | --- | --- | --- | --- | --- | --- | --- | --- | --- | --- | --- | --- | --- | --- | --- | --- | --- | --- | --- | --- | --- | --- | --- | --- | --- | --- | --- | --- | --- | --- | --- | --- | --- | --- | --- | --- | --- | --- | --- | --- | --- |
| A | |  |  |  | | --- | --- | --- | | a  (in PyDSTool.Symbolic) | ADMC\_ODEsystem'  (in PyDSTool.Generator) | arcsinh  (in PyDSTool.Toolbox.NineML) | | Abs  (in PyDSTool) | AICCriterion  (in PyDSTool.Toolbox.optimizers.criterion.information\_criteria) | arcsinh  (in PyDSTool.Toolbox.dataanalysis) | | Abs  (in PyDSTool.ModelSpec') | alg\_spec  (in PyDSTool.Toolbox.model\_primitives) | arcsinh  (in PyDSTool.Toolbox.phaseplane) | | Abs  (in PyDSTool.Symbolic) | all()  (in B\_Check) | arcsinh  (in PyDSTool.Toolbox.synthetic\_data) | | Abs  (in PyDSTool.Toolbox.ActivationFuncs) | all()  (in PyDSTool.Toolbox.optimizers.criterion.composite\_criteria) | arcsinh  (in PyDSTool.Toolbox.syntheticdata) | | Abs  (in PyDSTool.Toolbox.DSSRT\_tools) | all\_args\_list  (in PyDSTool.PyCont.Continuation) | arcsinh  (in matplotlib.pylab) | | Abs  (in PyDSTool.Toolbox.InputProfile) | all\_curve\_types  (in PyDSTool.PyCont.Continuation) | arctan  (in PyDSTool.PyCont.ContClass') | | Abs  (in PyDSTool.Toolbox.ModelHelper) | all\_curve\_types  (in PyDSTool.PyCont.Plotting) | arctan  (in PyDSTool.Symbolic) | | Abs  (in PyDSTool.Toolbox.NineML) | all\_curve\_types  (in PyDSTool.PyCont.misc) | arctan  (in PyDSTool.Toolbox.NineML) | | Abs  (in PyDSTool.Toolbox.adjointPRC) | all\_point\_types  (in PyDSTool.PyCont.Continuation) | arctan  (in PyDSTool.Toolbox.dataanalysis) | | Abs  (in PyDSTool.Toolbox.dataanalysis) | all\_point\_types  (in PyDSTool.PyCont.Plotting) | arctan  (in PyDSTool.Toolbox.phaseplane) | | Abs  (in PyDSTool.Toolbox.fracdim) | all\_point\_types  (in PyDSTool.PyCont.misc) | arctan  (in PyDSTool.Toolbox.synthetic\_data) | | Abs  (in PyDSTool.Toolbox.makeSloppyModel) | allCompTypes()  (in PyDSTool.ModelSpec') | arctan  (in PyDSTool.Toolbox.syntheticdata) | | Abs  (in PyDSTool.Toolbox.neuralcomp) | allimports  (in PyDSTool.Generator) | arctan  (in matplotlib.pylab) | | Abs  (in PyDSTool.Toolbox.phaseplane) | allmathnames  (in PyDSTool.ModelSpec') | arctan2  (in PyDSTool.PyCont.ContClass') | | Abs  (in PyDSTool.Toolbox.synthetic\_data) | allmathnames  (in PyDSTool.Symbolic) | arctan2  (in PyDSTool.Symbolic) | | Abs  (in PyDSTool.Toolbox.syntheticdata) | allmathnames  (in PyDSTool.Toolbox.NineML) | arctan2  (in PyDSTool.Toolbox.NineML) | | absolute  (in PyDSTool.PyCont.ContClass') | allmathnames  (in PyDSTool.Toolbox.dataanalysis) | arctan2  (in PyDSTool.Toolbox.dataanalysis) | | absolute  (in PyDSTool.Toolbox.ActivationFuncs) | allmathnames  (in PyDSTool.Toolbox.phaseplane) | arctan2  (in PyDSTool.Toolbox.phaseplane) | | absolute  (in PyDSTool.Toolbox.DSSRT\_tools) | allmathnames  (in PyDSTool.Toolbox.synthetic\_data) | arctan2  (in PyDSTool.Toolbox.synthetic\_data) | | absolute  (in PyDSTool.Toolbox.InputProfile) | allmathnames  (in PyDSTool.Toolbox.syntheticdata) | arctan2  (in PyDSTool.Toolbox.syntheticdata) | | absolute  (in PyDSTool.Toolbox.ModelHelper) | allmathnames\_symbolic  (in PyDSTool.ModelSpec') | arctan2  (in matplotlib.pylab) | | absolute  (in PyDSTool.Toolbox.NineML) | allmathnames\_symbolic  (in PyDSTool.Symbolic) | arctanh  (in PyDSTool.PyCont.ContClass') | | absolute  (in PyDSTool.Toolbox.adjointPRC) | allmathnames\_symbolic  (in PyDSTool.Toolbox.NineML) | arctanh  (in PyDSTool.Symbolic) | | absolute  (in PyDSTool.Toolbox.dataanalysis) | allmathnames\_symbolic  (in PyDSTool.Toolbox.dataanalysis) | arctanh  (in PyDSTool.Toolbox.NineML) | | absolute  (in PyDSTool.Toolbox.fracdim) | allmathnames\_symbolic  (in PyDSTool.Toolbox.phaseplane) | arctanh  (in PyDSTool.Toolbox.dataanalysis) | | absolute  (in PyDSTool.Toolbox.makeSloppyModel) | allmathnames\_symbolic  (in PyDSTool.Toolbox.synthetic\_data) | arctanh  (in PyDSTool.Toolbox.phaseplane) | | absolute  (in PyDSTool.Toolbox.neuralcomp) | allmathnames\_symbolic  (in PyDSTool.Toolbox.syntheticdata) | arctanh  (in PyDSTool.Toolbox.synthetic\_data) | | absolute  (in PyDSTool.Toolbox.phaseplane) | allODEgens  (in PyDSTool.Toolbox.ModelHelper) | arctanh  (in PyDSTool.Toolbox.syntheticdata) | | absolute  (in PyDSTool.Toolbox.synthetic\_data) | allODEgens  (in PyDSTool.Toolbox.NineML) | arctanh  (in matplotlib.pylab) | | absolute  (in PyDSTool.Toolbox.syntheticdata) | allODEgens  (in PyDSTool.Toolbox.makeSloppyModel) | args  (in PyDSTool.common) | | absolute  (in PyDSTool) | ALLOW\_THREADS  (in PyDSTool) | argstr  (in PyDSTool.Symbolic) | | absolute  (in matplotlib.pylab) | ALLOW\_THREADS  (in PyDSTool.PyCont.ContClass') | array\_bounds\_check()  (in PyDSTool.common) | | AbsoluteParametersCriterion  (in PyDSTool.Toolbox.optimizers.criterion.criteria) | ALLOW\_THREADS  (in PyDSTool.Toolbox.ActivationFuncs) | arraymax()  (in PyDSTool.common) | | AbsoluteValueCriterion  (in PyDSTool.Toolbox.optimizers.criterion.criteria) | ALLOW\_THREADS  (in PyDSTool.Toolbox.DSSRT\_tools) | arrayToPointset()  (in PyDSTool.Points) | | accrueCompTypes()  (in PyDSTool.ModelSpec') | ALLOW\_THREADS  (in PyDSTool.Toolbox.InputProfile) | as\_absolute\_ratios()  (in PyDSTool.Toolbox.dssrt) | | Acos  (in PyDSTool) | ALLOW\_THREADS  (in PyDSTool.Toolbox.ModelHelper) | as\_relative\_ratios()  (in PyDSTool.Toolbox.dssrt) | | Acos  (in PyDSTool.ModelSpec') | ALLOW\_THREADS  (in PyDSTool.Toolbox.NineML) | Asin  (in PyDSTool) | | Acos  (in PyDSTool.Symbolic) | ALLOW\_THREADS  (in PyDSTool.Toolbox.adjointPRC) | Asin  (in PyDSTool.ModelSpec') | | acos  (in PyDSTool.Symbolic) | ALLOW\_THREADS  (in PyDSTool.Toolbox.dataanalysis) | Asin  (in PyDSTool.Symbolic) | | Acos  (in PyDSTool.Toolbox.ActivationFuncs) | ALLOW\_THREADS  (in PyDSTool.Toolbox.fracdim) | asin  (in PyDSTool.Symbolic) | | Acos  (in PyDSTool.Toolbox.DSSRT\_tools) | ALLOW\_THREADS  (in PyDSTool.Toolbox.makeSloppyModel) | Asin  (in PyDSTool.Toolbox.ActivationFuncs) | | Acos  (in PyDSTool.Toolbox.InputProfile) | ALLOW\_THREADS  (in PyDSTool.Toolbox.neuralcomp) | Asin  (in PyDSTool.Toolbox.DSSRT\_tools) | | Acos  (in PyDSTool.Toolbox.ModelHelper) | ALLOW\_THREADS  (in PyDSTool.Toolbox.phaseplane) | Asin  (in PyDSTool.Toolbox.InputProfile) | | Acos  (in PyDSTool.Toolbox.NineML) | ALLOW\_THREADS  (in PyDSTool.Toolbox.synthetic\_data) | Asin  (in PyDSTool.Toolbox.ModelHelper) | | Acos  (in PyDSTool.Toolbox.adjointPRC) | ALLOW\_THREADS  (in PyDSTool.Toolbox.syntheticdata) | Asin  (in PyDSTool.Toolbox.NineML) | | Acos  (in PyDSTool.Toolbox.dataanalysis) | ALLOW\_THREADS  (in matplotlib.pylab) | Asin  (in PyDSTool.Toolbox.adjointPRC) | | Acos  (in PyDSTool.Toolbox.fracdim) | alphabet\_chars\_RE  (in PyDSTool.parseUtils) | Asin  (in PyDSTool.Toolbox.dataanalysis) | | Acos  (in PyDSTool.Toolbox.makeSloppyModel) | always\_feature  (in PyDSTool.MProject) | Asin  (in PyDSTool.Toolbox.fracdim) | | Acos  (in PyDSTool.Toolbox.neuralcomp) | and\_op  (in PyDSTool.common) | Asin  (in PyDSTool.Toolbox.makeSloppyModel) | | Acos  (in PyDSTool.Toolbox.phaseplane) | AndComposition  (in PyDSTool.Toolbox.optimizers.criterion.composite\_criteria) | Asin  (in PyDSTool.Toolbox.neuralcomp) | | Acos  (in PyDSTool.Toolbox.synthetic\_data) | angle\_to\_vertical()  (in PyDSTool.Toolbox.phaseplane) | Asin  (in PyDSTool.Toolbox.phaseplane) | | Acos  (in PyDSTool.Toolbox.syntheticdata) | any()  (in PyDSTool.Toolbox.optimizers.criterion.composite\_criteria) | Asin  (in PyDSTool.Toolbox.synthetic\_data) | | acosh  (in PyDSTool.Symbolic) | API  (in PyDSTool.FuncSpec') | Asin  (in PyDSTool.Toolbox.syntheticdata) | | activateBounds()  (in GeneratorConstructor) | API  (in PyDSTool.Generator.ADMC\_ODEsystem') | asinh  (in PyDSTool.Symbolic) | | activateBounds()  (in ModelConstructor) | API  (in PyDSTool.Generator.Dopri\_ODEsystem') | ast2shortlist()  (in PyDSTool.parseUtils) | | ActivationFuncs  (in PyDSTool.Toolbox) | API  (in PyDSTool.Generator.EmbeddedSysGen') | ast2string()  (in PyDSTool.parseUtils) | | adaptive\_last\_step\_modifier  (in PyDSTool.Toolbox.optimizers.line\_search) | API  (in PyDSTool.Generator.Euler\_ODEsystem') | Atan  (in PyDSTool) | | AdaptiveLastStepModifier  (in PyDSTool.Toolbox.optimizers.line\_search.adaptive\_last\_step\_modifier) | API  (in PyDSTool.Generator.ExplicitFnGen') | Atan  (in PyDSTool.ModelSpec') | | add  (in PyDSTool.PyCont.ContClass') | API  (in PyDSTool.Generator.ExtrapolateTable') | Atan  (in PyDSTool.Symbolic) | | add  (in PyDSTool.Toolbox.ActivationFuncs) | API  (in PyDSTool.Generator.ImplicitFnGen') | atan  (in PyDSTool.Symbolic) | | add  (in PyDSTool.Toolbox.DSSRT\_tools) | API  (in PyDSTool.Generator.InterpolateTable') | Atan  (in PyDSTool.Toolbox.ActivationFuncs) | | add  (in PyDSTool.Toolbox.InputProfile) | API  (in PyDSTool.Generator.LookupTable') | Atan  (in PyDSTool.Toolbox.DSSRT\_tools) | | add  (in PyDSTool.Toolbox.ModelHelper) | API  (in PyDSTool.Generator.MapSystem') | Atan  (in PyDSTool.Toolbox.InputProfile) | | add  (in PyDSTool.Toolbox.NineML) | API  (in PyDSTool.Generator.ODEsystem') | Atan  (in PyDSTool.Toolbox.ModelHelper) | | add  (in PyDSTool.Toolbox.adjointPRC) | API  (in PyDSTool.Generator.Radau\_ODEsystem') | Atan  (in PyDSTool.Toolbox.NineML) | | add  (in PyDSTool.Toolbox.dataanalysis) | API  (in PyDSTool.Generator.Vode\_ODEsystem') | Atan  (in PyDSTool.Toolbox.adjointPRC) | | add  (in PyDSTool.Toolbox.fracdim) | API  (in PyDSTool.Interval') | Atan  (in PyDSTool.Toolbox.dataanalysis) | | add  (in PyDSTool.Toolbox.makeSloppyModel) | API  (in PyDSTool.ModelConstructor') | Atan  (in PyDSTool.Toolbox.fracdim) | | add  (in PyDSTool.Toolbox.neuralcomp) | API  (in PyDSTool.ModelSpec') | Atan  (in PyDSTool.Toolbox.makeSloppyModel) | | add  (in PyDSTool.Toolbox.phaseplane) | API  (in PyDSTool.Symbolic) | Atan  (in PyDSTool.Toolbox.neuralcomp) | | add  (in PyDSTool.Toolbox.synthetic\_data) | API  (in PyDSTool.Toolbox.NineML) | Atan  (in PyDSTool.Toolbox.phaseplane) | | add  (in PyDSTool.Toolbox.syntheticdata) | API  (in PyDSTool.Toolbox.dataanalysis) | Atan  (in PyDSTool.Toolbox.synthetic\_data) | | add  (in PyDSTool) | API  (in PyDSTool.Toolbox.event\_driven\_simulator) | Atan  (in PyDSTool.Toolbox.syntheticdata) | | add  (in matplotlib.pylab) | API  (in PyDSTool.Toolbox.phaseplane) | Atan2  (in PyDSTool) | | add()  (in GenSpecHelper) | API  (in PyDSTool.Toolbox.synthetic\_data) | Atan2  (in PyDSTool.ModelSpec') | | add()  (in GenTransform) | API  (in PyDSTool.Toolbox.syntheticdata) | Atan2  (in PyDSTool.Symbolic) | | add()  (in MReg) | API  (in PyDSTool.Trajectory') | atan2  (in PyDSTool.Symbolic) | | add()  (in ModelManager) | API  (in PyDSTool.Variable') | Atan2  (in PyDSTool.Toolbox.ActivationFuncs) | | add()  (in ModelTransform) | API  (in PyDSTool.common) | Atan2  (in PyDSTool.Toolbox.DSSRT\_tools) | | add()  (in MDescriptor) | API  (in PyDSTool.parseUtils) | Atan2  (in PyDSTool.Toolbox.InputProfile) | | add()  (in LeafComponent) | API  (in PyDSTool.utils) | Atan2  (in PyDSTool.Toolbox.ModelHelper) | | add()  (in ModelSpec) | API\_class  (in PyDSTool.common) | Atan2  (in PyDSTool.Toolbox.NineML) | | add()  (in phaseplane) | APPEND  (in PyDSTool.fixedpickle) | Atan2  (in PyDSTool.Toolbox.adjointPRC) | | add\_spec()  (in ModelLibrary) | append()  (in Pointset) | Atan2  (in PyDSTool.Toolbox.dataanalysis) | | add\_transition()  (in FSM) | append()  (in PiecewisePolynomial) | Atan2  (in PyDSTool.Toolbox.fracdim) | | add\_transition()  (in ObjFSM) | APPENDS  (in PyDSTool.fixedpickle) | Atan2  (in PyDSTool.Toolbox.makeSloppyModel) | | add\_transition\_any()  (in FSM) | APPROX  (in PyDSTool.Toolbox.dssrt) | Atan2  (in PyDSTool.Toolbox.neuralcomp) | | add\_transition\_list()  (in FSM) | arccos  (in PyDSTool.PyCont.ContClass') | Atan2  (in PyDSTool.Toolbox.phaseplane) | | add\_xi()  (in BarycentricInterpolator) | arccos  (in PyDSTool.Symbolic) | Atan2  (in PyDSTool.Toolbox.synthetic\_data) | | addArgToCalls()  (in PyDSTool.parseUtils) | arccos  (in PyDSTool.Toolbox.NineML) | Atan2  (in PyDSTool.Toolbox.syntheticdata) | | addAuxFn()  (in auxfnDBclass) | arccos  (in PyDSTool.Toolbox.dataanalysis) | atanh  (in PyDSTool.Symbolic) | | addConnxnTarget()  (in ModelSpec) | arccos  (in PyDSTool.Toolbox.phaseplane) | atEndPoint()  (in Interval) | | addEvents()  (in GeneratorConstructor) | arccos  (in PyDSTool.Toolbox.synthetic\_data) | augment\_3\_vector()  (in PyDSTool.Toolbox.mechmatlib) | | addEvents()  (in ModelConstructor) | arccos  (in PyDSTool.Toolbox.syntheticdata) | augment\_3x3\_matrix()  (in PyDSTool.Toolbox.mechmatlib) | | addEvtPars()  (in Generator) | arccos  (in matplotlib.pylab) | auto\_list  (in ContClass) | | addFeatures()  (in ModelConstructor) | arccosh  (in PyDSTool.PyCont.ContClass') | auto\_list  (in PyDSTool.PyCont.ContClass') | | addFunctions()  (in GeneratorConstructor) | arccosh  (in PyDSTool.Symbolic) | auto\_point\_types  (in PyDSTool.PyCont.Continuation) | | addFunctions()  (in ModelConstructor) | arccosh  (in PyDSTool.Toolbox.NineML) | auxfn\_container  (in PyDSTool.Generator.baseclasses) | | addlabel()  (in Point) | arccosh  (in PyDSTool.Toolbox.dataanalysis) | auxfnDBclass  (in PyDSTool.parseUtils) | | addlabel()  (in Pointset) | arccosh  (in PyDSTool.Toolbox.phaseplane) | AuxFunc()  (in integrator) | | addMethods()  (in Event) | arccosh  (in PyDSTool.Toolbox.synthetic\_data) | AuxVars()  (in Dopri\_ODEsystem) | | addMethods()  (in Euler\_ODEsystem) | arccosh  (in PyDSTool.Toolbox.syntheticdata) | AuxVars()  (in Euler\_ODEsystem) | | addMethods()  (in ExplicitFnGen) | arccosh  (in matplotlib.pylab) | AuxVars()  (in ExplicitFnGen) | | addMethods()  (in MapSystem) | arcsin  (in PyDSTool.PyCont.ContClass') | AuxVars()  (in MapSystem) | | addMethods()  (in ODEsystem) | arcsin  (in PyDSTool.Symbolic) | AuxVars()  (in ODEsystem) | | addMethods()  (in Vode\_ODEsystem) | arcsin  (in PyDSTool.Toolbox.NineML) | AuxVars()  (in Radau\_ODEsystem) | | addMethods()  (in Variable) | arcsin  (in PyDSTool.Toolbox.dataanalysis) | AuxVars()  (in Vode\_ODEsystem) | | addModelInfo()  (in ModelConstructor) | arcsin  (in PyDSTool.Toolbox.phaseplane) | AuxVars()  (in GeneratorInterface) | | AddTestFunction  (in PyDSTool.PyCont.TestFunc) | arcsin  (in PyDSTool.Toolbox.synthetic\_data) | AuxVars()  (in ModelInterface) | | addToQ()  (in Event) | arcsin  (in PyDSTool.Toolbox.syntheticdata) | AuxVars()  (in HybridModel) | | adjointPRC  (in PyDSTool.Toolbox) | arcsin  (in matplotlib.pylab) | AuxVars()  (in NonHybridModel) | | adjointPRC()  (in PyDSTool.Toolbox.adjointPRC) | arcsinh  (in PyDSTool.PyCont.ContClass') |  | | ADMC\_ODEsystem  (in PyDSTool.Generator.ADMC\_ODEsystem') | arcsinh  (in PyDSTool.Symbolic) |  | |

  
  

| Home | Trees | Indices | Help | | PyDSTool | | --- | |
| --- | --- | --- | --- | --- | --- |

|  |  |
| --- | --- |
| Generated by Epydoc 3.0.1 on Fri May 4 15:24:00 2012 | http://epydoc.sourceforge.net |
