# Supplementary material for: Hybrid Models and Biological Model Reduction with PyDSTool
Source: PLoS Comput Biol. 2012 Aug 9;8(8):e1002628. doi: 10.1371/journal.pcbi.1002628 (PMC3415397; doi:10.1371/journal.pcbi.1002628)
Supplement: Text S4 — Complete source code for the PyDSTool package (version 0.88.120504). Includes API documentation and help files linking to web pages. This file is identical to the current public release on Sourceforge.net. (ZIP) [file pcbi.1002628.s004.zip › PyDSTool/html/matplotlib.pylab-module.html]

xml version="1.0" encoding="ascii"?


matplotlib.pylab


| Home | Trees | Indices | Help | | PyDSTool | | --- | |
| --- | --- | --- | --- | --- | --- |

|  |  |  |  |
| --- | --- | --- | --- |
| Package matplotlib :: Module pylab | |  | | --- | | [hide private] | | [frames] | no frames] | |

# Module pylab

source code

```
This is a procedural interface to the matplotlib object-oriented
plotting library.

The following plotting commands are provided; the majority have
Matlab(TM) analogs and similar argument.

_Plotting commands
  acorr     - plot the autocorrelation function
  annotate  - annotate something in the figure
  arrow     - add an arrow to the axes
  axes      - Create a new axes
  axhline   - draw a horizontal line across axes
  axvline   - draw a vertical line across axes
  axhspan   - draw a horizontal bar across axes
  axvspan   - draw a vertical bar across axes
  axis      - Set or return the current axis limits
  bar       - make a bar chart
  barh      - a horizontal bar chart
  broken_barh - a set of horizontal bars with gaps
  box       - set the axes frame on/off state
  boxplot   - make a box and whisker plot
  cla       - clear current axes
  clabel    - label a contour plot
  clf       - clear a figure window
  clim      - adjust the color limits of the current image
  close     - close a figure window
  colorbar  - add a colorbar to the current figure
  cohere    - make a plot of coherence
  contour   - make a contour plot
  contourf  - make a filled contour plot
  csd       - make a plot of cross spectral density
  delaxes   - delete an axes from the current figure
  draw      - Force a redraw of the current figure
  errorbar  - make an errorbar graph
  figlegend - make legend on the figure rather than the axes
  figimage  - make a figure image
  figtext   - add text in figure coords
  figure   - create or change active figure
  fill     - make filled polygons
  findobj  - recursively find all objects matching some criteria
  gca      - return the current axes
  gcf      - return the current figure
  gci      - get the current image, or None
  getp      - get a graphics property
  grid     - set whether gridding is on
  hist     - make a histogram
  hold     - set the axes hold state
  ioff     - turn interaction mode off
  ion      - turn interaction mode on
  isinteractive - return True if interaction mode is on
  imread   - load image file into array
  imsave   - save array as an image file
  imshow   - plot image data
  ishold   - return the hold state of the current axes
  legend   - make an axes legend
  loglog   - a log log plot
  matshow  - display a matrix in a new figure preserving aspect
  pcolor   - make a pseudocolor plot
  pcolormesh - make a pseudocolor plot using a quadrilateral mesh
  pie      - make a pie chart
  plot     - make a line plot
  plot_date - plot dates
  plotfile  - plot column data from an ASCII tab/space/comma delimited file
  pie      - pie charts
  polar    - make a polar plot on a PolarAxes
  psd      - make a plot of power spectral density
  quiver   - make a direction field (arrows) plot
  rc       - control the default params
  rgrids   - customize the radial grids and labels for polar
  savefig  - save the current figure
  scatter  - make a scatter plot
  setp      - set a graphics property
  semilogx - log x axis
  semilogy - log y axis
  show     - show the figures
  specgram - a spectrogram plot
  spy      - plot sparsity pattern using markers or image
  stem     - make a stem plot
  subplot  - make a subplot (numrows, numcols, axesnum)
  subplots_adjust - change the params controlling the subplot positions of current figure
  subplot_tool - launch the subplot configuration tool
  suptitle   - add a figure title
  table    - add a table to the plot
  text     - add some text at location x,y to the current axes
  thetagrids - customize the radial theta grids and labels for polar
  title    - add a title to the current axes
  xcorr   - plot the autocorrelation function of x and y
  xlim     - set/get the xlimits
  ylim     - set/get the ylimits
  xticks   - set/get the xticks
  yticks   - set/get the yticks
  xlabel   - add an xlabel to the current axes
  ylabel   - add a ylabel to the current axes

  autumn - set the default colormap to autumn
  bone   - set the default colormap to bone
  cool   - set the default colormap to cool
  copper - set the default colormap to copper
  flag   - set the default colormap to flag
  gray   - set the default colormap to gray
  hot    - set the default colormap to hot
  hsv    - set the default colormap to hsv
  jet    - set the default colormap to jet
  pink   - set the default colormap to pink
  prism  - set the default colormap to prism
  spring - set the default colormap to spring
  summer - set the default colormap to summer
  winter - set the default colormap to winter
  spectral - set the default colormap to spectral

_Event handling

  connect - register an event handler
  disconnect - remove a connected event handler

_Matrix commands

  cumprod   - the cumulative product along a dimension
  cumsum    - the cumulative sum along a dimension
  detrend   - remove the mean or besdt fit line from an array
  diag      - the k-th diagonal of matrix
  diff      - the n-th differnce of an array
  eig       - the eigenvalues and eigen vectors of v
  eye       - a matrix where the k-th diagonal is ones, else zero
  find      - return the indices where a condition is nonzero
  fliplr    - flip the rows of a matrix up/down
  flipud    - flip the columns of a matrix left/right
  linspace  - a linear spaced vector of N values from min to max inclusive
  logspace  - a log spaced vector of N values from min to max inclusive
  meshgrid  - repeat x and y to make regular matrices
  ones      - an array of ones
  rand      - an array from the uniform distribution [0,1]
  randn     - an array from the normal distribution
  rot90     - rotate matrix k*90 degress counterclockwise
  squeeze   - squeeze an array removing any dimensions of length 1
  tri       - a triangular matrix
  tril      - a lower triangular matrix
  triu      - an upper triangular matrix
  vander    - the Vandermonde matrix of vector x
  svd       - singular value decomposition
  zeros     - a matrix of zeros

_Probability

  levypdf   - The levy probability density function from the char. func.
  normpdf   - The Gaussian probability density function
  rand      - random numbers from the uniform distribution
  randn     - random numbers from the normal distribution

_Statistics

  amax       - the maximum along dimension m
  amin       - the minimum along dimension m
  corrcoef  - correlation coefficient
  cov       - covariance matrix
  mean      - the mean along dimension m
  median    - the median along dimension m
  norm      - the norm of vector x
  prod      - the product along dimension m
  ptp       - the max-min along dimension m
  std       - the standard deviation along dimension m
  asum       - the sum along dimension m

_Time series analysis

  bartlett  - M-point Bartlett window
  blackman  - M-point Blackman window
  cohere    - the coherence using average periodiogram
  csd       - the cross spectral density using average periodiogram
  fft       - the fast Fourier transform of vector x
  hamming   - M-point Hamming window
  hanning   - M-point Hanning window
  hist      - compute the histogram of x
  kaiser    - M length Kaiser window
  psd       - the power spectral density using average periodiogram
  sinc      - the sinc function of array x

_Dates

  date2num  - convert python datetimes to numeric representation
  drange    - create an array of numbers for date plots
  num2date  - convert numeric type (float days since 0001) to datetime

_Other

  angle     - the angle of a complex array
  griddata  - interpolate irregularly distributed data to a regular grid
  load      - Deprecated--please use loadtxt.
  loadtxt   - load ASCII data into array.
  polyfit   - fit x, y to an n-th order polynomial
  polyval   - evaluate an n-th order polynomial
  roots     - the roots of the polynomial coefficients in p
  save      - Deprecated--please use savetxt.
  savetxt   - save an array to an ASCII file.
  trapz     - trapezoidal integration

__end
```

---

**Version:**
1.4.1


|  |  |  |  |
| --- | --- | --- | --- |
| |  |  | | --- | --- | | Functions | [hide private] | | |
|  | |  |  | | --- | --- | | beta(a, b, size=None)  The Beta distribution over ``[0, 1]``. | source code | |
|  | |  |  | | --- | --- | | binomial(n, p, size=None)  Draw samples from a binomial distribution. | source code | |
|  | |  |  | | --- | --- | | bytes(length)  Return random bytes. | source code | |
|  | |  |  | | --- | --- | | chisquare(df, size=None)  Draw samples from a chi-square distribution. | source code | |
|  | |  |  | | --- | --- | | exponential(scale=1.0, size=None)  Exponential distribution. | source code | |
|  | |  |  | | --- | --- | | f(dfnum, dfden, size=None)  Draw samples from a F distribution. | source code | |
|  | |  |  | | --- | --- | | gamma(shape, scale=1.0, size=None)  Draw samples from a Gamma distribution. | source code | |
|  | |  |  | | --- | --- | | geometric(p, size=None)  Draw samples from the geometric distribution. | source code | |
|  | |  |  | | --- | --- | | get\_state()  Return a tuple representing the internal state of the generator. | source code | |
|  | |  |  | | --- | --- | | gumbel(loc=0.0, scale=1.0, size=None)  Gumbel distribution. | source code | |
|  | |  |  | | --- | --- | | hypergeometric(ngood, nbad, nsample, size=None)  Draw samples from a Hypergeometric distribution. | source code | |
|  | |  |  | | --- | --- | | laplace(loc=0.0, scale=1.0, size=None)  Draw samples from the Laplace or double exponential distribution with specified location (or mean) and scale (decay). | source code | |
|  | |  |  | | --- | --- | | load(\*args, \*\*kwargs)  pylab no longer provides a load function, though the old pylab function is still available as matplotlib.mlab.load (you can refer to it in pylab as "mlab.load"). | source code | |
|  | |  |  | | --- | --- | | logistic(loc=0.0, scale=1.0, size=None)  Draw samples from a Logistic distribution. | source code | |
|  | |  |  | | --- | --- | | lognormal(mean=0.0, sigma=1.0, size=None)  Return samples drawn from a log-normal distribution. | source code | |
|  | |  |  | | --- | --- | | logseries(p, size=None)  Draw samples from a Logarithmic Series distribution. | source code | |
|  | |  |  | | --- | --- | | multinomial(n, pvals, size=None)  Draw samples from a multinomial distribution. | source code | |
|  | |  |  | | --- | --- | | multivariate\_normal(mean, cov, size=...)  Draw random samples from a multivariate normal distribution. | source code | |
|  | |  |  | | --- | --- | | negative\_binomial(n, p, size=None)  Draw samples from a negative\_binomial distribution. | source code | |
|  | |  |  | | --- | --- | | noncentral\_chisquare(df, nonc, size=None)  Draw samples from a noncentral chi-square distribution. | source code | |
|  | |  |  | | --- | --- | | noncentral\_f(dfnum, dfden, nonc, size=None)  Draw samples from the noncentral F distribution. | source code | |
|  | |  |  | | --- | --- | | normal(loc=0.0, scale=1.0, size=None)  Draw random samples from a normal (Gaussian) distribution. | source code | |
|  | |  |  | | --- | --- | | pareto(a, size=None)  Draw samples from a Pareto distribution with specified shape. | source code | |
|  | |  |  | | --- | --- | | permutation(x)  Randomly permute a sequence, or return a permuted range. | source code | |
|  | |  |  | | --- | --- | | poisson(lam=1.0, size=None)  Draw samples from a Poisson distribution. | source code | |
|  | |  |  | | --- | --- | | power(a, size=None)  Draws samples in [0, 1] from a power distribution with positive exponent a - 1. | source code | |
|  | |  |  | | --- | --- | | rand(d0, d1, dn, ...)  Random values in a given shape. | source code | |
|  | |  |  | | --- | --- | | randint(low, high=None, size=None)  Return random integers from `low` (inclusive) to `high` (exclusive). | source code | |
|  | |  |  | | --- | --- | | randn(d1=..., dn=..., ...)  Return a sample (or samples) from the "standard normal" distribution. | source code | |
|  | |  |  | | --- | --- | | random(size=None)  Return random floats in the half-open interval [0.0, 1.0). | source code | |
|  | |  |  | | --- | --- | | random\_integers(low, high=None, size=None)  Return random integers between `low` and `high`, inclusive. | source code | |
|  | |  |  | | --- | --- | | random\_sample(size=None)  Return random floats in the half-open interval [0.0, 1.0). | source code | |
|  | |  |  | | --- | --- | | ranf(size=None)  Return random floats in the half-open interval [0.0, 1.0). | source code | |
|  | |  |  | | --- | --- | | rayleigh(scale=1.0, size=None)  Draw samples from a Rayleigh distribution. | source code | |
|  | |  |  | | --- | --- | | sample(size=None)  Return random floats in the half-open interval [0.0, 1.0). | source code | |
|  | |  |  | | --- | --- | | save(\*args, \*\*kwargs)  pylab no longer provides a save function, though the old pylab function is still available as matplotlib.mlab.save (you can still refer to it in pylab as "mlab.save"). | source code | |
|  | |  |  | | --- | --- | | seed(seed=None)  Seed the generator. | source code | |
|  | |  |  | | --- | --- | | set\_state(state)  Set the internal state of the generator from a tuple. | source code | |
|  | |  |  | | --- | --- | | shuffle(x)  Modify a sequence in-place by shuffling its contents. | source code | |
|  | |  |  | | --- | --- | | standard\_cauchy(size=None)  Standard Cauchy distribution with mode = 0. | source code | |
|  | |  |  | | --- | --- | | standard\_exponential(size=None)  Draw samples from the standard exponential distribution. | source code | |
|  | |  |  | | --- | --- | | standard\_gamma(shape, size=None)  Draw samples from a Standard Gamma distribution. | source code | |
|  | |  |  | | --- | --- | | standard\_normal(size=None)  Returns samples from a Standard Normal distribution (mean=0, stdev=1). | source code | |
|  | |  |  | | --- | --- | | standard\_t(df, size=None)  Standard Student's t distribution with df degrees of freedom. | source code | |
|  | |  |  | | --- | --- | | triangular(left, mode, right, size=None)  Draw samples from the triangular distribution. | source code | |
|  | |  |  | | --- | --- | | uniform(low=0.0, high=1.0, size=1)  Draw samples from a uniform distribution. | source code | |
|  | |  |  | | --- | --- | | vonmises(mu=0.0, kappa=1.0, size=None)  Draw samples from a von Mises distribution. | source code | |
|  | |  |  | | --- | --- | | wald(mean, scale, size=None)  Draw samples from a Wald, or Inverse Gaussian, distribution. | source code | |
|  | |  |  | | --- | --- | | weibull(a, size=None)  Weibull distribution. | source code | |
|  | |  |  | | --- | --- | | zipf(a, size=None)  Draw samples from a Zipf distribution. | source code | |


|  |  |  |  |
| --- | --- | --- | --- |
| |  |  | | --- | --- | | Variables | [hide private] | | |
|  | ALLOW\_THREADS = `1` |
|  | BUFSIZE = `10000` |
|  | CLIP = `0` |
|  | DAILY = `3` |
|  | ERR\_CALL = `3` |
|  | ERR\_DEFAULT = `0` |
|  | ERR\_DEFAULT2 = `2084` |
|  | ERR\_IGNORE = `0` |
|  | ERR\_LOG = `5` |
|  | ERR\_PRINT = `4` |
|  | ERR\_RAISE = `2` |
|  | ERR\_WARN = `1` |
|  | FLOATING\_POINT\_SUPPORT = `1` |
|  | FPE\_DIVIDEBYZERO = `1` |
|  | FPE\_INVALID = `8` |
|  | FPE\_OVERFLOW = `2` |
|  | FPE\_UNDERFLOW = `4` |
|  | FR = `FR` |
|  | False\_ = `False` |
|  | HOURLY = `4` |
|  | Inf = `inf` |
|  | Infinity = `inf` |
|  | MAXDIMS = `32` |
|  | MINUTELY = `5` |
|  | MO = `MO` |
|  | MONTHLY = `1` |
|  | NAN = `nan` |
|  | NINF = `-inf` |
|  | NZERO = `-0.0` |
|  | NaN = `nan` |
|  | PINF = `inf` |
|  | PZERO = `0.0` |
|  | RAISE = `2` |
|  | SA = `SA` |
|  | SECONDLY = `6` |
|  | SHIFT\_DIVIDEBYZERO = `0` |
|  | SHIFT\_INVALID = `9` |
|  | SHIFT\_OVERFLOW = `3` |
|  | SHIFT\_UNDERFLOW = `6` |
|  | SU = `SU` |
|  | ScalarType = `(<type 'int'>, <type 'float'>, <type 'complex'>, ...` |
|  | TH = `TH` |
|  | TU = `TU` |
|  | True\_ = `True` |
|  | UFUNC\_BUFSIZE\_DEFAULT = `10000` |
|  | UFUNC\_PYVALS\_NAME = `'UFUNC_PYVALS'` |
|  | WE = `WE` |
|  | WEEKLY = `2` |
|  | WRAP = `1` |
|  | YEARLY = `0` |
|  | absolute = `<ufunc 'absolute'>` |
|  | add = `<ufunc 'add'>` |
|  | arccos = `<ufunc 'arccos'>` |
|  | arccosh = `<ufunc 'arccosh'>` |
|  | arcsin = `<ufunc 'arcsin'>` |
|  | arcsinh = `<ufunc 'arcsinh'>` |
|  | arctan = `<ufunc 'arctan'>` |
|  | arctan2 = `<ufunc 'arctan2'>` |
|  | arctanh = `<ufunc 'arctanh'>` |
|  | bitwise\_and = `<ufunc 'bitwise_and'>` |
|  | bitwise\_not = `<ufunc 'invert'>` |
|  | bitwise\_or = `<ufunc 'bitwise_or'>` |
|  | bitwise\_xor = `<ufunc 'bitwise_xor'>` |
|  | c\_ = `<numpy.lib.index_tricks.CClass object at 0x1154330>` |
|  | cast = `{<type 'numpy.int64'>: <function <lambda> at 0x10a8bb0>...` |
|  | ceil = `<ufunc 'ceil'>` |
|  | colorbar\_doc = `'\n\nAdd a colorbar to a plot.\n\nFunction sign...` |
|  | conj = `<ufunc 'conjugate'>` |
|  | conjugate = `<ufunc 'conjugate'>` |
|  | copysign = `<ufunc 'copysign'>` |
|  | cos = `<ufunc 'cos'>` |
|  | cosh = `<ufunc 'cosh'>` |
|  | deg2rad = `<ufunc 'deg2rad'>` |
|  | degrees = `<ufunc 'degrees'>` |
|  | divide = `<ufunc 'divide'>` |
|  | e = `2.71828182846` |
|  | equal = `<ufunc 'equal'>` |
|  | exp = `<ufunc 'exp'>` |
|  | exp2 = `<ufunc 'exp2'>` |
|  | expm1 = `<ufunc 'expm1'>` |
|  | fabs = `<ufunc 'fabs'>` |
|  | floor = `<ufunc 'floor'>` |
|  | floor\_divide = `<ufunc 'floor_divide'>` |
|  | fmax = `<ufunc 'fmax'>` |
|  | fmin = `<ufunc 'fmin'>` |
|  | fmod = `<ufunc 'fmod'>` |
|  | frexp = `<ufunc 'frexp'>` |
|  | greater = `<ufunc 'greater'>` |
|  | greater\_equal = `<ufunc 'greater_equal'>` |
|  | hypot = `<ufunc 'hypot'>` |
|  | index\_exp = `<numpy.lib.index_tricks.IndexExpression object at ...` |
|  | inf = `inf` |
|  | infty = `inf` |
|  | invert = `<ufunc 'invert'>` |
|  | isfinite = `<ufunc 'isfinite'>` |
|  | isinf = `<ufunc 'isinf'>` |
|  | isnan = `<ufunc 'isnan'>` |
|  | ldexp = `<ufunc 'ldexp'>` |
|  | left\_shift = `<ufunc 'left_shift'>` |
|  | less = `<ufunc 'less'>` |
|  | less\_equal = `<ufunc 'less_equal'>` |
|  | little\_endian = `True` |
|  | log = `<ufunc 'log'>` |
|  | log10 = `<ufunc 'log10'>` |
|  | log1p = `<ufunc 'log1p'>` |
|  | logaddexp = `<ufunc 'logaddexp'>` |
|  | logaddexp2 = `<ufunc 'logaddexp2'>` |
|  | logical\_and = `<ufunc 'logical_and'>` |
|  | logical\_not = `<ufunc 'logical_not'>` |
|  | logical\_or = `<ufunc 'logical_or'>` |
|  | logical\_xor = `<ufunc 'logical_xor'>` |
|  | maximum = `<ufunc 'maximum'>` |
|  | mgrid = `<numpy.lib.index_tricks.nd_grid object at 0x113d490>` |
|  | minimum = `<ufunc 'minimum'>` |
|  | mod = `<ufunc 'remainder'>` |
|  | modf = `<ufunc 'modf'>` |
|  | multiply = `<ufunc 'multiply'>` |
|  | nan = `nan` |
|  | nbytes = `{<type 'numpy.int64'>: 8, <type 'numpy.int16'>: 2, <t...` |
|  | negative = `<ufunc 'negative'>` |
|  | newaxis = `None` |
|  | nextafter = `<ufunc 'nextafter'>` |
|  | not\_equal = `<ufunc 'not_equal'>` |
|  | ogrid = `<numpy.lib.index_tricks.nd_grid object at 0x113dbb0>` |
|  | ones\_like = `<ufunc 'ones_like'>` |
|  | pi = `3.14159265359` |
|  | r\_ = `<numpy.lib.index_tricks.RClass object at 0x11542f0>` |
|  | rad2deg = `<ufunc 'rad2deg'>` |
|  | radians = `<ufunc 'radians'>` |
|  | rcParams = `{'figure.subplot.right': 0.90000000000000002, 'math...` |
|  | rcParamsDefault = `{'figure.subplot.right': 0.90000000000000002...` |
|  | reciprocal = `<ufunc 'reciprocal'>` |
|  | remainder = `<ufunc 'remainder'>` |
|  | right\_shift = `<ufunc 'right_shift'>` |
|  | rint = `<ufunc 'rint'>` |
|  | s\_ = `<numpy.lib.index_tricks.IndexExpression object at 0x11543f0>` |
|  | sctypeDict = `{0: <type 'numpy.bool_'>, 1: <type 'numpy.int8'>,...` |
|  | sctypeNA = `{'?': 'Bool', 'B': 'UInt8', 'Bool': <type 'numpy.bo...` |
|  | sctypes = `{'complex': [<type 'numpy.complex64'>, <type 'numpy....` |
|  | sign = `<ufunc 'sign'>` |
|  | signbit = `<ufunc 'signbit'>` |
|  | sin = `<ufunc 'sin'>` |
|  | sinh = `<ufunc 'sinh'>` |
|  | spacing = `<ufunc 'spacing'>` |
|  | sqrt = `<ufunc 'sqrt'>` |
|  | square = `<ufunc 'square'>` |
|  | subtract = `<ufunc 'subtract'>` |
|  | tan = `<ufunc 'tan'>` |
|  | tanh = `<ufunc 'tanh'>` |
|  | true\_divide = `<ufunc 'true_divide'>` |
|  | trunc = `<ufunc 'trunc'>` |
|  | typeDict = `{0: <type 'numpy.bool_'>, 1: <type 'numpy.int8'>, 2...` |
|  | typeNA = `{'?': 'Bool', 'B': 'UInt8', 'Bool': <type 'numpy.bool...` |
|  | typecodes = `{'All': '?bhilqpBHILQPfdgFDGSUVOMm', 'AllFloat': '...` |


|  |  |  |  |
| --- | --- | --- | --- |
| |  |  | | --- | --- | | Function Details | [hide private] | | |

|  |  |  |
| --- | --- | --- |
| |  |  | | --- | --- | | beta(a, b, size=None) | source code |  ``` The Beta distribution over ``[0, 1]``.  The Beta distribution is a special case of the Dirichlet distribution, and is related to the Gamma distribution.  It has the probability distribution function  .. math:: f(x; a,b) = \frac{1}{B(\alpha, \beta)} x^{\alpha - 1}                                                  (1 - x)^{\beta - 1},  where the normalisation, B, is the beta function,  .. math:: B(\alpha, \beta) = \int_0^1 t^{\alpha - 1}                              (1 - t)^{\beta - 1} dt.  It is often seen in Bayesian inference and order statistics.  Parameters ---------- a : float     Alpha, non-negative. b : float     Beta, non-negative. size : tuple of ints, optional     The number of samples to draw.  The ouput is packed according to     the size given.  Returns ------- out : ndarray     Array of the given shape, containing values drawn from a     Beta distribution. ``` |

|  |  |  |
| --- | --- | --- |
| |  |  | | --- | --- | | binomial(n, p, size=None) | source code |  ``` Draw samples from a binomial distribution.  Samples are drawn from a Binomial distribution with specified parameters, n trials and p probability of success where n an integer > 0 and p is in the interval [0,1]. (n may be input as a float, but it is truncated to an integer in use)  Parameters ---------- n : float (but truncated to an integer)         parameter, > 0. p : float         parameter, >= 0 and <=1. size : {tuple, int}     Output shape.  If the given shape is, e.g., ``(m, n, k)``, then     ``m * n * k`` samples are drawn.  Returns ------- samples : {ndarray, scalar}           where the values are all integers in  [0, n].  See Also -------- scipy.stats.distributions.binom : probability density function,     distribution or cumulative density function, etc.  Notes ----- The probability density for the Binomial distribution is  .. math:: P(N) = \binom{n}{N}p^N(1-p)^{n-N},  where :math:`n` is the number of trials, :math:`p` is the probability of success, and :math:`N` is the number of successes.  When estimating the standard error of a proportion in a population by using a random sample, the normal distribution works well unless the product p*n <=5, where p = population proportion estimate, and n = number of samples, in which case the binomial distribution is used instead. For example, a sample of 15 people shows 4 who are left handed, and 11 who are right handed. Then p = 4/15 = 27%. 0.27*15 = 4, so the binomial distribution should be used in this case.  References ---------- .. [1] Dalgaard, Peter, "Introductory Statistics with R",        Springer-Verlag, 2002. .. [2] Glantz, Stanton A. "Primer of Biostatistics.", McGraw-Hill,        Fifth Edition, 2002. .. [3] Lentner, Marvin, "Elementary Applied Statistics", Bogden        and Quigley, 1972. .. [4] Weisstein, Eric W. "Binomial Distribution." From MathWorld--A        Wolfram Web Resource.        http://mathworld.wolfram.com/BinomialDistribution.html .. [5] Wikipedia, "Binomial-distribution",        http://en.wikipedia.org/wiki/Binomial_distribution  Examples -------- Draw samples from the distribution:  >>> n, p = 10, .5 # number of trials, probability of each trial >>> s = np.random.binomial(n, p, 1000) # result of flipping a coin 10 times, tested 1000 times.  A real world example. A company drills 9 wild-cat oil exploration wells, each with an estimated probability of success of 0.1. All nine wells fail. What is the probability of that happening?  Let's do 20,000 trials of the model, and count the number that generate zero positive results.  >>> sum(np.random.binomial(9,0.1,20000)==0)/20000. answer = 0.38885, or 38%. ``` |

|  |  |  |
| --- | --- | --- |
| |  |  | | --- | --- | | bytes(length) | source code |  ``` Return random bytes.  Parameters ---------- length : int     Number of random bytes.  Returns ------- out : str     String of length `N`.  Examples -------- >>> np.random.bytes(10) ' eh\x85\x022SZ\xbf\xa4' #random ``` |

|  |  |  |
| --- | --- | --- |
| |  |  | | --- | --- | | chisquare(df, size=None) | source code |  ``` Draw samples from a chi-square distribution.  When `df` independent random variables, each with standard normal distributions (mean 0, variance 1), are squared and summed, the resulting distribution is chi-square (see Notes).  This distribution is often used in hypothesis testing.  Parameters ---------- df : int      Number of degrees of freedom. size : tuple of ints, int, optional      Size of the returned array.  By default, a scalar is      returned.  Returns ------- output : ndarray     Samples drawn from the distribution, packed in a `size`-shaped     array.  Raises ------ ValueError     When `df` <= 0 or when an inappropriate `size` (e.g. ``size=-1``)     is given.  Notes ----- The variable obtained by summing the squares of `df` independent, standard normally distributed random variables:  .. math:: Q = \sum_{i=0}^{\mathtt{df}} X^2_i  is chi-square distributed, denoted  .. math:: Q \sim \chi^2_k.  The probability density function of the chi-squared distribution is  .. math:: p(x) = \frac{(1/2)^{k/2}}{\Gamma(k/2)}                  x^{k/2 - 1} e^{-x/2},  where :math:`\Gamma` is the gamma function,  .. math:: \Gamma(x) = \int_0^{-\infty} t^{x - 1} e^{-t} dt.  References ---------- .. [1] NIST/SEMATECH e-Handbook of Statistical Methods,        http://www.itl.nist.gov/div898/handbook/eda/section3/eda3666.htm .. [2] Wikipedia, "Chi-square distribution",        http://en.wikipedia.org/wiki/Chi-square_distribution  Examples -------- >>> np.random.chisquare(2,4) array([ 1.89920014,  9.00867716,  3.13710533,  5.62318272]) ``` |

|  |  |  |
| --- | --- | --- |
| |  |  | | --- | --- | | exponential(scale=1.0, size=None) | source code |  ``` Exponential distribution.  Its probability density function is  .. math:: f(x; \frac{1}{\beta}) = \frac{1}{\beta} \exp(-\frac{x}{\beta}),  for ``x > 0`` and 0 elsewhere. :math:`\beta` is the scale parameter, which is the inverse of the rate parameter :math:`\lambda = 1/\beta`. The rate parameter is an alternative, widely used parameterization of the exponential distribution [3]_.  The exponential distribution is a continuous analogue of the geometric distribution.  It describes many common situations, such as the size of raindrops measured over many rainstorms [1]_, or the time between page requests to Wikipedia [2]_.  Parameters ---------- scale : float     The scale parameter, :math:`\beta = 1/\lambda`. size : tuple of ints     Number of samples to draw.  The output is shaped     according to `size`.  References ---------- .. [1] Peyton Z. Peebles Jr., "Probability, Random Variables and        Random Signal Principles", 4th ed, 2001, p. 57. .. [2] "Poisson Process", Wikipedia,        http://en.wikipedia.org/wiki/Poisson_process .. [3] "Exponential Distribution, Wikipedia,        http://en.wikipedia.org/wiki/Exponential_distribution ``` |

|  |  |  |
| --- | --- | --- |
| |  |  | | --- | --- | | f(dfnum, dfden, size=None) | source code |  ``` Draw samples from a F distribution.  Samples are drawn from an F distribution with specified parameters, `dfnum` (degrees of freedom in numerator) and `dfden` (degrees of freedom in denominator), where both parameters should be greater than zero.  The random variate of the F distribution (also known as the Fisher distribution) is a continuous probability distribution that arises in ANOVA tests, and is the ratio of two chi-square variates.  Parameters ---------- dfnum : float     Degrees of freedom in numerator. Should be greater than zero. dfden : float     Degrees of freedom in denominator. Should be greater than zero. size : {tuple, int}, optional     Output shape.  If the given shape is, e.g., ``(m, n, k)``,     then ``m * n * k`` samples are drawn. By default only one sample     is returned.  Returns ------- samples : {ndarray, scalar}     Samples from the Fisher distribution.  See Also -------- scipy.stats.distributions.f : probability density function,     distribution or cumulative density function, etc.  Notes -----  The F statistic is used to compare in-group variances to between-group variances. Calculating the distribution depends on the sampling, and so it is a function of the respective degrees of freedom in the problem.  The variable `dfnum` is the number of samples minus one, the between-groups degrees of freedom, while `dfden` is the within-groups degrees of freedom, the sum of the number of samples in each group minus the number of groups.  References ---------- .. [1] Glantz, Stanton A. "Primer of Biostatistics.", McGraw-Hill,        Fifth Edition, 2002. .. [2] Wikipedia, "F-distribution",        http://en.wikipedia.org/wiki/F-distribution  Examples -------- An example from Glantz[1], pp 47-40. Two groups, children of diabetics (25 people) and children from people without diabetes (25 controls). Fasting blood glucose was measured, case group had a mean value of 86.1, controls had a mean value of 82.2. Standard deviations were 2.09 and 2.49 respectively. Are these data consistent with the null hypothesis that the parents diabetic status does not affect their children's blood glucose levels? Calculating the F statistic from the data gives a value of 36.01.  Draw samples from the distribution:  >>> dfnum = 1. # between group degrees of freedom >>> dfden = 48. # within groups degrees of freedom >>> s = np.random.f(dfnum, dfden, 1000)  The lower bound for the top 1% of the samples is :  >>> sort(s)[-10] 7.61988120985  So there is about a 1% chance that the F statistic will exceed 7.62, the measured value is 36, so the null hypothesis is rejected at the 1% level. ``` |

|  |  |  |
| --- | --- | --- |
| |  |  | | --- | --- | | gamma(shape, scale=1.0, size=None) | source code |  ``` Draw samples from a Gamma distribution.  Samples are drawn from a Gamma distribution with specified parameters, `shape` (sometimes designated "k") and `scale` (sometimes designated "theta"), where both parameters are > 0.  Parameters ---------- shape : scalar > 0     The shape of the gamma distribution. scale : scalar > 0, optional     The scale of the gamma distribution.  Default is equal to 1. size : shape_tuple, optional     Output shape.  If the given shape is, e.g., ``(m, n, k)``, then     ``m * n * k`` samples are drawn.  Returns ------- out : ndarray, float     Returns one sample unless `size` parameter is specified.  See Also -------- scipy.stats.distributions.gamma : probability density function,     distribution or cumulative density function, etc.  Notes ----- The probability density for the Gamma distribution is  .. math:: p(x) = x^{k-1}\frac{e^{-x/\theta}}{\theta^k\Gamma(k)},  where :math:`k` is the shape and :math:`\theta` the scale, and :math:`\Gamma` is the Gamma function.  The Gamma distribution is often used to model the times to failure of electronic components, and arises naturally in processes for which the waiting times between Poisson distributed events are relevant.  References ---------- .. [1] Weisstein, Eric W. "Gamma Distribution." From MathWorld--A        Wolfram Web Resource.        http://mathworld.wolfram.com/GammaDistribution.html .. [2] Wikipedia, "Gamma-distribution",        http://en.wikipedia.org/wiki/Gamma-distribution  Examples -------- Draw samples from the distribution:  >>> shape, scale = 2., 2. # mean and dispersion >>> s = np.random.gamma(shape, scale, 1000)  Display the histogram of the samples, along with the probability density function:  >>> import matplotlib.pyplot as plt >>> import scipy.special as sps >>> count, bins, ignored = plt.hist(s, 50, normed=True) >>> y = bins**(shape-1)*(np.exp(-bins/scale) / ...                      (sps.gamma(shape)*scale**shape)) >>> plt.plot(bins, y, linewidth=2, color='r') >>> plt.show() ``` |

|  |  |  |
| --- | --- | --- |
| |  |  | | --- | --- | | geometric(p, size=None) | source code |  ``` Draw samples from the geometric distribution.  Bernoulli trials are experiments with one of two outcomes: success or failure (an example of such an experiment is flipping a coin).  The geometric distribution models the number of trials that must be run in order to achieve success.  It is therefore supported on the positive integers, ``k = 1, 2, ...``.  The probability mass function of the geometric distribution is  .. math:: f(k) = (1 - p)^{k - 1} p  where `p` is the probability of success of an individual trial.  Parameters ---------- p : float     The probability of success of an individual trial. size : tuple of ints     Number of values to draw from the distribution.  The output     is shaped according to `size`.  Returns ------- out : ndarray     Samples from the geometric distribution, shaped according to     `size`.  Examples -------- Draw ten thousand values from the geometric distribution, with the probability of an individual success equal to 0.35:  >>> z = np.random.geometric(p=0.35, size=10000)  How many trials succeeded after a single run?  >>> (z == 1).sum() / 10000. 0.34889999999999999 #random ``` |

|  |  |  |
| --- | --- | --- |
| |  |  | | --- | --- | | get\_state() | source code |  ``` Return a tuple representing the internal state of the generator.  For more details, see `set_state`.  Returns ------- out : tuple(str, ndarray of 624 uints, int, int, float)     The returned tuple has the following items:      1. the string 'MT19937'.     2. a 1-D array of 624 unsigned integer keys.     3. an integer ``pos``.     4. an integer ``has_gauss``.     5. a float ``cached_gaussian``.  See Also -------- set_state  Notes ----- `set_state` and `get_state` are not needed to work with any of the random distributions in NumPy. If the internal state is manually altered, the user should know exactly what he/she is doing. ``` |

|  |  |  |
| --- | --- | --- |
| |  |  | | --- | --- | | gumbel(loc=0.0, scale=1.0, size=None) | source code |  ``` Gumbel distribution.  Draw samples from a Gumbel distribution with specified location (or mean) and scale (or standard deviation).  The Gumbel (or Smallest Extreme Value (SEV) or the Smallest Extreme Value Type I) distribution is one of a class of Generalized Extreme Value (GEV) distributions used in modeling extreme value problems.  The Gumbel is a special case of the Extreme Value Type I distribution for maximums from distributions with "exponential-like" tails, it may be derived by considering a Gaussian process of measurements, and generating the pdf for the maximum values from that set of measurements (see examples).  Parameters ---------- loc : float     The location of the mode of the distribution. scale : float     The scale parameter of the distribution. size : tuple of ints     Output shape.  If the given shape is, e.g., ``(m, n, k)``, then     ``m * n * k`` samples are drawn.  See Also -------- scipy.stats.gumbel : probability density function,     distribution or cumulative density function, etc. weibull, scipy.stats.genextreme  Notes ----- The probability density for the Gumbel distribution is  .. math:: p(x) = \frac{e^{-(x - \mu)/ \beta}}{\beta} e^{ -e^{-(x - \mu)/           \beta}},  where :math:`\mu` is the mode, a location parameter, and :math:`\beta` is the scale parameter.  The Gumbel (named for German mathematician Emil Julius Gumbel) was used very early in the hydrology literature, for modeling the occurrence of flood events. It is also used for modeling maximum wind speed and rainfall rates.  It is a "fat-tailed" distribution - the probability of an event in the tail of the distribution is larger than if one used a Gaussian, hence the surprisingly frequent occurrence of 100-year floods. Floods were initially modeled as a Gaussian process, which underestimated the frequency of extreme events.  It is one of a class of extreme value distributions, the Generalized Extreme Value (GEV) distributions, which also includes the Weibull and Frechet.  The function has a mean of :math:`\mu + 0.57721\beta` and a variance of :math:`\frac{\pi^2}{6}\beta^2`.  References ---------- .. [1] Gumbel, E.J. (1958). Statistics of Extremes. Columbia University        Press. .. [2] Reiss, R.-D. and Thomas M. (2001), Statistical Analysis of Extreme        Values, from Insurance, Finance, Hydrology and Other Fields,        Birkhauser Verlag, Basel: Boston : Berlin. .. [3] Wikipedia, "Gumbel distribution",        http://en.wikipedia.org/wiki/Gumbel_distribution  Examples -------- Draw samples from the distribution:  >>> mu, beta = 0, 0.1 # location and scale >>> s = np.random.gumbel(mu, beta, 1000)  Display the histogram of the samples, along with the probability density function:  >>> import matplotlib.pyplot as plt >>> count, bins, ignored = plt.hist(s, 30, normed=True) >>> plt.plot(bins, (1/beta)*np.exp(-(bins - mu)/beta) ...          * np.exp( -np.exp( -(bins - mu) /beta) ), ...          linewidth=2, color='r') >>> plt.show()  Show how an extreme value distribution can arise from a Gaussian process and compare to a Gaussian:  >>> means = [] >>> maxima = [] >>> for i in range(0,1000) : ...    a = np.random.normal(mu, beta, 1000) ...    means.append(a.mean()) ...    maxima.append(a.max()) >>> count, bins, ignored = plt.hist(maxima, 30, normed=True) >>> beta = np.std(maxima)*np.pi/np.sqrt(6) >>> mu = np.mean(maxima) - 0.57721*beta >>> plt.plot(bins, (1/beta)*np.exp(-(bins - mu)/beta) ...          * np.exp(-np.exp(-(bins - mu)/beta)), ...          linewidth=2, color='r') >>> plt.plot(bins, 1/(beta * np.sqrt(2 * np.pi)) ...          * np.exp(-(bins - mu)**2 / (2 * beta**2)), ...          linewidth=2, color='g') >>> plt.show() ``` |

|  |  |  |
| --- | --- | --- |
| |  |  | | --- | --- | | hypergeometric(ngood, nbad, nsample, size=None) | source code |  ``` Draw samples from a Hypergeometric distribution.  Samples are drawn from a Hypergeometric distribution with specified parameters, ngood (ways to make a good selection), nbad (ways to make a bad selection), and nsample = number of items sampled, which is less than or equal to the sum ngood + nbad.  Parameters ---------- ngood : float (but truncated to an integer)         parameter, > 0. nbad  : float         parameter, >= 0. nsample  : float            parameter, > 0 and <= ngood+nbad size : {tuple, int}     Output shape.  If the given shape is, e.g., ``(m, n, k)``, then     ``m * n * k`` samples are drawn.  Returns ------- samples : {ndarray, scalar}           where the values are all integers in  [0, n].  See Also -------- scipy.stats.distributions.hypergeom : probability density function,     distribution or cumulative density function, etc.  Notes ----- The probability density for the Hypergeometric distribution is  .. math:: P(x) = \frac{\binom{m}{n}\binom{N-m}{n-x}}{\binom{N}{n}},  where :math:`0 \le x \le m` and :math:`n+m-N \le x \le n`  for P(x) the probability of x successes, n = ngood, m = nbad, and N = number of samples.  Consider an urn with black and white marbles in it, ngood of them black and nbad are white. If you draw nsample balls without replacement, then the Hypergeometric distribution describes the distribution of black balls in the drawn sample.  Note that this distribution is very similar to the Binomial distribution, except that in this case, samples are drawn without replacement, whereas in the Binomial case samples are drawn with replacement (or the sample space is infinite). As the sample space becomes large, this distribution approaches the Binomial.  References ---------- .. [1] Lentner, Marvin, "Elementary Applied Statistics", Bogden        and Quigley, 1972. .. [2] Weisstein, Eric W. "Hypergeometric Distribution." From        MathWorld--A Wolfram Web Resource.        http://mathworld.wolfram.com/HypergeometricDistribution.html .. [3] Wikipedia, "Hypergeometric-distribution",        http://en.wikipedia.org/wiki/Hypergeometric-distribution  Examples -------- Draw samples from the distribution:  >>> ngood, nbad, nsamp = 100, 2, 10 # number of good, number of bad, and number of samples >>> s = np.random.hypergeometric(ngood, nbad, nsamp, 1000) >>> hist(s) #   note that it is very unlikely to grab both bad items  Suppose you have an urn with 15 white and 15 black marbles. If you pull 15 marbles at random, how likely is it that 12 or more of them are one color?  >>> s = np.random.hypergeometric(15, 15, 15, 100000) >>> sum(s>=12)/100000. + sum(s<=3)/100000. #   answer = 0.003 ... pretty unlikely! ``` |

|  |  |  |
| --- | --- | --- |
| |  |  | | --- | --- | | laplace(loc=0.0, scale=1.0, size=None) | source code |  ``` Draw samples from the Laplace or double exponential distribution with specified location (or mean) and scale (decay).  The Laplace distribution is similar to the Gaussian/normal distribution, but is sharper at the peak and has fatter tails. It represents the difference between two independent, identically distributed exponential random variables.  Parameters ---------- loc : float     The position, :math:`\mu`, of the distribution peak. scale : float     :math:`\lambda`, the exponential decay.  Notes ----- It has the probability density function  .. math:: f(x; \mu, \lambda) = \frac{1}{2\lambda}                                \exp\left(-\frac{|x - \mu|}{\lambda}\right).  The first law of Laplace, from 1774, states that the frequency of an error can be expressed as an exponential function of the absolute magnitude of the error, which leads to the Laplace distribution. For many problems in Economics and Health sciences, this distribution seems to model the data better than the standard Gaussian distribution   References ---------- .. [1] Abramowitz, M. and Stegun, I. A. (Eds.). Handbook of Mathematical        Functions with Formulas, Graphs, and Mathematical Tables, 9th        printing.  New York: Dover, 1972.  .. [2] The Laplace distribution and generalizations        By Samuel Kotz, Tomasz J. Kozubowski, Krzysztof Podgorski,        Birkhauser, 2001.  .. [3] Weisstein, Eric W. "Laplace Distribution."        From MathWorld--A Wolfram Web Resource.        http://mathworld.wolfram.com/LaplaceDistribution.html  .. [4] Wikipedia, "Laplace distribution",        http://en.wikipedia.org/wiki/Laplace_distribution  Examples -------- Draw samples from the distribution  >>> loc, scale = 0., 1. >>> s = np.random.laplace(loc, scale, 1000)  Display the histogram of the samples, along with the probability density function:  >>> import matplotlib.pyplot as plt >>> count, bins, ignored = plt.hist(s, 30, normed=True) >>> x = np.arange(-8., 8., .01) >>> pdf = np.exp(-abs(x-loc/scale))/(2.*scale) >>> plt.plot(x, pdf)  Plot Gaussian for comparison:  >>> g = (1/(scale * np.sqrt(2 * np.pi)) *  ...      np.exp( - (x - loc)**2 / (2 * scale**2) )) >>> plt.plot(x,g) ``` |

|  |  |  |
| --- | --- | --- |
| |  |  | | --- | --- | | load(\*args, \*\*kwargs) | source code |   pylab no longer provides a load function, though the old pylab function is still available as matplotlib.mlab.load (you can refer to it in pylab as "mlab.load"). However, for plain text files, we recommend numpy.loadtxt, which was inspired by the old pylab.load but now has more features. For loading numpy arrays, we recommend numpy.load, and its analog numpy.save, which are available in pylab as np.load and np.save. |

|  |  |  |
| --- | --- | --- |
| |  |  | | --- | --- | | logistic(loc=0.0, scale=1.0, size=None) | source code |  ``` Draw samples from a Logistic distribution.  Samples are drawn from a Logistic distribution with specified parameters, loc (location or mean, also median), and scale (>0).  Parameters ---------- loc : float  scale : float > 0.  size : {tuple, int}     Output shape.  If the given shape is, e.g., ``(m, n, k)``, then     ``m * n * k`` samples are drawn.  Returns ------- samples : {ndarray, scalar}           where the values are all integers in  [0, n].  See Also -------- scipy.stats.distributions.logistic : probability density function,     distribution or cumulative density function, etc.  Notes ----- The probability density for the Logistic distribution is  .. math:: P(x) = P(x) = \frac{e^{-(x-\mu)/s}}{s(1+e^{-(x-\mu)/s})^2},  where :math:`\mu` = location and :math:`s` = scale.  The Logistic distribution is used in Extreme Value problems where it can act as a mixture of Gumbel distributions, in Epidemiology, and by the World Chess Federation (FIDE) where it is used in the Elo ranking system, assuming the performance of each player is a logistically distributed random variable.  References ---------- .. [1] Reiss, R.-D. and Thomas M. (2001), Statistical Analysis of Extreme        Values, from Insurance, Finance, Hydrology and Other Fields,        Birkhauser Verlag, Basel, pp 132-133. .. [2] Weisstein, Eric W. "Logistic Distribution." From        MathWorld--A Wolfram Web Resource.        http://mathworld.wolfram.com/LogisticDistribution.html .. [3] Wikipedia, "Logistic-distribution",        http://en.wikipedia.org/wiki/Logistic-distribution  Examples -------- Draw samples from the distribution:  >>> loc, scale = 10, 1 >>> s = np.random.logistic(loc, scale, 10000) >>> count, bins, ignored = plt.hist(s, bins=50)  #   plot against distribution  >>> def logist(x, loc, scale): ...     return exp((loc-x)/scale)/(scale*(1+exp((loc-x)/scale))**2) >>> plt.plot(bins, logist(bins, loc, scale)*count.max()/\ ... logist(bins, loc, scale).max()) >>> plt.show() ``` |

|  |  |  |
| --- | --- | --- |
| |  |  | | --- | --- | | lognormal(mean=0.0, sigma=1.0, size=None) | source code |  ``` Return samples drawn from a log-normal distribution.  Draw samples from a log-normal distribution with specified mean, standard deviation, and shape. Note that the mean and standard deviation are not the values for the distribution itself, but of the underlying normal distribution it is derived from.   Parameters ---------- mean : float     Mean value of the underlying normal distribution sigma : float, >0.     Standard deviation of the underlying normal distribution size : tuple of ints     Output shape.  If the given shape is, e.g., ``(m, n, k)``, then     ``m * n * k`` samples are drawn.  See Also -------- scipy.stats.lognorm : probability density function, distribution,     cumulative density function, etc.  Notes ----- A variable `x` has a log-normal distribution if `log(x)` is normally distributed.  The probability density function for the log-normal distribution is  .. math:: p(x) = \frac{1}{\sigma x \sqrt{2\pi}}                  e^{(-\frac{(ln(x)-\mu)^2}{2\sigma^2})}  where :math:`\mu` is the mean and :math:`\sigma` is the standard deviation of the normally distributed logarithm of the variable.  A log-normal distribution results if a random variable is the *product* of a large number of independent, identically-distributed variables in the same way that a normal distribution results if the variable is the *sum* of a large number of independent, identically-distributed variables (see the last example). It is one of the so-called "fat-tailed" distributions.  The log-normal distribution is commonly used to model the lifespan of units with fatigue-stress failure modes. Since this includes most mechanical systems, the log-normal distribution has widespread application.  It is also commonly used to model oil field sizes, species abundance, and latent periods of infectious diseases.  References ---------- .. [1] Eckhard Limpert, Werner A. Stahel, and Markus Abbt, "Log-normal        Distributions across the Sciences: Keys and Clues", May 2001        Vol. 51 No. 5 BioScience        http://stat.ethz.ch/~stahel/lognormal/bioscience.pdf .. [2] Reiss, R.D., Thomas, M.(2001), Statistical Analysis of Extreme        Values, Birkhauser Verlag, Basel, pp 31-32. .. [3] Wikipedia, "Lognormal distribution",        http://en.wikipedia.org/wiki/Lognormal_distribution  Examples -------- Draw samples from the distribution:  >>> mu, sigma = 3., 1. # mean and standard deviation >>> s = np.random.lognormal(mu, sigma, 1000)  Display the histogram of the samples, along with the probability density function:  >>> import matplotlib.pyplot as plt >>> count, bins, ignored = plt.hist(s, 100, normed=True, align='mid')  >>> x = np.linspace(min(bins), max(bins), 10000) >>> pdf = (np.exp(-(np.log(x) - mu)**2 / (2 * sigma**2)) ...        / (x * sigma * np.sqrt(2 * np.pi)))  >>> plt.plot(x, pdf, linewidth=2, color='r') >>> plt.axis('tight') >>> plt.show()  Demonstrate that taking the products of random samples from a uniform distribution can be fit well by a log-normal probability density function.  >>> # Generate a thousand samples: each is the product of 100 random >>> # values, drawn from a normal distribution. >>> b = [] >>> for i in range(1000): ...    a = 10. + np.random.random(100) ...    b.append(np.product(a))  >>> b = np.array(b) / np.min(b) # scale values to be positive  >>> count, bins, ignored = plt.hist(b, 100, normed=True, align='center')  >>> sigma = np.std(np.log(b)) >>> mu = np.mean(np.log(b))  >>> x = np.linspace(min(bins), max(bins), 10000) >>> pdf = (np.exp(-(np.log(x) - mu)**2 / (2 * sigma**2)) ...        / (x * sigma * np.sqrt(2 * np.pi)))  >>> plt.plot(x, pdf, color='r', linewidth=2) >>> plt.show() ``` |

|  |  |  |
| --- | --- | --- |
| |  |  | | --- | --- | | logseries(p, size=None) | source code |  ``` Draw samples from a Logarithmic Series distribution.  Samples are drawn from a Log Series distribution with specified parameter, p (probability, 0 < p < 1).  Parameters ---------- loc : float  scale : float > 0.  size : {tuple, int}     Output shape.  If the given shape is, e.g., ``(m, n, k)``, then     ``m * n * k`` samples are drawn.  Returns ------- samples : {ndarray, scalar}           where the values are all integers in  [0, n].  See Also -------- scipy.stats.distributions.logser : probability density function,     distribution or cumulative density function, etc.  Notes ----- The probability density for the Log Series distribution is  .. math:: P(k) = \frac{-p^k}{k \ln(1-p)},  where p = probability.  The Log Series distribution is frequently used to represent species richness and occurrence, first proposed by Fisher, Corbet, and Williams in 1943 [2].  It may also be used to model the numbers of occupants seen in cars [3].  References ---------- .. [1] Buzas, Martin A.; Culver, Stephen J.,  Understanding regional        species diversity through the log series distribution of        occurrences: BIODIVERSITY RESEARCH Diversity & Distributions,        Volume 5, Number 5, September 1999 , pp. 187-195(9). .. [2] Fisher, R.A,, A.S. Corbet, and C.B. Williams. 1943. The        relation between the number of species and the number of        individuals in a random sample of an animal population.        Journal of Animal Ecology, 12:42-58. .. [3] D. J. Hand, F. Daly, D. Lunn, E. Ostrowski, A Handbook of Small        Data Sets, CRC Press, 1994. .. [4] Wikipedia, "Logarithmic-distribution",        http://en.wikipedia.org/wiki/Logarithmic-distribution  Examples -------- Draw samples from the distribution:  >>> a = .6 >>> s = np.random.logseries(a, 10000) >>> count, bins, ignored = plt.hist(s)  #   plot against distribution  >>> def logseries(k, p): ...     return -p**k/(k*log(1-p)) >>> plt.plot(bins, logseries(bins, a)*count.max()/              logseries(bins, a).max(), 'r') >>> plt.show() ``` |

|  |  |  |
| --- | --- | --- |
| |  |  | | --- | --- | | multinomial(n, pvals, size=None) | source code |  ``` Draw samples from a multinomial distribution.  The multinomial distribution is a multivariate generalisation of the binomial distribution.  Take an experiment with one of ``p`` possible outcomes.  An example of such an experiment is throwing a dice, where the outcome can be 1 through 6.  Each sample drawn from the distribution represents `n` such experiments.  Its values, ``X_i = [X_0, X_1, ..., X_p]``, represent the number of times the outcome was ``i``.  Parameters ---------- n : int     Number of experiments. pvals : sequence of floats, length p     Probabilities of each of the ``p`` different outcomes.  These     should sum to 1 (however, the last element is always assumed to     account for the remaining probability, as long as     ``sum(pvals[:-1]) <= 1)``. size : tuple of ints     Given a `size` of ``(M, N, K)``, then ``M*N*K`` samples are drawn,     and the output shape becomes ``(M, N, K, p)``, since each sample     has shape ``(p,)``.  Examples -------- Throw a dice 20 times:  >>> np.random.multinomial(20, [1/6.]*6, size=1) array([[4, 1, 7, 5, 2, 1]])  It landed 4 times on 1, once on 2, etc.  Now, throw the dice 20 times, and 20 times again:  >>> np.random.multinomial(20, [1/6.]*6, size=2) array([[3, 4, 3, 3, 4, 3],        [2, 4, 3, 4, 0, 7]])  For the first run, we threw 3 times 1, 4 times 2, etc.  For the second, we threw 2 times 1, 4 times 2, etc.  A loaded dice is more likely to land on number 6:  >>> np.random.multinomial(100, [1/7.]*5) array([13, 16, 13, 16, 42]) ``` |

|  |  |  |
| --- | --- | --- |
| |  |  | | --- | --- | | multivariate\_normal(mean, cov, size=...) | source code |  ``` Draw random samples from a multivariate normal distribution.  The multivariate normal, multinormal or Gaussian distribution is a generalisation of the one-dimensional normal distribution to higher dimensions.  Such a distribution is specified by its mean and covariance matrix, which are analogous to the mean (average or "centre") and variance (standard deviation squared or "width") of the one-dimensional normal distribution.  Parameters ---------- mean : (N,) ndarray     Mean of the N-dimensional distribution. cov : (N,N) ndarray     Covariance matrix of the distribution. size : tuple of ints, optional     Given a shape of, for example, (m,n,k), m*n*k samples are     generated, and packed in an m-by-n-by-k arrangement.  Because each     sample is N-dimensional, the output shape is (m,n,k,N).  If no     shape is specified, a single sample is returned.  Returns ------- out : ndarray     The drawn samples, arranged according to `size`.  If the     shape given is (m,n,...), then the shape of `out` is is     (m,n,...,N).      In other words, each entry ``out[i,j,...,:]`` is an N-dimensional     value drawn from the distribution.  Notes ----- The mean is a coordinate in N-dimensional space, which represents the location where samples are most likely to be generated.  This is analogous to the peak of the bell curve for the one-dimensional or univariate normal distribution.  Covariance indicates the level to which two variables vary together. From the multivariate normal distribution, we draw N-dimensional samples, :math:`X = [x_1, x_2, ... x_N]`.  The covariance matrix element :math:`C_{ij}` is the covariance of :math:`x_i` and :math:`x_j`. The element :math:`C_{ii}` is the variance of :math:`x_i` (i.e. its "spread").  Instead of specifying the full covariance matrix, popular approximations include:    - Spherical covariance (`cov` is a multiple of the identity matrix)   - Diagonal covariance (`cov` has non-negative elements, and only on     the diagonal)  This geometrical property can be seen in two dimensions by plotting generated data-points:  >>> mean = [0,0] >>> cov = [[1,0],[0,100]] # diagonal covariance, points lie on x or y-axis  >>> import matplotlib.pyplot as plt >>> x,y = np.random.multivariate_normal(mean,cov,5000).T >>> plt.plot(x,y,'x'); plt.axis('equal'); plt.show()  Note that the covariance matrix must be non-negative definite.  References ---------- .. [1] A. Papoulis, "Probability, Random Variables, and Stochastic        Processes," 3rd ed., McGraw-Hill Companies, 1991 .. [2] R.O. Duda, P.E. Hart, and D.G. Stork, "Pattern Classification,"        2nd ed., Wiley, 2001.  Examples -------- >>> mean = (1,2) >>> cov = [[1,0],[1,0]] >>> x = np.random.multivariate_normal(mean,cov,(3,3)) >>> x.shape (3, 3, 2)  The following is probably true, given that 0.6 is roughly twice the standard deviation:  >>> print list( (x[0,0,:] - mean) < 0.6 ) [True, True] ``` |

|  |  |  |
| --- | --- | --- |
| |  |  | | --- | --- | | negative\_binomial(n, p, size=None) | source code |  ``` Draw samples from a negative_binomial distribution.  Samples are drawn from a negative_Binomial distribution with specified parameters, `n` trials and `p` probability of success where `n` is an integer > 0 and `p` is in the interval [0, 1].  Parameters ---------- n : int     Parameter, > 0. p : float     Parameter, >= 0 and <=1. size : int or tuple of ints     Output shape. If the given shape is, e.g., ``(m, n, k)``, then     ``m * n * k`` samples are drawn.  Returns ------- samples : int or ndarray of ints     Drawn samples.  Notes ----- The probability density for the Negative Binomial distribution is  .. math:: P(N;n,p) = \binom{N+n-1}{n-1}p^{n}(1-p)^{N},  where :math:`n-1` is the number of successes, :math:`p` is the probability of success, and :math:`N+n-1` is the number of trials.  The negative binomial distribution gives the probability of n-1 successes and N failures in N+n-1 trials, and success on the (N+n)th trial.  If one throws a die repeatedly until the third time a "1" appears, then the probability distribution of the number of non-"1"s that appear before the third "1" is a negative binomial distribution.  References ---------- .. [1] Weisstein, Eric W. "Negative Binomial Distribution." From        MathWorld--A Wolfram Web Resource.        http://mathworld.wolfram.com/NegativeBinomialDistribution.html .. [2] Wikipedia, "Negative binomial distribution",        http://en.wikipedia.org/wiki/Negative_binomial_distribution  Examples -------- Draw samples from the distribution:  A real world example. A company drills wild-cat oil exploration wells, each with an estimated probability of success of 0.1.  What is the probability of having one success for each successive well, that is what is the probability of a single success after drilling 5 wells, after 6 wells, etc.?  >>> s = np.random.negative_binomial(1, 0.1, 100000) >>> for i in range(1, 11): ...    probability = sum(s<i) / 100000. ...    print i, "wells drilled, probability of one success =", probability ``` |

|  |  |  |
| --- | --- | --- |
| |  |  | | --- | --- | | noncentral\_chisquare(df, nonc, size=None) | source code |  ``` Draw samples from a noncentral chi-square distribution.  The noncentral :math:`\chi^2` distribution is a generalisation of the :math:`\chi^2` distribution.  Parameters ---------- df : int     Degrees of freedom, should be >= 1. nonc : float     Non-centrality, should be > 0. size : int or tuple of ints     Shape of the output.  Notes ----- The probability density function for the noncentral Chi-square distribution is  .. math:: P(x;df,nonc) = \sum^{\infty}_{i=0}                        \frac{e^{-nonc/2}(nonc/2)^{i}}{i!}P_{Y_{df+2i}}(x),  where :math:`Y_{q}` is the Chi-square with q degrees of freedom.  In Delhi (2007), it is noted that the noncentral chi-square is useful in bombing and coverage problems, the probability of killing the point target given by the noncentral chi-squared distribution.  References ---------- .. [1] Delhi, M.S. Holla, "On a noncentral chi-square distribution in the        analysis of weapon systems effectiveness", Metrika, Volume 15,        Number 1 / December, 1970. .. [2] Wikipedia, "Noncentral chi-square distribution"        http://en.wikipedia.org/wiki/Noncentral_chi-square_distribution  Examples -------- Draw values from the distribution and plot the histogram  >>> import matplotlib.pyplot as plt >>> values = plt.hist(np.random.noncentral_chisquare(3, 20, 100000), ...                   bins=200, normed=True) >>> plt.show()  Draw values from a noncentral chisquare with very small noncentrality, and compare to a chisquare.  >>> plt.figure() >>> values = plt.hist(np.random.noncentral_chisquare(3, .0000001, 100000), ...                   bins=np.arange(0., 25, .1), normed=True) >>> values2 = plt.hist(np.random.chisquare(3, 100000), ...                    bins=np.arange(0., 25, .1), normed=True) >>> plt.plot(values[1][0:-1], values[0]-values2[0], 'ob') >>> plt.show()  Demonstrate how large values of non-centrality lead to a more symmetric distribution.  >>> plt.figure() >>> values = plt.hist(np.random.noncentral_chisquare(3, 20, 100000), ...                   bins=200, normed=True) >>> plt.show() ``` |

|  |  |  |
| --- | --- | --- |
| |  |  | | --- | --- | | noncentral\_f(dfnum, dfden, nonc, size=None) | source code |  ``` Draw samples from the noncentral F distribution.  Samples are drawn from an F distribution with specified parameters, `dfnum` (degrees of freedom in numerator) and `dfden` (degrees of freedom in denominator), where both parameters > 1. `nonc` is the non-centrality parameter.  Parameters ---------- dfnum : int     Parameter, should be > 1. dfden : int     Parameter, should be > 1. nonc : float     Parameter, should be >= 0. size : int or tuple of ints     Output shape. If the given shape is, e.g., ``(m, n, k)``, then     ``m * n * k`` samples are drawn.  Returns ------- samples : scalar or ndarray     Drawn samples.  Notes ----- When calculating the power of an experiment (power = probability of rejecting the null hypothesis when a specific alternative is true) the non-central F statistic becomes important.  When the null hypothesis is true, the F statistic follows a central F distribution. When the null hypothesis is not true, then it follows a non-central F statistic.  References ---------- Weisstein, Eric W. "Noncentral F-Distribution." From MathWorld--A Wolfram Web Resource.  http://mathworld.wolfram.com/NoncentralF-Distribution.html  Wikipedia, "Noncentral F distribution", http://en.wikipedia.org/wiki/Noncentral_F-distribution  Examples -------- In a study, testing for a specific alternative to the null hypothesis requires use of the Noncentral F distribution. We need to calculate the area in the tail of the distribution that exceeds the value of the F distribution for the null hypothesis.  We'll plot the two probability distributions for comparison.  >>> dfnum = 3 # between group deg of freedom >>> dfden = 20 # within groups degrees of freedom >>> nonc = 3.0 >>> nc_vals = np.random.noncentral_f(dfnum, dfden, nonc, 1000000) >>> NF = np.histogram(nc_vals, bins=50, normed=True) >>> c_vals = np.random.f(dfnum, dfden, 1000000) >>> F = np.histogram(c_vals, bins=50, normed=True) >>> plt.plot(F[1][1:], F[0]) >>> plt.plot(NF[1][1:], NF[0]) >>> plt.show() ``` |

|  |  |  |
| --- | --- | --- |
| |  |  | | --- | --- | | normal(loc=0.0, scale=1.0, size=None) | source code |  ``` Draw random samples from a normal (Gaussian) distribution.  The probability density function of the normal distribution, first derived by De Moivre and 200 years later by both Gauss and Laplace independently [2]_, is often called the bell curve because of its characteristic shape (see the example below).  The normal distributions occurs often in nature.  For example, it describes the commonly occurring distribution of samples influenced by a large number of tiny, random disturbances, each with its own unique distribution [2]_.  Parameters ---------- loc : float     Mean ("centre") of the distribution. scale : float     Standard deviation (spread or "width") of the distribution. size : tuple of ints     Output shape.  If the given shape is, e.g., ``(m, n, k)``, then     ``m * n * k`` samples are drawn.  See Also -------- scipy.stats.distributions.norm : probability density function,     distribution or cumulative density function, etc.  Notes ----- The probability density for the Gaussian distribution is  .. math:: p(x) = \frac{1}{\sqrt{ 2 \pi \sigma^2 }}                  e^{ - \frac{ (x - \mu)^2 } {2 \sigma^2} },  where :math:`\mu` is the mean and :math:`\sigma` the standard deviation. The square of the standard deviation, :math:`\sigma^2`, is called the variance.  The function has its peak at the mean, and its "spread" increases with the standard deviation (the function reaches 0.607 times its maximum at :math:`x + \sigma` and :math:`x - \sigma` [2]_).  This implies that `numpy.random.normal` is more likely to return samples lying close to the mean, rather than those far away.  References ---------- .. [1] Wikipedia, "Normal distribution",        http://en.wikipedia.org/wiki/Normal_distribution .. [2] P. R. Peebles Jr., "Central Limit Theorem" in "Probability, Random        Variables and Random Signal Principles", 4th ed., 2001,        pp. 51, 51, 125.  Examples -------- Draw samples from the distribution:  >>> mu, sigma = 0, 0.1 # mean and standard deviation >>> s = np.random.normal(mu, sigma, 1000)  Verify the mean and the variance:  >>> abs(mu - np.mean(s)) < 0.01 True  >>> abs(sigma - np.std(s, ddof=1)) < 0.01 True  Display the histogram of the samples, along with the probability density function:  >>> import matplotlib.pyplot as plt >>> count, bins, ignored = plt.hist(s, 30, normed=True) >>> plt.plot(bins, 1/(sigma * np.sqrt(2 * np.pi)) * ...                np.exp( - (bins - mu)**2 / (2 * sigma**2) ), ...          linewidth=2, color='r') >>> plt.show() ``` |

|  |  |  |
| --- | --- | --- |
| |  |  | | --- | --- | | pareto(a, size=None) | source code |  ``` Draw samples from a Pareto distribution with specified shape.  This is a simplified version of the Generalized Pareto distribution (available in SciPy), with the scale set to one and the location set to zero. Most authors default the location to one.  The Pareto distribution must be greater than zero, and is unbounded above. It is also known as the "80-20 rule".  In this distribution, 80 percent of the weights are in the lowest 20 percent of the range, while the other 20 percent fill the remaining 80 percent of the range.  Parameters ---------- shape : float, > 0.     Shape of the distribution. size : tuple of ints     Output shape.  If the given shape is, e.g., ``(m, n, k)``, then     ``m * n * k`` samples are drawn.  See Also -------- scipy.stats.distributions.genpareto.pdf : probability density function,     distribution or cumulative density function, etc.  Notes ----- The probability density for the Pareto distribution is  .. math:: p(x) = \frac{am^a}{x^{a+1}}  where :math:`a` is the shape and :math:`m` the location  The Pareto distribution, named after the Italian economist Vilfredo Pareto, is a power law probability distribution useful in many real world problems. Outside the field of economics it is generally referred to as the Bradford distribution. Pareto developed the distribution to describe the distribution of wealth in an economy.  It has also found use in insurance, web page access statistics, oil field sizes, and many other problems, including the download frequency for projects in Sourceforge [1].  It is one of the so-called "fat-tailed" distributions.   References ---------- .. [1] Francis Hunt and Paul Johnson, On the Pareto Distribution of        Sourceforge projects. .. [2] Pareto, V. (1896). Course of Political Economy. Lausanne. .. [3] Reiss, R.D., Thomas, M.(2001), Statistical Analysis of Extreme        Values, Birkhauser Verlag, Basel, pp 23-30. .. [4] Wikipedia, "Pareto distribution",        http://en.wikipedia.org/wiki/Pareto_distribution  Examples -------- Draw samples from the distribution:  >>> a, m = 3., 1. # shape and mode >>> s = np.random.pareto(a, 1000) + m  Display the histogram of the samples, along with the probability density function:  >>> import matplotlib.pyplot as plt >>> count, bins, ignored = plt.hist(s, 100, normed=True, align='center') >>> fit = a*m**a/bins**(a+1) >>> plt.plot(bins, max(count)*fit/max(fit),linewidth=2, color='r') >>> plt.show() ``` |

|  |  |  |
| --- | --- | --- |
| |  |  | | --- | --- | | permutation(x) | source code |  ``` Randomly permute a sequence, or return a permuted range.  Parameters ---------- x : int or array_like     If `x` is an integer, randomly permute ``np.arange(x)``.     If `x` is an array, make a copy and shuffle the elements     randomly.  Returns ------- out : ndarray     Permuted sequence or array range.  Examples -------- >>> np.random.permutation(10) array([1, 7, 4, 3, 0, 9, 2, 5, 8, 6])  >>> np.random.permutation([1, 4, 9, 12, 15]) array([15,  1,  9,  4, 12]) ``` |

|  |  |  |
| --- | --- | --- |
| |  |  | | --- | --- | | poisson(lam=1.0, size=None) | source code |  ``` Draw samples from a Poisson distribution.  The Poisson distribution is the limit of the Binomial distribution for large N.  Parameters ---------- lam : float     Expectation of interval, should be >= 0. size : int or tuple of ints, optional     Output shape. If the given shape is, e.g., ``(m, n, k)``, then     ``m * n * k`` samples are drawn.  Notes ----- The Poisson distribution  .. math:: f(k; \lambda)=\frac{\lambda^k e^{-\lambda}}{k!}  For events with an expected separation :math:`\lambda` the Poisson distribution :math:`f(k; \lambda)` describes the probability of :math:`k` events occurring within the observed interval :math:`\lambda`.  References ---------- .. [1] Weisstein, Eric W. "Poisson Distribution." From MathWorld--A Wolfram        Web Resource. http://mathworld.wolfram.com/PoissonDistribution.html .. [2] Wikipedia, "Poisson distribution",    http://en.wikipedia.org/wiki/Poisson_distribution  Examples -------- Draw samples from the distribution:  >>> import numpy as np >>> s = np.random.poisson(5, 10000)  Display histogram of the sample:  >>> import matplotlib.pyplot as plt >>> count, bins, ignored = plt.hist(s, 14, normed=True) >>> plt.show() ``` |

|  |  |  |
| --- | --- | --- |
| |  |  | | --- | --- | | power(a, size=None) | source code |  ``` Draws samples in [0, 1] from a power distribution with positive exponent a - 1.  Also known as the power function distribution.  Parameters ---------- a : float     parameter, > 0 size : tuple of ints     Output shape.  If the given shape is, e.g., ``(m, n, k)``, then             ``m * n * k`` samples are drawn.  Returns ------- samples : {ndarray, scalar}     The returned samples lie in [0, 1].  Raises ------ ValueError     If a<1.  Notes ----- The probability density function is  .. math:: P(x; a) = ax^{a-1}, 0 \le x \le 1, a>0.  The power function distribution is just the inverse of the Pareto distribution. It may also be seen as a special case of the Beta distribution.  It is used, for example, in modeling the over-reporting of insurance claims.  References ---------- .. [1] Christian Kleiber, Samuel Kotz, "Statistical size distributions        in economics and actuarial sciences", Wiley, 2003. .. [2] Heckert, N. A. and Filliben, James J. (2003). NIST Handbook 148:        Dataplot Reference Manual, Volume 2: Let Subcommands and Library        Functions", National Institute of Standards and Technology Handbook        Series, June 2003.        http://www.itl.nist.gov/div898/software/dataplot/refman2/auxillar/powpdf.pdf  Examples -------- Draw samples from the distribution:  >>> a = 5. # shape >>> samples = 1000 >>> s = np.random.power(a, samples)  Display the histogram of the samples, along with the probability density function:  >>> import matplotlib.pyplot as plt >>> count, bins, ignored = plt.hist(s, bins=30) >>> x = np.linspace(0, 1, 100) >>> y = a*x**(a-1.) >>> normed_y = samples*np.diff(bins)[0]*y >>> plt.plot(x, normed_y) >>> plt.show()  Compare the power function distribution to the inverse of the Pareto.  >>> from scipy import stats >>> rvs = np.random.power(5, 1000000) >>> rvsp = np.random.pareto(5, 1000000) >>> xx = np.linspace(0,1,100) >>> powpdf = stats.powerlaw.pdf(xx,5)  >>> plt.figure() >>> plt.hist(rvs, bins=50, normed=True) >>> plt.plot(xx,powpdf,'r-') >>> plt.title('np.random.power(5)')  >>> plt.figure() >>> plt.hist(1./(1.+rvsp), bins=50, normed=True) >>> plt.plot(xx,powpdf,'r-') >>> plt.title('inverse of 1 + np.random.pareto(5)')  >>> plt.figure() >>> plt.hist(1./(1.+rvsp), bins=50, normed=True) >>> plt.plot(xx,powpdf,'r-') >>> plt.title('inverse of stats.pareto(5)') ``` |

|  |  |  |
| --- | --- | --- |
| |  |  | | --- | --- | | rand(d0, d1, dn, ...) | source code |  ``` Random values in a given shape.  Create an array of the given shape and propagate it with random samples from a uniform distribution over ``[0, 1)``.  Parameters ---------- d0, d1, ..., dn : int     Shape of the output.  Returns ------- out : ndarray, shape ``(d0, d1, ..., dn)``     Random values.  See Also -------- random  Notes ----- This is a convenience function. If you want an interface that takes a shape-tuple as the first argument, refer to `random`.  Examples -------- >>> np.random.rand(3,2) array([[ 0.14022471,  0.96360618],  #random        [ 0.37601032,  0.25528411],  #random        [ 0.49313049,  0.94909878]]) #random ``` |

|  |  |  |
| --- | --- | --- |
| |  |  | | --- | --- | | randint(low, high=None, size=None) | source code |  ``` Return random integers from `low` (inclusive) to `high` (exclusive).  Return random integers from the "discrete uniform" distribution in the "half-open" interval [`low`, `high`). If `high` is None (the default), then results are from [0, `low`).  Parameters ---------- low : int     Lowest (signed) integer to be drawn from the distribution (unless     ``high=None``, in which case this parameter is the *highest* such     integer). high : int, optional     If provided, one above the largest (signed) integer to be drawn     from the distribution (see above for behavior if ``high=None``). size : int or tuple of ints, optional     Output shape. Default is None, in which case a single int is     returned.  Returns ------- out : int or ndarray of ints     `size`-shaped array of random integers from the appropriate     distribution, or a single such random int if `size` not provided.  See Also -------- random.random_integers : similar to `randint`, only for the closed     interval [`low`, `high`], and 1 is the lowest value if `high` is     omitted. In particular, this other one is the one to use to generate     uniformly distributed discrete non-integers.  Examples -------- >>> np.random.randint(2, size=10) array([1, 0, 0, 0, 1, 1, 0, 0, 1, 0]) >>> np.random.randint(1, size=10) array([0, 0, 0, 0, 0, 0, 0, 0, 0, 0])  Generate a 2 x 4 array of ints between 0 and 4, inclusive:  >>> np.random.randint(5, size=(2, 4)) array([[4, 0, 2, 1],        [3, 2, 2, 0]]) ``` |

|  |  |  |
| --- | --- | --- |
| |  |  | | --- | --- | | randn(d1=..., dn=..., ...) | source code |  ``` Return a sample (or samples) from the "standard normal" distribution.  If positive, int_like or int-convertible arguments are provided, `randn` generates an array of shape ``(d1, ..., dn)``, filled with random floats sampled from a univariate "normal" (Gaussian) distribution of mean 0 and variance 1 (if any of the :math:`d_i` are floats, they are first converted to integers by truncation). A single float randomly sampled from the distribution is returned if no argument is provided.  This is a convenience function.  If you want an interface that takes a tuple as the first argument, use `numpy.random.standard_normal` instead.  Parameters ---------- d1, ..., dn : `n` ints, optional     The dimensions of the returned array, should be all positive.  Returns ------- Z : ndarray or float     A ``(d1, ..., dn)``-shaped array of floating-point samples from     the standard normal distribution, or a single such float if     no parameters were supplied.  See Also -------- random.standard_normal : Similar, but takes a tuple as its argument.  Notes ----- For random samples from :math:`N(\mu, \sigma^2)`, use:  ``sigma * np.random.randn(...) + mu``  Examples -------- >>> np.random.randn() 2.1923875335537315 #random  Two-by-four array of samples from N(3, 6.25):  >>> 2.5 * np.random.randn(2, 4) + 3 array([[-4.49401501,  4.00950034, -1.81814867,  7.29718677],  #random        [ 0.39924804,  4.68456316,  4.99394529,  4.84057254]]) #random ``` |

|  |  |  |
| --- | --- | --- |
| |  |  | | --- | --- | | random(size=None) | source code |  ``` Return random floats in the half-open interval [0.0, 1.0).  Results are from the "continuous uniform" distribution over the stated interval.  To sample :math:`Unif[a, b), b > a` multiply the output of `random_sample` by `(b-a)` and add `a`::    (b - a) * random_sample() + a  Parameters ---------- size : int or tuple of ints, optional     Defines the shape of the returned array of random floats. If None     (the default), returns a single float.  Returns ------- out : float or ndarray of floats     Array of random floats of shape `size` (unless ``size=None``, in which     case a single float is returned).  Examples -------- >>> np.random.random_sample() 0.47108547995356098 >>> type(np.random.random_sample()) <type 'float'> >>> np.random.random_sample((5,)) array([ 0.30220482,  0.86820401,  0.1654503 ,  0.11659149,  0.54323428])  Three-by-two array of random numbers from [-5, 0):  >>> 5 * np.random.random_sample((3, 2)) - 5 array([[-3.99149989, -0.52338984],        [-2.99091858, -0.79479508],        [-1.23204345, -1.75224494]]) ``` |

|  |  |  |
| --- | --- | --- |
| |  |  | | --- | --- | | random\_integers(low, high=None, size=None) | source code |  ``` Return random integers between `low` and `high`, inclusive.  Return random integers from the "discrete uniform" distribution in the closed interval [`low`, `high`].  If `high` is None (the default), then results are from [1, `low`].  Parameters ---------- low : int     Lowest (signed) integer to be drawn from the distribution (unless     ``high=None``, in which case this parameter is the *highest* such     integer). high : int, optional     If provided, the largest (signed) integer to be drawn from the     distribution (see above for behavior if ``high=None``). size : int or tuple of ints, optional     Output shape. Default is None, in which case a single int is returned.  Returns ------- out : int or ndarray of ints     `size`-shaped array of random integers from the appropriate     distribution, or a single such random int if `size` not provided.  See Also -------- random.randint : Similar to `random_integers`, only for the half-open     interval [`low`, `high`), and 0 is the lowest value if `high` is     omitted.  Notes ----- To sample from N evenly spaced floating-point numbers between a and b, use::    a + (b - a) * (np.random.random_integers(N) - 1) / (N - 1.)  Examples -------- >>> np.random.random_integers(5) 4 >>> type(np.random.random_integers(5)) <type 'int'> >>> np.random.random_integers(5, size=(3.,2.)) array([[5, 4],        [3, 3],        [4, 5]])  Choose five random numbers from the set of five evenly-spaced numbers between 0 and 2.5, inclusive (*i.e.*, from the set :math:`{0, 5/8, 10/8, 15/8, 20/8}`):  >>> 2.5 * (np.random.random_integers(5, size=(5,)) - 1) / 4. array([ 0.625,  1.25 ,  0.625,  0.625,  2.5  ])  Roll two six sided dice 1000 times and sum the results:  >>> d1 = np.random.random_integers(1, 6, 1000) >>> d2 = np.random.random_integers(1, 6, 1000) >>> dsums = d1 + d2  Display results as a histogram:  >>> import matplotlib.pyplot as plt >>> count, bins, ignored = plt.hist(dsums, 11, normed=True) >>> plt.show() ``` |

|  |  |  |
| --- | --- | --- |
| |  |  | | --- | --- | | random\_sample(size=None) | source code |  ``` Return random floats in the half-open interval [0.0, 1.0).  Results are from the "continuous uniform" distribution over the stated interval.  To sample :math:`Unif[a, b), b > a` multiply the output of `random_sample` by `(b-a)` and add `a`::    (b - a) * random_sample() + a  Parameters ---------- size : int or tuple of ints, optional     Defines the shape of the returned array of random floats. If None     (the default), returns a single float.  Returns ------- out : float or ndarray of floats     Array of random floats of shape `size` (unless ``size=None``, in which     case a single float is returned).  Examples -------- >>> np.random.random_sample() 0.47108547995356098 >>> type(np.random.random_sample()) <type 'float'> >>> np.random.random_sample((5,)) array([ 0.30220482,  0.86820401,  0.1654503 ,  0.11659149,  0.54323428])  Three-by-two array of random numbers from [-5, 0):  >>> 5 * np.random.random_sample((3, 2)) - 5 array([[-3.99149989, -0.52338984],        [-2.99091858, -0.79479508],        [-1.23204345, -1.75224494]]) ``` |

|  |  |  |
| --- | --- | --- |
| |  |  | | --- | --- | | ranf(size=None) | source code |  ``` Return random floats in the half-open interval [0.0, 1.0).  Results are from the "continuous uniform" distribution over the stated interval.  To sample :math:`Unif[a, b), b > a` multiply the output of `random_sample` by `(b-a)` and add `a`::    (b - a) * random_sample() + a  Parameters ---------- size : int or tuple of ints, optional     Defines the shape of the returned array of random floats. If None     (the default), returns a single float.  Returns ------- out : float or ndarray of floats     Array of random floats of shape `size` (unless ``size=None``, in which     case a single float is returned).  Examples -------- >>> np.random.random_sample() 0.47108547995356098 >>> type(np.random.random_sample()) <type 'float'> >>> np.random.random_sample((5,)) array([ 0.30220482,  0.86820401,  0.1654503 ,  0.11659149,  0.54323428])  Three-by-two array of random numbers from [-5, 0):  >>> 5 * np.random.random_sample((3, 2)) - 5 array([[-3.99149989, -0.52338984],        [-2.99091858, -0.79479508],        [-1.23204345, -1.75224494]]) ``` |

|  |  |  |
| --- | --- | --- |
| |  |  | | --- | --- | | rayleigh(scale=1.0, size=None) | source code |  ``` Draw samples from a Rayleigh distribution.  The :math:`\chi` and Weibull distributions are generalizations of the Rayleigh.  Parameters ---------- scale : scalar     Scale, also equals the mode. Should be >= 0. size : int or tuple of ints, optional     Shape of the output. Default is None, in which case a single     value is returned.  Notes ----- The probability density function for the Rayleigh distribution is  .. math:: P(x;scale) = \frac{x}{scale^2}e^{\frac{-x^2}{2 \cdotp scale^2}}  The Rayleigh distribution arises if the wind speed and wind direction are both gaussian variables, then the vector wind velocity forms a Rayleigh distribution. The Rayleigh distribution is used to model the expected output from wind turbines.  References ---------- ..[1] Brighton Webs Ltd., Rayleigh Distribution,       http://www.brighton-webs.co.uk/distributions/rayleigh.asp ..[2] Wikipedia, "Rayleigh distribution"       http://en.wikipedia.org/wiki/Rayleigh_distribution  Examples -------- Draw values from the distribution and plot the histogram  >>> values = hist(np.random.rayleigh(3, 100000), bins=200, normed=True)  Wave heights tend to follow a Rayleigh distribution. If the mean wave height is 1 meter, what fraction of waves are likely to be larger than 3 meters?  >>> meanvalue = 1 >>> modevalue = np.sqrt(2 / np.pi) * meanvalue >>> s = np.random.rayleigh(modevalue, 1000000)  The percentage of waves larger than 3 meters is:  >>> 100.*sum(s>3)/1000000. 0.087300000000000003 ``` |

|  |  |  |
| --- | --- | --- |
| |  |  | | --- | --- | | sample(size=None) | source code |  ``` Return random floats in the half-open interval [0.0, 1.0).  Results are from the "continuous uniform" distribution over the stated interval.  To sample :math:`Unif[a, b), b > a` multiply the output of `random_sample` by `(b-a)` and add `a`::    (b - a) * random_sample() + a  Parameters ---------- size : int or tuple of ints, optional     Defines the shape of the returned array of random floats. If None     (the default), returns a single float.  Returns ------- out : float or ndarray of floats     Array of random floats of shape `size` (unless ``size=None``, in which     case a single float is returned).  Examples -------- >>> np.random.random_sample() 0.47108547995356098 >>> type(np.random.random_sample()) <type 'float'> >>> np.random.random_sample((5,)) array([ 0.30220482,  0.86820401,  0.1654503 ,  0.11659149,  0.54323428])  Three-by-two array of random numbers from [-5, 0):  >>> 5 * np.random.random_sample((3, 2)) - 5 array([[-3.99149989, -0.52338984],        [-2.99091858, -0.79479508],        [-1.23204345, -1.75224494]]) ``` |

|  |  |  |
| --- | --- | --- |
| |  |  | | --- | --- | | save(\*args, \*\*kwargs) | source code |   pylab no longer provides a save function, though the old pylab function is still available as matplotlib.mlab.save (you can still refer to it in pylab as "mlab.save"). However, for plain text files, we recommend numpy.savetxt. For saving numpy arrays, we recommend numpy.save, and its analog numpy.load, which are available in pylab as np.save and np.load. |

|  |  |  |
| --- | --- | --- |
| |  |  | | --- | --- | | seed(seed=None) | source code |  ``` Seed the generator.  This method is called when `RandomState` is initialized. It can be called again to re-seed the generator. For details, see `RandomState`.  Parameters ---------- seed : int or array_like, optional     Seed for `RandomState`.  See Also -------- RandomState ``` |

|  |  |  |
| --- | --- | --- |
| |  |  | | --- | --- | | set\_state(state) | source code |  ``` Set the internal state of the generator from a tuple.  For use if one has reason to manually (re-)set the internal state of the "Mersenne Twister"[1]_ pseudo-random number generating algorithm.  Parameters ---------- state : tuple(str, ndarray of 624 uints, int, int, float)     The `state` tuple has the following items:      1. the string 'MT19937', specifying the Mersenne Twister algorithm.     2. a 1-D array of 624 unsigned integers ``keys``.     3. an integer ``pos``.     4. an integer ``has_gauss``.     5. a float ``cached_gaussian``.  Returns ------- out : None     Returns 'None' on success.  See Also -------- get_state  Notes ----- `set_state` and `get_state` are not needed to work with any of the random distributions in NumPy. If the internal state is manually altered, the user should know exactly what he/she is doing.  For backwards compatibility, the form (str, array of 624 uints, int) is also accepted although it is missing some information about the cached Gaussian value: ``state = ('MT19937', keys, pos)``.  References ---------- .. [1] M. Matsumoto and T. Nishimura, "Mersenne Twister: A    623-dimensionally equidistributed uniform pseudorandom number    generator," *ACM Trans. on Modeling and Computer Simulation*,    Vol. 8, No. 1, pp. 3-30, Jan. 1998. ``` |

|  |  |  |
| --- | --- | --- |
| |  |  | | --- | --- | | standard\_cauchy(size=None) | source code |  ``` Standard Cauchy distribution with mode = 0.  Also known as the Lorentz distribution.  Parameters ---------- size : int or tuple of ints     Shape of the output.  Returns ------- samples : ndarray or scalar     The drawn samples.  Notes ----- The probability density function for the full Cauchy distribution is  .. math:: P(x; x_0, \gamma) = \frac{1}{\pi \gamma \bigl[ 1+           (\frac{x-x_0}{\gamma})^2 \bigr] }  and the Standard Cauchy distribution just sets :math:`x_0=0` and :math:`\gamma=1`  The Cauchy distribution arises in the solution to the driven harmonic oscillator problem, and also describes spectral line broadening. It also describes the distribution of values at which a line tilted at a random angle will cut the x axis.  When studying hypothesis tests that assume normality, seeing how the tests perform on data from a Cauchy distribution is a good indicator of their sensitivity to a heavy-tailed distribution, since the Cauchy looks very much like a Gaussian distribution, but with heavier tails.  References ---------- ..[1] NIST/SEMATECH e-Handbook of Statistical Methods, "Cauchy       Distribution",       http://www.itl.nist.gov/div898/handbook/eda/section3/eda3663.htm ..[2] Weisstein, Eric W. "Cauchy Distribution." From MathWorld--A       Wolfram Web Resource.       http://mathworld.wolfram.com/CauchyDistribution.html ..[3] Wikipedia, "Cauchy distribution"       http://en.wikipedia.org/wiki/Cauchy_distribution  Examples -------- Draw samples and plot the distribution:  >>> s = np.random.standard_cauchy(1000000) >>> s = s[(s>-25) & (s<25)]  # truncate distribution so it plots well >>> plt.hist(s, bins=100) >>> plt.show() ``` |

|  |  |  |
| --- | --- | --- |
| |  |  | | --- | --- | | standard\_exponential(size=None) | source code |  ``` Draw samples from the standard exponential distribution.  `standard_exponential` is identical to the exponential distribution with a scale parameter of 1.  Parameters ---------- size : int or tuple of ints     Shape of the output.  Returns ------- out : float or ndarray     Drawn samples.  Examples -------- Output a 3x8000 array:  >>> n = np.random.standard_exponential((3, 8000)) ``` |

|  |  |  |
| --- | --- | --- |
| |  |  | | --- | --- | | standard\_gamma(shape, size=None) | source code |  ``` Draw samples from a Standard Gamma distribution.  Samples are drawn from a Gamma distribution with specified parameters, shape (sometimes designated "k") and scale=1.  Parameters ---------- shape : float     Parameter, should be > 0. size : int or tuple of ints     Output shape.  If the given shape is, e.g., ``(m, n, k)``, then     ``m * n * k`` samples are drawn.  Returns ------- samples : ndarray or scalar     The drawn samples.  See Also -------- scipy.stats.distributions.gamma : probability density function,     distribution or cumulative density function, etc.  Notes ----- The probability density for the Gamma distribution is  .. math:: p(x) = x^{k-1}\frac{e^{-x/\theta}}{\theta^k\Gamma(k)},  where :math:`k` is the shape and :math:`\theta` the scale, and :math:`\Gamma` is the Gamma function.  The Gamma distribution is often used to model the times to failure of electronic components, and arises naturally in processes for which the waiting times between Poisson distributed events are relevant.  References ---------- .. [1] Weisstein, Eric W. "Gamma Distribution." From MathWorld--A        Wolfram Web Resource.        http://mathworld.wolfram.com/GammaDistribution.html .. [2] Wikipedia, "Gamma-distribution",        http://en.wikipedia.org/wiki/Gamma-distribution  Examples -------- Draw samples from the distribution:  >>> shape, scale = 2., 1. # mean and width >>> s = np.random.standard_gamma(shape, 1000000)  Display the histogram of the samples, along with the probability density function:  >>> import matplotlib.pyplot as plt >>> import scipy.special as sps >>> count, bins, ignored = plt.hist(s, 50, normed=True) >>> y = bins**(shape-1) * ((np.exp(-bins/scale))/ \ ...                       (sps.gamma(shape) * scale**shape)) >>> plt.plot(bins, y, linewidth=2, color='r') >>> plt.show() ``` |

|  |  |  |
| --- | --- | --- |
| |  |  | | --- | --- | | standard\_normal(size=None) | source code |  ``` Returns samples from a Standard Normal distribution (mean=0, stdev=1).  Parameters ---------- size : int or tuple of ints, optional     Output shape. Default is None, in which case a single value is     returned.  Returns ------- out : float or ndarray     Drawn samples.  Examples -------- >>> s = np.random.standard_normal(8000) >>> s array([ 0.6888893 ,  0.78096262, -0.89086505, ...,  0.49876311, #random        -0.38672696, -0.4685006 ])                               #random >>> s.shape (8000,) >>> s = np.random.standard_normal(size=(3, 4, 2)) >>> s.shape (3, 4, 2) ``` |

|  |  |  |
| --- | --- | --- |
| |  |  | | --- | --- | | standard\_t(df, size=None) | source code |  ``` Standard Student's t distribution with df degrees of freedom.  A special case of the hyperbolic distribution. As `df` gets large, the result resembles that of the standard normal distribution (`standard_normal`).  Parameters ---------- df : int     Degrees of freedom, should be > 0. size : int or tuple of ints, optional     Output shape. Default is None, in which case a single value is     returned.  Returns ------- samples : ndarray or scalar     Drawn samples.  Notes ----- The probability density function for the t distribution is  .. math:: P(x, df) = \frac{\Gamma(\frac{df+1}{2})}{\sqrt{\pi df}           \Gamma(\frac{df}{2})}\Bigl( 1+\frac{x^2}{df} \Bigr)^{-(df+1)/2}  The t test is based on an assumption that the data come from a Normal distribution. The t test provides a way to test whether the sample mean (that is the mean calculated from the data) is a good estimate of the true mean.  The derivation of the t-distribution was forst published in 1908 by William Gisset while working for the Guinness Brewery in Dublin. Due to proprietary issues, he had to publish under a pseudonym, and so he used the name Student.  References ---------- .. [1] Dalgaard, Peter, "Introductory Statistics With R",        Springer, 2002. .. [2] Wikipedia, "Student's t-distribution"        http://en.wikipedia.org/wiki/Student's_t-distribution  Examples -------- From Dalgaard page 83 [1]_, suppose the daily energy intake for 11 women in Kj is:  >>> intake = np.array([5260., 5470, 5640, 6180, 6390, 6515, 6805, 7515, \ ...                    7515, 8230, 8770])  Does their energy intake deviate systematically from the recommended value of 7725 kJ?  We have 10 degrees of freedom, so is the sample mean within 95% of the recommended value?  >>> s = np.random.standard_t(10, size=100000) >>> np.mean(intake) 6753.636363636364 >>> intake.std(ddof=1) 1142.1232221373727  Calculate the t statistic, setting the ddof parameter to the unbiased value so the divisor in the standard deviation will be degrees of freedom, N-1.  >>> t = (np.mean(intake)-7725)/(intake.std(ddof=1)/np.sqrt(len(intake))) >>> import matplotlib.pyplot as plt >>> h = plt.hist(s, bins=100, normed=True)  For a one-sided t-test, how far out in the distribution does the t statistic appear?  >>> >>> np.sum(s<t) / float(len(s)) 0.0090699999999999999  #random  So the p-value is about 0.009, which says the null hypothesis has a probability of about 99% of being true. ``` |

|  |  |  |
| --- | --- | --- |
| |  |  | | --- | --- | | triangular(left, mode, right, size=None) | source code |  ``` Draw samples from the triangular distribution.  The triangular distribution is a continuous probability distribution with lower limit left, peak at mode, and upper limit right. Unlike the other distributions, these parameters directly define the shape of the pdf.  Parameters ---------- left : scalar     Lower limit. mode : scalar     The value where the peak of the distribution occurs.     The value should fulfill the condition ``left <= mode <= right``. right : scalar     Upper limit, should be larger than `left`. size : int or tuple of ints, optional     Output shape. Default is None, in which case a single value is     returned.  Returns ------- samples : ndarray or scalar     The returned samples all lie in the interval [left, right].  Notes ----- The probability density function for the Triangular distribution is  .. math:: P(x;l, m, r) = \begin{cases}           \frac{2(x-l)}{(r-l)(m-l)}& \text{for $l \leq x \leq m$},\\           \frac{2(m-x)}{(r-l)(r-m)}& \text{for $m \leq x \leq r$},\\           0& \text{otherwise}.           \end{cases}  The triangular distribution is often used in ill-defined problems where the underlying distribution is not known, but some knowledge of the limits and mode exists. Often it is used in simulations.  References ---------- ..[1] Wikipedia, "Triangular distribution"       http://en.wikipedia.org/wiki/Triangular_distribution  Examples -------- Draw values from the distribution and plot the histogram:  >>> import matplotlib.pyplot as plt >>> h = plt.hist(np.random.triangular(-3, 0, 8, 100000), bins=200, ...              normed=True) >>> plt.show() ``` |

|  |  |  |
| --- | --- | --- |
| |  |  | | --- | --- | | uniform(low=0.0, high=1.0, size=1) | source code |  ``` Draw samples from a uniform distribution.  Samples are uniformly distributed over the half-open interval ``[low, high)`` (includes low, but excludes high).  In other words, any value within the given interval is equally likely to be drawn by `uniform`.  Parameters ---------- low : float, optional     Lower boundary of the output interval.  All values generated will be     greater than or equal to low.  The default value is 0. high : float     Upper boundary of the output interval.  All values generated will be     less than high.  The default value is 1.0. size : tuple of ints, int, optional     Shape of output.  If the given size is, for example, (m,n,k),     m*n*k samples are generated.  If no shape is specified, a single sample     is returned.  Returns ------- out : ndarray     Drawn samples, with shape `size`.  See Also -------- randint : Discrete uniform distribution, yielding integers. random_integers : Discrete uniform distribution over the closed interval                   ``[low, high]``. random_sample : Floats uniformly distributed over ``[0, 1)``. random : Alias for `random_sample`. rand : Convenience function that accepts dimensions as input, e.g.,        ``rand(2,2)`` would generate a 2-by-2 array of floats, uniformly        distributed over ``[0, 1)``.  Notes ----- The probability density function of the uniform distribution is  .. math:: p(x) = \frac{1}{b - a}  anywhere within the interval ``[a, b)``, and zero elsewhere.  Examples -------- Draw samples from the distribution:  >>> s = np.random.uniform(-1,0,1000)  All values are within the given interval:  >>> np.all(s >= -1) True  >>> np.all(s < 0) True  Display the histogram of the samples, along with the probability density function:  >>> import matplotlib.pyplot as plt >>> count, bins, ignored = plt.hist(s, 15, normed=True) >>> plt.plot(bins, np.ones_like(bins), linewidth=2, color='r') >>> plt.show() ``` |

|  |  |  |
| --- | --- | --- |
| |  |  | | --- | --- | | vonmises(mu=0.0, kappa=1.0, size=None) | source code |  ``` Draw samples from a von Mises distribution.  Samples are drawn from a von Mises distribution with specified mode (mu) and dispersion (kappa), on the interval [-pi, pi].  The von Mises distribution (also known as the circular normal distribution) is a continuous probability distribution on the circle. It may be thought of as the circular analogue of the normal distribution.  Parameters ---------- mu : float     Mode ("center") of the distribution. kappa : float, >= 0.     Dispersion of the distribution. size : {tuple, int}     Output shape.  If the given shape is, e.g., ``(m, n, k)``, then     ``m * n * k`` samples are drawn.  Returns ------- samples : {ndarray, scalar}     The returned samples live on the unit circle [-\pi, \pi].  See Also -------- scipy.stats.distributions.vonmises : probability density function,     distribution or cumulative density function, etc.  Notes ----- The probability density for the von Mises distribution is  .. math:: p(x) = \frac{e^{\kappa cos(x-\mu)}}{2\pi I_0(\kappa)},  where :math:`\mu` is the mode and :math:`\kappa` the dispersion, and :math:`I_0(\kappa)` is the modified Bessel function of order 0.  The von Mises, named for Richard Edler von Mises, born in Austria-Hungary, in what is now the Ukraine. He fled to the United States in 1939 and became a professor at Harvard. He worked in probability theory, aerodynamics, fluid mechanics, and philosophy of science.  References ---------- .. [1] Abramowitz, M. and Stegun, I. A. (ed.), Handbook of Mathematical        Functions, National Bureau of Standards, 1964; reprinted Dover        Publications, 1965. .. [2] von Mises, Richard, 1964, Mathematical Theory of Probability        and Statistics (New York: Academic Press). .. [3] Wikipedia, "Von Mises distribution",        http://en.wikipedia.org/wiki/Von_Mises_distribution  Examples -------- Draw samples from the distribution:  >>> mu, kappa = 0.0, 4.0 # mean and dispersion >>> s = np.random.vonmises(mu, kappa, 1000)  Display the histogram of the samples, along with the probability density function:  >>> import matplotlib.pyplot as plt >>> import scipy.special as sps >>> count, bins, ignored = plt.hist(s, 50, normed=True) >>> x = np.arange(-np.pi, np.pi, 2*np.pi/50.) >>> y = -np.exp(kappa*np.cos(x-mu))/(2*np.pi*sps.jn(0,kappa)) >>> plt.plot(x, y/max(y), linewidth=2, color='r') >>> plt.show() ``` |

|  |  |  |
| --- | --- | --- |
| |  |  | | --- | --- | | wald(mean, scale, size=None) | source code |  ``` Draw samples from a Wald, or Inverse Gaussian, distribution.  As the scale approaches infinity, the distribution becomes more like a Gaussian.  Some references claim that the Wald is an Inverse Gaussian with mean=1, but this is by no means universal.  The Inverse Gaussian distribution was first studied in relationship to Brownian motion. In 1956 M.C.K. Tweedie used the name Inverse Gaussian because there is an inverse relationship between the time to cover a unit distance and distance covered in unit time.  Parameters ---------- mean : scalar     Distribution mean, should be > 0. scale : scalar     Scale parameter, should be >= 0. size : int or tuple of ints, optional     Output shape. Default is None, in which case a single value is     returned.  Returns ------- samples : ndarray or scalar     Drawn sample, all greater than zero.  Notes ----- The probability density function for the Wald distribution is  .. math:: P(x;mean,scale) = \sqrt{\frac{scale}{2\pi x^3}}e^                             \frac{-scale(x-mean)^2}{2\cdotp mean^2x}  As noted above the Inverse Gaussian distribution first arise from attempts to model Brownian Motion. It is also a competitor to the Weibull for use in reliability modeling and modeling stock returns and interest rate processes.  References ---------- ..[1] Brighton Webs Ltd., Wald Distribution,       http://www.brighton-webs.co.uk/distributions/wald.asp ..[2] Chhikara, Raj S., and Folks, J. Leroy, "The Inverse Gaussian       Distribution: Theory : Methodology, and Applications", CRC Press,       1988. ..[3] Wikipedia, "Wald distribution"       http://en.wikipedia.org/wiki/Wald_distribution  Examples -------- Draw values from the distribution and plot the histogram:  >>> import matplotlib.pyplot as plt >>> h = plt.hist(np.random.wald(3, 2, 100000), bins=200, normed=True) >>> plt.show() ``` |

|  |  |  |
| --- | --- | --- |
| |  |  | | --- | --- | | weibull(a, size=None) | source code |  ``` Weibull distribution.  Draw samples from a 1-parameter Weibull distribution with the given shape parameter.  .. math:: X = (-ln(U))^{1/a}  Here, U is drawn from the uniform distribution over (0,1].  The more common 2-parameter Weibull, including a scale parameter :math:`\lambda` is just :math:`X = \lambda(-ln(U))^{1/a}`.  The Weibull (or Type III asymptotic extreme value distribution for smallest values, SEV Type III, or Rosin-Rammler distribution) is one of a class of Generalized Extreme Value (GEV) distributions used in modeling extreme value problems.  This class includes the Gumbel and Frechet distributions.  Parameters ---------- a : float     Shape of the distribution. size : tuple of ints     Output shape.  If the given shape is, e.g., ``(m, n, k)``, then     ``m * n * k`` samples are drawn.  See Also -------- scipy.stats.distributions.weibull : probability density function,     distribution or cumulative density function, etc.  gumbel, scipy.stats.distributions.genextreme  Notes ----- The probability density for the Weibull distribution is  .. math:: p(x) = \frac{a}                  {\lambda}(\frac{x}{\lambda})^{a-1}e^{-(x/\lambda)^a},  where :math:`a` is the shape and :math:`\lambda` the scale.  The function has its peak (the mode) at :math:`\lambda(\frac{a-1}{a})^{1/a}`.  When ``a = 1``, the Weibull distribution reduces to the exponential distribution.  References ---------- .. [1] Waloddi Weibull, Professor, Royal Technical University, Stockholm,        1939 "A Statistical Theory Of The Strength Of Materials",        Ingeniorsvetenskapsakademiens Handlingar Nr 151, 1939,        Generalstabens Litografiska Anstalts Forlag, Stockholm. .. [2] Waloddi Weibull, 1951 "A Statistical Distribution Function of Wide        Applicability",  Journal Of Applied Mechanics ASME Paper. .. [3] Wikipedia, "Weibull distribution",        http://en.wikipedia.org/wiki/Weibull_distribution  Examples -------- Draw samples from the distribution:  >>> a = 5. # shape >>> s = np.random.weibull(a, 1000)  Display the histogram of the samples, along with the probability density function:  >>> import matplotlib.pyplot as plt >>> x = np.arange(1,100.)/50. >>> def weib(x,n,a): ...     return (a / n) * (x / n)**(a - 1) * np.exp(-(x / n)**a)  >>> count, bins, ignored = plt.hist(np.random.weibull(5.,1000)) >>> x = np.arange(1,100.)/50. >>> scale = count.max()/weib(x, 1., 5.).max() >>> plt.plot(x, weib(x, 1., 5.)*scale) >>> plt.show() ``` |

|  |  |  |
| --- | --- | --- |
| |  |  | | --- | --- | | zipf(a, size=None) | source code |  ``` Draw samples from a Zipf distribution.  Samples are drawn from a Zipf distribution with specified parameter (a), where a > 1.  The zipf distribution (also known as the zeta distribution) is a continuous probability distribution that satisfies Zipf's law, where the frequency of an item is inversely proportional to its rank in a frequency table.  Parameters ---------- a : float     parameter, > 1. size : {tuple, int}     Output shape.  If the given shape is, e.g., ``(m, n, k)``, then     ``m * n * k`` samples are drawn.  Returns ------- samples : {ndarray, scalar}     The returned samples are greater than or equal to one.  See Also -------- scipy.stats.distributions.zipf : probability density function,     distribution or cumulative density function, etc.  Notes ----- The probability density for the Zipf distribution is  .. math:: p(x) = \frac{x^{-a}}{\zeta(a)},  where :math:`\zeta` is the Riemann Zeta function.  Named after the American linguist George Kingsley Zipf, who noted that the frequency of any word in a sample of a language is inversely proportional to its rank in the frequency table.   References ---------- .. [1] Weisstein, Eric W. "Zipf Distribution." From MathWorld--A Wolfram        Web Resource. http://mathworld.wolfram.com/ZipfDistribution.html .. [2] Wikipedia, "Zeta distribution",        http://en.wikipedia.org/wiki/Zeta_distribution .. [3] Wikipedia, "Zipf's Law",        http://en.wikipedia.org/wiki/Zipf%27s_law .. [4] Zipf, George Kingsley (1932): Selected Studies of the Principle        of Relative Frequency in Language. Cambridge (Mass.).  Examples -------- Draw samples from the distribution:  >>> a = 2. # parameter >>> s = np.random.zipf(a, 1000)  Display the histogram of the samples, along with the probability density function:  >>> import matplotlib.pyplot as plt >>> import scipy.special as sps Truncate s values at 50 so plot is interesting >>> count, bins, ignored = plt.hist(s[s<50], 50, normed=True) >>> x = np.arange(1., 50.) >>> y = x**(-a)/sps.zetac(a) >>> plt.plot(x, y/max(y), linewidth=2, color='r') >>> plt.show() ``` |

  


|  |  |  |  |
| --- | --- | --- | --- |
| |  |  | | --- | --- | | Variables Details | [hide private] | | |

|  |  |
| --- | --- |
| ScalarType   Value:  |  | | --- | | ``` (<type 'int'>,  <type 'float'>,  <type 'complex'>,  <type 'long'>,  <type 'bool'>,  <type 'str'>,  <type 'unicode'>,  <type 'buffer'>, ... ``` | |

|  |  |
| --- | --- |
| cast   Value:  |  | | --- | | ``` {<type 'numpy.int64'>: <function <lambda> at 0x10a8bb0>, <type 'numpy. int16'>: <function <lambda> at 0x10a8bf0>, <type 'numpy.complex128'>:  <function <lambda> at 0x10a8c30>, <type 'numpy.uint64'>: <function <la mbda> at 0x10a8c70>, <type 'numpy.complex256'>: <function <lambda> at  0x10a8cf0>, <type 'numpy.float32'>: <function <lambda> at 0x10a8d30>,  <type 'numpy.bool_'>: <function <lambda> at 0x10a8cb0>, <type 'numpy.u int8'>: <function <lambda> at 0x10a8db0>, <type 'numpy.int32'>: <funct ion <lambda> at 0x10a8eb0>, <type 'numpy.int8'>: <function <lambda> at ... ``` | |

|  |  |
| --- | --- |
| colorbar\_doc   Value:  |  | | --- | | ``` '''  Add a colorbar to a plot.  Function signatures for the :mod:`~matplotlib.pyplot` interface; all but the first are also method signatures for the :meth:`~matplotlib.figure.Figure.colorbar` method::  ... ``` | |

|  |  |
| --- | --- |
| index\_exp   Value:  |  | | --- | | ``` <numpy.lib.index_tricks.IndexExpression object at 0x11543b0> ``` | |

|  |  |
| --- | --- |
| nbytes   Value:  |  | | --- | | ``` {<type 'numpy.int64'>: 8, <type 'numpy.int16'>: 2, <type 'numpy.comple x128'>: 16, <type 'numpy.uint64'>: 8, <type 'numpy.bool_'>: 1, <type ' numpy.complex256'>: 32, <type 'numpy.float32'>: 4, <type 'numpy.int8'> : 1, <type 'numpy.uint8'>: 1, <type 'numpy.uint16'>: 2, <type 'numpy.o bject_'>: 4, <type 'numpy.float64'>: 8, <type 'numpy.int32'>: 4, <type  'numpy.string_'>: 0, <type 'numpy.void'>: 0, <type 'numpy.float128'>:  16, <type 'numpy.int32'>: 4, <type 'numpy.uint32'>: 4, <type 'numpy.u nicode_'>: 0, <type 'numpy.complex64'>: 8, <type 'numpy.uint32'>: 4} ``` | |

|  |  |
| --- | --- |
| rcParams   Value:  |  | | --- | | ``` {'figure.subplot.right': 0.90000000000000002, 'mathtext.cal': 'cursive ', 'font.fantasy': ['Comic Sans MS', 'Chicago', 'Charcoal', 'ImpactWes tern', 'fantasy'], 'xtick.minor.pad': 4, 'tk.pythoninspect': False, 'i mage.aspect': 'equal', 'font.cursive': ['Apple Chancery', 'Textile', ' Zapf Chancery', 'Sand', 'cursive'], 'figure.subplot.hspace': 0.2000000 0000000001, 'xtick.direction': 'in', 'axes.facecolor': 'w', 'mathtext. fontset': 'cm', 'ytick.direction': 'in', 'axes.axisbelow': False, 'lin es.markersize': 6, 'figure.dpi': 100.0, 'text.usetex': True, 'text.fon ... ``` | |

|  |  |
| --- | --- |
| rcParamsDefault   Value:  |  | | --- | | ``` {'figure.subplot.right': 0.90000000000000002, 'mathtext.cal': 'cursive ', 'font.fantasy': ['Comic Sans MS', 'Chicago', 'Charcoal', 'ImpactWes tern', 'fantasy'], 'xtick.minor.pad': 4, 'tk.pythoninspect': False, 'i mage.aspect': 'equal', 'font.cursive': ['Apple Chancery', 'Textile', ' Zapf Chancery', 'Sand', 'cursive'], 'figure.subplot.hspace': 0.2000000 0000000001, 'xtick.direction': 'in', 'axes.facecolor': 'w', 'mathtext. fontset': 'cm', 'ytick.direction': 'in', 'axes.axisbelow': False, 'lin es.markersize': 6, 'figure.dpi': 80, 'text.usetex': False, 'text.fonta ... ``` | |

|  |  |
| --- | --- |
| sctypeDict   Value:  |  | | --- | | ``` {0: <type 'numpy.bool_'>,  1: <type 'numpy.int8'>,  2: <type 'numpy.uint8'>,  3: <type 'numpy.int16'>,  4: <type 'numpy.uint16'>,  5: <type 'numpy.int32'>,  6: <type 'numpy.uint32'>,  7: <type 'numpy.int32'>, ... ``` | |

|  |  |
| --- | --- |
| sctypeNA   Value:  |  | | --- | | ``` {'?': 'Bool',  'B': 'UInt8',  'Bool': <type 'numpy.bool_'>,  'Complex128': <type 'numpy.complex256'>,  'Complex32': <type 'numpy.complex64'>,  'Complex64': <type 'numpy.complex128'>,  'D': 'Complex64',  'F': 'Complex32', ... ``` | |

|  |  |
| --- | --- |
| sctypes   Value:  |  | | --- | | ``` {'complex': [<type 'numpy.complex64'>,              <type 'numpy.complex128'>,              <type 'numpy.complex256'>],  'float': [<type 'numpy.float32'>,            <type 'numpy.float64'>,            <type 'numpy.float128'>],  'int': [<type 'numpy.int8'>,          <type 'numpy.int16'>, ... ``` | |

|  |  |
| --- | --- |
| typeDict   Value:  |  | | --- | | ``` {0: <type 'numpy.bool_'>,  1: <type 'numpy.int8'>,  2: <type 'numpy.uint8'>,  3: <type 'numpy.int16'>,  4: <type 'numpy.uint16'>,  5: <type 'numpy.int32'>,  6: <type 'numpy.uint32'>,  7: <type 'numpy.int32'>, ... ``` | |

|  |  |
| --- | --- |
| typeNA   Value:  |  | | --- | | ``` {'?': 'Bool',  'B': 'UInt8',  'Bool': <type 'numpy.bool_'>,  'Complex128': <type 'numpy.complex256'>,  'Complex32': <type 'numpy.complex64'>,  'Complex64': <type 'numpy.complex128'>,  'D': 'Complex64',  'F': 'Complex32', ... ``` | |

|  |  |
| --- | --- |
| typecodes   Value:  |  | | --- | | ``` {'All': '?bhilqpBHILQPfdgFDGSUVOMm',  'AllFloat': 'fdgFDG',  'AllInteger': 'bBhHiIlLqQpP',  'Character': 'c',  'Complex': 'FDG',  'Datetime': 'Mm',  'Float': 'fdg',  'Integer': 'bhilqp', ... ``` | |

  


| Home | Trees | Indices | Help | | PyDSTool | | --- | |
| --- | --- | --- | --- | --- | --- |

|  |  |
| --- | --- |
| Generated by Epydoc 3.0.1 on Fri May 4 15:24:06 2012 | http://epydoc.sourceforge.net |
