# Supplementary material for: Hybrid Models and Biological Model Reduction with PyDSTool
Source: PLoS Comput Biol. 2012 Aug 9;8(8):e1002628. doi: 10.1371/journal.pcbi.1002628 (PMC3415397; doi:10.1371/journal.pcbi.1002628)
Supplement: Text S4 — Complete source code for the PyDSTool package (version 0.88.120504). Includes API documentation and help files linking to web pages. This file is identical to the current public release on Sourceforge.net. (ZIP) [file pcbi.1002628.s004.zip › PyDSTool/html/matplotlib.pylab-pysrc.html]

xml version="1.0" encoding="ascii"?


matplotlib.pylab


| Home | Trees | Indices | Help | | PyDSTool | | --- | |
| --- | --- | --- | --- | --- | --- |

|  |  |  |  |
| --- | --- | --- | --- |
| Package matplotlib :: Module pylab | |  | | --- | | [hide private] | | [frames] | no frames] | |

# Source Code for Module matplotlib.pylab

```
  1  """ 
  2  This is a procedural interface to the matplotlib object-oriented 
  3  plotting library. 
  4   
  5  The following plotting commands are provided; the majority have 
  6  Matlab(TM) analogs and similar argument. 
  7   
  8  _Plotting commands 
  9    acorr     - plot the autocorrelation function 
 10    annotate  - annotate something in the figure 
 11    arrow     - add an arrow to the axes 
 12    axes      - Create a new axes 
 13    axhline   - draw a horizontal line across axes 
 14    axvline   - draw a vertical line across axes 
 15    axhspan   - draw a horizontal bar across axes 
 16    axvspan   - draw a vertical bar across axes 
 17    axis      - Set or return the current axis limits 
 18    bar       - make a bar chart 
 19    barh      - a horizontal bar chart 
 20    broken_barh - a set of horizontal bars with gaps 
 21    box       - set the axes frame on/off state 
 22    boxplot   - make a box and whisker plot 
 23    cla       - clear current axes 
 24    clabel    - label a contour plot 
 25    clf       - clear a figure window 
 26    clim      - adjust the color limits of the current image 
 27    close     - close a figure window 
 28    colorbar  - add a colorbar to the current figure 
 29    cohere    - make a plot of coherence 
 30    contour   - make a contour plot 
 31    contourf  - make a filled contour plot 
 32    csd       - make a plot of cross spectral density 
 33    delaxes   - delete an axes from the current figure 
 34    draw      - Force a redraw of the current figure 
 35    errorbar  - make an errorbar graph 
 36    figlegend - make legend on the figure rather than the axes 
 37    figimage  - make a figure image 
 38    figtext   - add text in figure coords 
 39    figure   - create or change active figure 
 40    fill     - make filled polygons 
 41    findobj  - recursively find all objects matching some criteria 
 42    gca      - return the current axes 
 43    gcf      - return the current figure 
 44    gci      - get the current image, or None 
 45    getp      - get a graphics property 
 46    grid     - set whether gridding is on 
 47    hist     - make a histogram 
 48    hold     - set the axes hold state 
 49    ioff     - turn interaction mode off 
 50    ion      - turn interaction mode on 
 51    isinteractive - return True if interaction mode is on 
 52    imread   - load image file into array 
 53    imsave   - save array as an image file 
 54    imshow   - plot image data 
 55    ishold   - return the hold state of the current axes 
 56    legend   - make an axes legend 
 57    loglog   - a log log plot 
 58    matshow  - display a matrix in a new figure preserving aspect 
 59    pcolor   - make a pseudocolor plot 
 60    pcolormesh - make a pseudocolor plot using a quadrilateral mesh 
 61    pie      - make a pie chart 
 62    plot     - make a line plot 
 63    plot_date - plot dates 
 64    plotfile  - plot column data from an ASCII tab/space/comma delimited file 
 65    pie      - pie charts 
 66    polar    - make a polar plot on a PolarAxes 
 67    psd      - make a plot of power spectral density 
 68    quiver   - make a direction field (arrows) plot 
 69    rc       - control the default params 
 70    rgrids   - customize the radial grids and labels for polar 
 71    savefig  - save the current figure 
 72    scatter  - make a scatter plot 
 73    setp      - set a graphics property 
 74    semilogx - log x axis 
 75    semilogy - log y axis 
 76    show     - show the figures 
 77    specgram - a spectrogram plot 
 78    spy      - plot sparsity pattern using markers or image 
 79    stem     - make a stem plot 
 80    subplot  - make a subplot (numrows, numcols, axesnum) 
 81    subplots_adjust - change the params controlling the subplot positions of current figure 
 82    subplot_tool - launch the subplot configuration tool 
 83    suptitle   - add a figure title 
 84    table    - add a table to the plot 
 85    text     - add some text at location x,y to the current axes 
 86    thetagrids - customize the radial theta grids and labels for polar 
 87    title    - add a title to the current axes 
 88    xcorr   - plot the autocorrelation function of x and y 
 89    xlim     - set/get the xlimits 
 90    ylim     - set/get the ylimits 
 91    xticks   - set/get the xticks 
 92    yticks   - set/get the yticks 
 93    xlabel   - add an xlabel to the current axes 
 94    ylabel   - add a ylabel to the current axes 
 95   
 96    autumn - set the default colormap to autumn 
 97    bone   - set the default colormap to bone 
 98    cool   - set the default colormap to cool 
 99    copper - set the default colormap to copper 
100    flag   - set the default colormap to flag 
101    gray   - set the default colormap to gray 
102    hot    - set the default colormap to hot 
103    hsv    - set the default colormap to hsv 
104    jet    - set the default colormap to jet 
105    pink   - set the default colormap to pink 
106    prism  - set the default colormap to prism 
107    spring - set the default colormap to spring 
108    summer - set the default colormap to summer 
109    winter - set the default colormap to winter 
110    spectral - set the default colormap to spectral 
111   
112  _Event handling 
113   
114    connect - register an event handler 
115    disconnect - remove a connected event handler 
116   
117  _Matrix commands 
118   
119    cumprod   - the cumulative product along a dimension 
120    cumsum    - the cumulative sum along a dimension 
121    detrend   - remove the mean or besdt fit line from an array 
122    diag      - the k-th diagonal of matrix 
123    diff      - the n-th differnce of an array 
124    eig       - the eigenvalues and eigen vectors of v 
125    eye       - a matrix where the k-th diagonal is ones, else zero 
126    find      - return the indices where a condition is nonzero 
127    fliplr    - flip the rows of a matrix up/down 
128    flipud    - flip the columns of a matrix left/right 
129    linspace  - a linear spaced vector of N values from min to max inclusive 
130    logspace  - a log spaced vector of N values from min to max inclusive 
131    meshgrid  - repeat x and y to make regular matrices 
132    ones      - an array of ones 
133    rand      - an array from the uniform distribution [0,1] 
134    randn     - an array from the normal distribution 
135    rot90     - rotate matrix k*90 degress counterclockwise 
136    squeeze   - squeeze an array removing any dimensions of length 1 
137    tri       - a triangular matrix 
138    tril      - a lower triangular matrix 
139    triu      - an upper triangular matrix 
140    vander    - the Vandermonde matrix of vector x 
141    svd       - singular value decomposition 
142    zeros     - a matrix of zeros 
143   
144  _Probability 
145   
146    levypdf   - The levy probability density function from the char. func. 
147    normpdf   - The Gaussian probability density function 
148    rand      - random numbers from the uniform distribution 
149    randn     - random numbers from the normal distribution 
150   
151  _Statistics 
152   
153    amax       - the maximum along dimension m 
154    amin       - the minimum along dimension m 
155    corrcoef  - correlation coefficient 
156    cov       - covariance matrix 
157    mean      - the mean along dimension m 
158    median    - the median along dimension m 
159    norm      - the norm of vector x 
160    prod      - the product along dimension m 
161    ptp       - the max-min along dimension m 
162    std       - the standard deviation along dimension m 
163    asum       - the sum along dimension m 
164   
165  _Time series analysis 
166   
167    bartlett  - M-point Bartlett window 
168    blackman  - M-point Blackman window 
169    cohere    - the coherence using average periodiogram 
170    csd       - the cross spectral density using average periodiogram 
171    fft       - the fast Fourier transform of vector x 
172    hamming   - M-point Hamming window 
173    hanning   - M-point Hanning window 
174    hist      - compute the histogram of x 
175    kaiser    - M length Kaiser window 
176    psd       - the power spectral density using average periodiogram 
177    sinc      - the sinc function of array x 
178   
179  _Dates 
180   
181    date2num  - convert python datetimes to numeric representation 
182    drange    - create an array of numbers for date plots 
183    num2date  - convert numeric type (float days since 0001) to datetime 
184   
185  _Other 
186   
187    angle     - the angle of a complex array 
188    griddata  - interpolate irregularly distributed data to a regular grid 
189    load      - Deprecated--please use loadtxt. 
190    loadtxt   - load ASCII data into array. 
191    polyfit   - fit x, y to an n-th order polynomial 
192    polyval   - evaluate an n-th order polynomial 
193    roots     - the roots of the polynomial coefficients in p 
194    save      - Deprecated--please use savetxt. 
195    savetxt   - save an array to an ASCII file. 
196    trapz     - trapezoidal integration 
197   
198  __end 
199   
200  """ 
201  import sys, warnings 
202   
203  from cbook import flatten, is_string_like, exception_to_str, \ 
204       silent_list, iterable, dedent 
205   
206  from matplotlib import mpl  # pulls in most modules 
207   
208  from matplotlib.dates import date2num, num2date,\ 
209          datestr2num, strpdate2num, drange,\ 
210          epoch2num, num2epoch, mx2num,\ 
211          DateFormatter, IndexDateFormatter, DateLocator,\ 
212          RRuleLocator, YearLocator, MonthLocator, WeekdayLocator,\ 
213          DayLocator, HourLocator, MinuteLocator, SecondLocator,\ 
214          rrule, MO, TU, WE, TH, FR, SA, SU, YEARLY, MONTHLY,\ 
215          WEEKLY, DAILY, HOURLY, MINUTELY, SECONDLY, relativedelta 
216   
217  import matplotlib.dates  # Do we need this at all? 
218   
219  # bring all the  symbols in so folks can import them from 
220  # pylab in one fell swoop 
221   
222   
223  ## We are still importing too many things from mlab; more cleanup is needed. 
224   
225  from matplotlib.mlab import griddata, stineman_interp, slopes, \ 
226      inside_poly, poly_below, poly_between, \ 
227      is_closed_polygon, path_length, distances_along_curve, vector_lengths 
228   
229  from matplotlib.mlab import window_hanning, window_none,  detrend, demean, \ 
230       detrend_mean, detrend_none, detrend_linear, entropy, normpdf, levypdf, \ 
231       find, longest_contiguous_ones, longest_ones, prepca, prctile, prctile_rank, \ 
232       center_matrix, rk4, bivariate_normal, get_xyz_where, get_sparse_matrix, dist, \ 
233       dist_point_to_segment, segments_intersect, fftsurr, liaupunov, movavg, \ 
234       save, load, exp_safe, \ 
235       amap, rms_flat, l1norm, l2norm, norm_flat, frange,  identity, \ 
236       base_repr, binary_repr, log2, ispower2, \ 
237       rec_append_fields, rec_drop_fields, rec_join, csv2rec, rec2csv, isvector 
238   
239  import matplotlib.mlab as mlab 
240  import matplotlib.cbook as cbook 
241   
242  from numpy import * 
243  from numpy.fft import * 
244  from numpy.random import * 
245  from numpy.linalg import * 
246   
247  from matplotlib.pyplot import * 
248   
249  # provide the recommended module abbrevs in the pylab namespace 
250  import matplotlib.pyplot as plt 
251  import numpy as np 
252  import numpy.ma as ma 
253   


254 -def load(*args, **kwargs):

255      raise  NotImplementedError(load.__doc__)             
256  load.__doc__ = """\ 
257      pylab no longer provides a load function, though the old pylab 
258      function is still available as matplotlib.mlab.load (you can refer 
259      to it in pylab as "mlab.load").  However, for plain text files, we 
260      recommend numpy.loadtxt, which was inspired by the old pylab.load 
261      but now has more features.  For loading numpy arrays, we recommend 
262      numpy.load, and its analog numpy.save, which are available in 
263      pylab as np.load and np.save. 
264      """ 
265   
266   


267 -def save(*args, **kwargs):

268      raise  NotImplementedError(save.__doc__)             
269  save.__doc__ = """\ 
270      pylab no longer provides a save function, though the old pylab 
271      function is still available as matplotlib.mlab.save (you can still 
272      refer to it in pylab as "mlab.save").  However, for plain text 
273      files, we recommend numpy.savetxt.  For saving numpy arrays, 
274      we recommend numpy.save, and its analog numpy.load, which are 
275      available in pylab as np.save and np.load.""" 
276
```

  


| Home | Trees | Indices | Help | | PyDSTool | | --- | |
| --- | --- | --- | --- | --- | --- |

|  |  |
| --- | --- |
| Generated by Epydoc 3.0.1 on Fri May 4 15:24:25 2012 | http://epydoc.sourceforge.net |
