# Supplementary material for: Hybrid Models and Biological Model Reduction with PyDSTool
Source: PLoS Comput Biol. 2012 Aug 9;8(8):e1002628. doi: 10.1371/journal.pcbi.1002628 (PMC3415397; doi:10.1371/journal.pcbi.1002628)
Supplement: Text S4 — Complete source code for the PyDSTool package (version 0.88.120504). Includes API documentation and help files linking to web pages. This file is identical to the current public release on Sourceforge.net. (ZIP) [file pcbi.1002628.s004.zip › PyDSTool/html/module-tree.html]

xml version="1.0" encoding="ascii"?


Module Hierarchy


| Home | Trees | Indices | Help | | PyDSTool | | --- | |
| --- | --- | --- | --- | --- | --- |

|  |  |  |  |
| --- | --- | --- | --- |
|  | |  | | --- | | [hide private] | | [frames] | no frames] | |

**[ Module Hierarchy
| Class Hierarchy ]**  

# Module Hierarchy

- **PyDSTool**: *PyDSTool initialization script.*
  - **PyDSTool.Events**: *Event handling for python-based computations, and specification for
    both python and externally compiled code.*
  - **PyDSTool.FuncSpec**
  - **PyDSTool.FuncSpec'**: *Functional specification classes.*
  - **PyDSTool.Generator**: *Trajectory generator classes.*
    - **PyDSTool.Generator.ADMC\_ODEsystem**
    - **PyDSTool.Generator.ADMC\_ODEsystem'**
    - **PyDSTool.Generator.DDEsystem**
    - **PyDSTool.Generator.Dopri\_ODEsystem**
    - **PyDSTool.Generator.Dopri\_ODEsystem'**
    - **PyDSTool.Generator.EmbeddedSysGen**
    - **PyDSTool.Generator.EmbeddedSysGen'**
    - **PyDSTool.Generator.Euler\_ODEsystem**
    - **PyDSTool.Generator.Euler\_ODEsystem'**: *Euler integrator for ODE systems, with no step refinement for
      events.*
    - **PyDSTool.Generator.ExplicitFnGen**
    - **PyDSTool.Generator.ExplicitFnGen'**
    - **PyDSTool.Generator.ExtrapolateTable**
    - **PyDSTool.Generator.ExtrapolateTable'**
    - **PyDSTool.Generator.ImplicitFnGen**
    - **PyDSTool.Generator.ImplicitFnGen'**
    - **PyDSTool.Generator.InterpolateTable**
    - **PyDSTool.Generator.InterpolateTable'**
    - **PyDSTool.Generator.LookupTable**
    - **PyDSTool.Generator.LookupTable'**
    - **PyDSTool.Generator.MapSystem**
    - **PyDSTool.Generator.MapSystem'**
    - **PyDSTool.Generator.ODEsystem**
    - **PyDSTool.Generator.ODEsystem'**
    - **PyDSTool.Generator.Radau\_ODEsystem**
    - **PyDSTool.Generator.Radau\_ODEsystem'**
    - **PyDSTool.Generator.Vode\_ODEsystem**
    - **PyDSTool.Generator.Vode\_ODEsystem'**: *VODE integrator for ODE systems, imported from a mild modification
      of the scipy-wrapped VODE Fortran solver.*
    - **PyDSTool.Generator.allimports**
    - **PyDSTool.Generator.baseclasses**
    - **PyDSTool.Generator.messagecodes**: *Message code definitions for Generators*
  - **PyDSTool.Interval**
  - **PyDSTool.Interval'**: *Interval class*
  - **PyDSTool.MProject**: *Modelling project and associated classes.*
  - **PyDSTool.Model**: *General purpose (hybrid) model class, and associated hybrid trajectory
    and variable classes.*
  - **PyDSTool.ModelConstructor**
  - **PyDSTool.ModelConstructor'**: *Model Constructor classes.*
  - **PyDSTool.ModelSpec**
  - **PyDSTool.ModelSpec'**: *Structured model specification classes, and associated utilities.*
  - **PyDSTool.Points**: *Point and Pointset enhanced array classes.*
  - **PyDSTool.PyCont**
    - **PyDSTool.PyCont.BifPoint**: *Bifurcation point classes.*
    - **PyDSTool.PyCont.ContClass**
    - **PyDSTool.PyCont.ContClass'**: *ContClass stores continuation curves for a specified model.*
    - **PyDSTool.PyCont.Continuation**: *Curve classes: Continuation, EquilibriumCurve, FoldCurve,
      HopfCurveOne, HopfCurveTwo*
    - **PyDSTool.PyCont.Plotting**: *Plotting class and function*
    - **PyDSTool.PyCont.TestFunc**: *Test functions*
    - **PyDSTool.PyCont.misc**: *Common functions*
  - **PyDSTool.Redirector**: *Redirect stdout / stderr to temp file*
  - **PyDSTool.Symbolic**: *Symbolic expression support, and associated utilities.*
  - **PyDSTool.Toolbox**: *Toolbox utilities for applications.*
    - **PyDSTool.Toolbox.ActivationFuncs**
    - **PyDSTool.Toolbox.DSSRT\_tools**: *DSSRT interface tools.*
    - **PyDSTool.Toolbox.FR**
    - **PyDSTool.Toolbox.FSM**: *Finite State Machines.*
    - **PyDSTool.Toolbox.InputProfile**
    - **PyDSTool.Toolbox.ModelEst**: *Model estimation classes for ODEs.*
    - **PyDSTool.Toolbox.ModelHelper**: *A set of functions to help build ODE models with complete sets of
      standard events, etc.*
    - **PyDSTool.Toolbox.NineML**: *PySCes interface code for systems biology modeling and SBML model
      markup.*
    - **PyDSTool.Toolbox.PRCtools**: *Toolbox for phase response curves measured by finite perturbations*
    - **PyDSTool.Toolbox.ParamEst**: *Parameter estimation classes for ODEs.*
    - **PyDSTool.Toolbox.PySCes\_SBML**: *PySCes interface code for systems biology modeling and SBML model
      markup.*
    - **PyDSTool.Toolbox.adjointPRC**
    - **PyDSTool.Toolbox.data\_analysis**: *Data analysis utilities*
    - **PyDSTool.Toolbox.dataanalysis**: *Data analysis utilities*
    - **PyDSTool.Toolbox.dssrt**: *Implementation of dominant scale analysis techniques for python.*
    - **PyDSTool.Toolbox.event\_driven\_simulator**
    - **PyDSTool.Toolbox.fracdim**: *Fractal dimension estimates for analysis of datasets having rank 2
      or 3.*
    - **PyDSTool.Toolbox.makeSloppyModel**
    - **PyDSTool.Toolbox.mechmatlib**
    - **PyDSTool.Toolbox.model\_primitives**: *Library of common model equation primitives.*
    - **PyDSTool.Toolbox.neuralcomp**: *An example set of basic compartmental model ModelSpec classes
      for use in computational neuroscience modeling.*
    - **PyDSTool.Toolbox.neuro\_data**
    - **PyDSTool.Toolbox.optimizers**: *Optimization module*
      - **PyDSTool.Toolbox.optimizers.criterion**: *Module containing every criteria for converge test*
        - **PyDSTool.Toolbox.optimizers.criterion.composite\_criteria**: *Composite criteria allow to use several criteria together, with
          and/or composition*
        - **PyDSTool.Toolbox.optimizers.criterion.criteria**: *A list of standard convergence criteria based on the number of
          iterations, the last values taken by the cost function and the
          associated points*
        - **PyDSTool.Toolbox.optimizers.criterion.facilities**: *Proposes a way to create a composite criterion*
        - **PyDSTool.Toolbox.optimizers.criterion.information\_criteria**
      - **PyDSTool.Toolbox.optimizers.defaults**: *Defines the defaults parameters for the generic optimizer framework*
      - **PyDSTool.Toolbox.optimizers.helpers**: *Helper functions*
        - **PyDSTool.Toolbox.optimizers.helpers.finite\_difference**
        - **PyDSTool.Toolbox.optimizers.helpers.levenberg\_marquardt**
        - **PyDSTool.Toolbox.optimizers.helpers.quadratic**
      - **PyDSTool.Toolbox.optimizers.line\_search**: *Module containing the line searchers*
        - **PyDSTool.Toolbox.optimizers.line\_search.adaptive\_last\_step\_modifier**: *Line search decorator that overrides the default alpha\_step value
          with a factor times the last alpha\_step, the factor being a
          function of the current direction and the last direction*
        - **PyDSTool.Toolbox.optimizers.line\_search.backtracking\_search**
        - **PyDSTool.Toolbox.optimizers.line\_search.barzilai\_borwein\_non\_monotone\_search**
        - **PyDSTool.Toolbox.optimizers.line\_search.barzilai\_borwein\_search**
        - **PyDSTool.Toolbox.optimizers.line\_search.cubic\_interpolation**: *Line Search with the cubic interpolation method with the
          computation of the gradient of the function*
        - **PyDSTool.Toolbox.optimizers.line\_search.damped\_line\_search**: *A damped line search*
        - **PyDSTool.Toolbox.optimizers.line\_search.fibonacci\_section**: *Line Search with the Fibonacci section method*
        - **PyDSTool.Toolbox.optimizers.line\_search.fixed\_last\_step\_modifier**: *Line search decorator that overrides the default alpha\_step value
          with a factor times the last alpha\_step*
        - **PyDSTool.Toolbox.optimizers.line\_search.golden\_section**: *Line Search with the golden section method*
        - **PyDSTool.Toolbox.optimizers.line\_search.goldstein\_rule**
        - **PyDSTool.Toolbox.optimizers.line\_search.hyperbolic\_line\_search**: *An hyperbolic line search, in fact no searches at all*
        - **PyDSTool.Toolbox.optimizers.line\_search.quadratic\_interpolation**: *Line Search with the quadratic interpolation method with the
          computation of the gradient of the function*
        - **PyDSTool.Toolbox.optimizers.line\_search.scaled\_line\_search**
        - **PyDSTool.Toolbox.optimizers.line\_search.simple\_line\_search**: *A simple line search, in fact no searches at all*
        - **PyDSTool.Toolbox.optimizers.line\_search.strong\_wolfe\_powell\_rule**
        - **PyDSTool.Toolbox.optimizers.line\_search.wolfe\_powell\_rule**
      - **PyDSTool.Toolbox.optimizers.optimizer**: *Module containing the core optimizers*
        - **PyDSTool.Toolbox.optimizers.optimizer.optimizer**: *The core optimizer from which every other optimizer is derived*
        - **PyDSTool.Toolbox.optimizers.optimizer.standard\_optimizer**: *A standard optimizer*
        - **PyDSTool.Toolbox.optimizers.optimizer.standard\_optimizer\_modifying**: *A standard optimizer with a special object that modifies the
          resulting set of parameters*
      - **PyDSTool.Toolbox.optimizers.step**: *Module containing every step use to lower a cost function*
        - **PyDSTool.Toolbox.optimizers.step.conjugate\_gradient\_step**: *Computes the conjugate gradient steps for a specific function at a
          specific point*
        - **PyDSTool.Toolbox.optimizers.step.goldfeld\_step**: *Computes Goldfeld step for a specific function at a specific point*
        - **PyDSTool.Toolbox.optimizers.step.goldstein\_price\_step**: *Computes Goldstein-Price step for a specific function at a specific
          point*
        - **PyDSTool.Toolbox.optimizers.step.gradient\_step**: *Computes a gradient step for a specific function at a specific
          point*
        - **PyDSTool.Toolbox.optimizers.step.local\_brute\_force\_1dstep**
        - **PyDSTool.Toolbox.optimizers.step.marquardt\_step**: *Computes a Marquardt step for a specific function at a specific
          point*
        - **PyDSTool.Toolbox.optimizers.step.newton\_step**: *Computes a Newton step for a specific function at a specific point*
        - **PyDSTool.Toolbox.optimizers.step.partial\_step**: *Computes a partial step for a specific function at a specific
          point, acting like a decorator for other steps*
        - **PyDSTool.Toolbox.optimizers.step.quasi\_newton\_step**: *Computes a quasi-Newton step for a specific function at a specific
          point*
        - **PyDSTool.Toolbox.optimizers.step.restart\_conjugate\_gradient**: *Restarts a conjugate gradient search by deleting the step key in
          the state dictionary*
      - **PyDSTool.Toolbox.optimizers.tests**
        - **PyDSTool.Toolbox.optimizers.tests.test\_powell**: *Class defining the Powell function*
        - **PyDSTool.Toolbox.optimizers.tests.test\_quadratic**: *Class defining a quadratic function*
        - **PyDSTool.Toolbox.optimizers.tests.test\_rosenbrock**: *Class defining the Rosenbrock function*
    - **PyDSTool.Toolbox.phaseplane**: *Phase plane utilities.*
    - **PyDSTool.Toolbox.prep\_boxplot**
    - **PyDSTool.Toolbox.synthetic\_data**: *Helper functions for creating synthetic data*
    - **PyDSTool.Toolbox.syntheticdata**: *Helper functions for creating synthetic data*
    - **PyDSTool.Toolbox.test\_protocols**
  - **PyDSTool.Trajectory**
  - **PyDSTool.Trajectory'**: *Trajectory classes.*
  - **PyDSTool.Variable**
  - **PyDSTool.Variable'**: *Variable is a one-dimensional discrete and continuous real variable
    class.*
  - **PyDSTool.common**: *Internal utilities.*
  - **PyDSTool.conf**
  - **PyDSTool.errors**
  - **PyDSTool.fixedpickle**: *Create portable serialized representations of Python objects.*
  - **PyDSTool.integrator**
  - **PyDSTool.integrator'**: *Basic integrator interface class Erik Sherwood, September 2006*
  - **PyDSTool.matplotlib\_import**: *Plotting imports for PyDSTool, from Matplotlib.*
  - **PyDSTool.parseUtils**: *Parser utilities.*
  - **PyDSTool.scipy\_ode**: *User-friendly interface to various numerical integrators for solving a
    system of first order ODEs with prescribed initial conditions:*
  - **PyDSTool.utils**: *User utilities.*
- **cPickle**: *C implementation and optimization of the Python pickle module.*
- **matplotlib.pylab**: *This is a procedural interface to the matplotlib object-oriented
  plotting library.*
- **scipy.integrate.vode**: *This module 'vode' is auto-generated with f2py (version:2\_5540).*

| Home | Trees | Indices | Help | | PyDSTool | | --- | |
| --- | --- | --- | --- | --- | --- |

|  |  |
| --- | --- |
| Generated by Epydoc 3.0.1 on Fri May 4 15:24:00 2012 | http://epydoc.sourceforge.net |
