# Supplementary material for: Hybrid Models and Biological Model Reduction with PyDSTool
Source: PLoS Comput Biol. 2012 Aug 9;8(8):e1002628. doi: 10.1371/journal.pcbi.1002628 (PMC3415397; doi:10.1371/journal.pcbi.1002628)
Supplement: Text S4 — Complete source code for the PyDSTool package (version 0.88.120504). Includes API documentation and help files linking to web pages. This file is identical to the current public release on Sourceforge.net. (ZIP) [file pcbi.1002628.s004.zip › PyDSTool/html/PyDSTool-module.html]

xml version="1.0" encoding="ascii"?


PyDSTool


| Home | Trees | Indices | Help | | PyDSTool | | --- | |
| --- | --- | --- | --- | --- | --- |

|  |  |  |  |
| --- | --- | --- | --- |
| Package PyDSTool | |  | | --- | | [hide private] | | [frames] | no frames] | |

# Package PyDSTool

source code

PyDSTool initialization script.

Copyright (C) 2007-2012 Georgia State University

print PyDSTool.\_\_LICENSE\_\_ for the terms of use.

---

**Version:**
0.88

**Date:**
$Date: 2012/04/01 19:50:00 $


|  |  |  |  |
| --- | --- | --- | --- |
| |  |  | | --- | --- | | Submodules | [hide private] | | |
| - **PyDSTool.Events**: *Event handling for python-based computations, and specification for   both python and externally compiled code.* - **PyDSTool.FuncSpec** - **PyDSTool.FuncSpec'**: *Functional specification classes.* - **PyDSTool.Generator**: *Trajectory generator classes.*   - **PyDSTool.Generator.ADMC\_ODEsystem**   - **PyDSTool.Generator.ADMC\_ODEsystem'**   - **PyDSTool.Generator.DDEsystem**   - **PyDSTool.Generator.Dopri\_ODEsystem**   - **PyDSTool.Generator.Dopri\_ODEsystem'**   - **PyDSTool.Generator.EmbeddedSysGen**   - **PyDSTool.Generator.EmbeddedSysGen'**   - **PyDSTool.Generator.Euler\_ODEsystem**   - **PyDSTool.Generator.Euler\_ODEsystem'**: *Euler integrator for ODE systems, with no step refinement for     events.*   - **PyDSTool.Generator.ExplicitFnGen**   - **PyDSTool.Generator.ExplicitFnGen'**   - **PyDSTool.Generator.ExtrapolateTable**   - **PyDSTool.Generator.ExtrapolateTable'**   - **PyDSTool.Generator.ImplicitFnGen**   - **PyDSTool.Generator.ImplicitFnGen'**   - **PyDSTool.Generator.InterpolateTable**   - **PyDSTool.Generator.InterpolateTable'**   - **PyDSTool.Generator.LookupTable**   - **PyDSTool.Generator.LookupTable'**   - **PyDSTool.Generator.MapSystem**   - **PyDSTool.Generator.MapSystem'**   - **PyDSTool.Generator.ODEsystem**   - **PyDSTool.Generator.ODEsystem'**   - **PyDSTool.Generator.Radau\_ODEsystem**   - **PyDSTool.Generator.Radau\_ODEsystem'**   - **PyDSTool.Generator.Vode\_ODEsystem**   - **PyDSTool.Generator.Vode\_ODEsystem'**: *VODE integrator for ODE systems, imported from a mild modification     of the scipy-wrapped VODE Fortran solver.*   - **PyDSTool.Generator.allimports**   - **PyDSTool.Generator.baseclasses**   - **PyDSTool.Generator.messagecodes**: *Message code definitions for Generators* - **PyDSTool.Interval** - **PyDSTool.Interval'**: *Interval class* - **PyDSTool.MProject**: *Modelling project and associated classes.* - **PyDSTool.Model**: *General purpose (hybrid) model class, and associated hybrid trajectory   and variable classes.* - **PyDSTool.ModelConstructor** - **PyDSTool.ModelConstructor'**: *Model Constructor classes.* - **PyDSTool.ModelSpec** - **PyDSTool.ModelSpec'**: *Structured model specification classes, and associated utilities.* - **PyDSTool.Points**: *Point and Pointset enhanced array classes.* - **PyDSTool.PyCont**   - **PyDSTool.PyCont.BifPoint**: *Bifurcation point classes.*   - **PyDSTool.PyCont.ContClass**   - **PyDSTool.PyCont.ContClass'**: *ContClass stores continuation curves for a specified model.*   - **PyDSTool.PyCont.Continuation**: *Curve classes: Continuation, EquilibriumCurve, FoldCurve,     HopfCurveOne, HopfCurveTwo*   - **PyDSTool.PyCont.Plotting**: *Plotting class and function*   - **PyDSTool.PyCont.TestFunc**: *Test functions*   - **PyDSTool.PyCont.misc**: *Common functions* - **PyDSTool.Redirector**: *Redirect stdout / stderr to temp file* - **PyDSTool.Symbolic**: *Symbolic expression support, and associated utilities.* - **PyDSTool.Toolbox**: *Toolbox utilities for applications.*   - **PyDSTool.Toolbox.ActivationFuncs**   - **PyDSTool.Toolbox.DSSRT\_tools**: *DSSRT interface tools.*   - **PyDSTool.Toolbox.FR**   - **PyDSTool.Toolbox.FSM**: *Finite State Machines.*   - **PyDSTool.Toolbox.InputProfile**   - **PyDSTool.Toolbox.ModelEst**: *Model estimation classes for ODEs.*   - **PyDSTool.Toolbox.ModelHelper**: *A set of functions to help build ODE models with complete sets of     standard events, etc.*   - **PyDSTool.Toolbox.NineML**: *PySCes interface code for systems biology modeling and SBML model     markup.*   - **PyDSTool.Toolbox.PRCtools**: *Toolbox for phase response curves measured by finite perturbations*   - **PyDSTool.Toolbox.ParamEst**: *Parameter estimation classes for ODEs.*   - **PyDSTool.Toolbox.PySCes\_SBML**: *PySCes interface code for systems biology modeling and SBML model     markup.*   - **PyDSTool.Toolbox.adjointPRC**   - **PyDSTool.Toolbox.data\_analysis**: *Data analysis utilities*   - **PyDSTool.Toolbox.dataanalysis**: *Data analysis utilities*   - **PyDSTool.Toolbox.dssrt**: *Implementation of dominant scale analysis techniques for python.*   - **PyDSTool.Toolbox.event\_driven\_simulator**   - **PyDSTool.Toolbox.fracdim**: *Fractal dimension estimates for analysis of datasets having rank 2     or 3.*   - **PyDSTool.Toolbox.makeSloppyModel**   - **PyDSTool.Toolbox.mechmatlib**   - **PyDSTool.Toolbox.model\_primitives**: *Library of common model equation primitives.*   - **PyDSTool.Toolbox.neuralcomp**: *An example set of basic compartmental model ModelSpec classes     for use in computational neuroscience modeling.*   - **PyDSTool.Toolbox.neuro\_data**   - **PyDSTool.Toolbox.optimizers**: *Optimization module*     - **PyDSTool.Toolbox.optimizers.criterion**: *Module containing every criteria for converge test*       - **PyDSTool.Toolbox.optimizers.criterion.composite\_criteria**: *Composite criteria allow to use several criteria together, with         and/or composition*       - **PyDSTool.Toolbox.optimizers.criterion.criteria**: *A list of standard convergence criteria based on the number of         iterations, the last values taken by the cost function and the         associated points*       - **PyDSTool.Toolbox.optimizers.criterion.facilities**: *Proposes a way to create a composite criterion*       - **PyDSTool.Toolbox.optimizers.criterion.information\_criteria**     - **PyDSTool.Toolbox.optimizers.defaults**: *Defines the defaults parameters for the generic optimizer framework*     - **PyDSTool.Toolbox.optimizers.helpers**: *Helper functions*       - **PyDSTool.Toolbox.optimizers.helpers.finite\_difference**       - **PyDSTool.Toolbox.optimizers.helpers.levenberg\_marquardt**       - **PyDSTool.Toolbox.optimizers.helpers.quadratic**     - **PyDSTool.Toolbox.optimizers.line\_search**: *Module containing the line searchers*       - **PyDSTool.Toolbox.optimizers.line\_search.adaptive\_last\_step\_modifier**: *Line search decorator that overrides the default alpha\_step value         with a factor times the last alpha\_step, the factor being a         function of the current direction and the last direction*       - **PyDSTool.Toolbox.optimizers.line\_search.backtracking\_search**       - **PyDSTool.Toolbox.optimizers.line\_search.barzilai\_borwein\_non\_monotone\_search**       - **PyDSTool.Toolbox.optimizers.line\_search.barzilai\_borwein\_search**       - **PyDSTool.Toolbox.optimizers.line\_search.cubic\_interpolation**: *Line Search with the cubic interpolation method with the         computation of the gradient of the function*       - **PyDSTool.Toolbox.optimizers.line\_search.damped\_line\_search**: *A damped line search*       - **PyDSTool.Toolbox.optimizers.line\_search.fibonacci\_section**: *Line Search with the Fibonacci section method*       - **PyDSTool.Toolbox.optimizers.line\_search.fixed\_last\_step\_modifier**: *Line search decorator that overrides the default alpha\_step value         with a factor times the last alpha\_step*       - **PyDSTool.Toolbox.optimizers.line\_search.golden\_section**: *Line Search with the golden section method*       - **PyDSTool.Toolbox.optimizers.line\_search.goldstein\_rule**       - **PyDSTool.Toolbox.optimizers.line\_search.hyperbolic\_line\_search**: *An hyperbolic line search, in fact no searches at all*       - **PyDSTool.Toolbox.optimizers.line\_search.quadratic\_interpolation**: *Line Search with the quadratic interpolation method with the         computation of the gradient of the function*       - **PyDSTool.Toolbox.optimizers.line\_search.scaled\_line\_search**       - **PyDSTool.Toolbox.optimizers.line\_search.simple\_line\_search**: *A simple line search, in fact no searches at all*       - **PyDSTool.Toolbox.optimizers.line\_search.strong\_wolfe\_powell\_rule**       - **PyDSTool.Toolbox.optimizers.line\_search.wolfe\_powell\_rule**     - **PyDSTool.Toolbox.optimizers.optimizer**: *Module containing the core optimizers*       - **PyDSTool.Toolbox.optimizers.optimizer.optimizer**: *The core optimizer from which every other optimizer is derived*       - **PyDSTool.Toolbox.optimizers.optimizer.standard\_optimizer**: *A standard optimizer*       - **PyDSTool.Toolbox.optimizers.optimizer.standard\_optimizer\_modifying**: *A standard optimizer with a special object that modifies the         resulting set of parameters*     - **PyDSTool.Toolbox.optimizers.step**: *Module containing every step use to lower a cost function*       - **PyDSTool.Toolbox.optimizers.step.conjugate\_gradient\_step**: *Computes the conjugate gradient steps for a specific function at a         specific point*       - **PyDSTool.Toolbox.optimizers.step.goldfeld\_step**: *Computes Goldfeld step for a specific function at a specific point*       - **PyDSTool.Toolbox.optimizers.step.goldstein\_price\_step**: *Computes Goldstein-Price step for a specific function at a specific         point*       - **PyDSTool.Toolbox.optimizers.step.gradient\_step**: *Computes a gradient step for a specific function at a specific         point*       - **PyDSTool.Toolbox.optimizers.step.local\_brute\_force\_1dstep**       - **PyDSTool.Toolbox.optimizers.step.marquardt\_step**: *Computes a Marquardt step for a specific function at a specific         point*       - **PyDSTool.Toolbox.optimizers.step.newton\_step**: *Computes a Newton step for a specific function at a specific point*       - **PyDSTool.Toolbox.optimizers.step.partial\_step**: *Computes a partial step for a specific function at a specific         point, acting like a decorator for other steps*       - **PyDSTool.Toolbox.optimizers.step.quasi\_newton\_step**: *Computes a quasi-Newton step for a specific function at a specific         point*       - **PyDSTool.Toolbox.optimizers.step.restart\_conjugate\_gradient**: *Restarts a conjugate gradient search by deleting the step key in         the state dictionary*     - **PyDSTool.Toolbox.optimizers.tests**       - **PyDSTool.Toolbox.optimizers.tests.test\_powell**: *Class defining the Powell function*       - **PyDSTool.Toolbox.optimizers.tests.test\_quadratic**: *Class defining a quadratic function*       - **PyDSTool.Toolbox.optimizers.tests.test\_rosenbrock**: *Class defining the Rosenbrock function*   - **PyDSTool.Toolbox.phaseplane**: *Phase plane utilities.*   - **PyDSTool.Toolbox.prep\_boxplot**   - **PyDSTool.Toolbox.synthetic\_data**: *Helper functions for creating synthetic data*   - **PyDSTool.Toolbox.syntheticdata**: *Helper functions for creating synthetic data*   - **PyDSTool.Toolbox.test\_protocols** - **PyDSTool.Trajectory** - **PyDSTool.Trajectory'**: *Trajectory classes.* - **PyDSTool.Variable** - **PyDSTool.Variable'**: *Variable is a one-dimensional discrete and continuous real variable   class.* - **PyDSTool.common**: *Internal utilities.* - **PyDSTool.conf** - **PyDSTool.errors** - **PyDSTool.fixedpickle**: *Create portable serialized representations of Python objects.* - **PyDSTool.integrator** - **PyDSTool.integrator'**: *Basic integrator interface class Erik Sherwood, September 2006* - **PyDSTool.matplotlib\_import**: *Plotting imports for PyDSTool, from Matplotlib.* - **PyDSTool.parseUtils**: *Parser utilities.* - **PyDSTool.scipy\_ode**: *User-friendly interface to various numerical integrators for solving a   system of first order ODEs with prescribed initial conditions:* - **PyDSTool.utils**: *User utilities.* |

  


|  |  |  |  |
| --- | --- | --- | --- |
| |  |  | | --- | --- | | Functions | [hide private] | | |
|  | |  |  | | --- | --- | | who(typelist=None, objdict=None, verboselevel=0, returnlevel=0, deepSearch=False, \_localCall=False)  Information about the PyDSTool user-created objects of types specified by typelist (defaults to all PyDSTool types and numpy arrays), from the objdict dictionary (or from globals() if this is not given). | source code | |
|  | |  |  | | --- | --- | | saveSession(sessionName=None, force=False, silent=False, deepSearch=False) | source code | |
|  | |  |  | | --- | --- | | loadSession(sessionName, tolocals=False)  Use tolocals boolean option if loading a session into the local namespace of the caller (i.e. | source code | |
|  | |  |  | | --- | --- | | restart(delall=0)  restart clears out global databases of PyDSTool objects, and with the optional argument delall=1 will delete all PyDSTool objects found at the top-level of the caller's namespace (not including numpy arrays). | source code | |


|  |  |  |  |
| --- | --- | --- | --- |
| |  |  | | --- | --- | | Variables | [hide private] | | |
|  | \_\_LICENSE\_\_ = `'Copyright (C) 2007-2012, Georgia State Universi...` |
|  | \_\_revision\_\_ = `'$Revision: 120401 $'` |
|  | verstr\_parts = `['0', '7', '0']` |
|  | \_pyDSToolTypes = `[<type 'numpy.ndarray'>, <class 'PyDSTool.Gen...` |
|  | \_\_session\_ext = `'ses'` |
|  | \_\_symbolic\_ext = `'sym'` |
|  | ALLOW\_THREADS = `1` |
|  | Abs = `Abs (ModelSpec wrapper)` |
|  | Acos = `Acos (ModelSpec wrapper)` |
|  | Asin = `Asin (ModelSpec wrapper)` |
|  | Atan = `Atan (ModelSpec wrapper)` |
|  | Atan2 = `Atan2 (ModelSpec wrapper)` |
|  | BUFSIZE = `10000` |
|  | Betavariate = `Betavariate (ModelSpec wrapper)` |
|  | CLIP = `0` |
|  | Ceil = `Ceil (ModelSpec wrapper)` |
|  | Choice = `Choice (ModelSpec wrapper)` |
|  | Cos = `Cos (ModelSpec wrapper)` |
|  | Cosh = `Cosh (ModelSpec wrapper)` |
|  | Degrees = `Degrees (ModelSpec wrapper)` |
|  | E = `QuantSpec e (ExpFuncSpec)` |
|  | ERR\_CALL = `3` |
|  | ERR\_DEFAULT = `0` |
|  | ERR\_DEFAULT2 = `2084` |
|  | ERR\_IGNORE = `0` |
|  | ERR\_LOG = `5` |
|  | ERR\_PRINT = `4` |
|  | ERR\_RAISE = `2` |
|  | ERR\_WARN = `1` |
|  | Exp = `Exp (ModelSpec wrapper)` |
|  | Expovariate = `Expovariate (ModelSpec wrapper)` |
|  | FLOATING\_POINT\_SUPPORT = `1` |
|  | FPE\_DIVIDEBYZERO = `1` |
|  | FPE\_INVALID = `8` |
|  | FPE\_OVERFLOW = `2` |
|  | FPE\_UNDERFLOW = `4` |
|  | Fabs = `Fabs (ModelSpec wrapper)` |
|  | False\_ = `False` |
|  | Floor = `Floor (ModelSpec wrapper)` |
|  | Fmod = `Fmod (ModelSpec wrapper)` |
|  | Frexp = `Frexp (ModelSpec wrapper)` |
|  | Gammavariate = `Gammavariate (ModelSpec wrapper)` |
|  | Gauss = `Gauss (ModelSpec wrapper)` |
|  | Getrandbits = `Getrandbits (ModelSpec wrapper)` |
|  | Getstate = `Getstate (ModelSpec wrapper)` |
|  | Hypot = `Hypot (ModelSpec wrapper)` |
|  | Infinity = `inf` |
|  | Jumpahead = `Jumpahead (ModelSpec wrapper)` |
|  | Ldexp = `Ldexp (ModelSpec wrapper)` |
|  | Log = `Log (ModelSpec wrapper)` |
|  | Log10 = `Log10 (ModelSpec wrapper)` |
|  | Lognormvariate = `Lognormvariate (ModelSpec wrapper)` |
|  | MAXDIMS = `32` |
|  | Max = `Max (ModelSpec wrapper)` |
|  | Min = `Min (ModelSpec wrapper)` |
|  | Modf = `Modf (ModelSpec wrapper)` |
|  | NAN = `nan` |
|  | NINF = `-inf` |
|  | NZERO = `-0.0` |
|  | Normalvariate = `Normalvariate (ModelSpec wrapper)` |
|  | PINF = `inf` |
|  | PZERO = `0.0` |
|  | Paretovariate = `Paretovariate (ModelSpec wrapper)` |
|  | Pi = `QuantSpec pi (ExpFuncSpec)` |
|  | Pow = `Pow (ModelSpec wrapper)` |
|  | RAISE = `2` |
|  | Radians = `Radians (ModelSpec wrapper)` |
|  | Randint = `Randint (ModelSpec wrapper)` |
|  | Random = `Random (ModelSpec wrapper)` |
|  | Randrange = `Randrange (ModelSpec wrapper)` |
|  | SHIFT\_DIVIDEBYZERO = `0` |
|  | SHIFT\_INVALID = `9` |
|  | SHIFT\_OVERFLOW = `3` |
|  | SHIFT\_UNDERFLOW = `6` |
|  | Sample = `Sample (ModelSpec wrapper)` |
|  | ScalarType = `(<type 'int'>, <type 'float'>, <type 'complex'>, ...` |
|  | Seed = `Seed (ModelSpec wrapper)` |
|  | Setstate = `Setstate (ModelSpec wrapper)` |
|  | Shuffle = `Shuffle (ModelSpec wrapper)` |
|  | Sin = `Sin (ModelSpec wrapper)` |
|  | Sinh = `Sinh (ModelSpec wrapper)` |
|  | Sqrt = `Sqrt (ModelSpec wrapper)` |
|  | Sum = `Sum (ModelSpec wrapper)` |
|  | Systemrandom = `Systemrandom (ModelSpec wrapper)` |
|  | Tan = `Tan (ModelSpec wrapper)` |
|  | Tanh = `Tanh (ModelSpec wrapper)` |
|  | True\_ = `True` |
|  | UFUNC\_BUFSIZE\_DEFAULT = `10000` |
|  | UFUNC\_PYVALS\_NAME = `'UFUNC_PYVALS'` |
|  | Uniform = `Uniform (ModelSpec wrapper)` |
|  | Vonmisesvariate = `Vonmisesvariate (ModelSpec wrapper)` |
|  | WRAP = `1` |
|  | Weibullvariate = `Weibullvariate (ModelSpec wrapper)` |
|  | Wichmannhill = `Wichmannhill (ModelSpec wrapper)` |
|  | \_1DimplicitSolveMethods = `['newton', 'bisect', 'steffe']` |
|  | \_implicitSolveMethods = `['newton', 'bisect', 'steffe', 'fsolve']` |
|  | absolute = `<ufunc 'absolute'>` |
|  | add = `<ufunc 'add'>` |
|  | bitwise\_and = `<ufunc 'bitwise_and'>` |
|  | bitwise\_not = `<ufunc 'invert'>` |
|  | bitwise\_or = `<ufunc 'bitwise_or'>` |
|  | bitwise\_xor = `<ufunc 'bitwise_xor'>` |
|  | c\_ = `<numpy.lib.index_tricks.CClass object at 0x1154330>` |
|  | cast = `{<type 'numpy.int64'>: <function <lambda> at 0x10a8bb0>...` |
|  | conj = `<ufunc 'conjugate'>` |
|  | conjugate = `<ufunc 'conjugate'>` |
|  | copysign = `<ufunc 'copysign'>` |
|  | deg2rad = `<ufunc 'deg2rad'>` |
|  | divide = `<ufunc 'divide'>` |
|  | equal = `<ufunc 'equal'>` |
|  | exp2 = `<ufunc 'exp2'>` |
|  | expm1 = `<ufunc 'expm1'>` |
|  | floor\_divide = `<ufunc 'floor_divide'>` |
|  | fmax = `<ufunc 'fmax'>` |
|  | fmin = `<ufunc 'fmin'>` |
|  | greater\_equal = `<ufunc 'greater_equal'>` |
|  | index\_exp = `<numpy.lib.index_tricks.IndexExpression object at ...` |
|  | inf = `inf` |
|  | infty = `inf` |
|  | invert = `<ufunc 'invert'>` |
|  | isinf = `<ufunc 'isinf'>` |
|  | left\_shift = `<ufunc 'left_shift'>` |
|  | little\_endian = `True` |
|  | log1p = `<ufunc 'log1p'>` |
|  | logaddexp = `<ufunc 'logaddexp'>` |
|  | logaddexp2 = `<ufunc 'logaddexp2'>` |
|  | logical\_and = `<ufunc 'logical_and'>` |
|  | logical\_not = `<ufunc 'logical_not'>` |
|  | logical\_xor = `<ufunc 'logical_xor'>` |
|  | maximum = `<ufunc 'maximum'>` |
|  | mgrid = `<numpy.lib.index_tricks.nd_grid object at 0x113d490>` |
|  | minimum = `<ufunc 'minimum'>` |
|  | multiply = `<ufunc 'multiply'>` |
|  | n = `9` |
|  | nan = `nan` |
|  | nbytes = `{<type 'numpy.int64'>: 8, <type 'numpy.int16'>: 2, <t...` |
|  | negative = `<ufunc 'negative'>` |
|  | newaxis = `None` |
|  | nextafter = `<ufunc 'nextafter'>` |
|  | not\_equal = `<ufunc 'not_equal'>` |
|  | ogrid = `<numpy.lib.index_tricks.nd_grid object at 0x113dbb0>` |
|  | ones\_like = `<ufunc 'ones_like'>` |
|  | r\_ = `<numpy.lib.index_tricks.RClass object at 0x11542f0>` |
|  | rad2deg = `<ufunc 'rad2deg'>` |
|  | reciprocal = `<ufunc 'reciprocal'>` |
|  | remainder = `<ufunc 'remainder'>` |
|  | right\_shift = `<ufunc 'right_shift'>` |
|  | rint = `<ufunc 'rint'>` |
|  | s\_ = `<numpy.lib.index_tricks.IndexExpression object at 0x11543f0>` |
|  | sctypeDict = `{0: <type 'numpy.bool_'>, 1: <type 'numpy.int8'>,...` |
|  | sctypeNA = `{'?': 'Bool', 'B': 'UInt8', 'Bool': <type 'numpy.bo...` |
|  | sctypes = `{'complex': [<type 'numpy.complex64'>, <type 'numpy....` |
|  | signbit = `<ufunc 'signbit'>` |
|  | spacing = `<ufunc 'spacing'>` |
|  | square = `<ufunc 'square'>` |
|  | subtract = `<ufunc 'subtract'>` |
|  | t = `'0'` |
|  | true\_divide = `<ufunc 'true_divide'>` |
|  | trunc = `<ufunc 'trunc'>` |
|  | typeDict = `{0: <type 'numpy.bool_'>, 1: <type 'numpy.int8'>, 2...` |
|  | typeNA = `{'?': 'Bool', 'B': 'UInt8', 'Bool': <type 'numpy.bool...` |
|  | typecodes = `{'All': '?bhilqpBHILQPfdgFDGSUVOMm', 'AllFloat': '...` |


|  |  |  |  |
| --- | --- | --- | --- |
| |  |  | | --- | --- | | Function Details | [hide private] | | |

|  |  |  |
| --- | --- | --- |
| |  |  | | --- | --- | | who(typelist=None, objdict=None, verboselevel=0, returnlevel=0, deepSearch=False, \_localCall=False) | source code |   Information about the PyDSTool user-created objects of types specified by typelist (defaults to all PyDSTool types and numpy arrays), from the objdict dictionary (or from globals() if this is not given).  returnlevel > 0 puts who() into silent mode, and it just returns either (1) a list of the objects found, or (2) a dictionary of object names -> objects found.  deepSearch = True causes a shallow search of list, tuple and dictionary objects defined at the topmost level of objdict. Otherwise only PyDSTool objects visible at the topmost level will be seen. |

|  |  |  |
| --- | --- | --- |
| |  |  | | --- | --- | | loadSession(sessionName, tolocals=False) | source code |   Use tolocals boolean option if loading a session into the local namespace of the caller (i.e. if calling this from within a function rather than interactively at the prompt) |

|  |  |  |
| --- | --- | --- |
| |  |  | | --- | --- | | restart(delall=0) | source code |   restart clears out global databases of PyDSTool objects, and with the optional argument delall=1 will delete all PyDSTool objects found at the top-level of the caller's namespace (not including numpy arrays).  delall=2 will cause a one-level deeper search of lists, tuples, and dictionaries for PyDSTool objects, and the lists etc. will be deleted. Additionally, numpy arrays will be deleted. |

  


|  |  |  |  |
| --- | --- | --- | --- |
| |  |  | | --- | --- | | Variables Details | [hide private] | | |

|  |  |
| --- | --- |
| \_\_LICENSE\_\_   Value:  |  | | --- | | ``` '''Copyright (C) 2007-2012, Georgia State University All rights reserved.  Parts of this distribution that originate from different authors are individually marked as such. Copyright and licensing of those parts re mains with the original authors.  ... ``` | |

|  |  |
| --- | --- |
| \_pyDSToolTypes   Value:  |  | | --- | | ``` [<type 'numpy.ndarray'>,  <class 'PyDSTool.Generator.baseclasses.Generator'>,  <class 'PyDSTool.Variable.Variable'>,  <class 'PyDSTool.Trajectory.Trajectory'>,  <class 'PyDSTool.Events.Event'>,  <class 'PyDSTool.Events.EventStruct'>,  <class 'PyDSTool.Points.Point'>,  <class 'PyDSTool.Points.Pointset'>, ... ``` | |

|  |  |
| --- | --- |
| ScalarType   Value:  |  | | --- | | ``` (<type 'int'>,  <type 'float'>,  <type 'complex'>,  <type 'long'>,  <type 'bool'>,  <type 'str'>,  <type 'unicode'>,  <type 'buffer'>, ... ``` | |

|  |  |
| --- | --- |
| cast   Value:  |  | | --- | | ``` {<type 'numpy.int64'>: <function <lambda> at 0x10a8bb0>, <type 'numpy. int16'>: <function <lambda> at 0x10a8bf0>, <type 'numpy.complex128'>:  <function <lambda> at 0x10a8c30>, <type 'numpy.uint64'>: <function <la mbda> at 0x10a8c70>, <type 'numpy.complex256'>: <function <lambda> at  0x10a8cf0>, <type 'numpy.float32'>: <function <lambda> at 0x10a8d30>,  <type 'numpy.bool_'>: <function <lambda> at 0x10a8cb0>, <type 'numpy.u int8'>: <function <lambda> at 0x10a8db0>, <type 'numpy.int32'>: <funct ion <lambda> at 0x10a8eb0>, <type 'numpy.int8'>: <function <lambda> at ... ``` | |

|  |  |
| --- | --- |
| index\_exp   Value:  |  | | --- | | ``` <numpy.lib.index_tricks.IndexExpression object at 0x11543b0> ``` | |

|  |  |
| --- | --- |
| nbytes   Value:  |  | | --- | | ``` {<type 'numpy.int64'>: 8, <type 'numpy.int16'>: 2, <type 'numpy.comple x128'>: 16, <type 'numpy.uint64'>: 8, <type 'numpy.bool_'>: 1, <type ' numpy.complex256'>: 32, <type 'numpy.float32'>: 4, <type 'numpy.int8'> : 1, <type 'numpy.uint8'>: 1, <type 'numpy.uint16'>: 2, <type 'numpy.o bject_'>: 4, <type 'numpy.float64'>: 8, <type 'numpy.int32'>: 4, <type  'numpy.string_'>: 0, <type 'numpy.void'>: 0, <type 'numpy.float128'>:  16, <type 'numpy.int32'>: 4, <type 'numpy.uint32'>: 4, <type 'numpy.u nicode_'>: 0, <type 'numpy.complex64'>: 8, <type 'numpy.uint32'>: 4} ``` | |

|  |  |
| --- | --- |
| sctypeDict   Value:  |  | | --- | | ``` {0: <type 'numpy.bool_'>,  1: <type 'numpy.int8'>,  2: <type 'numpy.uint8'>,  3: <type 'numpy.int16'>,  4: <type 'numpy.uint16'>,  5: <type 'numpy.int32'>,  6: <type 'numpy.uint32'>,  7: <type 'numpy.int32'>, ... ``` | |

|  |  |
| --- | --- |
| sctypeNA   Value:  |  | | --- | | ``` {'?': 'Bool',  'B': 'UInt8',  'Bool': <type 'numpy.bool_'>,  'Complex128': <type 'numpy.complex256'>,  'Complex32': <type 'numpy.complex64'>,  'Complex64': <type 'numpy.complex128'>,  'D': 'Complex64',  'F': 'Complex32', ... ``` | |

|  |  |
| --- | --- |
| sctypes   Value:  |  | | --- | | ``` {'complex': [<type 'numpy.complex64'>,              <type 'numpy.complex128'>,              <type 'numpy.complex256'>],  'float': [<type 'numpy.float32'>,            <type 'numpy.float64'>,            <type 'numpy.float128'>],  'int': [<type 'numpy.int8'>,          <type 'numpy.int16'>, ... ``` | |

|  |  |
| --- | --- |
| typeDict   Value:  |  | | --- | | ``` {0: <type 'numpy.bool_'>,  1: <type 'numpy.int8'>,  2: <type 'numpy.uint8'>,  3: <type 'numpy.int16'>,  4: <type 'numpy.uint16'>,  5: <type 'numpy.int32'>,  6: <type 'numpy.uint32'>,  7: <type 'numpy.int32'>, ... ``` | |

|  |  |
| --- | --- |
| typeNA   Value:  |  | | --- | | ``` {'?': 'Bool',  'B': 'UInt8',  'Bool': <type 'numpy.bool_'>,  'Complex128': <type 'numpy.complex256'>,  'Complex32': <type 'numpy.complex64'>,  'Complex64': <type 'numpy.complex128'>,  'D': 'Complex64',  'F': 'Complex32', ... ``` | |

|  |  |
| --- | --- |
| typecodes   Value:  |  | | --- | | ``` {'All': '?bhilqpBHILQPfdgFDGSUVOMm',  'AllFloat': 'fdgFDG',  'AllInteger': 'bBhHiIlLqQpP',  'Character': 'c',  'Complex': 'FDG',  'Datetime': 'Mm',  'Float': 'fdg',  'Integer': 'bhilqp', ... ``` | |

  


| Home | Trees | Indices | Help | | PyDSTool | | --- | |
| --- | --- | --- | --- | --- | --- |

|  |  |
| --- | --- |
| Generated by Epydoc 3.0.1 on Fri May 4 15:24:02 2012 | http://epydoc.sourceforge.net |
