# Supplementary material for: Hybrid Models and Biological Model Reduction with PyDSTool
Source: PLoS Comput Biol. 2012 Aug 9;8(8):e1002628. doi: 10.1371/journal.pcbi.1002628 (PMC3415397; doi:10.1371/journal.pcbi.1002628)
Supplement: Text S4 — Complete source code for the PyDSTool package (version 0.88.120504). Includes API documentation and help files linking to web pages. This file is identical to the current public release on Sourceforge.net. (ZIP) [file pcbi.1002628.s004.zip › PyDSTool/html/PyDSTool-pysrc.html]

xml version="1.0" encoding="ascii"?


PyDSTool


| Home | Trees | Indices | Help | | PyDSTool | | --- | |
| --- | --- | --- | --- | --- | --- |

|  |  |  |  |
| --- | --- | --- | --- |
| Package PyDSTool | |  | | --- | | [hide private] | | [frames] | no frames] | |

# Source Code for Package PyDSTool

```
  1  """PyDSTool initialization script.
 
  2  
 
  3  Copyright (C) 2007-2012 Georgia State University
 
  4  
 
  5  print PyDSTool.__LICENSE__    for the terms of use.
 
  6  """ 
  7  
 
  8  __LICENSE__ = """\
 
  9  Copyright (C) 2007-2012, Georgia State University
 
 10  All rights reserved.
 
 11  
 
 12  Parts of this distribution that originate from different authors are
 
 13  individually marked as such. Copyright and licensing of those parts remains
 
 14  with the original authors.
 
 15  
 
 16  Redistribution and use in source and binary forms, with or without
 
 17  modification, are permitted provided that the following conditions are met:
 
 18  
 
 19      1. Redistributions of source code must retain the above copyright
 
 20        notice, this list of conditions and the following disclaimer.
 
 21  
 
 22      2. Redistributions in binary form must reproduce the above
 
 23        copyright notice, this list of conditions and the following
 
 24        disclaimer in the documentation and/or other materials provided
 
 25        with the distribution.
 
 26  
 
 27      3. The name of Georgia State University and its representatives may not
 
 28        be used to endorse or promote products derived from this
 
 29        software without specific prior written permission.
 
 30  
 
 31  THIS SOFTWARE IS PROVIDED BY GEORGIA STATE UNIVERSITY ``AS IS'' AND ANY
 
 32  EXPRESS OR IMPLIED WARRANTIES, INCLUDING, BUT NOT LIMITED TO, THE
 
 33  IMPLIED WARRANTIES OF MERCHANTABILITY AND FITNESS FOR A PARTICULAR
 
 34  PURPOSE ARE DISCLAIMED. IN NO EVENT SHALL GEORGIA STATE UNIVERSITY BE LIABLE
 
 35  FOR ANY DIRECT, INDIRECT, INCIDENTAL, SPECIAL, EXEMPLARY, OR
 
 36  CONSEQUENTIAL DAMAGES (INCLUDING, BUT NOT LIMITED TO, PROCUREMENT OF
 
 37  SUBSTITUTE GOODS OR SERVICES; LOSS OF USE, DATA, OR PROFITS; OR
 
 38  BUSINESS INTERRUPTION) HOWEVER CAUSED AND ON ANY THEORY OF LIABILITY,
 
 39  WHETHER IN CONTRACT, STRICT LIABILITY, OR TORT (INCLUDING NEGLIGENCE
 
 40  OR OTHERWISE) ARISING IN ANY WAY OUT OF THE USE OF THIS SOFTWARE, EVEN
 
 41  IF ADVISED OF THE POSSIBILITY OF SUCH DAMAGE.
 
 42  
 
 43  """ 
 44  
 
 45  vernum = '0.88' 
 46  __version__  = vernum 
 47  __revision__ = '$Revision: 120401 $' 
 48  __date__     = '$Date: 2012/04/01 19:50:00 $' 
 49  
 
 50  import sys, os, gc 
 51  
 
 52  try: 
 53      import scipy 
 54  except ImportError: 
 55      raise RuntimeError("SciPy is required") 
 56  try: 
 57      verstr_parts = scipy.__version__.split('.') 
 58  except AttributeError: 
 59      verstr_parts = scipy.__scipy_version__.split('.') 
 60  digits = [str(n) for n in range(10)] 
 61  vernums = [] 
 62  # filter out any trailing alphabetic chars in version number,
 
 63  # e.g. for release candidates
 
 64  temp_str = '' 
 65  for s in verstr_parts: 
 66      for t in s: 
 67          if t in digits: 
 68              temp_str += t 
 69          else: 
 70              break 
 71      try: 
 72          vernums.append(int(temp_str)) 
 73      except ValueError: 
 74          # wholly alphabetical component, e.g. in '0.5.2.dev'
 
 75          pass 
 76      temp_str = '' 
 77  assert len(vernums) > 0 
 78  if vernums[1] < 5: 
 79      raise RuntimeError("SciPy v0.5.1 or above is required") 
 80  del digits, vernums, temp_str 
 81  
 
 82  import math, random 
 83  import types, time 
 84  
 
 85  # PyDSTool imports
 
 86  from Events import * 
 87  from Interval import * 
 88  from Points import * 
 89  from Variable import * 
 90  from Trajectory import * 
 91  from FuncSpec import * 
 92  # \begin{hacksRus}
 
 93  import Generator as GenModule 
 94  from Generator import Generator as Generator_ 
 95  from Generator import * 
 96  Generator = GenModule 
 97  import Model as ModelModule 
 98  from Model import Model as Model_ 
 99  from Model import * 
100  Model = ModelModule 
101  # \end{hacksRus}
 
102  from ModelConstructor import * 
103  from Toolbox.ParamEst import ParamEst 
104  from Toolbox.ModelEst import ModelEst 
105  from MProject import * 
106  from Symbolic import * 
107  from ModelSpec import * 
108  from parseUtils import auxfnDBclass, protected_allnames, protected_auxnamesDB, \
 
109           convertPowers 
110  from PyCont import * 
111  import numpy 
112  import numpy as npy  # alternate 
113  # import scipy already done at top
 
114  import scipy as spy  # alternate 
115  from math import * 
116  from matplotlib import pyplot as plt 
117  from matplotlib.pyplot import figure, plot, show, draw, hold 
118  from numpy import *    # overwrites __version__ for PyDSTool, so fix now 
119  # Restore PyDSTool version
 
120  __version__ = vernum 
121  del vernum 
122  
 
123  #from numpy.linalg import *
 
124  from copy import copy 
125  # note that the names with leading underscores will not be exported by
 
126  # "from PyDSTool import *"
 
127  # diff overwrites numpy diff
 
128  from common import Verbose, Continuous, Discrete, targetLangs, _seq_types, \
 
129                _num_types, _int_types, _float_types, _complex_types, \
 
130                _real_types, _all_numpy_int, _all_numpy_float, \
 
131                _all_numpy_complex, _all_int, _all_float, _all_complex, \
 
132                LargestInt32, diff, diff2 
133  from scipy import who as scipy_who 
134  from utils import * 
135  
 
136  
 
137  # ------ Check Python version compatibility
 
138  major, minor1, minor2, s, tmp = sys.version_info 
139  _validpython = major==2 and minor1>=4 
140  
 
141  if not _validpython: 
142      raise RuntimeError("Python 2.4 or later is required to run PyDSTool") 
143  del _validpython, major, minor1, minor2, s, tmp 
144  
 
145  _pyDSToolTypes = [ndarray, Generator_, Variable, Trajectory, Event,
 
146                  EventStruct, Point, Pointset, Interval, ParamEst,
 
147                  Model_, Quantity, ModelSpec, QuantSpec, ModelEst,
 
148                  ModelConstructor, auxfnDBclass, nameResolverClass,
 
149                  ContClass, GeneratorConstructor, condition, feature,
 
150                  GeneratorInterface, ModelInterface, ModelManager,
 
151                  ModelTransform, GDescriptor, MDescriptor, context,
 
152                  ModelLibrary] 
153  
 


154 -def who(typelist=None, objdict=None, verboselevel=0, returnlevel=0,
 
155          deepSearch=False, _localCall=False):


156      """Information about the PyDSTool user-created objects of types
 
157      specified by typelist (defaults to all PyDSTool types and
 
158      numpy arrays), from the objdict dictionary (or from globals() if this
 
159      is not given).
 
160  
 
161      returnlevel > 0 puts who() into silent mode, and it just returns
 
162      either (1) a list of the objects found, or (2) a dictionary of object
 
163      names ->  objects found.
 
164  
 
165      deepSearch = True causes a shallow search of list, tuple and dictionary
 
166      objects defined at the topmost level of objdict. Otherwise only
 
167      PyDSTool objects visible at the topmost level will be seen.
 
168      """ 
169      objdict_out = {}   # for returning if returnlevel > 0 
170      if objdict is None: 
171          if _localCall: 
172              # e.g. from saveSession or restart, then need to look one
 
173              # stack frame further back.
 
174              frame = sys._getframe().f_back.f_back 
175          else: 
176              frame = sys._getframe().f_back 
177          objdict = frame.f_globals 
178      # Generator_ is an alias for PyDSTool.Generator.baseclasses.Generator
 
179      # Model_ is an alias for PyDSTool.Model.Model
 
180      if typelist is None: 
181          typelist_actual = _pyDSToolTypes 
182      elif isinstance(typelist, list): 
183          # hacks for taking care of class naming problems
 
184          typelist_actual = [] 
185          for t in typelist: 
186              if t == Model: 
187                  # user meant Model.Model, i.e. local name Model_
 
188                  typelist_actual.append(Model_) 
189              elif t == Generator: 
190                  # user meant Generator.Generator, i.e. local name Generator_
 
191                  typelist_actual.append(Generator_) 
192              elif t == array: 
193                  # user meant numpy.ndarray
 
194                  typelist_actual.append(numpy.ndarray) 
195              else: 
196                  typelist_actual.append(t) 
197                  #if compareClassAndBases(t, _pyDSToolTypes):
 
198                  #    typelist_actual.append(t)
 
199                  #else:
 
200                  #    raise TypeError("Invalid PyDSTool object types passed")
 
201      else: 
202          # hacks for taking care of class naming problems
 
203          # when typelist is a singleton (type)
 
204          if typelist == Model: 
205              # user meant Model.Model, i.e. local name Model_
 
206              typelist_actual = [Model_] 
207          elif typelist == Generator: 
208              # user meant Generator.Generator, i.e. local name Generator_
 
209              typelist_actual = [Generator_] 
210          elif typelist == array: 
211              typelist_actual = [numpy.ndarray] 
212          else: 
213              typelist_actual = [typelist] 
214          #elif compareClassAndBases(typelist, _pyDSToolTypes):
 
215          #    typelist_actual = [typelist]
 
216          #else:
 
217          #    raise TypeError("Invalid PyDSTool object types passed")
 
218      for objname, obj in objdict.iteritems(): 
219          if type(obj) not in [type, types.ClassType, types.ModuleType]: 
220              if compareClassAndBases(obj, typelist_actual): 
221                  if isinstance(obj, QuantSpec) and objname in protected_allnames: 
222                      # don't display internally-created QuantSpecs (i.e. all
 
223                      # of the wrappers of the math functions and constants)
 
224                      continue 
225                  objdict_out[objname] = obj 
226              elif deepSearch: 
227                  if isinstance(obj, (list, tuple)): 
228                      if any([compareClassAndBases(x, typelist_actual) \
 
229                                   for x in obj]): 
230                          objdict_out[objname] = obj 
231                  elif isinstance(obj, dict): 
232                      if any([compareClassAndBases(x, typelist_actual) \
 
233                                   for x in obj.values()]): 
234                          objdict_out[objname] = obj 
235      if returnlevel == 1: 
236          # silent mode -- just return the objects
 
237          return objdict_out.values() 
238      elif returnlevel == 2: 
239          # silent mode -- return the objects mapped by their names
 
240          return objdict_out 
241      else: 
242          for objname, obj in objdict_out.iteritems(): 
243              # make appropriate separation between output items
 
244              if verboselevel > 0: 
245                  print "\n"*(verboselevel-1) 
246              if hasattr(obj, '_infostr') and not isinstance(obj, type): 
247                  try: 
248                      print objname + ": " + obj._infostr(verboselevel) 
249                  except: 
250                      print "Problem with: ", objname, className(obj), \
 
251                            obj.info 
252                      raise 
253              else: 
254                  print objname + " (Class " + className(obj) + ")" \
 
255                        + (verboselevel > 0)*":" 
256                  if verboselevel > 0: 
257                      info(obj, objname, recurseDepthLimit=verboselevel-1)

258  
 
259  
 
260  __session_ext = 'ses' 
261  __symbolic_ext = 'sym' 
262  
 


263 -def saveSession(sessionName=None, force=False, silent=False, deepSearch=False):


264      if sessionName is None: 
265          datestr = time.strftime("%Y %m %d _ %Hh%Mm").replace(" ","")[2:] 
266          sessionName = "Session_" + datestr 
267      objdict = who(returnlevel=2, _localCall=True, deepSearch=deepSearch) 
268      objnamelist, objlist = sortedDictLists(objdict, byvalue=False) 
269      # objnamelist stores the original names of the objects saved, for use
 
270      # when restoring the session
 
271      objlist.append(objnamelist) 
272      saveObjects(objlist, sessionName+'.'+__session_ext, force) 
273      if not silent: 
274          print "Important!" 
275          print "If you used any user-defined classes for ModelSpec, these need to " 
276          print "be recreated by running their definition scripts when you restore " 
277          print "the session. saveSession only saves class _instances_."

278      #Symbolic.saveDiffs(sessionName+'.'+__symbolic_ext)
 
279  
 
280  
 


281 -def loadSession(sessionName, tolocals=False):


282      """Use tolocals boolean option if loading a session into the local
 
283      namespace of the caller (i.e. if calling this from within a function
 
284      rather than interactively at the prompt)""" 
285  
 
286      sessionName_split = sessionName.split('.') 
287      if sessionName_split[-1] != __session_ext: 
288          sessionName = sessionName + '.' + __session_ext 
289      try: 
290          loadlist = loadObjects(sessionName) 
291      except: 
292          print "Problem loading session " + sessionName 
293          raise 
294      numobjs = len(loadlist) - 1   # last entry is obj name list 
295      if len(loadlist) <= 0: 
296          raise ValueError, "Session was empty!" 
297      objnamelist = loadlist[-1] 
298      objlist = loadlist[:-1] 
299      frame = sys._getframe().f_back 
300      if tolocals: 
301          nspace = frame.f_locals 
302      else: 
303          nspace = frame.f_globals 
304      # bind the original session names for the objects to the objects
 
305      try: 
306          for i in xrange(numobjs): 
307              nspace[objnamelist[i]] = objlist[i] 
308      except: 
309          print "Problem recreating objects" 
310          print "Debug info: ", len(objnamelist), len(objlist), numobjs 
311          raise

312      # load any symbolic derivatives previously auto-saved
 
313      #symbolic.loadDiffs(sessionName+'.'+__symbolic_ext)
 
314  
 
315  
 


316 -def restart(delall=0):


317      """restart clears out global databases of PyDSTool objects,
 
318      and with the optional argument delall=1 will delete all PyDSTool
 
319      objects found at the top-level of the caller's namespace (not including
 
320      numpy arrays).
 
321  
 
322      delall=2 will cause a one-level deeper search of lists, tuples, and
 
323      dictionaries for PyDSTool objects, and the lists etc. will be deleted.
 
324      Additionally, numpy arrays will be deleted.
 
325      """ 
326      nameResolver.clearall() 
327      genDB.clearall() 
328      protected_auxnamesDB.clearall() 
329      if delall>0: 
330          deep = delall==2 
331          objdict = who(returnlevel=2, _localCall=True, deepSearch=deep) 
332          frame = sys._getframe().f_back 
333          nspace = frame.f_globals 
334          for objname, obj in objdict.iteritems(): 
335              if objname not in ['nameResolver', 'protected_auxnamesDB'] and \
 
336                 (not isinstance(obj, ndarray) or delall==2): 
337                  # don't delete those types of global objects
 
338                  del nspace[objname] 
339          del objdict, frame, nspace 
340          gc.collect()

341
```

  


| Home | Trees | Indices | Help | | PyDSTool | | --- | |
| --- | --- | --- | --- | --- | --- |

|  |  |
| --- | --- |
| Generated by Epydoc 3.0.1 on Fri May 4 15:24:23 2012 | http://epydoc.sourceforge.net |
