# Supplementary material for: Hybrid Models and Biological Model Reduction with PyDSTool
Source: PLoS Comput Biol. 2012 Aug 9;8(8):e1002628. doi: 10.1371/journal.pcbi.1002628 (PMC3415397; doi:10.1371/journal.pcbi.1002628)
Supplement: Text S4 — Complete source code for the PyDSTool package (version 0.88.120504). Includes API documentation and help files linking to web pages. This file is identical to the current public release on Sourceforge.net. (ZIP) [file pcbi.1002628.s004.zip › PyDSTool/html/PyDSTool.common-module.html]

xml version="1.0" encoding="ascii"?


PyDSTool.common


| Home | Trees | Indices | Help | | PyDSTool | | --- | |
| --- | --- | --- | --- | --- | --- |

|  |  |  |  |
| --- | --- | --- | --- |
| Package PyDSTool :: Module common | |  | | --- | | [hide private] | | [frames] | no frames] | |

# Module common

source code

Internal utilities.

Robert Clewley, September 2005.


|  |  |  |  |
| --- | --- | --- | --- |
| |  |  | | --- | --- | | Classes | [hide private] | | |
|  | API\_class  Adapted from . |
|  | Struct  The args class is a more sophisticated type of Struct. |
|  | DefaultDict  Dictionary with a default value for unknown keys. |
|  | predicate\_op |
|  | and\_op |
|  | or\_op |
|  | not\_op |
|  | predicate |
|  | null\_predicate\_class |
|  | metric  Abstract metric class for quantitatively comparing scalar or vector quantities. |
|  | metric\_float  Simple metric between two real-valued floats. |
|  | metric\_float\_1D  Simple metric between two real-valued floats. |
|  | metric\_L2  Measures the standard "distance" between two 1D pointsets or arrays using the L-2 norm. |
|  | metric\_L2\_1D  Measures the standard "distance" between two 1D pointsets or arrays using the L-2 norm. |
|  | metric\_weighted\_L2  Measures the standard "distance" between two 1D pointsets or arrays using the L-2 norm, after weighting by weights attribute (must set weights after creation, e.g. |
|  | metric\_weighted\_deadzone\_L2  Measures the standard "distance" between two 1D pointsets or arrays using the L-2 norm, after weighting by weights attribute. |
|  | args  Mapping object class for building arguments for class initialization calls. |
|  | Diagnostics  General purpose diagnostics manager. |
|  | Utility  Utility abstract class for manipulating and analyzing dynamical systems. |
|  | interpclass  Abstract class for interpolators. |
|  | interp0d  Design of this class based on SciPy's interp1d |
|  | interp1d |
|  | KroghInterpolator  The interpolating polynomial for a set of points |
|  | BarycentricInterpolator  The interpolating polynomial for a set of points |
|  | PiecewisePolynomial  Piecewise polynomial curve specified by points and derivatives. |
|  | fit\_function  Abstract super-class for fitting explicit functions to 1D arrays of data using least squares. |
|  | fit\_quadratic  Fit a quadratic function y=a\*x^2+b\*x+c to the (x,y) array data. |
|  | fit\_quadratic\_at\_vertex  Fit a quadratic function y=a\*(x+h)\*\*2+k to the (x,y) array data, constrained to have a vertex at (h, k), leaving only the free parameter a for the curvature. |
|  | fit\_cubic  Fit a cubic function y=a\*x^3+b\*x^2+c\*x+d to the (x,y) array data. |
|  | fit\_exponential  Fit an exponential function y=a\*exp(b\*x) to the (x,y) array data. |
|  | fit\_diff\_of\_exp  Fit a 'difference of two exponentials' function y = k\*a\*b\*(exp(-a\*x)-exp(-b\*x))/(b-a) to the (x,y) array data. |
|  | fit\_linear  Fit a linear function y=a\*x+b to the (x,y) array data. |
|  | DomainType |
|  | Verbose  A class to handle reporting. |


|  |  |  |  |
| --- | --- | --- | --- |
| |  |  | | --- | --- | | Functions | [hide private] | | |
|  | |  |  | | --- | --- | | n\_sigdigs\_str(x, n)  Return a string representation of float x with n significant digits, where n > 0 is an integer. | source code | |
|  | |  |  | | --- | --- | | get\_opt(argopt, attr, default=None)  Get option from args object otherwise default to the given value. | source code | |
|  | |  |  | | --- | --- | | compareNumTypes(t1, t2) | source code | |
|  | |  |  | | --- | --- | | filteredDict(d, keys, neg=False)  returns filtered dictionary containing specified keys, or \*not\* containing the specified keys if option neg=True. | source code | |
|  | |  |  | | --- | --- | | concatStrDict(d, order=`[``]`)  Concatenates all entries of a dictionary (assumed to be lists of strings), in optionally specified order. | source code | |
|  | |  |  | | --- | --- | | copyVarDict(vardict, only\_cts=False)  Copy dictionary of Variable objects. | source code | |
|  | |  |  | | --- | --- | | insertInOrder(sourcelist, inslist, return\_ixs=False, abseps=0)  Insert elements of inslist into sourcelist, sorting these lists in case they are not already in increasing order. | source code | |
|  | |  |  | | --- | --- | | arraymax(a1, a2, t=<type 'numpy.float64'>)  Element-wise comparison of maximum values for two arrays. | source code | |
|  | |  |  | | --- | --- | | simplifyMatrixRepr(m)  Convert matrix object to a compact array representation or numeric value. | source code | |
|  | |  |  | | --- | --- | | makeMultilinearRegrFn(arg, xs, ys)  Convert two lists or arrays mapping x intervals to y intervals into a string function definition of a multilinear regression scalar function that these define. | source code | |
|  | |  |  | | --- | --- | | \_scalar\_diff(func, x0, dx)  Numerical differentiation of scalar function by central differences. | source code | |
|  | |  |  | | --- | --- | | diff(func, x0, vars=None, axes=None, eps=None, output=None)  Numerical 1st derivative of R^N -> R^M scalar or array function about x0 by central finite differences. | source code | |
|  | |  |  | | --- | --- | | diff2(func, x0, vars=None, axes=None, dir=1, eps=None)  Numerical 1st derivative of R^N -> R^M scalar or array function about x0 by forward or backward finite differences. | source code | |
|  | |  |  | | --- | --- | | ensurefloat(v) | source code | |
|  | |  |  | | --- | --- | | verify\_values(name, value, values, list\_ok=False, list\_len=None)  Use list\_ok if a list of values of these types is acceptable. | source code | |
|  | |  |  | | --- | --- | | verify\_intbool(name, value, list\_ok=False, list\_len=None)  Use list\_ok if a list of values of these types is acceptable. | source code | |
|  | |  |  | | --- | --- | | verify\_nonneg(name, value, types, list\_ok=False, list\_len=None)  Use list\_ok if a list of values of these types is acceptable. | source code | |
|  | |  |  | | --- | --- | | verify\_pos(name, value, types, list\_ok=False, list\_len=None)  Use list\_ok if a list of values of these types is acceptable. | source code | |
|  | |  |  | | --- | --- | | array\_bounds\_check(a, bounds, dirn=1)  Internal utility function to test a 1D array for staying within given bounds (min val, max val). | source code | |
|  | |  |  | | --- | --- | | linearInterp(y0, ygoal, y1, x0, x1)  Internal utility function to linearly interpolate between two data points. | source code | |
|  | |  |  | | --- | --- | | makeUniqueFn(fstr, tdigits=0, idstr=None)  Add unique ID to function names. | source code | |
|  | |  |  | | --- | --- | | timestamp(tdigits=8)  Return a unique timestamp string for the session. | source code | |
|  | |  |  | | --- | --- | | isUniqueSeq(objlist)  Check that list contains items only once | source code | |
|  | |  |  | | --- | --- | | makeSeqUnique(seq, asarray=False)  Return a 1D sequence that only contains the unique values in seq. | source code | |
|  | |  |  | | --- | --- | | object2str(x)  Convert occurrences of types / classes, to pretty-printable strings. | source code | |
|  | |  |  | | --- | --- | | compareBaseClass(input, baseClass)  input may be a class or a class instance representing that class. | source code | |
|  | |  |  | | --- | --- | | compareClassAndBases(input, arg)  arg can be a single or sequence of classes | source code | |
|  | |  |  | | --- | --- | | getSuperClasses(obj, limitClasses=None)  Return string names of all super classes of a given object | source code | |
|  | |  |  | | --- | --- | | className(obj, addPrefix=False)  Return human-readable string of class name. | source code | |
|  | |  |  | | --- | --- | | listid(val) | source code | |
|  | |  |  | | --- | --- | | idfn(val) | source code | |
|  | |  |  | | --- | --- | | noneFn(x) | source code | |
|  | |  |  | | --- | --- | | makeArrayIxMap(a) | source code | |
|  | |  |  | | --- | --- | | invertMap(themap)  invert an index mapping or other form of mapping. | source code | |
|  | |  |  | | --- | --- | | isincreasing(theseq, withVal=False)  Check whether a sequence is in increasing order. | source code | |
|  | |  |  | | --- | --- | | ismonotonic(theseq, withVal=False)  Check whether a sequence is in strictly increasing or decreasing order. | source code | |
|  | |  |  | | --- | --- | | extent(data)  Returns a pair of the min and max values of a dataset, or just a numeric type if these are equal. | source code | |
|  | |  |  | | --- | --- | | uniquePoints(ar)  For an n by m array input, return only points that are unique | source code | |
|  | |  |  | | --- | --- | | sortedDictValues(d, onlykeys=None, reverse=False)  Return list of values from a dictionary in order of sorted key list. | source code | |
|  | |  |  | | --- | --- | | sortedDictKeys(d, onlykeys=None, reverse=False)  Return sorted list of keys from a dictionary. | source code | |
|  | |  |  | | --- | --- | | sortedDictLists(d, byvalue=True, onlykeys=None, reverse=False)  Return (key list, value list) pair from a dictionary, sorted by value (default) or key. | source code | |
|  | |  |  | | --- | --- | | sortedDictItems(d, byvalue=True, onlykeys=None, reverse=False)  Return list of (key, value) pairs of a dictionary, sorted by value (default) or key. | source code | |
|  | |  |  | | --- | --- | | intersect(a, b) | source code | |
|  | |  |  | | --- | --- | | remain(a, b) | source code | |
|  | |  |  | | --- | --- | | simple\_bisection(tlo, thi, f, tol, imax=100) | source code | |
|  | |  |  | | --- | --- | | make\_poly\_interpolated\_curve(pts, coord, model)  Only for a 1D curve from a Model object (that has an associated vector field for defining 1st derivative of curve). | source code | |
|  | |  |  | | --- | --- | | smooth\_pts(t, x, q=None)  Use a local quadratic fit on a set of nearby 1D points and obtain a function that represents that fit in that neighbourhood. | source code | |
|  | |  |  | | --- | --- | | nearest\_2n\_indices(x, i, n)  Calculates the nearest 2n indices centred at i in an array x, or as close as possible to i, taking into account that i might be within n indices of an endpoint of x. | source code | |


|  |  |  |  |
| --- | --- | --- | --- |
| |  |  | | --- | --- | | Variables | [hide private] | | |
|  | Inf = `inf` |
|  | NaN = `nan` |
|  | less = `<ufunc 'less'>` |
|  | greater = `<ufunc 'greater'>` |
|  | logical\_or = `<ufunc 'logical_or'>` |
|  | isfinite = `<ufunc 'isfinite'>` |
|  | sign = `<ufunc 'sign'>` |
|  | exp = `<ufunc 'exp'>` |
|  | log = `<ufunc 'log'>` |
|  | less\_equal = `<ufunc 'less_equal'>` |
|  | float96 |
|  | \_all\_numpy\_float = `(<type 'numpy.float64'>, <type 'numpy.float...` |
|  | complex192 |
|  | \_all\_numpy\_complex = `(<type 'numpy.complex128'>, <type 'numpy....` |
|  | \_classes = `['Verbose', 'interpclass', 'interp0d', 'interp1d', ...` |
|  | \_mappings = `['_num_type2name', '_num_name2type', '_num_equivty...` |
|  | \_functions = `['isUniqueSeq', 'makeArrayIxMap', 'className', 'c...` |
|  | \_constants = `['Continuous', 'Discrete', 'targetLangs', '_seq_t...` |
|  | targetLangs = `['c', 'python', 'matlab']` |
|  | \_num\_types = `(<type 'float'>, <type 'int'>, <type 'numpy.float...` |
|  | \_int\_types = `(<type 'int'>, <type 'numpy.integer'>)` |
|  | \_float\_types = `(<type 'float'>, <type 'numpy.floating'>)` |
|  | \_complex\_types = `(<type 'complex'>, <type 'numpy.complexfloati...` |
|  | \_real\_types = `(<type 'int'>, <type 'numpy.integer'>, <type 'fl...` |
|  | \_seq\_types = `(<type 'list'>, <type 'tuple'>, <type 'numpy.ndar...` |
|  | \_all\_numpy\_int = `(<type 'numpy.int32'>, <type 'numpy.int32'>, ...` |
|  | \_all\_int = `(<type 'int'>, <type 'numpy.integer'>, <type 'numpy...` |
|  | \_all\_float = `(<type 'float'>, <type 'numpy.floating'>, <type '...` |
|  | \_all\_complex = `(<type 'complex'>, <type 'numpy.complexfloating...` |
|  | LargestInt32 = `2147483647` |
|  | Macheps = `2.2204460492503131e-16` |
|  | \_num\_type2name = `{<type 'float'>: 'float', <type 'int'>: 'int'...` |
|  | \_num\_equivtype = `{<type 'float'>: <type 'numpy.float64'>, <typ...` |
|  | \_num\_name2equivtypes = `{'float': (<type 'float'>, <type 'numpy...` |
|  | \_num\_name2type = `{'float': <type 'numpy.float64'>, 'int': <typ...` |
|  | \_num\_maxmin = `{<type 'numpy.int32'>: [-2147483648, 2147483647]...` |
|  | \_typefrompytype = `{<type 'float'>: <type 'numpy.float64'>, <ty...` |
|  | \_pytypefromtype = `{<type 'numpy.int32'>: <type 'int'>, <type '...` |
|  | API = `API_class()` |
|  | null\_predicate = `null_predicate_class(None)` |
|  | \_verify\_type\_names = `{(<type 'complex'>, <type 'numpy.complexf...` |
|  | Continuous = `Continuous Domain` |
|  | Discrete = `Discrete Domain` |


|  |  |  |  |
| --- | --- | --- | --- |
| |  |  | | --- | --- | | Function Details | [hide private] | | |

|  |  |  |
| --- | --- | --- |
| |  |  | | --- | --- | | get\_opt(argopt, attr, default=None) | source code |   Get option from args object otherwise default to the given value. Can also specify that an AttributeError is raised by passing default=Exception. |

|  |  |  |
| --- | --- | --- |
| |  |  | | --- | --- | | copyVarDict(vardict, only\_cts=False) | source code |   Copy dictionary of Variable objects. Use the only\_cts Boolean optional argument (default False) to select only continuous-valued variables (mainly for internal use). |

|  |  |  |
| --- | --- | --- |
| |  |  | | --- | --- | | insertInOrder(sourcelist, inslist, return\_ixs=False, abseps=0) | source code |  ``` Insert elements of inslist into sourcelist, sorting these   lists in case they are not already in increasing order. The new   list is returned.  The function will not create duplicate entries in the list, and will   change neither the first or last entries of the list.  If sourcelist is an array, an array is returned. If optional return_ixs=True, the indices of the inserted elements   in the returned list is returned as an additional return argument. If abseps=0 (default) the comparison of elements is done exactly. For   abseps > 0 elements are compared up to an absolute difference no   greater than abseps for determining "equality". ``` |

|  |  |  |
| --- | --- | --- |
| |  |  | | --- | --- | | makeMultilinearRegrFn(arg, xs, ys) | source code |   Convert two lists or arrays mapping x intervals to y intervals into a string function definition of a multilinear regression scalar function that these define. A.k.a. makes a "piecewise linear" scalar function from the input data. The two input data sequences can each be either all numeric values or all strings/symbolic objects, but not a mixture. |

|  |  |  |
| --- | --- | --- |
| |  |  | | --- | --- | | \_scalar\_diff(func, x0, dx) | source code |   Numerical differentiation of scalar function by central differences. Returns tuple containing derivative evaluated at x0 and error estimate, using Ridders' method and Neville's algorithm. |

|  |  |  |
| --- | --- | --- |
| |  |  | | --- | --- | | diff(func, x0, vars=None, axes=None, eps=None, output=None) | source code |  ``` Numerical 1st derivative of R^N -> R^M scalar or array function about x0 by central finite differences. Uses Ridders' method of polynomial extrapolation, based on an implementation in the book "Numerical Recipes". Returns a matrix.  vars argument specifies which elements of x0 are to be treated as   variables for the purposes of taking the Jacobian. If axes argument is unused or set to be all axes, the Jacobian of the   function evaluated at x0 with respect to the variables is returned,   otherwise a sub-matrix of it is returned. eps is assumed to be the scale in x for which the function varies by O(1).   If eps is not given an appropriate step size is chosen. output = True returns an optional dictionary which will be updated   with error and derivative information. ``` |

|  |  |  |
| --- | --- | --- |
| |  |  | | --- | --- | | diff2(func, x0, vars=None, axes=None, dir=1, eps=None) | source code |  ``` Numerical 1st derivative of R^N -> R^M scalar or array function about x0 by forward or backward finite differences. Returns a matrix.  dir=1 uses finite forward difference. dir=-1 uses finite backward difference. List-valued eps rescales finite differencing in each axis separately. vars argument specifies which elements of x0 are to be treated as   variables for the purposes of taking the Jacobian. If axes argument is unused or set to be all axes, the Jacobian of the   function evaluated at x0 with respect to the variables is returned,   otherwise a sub-matrix of it is returned. eps is assumed to be the scale in x for which the function varies by O(1).   If eps is not given an appropriate step size is chosen   (proportional to sqrt(machine precision)). ``` |

|  |  |  |
| --- | --- | --- |
| |  |  | | --- | --- | | verify\_values(name, value, values, list\_ok=False, list\_len=None) | source code |   Use list\_ok if a list of values of these types is acceptable. list\_len can be used to specify that a list must be of a certain length, either a fixed integer or a variable integer value given as the first value of a pair, the second being the name of the variable (for use in error messages) |

|  |  |  |
| --- | --- | --- |
| |  |  | | --- | --- | | verify\_intbool(name, value, list\_ok=False, list\_len=None) | source code |   Use list\_ok if a list of values of these types is acceptable. list\_len can be used to specify that a list must be of a certain length, either a fixed integer or a variable integer value given as the first value of a pair, the second being the name of the variable (for use in error messages) |

|  |  |  |
| --- | --- | --- |
| |  |  | | --- | --- | | verify\_nonneg(name, value, types, list\_ok=False, list\_len=None) | source code |   Use list\_ok if a list of values of these types is acceptable. list\_len can be used to specify that a list must be of a certain length, either a fixed integer or a variable integer value given as the first value of a pair, the second being the name of the variable (for use in error messages) |

|  |  |  |
| --- | --- | --- |
| |  |  | | --- | --- | | verify\_pos(name, value, types, list\_ok=False, list\_len=None) | source code |   Use list\_ok if a list of values of these types is acceptable. list\_len can be used to specify that a list must be of a certain length, either a fixed integer or a variable integer value given as the first value of a pair, the second being the name of the variable (for use in error messages) |

|  |  |  |
| --- | --- | --- |
| |  |  | | --- | --- | | array\_bounds\_check(a, bounds, dirn=1) | source code |   Internal utility function to test a 1D array for staying within given bounds (min val, max val).  Returns the largest index +1 if the array is within bounds, otherwise the first offending index, where 'first' is the earliest in a if direction dirn=1, or the latest if dirn=-1. |

|  |  |  |
| --- | --- | --- |
| |  |  | | --- | --- | | makeUniqueFn(fstr, tdigits=0, idstr=None) | source code |   Add unique ID to function names.  Used when functions are executed in global namespace to avoid name clashes, and need to be distinguished when DS objects are copied. |

|  |  |  |
| --- | --- | --- |
| |  |  | | --- | --- | | timestamp(tdigits=8) | source code |   Return a unique timestamp string for the session. useful for ensuring unique function identifiers, etc. |

|  |  |  |
| --- | --- | --- |
| |  |  | | --- | --- | | makeSeqUnique(seq, asarray=False) | source code |   Return a 1D sequence that only contains the unique values in seq. Adapted from code by Raymond Hettinger, 2002 |

|  |  |  |
| --- | --- | --- |
| |  |  | | --- | --- | | compareBaseClass(input, baseClass) | source code |   input may be a class or a class instance representing that class. baseClass may be a class or a string name of a class.  Comparison is made using class names only. |

|  |  |  |
| --- | --- | --- |
| |  |  | | --- | --- | | invertMap(themap) | source code |   invert an index mapping or other form of mapping.  If argument is a dict or sequence type, returns a dictionary, but if argument is a parseUtils.symbolMapClass then that type is returned. |

|  |  |  |
| --- | --- | --- |
| |  |  | | --- | --- | | isincreasing(theseq, withVal=False) | source code |   Check whether a sequence is in increasing order. The withVal option (default False) causes the function to return the first two offending values that are not repeated. |

|  |  |  |
| --- | --- | --- |
| |  |  | | --- | --- | | ismonotonic(theseq, withVal=False) | source code |   Check whether a sequence is in strictly increasing or decreasing order. The withVal option (default False) causes the function to return the first two offending values that are not repeated. |

|  |  |  |
| --- | --- | --- |
| |  |  | | --- | --- | | extent(data) | source code |   Returns a pair of the min and max values of a dataset, or just a numeric type if these are equal. (Ignores NaNs.) |

|  |  |  |
| --- | --- | --- |
| |  |  | | --- | --- | | sortedDictValues(d, onlykeys=None, reverse=False) | source code |  ``` Return list of values from a dictionary in order of sorted key list.  Adapted from original function by Alex Martelli:  added filtering of keys. ``` |

|  |  |  |
| --- | --- | --- |
| |  |  | | --- | --- | | sortedDictKeys(d, onlykeys=None, reverse=False) | source code |  ``` Return sorted list of keys from a dictionary.  Adapted from original function by Alex Martelli:  added filtering of keys. ``` |

|  |  |  |
| --- | --- | --- |
| |  |  | | --- | --- | | sortedDictLists(d, byvalue=True, onlykeys=None, reverse=False) | source code |   Return (key list, value list) pair from a dictionary, sorted by value (default) or key. Adapted from an original function by Duncan Booth. |

|  |  |  |
| --- | --- | --- |
| |  |  | | --- | --- | | sortedDictItems(d, byvalue=True, onlykeys=None, reverse=False) | source code |   Return list of (key, value) pairs of a dictionary, sorted by value (default) or key. Adapted from an original function by Duncan Booth. |

|  |  |  |
| --- | --- | --- |
| |  |  | | --- | --- | | smooth\_pts(t, x, q=None) | source code |   Use a local quadratic fit on a set of nearby 1D points and obtain a function that represents that fit in that neighbourhood. Returns a structure (args object) with attributes ys\_fit, pars\_fit, info, and results. The function can be referenced as results.f  Assumed that pts is small enough that it is either purely concave up or down but that at it contains at least five points.  If this function is used repeatedly, pass a fit\_quadratic instance as the argument q |

|  |  |  |
| --- | --- | --- |
| |  |  | | --- | --- | | nearest\_2n\_indices(x, i, n) | source code |   Calculates the nearest 2n indices centred at i in an array x, or as close as possible to i, taking into account that i might be within n indices of an endpoint of x.  The function returns the limiting indices as a pair, and always returns an interval that contains 2n+1 indices, assuming x is long enough.  I.e., away from endpoints, the function returns (i-n, i+n). If i is within n of index 0, the function returns (0, 2n). If i is within n of last index L, the function returns (L-2n, L).  Remember to add one to the upper limit if using it in a slice. |

  


|  |  |  |  |
| --- | --- | --- | --- |
| |  |  | | --- | --- | | Variables Details | [hide private] | | |

|  |  |
| --- | --- |
| \_all\_numpy\_float   Value:  |  | | --- | | ``` (<type 'numpy.float64'>,  <type 'numpy.float32'>,  <type 'numpy.float64'>) ``` | |

|  |  |
| --- | --- |
| \_all\_numpy\_complex   Value:  |  | | --- | | ``` (<type 'numpy.complex128'>,  <type 'numpy.complex64'>,  <type 'numpy.complex128'>) ``` | |

|  |  |
| --- | --- |
| \_classes   Value:  |  | | --- | | ``` ['Verbose',  'interpclass',  'interp0d',  'interp1d',  'Utility',  'args',  'DefaultDict',  'Struct', ... ``` | |

|  |  |
| --- | --- |
| \_mappings   Value:  |  | | --- | | ``` ['_num_type2name',  '_num_name2type',  '_num_equivtype',  '_num_name2equivtypes',  '_pytypefromtype',  '_num_maxmin'] ``` | |

|  |  |
| --- | --- |
| \_functions   Value:  |  | | --- | | ``` ['isUniqueSeq',  'makeArrayIxMap',  'className',  'compareBaseClass',  'compareClassAndBases',  'timestamp',  'makeUniqueFn',  'copyVarDict', ... ``` | |

|  |  |
| --- | --- |
| \_constants   Value:  |  | | --- | | ``` ['Continuous',  'Discrete',  'targetLangs',  '_seq_types',  '_num_types',  '_int_types',  '_float_types',  '_complex_types', ... ``` | |

|  |  |
| --- | --- |
| \_num\_types   Value:  |  | | --- | | ``` (<type 'float'>,  <type 'int'>,  <type 'numpy.floating'>,  <type 'numpy.integer'>) ``` | |

|  |  |
| --- | --- |
| \_complex\_types   Value:  |  | | --- | | ``` (<type 'complex'>, <type 'numpy.complexfloating'>) ``` | |

|  |  |
| --- | --- |
| \_real\_types   Value:  |  | | --- | | ``` (<type 'int'>,  <type 'numpy.integer'>,  <type 'float'>,  <type 'numpy.floating'>) ``` | |

|  |  |
| --- | --- |
| \_seq\_types   Value:  |  | | --- | | ``` (<type 'list'>, <type 'tuple'>, <type 'numpy.ndarray'>) ``` | |

|  |  |
| --- | --- |
| \_all\_numpy\_int   Value:  |  | | --- | | ``` (<type 'numpy.int32'>,  <type 'numpy.int32'>,  <type 'numpy.int8'>,  <type 'numpy.int16'>,  <type 'numpy.int32'>,  <type 'numpy.int64'>) ``` | |

|  |  |
| --- | --- |
| \_all\_int   Value:  |  | | --- | | ``` (<type 'int'>,  <type 'numpy.integer'>,  <type 'numpy.int32'>,  <type 'numpy.int32'>,  <type 'numpy.int8'>,  <type 'numpy.int16'>,  <type 'numpy.int32'>,  <type 'numpy.int64'>) ``` | |

|  |  |
| --- | --- |
| \_all\_float   Value:  |  | | --- | | ``` (<type 'float'>,  <type 'numpy.floating'>,  <type 'numpy.float64'>,  <type 'numpy.float32'>,  <type 'numpy.float64'>) ``` | |

|  |  |
| --- | --- |
| \_all\_complex   Value:  |  | | --- | | ``` (<type 'complex'>,  <type 'numpy.complexfloating'>,  <type 'numpy.complex128'>,  <type 'numpy.complex64'>,  <type 'numpy.complex128'>) ``` | |

|  |  |
| --- | --- |
| \_num\_type2name   Value:  |  | | --- | | ``` {<type 'float'>: 'float',  <type 'int'>: 'int',  <type 'numpy.integer'>: 'int',  <type 'numpy.floating'>: 'float',  <type 'numpy.int8'>: 'int',  <type 'numpy.int16'>: 'int',  <type 'numpy.int32'>: 'int',  <type 'numpy.int32'>: 'int', ... ``` | |

|  |  |
| --- | --- |
| \_num\_equivtype   Value:  |  | | --- | | ``` {<type 'float'>: <type 'numpy.float64'>,  <type 'int'>: <type 'numpy.int32'>,  <type 'numpy.integer'>: <type 'numpy.int32'>,  <type 'numpy.floating'>: <type 'numpy.float64'>,  <type 'numpy.int8'>: <type 'numpy.int32'>,  <type 'numpy.int16'>: <type 'numpy.int32'>,  <type 'numpy.int32'>: <type 'numpy.int32'>,  <type 'numpy.int32'>: <type 'numpy.int32'>, ... ``` | |

|  |  |
| --- | --- |
| \_num\_name2equivtypes   Value:  |  | | --- | | ``` {'float': (<type 'float'>,            <type 'numpy.floating'>,            <type 'numpy.float64'>,            <type 'numpy.float32'>,            <type 'numpy.float64'>),  'int': (<type 'int'>,          <type 'numpy.integer'>,          <type 'numpy.int32'>, ... ``` | |

|  |  |
| --- | --- |
| \_num\_name2type   Value:  |  | | --- | | ``` {'float': <type 'numpy.float64'>, 'int': <type 'numpy.int32'>} ``` | |

|  |  |
| --- | --- |
| \_num\_maxmin   Value:  |  | | --- | | ``` {<type 'numpy.int32'>: [-2147483648, 2147483647],  <type 'numpy.float64'>: [-inf, inf]} ``` | |

|  |  |
| --- | --- |
| \_typefrompytype   Value:  |  | | --- | | ``` {<type 'float'>: <type 'numpy.float64'>,  <type 'int'>: <type 'numpy.int32'>} ``` | |

|  |  |
| --- | --- |
| \_pytypefromtype   Value:  |  | | --- | | ``` {<type 'numpy.int32'>: <type 'int'>,  <type 'numpy.float64'>: <type 'float'>} ``` | |

|  |  |
| --- | --- |
| \_verify\_type\_names   Value:  |  | | --- | | ``` {(<type 'complex'>,   <type 'numpy.complexfloating'>,   <type 'numpy.complex128'>,   <type 'numpy.complex64'>,   <type 'numpy.complex128'>): 'a complex number',  (<type 'float'>,   <type 'numpy.floating'>,   <type 'numpy.float64'>, ... ``` | |

  


| Home | Trees | Indices | Help | | PyDSTool | | --- | |
| --- | --- | --- | --- | --- | --- |

|  |  |
| --- | --- |
| Generated by Epydoc 3.0.1 on Fri May 4 15:24:05 2012 | http://epydoc.sourceforge.net |
