# Supplementary material for: Hybrid Models and Biological Model Reduction with PyDSTool
Source: PLoS Comput Biol. 2012 Aug 9;8(8):e1002628. doi: 10.1371/journal.pcbi.1002628 (PMC3415397; doi:10.1371/journal.pcbi.1002628)
Supplement: Text S4 — Complete source code for the PyDSTool package (version 0.88.120504). Includes API documentation and help files linking to web pages. This file is identical to the current public release on Sourceforge.net. (ZIP) [file pcbi.1002628.s004.zip › PyDSTool/html/PyDSTool.common-pysrc.html]

xml version="1.0" encoding="ascii"?


PyDSTool.common


| Home | Trees | Indices | Help | | PyDSTool | | --- | |
| --- | --- | --- | --- | --- | --- |

|  |  |  |  |
| --- | --- | --- | --- |
| Package PyDSTool :: Module common | |  | | --- | | [hide private] | | [frames] | no frames] | |

# Source Code for Module PyDSTool.common

```
   1  """
 
   2      Internal utilities.
 
   3  
 
   4      Robert Clewley, September 2005.
 
   5  """ 
   6  
 
   7  from errors import * 
   8  
 
   9  import sys, types 
  10  import numpy as npy 
  11  import scipy as spy 
  12  from scipy.optimize import minpack 
  13  # In future, will convert these specific imports to be referred as npy.X
 
  14  from numpy import Inf, NaN, atleast_1d, clip, less, greater, logical_or, \
 
  15       searchsorted, isfinite, shape, mat, sign, any, all, sometrue, alltrue, \
 
  16       array, swapaxes, zeros, ones, finfo, double, exp, log, \
 
  17       take, less_equal, putmask, ndarray, asarray, \
 
  18       int, float, complex, complexfloating, integer, floating, \
 
  19       int_, int0, int8, int16, int32, int64, float_, float32, float64, \
 
  20       complex_, complex64, complex128, argmin, argmax 
  21  from numpy.linalg import norm 
  22  from math import sqrt 
  23  
 
  24  try: 
  25      from numpy import float96 
  26  except ImportError: 
  27      _all_numpy_float = (float_, float32, float64) 
  28  else: 
  29      _all_numpy_float = (float_, float32, float64, float96) 
  30  
 
  31  
 
  32  try: 
  33      from numpy import complex192 
  34  except ImportError: 
  35      _all_numpy_complex = (complex_, complex64, complex128) 
  36  else: 
  37      _all_numpy_complex = (complex_, complex64, complex128, complex192) 
  38  
 
  39  
 
  40  import time 
  41  from copy import copy, deepcopy 
  42  import os 
  43  if os.name == 'nt': 
  44      # slow object copying for you guys
 
  45      import fixedpickle as pickle 
  46  else: 
  47      import cPickle as pickle 
  48  
 
  49  # ----------------------------------------------------------------------------
 
  50  ### EXPORTS
 
  51  
 
  52  _classes = ['Verbose', 'interpclass', 'interp0d', 'interp1d', 'Utility',
 
  53              'args', 'DefaultDict', 'Struct', 'pickle', 'Diagnostics',
 
  54              'metric', 'metric_float', 'metric_float_1D', 'metric_L2',
 
  55              'metric_L2_1D', 'metric_weighted_L2', 'metric_weighted_deadzone_L2',
 
  56              'predicate', 'null_predicate', 'and_op', 'or_op', 'not_op'] 
  57  
 
  58  _mappings = ['_num_type2name', '_num_name2type',
 
  59               '_num_equivtype', '_num_name2equivtypes',
 
  60               '_pytypefromtype', '_num_maxmin'
 
  61               ] 
  62  
 
  63  _functions = ['isUniqueSeq', 'makeArrayIxMap', 'className',
 
  64                'compareBaseClass', 'compareClassAndBases', 'timestamp',
 
  65                'makeUniqueFn', 'copyVarDict', 'concatStrDict',
 
  66                'invertMap', 'makeSeqUnique', 'insertInOrder', 'uniquePoints',
 
  67                'sortedDictKeys', 'sortedDictValues', 'sortedDictItems',
 
  68                'sortedDictLists', 'compareNumTypes', 'diff', 'diff2',
 
  69                'listid', 'idfn', 'noneFn', 'isincreasing', 'ismonotonic',
 
  70                'extent', 'n_sigdigs_str',
 
  71                'linearInterp', 'object2str', 'getSuperClasses',
 
  72                'filteredDict', 'arraymax', 'simplifyMatrixRepr',
 
  73                'makeMultilinearRegrFn', 'fit_quadratic', 'fit_quadratic_at_vertex',
 
  74                'fit_exponential', 'fit_diff_of_exp', 'fit_linear', 'fit_cubic',
 
  75                'smooth_pts', 'nearest_2n_indices',
 
  76                'KroghInterpolator', 'BarycentricInterpolator',
 
  77                'PiecewisePolynomial', 'make_poly_interpolated_curve',
 
  78                'simple_bisection', 'get_opt', 'array_bounds_check',
 
  79                'verify_intbool', 'verify_nonneg', 'verify_pos',
 
  80                'verify_values', 'ensurefloat', 'API'] 
  81  
 
  82  _constants = ['Continuous', 'Discrete', 'targetLangs', '_seq_types',
 
  83                '_num_types', '_int_types', '_float_types', '_complex_types',
 
  84                '_real_types', '_all_numpy_int', '_all_numpy_float',
 
  85                '_all_numpy_complex', '_all_int', '_all_float', '_all_complex',
 
  86                'LargestInt32'] 
  87  
 
  88  __all__ = _functions + _mappings + _classes + _constants 
  89  
 
  90  # ----------------------------------------------------------------------------
 
  91  
 
  92  # global reference for supported target languages
 
  93  targetLangs = ['c', 'python', 'matlab'] #, 'xpp', 'dstool' 
  94  
 
  95  
 
  96  # type mappings and groupings
 
  97  
 
  98  _num_types = (float, int, floating, integer) # complex, complexfloating 
  99  
 
 100  _int_types = (int, integer) 
 101  _float_types = (float, floating) 
 102  _complex_types = (complex, complexfloating) 
 103  _real_types = (int, integer, float, floating) 
 104  
 
 105  _seq_types = (list, tuple, ndarray) 
 106  
 
 107  _all_numpy_int = (int_, int0, int8, int16, int32, int64) 
 108  
 
 109  _all_int = (int, integer)+_all_numpy_int 
 110  _all_float = (float, floating)+_all_numpy_float 
 111  _all_complex = (complex, complexfloating)+_all_numpy_complex 
 112  
 
 113  LargestInt32 = 2147483647 
 114  Macheps = finfo(double).eps 
 115  
 
 116  # bind common names
 
 117  _num_type2name = {float: 'float', int: 'int'} #, complex: 'complex'} 
 118  _num_equivtype = {float: float64, int: int32} #, complex: complex128} 
 119  for f in _all_float: 
 120      _num_type2name[f] = 'float' 
 121      _num_equivtype[f] = float64 
 122  for i in _all_int: 
 123      _num_type2name[i] = 'int' 
 124      _num_equivtype[i] = int32 
 125  # Don't yet support complex numbers
 
 126  ##for c in _all_complex:
 
 127  ##    _num_type2name[c] = 'complex'
 
 128  ##    _num_equivtype[c] = complex128
 
 129  
 
 130  # equivalent types for comparison
 
 131  _num_name2equivtypes = {'float': _all_float,
 
 132                  'int': _all_int} 
 133  ##                'complex': _all_complex}
 
 134  
 
 135  # default types used by PyDSTool when named
 
 136  _num_name2type = {'float': float64, 'int': int32} #, 'complex': complex128} 
 137  
 
 138  _num_maxmin = {float64: [-Inf, Inf],
 
 139               int32: [-LargestInt32-1, LargestInt32],
 
 140  ##             complex128: [-Inf-Inf*1.0j, Inf+Inf*1.0j]
 
 141               } 
 142  
 
 143  _typefrompytype = {float: float64, int: int32} #, complex: complex128} 
 144  _pytypefromtype = {float64: float, int32: int} #, complex128: complex} 
 145  
 
 146  
 
 147  #-------------------------------------------------------------------------
 
 148  
 
 149  
 


150 -class API_class(object):


151      """Adapted from .""" 


152 -    def _print_values(self, obj):


153          def _print_value(key): 
 154              if key.startswith('_'): 
 155                  return '' 
 156              value = getattr(obj, key) 
 157              if not hasattr(value, 'im_func'): 
 158                  doc = type(value).__name__ 
 159              else: 
 160                  if value.__doc__ is None: 
 161                      doc = 'no docstring' 
 162                  else: 
 163                      doc = value.__doc__ 
 164              return '    %s : %s' % (key, doc)

 165          res = [_print_value(el) for el in dir(obj)] 
 166          return '\n'.join([el for el in res
 
 167                            if el != ''])

 168  
 


169 -    def __call__(self, obj):


170          if obj.__doc__ is None: 
 171              doc = 'No docstring' 
 172          else: 
 173              doc = obj.__doc__ 
 174          if hasattr(obj, '__name__'): 
 175              return obj.__name__ + " : " + doc + "\n\n" + \
 
 176                 self._print_values(obj) 
 177          else: 
 178              return doc + "\n\n" + self._print_values(obj)

 179  
 
 180  API = API_class() 
 181  
 
 182  
 


183 -class Struct(object):


184      """The args class is a more sophisticated type of Struct.
 
 185      """ 


186 -    def __init__(self, **entries):


187          self.__dict__.update(entries)

 188  
 


189 -    def __repr__(self):


190          attributes = [attr for attr in dir(self) if attr[0] != '_'] 
 191          return 'Struct(' + ', '.join(attributes) + ')'

 192  
 
 193  
 


194 -class DefaultDict(dict):


195      """Dictionary with a default value for unknown keys.
 
 196  
 
 197      Written by Peter Norvig.""" 


198 -    def __init__(self, default):


199          self.default = default

 200  
 


201 -    def __getitem__(self, key):


202          if key in self: return self.get(key) 
 203          return self.setdefault(key, deepcopy(self.default))

 204  
 
 205  
 
 206  ### PREDICATES ETC
 
 207  
 


208 -class predicate_op(object):


209 -    def __init__(self, predicates):


210          self.predicates = predicates 
 211          self.record = []

 212  
 


213 -    def precondition(self, objlist):


214          res = npy.all([p.precondition(objlist) for p in self.predicates]) 
 215          self.record = [(self.name, [p.record for p in self.predicates])] 
 216          return res

 217  
 


218 -    def __call__(self, obj):


219          res = self.evaluate(obj) 
 220          self.record = [(self.name, [p.record for p in self.predicates])] 
 221          return res

 222  
 


223 -    def evaluate(self, obj):


224          raise NotImplementedError

 225  
 
 226  
 


227 -class and_op(predicate_op):


228      name = 'AND' 
 229  
 


230 -    def evaluate(self, obj):


231          return npy.all([p(obj) for p in self.predicates])

 232  
 
 233  
 


234 -class or_op(predicate_op):


235      name = 'OR' 
 236  
 


237 -    def evaluate(self, obj):


238          return npy.any([p(obj) for p in self.predicates])

 239  
 
 240  
 


241 -class not_op(predicate_op):


242      name = 'NOT' 
 243  
 


244 -    def __init__(self, predicate):


245          self.predicate = predicate 
 246          self.record = []

 247  
 


248 -    def precondition(self, objlist):


249          res = self.predicate.precondition(objlist) 
 250          self.record = [self.name, self.predicate.record] 
 251          return res

 252  
 


253 -    def __call__(self, obj):


254          res = self.evaluate(obj) 
 255          self.record = [self.name, self.predicate.record] 
 256          return res

 257  
 


258 -    def evaluate(self, obj):


259          return not self.predicate(obj)

 260  
 
 261  
 


262 -class predicate(object):


263      # override name in subclass if needed
 
 264      name = '' 
 265  
 


266 -    def __init__(self, subject):


267          self.subject = subject 
 268          self.record = []

 269  
 


270 -    def precondition(self, objlist):


271          """Override if needed""" 
 272          return True

 273  
 


274 -    def __call__(self, obj):


275          res = self.evaluate(obj) 
 276          self.record = (self.name, self.subject, res) 
 277          return res

 278  
 


279 -    def evaluate(self, obj):


280          raise NotImplementedError

 281  
 
 282  
 


283 -class null_predicate_class(predicate):


284      name = 'null' 
 285  
 


286 -    def evaluate(self, obj):


287          return True

 288  
 
 289  null_predicate = null_predicate_class(None) 
 290  
 
 291  # ------------------------------------------------------
 
 292  
 
 293  
 


294 -class metric(object):


295      """Abstract metric class for quantitatively comparing scalar or vector
 
 296      quantities.
 
 297      Can include optional explicit Jacobian function.
 
 298  
 
 299      Create concrete sub-classes for specific applications.
 
 300      Store the measured (*1D array only*) value in self.results for use as part
 
 301      of a parameter estimation residual value. Residual norm will be taken
 
 302      by optimizer routines.
 
 303      """ 


304 -    def __init__(self):


305          self.results = None

 306  
 


307 -    def __call__(self, x, y):


308          raise NotImplementedError("Override with a concrete sub-class")

 309  
 


310 -    def Jac(self, x, y):


311          raise NotImplementedError("Override with a concrete sub-class")

 312  
 
 313  
 


314 -class metric_float(metric):


315      """Simple metric between two real-valued floats.
 
 316      """ 


317 -    def __call__(self, x, y):


318          self.results = asarray([x - y]).flatten() 
 319          return norm(self.results)

 320  
 


321 -class metric_float_1D(metric):


322      """Simple metric between two real-valued floats. Version that is suitable for
 
 323      scalar optimizers such as BoundMin.
 
 324      """ 


325 -    def __call__(self, x, y):


326          self.results = abs(asarray([x - y]).flatten()) 
 327          return self.results[0]

 328  
 


329 -class metric_L2(metric):


330      """Measures the standard "distance" between two 1D pointsets or arrays
 
 331      using the L-2 norm.""" 


332 -    def __call__(self, pts1, pts2):


333          self.results = asarray(pts1-pts2).flatten() 
 334          return norm(self.results)

 335  
 


336 -class metric_L2_1D(metric):


337      """Measures the standard "distance" between two 1D pointsets or arrays
 
 338      using the L-2 norm.""" 


339 -    def __call__(self, pts1, pts2):


340          norm_val = norm(asarray(pts1-pts2).flatten()) 
 341          self.results = array([norm_val]) 
 342          return norm_val

 343  
 


344 -class metric_weighted_L2(metric):


345      """Measures the standard "distance" between two 1D pointsets or arrays
 
 346      using the L-2 norm, after weighting by weights attribute
 
 347      (must set weights after creation, e.g. in a feature's _local_init
 
 348      method).""" 


349 -    def __call__(self, pts1, pts2):


350          self.results = array(pts1-pts2).flatten()*self.weights 
 351          return norm(self.results)

 352  
 


353 -class metric_weighted_deadzone_L2(metric):


354      """Measures the standard "distance" between two 1D pointsets or arrays
 
 355      using the L-2 norm, after weighting by weights attribute.
 
 356      Then, sets distance vector entries to zero if they fall
 
 357      below corresponding entries in the deadzone vector/scalar.
 
 358      (Must set weights and deadzone vectors/scalars after creation, e.g.
 
 359      in a feature's _local_init method).
 
 360      """ 


361 -    def __call__(self, pts1, pts2):


362          v = array(pts1-pts2).flatten()*self.weights 
 363          v = (abs(v) > self.deadzone).astype(int) * v 
 364          self.results = v 
 365          return norm(v)

 366  
 
 367  
 


368 -def n_sigdigs_str(x, n):


369      """Return a string representation of float x with n significant digits,
 
 370      where n > 0 is an integer.
 
 371      """ 
 372      format = "%." + str(int(n)) + "g" 
 373      s = '%s' % float(format % x) 
 374      if '.' in s: 
 375          # handle trailing ".0" when not one of the sig. digits
 
 376          pt_idx = s.index('.') 
 377          if s[0] == '-': 
 378              # pt_idx is one too large
 
 379              if pt_idx-1 >= n: 
 380                  return s[:pt_idx] 
 381          else: 
 382              if pt_idx >= n: 
 383                  return s[:pt_idx] 
 384      return s

 385  
 
 386  
 


387 -class args(object):


388      """Mapping object class for building arguments for class initialization
 
 389      calls. Treat as a dictionary.
 
 390      """ 
 391  
 


392 -    def __init__(self, **kw):


393          self.__dict__ = kw

 394  
 


395 -    def _infostr(self, verbose=1, attributeTitle='args'):


396          # removed offset=0 from arg list
 
 397          if len(self.__dict__) > 0: 
 398              res = "%s ("%attributeTitle 
 399              for k, v in self.__dict__.iteritems(): 
 400                  try: 
 401                      istr = v._infostr(verbose-1) #, offset+2) 
 402                  except AttributeError: 
 403                      istr = str(v) 
 404                  res += "\n%s%s = %s,"%(" ",k,istr) 
 405                  # was " "*offset
 
 406              # skip last comma
 
 407              res = res[:-1] + "\n)" 
 408              return res 
 409          else: 
 410              return "No %s defined"%attributeTitle

 411  
 


412 -    def __repr__(self):


413          return self._infostr()

 414  
 


415 -    def info(self):


416          print self._infostr()

 417  
 
 418      __str__ = __repr__ 
 419  
 


420 -    def values(self):


421          return self.__dict__.values()

 422  
 


423 -    def keys(self):


424          return self.__dict__.keys()

 425  
 


426 -    def items(self):


427          return self.__dict__.items()

 428  
 


429 -    def itervalues(self):


430          return self.__dict__.itervalues()

 431  
 


432 -    def iterkeys(self):


433          return self.__dict__.iterkeys()

 434  
 


435 -    def iteritems(self):


436          return self.__dict__.iteritems()

 437  
 


438 -    def __getitem__(self, k):


439          return self.__dict__[k]

 440  
 


441 -    def __setitem__(self, k, v):


442          self.__dict__.__setitem__(k, v)

 443  
 


444 -    def update(self, d):


445          self.__dict__.update(d)

 446  
 


447 -    def copy(self):


448          return copy(self)

 449  
 


450 -    def clear(self):


451          self.__dict__.clear()

 452  
 


453 -    def get(self, k, d=None):


454          return self.__dict__.get(k, d)

 455  
 


456 -    def has_key(self, k):


457          return self.__dict__.has_key(k)

 458  
 


459 -    def pop(self, k, d=None):


460          return self.__dict__.pop(k, d)

 461  
 


462 -    def popitem(self):


463          raise NotImplementedError

 464  
 


465 -    def __contains__(self, v):


466          return self.__dict__.__contains__(v)

 467  
 


468 -    def fromkeys(self, S, v=None):


469          raise NotImplementedError

 470  
 


471 -    def setdefault(self, d):


472          raise NotImplementedError

 473  
 


474 -    def __delitem__(self, k):


475          del self.__dict__[k]

 476  
 


477 -    def __cmp__(self, other):


478          return self.__dict__ == other

 479  
 


480 -    def __eq__(self, other):


481          return self.__dict__ == other

 482  
 


483 -    def __ne__(self, other):


484          return self.__dict__ != other

 485  
 


486 -    def __gt__(self, other):


487          return self.__dict__ > other

 488  
 


489 -    def __ge__(self, other):


490          return self.__dict__ >= other

 491  
 


492 -    def __lt__(self, other):


493          return self.__dict__ < other

 494  
 


495 -    def __le__(self, other):


496          return self.__dict__ <= other

 497  
 


498 -    def __len__(self):


499          return len(self.__dict__)

 500  
 


501 -    def __iter__(self):


502          return iter(self.__dict__)

 503  
 


504 -    def __add__(self, other):


505          d = self.__dict__.copy() 
 506          d.update(other.__dict__) 
 507          return args(**d)

 508  
 
 509  
 


510 -def get_opt(argopt, attr, default=None):


511      """Get option from args object otherwise default to the given value. Can
 
 512      also specify that an AttributeError is raised by passing default=Exception.
 
 513      """ 
 514      try: 
 515          return getattr(argopt, attr) 
 516      except AttributeError: 
 517          if default is Exception: 
 518              raise PyDSTool_AttributeError("Missing option: "+attr) 
 519          else: 
 520              return default

 521  
 
 522  
 


523 -class Diagnostics(object):


524      """General purpose diagnostics manager.""" 
 525  
 


526 -    def __init__(self, errmessages=None, errorfields=None, warnmessages=None,
 
 527                   warnfields=None, errorcodes=None, warncodes=None,
 
 528                   outputinfo=None, propagate_dict=None):


529          if warnfields is None: 
 530              warnfields = {} 
 531          if warnmessages is None: 
 532              warnmessages = {} 
 533          if warncodes is None: 
 534              warncodes = {} 
 535          if errorfields is None: 
 536              errorfields = {} 
 537          if errmessages is None: 
 538              errmessages = {} 
 539          if errorcodes is None: 
 540              errorcodes = {} 
 541          self._warnfields = warnfields 
 542          self._warnmessages = warnmessages 
 543          self._warncodes = warncodes 
 544          self._errorfields = errorfields 
 545          self._errmessages = errmessages 
 546          self._errorcodes = errorcodes 
 547          self.errors = [] 
 548          self.warnings = [] 
 549          # traceback may store information about variable state, pars, etc.
 
 550          # at time of an error that breaks the solver
 
 551          self.traceback = {} 
 552          self.outputStatsInfo = outputinfo 
 553          self.outputStats = {} 
 554          if propagate_dict is None: 
 555              # use dict so that un-initialized inputs attribute
 
 556              # of generator etc. can be passed-by-reference
 
 557              self.propagate_dict = {} 
 558          else: 
 559              self.propagate_dict = propagate_dict

 560  
 


561 -    def update(self, d):


562          """Update warnings and errors from another diagnostics object""" 
 563          self.traceback.update(d.traceback) 
 564          self.warnings.extend(d.warnings) 
 565          self.errors.extend(d.errors) 
 566          self.outputStats.update(d.outputStats) 
 567          self._warnfields.update(d._warnfields) 
 568          self._warnmessages.update(d._warnmessages) 
 569          self._warncodes.update(d._warncodes) 
 570          self._errorfields.update(d._errorfields) 
 571          self._errmessages.update(d._errmessages) 
 572          self._errorcodes.update(d._errorcodes)

 573  
 


574 -    def clearAll(self):


575          self.clearErrors() 
 576          self.clearWarnings() 
 577          self.outputStats = {} 
 578          self.traceback = {}

 579  
 


580 -    def clearWarnings(self):


581          self.warnings = [] 
 582          for obj in self.propagate_dict.values(): 
 583              try: 
 584                  obj.diagnostics.clearWarnings() 
 585              except AttributeError: 
 586                  if hasattr(obj, 'name'): 
 587                      name = obj.name 
 588                  else: 
 589                      name = str(obj) 
 590                  raise TypeError("Object %s has no diagnostics manager"%name)

 591  
 


592 -    def showWarnings(self):


593          if len(self.warnings)>0: 
 594              print self.getWarnings()

 595  
 


596 -    def getWarnings(self):


597          if len(self.warnings)>0: 
 598              output = 'Warnings: ' 
 599              for (w, d) in self.warnings: 
 600                  dstr = '' 
 601                  for i in range(len(d)): 
 602                      dentry = d[i] 
 603                      dstr += self._warnfields[w][i] + ' = ' + str(dentry) + ", " 
 604                  dstr = dstr[:-2]  # drop trailing comma 
 605                  output += ' Warning code %s:  %s\n Info:  %s ' %(w, \
 
 606                                                  self._warnmessages[w], dstr) 
 607          else: 
 608              output = '' 
 609          return output

 610  
 


611 -    def findWarnings(self, code):


612          """Return time-ordered list of warnings of kind specified using a
 
 613          single Generator warning code""" 
 614          res = [] 
 615          for wcode, (t, name) in self.warnings: 
 616              if wcode == code: 
 617                  res.append((t, name)) 
 618          res.sort()  # increasing order 
 619          return res

 620  
 


621 -    def hasWarnings(self):


622          return self.warnings != []

 623  
 


624 -    def hasErrors(self):


625          return self.errors != []

 626  
 


627 -    def clearErrors(self):


628          self.errors = [] 
 629          for obj in self.propagate_dict.values(): 
 630              try: 
 631                  obj.diagnostics.clearErrors() 
 632              except AttributeError: 
 633                  if hasattr(obj, 'name'): 
 634                      name = obj.name 
 635                  else: 
 636                      name = str(obj) 
 637                  raise TypeError("Object %s has no diagnostics manager"%name)

 638  
 


639 -    def showErrors(self):


640          if len(self.errors)>0: 
 641              print self.getErrors()

 642  
 


643 -    def getErrors(self):


644          if len(self.errors)>0: 
 645              output = 'Errors: ' 
 646              for (e, d) in self.errors: 
 647                  dstr = '' 
 648                  for i in range(len(d)): 
 649                      dentry = d[i] 
 650                      dstr += self._errorfields[e][i] + ' = ' + str(dentry) + ", " 
 651                  dstr = dstr[:-2]  # drop trailing comma 
 652                  output += ' Error code %s:  %s\n Info:\n  %s ' %(e, \
 
 653                                                      self._errmessages[e], dstr) 
 654          else: 
 655              output = '' 
 656          return output

 657  
 


658 -    def info(self, verboselevel=0):


659          self.showErrors() 
 660          self.showWarnings()

 661  
 
 662  
 
 663  ## ------------------------------------------------------------------
 
 664  
 
 665  ## Internally used functions
 
 666  
 


667 -def compareNumTypes(t1, t2):


668      try: 
 669          return sometrue([_num_type2name[t1] == _num_type2name[t] for t in t2]) 
 670      except TypeError: 
 671          # t2 not iterable, assume singleton
 
 672          try: 
 673              return _num_type2name[t1] == _num_type2name[t2] 
 674          except KeyError: 
 675              return False 
 676      except KeyError: 
 677          return False

 678  
 
 679  
 


680 -def filteredDict(d, keys, neg=False):


681      """returns filtered dictionary containing specified keys,
 
 682      or *not* containing the specified keys if option neg=True.""" 
 683      out_d = {} 
 684      if neg: 
 685          out_keys = remain(d.keys(), keys) 
 686      else: 
 687          out_keys = keys 
 688      for k in out_keys: 
 689          try: 
 690              out_d[k] = d[k] 
 691          except KeyError: 
 692              pass 
 693      return out_d

 694  
 
 695  
 


696 -def concatStrDict(d, order=[]):


697      """Concatenates all entries of a dictionary (assumed to be
 
 698      lists of strings), in optionally specified order.""" 
 699      retstr = '' 
 700      if d != {}: 
 701          if order == []: 
 702              order = d.keys() 
 703          for key in order: 
 704              itemlist = d[key] 
 705              for strlist in itemlist: 
 706                  retstr += ''.join(strlist) 
 707      return retstr

 708  
 
 709  
 


710 -def copyVarDict(vardict, only_cts=False):


711      """Copy dictionary of Variable objects.
 
 712      Use the only_cts Boolean optional argument (default False) to select only
 
 713      continuous-valued variables (mainly for internal use).
 
 714      """ 
 715      if only_cts: 
 716          out_vars = [] 
 717          out_varnames = [] 
 718          sorted_varnames = sortedDictKeys(vardict) 
 719          for varname in sorted_varnames: 
 720              var = vardict[varname] 
 721              if var.is_continuous_valued(): 
 722                  out_varnames.append(varname) 
 723                  out_vars.append(var) 
 724          return dict(zip(out_varnames, out_vars)) 
 725      else: 
 726          return dict(zip(sortedDictKeys(vardict), [copy(v) for v in \
 
 727                                                sortedDictValues(vardict)]))

 728  
 
 729  
 


730 -def insertInOrder(sourcelist, inslist, return_ixs=False, abseps=0):


731      """Insert elements of inslist into sourcelist, sorting these
 
 732        lists in case they are not already in increasing order. The new
 
 733        list is returned.
 
 734  
 
 735      The function will not create duplicate entries in the list, and will
 
 736        change neither the first or last entries of the list.
 
 737  
 
 738      If sourcelist is an array, an array is returned.
 
 739      If optional return_ixs=True, the indices of the inserted elements
 
 740        in the returned list is returned as an additional return argument.
 
 741      If abseps=0 (default) the comparison of elements is done exactly. For
 
 742        abseps > 0 elements are compared up to an absolute difference no
 
 743        greater than abseps for determining "equality".
 
 744      """ 
 745      try: 
 746          sorted_inslist = inslist.tolist() 
 747      except AttributeError: 
 748          sorted_inslist = copy(inslist) 
 749      sorted_inslist.sort() 
 750      try: 
 751          sorted_sourcelist = sourcelist.tolist() 
 752          was_array = True 
 753      except AttributeError: 
 754          sorted_sourcelist = copy(sourcelist) 
 755          was_array = False 
 756      sorted_sourcelist.sort() 
 757      close_ixs = [] 
 758      tix = 0 
 759      # optimize by having separate versions of loop
 
 760      if return_ixs: 
 761          ins_ixs = [] 
 762          for t in sorted_inslist: 
 763              tcond = less_equal(sorted_sourcelist[tix:], t).tolist() 
 764              try: 
 765                  tix = tcond.index(0) + tix  # lowest index for elt > t 
 766              except ValueError: 
 767                  # no 0 value in tcond, so t might be equal to the final value
 
 768                  if abs(sorted_sourcelist[-1] - t) < abseps: 
 769                      close_ixs.append((t,len(sorted_sourcelist)-1)) 
 770              else: 
 771                  if abs(sorted_sourcelist[tix-1] - t) >= abseps: 
 772                      if tix >= 0: 
 773                          sorted_sourcelist.insert(tix, t) 
 774                          ins_ixs.append(tix) 
 775                  else: 
 776                      close_ixs.append((t,tix-1)) 
 777          if was_array: 
 778              if abseps > 0: 
 779                  return array(sorted_sourcelist), ins_ixs, dict(close_ixs) 
 780              else: 
 781                  return array(sorted_sourcelist), ins_ixs 
 782          else: 
 783              if abseps > 0: 
 784                  return sorted_sourcelist, ins_ixs, dict(close_ixs) 
 785              else: 
 786                  return sorted_sourcelist, ins_ixs 
 787      else: 
 788          for t in sorted_inslist: 
 789              tcond = less_equal(sorted_sourcelist[tix:], t).tolist() 
 790              try: 
 791                  tix = tcond.index(0) + tix  # lowest index for elt > t 
 792              except ValueError: 
 793                  # no 0 value in tcond, so t might be equal to the final value
 
 794                  if abs(sorted_sourcelist[-1] - t) < abseps: 
 795                      close_ixs.append((t,len(sorted_sourcelist)-1)) 
 796              else: 
 797                  if abs(sorted_sourcelist[tix-1] - t) >= abseps: 
 798                      if tix >= 0: 
 799                          sorted_sourcelist.insert(tix, t) 
 800                  else: 
 801                      close_ixs.append((t,tix-1)) 
 802          if was_array: 
 803              if abseps > 0: 
 804                  return array(sorted_sourcelist), dict(close_ixs) 
 805              else: 
 806                  return array(sorted_sourcelist) 
 807          else: 
 808              if abseps > 0: 
 809                  return sorted_sourcelist, dict(close_ixs) 
 810              else: 
 811                  return sorted_sourcelist

 812  
 
 813  
 


814 -def arraymax(a1,a2,t=float64):


815      """Element-wise comparison of maximum values for two arrays.""" 
 816      o=[] 
 817      try: 
 818          for x, y in zip(a1,a2): 
 819              o.append(max(x,y)) 
 820      except TypeError: 
 821          print "Problem with type of arguments in arraymax:" 
 822          print "Received a1 =", a1 
 823          print "         a2 =", a2 
 824          raise 
 825      return array(o,t)

 826  
 
 827  
 


828 -def simplifyMatrixRepr(m):


829      """Convert matrix object to a compact array
 
 830      representation or numeric value.""" 
 831      ma=array(m) 
 832      l = len(shape(ma)) 
 833      if l == 0: 
 834          return m 
 835      elif l>0 and shape(ma)[0] == 1: 
 836          return simplifyMatrixRepr(ma[0]) 
 837      elif l>1 and shape(ma)[1] == 1: 
 838          return simplifyMatrixRepr(ma[:,0]) 
 839      else: 
 840          return ma

 841  
 
 842  
 


843 -def makeMultilinearRegrFn(arg, xs, ys):


844      """Convert two lists or arrays mapping x intervals to y intervals
 
 845      into a string function definition of a multilinear regression
 
 846      scalar function that these define. A.k.a. makes a "piecewise
 
 847      linear" scalar function from the input data. The two input data
 
 848      sequences can each be either all numeric values or all
 
 849      strings/symbolic objects, but not a mixture. """ 
 850      assert len(xs)==len(ys), \
 
 851             "You must give x and y lists that are the same length" 
 852      assert not isinstance(arg, _num_types), \
 
 853             "arg must be a string or symbolic object" 
 854      argname = str(arg) 
 855  
 
 856      def sub_str(a,b): 
 857          return '(' + str(a) + '-' + str(b) + ')'

 858      def sub_val(a,b): 
 859          return repr(a-b) 
 860  
 
 861      def interp(n): 
 862          return rep_y(ys[n-1]) +'+(' + argname + '-(' + rep_x(xs[n-1]) \
 
 863            + '))*' + sub_y(ys[n],ys[n-1]) +'/'+ sub_x(xs[n],xs[n-1]) 
 864  
 
 865      x_test = [isinstance(xs[n], _num_types) for n in range(len(xs))] 
 866      if all(x_test): 
 867          rep_x = lambda x: repr(x) 
 868          sub_x = sub_val 
 869      elif any(x_test): 
 870          raise TypeError("xlist must contain either all string/symbolic types "
 
 871                          "or all numeric values") 
 872      else: 
 873          rep_x = lambda x: str(x) 
 874          sub_x = sub_str 
 875      y_test = [isinstance(ys[n], _num_types) for n in range(len(ys))] 
 876      if all(y_test): 
 877          rep_y = lambda y: repr(y) 
 878          sub_y = sub_val 
 879      elif any(y_test): 
 880          raise TypeError("ylist must contain either all string/symbolic types "
 
 881                          "or all numeric values") 
 882      else: 
 883          rep_y = lambda y: str(y) 
 884          sub_y = sub_str 
 885      mLR = '+'.join(['heav(%s-%s)*(1-heav(%s-%s))*(%s)'%(argname, \
 
 886                       rep_x(xs[n-1]),argname,rep_x(xs[n]),interp(n)) \
 
 887                     for n in range(1,len(xs))]) 
 888      return ([argname], mLR) 
 889  
 
 890  
 


891 -def _scalar_diff(func, x0, dx):


892      """Numerical differentiation of scalar function by central differences.
 
 893      Returns tuple containing derivative evaluated at x0 and error estimate,
 
 894      using Ridders' method and Neville's algorithm.
 
 895      """ 
 896      max_order = 10 
 897      BIG = 1e50 
 898      CON = 1.4 
 899      CON2 = CON*CON 
 900      SAFE = 2 
 901      a=zeros((max_order,max_order),'f') 
 902      a[0,0] = (func(x0+dx)-func(x0-dx))/(2.*dx) 
 903      err=BIG 
 904      ans = NaN 
 905      for i in range(1,max_order): 
 906          dx /= CON 
 907          # try a smaller stepsize
 
 908          a[0,i] = (func(x0+dx)-func(x0-dx))/(2.*dx) 
 909          fac = CON2 
 910          for j in range(1,i): 
 911              # compute extrapolations of various orders, using Neville's
 
 912              # algorithm
 
 913              a[j,i] = (a[j-1,i]*fac-a[j-1,i-1])/(fac-1.) 
 914              fac *= CON2 
 915              errt = max([abs(a[j,i]-a[j-1,i]),abs(a[j,i]-a[j-1][i-1])]) 
 916              # error strategy:
 
 917              # compare each new extrapolation to one order lower, both at the
 
 918              # present stepsize and the previous one
 
 919              if errt <= err: 
 920                  err = errt 
 921                  ans  = a[j,i] 
 922          if abs(a[i,i] - a[i-1,i-1]) >= SAFE*err: 
 923              # if higher order is worse by a significant factor SAFE, then
 
 924              # quit early
 
 925              break 
 926      return (ans, err, dx)

 927  
 
 928  
 


929 -def diff(func, x0, vars=None, axes=None, eps=None, output=None):


930      """Numerical 1st derivative of R^N -> R^M scalar or array function
 
 931      about x0 by central finite differences. Uses Ridders' method of
 
 932      polynomial extrapolation, based on an implementation in the book
 
 933      "Numerical Recipes". Returns a matrix.
 
 934  
 
 935      vars argument specifies which elements of x0 are to be treated as
 
 936        variables for the purposes of taking the Jacobian.
 
 937      If axes argument is unused or set to be all axes, the Jacobian of the
 
 938        function evaluated at x0 with respect to the variables is returned,
 
 939        otherwise a sub-matrix of it is returned.
 
 940      eps is assumed to be the scale in x for which the function varies by O(1).
 
 941        If eps is not given an appropriate step size is chosen.
 
 942      output = True returns an optional dictionary which will be updated
 
 943        with error and derivative information.
 
 944      """ 
 945  
 
 946      if isinstance(x0, ndarray): 
 947          x0type = 'array' 
 948          if not compareNumTypes(x0.dtype.type, _all_float): 
 949              raise TypeError("Only real-valued arrays valid") 
 950      elif isinstance(x0, _real_types): 
 951          x0type = 'num' 
 952          x0 = float(x0) 
 953      else: 
 954          # Point type
 
 955          try: 
 956              assert compareNumTypes(x0.coordtype, _all_float) 
 957              x0.coordnames 
 958              x0.dimension 
 959          except (AssertionError, AttributeError): 
 960              raise TypeError("Function and x0 must use real-valued scalar,"
 
 961                              "array, or Point types only") 
 962          x0type = 'point' 
 963      output_info = {} 
 964      if vars is None: 
 965          if x0type == 'array': 
 966              dim = len(x0) 
 967              vars = range(dim) 
 968          elif x0type == 'num': 
 969              dim = 1 
 970              vars = [0] 
 971          else: 
 972              # Point type
 
 973              dim = x0.dimension 
 974              vars = x0.coordnames 
 975      else: 
 976          assert isinstance(vars, _seq_types), \
 
 977                 "vars argument must be a sequence type" 
 978          if x0type in ['array', 'num']: 
 979              assert all(vars>=0), \
 
 980                      "vars argument must hold non-negative integers" 
 981          else: 
 982              assert all([isinstance(vars[i], str) \
 
 983                   for i in range(len(vars))]), "vars argument must hold strings" 
 984          dim = len(vars) 
 985      fx0 = func(x0) 
 986      sfx0 = shape(fx0) 
 987      try: 
 988          # ensure fx0 is a vector or at least only a D x 1 matrix
 
 989          assert sfx0[1] == 1 
 990      except IndexError: 
 991          # if shape is of form (D,) then that's fine
 
 992          if len(sfx0) > 0: 
 993              if sfx0[0] == 0: 
 994                  raise TypeError("Invalid function return type") 
 995      except AssertionError: 
 996          print "fx0 shape is", sfx0 
 997          print fx0 
 998          raise ValueError("Function should return an N-vector or N x 1 matrix,"
 
 999                   " but it returned a matrix with shape %s" % str(sfx0)) 
1000      if isinstance(fx0, _float_types): 
1001          dimf = 1 
1002      elif isinstance(fx0, ndarray): 
1003          if not compareNumTypes(fx0.dtype.type, _all_float): 
1004              raise TypeError("Only real-valued functions valid") 
1005          try: 
1006              dimf = sfx0[0] 
1007          except IndexError: 
1008              dimf = 1 
1009      else: 
1010          try: 
1011              assert compareNumTypes(fx0.coordtype, _all_float) 
1012          except (AssertionError, AttributeError): 
1013              raise TypeError("Only real-valued functions valid") 
1014          dimf = sfx0[0] 
1015      if axes is None: 
1016          if x0type in ['array', 'num']: 
1017              try: 
1018                  axes = range(sfx0[0]) 
1019              except IndexError: 
1020                  # then singleton (scalar) was returned
 
1021                  axes = [0] 
1022          else: 
1023              axes = fx0.coordnames 
1024      else: 
1025          assert isinstance(axes, _seq_types), \
 
1026                 "axes argument must be a sequence type" 
1027          if x0type in ['array', 'num']: 
1028              assert all(axes>=0), \
 
1029                     "axes argument must hold non-negative integers" 
1030          else: 
1031              assert all([isinstance(axes[i], str) \
 
1032                   for i in range(len(axes))]), "axes argument must hold strings" 
1033      if eps is None: 
1034          eps = sqrt(Macheps) 
1035      else: 
1036          assert all(eps > 0), "eps scaling array must be strictly positive" 
1037          if isinstance(eps, _num_types): 
1038              eps = ones(dim)*eps 
1039          else: 
1040              assert len(eps) == len(vars), \
 
1041                     "eps scaling array has length mismatch with vars" 
1042  
 
1043      if x0type == 'array': 
1044          dx = eps*(abs(x0[vars]) + array(x0[vars]==zeros(dim),'float64')) 
1045      elif x0type == 'num': 
1046          dx = eps*(abs(x0) + int(x0==0)) 
1047      else: 
1048          # Point
 
1049          x0a = x0[vars].toarray() 
1050          dx = dict(zip(vars,
 
1051                        eps*(abs(x0a) + array(x0a==zeros(dim),'float64')))) 
1052      try: 
1053          dim_mat = len(axes) 
1054      except TypeError: 
1055          raise TypeError("axes argument must be a sequence type") 
1056      assert dim_mat <= dimf, "Number of axes greater than dimension of function" 
1057      df = zeros([dim_mat,dim], 'float64') 
1058      if x0type == 'array': 
1059          output_info['error'] = zeros([dim_mat,dim], 'float64') 
1060          output_info['dx'] = zeros([dim_mat,dim], 'float64') 
1061          def update(xa, i, x): 
1062              xa[i] = x 
1063              return xa

1064          for i, vix in enumerate(vars): 
1065              try: 
1066                  # for numpy arrays (otherwise copy returns a regular 'array'!)
 
1067                  x0_d = x0.copy() 
1068              except AttributeError: 
1069                  x0_d = copy(x0) 
1070              if dimf > 1: 
1071                  for j in range(dim_mat): 
1072                      f_d = lambda x: func(update(x0_d, vix, x))[axes[j]] 
1073                      df_d, errval, dx_d = _scalar_diff(f_d, x0_d[vix], dx[i]) 
1074                      df[j,i] = df_d 
1075                      output_info['error'][j,i] = errval 
1076                      output_info['dx'][j,i] = dx_d 
1077              else: 
1078                  for j in range(dim_mat): 
1079                      f_d = lambda x: func(update(x0_d, vix, x)) 
1080                      df_d, errval, dx_d = _scalar_diff(f_d, x0_d[vix], dx[i]) 
1081                      df[j,i] = df_d 
1082                      output_info['error'][j,i] = errval 
1083                      output_info['dx'][j,i] = dx_d 
1084          df = mat(df) 
1085          output_info['df'] = df 
1086          if output is not None: 
1087              try: 
1088                  output.update(output_info) 
1089              except: 
1090                  raise TypeError("Invalid type for 'output' argument") 
1091          return df 
1092      elif x0type == 'num': 
1093          df, errval, dx_d = _scalar_diff(func, x0, dx) 
1094          output_info['df'] = df 
1095          output_info['error'] = errval 
1096          output_info['dx'] = dx_d 
1097          if output is not None: 
1098              try: 
1099                  output.update(output_info) 
1100              except: 
1101                  raise TypeError("Invalid type for 'output' argument") 
1102          return df 
1103      else: 
1104          # Point type
 
1105          output_info['error'] = zeros([dim_mat,dim], 'float64') 
1106          output_info['dx'] = zeros([dim_mat,dim], 'float64') 
1107          def update(xa, vn, x): 
1108              xa[vn] = x 
1109              return xa 
1110          for i in range(dim): 
1111              vname = vars[i] 
1112              x0_d = copy(x0) 
1113              for j in range(dim_mat): 
1114                  f_d = lambda x: func(update(x0_d, vname, x))[axes[j]] 
1115                  df_d, errval, dx_d = _scalar_diff(f_d, x0_d[vname], dx[vname]) 
1116                  df[j,i] = df_d 
1117                  output_info['error'][j,i] = errval 
1118                  output_info['dx'][j,i] = dx_d 
1119          df = mat(df) 
1120          output_info['df'] = df 
1121          if output is not None: 
1122              try: 
1123                  output.update(output_info) 
1124              except: 
1125                  raise TypeError("Invalid type for 'output' argument") 
1126          return df 
1127  
 
1128  
 


1129 -def diff2(func, x0, vars=None, axes=None, dir=1, eps=None):


1130      """Numerical 1st derivative of R^N -> R^M scalar or array function
 
1131      about x0 by forward or backward finite differences. Returns a matrix.
 
1132  
 
1133      dir=1 uses finite forward difference.
 
1134      dir=-1 uses finite backward difference.
 
1135      List-valued eps rescales finite differencing in each axis separately.
 
1136      vars argument specifies which elements of x0 are to be treated as
 
1137        variables for the purposes of taking the Jacobian.
 
1138      If axes argument is unused or set to be all axes, the Jacobian of the
 
1139        function evaluated at x0 with respect to the variables is returned,
 
1140        otherwise a sub-matrix of it is returned.
 
1141      eps is assumed to be the scale in x for which the function varies by O(1).
 
1142        If eps is not given an appropriate step size is chosen
 
1143        (proportional to sqrt(machine precision)).
 
1144      """ 
1145  
 
1146      if isinstance(x0, ndarray): 
1147          x0type = 'array' 
1148          if not compareNumTypes(x0.dtype.type, _all_float): 
1149              try: 
1150                  x0 = x0.astype(float) 
1151              except: 
1152                  print "Found type:", x0.dtype.type 
1153                  raise TypeError("Only real-valued arrays valid") 
1154      elif isinstance(x0, _real_types): 
1155          x0type = 'num' 
1156          x0 = float(x0) 
1157      else: 
1158          # Point type
 
1159          try: 
1160              assert compareNumTypes(x0.coordtype, _all_float) 
1161              x0.coordnames 
1162              x0.dimension 
1163          except (AssertionError, AttributeError): 
1164              raise TypeError("Function and x0 must use real-valued scalar,"
 
1165                              "array, or Point types only") 
1166          x0type = 'point' 
1167      if vars is None: 
1168          if x0type == 'array': 
1169              dim = len(x0) 
1170              vars = range(dim) 
1171          elif x0type == 'num': 
1172              dim = 1 
1173              vars = [0] 
1174          else: 
1175              # Point type
 
1176              dim = x0.dimension 
1177              vars = x0.coordnames 
1178      else: 
1179          assert isinstance(vars, _seq_types), \
 
1180                 "vars argument must be a sequence type" 
1181          if x0type in ['array', 'num']: 
1182              assert all(vars>=0), \
 
1183                      "vars argument must hold non-negative integers" 
1184          else: 
1185              assert all([isinstance(vars[i], str) \
 
1186                   for i in range(len(vars))]), "vars argument must hold strings" 
1187          dim = len(vars) 
1188      fx0 = func(x0) 
1189      sfx0 = shape(fx0) 
1190      if isinstance(fx0, _float_types): 
1191          dimf = 1 
1192      elif isinstance(fx0, ndarray): 
1193          if not compareNumTypes(fx0.dtype.type, _all_float): 
1194              raise TypeError("Only real-valued functions valid") 
1195          try: 
1196              dimf = sfx0[0] 
1197          except IndexError: 
1198              dimf = 1 
1199          try: 
1200              # ensure fx0 is a vector or at least only a D x 1 matrix
 
1201              assert sfx0[1] == 1 
1202          except IndexError: 
1203              # if shape is of form (D,) then that's fine
 
1204              if len(sfx0) > 0: 
1205                  if sfx0[0] == 0: 
1206                      raise TypeError("Invalid function return type") 
1207              else: 
1208                  raise TypeError("Invalid function return type") 
1209          except AssertionError: 
1210              print "fx0 shape is", sfx0 
1211              print fx0 
1212              raise ValueError("Function should return an N-vector or N x 1 matrix,"
 
1213                       " but it returned a matrix with shape %s" % str(sfx0)) 
1214      else: 
1215          try: 
1216              assert compareNumTypes(fx0.coordtype, _all_float) 
1217          except (AssertionError, AttributeError): 
1218              raise TypeError("Only real-valued functions valid") 
1219          dimf = sfx0[0] 
1220      if axes is None: 
1221          if x0type in ['array', 'num']: 
1222              try: 
1223                  axes = range(sfx0[0]) 
1224              except IndexError: 
1225                  # then singleton (scalar) was returned
 
1226                  axes = [0] 
1227          else: 
1228              axes = fx0.coordnames 
1229      else: 
1230          assert isinstance(axes, _seq_types), \
 
1231                 "axes argument must be a sequence type" 
1232          if x0type in ['array', 'num']: 
1233              assert all(axes>=0), \
 
1234                     "axes argument must hold non-negative integers" 
1235          else: 
1236              assert all([isinstance(axes[i], str) \
 
1237                   for i in range(len(axes))]), "axes argument must hold strings" 
1238      if eps is None: 
1239          eps = sqrt(Macheps) 
1240      else: 
1241          assert all(eps > 0), "eps scaling array must be strictly positive" 
1242          if isinstance(eps, float): 
1243              if x0type in ['array', 'num']: 
1244                  eps = ones(dim)*eps 
1245          else: 
1246              assert len(eps) == len(vars), \
 
1247                     "eps scaling array has length mismatch with vars" 
1248              eps = asarray(eps, 'float64') 
1249      # ensure dx is not 0, and make into an appropriate length vector
 
1250      if x0type == 'array': 
1251          dx = eps*(abs(x0[vars]) + array(x0[vars]==zeros(dim),'float64')) 
1252      elif x0type == 'num': 
1253          dx = eps*(abs(x0)+int(x0==0)) 
1254      else: 
1255          # Point
 
1256          x0a = x0[vars].toarray() 
1257          dx = dict(zip(vars,
 
1258                        eps*(abs(x0a) + array(x0a==zeros(dim),'float64')))) 
1259  
 
1260      assert dir==1 or dir==-1, "Direction code must be -1 or 1" 
1261      try: 
1262          dim_mat = len(axes) 
1263      except TypeError: 
1264          raise TypeError("axes argument must be a sequence type") 
1265      assert dim_mat <= dimf, "Number of axes greater than dimension of function" 
1266      df = zeros([dim_mat,dim], 'float64') 
1267      if x0type == 'array': 
1268          for i in range(dim): 
1269              vix = vars[i] 
1270              try: 
1271                  # for numpy arrays (otherwise copy returns a regular 'array'!)
 
1272                  x0_d = x0.copy() 
1273              except AttributeError: 
1274                  x0_d = copy(x0) 
1275              x0_d[vix] += dir * dx[i] 
1276              fx0_d = func(x0_d) 
1277              if dim_mat > 1: 
1278                  fx0_d_v = array([fx0_d[n] for n in axes]) 
1279                  fx0_v = array([fx0[n] for n in axes]) 
1280              else: 
1281                  if dimf > 1: 
1282                      fx0_d_v = fx0_d[axes[0]] 
1283                      fx0_v = fx0[axes[0]] 
1284                  else: 
1285                      fx0_d_v = fx0_d 
1286                      fx0_v = fx0 
1287              df[:,i] = dir*(fx0_d_v - fx0_v)/dx[i] 
1288          return mat(df) 
1289      elif x0type == 'num': 
1290          x0_d = x0 + dir*dx 
1291          fx0_d = func(x0_d) 
1292          df = dir*(fx0_d - fx0)/dx 
1293          return df 
1294      else: 
1295          # Point type
 
1296          for i in range(dim): 
1297              vname = vars[i] 
1298              x0_d = copy(x0) 
1299              x0_d[vname] = x0_d(vname) + dir * dx[vname] 
1300              fx0_d = func(x0_d)[axes] 
1301              fx0_v = fx0[axes] 
1302              df[:,i] = dir*(fx0_d - fx0_v).toarray()/dx[vname] 
1303          return mat(df)

1304  
 
1305  
 


1306 -def ensurefloat(v):


1307      try: 
1308          # singleton Point will return scalar here
 
1309          v = v.toarray() 
1310      except AttributeError: 
1311          pass 
1312      try: 
1313          # numeric literal as Quantity will return scalar here
 
1314          v = v.tonumeric() 
1315      except AttributeError: 
1316          pass 
1317      return float(v)

1318  
 
1319  _verify_type_names = {_all_int: 'an integer',
 
1320                        _all_float: 'a float',
 
1321                        _real_types: 'a real number',
 
1322                        _all_complex: 'a complex number'} 
1323  
 
1324  # Only support lists because the primary use of these functions is for
 
1325  # checking input to SWIG-interfaced data structures passed down to C
 
1326  # and Fortran, which must be basic types only.
 
1327  
 


1328 -def verify_values(name, value, values, list_ok=False, list_len=None):


1329      """Use list_ok if a list of values of these types is acceptable.
 
1330      list_len can be used to specify that a list must be of a certain length,
 
1331      either a fixed integer or a variable integer value given as the first
 
1332      value of a pair, the second being the name of the variable (for use in
 
1333      error messages)
 
1334      """ 
1335      if list_ok: 
1336          if isinstance(value, list): 
1337              if list_len is not None: 
1338                  if isinstance(list_len, _all_int): 
1339                      ok = (len(value) == list_len) 
1340                      len_name = '%d' % list_len 
1341                  else: 
1342                      ok = (len(value) == list_len[0]) 
1343                      len_name = list_len[1] 
1344                  if not ok: 
1345                      raise ValueError("list "+name+" length must equal "+len_name) 
1346              for v in value: 
1347                  try: 
1348                      # make sure v is not a list too
 
1349                      verify_values(name, v, values) 
1350                  except ValueError: 
1351                      raise ValueError(name+" must be in " + str(values) + \
 
1352                                       " or a list of these") 
1353                  except TypeError: 
1354                      raise TypeError(name+" must be in " + str(values) + \
 
1355                                       " or a list of these") 
1356          else: 
1357              raise TypeError(name+" must be in " + str(values) + \
 
1358                              " or a list of these") 
1359      else: 
1360          if value not in values: 
1361              raise ValueError(name+" must be in " + str(values))

1362  
 
1363  
 


1364 -def verify_intbool(name, value, list_ok=False, list_len=None):


1365      """Use list_ok if a list of values of these types is acceptable.
 
1366      list_len can be used to specify that a list must be of a certain length,
 
1367      either a fixed integer or a variable integer value given as the first
 
1368      value of a pair, the second being the name of the variable (for use in
 
1369      error messages)
 
1370      """ 
1371      if list_ok: 
1372          if isinstance(value, list): 
1373              if list_len is not None: 
1374                  if isinstance(list_len, _all_int): 
1375                      ok = (len(value) == list_len) 
1376                      len_name = '%d' % list_len 
1377                  else: 
1378                      ok = (len(value) == list_len[0]) 
1379                      len_name = list_len[1] 
1380                  if not ok: 
1381                      raise ValueError("list "+name+" length must equal "+len_name) 
1382              for v in value: 
1383                  try: 
1384                      # make sure v is not a list too
 
1385                      verify_intbool(name, v) 
1386                  except ValueError: 
1387                      raise ValueError(name+" must be 0, 1, or a boolean," + \
 
1388                                       " or a list of these") 
1389                  except TypeError: 
1390                      raise TypeError(name+" must be 0, 1, or a boolean," + \
 
1391                                       " or a list of these") 
1392          else: 
1393              raise TypeError(name+" must be 0, 1, or a boolean," + \
 
1394                              " or a list of these") 
1395      elif isinstance(value, _all_int): 
1396          if value not in [0, 1]: 
1397              raise ValueError("integer "+name+" must be 0 or 1") 
1398      elif not isinstance(value, bool): 
1399          raise TypeError(name+" must be 0, 1 or a boolean")

1400  
 
1401  
 


1402 -def verify_nonneg(name, value, types, list_ok=False, list_len=None):


1403      """Use list_ok if a list of values of these types is acceptable.
 
1404      list_len can be used to specify that a list must be of a certain length,
 
1405      either a fixed integer or a variable integer value given as the first
 
1406      value of a pair, the second being the name of the variable (for use in
 
1407      error messages)
 
1408      """ 
1409      if isinstance(value, types): 
1410          if value < 0: 
1411              raise ValueError(name+" must be non-negative") 
1412      elif list_ok: 
1413          if isinstance(value, list): 
1414              if list_len is not None: 
1415                  if isinstance(list_len, _all_int): 
1416                      ok = (len(value) == list_len) 
1417                      len_name = '%d' % list_len 
1418                  else: 
1419                      ok = (len(value) == list_len[0]) 
1420                      len_name = list_len[1] 
1421                  if not ok: 
1422                      raise ValueError("list "+name+" length must equal "+len_name) 
1423              for v in value: 
1424                  try: 
1425                      # make sure v is not a list too
 
1426                      verify_nonneg(name, v, types) 
1427                  except ValueError: 
1428                      raise ValueError(name+" must be "+_verify_type_names[types]+ \
 
1429                                       " and non-negative, or a list of these") 
1430                  except TypeError: 
1431                      raise TypeError(name+" must be "+_verify_type_names[types]+ \
 
1432                                       " and non-negative, or a list of these") 
1433          else: 
1434              raise TypeError(name+" must be "+_verify_type_names[types]+ \
 
1435                              " and non-negative, or a list of these") 
1436      else: 
1437          raise TypeError(name+" must be "+_verify_type_names[types]+ \
 
1438                              " and non-negative")

1439  
 
1440  
 


1441 -def verify_pos(name, value, types, list_ok=False, list_len=None):


1442      """Use list_ok if a list of values of these types is acceptable.
 
1443      list_len can be used to specify that a list must be of a certain length,
 
1444      either a fixed integer or a variable integer value given as the first
 
1445      value of a pair, the second being the name of the variable (for use in
 
1446      error messages)
 
1447      """ 
1448      if isinstance(value, types): 
1449          if value <= 0: 
1450              raise ValueError(name+" must be positive") 
1451      elif list_ok: 
1452          if isinstance(value, list): 
1453              if list_len is not None: 
1454                  if isinstance(list_len, _all_int): 
1455                      ok = (len(value) == list_len) 
1456                      len_name = '%d' % list_len 
1457                  else: 
1458                      ok = (len(value) == list_len[0]) 
1459                      len_name = list_len[1] 
1460                  if not ok: 
1461                      raise ValueError("list "+name+" length must equal "+len_name) 
1462              for v in value: 
1463                  try: 
1464                      # make sure v is not a list too
 
1465                      verify_nonneg(name, v, types) 
1466                  except ValueError: 
1467                      raise ValueError(name+" must be "+_verify_type_names[types]+ \
 
1468                                       " and positive, or a list of these") 
1469                  except TypeError: 
1470                      raise TypeError(name+" must be "+_verify_type_names[types]+ \
 
1471                                       " and positive, or a list of these") 
1472          else: 
1473              raise TypeError(name+" must be "+_verify_type_names[types]+ \
 
1474                              " and positive, or a list of these") 
1475      else: 
1476          raise TypeError(name+" must be "+_verify_type_names[types]+ \
 
1477                              " and positive")

1478  
 
1479  
 


1480 -def array_bounds_check(a, bounds, dirn=1):


1481      """Internal utility function to test a 1D array for staying within given
 
1482      bounds (min val, max val).
 
1483  
 
1484      Returns the largest index +1 if the array is within bounds, otherwise the
 
1485      first offending index, where 'first' is the earliest in a if direction
 
1486      dirn=1, or the latest if dirn=-1.""" 
1487      if dirn == 1: 
1488          OK_ix = len(a) 
1489          alo = asarray(a<bounds, int) 
1490          ahi = asarray(a>bounds, int) 
1491          alo_first = alo.argmax() 
1492          ahi_first = ahi.argmax() 
1493          test_val = 0 
1494          compare = min 
1495      elif dirn == -1: 
1496          OK_ix = -1 
1497          alo = 1 - asarray(a<bounds, int) 
1498          ahi = 1 - asarray(a>bounds, int) 
1499          alo_first = alo.argmin() 
1500          ahi_first = ahi.argmin() 
1501          test_val = 1 
1502          compare = max 
1503      else: 
1504          raise ValueError("Invalid direction") 
1505      first_fail_ix = OK_ix 
1506      if alo[alo_first] != test_val: 
1507          # an element was below lower bound
 
1508          first_fail_ix = alo_first 
1509      if ahi[ahi_first] != test_val: 
1510          # an element was above upper bound
 
1511          if first_fail_ix == OK_ix: 
1512              first_fail_ix = ahi_first 
1513          else: 
1514              first_fail_ix = compare(first_fail_ix, ahi_first) 
1515      return first_fail_ix

1516  
 
1517  
 


1518 -def linearInterp(y0, ygoal, y1, x0, x1):


1519      """Internal utility function to linearly interpolate between two
 
1520      data points.""" 
1521      return ( x1 * (ygoal - y0) + x0 * ( y1 - ygoal) ) / (y1 - y0)

1522  
 
1523  
 


1524 -def makeUniqueFn(fstr, tdigits=0, idstr=None):


1525      """Add unique ID to function names.
 
1526  
 
1527      Used when functions are executed in global namespace to avoid name
 
1528      clashes, and need to be distinguished when DS objects are copied.""" 
1529      # check for syntax errors
 
1530      try: 
1531          code = compile(fstr, 'test', 'exec') 
1532      except: 
1533          print " Cannot make unique function because of a syntax (or other) error " \
 
1534                "in supplied code:\n" 
1535          print fstr 
1536          raise 
1537      bracepos = fstr.index("(") 
1538      if idstr is None: 
1539          idstr_insert = "" 
1540      else: 
1541          idstr_insert = "_" + idstr 
1542      if tdigits > 0: 
1543          fname = fstr[4:bracepos] + idstr_insert + "_" + timestamp(tdigits) 
1544      else: 
1545          fname = fstr[4:bracepos] 
1546      fstr_new = "def " + fname + fstr[bracepos:] 
1547      return (fstr_new, fname)

1548  
 
1549  
 


1550 -def timestamp(tdigits=8):


1551      """Return a unique timestamp string for the session. useful for ensuring
 
1552      unique function identifiers, etc.
 
1553      """ 
1554      return str(time.clock()).replace(".", "").replace("-","")[:tdigits+1]

1555  
 
1556  
 


1557 -def isUniqueSeq(objlist):


1558      """Check that list contains items only once""" 
1559      if len(objlist) > 0: 
1560          return alltrue([objlist.count(obj) == 1 for obj in objlist]) 
1561      else: 
1562          return True

1563  
 


1564 -def makeSeqUnique(seq, asarray=False):


1565      """Return a 1D sequence that only contains the unique values in seq.
 
1566      Adapted from code by Raymond Hettinger, 2002""" 
1567      set = {} 
1568      if asarray: 
1569          return array([set.setdefault(e,e) for e in seq if e not in set]) 
1570      else: 
1571          return [set.setdefault(e,e) for e in seq if e not in set]

1572  
 
1573  
 


1574 -def object2str(x):


1575      """Convert occurrences of types / classes,
 
1576      to pretty-printable strings.""" 
1577      try: 
1578          if type(x) in [types.InstanceType, types.TypeType]: 
1579              return className(x, True) 
1580          elif isinstance(x, list): 
1581              # search through any iterable parts (that aren't strings)
 
1582              rx = "[" 
1583              if len(x)>0: 
1584                  for o in x: 
1585                      rx += object2str(o) + ", " 
1586                  return rx[:-2]+"]" 
1587              else: 
1588                  return rx+"]" 
1589          elif isinstance(x, tuple): 
1590              rx = "(" 
1591              if len(x)>0: 
1592                  for o in x: 
1593                      rx += object2str(o) + ", " 
1594                  return rx[:-2]+")" 
1595              else: 
1596                  return rx+")" 
1597          elif isinstance(x, dict): 
1598              rx = "{" 
1599              if len(x)>0: 
1600                  for k, o in x.iteritems(): 
1601                      rx += object2str(k) + ": " + object2str(o) + ", " 
1602                  return rx[:-2]+"}" 
1603              else: 
1604                  return rx+"}" 
1605          elif isinstance(x, str): 
1606              # this removes extraneous single quotes around dict keys, for instance
 
1607              return x 
1608          else: 
1609              return repr(x) 
1610      except: 
1611          raise TypeError("object2str cannot format this object type")

1612  
 
1613  
 
1614  #  The class types can show different roots when they originate from
 
1615  #  different parts of the PyDSTool package -- it might be a bug.
 
1616  #  e.g. baseClass might be Generator.Generator, but here this type will be
 
1617  #  <class 'PyDSTool.Generator.baseclasses.Generator'>
 
1618  #  and input.__class__ will boil down to
 
1619  #  <class 'Generator.baseclasses.Generator'>
 
1620  #  even though these classes are identical (constructed from the same class
 
1621  #  in the same module!)
 


1622 -def compareBaseClass(input, baseClass):


1623      """input may be a class or a class instance representing that class.
 
1624      baseClass may be a class or a string name of a class.
 
1625  
 
1626      Comparison is made using class names only.""" 
1627      if isinstance(baseClass, type): 
1628          base_str = baseClass.__name__ 
1629      elif isinstance(baseClass, str): 
1630          base_str = baseClass 
1631      else: 
1632          raise TypeError("Must pass either a class or a class name (string)") 
1633      if isinstance(input, type): 
1634          bases = input.__bases__ 
1635      else: 
1636          try: 
1637              bases = input.__class__.__bases__ 
1638          except AttributeError: 
1639              # not the kind of baseClass PyDSTool is interested in
 
1640              # e.g. an exception type
 
1641              return False 
1642      return sometrue([base_str == c.__name__ for c in bases])

1643  
 
1644  
 


1645 -def compareClassAndBases(input, arg):


1646      """arg can be a single or sequence of classes""" 
1647      try: 
1648          # if arg is iterable
 
1649          return sometrue([compareClassAndBases(input, a) for a in arg]) 
1650      except TypeError: 
1651          try: 
1652              if isinstance(input, type): 
1653                  # input is a class
 
1654                  return issubclass(input, arg) 
1655              else: 
1656                  # input is an instance
 
1657                  return isinstance(input, arg) 
1658          except TypeError: 
1659              raise TypeError("Invalid class(es) provided: input %s vs. %s" \
 
1660                              %(str(input)+" of type "+className(input),className(arg,True)))

1661  
 
1662  
 


1663 -def getSuperClasses(obj, limitClasses=None):


1664      """Return string names of all super classes of a given object""" 
1665      if limitClasses == None: 
1666          limitClassNames = ['object'] 
1667      elif isinstance(limitClasses, list): 
1668          limitClassNames = [className(lc) for lc in limitClasses] 
1669      else: 
1670          # singleton class
 
1671          limitClassNames = [className(limitClasses)] 
1672      # ensure "object" safety net is present
 
1673      if 'object' not in limitClassNames: 
1674          limitClassNames.append('object') 
1675      search_obj = [obj.__class__] 
1676      sclasses = [className(search_obj[0])] 
1677      # don't start while loop if obj is already of a type in limitClasses
 
1678      done = (sclasses[0] in limitClassNames) 
1679      c = 0 
1680      while not done and c < 10: 
1681          c += 1 
1682          search_temp = [] 
1683          for so in search_obj: 
1684              search_temp.extend(list(so.__bases__)) 
1685          search_obj = search_temp 
1686          for b in search_obj: 
1687              sclass = className(b) 
1688              done = sclass in limitClassNames 
1689              if done: 
1690                  break 
1691              else: 
1692                  sclasses.append(sclass) 
1693      return sclasses

1694  
 
1695  
 


1696 -def className(obj, addPrefix=False):


1697      """Return human-readable string of class name.""" 
1698      if isinstance(obj, str): 
1699          class_str = obj 
1700          # don't add prefix -- it's unknown
 
1701          prefix = "" 
1702      elif isinstance(obj, type): 
1703          class_str = obj.__name__ 
1704          if addPrefix: 
1705              prefix = "Class " 
1706          else: 
1707              prefix = "" 
1708      elif isinstance(obj, types.ModuleType): 
1709          class_str = obj.__name__ 
1710          if addPrefix: 
1711              prefix = "Module " 
1712          else: 
1713              prefix = "" 
1714      else: 
1715          try: 
1716              class_str = obj.__class__.__name__ 
1717          except AttributeError: 
1718              class_str = str(type(obj)) 
1719          prefix = "" 
1720      return prefix + class_str

1721  
 
1722  
 
1723  # little utility function to wrap value as a singleton list
 


1724 -def listid(val):


1725      return [val]

1726  
 
1727  
 
1728  # the identity function
 


1729 -def idfn(val):


1730      return copy(val)

1731  
 
1732  
 
1733  # utility function representing a "none" function
 


1734 -def noneFn(x):


1735      return None

1736  
 
1737  
 
1738  # returns the mapping from the entries in an array or list to their indices
 


1739 -def makeArrayIxMap(a):


1740      return dict(zip(a, range(len(a))))

1741  
 
1742  
 
1743  # invert an index mapping or other form of mapping
 


1744 -def invertMap(themap):


1745      """invert an index mapping or other form of mapping.
 
1746  
 
1747      If argument is a dict or sequence type, returns a dictionary,
 
1748      but if argument is a parseUtils.symbolMapClass then that type is
 
1749      returned.""" 
1750      if isinstance(themap, dict): 
1751          return dict(map(lambda (k,v): (v,k), themap.iteritems())) 
1752      elif isinstance(themap, (list,tuple)): 
1753          # input domain is the position index
 
1754          return dict(zip(themap, range(len(themap)))) 
1755      elif isinstance(themap, ndarray): 
1756          # input domain is the position index
 
1757          return dict(zip(themap.tolist(), range(len(themap)))) 
1758      elif hasattr(themap, 'inverse'): 
1759          # symbolMapClass type
 
1760          return themap.inverse() 
1761      else: 
1762          raise TypeError("Unsupported type for map")

1763  
 
1764  
 


1765 -def isincreasing(theseq, withVal=False):


1766      """
 
1767      Check whether a sequence is in increasing order. The withVal
 
1768      option (default False) causes the function to return the first
 
1769      two offending values that are not repeated.
 
1770      """ 
1771      # Note: This version of the function has better speed on the
 
1772      # 'usual' case where this function is used internally by PyDSTool
 
1773      # -- which is where the sequence *is* increasing and the input is
 
1774      # already an array
 
1775      try: 
1776          v_old = theseq[0] 
1777      except IndexError: 
1778          raise ValueError("Problem with sequence passed to "
 
1779                           "function `isincreasing` -- is it empty?") 
1780      v = array(theseq) 
1781      res = v[1:] > v[:-1] 
1782      if withVal: 
1783          if all(res): 
1784              return True, None, None 
1785          else: 
1786              pos = res.tolist().index(False) 
1787              return False, theseq[pos], theseq[pos+1] 
1788      else: 
1789          return all(res)

1790  
 
1791  
 


1792 -def ismonotonic(theseq, withVal=False):


1793      """
 
1794      Check whether a sequence is in strictly increasing or decreasing
 
1795      order. The withVal option (default False) causes the function to
 
1796      return the first two offending values that are not repeated.
 
1797      """ 
1798      if withVal: 
1799          res_incr, pos1, pos2 = isincreasing(theseq, True) 
1800          res_decr = isincreasing(theseq[::-1], False) 
1801          if res_incr or res_decr: 
1802              return True, None, None 
1803          else: 
1804              return False, pos1, pos2 
1805      else: 
1806          res_incr = isincreasing(theseq) 
1807          res_decr = isincreasing(theseq[::-1]) 
1808          return res_incr or res_decr

1809  
 
1810  
 


1811 -def extent(data):


1812      """Returns a pair of the min and max values of a dataset, or just a numeric type if these are equal.
 
1813      (Ignores NaNs.)
 
1814      """ 
1815      minval = npy.nanmin(data) 
1816      maxval = npy.nanmax(data) 
1817      if minval == maxval: 
1818          return minval 
1819      else: 
1820          return [minval, maxval]

1821  
 


1822 -def uniquePoints(ar):


1823      """For an n by m array input, return only points that are unique""" 
1824      result = [] 
1825      seq = set() 
1826      for a in ar: 
1827          a = tuple(a) 
1828          if a not in seq: 
1829              result.append(a) 
1830              seq.add(a) 
1831      return array(result)

1832  
 
1833  
 


1834 -def sortedDictValues(d, onlykeys=None, reverse=False):


1835      """Return list of values from a dictionary in order of sorted key list.
 
1836  
 
1837      Adapted from original function by Alex Martelli:
 
1838       added filtering of keys.
 
1839      """ 
1840      if onlykeys is None: 
1841          keys = d.keys() 
1842      else: 
1843          keys = intersect(d.keys(), onlykeys) 
1844      keys.sort() 
1845      if reverse: 
1846          keys.reverse() 
1847      return map(d.get, keys)

1848  
 


1849 -def sortedDictKeys(d, onlykeys=None, reverse=False):


1850      """Return sorted list of keys from a dictionary.
 
1851  
 
1852      Adapted from original function by Alex Martelli:
 
1853       added filtering of keys.""" 
1854      if onlykeys is None: 
1855          keys = d.keys() 
1856      else: 
1857          keys = intersect(d.keys(), onlykeys) 
1858      keys.sort() 
1859      if reverse: 
1860          keys.reverse() 
1861      return keys

1862  
 


1863 -def sortedDictLists(d, byvalue=True, onlykeys=None, reverse=False):


1864      """Return (key list, value list) pair from a dictionary,
 
1865      sorted by value (default) or key.
 
1866      Adapted from an original function by Duncan Booth.
 
1867      """ 
1868      if onlykeys is None: 
1869          onlykeys = d.keys() 
1870      if byvalue: 
1871          i = [(val, key) for (key, val) in d.items() if key in onlykeys] 
1872          i.sort() 
1873          if reverse: 
1874              i.reverse() 
1875          rvals = [val for (val, key) in i] 
1876          rkeys = [key for (val, key) in i] 
1877      else: 
1878          # by key
 
1879          i = [(key, val) for (key, val) in d.items() if key in onlykeys] 
1880          i.sort() 
1881          if reverse: 
1882              i.reverse() 
1883          rvals = [val for (key, val) in i] 
1884          rkeys = [key for (key, val) in i] 
1885      return (rkeys, rvals)

1886  
 


1887 -def sortedDictItems(d, byvalue=True, onlykeys=None, reverse=False):


1888      """Return list of (key, value) pairs of a dictionary,
 
1889      sorted by value (default) or key.
 
1890      Adapted from an original function by Duncan Booth.
 
1891      """ 
1892      ks, vs = sortedDictLists(d, byvalue, onlykeys, reverse) 
1893      return zip(ks,vs)

1894  
 
1895  # ----------------------------------------------------------------------
 
1896  
 
1897  ## private versions of these utils (cannot import them from utils!)
 
1898  
 
1899  # find intersection of two lists, sequences, etc.
 


1900 -def intersect(a, b):


1901      return filter(lambda e : e in b, a)

1902  
 
1903  
 
1904  # find remainder of two lists, sequences, etc., after intersection
 


1905 -def remain(a, b):


1906      return filter(lambda e : e not in b, a)

1907  
 
1908  
 
1909  # ----------------------------------------------------------------------
 
1910  
 
1911  # The Utility class may be abandoned in future versions.
 


1912 -class Utility(object):


1913      """
 
1914      Utility abstract class for manipulating and analyzing dynamical systems.
 
1915  
 
1916      Robert Clewley, March 2005.
 
1917  
 
1918  Subclasses of Utility could include such things as continuation tools,
 
1919  dimension reduction tools, parameter estimation tools.
 
1920  """ 
1921      pass

1922  
 
1923  
 
1924  # --------------------------------------------------------------------
 
1925  # This section adapted from scipy.interpolate
 
1926  # --------------------------------------------------------------------
 
1927  
 
1928  
 


1929 -class interpclass(object):


1930      """Abstract class for interpolators.""" 
1931      interp_axis = -1    # used to set which is default interpolation

1932                          # axis.  DO NOT CHANGE OR CODE WILL BREAK.
 
1933  
 
1934  
 


1935 -class interp0d(interpclass):


1936      """Design of this class based on SciPy's interp1d""" 
1937  
 


1938 -    def __init__(self, x, y, axis=-1, makecopy=0, bounds_error=1,
 
1939                   fill_value=None):


1940          """Initialize a piecewise-constant interpolation class
 
1941  
 
1942          Description:
 
1943            x and y are arrays of values used to approximate some function f:
 
1944              y = f(x)
 
1945            This class returns a function whose call method uses piecewise-
 
1946            constant interpolation to find the value of new points.
 
1947  
 
1948          Inputs:
 
1949              x -- a 1d array of monotonically increasing real values.
 
1950                   x cannot include duplicate values. (otherwise f is
 
1951                   overspecified)
 
1952              y -- an nd array of real values.  y's length along the
 
1953                   interpolation axis must be equal to the length
 
1954                   of x.
 
1955              axis -- specifies the axis of y along which to
 
1956                      interpolate. Interpolation defaults to the last
 
1957                      axis of y.  (default: -1)
 
1958              makecopy -- If 1, the class makes internal copies of x and y.
 
1959                      If 0, references to x and y are used. The default
 
1960                      is NOT to copy. (default: 0)
 
1961              bounds_error -- If 1, an error is thrown any time interpolation
 
1962                              is attempted on a value outside of the range
 
1963                              of x (where extrapolation is necessary).
 
1964                              If 0, out of bounds values are assigned the
 
1965                              NaN (#INF) value.  By default, an error is
 
1966                              raised, although this is prone to change.
 
1967                              (default: 1)
 
1968          """ 
1969          self.datapoints = (array(x, float), array(y, float))   # RHC -- for access from PyDSTool 
1970          self.type = float   # RHC -- for access from PyDSTool 
1971          self.axis = axis 
1972          self.makecopy = makecopy   # RHC -- renamed from copy to avoid nameclash 
1973          self.bounds_error = bounds_error 
1974          if fill_value is None: 
1975              self.fill_value = NaN   # RHC -- was:   array(0.0) / array(0.0) 
1976          else: 
1977              self.fill_value = fill_value 
1978  
 
1979          # Check that both x and y are at least 1 dimensional.
 
1980          if len(shape(x)) == 0 or len(shape(y)) == 0: 
1981              raise ValueError("x and y arrays must have at least one dimension.") 
1982          # make a "view" of the y array that is rotated to the
 
1983          # interpolation axis.
 
1984          oriented_x = x 
1985          oriented_y = swapaxes(y,self.interp_axis,axis) 
1986          interp_axis = self.interp_axis 
1987          len_x,len_y = shape(oriented_x)[interp_axis], \
 
1988                              shape(oriented_y)[interp_axis] 
1989          if len_x != len_y: 
1990              raise ValueError("x and y arrays must be equal in length along "
 
1991                                "interpolation axis.") 
1992          if len_x < 2 or len_y < 2: 
1993              raise ValueError("x and y arrays must have more than 1 entry") 
1994          self.x = array(oriented_x,copy=self.makecopy) 
1995          self.y = array(oriented_y,copy=self.makecopy)

1996  
 
1997  
 


1998 -    def __call__(self,x_new):


1999          """Find piecewise-constant interpolated y_new = <name>(x_new).
 
2000  
 
2001          Inputs:
 
2002            x_new -- New independent variables.
 
2003  
 
2004          Outputs:
 
2005            y_new -- Piecewise-constant interpolated values corresponding to x_new.
 
2006          """ 
2007          # 1. Handle values in x_new that are outside of x.  Throw error,
 
2008          #    or return a list of mask array indicating the outofbounds values.
 
2009          #    The behavior is set by the bounds_error variable.
 
2010          ## RHC -- was   x_new = atleast_1d(x_new)
 
2011          x_new_1d = atleast_1d(x_new) 
2012          out_of_bounds = self._check_bounds(x_new_1d) 
2013          # 2. Find where in the orignal data, the values to interpolate
 
2014          #    would be inserted.
 
2015          #    Note: If x_new[n] = x[m], then m is returned by searchsorted.
 
2016          x_new_indices = searchsorted(self.x,x_new_1d) 
2017          # 3. Clip x_new_indices so that they are within the range of
 
2018          #    self.x indices and at least 1.  Removes mis-interpolation
 
2019          #    of x_new[n] = x[0]
 
2020          x_new_indices = clip(x_new_indices,1,len(self.x)-1).astype(int) 
2021          # 4. Calculate the region that each x_new value falls in.
 
2022          lo = x_new_indices - 1; hi = x_new_indices 
2023  
 
2024          # !! take() should default to the last axis (IMHO) and remove
 
2025          # !! the extra argument.
 
2026          # 5. Calculate the actual value for each entry in x_new.
 
2027          y_lo = take(self.y,lo,axis=self.interp_axis) 
2028          y_hi = take(self.y,hi,axis=self.interp_axis) 
2029          y_new = (y_lo+y_hi)/2. 
2030          # 6. Fill any values that were out of bounds with NaN
 
2031          # !! Need to think about how to do this efficiently for
 
2032          # !! mutli-dimensional Cases.
 
2033          yshape = y_new.shape 
2034          y_new = y_new.ravel() 
2035          new_shape = list(yshape) 
2036          new_shape[self.interp_axis] = 1 
2037          sec_shape = [1]*len(new_shape) 
2038          sec_shape[self.interp_axis] = len(out_of_bounds) 
2039          out_of_bounds.shape = sec_shape 
2040          new_out = ones(new_shape)*out_of_bounds 
2041          putmask(y_new, new_out.ravel(), self.fill_value) 
2042          y_new.shape = yshape 
2043          # Rotate the values of y_new back so that they correspond to the
 
2044          # correct x_new values.
 
2045          result = swapaxes(y_new,self.interp_axis,self.axis) 
2046          try: 
2047              len(x_new) 
2048              return result 
2049          except TypeError: 
2050              return result[0] 
2051          return result

2052  
 
2053  
 


2054 -    def _check_bounds(self,x_new):


2055          # If self.bounds_error = 1, we raise an error if any x_new values
 
2056          # fall outside the range of x.  Otherwise, we return an array indicating
 
2057          # which values are outside the boundary region.
 
2058          # !! Needs some work for multi-dimensional x !!
 
2059          below_bounds = less(x_new,self.x[0]) 
2060          above_bounds = greater(x_new,self.x[-1]) 
2061          #  Note: sometrue has been redefined to handle length 0 arrays
 
2062          # !! Could provide more information about which values are out of bounds
 
2063          # RHC -- Changed these ValueErrors to PyDSTool_BoundsErrors
 
2064          if self.bounds_error and any(sometrue(below_bounds)): 
2065  ##            print "Input:", x_new
 
2066  ##            print "Bound:", self.x[0]
 
2067  ##            print "Difference input - bound:", x_new-self.x[0]
 
2068              raise PyDSTool_BoundsError(" A value in x_new is below the"
 
2069                                " interpolation range.") 
2070          if self.bounds_error and any(sometrue(above_bounds)): 
2071  ##            print "Input:", x_new
 
2072  ##            print "Bound:", self.x[-1]
 
2073  ##            print "Difference input - bound:", x_new-self.x[-1]
 
2074              raise PyDSTool_BoundsError(" A value in x_new is above the"
 
2075                                " interpolation range.") 
2076          # !! Should we emit a warning if some values are out of bounds.
 
2077          # !! matlab does not.
 
2078          out_of_bounds = logical_or(below_bounds,above_bounds) 
2079          return out_of_bounds

2080  
 
2081  
 
2082      # RHC added
 


2083 -    def __getstate__(self):


2084          d = copy(self.__dict__) 
2085          # remove reference to Cfunc self.type
 
2086          d['type'] = _num_type2name[self.type] 
2087          return d

2088  
 
2089      # RHC added
 


2090 -    def __setstate__(self, state):


2091          self.__dict__.update(state) 
2092          # reinstate Cfunc self.type
 
2093          self.type = _num_name2type[self.type]

2094  
 
2095  
 
2096  
 


2097 -class interp1d(interpclass):    # RHC -- made this a new-style Python class


2098 -    def __init__(self, x, y, kind='linear', axis=-1,
 
2099                   makecopy = 0, bounds_error=1, fill_value=None):


2100          """Initialize a 1d piecewise-linear interpolation class
 
2101  
 
2102          Description:
 
2103            x and y are arrays of values used to approximate some function f:
 
2104              y = f(x)
 
2105            This class returns a function whose call method uses linear
 
2106            interpolation to find the value of new points.
 
2107  
 
2108          Inputs:
 
2109              x -- a 1d array of monotonically increasing real values.
 
2110                   x cannot include duplicate values. (otherwise f is
 
2111                   overspecified)
 
2112              y -- an nd array of real values.  y's length along the
 
2113                   interpolation axis must be equal to the length
 
2114                   of x.
 
2115              kind -- specify the kind of interpolation: 'nearest', 'linear',
 
2116                      'cubic', or 'spline'
 
2117              axis -- specifies the axis of y along which to
 
2118                      interpolate. Interpolation defaults to the last
 
2119                      axis of y.  (default: -1)
 
2120              makecopy -- If 1, the class makes internal copies of x and y.
 
2121                      If 0, references to x and y are used. The default
 
2122                      is NOT to copy. (default: 0)
 
2123              bounds_error -- If 1, an error is thrown any time interpolation
 
2124                              is attempted on a value outside of the range
 
2125                              of x (where extrapolation is necessary).
 
2126                              If 0, out of bounds values are assigned the
 
2127                              NaN (#INF) value.  By default, an error is
 
2128                              raised, although this is prone to change.
 
2129                              (default: 1)
 
2130          """ 
2131          self.datapoints = (array(x, float), array(y, float))   # RHC -- for access from PyDSTool 
2132          self.type = float   # RHC -- for access from PyDSTool 
2133          self.axis = axis 
2134          self.makecopy = makecopy   # RHC -- renamed from copy to avoid nameclash 
2135          self.bounds_error = bounds_error 
2136          if fill_value is None: 
2137              self.fill_value = NaN   # RHC -- was:   array(0.0) / array(0.0) 
2138          else: 
2139              self.fill_value = fill_value 
2140  
 
2141          if kind != 'linear': 
2142              raise NotImplementedError("Only linear supported for now. "
 
2143                                        "Use fitpack routines for other types.") 
2144  
 
2145          # Check that both x and y are at least 1 dimensional.
 
2146          if len(shape(x)) == 0 or len(shape(y)) == 0: 
2147              raise ValueError("x and y arrays must have at least one dimension.") 
2148          # make a "view" of the y array that is rotated to the
 
2149          # interpolation axis.
 
2150          oriented_x = x 
2151          oriented_y = swapaxes(y,self.interp_axis,axis) 
2152          interp_axis = self.interp_axis 
2153          len_x,len_y = shape(oriented_x)[interp_axis], \
 
2154                              shape(oriented_y)[interp_axis] 
2155          if len_x != len_y: 
2156              raise ValueError("x and y arrays must be equal in length along "
 
2157                                "interpolation axis.") 
2158          if len_x < 2 or len_y < 2: 
2159              raise ValueError("x and y arrays must have more than 1 entry") 
2160          self.x = array(oriented_x,copy=self.makecopy) 
2161          self.y = array(oriented_y,copy=self.makecopy)

2162  
 
2163  
 


2164 -    def __call__(self,x_new):


2165          """Find linearly interpolated y_new = <name>(x_new).
 
2166  
 
2167          Inputs:
 
2168            x_new -- New independent variables.
 
2169  
 
2170          Outputs:
 
2171            y_new -- Linearly interpolated values corresponding to x_new.
 
2172          """ 
2173          # 1. Handle values in x_new that are outside of x.  Throw error,
 
2174          #    or return a list of mask array indicating the outofbounds values.
 
2175          #    The behavior is set by the bounds_error variable.
 
2176          ## RHC -- was   x_new = atleast_1d(x_new)
 
2177          x_new_1d = atleast_1d(x_new) 
2178          out_of_bounds = self._check_bounds(x_new_1d) 
2179          # 2. Find where in the orignal data, the values to interpolate
 
2180          #    would be inserted.
 
2181          #    Note: If x_new[n] = x[m], then m is returned by searchsorted.
 
2182          x_new_indices = searchsorted(self.x,x_new_1d) 
2183          # 3. Clip x_new_indices so that they are within the range of
 
2184          #    self.x indices and at least 1.  Removes mis-interpolation
 
2185          #    of x_new[n] = x[0]
 
2186          x_new_indices = clip(x_new_indices,1,len(self.x)-1).astype(int) 
2187          # 4. Calculate the slope of regions that each x_new value falls in.
 
2188          lo = x_new_indices - 1; hi = x_new_indices 
2189  
 
2190          # !! take() should default to the last axis (IMHO) and remove
 
2191          # !! the extra argument.
 
2192          x_lo = take(self.x,lo,axis=self.interp_axis) 
2193          x_hi = take(self.x,hi,axis=self.interp_axis) 
2194          y_lo = take(self.y,lo,axis=self.interp_axis) 
2195          y_hi = take(self.y,hi,axis=self.interp_axis) 
2196          slope = (y_hi-y_lo)/(x_hi-x_lo) 
2197          # 5. Calculate the actual value for each entry in x_new.
 
2198          y_new = slope*(x_new_1d-x_lo) + y_lo 
2199          # 6. Fill any values that were out of bounds with NaN
 
2200          # !! Need to think about how to do this efficiently for
 
2201          # !! mutli-dimensional Cases.
 
2202          yshape = y_new.shape 
2203          y_new = y_new.ravel() 
2204          new_shape = list(yshape) 
2205          new_shape[self.interp_axis] = 1 
2206          sec_shape = [1]*len(new_shape) 
2207          sec_shape[self.interp_axis] = len(out_of_bounds) 
2208          out_of_bounds.shape = sec_shape 
2209          new_out = ones(new_shape)*out_of_bounds 
2210          putmask(y_new, new_out.ravel(), self.fill_value) 
2211          y_new.shape = yshape 
2212          # Rotate the values of y_new back so that they correspond to the
 
2213          # correct x_new values.
 
2214          result = swapaxes(y_new,self.interp_axis,self.axis) 
2215          try: 
2216              len(x_new) 
2217              return result 
2218          except TypeError: 
2219              return result[0] 
2220          return result

2221  
 
2222  
 


2223 -    def _check_bounds(self,x_new):


2224          # If self.bounds_error = 1, we raise an error if any x_new values
 
2225          # fall outside the range of x.  Otherwise, we return an array indicating
 
2226          # which values are outside the boundary region.
 
2227          # !! Needs some work for multi-dimensional x !!
 
2228          below_bounds = less(x_new,self.x[0]) 
2229          above_bounds = greater(x_new,self.x[-1]) 
2230          #  Note: sometrue has been redefined to handle length 0 arrays
 
2231          # !! Could provide more information about which values are out of bounds
 
2232          # RHC -- Changed these ValueErrors to PyDSTool_BoundsErrors
 
2233          if self.bounds_error and any(sometrue(below_bounds)): 
2234  ##            print "Input:", x_new
 
2235  ##            print "Bound:", self.x[0]
 
2236  ##            print "Difference input - bound:", x_new-self.x[0]
 
2237              raise PyDSTool_BoundsError("A value in x_new is below the"
 
2238                                " interpolation range.") 
2239          if self.bounds_error and any(sometrue(above_bounds)): 
2240  ##            print "Input:", x_new
 
2241  ##            print "Bound:", self.x[-1]
 
2242  ##            print "Difference input - bound:", x_new-self.x[-1]
 
2243              raise PyDSTool_BoundsError("A value in x_new is above the"
 
2244                                " interpolation range.") 
2245          # !! Should we emit a warning if some values are out of bounds.
 
2246          # !! matlab does not.
 
2247          out_of_bounds = logical_or(below_bounds,above_bounds) 
2248          return out_of_bounds

2249  
 
2250  
 
2251      # RHC added
 


2252 -    def __getstate__(self):


2253          d = copy(self.__dict__) 
2254          # remove reference to Cfunc self.type
 
2255          d['type'] = _num_type2name[self.type] 
2256          return d

2257  
 
2258      # RHC added
 


2259 -    def __setstate__(self, state):


2260          self.__dict__.update(state) 
2261          # reinstate Cfunc self.type
 
2262          self.type = _num_name2type[self.type]

2263  
 
2264  
 
2265  # The following interpolation functions were written and (c) Anne
 
2266  # Archibald.
 
2267  
 


2268 -class KroghInterpolator(object):


2269      """The interpolating polynomial for a set of points
 
2270  
 
2271      Constructs a polynomial that passes through a given set of points,
 
2272      optionally with specified derivatives at those points.
 
2273      Allows evaluation of the polynomial and all its derivatives.
 
2274      For reasons of numerical stability, this function does not compute
 
2275      the coefficients of the polynomial, although they can be obtained
 
2276      by evaluating all the derivatives.
 
2277  
 
2278      Be aware that the algorithms implemented here are not necessarily
 
2279      the most numerically stable known. Moreover, even in a world of
 
2280      exact computation, unless the x coordinates are chosen very
 
2281      carefully - Chebyshev zeros (e.g. cos(i*pi/n)) are a good choice -
 
2282      polynomial interpolation itself is a very ill-conditioned process
 
2283      due to the Runge phenomenon. In general, even with well-chosen
 
2284      x values, degrees higher than about thirty cause problems with
 
2285      numerical instability in this code.
 
2286  
 
2287      Based on Krogh 1970, "Efficient Algorithms for Polynomial Interpolation
 
2288      and Numerical Differentiation"
 
2289      """ 


2290 -    def __init__(self, xi, yi):


2291          """Construct an interpolator passing through the specified points
 
2292  
 
2293          The polynomial passes through all the pairs (xi,yi). One may additionally
 
2294          specify a number of derivatives at each point xi; this is done by
 
2295          repeating the value xi and specifying the derivatives as successive
 
2296          yi values.
 
2297  
 
2298          Parameters
 
2299          ----------
 
2300          xi : array-like, length N
 
2301              known x-coordinates
 
2302          yi : array-like, N by R
 
2303              known y-coordinates, interpreted as vectors of length R,
 
2304              or scalars if R=1
 
2305  
 
2306          Example
 
2307          -------
 
2308          To produce a polynomial that is zero at 0 and 1 and has
 
2309          derivative 2 at 0, call
 
2310  
 
2311          >>> KroghInterpolator([0,0,1],[0,2,0])
 
2312          """ 
2313          self.xi = npy.asarray(xi) 
2314          self.yi = npy.asarray(yi) 
2315          if len(self.yi.shape)==1: 
2316              self.vector_valued = False 
2317              self.yi = self.yi[:,npy.newaxis] 
2318          elif len(self.yi.shape)>2: 
2319              raise ValueError, "y coordinates must be either scalars or vectors" 
2320          else: 
2321              self.vector_valued = True 
2322  
 
2323          n = len(xi) 
2324          self.n = n 
2325          nn, r = self.yi.shape 
2326          if nn!=n: 
2327              raise ValueError, "%d x values provided and %d y values; must be equal" % (n, nn) 
2328          self.r = r 
2329  
 
2330          c = npy.zeros((n+1,r)) 
2331          c[0] = yi[0] 
2332          Vk = npy.zeros((n,r)) 
2333          for k in xrange(1,n): 
2334              s = 0 
2335              while s<=k and xi[k-s]==xi[k]: 
2336                  s += 1 
2337              s -= 1 
2338              Vk[0] = yi[k]/float(spy.factorial(s)) 
2339              for i in xrange(k-s): 
2340                  assert xi[i]!=xi[k] 
2341                  if s==0: 
2342                      Vk[i+1] = (c[i]-Vk[i])/(xi[i]-xi[k]) 
2343                  else: 
2344                      Vk[i+1] = (Vk[i+1]-Vk[i])/(xi[i]-xi[k]) 
2345              c[k] = Vk[k-s] 
2346          self.c = c

2347  
 


2348 -    def __call__(self,x):


2349          """Evaluate the polynomial at the point x
 
2350  
 
2351          Parameters
 
2352          ----------
 
2353          x : scalar or array-like of length N
 
2354  
 
2355          Returns
 
2356          -------
 
2357          y : scalar, array of length R, array of length N, or array of length N by R
 
2358              If x is a scalar, returns either a vector or a scalar depending on
 
2359              whether the interpolator is vector-valued or scalar-valued.
 
2360              If x is a vector, returns a vector of values.
 
2361          """ 
2362          if npy.isscalar(x): 
2363              scalar = True 
2364              m = 1 
2365          else: 
2366              scalar = False 
2367              m = len(x) 
2368          x = npy.asarray(x) 
2369  
 
2370          n = self.n 
2371          pi = 1 
2372          p = npy.zeros((m,self.r)) 
2373          p += self.c[0,npy.newaxis,:] 
2374          for k in xrange(1,n): 
2375              w = x - self.xi[k-1] 
2376              pi = w*pi 
2377              p = p + npy.multiply.outer(pi,self.c[k]) 
2378          if not self.vector_valued: 
2379              if scalar: 
2380                  return p[0,0] 
2381              else: 
2382                  return p[:,0] 
2383          else: 
2384              if scalar: 
2385                  return p[0] 
2386              else: 
2387                  return p

2388  
 


2389 -    def derivatives(self,x,der=None):


2390          """Evaluate many derivatives of the polynomial at the point x
 
2391  
 
2392          Produce an array of all derivative values at the point x.
 
2393  
 
2394          Parameters
 
2395          ----------
 
2396          x : scalar or array-like of length N
 
2397              Point or points at which to evaluate the derivatives
 
2398          der : None or integer
 
2399              How many derivatives to extract; None for all potentially
 
2400              nonzero derivatives (that is a number equal to the number
 
2401              of points). This number includes the function value as 0th
 
2402              derivative.
 
2403          Returns
 
2404          -------
 
2405          d : array
 
2406              If the interpolator's values are R-dimensional then the
 
2407              returned array will be der by N by R. If x is a scalar,
 
2408              the middle dimension will be dropped; if R is 1 then the
 
2409              last dimension will be dropped.
 
2410  
 
2411          Example
 
2412          -------
 
2413          >>> KroghInterpolator([0,0,0],[1,2,3]).derivatives(0)
 
2414          array([1.0,2.0,3.0])
 
2415          >>> KroghInterpolator([0,0,0],[1,2,3]).derivatives([0,0])
 
2416          array([[1.0,1.0],
 
2417                 [2.0,2.0],
 
2418                 [3.0,3.0]])
 
2419          """ 
2420          if npy.isscalar(x): 
2421              scalar = True 
2422              m = 1 
2423          else: 
2424              scalar = False 
2425              m = len(x) 
2426          x = npy.asarray(x) 
2427  
 
2428          n = self.n 
2429          r = self.r 
2430  
 
2431          if der is None: 
2432              der = self.n 
2433          dern = min(self.n,der) 
2434          pi = npy.zeros((n,m)) 
2435          w = npy.zeros((n,m)) 
2436          pi[0] = 1 
2437          p = npy.zeros((m,self.r)) 
2438          p += self.c[0,npy.newaxis,:] 
2439  
 
2440          for k in xrange(1,n): 
2441              w[k-1] = x - self.xi[k-1] 
2442              pi[k] = w[k-1]*pi[k-1] 
2443              p += npy.multiply.outer(pi[k],self.c[k]) 
2444  
 
2445          cn = npy.zeros((max(der,n+1),m,r)) 
2446          cn[:n+1,...] += self.c[:n+1,npy.newaxis,:] 
2447          cn[0] = p 
2448          for k in xrange(1,n): 
2449              for i in xrange(1,n-k+1): 
2450                  pi[i] = w[k+i-1]*pi[i-1]+pi[i] 
2451                  cn[k] = cn[k]+pi[i,:,npy.newaxis]*cn[k+i] 
2452              cn[k]*=factorial(k) 
2453  
 
2454          cn[n,...] = 0 
2455          if not self.vector_valued: 
2456              if scalar: 
2457                  return cn[:der,0,0] 
2458              else: 
2459                  return cn[:der,:,0] 
2460          else: 
2461              if scalar: 
2462                  return cn[:der,0] 
2463              else: 
2464                  return cn[:der]


2465 -    def derivative(self,x,der):


2466          """Evaluate one derivative of the polynomial at the point x
 
2467  
 
2468          Parameters
 
2469          ----------
 
2470          x : scalar or array-like of length N
 
2471              Point or points at which to evaluate the derivatives
 
2472          der : None or integer
 
2473              Which derivative to extract. This number includes the
 
2474              function value as 0th derivative.
 
2475          Returns
 
2476          -------
 
2477          d : array
 
2478              If the interpolator's values are R-dimensional then the
 
2479              returned array will be N by R. If x is a scalar,
 
2480              the middle dimension will be dropped; if R is 1 then the
 
2481              last dimension will be dropped.
 
2482  
 
2483          Notes
 
2484          -----
 
2485          This is computed by evaluating all derivatives up to the desired
 
2486          one and then discarding the rest.
 
2487          """ 
2488          return self.derivatives(x,der=der+1)[der]

2489  
 
2490  
 


2491 -class BarycentricInterpolator(object):


2492      """The interpolating polynomial for a set of points
 
2493  
 
2494      Constructs a polynomial that passes through a given set of points.
 
2495      Allows evaluation of the polynomial, efficient changing of the y
 
2496      values to be interpolated, and updating by adding more x values.
 
2497      For reasons of numerical stability, this function does not compute
 
2498      the coefficients of the polynomial.
 
2499  
 
2500      This class uses a "barycentric interpolation" method that treats
 
2501      the problem as a special case of rational function interpolation.
 
2502      This algorithm is quite stable, numerically, but even in a world of
 
2503      exact computation, unless the x coordinates are chosen very
 
2504      carefully - Chebyshev zeros (e.g. cos(i*pi/n)) are a good choice -
 
2505      polynomial interpolation itself is a very ill-conditioned process
 
2506      due to the Runge phenomenon.
 
2507  
 
2508      Based on Berrut and Trefethen 2004, "Barycentric Lagrange Interpolation".
 
2509      """ 


2510 -    def __init__(self, xi, yi=None):


2511          """Construct an object capable of interpolating functions sampled at xi
 
2512  
 
2513          The values yi need to be provided before the function is evaluated,
 
2514          but none of the preprocessing depends on them, so rapid updates
 
2515          are possible.
 
2516  
 
2517          Parameters
 
2518          ----------
 
2519          xi : array-like of length N
 
2520              The x coordinates of the points the polynomial should pass through
 
2521          yi : array-like N by R or None
 
2522              The y coordinates of the points the polynomial should pass through;
 
2523              if R>1 the polynomial is vector-valued. If None the y values
 
2524              will be supplied later.
 
2525          """ 
2526          self.n = len(xi) 
2527          self.xi = npy.asarray(xi) 
2528          if yi is not None and len(yi)!=len(self.xi): 
2529              raise ValueError, "yi dimensions do not match xi dimensions" 
2530          self.set_yi(yi) 
2531          self.wi = npy.zeros(self.n) 
2532          self.wi[0] = 1 
2533          for j in xrange(1,self.n): 
2534              self.wi[:j]*=(self.xi[j]-self.xi[:j]) 
2535              self.wi[j] = npy.multiply.reduce(self.xi[:j]-self.xi[j]) 
2536          self.wi**=-1

2537  
 


2538 -    def set_yi(self, yi):


2539          """Update the y values to be interpolated
 
2540  
 
2541          The barycentric interpolation algorithm requires the calculation
 
2542          of weights, but these depend only on the xi. The yi can be changed
 
2543          at any time.
 
2544  
 
2545          Parameters
 
2546          ----------
 
2547          yi : array-like N by R
 
2548              The y coordinates of the points the polynomial should pass through;
 
2549              if R>1 the polynomial is vector-valued. If None the y values
 
2550              will be supplied later.
 
2551          """ 
2552          if yi is None: 
2553              self.yi = None 
2554              return 
2555          yi = npy.asarray(yi) 
2556          if len(yi.shape)==1: 
2557              self.vector_valued = False 
2558              yi = yi[:,npy.newaxis] 
2559          elif len(yi.shape)>2: 
2560              raise ValueError, "y coordinates must be either scalars or vectors" 
2561          else: 
2562              self.vector_valued = True 
2563  
 
2564          n, r = yi.shape 
2565          if n!=len(self.xi): 
2566              raise ValueError, "yi dimensions do not match xi dimensions" 
2567          self.yi = yi 
2568          self.r = r

2569  
 
2570  
 


2571 -    def add_xi(self, xi, yi=None):


2572          """Add more x values to the set to be interpolated
 
2573  
 
2574          The barycentric interpolation algorithm allows easy updating by
 
2575          adding more points for the polynomial to pass through.
 
2576  
 
2577          Parameters
 
2578          ----------
 
2579          xi : array-like of length N1
 
2580              The x coordinates of the points the polynomial should pass through
 
2581          yi : array-like N1 by R or None
 
2582              The y coordinates of the points the polynomial should pass through;
 
2583              if R>1 the polynomial is vector-valued. If None the y values
 
2584              will be supplied later. The yi should be specified if and only if
 
2585              the interpolator has y values specified.
 
2586          """ 
2587          if yi is not None: 
2588              if self.yi is None: 
2589                  raise ValueError, "No previous yi value to update!" 
2590              yi = npy.asarray(yi) 
2591              if len(yi.shape)==1: 
2592                  if self.vector_valued: 
2593                      raise ValueError, "Cannot extend dimension %d y vectors with scalars" % self.r 
2594                  yi = yi[:,npy.newaxis] 
2595              elif len(yi.shape)>2: 
2596                  raise ValueError, "y coordinates must be either scalars or vectors" 
2597              else: 
2598                  n, r = yi.shape 
2599                  if r!=self.r: 
2600                      raise ValueError, "Cannot extend dimension %d y vectors with dimension %d y vectors" % (self.r, r) 
2601  
 
2602              self.yi = npy.vstack((self.yi,yi)) 
2603          else: 
2604              if self.yi is not None: 
2605                  raise ValueError, "No update to yi provided!" 
2606          old_n = self.n 
2607          self.xi = npy.concatenate((self.xi,xi)) 
2608          self.n = len(self.xi) 
2609          self.wi**=-1 
2610          old_wi = self.wi 
2611          self.wi = npy.zeros(self.n) 
2612          self.wi[:old_n] = old_wi 
2613          for j in xrange(old_n,self.n): 
2614              self.wi[:j]*=(self.xi[j]-self.xi[:j]) 
2615              self.wi[j] = npy.multiply.reduce(self.xi[:j]-self.xi[j]) 
2616          self.wi**=-1

2617  
 


2618 -    def __call__(self, x):


2619          """Evaluate the interpolating polynomial at the points x
 
2620  
 
2621          Parameters
 
2622          ----------
 
2623          x : scalar or array-like of length M
 
2624  
 
2625          Returns
 
2626          -------
 
2627          y : scalar or array-like of length R or length M or M by R
 
2628              The shape of y depends on the shape of x and whether the
 
2629              interpolator is vector-valued or scalar-valued.
 
2630  
 
2631          Notes
 
2632          -----
 
2633          Currently the code computes an outer product between x and the
 
2634          weights, that is, it constructs an intermediate array of size
 
2635          N by M, where N is the degree of the polynomial.
 
2636          """ 
2637          scalar = npy.isscalar(x) 
2638          x = npy.atleast_1d(x) 
2639          c = npy.subtract.outer(x,self.xi) 
2640          z = c==0 
2641          c[z] = 1 
2642          c = self.wi/c 
2643          p = npy.dot(c,self.yi)/npy.sum(c,axis=-1)[:,npy.newaxis] 
2644          i, j = npy.nonzero(z) 
2645          p[i] = self.yi[j] 
2646          if not self.vector_valued: 
2647              if scalar: 
2648                  return p[0,0] 
2649              else: 
2650                  return p[:,0] 
2651          else: 
2652              if scalar: 
2653                  return p[0] 
2654              else: 
2655                  return p

2656  
 
2657  # RHC - made a sub-class of interpclass
 


2658 -class PiecewisePolynomial(interpclass):


2659      """Piecewise polynomial curve specified by points and derivatives.
 
2660  
 
2661      This class represents a curve that is a piecewise polynomial. It
 
2662      passes through a list of points and has specified derivatives at
 
2663      each point. The degree of the polynomial may very from segment to
 
2664      segment, as may the number of derivatives available. The degree
 
2665      should not exceed about thirty.
 
2666  
 
2667      Appending points to the end of the curve is efficient.
 
2668      """ 


2669 -    def __init__(self, xi, yi, orders=None, direction=None):


2670          """Construct a piecewise polynomial
 
2671  
 
2672          Parameters
 
2673          ----------
 
2674          xi : array-like of length N
 
2675              a sorted list of x-coordinates
 
2676          yi : list of lists of length N
 
2677              yi[i] is the list of derivatives known at xi[i]
 
2678          orders : list of integers, or integer
 
2679              a list of polynomial orders, or a single universal order
 
2680          direction : {None, 1, -1}
 
2681              indicates whether the xi are increasing or decreasing
 
2682              +1 indicates increasing
 
2683              -1 indicates decreasing
 
2684              None indicates that it should be deduced from the first two xi
 
2685  
 
2686          Notes
 
2687          -----
 
2688          If orders is None, or orders[i] is None, then the degree of the
 
2689          polynomial segment is exactly the degree required to match all i
 
2690          available derivatives at both endpoints. If orders[i] is not None,
 
2691          then some derivatives will be ignored. The code will try to use an
 
2692          equal number of derivatives from each end; if the total number of
 
2693          derivatives needed is odd, it will prefer the rightmost endpoint. If
 
2694          not enough derivatives are available, an exception is raised.
 
2695          """ 
2696          # RHC added datapoints for use by PyDSTool
 
2697          # don't store any derivative information in datapoints
 
2698          self.datapoints = (array(xi, float), array(yi[:,0], float)) 
2699          self.type = float    # RHC -- for access from PyDSTool 
2700          yi0 = npy.asarray(yi[0]) 
2701          if len(yi0.shape)==2: 
2702              self.vector_valued = True 
2703              self.r = yi0.shape[1] 
2704          elif len(yi0.shape)==1: 
2705              self.vector_valued = False 
2706              self.r = 1 
2707          else: 
2708              raise ValueError, "Each derivative must be a vector, not a higher-rank array" 
2709  
 
2710          self.xi = [xi[0]] 
2711          self.yi = [yi0] 
2712          self.n = 1 
2713  
 
2714          self.direction = direction 
2715          self.orders = [] 
2716          self.polynomials = [] 
2717          self.extend(xi[1:],yi[1:],orders)

2718  
 


2719 -    def _make_polynomial(self,x1,y1,x2,y2,order,direction):


2720          """Construct the interpolating polynomial object
 
2721  
 
2722          Deduces the number of derivatives to match at each end
 
2723          from order and the number of derivatives available. If
 
2724          possible it uses the same number of derivatives from
 
2725          each end; if the number is odd it tries to take the
 
2726          extra one from y2. In any case if not enough derivatives
 
2727          are available at one end or another it draws enough to
 
2728          make up the total from the other end.
 
2729          """ 
2730          n = order+1 
2731          n1 = min(n//2,len(y1)) 
2732          n2 = min(n-n1,len(y2)) 
2733          n1 = min(n-n2,len(y1)) 
2734          if n1+n2!=n: 
2735              raise ValueError, "Point %g has %d derivatives, point %g has %d derivatives, but order %d requested" % (x1, len(y1), x2, len(y2), order) 
2736          assert n1<=len(y1) 
2737          assert n2<=len(y2) 
2738  
 
2739          xi = npy.zeros(n) 
2740          if self.vector_valued: 
2741              yi = npy.zeros((n,self.r)) 
2742          else: 
2743              yi = npy.zeros((n,)) 
2744  
 
2745          xi[:n1] = x1 
2746          yi[:n1] = y1[:n1] 
2747          xi[n1:] = x2 
2748          yi[n1:] = y2[:n2] 
2749  
 
2750          return KroghInterpolator(xi,yi)

2751  
 


2752 -    def append(self, xi, yi, order=None):


2753          """Append a single point with derivatives to the PiecewisePolynomial
 
2754  
 
2755          Parameters
 
2756          ----------
 
2757          xi : float
 
2758          yi : array-like
 
2759              yi is the list of derivatives known at xi
 
2760          order : integer or None
 
2761              a polynomial order, or instructions to use the highest
 
2762              possible order
 
2763          """ 
2764  
 
2765          yi = npy.asarray(yi) 
2766          if self.vector_valued: 
2767              if (len(yi.shape)!=2 or yi.shape[1]!=self.r): 
2768                  raise ValueError, "Each derivative must be a vector of length %d" % self.r 
2769          else: 
2770              if len(yi.shape)!=1: 
2771                  raise ValueError, "Each derivative must be a scalar" 
2772  
 
2773          if self.direction is None: 
2774              self.direction = npy.sign(xi-self.xi[-1]) 
2775          elif (xi-self.xi[-1])*self.direction < 0: 
2776              raise ValueError, "x coordinates must be in the %d direction: %s" % (self.direction, self.xi) 
2777  
 
2778          self.xi.append(xi) 
2779          self.yi.append(yi) 
2780  
 
2781  
 
2782          if order is None: 
2783              n1 = len(self.yi[-2]) 
2784              n2 = len(self.yi[-1]) 
2785              n = n1+n2 
2786              order = n-1 
2787  
 
2788          self.orders.append(order) 
2789          self.polynomials.append(self._make_polynomial(
 
2790              self.xi[-2], self.yi[-2],
 
2791              self.xi[-1], self.yi[-1],
 
2792              order, self.direction)) 
2793          self.n += 1

2794  
 
2795  
 


2796 -    def extend(self, xi, yi, orders=None):


2797          """Extend the PiecewisePolynomial by a list of points
 
2798  
 
2799          Parameters
 
2800          ----------
 
2801          xi : array-like of length N1
 
2802              a sorted list of x-coordinates
 
2803          yi : list of lists of length N1
 
2804              yi[i] is the list of derivatives known at xi[i]
 
2805          orders : list of integers, or integer
 
2806              a list of polynomial orders, or a single universal order
 
2807          direction : {None, 1, -1}
 
2808              indicates whether the xi are increasing or decreasing
 
2809              +1 indicates increasing
 
2810              -1 indicates decreasing
 
2811              None indicates that it should be deduced from the first two xi
 
2812          """ 
2813  
 
2814          for i in xrange(len(xi)): 
2815              if orders is None or npy.isscalar(orders): 
2816                  self.append(xi[i],yi[i],orders) 
2817              else: 
2818                  self.append(xi[i],yi[i],orders[i])

2819  
 


2820 -    def __call__(self, x):


2821          """Evaluate the piecewise polynomial
 
2822  
 
2823          Parameters
 
2824          ----------
 
2825          x : scalar or array-like of length N
 
2826  
 
2827          Returns
 
2828          -------
 
2829          y : scalar or array-like of length R or length N or N by R
 
2830          """ 
2831          if npy.isscalar(x): 
2832              pos = npy.clip(npy.searchsorted(self.xi, x) - 1, 0, self.n-2) 
2833              y = self.polynomials[pos](x) 
2834          else: 
2835              x = npy.asarray(x) 
2836              m = len(x) 
2837              pos = npy.clip(npy.searchsorted(self.xi, x) - 1, 0, self.n-2) 
2838              if self.vector_valued: 
2839                  y = npy.zeros((m,self.r)) 
2840              else: 
2841                  y = npy.zeros(m) 
2842              for i in xrange(self.n-1): 
2843                  c = pos==i 
2844                  y[c] = self.polynomials[i](x[c]) 
2845          return y

2846  
 


2847 -    def derivative(self, x, der):


2848          """Evaluate a derivative of the piecewise polynomial
 
2849  
 
2850          Parameters
 
2851          ----------
 
2852          x : scalar or array-like of length N
 
2853          der : integer
 
2854              which single derivative to extract
 
2855  
 
2856          Returns
 
2857          -------
 
2858          y : scalar or array-like of length R or length N or N by R
 
2859  
 
2860          Notes
 
2861          -----
 
2862          This currently computes all derivatives of the curve segment
 
2863          containing each x but returns only one. This is because the
 
2864          number of nonzero derivatives that a segment can have depends
 
2865          on the degree of the segment, which may vary.
 
2866          """ 
2867          return self.derivatives(x,der=der+1)[der]

2868  
 


2869 -    def derivatives(self, x, der):


2870          """Evaluate a derivative of the piecewise polynomial
 
2871  
 
2872          Parameters
 
2873          ----------
 
2874          x : scalar or array-like of length N
 
2875          der : integer
 
2876              how many derivatives (including the function value as
 
2877              0th derivative) to extract
 
2878  
 
2879          Returns
 
2880          -------
 
2881          y : array-like of shape der by R or der by N or der by N by R
 
2882  
 
2883          """ 
2884          if npy.isscalar(x): 
2885              pos = npy.clip(npy.searchsorted(self.xi, x) - 1, 0, self.n-2) 
2886              y = self.polynomials[pos].derivatives(x,der=der) 
2887          else: 
2888              x = npy.asarray(x) 
2889              m = len(x) 
2890              pos = npy.clip(npy.searchsorted(self.xi, x) - 1, 0, self.n-2) 
2891              if self.vector_valued: 
2892                  y = npy.zeros((der,m,self.r)) 
2893              else: 
2894                  y = npy.zeros((der,m)) 
2895              for i in xrange(self.n-1): 
2896                  c = pos==i 
2897                  y[:,c] = self.polynomials[i].derivatives(x[c],der=der) 
2898          return y

2899      # FIXME: provide multiderivative finder
 
2900  
 
2901      # RHC added
 


2902 -    def __getstate__(self):


2903          d = copy(self.__dict__) 
2904          # remove reference to Cfunc self.type
 
2905          d['type'] = _num_type2name[self.type] 
2906          return d

2907  
 
2908      # RHC added
 


2909 -    def __setstate__(self, state):


2910          self.__dict__.update(state) 
2911          # reinstate Cfunc self.type
 
2912          self.type = _num_name2type[self.type]

2913  
 
2914  # --------------------------------------------------------------------
 
2915  
 


2916 -def simple_bisection(tlo, thi, f, tol, imax=100):


2917      sol = None 
2918      flo = f(tlo) 
2919      fhi = f(thi) 
2920      i = 1 
2921      while i <= imax: 
2922          d = (thi - tlo)/2. 
2923          p = tlo + d 
2924          if d < tol: 
2925              sol = p 
2926              break 
2927          fp = f(p) 
2928          if fp == 0: 
2929              sol = p 
2930              break 
2931          i += 1 
2932          if fp*flo > 0: 
2933              tlo = p 
2934              flo = fp 
2935          else: 
2936              thi = p 
2937      if i == imax: 
2938          sol = p 
2939      return sol

2940  
 
2941  # Function fitting tools
 
2942  
 


2943 -class fit_function(object):


2944      """Abstract super-class for fitting explicit functions to 1D arrays of data
 
2945      using least squares.
 
2946  
 
2947      xs -- independent variable data
 
2948      ys -- dependent variable data
 
2949      pars_ic -- initial values defining the function
 
2950  
 
2951      Optional algorithmic parameters to minpack.leastsq can be passed in the
 
2952      algpars argument: e.g.,
 
2953      ftol -- Relative error desired in the sum of squares (default 1e-6).
 
2954      xtol -- Relative error desired in the approximate solution (default 1e-6).
 
2955      gtol -- Orthogonality desired between the function vector
 
2956              and the columns of the Jacobian (default 1e-8).
 
2957  
 
2958      Other parameters may be used for concrete sub-classes. Pass these as a dict
 
2959      or args object in the opts argument.
 
2960  
 
2961      Returns an args object with attributes:
 
2962  
 
2963      ys_fit --   the fitted y values corresponding to the given x data,
 
2964      pars_fit -- the function parameters at the fit
 
2965      info --     diagnostic feedback from the leastsq algorithm
 
2966      results --  dictionary of other function specific information (such as peak
 
2967                   position)
 
2968      """ 
2969  
 


2970 -    def __init__(self, pars_ic=None, algpars=None, opts=None,
 
2971                   verbose=False):


2972          # defaults
 
2973          self.algpars = args(ftol=1e-8, xtol=1e-6, gtol=1e-8, maxfev=100) 
2974          if algpars is not None: 
2975              self.algpars.update(algpars) 
2976          self.verbose = verbose 
2977          self.pars_ic = pars_ic 
2978          if hasattr(opts, 'weight'): 
2979              self.weight = opts.weight 
2980          else: 
2981              self.weight = 1

2982  
 


2983 -    def fn(self, x, *pars):


2984          raise NotImplementedError("Override in a concrete sub-class")

2985  
 


2986 -    def _do_fit(self, constraint, xs, ys, pars_ic):


2987          xs = asarray(xs) 
2988          ys = asarray(ys) 
2989          weight = self.weight 
2990  
 
2991          if constraint is None: 
2992              if self.verbose: 
2993                  def res_fn(p): 
2994                      print "\n",p 
2995                      r = self.fn(xs, *p) - ys 
2996                      print "Residual = %f"%norm(r*weight) 
2997                      return r*weight

2998              else: 
2999                  def res_fn(p): 
3000                      r = self.fn(xs, *p) - ys 
3001                      return r*weight

3002          else: 
3003              if self.verbose: 
3004                  def res_fn(p): 
3005                      print "\n",p 
3006                      r = npy.concatenate((constraint(*p), (self.fn(xs, *p) - ys)*weight)) 
3007                      print "Residual = %f"%norm(r) 
3008                      return r 
3009              else: 
3010                  def res_fn(p): 
3011                      return npy.concatenate((constraint(*p), (self.fn(xs, *p) - ys)*weight)) 
3012  
 
3013          try: 
3014              res = minpack.leastsq(res_fn, pars_ic,
 
3015                                full_output = True,
 
3016                                ftol = self.algpars.ftol,
 
3017                                xtol = self.algpars.xtol,
 
3018                                gtol = self.algpars.gtol,
 
3019                                maxfev = self.algpars.maxfev) 
3020          except: 
3021              print "Error at parameters", pars_ic 
3022              raise 
3023          if self.verbose: 
3024              print "Result: ", res 
3025          return res 
3026  
 


3027 -    def fit(self, xs, ys, pars_ic=None, opts=None):


3028          raise NotImplementedError("Override in a concrete sub-class")

3029  
 
3030  
 


3031 -class fit_quadratic(fit_function):


3032      """Fit a quadratic function y=a*x^2+b*x+c to the (x,y) array data.
 
3033      If initial parameter values = (a,b,c) are not given, the values
 
3034      (1,1,0) will be used.
 
3035  
 
3036      If peak_constraint is a tuple of values (x_index, y_value, weight_x,
 
3037      weight_y) for the approximate position of a turning point in the data,
 
3038      then this will be used as a soft constraint in the fit.
 
3039  
 
3040      result.peak is a (xpeak, ypeak) pair.
 
3041      result.f is the fitted function (accepts x values).
 
3042      """ 
3043  
 


3044 -    def fn(self, x, a, b, c):


3045          return a*x**2+b*x+c

3046  
 


3047 -    def fit(self, xs, ys, pars_ic=None, opts=None):


3048          try: 
3049              peak_constraint = opts.peak_constraint 
3050          except AttributeError: 
3051              peak_constraint = None 
3052  
 
3053          if pars_ic is None: 
3054              if self.pars_ic is None: 
3055                  pars_ic = array([1.,1.,0.]) 
3056              else: 
3057                  pars_ic = self.pars_ic 
3058  
 
3059          if peak_constraint is None: 
3060              constraint = None 
3061          else: 
3062              x_index, y_value, weight_x, weight_y = peak_constraint 
3063              def constraint(a,b,c): 
3064                  return array([weight_y*(self.fn(xs[x_index],a,b,c)-y_value),
 
3065                                weight_x*(xs[x_index]+b/(2*a))])

3066          res = self._do_fit(constraint, xs, ys, pars_ic) 
3067          sol = res[0] 
3068          a,b,c = sol 
3069          def f(x): 
3070              return a*x**2+b*x+c

3071          ys_fit = f(xs) 
3072          xpeak = -b/(2*a) 
3073          ypeak = f(xpeak) 
3074          return args(ys_fit=ys_fit, pars_fit=(a,b,c), info=res,
 
3075                            results=args(peak=(xpeak, ypeak),
 
3076                                         f=f)) 
3077  
 


3078 -class fit_quadratic_at_vertex(fit_function):


3079      """Fit a quadratic function y=a*(x+h)**2+k to the (x,y) array data,
 
3080      constrained to have a vertex at (h, k), leaving only the free parameter
 
3081      a for the curvature. (h, k) is specified through the peak_constraint
 
3082      option in the initialization argument 'opts'.
 
3083  
 
3084      If initial parameter value = a is not given, the value 1 will be used.
 
3085  
 
3086      result.peak is a (xpeak, ypeak) pair, but corresponds to (h,k).
 
3087      result.f is the fitted function (accepts x values).
 
3088      """ 
3089  
 


3090 -    def fn(self, x, a):


3091          return a*(x+self.h)**2+self.k

3092  
 


3093 -    def fit(self, xs, ys, pars_ic=None, opts=None):


3094          self.h, self.k = opts.peak_constraint 
3095          if pars_ic is None: 
3096              if self.pars_ic is None: 
3097                  pars_ic = (1,) 
3098              else: 
3099                  pars_ic = (self.pars_ic,) 
3100  
 
3101          res = self._do_fit(None, xs, ys, pars_ic) 
3102          sol = res[0] 
3103          a = sol 
3104          def f(x): 
3105              return a*(x+self.h)**2+self.k

3106          ys_fit = f(xs) 
3107          return args(ys_fit=ys_fit, pars_fit=a, info=res,
 
3108                            results=args(peak=(self.h, self.k),
 
3109                                         f=f))

3110  
 


3111 -class fit_cubic(fit_function):


3112      """Fit a cubic function y=a*x^3+b*x^2+c*x+d to the (x,y) array data.
 
3113      If initial parameter values = (a,b,c,d) are not given, the values
 
3114      (1,1,1,0) will be used.
 
3115  
 
3116      result.f is the fitted function (accepts x values).
 
3117      """ 
3118  
 


3119 -    def fn(self, x, a, b, c,d):


3120          return a*x**3+b*x*x+c*x+d

3121  
 


3122 -    def fit(self, xs, ys, pars_ic=None, opts=None):


3123          if pars_ic is None: 
3124              if self.pars_ic is None: 
3125                  pars_ic = array([1.,1.,1.,0.]) 
3126              else: 
3127                  pars_ic = self.pars_ic 
3128  
 
3129          res = self._do_fit(None, xs, ys, pars_ic) 
3130          sol = res[0] 
3131          a,b,c,d = sol 
3132          def f(x): 
3133              return a*x**3+b*x*x+c*x+d

3134          ys_fit = f(xs) 
3135          return args(ys_fit=ys_fit, pars_fit=(a,b,c,d), info=res,
 
3136                            results=args(f=f))

3137  
 
3138  
 
3139  
 


3140 -class fit_exponential(fit_function):


3141      """Fit an exponential function y=a*exp(b*x) to the (x,y) array data.
 
3142      If initial parameter values = (a,b) are not given, the values
 
3143      (1,-1) will be used.
 
3144  
 
3145      result.f is the fitted function (accepts x values).
 
3146      """ 
3147  
 


3148 -    def fn(self, x, a, b):


3149          return a*exp(b*x)

3150  
 


3151 -    def fit(self, xs, ys, pars_ic=None, opts=None):


3152          if pars_ic is None: 
3153              if self.pars_ic is None: 
3154                  pars_ic = array([1.,-1.]) 
3155              else: 
3156                  pars_ic = self.pars_ic 
3157  
 
3158          res = self._do_fit(None, xs, ys, pars_ic) 
3159          sol = res[0] 
3160          a,b = sol 
3161          def f(x): 
3162              return a*exp(b*x)

3163          ys_fit = f(xs) 
3164          return args(ys_fit=ys_fit, pars_fit=(a,b), info=res,
 
3165                            results=args(f=f))

3166  
 
3167  
 


3168 -class fit_diff_of_exp(fit_function):


3169      """Fit a 'difference of two exponentials' function
 
3170      y = k*a*b*(exp(-a*x)-exp(-b*x))/(b-a) to the (x,y) array data.
 
3171      If initial parameter values = (k,a,b) are not given, the values
 
3172      (1,1,1) will be used (where the function degenerates to
 
3173      y = k*a*a*x*exp(-a*x).
 
3174  
 
3175      Optional use_xoff feature adds offset to x, so that
 
3176      y = k*a*a*(x+xoff)*exp(-a*(x+xoff))     (yes, "+ xoff")
 
3177      etc., in case fitting data that starts at larger values than its tail.
 
3178      Then initial parameter values will be (1,1,1,0) unless given otherwise.
 
3179  
 
3180      If peak_constraint option is used, it is a tuple of values (x_index,
 
3181      y_value, weight_x, weight_y) for the approximate position of a turning point
 
3182      in the data, then this will be used as a soft constraint in the fit.
 
3183  
 
3184      result.peak_pos is a (xpeak, ypeak) pair.
 
3185      result.f is the fitted function (accepts x values).
 
3186      """ 
3187  
 


3188 -    def fn(self, x, k, a, b, xoff=0):


3189          if a==b: 
3190              # classic "alpha" function
 
3191              return k*a*a*((x+xoff)*exp(-a*(x+xoff)) - xoff*exp(-a*xoff)) 
3192          else: 
3193              return k*a*b*(exp(-a*(x+xoff))+exp(-a*xoff)-exp(-b*(x+xoff))-exp(-b*xoff))/(b-a)

3194  
 


3195 -    def fit(self, xs, ys, pars_ic=None, opts=None):


3196          try: 
3197              peak_constraint = opts.peak_constraint 
3198          except AttributeError: 
3199              peak_constraint = None 
3200          try: 
3201              use_xoff = opts.use_xoff 
3202          except AttributeError: 
3203              use_xoff = False 
3204  
 
3205          def peak_pos(k, a, b, xoff=0): 
3206              if a==b: 
3207                  return 1./a - xoff 
3208              else: 
3209                  return ((b-a)*xoff+log(a/b))/(a-b)

3210  
 
3211          if pars_ic is None: 
3212              if self.pars_ic is None: 
3213                  if use_xoff: 
3214                      pars_ic = array([1.,1.,1.,0.]) 
3215                  else: 
3216                      pars_ic = array([1.,1.,1.]) 
3217              else: 
3218                  pars_ic = self.pars_ic 
3219                  if (len(self.pars_ic) == 4 and not use_xoff) or \
 
3220                     (len(self.pars_ic) == 3 and use_xoff): 
3221                      raise ValueError("Inconsistent use_xoff setting with pars_ic") 
3222  
 
3223          if peak_constraint is None: 
3224              constraint = None 
3225          else: 
3226              x_index, y_value, weight_x, weight_y = peak_constraint 
3227              def constraint(k,a,b,xoff=0): 
3228                  return array([weight_y*(self.fn(xs[x_index],k,a,b,xoff)-y_value),
 
3229                                weight_x*(xs[x_index]-peak_pos(k,a,b,xoff))])

3230          res = self._do_fit(constraint, xs, ys, pars_ic) 
3231          sol = res[0] 
3232          if use_xoff: 
3233              k,a,b,xoff = sol 
3234          else: 
3235              k,a,b = sol 
3236              xoff = 0 
3237          if xoff == 0: 
3238              if a == b: 
3239                  # exceptional case
 
3240                  def f(x): 
3241                      return k*a*a*x*exp(-a*x) 
3242              else: 
3243                  def f(x): 
3244                      return k*a*b*(exp(-a*x)-exp(-b*x))/(b-a) 
3245          else: 
3246              if a == b: 
3247                  # exceptional case
 
3248                  def f(x): 
3249                      return k*a*a*((x+xoff)*exp(-a*(x+xoff)) - xoff*exp(-a*xoff)) 
3250              else: 
3251                  def f(x): 
3252                      return k*a*b*(exp(-a*(x+xoff))+exp(-a*xoff)-exp(-b*(x+xoff))-exp(-b*xoff))/(b-a) 
3253          ys_fit = f(xs) 
3254          xpeak = peak_pos(k,a,b,xoff) 
3255          ypeak = f(xpeak) 
3256          if use_xoff: 
3257              pars_fit = (k, a, b, xoff) 
3258          else: 
3259              pars_fit = (k, a, b) 
3260          return args(ys_fit=ys_fit, pars_fit=pars_fit, info=res,
 
3261                            results=args(peak=(xpeak, ypeak),
 
3262                                         f=f)) 
3263  
 


3264 -class fit_linear(fit_function):


3265      """Fit a linear function y=a*x+b to the (x,y) array data.
 
3266      If initial parameter values = (a,b) are not given, the values
 
3267      (1,0) will be used.
 
3268  
 
3269      result.f is the fitted function (accepts x values).
 
3270      """ 
3271  
 


3272 -    def fn(self, x, a, b):


3273          return a*x+b

3274  
 


3275 -    def fit(self, xs, ys, pars_ic=None, opts=None):


3276          if pars_ic is None: 
3277              if self.pars_ic is None: 
3278                  pars_ic = array([1.,0.]) 
3279              else: 
3280                  pars_ic = self.pars_ic 
3281  
 
3282          res = self._do_fit(None, xs, ys, pars_ic) 
3283          sol = res[0] 
3284          a,b = sol 
3285          def f(x): 
3286              return a*x+b

3287          ys_fit = f(xs) 
3288          return args(ys_fit=ys_fit, pars_fit=(a,b), info=res,
 
3289                            results=args(f=f))

3290  
 


3291 -def make_poly_interpolated_curve(pts, coord, model):


3292      """Only for a 1D curve from a Model object (that has an associated
 
3293      vector field for defining 1st derivative of curve).
 
3294      """ 
3295      coord_ix = pts.coordnames.index(coord) 
3296      x = pts[coord] 
3297      t = pts.indepvararray 
3298      p = model.query('pars') 
3299      dx = array([model.Rhs(tval, pts[tix], p, asarray=True)[coord_ix] for \
 
3300                  tix, tval in enumerate(t)]) 
3301      return PiecewisePolynomial(t, array([x, dx]).T, 2)

3302  
 


3303 -def smooth_pts(t, x, q=None):


3304      """Use a local quadratic fit on a set of nearby 1D points and obtain
 
3305      a function that represents that fit in that neighbourhood. Returns a
 
3306      structure (args object) with attributes ys_fit, pars_fit, info, and
 
3307      results. The function can be referenced as results.f
 
3308  
 
3309      Assumed that pts is small enough that it is either purely concave up or
 
3310      down but that at it contains at least five points.
 
3311  
 
3312      If this function is used repeatedly, pass a fit_quadratic instance
 
3313      as the argument q
 
3314      """ 
3315      ## Uncomment verbose-related statements for debugging
 
3316  #    verbose = True
 
3317      if q is None: 
3318          q = fit_quadratic(verbose=False)  # verbose=verbose 
3319      ixlo = 0 
3320      ixhi = len(t)-1 
3321      assert ixhi >= 4, "Provide at least five points" 
3322      # concavity assumed to be simple: whether midpoint of x
 
3323      # is above or below the chord between the endpoints
 
3324      midpoint_ix = int(ixhi/2.) 
3325      midpoint_chord = x[0]+(t[midpoint_ix]-t[0])*(x[-1]-x[0])/(t[-1]-t[0]) 
3326      midpoint_x = x[midpoint_ix] 
3327      # a_sign is -1 if concave down
 
3328      a_sign = sign(midpoint_chord - midpoint_x) 
3329      ixmax = argmax(x) 
3330      ixmin = argmin(x) 
3331      # to estimate |a| need to know where best to put centre for
 
3332      # central second difference formula:
 
3333      # if extremum not at endpoints then use one endpoint
 
3334      # else use central point
 
3335      if (ixmax in (ixhi, ixlo) and a_sign == -1) or \
 
3336         (ixmin in (ixhi, ixlo) and a_sign == 1): 
3337          # use central point, guaranteed to be at least 2 indices away from
 
3338          # ends
 
3339          ix_cent = midpoint_ix 
3340      else: 
3341          # use an endpoint + 1
 
3342          ix_cent = ixlo+2 
3343      # use mean of right and left t steps as h (should be safe for
 
3344      # smooth enough data)
 
3345      h = 0.25*(t[ix_cent+2]-t[ix_cent-2]) 
3346      second_diff = (-x[ix_cent-2]+16*x[ix_cent-1]-30*x[ix_cent]+\
 
3347                     +16*x[ix_cent+1]-x[ix_cent+2])/(12*h**2) 
3348      assert sign(second_diff) == a_sign, "Data insufficiently smooth" 
3349      # a_est based on second deriv of quadratic formula = 2a
 
3350      a_est = second_diff/2. 
3351      if a_sign == -1: 
3352          extreme_x = x[ixmin] 
3353          extreme_t = t[ixmin] 
3354      else: 
3355          extreme_x = x[ixmax] 
3356          extreme_t = t[ixmax] 
3357      # using vertex form of quadratic, x = a*( t-extreme_t )^2 + extreme_x
 
3358      # then in regular formula x = at^2 + bt + c used by quadratic fit class,
 
3359      # b = -2*extreme_t, and c = a*extreme_t^2 + extreme_x
 
3360      b_est = -2*extreme_t 
3361      c_est = a_est*extreme_t**2 + extreme_x 
3362      return q.fit(t, x, pars_ic=(a_est,b_est,c_est))

3363      # for debugging, set res = q.fit() and then return it after the following...
 
3364  #    if verbose:
 
3365  #        print "h =", h
 
3366  #        print "a_est =", a_est, "b_est =", b_est, "c_est =", c_est
 
3367  #        print "extremum estimate at (%f,%f)"%(extreme_t,extreme_x)
 
3368  #        plot(t, x, 'go-')
 
3369  #        tval, xval = res.results.peak
 
3370  #        plot(tval, xval, 'rx')
 
3371  #        xs_fit = res.ys_fit
 
3372  #        plot(t, xs_fit, 'k:')
 
3373  
 


3374 -def nearest_2n_indices(x, i, n):


3375      """Calculates the nearest 2n indices centred at i in an array x, or as close
 
3376      as possible to i, taking into account that i might be within n indices of
 
3377      an endpoint of x.
 
3378  
 
3379      The function returns the limiting indices as a pair, and always returns
 
3380      an interval that contains 2n+1 indices, assuming x is long enough.
 
3381  
 
3382      I.e., away from endpoints, the function returns (i-n, i+n).
 
3383      If i is within n of index 0, the function returns (0, 2n).
 
3384      If i is within n of last index L, the function returns (L-2n, L).
 
3385  
 
3386      Remember to add one to the upper limit if using it in a slice.
 
3387      """ 
3388      assert len(x) > 2*n, "x is not long enough" 
3389      # ixlo = 0
 
3390      ixhi = len(x)-1 
3391      if i < n: 
3392          # too close to low end
 
3393          return (0, 2*n) 
3394      elif i > ixhi - n: 
3395          # too close to high end
 
3396          return (ixhi-2*n, ixhi) 
3397      else: 
3398          return (i-n, i+n)

3399  
 
3400  
 
3401  # --------------------------------------------------------------------
 
3402  
 


3403 -class DomainType(object):


3404 -    def __init__(self, name):


3405          self.name = name

3406  
 


3407 -    def __eq__(self, other):


3408          try: 
3409              return self.name == other.name 
3410          except: 
3411              return False

3412  
 


3413 -    def __ne__(self, other):


3414          try: 
3415              return self.name != other.name 
3416          except: 
3417              return False

3418  
 


3419 -    def __repr__(self):


3420          return self.name

3421  
 
3422      __str__ = __repr__

3423  
 
3424  # treat these as "constants" as they are empty
 
3425  global Continuous, Discrete 
3426  
 
3427  Continuous = DomainType("Continuous Domain") 
3428  Discrete = DomainType("Discrete Domain") 
3429  
 
3430  
 
3431  #-----------------------------------------------------------------------------
 
3432  # The following code, in particular the Verbose class, was written by
 
3433  # John D. Hunter as part of the front end of MatplotLib.
 
3434  # (See matplotlib.sourceforge.net/license.html for details.)
 
3435  #
 
3436  # Copyright (c) 2002-2004 John D. Hunter; All Rights Reserved
 
3437  #-----------------------------------------------------------------------------
 
3438  
 
3439  # This is not yet used in PyDSTool
 


3440 -class Verbose(object):


3441      """
 
3442      A class to handle reporting.  Set the fileo attribute to any file
 
3443      instance to handle the output.  Default is sys.stdout
 
3444      """ 
3445      levels = ('silent', 'error', 'helpful', 'debug', 'debug-annoying') 
3446      vald = dict( [(level, i) for i,level in enumerate(levels)]) 
3447  
 
3448      # parse the verbosity from the command line; flags look like
 
3449      # --verbose-error or --verbose-helpful
 
3450      _commandLineVerbose = None 
3451  
 
3452  
 
3453      for arg in sys.argv[1:]: 
3454          if not arg.startswith('--verbose-'): continue 
3455          _commandLineVerbose = arg[10:] 
3456  
 


3457 -    def __init__(self, level):


3458          self.setLevel(level) 
3459          self.fileo = sys.stdout 
3460          self.erro = sys.stderr

3461  
 


3462 -    def setLevel(self, level):


3463          'set the verbosity to one of the Verbose.levels strings' 
3464  
 
3465          if self._commandLineVerbose is not None: 
3466              level = self._commandLineVerbose 
3467          if level not in self.levels: 
3468              raise ValueError('Illegal verbose string "%s".  Legal values are %s'%(level, self.levels)) 
3469          self.level = level

3470  
 


3471 -    def report(self, s, level='helpful'):


3472          """
 
3473          print message s to self.fileo if self.level>=level.  Return
 
3474          value indicates whether a message was issue.
 
3475          """ 
3476          if self.ge(level): 
3477              print >>self.fileo, s 
3478              return True 
3479          return False

3480  
 


3481 -    def report_error(self, s):


3482          """
 
3483          print message s to self.fileo if self.level>=level.  Return
 
3484          value indicates whether a message was issued
 
3485          """ 
3486          if self.ge('error'): 
3487              print >>self.erro, s 
3488              return True 
3489          return False

3490  
 
3491  
 


3492 -    def wrap(self, fmt, func, level='helpful', always=True):


3493          """
 
3494          return a callable function that wraps func and reports it
 
3495          output through the verbose handler if current verbosity level
 
3496          is higher than level
 
3497  
 
3498          if always is True, the report will occur on every function
 
3499          call; otherwise only on the first time the function is called
 
3500          """ 
3501          assert callable(func) 
3502          def wrapper(*args, **kwargs): 
3503              ret = func(*args, **kwargs) 
3504  
 
3505              if (always or not wrapper._spoke): 
3506                  spoke = self.report(fmt%ret, level) 
3507                  if not wrapper._spoke: wrapper._spoke = spoke 
3508              return ret

3509          wrapper._spoke = False 
3510          wrapper.__doc__ = func.__doc__ 
3511          return wrapper

3512  
 


3513 -    def ge(self, level):


3514          'return true if self.level is >= level' 
3515          return self.vald[self.level]>=self.vald[level]

3516
```

  


| Home | Trees | Indices | Help | | PyDSTool | | --- | |
| --- | --- | --- | --- | --- | --- |

|  |  |
| --- | --- |
| Generated by Epydoc 3.0.1 on Fri May 4 15:24:12 2012 | http://epydoc.sourceforge.net |
