# Supplementary material for: Hybrid Models and Biological Model Reduction with PyDSTool
Source: PLoS Comput Biol. 2012 Aug 9;8(8):e1002628. doi: 10.1371/journal.pcbi.1002628 (PMC3415397; doi:10.1371/journal.pcbi.1002628)
Supplement: Text S4 — Complete source code for the PyDSTool package (version 0.88.120504). Includes API documentation and help files linking to web pages. This file is identical to the current public release on Sourceforge.net. (ZIP) [file pcbi.1002628.s004.zip › PyDSTool/html/PyDSTool.common.args-class.html]

xml version="1.0" encoding="ascii"?


PyDSTool.common.args


| Home | Trees | Indices | Help | | PyDSTool | | --- | |
| --- | --- | --- | --- | --- | --- |

|  |  |  |  |
| --- | --- | --- | --- |
| Package PyDSTool :: Module common :: Class args | |  | | --- | | [hide private] | | [frames] | no frames] | |

# Class args

source code

```
object --+
         |
        args
```

Known Subclasses:
:   - ModelConstructor'.Descriptor
    - , PyCont.Plotting.pargs

---

Mapping object class for building arguments for class initialization
calls. Treat as a dictionary.


|  |  |  |  |
| --- | --- | --- | --- |
| |  |  | | --- | --- | | Instance Methods | [hide private] | | |
|  | |  |  | | --- | --- | | \_\_init\_\_(self, \*\*kw)  x.\_\_init\_\_(...) initializes x; see x.\_\_class\_\_.\_\_doc\_\_ for signature | source code | |
|  | |  |  | | --- | --- | | \_infostr(self, verbose=1, attributeTitle=`'``args``'`) | source code | |
|  | |  |  | | --- | --- | | \_\_repr\_\_(self)  str(x) | source code | |
|  | |  |  | | --- | --- | | info(self) | source code | |
|  | |  |  | | --- | --- | | \_\_str\_\_(self)  str(x) | source code | |
|  | |  |  | | --- | --- | | values(self) | source code | |
|  | |  |  | | --- | --- | | keys(self) | source code | |
|  | |  |  | | --- | --- | | items(self) | source code | |
|  | |  |  | | --- | --- | | itervalues(self) | source code | |
|  | |  |  | | --- | --- | | iterkeys(self) | source code | |
|  | |  |  | | --- | --- | | iteritems(self) | source code | |
|  | |  |  | | --- | --- | | \_\_getitem\_\_(self, k) | source code | |
|  | |  |  | | --- | --- | | \_\_setitem\_\_(self, k, v) | source code | |
|  | |  |  | | --- | --- | | update(self, d) | source code | |
|  | |  |  | | --- | --- | | copy(self) | source code | |
|  | |  |  | | --- | --- | | clear(self) | source code | |
|  | |  |  | | --- | --- | | get(self, k, d=None) | source code | |
|  | |  |  | | --- | --- | | has\_key(self, k) | source code | |
|  | |  |  | | --- | --- | | pop(self, k, d=None) | source code | |
|  | |  |  | | --- | --- | | popitem(self) | source code | |
|  | |  |  | | --- | --- | | \_\_contains\_\_(self, v) | source code | |
|  | |  |  | | --- | --- | | fromkeys(self, S, v=None) | source code | |
|  | |  |  | | --- | --- | | setdefault(self, d) | source code | |
|  | |  |  | | --- | --- | | \_\_delitem\_\_(self, k) | source code | |
|  | |  |  | | --- | --- | | \_\_cmp\_\_(self, other) | source code | |
|  | |  |  | | --- | --- | | \_\_eq\_\_(self, other) | source code | |
|  | |  |  | | --- | --- | | \_\_ne\_\_(self, other) | source code | |
|  | |  |  | | --- | --- | | \_\_gt\_\_(self, other) | source code | |
|  | |  |  | | --- | --- | | \_\_ge\_\_(self, other) | source code | |
|  | |  |  | | --- | --- | | \_\_lt\_\_(self, other) | source code | |
|  | |  |  | | --- | --- | | \_\_le\_\_(self, other) | source code | |
|  | |  |  | | --- | --- | | \_\_len\_\_(self) | source code | |
|  | |  |  | | --- | --- | | \_\_iter\_\_(self) | source code | |
|  | |  |  | | --- | --- | | \_\_add\_\_(self, other) | source code | |
| **Inherited from `object`**: `__delattr__`, `__getattribute__`, `__hash__`, `__new__`, `__reduce__`, `__reduce_ex__`, `__setattr__` | |


|  |  |  |  |
| --- | --- | --- | --- |
| |  |  | | --- | --- | | Properties | [hide private] | | |
| **Inherited from `object`**: `__class__` | |


|  |  |  |  |
| --- | --- | --- | --- |
| |  |  | | --- | --- | | Method Details | [hide private] | | |

|  |  |  |
| --- | --- | --- |
| |  |  | | --- | --- | | \_\_init\_\_(self, \*\*kw)  *(Constructor)* | source code |   x.\_\_init\_\_(...) initializes x; see x.\_\_class\_\_.\_\_doc\_\_ for signature  Overrides: object.\_\_init\_\_ *(inherited documentation)* |

|  |  |  |
| --- | --- | --- |
| |  |  | | --- | --- | | \_\_repr\_\_(self)  *(Representation operator)* | source code |   str(x)  Overrides: object.\_\_repr\_\_ *(inherited documentation)* |

|  |  |  |
| --- | --- | --- |
| |  |  | | --- | --- | | \_\_str\_\_(self)  *(Informal representation operator)* | source code |   str(x)  Overrides: object.\_\_str\_\_ *(inherited documentation)* |

  


| Home | Trees | Indices | Help | | PyDSTool | | --- | |
| --- | --- | --- | --- | --- | --- |

|  |  |
| --- | --- |
| Generated by Epydoc 3.0.1 on Fri May 4 15:24:10 2012 | http://epydoc.sourceforge.net |
