# Supplementary material for: Hybrid Models and Biological Model Reduction with PyDSTool
Source: PLoS Comput Biol. 2012 Aug 9;8(8):e1002628. doi: 10.1371/journal.pcbi.1002628 (PMC3415397; doi:10.1371/journal.pcbi.1002628)
Supplement: Text S4 — Complete source code for the PyDSTool package (version 0.88.120504). Includes API documentation and help files linking to web pages. This file is identical to the current public release on Sourceforge.net. (ZIP) [file pcbi.1002628.s004.zip › PyDSTool/html/PyDSTool.common.BarycentricInterpolator-class.html]

xml version="1.0" encoding="ascii"?


PyDSTool.common.BarycentricInterpolator


| Home | Trees | Indices | Help | | PyDSTool | | --- | |
| --- | --- | --- | --- | --- | --- |

|  |  |  |  |
| --- | --- | --- | --- |
| Package PyDSTool :: Module common :: Class BarycentricInterpolator | |  | | --- | | [hide private] | | [frames] | no frames] | |

# Class BarycentricInterpolator

source code

```
object --+
         |
        BarycentricInterpolator
```

---

The interpolating polynomial for a set of points

Constructs a polynomial that passes through a given set of points.
Allows evaluation of the polynomial, efficient changing of the y values
to be interpolated, and updating by adding more x values. For reasons of
numerical stability, this function does not compute the coefficients of
the polynomial.

This class uses a "barycentric interpolation" method that
treats the problem as a special case of rational function interpolation.
This algorithm is quite stable, numerically, but even in a world of exact
computation, unless the x coordinates are chosen very carefully -
Chebyshev zeros (e.g. cos(i\*pi/n)) are a good choice - polynomial
interpolation itself is a very ill-conditioned process due to the Runge
phenomenon.

Based on Berrut and Trefethen 2004, "Barycentric Lagrange
Interpolation".


|  |  |  |  |
| --- | --- | --- | --- |
| |  |  | | --- | --- | | Instance Methods | [hide private] | | |
|  | |  |  | | --- | --- | | \_\_init\_\_(self, xi, yi=None)  Construct an object capable of interpolating functions sampled at xi | source code | |
|  | |  |  | | --- | --- | | set\_yi(self, yi)  Update the y values to be interpolated | source code | |
|  | |  |  | | --- | --- | | add\_xi(self, xi, yi=None)  Add more x values to the set to be interpolated | source code | |
|  | |  |  | | --- | --- | | \_\_call\_\_(self, x)  Evaluate the interpolating polynomial at the points x | source code | |
| **Inherited from `object`**: `__delattr__`, `__getattribute__`, `__hash__`, `__new__`, `__reduce__`, `__reduce_ex__`, `__repr__`, `__setattr__`, `__str__` | |


|  |  |  |  |
| --- | --- | --- | --- |
| |  |  | | --- | --- | | Properties | [hide private] | | |
| **Inherited from `object`**: `__class__` | |


|  |  |  |  |
| --- | --- | --- | --- |
| |  |  | | --- | --- | | Method Details | [hide private] | | |

|  |  |  |
| --- | --- | --- |
| |  |  | | --- | --- | | \_\_init\_\_(self, xi, yi=None)  *(Constructor)* | source code |  ``` Construct an object capable of interpolating functions sampled at xi  The values yi need to be provided before the function is evaluated, but none of the preprocessing depends on them, so rapid updates are possible.  Parameters ---------- xi : array-like of length N     The x coordinates of the points the polynomial should pass through yi : array-like N by R or None     The y coordinates of the points the polynomial should pass through;     if R>1 the polynomial is vector-valued. If None the y values     will be supplied later. ```   Overrides: object.\_\_init\_\_ |

|  |  |  |
| --- | --- | --- |
| |  |  | | --- | --- | | set\_yi(self, yi) | source code |  ``` Update the y values to be interpolated  The barycentric interpolation algorithm requires the calculation of weights, but these depend only on the xi. The yi can be changed at any time.  Parameters ---------- yi : array-like N by R     The y coordinates of the points the polynomial should pass through;     if R>1 the polynomial is vector-valued. If None the y values     will be supplied later. ``` |

|  |  |  |
| --- | --- | --- |
| |  |  | | --- | --- | | add\_xi(self, xi, yi=None) | source code |  ``` Add more x values to the set to be interpolated  The barycentric interpolation algorithm allows easy updating by adding more points for the polynomial to pass through.  Parameters ---------- xi : array-like of length N1     The x coordinates of the points the polynomial should pass through yi : array-like N1 by R or None     The y coordinates of the points the polynomial should pass through;     if R>1 the polynomial is vector-valued. If None the y values     will be supplied later. The yi should be specified if and only if     the interpolator has y values specified. ``` |

|  |  |  |
| --- | --- | --- |
| |  |  | | --- | --- | | \_\_call\_\_(self, x)  *(Call operator)* | source code |  ``` Evaluate the interpolating polynomial at the points x  Parameters ---------- x : scalar or array-like of length M  Returns ------- y : scalar or array-like of length R or length M or M by R     The shape of y depends on the shape of x and whether the     interpolator is vector-valued or scalar-valued.  Notes ----- Currently the code computes an outer product between x and the weights, that is, it constructs an intermediate array of size N by M, where N is the degree of the polynomial. ``` |

  


| Home | Trees | Indices | Help | | PyDSTool | | --- | |
| --- | --- | --- | --- | --- | --- |

|  |  |
| --- | --- |
| Generated by Epydoc 3.0.1 on Fri May 4 15:24:10 2012 | http://epydoc.sourceforge.net |
