# Supplementary material for: Hybrid Models and Biological Model Reduction with PyDSTool
Source: PLoS Comput Biol. 2012 Aug 9;8(8):e1002628. doi: 10.1371/journal.pcbi.1002628 (PMC3415397; doi:10.1371/journal.pcbi.1002628)
Supplement: Text S4 — Complete source code for the PyDSTool package (version 0.88.120504). Includes API documentation and help files linking to web pages. This file is identical to the current public release on Sourceforge.net. (ZIP) [file pcbi.1002628.s004.zip › PyDSTool/html/PyDSTool.common.DefaultDict-class.html]

xml version="1.0" encoding="ascii"?


PyDSTool.common.DefaultDict


| Home | Trees | Indices | Help | | PyDSTool | | --- | |
| --- | --- | --- | --- | --- | --- |

|  |  |  |  |
| --- | --- | --- | --- |
| Package PyDSTool :: Module common :: Class DefaultDict | |  | | --- | | [hide private] | | [frames] | no frames] | |

# Class DefaultDict

source code

```
object --+    
         |    
      dict --+
             |
            DefaultDict
```

---

Dictionary with a default value for unknown keys.

Written by Peter Norvig.


|  |  |  |  |
| --- | --- | --- | --- |
| |  |  | | --- | --- | | Instance Methods | [hide private] | | |
| ``` new empty dictionary ``` | |  |  | | --- | --- | | \_\_init\_\_(self, default)  x.\_\_init\_\_(...) initializes x; see x.\_\_class\_\_.\_\_doc\_\_ for signature | source code | |
|  | |  |  | | --- | --- | | \_\_getitem\_\_(self, key)  x[y] | source code | |
| **Inherited from `dict`**: `__cmp__`, `__contains__`, `__delitem__`, `__eq__`, `__ge__`, `__getattribute__`, `__gt__`, `__hash__`, `__iter__`, `__le__`, `__len__`, `__lt__`, `__ne__`, `__new__`, `__repr__`, `__setitem__`, `clear`, `copy`, `fromkeys`, `get`, `has_key`, `items`, `iteritems`, `iterkeys`, `itervalues`, `keys`, `pop`, `popitem`, `setdefault`, `update`, `values`  **Inherited from `object`**: `__delattr__`, `__reduce__`, `__reduce_ex__`, `__setattr__`, `__str__` | |


|  |  |  |  |
| --- | --- | --- | --- |
| |  |  | | --- | --- | | Properties | [hide private] | | |
| **Inherited from `object`**: `__class__` | |


|  |  |  |  |
| --- | --- | --- | --- |
| |  |  | | --- | --- | | Method Details | [hide private] | | |

|  |  |  |
| --- | --- | --- |
| |  |  | | --- | --- | | \_\_init\_\_(self, default)  *(Constructor)* | source code |   x.\_\_init\_\_(...) initializes x; see x.\_\_class\_\_.\_\_doc\_\_ for signature  Returns: ``` new empty dictionary ```  Overrides: object.\_\_init\_\_ *(inherited documentation)* |

|  |  |  |
| --- | --- | --- |
| |  |  | | --- | --- | | \_\_getitem\_\_(self, key)  *(Indexing operator)* | source code |   x[y]  Overrides: dict.\_\_getitem\_\_ *(inherited documentation)* |

  


| Home | Trees | Indices | Help | | PyDSTool | | --- | |
| --- | --- | --- | --- | --- | --- |

|  |  |
| --- | --- |
| Generated by Epydoc 3.0.1 on Fri May 4 15:24:10 2012 | http://epydoc.sourceforge.net |
