# Supplementary material for: Hybrid Models and Biological Model Reduction with PyDSTool
Source: PLoS Comput Biol. 2012 Aug 9;8(8):e1002628. doi: 10.1371/journal.pcbi.1002628 (PMC3415397; doi:10.1371/journal.pcbi.1002628)
Supplement: Text S4 — Complete source code for the PyDSTool package (version 0.88.120504). Includes API documentation and help files linking to web pages. This file is identical to the current public release on Sourceforge.net. (ZIP) [file pcbi.1002628.s004.zip › PyDSTool/html/PyDSTool.common.fit_diff_of_exp-class.html]

xml version="1.0" encoding="ascii"?


PyDSTool.common.fit\_diff\_of\_exp


| Home | Trees | Indices | Help | | PyDSTool | | --- | |
| --- | --- | --- | --- | --- | --- |

|  |  |  |  |
| --- | --- | --- | --- |
| Package PyDSTool :: Module common :: Class fit\_diff\_of\_exp | |  | | --- | | [hide private] | | [frames] | no frames] | |

# Class fit\_diff\_of\_exp

source code

```
  object --+    
           |    
fit_function --+
               |
              fit_diff_of_exp
```

---

Fit a 'difference of two exponentials' function y =
k\*a\*b\*(exp(-a\*x)-exp(-b\*x))/(b-a) to the (x,y) array data. If initial
parameter values = (k,a,b) are not given, the values (1,1,1) will be used
(where the function degenerates to y = k\*a\*a\*x\*exp(-a\*x).

Optional use\_xoff feature adds offset to x, so that y =
k\*a\*a\*(x+xoff)\*exp(-a\*(x+xoff)) (yes, "+ xoff") etc., in
case fitting data that starts at larger values than its tail. Then
initial parameter values will be (1,1,1,0) unless given otherwise.

If peak\_constraint option is used, it is a tuple of values (x\_index,
y\_value, weight\_x, weight\_y) for the approximate position of a turning
point in the data, then this will be used as a soft constraint in the
fit.

result.peak\_pos is a (xpeak, ypeak) pair. result.f is the fitted
function (accepts x values).


|  |  |  |  |
| --- | --- | --- | --- |
| |  |  | | --- | --- | | Instance Methods | [hide private] | | |
|  | |  |  | | --- | --- | | fn(self, x, k, a, b, xoff=0) | source code | |
|  | |  |  | | --- | --- | | fit(self, xs, ys, pars\_ic=None, opts=None) | source code | |
| **Inherited from `fit_function`**: `__init__`  **Inherited from `fit_function`** (private): `_do_fit`  **Inherited from `object`**: `__delattr__`, `__getattribute__`, `__hash__`, `__new__`, `__reduce__`, `__reduce_ex__`, `__repr__`, `__setattr__`, `__str__` | |


|  |  |  |  |
| --- | --- | --- | --- |
| |  |  | | --- | --- | | Properties | [hide private] | | |
| **Inherited from `object`**: `__class__` | |


|  |  |  |  |
| --- | --- | --- | --- |
| |  |  | | --- | --- | | Method Details | [hide private] | | |

|  |  |  |
| --- | --- | --- |
| |  |  | | --- | --- | | fn(self, x, k, a, b, xoff=0) | source code |   Overrides: fit\_function.fn |

|  |  |  |
| --- | --- | --- |
| |  |  | | --- | --- | | fit(self, xs, ys, pars\_ic=None, opts=None) | source code |   Overrides: fit\_function.fit |

  


| Home | Trees | Indices | Help | | PyDSTool | | --- | |
| --- | --- | --- | --- | --- | --- |

|  |  |
| --- | --- |
| Generated by Epydoc 3.0.1 on Fri May 4 15:24:10 2012 | http://epydoc.sourceforge.net |
