# Supplementary material for: Hybrid Models and Biological Model Reduction with PyDSTool
Source: PLoS Comput Biol. 2012 Aug 9;8(8):e1002628. doi: 10.1371/journal.pcbi.1002628 (PMC3415397; doi:10.1371/journal.pcbi.1002628)
Supplement: Text S4 — Complete source code for the PyDSTool package (version 0.88.120504). Includes API documentation and help files linking to web pages. This file is identical to the current public release on Sourceforge.net. (ZIP) [file pcbi.1002628.s004.zip › PyDSTool/html/PyDSTool.common.fit_exponential-class.html]

xml version="1.0" encoding="ascii"?


PyDSTool.common.fit\_exponential


| Home | Trees | Indices | Help | | PyDSTool | | --- | |
| --- | --- | --- | --- | --- | --- |

|  |  |  |  |
| --- | --- | --- | --- |
| Package PyDSTool :: Module common :: Class fit\_exponential | |  | | --- | | [hide private] | | [frames] | no frames] | |

# Class fit\_exponential

source code

```
  object --+    
           |    
fit_function --+
               |
              fit_exponential
```

---

Fit an exponential function y=a\*exp(b\*x) to the (x,y) array data. If
initial parameter values = (a,b) are not given, the values (1,-1) will be
used.

result.f is the fitted function (accepts x values).


|  |  |  |  |
| --- | --- | --- | --- |
| |  |  | | --- | --- | | Instance Methods | [hide private] | | |
|  | |  |  | | --- | --- | | fn(self, x, a, b) | source code | |
|  | |  |  | | --- | --- | | fit(self, xs, ys, pars\_ic=None, opts=None) | source code | |
| **Inherited from `fit_function`**: `__init__`  **Inherited from `fit_function`** (private): `_do_fit`  **Inherited from `object`**: `__delattr__`, `__getattribute__`, `__hash__`, `__new__`, `__reduce__`, `__reduce_ex__`, `__repr__`, `__setattr__`, `__str__` | |


|  |  |  |  |
| --- | --- | --- | --- |
| |  |  | | --- | --- | | Properties | [hide private] | | |
| **Inherited from `object`**: `__class__` | |


|  |  |  |  |
| --- | --- | --- | --- |
| |  |  | | --- | --- | | Method Details | [hide private] | | |

|  |  |  |
| --- | --- | --- |
| |  |  | | --- | --- | | fn(self, x, a, b) | source code |   Overrides: fit\_function.fn |

|  |  |  |
| --- | --- | --- |
| |  |  | | --- | --- | | fit(self, xs, ys, pars\_ic=None, opts=None) | source code |   Overrides: fit\_function.fit |

  


| Home | Trees | Indices | Help | | PyDSTool | | --- | |
| --- | --- | --- | --- | --- | --- |

|  |  |
| --- | --- |
| Generated by Epydoc 3.0.1 on Fri May 4 15:24:10 2012 | http://epydoc.sourceforge.net |
