# Supplementary material for: Hybrid Models and Biological Model Reduction with PyDSTool
Source: PLoS Comput Biol. 2012 Aug 9;8(8):e1002628. doi: 10.1371/journal.pcbi.1002628 (PMC3415397; doi:10.1371/journal.pcbi.1002628)
Supplement: Text S4 — Complete source code for the PyDSTool package (version 0.88.120504). Includes API documentation and help files linking to web pages. This file is identical to the current public release on Sourceforge.net. (ZIP) [file pcbi.1002628.s004.zip › PyDSTool/html/PyDSTool.common.fit_function-class.html]

xml version="1.0" encoding="ascii"?


PyDSTool.common.fit\_function


| Home | Trees | Indices | Help | | PyDSTool | | --- | |
| --- | --- | --- | --- | --- | --- |

|  |  |  |  |
| --- | --- | --- | --- |
| Package PyDSTool :: Module common :: Class fit\_function | |  | | --- | | [hide private] | | [frames] | no frames] | |

# Class fit\_function

source code

```
object --+
         |
        fit_function
```

Known Subclasses:
:   - fit\_cubic
    - , fit\_diff\_of\_exp
    - , fit\_exponential
    - , fit\_linear
    - , fit\_quadratic
    - , fit\_quadratic\_at\_vertex

---

```
Abstract super-class for fitting explicit functions to 1D arrays of data
using least squares.

xs -- independent variable data
ys -- dependent variable data
pars_ic -- initial values defining the function

Optional algorithmic parameters to minpack.leastsq can be passed in the
algpars argument: e.g.,
ftol -- Relative error desired in the sum of squares (default 1e-6).
xtol -- Relative error desired in the approximate solution (default 1e-6).
gtol -- Orthogonality desired between the function vector
        and the columns of the Jacobian (default 1e-8).

Other parameters may be used for concrete sub-classes. Pass these as a dict
or args object in the opts argument.

Returns an args object with attributes:

ys_fit --   the fitted y values corresponding to the given x data,
pars_fit -- the function parameters at the fit
info --     diagnostic feedback from the leastsq algorithm
results --  dictionary of other function specific information (such as peak
             position)
```


|  |  |  |  |
| --- | --- | --- | --- |
| |  |  | | --- | --- | | Instance Methods | [hide private] | | |
|  | |  |  | | --- | --- | | \_\_init\_\_(self, pars\_ic=None, algpars=None, opts=None, verbose=False)  x.\_\_init\_\_(...) initializes x; see x.\_\_class\_\_.\_\_doc\_\_ for signature | source code | |
|  | |  |  | | --- | --- | | fn(self, x, \*pars) | source code | |
|  | |  |  | | --- | --- | | \_do\_fit(self, constraint, xs, ys, pars\_ic) | source code | |
|  | |  |  | | --- | --- | | fit(self, xs, ys, pars\_ic=None, opts=None) | source code | |
| **Inherited from `object`**: `__delattr__`, `__getattribute__`, `__hash__`, `__new__`, `__reduce__`, `__reduce_ex__`, `__repr__`, `__setattr__`, `__str__` | |


|  |  |  |  |
| --- | --- | --- | --- |
| |  |  | | --- | --- | | Properties | [hide private] | | |
| **Inherited from `object`**: `__class__` | |


|  |  |  |  |
| --- | --- | --- | --- |
| |  |  | | --- | --- | | Method Details | [hide private] | | |

|  |  |  |
| --- | --- | --- |
| |  |  | | --- | --- | | \_\_init\_\_(self, pars\_ic=None, algpars=None, opts=None, verbose=False)  *(Constructor)* | source code |   x.\_\_init\_\_(...) initializes x; see x.\_\_class\_\_.\_\_doc\_\_ for signature  Overrides: object.\_\_init\_\_ *(inherited documentation)* |

  


| Home | Trees | Indices | Help | | PyDSTool | | --- | |
| --- | --- | --- | --- | --- | --- |

|  |  |
| --- | --- |
| Generated by Epydoc 3.0.1 on Fri May 4 15:24:10 2012 | http://epydoc.sourceforge.net |
