# Supplementary material for: Hybrid Models and Biological Model Reduction with PyDSTool
Source: PLoS Comput Biol. 2012 Aug 9;8(8):e1002628. doi: 10.1371/journal.pcbi.1002628 (PMC3415397; doi:10.1371/journal.pcbi.1002628)
Supplement: Text S4 — Complete source code for the PyDSTool package (version 0.88.120504). Includes API documentation and help files linking to web pages. This file is identical to the current public release on Sourceforge.net. (ZIP) [file pcbi.1002628.s004.zip › PyDSTool/html/PyDSTool.common.fit_quadratic_at_vertex-class.html]

xml version="1.0" encoding="ascii"?


PyDSTool.common.fit\_quadratic\_at\_vertex


| Home | Trees | Indices | Help | | PyDSTool | | --- | |
| --- | --- | --- | --- | --- | --- |

|  |  |  |  |
| --- | --- | --- | --- |
| Package PyDSTool :: Module common :: Class fit\_quadratic\_at\_vertex | |  | | --- | | [hide private] | | [frames] | no frames] | |

# Class fit\_quadratic\_at\_vertex

source code

```
  object --+    
           |    
fit_function --+
               |
              fit_quadratic_at_vertex
```

---

Fit a quadratic function y=a\*(x+h)\*\*2+k to the (x,y) array data,
constrained to have a vertex at (h, k), leaving only the free parameter a
for the curvature. (h, k) is specified through the peak\_constraint option
in the initialization argument 'opts'.

If initial parameter value = a is not given, the value 1 will be
used.

result.peak is a (xpeak, ypeak) pair, but corresponds to (h,k).
result.f is the fitted function (accepts x values).


|  |  |  |  |
| --- | --- | --- | --- |
| |  |  | | --- | --- | | Instance Methods | [hide private] | | |
|  | |  |  | | --- | --- | | fn(self, x, a) | source code | |
|  | |  |  | | --- | --- | | fit(self, xs, ys, pars\_ic=None, opts=None) | source code | |
| **Inherited from `fit_function`**: `__init__`  **Inherited from `fit_function`** (private): `_do_fit`  **Inherited from `object`**: `__delattr__`, `__getattribute__`, `__hash__`, `__new__`, `__reduce__`, `__reduce_ex__`, `__repr__`, `__setattr__`, `__str__` | |


|  |  |  |  |
| --- | --- | --- | --- |
| |  |  | | --- | --- | | Properties | [hide private] | | |
| **Inherited from `object`**: `__class__` | |


|  |  |  |  |
| --- | --- | --- | --- |
| |  |  | | --- | --- | | Method Details | [hide private] | | |

|  |  |  |
| --- | --- | --- |
| |  |  | | --- | --- | | fn(self, x, a) | source code |   Overrides: fit\_function.fn |

|  |  |  |
| --- | --- | --- |
| |  |  | | --- | --- | | fit(self, xs, ys, pars\_ic=None, opts=None) | source code |   Overrides: fit\_function.fit |

  


| Home | Trees | Indices | Help | | PyDSTool | | --- | |
| --- | --- | --- | --- | --- | --- |

|  |  |
| --- | --- |
| Generated by Epydoc 3.0.1 on Fri May 4 15:24:10 2012 | http://epydoc.sourceforge.net |
