# Supplementary material for: Hybrid Models and Biological Model Reduction with PyDSTool
Source: PLoS Comput Biol. 2012 Aug 9;8(8):e1002628. doi: 10.1371/journal.pcbi.1002628 (PMC3415397; doi:10.1371/journal.pcbi.1002628)
Supplement: Text S4 — Complete source code for the PyDSTool package (version 0.88.120504). Includes API documentation and help files linking to web pages. This file is identical to the current public release on Sourceforge.net. (ZIP) [file pcbi.1002628.s004.zip › PyDSTool/html/PyDSTool.common.interp1d-class.html]

xml version="1.0" encoding="ascii"?


PyDSTool.common.interp1d


| Home | Trees | Indices | Help | | PyDSTool | | --- | |
| --- | --- | --- | --- | --- | --- |

|  |  |  |  |
| --- | --- | --- | --- |
| Package PyDSTool :: Module common :: Class interp1d | |  | | --- | | [hide private] | | [frames] | no frames] | |

# Class interp1d

source code

```
 object --+    
          |    
interpclass --+
              |
             interp1d
```

---


|  |  |  |  |
| --- | --- | --- | --- |
| |  |  | | --- | --- | | Instance Methods | [hide private] | | |
|  | |  |  | | --- | --- | | \_\_init\_\_(self, x, y, kind=`'``linear``'`, axis=-1, makecopy=0, bounds\_error=1, fill\_value=None)  Initialize a 1d piecewise-linear interpolation class | source code | |
|  | |  |  | | --- | --- | | \_\_call\_\_(self, x\_new)  Find linearly interpolated y\_new = <name>(x\_new). | source code | |
|  | |  |  | | --- | --- | | \_check\_bounds(self, x\_new) | source code | |
|  | |  |  | | --- | --- | | \_\_getstate\_\_(self) | source code | |
|  | |  |  | | --- | --- | | \_\_setstate\_\_(self, state) | source code | |
| **Inherited from `object`**: `__delattr__`, `__getattribute__`, `__hash__`, `__new__`, `__reduce__`, `__reduce_ex__`, `__repr__`, `__setattr__`, `__str__` | |


|  |  |  |  |
| --- | --- | --- | --- |
| |  |  | | --- | --- | | Class Variables | [hide private] | | |
| **Inherited from `interpclass`**: `interp_axis` | |


|  |  |  |  |
| --- | --- | --- | --- |
| |  |  | | --- | --- | | Properties | [hide private] | | |
| **Inherited from `object`**: `__class__` | |


|  |  |  |  |
| --- | --- | --- | --- |
| |  |  | | --- | --- | | Method Details | [hide private] | | |

|  |  |  |
| --- | --- | --- |
| |  |  | | --- | --- | | \_\_init\_\_(self, x, y, kind=`'``linear``'`, axis=-1, makecopy=0, bounds\_error=1, fill\_value=None)  *(Constructor)* | source code |  ``` Initialize a 1d piecewise-linear interpolation class  Description:   x and y are arrays of values used to approximate some function f:     y = f(x)   This class returns a function whose call method uses linear   interpolation to find the value of new points.  Inputs:     x -- a 1d array of monotonically increasing real values.          x cannot include duplicate values. (otherwise f is          overspecified)     y -- an nd array of real values.  y's length along the          interpolation axis must be equal to the length          of x.     kind -- specify the kind of interpolation: 'nearest', 'linear',             'cubic', or 'spline'     axis -- specifies the axis of y along which to             interpolate. Interpolation defaults to the last             axis of y.  (default: -1)     makecopy -- If 1, the class makes internal copies of x and y.             If 0, references to x and y are used. The default             is NOT to copy. (default: 0)     bounds_error -- If 1, an error is thrown any time interpolation                     is attempted on a value outside of the range                     of x (where extrapolation is necessary).                     If 0, out of bounds values are assigned the                     NaN (#INF) value.  By default, an error is                     raised, although this is prone to change.                     (default: 1) ```   Overrides: object.\_\_init\_\_ |

|  |  |  |
| --- | --- | --- |
| |  |  | | --- | --- | | \_\_call\_\_(self, x\_new)  *(Call operator)* | source code |  ``` Find linearly interpolated y_new = <name>(x_new).  Inputs:   x_new -- New independent variables.  Outputs:   y_new -- Linearly interpolated values corresponding to x_new. ``` |

  


| Home | Trees | Indices | Help | | PyDSTool | | --- | |
| --- | --- | --- | --- | --- | --- |

|  |  |
| --- | --- |
| Generated by Epydoc 3.0.1 on Fri May 4 15:24:10 2012 | http://epydoc.sourceforge.net |
