# Supplementary material for: Hybrid Models and Biological Model Reduction with PyDSTool
Source: PLoS Comput Biol. 2012 Aug 9;8(8):e1002628. doi: 10.1371/journal.pcbi.1002628 (PMC3415397; doi:10.1371/journal.pcbi.1002628)
Supplement: Text S4 — Complete source code for the PyDSTool package (version 0.88.120504). Includes API documentation and help files linking to web pages. This file is identical to the current public release on Sourceforge.net. (ZIP) [file pcbi.1002628.s004.zip › PyDSTool/html/PyDSTool.common.KroghInterpolator-class.html]

xml version="1.0" encoding="ascii"?


PyDSTool.common.KroghInterpolator


| Home | Trees | Indices | Help | | PyDSTool | | --- | |
| --- | --- | --- | --- | --- | --- |

|  |  |  |  |
| --- | --- | --- | --- |
| Package PyDSTool :: Module common :: Class KroghInterpolator | |  | | --- | | [hide private] | | [frames] | no frames] | |

# Class KroghInterpolator

source code

```
object --+
         |
        KroghInterpolator
```

---

The interpolating polynomial for a set of points

Constructs a polynomial that passes through a given set of points,
optionally with specified derivatives at those points. Allows evaluation
of the polynomial and all its derivatives. For reasons of numerical
stability, this function does not compute the coefficients of the
polynomial, although they can be obtained by evaluating all the
derivatives.

Be aware that the algorithms implemented here are not necessarily the
most numerically stable known. Moreover, even in a world of exact
computation, unless the x coordinates are chosen very carefully -
Chebyshev zeros (e.g. cos(i\*pi/n)) are a good choice - polynomial
interpolation itself is a very ill-conditioned process due to the Runge
phenomenon. In general, even with well-chosen x values, degrees higher
than about thirty cause problems with numerical instability in this
code.

Based on Krogh 1970, "Efficient Algorithms for Polynomial
Interpolation and Numerical Differentiation"


|  |  |  |  |
| --- | --- | --- | --- |
| |  |  | | --- | --- | | Instance Methods | [hide private] | | |
|  | |  |  | | --- | --- | | \_\_init\_\_(self, xi, yi)  Construct an interpolator passing through the specified points | source code | |
|  | |  |  | | --- | --- | | \_\_call\_\_(self, x)  Evaluate the polynomial at the point x | source code | |
|  | |  |  | | --- | --- | | derivatives(self, x, der=None)  Evaluate many derivatives of the polynomial at the point x | source code | |
|  | |  |  | | --- | --- | | derivative(self, x, der)  Evaluate one derivative of the polynomial at the point x | source code | |
| **Inherited from `object`**: `__delattr__`, `__getattribute__`, `__hash__`, `__new__`, `__reduce__`, `__reduce_ex__`, `__repr__`, `__setattr__`, `__str__` | |


|  |  |  |  |
| --- | --- | --- | --- |
| |  |  | | --- | --- | | Properties | [hide private] | | |
| **Inherited from `object`**: `__class__` | |


|  |  |  |  |
| --- | --- | --- | --- |
| |  |  | | --- | --- | | Method Details | [hide private] | | |

|  |  |  |
| --- | --- | --- |
| |  |  | | --- | --- | | \_\_init\_\_(self, xi, yi)  *(Constructor)* | source code |  ``` Construct an interpolator passing through the specified points  The polynomial passes through all the pairs (xi,yi). One may additionally specify a number of derivatives at each point xi; this is done by repeating the value xi and specifying the derivatives as successive yi values.  Parameters ---------- xi : array-like, length N     known x-coordinates yi : array-like, N by R     known y-coordinates, interpreted as vectors of length R,     or scalars if R=1  Example ------- To produce a polynomial that is zero at 0 and 1 and has derivative 2 at 0, call  >>> KroghInterpolator([0,0,1],[0,2,0]) ```   Overrides: object.\_\_init\_\_ |

|  |  |  |
| --- | --- | --- |
| |  |  | | --- | --- | | \_\_call\_\_(self, x)  *(Call operator)* | source code |  ``` Evaluate the polynomial at the point x  Parameters ---------- x : scalar or array-like of length N  Returns ------- y : scalar, array of length R, array of length N, or array of length N by R     If x is a scalar, returns either a vector or a scalar depending on     whether the interpolator is vector-valued or scalar-valued.     If x is a vector, returns a vector of values. ``` |

|  |  |  |
| --- | --- | --- |
| |  |  | | --- | --- | | derivatives(self, x, der=None) | source code |  ``` Evaluate many derivatives of the polynomial at the point x  Produce an array of all derivative values at the point x.  Parameters ---------- x : scalar or array-like of length N     Point or points at which to evaluate the derivatives der : None or integer     How many derivatives to extract; None for all potentially     nonzero derivatives (that is a number equal to the number     of points). This number includes the function value as 0th     derivative. Returns ------- d : array     If the interpolator's values are R-dimensional then the     returned array will be der by N by R. If x is a scalar,     the middle dimension will be dropped; if R is 1 then the     last dimension will be dropped.  Example ------- >>> KroghInterpolator([0,0,0],[1,2,3]).derivatives(0) array([1.0,2.0,3.0]) >>> KroghInterpolator([0,0,0],[1,2,3]).derivatives([0,0]) array([[1.0,1.0],        [2.0,2.0],        [3.0,3.0]]) ``` |

|  |  |  |
| --- | --- | --- |
| |  |  | | --- | --- | | derivative(self, x, der) | source code |  ``` Evaluate one derivative of the polynomial at the point x  Parameters ---------- x : scalar or array-like of length N     Point or points at which to evaluate the derivatives der : None or integer     Which derivative to extract. This number includes the     function value as 0th derivative. Returns ------- d : array     If the interpolator's values are R-dimensional then the     returned array will be N by R. If x is a scalar,     the middle dimension will be dropped; if R is 1 then the     last dimension will be dropped.  Notes ----- This is computed by evaluating all derivatives up to the desired one and then discarding the rest. ``` |

  


| Home | Trees | Indices | Help | | PyDSTool | | --- | |
| --- | --- | --- | --- | --- | --- |

|  |  |
| --- | --- |
| Generated by Epydoc 3.0.1 on Fri May 4 15:24:10 2012 | http://epydoc.sourceforge.net |
