# Supplementary material for: Hybrid Models and Biological Model Reduction with PyDSTool
Source: PLoS Comput Biol. 2012 Aug 9;8(8):e1002628. doi: 10.1371/journal.pcbi.1002628 (PMC3415397; doi:10.1371/journal.pcbi.1002628)
Supplement: Text S4 — Complete source code for the PyDSTool package (version 0.88.120504). Includes API documentation and help files linking to web pages. This file is identical to the current public release on Sourceforge.net. (ZIP) [file pcbi.1002628.s004.zip › PyDSTool/html/PyDSTool.common.metric-class.html]

xml version="1.0" encoding="ascii"?


PyDSTool.common.metric


| Home | Trees | Indices | Help | | PyDSTool | | --- | |
| --- | --- | --- | --- | --- | --- |

|  |  |  |  |
| --- | --- | --- | --- |
| Package PyDSTool :: Module common :: Class metric | |  | | --- | | [hide private] | | [frames] | no frames] | |

# Class metric

source code

```
object --+
         |
        metric
```

Known Subclasses:
:   - metric\_L2
    - , metric\_L2\_1D
    - , metric\_float
    - , metric\_float\_1D
    - , metric\_weighted\_L2
    - , metric\_weighted\_deadzone\_L2
    - , Toolbox.neuro\_data.spike\_metric

---

Abstract metric class for quantitatively comparing scalar or vector
quantities. Can include optional explicit Jacobian function.

Create concrete sub-classes for specific applications. Store the
measured (\*1D array only\*) value in self.results for use as part of a
parameter estimation residual value. Residual norm will be taken by
optimizer routines.


|  |  |  |  |
| --- | --- | --- | --- |
| |  |  | | --- | --- | | Instance Methods | [hide private] | | |
|  | |  |  | | --- | --- | | \_\_init\_\_(self)  x.\_\_init\_\_(...) initializes x; see x.\_\_class\_\_.\_\_doc\_\_ for signature | source code | |
|  | |  |  | | --- | --- | | \_\_call\_\_(self, x, y) | source code | |
|  | |  |  | | --- | --- | | Jac(self, x, y) | source code | |
| **Inherited from `object`**: `__delattr__`, `__getattribute__`, `__hash__`, `__new__`, `__reduce__`, `__reduce_ex__`, `__repr__`, `__setattr__`, `__str__` | |


|  |  |  |  |
| --- | --- | --- | --- |
| |  |  | | --- | --- | | Properties | [hide private] | | |
| **Inherited from `object`**: `__class__` | |


|  |  |  |  |
| --- | --- | --- | --- |
| |  |  | | --- | --- | | Method Details | [hide private] | | |

|  |  |  |
| --- | --- | --- |
| |  |  | | --- | --- | | \_\_init\_\_(self)  *(Constructor)* | source code |   x.\_\_init\_\_(...) initializes x; see x.\_\_class\_\_.\_\_doc\_\_ for signature  Overrides: object.\_\_init\_\_ *(inherited documentation)* |

  


| Home | Trees | Indices | Help | | PyDSTool | | --- | |
| --- | --- | --- | --- | --- | --- |

|  |  |
| --- | --- |
| Generated by Epydoc 3.0.1 on Fri May 4 15:24:10 2012 | http://epydoc.sourceforge.net |
