# Supplementary material for: Hybrid Models and Biological Model Reduction with PyDSTool
Source: PLoS Comput Biol. 2012 Aug 9;8(8):e1002628. doi: 10.1371/journal.pcbi.1002628 (PMC3415397; doi:10.1371/journal.pcbi.1002628)
Supplement: Text S4 — Complete source code for the PyDSTool package (version 0.88.120504). Includes API documentation and help files linking to web pages. This file is identical to the current public release on Sourceforge.net. (ZIP) [file pcbi.1002628.s004.zip › PyDSTool/html/PyDSTool.common.metric_weighted_deadzone_L2-class.html]

xml version="1.0" encoding="ascii"?


PyDSTool.common.metric\_weighted\_deadzone\_L2


| Home | Trees | Indices | Help | | PyDSTool | | --- | |
| --- | --- | --- | --- | --- | --- |

|  |  |  |  |
| --- | --- | --- | --- |
| Package PyDSTool :: Module common :: Class metric\_weighted\_deadzone\_L2 | |  | | --- | | [hide private] | | [frames] | no frames] | |

# Class metric\_weighted\_deadzone\_L2

source code

```
object --+    
         |    
    metric --+
             |
            metric_weighted_deadzone_L2
```

---

Measures the standard "distance" between two 1D pointsets or
arrays using the L-2 norm, after weighting by weights attribute. Then,
sets distance vector entries to zero if they fall below corresponding
entries in the deadzone vector/scalar. (Must set weights and deadzone
vectors/scalars after creation, e.g. in a feature's \_local\_init
method).


|  |  |  |  |
| --- | --- | --- | --- |
| |  |  | | --- | --- | | Instance Methods | [hide private] | | |
|  | |  |  | | --- | --- | | \_\_call\_\_(self, pts1, pts2) | source code | |
| **Inherited from `metric`**: `Jac`, `__init__`  **Inherited from `object`**: `__delattr__`, `__getattribute__`, `__hash__`, `__new__`, `__reduce__`, `__reduce_ex__`, `__repr__`, `__setattr__`, `__str__` | |


|  |  |  |  |
| --- | --- | --- | --- |
| |  |  | | --- | --- | | Properties | [hide private] | | |
| **Inherited from `object`**: `__class__` | |


|  |  |  |  |
| --- | --- | --- | --- |
| |  |  | | --- | --- | | Method Details | [hide private] | | |

|  |  |  |
| --- | --- | --- |
| |  |  | | --- | --- | | \_\_call\_\_(self, pts1, pts2)  *(Call operator)* | source code |   Overrides: metric.\_\_call\_\_ |

  


| Home | Trees | Indices | Help | | PyDSTool | | --- | |
| --- | --- | --- | --- | --- | --- |

|  |  |
| --- | --- |
| Generated by Epydoc 3.0.1 on Fri May 4 15:24:10 2012 | http://epydoc.sourceforge.net |
