# Supplementary material for: Hybrid Models and Biological Model Reduction with PyDSTool
Source: PLoS Comput Biol. 2012 Aug 9;8(8):e1002628. doi: 10.1371/journal.pcbi.1002628 (PMC3415397; doi:10.1371/journal.pcbi.1002628)
Supplement: Text S4 — Complete source code for the PyDSTool package (version 0.88.120504). Includes API documentation and help files linking to web pages. This file is identical to the current public release on Sourceforge.net. (ZIP) [file pcbi.1002628.s004.zip › PyDSTool/html/PyDSTool.common.PiecewisePolynomial-class.html]

xml version="1.0" encoding="ascii"?


PyDSTool.common.PiecewisePolynomial


| Home | Trees | Indices | Help | | PyDSTool | | --- | |
| --- | --- | --- | --- | --- | --- |

|  |  |  |  |
| --- | --- | --- | --- |
| Package PyDSTool :: Module common :: Class PiecewisePolynomial | |  | | --- | | [hide private] | | [frames] | no frames] | |

# Class PiecewisePolynomial

source code

```
 object --+    
          |    
interpclass --+
              |
             PiecewisePolynomial
```

---

Piecewise polynomial curve specified by points and derivatives.

This class represents a curve that is a piecewise polynomial. It
passes through a list of points and has specified derivatives at each
point. The degree of the polynomial may very from segment to segment, as
may the number of derivatives available. The degree should not exceed
about thirty.

Appending points to the end of the curve is efficient.


|  |  |  |  |
| --- | --- | --- | --- |
| |  |  | | --- | --- | | Instance Methods | [hide private] | | |
|  | |  |  | | --- | --- | | \_\_init\_\_(self, xi, yi, orders=None, direction=None)  Construct a piecewise polynomial | source code | |
|  | |  |  | | --- | --- | | \_make\_polynomial(self, x1, y1, x2, y2, order, direction)  Construct the interpolating polynomial object | source code | |
|  | |  |  | | --- | --- | | append(self, xi, yi, order=None)  Append a single point with derivatives to the PiecewisePolynomial | source code | |
|  | |  |  | | --- | --- | | extend(self, xi, yi, orders=None)  Extend the PiecewisePolynomial by a list of points | source code | |
|  | |  |  | | --- | --- | | \_\_call\_\_(self, x)  Evaluate the piecewise polynomial | source code | |
|  | |  |  | | --- | --- | | derivative(self, x, der)  Evaluate a derivative of the piecewise polynomial | source code | |
|  | |  |  | | --- | --- | | derivatives(self, x, der)  Evaluate a derivative of the piecewise polynomial | source code | |
|  | |  |  | | --- | --- | | \_\_getstate\_\_(self) | source code | |
|  | |  |  | | --- | --- | | \_\_setstate\_\_(self, state) | source code | |
| **Inherited from `object`**: `__delattr__`, `__getattribute__`, `__hash__`, `__new__`, `__reduce__`, `__reduce_ex__`, `__repr__`, `__setattr__`, `__str__` | |


|  |  |  |  |
| --- | --- | --- | --- |
| |  |  | | --- | --- | | Class Variables | [hide private] | | |
| **Inherited from `interpclass`**: `interp_axis` | |


|  |  |  |  |
| --- | --- | --- | --- |
| |  |  | | --- | --- | | Properties | [hide private] | | |
| **Inherited from `object`**: `__class__` | |


|  |  |  |  |
| --- | --- | --- | --- |
| |  |  | | --- | --- | | Method Details | [hide private] | | |

|  |  |  |
| --- | --- | --- |
| |  |  | | --- | --- | | \_\_init\_\_(self, xi, yi, orders=None, direction=None)  *(Constructor)* | source code |  ``` Construct a piecewise polynomial  Parameters ---------- xi : array-like of length N     a sorted list of x-coordinates yi : list of lists of length N     yi[i] is the list of derivatives known at xi[i] orders : list of integers, or integer     a list of polynomial orders, or a single universal order direction : {None, 1, -1}     indicates whether the xi are increasing or decreasing     +1 indicates increasing     -1 indicates decreasing     None indicates that it should be deduced from the first two xi  Notes ----- If orders is None, or orders[i] is None, then the degree of the polynomial segment is exactly the degree required to match all i available derivatives at both endpoints. If orders[i] is not None, then some derivatives will be ignored. The code will try to use an equal number of derivatives from each end; if the total number of derivatives needed is odd, it will prefer the rightmost endpoint. If not enough derivatives are available, an exception is raised. ```   Overrides: object.\_\_init\_\_ |

|  |  |  |
| --- | --- | --- |
| |  |  | | --- | --- | | \_make\_polynomial(self, x1, y1, x2, y2, order, direction) | source code |   Construct the interpolating polynomial object  Deduces the number of derivatives to match at each end from order and the number of derivatives available. If possible it uses the same number of derivatives from each end; if the number is odd it tries to take the extra one from y2. In any case if not enough derivatives are available at one end or another it draws enough to make up the total from the other end. |

|  |  |  |
| --- | --- | --- |
| |  |  | | --- | --- | | append(self, xi, yi, order=None) | source code |  ``` Append a single point with derivatives to the PiecewisePolynomial  Parameters ---------- xi : float yi : array-like     yi is the list of derivatives known at xi order : integer or None     a polynomial order, or instructions to use the highest     possible order ``` |

|  |  |  |
| --- | --- | --- |
| |  |  | | --- | --- | | extend(self, xi, yi, orders=None) | source code |  ``` Extend the PiecewisePolynomial by a list of points  Parameters ---------- xi : array-like of length N1     a sorted list of x-coordinates yi : list of lists of length N1     yi[i] is the list of derivatives known at xi[i] orders : list of integers, or integer     a list of polynomial orders, or a single universal order direction : {None, 1, -1}     indicates whether the xi are increasing or decreasing     +1 indicates increasing     -1 indicates decreasing     None indicates that it should be deduced from the first two xi ``` |

|  |  |  |
| --- | --- | --- |
| |  |  | | --- | --- | | \_\_call\_\_(self, x)  *(Call operator)* | source code |  ``` Evaluate the piecewise polynomial  Parameters ---------- x : scalar or array-like of length N  Returns ------- y : scalar or array-like of length R or length N or N by R ``` |

|  |  |  |
| --- | --- | --- |
| |  |  | | --- | --- | | derivative(self, x, der) | source code |  ``` Evaluate a derivative of the piecewise polynomial  Parameters ---------- x : scalar or array-like of length N der : integer     which single derivative to extract  Returns ------- y : scalar or array-like of length R or length N or N by R  Notes ----- This currently computes all derivatives of the curve segment containing each x but returns only one. This is because the number of nonzero derivatives that a segment can have depends on the degree of the segment, which may vary. ``` |

|  |  |  |
| --- | --- | --- |
| |  |  | | --- | --- | | derivatives(self, x, der) | source code |  ``` Evaluate a derivative of the piecewise polynomial  Parameters ---------- x : scalar or array-like of length N der : integer     how many derivatives (including the function value as     0th derivative) to extract  Returns ------- y : array-like of shape der by R or der by N or der by N by R ``` |

  


| Home | Trees | Indices | Help | | PyDSTool | | --- | |
| --- | --- | --- | --- | --- | --- |

|  |  |
| --- | --- |
| Generated by Epydoc 3.0.1 on Fri May 4 15:24:10 2012 | http://epydoc.sourceforge.net |
