# Supplementary material for: Hybrid Models and Biological Model Reduction with PyDSTool
Source: PLoS Comput Biol. 2012 Aug 9;8(8):e1002628. doi: 10.1371/journal.pcbi.1002628 (PMC3415397; doi:10.1371/journal.pcbi.1002628)
Supplement: Text S4 — Complete source code for the PyDSTool package (version 0.88.120504). Includes API documentation and help files linking to web pages. This file is identical to the current public release on Sourceforge.net. (ZIP) [file pcbi.1002628.s004.zip › PyDSTool/html/PyDSTool.common.Utility-class.html]

xml version="1.0" encoding="ascii"?


PyDSTool.common.Utility


| Home | Trees | Indices | Help | | PyDSTool | | --- | |
| --- | --- | --- | --- | --- | --- |

|  |  |  |  |
| --- | --- | --- | --- |
| Package PyDSTool :: Module common :: Class Utility | |  | | --- | | [hide private] | | [frames] | no frames] | |

# Class Utility

source code

```
object --+
         |
        Utility
```

Known Subclasses:
:   - PyCont.ContClass'.ContClass
    - , Toolbox.ModelEst.ModelEst
    - , Toolbox.ParamEst.ParamEst

---

```
    Utility abstract class for manipulating and analyzing dynamical systems.

    Robert Clewley, March 2005.

Subclasses of Utility could include such things as continuation tools,
dimension reduction tools, parameter estimation tools.
```


|  |  |  |  |
| --- | --- | --- | --- |
| |  |  | | --- | --- | | Instance Methods | [hide private] | | |
| **Inherited from `object`**: `__delattr__`, `__getattribute__`, `__hash__`, `__init__`, `__new__`, `__reduce__`, `__reduce_ex__`, `__repr__`, `__setattr__`, `__str__` | |


|  |  |  |  |
| --- | --- | --- | --- |
| |  |  | | --- | --- | | Properties | [hide private] | | |
| **Inherited from `object`**: `__class__` | |

| Home | Trees | Indices | Help | | PyDSTool | | --- | |
| --- | --- | --- | --- | --- | --- |

|  |  |
| --- | --- |
| Generated by Epydoc 3.0.1 on Fri May 4 15:24:10 2012 | http://epydoc.sourceforge.net |
