# Supplementary material for: Hybrid Models and Biological Model Reduction with PyDSTool
Source: PLoS Comput Biol. 2012 Aug 9;8(8):e1002628. doi: 10.1371/journal.pcbi.1002628 (PMC3415397; doi:10.1371/journal.pcbi.1002628)
Supplement: Text S4 — Complete source code for the PyDSTool package (version 0.88.120504). Includes API documentation and help files linking to web pages. This file is identical to the current public release on Sourceforge.net. (ZIP) [file pcbi.1002628.s004.zip › PyDSTool/html/PyDSTool.common.Verbose-class.html]

xml version="1.0" encoding="ascii"?


PyDSTool.common.Verbose


| Home | Trees | Indices | Help | | PyDSTool | | --- | |
| --- | --- | --- | --- | --- | --- |

|  |  |  |  |
| --- | --- | --- | --- |
| Package PyDSTool :: Module common :: Class Verbose | |  | | --- | | [hide private] | | [frames] | no frames] | |

# Class Verbose

source code

```
object --+
         |
        Verbose
```

---

A class to handle reporting. Set the fileo attribute to any file
instance to handle the output. Default is sys.stdout


|  |  |  |  |
| --- | --- | --- | --- |
| |  |  | | --- | --- | | Instance Methods | [hide private] | | |
|  | |  |  | | --- | --- | | \_\_init\_\_(self, level)  x.\_\_init\_\_(...) initializes x; see x.\_\_class\_\_.\_\_doc\_\_ for signature | source code | |
|  | |  |  | | --- | --- | | setLevel(self, level)  set the verbosity to one of the Verbose.levels strings | source code | |
|  | |  |  | | --- | --- | | report(self, s, level=`'``helpful``'`)  print message s to self.fileo if self.level>=level. | source code | |
|  | |  |  | | --- | --- | | report\_error(self, s)  print message s to self.fileo if self.level>=level. | source code | |
|  | |  |  | | --- | --- | | wrap(self, fmt, func, level=`'``helpful``'`, always=True)  return a callable function that wraps func and reports it output through the verbose handler if current verbosity level is higher than level | source code | |
|  | |  |  | | --- | --- | | ge(self, level)  return true if self.level is >= level | source code | |
| **Inherited from `object`**: `__delattr__`, `__getattribute__`, `__hash__`, `__new__`, `__reduce__`, `__reduce_ex__`, `__repr__`, `__setattr__`, `__str__` | |


|  |  |  |  |
| --- | --- | --- | --- |
| |  |  | | --- | --- | | Class Variables | [hide private] | | |
|  | levels = `('silent', 'error', 'helpful', 'debug', 'debug-annoyi...` |
|  | vald = `{'debug': 3, 'debug-annoying': 4, 'error': 1, 'helpful'...` |
|  | \_commandLineVerbose = `None` |
|  | i = `4` |
|  | level = `'debug-annoying'` |


|  |  |  |  |
| --- | --- | --- | --- |
| |  |  | | --- | --- | | Properties | [hide private] | | |
| **Inherited from `object`**: `__class__` | |


|  |  |  |  |
| --- | --- | --- | --- |
| |  |  | | --- | --- | | Method Details | [hide private] | | |

|  |  |  |
| --- | --- | --- |
| |  |  | | --- | --- | | \_\_init\_\_(self, level)  *(Constructor)* | source code |   x.\_\_init\_\_(...) initializes x; see x.\_\_class\_\_.\_\_doc\_\_ for signature  Overrides: object.\_\_init\_\_ *(inherited documentation)* |

|  |  |  |
| --- | --- | --- |
| |  |  | | --- | --- | | report(self, s, level=`'``helpful``'`) | source code |   print message s to self.fileo if self.level>=level. Return value indicates whether a message was issue. |

|  |  |  |
| --- | --- | --- |
| |  |  | | --- | --- | | report\_error(self, s) | source code |   print message s to self.fileo if self.level>=level. Return value indicates whether a message was issued |

|  |  |  |
| --- | --- | --- |
| |  |  | | --- | --- | | wrap(self, fmt, func, level=`'``helpful``'`, always=True) | source code |   return a callable function that wraps func and reports it output through the verbose handler if current verbosity level is higher than level  if always is True, the report will occur on every function call; otherwise only on the first time the function is called |

  


|  |  |  |  |
| --- | --- | --- | --- |
| |  |  | | --- | --- | | Class Variable Details | [hide private] | | |

|  |  |
| --- | --- |
| levels   Value:  |  | | --- | | ``` ('silent', 'error', 'helpful', 'debug', 'debug-annoying') ``` | |

|  |  |
| --- | --- |
| vald   Value:  |  | | --- | | ``` {'debug': 3,  'debug-annoying': 4,  'error': 1,  'helpful': 2,  'silent': 0} ``` | |

  


| Home | Trees | Indices | Help | | PyDSTool | | --- | |
| --- | --- | --- | --- | --- | --- |

|  |  |
| --- | --- |
| Generated by Epydoc 3.0.1 on Fri May 4 15:24:10 2012 | http://epydoc.sourceforge.net |
