# Supplementary material for: Hybrid Models and Biological Model Reduction with PyDSTool
Source: PLoS Comput Biol. 2012 Aug 9;8(8):e1002628. doi: 10.1371/journal.pcbi.1002628 (PMC3415397; doi:10.1371/journal.pcbi.1002628)
Supplement: Text S4 — Complete source code for the PyDSTool package (version 0.88.120504). Includes API documentation and help files linking to web pages. This file is identical to the current public release on Sourceforge.net. (ZIP) [file pcbi.1002628.s004.zip › PyDSTool/html/PyDSTool.conf-module.html]

xml version="1.0" encoding="ascii"?


PyDSTool.conf


| Home | Trees | Indices | Help | | PyDSTool | | --- | |
| --- | --- | --- | --- | --- | --- |

|  |  |  |  |
| --- | --- | --- | --- |
| Package PyDSTool :: Module conf | |  | | --- | | [hide private] | | [frames] | no frames] | |

# Module conf

source code


|  |  |  |  |
| --- | --- | --- | --- |
| |  |  | | --- | --- | | Variables | [hide private] | | |
|  | extensions = `['sphinx.ext.autodoc']` |
|  | templates\_path = `['_templates']` |
|  | source\_suffix = `'.rst'` |
|  | master\_doc = `'index'` |
|  | project = `u'PyDSTool'` |
|  | copyright = `u'2010, Robert Clewley'` |
|  | version = `'0.88'` |
|  | release = `'0.88'` |
|  | exclude\_patterns = `['_build']` |
|  | pygments\_style = `'sphinx'` |
|  | html\_theme = `'default'` |
|  | html\_static\_path = `['_static']` |
|  | htmlhelp\_basename = `'PyDSTooldoc'` |
|  | latex\_documents = `[('index', 'PyDSTool.tex', u'PyDSTool Docume...` |
|  | man\_pages = `[('index', 'pydstool', u'PyDSTool Documentation', ...` |


|  |  |  |  |
| --- | --- | --- | --- |
| |  |  | | --- | --- | | Variables Details | [hide private] | | |

|  |  |
| --- | --- |
| latex\_documents   Value:  |  | | --- | | ``` [('index',   'PyDSTool.tex',   u'PyDSTool Documentation',   u'Robert Clewley',   'manual')] ``` | |

|  |  |
| --- | --- |
| man\_pages   Value:  |  | | --- | | ``` [('index',   'pydstool',   u'PyDSTool Documentation',   [u'Robert Clewley'],   1)] ``` | |

  


| Home | Trees | Indices | Help | | PyDSTool | | --- | |
| --- | --- | --- | --- | --- | --- |

|  |  |
| --- | --- |
| Generated by Epydoc 3.0.1 on Fri May 4 15:24:05 2012 | http://epydoc.sourceforge.net |
