# Supplementary material for: Hybrid Models and Biological Model Reduction with PyDSTool
Source: PLoS Comput Biol. 2012 Aug 9;8(8):e1002628. doi: 10.1371/journal.pcbi.1002628 (PMC3415397; doi:10.1371/journal.pcbi.1002628)
Supplement: Text S4 — Complete source code for the PyDSTool package (version 0.88.120504). Includes API documentation and help files linking to web pages. This file is identical to the current public release on Sourceforge.net. (ZIP) [file pcbi.1002628.s004.zip › PyDSTool/html/PyDSTool.conf-pysrc.html]

xml version="1.0" encoding="ascii"?


PyDSTool.conf


| Home | Trees | Indices | Help | | PyDSTool | | --- | |
| --- | --- | --- | --- | --- | --- |

|  |  |  |  |
| --- | --- | --- | --- |
| Package PyDSTool :: Module conf | |  | | --- | | [hide private] | | [frames] | no frames] | |

# Source Code for Module PyDSTool.conf

```
  1  # -*- coding: utf-8 -*- 
  2  # 
  3  # PyDSTool documentation build configuration file, created by 
  4  # sphinx-quickstart on Mon Oct  4 23:37:02 2010. 
  5  # 
  6  # This file is execfile()d with the current directory set to its containing dir. 
  7  # 
  8  # Note that not all possible configuration values are present in this 
  9  # autogenerated file. 
 10  # 
 11  # All configuration values have a default; values that are commented out 
 12  # serve to show the default. 
 13   
 14  import sys, os 
 15   
 16  # If extensions (or modules to document with autodoc) are in another directory, 
 17  # add these directories to sys.path here. If the directory is relative to the 
 18  # documentation root, use os.path.abspath to make it absolute, like shown here. 
 19  #sys.path.insert(0, os.path.abspath('.')) 
 20   
 21  # -- General configuration ----------------------------------------------------- 
 22   
 23  # If your documentation needs a minimal Sphinx version, state it here. 
 24  #needs_sphinx = '1.0' 
 25   
 26  # Add any Sphinx extension module names here, as strings. They can be extensions 
 27  # coming with Sphinx (named 'sphinx.ext.*') or your custom ones. 
 28  extensions = ['sphinx.ext.autodoc'] 
 29   
 30  # Add any paths that contain templates here, relative to this directory. 
 31  templates_path = ['_templates'] 
 32   
 33  # The suffix of source filenames. 
 34  source_suffix = '.rst' 
 35   
 36  # The encoding of source files. 
 37  #source_encoding = 'utf-8-sig' 
 38   
 39  # The master toctree document. 
 40  master_doc = 'index' 
 41   
 42  # General information about the project. 
 43  project = u'PyDSTool' 
 44  copyright = u'2010, Robert Clewley' 
 45   
 46  # The version info for the project you're documenting, acts as replacement for 
 47  # |version| and |release|, also used in various other places throughout the 
 48  # built documents. 
 49  # 
 50  # The short X.Y version. 
 51  version = '0.88' 
 52  # The full version, including alpha/beta/rc tags. 
 53  release = '0.88' 
 54   
 55  # The language for content autogenerated by Sphinx. Refer to documentation 
 56  # for a list of supported languages. 
 57  #language = None 
 58   
 59  # There are two options for replacing |today|: either, you set today to some 
 60  # non-false value, then it is used: 
 61  #today = '' 
 62  # Else, today_fmt is used as the format for a strftime call. 
 63  #today_fmt = '%B %d, %Y' 
 64   
 65  # List of patterns, relative to source directory, that match files and 
 66  # directories to ignore when looking for source files. 
 67  exclude_patterns = ['_build'] 
 68   
 69  # The reST default role (used for this markup: `text`) to use for all documents. 
 70  #default_role = None 
 71   
 72  # If true, '()' will be appended to :func: etc. cross-reference text. 
 73  #add_function_parentheses = True 
 74   
 75  # If true, the current module name will be prepended to all description 
 76  # unit titles (such as .. function::). 
 77  #add_module_names = True 
 78   
 79  # If true, sectionauthor and moduleauthor directives will be shown in the 
 80  # output. They are ignored by default. 
 81  #show_authors = False 
 82   
 83  # The name of the Pygments (syntax highlighting) style to use. 
 84  pygments_style = 'sphinx' 
 85   
 86  # A list of ignored prefixes for module index sorting. 
 87  #modindex_common_prefix = [] 
 88   
 89   
 90  # -- Options for HTML output --------------------------------------------------- 
 91   
 92  # The theme to use for HTML and HTML Help pages.  See the documentation for 
 93  # a list of builtin themes. 
 94  html_theme = 'default' 
 95   
 96  # Theme options are theme-specific and customize the look and feel of a theme 
 97  # further.  For a list of options available for each theme, see the 
 98  # documentation. 
 99  #html_theme_options = {} 
100   
101  # Add any paths that contain custom themes here, relative to this directory. 
102  #html_theme_path = [] 
103   
104  # The name for this set of Sphinx documents.  If None, it defaults to 
105  # "<project> v<release> documentation". 
106  #html_title = None 
107   
108  # A shorter title for the navigation bar.  Default is the same as html_title. 
109  #html_short_title = None 
110   
111  # The name of an image file (relative to this directory) to place at the top 
112  # of the sidebar. 
113  #html_logo = None 
114   
115  # The name of an image file (within the static path) to use as favicon of the 
116  # docs.  This file should be a Windows icon file (.ico) being 16x16 or 32x32 
117  # pixels large. 
118  #html_favicon = None 
119   
120  # Add any paths that contain custom static files (such as style sheets) here, 
121  # relative to this directory. They are copied after the builtin static files, 
122  # so a file named "default.css" will overwrite the builtin "default.css". 
123  html_static_path = ['_static'] 
124   
125  # If not '', a 'Last updated on:' timestamp is inserted at every page bottom, 
126  # using the given strftime format. 
127  #html_last_updated_fmt = '%b %d, %Y' 
128   
129  # If true, SmartyPants will be used to convert quotes and dashes to 
130  # typographically correct entities. 
131  #html_use_smartypants = True 
132   
133  # Custom sidebar templates, maps document names to template names. 
134  #html_sidebars = {} 
135   
136  # Additional templates that should be rendered to pages, maps page names to 
137  # template names. 
138  #html_additional_pages = {} 
139   
140  # If false, no module index is generated. 
141  #html_domain_indices = True 
142   
143  # If false, no index is generated. 
144  #html_use_index = True 
145   
146  # If true, the index is split into individual pages for each letter. 
147  #html_split_index = False 
148   
149  # If true, links to the reST sources are added to the pages. 
150  #html_show_sourcelink = True 
151   
152  # If true, "Created using Sphinx" is shown in the HTML footer. Default is True. 
153  #html_show_sphinx = True 
154   
155  # If true, "(C) Copyright ..." is shown in the HTML footer. Default is True. 
156  #html_show_copyright = True 
157   
158  # If true, an OpenSearch description file will be output, and all pages will 
159  # contain a <link> tag referring to it.  The value of this option must be the 
160  # base URL from which the finished HTML is served. 
161  #html_use_opensearch = '' 
162   
163  # This is the file name suffix for HTML files (e.g. ".xhtml"). 
164  #html_file_suffix = None 
165   
166  # Output file base name for HTML help builder. 
167  htmlhelp_basename = 'PyDSTooldoc' 
168   
169   
170  # -- Options for LaTeX output -------------------------------------------------- 
171   
172  # The paper size ('letter' or 'a4'). 
173  #latex_paper_size = 'letter' 
174   
175  # The font size ('10pt', '11pt' or '12pt'). 
176  #latex_font_size = '10pt' 
177   
178  # Grouping the document tree into LaTeX files. List of tuples 
179  # (source start file, target name, title, author, documentclass [howto/manual]). 
180  latex_documents = [ 
181    ('index', 'PyDSTool.tex', u'PyDSTool Documentation', 
182     u'Robert Clewley', 'manual'), 
183  ] 
184   
185  # The name of an image file (relative to this directory) to place at the top of 
186  # the title page. 
187  #latex_logo = None 
188   
189  # For "manual" documents, if this is true, then toplevel headings are parts, 
190  # not chapters. 
191  #latex_use_parts = False 
192   
193  # If true, show page references after internal links. 
194  #latex_show_pagerefs = False 
195   
196  # If true, show URL addresses after external links. 
197  #latex_show_urls = False 
198   
199  # Additional stuff for the LaTeX preamble. 
200  #latex_preamble = '' 
201   
202  # Documents to append as an appendix to all manuals. 
203  #latex_appendices = [] 
204   
205  # If false, no module index is generated. 
206  #latex_domain_indices = True 
207   
208   
209  # -- Options for manual page output -------------------------------------------- 
210   
211  # One entry per manual page. List of tuples 
212  # (source start file, name, description, authors, manual section). 
213  man_pages = [ 
214      ('index', 'pydstool', u'PyDSTool Documentation', 
215       [u'Robert Clewley'], 1) 
216  ] 
217
```

  


| Home | Trees | Indices | Help | | PyDSTool | | --- | |
| --- | --- | --- | --- | --- | --- |

|  |  |
| --- | --- |
| Generated by Epydoc 3.0.1 on Fri May 4 15:24:14 2012 | http://epydoc.sourceforge.net |
