# Supplementary material for: Hybrid Models and Biological Model Reduction with PyDSTool
Source: PLoS Comput Biol. 2012 Aug 9;8(8):e1002628. doi: 10.1371/journal.pcbi.1002628 (PMC3415397; doi:10.1371/journal.pcbi.1002628)
Supplement: Text S4 — Complete source code for the PyDSTool package (version 0.88.120504). Includes API documentation and help files linking to web pages. This file is identical to the current public release on Sourceforge.net. (ZIP) [file pcbi.1002628.s004.zip › PyDSTool/html/PyDSTool.errors-pysrc.html]

xml version="1.0" encoding="ascii"?


PyDSTool.errors


| Home | Trees | Indices | Help | | PyDSTool | | --- | |
| --- | --- | --- | --- | --- | --- |

|  |  |  |  |
| --- | --- | --- | --- |
| Package PyDSTool :: Module errors | |  | | --- | | [hide private] | | [frames] | no frames] | |

# Source Code for Module PyDSTool.errors

```
 1  ## Exceptions
 
 2  
 
 3  __all__ = ['PyDSTool_Error', 'PyDSTool_BoundsError', 'PyDSTool_KeyError',
 
 4             'PyDSTool_UncertainValueError', 'PyDSTool_TypeError',
 
 5             'PyDSTool_ExistError', 'PyDSTool_AttributeError',
 
 6             'PyDSTool_ValueError', 'PyDSTool_UndefinedError',
 
 7             'PyDSTool_InitError', 'PyDSTool_ClearError',
 
 8             'PyDSTool_ContError'] 
 9  
 
10  
 


11 -class PyDSTool_Error(Exception):


12 -    def __init__(self, value=None):


13          self.value = value 
14          self.code = None


15 -    def __str__(self):


16          return repr(self.value)


17 -    def __repr__(self):


18          return repr(self.value)

19  
 


20 -class PyDSTool_UncertainValueError(PyDSTool_Error):


21 -    def __init__(self, value, varval=None):


22          if varval is None: 
23              valstr = '' 
24          else: 
25              valstr = ' at variable = '+str(varval) 
26          self.varval = varval 
27          PyDSTool_Error.__init__(self, value+valstr)

28  
 


29 -class PyDSTool_BoundsError(PyDSTool_Error):


30      pass

31  
 


32 -class PyDSTool_KeyError(PyDSTool_Error):


33      pass

34  
 


35 -class PyDSTool_ValueError(PyDSTool_Error):


36      pass

37  
 


38 -class PyDSTool_TypeError(PyDSTool_Error):


39      pass

40  
 


41 -class PyDSTool_ExistError(PyDSTool_Error):


42      pass

43  
 


44 -class PyDSTool_UndefinedError(PyDSTool_Error):


45      pass

46  
 


47 -class PyDSTool_AttributeError(PyDSTool_Error):


48      pass

49  
 


50 -class PyDSTool_InitError(PyDSTool_Error):


51      pass

52  
 


53 -class PyDSTool_ClearError(PyDSTool_Error):


54      pass

55  
 


56 -class PyDSTool_ContError(PyDSTool_Error):


57      pass

58
```

  


| Home | Trees | Indices | Help | | PyDSTool | | --- | |
| --- | --- | --- | --- | --- | --- |

|  |  |
| --- | --- |
| Generated by Epydoc 3.0.1 on Fri May 4 15:24:20 2012 | http://epydoc.sourceforge.net |
