# Supplementary material for: Hybrid Models and Biological Model Reduction with PyDSTool
Source: PLoS Comput Biol. 2012 Aug 9;8(8):e1002628. doi: 10.1371/journal.pcbi.1002628 (PMC3415397; doi:10.1371/journal.pcbi.1002628)
Supplement: Text S4 — Complete source code for the PyDSTool package (version 0.88.120504). Includes API documentation and help files linking to web pages. This file is identical to the current public release on Sourceforge.net. (ZIP) [file pcbi.1002628.s004.zip › PyDSTool/html/PyDSTool.errors.PyDSTool_Error-class.html]

xml version="1.0" encoding="ascii"?


PyDSTool.errors.PyDSTool\_Error


| Home | Trees | Indices | Help | | PyDSTool | | --- | |
| --- | --- | --- | --- | --- | --- |

|  |  |  |  |
| --- | --- | --- | --- |
| Package PyDSTool :: Module errors :: Class PyDSTool\_Error | |  | | --- | | [hide private] | | [frames] | no frames] | |

# Class PyDSTool\_Error

source code

```
              object --+        
                       |        
exceptions.BaseException --+    
                           |    
        exceptions.Exception --+
                               |
                              PyDSTool_Error
```

Known Subclasses:
:   - PyDSTool\_AttributeError
    - , PyDSTool\_BoundsError
    - , PyDSTool\_ClearError
    - , PyDSTool\_ContError
    - , PyDSTool\_ExistError
    - , PyDSTool\_InitError
    - , PyDSTool\_KeyError
    - , PyDSTool\_TypeError
    - , PyDSTool\_UncertainValueError
    - , PyDSTool\_UndefinedError
    - , PyDSTool\_ValueError
    - , Toolbox.ParamEst.ConstraintFail
    - , Toolbox.ParamEst.Converged

---


|  |  |  |  |
| --- | --- | --- | --- |
| |  |  | | --- | --- | | Instance Methods | [hide private] | | |
|  | |  |  | | --- | --- | | \_\_init\_\_(self, value=None)  x.\_\_init\_\_(...) initializes x; see x.\_\_class\_\_.\_\_doc\_\_ for signature | source code | |
|  | |  |  | | --- | --- | | \_\_str\_\_(self)  str(x) | source code | |
|  | |  |  | | --- | --- | | \_\_repr\_\_(self)  repr(x) | source code | |
| **Inherited from `exceptions.Exception`**: `__new__`  **Inherited from `exceptions.BaseException`**: `__delattr__`, `__getattribute__`, `__getitem__`, `__getslice__`, `__reduce__`, `__setattr__`, `__setstate__`  **Inherited from `object`**: `__hash__`, `__reduce_ex__` | |


|  |  |  |  |
| --- | --- | --- | --- |
| |  |  | | --- | --- | | Properties | [hide private] | | |
| **Inherited from `exceptions.BaseException`**: `args`, `message`  **Inherited from `object`**: `__class__` | |


|  |  |  |  |
| --- | --- | --- | --- |
| |  |  | | --- | --- | | Method Details | [hide private] | | |

|  |  |  |
| --- | --- | --- |
| |  |  | | --- | --- | | \_\_init\_\_(self, value=None)  *(Constructor)* | source code |   x.\_\_init\_\_(...) initializes x; see x.\_\_class\_\_.\_\_doc\_\_ for signature  Overrides: object.\_\_init\_\_ *(inherited documentation)* |

|  |  |  |
| --- | --- | --- |
| |  |  | | --- | --- | | \_\_str\_\_(self)  *(Informal representation operator)* | source code |   str(x)  Overrides: object.\_\_str\_\_ *(inherited documentation)* |

|  |  |  |
| --- | --- | --- |
| |  |  | | --- | --- | | \_\_repr\_\_(self)  *(Representation operator)* | source code |   repr(x)  Overrides: object.\_\_repr\_\_ *(inherited documentation)* |

  


| Home | Trees | Indices | Help | | PyDSTool | | --- | |
| --- | --- | --- | --- | --- | --- |

|  |  |
| --- | --- |
| Generated by Epydoc 3.0.1 on Fri May 4 15:24:10 2012 | http://epydoc.sourceforge.net |
