# Supplementary material for: Hybrid Models and Biological Model Reduction with PyDSTool
Source: PLoS Comput Biol. 2012 Aug 9;8(8):e1002628. doi: 10.1371/journal.pcbi.1002628 (PMC3415397; doi:10.1371/journal.pcbi.1002628)
Supplement: Text S4 — Complete source code for the PyDSTool package (version 0.88.120504). Includes API documentation and help files linking to web pages. This file is identical to the current public release on Sourceforge.net. (ZIP) [file pcbi.1002628.s004.zip › PyDSTool/html/PyDSTool.errors.PyDSTool_UncertainValueError-class.html]

xml version="1.0" encoding="ascii"?


PyDSTool.errors.PyDSTool\_UncertainValueError


| Home | Trees | Indices | Help | | PyDSTool | | --- | |
| --- | --- | --- | --- | --- | --- |

|  |  |  |  |
| --- | --- | --- | --- |
| Package PyDSTool :: Module errors :: Class PyDSTool\_UncertainValueError | |  | | --- | | [hide private] | | [frames] | no frames] | |

# Class PyDSTool\_UncertainValueError

source code

```
              object --+            
                       |            
exceptions.BaseException --+        
                           |        
        exceptions.Exception --+    
                               |    
                  PyDSTool_Error --+
                                   |
                                  PyDSTool_UncertainValueError
```

---


|  |  |  |  |
| --- | --- | --- | --- |
| |  |  | | --- | --- | | Instance Methods | [hide private] | | |
|  | |  |  | | --- | --- | | \_\_init\_\_(self, value, varval=None)  x.\_\_init\_\_(...) initializes x; see x.\_\_class\_\_.\_\_doc\_\_ for signature | source code | |
| **Inherited from `PyDSTool_Error`**: `__repr__`, `__str__`  **Inherited from `exceptions.Exception`**: `__new__`  **Inherited from `exceptions.BaseException`**: `__delattr__`, `__getattribute__`, `__getitem__`, `__getslice__`, `__reduce__`, `__setattr__`, `__setstate__`  **Inherited from `object`**: `__hash__`, `__reduce_ex__` | |


|  |  |  |  |
| --- | --- | --- | --- |
| |  |  | | --- | --- | | Properties | [hide private] | | |
| **Inherited from `exceptions.BaseException`**: `args`, `message`  **Inherited from `object`**: `__class__` | |


|  |  |  |  |
| --- | --- | --- | --- |
| |  |  | | --- | --- | | Method Details | [hide private] | | |

|  |  |  |
| --- | --- | --- |
| |  |  | | --- | --- | | \_\_init\_\_(self, value, varval=None)  *(Constructor)* | source code |   x.\_\_init\_\_(...) initializes x; see x.\_\_class\_\_.\_\_doc\_\_ for signature  Overrides: object.\_\_init\_\_ *(inherited documentation)* |

  


| Home | Trees | Indices | Help | | PyDSTool | | --- | |
| --- | --- | --- | --- | --- | --- |

|  |  |
| --- | --- |
| Generated by Epydoc 3.0.1 on Fri May 4 15:24:10 2012 | http://epydoc.sourceforge.net |
