# Supplementary material for: Hybrid Models and Biological Model Reduction with PyDSTool
Source: PLoS Comput Biol. 2012 Aug 9;8(8):e1002628. doi: 10.1371/journal.pcbi.1002628 (PMC3415397; doi:10.1371/journal.pcbi.1002628)
Supplement: Text S4 — Complete source code for the PyDSTool package (version 0.88.120504). Includes API documentation and help files linking to web pages. This file is identical to the current public release on Sourceforge.net. (ZIP) [file pcbi.1002628.s004.zip › PyDSTool/html/PyDSTool.Events-module.html]

xml version="1.0" encoding="ascii"?


PyDSTool.Events


| Home | Trees | Indices | Help | | PyDSTool | | --- | |
| --- | --- | --- | --- | --- | --- |

|  |  |  |  |
| --- | --- | --- | --- |
| Package PyDSTool :: Module Events | |  | | --- | | [hide private] | | [frames] | no frames] | |

# Module Events

source code

```
Event handling for python-based computations, and specification for
both python and externally compiled code. (Externally compiled code
may include its own event determination implementation.)

"High-level" events are built in native Python function format.
"Low-level" events are built for external platforms, e.g. C or Matlab code.

    Robert Clewley, October 2005.
```


|  |  |  |  |
| --- | --- | --- | --- |
| |  |  | | --- | --- | | Classes | [hide private] | | |
|  | EventStruct  A data structure to store and interface with multiple events. |
|  | Event  Generic Event. |
|  | HighLevelEvent  Event defined using python function code. |
|  | LowLevelEvent  Event defined using externally-compiled and linked function code (i.e. |
|  | MatlabEvent  Event defined using MATLAB syntax for use with ADMC++ |


|  |  |  |  |
| --- | --- | --- | --- |
| |  |  | | --- | --- | | Functions | [hide private] | | |
|  | |  |  | | --- | --- | | \_highlevel(arg) | source code | |
|  | |  |  | | --- | --- | | \_lowlevel(arg) | source code | |
|  | |  |  | | --- | --- | | \_term(arg) | source code | |
|  | |  |  | | --- | --- | | \_nonterm(arg) | source code | |
|  | |  |  | | --- | --- | | \_active(arg) | source code | |
|  | |  |  | | --- | --- | | \_notactive(arg) | source code | |
|  | |  |  | | --- | --- | | \_varlinked(arg) | source code | |
|  | |  |  | | --- | --- | | \_notvarlinked(arg) | source code | |
|  | |  |  | | --- | --- | | \_precise(arg) | source code | |
|  | |  |  | | --- | --- | | \_notprecise(arg) | source code | |
|  | |  |  | | --- | --- | | makeZeroCrossEvent(expr, dircode, argDict, varnames=`[``]`, parnames=`[``]`, inputnames=`[``]`, fnspecs=`{``}`, targetlang=`'``python``'`, reuseterms=`{``}`, flatspec=None)  Target language-independent user-defined event involving coordinates, parameters, and time. | source code | |
|  | |  |  | | --- | --- | | makePythonStateZeroCrossEvent(varname, targetvalue, dircode, argDict, var=None)  Python function-specified zero-crossing event in coordinate, or in time. | source code | |
|  | |  |  | | --- | --- | | collectReused(quant, allvars, allpars, allinputs, allfuns, varnames, parnames, inputnames, auxfns, auxVarDefMap, flatspec) | source code | |
|  | |  |  | | --- | --- | | processReusedPy(specnames, specdict, reuseterms, fspec, specials=`[``]`, dovars=True, dopars=True, doinps=True, illegal=`[``]`)  Process reused subexpression terms for Python code. | source code | |
|  | |  |  | | --- | --- | | processReusedC(specnames, specdict, reuseterms)  Process reused subexpression terms for C code. | source code | |
|  | |  |  | | --- | --- | | processReusedMatlab(specnames, specdict, reuseterms)  Process reused subexpression terms for matlab code. | source code | |
|  | |  |  | | --- | --- | | findpreciseroot(ev, tlo, thi, parDict=None, vars=None, inputs=None, globalt0=0, quadratic\_interp=None)  Find root more accurately from a Variable object using bisection. | source code | |


|  |  |  |  |
| --- | --- | --- | --- |
| |  |  | | --- | --- | | Variables | [hide private] | | |
|  | \_1DimplicitSolveMethods = `['newton', 'bisect', 'steffe']` |
|  | \_all\_complex = `(<type 'complex'>, <type 'numpy.complexfloating...` |
|  | \_all\_float = `(<type 'float'>, <type 'numpy.floating'>, <type '...` |
|  | \_all\_int = `(<type 'int'>, <type 'numpy.integer'>, <type 'numpy...` |
|  | \_all\_numpy\_complex = `(<type 'numpy.complex128'>, <type 'numpy....` |
|  | \_all\_numpy\_float = `(<type 'numpy.float64'>, <type 'numpy.float...` |
|  | \_all\_numpy\_int = `(<type 'numpy.int32'>, <type 'numpy.int32'>, ...` |
|  | \_complex\_types = `(<type 'complex'>, <type 'numpy.complexfloati...` |
|  | \_float\_types = `(<type 'float'>, <type 'numpy.floating'>)` |
|  | \_implicitSolveMethods = `['newton', 'bisect', 'steffe', 'fsolve']` |
|  | \_indentstr = `' '` |
|  | \_int\_types = `(<type 'int'>, <type 'numpy.integer'>)` |
|  | \_num\_equivtype = `{<type 'float'>: <type 'numpy.float64'>, <typ...` |
|  | \_num\_maxmin = `{<type 'numpy.int32'>: [-2147483648, 2147483647]...` |
|  | \_num\_name2equivtypes = `{'float': (<type 'float'>, <type 'numpy...` |
|  | \_num\_name2type = `{'float': <type 'numpy.float64'>, 'int': <typ...` |
|  | \_num\_type2name = `{<type 'float'>: 'float', <type 'int'>: 'int'...` |
|  | \_num\_types = `(<type 'float'>, <type 'int'>, <type 'numpy.float...` |
|  | \_pytypefromtype = `{<type 'numpy.int32'>: <type 'int'>, <type '...` |
|  | \_real\_types = `(<type 'int'>, <type 'numpy.integer'>, <type 'fl...` |
|  | \_seq\_types = `(<type 'list'>, <type 'tuple'>, <type 'numpy.ndar...` |


|  |  |  |  |
| --- | --- | --- | --- |
| |  |  | | --- | --- | | Function Details | [hide private] | | |

|  |  |  |
| --- | --- | --- |
| |  |  | | --- | --- | | makeZeroCrossEvent(expr, dircode, argDict, varnames=`[``]`, parnames=`[``]`, inputnames=`[``]`, fnspecs=`{``}`, targetlang=`'``python``'`, reuseterms=`{``}`, flatspec=None) | source code |   Target language-independent user-defined event involving coordinates, parameters, and time. Returns a non variable-linked event only.  List of used variable, parameter, and input names defaults to the empty list and can be omitted if these are not referenced. If variable names are omitted then the expression must only depend on time 't' and any declared parameters.  Auxiliary function dictionary is required if the event accesses them.  'targetlang' argument defaults to 'python'. The expression 'expr' may not use intermediate temporary variables (for which you should specify the body of the event function by hand.  Optional argument reuseterms is a dictionary of terms used in expr that map to their definitions in terms of the state variables, time, parameters, and inputs. |

|  |  |  |
| --- | --- | --- |
| |  |  | | --- | --- | | makePythonStateZeroCrossEvent(varname, targetvalue, dircode, argDict, var=None) | source code |   Python function-specified zero-crossing event in coordinate, or in time. Use 'var' argument to create a variable-linked event.  varname may be a Quantity object. dircode is -1, 0, or 1. varname may be the reserved word 't', for the independent variable. |

|  |  |  |
| --- | --- | --- |
| |  |  | | --- | --- | | processReusedPy(specnames, specdict, reuseterms, fspec, specials=`[``]`, dovars=True, dopars=True, doinps=True, illegal=`[``]`) | source code |   Process reused subexpression terms for Python code. (Similar to function of similar name in FuncSpec.py) |

|  |  |  |
| --- | --- | --- |
| |  |  | | --- | --- | | processReusedC(specnames, specdict, reuseterms) | source code |   Process reused subexpression terms for C code. (Similar to function processReusedC in FuncSpec.py) |

|  |  |  |
| --- | --- | --- |
| |  |  | | --- | --- | | processReusedMatlab(specnames, specdict, reuseterms) | source code |   Process reused subexpression terms for matlab code. (Similar to function of similar name in FuncSpec.py) |

|  |  |  |
| --- | --- | --- |
| |  |  | | --- | --- | | findpreciseroot(ev, tlo, thi, parDict=None, vars=None, inputs=None, globalt0=0, quadratic\_interp=None) | source code |   Find root more accurately from a Variable object using bisection.  (Adapted from scipy.optimize.minpack.bisection code to make use of quadratic interpolation, which assumes that tlo and thi are already known to be close enough together that the variable's curve is purely concave up or down in the neighbourhood, and so can be fitted accurately with a single quadratic).  To use quadratic interpolation, pass a fit\_quadratic instance as the quadratic\_interp argument. Interpolation will also be done on any inputs provided (\*\*not yet implemented\*\*). |

  


|  |  |  |  |
| --- | --- | --- | --- |
| |  |  | | --- | --- | | Variables Details | [hide private] | | |

|  |  |
| --- | --- |
| \_all\_complex   Value:  |  | | --- | | ``` (<type 'complex'>,  <type 'numpy.complexfloating'>,  <type 'numpy.complex128'>,  <type 'numpy.complex64'>,  <type 'numpy.complex128'>) ``` | |

|  |  |
| --- | --- |
| \_all\_float   Value:  |  | | --- | | ``` (<type 'float'>,  <type 'numpy.floating'>,  <type 'numpy.float64'>,  <type 'numpy.float32'>,  <type 'numpy.float64'>) ``` | |

|  |  |
| --- | --- |
| \_all\_int   Value:  |  | | --- | | ``` (<type 'int'>,  <type 'numpy.integer'>,  <type 'numpy.int32'>,  <type 'numpy.int32'>,  <type 'numpy.int8'>,  <type 'numpy.int16'>,  <type 'numpy.int32'>,  <type 'numpy.int64'>) ``` | |

|  |  |
| --- | --- |
| \_all\_numpy\_complex   Value:  |  | | --- | | ``` (<type 'numpy.complex128'>,  <type 'numpy.complex64'>,  <type 'numpy.complex128'>) ``` | |

|  |  |
| --- | --- |
| \_all\_numpy\_float   Value:  |  | | --- | | ``` (<type 'numpy.float64'>,  <type 'numpy.float32'>,  <type 'numpy.float64'>) ``` | |

|  |  |
| --- | --- |
| \_all\_numpy\_int   Value:  |  | | --- | | ``` (<type 'numpy.int32'>,  <type 'numpy.int32'>,  <type 'numpy.int8'>,  <type 'numpy.int16'>,  <type 'numpy.int32'>,  <type 'numpy.int64'>) ``` | |

|  |  |
| --- | --- |
| \_complex\_types   Value:  |  | | --- | | ``` (<type 'complex'>, <type 'numpy.complexfloating'>) ``` | |

|  |  |
| --- | --- |
| \_num\_equivtype   Value:  |  | | --- | | ``` {<type 'float'>: <type 'numpy.float64'>,  <type 'int'>: <type 'numpy.int32'>,  <type 'numpy.integer'>: <type 'numpy.int32'>,  <type 'numpy.floating'>: <type 'numpy.float64'>,  <type 'numpy.int8'>: <type 'numpy.int32'>,  <type 'numpy.int16'>: <type 'numpy.int32'>,  <type 'numpy.int32'>: <type 'numpy.int32'>,  <type 'numpy.int32'>: <type 'numpy.int32'>, ... ``` | |

|  |  |
| --- | --- |
| \_num\_maxmin   Value:  |  | | --- | | ``` {<type 'numpy.int32'>: [-2147483648, 2147483647],  <type 'numpy.float64'>: [-inf, inf]} ``` | |

|  |  |
| --- | --- |
| \_num\_name2equivtypes   Value:  |  | | --- | | ``` {'float': (<type 'float'>,            <type 'numpy.floating'>,            <type 'numpy.float64'>,            <type 'numpy.float32'>,            <type 'numpy.float64'>),  'int': (<type 'int'>,          <type 'numpy.integer'>,          <type 'numpy.int32'>, ... ``` | |

|  |  |
| --- | --- |
| \_num\_name2type   Value:  |  | | --- | | ``` {'float': <type 'numpy.float64'>, 'int': <type 'numpy.int32'>} ``` | |

|  |  |
| --- | --- |
| \_num\_type2name   Value:  |  | | --- | | ``` {<type 'float'>: 'float',  <type 'int'>: 'int',  <type 'numpy.integer'>: 'int',  <type 'numpy.floating'>: 'float',  <type 'numpy.int8'>: 'int',  <type 'numpy.int16'>: 'int',  <type 'numpy.int32'>: 'int',  <type 'numpy.int32'>: 'int', ... ``` | |

|  |  |
| --- | --- |
| \_num\_types   Value:  |  | | --- | | ``` (<type 'float'>,  <type 'int'>,  <type 'numpy.floating'>,  <type 'numpy.integer'>) ``` | |

|  |  |
| --- | --- |
| \_pytypefromtype   Value:  |  | | --- | | ``` {<type 'numpy.int32'>: <type 'int'>,  <type 'numpy.float64'>: <type 'float'>} ``` | |

|  |  |
| --- | --- |
| \_real\_types   Value:  |  | | --- | | ``` (<type 'int'>,  <type 'numpy.integer'>,  <type 'float'>,  <type 'numpy.floating'>) ``` | |

|  |  |
| --- | --- |
| \_seq\_types   Value:  |  | | --- | | ``` (<type 'list'>, <type 'tuple'>, <type 'numpy.ndarray'>) ``` | |

  


| Home | Trees | Indices | Help | | PyDSTool | | --- | |
| --- | --- | --- | --- | --- | --- |

|  |  |
| --- | --- |
| Generated by Epydoc 3.0.1 on Fri May 4 15:24:02 2012 | http://epydoc.sourceforge.net |
