# Supplementary material for: Hybrid Models and Biological Model Reduction with PyDSTool
Source: PLoS Comput Biol. 2012 Aug 9;8(8):e1002628. doi: 10.1371/journal.pcbi.1002628 (PMC3415397; doi:10.1371/journal.pcbi.1002628)
Supplement: Text S4 — Complete source code for the PyDSTool package (version 0.88.120504). Includes API documentation and help files linking to web pages. This file is identical to the current public release on Sourceforge.net. (ZIP) [file pcbi.1002628.s004.zip › PyDSTool/html/PyDSTool.Events-pysrc.html]

xml version="1.0" encoding="ascii"?


PyDSTool.Events


| Home | Trees | Indices | Help | | PyDSTool | | --- | |
| --- | --- | --- | --- | --- | --- |

|  |  |  |  |
| --- | --- | --- | --- |
| Package PyDSTool :: Module Events | |  | | --- | | [hide private] | | [frames] | no frames] | |

# Source Code for Module PyDSTool.Events

```
   1  """Event handling for python-based computations, and specification for
 
   2  both python and externally compiled code. (Externally compiled code
 
   3  may include its own event determination implementation.)
 
   4  
 
   5  "High-level" events are built in native Python function format.
 
   6  "Low-level" events are built for external platforms, e.g. C or Matlab code.
 
   7  
 
   8      Robert Clewley, October 2005.
 
   9  """ 
  10  
 
  11  # PyDSTool imports
 
  12  from Variable import * 
  13  from utils import * 
  14  from common import * 
  15  from parseUtils import * 
  16  from errors import * 
  17  from Interval import * 
  18  from utils import info as utils_info 
  19  from Symbolic import QuantSpec, Var, ensureStrArgDict 
  20  import FuncSpec 
  21  
 
  22  # Other imports
 
  23  import scipy, numpy, scipy, scipy.special 
  24  import math, random 
  25  import copy, types 
  26  
 
  27  __all__ = ['EventStruct', 'Event',
 
  28              'HighLevelEvent', 'LowLevelEvent', 'MatlabEvent',
 
  29              'makePythonStateZeroCrossEvent', 'makeZeroCrossEvent'] 
  30  
 
  31  
 
  32  # helper functions
 


33 -def _highlevel(arg):


34      return isinstance(arg[1], HighLevelEvent)

  35  
 


36 -def _lowlevel(arg):


37      return isinstance(arg[1], LowLevelEvent)

  38  
 


39 -def _term(arg):


40      return arg[1].termFlag

  41  
 


42 -def _nonterm(arg):


43      return not arg[1].termFlag

  44  
 


45 -def _active(arg):


46      return arg[1].activeFlag

  47  
 


48 -def _notactive(arg):


49      return not arg[1].activeFlag

  50  
 


51 -def _varlinked(arg):


52      return arg[1].varlinked

  53  
 


54 -def _notvarlinked(arg):


55      return not arg[1].varlinked

  56  
 


57 -def _precise(arg):


58      return arg[1].preciseFlag

  59  
 


60 -def _notprecise(arg):


61      return not arg[1].preciseFlag

  62  
 
  63  
 
  64  # ---------------------------------------------------------------------------
 
  65  
 


66 -class EventStruct(object):


67      """A data structure to store and interface with multiple events.""" 
  68  
 


69 -    def __init__(self):


70          self.events = {} 
  71          self._keylist = ['term', 'nonterm', 'active', 'varlinked', 'precise',
 
  72                           'notprecise', 'notvarlinked', 'notactive', 'highlevel',
 
  73                           'lowlevel'] 
  74          self._makeFilterDict() 
  75          # record of recent events (mainly for checking event interval
 
  76          # in high-level events)
 
  77          self.resetEvtimes()

  78  
 


79 -    def _makeFilterDict(self):


80          self._filterDict = {} 
  81          self._filterDict['highlevel'] = _highlevel 
  82          self._filterDict['lowlevel'] = _lowlevel 
  83          self._filterDict['term'] = _term 
  84          self._filterDict['nonterm'] = _nonterm 
  85          self._filterDict['active'] = _active 
  86          self._filterDict['notactive'] = _notactive 
  87          self._filterDict['varlinked'] = _varlinked 
  88          self._filterDict['notvarlinked'] = _notvarlinked 
  89          self._filterDict['precise'] = _precise 
  90          self._filterDict['notprecise'] = _notprecise

  91  
 


92 -    def resetEvtimes(self):


93          self.Evtimes = {}

  94  
 


95 -    def __deepcopy__(self, dummy):


96          # bug in deepcopy concerning _filterDict attribute, so work around
 
  97          # - self.events is the only thing to really deep copy anyway
 
  98          deepcopied_events = copy.deepcopy(self.events) 
  99          dcopy = copy.copy(self) 
 100          dcopy.events = deepcopied_events 
 101          return dcopy

 102  
 


103 -    def __del__(self):


104          # not sure if this is necessary in order to evoke Event.__del__
 
 105          for ev in self.events: 
 106              del ev

 107  
 


108 -    def __setitem__(self, ev):


109          if compareBaseClass(ev, Event): 
 110              if ev.name not in self.events: 
 111                  self.events[ev.name] = ev 
 112              else: 
 113                  print self 
 114                  raise ValueError("Event name '"+ev.name+"' already present "
 
 115                                      "in database") 
 116          elif isinstance(ev,list): 
 117              for ev_item in ev: 
 118                  self.__setitem__(ev_item) 
 119          else: 
 120              raise TypeError('Argument must be an Event: received type '
 
 121                              '%s'%str(type(ev)))

 122  
 
 123      add = __setitem__ 
 124  
 


125 -    def __delitem__(self, ename):


126          del(self.events[ename])

 127  
 
 128      delete = __delitem__ 
 129  
 


130 -    def __getitem__(self, ename):


131          return self.events[ename]

 132  
 


133 -    def sortedEventNames(self, eventlist=None):


134          if eventlist is None: 
 135              return sortedDictKeys(self.events) 
 136          else: 
 137              eventlist.sort() 
 138              return eventlist

 139  
 


140 -    def getHighLevelEvents(self):


141          hlList = [] 
 142          for epair in self.events.items(): 
 143              if isinstance(epair[1], HighLevelEvent): 
 144                  hlList.append(epair) 
 145          return hlList

 146  
 


147 -    def getLowLevelEvents(self):


148          llList = [] 
 149          for epair in self.events.items(): 
 150              if isinstance(epair[1], LowLevelEvent): 
 151                  llList.append(epair) 
 152          return llList

 153  
 


154 -    def getAllEvents(self):


155          return self.events.items()

 156  
 


157 -    def getTermEvents(self):


158          teList = [] 
 159          for epair in self.events.items(): 
 160              if epair[1].termFlag: 
 161                  teList.append(epair) 
 162          return teList

 163  
 


164 -    def getNonTermEvents(self):


165          neList = [] 
 166          for epair in self.events.items(): 
 167              if not epair[1].termFlag: 
 168                  neList.append(epair) 
 169          return neList

 170  
 


171 -    def getActiveEvents(self):


172          neList = [] 
 173          for epair in self.events.items(): 
 174              if epair[1].activeFlag: 
 175                  neList.append(epair) 
 176          return neList

 177  
 


178 -    def getNonActiveEvents(self):


179          neList = [] 
 180          for epair in self.events.items(): 
 181              if not epair[1].activeFlag: 
 182                  neList.append(epair) 
 183          return neList

 184  
 


185 -    def getNonPreciseEvents(self):


186          neList = [] 
 187          for epair in self.events.items(): 
 188              if not epair[1].preciseFlag: 
 189                  neList.append(epair) 
 190          return neList

 191  
 


192 -    def getPreciseEvents(self):


193          neList = [] 
 194          for epair in self.events.items(): 
 195              if epair[1].preciseFlag: 
 196                  neList.append(epair) 
 197          return neList

 198  
 


199 -    def setglobalt0(self, t0):


200          for epair in self.events.items(): 
 201              epair[1].globalt0 = t0

 202  
 


203 -    def query(self, keylist, eventlist=None):


204          """Return eventlist with results of queries corresponding
 
 205          to self._keylist keys.
 
 206  
 
 207          Multiple keys permitted in the query.
 
 208          """ 
 209          if not isinstance(keylist, list): 
 210              raise TypeError('Query argument must be a list of keys') 
 211          for key in keylist: 
 212              if key not in self._keylist: 
 213                  raise TypeError('Query keys must be in _keylist attribute') 
 214          filterFuncs = [self._filterDict[key] for key in keylist] 
 215          if eventlist is None: 
 216              if self.events == {}: 
 217                  return [] 
 218              else: 
 219                  eventlist = self.events.items() 
 220          if filterFuncs == []: 
 221              return [] 
 222          for f in filterFuncs: 
 223              eventlist = filter(f, eventlist) 
 224          return eventlist

 225  
 
 226  
 


227 -    def __call__(self):


228          # info is defined in utils.py
 
 229          utils_info(self.__dict__, "EventStruct")

 230  
 
 231  
 


232 -    def info(self, verboselevel=0):


233          if verboselevel > 0: 
 234              # info is defined in utils.py
 
 235              utils_info(self.__dict__, "EventStruct",
 
 236                          recurseDepthLimit=1+verboselevel) 
 237          else: 
 238              print self.__repr__()

 239  
 
 240  
 


241 -    def __contains__(self, evname):


242          return evname in self.events

 243  
 
 244      # If low level events are passed to this method, there will be an
 
 245      # exception because they do not have __call__ method defined!
 
 246      # This function is used for non-varlinked events, e.g. for use in
 
 247      # integration loops.
 
 248      # varDict must be supplied (not None) for this polling to work!
 


249 -    def pollHighLevelEvents(self, tval=None, varDict=None, parDict=None,
 
 250                              eventlist=None):


251          if eventlist is None: 
 252              eventlist = self.query(['highlevel', 'active', 'notvarlinked']) 
 253          return filter(lambda (n,e): e(t=tval, varDict=varDict,
 
 254                                          parDict=parDict), eventlist)

 255  
 
 256  
 


257 -    def resetHighLevelEvents(self, t0, eventlist=None, state=None):


258          if eventlist is None: 
 259              eventlist = self.query(['highlevel']) 
 260          if type(state)==list: 
 261              for i, (ev, s) in enumerate(zip(eventlist, state)): 
 262                  ev[1].reset(s) 
 263                  ev[1].starttime = t0 
 264          else: 
 265              for ev in eventlist: 
 266                  ev[1].reset(state) 
 267                  ev[1].starttime = t0

 268  
 
 269  
 


270 -    def validateEvents(self, database, eventlist):


271          """validateEvents is only used for high level events.""" 
 272          assert eventlist != [], 'Empty event list passed to validateEvents' 
 273          for ev in eventlist: 
 274              if isinstance(ev[1], HighLevelEvent): 
 275                  if not reduce(bool.__and__, [key in database for key in \
 
 276                                              ev[1].vars.keys()]): 
 277                      ek = ev[1].vars.keys() 
 278                      print "Missing keys: ", remain(ek, database) 
 279                      raise RuntimeError("Invalid keys in event '%s'" % ev[0])

 280              #else:
 
 281              #    print "Warning: Low level events should not be passed to " \
 
 282              #            + "validateEvents()"
 
 283              #    print "   (event '", ev[0], "')"
 
 284  
 
 285  
 


286 -    def setTermFlag(self, eventTarget, flagval):


287          if isinstance(flagval, bool): 
 288              if isinstance(eventTarget, list): 
 289                  for evTarg in intersect(eventTarget, self.events.keys()): 
 290                      self.events[evTarg].termFlag = flagval 
 291              else: 
 292                  if eventTarget in self.events.keys(): 
 293                      self.events[eventTarget].termFlag = flagval 
 294          else: 
 295              raise TypeError("Invalid flag type")

 296  
 
 297  
 


298 -    def setActiveFlag(self, eventTarget, flagval):


299          if isinstance(flagval, bool): 
 300              if isinstance(eventTarget, list): 
 301                  for evTarg in intersect(eventTarget, self.events.keys()): 
 302                      self.events[evTarg].activeFlag = flagval 
 303              else: 
 304                  if eventTarget in self.events.keys(): 
 305                      self.events[eventTarget].activeFlag = flagval 
 306          else: 
 307              raise TypeError("Invalid flag type")

 308  
 


309 -    def setPreciseFlag(self, eventTarget, flagval):


310          if isinstance(flagval, bool): 
 311              if isinstance(eventTarget, list): 
 312                  for evTarg in intersect(eventTarget, self.events.keys()): 
 313                      self.events[evTarg].preciseFlag = flagval 
 314              else: 
 315                  if eventTarget in self.events.keys(): 
 316                      self.events[eventTarget].preciseFlag = flagval 
 317          else: 
 318              raise TypeError("Invalid flag type")

 319  
 


320 -    def setEventICs(self, eventTarget, val):


321          if isinstance(val, dict): 
 322              if isinstance(eventTarget, list): 
 323                  for evTarg in intersect(eventTarget, self.events.keys()): 
 324                      for name in val.keys(): 
 325                          if name in self.events[evTarg].initialconditions.keys(): 
 326                              self.events[evTarg].initialconditions[name] = val[name] 
 327              else: 
 328                  if eventTarget in self.events.keys(): 
 329                      for name in val.keys(): 
 330                          if name in self.events[eventTarget].initialconditions.keys(): 
 331                              self.events[eventTarget].initialconditions[name] = val[name] 
 332          else: 
 333              raise TypeError("Invalid ICs type -- must be dict of varname, value pairs")

 334  
 
 335  
 


336 -    def setEventDelay(self, eventTarget, val):


337          if isinstance(val, int) or isinstance(val, float): 
 338              if isinstance(eventTarget, list): 
 339                  for evTarg in intersect(eventTarget, self.events.keys()): 
 340                      self.events[evTarg].eventdelay = val 
 341              else: 
 342                  if eventTarget in self.events.keys(): 
 343                      self.events[eventTarget].eventdelay = val 
 344          else: 
 345              raise TypeError("Invalid eventdelay type/value")

 346  
 


347 -    def setEventInterval(self, eventTarget, val):


348          if isinstance(val, int) or isinstance(val, float): 
 349              if isinstance(eventTarget, list): 
 350                  for evTarg in intersect(eventTarget, self.events.keys()): 
 351                      self.events[evTarg].eventinterval = val 
 352              else: 
 353                  if eventTarget in self.events.keys(): 
 354                      self.events[eventTarget].eventinterval = val 
 355          else: 
 356              raise TypeError("Invalid eventinterval type/value")

 357  
 


358 -    def setEventTol(self, eventTarget, val):


359          if val > 0: 
 360              if isinstance(eventTarget, list): 
 361                  for evTarg in intersect(eventTarget, self.events.keys()): 
 362                      self.events[evTarg].eventtol = val 
 363              else: 
 364                  if eventTarget in self.events.keys(): 
 365                      self.events[eventTarget].eventtol = val 
 366          else: 
 367              raise TypeError("Invalid eventtol type/value")

 368  
 


369 -    def setEventDir(self, eventTarget, val):


370          if val in [-1,0,1] and isinstance(val, int): 
 371              if isinstance(eventTarget, list): 
 372                  for evTarg in intersect(eventTarget, self.events.keys()): 
 373                      self.events[evTarg].dircode = val 
 374              else: 
 375                  if eventTarget in self.events.keys(): 
 376                      self.events[eventTarget].dircode = val 
 377          else: 
 378              raise TypeError("Invalid eventdir type/value")

 379  
 


380 -    def setStartTime(self, eventTarget, val):


381          if isinstance(val, int) or isinstance(val, float): 
 382              if isinstance(eventTarget, list): 
 383                  for evTarg in intersect(eventTarget, self.events.keys()): 
 384                      self.events[evTarg].starttime = val 
 385              else: 
 386                  if eventTarget in self.events.keys(): 
 387                      self.events[eventTarget].starttime = val 
 388          else: 
 389              raise TypeError("Invalid starttime type")

 390  
 
 391  
 


392 -    def setBisect(self, eventTarget, val):


393          if isinstance(val, int) and val > 0: 
 394              if isinstance(eventTarget, list): 
 395                  for evTarg in intersect(eventTarget, self.events.keys()): 
 396                      self.events[evTarg].bisectlimit = val 
 397              else: 
 398                  if eventTarget in self.events.keys(): 
 399                      self.events[eventTarget].bisectlimit = val 
 400          else: 
 401              raise TypeError("Invalid bisectlimit type/value")

 402  
 
 403  
 
 404  
 


405 -class Event(object):


406      """Generic Event.
 
 407  
 
 408      Possible keys in argument dictionary at initialization:
 
 409          name, eventtol, eventdelay, starttime, bisectlimit, term, active,
 
 410          precise, vars, expr.
 
 411      """ 
 412  
 


413 -    def __init__(self, kw):


414          if 'name' in kw: 
 415              self.name = kw['name'] 
 416          else: 
 417              raise KeyError('Name must be supplied to event') 
 418          # absolute tolerance for event location (in dependent variable/expression)
 
 419          if 'eventtol' in kw: 
 420              self.eventtol = kw['eventtol'] 
 421          else: 
 422              self.eventtol = 1e-9 
 423          # time interval before event detection begins on each run
 
 424          if 'eventdelay' in kw: 
 425              self.eventdelay = kw['eventdelay'] 
 426          else: 
 427              self.eventdelay = 1e-3 
 428          # time interval between event detections restart
 
 429          if 'eventinterval' in kw: 
 430              self.eventinterval = kw['eventinterval'] 
 431          else: 
 432              self.eventinterval = 1e-3 
 433          # number of bisection steps to take when finding events
 
 434          if 'bisectlimit' in kw: 
 435              self.bisectlimit = kw['bisectlimit'] 
 436          else: 
 437              self.bisectlimit = 100 
 438          # terminating event flag
 
 439          if 'term' in kw: 
 440              self.termFlag = kw['term'] 
 441          else: 
 442              self.termFlag = False 
 443          # active event flag
 
 444          if 'active' in kw: 
 445              self.activeFlag = kw['active'] 
 446          else: 
 447              self.activeFlag = True 
 448          # determines whether event must be computed precisely
 
 449          if 'precise' in kw: 
 450              self.preciseFlag = kw['precise'] 
 451          else: 
 452              self.preciseFlag = True 
 453          # store 'plain text' definition of event as string, if provided
 
 454          if 'expr' in kw: 
 455              assert isinstance(kw['expr'],str), \
 
 456                      "Invalid type for event definition string" 
 457              self._expr = kw['expr'] 
 458          else: 
 459              self._expr = None 
 460          # list of error structures
 
 461  ##        self.errors = []
 
 462          try: 
 463              self.dircode = kw['dircode'] 
 464              assert self.dircode in [-1, 0, 1]  # direction codes 
 465          except AssertionError: 
 466              print 'invalid value for direction code -- must be -1, 0, or 1' 
 467              raise 
 468          except KeyError: 
 469              self.dircode = 0 
 470          # effective time zero for searches (used for eventdelay)
 
 471          if 'starttime' in kw: 
 472              self.starttime = kw['starttime'] 
 473          else: 
 474              self.starttime = 0 
 475          # optional variable and parameter bounds information, in case an event
 
 476          # wishes to refer to them
 
 477          if 'xdomain' in kw: 
 478              assert type(kw['xdomain'])==dict, \
 
 479                      "Invalid type for variable bounds information" 
 480              self.xdomain = kw['xdomain'] 
 481          else: 
 482              self.xdomain = {} 
 483          if 'pdomain' in kw: 
 484              assert type(kw['pdomain'])==dict, \
 
 485                      "Invalid type for parameter bounds information" 
 486              self.pdomain = kw['pdomain'] 
 487          else: 
 488              self.pdomain = {} 
 489          # var dictionary (can be just dict of keys for individual
 
 490          # value calls only) -- only for purely high level events
 
 491          if 'vars' in kw: 
 492              self.vars = kw['vars'] 
 493              assert len(self.vars) > 0, 'vars dictionary must be non-empty' 
 494              if reduce(bool.__and__, [isinstance(var, Variable) for \
 
 495                                          var in self.vars.itervalues()]): 
 496                  self.varlinked = True 
 497                  # doesn't check that only argument is spec'd for _fn method
 
 498              else: 
 499                  self.varlinked = False 
 500          else: 
 501              raise KeyError('vars dictionary not present') 
 502          # _funcreg is a register of dynamically created Event method names
 
 503          # in case of object copying (requiring destruction and re-creation
 
 504          # of dynamically created methods)
 
 505          self._funcreg = [] 
 506          self._funcstr = kw['funcspec'][0] 
 507          self._funcname = kw['funcspec'][1] 
 508          if 'auxfnspec' in kw: 
 509              self._fnspecs = ensureStrArgDict(kw['auxfnspec']) 
 510          else: 
 511              self._fnspecs = {} 
 512          if 'noHighLevel' in kw: 
 513              # Boolean to indicate whether the non-Python event type can make
 
 514              # a high-level image of the event function
 
 515              if not kw['noHighLevel']: 
 516                  self.addMethods() 
 517          else: 
 518              # assume is high level
 
 519              self.addMethods() 
 520          if 'prevsign_IC' in kw: 
 521              # set initial value -- useful for one-off tests or as part of
 
 522              # map-based hybrid models with only one integer timestep
 
 523              self.prevsign = kw['prevsign_IC'] 
 524              self.prevsign_IC = self.prevsign 
 525          else: 
 526              self.prevsign = None 
 527              self.prevsign_IC = None 
 528          # additional history for previous sign, in case of resetting
 
 529          self.prevprevsign = None 
 530          self.fval = None 
 531          # placeholder for variables' initial conditions. If events use
 
 532          # auxiliary functions that access 'initcond' auxiliary function
 
 533          # then caller had better assign the current initial conditions
 
 534          # to this dictionary first.
 
 535          self.initialconditions = {} 
 536          # placeholder for global independent variable value, for use in
 
 537          # hybrid systems
 
 538          self.globalt0 = 0 
 539          # event queue information (used for discrete delays, recording
 
 540          # features, etc.)
 
 541          self.queues = {} 
 542          self._sorted_queues = [] 
 543          # event internal parameter information (for temp usage by
 
 544          # event mappings)
 
 545          self.evpars = {} 
 546          if 'evpars' in kw: 
 547              self.evpars.update(kw['evpars']) 
 548          # used for quadratic interpolation, if requested in searchForEvents
 
 549          self.quadratic = None  #fit_quadratic()

 550  
 
 551  
 
 552      # Queues are intended to be an ordered sequence of numeric types, e.g.
 
 553      # for times at which next terminal event should occur.
 
 554      # Queues are first in, first out, and may be sorted or not.
 
 555      # !! This feature is in development.
 


556 -    def addToQ(self, qname, item):


557          try: 
 558              self.queues[qname].append(item) 
 559          except KeyError: 
 560              raise PyDSTool_ExistError("Queue %s was not declared"%qname) 
 561          else: 
 562              if qname in self._sorted_queues: 
 563                  self.queues[qname].sort()

 564  
 


565 -    def createQ(self, qname, sorted=True, seq=None):


566          """Also use to reset a queue.""" 
 567          if seq is None: 
 568              self.queues[qname] = [] 
 569          else: 
 570              # ensure list argument (for pop to work)
 
 571              self.queues[qname] = list(seq) 
 572          if sorted: 
 573              self._sorted_queues.append(qname)

 574  
 


575 -    def popFromQ(self, qname):


576          return self.queues[qname].pop(0)

 577  
 


578 -    def deleteQ(self, qname):


579          if qname in self._sorted_queues: 
 580              i = self._sorted_queues.index(qname) 
 581              del self._sorted_queues[i] 
 582          try: 
 583              del self.queues[qname] 
 584          except KeyError: 
 585              pass

 586  
 


587 -    def _infostr(self, verbose=1):


588          dirstr = ["decreasing", "either", "increasing"] 
 589          if verbose <= 0: 
 590              outputStr = "Event "+self.name 
 591          elif verbose > 0: 
 592              outputStr = "Event "+self.name+"\n" + \
 
 593                          "  active: " + str(self.activeFlag) + "\n" + \
 
 594                          "  terminal: " + str(self.termFlag) + "\n" + \
 
 595                          "  precise: " + str(self.preciseFlag) + "\n" + \
 
 596                          "  direction: " + dirstr[self.dircode+1] + "\n" + \
 
 597                          "  event tol: " + str(self.eventtol) + "\n" + \
 
 598                          "  definition: " + self._expr 
 599              if verbose >= 2: 
 600                  outputStr += "\n  bisect limit: " + str(self.bisectlimit) \
 
 601                                  + "\n" + \
 
 602                                  "  event delay: " + str(self.eventdelay) + "\n" +\
 
 603                                  "  event interval: " + str(self.eventinterval) 
 604          return outputStr

 605  
 
 606  
 


607 -    def info(self, verboselevel=1):


608          print self._infostr(verboselevel)

 609  
 
 610  
 


611 -    def __repr__(self):


612          return self._infostr(verbose=0)

 613  
 
 614      __str__ = __repr__ 
 615  
 
 616  
 


617 -    def addMethods(self):


618          # clean up FuncSpec usage of parsinps and x for pars/inputs and
 
 619          # variables
 
 620          try: 
 621              exec self._funcstr 
 622          except: 
 623              print 'Invalid event function definition:' 
 624              print self._funcstr 
 625              raise 
 626          try: 
 627              setattr(self, '_fn', types.MethodType(locals()[self._funcname],
 
 628                                                      self, self.__class__)) 
 629          except KeyError: 
 630              print 'Must pass objective function for event at initialization' 
 631              raise 
 632          if '_fn' not in self._funcreg: 
 633              self._funcreg.append('_fn') 
 634          # _fnspecs keys are convenient, user-readable, short-hand names
 
 635          # for the functions, whose actual function names are the second entry
 
 636          # of the pair funcstr
 
 637          for funcpair in self._fnspecs.values(): 
 638              # clean up FuncSpec usage of parsinps and x for pars/inputs and
 
 639              # variables
 
 640              try: 
 641                  exec funcpair[0] 
 642              except: 
 643                  print 'Invalid auxiliary function definition:' 
 644                  print funcpair[0] 
 645                  raise 
 646              try: 
 647                  setattr(self, funcpair[1], types.MethodType(locals()[funcpair[1]],
 
 648                                                              self,
 
 649                                                              self.__class__)) 
 650              except KeyError: 
 651                  print 'Must pass objective function for event at initialization' 
 652                  raise 
 653              if funcpair[1] not in self._funcreg: 
 654                  self._funcreg.append(funcpair[1])

 655  
 
 656  
 


657 -    def reset(self, state=None):


658          """Reset event`s prevsign attribute to a certain state (defaults to None)
 
 659          """ 
 660          self.fval = None 
 661          if state is None or self.dircode == 0: 
 662              self.prevsign = self.prevsign_IC 
 663          elif state == 'prev': 
 664              self.prevsign = self.prevprevsign 
 665          elif state == 'on': 
 666              self.prevsign = self.dircode 
 667          elif state == 'off': 
 668              self.prevsign = -self.dircode 
 669          else: 
 670              raise ValueError("Invalid state passed to event reset method")

 671  
 
 672  
 


673 -    def __call__(self, t=None, varDict=None, parDict=None):


674          """Report on correct sign change.
 
 675          For external inputs, add input names and vales at time t to parDict
 
 676          """ 
 677          assert self.activeFlag, "Event cannot be called when inactivated" 
 678          if varDict is None: 
 679              if t is not None: 
 680                  assert self.varlinked, ('wrong type of call for non var-'
 
 681                                              ' linked event') 
 682                  if t < self.starttime + self.eventdelay: 
 683                      return False 
 684              else: 
 685                  raise ValueError('t must be specified for this type of call') 
 686              if self.prevsign is None: 
 687                  try: 
 688                      self.fval = self._fn(t, parDict) 
 689                  except: 
 690                      print "Error in event", self.name 
 691                      info(parDict, "Parameters") 
 692                      raise 
 693                  self.prevsign = scipy.sign(self.fval) 
 694                  self.prevprevsign = None 
 695                  return False 
 696              else: 
 697                  try: 
 698                      self.fval = self._fn(t, parDict) 
 699                  except: 
 700                      print "Error in event", self.name 
 701                      info(parDict, "Parameters") 
 702                      raise 
 703                  sval = scipy.sign(self.fval) 
 704                  if self.dircode == 0: 
 705                      result = self.prevsign != sval 
 706                  else: 
 707                      result = self.prevsign != sval and \
 
 708                              self.prevsign * self.dircode < 0 
 709                  self.prevprevsign = self.prevsign 
 710                  self.prevsign = sval 
 711                  return result 
 712          else: 
 713              # then calling as individual value, not var object
 
 714              varDict_temp = dict(varDict) 
 715              if t is None: 
 716                  t = varDict['t'] 
 717              else: 
 718                  assert 't' not in varDict, ('`t` key already present in '
 
 719                                          'varDict argument. Cannot redefine.') 
 720                  varDict_temp['t'] = t 
 721              if t < self.starttime + self.eventdelay: 
 722                  return False 
 723              assert not self.varlinked, ('wrong type of call for var-linked'
 
 724                                          ' event') 
 725              if self.prevsign is None: 
 726                  try: 
 727                      self.fval = self._fn(varDict_temp, parDict) 
 728                  except: 
 729                      print "Error in event", self.name 
 730                      info(parDict, "Parameters") 
 731                      print "\n" 
 732                      info(varDict_temp, "Variables") 
 733                      raise 
 734                  self.prevsign = scipy.sign(self.fval) 
 735                  self.prevprevsign = None 
 736                  return False 
 737              else: 
 738                  try: 
 739                      self.fval = self._fn(varDict_temp, parDict) 
 740                  except: 
 741                      print "Error in event", self.name 
 742                      info(parDict, "Parameters") 
 743                      print "\n" 
 744                      info(varDict_temp, "Variables") 
 745                      raise 
 746                  sval = scipy.sign(self.fval) 
 747                  if self.dircode == 0: 
 748                      result = self.prevsign != sval 
 749                  else: 
 750                      result = self.prevsign != sval and \
 
 751                              self.prevsign * self.dircode < 0 
 752                  self.prevprevsign = self.prevsign 
 753                  self.prevsign = sval 
 754                  return result

 755  
 
 756  
 


757 -    def searchForEvents(self, trange=None, dt=None, checklevel=2,
 
 758                          parDict=None, vars=None, inputs=None,
 
 759                          abseps=1e-13, eventdelay=True, globalt0=0):


760          """Search a variable-linked event, or an event with supplied vars
 
 761          dictionary and relevant parameters, for zero crossings.
 
 762  
 
 763          (Variable-linked search not applicable to low level events.)
 
 764  
 
 765          trange=None, dt=None, checklevel=2, parDict=None, vars=None, inputs=None,
 
 766              abseps=1e-13, eventdelay=True -> (ev_t, (ev_tlo, ev_thi))
 
 767          where the lo-hi tuple is the smallest bound around ev_t (in
 
 768          case it is None because event was not found accurately).
 
 769  
 
 770          dt will default to 1e-3 * the time interval of the variables.
 
 771          'eventinterval' inherited from the event will be used to separate
 
 772          detected events.
 
 773  
 
 774          Only pass vars dictionary when event.varlinked is False.
 
 775          """ 
 776  
 
 777          # flags indicating whether continuous and discrete variables are present
 
 778          discretevars = False 
 779          continuousvars = False 
 780          if self.varlinked: 
 781              assert vars is None, ("event is variable-linked already -- "
 
 782                                      "do not pass vars argument") 
 783              varDict = self.vars 
 784          else: 
 785              assert vars is not None, ("event is not variable-linked "
 
 786                                          "already -- require a vars argument") 
 787              varDict = vars 
 788          precise = self.preciseFlag 
 789          try: 
 790              for var in varDict.itervalues(): 
 791                  if isdiscrete(var): 
 792                      discretevars = True 
 793                  elif iscontinuous(var): 
 794                      continuousvars = True 
 795                  else: 
 796                      raise TypeError('varDict must consist of Variable objects') 
 797          except AttributeError: 
 798              print 'event must contain a dictionary of vars' 
 799              raise 
 800          if discretevars and continuousvars: 
 801              raise TypeError('Cannot mix discrete and continuous Variable types') 
 802          if discretevars: 
 803              if precise: 
 804                  print 'argument precise cannot be used for discrete Variable objects' 
 805                  precise = False 
 806              varnames = varDict.keys() 
 807              if dt is not None: 
 808                  print 'argument dt is unused for discrete Variable objects' 
 809              if trange is not None: 
 810                  # trange had better be contained in all var.indepdomain ranges
 
 811                  assert len(varDict) > 0, 'varDict was empty!' 
 812                  if not reduce(bool.__and__, [trange[0] in var.indepdomain \
 
 813                                  and trange[1] in var.indepdomain for var \
 
 814                                  in varDict.itervalues()]): 
 815                      raise ValueError('trange not contained in all var ranges') 
 816              else: 
 817                  raise ValueError('trange must be defined for discrete'
 
 818                                      ' Variable objects') 
 819              tlist = varDict[varnames[0]].indepdomain 
 820              tlist = tlist[tlist.index(trange[0]):tlist.index(trange[1])] 
 821          elif continuousvars: 
 822              # trange had better be contained in all var.indepdomain ranges
 
 823              tlimits = None  # initial value 
 824              for var in varDict.itervalues(): 
 825                  if tlimits is None: 
 826                      tlimits = var.indepdomain.get() 
 827                  else: 
 828                      temp = var.indepdomain.get() 
 829                      if temp[0] < tlimits[0]: 
 830                          tlimits[0] = temp[0] 
 831                      if temp[1] > tlimits[1]: 
 832                          tlimits[1] = temp[1] 
 833              if trange is not None: 
 834                  # compare trange and tlimits using Interval inclusion,
 
 835                  # so that can make use of uncertain values at boundaries
 
 836                  trange_int = Interval('trange', float, trange, abseps) 
 837                  tlimits_int = Interval('tlimits', float, tlimits, abseps) 
 838                  if not self.contains(tlimits_int, trange_int, checklevel): 
 839                      raise ValueError('trange not contained in all var ranges') 
 840              else: 
 841                  trange = tlimits 
 842              if dt is None: 
 843                  dt = max(self.eventtol, 1e-3*(trange[1]-trange[0])) 
 844              if dt > trange[1]-trange[0]: 
 845                  raise ValueError('dt (eventtol if not specified) is too large'
 
 846                                   ' for trange in event %s'%self.name) 
 847              ttemp = copy.copy(var.indepdomain) 
 848              ttemp.set(trange) 
 849              tlist = ttemp.sample(dt, avoidendpoints=True) 
 850          else: 
 851              raise TypeError('var must be a Variable object') 
 852          if inputs is None: 
 853              test_fn = self._fn 
 854              self_caller = self.__call__ 
 855          else: 
 856              def test_fn(ts, ps): 
 857                  if ps is None: 
 858                      pidict = {} 
 859                  else: 
 860                      pidict = copy.copy(ps) 
 861                  try: 
 862                      for t in ts: 
 863                          idict = dict([(n,i(t+globalt0)) for n,i in inputs.iteritems()]) 
 864                          pidict.update(idict) 
 865                          fvals.append(self._fn(t, pidict)) 
 866                  except TypeError: 
 867                      # Iteration over non sequence, so assume ts is just a single
 
 868                      # numeric value
 
 869                      try: 
 870                          tvals = array(ts)+globalt0 
 871                      except: 
 872                          print "\n Found type", type(ts) 
 873                          raise TypeError("t values invalid") 
 874                      idict = dict([(n,i(tvals)) for n,i in inputs.iteritems()]) 
 875                      pidict.update(idict) 
 876                      fvals = self._fn(ts, pidict) 
 877                  return fvals

 878              def self_caller(t=None, varDict=None, parDict=None): 
 879                  if parDict is None: 
 880                      pidict = dict([(n,i(t+globalt0)) for n,i in inputs.iteritems()]) 
 881                  else: 
 882                      pidict = copy.copy(parDict) 
 883                      pidict.update(dict([(n,i(t+globalt0)) for n,i in inputs.iteritems()])) 
 884                  return self(t, varDict=varDict, parDict=pidict)

 885          # before search, check that start after 'eventdelay', if option set
 
 886          if eventdelay: 
 887              while tlist[0] <= self.eventdelay: 
 888                  try: 
 889                      tlist.pop(0) 
 890                  except IndexError: 
 891                      raise ValueError('eventdelay too large -- '
 
 892                                          'no search interval') 
 893          # now do the search
 
 894          try: 
 895              if self.varlinked: 
 896                  try: 
 897                      fvals = test_fn(tlist, parDict) 
 898                      boollist = [False]  # initial point is always False 
 899                      if self.dircode == 0: 
 900                          boollist.extend([fvals[i] != fvals[i+1] for i in \
 
 901                                              range(len(fvals)-1)]) 
 902                      else: 
 903                          boollist.extend([fvals[i] * fvals[i+1] < 0 and \
 
 904                              fvals[i] * self.dircode < 0 for i in \
 
 905                                              range(len(fvals)-1)]) 
 906                  except: 
 907                      # event does not support vectorized t calls
 
 908  ##                    print "Warning: event did not support vectorized t calls"
 
 909                      # set start time for self-calling purposes
 
 910                      self.starttime = trange[0] 
 911                      boollist = [self_caller(t, parDict=parDict) for t in tlist] 
 912                      # first bool may be True because event hasn't been called
 
 913                      # before and has prevsign unset (or mis-set)...
 
 914                      # we can safely overwrite it (bit of a hack) because
 
 915                      # it's always really False
 
 916                      boollist[0] = False 
 917              else: 
 918                  # vallist is a list of 1D lists
 
 919                  vallist = [v(tlist) for v in sortedDictValues(varDict)] 
 920                  varnames = sortedDictKeys(varDict) 
 921                  # set start time for self-calling purposes
 
 922                  self.starttime = trange[0] 
 923                  if not eventdelay: 
 924                      # switch off event delay temporarily
 
 925                      restore_val = self.eventdelay 
 926                      self.eventdelay = 0. 
 927                  boollist = [] 
 928                  for tix in xrange(len(tlist)): 
 929                      boollist.append(self_caller(t=tlist[tix],
 
 930                                              varDict=dict(zip(varnames,
 
 931                                                              [vallist[i][tix] for\
 
 932                                                      i in range(len(vallist))])),
 
 933                                              parDict=parDict)) 
 934                  # first bool may be True because event hasn't been called
 
 935                  # before and has prevsign unset (or mis-set)...
 
 936                  # we can safely overwrite it (bit of a hack) because
 
 937                  # it's always really False
 
 938                  boollist[0] = False 
 939                  if not eventdelay: 
 940                      # restore value for future use
 
 941                      self.eventdelay = restore_val 
 942          except KeyError, e: 
 943              # for discretevars if not all var.indepdomains equal over trange
 
 944              if discretevars: 
 945                  print 'Note: All discrete Variables must have identical ' \
 
 946                          'independent variable domains over trange' 
 947              print "Check that all variable references in events are legitimate" 
 948              raise 
 949          # loop through boollist and find all events unless terminating at tpos!
 
 950          tpos = -1 
 951          eventsfound = [] 
 952          t_last = -numpy.inf 
 953          t_interval = self.eventinterval 
 954          while True: 
 955              try: 
 956                  tpos += boollist[tpos+1:].index(True)+1 
 957              except (ValueError, IndexError): 
 958                  # no event found in this slice, at this dt
 
 959                  break 
 960              if precise and continuousvars: 
 961                  if self.varlinked: 
 962                      result = findpreciseroot(self, tlist[tpos-1], tlist[tpos],
 
 963                                                parDict, inputs=inputs,
 
 964                                                globalt0=globalt0,
 
 965                                                quadratic_interp=self.quadratic) 
 966                  else: 
 967                      result = findpreciseroot(self, tlist[tpos-1], tlist[tpos],
 
 968                                                parDict, varDict, inputs,
 
 969                                                globalt0=globalt0,
 
 970                                                quadratic_interp=self.quadratic) 
 971                  if result is not None and result[0] > t_last + t_interval: 
 972                      eventsfound.append(result) 
 973                      t_last = result[0] 
 974              else: 
 975                  result = tlist[tpos] 
 976                  if result > t_last + t_interval: 
 977                      eventsfound.append((result, (tlist[tpos-1], result))) 
 978                      t_last = result 
 979              if self.termFlag:  # quit searching now! 
 980                  break 
 981          return eventsfound 
 982  
 
 983  
 


984 -    def contains(self, interval, val, checklevel=2):


985          # NB. val may be another interval
 
 986          if checklevel == 0: 
 987              # level 0 -- no bounds checking at all
 
 988              # code should avoid calling this function with checklevel = 0
 
 989              # if possible, but this case is left here for completeness and
 
 990              # consistency
 
 991              return True 
 992          elif checklevel in [1,2]: 
 993              # level 1 -- ignore uncertain cases (treat as contained)
 
 994              # level 2 -- warn on uncertain and continue (but warnings not
 
 995              #  used in Events, so treat as #1)
 
 996              if interval.contains(val) is not notcontained: 
 997                  return True 
 998              else: 
 999                  return False 
1000          else: 
1001              # level 3 -- exception will be raised for uncertain case
 
1002              if val in interval: 
1003                  return True 
1004              else: 
1005                  return False

1006  
 


1007 -    def __getstate__(self):


1008          d = copy.copy(self.__dict__) 
1009          for fname in self._funcreg: 
1010              del d[fname] 
1011          return d

1012  
 


1013 -    def __setstate__(self, state):


1014          self.__dict__.update(state) 
1015          self.addMethods()

1016  
 


1017 -    def __copy__(self):


1018          pickledself = pickle.dumps(self) 
1019          return pickle.loads(pickledself)

1020  
 


1021 -    def __deepcopy__(self, memo=None, _nil=[]):


1022          pickledself = pickle.dumps(self) 
1023          return pickle.loads(pickledself)

1024  
 
1025  
 
1026  
 


1027 -class HighLevelEvent(Event):


1028      """Event defined using python function code.
 
1029  
 
1030      First argument to function must always be 'self'
 
1031      """ 


1032 -    def __init__(self, kw):


1033          assert 'LLfuncspec' not in kw, \
 
1034              "'LLfuncspec' key is invalid for purely high level events" 
1035          Event.__init__(self, kw)

1036  
 
1037  
 


1038 -class LowLevelEvent(Event):


1039      """Event defined using externally-compiled and linked function code (i.e. C)
 
1040  
 
1041      Specification of the function body as a string must include
 
1042      necessary temporary variable declarations and a return statement.""" 


1043 -    def __init__(self, kw):


1044          if 'vars' in kw: 
1045              for v in kw['vars'].itervalues(): 
1046                  if v is not None: 
1047                      raise TypeError("Low level events cannot be linked to a"
 
1048                                      " variable") 
1049          Event.__init__(self, kw) 
1050          assert isinstance(kw['LLfuncspec'], str), ("For low level events, must "
 
1051                                  "pass string for 'LLfuncspec' in initialization") 
1052          LLfuncstr = kw['LLfuncspec'] 
1053          dummyQ = QuantSpec('dummy', LLfuncstr, preserveSpace=True) 
1054          dummyQ.mapNames({'abs': 'fabs', 'sign': 'signum', 'mod': 'fmod'}) 
1055          if dummyQ[0] != 'return': 
1056              print "Found: ", dummyQ[0] 
1057              print "in event specification: ", dummyQ() 
1058              raise ValueError("'return' must be first token in low level "
 
1059                                  "event specification") 
1060          # take out 'return' from tokenized because whitespace will be lost
 
1061          # (parser not designed for non-math expressions)
 
1062          self._LLfuncstr = "return " + "".join(dummyQ[1:]) 
1063          self._LLfuncname = self.name 
1064          self._LLreturnstr = "double" 
1065          self._LLargstr = "(unsigned n_, double t, double *Y_, double *p_, unsigned wkn_, double *wk_, unsigned xvn_, double *xv_)" 
1066          self.varlinked = False  # for compatibility with query calls

1067  
 
1068  
 


1069 -class MatlabEvent(LowLevelEvent):


1070      """Event defined using MATLAB syntax for use with ADMC++
 
1071  
 
1072      Specification of the function body as a string must include
 
1073      necessary temporary variable declarations.""" 


1074 -    def __init__(self, kw):


1075          if 'vars' in kw: 
1076              for v in kw['vars'].itervalues(): 
1077                  if v is not None: 
1078                      raise TypeError("Low level events cannot be linked to a variable") 
1079          kw['noHighLevel'] = True 
1080          Event.__init__(self, kw) 
1081          assert isinstance(kw['Matlabfuncspec'], str), ("For low level events, must "
 
1082                                  "pass string for 'LLfuncspec' in initialization") 
1083          LLfuncstr = kw['Matlabfuncspec'] 
1084          dummyQ = QuantSpec('dummy', LLfuncstr, preserveSpace=True) 
1085          if dummyQ[0] != 'return': 
1086              print "Found: ", dummyQ[0] 
1087              print "in event specification: ", dummyQ() 
1088              raise ValueError("'return' must be first token in low level "
 
1089                                  "event specification") 
1090          # take out 'return' from tokenized because whitespace will be lost
 
1091          # (parser not designed for non-math expressions)
 
1092          self._LLfuncstr = "Y_ =  " + "".join(dummyQ[1:]) 
1093          self._LLfuncname = self.name 
1094          self._LLreturnstr = "Y_ = " 
1095          self._LLargstr = "(t_, x_, p_)" 
1096          self.varlinked = False  # for compatibility with query calls

1097  
 
1098  
 
1099  # -------------------------------------------------------------
 
1100  
 
1101  ## Public exported functions
 


1102 -def makeZeroCrossEvent(expr, dircode, argDict, varnames=[], parnames=[],
 
1103                          inputnames=[], fnspecs={}, targetlang='python',
 
1104                          reuseterms={}, flatspec=None):


1105      """Target language-independent user-defined event involving coordinates,
 
1106      parameters, and time. Returns a non variable-linked event only.
 
1107  
 
1108      List of used variable, parameter, and input names defaults to
 
1109      the empty list and can be omitted if these are not referenced. If
 
1110      variable names are omitted then the expression must only depend on time
 
1111      't' and any declared parameters.
 
1112  
 
1113      Auxiliary function dictionary is required if the event accesses them.
 
1114  
 
1115      'targetlang' argument defaults to 'python'.
 
1116      The expression 'expr' may not use intermediate temporary variables (for
 
1117      which you should specify the body of the event function by hand.
 
1118  
 
1119      Optional argument reuseterms is a dictionary of terms used in expr
 
1120      that map to their definitions in terms of the state variables, time,
 
1121      parameters, and inputs.
 
1122      """ 
1123      # preprocess expression for varnames -> v['varname'] and similarly
 
1124      # for parameter names
 
1125      try: 
1126          exprname = argDict['name'] 
1127      except KeyError: 
1128          raise PyDSTool_KeyError("name key must be present in argDict") 
1129      # support ModelSpec definitions, so take str() of expr
 
1130      expQS = QuantSpec('__ev_expr__', str(expr)) 
1131      # make copies of arguments to prevent defaults getting messed up
 
1132      varnames = copy.copy(varnames) 
1133      parnames = copy.copy(parnames) 
1134      inputnames = copy.copy(inputnames) 
1135      auxfns = copy.copy(fnspecs) 
1136      auxVarDefMap = {} 
1137      if flatspec is not None: 
1138          # transfer used definitions in flatpsec into varnames, parnames, auxfns
 
1139          # etc.
 
1140          assert varnames==parnames==[], 'flatspec argument requires that varnames and parnames lists are not used' 
1141          assert fnspecs=={}, 'flatspec argument requires that fnspecs is not used' 
1142          try: 
1143              allvars = flatspec['vars'] 
1144          except KeyError: 
1145              allvars = {} 
1146          try: 
1147              allinputs = flatspec['inputs'] 
1148          except KeyError: 
1149              allinputs = {} 
1150          try: 
1151              allpars = flatspec['pars'] 
1152          except KeyError: 
1153              allpars = {} 
1154  
 
1155          try: 
1156              allfuns = flatspec['auxfns'] 
1157          except KeyError: 
1158              allfuns = {} 
1159          collectReused(expQS, allvars, allpars, allinputs, allfuns,
 
1160                      varnames, parnames, inputnames, auxfns,
 
1161                      auxVarDefMap, flatspec) 
1162  ##        for symb in expQS.freeSymbols:
 
1163  ##            if symb in allvars:
 
1164  ##                spectype = flatspec['spectypes'][symb]
 
1165  ##                if spectype == 'RHSfuncSpec':
 
1166  ##                    if symb not in varnames:
 
1167  ##                        varnames.append(symb)
 
1168  ##                elif spectype == 'ExpFuncSpec':
 
1169  ##                    auxVarDef = allvars[symb]
 
1170  ##                    auxVarDefMap[symb] = auxVarDef
 
1171  ##                    # only need to look one level deeper because auxvars
 
1172  ##                    # cannot depend on each other, in a well-defined system.
 
1173  ##                    symbQS = QuantSpec('__symb_expr__', auxVarDef)
 
1174  ##                    for auxsymb in symbQS.freeSymbols:
 
1175  ##                        if auxsymb in allvars:
 
1176  ##                            if auxsymb not in varnames:
 
1177  ##                                varnames.append(auxsymb)
 
1178  ##                                spectype = flatspec['spectypes'][auxsymb]
 
1179  ##                                if spectype == 'ExpFuncSpec':
 
1180  ##                                    auxVarDefMap[auxsymb] = allvars[auxsymb]
 
1181  ##                        elif auxsymb in allpars:
 
1182  ##                            if auxsymb not in parnames:
 
1183  ##                                parnames.append(auxsymb)
 
1184  ##                        elif auxsymb in allinputs:
 
1185  ##                            if auxsymb not in inputnames:
 
1186  ##                                inputnames.append(auxsymb)
 
1187  ##                        elif auxsymb in allfuns:
 
1188  ##                            if auxsymb not in auxfns:
 
1189  ##                                auxfns[auxsymb] = allfuns[auxsymb]
 
1190  ##            elif symb in allpars:
 
1191  ##                if symb not in parnames:
 
1192  ##                    parnames.append(symb)
 
1193  ##            elif symb in allinputs:
 
1194  ##                if symb not in inputnames:
 
1195  ##                    inputnames.append(symb)
 
1196  ##            elif symb in allfuns:
 
1197  ##                if symb not in auxfns:
 
1198  ##                    auxfns[symb] = allfuns[symb]
 
1199  ##                    # Add any dependencies of the function
 
1200  ##                    symbQS = QuantSpec('__symb_expr__', allfuns[symb][1])
 
1201  ##                    for fnsymb in symbQS.freeSymbols:
 
1202  ##                        if fnsymb in allpars:
 
1203  ##                            if fnsymb not in parnames:
 
1204  ##                                parnames.append(fnsymb)
 
1205  ##                        if fnsymb in allinputs:
 
1206  ##                            if fnsymb not in inputnames:
 
1207  ##                                inputnames.append(fnsymb)
 
1208  ##                        elif fnsymb in allfuns:
 
1209  ##                            raise "Ask Rob to add support for function-to-function calls which he didn't anticipate here!"
 
1210      expr = '' 
1211      done = False 
1212      while not done: 
1213          expQS = expQS.eval(auxVarDefMap) 
1214          expr_new = str(expQS) 
1215          if expr_new == expr: 
1216              done = True 
1217          else: 
1218              expr = expr_new 
1219      # support ModelSpec definitions, so take str() of list elements
 
1220      varnames = [str(v) for v in varnames] 
1221      parnames = [str(p) for p in parnames] 
1222  ##    if inputnames != [] and targetlang != 'c':
 
1223  ##        raise NotImplementedError("Inputs to non C-based events are not "
 
1224  ##                                  "yet supported.")
 
1225      if exprname in varnames: 
1226          raise ValueError("Expression name %s coincides with a variable name"%exprname) 
1227      if exprname in parnames: 
1228          raise ValueError("Expression name %s coincides with a parameter name"%exprname) 
1229      if varnames == [] and inputnames == [] and 't' not in expQS: 
1230          raise ValueError("In absence of variable or input names, time must appear in expression: %s"%expr) 
1231      # zeros for t and varnames are unused placeholders. the names have
 
1232      # to be declared to funcspec with some value in order for the
 
1233      # object to be properly created. this is a kludge!
 
1234      specdict = {exprname: expr, 't': '0'} 
1235      specdict.update({}.fromkeys(varnames, '0')) 
1236      if 't' not in varnames: 
1237          varnames.append('t') 
1238      varnames.append(exprname) 
1239      # Thus, if Jacobian etc. listed in funcspec then need to make a copy of the auxfns
 
1240      # and filter these out
 
1241      auxfns_temp = filteredDict(auxfns, ['Jacobian', 'Jacobian_pars', 'massMatrix'],
 
1242                                 neg=True) 
1243      # Hack: targetLang must be python if (actually) c or python; matlab otherwise
 
1244      if targetlang == 'matlab': 
1245          dummytlang = 'matlab' 
1246      else: 
1247          dummytlang = 'python' 
1248      dummyfs = {'varspecs': specdict,
 
1249                 'name': exprname, 'vars': varnames,
 
1250                 'fnspecs': auxfns_temp,
 
1251                 'pars': parnames, 'inputs': inputnames,
 
1252                 'targetlang': dummytlang
 
1253                 } 
1254      dummyfuncspec = FuncSpec.RHSfuncSpec(dummyfs) 
1255      parsedstr = dummyfuncspec._specStrParse([exprname],
 
1256                                              dummyfuncspec.varspecs,
 
1257                                              noreturndefs=True,
 
1258                                              forexternal=True) 
1259      # alter par and var name dictionaries used by Events.py
 
1260      parsedstr = parsedstr.replace("parsinps[","p[").replace("x[","v[") 
1261      if 'parsinps' in parsedstr: 
1262          # then there are aux fns that use it
 
1263          funcstr = "parsinps=sortedDictValues(p,%s)+sortedDictValues(p,%s)"%(str(parnames),str(inputnames)) +\
 
1264                      "\n\treturn "+parsedstr+"\n" 
1265      else: 
1266          funcstr = "return "+parsedstr+"\n" 
1267      if reuseterms != {}: 
1268          illegalterms = ['globalindepvar', 'initcond', 'getindex', 'Jacobian',
 
1269                          'Jacobian_pars'] 
1270          reusestr, body_processed_dict = processReusedPy([exprname],
 
1271                                                  {exprname: funcstr},
 
1272                                                  copy.copy(reuseterms),
 
1273                                                  dummyfuncspec,
 
1274                                                  illegal=illegalterms) 
1275          funcstr_processed = (len(reusestr)>0)*"# local definitions\n" \
 
1276              + reusestr + (len(reusestr)>0)*"\n" \
 
1277              + body_processed_dict[exprname] 
1278      else: 
1279          funcstr_processed = funcstr 
1280      # find used variable names (assumes matching braces in supplied spec!)
 
1281      # so use original parsedstr (before being processed for reused terms)
 
1282      varnames_found = [] 
1283      currpos = 0 
1284      done = False 
1285      while not done: 
1286          relfindpos = parsedstr[currpos:].find("v[") 
1287          if relfindpos >= 0: 
1288              findpos = relfindpos + currpos 
1289              lbpos = findpos+1 
1290              rbpos = findEndBrace(parsedstr[lbpos:], '[', ']')+lbpos 
1291              varnames_found.append(parsedstr[lbpos+2:rbpos-1]) 
1292              currpos = rbpos 
1293          else: 
1294              done = True 
1295      inputnames_found = [] 
1296      currpos = 0 
1297      done = False 
1298      while not done and inputnames != []: 
1299          relfindpos = parsedstr[currpos:].find("p[") 
1300          if relfindpos >= 0: 
1301              findpos = relfindpos + currpos 
1302              lbpos = findpos+1 
1303              rbpos = findEndBrace(parsedstr[lbpos:], '[', ']')+lbpos 
1304              in_name = parsedstr[lbpos+2:rbpos-1] 
1305              if in_name in inputnames: 
1306                  inputnames_found.append(in_name) 
1307              currpos = rbpos 
1308          else: 
1309              done = True 
1310      newargs = ['dircode', 'funcspec', 'auxfnspec'] 
1311      if targetlang in ['c', 'C']: 
1312          newargs.append('LLfuncspec') 
1313          # convert any special C-specific functions
 
1314          expr = dummyfuncspec._processSpecialC(expr) 
1315      elif targetlang == 'matlab': 
1316          newargs.append('Matlabfuncspec') 
1317          # convert any special C-specific functions
 
1318          expr = dummyfuncspec._processSpecialC(expr) 
1319      for arg in newargs: 
1320          if arg in argDict: 
1321              print 'Warning: `' + arg + '` already appears in argDict!' 
1322              print '  This value will be overwritten.' 
1323      argDict_out = copy.copy(argDict) 
1324      funcname = "_f_"+exprname+"_ud" 
1325      if parnames == []: 
1326          pdefstr = "=None" 
1327      else: 
1328          pdefstr = "" 
1329      # 'ds' plays role of 'self' (named for compatibility with FuncSpec's
 
1330      # automatic name resolution of auxiliary functions during parsing.
 
1331      if targetlang == 'matlab': 
1332          funcstr_full = funcstr_processed 
1333      else: 
1334          funcstr_full = makeUniqueFn("def "+funcname+"(ds, v, p%s):\n\t"%pdefstr + \
 
1335                                      funcstr_processed, idstr="event") 
1336      if varnames_found+inputnames_found != []: 
1337          argDict_out['vars'] = {}.fromkeys(varnames_found+inputnames_found, None) 
1338      argDict_out.update({'funcspec': funcstr_full,
 
1339                      'auxfnspec': dummyfuncspec.auxfns,
 
1340                      'expr': expr,
 
1341                      'dircode': dircode}) 
1342      if targetlang in ['c', 'C']: 
1343          LLfuncstr = "return "+expr+";\n" 
1344          if reuseterms != {}: 
1345  ##          illegalterms = ['globalindepvar', 'initcond', 'getindex', 'Jacobian',
 
1346  ##                          'Jacobian_pars']
 
1347              LLreusestr, LLbody_processed_dict = processReusedC(['ev'],
 
1348                                                          {'ev': LLfuncstr},
 
1349                                                          copy.copy(reuseterms)) #, 
1350  ##                                                       illegal=illegalterms)
 
1351              LLfuncstr_processed = (len(LLreusestr)>0)*"/* local definitions */\n" \
 
1352                  + LLreusestr + (len(LLreusestr)>0)*"\n" + LLbody_processed_dict['ev'] 
1353          else: 
1354              LLfuncstr_processed = LLfuncstr 
1355          argDict_out['LLfuncspec'] = LLfuncstr_processed 
1356          return LowLevelEvent(argDict_out) 
1357      elif targetlang == 'matlab': 
1358          LLfuncstr = "return "+expr+";\n" 
1359          if reuseterms != {}: 
1360              LLreusestr, LLbody_processed_dict = processReusedMatlab(['ev'],
 
1361                                                          {'ev': LLfuncstr},
 
1362                                                          copy.copy(reuseterms)) 
1363  
 
1364              LLfuncstr_processed = (len(LLreusestr)>0)*"% local definitions \n" \
 
1365                  + LLreusestr + (len(LLreusestr)>0)*"\n" + LLbody_processed_dict['ev'] 
1366          else: 
1367              LLfuncstr_processed = LLfuncstr 
1368          argDict_out['Matlabfuncspec'] = LLfuncstr_processed 
1369          return MatlabEvent(argDict_out) 
1370      else: 
1371          return HighLevelEvent(argDict_out)

1372  
 
1373  
 


1374 -def makePythonStateZeroCrossEvent(varname, targetvalue, dircode, argDict,
 
1375                                      var=None):


1376      """Python function-specified zero-crossing event in coordinate, or in
 
1377      time. Use 'var' argument to create a variable-linked event.
 
1378  
 
1379      varname may be a Quantity object. dircode is -1, 0, or 1.
 
1380      varname may be the reserved word 't', for the independent variable.
 
1381      """ 
1382      # set `var` only for non-var linked events
 
1383      newargs = ['dircode', 'funcspec', 'vars'] 
1384      for arg in newargs: 
1385          if arg in argDict: 
1386              print 'Warning: `' + arg + '` already appears in argDict!' 
1387              print '  This value will be overwritten.' 
1388      if isinstance(varname, Var): 
1389          # supporting a Variable object
 
1390          varname = varname.name 
1391      elif not isinstance(varname, str): 
1392          raise TypeError("Invalid type for event variable") 
1393      funcname = "_f_"+varname+"_zc" 
1394      if isinstance(targetvalue, str): 
1395          # this is assumed to be a parameter name if not a numeric value,
 
1396          # but user preferred to add the p[' '] wrapping around a
 
1397          # parameter name
 
1398          if not (targetvalue[0:3] == "p['" and targetvalue[-2:] == "']"): 
1399                  targstr = "p['"+targetvalue+"']" 
1400          else: 
1401              targstr = targetvalue 
1402          pdefstr = "=None" 
1403      else: 
1404          targstr = str(targetvalue) 
1405          pdefstr = "" 
1406      # 'ds' plays role of 'self' (named for compatibility with FuncSpec's
 
1407      # automatic name resolution of auxiliary functions during parsing.
 
1408      if isinstance(var, Variable): 
1409          if not isinstance(targetvalue, str): 
1410              assert targetvalue in var.depdomain, 'targetvalue not in var.depdomain' 
1411          funcstr = "def "+funcname+"(ds, t, p%s):\n\treturn "%pdefstr + \
 
1412                      "ds.vars['"+varname+"'](t) - "+targstr+"\n" 
1413      elif var is None: 
1414          funcstr = "def "+funcname+"(ds, v, p%s):\n\treturn v['"%pdefstr + \
 
1415                      varname+"'] - " + targstr + "\n" 
1416      else: 
1417          raise ValueError('var must be a Variable object or None') 
1418      argDict_out = copy.copy(argDict) 
1419      argDict_out.update({'vars': {varname: var},
 
1420                      'funcspec': makeUniqueFn(funcstr, idstr="event"),
 
1421                      'expr': varname + ' - ' + targstr,
 
1422                      'dircode': dircode}) 
1423      return HighLevelEvent(argDict_out)

1424  
 
1425  
 


1426 -def collectReused(quant, allvars, allpars, allinputs, allfuns,
 
1427                    varnames, parnames, inputnames, auxfns, auxVarDefMap, flatspec):


1428      for symb in quant.freeSymbols: 
1429          if symb in allvars: 
1430              spectype = flatspec['spectypes'][symb] 
1431              if spectype == 'RHSfuncSpec': 
1432                  if symb not in varnames: 
1433                      varnames.append(symb) 
1434              elif spectype == 'ExpFuncSpec': 
1435                  auxVarDef = allvars[symb] 
1436                  auxVarDefMap[symb] = auxVarDef 
1437                  # need to recurse in case auxvar depends on another
 
1438                  symbQS = QuantSpec('__symb_expr__', auxVarDef) 
1439                  collectReused(symbQS, allvars, allpars, allinputs, allfuns,
 
1440                                varnames, parnames, inputnames, auxfns,
 
1441                                auxVarDefMap, flatspec) 
1442          elif symb in allpars: 
1443              if symb not in parnames: 
1444                  parnames.append(symb) 
1445          elif symb in allinputs: 
1446              if symb not in inputnames: 
1447                  inputnames.append(symb) 
1448          elif symb in allfuns: 
1449              if symb not in auxfns: 
1450                  auxfns[symb] = allfuns[symb] 
1451                  # Add any dependencies of the function
 
1452                  symbQS = QuantSpec('__symb_expr__', allfuns[symb][1]) 
1453                  collectReused(symbQS, allvars, allpars, allinputs, allfuns,
 
1454                                varnames, parnames, inputnames, auxfns,
 
1455                                auxVarDefMap, flatspec)

1456  
 
1457  
 


1458 -def processReusedPy(specnames, specdict, reuseterms, fspec, specials=[],
 
1459                          dovars=True, dopars=True, doinps=True, illegal=[]):


1460      """Process reused subexpression terms for Python code.
 
1461      (Similar to function of similar name in FuncSpec.py)
 
1462      """ 
1463      reused, specupdated, new_protected, order = FuncSpec._processReused(specnames,
 
1464                                                              specdict,
 
1465                                                              reuseterms,
 
1466                                                              _indentstr) 
1467      fspec._protected_reusenames = new_protected 
1468      fspec.varspecs.update(specupdated) 
1469      # symbols to parse are at indices 2 and 4 of 'reused' dictionary
 
1470      reusedParsed = fspec._parseReusedTermsPy(reused, [2,4],
 
1471                                      specials=specials, dovars=dovars,
 
1472                                      dopars=dopars, doinps=doinps,
 
1473                                                  illegal=illegal) 
1474      reusedefs = {}.fromkeys(new_protected) 
1475      for vname, deflist in reusedParsed.iteritems(): 
1476          for d in deflist: 
1477              reusedefs[d[2]] = d 
1478      return (concatStrDict(reusedefs, intersect(order,reusedefs.keys())),
 
1479                      specupdated)

1480  
 
1481  
 


1482 -def processReusedC(specnames, specdict, reuseterms):


1483      """Process reused subexpression terms for C code.
 
1484      (Similar to function processReusedC in FuncSpec.py)
 
1485      """ 
1486      reused, specupdated, new_protected, order = FuncSpec._processReused(specnames,
 
1487                                                      specdict,
 
1488                                                      reuseterms,
 
1489                                                      '', 'double', ';') 
1490      reusedefs = {}.fromkeys(new_protected) 
1491      for vname, deflist in reused.iteritems(): 
1492          for d in deflist: 
1493              reusedefs[d[2]] = d 
1494      return (concatStrDict(reusedefs, intersect(order, reusedefs.keys())),
 
1495                      specupdated)

1496  
 
1497  
 


1498 -def processReusedMatlab(specnames, specdict, reuseterms):


1499      """Process reused subexpression terms for matlab code.
 
1500      (Similar to function of similar name in FuncSpec.py)
 
1501      """ 
1502      reused, specupdated, new_protected, order = FuncSpec._processReused(specnames,
 
1503                                                      specdict,
 
1504                                                      reuseterms,
 
1505                                                      '', '', ';') 
1506      reusedefs = {}.fromkeys(new_protected) 
1507      for vname, deflist in reused.iteritems(): 
1508          for d in deflist: 
1509              reusedefs[d[2]] = d 
1510      return (concatStrDict(reusedefs, intersect(order, reusedefs.keys())),
 
1511                      specupdated)

1512  
 
1513  
 


1514 -def findpreciseroot(ev, tlo, thi, parDict=None, vars=None, inputs=None,
 
1515                      globalt0=0, quadratic_interp=None):


1516      """Find root more accurately from a Variable object using bisection.
 
1517  
 
1518      (Adapted from scipy.optimize.minpack.bisection code to make use of
 
1519      quadratic interpolation, which assumes that tlo and thi are already known
 
1520      to be close enough together that the variable's curve is purely concave
 
1521      up or down in the neighbourhood, and so can be fitted accurately with a
 
1522      single quadratic).
 
1523  
 
1524      To use quadratic interpolation, pass a fit_quadratic instance as the
 
1525      quadratic_interp argument. Interpolation will also be done on any inputs
 
1526      provided (**not yet implemented**).
 
1527      """ 
1528      if tlo >= thi: 
1529          raise ValueError('time limits are not finitely separated'
 
1530                              ' or are given in wrong order') 
1531      if inputs is None: 
1532          if quadratic_interp is not None: 
1533              raise NotImplementedError 
1534              #
 
1535              q = quadratic_interp 
1536              dt = thi-tlo #??? # may not be variable's mesh dt 
1537              # and so far Event has not assumed that variable is either defined
 
1538              # using a mesh or not !!!
 
1539              ts = linspace(tlo, thi, 8) 
1540              res = smooth_pts(ts, [ev._fn(t) for t in ts]) 
1541              test_fn = res.results.f 
1542          else: 
1543              test_fn = ev._fn 
1544      else: 
1545          # only going to be calling this with individual t values
 
1546          if ev.varlinked: 
1547              if quadratic_interp is not None: 
1548                  raise NotImplementedError 
1549              else: 
1550                  def test_fn(x, p): 
1551                      if p is None: 
1552                          pidict = dict([(n,i(t+globalt0)) for n,i in inputs.iteritems()]) 
1553                      else: 
1554                          pidict = copy.copy(p) 
1555                          pidict.update(dict([(n,i(t+globalt0)) for n,i in inputs.iteritems()])) 
1556                      return ev._fn(t, pidict)

1557          else: 
1558              if quadratic_interp is not None: 
1559                  raise NotImplementedError 
1560              else: 
1561                  def test_fn(x, p): 
1562                      if p is None: 
1563                          pidict = dict([(n,i(x['t']+globalt0)) for n,i in inputs.iteritems()]) 
1564                      else: 
1565                          pidict = copy.copy(p) 
1566                          pidict.update(dict([(n,i(x['t']+globalt0)) for n,i in inputs.iteritems()])) 
1567                      return ev._fn(x, pidict) 
1568      if ev.varlinked: 
1569          assert vars is None, ("Only pass vars argument when event is not "
 
1570                                  "linked to a variable") 
1571          assert reduce(bool.__and__, [iscontinuous(var) \
 
1572                                      for var in ev.vars.itervalues()]), \
 
1573                              'Only pass continously-defined Variables in event' 
1574          elo = test_fn(tlo, parDict) 
1575          ehi = test_fn(thi, parDict) 
1576          if elo == 0: 
1577              return (tlo, (tlo, thi)) 
1578          elif ehi == 0: 
1579              return (thi, (tlo, thi)) 
1580          elif elo * ehi > 0: 
1581              # event cannot be present
 
1582              return None 
1583          a = tlo 
1584          b = thi 
1585          i = 1 
1586          eva = test_fn(a, parDict) 
1587          rootival = (a,b) 
1588          while i <= ev.bisectlimit: 
1589              d = (b-a)/2.0 
1590              p = a + d 
1591              evp = test_fn(p, parDict) 
1592              if abs(evp-eva) < ev.eventtol or evp == 0: 
1593                  return (p, rootival) 
1594              i += 1 
1595              if evp*eva > 0: 
1596                  a = p 
1597                  eva = evp 
1598              else: 
1599                  b = p 
1600              # do this at end of while loop in case i > bisectlimit
 
1601              rootival = (a,b) 
1602          # search failed after bisectlimit
 
1603          return (None, rootival) 
1604      else: 
1605          assert vars is not None, ("vars argument is required when event is not"
 
1606                                      " linked to a variable") 
1607          assert reduce(bool.__and__, [iscontinuous(var) \
 
1608                                      for var in vars.itervalues()]), \
 
1609                              'Only pass continously-defined Variables' 
1610          varnames = sortedDictKeys(vars)+['t'] 
1611          dlo = dict(zip(varnames, [v(tlo) for v in sortedDictValues(vars)]+[tlo])) 
1612          dhi = dict(zip(varnames, [v(thi) for v in sortedDictValues(vars)]+[thi])) 
1613          elo = test_fn(dlo, parDict) 
1614          ehi = test_fn(dhi, parDict) 
1615          if elo == 0: 
1616              return (tlo, (tlo, thi)) 
1617          elif ehi == 0: 
1618              return (thi, (tlo, thi)) 
1619          elif elo * ehi > 0: 
1620              # event cannot be present
 
1621              return None 
1622          a = tlo 
1623          b = thi 
1624          i = 1 
1625          da = dict(zip(varnames, [v(a) for v in sortedDictValues(vars)]+[a])) 
1626          db = dict(zip(varnames, [v(b) for v in sortedDictValues(vars)]+[b])) 
1627          eva = test_fn(da, parDict) 
1628          rootival = (a,b) 
1629          while i <= ev.bisectlimit: 
1630              d = (b-a)/2.0 
1631              p = a + d 
1632              dp = dict(zip(varnames, [v(p) for v in sortedDictValues(vars)]+[p])) 
1633              evp = test_fn(dp, parDict) 
1634              if abs(evp-eva) < ev.eventtol or evp == 0: 
1635                  return (p, rootival) 
1636              i += 1 
1637              if evp*eva > 0: 
1638                  a = p 
1639                  eva = evp 
1640              else: 
1641                  b = p 
1642              # do this at end of while loop in case i > bisectlimit
 
1643              rootival = (a,b) 
1644          # search failed after bisectlimit
 
1645          return (None, rootival) 
1646
```

  


| Home | Trees | Indices | Help | | PyDSTool | | --- | |
| --- | --- | --- | --- | --- | --- |

|  |  |
| --- | --- |
| Generated by Epydoc 3.0.1 on Fri May 4 15:24:17 2012 | http://epydoc.sourceforge.net |
