# Supplementary material for: Hybrid Models and Biological Model Reduction with PyDSTool
Source: PLoS Comput Biol. 2012 Aug 9;8(8):e1002628. doi: 10.1371/journal.pcbi.1002628 (PMC3415397; doi:10.1371/journal.pcbi.1002628)
Supplement: Text S4 — Complete source code for the PyDSTool package (version 0.88.120504). Includes API documentation and help files linking to web pages. This file is identical to the current public release on Sourceforge.net. (ZIP) [file pcbi.1002628.s004.zip › PyDSTool/html/PyDSTool.Events.Event-class.html]

xml version="1.0" encoding="ascii"?


PyDSTool.Events.Event


| Home | Trees | Indices | Help | | PyDSTool | | --- | |
| --- | --- | --- | --- | --- | --- |

|  |  |  |  |
| --- | --- | --- | --- |
| Package PyDSTool :: Module Events :: Class Event | |  | | --- | | [hide private] | | [frames] | no frames] | |

# Class Event

source code

```
object --+
         |
        Event
```

Known Subclasses:
:   - HighLevelEvent
    - , LowLevelEvent

---

```
Generic Event.

Possible keys in argument dictionary at initialization:
    name, eventtol, eventdelay, starttime, bisectlimit, term, active,
    precise, vars, expr.
```


|  |  |  |  |
| --- | --- | --- | --- |
| |  |  | | --- | --- | | Instance Methods | [hide private] | | |
|  | |  |  | | --- | --- | | \_\_init\_\_(self, kw)  x.\_\_init\_\_(...) initializes x; see x.\_\_class\_\_.\_\_doc\_\_ for signature | source code | |
|  | |  |  | | --- | --- | | addToQ(self, qname, item) | source code | |
|  | |  |  | | --- | --- | | createQ(self, qname, sorted=True, seq=None)  Also use to reset a queue. | source code | |
|  | |  |  | | --- | --- | | popFromQ(self, qname) | source code | |
|  | |  |  | | --- | --- | | deleteQ(self, qname) | source code | |
|  | |  |  | | --- | --- | | \_infostr(self, verbose=1) | source code | |
|  | |  |  | | --- | --- | | info(self, verboselevel=1) | source code | |
|  | |  |  | | --- | --- | | \_\_repr\_\_(self)  str(x) | source code | |
|  | |  |  | | --- | --- | | \_\_str\_\_(self)  str(x) | source code | |
|  | |  |  | | --- | --- | | addMethods(self) | source code | |
|  | |  |  | | --- | --- | | reset(self, state=None)  Reset event`s prevsign attribute to a certain state (defaults to None) | source code | |
|  | |  |  | | --- | --- | | \_\_call\_\_(self, t=None, varDict=None, parDict=None)  Report on correct sign change. | source code | |
|  | |  |  | | --- | --- | | searchForEvents(self, trange=None, dt=None, checklevel=2, parDict=None, vars=None, inputs=None, abseps=1e-13, eventdelay=True, globalt0=0)  Search a variable-linked event, or an event with supplied vars dictionary and relevant parameters, for zero crossings. | source code | |
|  | |  |  | | --- | --- | | contains(self, interval, val, checklevel=2) | source code | |
|  | |  |  | | --- | --- | | \_\_getstate\_\_(self) | source code | |
|  | |  |  | | --- | --- | | \_\_setstate\_\_(self, state) | source code | |
|  | |  |  | | --- | --- | | \_\_copy\_\_(self) | source code | |
|  | |  |  | | --- | --- | | \_\_deepcopy\_\_(self, memo=None, \_nil=`[``]`) | source code | |
| **Inherited from `object`**: `__delattr__`, `__getattribute__`, `__hash__`, `__new__`, `__reduce__`, `__reduce_ex__`, `__setattr__` | |


|  |  |  |  |
| --- | --- | --- | --- |
| |  |  | | --- | --- | | Properties | [hide private] | | |
| **Inherited from `object`**: `__class__` | |


|  |  |  |  |
| --- | --- | --- | --- |
| |  |  | | --- | --- | | Method Details | [hide private] | | |

|  |  |  |
| --- | --- | --- |
| |  |  | | --- | --- | | \_\_init\_\_(self, kw)  *(Constructor)* | source code |   x.\_\_init\_\_(...) initializes x; see x.\_\_class\_\_.\_\_doc\_\_ for signature  Overrides: object.\_\_init\_\_ *(inherited documentation)* |

|  |  |  |
| --- | --- | --- |
| |  |  | | --- | --- | | \_\_repr\_\_(self)  *(Representation operator)* | source code |   str(x)  Overrides: object.\_\_repr\_\_ *(inherited documentation)* |

|  |  |  |
| --- | --- | --- |
| |  |  | | --- | --- | | \_\_str\_\_(self)  *(Informal representation operator)* | source code |   str(x)  Overrides: object.\_\_str\_\_ *(inherited documentation)* |

|  |  |  |
| --- | --- | --- |
| |  |  | | --- | --- | | \_\_call\_\_(self, t=None, varDict=None, parDict=None)  *(Call operator)* | source code |   Report on correct sign change. For external inputs, add input names and vales at time t to parDict |

|  |  |  |
| --- | --- | --- |
| |  |  | | --- | --- | | searchForEvents(self, trange=None, dt=None, checklevel=2, parDict=None, vars=None, inputs=None, abseps=1e-13, eventdelay=True, globalt0=0) | source code |  ``` Search a variable-linked event, or an event with supplied vars dictionary and relevant parameters, for zero crossings.  (Variable-linked search not applicable to low level events.)  trange=None, dt=None, checklevel=2, parDict=None, vars=None, inputs=None,     abseps=1e-13, eventdelay=True -> (ev_t, (ev_tlo, ev_thi)) where the lo-hi tuple is the smallest bound around ev_t (in case it is None because event was not found accurately).  dt will default to 1e-3 * the time interval of the variables. 'eventinterval' inherited from the event will be used to separate detected events.  Only pass vars dictionary when event.varlinked is False. ``` |

  


| Home | Trees | Indices | Help | | PyDSTool | | --- | |
| --- | --- | --- | --- | --- | --- |

|  |  |
| --- | --- |
| Generated by Epydoc 3.0.1 on Fri May 4 15:24:06 2012 | http://epydoc.sourceforge.net |
