# Supplementary material for: Hybrid Models and Biological Model Reduction with PyDSTool
Source: PLoS Comput Biol. 2012 Aug 9;8(8):e1002628. doi: 10.1371/journal.pcbi.1002628 (PMC3415397; doi:10.1371/journal.pcbi.1002628)
Supplement: Text S4 — Complete source code for the PyDSTool package (version 0.88.120504). Includes API documentation and help files linking to web pages. This file is identical to the current public release on Sourceforge.net. (ZIP) [file pcbi.1002628.s004.zip › PyDSTool/html/PyDSTool.Events.EventStruct-class.html]

xml version="1.0" encoding="ascii"?


PyDSTool.Events.EventStruct


| Home | Trees | Indices | Help | | PyDSTool | | --- | |
| --- | --- | --- | --- | --- | --- |

|  |  |  |  |
| --- | --- | --- | --- |
| Package PyDSTool :: Module Events :: Class EventStruct | |  | | --- | | [hide private] | | [frames] | no frames] | |

# Class EventStruct

source code

```
object --+
         |
        EventStruct
```

---

A data structure to store and interface with multiple events.


|  |  |  |  |
| --- | --- | --- | --- |
| |  |  | | --- | --- | | Instance Methods | [hide private] | | |
|  | |  |  | | --- | --- | | \_\_init\_\_(self)  x.\_\_init\_\_(...) initializes x; see x.\_\_class\_\_.\_\_doc\_\_ for signature | source code | |
|  | |  |  | | --- | --- | | \_makeFilterDict(self) | source code | |
|  | |  |  | | --- | --- | | resetEvtimes(self) | source code | |
|  | |  |  | | --- | --- | | \_\_deepcopy\_\_(self, dummy) | source code | |
|  | |  |  | | --- | --- | | \_\_del\_\_(self) | source code | |
|  | |  |  | | --- | --- | | \_\_setitem\_\_(self, ev) | source code | |
|  | |  |  | | --- | --- | | add(self, ev) | source code | |
|  | |  |  | | --- | --- | | \_\_delitem\_\_(self, ename) | source code | |
|  | |  |  | | --- | --- | | delete(self, ename) | source code | |
|  | |  |  | | --- | --- | | \_\_getitem\_\_(self, ename) | source code | |
|  | |  |  | | --- | --- | | sortedEventNames(self, eventlist=None) | source code | |
|  | |  |  | | --- | --- | | getHighLevelEvents(self) | source code | |
|  | |  |  | | --- | --- | | getLowLevelEvents(self) | source code | |
|  | |  |  | | --- | --- | | getAllEvents(self) | source code | |
|  | |  |  | | --- | --- | | getTermEvents(self) | source code | |
|  | |  |  | | --- | --- | | getNonTermEvents(self) | source code | |
|  | |  |  | | --- | --- | | getActiveEvents(self) | source code | |
|  | |  |  | | --- | --- | | getNonActiveEvents(self) | source code | |
|  | |  |  | | --- | --- | | getNonPreciseEvents(self) | source code | |
|  | |  |  | | --- | --- | | getPreciseEvents(self) | source code | |
|  | |  |  | | --- | --- | | setglobalt0(self, t0) | source code | |
|  | |  |  | | --- | --- | | query(self, keylist, eventlist=None)  Return eventlist with results of queries corresponding to self.\_keylist keys. | source code | |
|  | |  |  | | --- | --- | | \_\_call\_\_(self) | source code | |
|  | |  |  | | --- | --- | | info(self, verboselevel=0) | source code | |
|  | |  |  | | --- | --- | | \_\_contains\_\_(self, evname) | source code | |
|  | |  |  | | --- | --- | | pollHighLevelEvents(self, tval=None, varDict=None, parDict=None, eventlist=None) | source code | |
|  | |  |  | | --- | --- | | resetHighLevelEvents(self, t0, eventlist=None, state=None) | source code | |
|  | |  |  | | --- | --- | | validateEvents(self, database, eventlist)  validateEvents is only used for high level events. | source code | |
|  | |  |  | | --- | --- | | setTermFlag(self, eventTarget, flagval) | source code | |
|  | |  |  | | --- | --- | | setActiveFlag(self, eventTarget, flagval) | source code | |
|  | |  |  | | --- | --- | | setPreciseFlag(self, eventTarget, flagval) | source code | |
|  | |  |  | | --- | --- | | setEventICs(self, eventTarget, val) | source code | |
|  | |  |  | | --- | --- | | setEventDelay(self, eventTarget, val) | source code | |
|  | |  |  | | --- | --- | | setEventInterval(self, eventTarget, val) | source code | |
|  | |  |  | | --- | --- | | setEventTol(self, eventTarget, val) | source code | |
|  | |  |  | | --- | --- | | setEventDir(self, eventTarget, val) | source code | |
|  | |  |  | | --- | --- | | setStartTime(self, eventTarget, val) | source code | |
|  | |  |  | | --- | --- | | setBisect(self, eventTarget, val) | source code | |
| **Inherited from `object`**: `__delattr__`, `__getattribute__`, `__hash__`, `__new__`, `__reduce__`, `__reduce_ex__`, `__repr__`, `__setattr__`, `__str__` | |


|  |  |  |  |
| --- | --- | --- | --- |
| |  |  | | --- | --- | | Properties | [hide private] | | |
| **Inherited from `object`**: `__class__` | |


|  |  |  |  |
| --- | --- | --- | --- |
| |  |  | | --- | --- | | Method Details | [hide private] | | |

|  |  |  |
| --- | --- | --- |
| |  |  | | --- | --- | | \_\_init\_\_(self)  *(Constructor)* | source code |   x.\_\_init\_\_(...) initializes x; see x.\_\_class\_\_.\_\_doc\_\_ for signature  Overrides: object.\_\_init\_\_ *(inherited documentation)* |

|  |  |  |
| --- | --- | --- |
| |  |  | | --- | --- | | query(self, keylist, eventlist=None) | source code |   Return eventlist with results of queries corresponding to self.\_keylist keys.  Multiple keys permitted in the query. |

  


| Home | Trees | Indices | Help | | PyDSTool | | --- | |
| --- | --- | --- | --- | --- | --- |

|  |  |
| --- | --- |
| Generated by Epydoc 3.0.1 on Fri May 4 15:24:06 2012 | http://epydoc.sourceforge.net |
