# Supplementary material for: Hybrid Models and Biological Model Reduction with PyDSTool
Source: PLoS Comput Biol. 2012 Aug 9;8(8):e1002628. doi: 10.1371/journal.pcbi.1002628 (PMC3415397; doi:10.1371/journal.pcbi.1002628)
Supplement: Text S4 — Complete source code for the PyDSTool package (version 0.88.120504). Includes API documentation and help files linking to web pages. This file is identical to the current public release on Sourceforge.net. (ZIP) [file pcbi.1002628.s004.zip › PyDSTool/html/PyDSTool.fixedpickle-module.html]

xml version="1.0" encoding="ascii"?


PyDSTool.fixedpickle


| Home | Trees | Indices | Help | | PyDSTool | | --- | |
| --- | --- | --- | --- | --- | --- |

|  |  |  |  |
| --- | --- | --- | --- |
| Package PyDSTool :: Module fixedpickle | |  | | --- | | [hide private] | | [frames] | no frames] | |

# Module fixedpickle

source code

```
Create portable serialized representations of Python objects.

See module cPickle for a (much) faster implementation.
See module copy_reg for a mechanism for registering custom picklers.
See module pickletools source for extensive comments.

Classes:

    Pickler
    Unpickler

Functions:

    dump(object, file)
    dumps(object) -> string
    load(file) -> object
    loads(string) -> object

Misc variables:

    __version__
    format_version
    compatible_formats
```

---

**Version:**
$Revision: 1.156 $


|  |  |  |  |
| --- | --- | --- | --- |
| |  |  | | --- | --- | | Classes | [hide private] | | |
|  | PickleError  A common base class for the other pickling exceptions. |
|  | PicklingError  This exception is raised when an unpicklable object is passed to the dump() method. |
|  | UnpicklingError  This exception is raised when there is a problem unpickling an object, such as a security violation. |
|  | \_Stop |
|  | Pickler |
|  | Unpickler |
|  | \_EmptyClass |


|  |  |  |  |
| --- | --- | --- | --- |
| |  |  | | --- | --- | | Functions | [hide private] | | |
|  | |  |  | | --- | --- | | \_keep\_alive(x, memo)  Keeps a reference to the object x in the memo. | source code | |
|  | |  |  | | --- | --- | | whichmodule(func, funcname)  Figure out the module in which a function occurs. | source code | |
|  | |  |  | | --- | --- | | encode\_long(x)  Encode a long to a two's complement little-endian binary string. | source code | |
|  | |  |  | | --- | --- | | decode\_long(data)  Decode a long from a two's complement little-endian binary string. | source code | |
|  | |  |  | | --- | --- | | dump(obj, file, protocol=None, bin=None) | source code | |
|  | |  |  | | --- | --- | | dumps(obj, protocol=None, bin=None) | source code | |
|  | |  |  | | --- | --- | | load(file) | source code | |
|  | |  |  | | --- | --- | | loads(str) | source code | |
|  | |  |  | | --- | --- | | \_test() | source code | |


|  |  |  |  |
| --- | --- | --- | --- |
| |  |  | | --- | --- | | Variables | [hide private] | | |
|  | format\_version = `'2.0'` |
|  | compatible\_formats = `['1.0', '1.1', '1.2', '1.3', '2.0']` |
|  | HIGHEST\_PROTOCOL = `2` |
|  | PyStringMap = `None` |
|  | MARK = `'('` |
|  | STOP = `'.'` |
|  | POP = `'0'` |
|  | POP\_MARK = `'1'` |
|  | DUP = `'2'` |
|  | FLOAT = `'F'` |
|  | INT = `'I'` |
|  | BININT = `'J'` |
|  | BININT1 = `'K'` |
|  | LONG = `'L'` |
|  | BININT2 = `'M'` |
|  | NONE = `'N'` |
|  | PERSID = `'P'` |
|  | BINPERSID = `'Q'` |
|  | REDUCE = `'R'` |
|  | STRING = `'S'` |
|  | BINSTRING = `'T'` |
|  | SHORT\_BINSTRING = `'U'` |
|  | UNICODE = `'V'` |
|  | BINUNICODE = `'X'` |
|  | APPEND = `'a'` |
|  | BUILD = `'b'` |
|  | GLOBAL = `'c'` |
|  | DICT = `'d'` |
|  | EMPTY\_DICT = `'}'` |
|  | APPENDS = `'e'` |
|  | GET = `'g'` |
|  | BINGET = `'h'` |
|  | INST = `'i'` |
|  | LONG\_BINGET = `'j'` |
|  | LIST = `'l'` |
|  | EMPTY\_LIST = `']'` |
|  | OBJ = `'o'` |
|  | PUT = `'p'` |
|  | BINPUT = `'q'` |
|  | LONG\_BINPUT = `'r'` |
|  | SETITEM = `'s'` |
|  | TUPLE = `'t'` |
|  | EMPTY\_TUPLE = `')'` |
|  | SETITEMS = `'u'` |
|  | BINFLOAT = `'G'` |
|  | TRUE = `'I01\n'` |
|  | FALSE = `'I00\n'` |
|  | PROTO = `'\x80'` |
|  | NEWOBJ = `'\x81'` |
|  | EXT1 = `'\x82'` |
|  | EXT2 = `'\x83'` |
|  | EXT4 = `'\x84'` |
|  | TUPLE1 = `'\x85'` |
|  | TUPLE2 = `'\x86'` |
|  | TUPLE3 = `'\x87'` |
|  | NEWTRUE = `'\x88'` |
|  | NEWFALSE = `'\x89'` |
|  | LONG1 = `'\x8a'` |
|  | LONG4 = `'\x8b'` |
|  | \_tuplesize2code = `[')', '\x85', '\x86', '\x87']` |
|  | classmap = `{}` |


|  |  |  |  |
| --- | --- | --- | --- |
| |  |  | | --- | --- | | Function Details | [hide private] | | |

|  |  |  |
| --- | --- | --- |
| |  |  | | --- | --- | | \_keep\_alive(x, memo) | source code |   Keeps a reference to the object x in the memo.  Because we remember objects by their id, we have to assure that possibly temporary objects are kept alive by referencing them. We store a reference at the id of the memo, which should normally not be used unless someone tries to deepcopy the memo itself... |

|  |  |  |
| --- | --- | --- |
| |  |  | | --- | --- | | whichmodule(func, funcname) | source code |   Figure out the module in which a function occurs.  Search sys.modules for the module. Cache in classmap. Return a module name. If the function cannot be found, return "\_\_main\_\_". |

|  |  |  |
| --- | --- | --- |
| |  |  | | --- | --- | | encode\_long(x) | source code |   Encode a long to a two's complement little-endian binary string. Note that 0L is a special case, returning an empty string, to save a byte in the LONG1 pickling context.   ``` >>> encode_long(0L) '' >>> encode_long(255L) '\xff\x00' >>> encode_long(32767L) '\xff\x7f' >>> encode_long(-256L) '\x00\xff' >>> encode_long(-32768L) '\x00\x80' >>> encode_long(-128L) '\x80' >>> encode_long(127L) '\x7f' >>> ``` |

|  |  |  |
| --- | --- | --- |
| |  |  | | --- | --- | | decode\_long(data) | source code |   Decode a long from a two's complement little-endian binary string.   ``` >>> decode_long('') 0L >>> decode_long("\xff\x00") 255L >>> decode_long("\xff\x7f") 32767L >>> decode_long("\x00\xff") -256L >>> decode_long("\x00\x80") -32768L >>> decode_long("\x80") -128L >>> decode_long("\x7f") 127L ``` |

  


| Home | Trees | Indices | Help | | PyDSTool | | --- | |
| --- | --- | --- | --- | --- | --- |

|  |  |
| --- | --- |
| Generated by Epydoc 3.0.1 on Fri May 4 15:24:05 2012 | http://epydoc.sourceforge.net |
