# Supplementary material for: Hybrid Models and Biological Model Reduction with PyDSTool
Source: PLoS Comput Biol. 2012 Aug 9;8(8):e1002628. doi: 10.1371/journal.pcbi.1002628 (PMC3415397; doi:10.1371/journal.pcbi.1002628)
Supplement: Text S4 — Complete source code for the PyDSTool package (version 0.88.120504). Includes API documentation and help files linking to web pages. This file is identical to the current public release on Sourceforge.net. (ZIP) [file pcbi.1002628.s004.zip › PyDSTool/html/PyDSTool.fixedpickle-pysrc.html]

xml version="1.0" encoding="ascii"?


PyDSTool.fixedpickle


| Home | Trees | Indices | Help | | PyDSTool | | --- | |
| --- | --- | --- | --- | --- | --- |

|  |  |  |  |
| --- | --- | --- | --- |
| Package PyDSTool :: Module fixedpickle | |  | | --- | | [hide private] | | [frames] | no frames] | |

# Source Code for Module PyDSTool.fixedpickle

```
   1  """Create portable serialized representations of Python objects.
 
   2  
 
   3  See module cPickle for a (much) faster implementation.
 
   4  See module copy_reg for a mechanism for registering custom picklers.
 
   5  See module pickletools source for extensive comments.
 
   6  
 
   7  Classes:
 
   8  
 
   9      Pickler
 
  10      Unpickler
 
  11  
 
  12  Functions:
 
  13  
 
  14      dump(object, file)
 
  15      dumps(object) -> string
 
  16      load(file) -> object
 
  17      loads(string) -> object
 
  18  
 
  19  Misc variables:
 
  20  
 
  21      __version__
 
  22      format_version
 
  23      compatible_formats
 
  24  
 
  25  """ 
  26  
 
  27  # Patched version to allow pickling of IEE754 special values NaN and Inf
 
  28  # (original pickle cannot handle loading these on Win32 platforms)
 
  29  
 
  30  __version__ = "$Revision: 1.156 $"       # Code version 
  31  
 
  32  from types import * 
  33  from copy_reg import dispatch_table 
  34  from copy_reg import _extension_registry, _inverted_registry, _extension_cache 
  35  import marshal 
  36  import sys 
  37  import struct 
  38  import re 
  39  import warnings 
  40  
 
  41  __all__ = ["PickleError", "PicklingError", "UnpicklingError", "Pickler",
 
  42             "Unpickler", "dump", "dumps", "load", "loads"] 
  43  
 
  44  # These are purely informational; no code uses these.
 
  45  format_version = "2.0"                  # File format version we write 
  46  compatible_formats = ["1.0",            # Original protocol 0
 
  47                        "1.1",            # Protocol 0 with INST added
 
  48                        "1.2",            # Original protocol 1
 
  49                        "1.3",            # Protocol 1 with BINFLOAT added
 
  50                        "2.0",            # Protocol 2
 
  51                        ]                 # Old format versions we can read 
  52  
 
  53  # Keep in synch with cPickle.  This is the highest protocol number we
 
  54  # know how to read.
 
  55  HIGHEST_PROTOCOL = 2 
  56  
 
  57  # Why use struct.pack() for pickling but marshal.loads() for
 
  58  # unpickling?  struct.pack() is 40% faster than marshal.dumps(), but
 
  59  # marshal.loads() is twice as fast as struct.unpack()!
 
  60  mloads = marshal.loads 
  61  
 


62 -class PickleError(Exception):


63      """A common base class for the other pickling exceptions.""" 
  64      pass

  65  
 


66 -class PicklingError(PickleError):


67      """This exception is raised when an unpicklable object is passed to the
 
  68      dump() method.
 
  69  
 
  70      """ 
  71      pass

  72  
 


73 -class UnpicklingError(PickleError):


74      """This exception is raised when there is a problem unpickling an object,
 
  75      such as a security violation.
 
  76  
 
  77      Note that other exceptions may also be raised during unpickling, including
 
  78      (but not necessarily limited to) AttributeError, EOFError, ImportError,
 
  79      and IndexError.
 
  80  
 
  81      """ 
  82      pass

  83  
 
  84  # An instance of _Stop is raised by Unpickler.load_stop() in response to
 
  85  # the STOP opcode, passing the object that is the result of unpickling.
 


86 -class _Stop(Exception):


87 -    def __init__(self, value):


88          self.value = value

  89  
 
  90  # Jython has PyStringMap; it's a dict subclass with string keys
 
  91  try: 
  92      from org.python.core import PyStringMap 
  93  except ImportError: 
  94      PyStringMap = None 
  95  
 
  96  # UnicodeType may or may not be exported (normally imported from types)
 
  97  try: 
  98      UnicodeType 
  99  except NameError: 
 100      UnicodeType = None 
 101  
 
 102  # Pickle opcodes.  See pickletools.py for extensive docs.  The listing
 
 103  # here is in kind-of alphabetical order of 1-character pickle code.
 
 104  # pickletools groups them by purpose.
 
 105  
 
 106  MARK            = '('   # push special markobject on stack 
 107  STOP            = '.'   # every pickle ends with STOP 
 108  POP             = '0'   # discard topmost stack item 
 109  POP_MARK        = '1'   # discard stack top through topmost markobject 
 110  DUP             = '2'   # duplicate top stack item 
 111  FLOAT           = 'F'   # push float object; decimal string argument 
 112  INT             = 'I'   # push integer or bool; decimal string argument 
 113  BININT          = 'J'   # push four-byte signed int 
 114  BININT1         = 'K'   # push 1-byte unsigned int 
 115  LONG            = 'L'   # push long; decimal string argument 
 116  BININT2         = 'M'   # push 2-byte unsigned int 
 117  NONE            = 'N'   # push None 
 118  PERSID          = 'P'   # push persistent object; id is taken from string arg 
 119  BINPERSID       = 'Q'   #  "       "         "  ;  "  "   "     "  stack 
 120  REDUCE          = 'R'   # apply callable to argtuple, both on stack 
 121  STRING          = 'S'   # push string; NL-terminated string argument 
 122  BINSTRING       = 'T'   # push string; counted binary string argument 
 123  SHORT_BINSTRING = 'U'   #  "     "   ;    "      "       "      " < 256 bytes 
 124  UNICODE         = 'V'   # push Unicode string; raw-unicode-escaped'd argument 
 125  BINUNICODE      = 'X'   #   "     "       "  ; counted UTF-8 string argument 
 126  APPEND          = 'a'   # append stack top to list below it 
 127  BUILD           = 'b'   # call __setstate__ or __dict__.update() 
 128  GLOBAL          = 'c'   # push self.find_class(modname, name); 2 string args 
 129  DICT            = 'd'   # build a dict from stack items 
 130  EMPTY_DICT      = '}'   # push empty dict 
 131  APPENDS         = 'e'   # extend list on stack by topmost stack slice 
 132  GET             = 'g'   # push item from memo on stack; index is string arg 
 133  BINGET          = 'h'   #   "    "    "    "   "   "  ;   "    " 1-byte arg 
 134  INST            = 'i'   # build & push class instance 
 135  LONG_BINGET     = 'j'   # push item from memo on stack; index is 4-byte arg 
 136  LIST            = 'l'   # build list from topmost stack items 
 137  EMPTY_LIST      = ']'   # push empty list 
 138  OBJ             = 'o'   # build & push class instance 
 139  PUT             = 'p'   # store stack top in memo; index is string arg 
 140  BINPUT          = 'q'   #   "     "    "   "   " ;   "    " 1-byte arg 
 141  LONG_BINPUT     = 'r'   #   "     "    "   "   " ;   "    " 4-byte arg 
 142  SETITEM         = 's'   # add key+value pair to dict 
 143  TUPLE           = 't'   # build tuple from topmost stack items 
 144  EMPTY_TUPLE     = ')'   # push empty tuple 
 145  SETITEMS        = 'u'   # modify dict by adding topmost key+value pairs 
 146  BINFLOAT        = 'G'   # push float; arg is 8-byte float encoding 
 147  
 
 148  TRUE            = 'I01\n'  # not an opcode; see INT docs in pickletools.py 
 149  FALSE           = 'I00\n'  # not an opcode; see INT docs in pickletools.py 
 150  
 
 151  # Protocol 2
 
 152  
 
 153  PROTO           = '\x80'  # identify pickle protocol 
 154  NEWOBJ          = '\x81'  # build object by applying cls.__new__ to argtuple 
 155  EXT1            = '\x82'  # push object from extension registry; 1-byte index 
 156  EXT2            = '\x83'  # ditto, but 2-byte index 
 157  EXT4            = '\x84'  # ditto, but 4-byte index 
 158  TUPLE1          = '\x85'  # build 1-tuple from stack top 
 159  TUPLE2          = '\x86'  # build 2-tuple from two topmost stack items 
 160  TUPLE3          = '\x87'  # build 3-tuple from three topmost stack items 
 161  NEWTRUE         = '\x88'  # push True 
 162  NEWFALSE        = '\x89'  # push False 
 163  LONG1           = '\x8a'  # push long from < 256 bytes 
 164  LONG4           = '\x8b'  # push really big long 
 165  
 
 166  _tuplesize2code = [EMPTY_TUPLE, TUPLE1, TUPLE2, TUPLE3] 
 167  
 
 168  
 
 169  __all__.extend([x for x in dir() if re.match("[A-Z][A-Z0-9_]+$",x)]) 
 170  del x 
 171  
 
 172  
 
 173  # Pickling machinery
 
 174  
 


175 -class Pickler:


176  
 


177 -    def __init__(self, file, protocol=None, bin=None):


178          """This takes a file-like object for writing a pickle data stream.
 
 179  
 
 180          The optional protocol argument tells the pickler to use the
 
 181          given protocol; supported protocols are 0, 1, 2.  The default
 
 182          protocol is 0, to be backwards compatible.  (Protocol 0 is the
 
 183          only protocol that can be written to a file opened in text
 
 184          mode and read back successfully.  When using a protocol higher
 
 185          than 0, make sure the file is opened in binary mode, both when
 
 186          pickling and unpickling.)
 
 187  
 
 188          Protocol 1 is more efficient than protocol 0; protocol 2 is
 
 189          more efficient than protocol 1.
 
 190  
 
 191          Specifying a negative protocol version selects the highest
 
 192          protocol version supported.  The higher the protocol used, the
 
 193          more recent the version of Python needed to read the pickle
 
 194          produced.
 
 195  
 
 196          The file parameter must have a write() method that accepts a single
 
 197          string argument.  It can thus be an open file object, a StringIO
 
 198          object, or any other custom object that meets this interface.
 
 199  
 
 200          """ 
 201          if protocol is not None and bin is not None: 
 202              raise ValueError, "can't specify both 'protocol' and 'bin'" 
 203          if bin is not None: 
 204              warnings.warn("The 'bin' argument to Pickler() is deprecated",
 
 205                            PendingDeprecationWarning) 
 206              protocol = bin 
 207          if protocol is None: 
 208              protocol = 0 
 209          if protocol < 0: 
 210              protocol = HIGHEST_PROTOCOL 
 211          elif not 0 <= protocol <= HIGHEST_PROTOCOL: 
 212              raise ValueError("pickle protocol must be <= %d" % HIGHEST_PROTOCOL) 
 213          self.write = file.write 
 214          self.memo = {} 
 215          self.proto = int(protocol) 
 216          self.bin = protocol >= 1 
 217          self.fast = 0

 218  
 


219 -    def clear_memo(self):


220          """Clears the pickler's "memo".
 
 221  
 
 222          The memo is the data structure that remembers which objects the
 
 223          pickler has already seen, so that shared or recursive objects are
 
 224          pickled by reference and not by value.  This method is useful when
 
 225          re-using picklers.
 
 226  
 
 227          """ 
 228          self.memo.clear()

 229  
 


230 -    def dump(self, obj):


231          """Write a pickled representation of obj to the open file.""" 
 232          if self.proto >= 2: 
 233              self.write(PROTO + chr(self.proto)) 
 234          self.save(obj) 
 235          self.write(STOP)

 236  
 


237 -    def memoize(self, obj):


238          """Store an object in the memo.""" 
 239  
 
 240          # The Pickler memo is a dictionary mapping object ids to 2-tuples
 
 241          # that contain the Unpickler memo key and the object being memoized.
 
 242          # The memo key is written to the pickle and will become
 
 243          # the key in the Unpickler's memo.  The object is stored in the
 
 244          # Pickler memo so that transient objects are kept alive during
 
 245          # pickling.
 
 246  
 
 247          # The use of the Unpickler memo length as the memo key is just a
 
 248          # convention.  The only requirement is that the memo values be unique.
 
 249          # But there appears no advantage to any other scheme, and this
 
 250          # scheme allows the Unpickler memo to be implemented as a plain (but
 
 251          # growable) array, indexed by memo key.
 
 252          if self.fast: 
 253              return 
 254          assert id(obj) not in self.memo 
 255          memo_len = len(self.memo) 
 256          self.write(self.put(memo_len)) 
 257          self.memo[id(obj)] = memo_len, obj

 258  
 
 259      # Return a PUT (BINPUT, LONG_BINPUT) opcode string, with argument i.
 


260 -    def put(self, i, pack=struct.pack):


261          if self.bin: 
 262              if i < 256: 
 263                  return BINPUT + chr(i) 
 264              else: 
 265                  return LONG_BINPUT + pack("<i", i) 
 266  
 
 267          return PUT + `i` + '\n'

 268  
 
 269      # Return a GET (BINGET, LONG_BINGET) opcode string, with argument i.
 


270 -    def get(self, i, pack=struct.pack):


271          if self.bin: 
 272              if i < 256: 
 273                  return BINGET + chr(i) 
 274              else: 
 275                  return LONG_BINGET + pack("<i", i) 
 276  
 
 277          return GET + `i` + '\n'

 278  
 


279 -    def save(self, obj):


280          # Check for persistent id (defined by a subclass)
 
 281          pid = self.persistent_id(obj) 
 282          if pid: 
 283              self.save_pers(pid) 
 284              return 
 285  
 
 286          # Check the memo
 
 287          x = self.memo.get(id(obj)) 
 288          if x: 
 289              self.write(self.get(x[0])) 
 290              return 
 291  
 
 292          # Check the type dispatch table
 
 293          t = type(obj) 
 294          f = self.dispatch.get(t) 
 295          if f: 
 296              f(self, obj) # Call unbound method with explicit self 
 297              return 
 298  
 
 299          # Check for a class with a custom metaclass; treat as regular class
 
 300          try: 
 301              issc = issubclass(t, TypeType) 
 302          except TypeError: # t is not a class (old Boost; see SF #502085) 
 303              issc = 0 
 304          if issc: 
 305              self.save_global(obj) 
 306              return 
 307  
 
 308          # Check copy_reg.dispatch_table
 
 309          reduce = dispatch_table.get(t) 
 310          if reduce: 
 311              rv = reduce(obj) 
 312          else: 
 313              # Check for a __reduce_ex__ method, fall back to __reduce__
 
 314              reduce = getattr(obj, "__reduce_ex__", None) 
 315              if reduce: 
 316                  # TEMP
 
 317                  try: 
 318                      rv = reduce(self.proto) 
 319                  except: 
 320                      print "Problem with ", obj 
 321                      raise 
 322              else: 
 323                  reduce = getattr(obj, "__reduce__", None) 
 324                  if reduce: 
 325                      rv = reduce() 
 326                  else: 
 327                      raise PicklingError("Can't pickle %r object: %r" %
 
 328                                          (t.__name__, obj)) 
 329  
 
 330          # Check for string returned by reduce(), meaning "save as global"
 
 331          if type(rv) is StringType: 
 332              self.save_global(obj, rv) 
 333              return 
 334  
 
 335          # Assert that reduce() returned a tuple
 
 336          if type(rv) is not TupleType: 
 337              raise PicklingError("%s must return string or tuple" % reduce) 
 338  
 
 339          # Assert that it returned an appropriately sized tuple
 
 340          l = len(rv) 
 341          if not (2 <= l <= 5): 
 342              raise PicklingError("Tuple returned by %s must have "
 
 343                                  "two to five elements" % reduce) 
 344  
 
 345          # Save the reduce() output and finally memoize the object
 
 346          self.save_reduce(obj=obj, *rv)

 347  
 


348 -    def persistent_id(self, obj):


349          # This exists so a subclass can override it
 
 350          return None

 351  
 


352 -    def save_pers(self, pid):


353          # Save a persistent id reference
 
 354          if self.bin: 
 355              self.save(pid) 
 356              self.write(BINPERSID) 
 357          else: 
 358              self.write(PERSID + str(pid) + '\n')

 359  
 


360 -    def save_reduce(self, func, args, state=None,
 
 361                      listitems=None, dictitems=None, obj=None):


362          # This API is called by some subclasses
 
 363  
 
 364          # Assert that args is a tuple or None
 
 365          if not isinstance(args, TupleType): 
 366              if args is None: 
 367                  # A hack for Jim Fulton's ExtensionClass, now deprecated.
 
 368                  # See load_reduce()
 
 369                  warnings.warn("__basicnew__ special case is deprecated",
 
 370                                DeprecationWarning) 
 371              else: 
 372                  raise PicklingError(
 
 373                      "args from reduce() should be a tuple") 
 374  
 
 375          # Assert that func is callable
 
 376          if not callable(func): 
 377              raise PicklingError("func from reduce should be callable") 
 378  
 
 379          save = self.save 
 380          write = self.write 
 381  
 
 382          # Protocol 2 special case: if func's name is __newobj__, use NEWOBJ
 
 383          if self.proto >= 2 and getattr(func, "__name__", "") == "__newobj__": 
 384              # A __reduce__ implementation can direct protocol 2 to
 
 385              # use the more efficient NEWOBJ opcode, while still
 
 386              # allowing protocol 0 and 1 to work normally.  For this to
 
 387              # work, the function returned by __reduce__ should be
 
 388              # called __newobj__, and its first argument should be a
 
 389              # new-style class.  The implementation for __newobj__
 
 390              # should be as follows, although pickle has no way to
 
 391              # verify this:
 
 392              #
 
 393              # def __newobj__(cls, *args):
 
 394              #     return cls.__new__(cls, *args)
 
 395              #
 
 396              # Protocols 0 and 1 will pickle a reference to __newobj__,
 
 397              # while protocol 2 (and above) will pickle a reference to
 
 398              # cls, the remaining args tuple, and the NEWOBJ code,
 
 399              # which calls cls.__new__(cls, *args) at unpickling time
 
 400              # (see load_newobj below).  If __reduce__ returns a
 
 401              # three-tuple, the state from the third tuple item will be
 
 402              # pickled regardless of the protocol, calling __setstate__
 
 403              # at unpickling time (see load_build below).
 
 404              #
 
 405              # Note that no standard __newobj__ implementation exists;
 
 406              # you have to provide your own.  This is to enforce
 
 407              # compatibility with Python 2.2 (pickles written using
 
 408              # protocol 0 or 1 in Python 2.3 should be unpicklable by
 
 409              # Python 2.2).
 
 410              cls = args[0] 
 411              if not hasattr(cls, "__new__"): 
 412                  raise PicklingError(
 
 413                      "args[0] from __newobj__ args has no __new__") 
 414              if obj is not None and cls is not obj.__class__: 
 415                  raise PicklingError(
 
 416                      "args[0] from __newobj__ args has the wrong class") 
 417              args = args[1:] 
 418              save(cls) 
 419              save(args) 
 420              write(NEWOBJ) 
 421          else: 
 422              save(func) 
 423              save(args) 
 424              write(REDUCE) 
 425  
 
 426          if obj is not None: 
 427              self.memoize(obj) 
 428  
 
 429          # More new special cases (that work with older protocols as
 
 430          # well): when __reduce__ returns a tuple with 4 or 5 items,
 
 431          # the 4th and 5th item should be iterators that provide list
 
 432          # items and dict items (as (key, value) tuples), or None.
 
 433  
 
 434          if listitems is not None: 
 435              self._batch_appends(listitems) 
 436  
 
 437          if dictitems is not None: 
 438              self._batch_setitems(dictitems) 
 439  
 
 440          if state is not None: 
 441              save(state) 
 442              write(BUILD)

 443  
 
 444      # Methods below this point are dispatched through the dispatch table
 
 445  
 
 446      dispatch = {} 
 447  
 


448 -    def save_none(self, obj):


449          self.write(NONE)

 450      dispatch[NoneType] = save_none 
 451  
 


452 -    def save_bool(self, obj):


453          if self.proto >= 2: 
 454              self.write(obj and NEWTRUE or NEWFALSE) 
 455          else: 
 456              self.write(obj and TRUE or FALSE)

 457      dispatch[bool] = save_bool 
 458  
 


459 -    def save_int(self, obj, pack=struct.pack):


460          if self.bin: 
 461              # If the int is small enough to fit in a signed 4-byte 2's-comp
 
 462              # format, we can store it more efficiently than the general
 
 463              # case.
 
 464              # First one- and two-byte unsigned ints:
 
 465              if obj >= 0: 
 466                  if obj <= 0xff: 
 467                      self.write(BININT1 + chr(obj)) 
 468                      return 
 469                  if obj <= 0xffff: 
 470                      self.write("%c%c%c" % (BININT2, obj&0xff, obj>>8)) 
 471                      return 
 472              # Next check for 4-byte signed ints:
 
 473              high_bits = obj >> 31  # note that Python shift sign-extends 
 474              if high_bits == 0 or high_bits == -1: 
 475                  # All high bits are copies of bit 2**31, so the value
 
 476                  # fits in a 4-byte signed int.
 
 477                  self.write(BININT + pack("<i", obj)) 
 478                  return 
 479          # Text pickle, or int too big to fit in signed 4-byte format.
 
 480          self.write(INT + `obj` + '\n')

 481      dispatch[IntType] = save_int 
 482  
 


483 -    def save_long(self, obj, pack=struct.pack):


484          if self.proto >= 2: 
 485              bytes = encode_long(obj) 
 486              n = len(bytes) 
 487              if n < 256: 
 488                  self.write(LONG1 + chr(n) + bytes) 
 489              else: 
 490                  self.write(LONG4 + pack("<i", n) + bytes) 
 491              return 
 492          self.write(LONG + `obj` + '\n')

 493      dispatch[LongType] = save_long 
 494  
 


495 -    def save_float(self, obj, pack=struct.pack):


496          if self.bin: 
 497              self.write(BINFLOAT + pack('>d', obj)) 
 498          else: 
 499              self.write(FLOAT + `obj` + '\n')

 500      dispatch[FloatType] = save_float 
 501  
 


502 -    def save_string(self, obj, pack=struct.pack):


503          if self.bin: 
 504              n = len(obj) 
 505              if n < 256: 
 506                  self.write(SHORT_BINSTRING + chr(n) + obj) 
 507              else: 
 508                  self.write(BINSTRING + pack("<i", n) + obj) 
 509          else: 
 510              self.write(STRING + `obj` + '\n') 
 511          self.memoize(obj)

 512      dispatch[StringType] = save_string 
 513  
 


514 -    def save_unicode(self, obj, pack=struct.pack):


515          if self.bin: 
 516              encoding = obj.encode('utf-8') 
 517              n = len(encoding) 
 518              self.write(BINUNICODE + pack("<i", n) + encoding) 
 519          else: 
 520              obj = obj.replace("\\", "\\u005c") 
 521              obj = obj.replace("\n", "\\u000a") 
 522              self.write(UNICODE + obj.encode('raw-unicode-escape') + '\n') 
 523          self.memoize(obj)

 524      dispatch[UnicodeType] = save_unicode 
 525  
 
 526      if StringType == UnicodeType: 
 527          # This is true for Jython
 


528 -        def save_string(self, obj, pack=struct.pack):


529              unicode = obj.isunicode() 
 530  
 
 531              if self.bin: 
 532                  if unicode: 
 533                      obj = obj.encode("utf-8") 
 534                  l = len(obj) 
 535                  if l < 256 and not unicode: 
 536                      self.write(SHORT_BINSTRING + chr(l) + obj) 
 537                  else: 
 538                      s = pack("<i", l) 
 539                      if unicode: 
 540                          self.write(BINUNICODE + s + obj) 
 541                      else: 
 542                          self.write(BINSTRING + s + obj) 
 543              else: 
 544                  if unicode: 
 545                      obj = obj.replace("\\", "\\u005c") 
 546                      obj = obj.replace("\n", "\\u000a") 
 547                      obj = obj.encode('raw-unicode-escape') 
 548                      self.write(UNICODE + obj + '\n') 
 549                  else: 
 550                      self.write(STRING + `obj` + '\n') 
 551              self.memoize(obj)

 552          dispatch[StringType] = save_string 
 553  
 


554 -    def save_tuple(self, obj):


555          write = self.write 
 556          proto = self.proto 
 557  
 
 558          n = len(obj) 
 559          if n == 0: 
 560              if proto: 
 561                  write(EMPTY_TUPLE) 
 562              else: 
 563                  write(MARK + TUPLE) 
 564              return 
 565  
 
 566          save = self.save 
 567          memo = self.memo 
 568          if n <= 3 and proto >= 2: 
 569              for element in obj: 
 570                  save(element) 
 571              # Subtle.  Same as in the big comment below.
 
 572              if id(obj) in memo: 
 573                  get = self.get(memo[id(obj)][0]) 
 574                  write(POP * n + get) 
 575              else: 
 576                  write(_tuplesize2code[n]) 
 577                  self.memoize(obj) 
 578              return 
 579  
 
 580          # proto 0 or proto 1 and tuple isn't empty, or proto > 1 and tuple
 
 581          # has more than 3 elements.
 
 582          write(MARK) 
 583          for element in obj: 
 584              save(element) 
 585  
 
 586          if id(obj) in memo: 
 587              # Subtle.  d was not in memo when we entered save_tuple(), so
 
 588              # the process of saving the tuple's elements must have saved
 
 589              # the tuple itself:  the tuple is recursive.  The proper action
 
 590              # now is to throw away everything we put on the stack, and
 
 591              # simply GET the tuple (it's already constructed).  This check
 
 592              # could have been done in the "for element" loop instead, but
 
 593              # recursive tuples are a rare thing.
 
 594              get = self.get(memo[id(obj)][0]) 
 595              if proto: 
 596                  write(POP_MARK + get) 
 597              else:   # proto 0 -- POP_MARK not available 
 598                  write(POP * (n+1) + get) 
 599              return 
 600  
 
 601          # No recursion.
 
 602          self.write(TUPLE) 
 603          self.memoize(obj)

 604  
 
 605      dispatch[TupleType] = save_tuple 
 606  
 
 607      # save_empty_tuple() isn't used by anything in Python 2.3.  However, I
 
 608      # found a Pickler subclass in Zope3 that calls it, so it's not harmless
 
 609      # to remove it.
 


610 -    def save_empty_tuple(self, obj):


611          self.write(EMPTY_TUPLE)

 612  
 


613 -    def save_list(self, obj):


614          write = self.write 
 615  
 
 616          if self.bin: 
 617              write(EMPTY_LIST) 
 618          else:   # proto 0 -- can't use EMPTY_LIST 
 619              write(MARK + LIST) 
 620  
 
 621          self.memoize(obj) 
 622          self._batch_appends(iter(obj))

 623  
 
 624      dispatch[ListType] = save_list 
 625  
 
 626      # Keep in synch with cPickle's BATCHSIZE.  Nothing will break if it gets
 
 627      # out of synch, though.
 
 628      _BATCHSIZE = 1000 
 629  
 


630 -    def _batch_appends(self, items):


631          # Helper to batch up APPENDS sequences
 
 632          save = self.save 
 633          write = self.write 
 634  
 
 635          if not self.bin: 
 636              for x in items: 
 637                  save(x) 
 638                  write(APPEND) 
 639              return 
 640  
 
 641          r = xrange(self._BATCHSIZE) 
 642          while items is not None: 
 643              tmp = [] 
 644              for i in r: 
 645                  try: 
 646                      x = items.next() 
 647                      tmp.append(x) 
 648                  except StopIteration: 
 649                      items = None 
 650                      break 
 651              n = len(tmp) 
 652              if n > 1: 
 653                  write(MARK) 
 654                  for x in tmp: 
 655                      save(x) 
 656                  write(APPENDS) 
 657              elif n: 
 658                  save(tmp[0]) 
 659                  write(APPEND)

 660              # else tmp is empty, and we're done
 
 661  
 


662 -    def save_dict(self, obj):


663          write = self.write 
 664  
 
 665          if self.bin: 
 666              write(EMPTY_DICT) 
 667          else:   # proto 0 -- can't use EMPTY_DICT 
 668              write(MARK + DICT) 
 669  
 
 670          self.memoize(obj) 
 671          self._batch_setitems(obj.iteritems())

 672  
 
 673      dispatch[DictionaryType] = save_dict 
 674      if not PyStringMap is None: 
 675          dispatch[PyStringMap] = save_dict 
 676  
 


677 -    def _batch_setitems(self, items):


678          # Helper to batch up SETITEMS sequences; proto >= 1 only
 
 679          save = self.save 
 680          write = self.write 
 681  
 
 682          if not self.bin: 
 683              for k, v in items: 
 684                  save(k) 
 685                  save(v) 
 686                  write(SETITEM) 
 687              return 
 688  
 
 689          r = xrange(self._BATCHSIZE) 
 690          while items is not None: 
 691              tmp = [] 
 692              for i in r: 
 693                  try: 
 694                      tmp.append(items.next()) 
 695                  except StopIteration: 
 696                      items = None 
 697                      break 
 698              n = len(tmp) 
 699              if n > 1: 
 700                  write(MARK) 
 701                  for k, v in tmp: 
 702                      save(k) 
 703                      save(v) 
 704                  write(SETITEMS) 
 705              elif n: 
 706                  k, v = tmp[0] 
 707                  save(k) 
 708                  save(v) 
 709                  write(SETITEM)

 710              # else tmp is empty, and we're done
 
 711  
 


712 -    def save_inst(self, obj):


713          cls = obj.__class__ 
 714  
 
 715          memo  = self.memo 
 716          write = self.write 
 717          save  = self.save 
 718  
 
 719          if hasattr(obj, '__getinitargs__'): 
 720              args = obj.__getinitargs__() 
 721              len(args) # XXX Assert it's a sequence 
 722              _keep_alive(args, memo) 
 723          else: 
 724              args = () 
 725  
 
 726          write(MARK) 
 727  
 
 728          if self.bin: 
 729              save(cls) 
 730              for arg in args: 
 731                  save(arg) 
 732              write(OBJ) 
 733          else: 
 734              for arg in args: 
 735                  save(arg) 
 736              write(INST + cls.__module__ + '\n' + cls.__name__ + '\n') 
 737  
 
 738          self.memoize(obj) 
 739  
 
 740          try: 
 741              getstate = obj.__getstate__ 
 742          except AttributeError: 
 743              stuff = obj.__dict__ 
 744          else: 
 745              stuff = getstate() 
 746              _keep_alive(stuff, memo) 
 747          save(stuff) 
 748          write(BUILD)

 749  
 
 750      dispatch[InstanceType] = save_inst 
 751  
 


752 -    def save_global(self, obj, name=None, pack=struct.pack):


753          write = self.write 
 754          memo = self.memo 
 755  
 
 756          if name is None: 
 757              name = obj.__name__ 
 758  
 
 759          module = getattr(obj, "__module__", None) 
 760          if module is None: 
 761              module = whichmodule(obj, name) 
 762  
 
 763          try: 
 764              __import__(module) 
 765              mod = sys.modules[module] 
 766              klass = getattr(mod, name) 
 767          except (ImportError, KeyError, AttributeError): 
 768              raise PicklingError(
 
 769                  "Can't pickle %r: it's not found as %s.%s" %
 
 770                  (obj, module, name)) 
 771          else: 
 772              if klass is not obj: 
 773                  raise PicklingError(
 
 774                      "Can't pickle %r: it's not the same object as %s.%s" %
 
 775                      (obj, module, name)) 
 776  
 
 777          if self.proto >= 2: 
 778              code = _extension_registry.get((module, name)) 
 779              if code: 
 780                  assert code > 0 
 781                  if code <= 0xff: 
 782                      write(EXT1 + chr(code)) 
 783                  elif code <= 0xffff: 
 784                      write("%c%c%c" % (EXT2, code&0xff, code>>8)) 
 785                  else: 
 786                      write(EXT4 + pack("<i", code)) 
 787                  return 
 788  
 
 789          write(GLOBAL + module + '\n' + name + '\n') 
 790          self.memoize(obj)

 791  
 
 792      dispatch[ClassType] = save_global 
 793      dispatch[FunctionType] = save_global 
 794      dispatch[BuiltinFunctionType] = save_global 
 795      dispatch[TypeType] = save_global

 796  
 
 797  # Pickling helpers
 
 798  
 


799 -def _keep_alive(x, memo):


800      """Keeps a reference to the object x in the memo.
 
 801  
 
 802      Because we remember objects by their id, we have
 
 803      to assure that possibly temporary objects are kept
 
 804      alive by referencing them.
 
 805      We store a reference at the id of the memo, which should
 
 806      normally not be used unless someone tries to deepcopy
 
 807      the memo itself...
 
 808      """ 
 809      try: 
 810          memo[id(memo)].append(x) 
 811      except KeyError: 
 812          # aha, this is the first one :-)
 
 813          memo[id(memo)]=[x]

 814  
 
 815  
 
 816  # A cache for whichmodule(), mapping a function object to the name of
 
 817  # the module in which the function was found.
 
 818  
 
 819  classmap = {} # called classmap for backwards compatibility 
 820  
 


821 -def whichmodule(func, funcname):


822      """Figure out the module in which a function occurs.
 
 823  
 
 824      Search sys.modules for the module.
 
 825      Cache in classmap.
 
 826      Return a module name.
 
 827      If the function cannot be found, return "__main__".
 
 828      """ 
 829      # Python functions should always get an __module__ from their globals.
 
 830      mod = getattr(func, "__module__", None) 
 831      if mod is not None: 
 832          return mod 
 833      if func in classmap: 
 834          return classmap[func] 
 835  
 
 836      for name, module in sys.modules.items(): 
 837          if module is None: 
 838              continue # skip dummy package entries 
 839          if name != '__main__' and getattr(module, funcname, None) is func: 
 840              break 
 841      else: 
 842          name = '__main__' 
 843      classmap[func] = name 
 844      return name

 845  
 
 846  
 
 847  # Unpickling machinery
 
 848  
 


849 -class Unpickler:


850  
 


851 -    def __init__(self, file):


852          """This takes a file-like object for reading a pickle data stream.
 
 853  
 
 854          The protocol version of the pickle is detected automatically, so no
 
 855          proto argument is needed.
 
 856  
 
 857          The file-like object must have two methods, a read() method that
 
 858          takes an integer argument, and a readline() method that requires no
 
 859          arguments.  Both methods should return a string.  Thus file-like
 
 860          object can be a file object opened for reading, a StringIO object,
 
 861          or any other custom object that meets this interface.
 
 862          """ 
 863          self.readline = file.readline 
 864          self.read = file.read 
 865          self.memo = {}

 866  
 


867 -    def load(self):


868          """Read a pickled object representation from the open file.
 
 869  
 
 870          Return the reconstituted object hierarchy specified in the file.
 
 871          """ 
 872          self.mark = object() # any new unique object 
 873          self.stack = [] 
 874          self.append = self.stack.append 
 875          read = self.read 
 876          dispatch = self.dispatch 
 877          try: 
 878              while 1: 
 879                  key = read(1) 
 880                  dispatch[key](self) 
 881          except _Stop, stopinst: 
 882              return stopinst.value

 883  
 
 884      # Return largest index k such that self.stack[k] is self.mark.
 
 885      # If the stack doesn't contain a mark, eventually raises IndexError.
 
 886      # This could be sped by maintaining another stack, of indices at which
 
 887      # the mark appears.  For that matter, the latter stack would suffice,
 
 888      # and we wouldn't need to push mark objects on self.stack at all.
 
 889      # Doing so is probably a good thing, though, since if the pickle is
 
 890      # corrupt (or hostile) we may get a clue from finding self.mark embedded
 
 891      # in unpickled objects.
 


892 -    def marker(self):


893          stack = self.stack 
 894          mark = self.mark 
 895          k = len(stack)-1 
 896          while stack[k] is not mark: k = k-1 
 897          return k

 898  
 
 899      dispatch = {} 
 900  
 


901 -    def load_eof(self):


902          raise EOFError

 903      dispatch[''] = load_eof 
 904  
 


905 -    def load_proto(self):


906          proto = ord(self.read(1)) 
 907          if not 0 <= proto <= 2: 
 908              raise ValueError, "unsupported pickle protocol: %d" % proto

 909      dispatch[PROTO] = load_proto 
 910  
 


911 -    def load_persid(self):


912          pid = self.readline()[:-1] 
 913          self.append(self.persistent_load(pid))

 914      dispatch[PERSID] = load_persid 
 915  
 


916 -    def load_binpersid(self):


917          pid = self.stack.pop() 
 918          self.append(self.persistent_load(pid))

 919      dispatch[BINPERSID] = load_binpersid 
 920  
 


921 -    def load_none(self):


922          self.append(None)

 923      dispatch[NONE] = load_none 
 924  
 


925 -    def load_false(self):


926          self.append(False)

 927      dispatch[NEWFALSE] = load_false 
 928  
 


929 -    def load_true(self):


930          self.append(True)

 931      dispatch[NEWTRUE] = load_true 
 932  
 


933 -    def load_int(self):


934          data = self.readline() 
 935          if data == FALSE[1:]: 
 936              val = False 
 937          elif data == TRUE[1:]: 
 938              val = True 
 939          else: 
 940              try: 
 941                  val = int(data) 
 942              except ValueError: 
 943                  val = long(data) 
 944          self.append(val)

 945      dispatch[INT] = load_int 
 946  
 


947 -    def load_binint(self):


948          self.append(mloads('i' + self.read(4)))

 949      dispatch[BININT] = load_binint 
 950  
 


951 -    def load_binint1(self):


952          self.append(ord(self.read(1)))

 953      dispatch[BININT1] = load_binint1 
 954  
 


955 -    def load_binint2(self):


956          self.append(mloads('i' + self.read(2) + '\000\000'))

 957      dispatch[BININT2] = load_binint2 
 958  
 


959 -    def load_long(self):


960          self.append(long(self.readline()[:-1], 0))

 961      dispatch[LONG] = load_long 
 962  
 


963 -    def load_long1(self):


964          n = ord(self.read(1)) 
 965          bytes = self.read(n) 
 966          self.append(decode_long(bytes))

 967      dispatch[LONG1] = load_long1 
 968  
 


969 -    def load_long4(self):


970          n = mloads('i' + self.read(4)) 
 971          bytes = self.read(n) 
 972          self.append(decode_long(bytes))

 973      dispatch[LONG4] = load_long4 
 974  
 


975 -    def load_float(self):


976          s = self.readline()[:-1] 
 977          try: 
 978              f = float(s) 
 979          except ValueError: 
 980              s = s.upper() 
 981              if s in ["1.#INF", "INF"]: 
 982                  f = 1e300*1e300 
 983              elif s in ["-1.#INF", "-INF"]: 
 984                  f = -1e300*1e300 
 985              elif s in ["NAN","1.#QNAN","QNAN","1.#IND","IND","-1.#IND"]: 
 986                  f = -((1e300*1e300)/(1e300*1e300)) 
 987              else: 
 988                  raise ValueError, "Don't know what to do with "+`s` 
 989          self.append(f)

 990      dispatch[FLOAT] = load_float 
 991  
 


992 -    def load_binfloat(self, unpack=struct.unpack):


993          self.append(unpack('>d', self.read(8))[0])

 994      dispatch[BINFLOAT] = load_binfloat 
 995  
 


996 -    def load_string(self):


997          rep = self.readline()[:-1] 
 998          for q in "\"'": # double or single quote 
 999              if rep.startswith(q): 
1000                  if not rep.endswith(q): 
1001                      raise ValueError, "insecure string pickle" 
1002                  rep = rep[len(q):-len(q)] 
1003                  break 
1004          else: 
1005              raise ValueError, "insecure string pickle" 
1006          self.append(rep.decode("string-escape"))

1007      dispatch[STRING] = load_string 
1008  
 


1009 -    def load_binstring(self):


1010          len = mloads('i' + self.read(4)) 
1011          self.append(self.read(len))

1012      dispatch[BINSTRING] = load_binstring 
1013  
 


1014 -    def load_unicode(self):


1015          self.append(unicode(self.readline()[:-1],'raw-unicode-escape'))

1016      dispatch[UNICODE] = load_unicode 
1017  
 


1018 -    def load_binunicode(self):


1019          len = mloads('i' + self.read(4)) 
1020          self.append(unicode(self.read(len),'utf-8'))

1021      dispatch[BINUNICODE] = load_binunicode 
1022  
 


1023 -    def load_short_binstring(self):


1024          len = ord(self.read(1)) 
1025          self.append(self.read(len))

1026      dispatch[SHORT_BINSTRING] = load_short_binstring 
1027  
 


1028 -    def load_tuple(self):


1029          k = self.marker() 
1030          self.stack[k:] = [tuple(self.stack[k+1:])]

1031      dispatch[TUPLE] = load_tuple 
1032  
 


1033 -    def load_empty_tuple(self):


1034          self.stack.append(())

1035      dispatch[EMPTY_TUPLE] = load_empty_tuple 
1036  
 


1037 -    def load_tuple1(self):


1038          self.stack[-1] = (self.stack[-1],)

1039      dispatch[TUPLE1] = load_tuple1 
1040  
 


1041 -    def load_tuple2(self):


1042          self.stack[-2:] = [(self.stack[-2], self.stack[-1])]

1043      dispatch[TUPLE2] = load_tuple2 
1044  
 


1045 -    def load_tuple3(self):


1046          self.stack[-3:] = [(self.stack[-3], self.stack[-2], self.stack[-1])]

1047      dispatch[TUPLE3] = load_tuple3 
1048  
 


1049 -    def load_empty_list(self):


1050          self.stack.append([])

1051      dispatch[EMPTY_LIST] = load_empty_list 
1052  
 


1053 -    def load_empty_dictionary(self):


1054          self.stack.append({})

1055      dispatch[EMPTY_DICT] = load_empty_dictionary 
1056  
 


1057 -    def load_list(self):


1058          k = self.marker() 
1059          self.stack[k:] = [self.stack[k+1:]]

1060      dispatch[LIST] = load_list 
1061  
 


1062 -    def load_dict(self):


1063          k = self.marker() 
1064          d = {} 
1065          items = self.stack[k+1:] 
1066          for i in range(0, len(items), 2): 
1067              key = items[i] 
1068              value = items[i+1] 
1069              d[key] = value 
1070          self.stack[k:] = [d]

1071      dispatch[DICT] = load_dict 
1072  
 
1073      # INST and OBJ differ only in how they get a class object.  It's not
 
1074      # only sensible to do the rest in a common routine, the two routines
 
1075      # previously diverged and grew different bugs.
 
1076      # klass is the class to instantiate, and k points to the topmost mark
 
1077      # object, following which are the arguments for klass.__init__.
 


1078 -    def _instantiate(self, klass, k):


1079          args = tuple(self.stack[k+1:]) 
1080          del self.stack[k:] 
1081          instantiated = 0 
1082          if (not args and
 
1083                  type(klass) is ClassType and
 
1084                  not hasattr(klass, "__getinitargs__")): 
1085              try: 
1086                  value = _EmptyClass() 
1087                  value.__class__ = klass 
1088                  instantiated = 1 
1089              except RuntimeError: 
1090                  # In restricted execution, assignment to inst.__class__ is
 
1091                  # prohibited
 
1092                  pass 
1093          if not instantiated: 
1094              try: 
1095                  value = klass(*args) 
1096              except TypeError, err: 
1097                  raise TypeError, "in constructor for %s: %s" % (
 
1098                      klass.__name__, str(err)), sys.exc_info()[2] 
1099          self.append(value)

1100  
 


1101 -    def load_inst(self):


1102          module = self.readline()[:-1] 
1103          name = self.readline()[:-1] 
1104          klass = self.find_class(module, name) 
1105          self._instantiate(klass, self.marker())

1106      dispatch[INST] = load_inst 
1107  
 


1108 -    def load_obj(self):


1109          # Stack is ... markobject classobject arg1 arg2 ...
 
1110          k = self.marker() 
1111          klass = self.stack.pop(k+1) 
1112          self._instantiate(klass, k)

1113      dispatch[OBJ] = load_obj 
1114  
 


1115 -    def load_newobj(self):


1116          args = self.stack.pop() 
1117          cls = self.stack[-1] 
1118          obj = cls.__new__(cls, *args) 
1119          self.stack[-1] = obj

1120      dispatch[NEWOBJ] = load_newobj 
1121  
 


1122 -    def load_global(self):


1123          module = self.readline()[:-1] 
1124          name = self.readline()[:-1] 
1125          klass = self.find_class(module, name) 
1126          self.append(klass)

1127      dispatch[GLOBAL] = load_global 
1128  
 


1129 -    def load_ext1(self):


1130          code = ord(self.read(1)) 
1131          self.get_extension(code)

1132      dispatch[EXT1] = load_ext1 
1133  
 


1134 -    def load_ext2(self):


1135          code = mloads('i' + self.read(2) + '\000\000') 
1136          self.get_extension(code)

1137      dispatch[EXT2] = load_ext2 
1138  
 


1139 -    def load_ext4(self):


1140          code = mloads('i' + self.read(4)) 
1141          self.get_extension(code)

1142      dispatch[EXT4] = load_ext4 
1143  
 


1144 -    def get_extension(self, code):


1145          nil = [] 
1146          obj = _extension_cache.get(code, nil) 
1147          if obj is not nil: 
1148              self.append(obj) 
1149              return 
1150          key = _inverted_registry.get(code) 
1151          if not key: 
1152              raise ValueError("unregistered extension code %d" % code) 
1153          obj = self.find_class(*key) 
1154          _extension_cache[code] = obj 
1155          self.append(obj)

1156  
 


1157 -    def find_class(self, module, name):


1158          # Subclasses may override this
 
1159          __import__(module) 
1160          mod = sys.modules[module] 
1161          klass = getattr(mod, name) 
1162          return klass

1163  
 


1164 -    def load_reduce(self):


1165          stack = self.stack 
1166          args = stack.pop() 
1167          func = stack[-1] 
1168          if args is None: 
1169              # A hack for Jim Fulton's ExtensionClass, now deprecated
 
1170              warnings.warn("__basicnew__ special case is deprecated",
 
1171                            DeprecationWarning) 
1172              value = func.__basicnew__() 
1173          else: 
1174              value = func(*args) 
1175          stack[-1] = value

1176      dispatch[REDUCE] = load_reduce 
1177  
 


1178 -    def load_pop(self):


1179          del self.stack[-1]

1180      dispatch[POP] = load_pop 
1181  
 


1182 -    def load_pop_mark(self):


1183          k = self.marker() 
1184          del self.stack[k:]

1185      dispatch[POP_MARK] = load_pop_mark 
1186  
 


1187 -    def load_dup(self):


1188          self.append(self.stack[-1])

1189      dispatch[DUP] = load_dup 
1190  
 


1191 -    def load_get(self):


1192          self.append(self.memo[self.readline()[:-1]])

1193      dispatch[GET] = load_get 
1194  
 


1195 -    def load_binget(self):


1196          i = ord(self.read(1)) 
1197          self.append(self.memo[`i`])

1198      dispatch[BINGET] = load_binget 
1199  
 


1200 -    def load_long_binget(self):


1201          i = mloads('i' + self.read(4)) 
1202          self.append(self.memo[`i`])

1203      dispatch[LONG_BINGET] = load_long_binget 
1204  
 


1205 -    def load_put(self):


1206          self.memo[self.readline()[:-1]] = self.stack[-1]

1207      dispatch[PUT] = load_put 
1208  
 


1209 -    def load_binput(self):


1210          i = ord(self.read(1)) 
1211          self.memo[`i`] = self.stack[-1]

1212      dispatch[BINPUT] = load_binput 
1213  
 


1214 -    def load_long_binput(self):


1215          i = mloads('i' + self.read(4)) 
1216          self.memo[`i`] = self.stack[-1]

1217      dispatch[LONG_BINPUT] = load_long_binput 
1218  
 


1219 -    def load_append(self):


1220          stack = self.stack 
1221          value = stack.pop() 
1222          list = stack[-1] 
1223          list.append(value)

1224      dispatch[APPEND] = load_append 
1225  
 


1226 -    def load_appends(self):


1227          stack = self.stack 
1228          mark = self.marker() 
1229          list = stack[mark - 1] 
1230          list.extend(stack[mark + 1:]) 
1231          del stack[mark:]

1232      dispatch[APPENDS] = load_appends 
1233  
 


1234 -    def load_setitem(self):


1235          stack = self.stack 
1236          value = stack.pop() 
1237          key = stack.pop() 
1238          dict = stack[-1] 
1239          dict[key] = value

1240      dispatch[SETITEM] = load_setitem 
1241  
 


1242 -    def load_setitems(self):


1243          stack = self.stack 
1244          mark = self.marker() 
1245          dict = stack[mark - 1] 
1246          for i in range(mark + 1, len(stack), 2): 
1247              dict[stack[i]] = stack[i + 1] 
1248  
 
1249          del stack[mark:]

1250      dispatch[SETITEMS] = load_setitems 
1251  
 


1252 -    def load_build(self):


1253          stack = self.stack 
1254          state = stack.pop() 
1255          inst = stack[-1] 
1256          setstate = getattr(inst, "__setstate__", None) 
1257          if setstate: 
1258              setstate(state) 
1259              return 
1260          slotstate = None 
1261          if isinstance(state, tuple) and len(state) == 2: 
1262              state, slotstate = state 
1263          if state: 
1264              try: 
1265                  inst.__dict__.update(state) 
1266              except RuntimeError: 
1267                  # XXX In restricted execution, the instance's __dict__
 
1268                  # is not accessible.  Use the old way of unpickling
 
1269                  # the instance variables.  This is a semantic
 
1270                  # difference when unpickling in restricted
 
1271                  # vs. unrestricted modes.
 
1272                  # Note, however, that cPickle has never tried to do the
 
1273                  # .update() business, and always uses
 
1274                  #     PyObject_SetItem(inst.__dict__, key, value) in a
 
1275                  # loop over state.items().
 
1276                  for k, v in state.items(): 
1277                      setattr(inst, k, v) 
1278          if slotstate: 
1279              for k, v in slotstate.items(): 
1280                  setattr(inst, k, v)

1281      dispatch[BUILD] = load_build 
1282  
 


1283 -    def load_mark(self):


1284          self.append(self.mark)

1285      dispatch[MARK] = load_mark 
1286  
 


1287 -    def load_stop(self):


1288          value = self.stack.pop() 
1289          raise _Stop(value)

1290      dispatch[STOP] = load_stop

1291  
 
1292  # Helper class for load_inst/load_obj
 
1293  
 


1294 -class _EmptyClass:


1295      pass

1296  
 
1297  # Encode/decode longs in linear time.
 
1298  
 
1299  import binascii as _binascii 
1300  
 


1301 -def encode_long(x):


1302      r"""Encode a long to a two's complement little-endian binary string.
 
1303      Note that 0L is a special case, returning an empty string, to save a
 
1304      byte in the LONG1 pickling context.
 
1305  
 
1306      >>> encode_long(0L)
 
1307      ''
 
1308      >>> encode_long(255L)
 
1309      '\xff\x00'
 
1310      >>> encode_long(32767L)
 
1311      '\xff\x7f'
 
1312      >>> encode_long(-256L)
 
1313      '\x00\xff'
 
1314      >>> encode_long(-32768L)
 
1315      '\x00\x80'
 
1316      >>> encode_long(-128L)
 
1317      '\x80'
 
1318      >>> encode_long(127L)
 
1319      '\x7f'
 
1320      >>>
 
1321      """ 
1322  
 
1323      if x == 0: 
1324          return '' 
1325      if x > 0: 
1326          ashex = hex(x) 
1327          assert ashex.startswith("0x") 
1328          njunkchars = 2 + ashex.endswith('L') 
1329          nibbles = len(ashex) - njunkchars 
1330          if nibbles & 1: 
1331              # need an even # of nibbles for unhexlify
 
1332              ashex = "0x0" + ashex[2:] 
1333          elif int(ashex[2], 16) >= 8: 
1334              # "looks negative", so need a byte of sign bits
 
1335              ashex = "0x00" + ashex[2:] 
1336      else: 
1337          # Build the 256's-complement:  (1L << nbytes) + x.  The trick is
 
1338          # to find the number of bytes in linear time (although that should
 
1339          # really be a constant-time task).
 
1340          ashex = hex(-x) 
1341          assert ashex.startswith("0x") 
1342          njunkchars = 2 + ashex.endswith('L') 
1343          nibbles = len(ashex) - njunkchars 
1344          if nibbles & 1: 
1345              # Extend to a full byte.
 
1346              nibbles += 1 
1347          nbits = nibbles * 4 
1348          x += 1L << nbits 
1349          assert x > 0 
1350          ashex = hex(x) 
1351          njunkchars = 2 + ashex.endswith('L') 
1352          newnibbles = len(ashex) - njunkchars 
1353          if newnibbles < nibbles: 
1354              ashex = "0x" + "0" * (nibbles - newnibbles) + ashex[2:] 
1355          if int(ashex[2], 16) < 8: 
1356              # "looks positive", so need a byte of sign bits
 
1357              ashex = "0xff" + ashex[2:] 
1358  
 
1359      if ashex.endswith('L'): 
1360          ashex = ashex[2:-1] 
1361      else: 
1362          ashex = ashex[2:] 
1363      assert len(ashex) & 1 == 0, (x, ashex) 
1364      binary = _binascii.unhexlify(ashex) 
1365      return binary[::-1]

1366  
 


1367 -def decode_long(data):


1368      r"""Decode a long from a two's complement little-endian binary string.
 
1369  
 
1370      >>> decode_long('')
 
1371      0L
 
1372      >>> decode_long("\xff\x00")
 
1373      255L
 
1374      >>> decode_long("\xff\x7f")
 
1375      32767L
 
1376      >>> decode_long("\x00\xff")
 
1377      -256L
 
1378      >>> decode_long("\x00\x80")
 
1379      -32768L
 
1380      >>> decode_long("\x80")
 
1381      -128L
 
1382      >>> decode_long("\x7f")
 
1383      127L
 
1384      """ 
1385  
 
1386      nbytes = len(data) 
1387      if nbytes == 0: 
1388          return 0L 
1389      ashex = _binascii.hexlify(data[::-1]) 
1390      n = long(ashex, 16) # quadratic time before Python 2.3; linear now 
1391      if data[-1] >= '\x80': 
1392          n -= 1L << (nbytes * 8) 
1393      return n

1394  
 
1395  # Shorthands
 
1396  
 
1397  try: 
1398      from cStringIO import StringIO 
1399  except ImportError: 
1400      from StringIO import StringIO 
1401  
 


1402 -def dump(obj, file, protocol=None, bin=None):


1403      Pickler(file, protocol, bin).dump(obj)

1404  
 


1405 -def dumps(obj, protocol=None, bin=None):


1406      file = StringIO() 
1407      Pickler(file, protocol, bin).dump(obj) 
1408      return file.getvalue()

1409  
 


1410 -def load(file):


1411      return Unpickler(file).load()

1412  
 


1413 -def loads(str):


1414      file = StringIO(str) 
1415      return Unpickler(file).load()

1416  
 
1417  # Doctest
 
1418  
 


1419 -def _test():


1420      import doctest 
1421      return doctest.testmod()

1422  
 
1423  if __name__ == "__main__": 
1424      _test() 
1425
```

  


| Home | Trees | Indices | Help | | PyDSTool | | --- | |
| --- | --- | --- | --- | --- | --- |

|  |  |
| --- | --- |
| Generated by Epydoc 3.0.1 on Fri May 4 15:24:11 2012 | http://epydoc.sourceforge.net |
