# Supplementary material for: Hybrid Models and Biological Model Reduction with PyDSTool
Source: PLoS Comput Biol. 2012 Aug 9;8(8):e1002628. doi: 10.1371/journal.pcbi.1002628 (PMC3415397; doi:10.1371/journal.pcbi.1002628)
Supplement: Text S4 — Complete source code for the PyDSTool package (version 0.88.120504). Includes API documentation and help files linking to web pages. This file is identical to the current public release on Sourceforge.net. (ZIP) [file pcbi.1002628.s004.zip › PyDSTool/html/PyDSTool.fixedpickle.Pickler-class.html]

xml version="1.0" encoding="ascii"?


PyDSTool.fixedpickle.Pickler


| Home | Trees | Indices | Help | | PyDSTool | | --- | |
| --- | --- | --- | --- | --- | --- |

|  |  |  |  |
| --- | --- | --- | --- |
| Package PyDSTool :: Module fixedpickle :: Class Pickler | |  | | --- | | [hide private] | | [frames] | no frames] | |

# Class Pickler

source code


|  |  |  |  |
| --- | --- | --- | --- |
| |  |  | | --- | --- | | Instance Methods | [hide private] | | |
|  | |  |  | | --- | --- | | \_\_init\_\_(self, file, protocol=None, bin=None)  This takes a file-like object for writing a pickle data stream. | source code | |
|  | |  |  | | --- | --- | | clear\_memo(self)  Clears the pickler's "memo". | source code | |
|  | |  |  | | --- | --- | | dump(self, obj)  Write a pickled representation of obj to the open file. | source code | |
|  | |  |  | | --- | --- | | memoize(self, obj)  Store an object in the memo. | source code | |
|  | |  |  | | --- | --- | | put(self, i, pack=<function pack at 0x33df30>) | source code | |
|  | |  |  | | --- | --- | | get(self, i, pack=<function pack at 0x33df30>) | source code | |
|  | |  |  | | --- | --- | | save(self, obj) | source code | |
|  | |  |  | | --- | --- | | persistent\_id(self, obj) | source code | |
|  | |  |  | | --- | --- | | save\_pers(self, pid) | source code | |
|  | |  |  | | --- | --- | | save\_reduce(self, func, args, state=None, listitems=None, dictitems=None, obj=None) | source code | |
|  | |  |  | | --- | --- | | save\_none(self, obj) | source code | |
|  | |  |  | | --- | --- | | save\_bool(self, obj) | source code | |
|  | |  |  | | --- | --- | | save\_int(self, obj, pack=<function pack at 0x33df30>) | source code | |
|  | |  |  | | --- | --- | | save\_long(self, obj, pack=<function pack at 0x33df30>) | source code | |
|  | |  |  | | --- | --- | | save\_float(self, obj, pack=<function pack at 0x33df30>) | source code | |
|  | |  |  | | --- | --- | | save\_unicode(self, obj, pack=<function pack at 0x33df30>) | source code | |
|  | |  |  | | --- | --- | | save\_string(self, obj, pack=<function pack at 0x33df30>) | source code | |
|  | |  |  | | --- | --- | | save\_tuple(self, obj) | source code | |
|  | |  |  | | --- | --- | | save\_empty\_tuple(self, obj) | source code | |
|  | |  |  | | --- | --- | | save\_list(self, obj) | source code | |
|  | |  |  | | --- | --- | | \_batch\_appends(self, items) | source code | |
|  | |  |  | | --- | --- | | save\_dict(self, obj) | source code | |
|  | |  |  | | --- | --- | | \_batch\_setitems(self, items) | source code | |
|  | |  |  | | --- | --- | | save\_inst(self, obj) | source code | |
|  | |  |  | | --- | --- | | save\_global(self, obj, name=None, pack=<function pack at 0x33df30>) | source code | |


|  |  |  |  |
| --- | --- | --- | --- |
| |  |  | | --- | --- | | Class Variables | [hide private] | | |
|  | dispatch = `{}` |
|  | \_BATCHSIZE = `1000` |


|  |  |  |  |
| --- | --- | --- | --- |
| |  |  | | --- | --- | | Method Details | [hide private] | | |

|  |  |  |
| --- | --- | --- |
| |  |  | | --- | --- | | \_\_init\_\_(self, file, protocol=None, bin=None)  *(Constructor)* | source code |   This takes a file-like object for writing a pickle data stream.  The optional protocol argument tells the pickler to use the given protocol; supported protocols are 0, 1, 2. The default protocol is 0, to be backwards compatible. (Protocol 0 is the only protocol that can be written to a file opened in text mode and read back successfully. When using a protocol higher than 0, make sure the file is opened in binary mode, both when pickling and unpickling.)  Protocol 1 is more efficient than protocol 0; protocol 2 is more efficient than protocol 1.  Specifying a negative protocol version selects the highest protocol version supported. The higher the protocol used, the more recent the version of Python needed to read the pickle produced.  The file parameter must have a write() method that accepts a single string argument. It can thus be an open file object, a StringIO object, or any other custom object that meets this interface. |

|  |  |  |
| --- | --- | --- |
| |  |  | | --- | --- | | clear\_memo(self) | source code |   Clears the pickler's "memo".  The memo is the data structure that remembers which objects the pickler has already seen, so that shared or recursive objects are pickled by reference and not by value. This method is useful when re-using picklers. |

  


| Home | Trees | Indices | Help | | PyDSTool | | --- | |
| --- | --- | --- | --- | --- | --- |

|  |  |
| --- | --- |
| Generated by Epydoc 3.0.1 on Fri May 4 15:24:10 2012 | http://epydoc.sourceforge.net |
