# Supplementary material for: Hybrid Models and Biological Model Reduction with PyDSTool
Source: PLoS Comput Biol. 2012 Aug 9;8(8):e1002628. doi: 10.1371/journal.pcbi.1002628 (PMC3415397; doi:10.1371/journal.pcbi.1002628)
Supplement: Text S4 — Complete source code for the PyDSTool package (version 0.88.120504). Includes API documentation and help files linking to web pages. This file is identical to the current public release on Sourceforge.net. (ZIP) [file pcbi.1002628.s004.zip › PyDSTool/html/PyDSTool.fixedpickle.Unpickler-class.html]

xml version="1.0" encoding="ascii"?


PyDSTool.fixedpickle.Unpickler


| Home | Trees | Indices | Help | | PyDSTool | | --- | |
| --- | --- | --- | --- | --- | --- |

|  |  |  |  |
| --- | --- | --- | --- |
| Package PyDSTool :: Module fixedpickle :: Class Unpickler | |  | | --- | | [hide private] | | [frames] | no frames] | |

# Class Unpickler

source code


|  |  |  |  |
| --- | --- | --- | --- |
| |  |  | | --- | --- | | Instance Methods | [hide private] | | |
|  | |  |  | | --- | --- | | \_\_init\_\_(self, file)  This takes a file-like object for reading a pickle data stream. | source code | |
|  | |  |  | | --- | --- | | load(self)  Read a pickled object representation from the open file. | source code | |
|  | |  |  | | --- | --- | | marker(self) | source code | |
|  | |  |  | | --- | --- | | load\_eof(self) | source code | |
|  | |  |  | | --- | --- | | load\_proto(self) | source code | |
|  | |  |  | | --- | --- | | load\_persid(self) | source code | |
|  | |  |  | | --- | --- | | load\_binpersid(self) | source code | |
|  | |  |  | | --- | --- | | load\_none(self) | source code | |
|  | |  |  | | --- | --- | | load\_false(self) | source code | |
|  | |  |  | | --- | --- | | load\_true(self) | source code | |
|  | |  |  | | --- | --- | | load\_int(self) | source code | |
|  | |  |  | | --- | --- | | load\_binint(self) | source code | |
|  | |  |  | | --- | --- | | load\_binint1(self) | source code | |
|  | |  |  | | --- | --- | | load\_binint2(self) | source code | |
|  | |  |  | | --- | --- | | load\_long(self) | source code | |
|  | |  |  | | --- | --- | | load\_long1(self) | source code | |
|  | |  |  | | --- | --- | | load\_long4(self) | source code | |
|  | |  |  | | --- | --- | | load\_float(self) | source code | |
|  | |  |  | | --- | --- | | load\_binfloat(self, unpack=<function unpack at 0x33dfb0>) | source code | |
|  | |  |  | | --- | --- | | load\_string(self) | source code | |
|  | |  |  | | --- | --- | | load\_binstring(self) | source code | |
|  | |  |  | | --- | --- | | load\_unicode(self) | source code | |
|  | |  |  | | --- | --- | | load\_binunicode(self) | source code | |
|  | |  |  | | --- | --- | | load\_short\_binstring(self) | source code | |
|  | |  |  | | --- | --- | | load\_tuple(self) | source code | |
|  | |  |  | | --- | --- | | load\_empty\_tuple(self) | source code | |
|  | |  |  | | --- | --- | | load\_tuple1(self) | source code | |
|  | |  |  | | --- | --- | | load\_tuple2(self) | source code | |
|  | |  |  | | --- | --- | | load\_tuple3(self) | source code | |
|  | |  |  | | --- | --- | | load\_empty\_list(self) | source code | |
|  | |  |  | | --- | --- | | load\_empty\_dictionary(self) | source code | |
|  | |  |  | | --- | --- | | load\_list(self) | source code | |
|  | |  |  | | --- | --- | | load\_dict(self) | source code | |
|  | |  |  | | --- | --- | | \_instantiate(self, klass, k) | source code | |
|  | |  |  | | --- | --- | | load\_inst(self) | source code | |
|  | |  |  | | --- | --- | | load\_obj(self) | source code | |
|  | |  |  | | --- | --- | | load\_newobj(self) | source code | |
|  | |  |  | | --- | --- | | load\_global(self) | source code | |
|  | |  |  | | --- | --- | | load\_ext1(self) | source code | |
|  | |  |  | | --- | --- | | load\_ext2(self) | source code | |
|  | |  |  | | --- | --- | | load\_ext4(self) | source code | |
|  | |  |  | | --- | --- | | get\_extension(self, code) | source code | |
|  | |  |  | | --- | --- | | find\_class(self, module, name) | source code | |
|  | |  |  | | --- | --- | | load\_reduce(self) | source code | |
|  | |  |  | | --- | --- | | load\_pop(self) | source code | |
|  | |  |  | | --- | --- | | load\_pop\_mark(self) | source code | |
|  | |  |  | | --- | --- | | load\_dup(self) | source code | |
|  | |  |  | | --- | --- | | load\_get(self) | source code | |
|  | |  |  | | --- | --- | | load\_binget(self) | source code | |
|  | |  |  | | --- | --- | | load\_long\_binget(self) | source code | |
|  | |  |  | | --- | --- | | load\_put(self) | source code | |
|  | |  |  | | --- | --- | | load\_binput(self) | source code | |
|  | |  |  | | --- | --- | | load\_long\_binput(self) | source code | |
|  | |  |  | | --- | --- | | load\_append(self) | source code | |
|  | |  |  | | --- | --- | | load\_appends(self) | source code | |
|  | |  |  | | --- | --- | | load\_setitem(self) | source code | |
|  | |  |  | | --- | --- | | load\_setitems(self) | source code | |
|  | |  |  | | --- | --- | | load\_build(self) | source code | |
|  | |  |  | | --- | --- | | load\_mark(self) | source code | |
|  | |  |  | | --- | --- | | load\_stop(self) | source code | |


|  |  |  |  |
| --- | --- | --- | --- |
| |  |  | | --- | --- | | Class Variables | [hide private] | | |
|  | dispatch = `{}` |


|  |  |  |  |
| --- | --- | --- | --- |
| |  |  | | --- | --- | | Method Details | [hide private] | | |

|  |  |  |
| --- | --- | --- |
| |  |  | | --- | --- | | \_\_init\_\_(self, file)  *(Constructor)* | source code |   This takes a file-like object for reading a pickle data stream.  The protocol version of the pickle is detected automatically, so no proto argument is needed.  The file-like object must have two methods, a read() method that takes an integer argument, and a readline() method that requires no arguments. Both methods should return a string. Thus file-like object can be a file object opened for reading, a StringIO object, or any other custom object that meets this interface. |

|  |  |  |
| --- | --- | --- |
| |  |  | | --- | --- | | load(self) | source code |   Read a pickled object representation from the open file.  Return the reconstituted object hierarchy specified in the file. |

  


| Home | Trees | Indices | Help | | PyDSTool | | --- | |
| --- | --- | --- | --- | --- | --- |

|  |  |
| --- | --- |
| Generated by Epydoc 3.0.1 on Fri May 4 15:24:10 2012 | http://epydoc.sourceforge.net |
