# Supplementary material for: Hybrid Models and Biological Model Reduction with PyDSTool
Source: PLoS Comput Biol. 2012 Aug 9;8(8):e1002628. doi: 10.1371/journal.pcbi.1002628 (PMC3415397; doi:10.1371/journal.pcbi.1002628)
Supplement: Text S4 — Complete source code for the PyDSTool package (version 0.88.120504). Includes API documentation and help files linking to web pages. This file is identical to the current public release on Sourceforge.net. (ZIP) [file pcbi.1002628.s004.zip › PyDSTool/html/PyDSTool.FuncSpec'-module.html]

xml version="1.0" encoding="ascii"?


PyDSTool.FuncSpec'


| Home | Trees | Indices | Help | | PyDSTool | | --- | |
| --- | --- | --- | --- | --- | --- |

|  |  |  |  |
| --- | --- | --- | --- |
| Package PyDSTool :: Module FuncSpec' | |  | | --- | | [hide private] | | [frames] | no frames] | |

# Module FuncSpec'

source code

```
Functional specification classes.

   Robert Clewley, August 2005.

This module aids in building internal representations of ODEs, etc.,
particularly for the benefit of Automatic Differentiation
and for manipulation of abstraction digraphs.
```


|  |  |  |  |
| --- | --- | --- | --- |
| |  |  | | --- | --- | | Classes | [hide private] | | |
|  | ExpFuncSpec  Explicit definition of vars defined. |
|  | FuncSpec  Functional specification of dynamics: abstract class. |
|  | ImpFuncSpec  Assumes this will be set to equal zero when solving for vars defined. |
|  | RHSfuncSpec  Right-hand side definition for vars defined. |


|  |  |  |  |
| --- | --- | --- | --- |
| |  |  | | --- | --- | | Functions | [hide private] | | |
|  | |  |  | | --- | --- | | \_processReused(specnames, specdict, reuseterms, indentstr=`'``'`, typestr=`'``'`, endstatementchar=`'``'`, parseFunc=<function idfn at 0x1565470>)  Process substitutions of reused terms. | source code | |
|  | |  |  | | --- | --- | | getSpecFromFile(specfilename)  Read text specs from a file | source code | |
|  | |  |  | | --- | --- | | makePartialJac(spec\_pair, varnames, select=None)  Use this when parameters have been added to a modified Generator which might clash with aux fn argument names. | source code | |
|  | |  |  | | --- | --- | | resolveClashingAuxFnPars(fnspecs, varspecs, parnames)  Use this when parameters have been added to a modified Generator which might clash with aux fn argument names. | source code | |


|  |  |  |  |
| --- | --- | --- | --- |
| |  |  | | --- | --- | | Variables | [hide private] | | |
|  | API = `API_class()` |
|  | Continuous = `Continuous Domain` |
|  | Discrete = `Discrete Domain` |
|  | LargestInt32 = `2147483647` |
|  | NAMESEP = `'.'` |
|  | ONES = `['1', '1.0', '1.', '(1)', '(1.0)', '(1.)']` |
|  | ZEROS = `['0', '0.0', '0.', '(0)', '(0.0)', '(0.)']` |
|  | \_1DimplicitSolveMethods = `['newton', 'bisect', 'steffe']` |
|  | \_all\_complex = `(<type 'complex'>, <type 'numpy.complexfloating...` |
|  | \_all\_float = `(<type 'float'>, <type 'numpy.floating'>, <type '...` |
|  | \_all\_int = `(<type 'int'>, <type 'numpy.integer'>, <type 'numpy...` |
|  | \_all\_numpy\_complex = `(<type 'numpy.complex128'>, <type 'numpy....` |
|  | \_all\_numpy\_float = `(<type 'numpy.float64'>, <type 'numpy.float...` |
|  | \_all\_numpy\_int = `(<type 'numpy.int32'>, <type 'numpy.int32'>, ...` |
|  | \_complex\_types = `(<type 'complex'>, <type 'numpy.complexfloati...` |
|  | \_float\_types = `(<type 'float'>, <type 'numpy.floating'>)` |
|  | \_implicitSolveMethods = `['newton', 'bisect', 'steffe', 'fsolve']` |
|  | \_indentstr = `' '` |
|  | \_int\_types = `(<type 'int'>, <type 'numpy.integer'>)` |
|  | \_num\_equivtype = `{<type 'float'>: <type 'numpy.float64'>, <typ...` |
|  | \_num\_maxmin = `{<type 'numpy.int32'>: [-2147483648, 2147483647]...` |
|  | \_num\_name2equivtypes = `{'float': (<type 'float'>, <type 'numpy...` |
|  | \_num\_name2type = `{'float': <type 'numpy.float64'>, 'int': <typ...` |
|  | \_num\_type2name = `{<type 'float'>: 'float', <type 'int'>: 'int'...` |
|  | \_num\_types = `(<type 'float'>, <type 'int'>, <type 'numpy.float...` |
|  | \_pytypefromtype = `{<type 'numpy.int32'>: <type 'int'>, <type '...` |
|  | \_real\_types = `(<type 'int'>, <type 'numpy.integer'>, <type 'fl...` |
|  | \_seq\_types = `(<type 'list'>, <type 'tuple'>, <type 'numpy.ndar...` |
|  | builtinFnSigInfo = `{'for': 4, 'getbound': 2, 'getindex': 1, 'g...` |
|  | builtin\_auxnames = `['globalindepvar', 'initcond', 'heav', 'if'...` |
|  | name\_chars\_RE = `re.compile(r'\w')` |
|  | null\_predicate = `null_predicate_class(None)` |
|  | num\_chars = `['0', '1', '2', '3', '4', '5', '6', '7', '8', '9']` |
|  | protected\_allnames = `['acos', 'asin', 'atan', 'atan2', 'ceil',...` |
|  | protected\_auxnamesDB = `ModelSpec internal helper class: auxfnD...` |
|  | protected\_macronames = `['for', 'if', 'max', 'min', 'sum']` |
|  | protected\_mathnames = `['acos', 'asin', 'atan', 'atan2', 'ceil'...` |
|  | protected\_randomnames = `['BPF', 'LOG4', 'NV_MAGICCONST', 'RECI...` |
|  | protected\_scipynames = `['sign', 'mod']` |
|  | protected\_specialfns = `['special_airy', 'special_airye', 'spec...` |
|  | syms = `{0: 'ENDMARKER', 1: 'NAME', 2: 'NUMBER', 3: 'STRING', 4...` |
|  | targetLangs = `['c', 'python', 'matlab']` |


|  |  |  |  |
| --- | --- | --- | --- |
| |  |  | | --- | --- | | Function Details | [hide private] | | |

|  |  |  |
| --- | --- | --- |
| |  |  | | --- | --- | | makePartialJac(spec\_pair, varnames, select=None) | source code |  ``` Use this when parameters have been added to a modified Generator which might clash with aux fn argument names. (E.g., used by find_nullclines).  'select' option (list of varnames) selects those entries from the Jac of the varnames,    e.g. for constructing Jacobian w.r.t. 'parameters' using a parameter formerly    a variable (e.g. for find_nullclines). ``` |

|  |  |  |
| --- | --- | --- |
| |  |  | | --- | --- | | resolveClashingAuxFnPars(fnspecs, varspecs, parnames) | source code |   Use this when parameters have been added to a modified Generator which might clash with aux fn argument names. (E.g., used by find\_nullclines). Will remove arguments that are now considered parameters by the system, in both the function definitions and their use in specs for the variables. |

  


|  |  |  |  |
| --- | --- | --- | --- |
| |  |  | | --- | --- | | Variables Details | [hide private] | | |

|  |  |
| --- | --- |
| \_all\_complex   Value:  |  | | --- | | ``` (<type 'complex'>,  <type 'numpy.complexfloating'>,  <type 'numpy.complex128'>,  <type 'numpy.complex64'>,  <type 'numpy.complex128'>) ``` | |

|  |  |
| --- | --- |
| \_all\_float   Value:  |  | | --- | | ``` (<type 'float'>,  <type 'numpy.floating'>,  <type 'numpy.float64'>,  <type 'numpy.float32'>,  <type 'numpy.float64'>) ``` | |

|  |  |
| --- | --- |
| \_all\_int   Value:  |  | | --- | | ``` (<type 'int'>,  <type 'numpy.integer'>,  <type 'numpy.int32'>,  <type 'numpy.int32'>,  <type 'numpy.int8'>,  <type 'numpy.int16'>,  <type 'numpy.int32'>,  <type 'numpy.int64'>) ``` | |

|  |  |
| --- | --- |
| \_all\_numpy\_complex   Value:  |  | | --- | | ``` (<type 'numpy.complex128'>,  <type 'numpy.complex64'>,  <type 'numpy.complex128'>) ``` | |

|  |  |
| --- | --- |
| \_all\_numpy\_float   Value:  |  | | --- | | ``` (<type 'numpy.float64'>,  <type 'numpy.float32'>,  <type 'numpy.float64'>) ``` | |

|  |  |
| --- | --- |
| \_all\_numpy\_int   Value:  |  | | --- | | ``` (<type 'numpy.int32'>,  <type 'numpy.int32'>,  <type 'numpy.int8'>,  <type 'numpy.int16'>,  <type 'numpy.int32'>,  <type 'numpy.int64'>) ``` | |

|  |  |
| --- | --- |
| \_complex\_types   Value:  |  | | --- | | ``` (<type 'complex'>, <type 'numpy.complexfloating'>) ``` | |

|  |  |
| --- | --- |
| \_num\_equivtype   Value:  |  | | --- | | ``` {<type 'float'>: <type 'numpy.float64'>,  <type 'int'>: <type 'numpy.int32'>,  <type 'numpy.integer'>: <type 'numpy.int32'>,  <type 'numpy.floating'>: <type 'numpy.float64'>,  <type 'numpy.int8'>: <type 'numpy.int32'>,  <type 'numpy.int16'>: <type 'numpy.int32'>,  <type 'numpy.int32'>: <type 'numpy.int32'>,  <type 'numpy.int32'>: <type 'numpy.int32'>, ... ``` | |

|  |  |
| --- | --- |
| \_num\_maxmin   Value:  |  | | --- | | ``` {<type 'numpy.int32'>: [-2147483648, 2147483647],  <type 'numpy.float64'>: [-inf, inf]} ``` | |

|  |  |
| --- | --- |
| \_num\_name2equivtypes   Value:  |  | | --- | | ``` {'float': (<type 'float'>,            <type 'numpy.floating'>,            <type 'numpy.float64'>,            <type 'numpy.float32'>,            <type 'numpy.float64'>),  'int': (<type 'int'>,          <type 'numpy.integer'>,          <type 'numpy.int32'>, ... ``` | |

|  |  |
| --- | --- |
| \_num\_name2type   Value:  |  | | --- | | ``` {'float': <type 'numpy.float64'>, 'int': <type 'numpy.int32'>} ``` | |

|  |  |
| --- | --- |
| \_num\_type2name   Value:  |  | | --- | | ``` {<type 'float'>: 'float',  <type 'int'>: 'int',  <type 'numpy.integer'>: 'int',  <type 'numpy.floating'>: 'float',  <type 'numpy.int8'>: 'int',  <type 'numpy.int16'>: 'int',  <type 'numpy.int32'>: 'int',  <type 'numpy.int32'>: 'int', ... ``` | |

|  |  |
| --- | --- |
| \_num\_types   Value:  |  | | --- | | ``` (<type 'float'>,  <type 'int'>,  <type 'numpy.floating'>,  <type 'numpy.integer'>) ``` | |

|  |  |
| --- | --- |
| \_pytypefromtype   Value:  |  | | --- | | ``` {<type 'numpy.int32'>: <type 'int'>,  <type 'numpy.float64'>: <type 'float'>} ``` | |

|  |  |
| --- | --- |
| \_real\_types   Value:  |  | | --- | | ``` (<type 'int'>,  <type 'numpy.integer'>,  <type 'float'>,  <type 'numpy.floating'>) ``` | |

|  |  |
| --- | --- |
| \_seq\_types   Value:  |  | | --- | | ``` (<type 'list'>, <type 'tuple'>, <type 'numpy.ndarray'>) ``` | |

|  |  |
| --- | --- |
| builtinFnSigInfo   Value:  |  | | --- | | ``` {'for': 4,  'getbound': 2,  'getindex': 1,  'globalindepvar': 1,  'heav': 1,  'if': 3,  'initcond': 1,  'max': 1, ... ``` | |

|  |  |
| --- | --- |
| builtin\_auxnames   Value:  |  | | --- | | ``` ['globalindepvar', 'initcond', 'heav', 'if', 'getindex', 'getbound'] ``` | |

|  |  |
| --- | --- |
| protected\_allnames   Value:  |  | | --- | | ``` ['acos',  'asin',  'atan',  'atan2',  'ceil',  'cos',  'cosh',  'degrees', ... ``` | |

|  |  |
| --- | --- |
| protected\_auxnamesDB   Value:  |  | | --- | | ``` ModelSpec internal helper class: auxfnDBclass object ``` | |

|  |  |
| --- | --- |
| protected\_mathnames   Value:  |  | | --- | | ``` ['acos',  'asin',  'atan',  'atan2',  'ceil',  'cos',  'cosh',  'degrees', ... ``` | |

|  |  |
| --- | --- |
| protected\_randomnames   Value:  |  | | --- | | ``` ['BPF',  'LOG4',  'NV_MAGICCONST',  'RECIP_BPF',  'Random',  'SG_MAGICCONST',  'SystemRandom',  'TWOPI', ... ``` | |

|  |  |
| --- | --- |
| protected\_specialfns   Value:  |  | | --- | | ``` ['special_airy',  'special_airye',  'special_ai_zeros',  'special_bi_zeros',  'special_ellipj',  'special_ellipk',  'special_ellipkinc',  'special_ellipe', ... ``` | |

|  |  |
| --- | --- |
| syms   Value:  |  | | --- | | ``` {0: 'ENDMARKER',  1: 'NAME',  2: 'NUMBER',  3: 'STRING',  4: 'NEWLINE',  5: 'INDENT',  6: 'DEDENT',  7: 'LPAR', ... ``` | |

  


| Home | Trees | Indices | Help | | PyDSTool | | --- | |
| --- | --- | --- | --- | --- | --- |

|  |  |
| --- | --- |
| Generated by Epydoc 3.0.1 on Fri May 4 15:24:02 2012 | http://epydoc.sourceforge.net |
