# Supplementary material for: Hybrid Models and Biological Model Reduction with PyDSTool
Source: PLoS Comput Biol. 2012 Aug 9;8(8):e1002628. doi: 10.1371/journal.pcbi.1002628 (PMC3415397; doi:10.1371/journal.pcbi.1002628)
Supplement: Text S4 — Complete source code for the PyDSTool package (version 0.88.120504). Includes API documentation and help files linking to web pages. This file is identical to the current public release on Sourceforge.net. (ZIP) [file pcbi.1002628.s004.zip › PyDSTool/html/PyDSTool.FuncSpec'-pysrc.html]

xml version="1.0" encoding="ascii"?


PyDSTool.FuncSpec'


| Home | Trees | Indices | Help | | PyDSTool | | --- | |
| --- | --- | --- | --- | --- | --- |

|  |  |  |  |
| --- | --- | --- | --- |
| Package PyDSTool :: Module FuncSpec' | |  | | --- | | [hide private] | | [frames] | no frames] | |

# Source Code for Module PyDSTool.FuncSpec'

```
   1  """Functional specification classes.
 
   2  
 
   3     Robert Clewley, August 2005.
 
   4  
 
   5  This module aids in building internal representations of ODEs, etc.,
 
   6  particularly for the benefit of Automatic Differentiation
 
   7  and for manipulation of abstraction digraphs.
 
   8  """ 
   9  
 
  10  # PyDSTool imports
 
  11  from __future__ import division 
  12  from utils import * 
  13  from common import * 
  14  from parseUtils import * 
  15  from errors import * 
  16  from utils import info as utils_info 
  17  from Symbolic import QuantSpec 
  18  
 
  19  # Other imports
 
  20  from copy import copy, deepcopy 
  21  import math, random, numpy, scipy, scipy.special 
  22  from numpy import any 
  23  
 
  24  __all__ = ['RHSfuncSpec', 'ImpFuncSpec', 'ExpFuncSpec', 'FuncSpec',
 
  25             'getSpecFromFile', 'resolveClashingAuxFnPars', 'makePartialJac'] 
  26  
 
  27  # ---------------------------------------------------------------
 
  28  
 


29 -class FuncSpec(object):


30      """Functional specification of dynamics: abstract class.
 
  31  
 
  32      NOTES ON BUILT-IN AUX FUNCTIONS (WITH SYNTAX AS USED IN SPEC STRING):
 
  33  
 
  34      globalindepvar(t) -> global independent variable (time) reference
 
  35  
 
  36      initcond(varname) -> initial condition of that variable in this DS
 
  37  
 
  38      heav(x) = 1 if x > 0, 0 otherwise
 
  39  
 
  40      getindex(varname) -> index of varname in internal representation of
 
  41       variables as array
 
  42  
 
  43      getbound(name, which_bd) -> value of user-defined bound on the named
 
  44       variable or parameter, either the lower (which_bd=0) or higher
 
  45       (which_bd=1)
 
  46  
 
  47      if(condition, expr1, expr2) -> if condition as a function of state,
 
  48       parameters and time is true, then evaluate <expr1>, else evaluate
 
  49       <expr2>.
 
  50  
 
  51      MACRO `for` SYNTAX:
 
  52  
 
  53       for(i, ilo, ihi, expr_in_i) -> list of expressions where each
 
  54        occurrence of `[i]` is replaced with the appropriate integer.
 
  55        The letter i can be replaced with any other single character.
 
  56  
 
  57      MACRO `sum` SYNTAX:
 
  58  
 
  59       sum(i, ilo, ihi, expr_in_i) -> an expression that sums
 
  60        over the expression replacing any occurrence of `[i]` with
 
  61        the appropriate integer.
 
  62      """ 
  63  
 


64 -    def __init__(self, kw):


65          # All math package names are reserved
 
  66          self._protected_mathnames = protected_mathnames 
  67          self._protected_randomnames = protected_randomnames 
  68          self._protected_scipynames = protected_scipynames 
  69          self._protected_specialfns = protected_specialfns 
  70          # We add internal default auxiliary function names for use by
 
  71          # functional specifications.
 
  72          self._builtin_auxnames = builtin_auxnames 
  73          self._protected_macronames = protected_macronames 
  74          self._protected_auxnames = copy(self._builtin_auxnames) 
  75          self._protected_reusenames = []   # for reusable sub-expression terms 
  76          needKeys = ['name', 'vars'] 
  77          optionalKeys = ['pars', 'inputs', 'varspecs', 'spec',
 
  78                     'targetlang', 'fnspecs', 'auxvars', 'reuseterms',
 
  79                     'codeinsert_start', 'codeinsert_end', 'ignorespecial'] 
  80          self._initargs = deepcopy(kw) 
  81          # PROCESS NECESSARY KEYS -------------------
 
  82          try: 
  83              # spec name
 
  84              if 'name' in kw: 
  85                  self.name = kw['name'] 
  86              else: 
  87                  self.name = 'untitled' 
  88              # declare variables (name list)
 
  89              if isinstance(kw['vars'], list): 
  90                  vars = kw['vars'][:]  # take copy 
  91              else: 
  92                  assert isinstance(kw['vars'], str), 'Invalid variable name' 
  93                  vars = [kw['vars']] 
  94          except KeyError: 
  95              raise PyDSTool_KeyError('Necessary keys missing from argument dict') 
  96          foundKeys = len(needKeys) 
  97          # PROCESS OPTIONAL KEYS --------------------
 
  98          # declare pars (name list)
 
  99          if 'pars' in kw: 
 100              if isinstance(kw['pars'], list): 
 101                  pars = kw['pars'][:]  # take copy 
 102              else: 
 103                  assert isinstance(kw['pars'], str), 'Invalid parameter name' 
 104                  pars = [kw['pars']] 
 105              foundKeys += 1 
 106          else: 
 107              pars = [] 
 108          # declare external inputs (name list)
 
 109          if 'inputs' in kw: 
 110              if isinstance(kw['inputs'], list): 
 111                  inputs = kw['inputs'][:]   # take copy 
 112              else: 
 113                  assert isinstance(kw['inputs'], str), 'Invalid input name' 
 114                  inputs = [kw['inputs']] 
 115              foundKeys += 1 
 116          else: 
 117              inputs = [] 
 118          if 'targetlang' in kw: 
 119              try: 
 120                  tlang = kw['targetlang'].lower() 
 121              except AttributeError: 
 122                  raise TypeError("Expected string type for target language") 
 123              if tlang not in targetLangs: 
 124                  raise ValueError('Invalid specification for targetlang') 
 125              self.targetlang = tlang 
 126              foundKeys += 1 
 127          else: 
 128              self.targetlang = 'python'  # default 
 129          if self.targetlang == 'c': 
 130              self._defstr = "#define" 
 131              self._undefstr = "#undef" 
 132          else: 
 133              self._defstr = "" 
 134              self._undefstr = "" 
 135          if 'ignorespecial' in kw: 
 136              self._ignorespecial = kw['ignorespecial'] 
 137              foundKeys += 1 
 138          else: 
 139              self._ignorespecial = [] 
 140          # ------------------------------------------
 
 141          # reusable terms in function specs
 
 142          if 'reuseterms' in kw: 
 143              if isinstance(kw['reuseterms'], dict): 
 144                  self.reuseterms = deepcopy(kw['reuseterms']) 
 145              else: 
 146                  raise ValueError('reuseterms must be a dictionary of strings ->'
 
 147                                     ' replacement strings') 
 148              ignore_list = [] 
 149              for term, repterm in self.reuseterms.iteritems(): 
 150                  assert isinstance(term, str), \
 
 151                         "terms in 'reuseterms' dictionary must be strings" 
 152  ##                if term[0] in num_chars+['.']:
 
 153  ##                    raise ValueError('terms must not be numerical values')
 
 154                  if isNumericToken(term): 
 155                      # don't replace numeric terms (sometimes these are
 
 156                      # generated automatically by Constructors when resolving
 
 157                      # explicit variable inter-dependencies)
 
 158                      ignore_list.append(term) 
 159                  # not sure about the next check any more...
 
 160                  # what is the point of not allowing subs terms to begin with op?
 
 161                  if term[0] in '+/*': 
 162                      print "Error in term:", term 
 163                      raise ValueError('terms to be substituted must not begin '
 
 164                                       'with arithmetic operators') 
 165                  if term[0] == '-': 
 166                      term = '(' + term + ')' 
 167                  if term[-1] in '+-/*': 
 168                      print "Error in term:", term 
 169                      raise ValueError('terms to be substituted must not end with '
 
 170                                       'arithmetic operators') 
 171                  for s in term: 
 172                      if self.targetlang == 'python': 
 173                          if s in '[]{}~!@#$%&\|?><^': 
 174                              print "Error in term:", term 
 175                              raise ValueError('terms to be substituted must be '
 
 176                                  'alphanumeric or contain arithmetic operators '
 
 177                                  '+ - / *') 
 178                      else: 
 179                          if s in '[]{}~!@#$%&\|?><': # removed ^ from this list 
 180                              print "Error in term:", term 
 181                              raise ValueError('terms to be substituted must be alphanumeric or contain arithmetic operators + - / *') 
 182                  if repterm[0] in num_chars: 
 183                      print "Error in replacement term:", repterm 
 184                      raise ValueError('replacement terms must not begin with numbers') 
 185                  for s in repterm: 
 186                      if s in '+-/*.()[]{}~!@#$%^&\|?><,': 
 187                          print "Error in replacement term:", repterm 
 188                          raise ValueError('replacement terms must be alphanumeric') 
 189              for t in ignore_list: 
 190                  del self.reuseterms[t] 
 191              foundKeys += 1 
 192          else: 
 193              self.reuseterms = {} 
 194          # auxiliary variables declaration
 
 195          if 'auxvars' in kw: 
 196              if isinstance(kw['auxvars'], list): 
 197                  auxvars = kw['auxvars'][:]   # take copy 
 198              else: 
 199                  assert isinstance(kw['auxvars'], str), 'Invalid variable name' 
 200                  auxvars = [kw['auxvars']] 
 201              foundKeys += 1 
 202          else: 
 203              auxvars = [] 
 204          # auxfns dict of functionality for auxiliary functions (in
 
 205          # either python or C). for instance, these are used for global
 
 206          # time reference, access of regular variables to initial
 
 207          # conditions, and user-defined quantities.
 
 208          self.auxfns = {} 
 209          if 'fnspecs' in kw: 
 210              self._auxfnspecs = deepcopy(kw['fnspecs']) 
 211              foundKeys += 1 
 212          else: 
 213              self._auxfnspecs = {} 
 214          # spec dict of functionality, as a string for each var
 
 215          # (in either python or C, or just for python?)
 
 216          assert 'varspecs' in kw or 'spec' in kw, ("Require a functional "
 
 217                                  "specification key -- 'spec' or 'varspecs'") 
 218          if 'varspecs' in kw: 
 219              if auxvars == []: 
 220                  numaux = 0 
 221              else: 
 222                  numaux = len(auxvars) 
 223              if len(kw['varspecs']) != len(vars)+numaux: 
 224                  print "# state variables: ", len(vars) 
 225                  print "# auxiliary variables: ", numaux 
 226                  print "# of variable specs: ", len(kw['varspecs']) 
 227                  raise ValueError('Incorrect size of varspecs') 
 228              self.varspecs = deepcopy(kw['varspecs']) 
 229              foundKeys += 1 
 230          else: 
 231              self.varspecs = {} 
 232          self.codeinserts = {'start': '', 'end': ''} 
 233          if 'codeinsert_start' in kw: 
 234              codestr = kw['codeinsert_start'] 
 235              assert isinstance(codestr, str), 'code insert must be a string' 
 236              if self.targetlang == 'python': 
 237                  # check initial indentation (as early predictor of whether
 
 238                  # indentation has been done properly)
 
 239                  if codestr[:4] != _indentstr: 
 240                      codestr = _indentstr+codestr 
 241              # additional spacing in function spec
 
 242              if codestr[-1] != '\n': 
 243                  addnl = '\n' 
 244              else: 
 245                  addnl = '' 
 246              self.codeinserts['start'] = codestr+addnl 
 247              foundKeys += 1 
 248          if 'codeinsert_end' in kw: 
 249              codestr = kw['codeinsert_end'] 
 250              assert isinstance(codestr, str), 'code insert must be a string' 
 251              if self.targetlang == 'python': 
 252                  # check initial indentation (as early predictor of whether
 
 253                  # indentation has been done properly)
 
 254                  assert codestr[:4] == "    ", ("First line of inserted "
 
 255                                          "python code at start of spec was "
 
 256                                                 "wrongly indented") 
 257              # additional spacing in function spec
 
 258              if codestr[-1] != '\n': 
 259                  addnl = '\n' 
 260              else: 
 261                  addnl = '' 
 262              self.codeinserts['end'] = codestr+addnl 
 263              foundKeys += 1 
 264          # spec dict of functionality, as python functions,
 
 265          # or the paths/names of C dynamic linked library files
 
 266          # can be user-defined or generated from generateSpec
 
 267          if 'spec' in kw: 
 268              if 'varspecs' in kw: 
 269                  raise PyDSTool_KeyError, \
 
 270                        "Cannot provide both 'spec' and 'varspecs' keys" 
 271              assert isinstance(kw['spec'], tuple), ("'spec' must be a pair:"
 
 272                                      " (spec body, spec name)") 
 273              assert len(kw['spec'])==2, ("'spec' must be a pair:"
 
 274                                      " (spec body, spec name)") 
 275              self.spec = deepcopy(kw['spec']) 
 276              # auxspec not used for explicitly-given specs. it's only for
 
 277              # auto-generated python auxiliary variable specs (as py functions)
 
 278              self.auxspec = {} 
 279              if 'dependencies' in kw: 
 280                  self.dependencies = kw['dependencies'] 
 281              else: 
 282                  raise PyDSTool_KeyError("Dependencies must be provided "
 
 283                           "explicitly when using 'spec' form of initialization") 
 284              foundKeys += 2 
 285          else: 
 286              self.spec = {} 
 287              self.auxspec = {} 
 288              self.dependencies = [] 
 289          if len(kw) > foundKeys: 
 290              raise PyDSTool_KeyError('Invalid keys passed in argument dict') 
 291          self.defined = False  # initial value 
 292          self.validateDef(vars, pars, inputs, auxvars, self._auxfnspecs.keys()) 
 293          # ... exception if not valid
 
 294          # Fine to do the following if we get this far:
 
 295          # sort for final order that will be used for determining array indices
 
 296          vars.sort() 
 297          pars.sort() 
 298          inputs.sort() 
 299          auxvars.sort() 
 300          self.vars = vars 
 301          self.pars = pars 
 302          self.inputs = inputs 
 303          self.auxvars = auxvars 
 304          # pre-process specification string for built-in macros (like `for`,
 
 305          # i.e. that are not also auxiliary functions, like the in-line `if`)
 
 306          self.doPreMacros() 
 307          # !!!
 
 308          # want to create _pyauxfns but not C versions until after main spec
 
 309          # !!!
 
 310          self.generateAuxFns() 
 311          if self.spec == {}: 
 312              assert self.varspecs != {}, \
 
 313                     'No functional specification provided!' 
 314              self.generateSpec() 
 315              # exception if the following is not successful
 
 316              self.validateDependencies(self.dependencies) 
 317          #self.generateAuxFns()
 
 318  ##        self.validateSpecs()
 
 319          # algparams is only used by ImplicitFnGen to pass extra info to Variable
 
 320          self.algparams = {} 
 321          self.defined = True

 322  
 


323 -    def __hash__(self):


324          """Unique identifier for this specification.""" 
 325          deflist = [self.name, self.targetlang] 
 326          # lists
 
 327          for l in [self.pars, self.vars, self.auxvars, self.inputs,
 
 328                    self.spec, self.auxspec]: 
 329              deflist.append(tuple(l)) 
 330          # dicts
 
 331          for d in [self.auxfns, self.codeinserts]: 
 332              deflist.append(tuple(sortedDictItems(d, byvalue=False))) 
 333          return hash(tuple(deflist))

 334  
 


335 -    def recreate(self, targetlang):


336          if targetlang == self.targetlang: 
 337  #            print "Returning a deep copy of self"
 
 338              return deepcopy(self) 
 339          fs = FuncSpec.__new__(self.__class__) 
 340          new_args = deepcopy(self._initargs) 
 341          if self.codeinserts['start'] != '': 
 342              del new_args['codeinsert_start'] 
 343              print "Warning: code insert (start) ignored for new target" 
 344          if self.codeinserts['end'] != '': 
 345              del new_args['codeinsert_end'] 
 346              print "Warning: code insert (end) ignored for new target" 
 347          new_args['targetlang'] = targetlang 
 348          fs.__init__(new_args) 
 349          return fs

 350  
 
 351  
 


352 -    def __call__(self):


353          # info is defined in utils.py
 
 354          utils_info(self.__dict__, "FuncSpec " + self.name)

 355  
 
 356  
 
 357  #    def info(self, verbose=1):
 
 358  #        if verbose > 0:
 
 359  #            # info is defined in utils.py
 
 360  #            utils_info(self.__dict__, "FuncSpec " + self.name,
 
 361  #                 recurseDepthLimit=1+verbose)
 
 362  #        else:
 
 363  #            print self.__repr__()
 
 364  
 
 365  
 
 366      # This function doesn't work -- it generates:
 
 367      #    global name 'self' is not defined
 
 368      # in the _specfn call
 
 369  ##    def validateSpecs(self):
 
 370  ##        # dummy values for internal values possibly needed by auxiliary fns
 
 371  ##        self.globalt0 = 0
 
 372  ##        self.initialconditions = {}.fromkeys(self.vars, 0)
 
 373  ##        lenparsinps = len(self.pars)+len(self.inputs)
 
 374  ##        pi_vals = zeros(lenparsinps, float64)
 
 375  ##        _specfn(1, self.initialconditions.values(), pi_vals)
 
 376  
 
 377  
 


378 -    def validateDef(self, vars, pars, inputs, auxvars, auxfns):


379          """Validate definition of the functional specification.""" 
 380          # verify that vars, pars, and inputs are non-overlapping lists
 
 381          assert not intersect(vars, pars), 'variable and param names overlap' 
 382          assert not intersect(vars, inputs), 'variable and input names overlap' 
 383          assert not intersect(pars, inputs), 'param and input names overlap' 
 384          assert not intersect(vars, auxfns), ('variable and auxiliary function '
 
 385                                               'names overlap') 
 386          assert not intersect(pars, auxfns), ('param and auxiliary function '
 
 387                                               'names overlap') 
 388          assert not intersect(inputs, auxfns), ('input and auxiliary function '
 
 389                                                 'names overlap') 
 390          assert not intersect(vars, auxvars), ('variable and auxiliary variable '
 
 391                                               'names overlap') 
 392          assert not intersect(pars, auxvars), ('param and auxiliary variable '
 
 393                                               'names overlap') 
 394          assert not intersect(inputs, auxvars), ('input and auxiliary variable '
 
 395                                                 'names overlap') 
 396          # verify uniqueness of all names
 
 397          assert isUniqueSeq(vars), 'variable names are repeated' 
 398          assert isUniqueSeq(pars), 'parameter names are repeated' 
 399          assert isUniqueSeq(inputs), 'input names are repeated' 
 400          if auxvars != []: 
 401              assert isUniqueSeq(auxvars), 'auxiliary variable names are repeated' 
 402          if auxfns != []: 
 403              assert isUniqueSeq(auxfns), 'auxiliary function names are repeated' 
 404          allnames = vars+pars+inputs+auxvars 
 405          allprotectednames = self._protected_mathnames + \
 
 406                              self._protected_scipynames + \
 
 407                              self._protected_specialfns + \
 
 408                              self._protected_randomnames + \
 
 409                              self._protected_auxnames + \
 
 410                              ['abs', 'min', 'max', 'and', 'or', 'not',
 
 411                               'True', 'False'] 
 412          # other checks
 
 413          assert reduce(bool.__and__, [name_chars_RE.match(n[0]) \
 
 414                                       is not None for n in allnames]), \
 
 415                          ('variable, parameter, and input names must not '
 
 416                           'begin with non-alphabetic chars') 
 417          assert reduce(bool.__and__, [n not in allnames for n in \
 
 418                                       allprotectednames]), \
 
 419                          ('variable, parameter, and input names must not '
 
 420                           'overlap with protected math / aux function names')

 421          ## Not yet implemented ?
 
 422          # verify that targetlang is consistent with spec contents?
 
 423          # verify that spec is consistent with specstring (if not empty)?
 
 424  
 
 425  
 


426 -    def validateDependencies(self, dependencies):


427          """Validate the stored dependency pairs for self-consistency.""" 
 428          # dependencies is a list of unique ordered pairs (i,o)
 
 429          # where (i,o) means 'variable i directly depends on variable o'
 
 430          # (o can include inputs)
 
 431          assert isinstance(dependencies, list), ('dependencies must be a list '
 
 432                                                  'of unique ordered pairs') 
 433          # Verify all names in dependencies are in self.vars
 
 434          # and that (i,o) pairs are unique in dependencies
 
 435          for d in dependencies: 
 436              assert len(d) == 2, 'dependencies must be ordered pairs' 
 437              i = d[0] 
 438              o = d[1] 
 439              firstpos = dependencies.index(d) 
 440              assert d not in dependencies[firstpos+1:], \
 
 441                     'dependency pairs must be unique' 
 442              assert i in self.vars+self.auxvars, 'unknown variable name in dependencies' 
 443              assert o in self.vars or o in self.inputs, \
 
 444                     'unknown variable name in dependencies'

 445          # No need to verify that dependencies are consistent with spec,
 
 446          # if spec was generated automatically
 
 447  
 
 448  
 


449 -    def generateAuxFns(self):


450          # Always makes a set of python versions of the functions for future
 
 451          # use by user at python level
 
 452          if self.targetlang == 'python': 
 453              self._genAuxFnPy(pytarget=True) 
 454          elif self.targetlang == 'c': 
 455              self._genAuxFnC() 
 456              self._genAuxFnPy() 
 457          elif self.targetlang == 'matlab': 
 458              self._genAuxFnMatlab() 
 459              self._genAuxFnPy() 
 460          elif self.targetlang == 'dstool': 
 461              raise NotImplementedError 
 462          elif self.targetlang == 'xpp': 
 463              raise NotImplementedError 
 464          else: 
 465              raise ValueError('targetlang attribute must be in '+str(targetLangs))

 466  
 
 467  
 


468 -    def generateSpec(self):


469          """Automatically generate callable target-language functions from
 
 470          the user-defined specification strings.""" 
 471          if self.targetlang == 'python': 
 472              self._genSpecPy() 
 473          elif self.targetlang == 'c': 
 474              self._genSpecC() 
 475          elif self.targetlang == 'matlab': 
 476              self._genSpecMatlab() 
 477          elif self.targetlang == 'odetools': 
 478              raise NotImplementedError 
 479          elif self.targetlang == 'xpp': 
 480              raise NotImplementedError 
 481          else: 
 482              raise ValueError('targetlang attribute must be in '+str(targetLangs))

 483  
 
 484  
 


485 -    def doPreMacros(self):


486          """Pre-process any macro spec definitions (e.g. `for` loops).""" 
 487  
 
 488          assert self.varspecs != {}, 'varspecs attribute must be defined' 
 489          specnames_unsorted = self.varspecs.keys() 
 490          specname_vars = intersect(self.vars, specnames_unsorted) 
 491          specname_vars.sort() 
 492          assert self.vars == specname_vars, ('Mismatch between declared '
 
 493                                          ' variable names and varspecs keys') 
 494          specnames_unsorted = self.varspecs.keys() 
 495          specname_auxvars = intersect(self.auxvars, specnames_unsorted) 
 496          specname_auxvars.sort() 
 497          specnames = specname_vars + specname_auxvars  # sorted *individually* 
 498          specnames_temp = copy(specnames) 
 499          for specname in specnames_temp: 
 500              leftbrack_ix = specname.find('[') 
 501              rightbrack_ix = specname.find(']') 
 502              test_sum = leftbrack_ix + rightbrack_ix 
 503              if test_sum > 0: 
 504                  # both brackets found -- we expect a `for` macro in specstr
 
 505                  assert rightbrack_ix - leftbrack_ix == 2, ('Misuse of square '
 
 506                                   'brackets in spec definition. Expected single'
 
 507                                   ' character between left and right brackets.') 
 508                  if specname in self.vars: 
 509                      foundvar = True 
 510                  else: 
 511                      foundvar = False  # auxiliary variable instead 
 512                  rootstr = specname[:leftbrack_ix] 
 513                  istr = specname[leftbrack_ix+1] 
 514                  specstr = self.varspecs[specname] 
 515                  assert specstr[:4] == 'for(', ('Expected `for` macro when '
 
 516                                  'square brackets used in name definition') 
 517                  # read contents of braces
 
 518                  arginfo = readArgs(specstr[3:]) 
 519                  if not arginfo[0]: 
 520                      raise ValueError('Error finding '
 
 521                              'arguments applicable to `for` '
 
 522                              'macro') 
 523                  arglist = arginfo[1] 
 524                  assert len(arglist) == 4, ('Wrong number of arguments passed '
 
 525                                             'to `for` macro. Expected 4') 
 526                  istr = arglist[0] 
 527                  allnames = self.vars + self.pars + self.inputs + self.auxvars \
 
 528                             + self._protected_mathnames \
 
 529                             + self._protected_randomnames \
 
 530                             + self._protected_auxnames \
 
 531                             + self._protected_scipynames \
 
 532                             + self._protected_specialfns \
 
 533                             + self._protected_macronames \
 
 534                             + ['abs', 'and', 'or', 'not', 'True', 'False'] 
 535                  assert istr not in allnames, ('loop index in `for` macro '
 
 536                                                'must not be a reserved name') 
 537                  for ichar in istr: 
 538                      assert name_chars_RE.match(ichar) is not None, \
 
 539                                                 ('loop index in `for` macro '
 
 540                                                  'must be alphanumeric') 
 541                  ilo = int(arglist[1]) 
 542                  ihi = int(arglist[2]) 
 543                  # NOTE: rootstr + '['+istr+'] = ' + arglist[3]
 
 544                  expr = arglist[3] 
 545                  # add macro text
 
 546                  varspecs = self._macroFor(rootstr, istr, ilo, ihi, expr) 
 547                  specnames_gen = varspecs.keys() 
 548                  # now we update the dictionary of specnames with the
 
 549                  # processed, expanded versions
 
 550                  specnames.remove(specname) 
 551                  if foundvar: 
 552                      assert rootstr+'['+istr+']' in self.vars, ('Mismatch '
 
 553                                                   'between declared variables '
 
 554                                                 'and loop index in `for` macro') 
 555                      self.vars.remove(specname) 
 556                  else: 
 557                      assert rootstr+'['+istr+']' in self.auxvars, ('Mismatch '
 
 558                                                   'between declared variables '
 
 559                                                 'and loop index in `for` macro') 
 560                      self.auxvars.remove(specname) 
 561                  del(self.varspecs[specname]) 
 562                  for sname in specnames_gen: 
 563                      self.varspecs[sname] = varspecs[sname] 
 564                      specnames.append(sname) 
 565                      if foundvar: 
 566                          self.vars.append(sname) 
 567                      else: 
 568                          self.auxvars.append(sname) 
 569              elif test_sum == -2: 
 570                  pass 
 571                  # no brackets found. regular definition line. take no action.
 
 572              else: 
 573                  raise AssertionError('Misuse of square brackets in spec '
 
 574                                         'definition. Expected single'
 
 575                                   ' character between left and right brackets.')

 576  
 
 577  
 


578 -    def _macroFor(self, rootstr, istr, ilo, ihi, expr_in_i):


579          """Internal utility function to build multiple instances of expression
 
 580          'expr_in_i' where integer i has been substituted for values from ilo to ihi.
 
 581          Returns dictionary keyed by rootstr+str(i) for each i.
 
 582          """ 
 583          # already tested for the same number of [ and ] occurrences
 
 584          retdict = {} 
 585          q = QuantSpec('__temp__', expr_in_i) 
 586          eval_pieces = {} 
 587          avoid_toks = [] 
 588          for ix, tok in enumerate(q): 
 589              if tok[0] == '[': 
 590                  eval_str = tok[1:-1] 
 591                  if istr in eval_str: 
 592                      eval_pieces[ix] = eval_str 
 593                  # otherwise may be a different, embedded temp index for another
 
 594                  # sum, etc., so don't touch it
 
 595          keys = eval_pieces.keys() 
 596          keys.sort() 
 597          ranges = remove_indices_from_range(keys, len(q.parser.tokenized)-1) 
 598          # By virtue of this syntax, the first [] cannot be before some other text
 
 599          pieces = [] 
 600          eval_ixs = [] 
 601          for ri, r in enumerate(ranges): 
 602              if len(r) == 1: 
 603                  pieces.append(q[r[0]]) 
 604              else: 
 605                  # len(r) == 2
 
 606                  pieces.append(''.join(q[r[0]:r[1]])) 
 607              if ri+1 == len(ranges): 
 608                  # last one - check if there's an eval piece placeholder to append at the end
 
 609                  if len(keys) > 0 and keys[-1] == r[-1]: 
 610                      pieces.append('') 
 611                      eval_ixs.append(len(pieces)-1) 
 612                  # else do nothing
 
 613              else: 
 614                  # in-between pieces, so append a placeholder for an eval piece
 
 615                  pieces.append('') 
 616                  eval_ixs.append(len(pieces)-1) 
 617          for i in range(ilo, ihi+1): 
 618              for k, ei in zip(keys, eval_ixs): 
 619                  s = eval_pieces[k].replace(istr, str(i)) 
 620                  try: 
 621                      pieces[ei] = str(int(eval(s))) 
 622                  except NameError: 
 623                      # maybe recursive 'sum' syntax, so a different index letter
 
 624                      pieces[ei] = s 
 625              retdict[rootstr+str(i)] = ''.join(pieces)+'\n' 
 626          return retdict

 627  
 


628 -    def _macroSum(self, istr, ilo, ihi, expr_in_i):


629          def_dict = self._macroFor('', istr, int(ilo), int(ihi), expr_in_i) 
 630          retstr = '(' + "+".join([term.strip() for term in def_dict.values()]) + ')' 
 631          return retstr

 632  
 
 633      # ----------------- Python specifications ----------------
 
 634  
 


635 -    def _genAuxFnPy(self, pytarget=False):


636          if pytarget: 
 637              assert self.targetlang == 'python', \
 
 638                 'Wrong target language for this call' 
 639          auxnames = self._auxfnspecs.keys() 
 640          # User aux fn interface
 
 641          uafi = {} 
 642  ##        protectednames = auxnames + self._protected_mathnames + \
 
 643  ##                         self._protected_randomnames + \
 
 644  ##                         self._protected_scipynames + \
 
 645  ##                         self._protected_specialfns + \
 
 646  ##                         ['abs', 'and', 'or', 'not', 'True', 'False']
 
 647          # Deal with built-in auxiliary functions (don't make their names unique)
 
 648          # In this version, the textual code here doesn't get executed. Only
 
 649          # the function names in the second position of the tuple are needed.
 
 650          # Later, the text will probably be removed.
 
 651          auxfns = {} 
 652          auxfns['globalindepvar'] = \
 
 653                     ("def _auxfn_globalindepvar(ds, parsinps, t):\n" \
 
 654                      + _indentstr \
 
 655                      + "return ds.globalt0 + t", '_auxfn_globalindepvar') 
 656          auxfns['initcond'] = \
 
 657                     ("def _auxfn_initcond(ds, parsinps, varname):\n" \
 
 658                      + _indentstr \
 
 659                      + "return ds.initialconditions[varname]",'_auxfn_initcond') 
 660          auxfns['heav'] = \
 
 661                     ("def _auxfn_heav(ds, parsinps, x):\n" + _indentstr \
 
 662                        + "if x>0:\n" + 2*_indentstr \
 
 663                        + "return 1\n" + _indentstr + "else:\n" \
 
 664                        + 2*_indentstr + "return 0", '_auxfn_heav') 
 665          auxfns['if'] = \
 
 666                     ("def _auxfn_if(ds, parsinps, c, e1, e2):\n" \
 
 667                      + _indentstr + "if c:\n" + 2*_indentstr \
 
 668                      + "return e1\n" + _indentstr \
 
 669                      + "else:\n" + 2*_indentstr + "return e2", '_auxfn_if') 
 670          auxfns['getindex'] = \
 
 671                     ("def _auxfn_getindex(ds, parsinps, varname):\n" \
 
 672                      + _indentstr \
 
 673                      + "return ds._var_namemap[varname]", '_auxfn_getindex') 
 674          auxfns['getbound'] = \
 
 675                     ("def _auxfn_getbound(ds, parsinps, name, bd):\n" \
 
 676                      + _indentstr + "try:\n" \
 
 677                      + 2*_indentstr + "return ds.xdomain[name][bd]\n" \
 
 678                      + _indentstr + "except KeyError:\n" + 2*_indentstr \
 
 679                      + "try:\n" + 3*_indentstr \
 
 680                      + "return ds.pdomain[name][bd]\n" + 2*_indentstr \
 
 681                      + "except KeyError, e:\n" + 3*_indentstr \
 
 682                      + "print 'Invalid var / par name %s'%name,\n" \
 
 683                      + 3*_indentstr + "print 'or bounds not well defined:'\n" \
 
 684                      + 3*_indentstr + "print ds.xdomain, ds.pdomain\n" \
 
 685                      + 3*_indentstr + "raise (RuntimeError, e)",
 
 686                      '_auxfn_getbound') 
 687          # the internal functions may be used by user-defined functions,
 
 688          # so need them to be accessible to __processTokens when parsing
 
 689          self._pyauxfns = auxfns 
 690          # add the user-defined function names for cross-referencing checks
 
 691          # (without their definitions)
 
 692          for auxname in auxnames: 
 693              self._pyauxfns[auxname] = None 
 694          # don't process the built-in functions -> unique fns because
 
 695          # they are global definitions existing throughout the
 
 696          # namespace
 
 697          self._protected_auxnames.extend(['Jacobian','Jacobian_pars']) 
 698          # protected names are the names that must not be used for
 
 699          # user-specified auxiliary fn arguments
 
 700          protectednames = self.pars + self.inputs \
 
 701                     + ['abs', 'pow', 'and', 'or', 'not', 'True', 'False'] \
 
 702                     + self._protected_auxnames + auxnames \
 
 703                     + self._protected_scipynames + self._protected_specialfns \
 
 704                     + self._protected_macronames + self._protected_mathnames \
 
 705                     + self._protected_randomnames + self._protected_reusenames 
 706          ### checks for user-defined auxiliary fns
 
 707          # name map for fixing inter-auxfn references
 
 708          auxfn_namemap = {} 
 709          specials_base = self.pars + self._protected_auxnames \
 
 710                     + ['abs', 'pow', 'and', 'or', 'not', 'True', 'False'] \
 
 711                     + auxnames + self._protected_scipynames \
 
 712                     + self._protected_specialfns \
 
 713                     + self._protected_macronames + self._protected_mathnames \
 
 714                     + self._protected_randomnames + self._protected_reusenames 
 715          for auxname in auxnames: 
 716              auxinfo = self._auxfnspecs[auxname] 
 717              try: 
 718                  if len(auxinfo) != 2: 
 719                      raise ValueError('auxinfo tuple must be of length 2') 
 720              except TypeError: 
 721                  raise TypeError('fnspecs argument must contain pairs') 
 722              # auxinfo[0] = tuple or list of parameter names
 
 723              # auxinfo[1] = string containing body of function definition
 
 724              assert isinstance(auxinfo[0], list), ('aux function arguments '
 
 725                                                    'must be given as a list') 
 726              assert isinstance(auxinfo[1], str), ('aux function specification '
 
 727                                                   'must be a string '
 
 728                                                   'of the function code') 
 729              # Process Jacobian functions, etc., specially, if present
 
 730              if auxname == 'Jacobian': 
 731                  if not compareList(auxinfo[0],['t']+self.vars): 
 732                      print ['t']+self.vars 
 733                      print "Auxinfo =", auxinfo[0] 
 734                      raise ValueError("Invalid argument list given in Jacobian.") 
 735                  auxparlist = ["t","x","parsinps"] 
 736                  # special symbols to allow in parsing function body
 
 737                  specials = ["t","x"] 
 738                  auxstr = auxinfo[1] 
 739                  if any([pt in auxstr for pt in ('^', '**')]): 
 740                      auxstr = convertPowers(auxstr, 'pow') 
 741                  specvars = self.vars 
 742                  specvars.sort() 
 743                  specdict = {}.fromkeys(specvars) 
 744                  if len(specvars) == 1: 
 745                      assert '[' not in auxstr, \
 
 746                             "'[' character invalid in Jacobian for 1D system" 
 747                      assert ']' not in auxstr, \
 
 748                             "']' character invalid in Jacobian for 1D system" 
 749                      specdict[specvars[0]] = auxstr 
 750                  else: 
 751                      specdict = parseMatrixStrToDictStr(auxstr, specvars) 
 752                  reusestr, body_processed_dict = self._processReusedPy(specvars,
 
 753                                                 specdict,
 
 754                                                 specials=specials+specials_base) 
 755                  body_processed = self._specStrParse(specvars,
 
 756                                            body_processed_dict, 'xjac',
 
 757                                            specials=specials+specials_base) 
 758                  auxstr_py = self._genSpecFnPy('_auxfn_Jac',
 
 759                                                 reusestr+body_processed,
 
 760                                                 'xjac', specvars) 
 761                  # check Jacobian
 
 762                  m = n = len(specvars) 
 763                  specdict_check = {}.fromkeys(specvars) 
 764                  for specname in specvars: 
 765                      temp = body_processed_dict[specname] 
 766                      specdict_check[specname] = \
 
 767                              count_sep(temp.replace("[","").replace("]",""))+1 
 768                  body_processed = "" 
 769                  for row in range(m): 
 770                      if specdict_check[specvars[row]] != n: 
 771                          print "Row %i: "%m, specdict[specvars[row]] 
 772                          print "Found length %i"%specdict_check[specvars[row]] 
 773                          raise ValueError("Jacobian should be %sx%s"%(m,n)) 
 774              elif auxname == 'Jacobian_pars': 
 775                  if not compareList(auxinfo[0],['t']+self.vars): 
 776                      print ['t']+self.vars 
 777                      print "Auxinfo =", auxinfo[0] 
 778                      raise ValueError("Invalid argument list given in Jacobian.") 
 779                  auxparlist = ["t","x","parsinps"] 
 780                  # special symbols to allow in parsing function body
 
 781                  specials = ["t","x"] 
 782                  auxstr = auxinfo[1] 
 783                  if any([pt in auxstr for pt in ('^', '**')]): 
 784                      auxstr = convertPowers(auxstr, 'pow') 
 785                  specvars = self.vars 
 786                  specvars.sort() 
 787                  specdict = {}.fromkeys(self.vars) 
 788                  if len(specvars) == len(self.vars) == 1: 
 789                      assert '[' not in auxstr, \
 
 790                             "'[' character invalid in Jacobian for 1D system" 
 791                      assert ']' not in auxstr, \
 
 792                             "']' character invalid in Jacobian for 1D system" 
 793                      specdict[specvars[0]] = auxstr 
 794                  else: 
 795                      specdict = parseMatrixStrToDictStr(auxstr, self.vars) 
 796                  reusestr, body_processed_dict = self._processReusedPy(self.vars,
 
 797                                                 specdict,
 
 798                                                 specials=specials+specials_base) 
 799                  body_processed = self._specStrParse(self.vars,
 
 800                                            body_processed_dict, 'pjac',
 
 801                                            specials=specials+specials_base) 
 802                  auxstr_py = self._genSpecFnPy('_auxfn_Jac_p',
 
 803                                                 reusestr+body_processed,
 
 804                                                 'pjac', self.vars) 
 805                  # check Jacobian
 
 806                  n = len(specvars) 
 807                  m = len(self.vars) 
 808                  specdict_check = {}.fromkeys(self.vars) 
 809                  for specname in self.vars: 
 810                      temp = body_processed_dict[specname] 
 811                      specdict_check[specname] = \
 
 812                              count_sep(temp.replace("[","").replace("]",""))+1 
 813                  body_processed = "" 
 814                  for row in range(m): 
 815                      try: 
 816                          if specdict_check[self.vars[row]] != n: 
 817                              print "Row %i: "%m, specdict[self.vars[row]] 
 818                              print "Found length %i"%specdict_check[self.vars[row]] 
 819                              raise ValueError("Jacobian w.r.t. pars should be %sx%s"%(m,n)) 
 820                      except IndexError: 
 821                          print "\nFound:\n" 
 822                          info(specdict) 
 823                          raise ValueError("Jacobian w.r.t. pars should be %sx%s"%(m,n)) 
 824              elif auxname == 'massMatrix': 
 825                  if not compareList(auxinfo[0],['t']+self.vars): 
 826                      print ['t']+self.vars 
 827                      print "Auxinfo =", auxinfo[0] 
 828                      raise ValueError("Invalid argument list given in Mass Matrix.") 
 829                  auxparlist = ["t","x","parsinps"] 
 830                  # special symbols to allow in parsing function body
 
 831                  specials = ["t","x"] 
 832                  auxstr = auxinfo[1] 
 833                  if any([pt in auxstr for pt in ('^', '**')]): 
 834                      auxstr = convertPowers(auxstr, 'pow') 
 835                  specvars = self.vars 
 836                  specvars.sort() 
 837                  specdict = {}.fromkeys(specvars) 
 838                  if len(specvars) == 1: 
 839                      assert '[' not in auxstr, \
 
 840                             "'[' character invalid in mass matrix for 1D system" 
 841                      assert ']' not in auxstr, \
 
 842                             "']' character invalid in mass matrix for 1D system" 
 843                      specdict[specvars.values()[0]] = auxstr 
 844                  else: 
 845                      specdict = parseMatrixStrToDictStr(auxstr, specvars) 
 846                  reusestr, body_processed_dict = self._processReusedPy(specvars,
 
 847                                                 specdict,
 
 848                                                 specials=specials+specials_base) 
 849                  body_processed = self._specStrParse(specvars,
 
 850                                            body_processed_dict, 'xmat',
 
 851                                            specials=specials+specials_base) 
 852                  auxstr_py = self._genSpecFnPy('_auxfn_massMatrix',
 
 853                                                 reusestr+body_processed,
 
 854                                                 'xmat', specvars) 
 855                  # check matrix
 
 856                  m = n = len(specvars) 
 857                  specdict_check = {}.fromkeys(specvars) 
 858                  for specname in specvars: 
 859                      specdict_check[specname] = 1 + \
 
 860                          count_sep(body_processed_dict[specname].replace("[","").replace("]","")) 
 861                  body_processed = "" 
 862                  for row in range(m): 
 863                      if specdict_check[specvars[row]] != n: 
 864                          print "Row %i: "%m, specdict[specvars[row]] 
 865                          print "Found length %i"%specdict_check[specvars[row]] 
 866                          raise ValueError("Mass matrix should be %sx%s"%(m,n)) 
 867              else: 
 868                  user_parstr = makeParList(auxinfo[0]) 
 869                  # `parsinps` is always added to allow reference to own
 
 870                  # parameters
 
 871                  if user_parstr == '': 
 872                      # no arguments, user calls as fn()
 
 873                      auxparstr = 'parsinps' 
 874                  else: 
 875                      auxparstr = 'parsinps, ' + user_parstr 
 876                  auxstr_py = 'def _auxfn_' + auxname + '(ds, ' + auxparstr \
 
 877                              +'):\n' 
 878                  auxparlist = auxparstr.replace(" ","").split(",") 
 879                  badparnames = intersect(auxparlist,
 
 880                                          remain(protectednames,auxnames)) 
 881                  if badparnames != []: 
 882                      print "Bad parameter names in auxiliary function", \
 
 883                              auxname, ":", badparnames 
 884                      #print auxinfo[0]
 
 885                      #print auxparlist
 
 886                      raise ValueError("Cannot use protected names for auxiliary "
 
 887                                         "function parameters") 
 888                  # special symbols to allow in parsing function body
 
 889                  specials = auxparlist 
 890                  specials.remove('parsinps') 
 891                  illegalterms = remain(self.vars + self.auxvars, specials) 
 892                  auxstr = auxinfo[1] 
 893                  if any([pt in auxstr for pt in ('^', '**')]): 
 894                      auxstr = convertPowers(auxstr, 'pow') 
 895                  reusestr, body_processed_dict = self._processReusedPy([auxname],
 
 896                                                 {auxname:auxstr},
 
 897                                                 specials=specials+specials_base,
 
 898                                                 dovars=False,
 
 899                                                 illegal=illegalterms) 
 900                  body_processed = self._specStrParse([auxname],
 
 901                                            body_processed_dict,
 
 902                                            specials=specials+specials_base,
 
 903                                            dovars=False,
 
 904                                            noreturndefs=True,
 
 905                                            illegal=illegalterms) 
 906                  auxstr_py += reusestr + _indentstr + 'return ' \
 
 907                            + body_processed 
 908              # syntax validation done in makeUniqueFn
 
 909              try: 
 910                  auxfns[auxname] = makeUniqueFn(auxstr_py) 
 911                  # Note: this automatically updates self._pyauxfns too
 
 912              except: 
 913                  print 'Error in supplied auxiliary spec dictionary code' 
 914                  raise 
 915              auxfn_namemap['ds.'+auxname] = 'ds.'+auxfns[auxname][1] 
 916              # prepare user-interface wrapper function (not method)
 
 917              if specials == [''] or specials == []: 
 918                  fn_args = '' 
 919              else: 
 920                  fn_args = ','+','.join(specials) 
 921              fn_elts = ['def ', auxname, '(self', fn_args,
 
 922                         ',__parsinps__=None):\n\t', 'if __parsinps__ is None:\n\t\t',
 
 923                         '__parsinps__=self.map_ixs(self.genref)\n\t',
 
 924                         'return self.genref.', auxfns[auxname][1],
 
 925                         '(__parsinps__', fn_args, ')\n'] 
 926              uafi[auxname] =  ''.join(fn_elts) 
 927          # resolve inter-auxiliary function references
 
 928          for auxname, auxspec in auxfns.iteritems(): 
 929              dummyQ = QuantSpec('dummy', auxspec[0], preserveSpace=True,
 
 930                                 treatMultiRefs=False) 
 931              dummyQ.mapNames(auxfn_namemap) 
 932              auxfns[auxname] = (dummyQ(), auxspec[1]) 
 933          if pytarget: 
 934              self.auxfns = auxfns 
 935          # keep _pyauxfns handy for users to access python versions of functions
 
 936          # from python, even using non-python target languages
 
 937          #
 
 938          # Changes to auxfns was already changing self._pyauxfns so the following line
 
 939          # is not needed
 
 940          #self._pyauxfns.update(auxfns)  # same thing if pytarget==True
 
 941          self._user_auxfn_interface = uafi 
 942          self._protected_auxnames.extend(auxnames)

 943  
 
 944  
 
 945  
 


946 -    def _genSpecFnPy(self, name, specstr, resname, specnames,
 
 947                       docodeinserts=False):


948          # Set up function header
 
 949          retstr = 'def '+name+'(ds, t, x, parsinps):\n' #    print t, x, parsinps\n' 
 950          # add arbitrary code inserts, if present and option is switched on
 
 951          # (only used for vector field definitions)
 
 952          lstart = len(self.codeinserts['start']) 
 953          lend = len(self.codeinserts['end']) 
 954          if docodeinserts: 
 955              if lstart>0: 
 956                  start_code = self._specStrParse(['inserts'],
 
 957                                 {'inserts':self.codeinserts['start']}, '',
 
 958                                  noreturndefs=True, ignoreothers=True,
 
 959                                  doing_inserts=True) 
 960              else: 
 961                  start_code = '' 
 962              if lend > 0: 
 963                  end_code = self._specStrParse(['inserts'],
 
 964                                 {'inserts':self.codeinserts['end']}, '',
 
 965                                  noreturndefs=True, ignoreothers=True,
 
 966                                  doing_inserts=True) 
 967              else: 
 968                  end_code = '' 
 969          else: 
 970              start_code = end_code = '' 
 971          retstr += start_code + specstr + end_code 
 972          # Add the return line to the function
 
 973          if len(specnames) == 1: 
 974              retstr += _indentstr + 'return array([' + resname + '0])\n' 
 975          else: 
 976              retstr += _indentstr + 'return array([' \
 
 977                        + makeParList(range(len(specnames)), resname) + '])\n' 
 978          return retstr

 979  
 
 980  
 


981 -    def _genSpecPy(self):


982          assert self.targetlang == 'python', ('Wrong target language for this'
 
 983                                               ' call') 
 984          assert self.varspecs != {}, 'varspecs attribute must be defined' 
 985          specnames_unsorted = self.varspecs.keys() 
 986          # Process state variable specifications
 
 987          specname_vars = intersect(self.vars, specnames_unsorted) 
 988          specname_vars.sort() 
 989          for vn, vs in self.varspecs.items(): 
 990              if any([pt in vs for pt in ('^', '**')]): 
 991                  self.varspecs[vn] = convertPowers(vs, 'pow') 
 992          self.vars.sort() 
 993          assert self.vars == specname_vars, ('Mismatch between declared '
 
 994                                          ' variable names and varspecs keys') 
 995          reusestr, specupdated = self._processReusedPy(specname_vars,
 
 996                                                        self.varspecs) 
 997          self.varspecs.update(specupdated) 
 998          temp = self._specStrParse(specname_vars, self.varspecs, 'xnew') 
 999          specstr_py = self._genSpecFnPy('_specfn', reusestr+temp, 'xnew',
 
1000                                         specname_vars, docodeinserts=True) 
1001          # Process auxiliary variable specifications
 
1002          specname_auxvars = intersect(self.auxvars, specnames_unsorted) 
1003          specname_auxvars.sort() 
1004          assert self.auxvars == specname_auxvars, \
 
1005                     ('Mismatch between declared auxiliary'
 
1006                      ' variable names and varspecs keys') 
1007          reusestraux, specupdated = self._processReusedPy(specname_auxvars,
 
1008                                                           self.varspecs) 
1009          self.varspecs.update(specupdated) 
1010          tempaux = self._specStrParse(specname_auxvars, self.varspecs, 'auxvals') 
1011          auxspecstr_py = self._genSpecFnPy('_auxspecfn', reusestraux+tempaux,
 
1012                                            'auxvals', specname_auxvars) 
1013          try: 
1014              spec_info = makeUniqueFn(specstr_py) 
1015          except SyntaxError: 
1016              print "Syntax error in specification:\n", specstr_py 
1017              raise 
1018          try: 
1019              auxspec_info = makeUniqueFn(auxspecstr_py) 
1020          except SyntaxError: 
1021              print "Syntax error in auxiliary spec:\n", auxspecstr_py 
1022              raise 
1023          self.spec = spec_info 
1024          self.auxspec = auxspec_info

1025  
 
1026  
 


1027 -    def _processReusedPy(self, specnames, specdict, specials=[],
 
1028                          dovars=True, dopars=True, doinps=True, illegal=[]):


1029          """Process reused subexpression terms for Python code.""" 
1030  
 
1031          reused, specupdated, new_protected, order = _processReused(specnames,
 
1032                                                          specdict,
 
1033                                                          self.reuseterms,
 
1034                                                          _indentstr) 
1035          self._protected_reusenames = new_protected 
1036          # symbols to parse are at indices 2 and 4 of 'reused' dictionary
 
1037          reusedParsed = self._parseReusedTermsPy(reused, [2,4],
 
1038                                          specials=specials, dovars=dovars,
 
1039                                          dopars=dopars, doinps=doinps,
 
1040                                                    illegal=illegal) 
1041          reusedefs = {}.fromkeys(new_protected) 
1042          for vname, deflist in reusedParsed.iteritems(): 
1043              for d in deflist: 
1044                  reusedefs[d[2]] = d 
1045          return (concatStrDict(reusedefs, intersect(order,reusedefs.keys())),
 
1046                         specupdated)

1047  
 
1048  
 


1049 -    def _parseReusedTermsPy(self, d, symbol_ixs, specials=[],
 
1050                          dovars=True, dopars=True, doinps=True, illegal=[]):


1051          """Process dictionary of reused term definitions (in spec syntax).""" 
1052          # ... to parse special symbols to actual Python.
 
1053          # expect symbols to be processed at d list's entries given in
 
1054          # symbol_ixs.
 
1055          allnames = self.vars + self.pars + self.inputs + self.auxvars \
 
1056                     + ['abs'] + self._protected_auxnames \
 
1057                     + self._protected_scipynames + self._protected_specialfns \
 
1058                     + self._protected_macronames + self._protected_mathnames \
 
1059                     + self._protected_randomnames + self._protected_reusenames 
1060          allnames = remain(allnames, illegal) 
1061          if dovars: 
1062              var_arrayixstr = dict(zip(self.vars, map(lambda i: str(i), \
 
1063                                       range(len(self.vars))) )) 
1064              aux_arrayixstr = dict(zip(self.auxvars, map(lambda i: str(i), \
 
1065                                       range(len(self.auxvars))) )) 
1066          else: 
1067              var_arrayixstr = {} 
1068              aux_arrayixstr = {} 
1069          if dopars: 
1070              if doinps: 
1071                  # parsinps_names is pars and inputs, each sorted
 
1072                  # *individually*
 
1073                  parsinps_names = self.pars+self.inputs 
1074              else: 
1075                  parsinps_names = self.pars 
1076              parsinps_arrayixstr = dict(zip(parsinps_names,
 
1077                                          map(lambda i: str(i), \
 
1078                                          range(len(parsinps_names))) )) 
1079          else: 
1080              parsinps_names = [] 
1081              parsinps_arrayixstr = {} 
1082          specialtokens = remain(allnames,specials) + ['(', 't'] + specials 
1083          for specname, itemlist in d.iteritems(): 
1084              listix = -1 
1085              for strlist in itemlist: 
1086                  listix += 1 
1087                  if strlist == []: 
1088                      continue 
1089                  if len(strlist) < max(symbol_ixs): 
1090                      raise ValueError("Symbol indices out of range in "
 
1091                                         "call to _parseReusedTermsPy") 
1092                  for ix in symbol_ixs: 
1093                      symbol = strlist[ix] 
1094                      parsedsymbol = self.__processTokens(allnames,
 
1095                                      specialtokens, symbol,
 
1096                                      var_arrayixstr, aux_arrayixstr,
 
1097                                      parsinps_names, parsinps_arrayixstr,
 
1098                                      specname) 
1099                      # must strip possible trailing whitespace!
 
1100                      d[specname][listix][ix] = parsedsymbol.strip() 
1101          return d

1102  
 
1103  
 


1104 -    def _specStrParse(self, specnames, specdict, resname='', specials=[],
 
1105                          dovars=True, dopars=True, doinps=True,
 
1106                          noreturndefs=False, forexternal=False, illegal=[],
 
1107                          ignoreothers=False, doing_inserts=False):


1108          # use 'noreturndefs' switch if calling this function just to "parse"
 
1109          # a spec string for other purposes, e.g. for using in an event setup
 
1110          # or an individual auxiliary function spec
 
1111          assert isinstance(specnames, list), "specnames must be a list" 
1112          if noreturndefs or forexternal: 
1113              assert len(specnames) == 1, ("can only pass a single specname for "
 
1114                                       "'forexternal' or 'noreturndefs' options") 
1115          allnames = self.vars + self.pars + self.inputs + self.auxvars \
 
1116                     + ['abs', 'and', 'or', 'not', 'True', 'False'] \
 
1117                     + self._protected_auxnames \
 
1118                     + self._protected_scipynames + self._protected_specialfns \
 
1119                     + self._protected_macronames + self._protected_mathnames \
 
1120                     + self._protected_randomnames + self._protected_reusenames 
1121          allnames = remain(allnames, illegal) 
1122          if dovars: 
1123              if forexternal: 
1124                  var_arrayixstr = dict(zip(self.vars,
 
1125                                            ["'"+v+"'" for v in self.vars])) 
1126                  aux_arrayixstr = dict(zip(self.auxvars,
 
1127                                            ["'"+v+"'" for v in self.auxvars])) 
1128              else: 
1129                  var_arrayixstr = dict(zip(self.vars, map(lambda i: str(i), \
 
1130                                           range(len(self.vars))) )) 
1131                  aux_arrayixstr = dict(zip(self.auxvars, map(lambda i: str(i),\
 
1132                                           range(len(self.auxvars))) )) 
1133          else: 
1134              var_arrayixstr = {} 
1135              aux_arrayixstr = {} 
1136          # ODE solvers typically don't recognize external inputs
 
1137          # so they have to be lumped in with the parameters
 
1138          # argument `parsinps` holds the combined pars and inputs
 
1139          if dopars: 
1140              if forexternal: 
1141                  if doinps: 
1142                      # parsinps_names is pars and inputs, each sorted
 
1143                      # *individually*
 
1144                      parsinps_names = self.pars+self.inputs 
1145                  else: 
1146                      parsinps_names = self.pars 
1147                  # for external calls we want parname -> 'parname'
 
1148                  parsinps_arrayixstr = dict(zip(parsinps_names,
 
1149                                         ["'"+pn+"'" for pn in parsinps_names])) 
1150              else: 
1151                  if doinps: 
1152                      # parsinps_names is pars and inputs, each sorted
 
1153                      # *individually*
 
1154                      parsinps_names = self.pars+self.inputs 
1155                  else: 
1156                      parsinps_names = self.pars 
1157                  parsinps_arrayixstr = dict(zip(parsinps_names,
 
1158                                              map(lambda i: str(i), \
 
1159                                              range(len(parsinps_names))) )) 
1160          else: 
1161              parsinps_names = [] 
1162              parsinps_arrayixstr = {} 
1163          specialtokens = remain(allnames,specials) + ['(', 't'] \
 
1164                          + remain(specials,['t']) 
1165          specstr_lang = '' 
1166          specname_count = 0 
1167          for specname in specnames: 
1168              specstr = specdict[specname] 
1169              assert type(specstr)==str, "Specification for %s was not a string"%specname 
1170              if not noreturndefs: 
1171                  specstr_lang += _indentstr + resname+str(specname_count)+' = ' 
1172              specname_count += 1 
1173              specstr_lang += self.__processTokens(allnames, specialtokens,
 
1174                                      specstr, var_arrayixstr,
 
1175                                      aux_arrayixstr, parsinps_names,
 
1176                                      parsinps_arrayixstr, specname, ignoreothers,
 
1177                                      doing_inserts) 
1178              if not noreturndefs or not forexternal: 
1179                  specstr_lang += '\n'  # prepare for next line 
1180          return specstr_lang

1181  
 
1182  
 


1183 -    def __processTokens(self, allnames, specialtokens, specstr,
 
1184                          var_arrayixstr, aux_arrayixstr, parsinps_names,
 
1185                          parsinps_arrayixstr, specname, ignoreothers=False,
 
1186                          doing_inserts=False):


1187          # This function is an earlier version of parseUtils.py's
 
1188          # parse method of a parserObject.
 
1189          # This function should be replaced with an adapted version
 
1190          # of parserObject that can handle auxiliary function call
 
1191          # parsing and the inbuilt macros. This is some of the worst-organized
 
1192          # code I ever wrote, early in my experience with Python. My apologies...
 
1193          returnstr = '' 
1194          if specstr[-1] != ')': 
1195              # temporary hack because strings not ending in ) lose their last
 
1196              # character!
 
1197              specstr += ' ' 
1198          scount = 0 
1199          speclen = len(specstr) 
1200          valid_depnames = self.vars+self.auxvars 
1201          s = '' 
1202          ignore_list = ['', ' ', '\n'] + allnames 
1203          foundtoken = False 
1204          # initial value for special treatment of the 'initcond' built-in
 
1205          # auxiliary function's argument
 
1206          strname_arg_imminent = False 
1207          auxfn_args_imminent = False 
1208          while scount < speclen: 
1209              stemp = specstr[scount] 
1210              scount += 1 
1211              if name_chars_RE.match(stemp) is None: 
1212                  # found a non-alphanumeric char
 
1213                  # so just add to returnstr with accumulated s characters
 
1214                  # (these will have been deleted if s contained a target
 
1215                  # name)
 
1216                  if not ignoreothers and s not in ignore_list: 
1217                      # adding allnames catches var names etc. that are valid
 
1218                      # in auxiliary functions but are not special tokens
 
1219                      # and must be left alone
 
1220                      print "Error in specification `" + specname + \
 
1221                            "` with token `"+s+"` :\n", specstr 
1222                      raise ValueError('Undeclared or illegal token `'+s+'` in'
 
1223                                         ' spec string `'+specname+'`') 
1224                  if stemp == '^' and self.targetlang == 'python': 
1225                      raise ValueError('Character `^` is not allowed. '
 
1226                                         'Please use the pow() call') 
1227                  if stemp == '(': 
1228                      returnstr += s 
1229                      s = stemp 
1230                  else: 
1231                      returnstr += s 
1232                      if len(returnstr)>1 and stemp == returnstr[-1] == "*": 
1233                          # check for ** case
 
1234                          raise ValueError('Operator ** is not allowed. '
 
1235                                     'Please use the pow() call') 
1236                      returnstr += stemp 
1237                      s = '' 
1238                      continue 
1239              else: 
1240                  if s == '' and stemp not in num_chars: 
1241                      s += stemp 
1242                  elif s != '': 
1243                      s += stemp 
1244                  else: 
1245                      returnstr += stemp 
1246                      continue 
1247              if s in specialtokens + self._ignorespecial: 
1248                  if s != '(': 
1249                      if scount < speclen - 1: 
1250                          if name_chars_RE.match(specstr[scount]) is None: 
1251                              foundtoken = True 
1252                          else: 
1253                              if s in ['e','E'] and \
 
1254                                 name_chars_RE.match(specstr[scount]).group() \
 
1255                                         in num_chars+['-']: 
1256                                  # not expecting an arithmetic symbol or space
 
1257                                  # ... we *are* expecting a numeric
 
1258                                  foundtoken = True 
1259                      else: 
1260                          foundtoken = True 
1261                  else: 
1262                      foundtoken = True 
1263                  if foundtoken: 
1264                      if s == '(': 
1265                          if auxfn_args_imminent: 
1266                              returnstr += s+'parsinps, ' 
1267                              auxfn_args_imminent = False 
1268                          else: 
1269                              returnstr += s 
1270                      elif s == 'abs': 
1271                          returnstr += s 
1272                      elif s in var_arrayixstr and \
 
1273                           (len(returnstr)==0 or len(returnstr)>0 and \
 
1274                            returnstr[-1] not in ["'", '"']): 
1275                          if strname_arg_imminent: 
1276                              returnstr += "'"+s+"'" 
1277                              strname_arg_imminent = False 
1278                          else: 
1279                              if specname in valid_depnames \
 
1280                                 and (specname, s) not in self.dependencies: 
1281                                  self.dependencies.append((specname,s)) 
1282                              returnstr += 'x['+var_arrayixstr[s]+']' 
1283                      elif s in aux_arrayixstr: 
1284                          if strname_arg_imminent: 
1285                              returnstr += "'"+s+"'" 
1286                              strname_arg_imminent = False 
1287                          else: 
1288                              print "Spec name:", specname 
1289                              print "Spec string:", specstr 
1290                              print "Problem symbol:", s 
1291                              raise NameError('auxiliary variables cannot '
 
1292                                           'appear on any right-hand side '
 
1293                                           'except their initial value') 
1294                      elif s in parsinps_arrayixstr and \
 
1295                           (len(returnstr)==0 or len(returnstr)>0 and \
 
1296                            returnstr[-1] not in ["'", '"']): 
1297                          if s in self.inputs: 
1298                              if specname in valid_depnames and \
 
1299                                     (specname, s) not in self.dependencies: 
1300                                  self.dependencies.append((specname,s)) 
1301                          if strname_arg_imminent: 
1302                              returnstr += "'"+s+"'" 
1303                              strname_arg_imminent = False 
1304                          else: 
1305                              returnstr += 'parsinps[' + \
 
1306                                        parsinps_arrayixstr[s] + ']' 
1307                      elif s in self._protected_mathnames: 
1308                          if s in ['e','E']: 
1309                              # special case where e is either = exp(0)
 
1310                              # as a constant or it's an exponent in 1e-4
 
1311                              if len(returnstr)>0: 
1312                                  if returnstr[-1] not in num_chars+['.']: 
1313                                      returnstr += 'math.'+s.lower() 
1314                                  else: 
1315                                      returnstr += s 
1316                              else: 
1317                                  returnstr += 'math.'+s.lower() 
1318                          else: 
1319                              returnstr += 'math.'+s 
1320                      elif s in self._protected_randomnames: 
1321                          if len(returnstr) > 0: 
1322                              if returnstr[-1] == '.': 
1323                                  # not a standalone name (e.g. "sample" may be a method call
 
1324                                  # in an embedded system)
 
1325                                  returnstr += s 
1326                              else: 
1327                                  returnstr += 'random.'+s 
1328                          else: 
1329                              returnstr += 'random.'+s 
1330                      elif s in self._protected_scipynames: 
1331                          if len(returnstr) > 0: 
1332                              if returnstr[-1] == '.': 
1333                                  # not a standalone name (e.g. may be a method call in an
 
1334                                  # embedded system)
 
1335                                  returnstr += s 
1336                              else: 
1337                                  returnstr += 'scipy.'+s 
1338                          else: 
1339                              returnstr += 'scipy.'+s 
1340                      elif s in self._protected_specialfns: 
1341                          if self.targetlang != 'python': 
1342                              print "Function %s is currently not supported "%s, \
 
1343                                  "outside of python target language definitions" 
1344                              raise ValueError("Invalid special function for "
 
1345                                               "non-python target definition") 
1346                          # replace the underscore in the name with a dot
 
1347                          # to access scipy.special
 
1348                          returnstr += 'scipy.'+s.replace('_','.') 
1349                      elif s in self._protected_macronames: 
1350                          if doing_inserts: 
1351                              # Code inserts don't use macro versions of "if", "for", etc.
 
1352                              # They are interpreted as regular python
 
1353                              returnstr += s 
1354                          else: 
1355                              if specname in self._pyauxfns: 
1356                                  # remove vars, auxs, inputs
 
1357                                  to_remove = self.vars + self.auxvars + self.inputs 
1358                                  filtfunc = lambda n: n not in to_remove 
1359                                  specialtokens_temp = filter(filtfunc,
 
1360                                                          specialtokens+self._ignorespecial) 
1361                              else: 
1362                                  specialtokens_temp = specialtokens+self._ignorespecial 
1363                              if s == 'if': 
1364                                  # hack for special 'if' case
 
1365                                  # read contents of braces
 
1366                                  endargbrace = findEndBrace(specstr[scount:]) \
 
1367                                                   + scount + 1 
1368                                  argstr = specstr[scount:endargbrace] 
1369                                  procstr = self.__processTokens(allnames,
 
1370                                                  specialtokens_temp, argstr,
 
1371                                                  var_arrayixstr,
 
1372                                                  aux_arrayixstr, parsinps_names,
 
1373                                                  parsinps_arrayixstr, specname) 
1374                                  arginfo = readArgs(procstr) 
1375                                  if not arginfo[0]: 
1376                                      raise ValueError('Error finding '
 
1377                                              'arguments applicable to `if` '
 
1378                                              'macro') 
1379                                  # advance pointer in specstr according to
 
1380                                  # how many tokens/characters were read in for
 
1381                                  # the argument list
 
1382                                  scount += len(argstr) # not arginfo[2] 
1383                                  arglist = arginfo[1] 
1384                                  assert len(arglist) == 3, ('Wrong number of'
 
1385                                                  ' arguments passed to `if`'
 
1386                                                  ' macro. Expected 3') 
1387                                  returnstr += 'ds.' + self._pyauxfns[s][1] + \
 
1388                                               '(parsinps, '+procstr[1:] 
1389                              elif s == 'for': 
1390                                  raise ValueError('Macro '+s+' cannot '
 
1391                                          'be used here') 
1392                              elif s == 'sum': 
1393                                  endargbrace = findEndBrace(specstr[scount:]) \
 
1394                                                   + scount + 1 
1395                                  argstr = specstr[scount:endargbrace] 
1396                                  arginfo = readArgs(argstr) 
1397                                  if not arginfo[0]: 
1398                                      raise ValueError('Error finding '
 
1399                                              'arguments applicable to `sum` '
 
1400                                              'macro') 
1401                                  arglist = arginfo[1] 
1402                                  assert len(arglist) == 4, ('Wrong number of'
 
1403                                                  ' arguments passed to `sum`'
 
1404                                                  ' macro. Expected 4') 
1405                                  # advance pointer in specstr according to
 
1406                                  # how many tokens/characters were read in for
 
1407                                  # the argument list
 
1408                                  scount += len(argstr) 
1409                                  # recursively process main argument
 
1410                                  returnstr += self.__processTokens(allnames,
 
1411                                                  specialtokens_temp,
 
1412                                                  self._macroSum(*arglist), var_arrayixstr,
 
1413                                                  aux_arrayixstr, parsinps_names,
 
1414                                                  parsinps_arrayixstr, specname) 
1415                              else: 
1416                                  # max and min just pass through
 
1417                                  returnstr += s 
1418                      elif s in self._protected_auxnames: 
1419                          if s in ['initcond', 'getbound']: 
1420                              # must prepare parser for upcoming variable
 
1421                              # name in argument that must only be
 
1422                              # converted to its index in x[]
 
1423                              strname_arg_imminent = True 
1424                          # add internal prefix (to avoid method name clashes
 
1425                          # in DS objects, for instance) unless built-in function
 
1426                          returnstr += 'ds.' + self._pyauxfns[s][1] 
1427                          auxfn_args_imminent = True 
1428                      elif s in self._pyauxfns: 
1429                          # treat inter-aux function dependencies:
 
1430                          # any protected auxnames will already have been
 
1431                          # processed because this is placed after that check.
 
1432                          # don't reference self._pyauxfns[s] because it doesn't
 
1433                          # contain the processed definition of the function.
 
1434                          if s in ['initcond', 'getbound']: 
1435                              # must prepare parser for upcoming variable
 
1436                              # name in argument that must only be
 
1437                              # converted to its index in x[]
 
1438                              strname_arg_imminent = True 
1439                          # add internal prefix (to avoid method name clashes
 
1440                          # in DS objects, for instance) unless built-in function
 
1441                          returnstr += 'ds.' + s 
1442                          auxfn_args_imminent = True 
1443                      elif s in self._protected_reusenames: 
1444                          returnstr += s 
1445                      else: 
1446                          # s is e.g. a declared argument to an aux fn but
 
1447                          # only want to ensure it is present. no action to take.
 
1448                          returnstr += s 
1449                      # reset for next iteration
 
1450                      s = '' 
1451                      foundtoken = False 
1452          # end of scount while loop
 
1453          return returnstr

1454  
 
1455  
 
1456      # --------------------- C code specifications -----------------------
 
1457  
 


1458 -    def _processReusedC(self, specnames, specdict):


1459          """Process reused subexpression terms for C code.""" 
1460  
 
1461          if self.auxfns: 
1462              def addParToCall(s): 
1463                  return addArgToCalls(self._processSpecialC(s),
 
1464                                        self.auxfns.keys(), "p_, wk_, xv_")

1465              parseFunc = addParToCall 
1466          else: 
1467              parseFunc = self._processSpecialC 
1468          reused, specupdated, new_protected, order = _processReused(specnames,
 
1469                                                            specdict,
 
1470                                                            self.reuseterms,
 
1471                                                            '', 'double', ';',
 
1472                                                            parseFunc) 
1473          self._protected_reusenames = new_protected 
1474          reusedefs = {}.fromkeys(new_protected) 
1475          for vname, deflist in reused.iteritems(): 
1476              for d in deflist: 
1477                  reusedefs[d[2]] = d 
1478          return (concatStrDict(reusedefs, intersect(order, reusedefs.keys())),
 
1479                         specupdated)

1480  
 
1481  
 


1482 -    def _genAuxFnC(self):


1483          auxnames = self._auxfnspecs.keys() 
1484          # parameter and variable definitions
 
1485          # sorted version of var and par names sorted version of par
 
1486          # names (vars not #define'd in aux functions unless Jacobian)
 
1487          vnames = self.vars 
1488          pnames = self.pars 
1489          vnames.sort() 
1490          pnames.sort() 
1491          for auxname in auxnames: 
1492              assert auxname not in ['auxvars', 'vfieldfunc'], \
 
1493                 ("auxiliary function name '" +auxname+ "' clashes with internal"
 
1494                  " names") 
1495          # must add parameter argument so that we can name
 
1496          # parameters inside the functions! this would either
 
1497          # require all calls to include this argument (yuk!) or
 
1498          # else we add these extra parameters automatically to
 
1499          # every call found in the .c code (as is done currently.
 
1500          # this is still an untidy solution, but there you go...)
 
1501          for auxname in auxnames: 
1502              auxspec = self._auxfnspecs[auxname] 
1503              assert len(auxspec) == 2, 'auxspec tuple must be of length 2' 
1504              if not isinstance(auxspec[0], list): 
1505                  print "Found type ", type(auxspec[0]) 
1506                  print "Containing: ", auxspec[0] 
1507                  raise TypeError('aux function arguments '
 
1508                                  'must be given as a list') 
1509              if not isinstance(auxspec[1], str): 
1510                  print "Found type ", type(auxspec[1]) 
1511                  print "Containing: ", auxspec[1] 
1512                  raise TypeError('aux function specification '
 
1513                                  'must be a string of the function code') 
1514              # Process Jacobian functions specially, if present
 
1515              if auxname == 'Jacobian': 
1516                  sig = "void jacobian(" 
1517                  if not compareList(auxspec[0],['t']+self.vars): 
1518                      print ['t']+self.vars 
1519                      print "Auxspec =", auxspec[0] 
1520                      raise ValueError("Invalid argument list given in Jacobian.") 
1521                  if any([pt in auxspec[1] for pt in ('^', '**')]): 
1522                      auxstr = convertPowers(auxspec[1], 'pow') 
1523                  else: 
1524                      auxstr = auxspec[1] 
1525                  parlist = "unsigned n_, unsigned np_, double t, double *Y_," 
1526                  ismat = True 
1527                  sig += parlist + " double *p_, double **f_, unsigned wkn_, double *wk_, unsigned xvn_, double *xv_)" 
1528                  specvars = self.vars 
1529                  specvars.sort() 
1530                  n = len(specvars) 
1531                  m = n 
1532                  specdict_temp = {}.fromkeys(specvars) 
1533                  if m == 1: 
1534                      assert '[' not in auxstr, \
 
1535                             "'[' character invalid in Jacobian for 1D system" 
1536                      assert ']' not in auxstr, \
 
1537                             "']' character invalid in Jacobian for 1D system" 
1538                      specdict_temp[specvars[0]] = auxstr 
1539                  else: 
1540                      specdict_temp = parseMatrixStrToDictStr(auxstr, specvars) 
1541                  reusestr, body_processed_dict = self._processReusedC(specvars,
 
1542                                                     specdict_temp) 
1543                  specdict = {}.fromkeys(specvars) 
1544                  for specname in specvars: 
1545                      temp = body_processed_dict[specname] 
1546                      specdict[specname] = splitargs(temp.replace("[","").replace("]","")) 
1547                  body_processed = "" 
1548                  # C integrators expect column-major matrices
 
1549                  for col in range(n): 
1550                      for row in range(m): 
1551                          try: 
1552                              body_processed += "f_[" + str(col) + "][" + str(row) \
 
1553                              + "] = " + specdict[specvars[row]][col] + ";\n" 
1554                          except IndexError: 
1555                              raise ValueError("Jacobian should be %sx%s"%(m,n)) 
1556                  body_processed += "\n" 
1557                  auxspec_processedDict = {auxname: body_processed} 
1558              elif auxname == 'Jacobian_pars': 
1559                  sig = "void jacobianParam(" 
1560                  if not compareList(auxspec[0],['t']+self.vars): 
1561                      print ['t']+self.vars 
1562                      print "Auxspec =", auxspec[0] 
1563                      raise ValueError("Invalid argument list given in Jacobian.") 
1564                  parlist = "unsigned n_, unsigned np_, double t, double *Y_," 
1565                  if any([pt in auxspec[1] for pt in ('^', '**')]): 
1566                      auxstr = convertPowers(auxspec[1], 'pow') 
1567                  else: 
1568                      auxstr = auxspec[1] 
1569                  ismat = True 
1570  #                specials = ["t","Y_","n_","np_","wkn_","wk_"]
 
1571                  sig += parlist + " double *p_, double **f_, unsigned wkn_, double *wk_, unsigned xvn_, double *xv_)" 
1572                  specvars = self.vars 
1573                  specvars.sort() 
1574                  n = len(specvars) 
1575                  if n == 0: 
1576                      raise ValueError("Cannot have a Jacobian w.r.t. pars"
 
1577                                       " because no pars are defined") 
1578                  m = len(self.vars) 
1579                  specdict_temp = {}.fromkeys(self.vars) 
1580                  if m == n == 1: 
1581                      assert '[' not in auxstr, \
 
1582                             "'[' character invalid in Jacobian for 1D system" 
1583                      assert ']' not in auxstr, \
 
1584                             "']' character invalid in Jacobian for 1D system" 
1585                      specdict_temp[self.vars.values()[0]] = auxstr 
1586                  else: 
1587                      specdict_temp = parseMatrixStrToDictStr(auxstr, self.vars, m) 
1588                  reusestr, body_processed_dict = self._processReusedC(self.vars,
 
1589                                                     specdict_temp) 
1590                  specdict = {}.fromkeys(self.vars) 
1591                  for specname in self.vars: 
1592                      temp = body_processed_dict[specname] 
1593                      specdict[specname] = splitargs(temp.replace("[","").replace("]","")) 
1594                  body_processed = "" 
1595                  # C integrators expect column-major matrices
 
1596                  for col in range(n): 
1597                      for row in range(m): 
1598                          try: 
1599                              body_processed += "f_[" + str(col) + "][" + str(row) \
 
1600                              + "] = " + specdict[self.vars[row]][col] + ";\n" 
1601                          except (IndexError, KeyError): 
1602                              print "\nFound matrix:\n" 
1603                              info(specdict) 
1604                              raise ValueError("Jacobian should be %sx%s"%(m,n)) 
1605                  body_processed += "\n" 
1606                  auxspec_processedDict = {auxname: body_processed} 
1607              elif auxname == 'massMatrix': 
1608                  sig = "void massMatrix(" 
1609                  if not compareList(auxspec[0],['t']+self.vars): 
1610                      raise ValueError("Invalid argument list given in Mass Matrix.") 
1611                  if any([pt in auxspec[1] for pt in ('^', '**')]): 
1612                      auxstr = convertPowers(auxspec[1], 'pow') 
1613                  else: 
1614                      auxstr = auxspec[1] 
1615                  parlist = "unsigned n_, unsigned np_," 
1616                  ismat = True 
1617  #                specials = ["n_","np_","wkn_","wk_"]
 
1618                  sig += parlist + " double t, double *Y_, double *p_, double **f_, unsigned wkn_, double *wk_, unsigned xvn_, double *xv_)" 
1619                  specvars = self.vars 
1620                  specvars.sort() 
1621                  n = len(specvars) 
1622                  m = n 
1623                  specdict_temp = {}.fromkeys(specvars) 
1624                  if m == 1: 
1625                      assert '[' not in auxstr, \
 
1626                             "'[' character invalid in mass matrix for 1D system" 
1627                      assert ']' not in auxstr, \
 
1628                             "']' character invalid in mass matrix for 1D system" 
1629                      specdict_temp[specvars.values()[0]] = auxstr 
1630                  else: 
1631                      specdict_temp = parseMatrixStrToDictStr(auxstr, specvars, m) 
1632                  reusestr, body_processed_dict = self._processReusedC(specvars,
 
1633                                                     specdict_temp) 
1634                  specdict = {}.fromkeys(specvars) 
1635                  for specname in specvars: 
1636                      temp = body_processed_dict[specname].replace("[","").replace("]","") 
1637                      specdict[specname] = splitargs(temp) 
1638                  body_processed = "" 
1639                  # C integrators expect column-major matrices
 
1640                  for col in range(n): 
1641                      for row in range(m): 
1642                          try: 
1643                              body_processed += "f_[" + str(col) + "][" + str(row) \
 
1644                              + "] = " + specdict[specvars[row]][col] + ";\n" 
1645                          except KeyError: 
1646                              raise ValueError("Mass matrix should be %sx%s"%(m,n)) 
1647                  body_processed += "\n" 
1648                  auxspec_processedDict = {auxname: body_processed} 
1649              else: 
1650                  ismat = False 
1651                  sig = "double " + auxname + "(" 
1652                  parlist = "" 
1653                  namemap = {} 
1654                  for parname in auxspec[0]: 
1655                      if parname == '': 
1656                          continue 
1657                      parlist += "double " + "__" + parname + "__, " 
1658                      namemap[parname] = '__'+parname+'__' 
1659                  sig += parlist + "double *p_, double *wk_, double *xv_)" 
1660                  auxstr = auxspec[1] 
1661                  if any([pt in auxspec[1] for pt in ('^', '**')]): 
1662                      auxstr = convertPowers(auxstr, 'pow') 
1663                  prep_auxstr = self._processSpecialC(auxstr) 
1664                  prep_auxstr_quant = QuantSpec('prep_q',
 
1665                                    prep_auxstr.replace(' ','').replace('\n',''),
 
1666                                    treatMultiRefs=False, preserveSpace=True) 
1667                  # have to do name map now in case function's formal arguments
 
1668                  # coincide with state variable names, which may get tied up
 
1669                  # in reused terms and not properly matched to the formal args.
 
1670                  prep_auxstr_quant.mapNames(namemap) 
1671                  auxspec = (auxspec[0], prep_auxstr_quant()) 
1672                  reusestr, auxspec_processedDict = self._processReusedC([auxname],
 
1673                                                       {auxname:auxspec[1]}) 
1674                  # addition of parameter done in Generator code
 
1675  #                dummyQ = QuantSpec('dummy', auxspec_processedDict[auxname])
 
1676  #                auxspec_processed = ""
 
1677  #                # add pars argument to inter-aux fn call
 
1678  #                auxfn_found = False   # then expect a left brace next
 
1679  #                for tok in dummyQ:
 
1680  #                    if auxfn_found:
 
1681  #                        # expect left brace in this tok
 
1682  #                        if tok == '(':
 
1683  #                            auxspec_processed += tok + 'p_, '
 
1684  #                            auxfn_found = False
 
1685  #                        else:
 
1686  #                            raise ValueError("Problem parsing inter-auxiliary"
 
1687  #                                             " function call")
 
1688  #                    elif tok in self.auxfns and tok not in \
 
1689  #                            ['Jacobian', 'Jacobian_pars']:
 
1690  #                        auxfn_found = True
 
1691  #                        auxspec_processed += tok
 
1692  #                    else:
 
1693  #                        auxspec_processed += tok
 
1694  #                body_processed = "return "+auxspec_processed + ";\n\n"
 
1695              # add underscore to local names, to avoid clash with global
 
1696              # '#define' names
 
1697              dummyQ = QuantSpec('dummy', auxspec_processedDict[auxname],
 
1698                                 treatMultiRefs=False, preserveSpace=True) 
1699              body_processed = "return "*(not ismat) + dummyQ() + ";\n\n" 
1700  #            auxspecstr = sig + " {\n\n" + pardefines + vardefines*ismat \
 
1701              auxspecstr = sig + " {\n\n" \
 
1702                  + "\n" + (len(reusestr)>0)*"/* reused term definitions */\n" \
 
1703                  + reusestr + (len(reusestr)>0)*"\n" + body_processed \
 
1704                  + "}" 
1705  #                + parundefines + varundefines*ismat + "}"
 
1706              # sig as second entry, whereas Python-coded specifications
 
1707              # have the fn name there
 
1708              self.auxfns[auxname] = (auxspecstr, sig) 
1709          # Don't apply #define's for built-in functions
 
1710          self.auxfns['heav'] = ("int heav(double x_, double *p_, double *wk_, double *xv_) {\n" \
 
1711                               + "  if (x_>0.0) {return 1;} else {return 0;}\n}",
 
1712                    "int heav(double x_, double *p_, double *wk_, double *xv_)") 
1713          self.auxfns['__rhs_if'] = ("double __rhs_if(int cond_, double e1_, " \
 
1714                          + "double e2_, double *p_, double *wk_, double *xv_) {\n" \
 
1715                          + "  if (cond_) {return e1_;} else {return e2_;};\n}",
 
1716                "double __rhs_if(int cond_, double e1_, double e2_, double *p_, double *wk_, double *xv_)") 
1717          self.auxfns['__maxof2'] = ("double __maxof2(double e1_, double e2_, double *p_, double *wk_, double *xv_) {\n" \
 
1718                                  + "if (e1_ > e2_) {return e1_;} else {return e2_;};\n}",
 
1719                  "double __maxof2(double e1_, double e2_, double *p_, double *wk_, double *xv_)") 
1720          self.auxfns['__minof2'] = ("double __minof2(double e1_, double e2_, double *p_, double *wk_, double *xv_) {\n" \
 
1721                                  + "if (e1_ < e2_) {return e1_;} else {return e2_;};\n}",
 
1722                  "double __minof2(double e1_, double e2_, double *p_, double *wk_, double *xv_)") 
1723          self.auxfns['__maxof3'] = ("double __maxof3(double e1_, double e2_, double e3_, double *p_, double *wk_, double *xv_) {\n" \
 
1724                                 + "double temp_;\nif (e1_ > e2_) {temp_ = e1_;} else {temp_ = e2_;};\n" \
 
1725                                 + "if (e3_ > temp_) {return e3_;} else {return temp_;};\n}",
 
1726                  "double __maxof3(double e1_, double e2_, double e3_, double *p_, double *wk_, double *xv_)") 
1727          self.auxfns['__minof3'] = ("double __minof3(double e1_, double e2_, double e3_, double *p_, double *wk_, double *xv_) {\n" \
 
1728                                 + "double temp_;\nif (e1_ < e2_) {temp_ = e1_;} else {temp_ = e2_;};\n" \
 
1729                                 + "if (e3_ < temp_) {return e3_;} else {return temp_;};\n}",
 
1730                  "double __minof3(double e1_, double e2_, double e3_, double *p_, double *wk_, double *xv_)") 
1731          self.auxfns['__maxof4'] = ("double __maxof4(double e1_, double e2_, double e3_, double e4_, double *p_, double *wk_, double *xv_) {\n" \
 
1732                                 + "double temp_;\nif (e1_ > e2_) {temp_ = e1_;} else {temp_ = e2_;};\n" \
 
1733                                 + "if (e3_ > temp_) {temp_ = e3_;};\nif (e4_ > temp_) {return e4_;} else {return temp_;};\n}",
 
1734                  "double __maxof4(double e1_, double e2_, double e3_, double e4_, double *p_, double *wk_, double *xv_)") 
1735          self.auxfns['__minof4'] = ("double __minof4(double e1_, double e2_, double e3_, double e4_, double *p_, double *wk_, double *xv_) {\n" \
 
1736                                 + "double temp_;\nif (e1_ < e2_) {temp_ = e1_;} else {temp_ = e2_;};\n" \
 
1737                                 + "if (e3_ < temp_) {temp_ = e3_;};\nif (e4_ < temp_) {return e4_;} else {return temp_;};\n}",
 
1738                  "double __minof4(double e1_, double e2_, double e3_, double e4_, double *p_, double *wk_, double *xv_)") 
1739          # temporary placeholders for these built-ins...
 
1740          cases_ic = "" 
1741          cases_index = "" 
1742          for i in xrange(len(self.vars)): 
1743              if i == 0: 
1744                  command = 'if' 
1745              else: 
1746                  command = 'else if' 
1747              vname = self.vars[i] 
1748              cases_ic += "  " + command + " (strcmp(varname, " + '"' + vname + '"'\
 
1749                       + ")==0)\n\treturn gICs[" + str(i) + "];\n" 
1750              cases_index += "  " + command + " (strcmp(name, " + '"' + vname + '"'\
 
1751                       + ")==0)\n\treturn " + str(i) + ";\n" 
1752          # add remaining par names for getindex
 
1753          for i in xrange(len(self.pars)): 
1754              pname = self.pars[i] 
1755              cases_index += "  else if" + " (strcmp(name, " + '"' + pname + '"'\
 
1756                             +")==0)\n\treturn " + str(i+len(self.vars)) + ";\n" 
1757          cases_ic += """  else {\n\tfprintf(stderr, "Invalid variable name %s for """ \
 
1758                   + """initcond call\\n", varname);\n\treturn 0.0/0.0;\n\t}\n""" 
1759          cases_index += """  else {\n\tfprintf(stderr, "Invalid name %s for """ \
 
1760                   + """getindex call\\n", name);\n\treturn 0.0/0.0;\n\t}\n""" 
1761          self.auxfns['initcond'] = ("double initcond(char *varname, double *p_, double *wk_, double *xv_) {\n" \
 
1762                                     + "\n" + cases_ic + "}",
 
1763                                     'double initcond(char *varname, double *p_, double *wk_, double *xv_)') 
1764          self.auxfns['getindex'] = ("int getindex(char *name, double *p_, double *wk_, double *xv_) {\n" \
 
1765                                     + "\n" + cases_index + "}",
 
1766                                     'int getindex(char *name, double *p_, double *wk_, double *xv_)') 
1767          self.auxfns['globalindepvar'] = ("double globalindepvar(double t, double *p_, double *wk_, double *xv_)" \
 
1768                                            + " {\n  return globalt0+t;\n}",
 
1769                                           'double globalindepvar(double t, double *p_, double *wk_, double *xv_)') 
1770          self.auxfns['getbound'] = \
 
1771                      ("double getbound(char *name, int which_bd, double *p_, double *wk_, double *xv_) {\n" \
 
1772                       + "  return gBds[which_bd][getindex(name)];\n}",
 
1773                   'double getbound(char *name, int which_bd, double *p_, double *wk_, double *xv_)')

1774  
 
1775  
 


1776 -    def _genSpecC(self):


1777          assert self.targetlang == 'c', ('Wrong target language for this'
 
1778                                               ' call') 
1779          assert self.varspecs != {}, 'varspecs attribute must be defined' 
1780          specnames_unsorted = self.varspecs.keys() 
1781          specname_vars = intersect(self.vars, specnames_unsorted) 
1782          specname_vars.sort() 
1783          # sorted version of var and par names
 
1784          vnames = specname_vars 
1785          pnames = self.pars 
1786          inames = self.inputs 
1787          pnames.sort() 
1788          inames.sort() 
1789          pardefines = "" 
1790          vardefines = "" 
1791          inpdefines = "" 
1792          parundefines = "" 
1793          varundefines = "" 
1794          inpundefines = "" 
1795          # produce vector field specification
 
1796          assert self.vars == specname_vars, ('Mismatch between declared '
 
1797                                          ' variable names and varspecs keys') 
1798          valid_depTargNames = self.inputs+self.vars+self.auxvars 
1799          for specname, specstr in self.varspecs.iteritems(): 
1800              assert type(specstr)==str, "Specification for %s was not a string"%specname 
1801              if any([pt in specstr for pt in ('^', '**')]): 
1802                  specstr = convertPowers(specstr, 'pow') 
1803              specQS = QuantSpec('__spectemp__',  specstr) 
1804              for s in specQS: 
1805                  if s in valid_depTargNames and (specname, s) not in \
 
1806                         self.dependencies: # and specname != s: 
1807                      self.dependencies.append((specname, s)) 
1808          # pre-process reused sub-expression dictionary to adapt for
 
1809          # known calling sequence in C
 
1810          reusestr, specupdated = self._processReusedC(specname_vars,
 
1811                                                       self.varspecs) 
1812          self.varspecs.update(specupdated) 
1813          specstr_C = self._genSpecFnC('vfieldfunc', reusestr, specname_vars,
 
1814                                         pardefines, vardefines, inpdefines,
 
1815                                         parundefines, varundefines, inpundefines,
 
1816                                         True) 
1817          self.spec = specstr_C 
1818          # produce auxiliary variables specification
 
1819          specname_auxvars = intersect(self.auxvars, specnames_unsorted) 
1820          specname_auxvars.sort() 
1821          assert self.auxvars == specname_auxvars, \
 
1822                     ('Mismatch between declared auxiliary'
 
1823                      ' variable names and varspecs keys') 
1824          if self.auxvars != []: 
1825              reusestraux, specupdated = self._processReusedC(specname_auxvars,
 
1826                                                          self.varspecs) 
1827              self.varspecs.update(specupdated) 
1828          if self.auxvars == []: 
1829              auxspecstr_C = self._genSpecFnC('auxvars', '',
 
1830                                          specname_auxvars,
 
1831                                          '', '', '',
 
1832                                          '', '', '', False) 
1833          else: 
1834              auxspecstr_C = self._genSpecFnC('auxvars', reusestraux,
 
1835                                          specname_auxvars, pardefines,
 
1836                                          vardefines, inpdefines, parundefines,
 
1837                                          varundefines, inpundefines,
 
1838                                          False) 
1839          self.auxspec = auxspecstr_C

1840  
 
1841  
 


1842 -    def _genSpecFnC(self, funcname, reusestr, specnames, pardefines,
 
1843                      vardefines, inpdefines, parundefines, varundefines,
 
1844                      inpundefines, docodeinserts):


1845          sig = "void " + funcname + "(unsigned n_, unsigned np_, double t, double *Y_, " \
 
1846                + "double *p_, double *f_, unsigned wkn_, double *wk_, unsigned xvn_, double *xv_)" 
1847  #        specstr = sig + "{\n\n" + pardefines + vardefines + "\n"
 
1848          specstr = sig + "{" + pardefines + vardefines + inpundefines + "\n" 
1849          if docodeinserts and self.codeinserts['start'] != '': 
1850              specstr += '/* Verbose code insert -- begin */\n' \
 
1851                              + self.codeinserts['start'] \
 
1852                              + '/* Verbose code insert -- end */\n\n' 
1853          specstr += (len(reusestr)>0)*"/* reused term definitions */\n" \
 
1854                     + reusestr + "\n" 
1855          auxdefs_parsed = {} 
1856          # add function body
 
1857          for i in xrange(len(specnames)): 
1858              xname = specnames[i] 
1859              fbody = self.varspecs[xname] 
1860              fbody_parsed = self._processSpecialC(fbody) 
1861              if self.auxfns: 
1862                  fbody_parsed = addArgToCalls(fbody_parsed,
 
1863                                              self.auxfns.keys(),
 
1864                                              "p_, wk_, xv_") 
1865                  if 'initcond' in self.auxfns: 
1866                      # convert 'initcond(x)' to 'initcond("x")' for
 
1867                      # compatibility with C syntax
 
1868                      fbody_parsed = wrapArgInCall(fbody_parsed,
 
1869                                      'initcond', '"') 
1870              specstr += "f_[" + str(i) + "] = " + fbody_parsed + ";\n" 
1871              auxdefs_parsed[xname] = fbody_parsed 
1872          if docodeinserts and self.codeinserts['end'] != '': 
1873              specstr += '\n/* Verbose code insert -- begin */\n' \
 
1874                      + self.codeinserts['end'] \
 
1875                      + '/* Verbose code insert -- end */\n' 
1876          specstr += "\n" + parundefines + varundefines + inpundefines + "}\n\n" 
1877          self._auxdefs_parsed = auxdefs_parsed 
1878          return (specstr, funcname)

1879  
 
1880  
 


1881 -    def _doPreMacrosC(self):


1882          # Pre-processor macros are presently not available for C-code
 
1883          # specifications
 
1884          pass

1885  
 
1886  
 


1887 -    def _processSpecialC(self, specStr):


1888          """Pre-process 'if' statements and names of 'abs' and 'sign' functions,
 
1889          as well as logical operators.
 
1890          """ 
1891          qspec = QuantSpec('spec', specStr, treatMultiRefs=False) 
1892          qspec.mapNames({'abs': 'fabs', 'sign': 'signum', 'mod': 'fmod',
 
1893                          'and': '&&', 'or': '||', 'not': '!',
 
1894                          'True': 1, 'False': 0,
 
1895                          'max': '__maxof', 'min': '__minof'}) 
1896          qtoks = qspec.parser.tokenized 
1897          # default value
 
1898          new_specStr = str(qspec) 
1899          if 'if' in qtoks: 
1900              new_specStr = "" 
1901              num_ifs = qtoks.count('if') 
1902              if_ix = -1 
1903              ix_continue = 0 
1904              for ifstmt in range(num_ifs): 
1905                  if_ix = qtoks[if_ix+1:].index('if')+if_ix+1 
1906                  new_specStr += "".join(qtoks[ix_continue:if_ix]) + "__rhs_if(" 
1907                  rbrace_ix = findEndBrace(qtoks[if_ix+1:])+if_ix+1 
1908                  ix_continue = rbrace_ix+1 
1909                  new_specStr += "".join(qtoks[if_ix+2:ix_continue]) 
1910              new_specStr += "".join(qtoks[ix_continue:]) 
1911              qspec = QuantSpec('spec', new_specStr) 
1912              qtoks = qspec.parser.tokenized 
1913          if '__minof' in qtoks: 
1914              new_specStr = "" 
1915              num = qtoks.count('__minof') 
1916              n_ix = -1 
1917              ix_continue = 0 
1918              for stmt in range(num): 
1919                  n_ix = qtoks[n_ix+1:].index('__minof')+n_ix+1 
1920                  new_specStr += "".join(qtoks[ix_continue:n_ix]) 
1921                  rbrace_ix = findEndBrace(qtoks[n_ix+1:])+n_ix+1 
1922                  ix_continue = rbrace_ix+1 
1923                  #assert qtoks[n_ix+2] == '[', "Error in min() syntax"
 
1924                  #assert qtoks[rbrace_ix-1] == ']', "Error in min() syntax"
 
1925                  #new_specStr += "".join(qtoks[n_ix+3:rbrace_ix-1]) + ")"
 
1926                  num_args = qtoks[n_ix+2:ix_continue].count(',') + 1 
1927                  if num_args > 4: 
1928                      raise NotImplementedError("Max of more than 4 arguments not currently supported in C") 
1929                  new_specStr += '__minof%s(' % str(num_args) 
1930                  new_specStr += "".join([q for q in qtoks[n_ix+2:ix_continue] if q not in ('[',']')]) 
1931              new_specStr += "".join(qtoks[ix_continue:]) 
1932              qspec = QuantSpec('spec', new_specStr) 
1933              qtoks = qspec.parser.tokenized 
1934          if '__maxof' in qtoks: 
1935              new_specStr = "" 
1936              num = qtoks.count('__maxof') 
1937              n_ix = -1 
1938              ix_continue = 0 
1939              for stmt in range(num): 
1940                  n_ix = qtoks[n_ix+1:].index('__maxof')+n_ix+1 
1941                  new_specStr += "".join(qtoks[ix_continue:n_ix]) 
1942                  rbrace_ix = findEndBrace(qtoks[n_ix+1:])+n_ix+1 
1943                  ix_continue = rbrace_ix+1 
1944                  #assert qtoks[n_ix+2] == '[', "Error in max() syntax"
 
1945                  #assert qtoks[rbrace_ix-1] == ']', "Error in max() syntax"
 
1946                  #new_specStr += "".join(qtoks[n_ix+3:rbrace_ix-1]) + ")"
 
1947                  num_args = qtoks[n_ix+2:ix_continue].count(',') + 1 
1948                  if num_args > 4: 
1949                      raise NotImplementedError("Min of more than 4 arguments not currently supported in C") 
1950                  new_specStr += '__maxof%s(' % str(num_args) 
1951                  new_specStr += "".join([q for q in qtoks[n_ix+2:ix_continue] if q not in ('[',']')]) 
1952              new_specStr += "".join(qtoks[ix_continue:]) 
1953              qspec = QuantSpec('spec', new_specStr) 
1954              qtoks = qspec.parser.tokenized 
1955          return new_specStr

1956  
 
1957      # ------------ Matlab code specifications -----------------------
 
1958  
 


1959 -    def _genAuxFnMatlab(self):


1960          auxnames = self.auxfns.keys() 
1961          # parameter and variable definitions
 
1962  
 
1963          # sorted version of var and par names sorted version of par
 
1964          # names (vars not #define'd in aux functions unless Jacobian)
 
1965          vnames = self.vars 
1966          pnames = self.pars 
1967          vnames.sort() 
1968          pnames.sort() 
1969  
 
1970          for auxname in auxnames: 
1971              assert auxname not in ['auxvars', 'vfield'], \
 
1972                 ("auxiliary function name '" +auxname+ "' clashes with internal"
 
1973                  " names") 
1974          # must add parameter argument so that we can name
 
1975          # pars inside the functions! this would either
 
1976          # require all calls to include this argument (yuk!) or
 
1977          # else we add these extra pars automatically to
 
1978          # every call found in the .c code (as is done currently.
 
1979          # this is still an untidy solution, but there you go...)
 
1980          for auxname, auxspec in self._auxfnspecs.iteritems(): 
1981              assert len(auxspec) == 2, 'auxspec tuple must be of length 2' 
1982              if not isinstance(auxspec[0], list): 
1983                  print "Found type ", type(auxspec[0]) 
1984                  print "Containing: ", auxspec[0] 
1985                  raise TypeError('aux function arguments '
 
1986                                  'must be given as a list') 
1987              if not isinstance(auxspec[1], str): 
1988                  print "Found type ", type(auxspec[1]) 
1989                  print "Containing: ", auxspec[1] 
1990                  raise TypeError('aux function specification '
 
1991                                  'must be a string of the function code') 
1992  ##            assert auxspec[1].find('^') == -1, ('carat character ^ is not '
 
1993  ##                                            'permitted in function definitions'
 
1994  ##                                            '-- use pow(x,p) syntax instead')
 
1995              # Process Jacobian functions specially, if present
 
1996              if auxname == 'Jacobian': 
1997                  raise NotImplementedError 
1998              elif auxname == 'Jacobian_pars': 
1999                  raise NotImplementedError 
2000              elif auxname == 'massMatrix': 
2001                  raise NotImplementedError 
2002              else: 
2003                  ismat = False 
2004                  topstr = "function y_ = " + auxname + "(" 
2005                  commentstr = "% Auxilliary function " + auxname + " for model " + self.name + "\n% Generated by PyDSTool for ADMC++ target\n\n" 
2006                  parlist = "" 
2007                  namemap = {} 
2008                  for parname in auxspec[0]: 
2009                      parlist += parname + "__, " 
2010                      namemap[parname] = parname+'__' 
2011                  topstr += parlist + " p_)\n" 
2012                  sig = topstr + commentstr 
2013                  pardefines = self._prepareMatlabPDefines(pnames) 
2014                  auxstr = auxspec[1] 
2015                  if any([pt in auxstr for pt in ('pow', '**')]): 
2016                      auxstr = convertPowers(auxstr, '^') 
2017                  reusestr, auxspec_processedDict = self._processReusedMatlab([auxname],
 
2018                          {auxname:auxstr.replace(' ','').replace('\n','')}) 
2019                  # addition of parameter done in Generator code
 
2020  
 
2021              dummyQ = QuantSpec('dummy', auxspec_processedDict[auxname],
 
2022                                 treatMultiRefs=False, preserveSpace=True) 
2023              if not ismat: 
2024                  dummyQ.mapNames(namemap) 
2025              body_processed = "y_ = "*(not ismat) + dummyQ() + ";\n\n" 
2026  #            auxspecstr = sig + " {\n\n" + pardefines + vardefines*ismat \
 
2027              auxspecstr = sig + pardefines + " \n\n" \
 
2028                  + "\n" + (len(reusestr)>0)*"% reused term definitions \n" \
 
2029                  + reusestr + (len(reusestr)>0)*"\n" + body_processed 
2030              # sig as second entry, whereas Python-coded specifications
 
2031              # have the fn name there
 
2032              self.auxfns[auxname] = (auxspecstr, sig) 
2033          self._protected_auxnames.extend(auxnames)

2034          # Don't apply #define's for built-in functions
 
2035  
 
2036  
 


2037 -    def _genSpecMatlab(self):


2038          assert self.targetlang == 'matlab', ('Wrong target language for this'
 
2039                                               ' call') 
2040          assert self.varspecs != {}, 'varspecs attribute must be defined' 
2041          specnames_unsorted = self.varspecs.keys() 
2042          specname_vars = intersect(self.vars, specnames_unsorted) 
2043          specname_vars.sort() 
2044          # parameter and variable definitions
 
2045          # sorted version of var and par names
 
2046          vnames = specname_vars 
2047          pnames = self.pars 
2048          pnames.sort() 
2049          pardefines = self._prepareMatlabPDefines(pnames) 
2050          vardefines = self._prepareMatlabVDefines(vnames) 
2051          # produce vector field specification
 
2052          assert self.vars == specname_vars, ('Mismatch between declared '
 
2053                                          ' variable names and varspecs keys') 
2054          valid_depTargNames = self.inputs+self.vars+self.auxvars 
2055          for specname, specstr in self.varspecs.iteritems(): 
2056              assert type(specstr)==str, "Specification for %s was not a string"%specname 
2057              if any([pt in specstr for pt in ('pow', '**')]): 
2058                  specstr = convertPowers(specstr, '^') 
2059              specQS = QuantSpec('__spectemp__',  specstr) 
2060              for s in specQS: 
2061                  if s in valid_depTargNames and (specname, s) not in \
 
2062                         self.dependencies: # and specname != s: 
2063                      self.dependencies.append((specname, s)) 
2064          # pre-process reused sub-expression dictionary to adapt for
 
2065          # known calling sequence in Matlab
 
2066          reusestr, specupdated = self._processReusedMatlab(specname_vars,
 
2067                                                       self.varspecs) 
2068          self.varspecs.update(specupdated) 
2069          specstr_Matlab = self._genSpecFnMatlab('vfield', reusestr, specname_vars,
 
2070                                            pardefines, vardefines, True) 
2071          self.spec = specstr_Matlab

2072          # do not produce auxiliary variables specification
 
2073  
 
2074  
 


2075 -    def _genSpecFnMatlab(self, funcname, reusestr, specnames, pardefines,
 
2076                           vardefines, docodeinserts):


2077          topstr = "function [vf_, y_] = " + funcname + "(vf_, t_, x_, p_)\n" 
2078          commentstr = "% Vector field definition for model " + self.name + "\n% Generated by PyDSTool for ADMC++ target\n\n" 
2079  
 
2080          specstr = topstr + commentstr + pardefines + vardefines + "\n" 
2081          if docodeinserts and self.codeinserts['start'] != '': 
2082              specstr += '% Verbose code insert -- begin \n' \
 
2083                              + self.codeinserts['start'] \
 
2084                              + '% Verbose code insert -- end \n\n' 
2085          specstr += (len(reusestr)>0)*"% reused term definitions \n" \
 
2086                     + reusestr + "\n" 
2087          # add function body
 
2088          for i in xrange(len(specnames)): 
2089              xname = specnames[i] 
2090              fbody = self.varspecs[xname] 
2091              fbody_parsed = self._processIfMatlab(fbody) 
2092              if self.auxfns: 
2093                  fbody_parsed = addArgToCalls(fbody_parsed,
 
2094                                              self.auxfns.keys(),
 
2095                                              "p_") 
2096                 # if 'initcond' in self.auxfns:
 
2097                      # convert 'initcond(x)' to 'initcond("x")' for
 
2098                      # compatibility with C syntax
 
2099                  #    fbody_parsed = wrapArgInCall(fbody_parsed,
 
2100                   #                   'initcond', '"')
 
2101              specstr += "y_(" + str(i+1) + ") = " + fbody_parsed + ";\n" 
2102          if docodeinserts and self.codeinserts['end'] != '': 
2103              specstr += '\n% Verbose code insert -- begin \n' \
 
2104                      + self.codeinserts['end'] \
 
2105                      + '% Verbose code insert -- end \n' 
2106          specstr += "\n\n" 
2107          return (specstr, funcname)

2108  
 
2109  
 


2110 -    def _processReusedMatlab(self, specnames, specdict):


2111          """Process reused subexpression terms for Matlab code.""" 
2112  
 
2113          if self.auxfns: 
2114              def addParToCall(s): 
2115                  return addArgToCalls(s, self.auxfns.keys(), "p_")

2116              parseFunc = addParToCall 
2117          else: 
2118              parseFunc = idfn 
2119          reused, specupdated, new_protected, order = _processReused(specnames,
 
2120                                                            specdict,
 
2121                                                            self.reuseterms,
 
2122                                                            '', '', ';',
 
2123                                                            parseFunc) 
2124          self._protected_reusenames = new_protected 
2125          reusedefs = {}.fromkeys(new_protected) 
2126          for vname, deflist in reused.iteritems(): 
2127              for d in deflist: 
2128                  reusedefs[d[2]] = d 
2129          return (concatStrDict(reusedefs, intersect(order, reusedefs.keys())),
 
2130                         specupdated) 
2131      # NEED TO CHECK WHETHER THIS IS NECESSARY AND WORKS
 
2132      # IF STATEMENTS LOOK DIFFERENT IN MATLAB
 


2133 -    def _processIfMatlab(self, specStr):


2134          qspec = QuantSpec('spec', specStr) 
2135          qtoks = qspec[:] 
2136          if 'if' in qtoks: 
2137              raise NotImplementedError 
2138          else: 
2139              new_specStr = specStr 
2140          return new_specStr

2141  
 
2142  
 


2143 -    def _prepareMatlabPDefines(self, pnames):


2144          pardefines = "" 
2145          for i in xrange(len(pnames)): 
2146              p = pnames[i] 
2147              pardefines += "\t" + p + " = p_(" + str(i+1) + ");\n" 
2148  
 
2149          alldefines = "\n% Parameter definitions\n\n" + pardefines 
2150          return alldefines

2151  
 
2152  
 


2153 -    def _prepareMatlabVDefines(self, vnames):


2154          vardefines = "" 
2155          for i in xrange(len(vnames)): 
2156              v = vnames[i] 
2157              vardefines += "\t" + v + " = x_(" + str(i+1) + ");\n" 
2158          alldefines = "\n% Variable definitions\n\n" + vardefines 
2159          return alldefines

2160  
 
2161  
 
2162      # ------------ Other utilities -----------------------
 
2163  
 


2164 -    def _infostr(self, verbose=1):


2165          if verbose == 0: 
2166              outputStr = "FuncSpec " + self.name 
2167          else: 
2168              outputStr = '*********** FuncSpec:  '+self.name + ' ***********' 
2169              outputStr += '\nTarget lang:  '+ self.targetlang 
2170              outputStr += '\nVariables:  ' 
2171              for v in self.vars: 
2172                  outputStr += v+'  ' 
2173              outputStr += '\nParameters:  ' 
2174              if len(self.pars): 
2175                  for p in self.pars: 
2176                      outputStr += p+'  ' 
2177              else: 
2178                  outputStr += '[]' 
2179              outputStr += '\nExternal inputs:  ' 
2180              if len(self.inputs): 
2181                  for i in self.inputs: 
2182                      outputStr += i+'  ' 
2183              else: 
2184                  outputStr += '[]' 
2185          if verbose == 2: 
2186              outputStr += "\nSpecification functions (in target language):" 
2187              outputStr += "\n  (ignore any arguments `ds` and `parsinps`," \
 
2188                         + "\n   which are for internal use only)\n" 
2189              if self.spec == {}: 
2190                  outputStr += "\n None\n" 
2191              else: 
2192                  outputStr += "\n  "+self.spec[0]+"\n" 
2193              if len(self.auxvars) and self.auxspec != {}: 
2194                  outputStr += " "+self.auxspec[0] 
2195              if self._protected_auxnames != []: 
2196                  outputStr += '\n\nUser-defined auxiliary variables:  ' 
2197                  for v in self.auxvars: 
2198                      outputStr += v+'  ' 
2199                  outputStr += '\n\nUser-defined auxiliary functions (in target ' + \
 
2200                               'language):' 
2201                  for auxname in self.auxfns: 
2202                      # verbose option shows up builtin auxiliary func definitions
 
2203                      if auxname not in self._builtin_auxnames or verbose>0: 
2204                          outputStr += '\n  '+self.auxfns[auxname][0]+'\n' 
2205              outputStr += "\n\nDependencies in specification functions - pair (i, o)"\
 
2206                      " means i depends on o:\n  " + str(self.dependencies) 
2207          return outputStr

2208  
 


2209 -    def info(self, verbose=0):


2210          print self._infostr(verbose)

2211  
 


2212 -    def __repr__(self):


2213          return self._infostr(verbose=0)

2214  
 
2215      __str__ = __repr__ 
2216  
 
2217  
 
2218  
 
2219  # -----------------------------------
 
2220  
 
2221  # Sub-classes of FuncSpec
 
2222  
 


2223 -class RHSfuncSpec(FuncSpec):


2224      """Right-hand side definition for vars defined.""" 
2225  
 


2226 -    def __init__(self, kw):


2227          FuncSpec.__init__(self, kw)

2228  
 
2229  
 
2230  
 


2231 -class ExpFuncSpec(FuncSpec):


2232      """Explicit definition of vars defined.""" 
2233  
 


2234 -    def __init__(self, kw):


2235          assert 'codeinsert_start' not in kw, ('code inserts invalid for '
 
2236                                              'explicit function specification') 
2237          assert 'codeinsert_end' not in kw, ('code inserts invalid for '
 
2238                                              'explicit function specification') 
2239          FuncSpec.__init__(self, kw)

2240  
 
2241  
 
2242  
 


2243 -class ImpFuncSpec(FuncSpec):


2244      """Assumes this will be set to equal zero when solving for vars defined.""" 
2245  
 
2246      # funcspec will possibly be the same for several variables
 
2247      # so it's repeated, but must be checked so that only solved
 
2248      # once for all relevant variables
 


2249 -    def __init__(self, kw):


2250          assert 'codeinsert_start' not in kw, ('code inserts invalid for '
 
2251                                              'implicit function specification') 
2252          assert 'codeinsert_end' not in kw, ('code inserts invalid for '
 
2253                                              'implicit function specification') 
2254          FuncSpec.__init__(self, kw)

2255  
 
2256  
 
2257  
 


2258 -def _processReused(specnames, specdict, reuseterms, indentstr='',
 
2259                      typestr='', endstatementchar='', parseFunc=idfn):


2260      """Process substitutions of reused terms.""" 
2261  
 
2262      seenrepterms = []  # for new protected names (global to all spec names) 
2263      reused = {}.fromkeys(specnames) 
2264      reuseterms_inv = invertMap(reuseterms) 
2265      # establish order for reusable terms, in case of inter-dependencies
 
2266      are_dependent = [] 
2267      deps = {} 
2268      for origterm, rterm in reuseterms.iteritems(): 
2269          for ot, rt in reuseterms.iteritems(): 
2270              if proper_match(origterm, rt): 
2271                  if rterm not in are_dependent: 
2272                      are_dependent.append(rterm) 
2273                  try: 
2274                      deps[rterm].append(rt) 
2275                  except KeyError: 
2276                      # new list
 
2277                      deps[rterm] = [rt] 
2278      order = remain(reuseterms.values(), are_dependent) + are_dependent 
2279      for specname in specnames: 
2280          reused[specname] = [] 
2281          specstr = specdict[specname] 
2282          repeatkeys = [] 
2283          for origterm, repterm in reuseterms.iteritems(): 
2284              # only add definitions if string found
 
2285              if proper_match(specstr, origterm): 
2286                  specstr = specstr.replace(origterm, repterm) 
2287                  if repterm not in seenrepterms: 
2288                      reused[specname].append([indentstr,
 
2289                                          typestr+' '*(len(typestr)>0),
 
2290                                          repterm, " = ",
 
2291                                          parseFunc(origterm),
 
2292                                          endstatementchar, "\n"]) 
2293                      seenrepterms.append(repterm) 
2294              else: 
2295                  # look for this term on second pass
 
2296                  repeatkeys.append(origterm) 
2297          if len(seenrepterms) > 0: 
2298              # don't bother with a second pass if specstr has not changed
 
2299              for origterm in repeatkeys: 
2300                  # second pass
 
2301                  repterm = reuseterms[origterm] 
2302                  if proper_match(specstr, origterm): 
2303                      specstr = specstr.replace(origterm, repterm) 
2304                      if repterm not in seenrepterms: 
2305                          seenrepterms.append(repterm) 
2306                          reused[specname].append([indentstr,
 
2307                                                   typestr+' '*(len(typestr)>0),
 
2308                                                   repterm, " = ",
 
2309                                                   parseFunc(origterm),
 
2310                                                   endstatementchar, "\n"]) 
2311          # if replacement terms have already been used in the specifications
 
2312          # and there are no occurrences of the terms meant to be replaced then
 
2313          # just log the definitions that will be needed without replacing
 
2314          # any strings.
 
2315          if reused[specname] == [] and len(reuseterms) > 0: 
2316              for origterm, repterm in reuseterms.iteritems(): 
2317                  # add definition if *replacement* string found in specs
 
2318                  if proper_match(specstr, repterm) and repterm not in seenrepterms: 
2319                      reused[specname].append([indentstr,
 
2320                                          typestr+' '*(len(typestr)>0),
 
2321                                          repterm, " = ",
 
2322                                          parseFunc(origterm),
 
2323                                          endstatementchar, "\n"]) 
2324                      seenrepterms.append(repterm) 
2325          specdict[specname] = specstr 
2326          # add any dependencies for repeated terms to those that will get
 
2327          # defined when functions are instantiated
 
2328          add_reps = [] 
2329          for r in seenrepterms: 
2330              if r in are_dependent: 
2331                  for repterm in deps[r]: 
2332                      if repterm not in seenrepterms: 
2333                          reused[specname].append([indentstr,
 
2334                                          typestr+' '*(len(typestr)>0),
 
2335                                          repterm, " = ",
 
2336                                          parseFunc(reuseterms_inv[repterm]),
 
2337                                          endstatementchar, "\n"]) 
2338                          seenrepterms.append(repterm) 
2339      # reuseterms may be automatically provided for a range of definitions
 
2340      # that may or may not contain instances, and it's too inefficient to
 
2341      # check in advance, so we'll not cause an error here if none show up.
 
2342  ##    if len(seenrepterms) == 0 and len(reuseterms) > 0:
 
2343  ##        print "Reuse terms expected:", reuseterms
 
2344  ##        info(specdict)
 
2345  ##        raise RuntimeError("Declared reusable term definitions did not match"
 
2346  ##                           " any occurrences in the specifications")
 
2347      return (reused, specdict, seenrepterms, order)

2348  
 
2349  
 
2350  
 
2351  
 
2352  # ----------------------------------------------
 
2353  ## Public exported functions
 
2354  # ----------------------------------------------
 
2355  
 


2356 -def makePartialJac(spec_pair, varnames, select=None):


2357      """Use this when parameters have been added to a modified Generator which
 
2358      might clash with aux fn argument names. (E.g., used by find_nullclines).
 
2359  
 
2360      'select' option (list of varnames) selects those entries from the Jac of the varnames,
 
2361         e.g. for constructing Jacobian w.r.t. 'parameters' using a parameter formerly
 
2362         a variable (e.g. for find_nullclines).
 
2363      """ 
2364      fargs, fspec = spec_pair 
2365      J = QuantSpec('J', fspec) 
2366      # find positions of actual varnames in f argument list
 
2367      # then extract terms from the Jacobian matrix, simplifying to a scalar if 1D
 
2368      dim = len(varnames) 
2369      if J.dim == dim: 
2370          # nothing to do
 
2371          return (fargs, fspec) 
2372      assert J.dim > dim, "Cannot add variable names to system while using its old Jacobian aux function" 
2373      assert remain(varnames, fargs) == [], "Invalid variable names to resolve Jacobian aux function" 
2374      assert fargs[0] == 't' 
2375      # -1 adjusts for 't' being the first argument
 
2376      vixs = [fargs.index(v)-1 for v in varnames] 
2377      vixs.sort() 
2378      if select is None: 
2379          select = varnames 
2380          sixs = vixs 
2381      else: 
2382          sixs = [fargs.index(v)-1 for v in select] 
2383      if dim == 1: 
2384          fspec = str(J.fromvector(vixs[0]).fromvector(sixs[0])) 
2385      else: 
2386          terms = [] 
2387          for i in vixs: 
2388              Ji = J.fromvector(i) 
2389              subterms = [] 
2390              for j in sixs: 
2391                  subterms.append( str(Ji.fromvector(j)) ) 
2392              terms.append( "[" + ",".join(subterms) + "]" ) 
2393          fspec = "[" + ",".join(terms) + "]" 
2394      # retain order of arguments
 
2395      fargs_new = ['t'] + [fargs[ix+1] for ix in vixs] 
2396      return (fargs_new, fspec)

2397  
 
2398  
 


2399 -def resolveClashingAuxFnPars(fnspecs, varspecs, parnames):


2400      """Use this when parameters have been added to a modified Generator which
 
2401      might clash with aux fn argument names. (E.g., used by find_nullclines).
 
2402      Will remove arguments that are now considered parameters by the system,
 
2403      in both the function definitions and their use in specs for the variables.
 
2404      """ 
2405      changed_fns = [] 
2406      new_fnspecs = {} 
2407      for fname, (fargs, fspec) in fnspecs.iteritems(): 
2408          common_names = intersect(fargs, parnames) 
2409          if fname in parnames: 
2410              print "Problem with function definition", fname 
2411              raise ValueError("Unrecoverable clash between parameter names and aux fn name") 
2412          if common_names == []: 
2413              new_fnspecs[fname] = (fargs, fspec) 
2414          else: 
2415              changed_fns.append(fname) 
2416              new_fnspecs[fname] = (remain(fargs, parnames), fspec) 
2417  
 
2418      new_varspecs = {} 
2419      for vname, vspec in varspecs.iteritems(): 
2420          q = QuantSpec('__temp__', vspec) 
2421          # only update use of functions both changed and used in the varspecs
 
2422          used_fns = intersect(q.parser.tokenized, changed_fns) 
2423          for f in used_fns: 
2424              ix = q.parser.tokenized.index(f) 
2425              # identify arg list for this fn call
 
2426              rest = ''.join(q.parser.tokenized[ix+1:]) 
2427              end_ix = findEndBrace(rest) 
2428              # get string of this arg list
 
2429              argstr = rest[:end_ix+1] 
2430              # split
 
2431              success, args_list, arglen = readArgs(argstr) 
2432              assert success, "Parsing arguments failed" 
2433              new_args_list = [] 
2434              # remove parnames
 
2435              for arg in args_list: 
2436                  qarg = QuantSpec('a', arg) 
2437                  # if parameter appears in a compound expression in the argument,
 
2438                  # then we don't know how to process it, so raise exception
 
2439                  if len(qarg) > 1 and any([p in qarg for p in parnames]): 
2440                      raise ValueError("Cannot process argument to aux fn %s"%f) 
2441                  # do not put raw parameter name arguments into new arg list
 
2442                  if arg not in parnames: 
2443                      new_args_list.append(arg) 
2444              new_argstr = ','.join(new_args_list) 
2445              # update vspec and q for next f
 
2446              vspec = ''.join(q[:ix+1]) + '(' + new_argstr + ')' + rest[end_ix+1:] 
2447              q = QuantSpec('__temp__', vspec) 
2448          new_varspecs[vname] = vspec 
2449      return new_fnspecs, new_varspecs

2450  
 
2451  
 
2452  
 


2453 -def getSpecFromFile(specfilename):


2454      """Read text specs from a file""" 
2455      try: 
2456          f = open(specfilename, 'r') 
2457          s = f.read() 
2458      except IOError, e: 
2459          print 'File error:', str(e) 
2460          raise 
2461      f.close() 
2462      return s

2463
```

  


| Home | Trees | Indices | Help | | PyDSTool | | --- | |
| --- | --- | --- | --- | --- | --- |

|  |  |
| --- | --- |
| Generated by Epydoc 3.0.1 on Fri May 4 15:24:19 2012 | http://epydoc.sourceforge.net |
