# Supplementary material for: Hybrid Models and Biological Model Reduction with PyDSTool
Source: PLoS Comput Biol. 2012 Aug 9;8(8):e1002628. doi: 10.1371/journal.pcbi.1002628 (PMC3415397; doi:10.1371/journal.pcbi.1002628)
Supplement: Text S4 — Complete source code for the PyDSTool package (version 0.88.120504). Includes API documentation and help files linking to web pages. This file is identical to the current public release on Sourceforge.net. (ZIP) [file pcbi.1002628.s004.zip › PyDSTool/html/PyDSTool.FuncSpec'.FuncSpec-class.html]

xml version="1.0" encoding="ascii"?


PyDSTool.FuncSpec'.FuncSpec


| Home | Trees | Indices | Help | | PyDSTool | | --- | |
| --- | --- | --- | --- | --- | --- |

|  |  |  |  |
| --- | --- | --- | --- |
| Package PyDSTool :: Module FuncSpec' :: Class FuncSpec | |  | | --- | | [hide private] | | [frames] | no frames] | |

# Class FuncSpec

source code

```
object --+
         |
        FuncSpec
```

Known Subclasses:
:   - ExpFuncSpec
    - , ImpFuncSpec
    - , RHSfuncSpec

---

```
Functional specification of dynamics: abstract class.

NOTES ON BUILT-IN AUX FUNCTIONS (WITH SYNTAX AS USED IN SPEC STRING):

globalindepvar(t) -> global independent variable (time) reference

initcond(varname) -> initial condition of that variable in this DS

heav(x) = 1 if x > 0, 0 otherwise

getindex(varname) -> index of varname in internal representation of
 variables as array

getbound(name, which_bd) -> value of user-defined bound on the named
 variable or parameter, either the lower (which_bd=0) or higher
 (which_bd=1)

if(condition, expr1, expr2) -> if condition as a function of state,
 parameters and time is true, then evaluate <expr1>, else evaluate
 <expr2>.

MACRO `for` SYNTAX:

 for(i, ilo, ihi, expr_in_i) -> list of expressions where each
  occurrence of `[i]` is replaced with the appropriate integer.
  The letter i can be replaced with any other single character.

MACRO `sum` SYNTAX:

 sum(i, ilo, ihi, expr_in_i) -> an expression that sums
  over the expression replacing any occurrence of `[i]` with
  the appropriate integer.
```


|  |  |  |  |
| --- | --- | --- | --- |
| |  |  | | --- | --- | | Instance Methods | [hide private] | | |
|  | |  |  | | --- | --- | | \_\_call\_\_(self) | source code | |
|  | |  |  | | --- | --- | | \_\_hash\_\_(self)  Unique identifier for this specification. | source code | |
|  | |  |  | | --- | --- | | \_\_init\_\_(self, kw)  x.\_\_init\_\_(...) initializes x; see x.\_\_class\_\_.\_\_doc\_\_ for signature | source code | |
|  | |  |  | | --- | --- | | \_\_processTokens(self, allnames, specialtokens, specstr, var\_arrayixstr, aux\_arrayixstr, parsinps\_names, parsinps\_arrayixstr, specname, ignoreothers=False, doing\_inserts=False) | source code | |
|  | |  |  | | --- | --- | | \_\_repr\_\_(self)  str(x) | source code | |
|  | |  |  | | --- | --- | | \_\_str\_\_(self)  str(x) | source code | |
|  | |  |  | | --- | --- | | \_doPreMacrosC(self) | source code | |
|  | |  |  | | --- | --- | | \_genAuxFnC(self) | source code | |
|  | |  |  | | --- | --- | | \_genAuxFnMatlab(self) | source code | |
|  | |  |  | | --- | --- | | \_genAuxFnPy(self, pytarget=False) | source code | |
|  | |  |  | | --- | --- | | \_genSpecC(self) | source code | |
|  | |  |  | | --- | --- | | \_genSpecFnC(self, funcname, reusestr, specnames, pardefines, vardefines, inpdefines, parundefines, varundefines, inpundefines, docodeinserts) | source code | |
|  | |  |  | | --- | --- | | \_genSpecFnMatlab(self, funcname, reusestr, specnames, pardefines, vardefines, docodeinserts) | source code | |
|  | |  |  | | --- | --- | | \_genSpecFnPy(self, name, specstr, resname, specnames, docodeinserts=False) | source code | |
|  | |  |  | | --- | --- | | \_genSpecMatlab(self) | source code | |
|  | |  |  | | --- | --- | | \_genSpecPy(self) | source code | |
|  | |  |  | | --- | --- | | \_infostr(self, verbose=1) | source code | |
|  | |  |  | | --- | --- | | \_macroFor(self, rootstr, istr, ilo, ihi, expr\_in\_i)  Internal utility function to build multiple instances of expression 'expr\_in\_i' where integer i has been substituted for values from ilo to ihi. | source code | |
|  | |  |  | | --- | --- | | \_macroSum(self, istr, ilo, ihi, expr\_in\_i) | source code | |
|  | |  |  | | --- | --- | | \_parseReusedTermsPy(self, d, symbol\_ixs, specials=`[``]`, dovars=True, dopars=True, doinps=True, illegal=`[``]`)  Process dictionary of reused term definitions (in spec syntax). | source code | |
|  | |  |  | | --- | --- | | \_prepareMatlabPDefines(self, pnames) | source code | |
|  | |  |  | | --- | --- | | \_prepareMatlabVDefines(self, vnames) | source code | |
|  | |  |  | | --- | --- | | \_processIfMatlab(self, specStr) | source code | |
|  | |  |  | | --- | --- | | \_processReusedC(self, specnames, specdict)  Process reused subexpression terms for C code. | source code | |
|  | |  |  | | --- | --- | | \_processReusedMatlab(self, specnames, specdict)  Process reused subexpression terms for Matlab code. | source code | |
|  | |  |  | | --- | --- | | \_processReusedPy(self, specnames, specdict, specials=`[``]`, dovars=True, dopars=True, doinps=True, illegal=`[``]`)  Process reused subexpression terms for Python code. | source code | |
|  | |  |  | | --- | --- | | \_processSpecialC(self, specStr)  Pre-process 'if' statements and names of 'abs' and 'sign' functions, as well as logical operators. | source code | |
|  | |  |  | | --- | --- | | \_specStrParse(self, specnames, specdict, resname=`'``'`, specials=`[``]`, dovars=True, dopars=True, doinps=True, noreturndefs=False, forexternal=False, illegal=`[``]`, ignoreothers=False, doing\_inserts=False) | source code | |
|  | |  |  | | --- | --- | | doPreMacros(self)  Pre-process any macro spec definitions (e.g. | source code | |
|  | |  |  | | --- | --- | | generateAuxFns(self) | source code | |
|  | |  |  | | --- | --- | | generateSpec(self)  Automatically generate callable target-language functions from the user-defined specification strings. | source code | |
|  | |  |  | | --- | --- | | info(self, verbose=0) | source code | |
|  | |  |  | | --- | --- | | recreate(self, targetlang) | source code | |
|  | |  |  | | --- | --- | | validateDef(self, vars, pars, inputs, auxvars, auxfns)  Validate definition of the functional specification. | source code | |
|  | |  |  | | --- | --- | | validateDependencies(self, dependencies)  Validate the stored dependency pairs for self-consistency. | source code | |
| **Inherited from `object`**: `__delattr__`, `__getattribute__`, `__new__`, `__reduce__`, `__reduce_ex__`, `__setattr__` | |


|  |  |  |  |
| --- | --- | --- | --- |
| |  |  | | --- | --- | | Properties | [hide private] | | |
| **Inherited from `object`**: `__class__` | |


|  |  |  |  |
| --- | --- | --- | --- |
| |  |  | | --- | --- | | Method Details | [hide private] | | |

|  |  |  |
| --- | --- | --- |
| |  |  | | --- | --- | | \_\_hash\_\_(self)  *(Hashing function)* | source code |   Unique identifier for this specification.  Overrides: object.\_\_hash\_\_ |

|  |  |  |
| --- | --- | --- |
| |  |  | | --- | --- | | \_\_init\_\_(self, kw)  *(Constructor)* | source code |   x.\_\_init\_\_(...) initializes x; see x.\_\_class\_\_.\_\_doc\_\_ for signature  Overrides: object.\_\_init\_\_ *(inherited documentation)* |

|  |  |  |
| --- | --- | --- |
| |  |  | | --- | --- | | \_\_repr\_\_(self)  *(Representation operator)* | source code |   str(x)  Overrides: object.\_\_repr\_\_ *(inherited documentation)* |

|  |  |  |
| --- | --- | --- |
| |  |  | | --- | --- | | \_\_str\_\_(self)  *(Informal representation operator)* | source code |   str(x)  Overrides: object.\_\_str\_\_ *(inherited documentation)* |

|  |  |  |
| --- | --- | --- |
| |  |  | | --- | --- | | \_macroFor(self, rootstr, istr, ilo, ihi, expr\_in\_i) | source code |   Internal utility function to build multiple instances of expression 'expr\_in\_i' where integer i has been substituted for values from ilo to ihi. Returns dictionary keyed by rootstr+str(i) for each i. |

|  |  |  |
| --- | --- | --- |
| |  |  | | --- | --- | | doPreMacros(self) | source code |   Pre-process any macro spec definitions (e.g. `for` loops). |

  


| Home | Trees | Indices | Help | | PyDSTool | | --- | |
| --- | --- | --- | --- | --- | --- |

|  |  |
| --- | --- |
| Generated by Epydoc 3.0.1 on Fri May 4 15:24:06 2012 | http://epydoc.sourceforge.net |
