# Supplementary material for: Hybrid Models and Biological Model Reduction with PyDSTool
Source: PLoS Comput Biol. 2012 Aug 9;8(8):e1002628. doi: 10.1371/journal.pcbi.1002628 (PMC3415397; doi:10.1371/journal.pcbi.1002628)
Supplement: Text S4 — Complete source code for the PyDSTool package (version 0.88.120504). Includes API documentation and help files linking to web pages. This file is identical to the current public release on Sourceforge.net. (ZIP) [file pcbi.1002628.s004.zip › PyDSTool/html/PyDSTool.Generator-module.html]

xml version="1.0" encoding="ascii"?


PyDSTool.Generator


| Home | Trees | Indices | Help | | PyDSTool | | --- | |
| --- | --- | --- | --- | --- | --- |

|  |  |  |  |
| --- | --- | --- | --- |
| Package PyDSTool :: Package Generator | |  | | --- | | [hide private] | | [frames] | no frames] | |

# Package Generator

source code

Trajectory generator classes.

Robert Clewley, September 2005


|  |  |  |  |
| --- | --- | --- | --- |
| |  |  | | --- | --- | | Submodules | [hide private] | | |
| - **PyDSTool.Generator.ADMC\_ODEsystem** - **PyDSTool.Generator.ADMC\_ODEsystem'** - **PyDSTool.Generator.DDEsystem** - **PyDSTool.Generator.Dopri\_ODEsystem** - **PyDSTool.Generator.Dopri\_ODEsystem'** - **PyDSTool.Generator.EmbeddedSysGen** - **PyDSTool.Generator.EmbeddedSysGen'** - **PyDSTool.Generator.Euler\_ODEsystem** - **PyDSTool.Generator.Euler\_ODEsystem'**: *Euler integrator for ODE systems, with no step refinement for   events.* - **PyDSTool.Generator.ExplicitFnGen** - **PyDSTool.Generator.ExplicitFnGen'** - **PyDSTool.Generator.ExtrapolateTable** - **PyDSTool.Generator.ExtrapolateTable'** - **PyDSTool.Generator.ImplicitFnGen** - **PyDSTool.Generator.ImplicitFnGen'** - **PyDSTool.Generator.InterpolateTable** - **PyDSTool.Generator.InterpolateTable'** - **PyDSTool.Generator.LookupTable** - **PyDSTool.Generator.LookupTable'** - **PyDSTool.Generator.MapSystem** - **PyDSTool.Generator.MapSystem'** - **PyDSTool.Generator.ODEsystem** - **PyDSTool.Generator.ODEsystem'** - **PyDSTool.Generator.Radau\_ODEsystem** - **PyDSTool.Generator.Radau\_ODEsystem'** - **PyDSTool.Generator.Vode\_ODEsystem** - **PyDSTool.Generator.Vode\_ODEsystem'**: *VODE integrator for ODE systems, imported from a mild modification   of the scipy-wrapped VODE Fortran solver.* - **PyDSTool.Generator.allimports** - **PyDSTool.Generator.baseclasses** - **PyDSTool.Generator.messagecodes**: *Message code definitions for Generators* |

  


|  |  |  |  |
| --- | --- | --- | --- |
| |  |  | | --- | --- | | Functions | [hide private] | | |
|  | |  |  | | --- | --- | | findGenSubClasses(superclass)  Find all Generator sub-classes of a certain class, e.g. | source code | |


|  |  |  |  |
| --- | --- | --- | --- |
| |  |  | | --- | --- | | Variables | [hide private] | | |
|  | e = `2.71828182846` |
|  | pi = `3.14159265359` |


|  |  |  |  |
| --- | --- | --- | --- |
| |  |  | | --- | --- | | Function Details | [hide private] | | |

|  |  |  |
| --- | --- | --- |
| |  |  | | --- | --- | | findGenSubClasses(superclass) | source code |   Find all Generator sub-classes of a certain class, e.g. ODEsystem. |

  


| Home | Trees | Indices | Help | | PyDSTool | | --- | |
| --- | --- | --- | --- | --- | --- |

|  |  |
| --- | --- |
| Generated by Epydoc 3.0.1 on Fri May 4 15:24:02 2012 | http://epydoc.sourceforge.net |
