# Supplementary material for: Hybrid Models and Biological Model Reduction with PyDSTool
Source: PLoS Comput Biol. 2012 Aug 9;8(8):e1002628. doi: 10.1371/journal.pcbi.1002628 (PMC3415397; doi:10.1371/journal.pcbi.1002628)
Supplement: Text S4 — Complete source code for the PyDSTool package (version 0.88.120504). Includes API documentation and help files linking to web pages. This file is identical to the current public release on Sourceforge.net. (ZIP) [file pcbi.1002628.s004.zip › PyDSTool/html/PyDSTool.Generator-pysrc.html]

xml version="1.0" encoding="ascii"?


PyDSTool.Generator


| Home | Trees | Indices | Help | | PyDSTool | | --- | |
| --- | --- | --- | --- | --- | --- |

|  |  |  |  |
| --- | --- | --- | --- |
| Package PyDSTool :: Package Generator | |  | | --- | | [hide private] | | [frames] | no frames] | |

# Source Code for Package PyDSTool.Generator

```
 1  """Trajectory generator classes.
 
 2  
 
 3     Robert Clewley, September 2005
 
 4  """ 
 5  
 
 6  from baseclasses import * 
 7  from ODEsystem import * 
 8  from Euler_ODEsystem import * 
 9  from Vode_ODEsystem import * 
10  from Dopri_ODEsystem import * 
11  from Radau_ODEsystem import * 
12  from ADMC_ODEsystem import * 
13  from ExplicitFnGen import * 
14  from ImplicitFnGen import * 
15  from EmbeddedSysGen import * 
16  from LookupTable import * 
17  from InterpolateTable import * 
18  from ExtrapolateTable import * 
19  from MapSystem import * 
20  
 
21  
 


22 -def findGenSubClasses(superclass):


23      """Find all Generator sub-classes of a certain class, e.g. ODEsystem.""" 
24      assert isinstance(superclass, str), \
 
25             "findGenSubClasses requires a string as the name of the class to search for subclasses." 
26      subclasslist = [] 
27      sc = eval(superclass) 
28      for x in theGenSpecHelper.gshDB.keys(): 
29          if compareClassAndBases(theGenSpecHelper.gshDB[x].genClass,sc): 
30              subclasslist.append(x) 
31      return subclasslist

32
```

  


| Home | Trees | Indices | Help | | PyDSTool | | --- | |
| --- | --- | --- | --- | --- | --- |

|  |  |
| --- | --- |
| Generated by Epydoc 3.0.1 on Fri May 4 15:24:11 2012 | http://epydoc.sourceforge.net |
