# Supplementary material for: Hybrid Models and Biological Model Reduction with PyDSTool
Source: PLoS Comput Biol. 2012 Aug 9;8(8):e1002628. doi: 10.1371/journal.pcbi.1002628 (PMC3415397; doi:10.1371/journal.pcbi.1002628)
Supplement: Text S4 — Complete source code for the PyDSTool package (version 0.88.120504). Includes API documentation and help files linking to web pages. This file is identical to the current public release on Sourceforge.net. (ZIP) [file pcbi.1002628.s004.zip › PyDSTool/html/PyDSTool.Generator.ADMC_ODEsystem'-pysrc.html]

xml version="1.0" encoding="ascii"?


PyDSTool.Generator.ADMC\_ODEsystem'


| Home | Trees | Indices | Help | | PyDSTool | | --- | |
| --- | --- | --- | --- | --- | --- |

|  |  |  |  |
| --- | --- | --- | --- |
| Package PyDSTool :: Package Generator :: Module ADMC\_ODEsystem' | |  | | --- | | [hide private] | | [frames] | no frames] | |

# Source Code for Module PyDSTool.Generator.ADMC\_ODEsystem'

```
  1  # ADMC++ ODE system
 
  2  from __future__ import division 
  3  
 
  4  from allimports import * 
  5  from PyDSTool.Generator import ODEsystem as ODEsystem 
  6  from baseclasses import Generator, theGenSpecHelper 
  7  from PyDSTool.utils import * 
  8  from PyDSTool.common import * 
  9  from PyDSTool.integrator import integrator 
 10  import PyDSTool.fixedpickle as fpickle 
 11  
 
 12  # Other imports
 
 13  from numpy import Inf, NaN, isfinite, sometrue, alltrue, isnan, zeros 
 14  import math, random 
 15  from copy import copy, deepcopy 
 16  import os, platform, shutil, sys, gc 
 17  import distutils 
 18  from distutils.core import setup, Extension 
 19  from distutils.sysconfig import get_python_inc 
 20  from time import clock, sleep 
 21  
 
 22  # path to the installation
 
 23  import PyDSTool 
 24  _pydstool_path = PyDSTool.__path__[0] 
 25  
 
 26  # Removed integrator subclass since this just generates code for
 
 27  # ADMC++ for matlab to deal with
 
 28  
 


29 -class ADMC_ODEsystem(ODEsystem):


30      """Wrapper for code generator for ADMC++32 and Matlab.
 
 31      Uses Matlab functional specifications only.""" 
 32  
 


33 -    def __init__(self, kw):


34          """Use the nobuild key to postpone building of the library, e.g. in
 
 35          order to provide additional build options to makeLibSource and
 
 36          compileLib methods or to make changes to the C code by hand.
 
 37          No build options can be specified otherwise.""" 
 38  
 
 39          # Building is just doing make
 
 40          if 'nobuild' in kw: 
 41              nobuild = kw['nobuild'] 
 42              del kw['nobuild'] 
 43          else: 
 44              nobuild = False 
 45          ODEsystem.__init__(self, kw) 
 46          self._solver = None 
 47          assert self.funcspec.targetlang == 'matlab', \
 
 48                 ('Wrong target language for functional specification. '
 
 49                  'matlab needed for this class') 
 50          assert isinstance(self.funcspec, RHSfuncSpec), ('ADMC++ '
 
 51                                      'requires RHSfuncSpec type to proceed') 
 52          assert not self.inputs, \
 
 53                          'ADMC++ does not support external inputs feature' 
 54          self._errorcodes = {} 
 55          self._paraminfo = {} 
 56  
 
 57          self.vftype = 'vfieldts' 
 58  
 
 59          # currently the final four of these params are for event handling
 
 60          # NEED TO CHECK WHICH ONES ARE SUPPORTED BY ADMC -- LOOKS LIKE EVTOLS ONLY FOR NOW
 
 61          # HACK: vftype is alg param for now, tells us whether parent class is hybridvf, vfieldts, etc.
 
 62          algparams_def = {'evtols' : 0.0001, 'vftype' : 'vfieldts'} 
 63  
 
 64          # Remove this later
 
 65          for k, v in algparams_def.iteritems(): 
 66              if k not in self.algparams: 
 67                  self.algparams[k] = v 
 68  
 
 69          # verify that no additional keys are present in algparams, after
 
 70          # defaults are added above
 
 71          if len(self.algparams) != len(algparams_def): 
 72              raise ValueError("Invalid keys present in algparams argument: " \
 
 73                       + str(remain(self.algparams.keys(),algparams_def.keys()))) 
 74  
 
 75          thisplatform = platform.system() 
 76  
 
 77          self._compilation_tempdir = os.path.join(os.getcwd(),
 
 78                                                        "admcpp_temp") 
 79          if not os.path.isdir(self._compilation_tempdir): 
 80              try: 
 81                  assert not os.path.isfile(self._compilation_tempdir), \
 
 82                       "A file already exists with the same name" 
 83                  os.mkdir(self._compilation_tempdir) 
 84              except: 
 85                  print "Could not create compilation temp directory " + \
 
 86                        self._compilation_tempdir 
 87                  raise 
 88  
 
 89          # ADMC targets must go in their own directories with appropriate names
 
 90          self._model_dir = "@"+self.name 
 91          self._target_dir = os.path.join(self._compilation_tempdir,self._model_dir) 
 92          # Make the target directory
 
 93          if not os.path.isdir(self._target_dir): 
 94              try: 
 95                  assert not os.path.isfile(self._target_dir), \
 
 96                         "A file already exists with the same name" 
 97                  os.mkdir(self._target_dir) 
 98              except: 
 99                  print "Could not creat target ADMC model directory " + \
 
100                        self._target_dir 
101                  raise 
102  
 
103  
 
104          """ An ADMC model has the following files:
 
105          vfield.m -- contains code for the RHS of the vector field
 
106          set.m -- a generic method that overload matlab's set method; only need to insert vfield name
 
107          get.m -- a generic method that overloads matlab's get method; only need to insert appropriate parent name
 
108          """ 
109  
 
110          # model.m, get.m, set.m, vfield.m are minimal files required. TO DO: EVENTS
 
111          self._model_file = self.name+".m" 
112          self._ic_file = self.name+"_ics.m" 
113          self._param_file = self.name+"_params.m" 
114          self._set_file = "set.m" 
115          self._get_file = "get.m" 
116          self._vfield_file = "vfield.m" 
117          self._events_file = "events.m" 
118  
 
119          self._vf_filename_ext = "_"+self._model_file[:-2] 
120  
 
121          if not nobuild: 
122              self.makeLibSource() 
123          else: 
124              print "Build the library using the makeLib method, or in " 
125              print "stages using the makeLibSource and compileLib methods."

126  
 
127  
 


128 -    def _prepareEventSpecs(self):


129          # in admc++, all events are terminal, must be 0 or 1 for direction, no delay, no tolerances, etc.
 
130          eventDir = [] 
131  
 
132  #       eventTol = []
 
133  
 
134          # convert event specs (term, active, etc.) into integparam specs
 
135          self._eventNames = self.eventstruct.sortedEventNames() 
136          for evname in self._eventNames: 
137              ev = self.eventstruct.events[evname] 
138              assert isinstance(ev, MatlabEvent), ("ADMC++ can only "
 
139                                                   "accept matlab events") 
140  
 
141          for evname in self._eventNames: 
142              ev = self.eventstruct.events[evname]

143              #assert ev.dircode in [-1,1], ("ADMC++ requires events to have direction -1 or 1")
 
144              #eventDir.append(ev.dircode)
 
145  
 
146          #self.algparams['eventDir'] = eventDir
 
147          #self.algparams['eventTol'] = eventTol
 
148          #self.algparams['eventDelay'] = eventDelay
 
149          #self.algparams['eventInt'] = eventInt
 
150          #self.algparams['maxbisect'] = maxbisect
 
151          #self.algparams['eventActive'] = eventActive
 
152          #self.algparams['eventTerm'] = eventTerm
 
153  
 


154 -    def _prepareEventsFileContents(self):


155  
 
156          allfilestr = "" 
157  
 
158          evname = self._eventNames 
159          evcount = len(evname) 
160          if evcount < 1: 
161              return allfilestr 
162  
 
163          topstr = "function [vf_, ev_] = events(vf_, t_, x_, p_, state_)" 
164          commentstr = "\n% Events method for model " + self.name + "\n% Generated by PyDSTool for ADMC++ target\n\n" 
165          alldefines = self._prepareVfieldDefines() 
166  
 
167          allfilestr = topstr + commentstr + alldefines 
168  
 
169          evassign = "\tev_ = zeros(1," + str(evcount) + ");\n" 
170          for x in range(evcount): 
171              ev = self.eventstruct.events[evname[x]] 
172              evassign += "\tev_(" + str(x+1) + ") = " + ev.name + ev._LLargstr + ";\n" 
173  
 
174          allfilestr += evassign + "\n\n" 
175  
 
176          for x in range(evcount): 
177  
 
178              ev = self.eventstruct.events[evname[x]] 
179              evfullfn = "" 
180              evsig = "function " + ev._LLreturnstr + ev.name + ev._LLargstr + "\n" 
181              assert ev._LLfuncstr.index(';') > 1, ("End your event function with a ';'") 
182  
 
183              fbody =  "%BEGIN func " + ev.name + "\n" + alldefines 
184              fbody += ev._LLfuncstr 
185  
 
186              if self.funcspec.auxfns: 
187                  fbody_parsed = addArgToCalls(fbody, self.funcspec.auxfns.keys(), "p_") 
188              else: 
189                  fbody_parsed = fbody 
190  
 
191              evbody = "\n % Function definition\n" + fbody_parsed + "\n\n\n" 
192              allevs = evsig + evbody 
193              allfilestr += allevs 
194  
 
195          return allfilestr

196  
 


197 -    def _prepareSetFileContents(self):


198          allfilestr = "" 
199  
 
200          topstr = "function a = set(ain, varargin)\n" 
201          commentstr = "% Set method for model " + self.name + "\n% Generated by PyDSTool for ADMC++ target\n\n" 
202  
 
203          bodystr = "if nargin == 1\n" \
 
204                    + "\t % Show input fields\n" \
 
205                    + "\t todisp = structsub(struct(ain), ain.protectedfields{:});\n" \
 
206                    + "\t disp(todisp);\n\t return \n end\n\n" \
 
207                    + "a = ain;\n\n" \
 
208                    + "if nargout < 1\n" + "\t warning('SET method invoked without output.');\n" \
 
209                    + "\t disp(' ');\n" + "\t return\n" + "end\n\n" \
 
210                    + "if rem(nargin-1, 2)\n" + "\t error('Wrong number of input arguments.');\n" + "end\n\n" \
 
211                    + "args = {varargin{:}};\n\n" \
 
212                    + "% Process input arguments\n" \
 
213                    + "while ~isempty(args)\n" + "\t nam = args{1};\n\t val = args{2};\n\t args = args(3:end);\n\n" \
 
214                    + "\t if any( strcmp( nam, a.privatefields ) )\n" + "\t\t warning(['Field ' nam ' is private -- unchanged.']);\n\n" \
 
215                    + "\t elseif isfield(struct(a), nam)\n\t\t eval(['a.' name '= val;']);\n\n" 
216  
 
217          parentfieldstr = "\t % Set parent field\n" + "\t else\n" \
 
218                           + "\t\t a." + str(self.vftype) + " = set(a." + str(self.vftype) + ", nam, val);\n\n" \
 
219                           + "\t end\n" + "end\n\n" + "varargout{1} = a;\n" 
220  
 
221          allfilestr = topstr + commentstr + bodystr + parentfieldstr 
222  
 
223          return allfilestr

224  
 


225 -    def _prepareGetFileContents(self):


226          allfilestr = "" 
227  
 
228          topstr = "function varargout = get(a, nam)\n" 
229          commentstr = "% Get method for model " + self.name + "\n% Generated by PyDSTool for ADMC++ target\n\n" 
230  
 
231          bodystr = "if nargin < 2\n" \
 
232                    + "\t % Show structure info\n" \
 
233                    + "\t disp(struct(a));\n\t return \n end\n\n" \
 
234                    + "if nargin > 2\n" + "\t warning('GET method invoked with more than two input arguments.');\n" \
 
235                    + "\t disp(' ');\n" + "end\n\n" \
 
236                    + "% Check for fields in this class; return values\n" \
 
237                    + "if any( strcmp( nam, fieldnames(a) ) )\n" + "\t v = getfield(struct(a), nam);\n\n" \
 
238   
239          parentfieldstr = "% Try parent field\n" + "else\n" \
 
240                           + "\t v = get(a." + str(self.vftype) + ", nam);\n\n" \
 
241                           + "end\n\n" + "varargout{1} = v;\n" 
242  
 
243          allfilestr = topstr + commentstr + bodystr + parentfieldstr 
244  
 
245          return allfilestr

246  
 
247  
 


248 -    def _prepareVfieldDefines(self):


249          pardefines = "" 
250          vardefines = "" 
251  
 
252          vnames = self._var_ixmap 
253          pnames = self.funcspec.pars 
254          pnames.sort() 
255  
 
256          for i in xrange(self.numpars): 
257              p = pnames[i] 
258              pardefines += "\t" + p + " = p_(" + str(i+1) + ");\n" 
259  
 
260          for i in xrange(self.dimension): 
261              v = vnames[i] 
262              vardefines += "\t" + v + " = x_(" + str(i+1) + ");\n" 
263  
 
264          alldefines = "\n% Parameter definitions\n\n" + pardefines \
 
265                       + "\n% Variable definitions\n\n" + vardefines 
266  
 
267          allfilestr = alldefines 
268  
 
269          return allfilestr

270  
 


271 -    def _prepareVfieldContents(self, vfdefines):


272          allfilestr = "" 
273  
 
274          topstr = "function [vf_, y_] = vfield(vf_, t_, x_, p_)\n" 
275          commentstr = "% Vector field definition for model " + self.name + "\n% Generated by PyDSTool for ADMC++ target\n\n" 
276  
 
277          bodystr = vfdefines 
278  
 
279          # Process the vector field stuff here
 
280  
 
281          # That's it, except we may need aux functions as well!
 
282          allfilestr = topstr + commentstr + bodystr + self.funcspec.spec[0] 
283  
 
284          return allfilestr

285  
 


286 -    def _prepareAuxContents(self):


287  
 
288          allfilestr = "" 
289  
 
290          if self.funcspec.auxfns: 
291              for finfo in self.funcspec.auxfns.values(): 
292                  fbody = finfo[0] 
293                  # subs _p into auxfn-to-auxfn calls (but not to the signature)
 
294                  fbody_parsed = addArgToCalls(fbody,
 
295                                          self.funcspec.auxfns.keys(),
 
296                                          "p_", notFirst=True) 
297  
 
298                  allfilestr += "\n" + fbody_parsed + "\n\n" 
299          # add auxiliary variables (shell of the function always present)
 
300          # add event functions
 
301          # allfilestr += self.funcspec.auxspec[0]
 
302  
 
303          return allfilestr

304  
 
305  
 
306  
 


307 -    def _prepareModelContents(self):


308          allfilestr = "" 
309          topstr = "function a = " + self.name + "(varargin)\n" 
310          commentstr = "% Vf object definition for model " + self.name + "\n% Generated by PyDSTool for ADMC++ target\n\n" 
311  
 
312          if len(self._eventNames) > 0: 
313              objectstr =  "\t\t vf = vfieldts('dimx'," + str(self.dimension) + ", 'eventdim', " \
 
314                          + str(len(self._eventNames)) + ");\n\n" 
315          else: 
316              objectstr = "\t\t vf = vfieldts('dimx'," + str(self.dimension) + ");\n\n" 
317  
 
318          bodystr = "nargs = nargin\n\n" + "switch nargs\n" \
 
319                    + "\t case 0\n" + "\t\t a.publicfields = {};\n" \
 
320                    + "\t\t a.protectedfields = {};\n" + "\t\t a.privatefields = {};\n\n" \
 
321                    + objectstr \
 
322                    + "\t\t a = class(a, '" + self.name + "', vf);\n\n" \
 
323                    + "\t case 1\n" + "\t\t if (isa(varargin{1}, '" + self.name + "'))\n" \
 
324                    + "\t\t\t a = varargin{1};\n" + "\t\t else\n" \
 
325                    + "\t\t\t error('Wrong argument type');\n" + "\t\t end\n\n" \
 
326                    + "\t otherwise\n" + "\t\t a = " + self.name + ";\n" \
 
327                    + "\t\t a = set(a, varargin{:});\n" + "end\n" 
328  
 
329          allfilestr = topstr + commentstr + bodystr 
330  
 
331          return allfilestr

332  
 


333 -    def _prepareICContents(self):


334          allfilestr = "" 
335          topstr = "function ics_ = " + self.name +"_ics()\n" 
336          commentstr = "% Initial conditions for model " + self.name + "\n% Generated by PyDSTool for ADMC++ target\n\n" 
337  
 
338          bodystr = "ics_ = [ ...\n" 
339          if self.initialconditions: 
340              icnames = self.initialconditions.keys() 
341              icnames.sort() 
342  
 
343              for i in range(len(icnames)-1): 
344                  if isnan(self.initialconditions[icnames[i]]): 
345                      val = str(0.0) 
346                  else: 
347                      val = str(self.initialconditions[icnames[i]]) 
348  
 
349                  bodystr += val + ", ... % " + icnames[i] + "\n" 
350  
 
351              if isnan(self.initialconditions[icnames[len(icnames)-1]]): 
352                  val = str(0.0) 
353              else: 
354                  val = self.initialconditions[icnames[len(icnames)-1]] 
355  
 
356              bodystr += val + " % " + icnames[len(icnames)-1] + " ...\n" 
357  
 
358          bodystr += "];\n" 
359  
 
360          allfilestr = topstr + commentstr + bodystr 
361  
 
362          return allfilestr

363  
 


364 -    def _prepareParamContents(self):


365          allfilestr = "" 
366          topstr = "function pars__ = " + self.name +"_params()\n" 
367          commentstr = "% Parameters for model " + self.name + "\n% Generated by PyDSTool for ADMC++ target\n\n" 
368  
 
369          bodystr = "pars_ = [ ...\n" 
370          if self.pars: 
371              pnames = self.pars.keys() 
372              pnames.sort() 
373  
 
374              for i in range(len(pnames)-1): 
375                  bodystr += str(self.pars[pnames[i]]) + ", ... % " + pnames[i] + "\n" 
376  
 
377              bodystr += str(self.pars[pnames[len(pnames)-1]]) + " % " + pnames[len(pnames)-1] + " ...\n" 
378  
 
379          bodystr += "];\n" 
380  
 
381          allfilestr = topstr + commentstr + bodystr 
382  
 
383          return allfilestr

384  
 
385  
 


386 -    def _prepareEventFuncStrings(self, vfdefines):


387          allevs = "" 
388  
 
389          if self._eventNames == []: 
390              numevs = 0 
391          else: 
392              numevs = len(self._eventNames) 
393          for ev in self._eventNames: 
394              ev = self.eventstruct.events[evname] 
395              evfullfn = "" 
396              assert isinstance(ev, MatlabEvent), ("ADMC can only accept matlab events") 
397              evsig = "function y_ = " + ev.name + "(vf_, t_, x_, p_)\n" 
398  
 
399              assert ev._LLfuncstr.index(';') > 1, ("Event function code error: "
 
400                                                    "Have you included a ';' character at the end of "
 
401                                                    "your function?") 
402              fbody = ev._LLfuncstr 
403              # NEED TO CHECK WHETHER THIS IS APPROPRIATELY DEFINED
 
404              # check for calls to user-defined functions and add hidden p_ argument
 
405              if self.funcspec.auxfns: 
406                  fbody_parsed = addArgToCalls(fbody, self.funcspec.auxfns.keys(), "p_") 
407                  if 'initcond' in self.funcspec.auxfns: 
408                      fbody_parsed = wrapArgInCall(fbody_parsed, 'initcond', ' ') 
409  
 
410              else: 
411                  fbody_parsed = fbody 
412  
 
413              allevs += evsig + vfdefines + fbody_parsed + "\n\n" 
414  
 
415              return allevs

416  
 
417  
 
418  #    def _prepareAuxFuncStrings(self, vfdefines):
 
419  #        allaux =
 
420  
 
421  
 


422 -    def makeLib(self, libsources=[], libdirs=[], include=[]):


423          """makeLib calls makeLibSource and then the compileLib method.
 
424          To postpone compilation of the source to a DLL, call makelibsource()
 
425          separately.""" 
426          self.makeLibSource()

427  
 
428  
 


429 -    def makeLibSource(self):


430          """makeLibSource generates the MATLAB source for the vector field specification.
 
431          It should be called only once per vector field.""" 
432  
 
433          # Make vector field (and event) file for compilation
 
434          # This sets the field self._eventNames
 
435          self._prepareEventSpecs() 
436  
 
437          # Write the model.m file
 
438          allfilestr = self._prepareModelContents() 
439          modelfile = os.path.join(self._target_dir, self._model_file) 
440          try: 
441              file = open(modelfile, 'w') 
442              file.write(allfilestr) 
443              file.close() 
444          except IOError, e: 
445              print "Error opening file "+self._model_file+" for writing" 
446              raise IOError, e 
447  
 
448          # Write the events.m file
 
449          if len(self._eventNames) > 0: 
450              allfilestr = self._prepareEventsFileContents() + self._prepareAuxContents() 
451              eventsfile = os.path.join(self._target_dir, self._events_file) 
452              try: 
453                  file = open(eventsfile, 'w') 
454                  file.write(allfilestr) 
455                  file.close() 
456              except IOError, e: 
457                  print "Error opening file "+self._events_file+" for writing" 
458                  raise IOError, e 
459  
 
460  
 
461          # Write the initialconditions.m file
 
462          allfilestr = self._prepareICContents() 
463          icfile = os.path.join(self._target_dir, self._ic_file) 
464          try: 
465              file = open(icfile, 'w') 
466              file.write(allfilestr) 
467              file.close() 
468          except IOError, e: 
469              print "Error opening file "+self._ic_file+" for writing" 
470              raise IOError, e 
471  
 
472          # Write the pars.m file
 
473          allfilestr = self._prepareParamContents() 
474          paramfile = os.path.join(self._target_dir, self._param_file) 
475          try: 
476              file = open(paramfile, 'w') 
477              file.write(allfilestr) 
478              file.close() 
479          except IOError, e: 
480              print "Error opening file "+self._param_file+" for writing" 
481              raise IOError, e 
482  
 
483          # Write the get.m file
 
484          allfilestr = self._prepareGetFileContents() 
485          getfile = os.path.join(self._target_dir, self._get_file) 
486          try: 
487              file = open(getfile, 'w') 
488              file.write(allfilestr) 
489              file.close() 
490          except IOError, e: 
491              print "Error opening file "+self._get_file+" for writing" 
492              raise IOError, e 
493  
 
494          # Write the set.m file
 
495          allfilestr = self._prepareSetFileContents() 
496          setfile = os.path.join(self._target_dir, self._set_file) 
497          try: 
498              file = open(setfile, 'w') 
499              file.write(allfilestr) 
500              file.close() 
501          except IOError, e: 
502              print "Error opening file "+self._set_file+" for writing" 
503              raise IOError, e 
504  
 
505          # Write the vfield.m file
 
506  #        vfdefines = self._prepareVfieldDefines()
 
507  #        allfilestr = self._prepareVfieldContents(vfdefines)
 
508          allfilestr = self.funcspec.spec[0] + self._prepareAuxContents() 
509          vffile = os.path.join(self._target_dir, self._vfield_file) 
510          try: 
511              file = open(vffile, 'w') 
512              file.write(allfilestr) 
513              file.close() 
514          except IOError, e: 
515              print "Error opening file "+self._vfield_file+" for writing" 
516              raise IOError, e

517  
 
518  
 
519      # We have omitted methods: RHS, compute, etc. because this
 
520      # class is intended solely to generate code for ADMC++, not do any integrations
 
521      # etc.
 
522  
 


523 -    def __del__(self):


524          ODEsystem.__del__(self)

525  
 
526  
 
527  # Register this Generator with the database
 
528  
 
529  symbolMapDict = {} 
530  # in future, provide appropriate mappings for libraries math,
 
531  # random, etc. (for now it's left to FuncSpec)
 
532  theGenSpecHelper.add(ADMC_ODEsystem, symbolMapDict, 'matlab') 
533
```

  


| Home | Trees | Indices | Help | | PyDSTool | | --- | |
| --- | --- | --- | --- | --- | --- |

|  |  |
| --- | --- |
| Generated by Epydoc 3.0.1 on Fri May 4 15:24:18 2012 | http://epydoc.sourceforge.net |
