# Supplementary material for: Hybrid Models and Biological Model Reduction with PyDSTool
Source: PLoS Comput Biol. 2012 Aug 9;8(8):e1002628. doi: 10.1371/journal.pcbi.1002628 (PMC3415397; doi:10.1371/journal.pcbi.1002628)
Supplement: Text S4 — Complete source code for the PyDSTool package (version 0.88.120504). Includes API documentation and help files linking to web pages. This file is identical to the current public release on Sourceforge.net. (ZIP) [file pcbi.1002628.s004.zip › PyDSTool/html/PyDSTool.Generator.ADMC_ODEsystem'.ADMC_ODEsystem-class.html]

xml version="1.0" encoding="ascii"?


PyDSTool.Generator.ADMC\_ODEsystem'.ADMC\_ODEsystem


| Home | Trees | Indices | Help | | PyDSTool | | --- | |
| --- | --- | --- | --- | --- | --- |

|  |  |  |  |
| --- | --- | --- | --- |
| Package PyDSTool :: Package Generator :: Module ADMC\_ODEsystem' :: Class ADMC\_ODEsystem | |  | | --- | | [hide private] | | [frames] | no frames] | |

# Class ADMC\_ODEsystem

source code

```
           object --+            
                    |            
baseclasses.Generator --+        
                        |        
       baseclasses.ctsGen --+    
                            |    
         ODEsystem'.ODEsystem --+
                                |
                               ADMC_ODEsystem
```

---

Wrapper for code generator for ADMC++32 and Matlab. Uses Matlab
functional specifications only.


|  |  |  |  |
| --- | --- | --- | --- |
| |  |  | | --- | --- | | Instance Methods | [hide private] | | |
|  | |  |  | | --- | --- | | \_\_del\_\_(self) | source code | |
|  | |  |  | | --- | --- | | \_\_init\_\_(self, kw)  Use the nobuild key to postpone building of the library, e.g. | source code | |
|  | |  |  | | --- | --- | | \_prepareAuxContents(self) | source code | |
|  | |  |  | | --- | --- | | \_prepareEventFuncStrings(self, vfdefines) | source code | |
|  | |  |  | | --- | --- | | \_prepareEventSpecs(self) | source code | |
|  | |  |  | | --- | --- | | \_prepareEventsFileContents(self) | source code | |
|  | |  |  | | --- | --- | | \_prepareGetFileContents(self) | source code | |
|  | |  |  | | --- | --- | | \_prepareICContents(self) | source code | |
|  | |  |  | | --- | --- | | \_prepareModelContents(self) | source code | |
|  | |  |  | | --- | --- | | \_prepareParamContents(self) | source code | |
|  | |  |  | | --- | --- | | \_prepareSetFileContents(self) | source code | |
|  | |  |  | | --- | --- | | \_prepareVfieldContents(self, vfdefines) | source code | |
|  | |  |  | | --- | --- | | \_prepareVfieldDefines(self) | source code | |
|  | |  |  | | --- | --- | | makeLib(self, libsources=`[``]`, libdirs=`[``]`, include=`[``]`)  makeLib calls makeLibSource and then the compileLib method. | source code | |
|  | |  |  | | --- | --- | | makeLibSource(self)  makeLibSource generates the MATLAB source for the vector field specification. | source code | |
| **Inherited from `ODEsystem'.ODEsystem`**: `AuxVars`, `Jacobian`, `JacobianP`, `Rhs`, `__getstate__`, `__setstate__`, `addMethods`, `checkInitialConditions`, `cleanupMemory`, `compute`, `haveJacobian`, `haveJacobian_pars`, `haveMass`, `prepDirection`, `set`, `validateICs`  **Inherited from `baseclasses.ctsGen`**: `validateSpec`  **Inherited from `baseclasses.Generator`**: `__copy__`, `__deepcopy__`, `__repr__`, `__str__`, `addEvtPars`, `checkArgs`, `contains`, `get`, `getEventTimes`, `getEvents`, `info`, `query`, `resetEventTimes`, `resetEvents`, `setEventICs`, `showAuxFnSpec`, `showAuxSpec`, `showEventSpec`, `showSpec`  **Inherited from `baseclasses.Generator`** (private): `_addEvents`, `_auxfn_getindex`, `_auxfn_globalindepvar`, `_auxfn_heav`, `_auxfn_if`, `_auxfn_initcond`, `_generate_ixmaps`, `_infostr`, `_kw_process_algparams`, `_kw_process_allvars`, `_kw_process_dispatch`, `_kw_process_events`, `_kw_process_fnspecs`, `_kw_process_ics`, `_kw_process_ignorespecial`, `_kw_process_inputs`, `_kw_process_pars`, `_kw_process_pdomain`, `_kw_process_reuseterms`, `_kw_process_system`, `_kw_process_target`, `_kw_process_tdata`, `_kw_process_tdomain`, `_kw_process_tstep`, `_kw_process_ttype`, `_kw_process_varspecs`, `_kw_process_vfcodeinserts`, `_kw_process_xdomain`, `_kw_process_xtype`, `_makeBoundsEvents`, `_register`, `_set_for_hybrid_DS`  **Inherited from `object`**: `__delattr__`, `__getattribute__`, `__hash__`, `__new__`, `__reduce__`, `__reduce_ex__`, `__setattr__` | |


|  |  |  |  |
| --- | --- | --- | --- |
| |  |  | | --- | --- | | Class Variables | [hide private] | | |
| **Inherited from `ODEsystem'.ODEsystem`** (private): `_needKeys`, `_optionalKeys`, `_validKeys`  **Inherited from `baseclasses.Generator`** (private): `_querykeys` | |


|  |  |  |  |
| --- | --- | --- | --- |
| |  |  | | --- | --- | | Properties | [hide private] | | |
| **Inherited from `object`**: `__class__` | |


|  |  |  |  |
| --- | --- | --- | --- |
| |  |  | | --- | --- | | Method Details | [hide private] | | |

|  |  |  |
| --- | --- | --- |
| |  |  | | --- | --- | | \_\_del\_\_(self)  *(Destructor)* | source code |   Overrides: baseclasses.Generator.\_\_del\_\_ |

|  |  |  |
| --- | --- | --- |
| |  |  | | --- | --- | | \_\_init\_\_(self, kw)  *(Constructor)* | source code |   Use the nobuild key to postpone building of the library, e.g. in order to provide additional build options to makeLibSource and compileLib methods or to make changes to the C code by hand. No build options can be specified otherwise.  Overrides: object.\_\_init\_\_ |

|  |  |  |
| --- | --- | --- |
| |  |  | | --- | --- | | makeLib(self, libsources=`[``]`, libdirs=`[``]`, include=`[``]`) | source code |   makeLib calls makeLibSource and then the compileLib method. To postpone compilation of the source to a DLL, call makelibsource() separately. |

|  |  |  |
| --- | --- | --- |
| |  |  | | --- | --- | | makeLibSource(self) | source code |   makeLibSource generates the MATLAB source for the vector field specification. It should be called only once per vector field. |

  


| Home | Trees | Indices | Help | | PyDSTool | | --- | |
| --- | --- | --- | --- | --- | --- |

|  |  |
| --- | --- |
| Generated by Epydoc 3.0.1 on Fri May 4 15:24:06 2012 | http://epydoc.sourceforge.net |
