# Supplementary material for: Hybrid Models and Biological Model Reduction with PyDSTool
Source: PLoS Comput Biol. 2012 Aug 9;8(8):e1002628. doi: 10.1371/journal.pcbi.1002628 (PMC3415397; doi:10.1371/journal.pcbi.1002628)
Supplement: Text S4 — Complete source code for the PyDSTool package (version 0.88.120504). Includes API documentation and help files linking to web pages. This file is identical to the current public release on Sourceforge.net. (ZIP) [file pcbi.1002628.s004.zip › PyDSTool/html/PyDSTool.Generator.allimports-pysrc.html]

xml version="1.0" encoding="ascii"?


PyDSTool.Generator.allimports


| Home | Trees | Indices | Help | | PyDSTool | | --- | |
| --- | --- | --- | --- | --- | --- |

|  |  |  |  |
| --- | --- | --- | --- |
| Package PyDSTool :: Package Generator :: Module allimports | |  | | --- | | [hide private] | | [frames] | no frames] | |

# Source Code for Module PyDSTool.Generator.allimports

```
 1  # PyDSTool imports
 
 2  
 
 3  # Imports of variables from these modules are not transferred to the caller
 
 4  # of this script, so those modules have to imported there specially.
 
 5  # Presently, this refers to utils and common
 
 6  from PyDSTool.errors import * 
 7  from PyDSTool.Interval import * 
 8  from PyDSTool.Points import * 
 9  from PyDSTool.Variable import * 
10  from PyDSTool.Trajectory import * 
11  from PyDSTool.FuncSpec import * 
12  from PyDSTool.Events import * 
13  from messagecodes import * 
14  from math import * 
15  import math, random, scipy 
16
```

  


| Home | Trees | Indices | Help | | PyDSTool | | --- | |
| --- | --- | --- | --- | --- | --- |

|  |  |
| --- | --- |
| Generated by Epydoc 3.0.1 on Fri May 4 15:24:20 2012 | http://epydoc.sourceforge.net |
