# Supplementary material for: Hybrid Models and Biological Model Reduction with PyDSTool
Source: PLoS Comput Biol. 2012 Aug 9;8(8):e1002628. doi: 10.1371/journal.pcbi.1002628 (PMC3415397; doi:10.1371/journal.pcbi.1002628)
Supplement: Text S4 — Complete source code for the PyDSTool package (version 0.88.120504). Includes API documentation and help files linking to web pages. This file is identical to the current public release on Sourceforge.net. (ZIP) [file pcbi.1002628.s004.zip › PyDSTool/html/PyDSTool.Generator.baseclasses-module.html]

xml version="1.0" encoding="ascii"?


PyDSTool.Generator.baseclasses


| Home | Trees | Indices | Help | | PyDSTool | | --- | |
| --- | --- | --- | --- | --- | --- |

|  |  |  |  |
| --- | --- | --- | --- |
| Package PyDSTool :: Package Generator :: Module baseclasses | |  | | --- | | [hide private] | | [frames] | no frames] | |

# Module baseclasses

source code


|  |  |  |  |
| --- | --- | --- | --- |
| |  |  | | --- | --- | | Classes | [hide private] | | |
|  | genDBClass  This class keeps a record of which non-Python Generators have been created in a session. |
|  | auxfn\_container  Auxiliary function interface for python user |
|  | ixmap |
|  | Generator  Trajectory Generator abstract class. |
|  | ctsGen  Abstract class for continuously-parameterized trajectory generators. |
|  | discGen  Abstract class for discretely-parameterized trajectory generators. |
|  | GenSpecInfoObj |
|  | GenSpecHelper  Generator specification helper - abstract class. |


|  |  |  |  |
| --- | --- | --- | --- |
| |  |  | | --- | --- | | Functions | [hide private] | | |
|  | |  |  | | --- | --- | | \_pollInputs(inputVarList, t, checklevel) | source code | |


|  |  |  |  |
| --- | --- | --- | --- |
| |  |  | | --- | --- | | Variables | [hide private] | | |
|  | HAVE\_PSYCO = `True` |
|  | genDB = `Generator internal database class: []` |
|  | theGenSpecHelper = `GenSpecHelper()` |
|  | \_1DimplicitSolveMethods = `['newton', 'bisect', 'steffe']` |
|  | \_all\_complex = `(<type 'complex'>, <type 'numpy.complexfloating...` |
|  | \_all\_float = `(<type 'float'>, <type 'numpy.floating'>, <type '...` |
|  | \_all\_int = `(<type 'int'>, <type 'numpy.integer'>, <type 'numpy...` |
|  | \_all\_numpy\_complex = `(<type 'numpy.complex128'>, <type 'numpy....` |
|  | \_all\_numpy\_float = `(<type 'numpy.float64'>, <type 'numpy.float...` |
|  | \_all\_numpy\_int = `(<type 'numpy.int32'>, <type 'numpy.int32'>, ...` |
|  | \_complex\_types = `(<type 'complex'>, <type 'numpy.complexfloati...` |
|  | \_float\_types = `(<type 'float'>, <type 'numpy.floating'>)` |
|  | \_implicitSolveMethods = `['newton', 'bisect', 'steffe', 'fsolve']` |
|  | \_int\_types = `(<type 'int'>, <type 'numpy.integer'>)` |
|  | \_num\_equivtype = `{<type 'float'>: <type 'numpy.float64'>, <typ...` |
|  | \_num\_maxmin = `{<type 'numpy.int32'>: [-2147483648, 2147483647]...` |
|  | \_num\_name2equivtypes = `{'float': (<type 'float'>, <type 'numpy...` |
|  | \_num\_name2type = `{'float': <type 'numpy.float64'>, 'int': <typ...` |
|  | \_num\_type2name = `{<type 'float'>: 'float', <type 'int'>: 'int'...` |
|  | \_num\_types = `(<type 'float'>, <type 'int'>, <type 'numpy.float...` |
|  | \_pytypefromtype = `{<type 'numpy.int32'>: <type 'int'>, <type '...` |
|  | \_real\_types = `(<type 'int'>, <type 'numpy.integer'>, <type 'fl...` |
|  | \_seq\_types = `(<type 'list'>, <type 'tuple'>, <type 'numpy.ndar...` |
|  | e = `2.71828182846` |
|  | pi = `3.14159265359` |


|  |  |  |  |
| --- | --- | --- | --- |
| |  |  | | --- | --- | | Variables Details | [hide private] | | |

|  |  |
| --- | --- |
| \_all\_complex   Value:  |  | | --- | | ``` (<type 'complex'>,  <type 'numpy.complexfloating'>,  <type 'numpy.complex128'>,  <type 'numpy.complex64'>,  <type 'numpy.complex128'>) ``` | |

|  |  |
| --- | --- |
| \_all\_float   Value:  |  | | --- | | ``` (<type 'float'>,  <type 'numpy.floating'>,  <type 'numpy.float64'>,  <type 'numpy.float32'>,  <type 'numpy.float64'>) ``` | |

|  |  |
| --- | --- |
| \_all\_int   Value:  |  | | --- | | ``` (<type 'int'>,  <type 'numpy.integer'>,  <type 'numpy.int32'>,  <type 'numpy.int32'>,  <type 'numpy.int8'>,  <type 'numpy.int16'>,  <type 'numpy.int32'>,  <type 'numpy.int64'>) ``` | |

|  |  |
| --- | --- |
| \_all\_numpy\_complex   Value:  |  | | --- | | ``` (<type 'numpy.complex128'>,  <type 'numpy.complex64'>,  <type 'numpy.complex128'>) ``` | |

|  |  |
| --- | --- |
| \_all\_numpy\_float   Value:  |  | | --- | | ``` (<type 'numpy.float64'>,  <type 'numpy.float32'>,  <type 'numpy.float64'>) ``` | |

|  |  |
| --- | --- |
| \_all\_numpy\_int   Value:  |  | | --- | | ``` (<type 'numpy.int32'>,  <type 'numpy.int32'>,  <type 'numpy.int8'>,  <type 'numpy.int16'>,  <type 'numpy.int32'>,  <type 'numpy.int64'>) ``` | |

|  |  |
| --- | --- |
| \_complex\_types   Value:  |  | | --- | | ``` (<type 'complex'>, <type 'numpy.complexfloating'>) ``` | |

|  |  |
| --- | --- |
| \_num\_equivtype   Value:  |  | | --- | | ``` {<type 'float'>: <type 'numpy.float64'>,  <type 'int'>: <type 'numpy.int32'>,  <type 'numpy.integer'>: <type 'numpy.int32'>,  <type 'numpy.floating'>: <type 'numpy.float64'>,  <type 'numpy.int8'>: <type 'numpy.int32'>,  <type 'numpy.int16'>: <type 'numpy.int32'>,  <type 'numpy.int32'>: <type 'numpy.int32'>,  <type 'numpy.int32'>: <type 'numpy.int32'>, ... ``` | |

|  |  |
| --- | --- |
| \_num\_maxmin   Value:  |  | | --- | | ``` {<type 'numpy.int32'>: [-2147483648, 2147483647],  <type 'numpy.float64'>: [-inf, inf]} ``` | |

|  |  |
| --- | --- |
| \_num\_name2equivtypes   Value:  |  | | --- | | ``` {'float': (<type 'float'>,            <type 'numpy.floating'>,            <type 'numpy.float64'>,            <type 'numpy.float32'>,            <type 'numpy.float64'>),  'int': (<type 'int'>,          <type 'numpy.integer'>,          <type 'numpy.int32'>, ... ``` | |

|  |  |
| --- | --- |
| \_num\_name2type   Value:  |  | | --- | | ``` {'float': <type 'numpy.float64'>, 'int': <type 'numpy.int32'>} ``` | |

|  |  |
| --- | --- |
| \_num\_type2name   Value:  |  | | --- | | ``` {<type 'float'>: 'float',  <type 'int'>: 'int',  <type 'numpy.integer'>: 'int',  <type 'numpy.floating'>: 'float',  <type 'numpy.int8'>: 'int',  <type 'numpy.int16'>: 'int',  <type 'numpy.int32'>: 'int',  <type 'numpy.int32'>: 'int', ... ``` | |

|  |  |
| --- | --- |
| \_num\_types   Value:  |  | | --- | | ``` (<type 'float'>,  <type 'int'>,  <type 'numpy.floating'>,  <type 'numpy.integer'>) ``` | |

|  |  |
| --- | --- |
| \_pytypefromtype   Value:  |  | | --- | | ``` {<type 'numpy.int32'>: <type 'int'>,  <type 'numpy.float64'>: <type 'float'>} ``` | |

|  |  |
| --- | --- |
| \_real\_types   Value:  |  | | --- | | ``` (<type 'int'>,  <type 'numpy.integer'>,  <type 'float'>,  <type 'numpy.floating'>) ``` | |

|  |  |
| --- | --- |
| \_seq\_types   Value:  |  | | --- | | ``` (<type 'list'>, <type 'tuple'>, <type 'numpy.ndarray'>) ``` | |

  


| Home | Trees | Indices | Help | | PyDSTool | | --- | |
| --- | --- | --- | --- | --- | --- |

|  |  |
| --- | --- |
| Generated by Epydoc 3.0.1 on Fri May 4 15:24:03 2012 | http://epydoc.sourceforge.net |
