# Supplementary material for: Hybrid Models and Biological Model Reduction with PyDSTool
Source: PLoS Comput Biol. 2012 Aug 9;8(8):e1002628. doi: 10.1371/journal.pcbi.1002628 (PMC3415397; doi:10.1371/journal.pcbi.1002628)
Supplement: Text S4 — Complete source code for the PyDSTool package (version 0.88.120504). Includes API documentation and help files linking to web pages. This file is identical to the current public release on Sourceforge.net. (ZIP) [file pcbi.1002628.s004.zip › PyDSTool/html/PyDSTool.Generator.baseclasses-pysrc.html]

xml version="1.0" encoding="ascii"?


PyDSTool.Generator.baseclasses


| Home | Trees | Indices | Help | | PyDSTool | | --- | |
| --- | --- | --- | --- | --- | --- |

|  |  |  |  |
| --- | --- | --- | --- |
| Package PyDSTool :: Package Generator :: Module baseclasses | |  | | --- | | [hide private] | | [frames] | no frames] | |

# Source Code for Module PyDSTool.Generator.baseclasses

```
   1  # Generator base classes: Generator, ctsGen, discGen
 
   2  from __future__ import division 
   3  
 
   4  from allimports import * 
   5  from PyDSTool.utils import * 
   6  from PyDSTool.common import * 
   7  from PyDSTool.Symbolic import ensureStrArgDict, Quantity, QuantSpec 
   8  from PyDSTool.Trajectory import Trajectory 
   9  from PyDSTool.parseUtils import symbolMapClass 
  10  from PyDSTool.Variable import Variable, iscontinuous 
  11  from PyDSTool.Points import Pointset 
  12  import PyDSTool.Events as Events 
  13  
 
  14  # Other imports
 
  15  from numpy import Inf, NaN, isfinite, sometrue, alltrue 
  16  import math, random 
  17  import os 
  18  from copy import copy, deepcopy 
  19  try: 
  20      # use pscyo JIT byte-compiler optimization, if available
 
  21      import psyco 
  22      HAVE_PSYCO = True 
  23  except ImportError: 
  24      HAVE_PSYCO = False 
  25  
 
  26  # -----------------------------------------------------------------------------
 
  27  
 
  28  __all__ = ['ctsGen', 'discGen', 'theGenSpecHelper', 'Generator',
 
  29             'genDB', 'auxfn_container', '_pollInputs'] 
  30  
 
  31  # -----------------------------------------------------------------------------
 
  32  
 


33 -class genDBClass(object):


34      """This class keeps a record of which non-Python Generators have been
 
  35      created in a session. A single global instance of this class is created,
 
  36      and prevents the user from re-using the name of a low-level
 
  37      DLL (such as that for a Dopri_ODEsystem vector field) unless the
 
  38      system is identical (according to its FuncSpec).""" 
  39  
 


40 -    def __init__(self):


41          self.database = {}

  42  
 


43 -    def check(self, gen):


44          """Look up generator instance and return Boolean of whether
 
  45          it is already registered. Only non-python based generators
 
  46          are registered, so all others will return False.
 
  47          """ 
  48          if gen.funcspec.targetlang == 'python': 
  49              # these do not need to be checked
 
  50              return False 
  51          try: 
  52              if gen._solver.rhs in self.database: 
  53                  entry = self.database[gen._solver.rhs] 
  54                  return className(gen) == entry['class'] \
 
  55                     and hash(gen.funcspec) == entry['hash'] 
  56              else: 
  57                  return False 
  58          except AttributeError: 
  59              # these do not need to be checked
 
  60              return False

  61  
 


62 -    def unregister(self, gen):


63          try: 
  64              del self.database[gen._solver.rhs] 
  65          except (AttributeError, KeyError): 
  66              # invalid type of generator or not present
 
  67              # so do nothing
 
  68              return

  69  
 


70 -    def register(self, gen):


71          if gen.funcspec.targetlang == 'python': 
  72              # these do not need to be checked
 
  73              return 
  74          # verify name of vector field in database
 
  75          try: 
  76              if gen._solver.rhs not in self.database: 
  77                  self.database[gen._solver.rhs] = {'name': gen.name,
 
  78                                                    'class': className(gen),
 
  79                                                    'hash': hash(gen.funcspec)} 
  80              else: 
  81                  if hash(gen.funcspec) != self.database[gen._solver.rhs]['hash']: 
  82                      raise PyDSTool_KeyError("Generator named %s already exists"%gen.name) 
  83                  # otherwise it's identical and can ignore
 
  84          except AttributeError: 
  85              # these do not need to be checked
 
  86              return

  87  
 


88 -    def __repr__(self):


89          s = "Generator internal database class: " 
  90          s += str(self.database.keys()) 
  91          return s

  92  
 
  93      __str__ = __repr__ 
  94  
 
  95  
 


96 -    def clearall(self):


97          self.database = {}

  98  
 
  99  
 
 100  # use single instance of nameResolver per session
 
 101  global genDB 
 102  genDB = genDBClass() 
 103  
 
 104  
 
 105  # ----------------------------------------------------------------------
 
 106  
 


107 -class auxfn_container(object):


108      """
 
 109      Auxiliary function interface for python user
 
 110      """ 


111 -    def __init__(self, genref):


112          self.genref = genref 
 113          self.map_ixs = ixmap

 114  
 


115 -    def __getitem__(self, k):


116          try: 
 117              return self.__dict__[k] 
 118          except KeyError: 
 119              raise KeyError("Function %s not present"%k)

 120  
 


121 -    def keys(self):


122          return [k for k in self.__dict__.keys() \
 
 123                  if k not in ['genref', 'map_ixs']]

 124  
 


125 -    def __contains__(self, k):


126          return k in self.keys()

 127  
 


128 -    def values(self):


129          return [self.__dict__[k] for k in self.__dict__.keys() \
 
 130                  if k not in ['genref', 'map_ixs']]

 131  
 


132 -    def items(self):


133          return zip(self.keys(), self.values())

 134  
 


135 -    def __repr__(self):


136          return "Aux functions: " + ", ".join(self.keys())

 137  
 
 138  
 


139 -class ixmap(dict):


140 -    def __init__(self, genref):


141          self.parixmap = {} 
 142          self.pars = genref.pars 
 143          i = genref.inputs.keys() 
 144          i.sort() 
 145          p = genref.pars.keys() 
 146          p.sort() 
 147          allnames = p + i 
 148          for pair in enumerate(allnames): 
 149              self.parixmap[pair[0]] = pair[1]

 150  
 


151 -    def __getitem__(self, k):


152          try: 
 153              return self.pars[self.parixmap[k]] 
 154          except: 
 155              raise "Cannot access external input values using ixmap class"

 156  
 


157 -    def __repr__(self):


158          return "Index mapping: " + str(self.parixmap)

 159  
 
 160  
 


161 -class Generator(object):


162      """
 
 163      Trajectory Generator abstract class.
 
 164      """ 
 165      # query keys for 'query' method
 
 166      _querykeys = ['pars', 'parameters', 'events', 'abseps',
 
 167                    'ics', 'initialconditions', 'vars', 'variables',
 
 168                    'auxvariables', 'auxvars', 'vardomains', 'pardomains'] 
 169      # initialization keyword keys
 
 170      _needKeys = ['name'] 
 171      _optionalKeys = ['globalt0', 'checklevel', 'model', 'abseps',
 
 172                                'eventPars', 'FScompatibleNames',
 
 173                                'FScompatibleNamesInv'] 
 174  
 


175 -    def __init__(self, kw):


176          # _funcreg stores function instances (and base classes) that are
 
 177          # passed from self.funcspec, as they are not defined in __main__.
 
 178          # A Generator object cannot be deep copied or
 
 179          # pickled without the additional __getstate__ and __setstate__
 
 180          # methods which know how to reconstruct these functions.
 
 181          self._funcreg = {} 
 182          try: 
 183              # sometimes certain keys are checked prior to calling this base
 
 184              # class so we don't want to tread on the feet of those __init__
 
 185              # methods
 
 186              dummy = self.foundKeys 
 187              # if this works then we won't reset to zero
 
 188          except: 
 189              self.foundKeys = 0 
 190          try: 
 191              self.name = kw['name'] 
 192              self.foundKeys += 1 
 193          except KeyError: 
 194              raise PyDSTool_KeyError("name must be supplied as a keyword in arguments") 
 195          # dimension value is set later
 
 196          self.dimension = None 
 197          # Regular Variable objects, and auxiliary variables.
 
 198          # These are callable and can even appear in
 
 199          # inputs. They are defined internally by the FuncSpec object.
 
 200          # These self.variables are generally placeholders containing domain
 
 201          # information only, except for the special Generator types LookupTable
 
 202          # and InterpTable. The actual variable contents from a computed
 
 203          # trajectory are created locally during computeTraj and immediately
 
 204          # exported to a Trajectory object, not affecting these variable objects.
 
 205          self.variables = {} 
 206          # initial conditions for each regular and auxiliary variable
 
 207          self.initialconditions = {} 
 208          # Generator's functional specification for variables, e.g.
 
 209          # right-hand side of ODE, or data lists for look-up table
 
 210          self.funcspec = None 
 211          # Generator's internal pars - for the functional specification
 
 212          self.pars = {} 
 213          # Place-holder for event structure, if used
 
 214          self.eventstruct = None 
 215          # Place-holder for latest trajectory events
 
 216          self.trajevents = None 
 217          # Autonomous external inputs (dictionary of callable Variables)
 
 218          self.inputs = {} 
 219          # Local independent variable interval and validity range (relative to global t0)
 
 220          self.indepvariable = None 
 221          # Sorted list of callable names
 
 222          self._callnames = [] 
 223          # Registry of object names and types
 
 224          self._registry = {} 
 225          # Internal flag for whether the generator is being used as part of a hybrid DS
 
 226          # (useful for control over high level event resetting between trajectory
 
 227          # segments when generators are reused) -- HybridModel class must keep
 
 228          # track of this and set using the _set_for_hybrid_DS method
 
 229          self._for_hybrid_DS = False 
 230          # Absolute tolerance for Interval endpoints
 
 231          if 'abseps' in kw: 
 232              self._abseps = kw['abseps'] 
 233              self.foundKeys += 1 
 234          else: 
 235              self._abseps = 1e-13 
 236          # `Checklevel` code determining response to uncertain float comparisons
 
 237          if 'checklevel' in kw: 
 238              if kw['checklevel'] not in range(4): 
 239                      raise ValueError('Invalid interval endpoint checking option') 
 240              self.checklevel = kw['checklevel'] 
 241              self.foundKeys += 1 
 242          else: 
 243              # default: no checking
 
 244              self.checklevel = 0 
 245  
 
 246          self.diagnostics = Diagnostics(errmessages, errorfields, warnmessages,
 
 247                                         warnfields, propagate_dict=self.inputs) 
 248  
 
 249          # global independent variable reference (for non-autonomous systems,
 
 250          # especially when a Generator is embedded in a hybrid Model object)
 
 251          if 'globalt0' in kw: 
 252              v = kw['globalt0'] 
 253              assert isinstance(v, _num_types), 'Incorrect type of globalt0' 
 254              self.globalt0 = v 
 255              self.foundKeys += 1 
 256          else: 
 257              self.globalt0 = 0 
 258  
 
 259          # If part of a Model, keep a reference to which one this
 
 260          # Generator belongs to
 
 261          if 'model' in kw: 
 262              assert isinstance(kw['model'], str), "model tag must be a string" 
 263              self._modeltag = kw['model'] 
 264              self.foundKeys += 1 
 265          else: 
 266              self._modeltag = None 
 267          if 'FScompatibleNames' in kw: 
 268              sm = kw['FScompatibleNames'] 
 269              if sm is None: 
 270                  sm = symbolMapClass() 
 271              self._FScompatibleNames = sm 
 272              self.foundKeys += 1 
 273          else: 
 274              self._FScompatibleNames = symbolMapClass() 
 275          if 'FScompatibleNamesInv' in kw: 
 276              sm = kw['FScompatibleNamesInv'] 
 277              if sm is None: 
 278                  sm = symbolMapClass() 
 279              self._FScompatibleNamesInv = sm 
 280              self.foundKeys += 1 
 281          else: 
 282              self._FScompatibleNamesInv = symbolMapClass() 
 283  
 
 284          # If there are eventPars, keep track of them (name list)
 
 285          self._eventPars = [] 
 286          if 'eventPars' in kw: 
 287              if isinstance(kw['eventPars'], list): 
 288                  self._eventPars = kw['eventPars'] 
 289              elif isinstance(kw['eventPars'], str): 
 290                  self._eventPars.append(kw['eventPars']) 
 291              self.foundKeys += 1 
 292              self._eventPars = self._FScompatibleNames(self._eventPars) 
 293          # Indicator of whether a trajectory has been successfully computed
 
 294          self.defined = False

 295  
 
 296  
 


297 -    def addEvtPars(self, eventPars):


298          """Register parameter names as event specific parameters.""" 
 299          if isinstance(eventPars, list): 
 300              self._eventPars.extend(self._FScompatibleNames(eventPars)) 
 301          elif isinstance(eventPars, str): 
 302              self._eventPars.append(self._FScompatibleNames(eventPars))

 303  
 


304 -    def getEvents(self, evnames=None, asGlobalTime=True):


305          """Produce dictionary of pointsets of all flagged events' independent
 
 306          and dependent variable values, for each event (whether terminal or not).
 
 307          Times will be globalized if optional asGlobalTime argument is True
 
 308          (default behavior). If a single event name is passed, only the pointset
 
 309          is returned (not a dictionary).
 
 310  
 
 311          evnames may be a singleton string or list of strings, or left blank to
 
 312          return data for all events.
 
 313  
 
 314          The events are not guaranteed to be ordered by the value of the
 
 315          independent variable.
 
 316          """ 
 317          compat_evnames = self._FScompatibleNamesInv(self.trajevents.keys()) 
 318          if evnames is None: 
 319              evnames = compat_evnames 
 320          if asGlobalTime: 
 321              t_offset = self.globalt0 
 322          else: 
 323              t_offset = 0 
 324          if isinstance(evnames, str): 
 325              # singleton
 
 326              assert evnames in compat_evnames, "Invalid event name provided: %s"%evnames 
 327              try: 
 328                  result = self.trajevents[self._FScompatibleNames(evnames)] 
 329              except AttributeError: 
 330                  # empty pointset
 
 331                  return None 
 332              else: 
 333                  result.indepvararray += t_offset 
 334                  return result 
 335          else: 
 336              # assume a sequence of strings
 
 337              assert all([ev in compat_evnames for ev in evnames]), \
 
 338                     "Invalid event name(s) provided: %s"%str(evnames) 
 339              result = {} 
 340              for (evname, evptset) in self.trajevents.iteritems(): 
 341                  compat_evname = self._FScompatibleNamesInv(evname) 
 342                  if compat_evname not in evnames: 
 343                      continue 
 344                  result[compat_evname] = copy(evptset) 
 345                  try: 
 346                      result[compat_evname].indepvararray += t_offset 
 347                  except AttributeError: 
 348                      # empty pointset
 
 349                      pass 
 350              return result

 351  
 


352 -    def getEventTimes(self, evnames=None, asGlobalTime=True):


353          """Produce dictionary of lists of all flagged events' independent
 
 354          variable values, for each event (whether terminal or not).
 
 355          Times will be globalized if optional asGlobalTime argument is True
 
 356          (default behavior). If a single event name is passed, only the pointset
 
 357          is returned (not a dictionary).
 
 358  
 
 359          evnames may be a singleton string or list of strings, or left blank to
 
 360          return data for all events.
 
 361  
 
 362          The events are guaranteed to be ordered by the value of the
 
 363          independent variable.
 
 364          """ 
 365          result = {} 
 366          if asGlobalTime: 
 367              t_offset = self.globalt0 
 368          else: 
 369              t_offset = 0 
 370          compat_evnames = self._FScompatibleNamesInv(self.trajevents.keys()) 
 371          if evnames is None: 
 372              evnames = compat_evnames 
 373          if isinstance(evnames, str): 
 374              # singleton
 
 375              assert evnames in compat_evnames, "Invalid event name provided: %s"%evnames 
 376              try: 
 377                  return self.trajevents[self._FScompatibleNames(evnames)].indepvararray \
 
 378                                                 + t_offset 
 379              except AttributeError: 
 380                  # empty pointset
 
 381                  return [] 
 382          else: 
 383              # assume a sequence of strings
 
 384              assert all([ev in compat_evnames for ev in evnames]), \
 
 385                     "Invalid event name(s) provided: %s"%str(evnames) 
 386              for (evname, evptset) in self.trajevents.iteritems(): 
 387                  compat_evname = self._FScompatibleNamesInv(evname) 
 388                  if compat_evname not in compat_evnames: 
 389                      continue 
 390                  try: 
 391                      result[compat_evname] = evptset.indepvararray + t_offset 
 392                  except AttributeError: 
 393                      # empty pointset
 
 394                      result[compat_evname] = [] 
 395              return result

 396  
 
 397  
 


398 -    def query(self, querykey=''):


399          """Return info about Generator set-up.
 
 400          Valid query key: 'pars', 'parameters', 'pardomains', 'events',
 
 401           'ics', 'initialconditions', 'vars', 'variables',
 
 402           'auxvars', 'auxvariables', 'vardomains'
 
 403           """ 
 404          assert isinstance(querykey, str), \
 
 405                         ("Query argument must be a single string") 
 406          if querykey not in self._querykeys: 
 407              print 'Valid query keys are:', self._querykeys 
 408              print "('events' key only queries model-level events, not those" 
 409              print " inside sub-models)" 
 410              raise ValueError('Query key '+querykey+' is not valid') 
 411          if querykey in ['pars', 'parameters']: 
 412              result = self._FScompatibleNamesInv(self.pars) 
 413          elif querykey in ['ics', 'initialconditions']: 
 414              try: 
 415                  result = self._FScompatibleNamesInv(self.initialconditions) 
 416              except AttributeError: 
 417                  result = None 
 418          elif querykey == 'events': 
 419              result = self.eventstruct.events 
 420          elif querykey in ['vars', 'variables']: 
 421              result = self._FScompatibleNamesInv(self.funcspec.vars) 
 422          elif querykey in ['auxvars', 'auxvariables']: 
 423              result = self._FScompatibleNamesInv(self.funcspec.auxvars) 
 424          elif querykey == 'vardomains': 
 425              result = {} 
 426              for varname, var in self.variables.iteritems(): 
 427                  result[self._FScompatibleNamesInv(varname)] = \
 
 428                                 var.depdomain 
 429          elif querykey == 'pardomains': 
 430              result = {} 
 431              for parname, pardom in self.parameterDomains.iteritems(): 
 432                  result[self._FScompatibleNamesInv(parname)] = \
 
 433                                 pardom 
 434          elif querykey == 'abseps': 
 435              result = self._abseps 
 436          return result

 437  
 


438 -    def get(self, key):


439          """For API compatibility with ModelInterface: get will make a copy of
 
 440          the key and pass it through the inverse FuncSpec-compatible name map.
 
 441          """ 
 442          return self._FScompatibleNamesInv(getattr(self, key))

 443  
 
 444  
 


445 -    def haveJacobian(self):


446          """Default method. Can be overridden by subclasses.""" 
 447          return False

 448  
 
 449  
 


450 -    def haveJacobian_pars(self):


451          """Default method. Can be overridden by subclasses.""" 
 452          return False

 453  
 
 454  
 


455 -    def info(self, verbose=1):


456          print self._infostr(verbose)

 457  
 
 458  
 


459 -    def _kw_process_dispatch(self, keys, kw):


460          # compile funcspec arguments by processing init keys
 
 461          # make ignorespecial initially empty so that it can safely be
 
 462          # extended by its own dispatch method and by process_system
 
 463          fs_args = {'name': self.name,
 
 464                     'ignorespecial': []} 
 465          if 'varspecs' not in keys: 
 466              fs_args['varspecs'] = kw['varspecs'] 
 467          for key in keys: 
 468              f = getattr(self, '_kw_process_'+key) 
 469              # f can update fs_args in place
 
 470              f(kw, fs_args) 
 471          return fs_args

 472  
 


473 -    def _kw_process_varspecs(self, kw, fs_args):


474          if 'varspecs' in kw: 
 475              for varname, varspec in kw['varspecs'].items(): 
 476                  assert isinstance(varname, (str, QuantSpec, Quantity)), "Invalid type for Variable name: %s"%str(varname) 
 477                  assert isinstance(varspec, (str, QuantSpec, Quantity)), "Invalid type for Variable %s's specification"%varname 
 478              self.foundKeys += 1 
 479              fs_args['varspecs'] = \
 
 480                      self._FScompatibleNames(ensureStrArgDict(kw['varspecs'])) 
 481          else: 
 482              raise PyDSTool_KeyError("Keyword 'varspecs' missing from "
 
 483                                      "argument")

 484  
 


485 -    def _kw_process_tdomain(self, kw, fs_args):


486          if 'tdomain' in kw: 
 487              self.tdomain = kw['tdomain'] 
 488              if self.tdomain[0] >= self.tdomain[1]: 
 489                  print "Time domain specified: [%s, %s]"%(self.tdomain[0],
 
 490                                                           self.tdomain[1]) 
 491                  raise PyDSTool_ValueError("tdomain values must be in order of "
 
 492                                  "increasing size") 
 493              self.foundKeys += 1 
 494          else: 
 495              self.tdomain = [-Inf, Inf]

 496  
 


497 -    def _kw_process_ttype(self, kw, fs_args):


498          # e.g. for map system
 
 499          if 'ttype' in kw: 
 500              try: 
 501                  self.indepvartype = _num_equivtype[kw['ttype']] 
 502              except KeyError: 
 503                  raise TypeError('Invalid ttype: %s'%str(kw['ttype'])) 
 504              self.foundKeys += 1 
 505          else: 
 506              self.indepvartype = float

 507  
 


508 -    def _kw_process_tdata(self, kw, fs_args):


509          # set tdomain first
 
 510          if 'tdata' in kw: 
 511              self.tdata = kw['tdata'] 
 512              if self.tdata[0] >= self.tdata[1]: 
 513                  raise PyDSTool_ValueError("tdata values must be in order of "
 
 514                                            "increasing size") 
 515              # _tdata is made into list to be consistent with
 
 516              # other uses of it in other Generators...
 
 517              if self.tdomain[0] > self.tdata[0]: 
 518                  raise ValueError('tdata cannot be specified below smallest '\
 
 519                        'value in tdomain\n (possibly due to uncertain'\
 
 520                        'bounding)') 
 521              if self.tdomain[1] < self.tdata[1]: 
 522                  raise ValueError('tdata cannot be specified above largest '\
 
 523                        'value in tdomain\n (possibly due to uncertain '\
 
 524                        'bounding)') 
 525              self.foundKeys += 1 
 526          else: 
 527              self.tdata = self.tdomain  # default needed

 528  
 


529 -    def _kw_process_tstep(self, kw, fs_args):


530          # requires self.indepvartype (e.g. for map system)
 
 531          if 'tstep' in kw: 
 532              self.tstep = kw['tstep'] 
 533              if self.tstep > self.tdata[1]-self.tdata[0]: 
 534                  raise PyDSTool_ValueError('tstep too large') 
 535              if compareNumTypes(self.indepvartype, _all_int) and round(self.tstep) != self.tstep: 
 536                  raise PyDSTool_ValueError('tstep must be an integer for integer ttype') 
 537              self.foundKeys += 1 
 538          else: 
 539              if compareNumTypes(self.indepvartype, _all_int): 
 540                  # default to 1 for integer types
 
 541                  self.tstep = 1 
 542              else: 
 543                  # no reasonable default - so raise error
 
 544                  raise PyDSTool_KeyError('tstep key needed for float ttype')

 545  
 


546 -    def _kw_process_inputs(self, kw, fs_args):


547          if 'inputs' in kw: 
 548              inputs = copy(kw['inputs']) 
 549              if isinstance(inputs, Trajectory): 
 550                  # extract the variables
 
 551                  self.inputs.update(self._FScompatibleNames(inputs.variables)) 
 552              elif isinstance(inputs, Variable): 
 553                  self.inputs.update({self._FScompatibleNames(inputs.name): \
 
 554                                      inputs}) 
 555              elif isinstance(inputs, Pointset): 
 556                  # turn into Variables with linear interpoolation between
 
 557                  # independent variable values
 
 558                  for n in inputs.coordnames: 
 559                      x_array = inputs[n] 
 560                      nFS = self._FScompatibleNames(n) 
 561                      self.inputs[nFS] = \
 
 562                          Variable(interp1d(inputs.indepvararray,
 
 563                                                         x_array), 't',
 
 564                                           Interval(nFS, float, extent(x_array),
 
 565                                                    abseps=self._abseps),
 
 566                                           name=n)  # keep original name here 
 567              elif isinstance(inputs, dict): 
 568                  self.inputs.update(self._FScompatibleNames(inputs)) 
 569                  # ensure values are Variables or Pointsets
 
 570                  for k, v in self.inputs.iteritems(): 
 571                      if not isinstance(v, Variable): 
 572                          try: 
 573                              self.inputs[k]=Variable(v) 
 574                          except: 
 575                              raise TypeError("Invalid specification of inputs") 
 576              else: 
 577                  raise TypeError("Invalid specification of inputs") 
 578              self._register(self.inputs) 
 579              self.foundKeys += 1 
 580              # only signal that _extInputsChanged if there are actually some
 
 581              # defined, e.g. inputs may be formally present in the keys but in
 
 582              # fact unused
 
 583              self._extInputsChanged = (self.inputs != {}) 
 584              fs_args['inputs'] = self.inputs.keys() 
 585          else: 
 586              self._extInputsChanged = False

 587  
 


588 -    def _kw_process_ics(self, kw, fs_args):


589          if 'ics' in kw: 
 590              self._xdatadict = {} 
 591              for k, v in dict(kw['ics']).iteritems(): 
 592                  self._xdatadict[self._FScompatibleNames(str(k))] = ensurefloat(v) 
 593              self.initialconditions = self._xdatadict.copy() 
 594              unspecd = remain(self._xdatadict.keys(), fs_args['varspecs'].keys()) 
 595              if unspecd != []: 
 596                  # ics were declared for variables not in varspecs
 
 597                  raise ValueError("Missing varspec entries for declared ICs: " + str(unspecd)) 
 598              for name in remain(fs_args['varspecs'].keys(),
 
 599                                 self._xdatadict.keys()): 
 600                  self.initialconditions[name] = NaN 
 601              self.foundKeys += 1 
 602          else: 
 603              self._xdatadict = {} 
 604              for name in fs_args['varspecs']: 
 605                  self.initialconditions[name] = NaN

 606  
 


607 -    def _kw_process_allvars(self, kw, fs_args):


608          if 'auxvars' in kw: 
 609              assert 'vars' not in kw, ("Cannot use both 'auxvars' and 'vars' "
 
 610                                        "keywords") 
 611              if isinstance(kw['auxvars'], list): 
 612                  auxvars = self._FScompatibleNames([str(v) for v in kw['auxvars']]) 
 613              else: 
 614                  auxvars = self._FScompatibleNames([str(kw['auxvars'])]) 
 615              vars = remain(fs_args['varspecs'].keys(), auxvars) 
 616              self.foundKeys += 1 
 617          elif 'vars' in kw: 
 618              assert 'auxvars' not in kw, \
 
 619                     "Cannot use both 'auxvars' and 'vars' keywords" 
 620              if isinstance(kw['vars'], list): 
 621                  vars = self._FScompatibleNames([str(v) for v in kw['vars']]) 
 622              else: 
 623                  vars = self._FScompatibleNames([str(kw['vars'])]) 
 624              auxvars = remain(fs_args['varspecs'].keys(), vars) 
 625              self.foundKeys += 1 
 626          else: 
 627              # default is that all are considered regular vars
 
 628              auxvars = [] 
 629              vars = fs_args['varspecs'].keys() 
 630          fs_args['vars'] = vars 
 631          self.dimension = len(vars) 
 632          if auxvars != []: 
 633              fs_args['auxvars'] = auxvars

 634  
 


635 -    def _kw_process_xtype(self, kw, fs_args):


636          # requires varspecs to have been set
 
 637          # default types are float
 
 638          self.xtype = {} 
 639          if 'xtype' in kw: 
 640              xts = kw['xtype'] 
 641              for name_temp, xt in dict(xts).iteritems(): 
 642                  if compareNumTypes(xt, _all_int): 
 643                      xt_actual = int 
 644                  elif compareNumTypes(xt, _all_float): 
 645                      xt_actual = float 
 646                  else: 
 647                      raise TypeError("Invalid variable type %s"%str(xt)) 
 648                  name = self._FScompatibleNames(name_temp) 
 649                  if name[-1] == ']': 
 650                      # for macro -- FuncSpec.py will double check for correct syntax
 
 651                      base = name[:name.index('[')] 
 652                      # pull out everything in parentheses
 
 653                      for_spec = fs_args['varspecs'][name][4:-1].replace(' ', '').split(',') 
 654                      for name_i in range(int(for_spec[1]), int(for_spec[2])+1): 
 655                          self.xtype[base+str(name_i)] = xt_actual 
 656                  else: 
 657                      self.xtype[name] = xt_actual 
 658              for name in remain(fs_args['varspecs'].keys(), self.xtype.keys()): 
 659                  self.xtype[name] = float 
 660              self.foundKeys += 1 
 661          else: 
 662              for name in fs_args['varspecs']: 
 663                  if name[-1] == ']': 
 664                      # for macro -- FuncSpec.py will double check for correct syntax
 
 665                      base = name[:name.index('[')] 
 666                      # pull out everything in parentheses
 
 667                      for_spec = fs_args['varspecs'][name][4:-1].replace(' ', '').split(',') 
 668                      for name_i in range(int(for_spec[1]), int(for_spec[2])+1): 
 669                          self.xtype[base+str(name_i)] = float 
 670                  else: 
 671                      self.xtype[name] = float

 672  
 


673 -    def _kw_process_xdomain(self, kw, fs_args):


674          if 'xdomain' in kw: 
 675              self.xdomain = {} 
 676              for k, v in dict(kw['xdomain']).iteritems(): 
 677                  name = self._FScompatibleNames(str(k)) 
 678                  if isinstance(v, _seq_types): 
 679                      assert len(v) == 2, \
 
 680                             "Invalid size of domain specification for "+name 
 681                      if v[0] >= v[1]: 
 682                          raise PyDSTool_ValueError('xdomain values must be in'
 
 683                                                    'order of increasing size') 
 684                      else: 
 685                          self.xdomain[name] = copy(v) 
 686                  elif isinstance(v, _num_types): 
 687                      self.xdomain[name] = [v, v] 
 688                  else: 
 689                      raise PyDSTool_TypeError('Invalid type for xdomain spec'
 
 690                                               ' '+name) 
 691              for name in remain(fs_args['varspecs'].keys(), self.xdomain.keys()): 
 692                  if name[-1] == ']': 
 693                      # for macro -- FuncSpec.py will double check for correct syntax
 
 694                      base = name[:name.index('[')] 
 695                      # pull out everything in parentheses
 
 696                      for_spec = fs_args['varspecs'][name][4:-1].replace(' ', '').split(',') 
 697                      for name_i in range(int(for_spec[1]), int(for_spec[2])+1): 
 698                          self.xdomain[base+str(name_i)] = [-Inf, Inf] 
 699                  else: 
 700                      self.xdomain[name] = [-Inf, Inf] 
 701              self.foundKeys += 1 
 702          else: 
 703              self.xdomain = {} 
 704              for name in fs_args['varspecs']: 
 705                  if name[-1] == ']': 
 706                      # for macro -- FuncSpec.py will double check for correct syntax
 
 707                      base = name[:name.index('[')] 
 708                      # pull out everything in parentheses
 
 709                      for_spec = fs_args['varspecs'][name][4:-1].replace(' ', '').split(',') 
 710                      for name_i in range(int(for_spec[1]), int(for_spec[2])+1): 
 711                          self.xdomain[base+str(name_i)] = [-Inf, Inf] 
 712                  else: 
 713                      self.xdomain[name] = [-Inf, Inf]

 714  
 


715 -    def _kw_process_reuseterms(self, kw, fs_args):


716          if 'reuseterms' in kw: 
 717              self.foundKeys += 1 
 718              fs_args['reuseterms'] = kw['reuseterms']

 719  
 


720 -    def _kw_process_ignorespecial(self, kw, fs_args):


721          if 'ignorespecial' in kw: 
 722              self.foundKeys += 1 
 723              fs_args['ignorespecial'].extend(kw['ignorespecial'])

 724  
 


725 -    def _kw_process_algparams(self, kw, fs_args):


726          if 'algparams' in kw: 
 727              self.algparams = copy(kw['algparams']) 
 728              self.foundKeys += 1 
 729          else: 
 730              self.algparams = {}

 731  
 


732 -    def _kw_process_pars(self, kw, fs_args):


733          if 'pars' in kw: 
 734              self.pars = {} 
 735              if isinstance(kw['pars'], list): 
 736                  # may be a list of symbolic definitions
 
 737                  for p in kw['pars']: 
 738                      try: 
 739                          self.pars[self._FScompatibleNames(p.name)] = p.tonumeric() 
 740                      except (AttributeError, TypeError): 
 741                          raise TypeError("Invalid parameter symbolic definition") 
 742              else: 
 743                  for k, v in dict(kw['pars']).iteritems(): 
 744                      self.pars[self._FScompatibleNames(str(k))] = ensurefloat(v) 
 745              fs_args['pars'] = self.pars.keys() 
 746              self._register(self.pars) 
 747              self.foundKeys += 1 
 748          self.numpars = len(self.pars)

 749  
 


750 -    def _kw_process_pdomain(self, kw, fs_args):


751          if 'pdomain' in kw: 
 752              if self.pars: 
 753                  self.pdomain = {} 
 754                  for k, v in dict(kw['pdomain']).iteritems(): 
 755                      assert len(v) == 2, \
 
 756                                 "Invalid size of domain specification for "+k 
 757                      self.pdomain[self._FScompatibleNames(str(k))] = v 
 758                  for name in self.pdomain: 
 759                      if self.pdomain[name][0] >= self.pdomain[name][1]: 
 760                          raise PyDSTool_ValueError('pdomain values must be in order of increasing size') 
 761                  for name in remain(self.pars.keys(), self.pdomain.keys()): 
 762                      self.pdomain[name] = [-Inf, Inf] 
 763                  self.foundKeys += 1 
 764              else: 
 765                  raise ValueError('Cannot specify pdomain because no pars declared') 
 766          else: 
 767              if self.pars: 
 768                  self.pdomain = {} 
 769                  for pname in self.pars: 
 770                      self.pdomain[pname] = [-Inf, Inf] 
 771          if self.pars: 
 772              self.parameterDomains = {} 
 773              for pname in self.pdomain: 
 774                  self.parameterDomains[pname] = Interval(pname, float,
 
 775                                                          self.pdomain[pname],
 
 776                                                          self._abseps) 
 777                  try: 
 778                      cval = self.parameterDomains[pname].contains(self.pars[pname]) 
 779                  except KeyError: 
 780                      raise ValueError("Parameter %s is missing a value"%pname) 
 781                  if self.checklevel < 3: 
 782                      if cval is not notcontained: 
 783                          if cval is uncertain and self.checklevel == 2: 
 784                              print 'Warning: Parameter value at bound' 
 785                      else: 
 786                          print self.pars[pname], "not in", self.parameterDomains[pname].get() 
 787                          raise PyDSTool_ValueError('Parameter %s: value out of bounds'%pname) 
 788                  else: 
 789                      if cval is uncertain: 
 790                          raise PyDSTool_UncertainValueError('Parameter %s: value at bound'%pname) 
 791                      elif cval is notcontained: 
 792                          raise PyDSTool_ValueError('Parameter %s: value out of bounds'%pname)

 793  
 


794 -    def _kw_process_fnspecs(self, kw, fs_args):


795          if 'fnspecs' in kw: 
 796              fs_args['fnspecs'] = ensureStrArgDict(kw['fnspecs']) 
 797              self.foundKeys += 1

 798  
 


799 -    def _kw_process_target(self, kw, fs_args):


800          fs_args['targetlang'] = theGenSpecHelper(self).lang 
 801          if 'compiler' in kw: 
 802              if fs_args['targetlang'] == 'python': 
 803                  print "Warning: redundant option 'compiler' for python target" 
 804              self._compiler = kw['compiler'] 
 805              self.foundKeys += 1 
 806          elif fs_args['targetlang'] != 'python': 
 807              osname = os.name 
 808              # os-specific defaults for C compiler
 
 809              if osname == 'nt': 
 810                  self._compiler = 'mingw32' 
 811              elif osname == 'mac': 
 812                  self._compiler = 'mwerks' 
 813              elif osname == 'posix' or osname == 'unix': 
 814                  self._compiler = 'unix' 
 815              elif osname == 'os2emx': 
 816                  self._compiler = 'emx' 
 817              else: 
 818                  self._compiler = '' 
 819          else: 
 820              self._compiler = ''

 821  
 


822 -    def _kw_process_vfcodeinserts(self, kw, fs_args):


823          if 'vfcodeinsert_start' in kw: 
 824              fs_args['codeinsert_start'] = kw['vfcodeinsert_start'] 
 825              self.foundKeys += 1 
 826          if 'vfcodeinsert_end' in kw: 
 827              fs_args['codeinsert_end'] = kw['vfcodeinsert_end'] 
 828              self.foundKeys += 1

 829  
 


830 -    def _kw_process_system(self, kw, fs_args):


831          # for python-based solvers, esp. map system
 
 832          if 'system' in kw: 
 833              self._solver = kw['system'] 
 834              try: 
 835                  fs_args['ignorespecial'].append(self._solver.name) 
 836              except: 
 837                  raise TypeError("Invalid solver system provided") 
 838              self.foundKeys += 1 
 839              if self.pars: 
 840                  # automatically pass par values on to embedded system
 
 841                  # when Rhs called
 
 842                  parlist = self.pars.keys() 
 843                  parstr = "".join(["'%s': %s, "%(parname,parname) \
 
 844                                    for parname in parlist]) 
 845              if 'codeinsert_start' in fs_args: 
 846                  fs_args['codeinsert_start'] = \
 
 847                      '    %s.set(pars={%s})\n'%(self._solver.name, parstr) \
 
 848                      + fs_args['codeinsert_start'] 
 849              else: 
 850                  fs_args['codeinsert_start'] = \
 
 851                      '    %s.set(pars={%s})\n'%(self._solver.name, parstr) 
 852          else: 
 853              self._solver = None

 854  
 
 855  
 


856 -    def _infostr(self, verbose=1):


857          """Return detailed information about the Generator
 
 858          specification.""" 
 859  
 
 860          if verbose == 0: 
 861              outputStr = "Generator "+self.name 
 862          else: 
 863              outputStr = '**************************************************' 
 864              outputStr += '\n           Generator  '+self.name 
 865              outputStr +='\n**************************************************' 
 866              outputStr +='\nType : ' + className(self) 
 867              outputStr +='\nIndependent variable interval: ' + str(self.indepvariable.depdomain) 
 868              outputStr +='\nGlobal t0 = ' + str(self.globalt0) 
 869              outputStr +='\nInterval endpoint check level = ' + str(self.checklevel) 
 870              outputStr +='\nDimension = ' + str(self.dimension) 
 871              outputStr +='\n' 
 872              if isinstance(self.funcspec, FuncSpec): 
 873                  outputStr += self.funcspec._infostr(verbose) 
 874          if verbose == 2: 
 875              outputStr += '\nVariables` validity intervals:' 
 876              for v in self.variables.values(): 
 877                  outputStr += '\n  ' + str(v.depdomain) 
 878              if self.eventstruct is not None: 
 879                  outputStr += '\nEvents defined:' 
 880                  outputStr += '\n  ' + str(self.eventstruct.events.keys()) 
 881          if self._modeltag is not None: 
 882              outputStr += '\nAssociated Model: ' + self._modeltag.name 
 883          if verbose > 0: 
 884              outputStr += '\n' 
 885          return outputStr

 886  
 
 887  
 


888 -    def showEventSpec(self):


889          if self.eventstruct is not None: 
 890              for evname, ev in self.eventstruct.events.iteritems(): 
 891                  print evname + ":\n" + ev._funcstr 
 892                  print "\n"

 893  
 
 894  
 


895 -    def showSpec(self):


896          print self.funcspec.spec[0]

 897  
 
 898  
 


899 -    def showAuxSpec(self):


900          print self.funcspec.auxspec[0]

 901  
 
 902  
 


903 -    def showAuxFnSpec(self, auxfnname=None):


904          if auxfnname is None: 
 905              retdict = {} 
 906              for aname, aspec in self.funcspec.auxfns.iteritems(): 
 907                  retdict[aname] = aspec[0] 
 908              info(retdict) 
 909          else: 
 910              try: 
 911                  print self.funcspec.auxfns[auxfnname][0] 
 912              except KeyError: 
 913                  raise NameError("Aux function %s not found"%auxfnname)

 914  
 
 915  
 


916 -    def __repr__(self):


917          return self._infostr(verbose=0)

 918  
 
 919  
 
 920      __str__ = __repr__ 
 921  
 
 922  
 


923 -    def validateSpec(self):


924          try: 
 925              assert self.dimension > 0 
 926              assert len(self.variables) == self.dimension 
 927              # don't assert self.pars because not all systems need them
 
 928              assert self.indepvariable.name == 't' 
 929              assert self.funcspec 
 930              assert self.checklevel in range(4) 
 931              #  check that all names in individual dicts are all in _registry
 
 932              if self.pars: 
 933                  for name in self.pars: 
 934                      assert isinstance(self.pars[name], _num_types) 
 935                      assert type(self.pars[name]) == self._registry[name] 
 936              if self.inputs: 
 937                  for subjectname, obj in self.inputs.iteritems(): 
 938                      # test for containment of input's interval in independent
 
 939                      # variable interval
 
 940                      # (use checklevel = 1 for this o/w could get errors)
 
 941                      assert self.contains(obj.indepdomain,
 
 942                                           self.indepvariable.indepdomain, 0) 
 943                      # test that types entered in registry are still correct
 
 944                      assert type(obj) == self._registry[subjectname] 
 945              for name in self.variables: 
 946                  assert self.variables[name].__class__ == self._registry[name] 
 947              dummy = self.indepvariable(self.tdata[0]) # exception if this call is ill-defined 
 948              # check consistency with FuncSpec type of self.funcspec
 
 949              # (unnecessary for dictionary version of FuncSpec)
 
 950              if isinstance(self.funcspec, FuncSpec): 
 951                  varnames = self.variables.keys() 
 952                  fsvars = self.funcspec.vars 
 953                  if len(varnames) > 1: 
 954                      varnames.sort() 
 955                      fsvars.sort() 
 956                      assert varnames == fsvars, ('Inconsistency with funcspec '
 
 957                                                  'variable names') 
 958                  else: 
 959                      assert varnames == fsvars 
 960                  parnames = self.pars.keys() 
 961                  fspars = self.funcspec.pars 
 962                  if len(parnames) > 1: 
 963                      parnames.sort() 
 964                      fspars.sort() 
 965                      assert parnames == fspars, ('Inconsistency with funcspec '
 
 966                                                  'parameter names') 
 967                  else: 
 968                      assert parnames == fspars 
 969                  if self.inputs: 
 970                      inputnames = self.inputs.keys() 
 971                      fsinputs = self.funcspec.inputs 
 972                      if len(inputnames) > 1: 
 973                          inputnames.sort() 
 974                          fsinputs.sort() 
 975                          assert inputnames == fsinputs, ('Inconsistency with funcspec'
 
 976                                                          ' input names') 
 977                      else: 
 978                          assert inputnames == fsinputs 
 979              else: 
 980                  assert len(self.funcspec) == self.dimension 
 981          except: 
 982              print 'Invalid system specification' 
 983              raise

 984  
 
 985  
 
 986      # call this after all expected keywords have been processed
 


987 -    def checkArgs(self, kw):


988          if len(kw) == self.foundKeys: 
 989              for name in self._needKeys: 
 990                  if name not in kw: 
 991                      raise PyDSTool_KeyError('Necessary key missing: ' + name) 
 992              for name in kw: 
 993                  if name not in self._needKeys + self._optionalKeys: 
 994                      raise PyDSTool_KeyError('Key name ' + name + ' is invalid') 
 995          else: 
 996              print 'Keywords supplied:\n\t' + str(kw.keys()) 
 997              print '# keywords found: ' + str(self.foundKeys) 
 998              print 'Needed:\n\t' + str(self._needKeys) 
 999              print 'Optional:\n\t' + str(self._optionalKeys) 
1000              raise PyDSTool_KeyError('Invalid keyword arguments for this class') 
1001          del self.foundKeys

1002  
 
1003  
 


1004 -    def _set_for_hybrid_DS(self, state):


1005          """Internal method for indicating whether this Generator is currently
 
1006          being used as part of a hybrid dybnamical system calculation""" 
1007          self._for_hybrid_DS = state

1008  
 
1009  
 


1010 -    def _register(self, items):


1011          """_register names and types of sub-system variables (including
 
1012          Generator variables), pars and external inputs.
 
1013  
 
1014          Names must be unique for the Generator.
 
1015          """ 
1016  
 
1017          if isinstance(items, dict): 
1018              # for parameter and variable dictionaries
 
1019              for name, v in items.iteritems(): 
1020                  if isinstance(self.funcspec, FuncSpec): 
1021                      assert name in self.funcspec.vars \
 
1022                         or name in self.funcspec.auxvars \
 
1023                         or name in self.funcspec.pars \
 
1024                         or name in self.funcspec.inputs, \
 
1025                         ("Generator = '"
 
1026                         +self.name+"': name "+name+" not found in "
 
1027                         "functional specification declaration") 
1028                  if name not in self._registry: 
1029                      self._registry[name] = type(v) 
1030                  else: 
1031                      raise ValueError('The name `' + name + '` of type `'
 
1032                                         + type(v).__name__ +
 
1033                                         '` already exists in the registry') 
1034                  if isinstance(v, Variable) and \
 
1035                     name in self.variables or name in self.inputs: 
1036                      self._callnames.append(name) 
1037              self._callnames.sort() 
1038          elif isinstance(items, Variable) and items.name == 't': 
1039              # for self.indepvariable
 
1040              if items.name not in self._registry: 
1041                  self._registry[items.name] = type(items) 
1042              else: 
1043                  raise ValueError('The reserved name `t` has already'
 
1044                                     ' been declared to the registry') 
1045          else: 
1046              raise TypeError('Expected dictionary or independent variable in '
 
1047                                'argument to _register()')

1048  
 


1049 -    def _kw_process_events(self, kw):


1050          # Only call once funcspec built
 
1051          #
 
1052          # Holder and interface for events
 
1053          self.eventstruct = EventStruct() 
1054          if 'enforcebounds' in kw: 
1055              if 'activatedbounds' in kw: 
1056                  ab = kw['activatedbounds'] 
1057                  self.foundKeys += 1 
1058              else: 
1059                  ab = None 
1060              if kw['enforcebounds']: 
1061                  self._makeBoundsEvents(precise=True, activatedbounds=ab) 
1062              self.foundKeys += 1 
1063          if 'events' in kw: 
1064              self._addEvents(kw['events']) 
1065              self.foundKeys += 1

1066  
 
1067  
 


1068 -    def _addEvents(self, evs):


1069          if isinstance(evs, list): 
1070              map(self.eventstruct.add, copy(evs)) 
1071          elif isinstance(evs, Event): 
1072              # singleton
 
1073              self.eventstruct.add(evs) 
1074          else: 
1075              raise TypeError('Unsupported type of argument for event '
 
1076                                'structure')

1077  
 
1078  
 


1079 -    def _makeBoundsEvents(self, precise=True, eventtol=1e-6,
 
1080                            activatedbounds=None):


1081          events = [] 
1082          # pars + vars (pars used only by PyCont during continuation)
 
1083          alldoms = copy(self.pdomain) 
1084          alldoms.update(self.xdomain) 
1085          if self._eventPars != []: 
1086              # This exclusion doesn't work at the moment.
 
1087              #            nonEvtPars = remain(self.funcspec.pars, self._eventPars)
 
1088              nonEvtPars = self.funcspec.pars 
1089          else: 
1090              nonEvtPars = self.funcspec.pars 
1091          allnames = self.funcspec.vars + nonEvtPars 
1092          if activatedbounds in (None,{}): 
1093              activatedbounds = {}.fromkeys(allnames, (False,False)) 
1094          for xname, xdom in alldoms.iteritems(): 
1095              if xname not in allnames: 
1096                  # don't make bound constraints for non-state variables
 
1097                  continue 
1098              xdlo = xdom[0] 
1099              xdhi = xdom[1] 
1100              evname = xname+"_domlo" 
1101              evargs = {'term': True,
 
1102                        'precise': precise,
 
1103                        'name': evname,
 
1104                        'eventtol': eventtol,
 
1105                        'eventdelay': eventtol*10,
 
1106                        'eventinterval': eventtol*20,
 
1107                        'xdomain': self.xdomain,
 
1108                        'pdomain': self.pdomain} 
1109              try: 
1110                  evargs['active'] = activatedbounds[xname][0] and isfinite(xdlo) 
1111              except KeyError: 
1112                  evargs['active'] = False 
1113              evstr = xname + '-' + 'getbound("%s", 0)'%xname 
1114              ev = Events.makeZeroCrossEvent(evstr, -1, evargs, [xname],
 
1115                                         targetlang=self.funcspec.targetlang,
 
1116                                         reuseterms=self.funcspec.reuseterms) 
1117              events.append(ev) 
1118              evname = xname+"_domhi" 
1119              evargs = {'term': True,
 
1120                        'precise': precise,
 
1121                        'name': evname,
 
1122                        'eventtol': eventtol,
 
1123                        'eventdelay': eventtol*10,
 
1124                        'eventinterval': eventtol*20,
 
1125                        'xdomain': self.xdomain,
 
1126                        'pdomain': self.pdomain} 
1127              try: 
1128                  evargs['active'] = activatedbounds[xname][1] and isfinite(xdhi) 
1129              except KeyError: 
1130                  evargs['active'] = False 
1131              evstr = xname + '-' + 'getbound("%s", 1)'%xname 
1132              ev = Events.makeZeroCrossEvent(evstr, 1, evargs, [xname],
 
1133                                         targetlang=self.funcspec.targetlang,
 
1134                                         reuseterms=self.funcspec.reuseterms) 
1135              events.append(ev) 
1136          if events != []: 
1137              self._addEvents(events)

1138  
 
1139  
 


1140 -    def set(self, **kw):


1141          """Set generic parameters.""" 
1142          # Note: globalt0 may be unused by many classes of Generator
 
1143          if len(kw) > 0: 
1144              if 'globalt0' in kw: 
1145                  self.globalt0 = kw['globalt0'] 
1146                  try: 
1147                      self.eventstruct.setglobalt0(self.globalt0) 
1148                  except AttributeError: 
1149                      # no events present
 
1150                      pass 
1151              if 'checklevel' in kw: 
1152                  self.checklevel = kw['checklevel'] 
1153                  if hasattr(self, 'algparams'): 
1154                      self.algparams['checkBounds'] = kw['checklevel'] 
1155              if 'abseps' in kw: 
1156                  self._abseps = kw['abseps'] 
1157              if remain(kw.keys(), ['globalt0', 'checklevel', 'abseps']) != []: 
1158                  raise PyDSTool_KeyError('Invalid keywords passed')

1159  
 
1160  
 


1161 -    def setEventICs(self, ics, gt0=0):


1162          """Set initialconditions attribute of all generator's events, in
 
1163          case event uses auxiliary functions that access this information.""" 
1164          try: 
1165              evs = self.eventstruct.events.values() 
1166          except AttributeError: 
1167              # no events present
 
1168              pass 
1169          else: 
1170              for ev in evs: 
1171                  ev.initialconditions = self._FScompatibleNames(ics) 
1172                  ev.globalt0 = gt0

1173  
 
1174  
 


1175 -    def resetEventTimes(self):


1176          try: 
1177              self.eventstruct.resetEvtimes() 
1178          except AttributeError: 
1179              # no events present
 
1180              pass

1181  
 


1182 -    def resetEvents(self, state=None):


1183          """Reset any high level (Python) events in Generator""" 
1184          try: 
1185              # time is OK to be 0 here, it will be overwritten before use anyway
 
1186              # e.g. by VODE or map system
 
1187              self.eventstruct.resetHighLevelEvents(0, state=state) 
1188          except AttributeError: 
1189              # no events present
 
1190              pass

1191  
 
1192      # Auxiliary functions for user-defined code to call
 
1193  
 


1194 -    def _auxfn_globalindepvar(self, parsinps, t):


1195          return self.globalt0 + t

1196  
 


1197 -    def _auxfn_initcond(self, parsinps, varname):


1198          return self.initialconditions[varname]

1199  
 


1200 -    def _auxfn_heav(self, parsinps, x):


1201          if x>0: 
1202              return 1 
1203          else: 
1204              return 0

1205  
 


1206 -    def _auxfn_if(self, parsinps, c, e1, e2):


1207          if c: 
1208              return e1 
1209          else: 
1210              return e2

1211  
 


1212 -    def _auxfn_getindex(self, parsinps, varname):


1213          return self._var_namemap[varname]

1214  
 
1215  
 


1216 -    def _generate_ixmaps(self, gentypes=None):


1217          """Generate indices mapping.
 
1218  
 
1219          This creates a mapping from the names of variables,
 
1220          pars and inputs, to indices in the arrays used for
 
1221          refering to the internal (dynamic) call methods.""" 
1222  
 
1223          if gentypes is not None: 
1224              if isinstance(gentypes, str): 
1225                  gentypes = [gentypes] 
1226              for s in gentypes: 
1227                  assert s in ['variables', 'inputs', 'pars'], \
 
1228                         ('Incorrect type string for _generate_ixmaps') 
1229          else: 
1230              # default to all
 
1231              gentypes = ['variables', 'inputs', 'pars'] 
1232          # ixmap (list) : int -> str
 
1233          # namemap (dict) : str -> int
 
1234          if 'variables' in gentypes: 
1235              self._var_ixmap = sortedDictKeys(self.variables,
 
1236                                               self.funcspec.vars) 
1237              self._var_namemap = invertMap(self._var_ixmap) 
1238          if 'pars' in gentypes: 
1239              if self.pars: 
1240                  self._parameter_ixmap = sortedDictKeys(self.pars) 
1241                  self._parameter_namemap = invertMap(self._parameter_ixmap) 
1242              else: 
1243                  self._parameter_ixmap = [] 
1244                  self._parameter_namemap = {} 
1245          if 'inputs' in gentypes: 
1246              if self.inputs: 
1247                  self._inputs_ixmap = \
 
1248                      sortedDictKeys(self.inputs) 
1249                  self._inputs_namemap = invertMap(self._inputs_ixmap) 
1250              else: 
1251                  self._inputs_ixmap = [] 
1252                  self._inputs_namemap = {}

1253  
 
1254  
 


1255 -    def contains(self, interval, val, checklevel=2):


1256          """Interval containment test""" 
1257          # NB. val may be another interval
 
1258          if checklevel == 0: 
1259              # level 0 -- no bounds checking at all
 
1260              # code should avoid calling this function with checklevel = 0
 
1261              # if possible, but this case is left here for completeness and
 
1262              # consistency
 
1263              return True 
1264          elif checklevel == 2: 
1265              # level 2 -- warn on uncertain and continue
 
1266              testresult = interval.contains(val) 
1267              if testresult is contained: 
1268                  return True 
1269              elif testresult is uncertain: 
1270                  self.diagnostics.warnings.append((W_UNCERTVAL, (val,interval))) 
1271                  return True 
1272              else: 
1273                  return False 
1274          elif checklevel == 1: 
1275              # level 1 -- ignore uncertain cases (treat as contained)
 
1276              if interval.contains(val) is not notcontained: 
1277                  return True 
1278              else: 
1279                  return False 
1280          else: 
1281              # level 3 -- exception will be raised for uncertain case
 
1282              if val in interval: 
1283                  return True 
1284              else: 
1285                  return False

1286  
 
1287  
 
1288      # Methods for pickling protocol
 


1289 -    def __getstate__(self):


1290          d = copy(self.__dict__) 
1291          for fname, finfo in self._funcreg.iteritems(): 
1292              try: 
1293                  del d[fname] 
1294              except KeyError: 
1295                  pass 
1296          return d

1297  
 
1298  
 


1299 -    def __setstate__(self, state):


1300          self.__dict__.update(state) 
1301          if self._funcreg != {}: 
1302              self.addMethods()

1303  
 
1304  
 


1305 -    def __del__(self):


1306          # delete object-specific class methods etc. before deleting
 
1307          # to avoid crowding namespace
 
1308          try: 
1309              for fname, finfo in self._funcreg.iteritems(): 
1310                  try: 
1311                      delattr(eval(finfo[0]), fname) 
1312                  except AttributeError: 
1313                      pass 
1314                  except NameError: 
1315                      # not sure what happens here, but some other names
 
1316                      # may be deleted before all references to them have
 
1317                      # been deleted, but it's very non-fatal to ignore.
 
1318                      pass 
1319              if hasattr(self, 'eventstruct'): 
1320                  if self.eventstruct is not None: 
1321                      self.eventstruct.__del__() 
1322              if self.indepvariable is not None: 
1323                  del self.indepvariable 
1324              for v in self.variables.values(): 
1325                  v.__del__() 
1326              if hasattr(self, 'inputs'): 
1327                  for v in self.inputs.values(): 
1328                      v.__del__() 
1329          except AttributeError: 
1330              # self does not have _funcreg
 
1331              pass 
1332          except NameError: 
1333              # see above notes for NameError catch
 
1334              pass

1335  
 
1336  
 


1337 -    def __copy__(self):


1338          pickledself = pickle.dumps(self) 
1339          return pickle.loads(pickledself)

1340  
 
1341  
 


1342 -    def __deepcopy__(self, memo=None, _nil=[]):


1343          pickledself = pickle.dumps(self) 
1344          return pickle.loads(pickledself)

1345  
 
1346  
 
1347  #--------------------------------------------------------------------------
 
1348  
 
1349  
 


1350 -class ctsGen(Generator):


1351      "Abstract class for continuously-parameterized trajectory generators." 
1352  
 


1353 -    def validateSpec(self):


1354          # only check that domain is cts, range may be a finite subset of points
 
1355          assert isinputcts(self.indepvariable), ("self.indepvariable must be continuously-"
 
1356                                           "defined for this class")

1357  
 


1358 -    def __del__(self):


1359          Generator.__del__(self)

1360  
 
1361  
 
1362  
 


1363 -class discGen(Generator):


1364      "Abstract class for discretely-parameterized trajectory generators." 
1365  
 


1366 -    def validateSpec(self):


1367          assert isdiscrete(self.indepvariable), ("self.indepvariable must be discretely-"
 
1368                                           "defined for this class")

1369  
 


1370 -    def __del__(self):


1371          Generator.__del__(self)

1372  
 
1373  
 
1374  #--------------------------------------------------------------------------
 
1375  
 
1376  
 


1377 -class GenSpecInfoObj(object):


1378      # empty class struct for GenSpecHelper
 
1379      pass

1380  
 
1381  
 


1382 -class GenSpecHelper(object):


1383      """Generator specification helper - abstract class.
 
1384  
 
1385      Used to help ModelConstructor translate abstract model specifications
 
1386      into concrete specifications specific to individual Generators.""" 
1387  
 


1388 -    def __init__(self):


1389          self.gshDB = {}

1390  
 


1391 -    def add(self, genClass, symbolMapDict, lang, specType='RHSfuncSpec'):


1392          genName = className(genClass) 
1393          if genName in self.gshDB: 
1394              raise ValueError("Generator %s has already been declared"%genName) 
1395          else: 
1396              infoObj = GenSpecInfoObj() 
1397              infoObj.genClass = genClass 
1398              infoObj.symbolMap = symbolMapClass(symbolMapDict) 
1399              infoObj.lang = lang 
1400              infoObj.specType = specType 
1401              if issubclass(genClass, ctsGen): 
1402                  infoObj.domain = Continuous 
1403              elif issubclass(genClass, discGen): 
1404                  infoObj.domain = Discrete 
1405              else: 
1406                  raise TypeError("Invalid Generator class") 
1407              self.gshDB[genName] = infoObj

1408  
 


1409 -    def __call__(self, subject):


1410          try: 
1411              if isinstance(subject, str): 
1412                  return self.gshDB[subject] 
1413              else: 
1414                  return self.gshDB[className(subject)] 
1415          except KeyError: 
1416              raise KeyError("Generator %s was not found in database"%str(subject))

1417  
 


1418 -    def __contains__(self, subject):


1419          return subject in self.gshDB or className(subject) in self.gshDB

1420  
 
1421  
 


1422 -def _pollInputs(inputVarList, t, checklevel):


1423      ilist = [] 
1424      try: 
1425          for f in inputVarList: 
1426              f.diagnostics.clearWarnings() 
1427              ilist.append(f(t, checklevel)) 
1428      except AssertionError: 
1429          print 'External input call has t out of range: t = ', t 
1430          print 'Maybe checklevel is 3 and initial time is not', \
 
1431                      'completely inside valid time interval' 
1432          raise 
1433      except ValueError: 
1434          print 'External input call has value out of range: t = ', t 
1435          print 'Check beginning and end time of integration' 
1436          for f in inputVarList: 
1437              if f.diagnostics.hasWarnings(): 
1438                  print 'External input %s out of range:' % f.name 
1439                  print '   t = ', repr(f.diagnostics.warnings[-1][0]), ', ', \
 
1440                        f.name, ' = ', repr(f.diagnostics.warnings[-1][1]) 
1441          raise 
1442      return ilist

1443  
 
1444  global theGenSpecHelper 
1445  theGenSpecHelper = GenSpecHelper() 
1446
```

  


| Home | Trees | Indices | Help | | PyDSTool | | --- | |
| --- | --- | --- | --- | --- | --- |

|  |  |
| --- | --- |
| Generated by Epydoc 3.0.1 on Fri May 4 15:24:25 2012 | http://epydoc.sourceforge.net |
