# Supplementary material for: Hybrid Models and Biological Model Reduction with PyDSTool
Source: PLoS Comput Biol. 2012 Aug 9;8(8):e1002628. doi: 10.1371/journal.pcbi.1002628 (PMC3415397; doi:10.1371/journal.pcbi.1002628)
Supplement: Text S4 — Complete source code for the PyDSTool package (version 0.88.120504). Includes API documentation and help files linking to web pages. This file is identical to the current public release on Sourceforge.net. (ZIP) [file pcbi.1002628.s004.zip › PyDSTool/html/PyDSTool.Generator.baseclasses.ctsGen-class.html]

xml version="1.0" encoding="ascii"?


PyDSTool.Generator.baseclasses.ctsGen


| Home | Trees | Indices | Help | | PyDSTool | | --- | |
| --- | --- | --- | --- | --- | --- |

|  |  |  |  |
| --- | --- | --- | --- |
| Package PyDSTool :: Package Generator :: Module baseclasses :: Class ctsGen | |  | | --- | | [hide private] | | [frames] | no frames] | |

# Class ctsGen

source code

```
object --+    
         |    
 Generator --+
             |
            ctsGen
```

Known Subclasses:
:   - ImplicitFnGen'.ImplicitFnGen
    - , ODEsystem'.ODEsystem
    - , ExplicitFnGen'.ExplicitFnGen
    - , EmbeddedSysGen'.EmbeddedSysGen
    - , DDEsystem.DDEsystem
    - , InterpolateTable'.InterpolateTable
    - , ExtrapolateTable'.ExtrapolateTable

---

Abstract class for continuously-parameterized trajectory
generators.


|  |  |  |  |
| --- | --- | --- | --- |
| |  |  | | --- | --- | | Instance Methods | [hide private] | | |
|  | |  |  | | --- | --- | | validateSpec(self) | source code | |
|  | |  |  | | --- | --- | | \_\_del\_\_(self) | source code | |
| **Inherited from `Generator`**: `__copy__`, `__deepcopy__`, `__getstate__`, `__init__`, `__repr__`, `__setstate__`, `__str__`, `addEvtPars`, `checkArgs`, `contains`, `get`, `getEventTimes`, `getEvents`, `haveJacobian`, `haveJacobian_pars`, `info`, `query`, `resetEventTimes`, `resetEvents`, `set`, `setEventICs`, `showAuxFnSpec`, `showAuxSpec`, `showEventSpec`, `showSpec`  **Inherited from `Generator`** (private): `_addEvents`, `_auxfn_getindex`, `_auxfn_globalindepvar`, `_auxfn_heav`, `_auxfn_if`, `_auxfn_initcond`, `_generate_ixmaps`, `_infostr`, `_kw_process_algparams`, `_kw_process_allvars`, `_kw_process_dispatch`, `_kw_process_events`, `_kw_process_fnspecs`, `_kw_process_ics`, `_kw_process_ignorespecial`, `_kw_process_inputs`, `_kw_process_pars`, `_kw_process_pdomain`, `_kw_process_reuseterms`, `_kw_process_system`, `_kw_process_target`, `_kw_process_tdata`, `_kw_process_tdomain`, `_kw_process_tstep`, `_kw_process_ttype`, `_kw_process_varspecs`, `_kw_process_vfcodeinserts`, `_kw_process_xdomain`, `_kw_process_xtype`, `_makeBoundsEvents`, `_register`, `_set_for_hybrid_DS`  **Inherited from `object`**: `__delattr__`, `__getattribute__`, `__hash__`, `__new__`, `__reduce__`, `__reduce_ex__`, `__setattr__` | |


|  |  |  |  |
| --- | --- | --- | --- |
| |  |  | | --- | --- | | Class Variables | [hide private] | | |
| **Inherited from `Generator`** (private): `_needKeys`, `_optionalKeys`, `_querykeys` | |


|  |  |  |  |
| --- | --- | --- | --- |
| |  |  | | --- | --- | | Properties | [hide private] | | |
| **Inherited from `object`**: `__class__` | |


|  |  |  |  |
| --- | --- | --- | --- |
| |  |  | | --- | --- | | Method Details | [hide private] | | |

|  |  |  |
| --- | --- | --- |
| |  |  | | --- | --- | | validateSpec(self) | source code |   Overrides: Generator.validateSpec |

|  |  |  |
| --- | --- | --- |
| |  |  | | --- | --- | | \_\_del\_\_(self)  *(Destructor)* | source code |   Overrides: Generator.\_\_del\_\_ |

  


| Home | Trees | Indices | Help | | PyDSTool | | --- | |
| --- | --- | --- | --- | --- | --- |

|  |  |
| --- | --- |
| Generated by Epydoc 3.0.1 on Fri May 4 15:24:06 2012 | http://epydoc.sourceforge.net |
