# Supplementary material for: Hybrid Models and Biological Model Reduction with PyDSTool
Source: PLoS Comput Biol. 2012 Aug 9;8(8):e1002628. doi: 10.1371/journal.pcbi.1002628 (PMC3415397; doi:10.1371/journal.pcbi.1002628)
Supplement: Text S4 — Complete source code for the PyDSTool package (version 0.88.120504). Includes API documentation and help files linking to web pages. This file is identical to the current public release on Sourceforge.net. (ZIP) [file pcbi.1002628.s004.zip › PyDSTool/html/PyDSTool.Generator.baseclasses.genDBClass-class.html]

xml version="1.0" encoding="ascii"?


PyDSTool.Generator.baseclasses.genDBClass


| Home | Trees | Indices | Help | | PyDSTool | | --- | |
| --- | --- | --- | --- | --- | --- |

|  |  |  |  |
| --- | --- | --- | --- |
| Package PyDSTool :: Package Generator :: Module baseclasses :: Class genDBClass | |  | | --- | | [hide private] | | [frames] | no frames] | |

# Class genDBClass

source code

```
object --+
         |
        genDBClass
```

---

This class keeps a record of which non-Python Generators have been
created in a session. A single global instance of this class is created,
and prevents the user from re-using the name of a low-level DLL (such as
that for a Dopri\_ODEsystem vector field) unless the system is identical
(according to its FuncSpec).


|  |  |  |  |
| --- | --- | --- | --- |
| |  |  | | --- | --- | | Instance Methods | [hide private] | | |
|  | |  |  | | --- | --- | | \_\_init\_\_(self)  x.\_\_init\_\_(...) initializes x; see x.\_\_class\_\_.\_\_doc\_\_ for signature | source code | |
|  | |  |  | | --- | --- | | check(self, gen)  Look up generator instance and return Boolean of whether it is already registered. | source code | |
|  | |  |  | | --- | --- | | unregister(self, gen) | source code | |
|  | |  |  | | --- | --- | | register(self, gen) | source code | |
|  | |  |  | | --- | --- | | \_\_repr\_\_(self)  str(x) | source code | |
|  | |  |  | | --- | --- | | \_\_str\_\_(self)  str(x) | source code | |
|  | |  |  | | --- | --- | | clearall(self) | source code | |
| **Inherited from `object`**: `__delattr__`, `__getattribute__`, `__hash__`, `__new__`, `__reduce__`, `__reduce_ex__`, `__setattr__` | |


|  |  |  |  |
| --- | --- | --- | --- |
| |  |  | | --- | --- | | Properties | [hide private] | | |
| **Inherited from `object`**: `__class__` | |


|  |  |  |  |
| --- | --- | --- | --- |
| |  |  | | --- | --- | | Method Details | [hide private] | | |

|  |  |  |
| --- | --- | --- |
| |  |  | | --- | --- | | \_\_init\_\_(self)  *(Constructor)* | source code |   x.\_\_init\_\_(...) initializes x; see x.\_\_class\_\_.\_\_doc\_\_ for signature  Overrides: object.\_\_init\_\_ *(inherited documentation)* |

|  |  |  |
| --- | --- | --- |
| |  |  | | --- | --- | | check(self, gen) | source code |   Look up generator instance and return Boolean of whether it is already registered. Only non-python based generators are registered, so all others will return False. |

|  |  |  |
| --- | --- | --- |
| |  |  | | --- | --- | | \_\_repr\_\_(self)  *(Representation operator)* | source code |   str(x)  Overrides: object.\_\_repr\_\_ *(inherited documentation)* |

|  |  |  |
| --- | --- | --- |
| |  |  | | --- | --- | | \_\_str\_\_(self)  *(Informal representation operator)* | source code |   str(x)  Overrides: object.\_\_str\_\_ *(inherited documentation)* |

  


| Home | Trees | Indices | Help | | PyDSTool | | --- | |
| --- | --- | --- | --- | --- | --- |

|  |  |
| --- | --- |
| Generated by Epydoc 3.0.1 on Fri May 4 15:24:06 2012 | http://epydoc.sourceforge.net |
