# Supplementary material for: Hybrid Models and Biological Model Reduction with PyDSTool
Source: PLoS Comput Biol. 2012 Aug 9;8(8):e1002628. doi: 10.1371/journal.pcbi.1002628 (PMC3415397; doi:10.1371/journal.pcbi.1002628)
Supplement: Text S4 — Complete source code for the PyDSTool package (version 0.88.120504). Includes API documentation and help files linking to web pages. This file is identical to the current public release on Sourceforge.net. (ZIP) [file pcbi.1002628.s004.zip › PyDSTool/html/PyDSTool.Generator.baseclasses.Generator-class.html]

xml version="1.0" encoding="ascii"?


PyDSTool.Generator.baseclasses.Generator


| Home | Trees | Indices | Help | | PyDSTool | | --- | |
| --- | --- | --- | --- | --- | --- |

|  |  |  |  |
| --- | --- | --- | --- |
| Package PyDSTool :: Package Generator :: Module baseclasses :: Class Generator | |  | | --- | | [hide private] | | [frames] | no frames] | |

# Class Generator

source code

```
object --+
         |
        Generator
```

Known Subclasses:
:   - ctsGen
    - , discGen

---

Trajectory Generator abstract class.


|  |  |  |  |
| --- | --- | --- | --- |
| |  |  | | --- | --- | | Instance Methods | [hide private] | | |
|  | |  |  | | --- | --- | | \_\_init\_\_(self, kw)  x.\_\_init\_\_(...) initializes x; see x.\_\_class\_\_.\_\_doc\_\_ for signature | source code | |
|  | |  |  | | --- | --- | | addEvtPars(self, eventPars)  Register parameter names as event specific parameters. | source code | |
|  | |  |  | | --- | --- | | getEvents(self, evnames=None, asGlobalTime=True)  Produce dictionary of pointsets of all flagged events' independent and dependent variable values, for each event (whether terminal or not). | source code | |
|  | |  |  | | --- | --- | | getEventTimes(self, evnames=None, asGlobalTime=True)  Produce dictionary of lists of all flagged events' independent variable values, for each event (whether terminal or not). | source code | |
|  | |  |  | | --- | --- | | query(self, querykey=`'``'`)  Return info about Generator set-up. | source code | |
|  | |  |  | | --- | --- | | get(self, key)  For API compatibility with ModelInterface: get will make a copy of the key and pass it through the inverse FuncSpec-compatible name map. | source code | |
|  | |  |  | | --- | --- | | haveJacobian(self)  Default method. | source code | |
|  | |  |  | | --- | --- | | haveJacobian\_pars(self)  Default method. | source code | |
|  | |  |  | | --- | --- | | info(self, verbose=1) | source code | |
|  | |  |  | | --- | --- | | \_kw\_process\_dispatch(self, keys, kw) | source code | |
|  | |  |  | | --- | --- | | \_kw\_process\_varspecs(self, kw, fs\_args) | source code | |
|  | |  |  | | --- | --- | | \_kw\_process\_tdomain(self, kw, fs\_args) | source code | |
|  | |  |  | | --- | --- | | \_kw\_process\_ttype(self, kw, fs\_args) | source code | |
|  | |  |  | | --- | --- | | \_kw\_process\_tdata(self, kw, fs\_args) | source code | |
|  | |  |  | | --- | --- | | \_kw\_process\_tstep(self, kw, fs\_args) | source code | |
|  | |  |  | | --- | --- | | \_kw\_process\_inputs(self, kw, fs\_args) | source code | |
|  | |  |  | | --- | --- | | \_kw\_process\_ics(self, kw, fs\_args) | source code | |
|  | |  |  | | --- | --- | | \_kw\_process\_allvars(self, kw, fs\_args) | source code | |
|  | |  |  | | --- | --- | | \_kw\_process\_xtype(self, kw, fs\_args) | source code | |
|  | |  |  | | --- | --- | | \_kw\_process\_xdomain(self, kw, fs\_args) | source code | |
|  | |  |  | | --- | --- | | \_kw\_process\_reuseterms(self, kw, fs\_args) | source code | |
|  | |  |  | | --- | --- | | \_kw\_process\_ignorespecial(self, kw, fs\_args) | source code | |
|  | |  |  | | --- | --- | | \_kw\_process\_algparams(self, kw, fs\_args) | source code | |
|  | |  |  | | --- | --- | | \_kw\_process\_pars(self, kw, fs\_args) | source code | |
|  | |  |  | | --- | --- | | \_kw\_process\_pdomain(self, kw, fs\_args) | source code | |
|  | |  |  | | --- | --- | | \_kw\_process\_fnspecs(self, kw, fs\_args) | source code | |
|  | |  |  | | --- | --- | | \_kw\_process\_target(self, kw, fs\_args) | source code | |
|  | |  |  | | --- | --- | | \_kw\_process\_vfcodeinserts(self, kw, fs\_args) | source code | |
|  | |  |  | | --- | --- | | \_kw\_process\_system(self, kw, fs\_args) | source code | |
|  | |  |  | | --- | --- | | \_infostr(self, verbose=1)  Return detailed information about the Generator specification. | source code | |
|  | |  |  | | --- | --- | | showEventSpec(self) | source code | |
|  | |  |  | | --- | --- | | showSpec(self) | source code | |
|  | |  |  | | --- | --- | | showAuxSpec(self) | source code | |
|  | |  |  | | --- | --- | | showAuxFnSpec(self, auxfnname=None) | source code | |
|  | |  |  | | --- | --- | | \_\_repr\_\_(self)  str(x) | source code | |
|  | |  |  | | --- | --- | | \_\_str\_\_(self)  str(x) | source code | |
|  | |  |  | | --- | --- | | validateSpec(self) | source code | |
|  | |  |  | | --- | --- | | checkArgs(self, kw) | source code | |
|  | |  |  | | --- | --- | | \_set\_for\_hybrid\_DS(self, state)  Internal method for indicating whether this Generator is currently being used as part of a hybrid dybnamical system calculation | source code | |
|  | |  |  | | --- | --- | | \_register(self, items)  \_register names and types of sub-system variables (including Generator variables), pars and external inputs. | source code | |
|  | |  |  | | --- | --- | | \_kw\_process\_events(self, kw) | source code | |
|  | |  |  | | --- | --- | | \_addEvents(self, evs) | source code | |
|  | |  |  | | --- | --- | | \_makeBoundsEvents(self, precise=True, eventtol=1e-06, activatedbounds=None) | source code | |
|  | |  |  | | --- | --- | | set(self, \*\*kw)  Set generic parameters. | source code | |
|  | |  |  | | --- | --- | | setEventICs(self, ics, gt0=0)  Set initialconditions attribute of all generator's events, in case event uses auxiliary functions that access this information. | source code | |
|  | |  |  | | --- | --- | | resetEventTimes(self) | source code | |
|  | |  |  | | --- | --- | | resetEvents(self, state=None)  Reset any high level (Python) events in Generator | source code | |
|  | |  |  | | --- | --- | | \_auxfn\_globalindepvar(self, parsinps, t) | source code | |
|  | |  |  | | --- | --- | | \_auxfn\_initcond(self, parsinps, varname) | source code | |
|  | |  |  | | --- | --- | | \_auxfn\_heav(self, parsinps, x) | source code | |
|  | |  |  | | --- | --- | | \_auxfn\_if(self, parsinps, c, e1, e2) | source code | |
|  | |  |  | | --- | --- | | \_auxfn\_getindex(self, parsinps, varname) | source code | |
|  | |  |  | | --- | --- | | \_generate\_ixmaps(self, gentypes=None)  Generate indices mapping. | source code | |
|  | |  |  | | --- | --- | | contains(self, interval, val, checklevel=2)  Interval containment test | source code | |
|  | |  |  | | --- | --- | | \_\_getstate\_\_(self) | source code | |
|  | |  |  | | --- | --- | | \_\_setstate\_\_(self, state) | source code | |
|  | |  |  | | --- | --- | | \_\_del\_\_(self) | source code | |
|  | |  |  | | --- | --- | | \_\_copy\_\_(self) | source code | |
|  | |  |  | | --- | --- | | \_\_deepcopy\_\_(self, memo=None, \_nil=`[``]`) | source code | |
| **Inherited from `object`**: `__delattr__`, `__getattribute__`, `__hash__`, `__new__`, `__reduce__`, `__reduce_ex__`, `__setattr__` | |


|  |  |  |  |
| --- | --- | --- | --- |
| |  |  | | --- | --- | | Class Variables | [hide private] | | |
|  | \_querykeys = `['pars', 'parameters', 'events', 'abseps', 'ics',...` |
|  | \_needKeys = `['name']` |
|  | \_optionalKeys = `['globalt0', 'checklevel', 'model', 'abseps', ...` |


|  |  |  |  |
| --- | --- | --- | --- |
| |  |  | | --- | --- | | Properties | [hide private] | | |
| **Inherited from `object`**: `__class__` | |


|  |  |  |  |
| --- | --- | --- | --- |
| |  |  | | --- | --- | | Method Details | [hide private] | | |

|  |  |  |
| --- | --- | --- |
| |  |  | | --- | --- | | \_\_init\_\_(self, kw)  *(Constructor)* | source code |   x.\_\_init\_\_(...) initializes x; see x.\_\_class\_\_.\_\_doc\_\_ for signature  Overrides: object.\_\_init\_\_ *(inherited documentation)* |

|  |  |  |
| --- | --- | --- |
| |  |  | | --- | --- | | getEvents(self, evnames=None, asGlobalTime=True) | source code |   Produce dictionary of pointsets of all flagged events' independent and dependent variable values, for each event (whether terminal or not). Times will be globalized if optional asGlobalTime argument is True (default behavior). If a single event name is passed, only the pointset is returned (not a dictionary).  evnames may be a singleton string or list of strings, or left blank to return data for all events.  The events are not guaranteed to be ordered by the value of the independent variable. |

|  |  |  |
| --- | --- | --- |
| |  |  | | --- | --- | | getEventTimes(self, evnames=None, asGlobalTime=True) | source code |   Produce dictionary of lists of all flagged events' independent variable values, for each event (whether terminal or not). Times will be globalized if optional asGlobalTime argument is True (default behavior). If a single event name is passed, only the pointset is returned (not a dictionary).  evnames may be a singleton string or list of strings, or left blank to return data for all events.  The events are guaranteed to be ordered by the value of the independent variable. |

|  |  |  |
| --- | --- | --- |
| |  |  | | --- | --- | | query(self, querykey=`'``'`) | source code |  ``` Return info about Generator set-up. Valid query key: 'pars', 'parameters', 'pardomains', 'events',  'ics', 'initialconditions', 'vars', 'variables',  'auxvars', 'auxvariables', 'vardomains' ``` |

|  |  |  |
| --- | --- | --- |
| |  |  | | --- | --- | | haveJacobian(self) | source code |   Default method. Can be overridden by subclasses. |

|  |  |  |
| --- | --- | --- |
| |  |  | | --- | --- | | haveJacobian\_pars(self) | source code |   Default method. Can be overridden by subclasses. |

|  |  |  |
| --- | --- | --- |
| |  |  | | --- | --- | | \_\_repr\_\_(self)  *(Representation operator)* | source code |   str(x)  Overrides: object.\_\_repr\_\_ *(inherited documentation)* |

|  |  |  |
| --- | --- | --- |
| |  |  | | --- | --- | | \_\_str\_\_(self)  *(Informal representation operator)* | source code |   str(x)  Overrides: object.\_\_str\_\_ *(inherited documentation)* |

|  |  |  |
| --- | --- | --- |
| |  |  | | --- | --- | | \_register(self, items) | source code |   \_register names and types of sub-system variables (including Generator variables), pars and external inputs.  Names must be unique for the Generator. |

|  |  |  |
| --- | --- | --- |
| |  |  | | --- | --- | | \_generate\_ixmaps(self, gentypes=None) | source code |   Generate indices mapping.  This creates a mapping from the names of variables, pars and inputs, to indices in the arrays used for refering to the internal (dynamic) call methods. |

  


|  |  |  |  |
| --- | --- | --- | --- |
| |  |  | | --- | --- | | Class Variable Details | [hide private] | | |

|  |  |
| --- | --- |
| \_querykeys   Value:  |  | | --- | | ``` ['pars',  'parameters',  'events',  'abseps',  'ics',  'initialconditions',  'vars',  'variables', ... ``` | |

|  |  |
| --- | --- |
| \_optionalKeys   Value:  |  | | --- | | ``` ['globalt0',  'checklevel',  'model',  'abseps',  'eventPars',  'FScompatibleNames',  'FScompatibleNamesInv'] ``` | |

  


| Home | Trees | Indices | Help | | PyDSTool | | --- | |
| --- | --- | --- | --- | --- | --- |

|  |  |
| --- | --- |
| Generated by Epydoc 3.0.1 on Fri May 4 15:24:06 2012 | http://epydoc.sourceforge.net |
