# Supplementary material for: Hybrid Models and Biological Model Reduction with PyDSTool
Source: PLoS Comput Biol. 2012 Aug 9;8(8):e1002628. doi: 10.1371/journal.pcbi.1002628 (PMC3415397; doi:10.1371/journal.pcbi.1002628)
Supplement: Text S4 — Complete source code for the PyDSTool package (version 0.88.120504). Includes API documentation and help files linking to web pages. This file is identical to the current public release on Sourceforge.net. (ZIP) [file pcbi.1002628.s004.zip › PyDSTool/html/PyDSTool.Generator.baseclasses.ixmap-class.html]

xml version="1.0" encoding="ascii"?


PyDSTool.Generator.baseclasses.ixmap


| Home | Trees | Indices | Help | | PyDSTool | | --- | |
| --- | --- | --- | --- | --- | --- |

|  |  |  |  |
| --- | --- | --- | --- |
| Package PyDSTool :: Package Generator :: Module baseclasses :: Class ixmap | |  | | --- | | [hide private] | | [frames] | no frames] | |

# Class ixmap

source code

```
object --+    
         |    
      dict --+
             |
            ixmap
```

---


|  |  |  |  |
| --- | --- | --- | --- |
| |  |  | | --- | --- | | Instance Methods | [hide private] | | |
| ``` new empty dictionary ``` | |  |  | | --- | --- | | \_\_init\_\_(self, genref)  x.\_\_init\_\_(...) initializes x; see x.\_\_class\_\_.\_\_doc\_\_ for signature | source code | |
|  | |  |  | | --- | --- | | \_\_getitem\_\_(self, k)  x[y] | source code | |
|  | |  |  | | --- | --- | | \_\_repr\_\_(self)  repr(x) | source code | |
| **Inherited from `dict`**: `__cmp__`, `__contains__`, `__delitem__`, `__eq__`, `__ge__`, `__getattribute__`, `__gt__`, `__hash__`, `__iter__`, `__le__`, `__len__`, `__lt__`, `__ne__`, `__new__`, `__setitem__`, `clear`, `copy`, `fromkeys`, `get`, `has_key`, `items`, `iteritems`, `iterkeys`, `itervalues`, `keys`, `pop`, `popitem`, `setdefault`, `update`, `values`  **Inherited from `object`**: `__delattr__`, `__reduce__`, `__reduce_ex__`, `__setattr__`, `__str__` | |


|  |  |  |  |
| --- | --- | --- | --- |
| |  |  | | --- | --- | | Properties | [hide private] | | |
| **Inherited from `object`**: `__class__` | |


|  |  |  |  |
| --- | --- | --- | --- |
| |  |  | | --- | --- | | Method Details | [hide private] | | |

|  |  |  |
| --- | --- | --- |
| |  |  | | --- | --- | | \_\_init\_\_(self, genref)  *(Constructor)* | source code |   x.\_\_init\_\_(...) initializes x; see x.\_\_class\_\_.\_\_doc\_\_ for signature  Returns: ``` new empty dictionary ```  Overrides: object.\_\_init\_\_ *(inherited documentation)* |

|  |  |  |
| --- | --- | --- |
| |  |  | | --- | --- | | \_\_getitem\_\_(self, k)  *(Indexing operator)* | source code |   x[y]  Overrides: dict.\_\_getitem\_\_ *(inherited documentation)* |

|  |  |  |
| --- | --- | --- |
| |  |  | | --- | --- | | \_\_repr\_\_(self)  *(Representation operator)* | source code |   repr(x)  Overrides: object.\_\_repr\_\_ *(inherited documentation)* |

  


| Home | Trees | Indices | Help | | PyDSTool | | --- | |
| --- | --- | --- | --- | --- | --- |

|  |  |
| --- | --- |
| Generated by Epydoc 3.0.1 on Fri May 4 15:24:06 2012 | http://epydoc.sourceforge.net |
