# Supplementary material for: Hybrid Models and Biological Model Reduction with PyDSTool
Source: PLoS Comput Biol. 2012 Aug 9;8(8):e1002628. doi: 10.1371/journal.pcbi.1002628 (PMC3415397; doi:10.1371/journal.pcbi.1002628)
Supplement: Text S4 — Complete source code for the PyDSTool package (version 0.88.120504). Includes API documentation and help files linking to web pages. This file is identical to the current public release on Sourceforge.net. (ZIP) [file pcbi.1002628.s004.zip › PyDSTool/html/PyDSTool.Generator.DDEsystem-pysrc.html]

xml version="1.0" encoding="ascii"?


PyDSTool.Generator.DDEsystem


| Home | Trees | Indices | Help | | PyDSTool | | --- | |
| --- | --- | --- | --- | --- | --- |

|  |  |  |  |
| --- | --- | --- | --- |
| Package PyDSTool :: Package Generator :: Module DDEsystem | |  | | --- | | [hide private] | | [frames] | no frames] | |

# Source Code for Module PyDSTool.Generator.DDEsystem

```
 1  # Differential-delay system (incomplete)
 
 2  
 
 3  from allimports import * 
 4  from baseclasses import ctsGen 
 5  from PyDSTool.utils import * 
 6  from PyDSTool.common import * 
 7  
 
 8  # Other imports
 
 9  from numpy import Inf, NaN, isfinite, sometrue, alltrue, array 
10  import math, random 
11  from copy import copy, deepcopy 
12  try: 
13      # use pscyo JIT byte-compiler optimization, if available
 
14      import psyco 
15      HAVE_PSYCO = True 
16  except ImportError: 
17      HAVE_PSYCO = False 
18  
 
19  # -----------------------------------------------------------------------------
 
20  
 


21 -class DDEsystem(ctsGen):


22      """Delay-differential equations.
 
23  
 
24      (incomplete)""" 
25  
 
26      # spec of initial condition _interval_ will be interesting!
 
27      # Use a Variable trajectory over that interval?
 


28 -    def __init__(self, kw):


29          ctsGen.__init__(self, kw) 
30          raise NotImplementedError

31  
 
32  
 


33 -    def validateSpec(self):


34          ctsGen.validateSpec(self)

35  
 
36  
 


37 -    def __del__(self):


38          ctsGen.__del__(self)

39
```

  


| Home | Trees | Indices | Help | | PyDSTool | | --- | |
| --- | --- | --- | --- | --- | --- |

|  |  |
| --- | --- |
| Generated by Epydoc 3.0.1 on Fri May 4 15:24:18 2012 | http://epydoc.sourceforge.net |
