# Supplementary material for: Hybrid Models and Biological Model Reduction with PyDSTool
Source: PLoS Comput Biol. 2012 Aug 9;8(8):e1002628. doi: 10.1371/journal.pcbi.1002628 (PMC3415397; doi:10.1371/journal.pcbi.1002628)
Supplement: Text S4 — Complete source code for the PyDSTool package (version 0.88.120504). Includes API documentation and help files linking to web pages. This file is identical to the current public release on Sourceforge.net. (ZIP) [file pcbi.1002628.s004.zip › PyDSTool/html/PyDSTool.Generator.Dopri_ODEsystem'-pysrc.html]

xml version="1.0" encoding="ascii"?


PyDSTool.Generator.Dopri\_ODEsystem'


| Home | Trees | Indices | Help | | PyDSTool | | --- | |
| --- | --- | --- | --- | --- | --- |

|  |  |  |  |
| --- | --- | --- | --- |
| Package PyDSTool :: Package Generator :: Module Dopri\_ODEsystem' | |  | | --- | | [hide private] | | [frames] | no frames] | |

# Source Code for Module PyDSTool.Generator.Dopri\_ODEsystem'

```
   1  # Dopri ODE system
 
   2  from __future__ import division 
   3  
 
   4  from allimports import * 
   5  from PyDSTool.Generator import ODEsystem as ODEsystem 
   6  from baseclasses import Generator, theGenSpecHelper, genDB, _pollInputs 
   7  from PyDSTool.utils import * 
   8  from PyDSTool.common import * 
   9  from PyDSTool.integrator import integrator 
  10  from PyDSTool.parseUtils import addArgToCalls, wrapArgInCall 
  11  import PyDSTool.Redirector as redirc 
  12  import numpy as npy 
  13  
 
  14  # Other imports
 
  15  from numpy import Inf, NaN, isfinite, int, int32, float, float64, \
 
  16       sometrue, alltrue, any, all, concatenate, transpose, array, zeros 
  17  import math, random 
  18  import operator 
  19  from copy import copy, deepcopy 
  20  import os, platform, shutil, sys, gc 
  21  #import distutils
 
  22  from numpy.distutils.core import setup, Extension 
  23  from distutils.sysconfig import get_python_inc 
  24  from time import clock, sleep 
  25  
 
  26  # path to the installation
 
  27  import PyDSTool 
  28  _pydstool_path = PyDSTool.__path__[0] 
  29  
 
  30  
 
  31  rout = redirc.Redirector(redirc.STDOUT) 
  32  rerr = redirc.Redirector(redirc.STDERR) 
  33  
 


34 -def distutil_destination():


35      """Makes the goofy destination directory string so that we can find where the distutils
 
  36      fortran compiler puts things.""" 
  37      osname = str.lower(platform.system()) 
  38      pyname = platform.python_version_tuple() 
  39      machinename = platform.machine() 
  40      if osname == 'linux': 
  41          destdir = 'src.'+osname+'-'+machinename+'-'+pyname[0] + '.' + pyname[1] 
  42      elif osname == 'darwin': 
  43          osver = platform.mac_ver()[0].split('.') 
  44          if int(scipy.__version__.split('.')[1]) > 5 and len(osver)>1 and osver != ['']: 
  45              destdir = 'src.macosx-'+osver[0]+'.'+osver[1]+'-'+machinename+'-'+pyname[0] + '.' + pyname[1] 
  46          else: 
  47              destdir = 'src.'+osname+'-'+platform.release()+'-'+machinename+'-'+pyname[0] + '.' + pyname[1] 
  48      elif osname == 'windows': 
  49          destdir = 'src.win32-'+pyname[0]+'.'+pyname[1] 
  50      else: 
  51          destdir = '' 
  52  
 
  53      return destdir

  54  
 
  55  
 


56 -class dopri(integrator):


57      """Dopri 853 specialization of the basic integrator class.""" 
  58  
 


59 -    def __init__(self, modname, rhs='default_name', phaseDim=0, paramDim=0,
 
  60                   nAux=0, nEvents=0, nExtInputs=0,
 
  61                   hasJac=0, hasJacP=0, hasMass=0, extraSpace=0,
 
  62                   defaultBound=1e8):


63  
 
  64          integrator.__init__(self, rhs=rhs, phaseDim=phaseDim, paramDim=paramDim,
 
  65                              nAux=nAux, nEvents=nEvents, nExtInputs=nExtInputs,
 
  66                              hasJac=hasJac, hasJacP=hasJacP, hasMass=hasMass,
 
  67                              extraSpace=extraSpace, defaultBound=defaultBound) 
  68          self.modname = modname 
  69          try: 
  70              self._integMod = __import__(modname, globals()) 
  71          except: 
  72              print "Error in importing compiled vector field and integrator." 
  73              print "Did you compile the RHS C code?" 
  74              raise 
  75          # check module's directory
 
  76          assert 'Integrate' in dir(self._integMod), \
 
  77                 "dopri853 library does not contain Integrate()" 
  78  
 
  79          self.fac1 = [] 
  80          self.fac2 = [] 
  81          self.safety = [] 
  82          self.beta = [] 
  83          self.checkBounds = 0 
  84          self.boundsCheckMaxSteps = 1000 
  85          self.magBound = 1000000 
  86  
 
  87          retval = self._integMod.InitBasic(self.phaseDim, self.paramDim, self.nAux,
 
  88                                            self.nEvents, self.nExtInputs, self.hasJac,
 
  89                                            self.hasJacP, self.hasMass, self.extraSpace) 
  90  
 
  91          if retval[0] != 1: 
  92              raise PyDSTool_InitError('Call to InitBasic failed! (dopri)') 
  93  
 
  94          self.initBasic = True

  95  
 
  96  
 


97 -    def Run(self, hinit=0, hmax=1.0, checkAux=0, calcSpecTimes=0, verbose=0,
 
  98              fac1=0.2, fac2=10.0, safety=0.9, beta=0.04, checkBounds=0,
 
  99              boundsCheckMaxSteps=1000, magBound=1000000):


100          if not self.initBasic: 
 101              raise PyDSTool_InitError('initBasic is False (dopri)') 
 102          if not self.initEvents: 
 103              raise PyDSTool_InitError('initEvents is False (dopri)') 
 104          if not self.initIntegrate: 
 105              raise PyDSTool_InitError('initInteg is False (dopri)') 
 106          if not self.setParams: 
 107              raise PyDSTool_InitError('setParams is False (dopri)') 
 108          if self.nExtInputs > 0 and not self.initExtInputs: 
 109              raise PyDSTool_InitError('initExtInputs is False (dopri)') 
 110  
 
 111          self.setDopriParams(hinit=hinit, hmax=hmax, checkAux=checkAux,
 
 112                              calcSpecTimes=calcSpecTimes,
 
 113                              verbose=verbose, fac1=fac1,
 
 114                              fac2=fac2, safety=safety, beta=beta,
 
 115                              checkBounds=checkBounds,
 
 116                              boundsCheckMaxSteps=boundsCheckMaxSteps,
 
 117                              magBound=magBound) 
 118  
 
 119          # For a run, we want to ensure indices are set to 0
 
 120          self.Reset() 
 121          T, P, A, Stats, H, Err, EvtT, EvtP = self._integMod.Integrate(self.ic,
 
 122                                                            self.t0,
 
 123                                                            self.hinit,
 
 124                                                            self.hmax,
 
 125                                                            self.safety,
 
 126                                                            self.fac1,
 
 127                                                            self.fac2,
 
 128                                                            self.beta,
 
 129                                                            self.verbose,
 
 130                                                            self.checkAux,
 
 131                                                            self.calcSpecTimes,
 
 132                                                            self.checkBounds,
 
 133                                                            self.boundsCheckMaxSteps,
 
 134                                                            self.magBound) 
 135          self.points = P 
 136          self.times = T 
 137          self.auxPoints = A 
 138          self.eventTimes = EvtT 
 139          self.eventPoints = EvtP 
 140          self.errors = Err 
 141          self.stats = Stats 
 142          self.step = H 
 143  
 
 144          try: 
 145              self.lastTime = self.times[-1] 
 146              self.lastPoint = [self.points[i][-1] for i in range(self.phaseDim)] 
 147              self.lastStep = self.step 
 148          except IndexError: 
 149              self.lastTime = self.t0 
 150              self.lastPoint = self.ic 
 151              self.lastStep = self.hinit 
 152          self.numRuns += 1 
 153          self.canContinue = True 
 154  
 
 155          return T, P, A, Stats, H, Err, EvtT, EvtP

 156  
 
 157  
 


158 -    def Continue(self, tend, params=[], calcSpecTimes=0, verbose=0, extInputChanged=False,
 
 159                   extInputVals=[], extInputTimes=[], bounds=[]):


160          if not self.initBasic: 
 161              raise PyDSTool_InitError('initBasic is False (dopri)') 
 162          if not self.initEvents: 
 163              raise PyDSTool_InitError('initEvents is False (dopri)') 
 164          if not self.initIntegrate: 
 165              raise PyDSTool_InitError('initInteg is False (dopri)') 
 166          if not self.setParams: 
 167              raise PyDSTool_InitError('setParams is False (dopri)') 
 168          if self.nExtInputs > 0 and not self.initExtInputs: 
 169              raise PyDSTool_InitError('initExtInputs is False (dopri)') 
 170  
 
 171          if not self.canContinue: 
 172              raise PyDSTool_ContError('Unable to continue trajectory -- '
 
 173                      'have you run the integrator and reset events, etc?') 
 174  
 
 175          self.setContParams(tend=tend, params=copy(params),
 
 176                             calcSpecTimes=calcSpecTimes, verbose=verbose,
 
 177                             extInputChanged=extInputChanged,
 
 178                             extInputVals=copy(extInputVals),
 
 179                             extInputTimes=copy(extInputTimes),
 
 180                             bounds=copy(bounds)) 
 181  
 
 182          # For a continue, we do not set indices to 0
 
 183          T, P, A, Stats, H, Err, EvtT, EvtP = self._integMod.Integrate(self.lastPoint,
 
 184                                                            self.lastTime,
 
 185                                                            self.lastStep,
 
 186                                                            self.hmax,
 
 187                                                            self.safety,
 
 188                                                            self.fac1,
 
 189                                                            self.fac2,
 
 190                                                            self.beta,
 
 191                                                            self.verbose,
 
 192                                                            self.checkAux,
 
 193                                                            self.calcSpecTimes,
 
 194                                                            self.checkBounds,
 
 195                                                            self.boundsCheckMaxSteps,
 
 196                                                            self.magBound) 
 197          self.points = P 
 198          self.times = T 
 199          self.auxPoints = A 
 200          self.eventTimes = EvtT 
 201          self.eventPoints = EvtP 
 202          self.errors = Err 
 203          self.stats = Stats 
 204          self.step = H 
 205  
 
 206          try: 
 207              self.lastTime = self.times[-1] 
 208              self.lastPoint = [self.points[i][-1] for i in range(self.phaseDim)] 
 209              self.lastStep = self.step 
 210          except IndexError: 
 211              self.lastTime = self.t0 
 212              self.lastPoint = self.ic 
 213              self.lastStep = self.hinit 
 214          self.numRuns += 1 
 215          self.numContinues += 1 
 216          self.canContinue = True 
 217  
 
 218          return T, P, A, Stats, H, Err, EvtT, EvtP

 219  
 
 220  
 


221 -    def setDopriParams(self,hinit,hmax,checkAux,calcSpecTimes,verbose,
 
 222                         fac1,fac2,safety,beta,checkBounds,boundsCheckMaxSteps,
 
 223                         magBound):


224          checkAux = int(checkAux) 
 225          calcSpecTimes = int(calcSpecTimes) 
 226  
 
 227          if not isinstance(hinit, _num_types): 
 228              raise TypeError("hinit must be int, float") 
 229  
 
 230          if not isinstance(hmax, _num_types): 
 231              raise TypeError("hmax must be int, float") 
 232  
 
 233          if abs(hinit) > abs(hmax): 
 234              raise ValueError("Abs value of hinit (%g) must be less than hmax (%g)"%(hinit,hmax)) 
 235  
 
 236          if not isinstance(checkAux, _int_types): 
 237              raise TypeError("checkAux must be int") 
 238          if checkAux not in (0,1): 
 239              raise TypeError("checkAux must be 0 or 1") 
 240          if checkAux == 1 and self.nAux <= 0: 
 241              raise ValueError("checkAux cannot be 1 if nAux is 0") 
 242  
 
 243          if not isinstance(verbose, _int_types): 
 244              raise TypeError("verbose must be int") 
 245          if verbose not in (0,1): 
 246              if verbose >= 2: 
 247                  # interpret all greater values as 1
 
 248                  verbose = 1 
 249              else: 
 250                  raise TypeError("verbose must be 0 or 1") 
 251  
 
 252          if not isinstance(calcSpecTimes, _int_types): 
 253              raise TypeError("calcSpecTimes must be int") 
 254          if calcSpecTimes not in (0,1): 
 255              raise TypeError("calcSpecTimes must be 0 or 1") 
 256          if calcSpecTimes == 1 and len(self.specTimes) <= 0: 
 257              raise ValueError("calcSpecTimes cannot be 1 if specTimes is empty") 
 258  
 
 259          if fac1 < 0: 
 260              raise ValueError("fac1 must be non-negative") 
 261          if fac2 < 0: 
 262              raise ValueError("fac2 must be non-negative") 
 263          if beta < 0: 
 264              raise ValueError("beta must be non-negative") 
 265          if safety < 0: 
 266              raise ValueError("safety must be non-negative") 
 267  
 
 268          if not isinstance(checkBounds, _int_types): 
 269              raise TypeError("checkBounds must be int") 
 270          if checkBounds not in (0,1,2): 
 271              raise ValueError("checkBounds must be 0, 1, or 2") 
 272  
 
 273          if not isinstance(boundsCheckMaxSteps, _int_types): 
 274              raise TypeError("boundsCheckMaxSteps must be int") 
 275          if boundsCheckMaxSteps < 0: 
 276              raise ValueError("boundsCheckMaxSteps must be non-negative") 
 277  
 
 278          if isinstance(magBound, _num_types): 
 279              if magBound <= 0: 
 280                  raise ValueError("magBound must be positive") 
 281              mbound = [float(magBound) for x in range(self.phaseDim)] 
 282              self.magBound = mbound 
 283          else: 
 284              for x in magBound: 
 285                  if x <= 0: 
 286                      raise ValueError("All magBound components must be positive") 
 287              self.magBound = magBound 
 288  
 
 289          self.boundsCheckMaxSteps = boundsCheckMaxSteps 
 290          self.checkBounds = checkBounds 
 291          self.hinit = hinit 
 292          self.hmax = hmax 
 293          self.checkAux = checkAux 
 294          self.verbose = verbose 
 295          self.calcSpecTimes = calcSpecTimes 
 296          self.fac1 = fac1 
 297          self.fac2 = fac2 
 298          self.beta = beta 
 299          self.safety = safety

 300  
 
 301  
 
 302  
 


303 -class Dopri_ODEsystem(ODEsystem):


304      """Wrapper for Dopri853 integrator.
 
 305  
 
 306      Uses C target language only for functional specifications.""" 
 307      _paraminfo = {'rtol': 'Relative error tolerance.',
 
 308                    'atol': 'Absolute error tolerance.',
 
 309                    'safety': 'Safety factor in the step size prediction, default 0.9.',
 
 310                    'fac1': 'Parameter for step size selection; the new step size is chosen subject to the restriction  fac1 <= new_step/old_step <= fac2. Default value is 0.333.',
 
 311                    'fac2': 'Parameter for step size selection; the new step size is chosen subject to the restriction  fac1 <= new_step/old_step <= fac2. Default value is 6.0.',
 
 312                    'beta': 'The "beta" for stabilized step size control. Larger values for beta ( <= 0.1 ) make the step size control more stable. Negative initial value provoke beta=0; default beta=0.04',
 
 313                    'max_step': 'Maximal step size, default tend-tstart.',
 
 314                    'init_step': 'Initial step size, default is a guess computed by the function init_step.',
 
 315                    'refine': 'Refine output by adding points interpolated using the RK4 polynomial (0, 1 or 2).',
 
 316                    'use_special': "Switch for using special times",
 
 317                    'specialtimes': "List of special times to use during integration",
 
 318                    'check_aux': "Switch",
 
 319                    'extraspace': "",
 
 320                    'magBound': "The largest variable magnitude before a bounds error flags (if checkBound > 0). Defaults to 1e7",
 
 321                    'checkBounds': "Switch to check variable bounds: 0 = no check, 1 = check up to 'boundsCheckMaxSteps', 2 = check for every point",
 
 322                    'boundsCheckMaxSteps': "Last step to bounds check if checkBound==1. Defaults to 1000."
 
 323                    } 
 324  
 


325 -    def __init__(self, kw):


326          """Use the nobuild key to postpone building of the library, e.g. in
 
 327          order to provide additional build options to makeLibSource and
 
 328          compileLib methods or to make changes to the C code by hand.
 
 329          No build options can be specified otherwise.""" 
 330  
 
 331          if 'nobuild' in kw: 
 332              nobuild = kw['nobuild'] 
 333              # delete because not covered in ODEsystem
 
 334              del kw['nobuild'] 
 335          else: 
 336              nobuild = False 
 337          ODEsystem.__init__(self, kw) 
 338          self.diagnostics.outputStatsInfo = {
 
 339              'last_step': 'Predicted step size of the last accepted step (useful for a subsequent call to dop853).',
 
 340              'num_steps': 'Number of used steps.',
 
 341              'num_accept': 'Number of accepted steps.',
 
 342              'num_reject': 'Number of rejected steps.',
 
 343              'num_fcns': 'Number of function calls.',
 
 344              'errorStatus': 'Error status on completion.'
 
 345                          } 
 346          self.diagnostics._errorcodes = {
 
 347               0 : 'Unrecognized error code returned (see stderr output)',
 
 348              -1 : 'input is not consistent',
 
 349              -2 : 'larger nmax is needed',
 
 350              2 : 'larger nmax or maxevtpts is probably needed (error raised by solout)',
 
 351              -3 : 'step size becomes too small',
 
 352              -4 : 'the problem is probably stiff (interrupted)',
 
 353              -8 : 'The solution exceeded a magbound (poor choice of initial step)'} 
 354          self._solver = None 
 355          algparams_def = {'poly_interp': False,
 
 356                          'init_step': 0,
 
 357                          'max_step': 0,
 
 358                          'rtol': [1e-9 for i in range(self.dimension)],
 
 359                          'atol': [1e-12 for i in range(self.dimension)],
 
 360                          'fac1': 0.2,
 
 361                          'fac2': 10.0,
 
 362                          'safety': 0.9,
 
 363                          'beta': 0.04,
 
 364                          'max_pts': 10000,
 
 365                          'refine': 0,
 
 366                          'maxbisect': [], # for events
 
 367                          'maxevtpts': 1000, # for events
 
 368                          'eventInt': [],  # set using setEventInterval only
 
 369                          'eventDelay': [], # set using setEventDelay only
 
 370                          'eventTol': [], # set using setEventTol only
 
 371                          'use_special': 0,
 
 372                          'specialtimes': [],
 
 373                          'check_aux': 1,
 
 374                          'extraspace': 100,
 
 375                          'verbose': 0,
 
 376                          'hasJac': 0,
 
 377                          'hasJacP': 0,
 
 378                          'magBound': 1e7,
 
 379                          'boundsCheckMaxSteps': 1000,
 
 380                          'checkBounds': self.checklevel
 
 381                          } 
 382          for k, v in algparams_def.iteritems(): 
 383              if k not in self.algparams: 
 384                  self.algparams[k] = v 
 385          # verify that no additional keys are present in algparams, after
 
 386          # defaults are added above
 
 387          if len(self.algparams) != len(algparams_def): 
 388              raise ValueError("Invalid keys present in algparams argument: " \
 
 389                       + str(remain(self.algparams.keys(),algparams_def.keys()))) 
 390          thisplatform = platform.system() 
 391          if thisplatform == 'Windows': 
 392              self._dllext = ".pyd" 
 393          elif thisplatform in ['Linux', 'IRIX', 'Solaris', 'SunOS', 'MacOS', 'Darwin']: 
 394              self._dllext = '.so' 
 395          else: 
 396              print "Shared library extension not tested on this platform." 
 397              print "If this process fails please report the errors to the" 
 398              print "developers." 
 399              self._dllext = '.so' 
 400          self._compilation_tempdir = os.path.join(os.getcwd(),
 
 401                                                        "dopri853_temp") 
 402          if not os.path.isdir(self._compilation_tempdir): 
 403              try: 
 404                  assert not os.path.isfile(self._compilation_tempdir), \
 
 405                       "A file already exists with the same name" 
 406                  os.mkdir(self._compilation_tempdir) 
 407              except: 
 408                  print "Could not create compilation temp directory " + \
 
 409                        self._compilation_tempdir 
 410                  raise 
 411          self._compilation_sourcedir = os.path.join(_pydstool_path,"integrator") 
 412          self._vf_file = self.name+"_vf.c" 
 413          self._vf_filename_ext = "_"+self._vf_file[:-2] 
 414          self._prepareEventSpecs() 
 415          if not (os.path.isfile(os.path.join(os.getcwd(),
 
 416                                  "dop853"+self._vf_filename_ext+".py")) and \
 
 417                  os.path.isfile(os.path.join(os.getcwd(),
 
 418                                  "_dop853"+self._vf_filename_ext+self._dllext))): 
 419              if not nobuild: 
 420                  self.makeLibSource() 
 421                  self.compileLib() 
 422              else: 
 423                  print "Build the library using the makeLib method, or in " 
 424                  print "stages using the makeLibSource and compileLib methods." 
 425          self._inputVarList = [] 
 426          self._inputTimeList = []

 427  
 
 428  
 


429 -    def forceLibRefresh(self):


430          """forceLibRefresh should be called after event contents are changed,
 
 431          or alterations are made to the right-hand side of the ODEs.
 
 432  
 
 433          Currently this function does NOT work!""" 
 434  
 
 435          # (try to) free dopri module from namespace
 
 436          delfiles = True 
 437          self._solver = None 
 438          try: 
 439              del(sys.modules["_dop853"+self._vf_filename_ext]) 
 440              del(sys.modules["dop853"+self._vf_filename_ext]) 
 441  ##            del(self._integMod)
 
 442          except NameError: 
 443              # modules weren't loaded, so nothing to do
 
 444              delfiles = False 
 445          if delfiles: 
 446              gc.collect() 
 447              # still not able to delete these files!!!!! Argh!
 
 448  ##            if os.path.isfile(os.path.join(os.getcwd(),
 
 449  ##                                    "dop853"+self._vf_filename_ext+".py")):
 
 450  ##                os.remove(os.path.join(os.getcwd(),
 
 451  ##                                    "dop853"+self._vf_filename_ext+".py"))
 
 452  ##            if os.path.isfile(os.path.join(os.getcwd(),
 
 453  ##                                 "_dop853"+self._vf_filename_ext+self._dllext)):
 
 454  ##                os.remove(os.path.join(os.getcwd(),
 
 455  ##                                 "_dop853"+self._vf_filename_ext+self._dllext))
 
 456          print "Cannot rebuild library without restarting session. Sorry." 
 457          print "Try asking the Python developers to make a working module" 
 458          print "unimport function!"

 459  ##        self.makeLibSource()
 
 460  
 
 461  
 


462 -    def _prepareEventSpecs(self):


463          eventActive = [] 
 464          eventTerm = [] 
 465          eventDir = [] 
 466          eventDelay = [] 
 467          eventTol = [] 
 468          maxbisect = [] 
 469          eventInt = [] 
 470          # convert event specs (term, active, etc.) into integparam specs
 
 471          self._eventNames = self.eventstruct.sortedEventNames() 
 472          for evname in self._eventNames: 
 473              ev = self.eventstruct.events[evname] 
 474              assert isinstance(ev, LowLevelEvent), ("Dopri can only "
 
 475                                                  "accept low level events") 
 476          # if event 'precise' flags set to False then set their tolerances
 
 477          # to be > max_step
 
 478          maxstep = self.algparams['max_step'] 
 479          for evname in self._eventNames: 
 480              ev = self.eventstruct.events[evname] 
 481              eventActive.append(int(ev.activeFlag)) 
 482              eventTerm.append(int(ev.termFlag)) 
 483              eventDir.append(ev.dircode) 
 484              eventInt.append(ev.eventinterval) 
 485              eventDelay.append(ev.eventdelay) 
 486              if ev.preciseFlag: 
 487                  eventTol.append(ev.eventtol) 
 488                  maxbisect.append(ev.bisectlimit) 
 489              else: 
 490                  eventTol.append(maxstep*1.5) 
 491                  maxbisect.append(1) 
 492          self.algparams['eventTol'] = eventTol 
 493          self.algparams['eventDelay'] = eventDelay 
 494          self.algparams['eventInt'] = eventInt 
 495          self.algparams['maxbisect'] = maxbisect 
 496          self.algparams['eventActive'] = eventActive 
 497          self.algparams['eventTerm'] = eventTerm 
 498          self.algparams['eventDir'] = eventDir

 499  
 
 500  
 


501 -    def makeLib(self, libsources=[], libdirs=[], include=[]):


502          """makeLib calls makeLibSource and then the compileLib method.
 
 503          To postpone compilation of the source to a DLL, call makelibsource()
 
 504          separately.""" 
 505          self.makeLibSource(include) 
 506          self.compileLib(libsources, libdirs)

 507  
 
 508  
 


509 -    def makeLibSource(self, include=[]):


510          """makeLibSource generates the C source for the vector field specification.
 
 511          It should be called only once per vector field.""" 
 512  
 
 513          # Make vector field (and event) file for compilation
 
 514          assert isinstance(include, list), "includes must be in form of a list" 
 515          # codes for library types (default is USERLIB, since compiler will look in standard library d
 
 516          STDLIB = 0 
 517          USERLIB = 1 
 518          libinclude = dict([('Python.h', STDLIB), ('math.h', STDLIB), ('stdio.h', STDLIB),
 
 519                      ('stdlib.h', STDLIB), ('string.h', STDLIB), ('vfield.h', USERLIB),
 
 520                      ('events.h', USERLIB), ('signum.h', USERLIB), ('maxmin.h', USERLIB)]) 
 521          include_str = '' 
 522          for libstr, libtype in libinclude.iteritems(): 
 523              if libtype == STDLIB: 
 524                  quoteleft = '<' 
 525                  quoteright = '>' 
 526              else: 
 527                  quoteleft = '"' 
 528                  quoteright = '"' 
 529              include_str += "#include " + quoteleft + libstr + quoteright + "\n" 
 530          if include != []: 
 531              assert isUniqueSeq(include), "list of library includes must not contain repeats" 
 532              for libstr in include: 
 533                  if libstr in libinclude: 
 534                      # don't repeat libraries
 
 535                      print "Warning: library '" + libstr + "' already appears in list"\
 
 536                            + " of imported libraries" 
 537                  else: 
 538                      include_str += "#include " + '"' + libstr + '"\n' 
 539          allfilestr = "/*  Vector field function and events for Dopri853 integrator.\n " \
 
 540              + "  This code was automatically generated by PyDSTool, but may be modified " \
 
 541              + "by hand. */\n\n" + include_str + """
 
 542  extern double *gICs;
 
 543  extern double **gBds;
 
 544  extern double globalt0;
 
 545  
 
 546  static double pi = 3.1415926535897931;
 
 547  
 
 548  double signum(double x)
 
 549  {
 
 550    if (x<0) {
 
 551      return -1;
 
 552    }
 
 553    else if (x==0) {
 
 554      return 0;
 
 555    }
 
 556    else if (x>0) {
 
 557      return 1;
 
 558    }
 
 559    else {
 
 560      /* must be that x is Not-a-Number */
 
 561      return x;
 
 562    }
 
 563  }
 
 564  
 
 565  """ 
 566          pardefines = "" 
 567          vardefines = "" 
 568          auxvardefines = "" 
 569          inpdefines = "" 
 570          # sorted version of var, par, and input names
 
 571          vnames = self._var_ixmap 
 572          pnames = self.funcspec.pars 
 573          inames = self.funcspec.inputs 
 574          pnames.sort() 
 575          inames.sort() 
 576          for i in xrange(self.numpars): 
 577              p = pnames[i] 
 578              # add to defines
 
 579              pardefines += self.funcspec._defstr+" "+p+"\tp_["+str(i)+"]\n" 
 580          for i in xrange(self.dimension): 
 581              v = vnames[i] 
 582              # add to defines
 
 583              vardefines += self.funcspec._defstr+" "+v+"\tY_["+str(i)+"]\n" 
 584          for i, v in enumerate(self.funcspec.auxvars): 
 585              auxvardefines += self.funcspec._defstr+" "+v+"\t("+self.funcspec._auxdefs_parsed[v]+")\n" 
 586          for i in xrange(len(self.funcspec.inputs)): 
 587              inp = inames[i] 
 588              # add to defines
 
 589              inpdefines += self.funcspec._defstr+" "+inp+"\txv_["+str(i)+"]\n" 
 590          allfilestr += "\n/* Variable, aux variable, parameter, and input definitions: */ \n" \
 
 591                        + pardefines + vardefines + auxvardefines + inpdefines + "\n" 
 592          # preprocess event code
 
 593          allevs = "" 
 594          if self._eventNames == []: 
 595              numevs = 0 
 596          else: 
 597              numevs = len(self._eventNames) 
 598          for evname in self._eventNames: 
 599              ev = self.eventstruct.events[evname] 
 600              evfullfn = "" 
 601              assert isinstance(ev, LowLevelEvent), ("Dopri can only "
 
 602                                                  "accept low level events") 
 603              evsig = ev._LLreturnstr + " " + ev.name + ev._LLargstr 
 604              assert ev._LLfuncstr.index(';') > 1, ("Event function code "
 
 605                      "error: Have you included a ';' character at the end of"
 
 606                                              "your 'return' statement?") 
 607              fbody = ev._LLfuncstr 
 608              # check fbody for calls to user-defined aux fns
 
 609              # and add hidden p argument
 
 610              if self.funcspec.auxfns: 
 611                  fbody_parsed = addArgToCalls(fbody,
 
 612                                          self.funcspec.auxfns.keys(),
 
 613                                          "p_, wk_, xv_") 
 614                  if 'initcond' in self.funcspec.auxfns: 
 615                      # convert 'initcond(x)' to 'initcond("x")' for
 
 616                      # compatibility with C syntax
 
 617                      fbody_parsed = wrapArgInCall(fbody_parsed,
 
 618                                          'initcond', '"') 
 619              else: 
 620                  fbody_parsed = fbody 
 621              evbody = " {\n" + fbody_parsed + "\n}\n\n" 
 622              allevs += evsig + evbody 
 623              allfilestr += evsig + ";\n" 
 624          # add signature for auxiliary functions
 
 625          if self.funcspec.auxfns: 
 626              allfilestr += "\n" 
 627              for finfo in self.funcspec.auxfns.values(): 
 628                  allfilestr += finfo[1] + ";\n" 
 629          assignEvBody = "" 
 630          for evix in range(numevs): 
 631              assignEvBody += "events[%d] = &%s;\n"%(evix,self._eventNames[evix]) 
 632          allfilestr += "\nint N_EVENTS = " + str(numevs) + ";\nvoid assignEvents(" \
 
 633                + "EvFunType *events){\n " + assignEvBody  \
 
 634                + "\n}\n\nvoid auxvars(unsigned, unsigned, double, double*, double*, " \
 
 635                + "double*, unsigned, double*, unsigned, double*);\n" \
 
 636                + """void jacobian(unsigned, unsigned, double, double*, double*, double**, unsigned, double*, unsigned, double*);
 
 637  void jacobianParam(unsigned, unsigned, double, double*, double*, double**, unsigned, double*, unsigned, double*);
 
 638  """ 
 639          if self.funcspec.auxvars == []: 
 640              allfilestr += "int N_AUXVARS = 0;\n\n\n" 
 641          else: 
 642              allfilestr += "int N_AUXVARS = " + str(len(self.funcspec.auxvars)) \
 
 643                         + ";\n\n\n" 
 644          if self.funcspec.inputs == []: 
 645              allfilestr += "int N_EXTINPUTS = 0;\n\n\n" 
 646          else: 
 647              allfilestr += "int N_EXTINPUTS = " + str(len(self.funcspec.inputs)) \
 
 648                         + ";\n\n\n" 
 649          allfilestr += self.funcspec.spec[0] + "\n\n" 
 650          if self.funcspec.auxfns: 
 651              for fname, finfo in self.funcspec.auxfns.iteritems(): 
 652                  fbody = finfo[0] 
 653                  # subs _p into auxfn-to-auxfn calls (but not to the signature)
 
 654                  fbody_parsed = addArgToCalls(fbody,
 
 655                                          self.funcspec.auxfns.keys(),
 
 656                                          "p_, wk_, xv_", notFirst=fname) 
 657                  if 'initcond' in self.funcspec.auxfns: 
 658                      # convert 'initcond(x)' to 'initcond("x")' for
 
 659                      # compatibility with C syntax, but don't affect the
 
 660                      # function signature!
 
 661                      fbody_parsed = wrapArgInCall(fbody_parsed,
 
 662                                          'initcond', '"', notFirst=True) 
 663                  allfilestr += "\n" + fbody_parsed + "\n\n" 
 664          # add auxiliary variables (shell of the function always present)
 
 665          # add event functions
 
 666          allfilestr += self.funcspec.auxspec[0] + allevs 
 667          # if jacobians or mass matrix not present, fill in dummy
 
 668          if self.haveMass(): 
 669              raise ValueError("Mass matrix declaration is incompatible with "
 
 670                               "Dopri integrator system specification") 
 671          else: 
 672              allfilestr += """
 
 673  void massMatrix(unsigned n_, unsigned np_, double t, double *Y_, double *p_, double **f_, unsigned wkn_, double *wk_, unsigned xvn_, double *xv_) {
 
 674  }
 
 675  """ 
 676          if not self.haveJacobian(): 
 677              allfilestr += """
 
 678  void jacobian(unsigned n_, unsigned np_, double t, double *Y_, double *p_, double **f_, unsigned wkn_, double *wk_, unsigned xvn_, double *xv_) {
 
 679  }
 
 680  """ 
 681          if not self.haveJacobian_pars(): 
 682              allfilestr += """
 
 683  void jacobianParam(unsigned n_, unsigned np_, double t, double *Y_, double *p_, double **f_, unsigned wkn_, double *wk_, unsigned xvn_, double *xv_) {
 
 684  }
 
 685  """ #+ "\n/* Variable and parameter substitutions undefined:*/\n" + parundefines + varundefines + "\n" 
 686          # write out C file
 
 687          vffile = os.path.join(self._compilation_tempdir, self._vf_file) 
 688          try: 
 689              file = open(vffile, 'w') 
 690              file.write(allfilestr) 
 691              file.close() 
 692          except IOError, e: 
 693              print "Error opening file "+self._vf_file+" for writing" 
 694              raise IOError, e

 695  
 
 696  
 


697 -    def compileLib(self, libsources=[], libdirs=[]):


698          """compileLib generates a python extension DLL with integrator and vector
 
 699          field compiled and linked.
 
 700  
 
 701          libsources list allows additional library sources to be linked.
 
 702          libdirs list allows additional directories to be searched for
 
 703            precompiled libraries.""" 
 704  
 
 705          if os.path.isfile(os.path.join(os.getcwd(),
 
 706                                  "_dop853"+self._vf_filename_ext+self._dllext)): 
 707              # then DLL file already exists and we can't overwrite it at this
 
 708              # time
 
 709              proceed = False 
 710              print "\n" 
 711              print "-----------------------------------------------------------" 
 712              print "Present limitation of Python: Cannot rebuild library" 
 713              print "without exiting Python and deleting the shared library" 
 714              print "   " + str(os.path.join(os.getcwd(),
 
 715                                  "_dop853"+self._vf_filename_ext+self._dllext)) 
 716              print "by hand! If you made any changes to the system you should" 
 717              print "not proceed with running the integrator until you quit" 
 718              print "and rebuild." 
 719              print "-----------------------------------------------------------" 
 720              print "\n" 
 721          else: 
 722              proceed = True 
 723          if not proceed: 
 724              print "Did not compile shared library." 
 725              return 
 726          if self._solver is not None: 
 727              self.forceLibRefresh() 
 728          vffile = os.path.join(self._compilation_tempdir, self._vf_file) 
 729          try: 
 730              ifacefile_orig = open(os.path.join(self._compilation_sourcedir,
 
 731                                                 "dop853.i"), 'r') 
 732              ifacefile_copy = open(os.path.join(self._compilation_tempdir,
 
 733                                         "dop853_"+self._vf_file[:-2]+".i"), 'w') 
 734              firstline = ifacefile_orig.readline() 
 735              ifacefile_copy.write('%module dop853_'+self._vf_file[:-2]+'\n') 
 736              iffilestr = ifacefile_orig.read() 
 737              ifacefile_copy.write(iffilestr) 
 738              ifacefile_orig.close() 
 739              ifacefile_copy.close() 
 740          except IOError: 
 741              print "dop853.i copying error in dopri853 compilation directory" 
 742              raise 
 743          swigfile = os.path.join(self._compilation_tempdir,
 
 744                                  "dop853"+self._vf_filename_ext+".i") 
 745          dopwrapfile = os.path.join(self._compilation_sourcedir, "dop853mod.c") 
 746          dopfile = os.path.join(self._compilation_sourcedir, "dop853.c") 
 747          integfile = os.path.join(self._compilation_sourcedir, "integration.c") 
 748          interfacefile = os.path.join(self._compilation_sourcedir, "interface.c") 
 749          eventfile = os.path.join(self._compilation_sourcedir, "eventFinding.c") 
 750          memfile = os.path.join(self._compilation_sourcedir, "memory.c") 
 751          # The following if statement attempts to avoid recompiling the SWIG wrapper
 
 752          # if the files mentioned already exist, because in principle the SWIG interface
 
 753          # only needs compiling once. But this step doesn't seem to work yet.
 
 754          # Instead, it seems that SWIG always gets recompiled with everything else
 
 755          # (at least on Win32). Maybe the list of files is incorrect...
 
 756          if not (all([os.path.isfile(os.path.join(self._compilation_tempdir,
 
 757                             sf)) for sf in ['dop853'+self._vf_filename_ext+'_wrap.o',
 
 758                                             'lib_dop853'+self._vf_filename_ext+'.a',
 
 759                                             'dop853'+self._vf_filename_ext+'.py',
 
 760                                             '_dop853'+self._vf_filename_ext+'.def']])): 
 761              modfilelist = [swigfile] 
 762          else: 
 763              modfilelist = [] 
 764          modfilelist.extend([dopwrapfile, dopfile, vffile, integfile, eventfile,
 
 765                             interfacefile, memfile]) 
 766          modfilelist.extend(libsources) 
 767          script_args = ['-q', 'build', '--build-lib=.', #+os.getcwd(), # '-t/',
 
 768                   '-tdopri853_temp',#+self._compilation_tempdir,
 
 769                   '--build-base=dopri853_temp', '--build-purelib=dopri853_temp']#+self._compilation_sourcedir] 
 770          #script_args = ['-q', 'build', '--build-lib='+os.getcwd(), '-t/',
 
 771          #               '--build-base='+self._compilation_tempdir]
 
 772          if self._compiler != '': 
 773              script_args.append('-c'+str(self._compiler)) 
 774  
 
 775          # include directories for libraries
 
 776          narraydir = npy.get_numarray_include() 
 777          npydir = npy.get_include() 
 778  
 
 779          incdirs = [npydir, narraydir, os.getcwd(), self._compilation_sourcedir] #_compilation_tempdir] 
 780          incdirs.extend(libdirs) 
 781          # Use distutils to perform the compilation of the selected files
 
 782          try: 
 783              distobject = setup(name = "Dopri 853 integrator",
 
 784                    author = "PyDSTool (automatically generated)",
 
 785                    script_args = script_args,
 
 786                    ext_modules = [Extension("_dop853"+self._vf_filename_ext,
 
 787                                   sources=modfilelist,
 
 788                                   include_dirs=incdirs,
 
 789  #                                 library_dirs=['./'],
 
 790                                   extra_compile_args=['-w', '-D__DOPRI__', '-m32'],
 
 791                                   extra_link_args=['-w', '-m32'])]) 
 792          except: 
 793              print "\nError occurred in generating Dopri system..." 
 794              print sys.exc_info()[0], sys.exc_info()[1] 
 795              raise RuntimeError 
 796          # Attempt to unload module through a shutdown() function being
 
 797          # added to the SWIG module file. But it didn't work!
 
 798  ##        try:
 
 799  ##            modfilepy = open(os.path.join(self._compilation_tempdir,
 
 800  ##                                    "dop853"+self._vf_filename_ext+".py"), 'a')
 
 801  ##            extfilename = "_dop853"+self._vf_filename_ext
 
 802  ##            modfilepy.write("""# The following addition made by PyDSTool:
 
 803  ##def shutdown():
 
 804  ##    import sys
 
 805  ##    del sys.modules['""" + extfilename + """']
 
 806  ##            """)
 
 807  ####    del """ + extfilename + """
 
 808  ####    del new_doubleArray
 
 809  ####    del delete_doubleArray
 
 810  ####    del doubleArray_getitem
 
 811  ####    del doubleArray_setitem
 
 812  ####    del new_intArray
 
 813  ####    del delete_intArray
 
 814  ####    del intArray_getitem
 
 815  ####    del intArray_setitem
 
 816  ####    del Integrate
 
 817  ##            modfilepy.close()
 
 818  ##        except IOError:
 
 819  ##            print "dop853.py modifying error in dopri853 temp compilation " \
 
 820  ##                  + "directory"
 
 821  ##            raise
 
 822          rout.start()    # redirect stdout 
 823          try: 
 824              # move library files into the user's CWD
 
 825              distdestdir = distutil_destination() 
 826              if swigfile in modfilelist or not \
 
 827                 os.path.isfile(os.path.join(self._compilation_tempdir,
 
 828                                  "dop853"+self._vf_filename_ext+".py")): 
 829                  # temporary hack to fix numpy_distutils bug
 
 830                  shutil.move(os.path.join(os.getcwd(),
 
 831                                    self._compilation_tempdir, distdestdir,
 
 832                                    "dopri853_temp",
 
 833                                   "dop853"+self._vf_filename_ext+".py"),
 
 834                              os.path.join(os.getcwd(),
 
 835                                   "dop853"+self._vf_filename_ext+".py")) 
 836          except: 
 837              rout.stop() 
 838              print "\nError occurred in generating Dopri system" 
 839              print "(while moving library extension modules to CWD)" 
 840              print sys.exc_info()[0], sys.exc_info()[1] 
 841              raise RuntimeError 
 842          rout.stop()    # restore stdout

 843  
 
 844  
 
 845  #    def _ensureLoaded(self, modname):
 
 846  ##        if modname in sys.modules:
 
 847  ##            _integMod = reload(sys.modules[modname])
 
 848  ##        else:
 
 849  #        try:
 
 850  #            _integMod = __import__(modname, globals())
 
 851  #        except:
 
 852  #            print "Error in importing compiled vector field and integrator."
 
 853  #            print "Did you compile the RHS C code?"
 
 854  #            raise
 
 855  #        # Initialize integrator
 
 856  #        assert 'Integrate' in dir(_integMod), \
 
 857  #               "dopri853 library does not contain Integrate()"
 
 858  #        return _integMod
 
 859  
 
 860  
 


861 -    def compute(self, trajname, dirn='f', ics=None):


862          continue_integ = ODEsystem.prepDirection(self, dirn) 
 863  #        _integMod = __import__("dop853"+self._vf_filename_ext, globals())
 
 864          if ics is not None: 
 865              self.set(ics=ics) 
 866          self.validateICs() 
 867          self.diagnostics.clearWarnings() 
 868          self.diagnostics.clearErrors() 
 869          if isinstance(self.algparams['rtol'], list): 
 870              if len(self.algparams['rtol']) != self.dimension: 
 871                  raise ValueError('rtol list must have same length as phase dimension') 
 872          else: 
 873              rtol = self.algparams['rtol'] 
 874              self.algparams['rtol'] = [rtol for dimix in xrange(self.dimension)] 
 875          if isinstance(self.algparams['atol'], list): 
 876              if len(self.algparams['atol']) != self.dimension: 
 877                  raise ValueError('atol list must have same length as phase dimension') 
 878          else: 
 879              atol = self.algparams['atol'] 
 880              self.algparams['atol'] = [atol for dimix in xrange(self.dimension)] 
 881          anames = self.funcspec.auxvars 
 882          # Check i.c.'s are well defined (finite)
 
 883          self.checkInitialConditions() 
 884          self.setEventICs(self.initialconditions, self.globalt0) 
 885          # update event params in case changed since last run
 
 886          self._prepareEventSpecs() 
 887          # Main integration
 
 888          t0 = self.indepvariable.depdomain[0] 
 889          t1 = self.indepvariable.depdomain[1] 
 890          plist = sortedDictValues(self.pars) 
 891          self.algparams['hasJac'] = self.haveJacobian() 
 892          self.algparams['hasJacP'] = self.haveJacobian_pars() 
 893          if self._solver is None: 
 894  #            _integMod = self._ensureLoaded("dop853"+self._vf_filename_ext)
 
 895              self._solver = dopri("dop853"+self._vf_filename_ext,
 
 896                                   rhs=self.name, phaseDim=self.dimension,
 
 897                                   paramDim=len(plist), nAux=len(anames),
 
 898                                   nEvents=len(self._eventNames),
 
 899                                   nExtInputs=len(self.inputs),
 
 900                                   hasJac=self.algparams['hasJac'],
 
 901                                   hasJacP=self.algparams['hasJacP'],
 
 902                                   hasMass=self.haveMass(),
 
 903                                   extraSpace=self.algparams['extraspace'],
 
 904                                   ) 
 905              try: 
 906                  genDB.register(self) 
 907              except PyDSTool_KeyError: 
 908                  errstr = "Generator " + self.name + ": this vector field's " +\
 
 909                           "DLL is already in use" 
 910                  raise RuntimeError(errstr) 
 911          if self._dircode == 1: 
 912              tbegin = t0 
 913              tend = t1 
 914          elif self._dircode == -1: 
 915              # dopri does reverse time integration simply by switching t0 and t1
 
 916              # and using negative steps
 
 917              tbegin = t1 
 918              tend = t0 
 919          if len(self.algparams['specialtimes'])>0: 
 920              use_special = self.algparams['use_special'] 
 921          else: 
 922              use_special = 0 
 923          bounds = [[],[]]  # lower, then upper 
 924          for v in self.funcspec.vars: 
 925              bds = self.xdomain[v] 
 926              bounds[0].append(bds[0]) 
 927              bounds[1].append(bds[1]) 
 928          for p in self.funcspec.pars: 
 929              bds = self.pdomain[p] 
 930              bounds[0].append(bds[0]) 
 931              bounds[1].append(bds[1]) 
 932          if continue_integ: 
 933              x0 = self._solver.lastPoint 
 934              # overwrite t0 from self.indepvariable.domain, but use its t1
 
 935              tbegin = self._solver.lastTime 
 936              if abs(self._solver.lastStep) < abs(self.algparams['init_step']): 
 937                  self.algparams['init_step'] = self._solver.lastStep 
 938              if abs(t1-tbegin) < abs(self.algparams['init_step']): 
 939                  raise ValueError("Integration end point too close to initial "
 
 940                                   "point") 
 941  #            if self.inputs and self._extInputsChanged:
 
 942  #                self._extInputsChanged = False
 
 943  #                self._solver.setContParams(tend, plist,
 
 944  #                                           use_special,
 
 945  #                                           self.algparams['verbose'],
 
 946  #                                           True, deepcopy(self._inputVarList),
 
 947  #                                           deepcopy(self._inputTimeList))
 
 948          else: 
 949              if self._solver.numRuns > 0: 
 950                  self._solver.clearAll() 
 951              x0 = sortedDictValues(self.initialconditions, self.funcspec.vars) 
 952              self._solver.setInteg(maxpts=self.algparams['max_pts'],
 
 953                  rtol=self.algparams['rtol'], atol=self.algparams['atol']) 
 954              self._solver.setRunParams(ic=x0, params=plist,
 
 955                                    t0=tbegin, tend=tend, gt0=self.globalt0,
 
 956                                    refine=self.algparams['refine'],
 
 957                                    specTimes=self.algparams['specialtimes'],
 
 958                                    bounds=bounds) 
 959          if self.inputs: 
 960              # self._extInputsChanged if global t0 changed so that can
 
 961              # adjust times given to the integrator (it is blind to global t0
 
 962              # when accesses input variable times)
 
 963              self._ensure_inputs(self._extInputsChanged) 
 964          # hinit only set if not continue_integ
 
 965          if len(anames)>0: 
 966              check_aux = self.algparams['check_aux'] 
 967          else: 
 968              check_aux = 0 
 969          if self.algparams['max_step'] == 0: 
 970              max_step = tend-tbegin 
 971          else: 
 972              max_step = self.algparams['max_step'] 
 973          init_step = self.algparams['init_step'] 
 974          if self._dircode == 1: 
 975              if init_step < 0: 
 976                  init_step = -init_step 
 977              if max_step < 0: 
 978                  max_step = -max_step 
 979          else: 
 980              if init_step > 0: 
 981                  init_step = -init_step 
 982              if max_step > 0: 
 983                  max_step = -max_step 
 984          if continue_integ: 
 985              # record needed for bounds checking and truncation
 
 986              old_highest_ix = self._solver.points.shape[1] 
 987              alltData, X, A, Stats, H, Err, Evtimes, \
 
 988                   Evpoints = self._solver.Continue(tend, plist,
 
 989                                    use_special, self.algparams['verbose'],
 
 990                                    self._extInputsChanged,
 
 991                                    deepcopy(self._inputVarList),
 
 992                                    deepcopy(self._inputTimeList),
 
 993                                    bounds) 
 994          else: 
 995              old_highest_ix = 0 
 996              self._solver.setEvents(eventActive=self.algparams['eventActive'],
 
 997                  eventTerm=self.algparams['eventTerm'],
 
 998                  eventDir=self.algparams['eventDir'],
 
 999                  eventDelay=self.algparams['eventDelay'],
 
1000                  eventInt=self.algparams['eventInt'],
 
1001                  eventTol=self.algparams['eventTol'],
 
1002                  maxevtpts=self.algparams['maxevtpts'],
 
1003                  maxbisect=self.algparams['maxbisect']) 
1004              alltData, X, A, Stats, H, Err, Evtimes, \
 
1005                   Evpoints = self._solver.Run(init_step,
 
1006                                      max_step,
 
1007                                      check_aux,
 
1008                                      use_special,
 
1009                                      self.algparams['verbose'],
 
1010                                      self.algparams['fac1'],
 
1011                                      self.algparams['fac2'],
 
1012                                      self.algparams['safety'],
 
1013                                      self.algparams['beta'],
 
1014                                      self.algparams['checkBounds'],
 
1015                                      self.algparams['boundsCheckMaxSteps'],
 
1016                                      self.algparams['magBound']) 
1017          self._extInputsChanged = False    # reset this now 
1018          self.diagnostics.outputStats = {'last_step': H,
 
1019                              'last_time': self._solver.lastTime,
 
1020                              'last_point': self._solver.lastPoint,
 
1021                              'num_fcns': Stats[0],
 
1022                              'num_steps': Stats[1],
 
1023                              'num_accept': Stats[2],
 
1024                              'num_reject': Stats[3],
 
1025                              'errorStatus': Err
 
1026                              } 
1027          if self._dircode == -1: 
1028              # reverse the array object (no reverse method!)
 
1029              alltData = alltData[::-1] 
1030              X = X[:,::-1] 
1031              if anames != []: 
1032                  A = A[:,::-1] 
1033          xnames = self._var_ixmap 
1034          # Package up computed trajectory in Variable variables
 
1035          # Add external inputs warnings to self.diagnostics.warnings, if any
 
1036          # (not presently supported)
 
1037  ##        for f in inputVarList:
 
1038  ##            for winfo in f.diagnostics.warnings:
 
1039  ##                self.diagnostics.warnings.append((W_NONTERMSTATEBD,
 
1040  ##                                     (winfo[0], f.name, winfo[1],
 
1041  ##                                      f.depdomain)))
 
1042          eventslist = self.eventstruct.query(['lowlevel', 'active']) 
1043          termevents = self.eventstruct.query(['term'], eventslist) 
1044          if self._eventNames != []: 
1045              # build self.diagnostics.warnings because events happened --
 
1046              # and keep a record of which times terminal events happened because
 
1047              # Model.py's event handling procedure assumes multiple events
 
1048              # happening at one time are listed in one warning
 
1049              termevtimes = {} 
1050              nontermevtimes = {} 
1051              try: 
1052                  for evix in range(len(self._eventNames)): 
1053                      if Evpoints[evix] is None: 
1054                          continue 
1055                      evname = self._eventNames[evix] 
1056                      numevs = len(Evtimes[evix]) 
1057                      if self.algparams['eventTerm'][evix]: 
1058                          if numevs > 1: 
1059                              print "Event info:", Evpoints, Evtimes 
1060                          assert numevs <= 1, ("Internal error: more than one "
 
1061                                           "terminal event of same type found") 
1062                          # For safety, we should assert that this event
 
1063                          # also appears in termevents, but we don't
 
1064                          if Evtimes[evix][0] in termevtimes.keys(): 
1065                              # append event name to this warning
 
1066                              warning_ix = termevtimes[Evtimes[evix][0]] 
1067                              self.diagnostics.warnings[warning_ix][1][1].append(evname) 
1068                          else: 
1069                              # make new termevtime entry for the new warning
 
1070                              termevtimes[Evtimes[evix][0]] = len(self.diagnostics.warnings) 
1071                              self.diagnostics.warnings.append((W_TERMEVENT,
 
1072                                               (Evtimes[evix][0],
 
1073                                               [self._eventNames[evix]]))) 
1074                      else: 
1075                          for ev in range(numevs): 
1076                              if Evtimes[evix][ev] in nontermevtimes.keys(): 
1077                                  # append event name to this warning
 
1078                                  warning_ix = nontermevtimes[Evtimes[evix][ev]] 
1079                                  self.diagnostics.warnings[warning_ix][1][1].append(evname) 
1080                              else: 
1081                                  # make new nontermevtime entry for the new warning
 
1082                                  nontermevtimes[Evtimes[evix][ev]] = \
 
1083                                                              len(self.diagnostics.warnings) 
1084                                  self.diagnostics.warnings.append((W_NONTERMEVENT,
 
1085                                                   (Evtimes[evix][ev],
 
1086                                                    [evname]))) 
1087              except IndexError: 
1088                  print "Events returned from integrator are the wrong size." 
1089                  print "  Did you change the system and not refresh the C " \
 
1090                        + "library using the forcelibrefresh() method?" 
1091                  raise 
1092          termcount = 0 
1093          for (w,i) in self.diagnostics.warnings: 
1094              if w == W_TERMEVENT or w == W_TERMSTATEBD: 
1095                  if termcount > 0: 
1096                      raise ValueError("Internal error: more than one terminal "
 
1097                                       "event found") 
1098                  termcount += 1 
1099          # post-process check of variable bounds (if defined and algparams['checkBounds'] True)
 
1100          if self._dircode > 0: 
1101              compare = operator.lt 
1102              last_ix = Inf 
1103          else: 
1104              compare = operator.gt 
1105              last_ix = -Inf 
1106          highest_ix = X.shape[1]-1 
1107          last_t = Inf 
1108          if self.algparams['checkBounds'] > 0: 
1109              # temp storage for repeatedly used object attributes (for lookup efficiency)
 
1110              depdomains = dict(zip(range(self.dimension),
 
1111                          [self.variables[xn].depdomain for xn in xnames])) 
1112              offender_ix = None 
1113              for xi in xrange(self.dimension): 
1114                  if not any(depdomains[xi].isfinite()): 
1115                      # no point in checking when the bounds are +/- infinity
 
1116                      continue 
1117                  next_last_ix = array_bounds_check(X[xi][old_highest_ix:],
 
1118                                      depdomains[xi], self._dircode) + old_highest_ix 
1119                  if compare(next_last_ix, last_ix): 
1120                      # won't count as truncating unless the following checks
 
1121                      # hold
 
1122                      last_ix = next_last_ix 
1123                      offender_ix = xi 
1124              if not isfinite(last_ix) and last_ix < 0: 
1125                  # only use +Inf hereon to flag no truncation needed
 
1126                  last_ix = Inf 
1127              elif last_ix >= 0 and last_ix < highest_ix: 
1128                  # truncate data
 
1129                  last_t = alltData[last_ix] 
1130                  print "Warning; domain bound reached (because algparams['checkBounds'] > 0)" 
1131                  self.diagnostics.warnings.append((W_TERMSTATEBD,
 
1132                                      (last_t, xnames[offender_ix],
 
1133                                       X[offender_ix, last_ix],
 
1134                                       depdomains[offender_ix].get()))) 
1135          # Create variables (self.variables contains no actual data)
 
1136          variables = copyVarDict(self.variables) 
1137          # build event pointset information (reset previous trajectory's)
 
1138          # don't include events after any truncation due to state bound violation
 
1139          self.trajevents = {} 
1140          for evix in range(len(self._eventNames)): 
1141              evname = self._eventNames[evix] 
1142              if Evpoints[evix] is None: 
1143                  self.trajevents[evname] = None 
1144              else: 
1145                  try: 
1146                      ev_a_list = [] 
1147                      for t in Evtimes[evix]: 
1148                          tix = find(alltData, t) 
1149                          ev_a_list.append(A[:,tix]) 
1150                      ev_array = concatenate((Evpoints[evix],
 
1151                                           transpose(array(ev_a_list, 'd')))) 
1152                      del ev_a_list, tix 
1153                  except TypeError: 
1154                      # A is empty
 
1155                      ev_array = Evpoints[evix] 
1156                  if last_ix >= 0 and last_ix < highest_ix: 
1157                      # don't count last_ix = -1 which is the same as highest_ix
 
1158                      last_ev_tix = npy.argmax(Evtimes[evix] >= alltData[last_ix]) 
1159                      if last_ev_tix == 0 and Evtimes[evix][0] >= last_t: 
1160                          # checks that there was actually a violation
 
1161                          # - so no events to record
 
1162                          self.trajevents[evname] = None 
1163                      else: 
1164                          # truncation needed
 
1165                          ev_array = ev_array[:, :last_ev_tix+1] 
1166                          ev_times = Evtimes[evix][:last_ev_tix+1] 
1167                          self.trajevents[evname] = Pointset({'coordnames': xnames+anames,
 
1168                                                 'indepvarname': 't',
 
1169                                                 'coordarray': ev_array,
 
1170                                                 'indepvararray': ev_times}) 
1171                  else: 
1172                      # no truncation needed
 
1173                      self.trajevents[evname] = Pointset({'coordnames': xnames+anames,
 
1174                                                 'indepvarname': 't',
 
1175                                                 'coordarray': ev_array,
 
1176                                                 'indepvararray': Evtimes[evix]}) 
1177          if last_ix >= 0 and last_ix < highest_ix: 
1178              # truncate
 
1179              X = X[:, :last_ix] 
1180              alltData = alltData[:last_ix] 
1181          try: 
1182              allxDataDict = dict(zip(xnames,X)) 
1183          except IndexError: 
1184              print "Integration returned variable values of unexpected dimensions." 
1185              print "  Did you change the system and not refresh the C library" \
 
1186                    + " using the forcelibrefresh() method?" 
1187              raise 
1188          # storage of all auxiliary variable data
 
1189          try: 
1190              if anames != []: 
1191                  if last_ix < highest_ix: 
1192                      A = A[:, :last_ix] 
1193                  try: 
1194                      allaDataDict = dict(zip(anames,A)) 
1195                  except TypeError: 
1196                      print "Internal error!  Type of A: ", type(A) 
1197                      raise 
1198          except IndexError: 
1199              print "Integration returned auxiliary values of unexpected dimensions." 
1200              print "  Did you change the system and not refresh the C library" \
 
1201                    + " using the forcelibrefresh() method?" 
1202              raise 
1203          if int(Err) == 1 or (int(Err) == 2 and termcount == 1): 
1204              # output OK
 
1205              if self.algparams['poly_interp']: 
1206                  rhsfn = self._solver.Rhs 
1207                  # when Dopri can output the Rhs values alongside variable
 
1208                  # values then this won't be necessary
 
1209                  dxvals = zeros((len(alltData),self.dimension),float) 
1210                  for tix, tval in enumerate(alltData): 
1211                      # solver's Rhs function already contains the inputs so no
 
1212                      # need to recompute and provide here.
 
1213                      #i = _pollInputs(sortedDictValues(self.inputs), tval,
 
1214                      #                        self.checklevel)
 
1215                      # X is the output variable array, but rhsfn demands a list
 
1216                      dxvals[tix] = rhsfn(tval, list(X[:,tix]), plist)[0] 
1217              for xi, x in enumerate(xnames): 
1218                  if len(alltData) > 1: 
1219                      if self.algparams['poly_interp']: 
1220                          interp = PiecewisePolynomial(alltData,
 
1221                                      array([allxDataDict[x], dxvals[:,xi]]).T, 2) 
1222                      else: 
1223                          interp = interp1d(alltData, allxDataDict[x]) 
1224                      variables[x] = Variable(interp, 't', x, x) 
1225                  else: 
1226                      raise PyDSTool_ValueError("Fewer than 2 data points "
 
1227                                                "computed") 
1228              for a in anames: 
1229                  if len(alltData) > 1: 
1230                      variables[a] = Variable(interp1d(alltData,allaDataDict[a]),
 
1231                                               't', a, a) 
1232                  else: 
1233                      raise PyDSTool_ValueError("Fewer than 2 data points "
 
1234                                                "computed") 
1235              # final checks
 
1236              #self.validateSpec()
 
1237              self.defined = True 
1238              return Trajectory(trajname, variables.values(),
 
1239                                abseps=self._abseps, globalt0=self.globalt0,
 
1240                                checklevel=self.checklevel,
 
1241                                FScompatibleNames=self._FScompatibleNames,
 
1242                                FScompatibleNamesInv=self._FScompatibleNamesInv,
 
1243                                modelNames=self.name, events=self.trajevents,
 
1244                                modelEventStructs=self.eventstruct) 
1245          else: 
1246              try: 
1247                  diagnost_info = self.diagnostics._errorcodes[int(Err)] 
1248              except TypeError: 
1249                  # errcode messed up from Dopri
 
1250                  print "Error code: ", Err 
1251                  diagnost_info = self.diagnostics._errorcodes[0] 
1252              if self._solver.verbose: 
1253                  info(self.diagnostics.outputStats, "Output statistics") 
1254              self.defined = False 
1255              # Did the solver run out of memory?
 
1256              if (len(alltData) == self.algparams['max_pts'] or \
 
1257                  self.diagnostics.outputStats['num_steps'] >= self.algparams['max_pts']) \
 
1258                     and alltData[-1] < tend: 
1259                  print "max_pts algorithmic parameter too small: current " + \
 
1260                        "value is %i"%self.algparams['max_pts'] 
1261  #                avstep = (self.algparams['init_step']+self.diagnostics.outputStats['last_step'])/2.
 
1262                  if self.diagnostics.outputStats['last_time']-tbegin > 0: 
1263                      ms = int(round(self.algparams['max_pts'] / \
 
1264                                (self.diagnostics.outputStats['last_time'] - \
 
1265                                 tbegin)*(tend-tbegin))) 
1266                  else: 
1267                      ms = Inf 
1268                  print "(recommended value for this trajectory segment is " + \
 
1269                        "estimated to be %s (saved in diagnostics.errors attribute))"%str(ms) 
1270                  diagnost_info += " -- recommended value is %i" % ms 
1271              self.diagnostics.errors.append((E_COMPUTFAIL,
 
1272                                      (self._solver.lastTime, diagnost_info))) 
1273              raise PyDSTool_ExistError("No trajectory created")

1274  
 
1275  
 


1276 -    def Rhs(self, t, xdict, pdict=None, asarray=True):


1277          """asarray is an unused, dummy argument for compatibility with Model.Rhs""" 
1278          # don't need to convert names to FS-compatible as they sort
 
1279          # the same
 
1280          x = sortedDictValues(filteredDict(xdict, self.funcspec.vars)) 
1281          if pdict is None: 
1282              pdict = self.pars 
1283          p = sortedDictValues(pdict) 
1284          i = _pollInputs(sortedDictValues(self.inputs), t, self.checklevel) 
1285          self._ensure_solver({'params': p, 't0': 0, 'tend': 1}) 
1286          self._ensure_inputs() 
1287          return self._solver.Rhs(t, x, p+i)[0]

1288  
 
1289  
 


1290 -    def Jacobian(self, t, xdict, pdict=None, asarray=True):


1291          """asarray is an unused, dummy argument for compatibility with
 
1292          Model.Jacobian""" 
1293          if self.haveJacobian(): 
1294              x = sortedDictValues(filteredDict(xdict, self.funcspec.vars)) 
1295              if pdict is None: 
1296                  pdict = self.pars 
1297              p = sortedDictValues(pdict) 
1298              i = _pollInputs(sortedDictValues(self.inputs), t, self.checklevel) 
1299              self._ensure_solver({'params': p, 't0': 0, 'tend': 1}) 
1300              self._ensure_inputs() 
1301              return self._solver.Jacobian(t, x, p+i)[0] 
1302          else: 
1303              raise PyDSTool_ExistError("Jacobian not defined")

1304  
 
1305  
 


1306 -    def JacobianP(self, t, xdict, pdict=None, asarray=True):


1307          """asarray is an unused, dummy argument for compatibility with
 
1308          Model.JacobianP""" 
1309          if self.haveJacobian_pars(): 
1310              x = sortedDictValues(filteredDict(xdict, self.funcspec.vars)) 
1311              if pdict is None: 
1312                  pdict = self.pars 
1313              p = sortedDictValues(pdict) 
1314              i = _pollInputs(sortedDictValues(self.inputs), t, self.checklevel) 
1315              self._ensure_solver({'params': p, 't0': 0, 'tend': 1}) 
1316              self._ensure_inputs() 
1317              return self._solver.JacobianP(t, x, p+i)[0] 
1318          else: 
1319              raise PyDSTool_ExistError("Jacobian w.r.t. parameters not defined")

1320  
 
1321  
 


1322 -    def AuxVars(self, t, xdict, pdict=None, asarray=True):


1323          """asarray is an unused, dummy argument for compatibility with
 
1324          Model.AuxVars""" 
1325          x = sortedDictValues(filteredDict(xdict, self.funcspec.vars)) 
1326          if pdict is None: 
1327              pdict = self.pars 
1328          p = sortedDictValues(pdict) 
1329          i = _pollInputs(sortedDictValues(self.inputs), t, self.checklevel) 
1330          self._ensure_solver({'params': p, 't0': 0, 'tend': 1}) 
1331          self._ensure_inputs() 
1332          return self._solver.AuxFunc(t, x, p+i)[0]

1333  
 
1334  
 


1335 -    def _ensure_solver(self, pars=None):


1336          if self._solver is None: 
1337              sortedDictValues(filteredDict(self.initialconditions, self.funcspec.vars)) 
1338  #            _integMod = self._ensureLoaded("dopri853"+self._vf_filename_ext)
 
1339              self._solver = dopri("dop853"+self._vf_filename_ext,
 
1340                                   rhs=self.name, phaseDim=self.dimension,
 
1341                                   paramDim=self.numpars,
 
1342                                   nAux=len(self.funcspec.auxvars),
 
1343                                   nEvents=len(self._eventNames),
 
1344                                   nExtInputs=len(self.inputs),
 
1345                                   hasJac=self.haveJacobian(),
 
1346                                   hasJacP=self.haveJacobian_pars(),
 
1347                                   hasMass=self.haveMass(),
 
1348                                   extraSpace=self.algparams['extraspace']) 
1349              try: 
1350                  genDB.register(self) 
1351              except PyDSTool_KeyError: 
1352                  errstr = "Generator " + self.name + ": this vector field's " +\
 
1353                           "DLL is already in use" 
1354                  raise RuntimeError(errstr) 
1355              if pars is not None: 
1356                  # tend value doesn't matter
 
1357                  self._solver.setRunParams(
 
1358                                ic=sortedDictValues(filteredDict(self.initialconditions,
 
1359                                                                 self.funcspec.vars)),
 
1360                                params=pars['params'],
 
1361                                t0=pars['t0'], tend=pars['tend'],
 
1362                                gt0=self.globalt0,
 
1363                                refine=0, specTimes=[])

1364  
 


1365 -    def _ensure_inputs(self, force=False):


1366          if not self.inputs: 
1367              return 
1368          if force: 
1369              listOK = False 
1370          else: 
1371              try: 
1372                  listOK = self._inputTimest0 == self.globalt0 
1373              except AttributeError: 
1374                  # not yet defined, so proceed
 
1375                  listOK = False 
1376          if not listOK: 
1377              self._inputVarList = [] 
1378              self._inputTimeList = [] 
1379              self._inputTimest0 = self.globalt0 
1380              # inputVarList is a list of Variables or Pointsets
 
1381              for inp in sortedDictValues(self.inputs): 
1382                  if isinstance(inp, Variable): 
1383                      pts = inp.getDataPoints() 
1384                      if pts is None: 
1385                          raise TypeError("Can only pass external input Variable objects if based on"
 
1386                                          " an underlying mesh") 
1387                      else: 
1388                          tvals = copy(pts[inp.indepvarname]) 
1389                          tvals -= self.globalt0 
1390                      self._inputVarList.append(pts[inp.coordname].tolist()) 
1391                      self._inputTimeList.append(tvals.tolist()) 
1392                  elif isinstance(inp, Pointset): 
1393                      tvals = copy(inp.indepvararray) 
1394                      tvals -= self.globalt0 
1395                      self._inputVarList.append(inp[inp.coordname].tolist()) 
1396                      self._inputTimeList.append(tvals.tolist()) 
1397                  else: 
1398                      raise TypeError("Invalid type of input") 
1399          if not self._solver.initExtInputs: 
1400              self._solver.setExtInputs(True, deepcopy(self._inputVarList),
 
1401                                          deepcopy(self._inputTimeList)) 
1402          elif not listOK: 
1403              self._solver.clearExtInputs() 
1404              self._solver.setExtInputs(True, deepcopy(self._inputVarList),
 
1405                                      deepcopy(self._inputTimeList)) 
1406              self._solver.canContinue=True

1407  
 
1408  
 


1409 -    def __del__(self):


1410          genDB.unregister(self) 
1411          ODEsystem.__del__(self)

1412  
 
1413  
 
1414  
 
1415  # Register this Generator with the database
 
1416  
 
1417  symbolMapDict = {'abs': 'fabs', 'sign': 'signum', 'mod': 'fmod'} 
1418  # in future, provide appropriate mappings for libraries math,
 
1419  # random, etc. (for now it's left to FuncSpec)
 
1420  theGenSpecHelper.add(Dopri_ODEsystem, symbolMapDict, 'c') 
1421
```

  


| Home | Trees | Indices | Help | | PyDSTool | | --- | |
| --- | --- | --- | --- | --- | --- |

|  |  |
| --- | --- |
| Generated by Epydoc 3.0.1 on Fri May 4 15:24:19 2012 | http://epydoc.sourceforge.net |
