# Supplementary material for: Hybrid Models and Biological Model Reduction with PyDSTool
Source: PLoS Comput Biol. 2012 Aug 9;8(8):e1002628. doi: 10.1371/journal.pcbi.1002628 (PMC3415397; doi:10.1371/journal.pcbi.1002628)
Supplement: Text S4 — Complete source code for the PyDSTool package (version 0.88.120504). Includes API documentation and help files linking to web pages. This file is identical to the current public release on Sourceforge.net. (ZIP) [file pcbi.1002628.s004.zip › PyDSTool/html/PyDSTool.Generator.Dopri_ODEsystem'.Dopri_ODEsystem-class.html]

xml version="1.0" encoding="ascii"?


PyDSTool.Generator.Dopri\_ODEsystem'.Dopri\_ODEsystem


| Home | Trees | Indices | Help | | PyDSTool | | --- | |
| --- | --- | --- | --- | --- | --- |

|  |  |  |  |
| --- | --- | --- | --- |
| Package PyDSTool :: Package Generator :: Module Dopri\_ODEsystem' :: Class Dopri\_ODEsystem | |  | | --- | | [hide private] | | [frames] | no frames] | |

# Class Dopri\_ODEsystem

source code

```
           object --+            
                    |            
baseclasses.Generator --+        
                        |        
       baseclasses.ctsGen --+    
                            |    
         ODEsystem'.ODEsystem --+
                                |
                               Dopri_ODEsystem
```

---

Wrapper for Dopri853 integrator.

Uses C target language only for functional specifications.


|  |  |  |  |
| --- | --- | --- | --- |
| |  |  | | --- | --- | | Instance Methods | [hide private] | | |
|  | |  |  | | --- | --- | | AuxVars(self, t, xdict, pdict=None, asarray=True)  asarray is an unused, dummy argument for compatibility with Model.AuxVars | source code | |
|  | |  |  | | --- | --- | | Jacobian(self, t, xdict, pdict=None, asarray=True)  asarray is an unused, dummy argument for compatibility with Model.Jacobian | source code | |
|  | |  |  | | --- | --- | | JacobianP(self, t, xdict, pdict=None, asarray=True)  asarray is an unused, dummy argument for compatibility with Model.JacobianP | source code | |
|  | |  |  | | --- | --- | | Rhs(self, t, xdict, pdict=None, asarray=True)  asarray is an unused, dummy argument for compatibility with Model.Rhs | source code | |
|  | |  |  | | --- | --- | | \_\_del\_\_(self) | source code | |
|  | |  |  | | --- | --- | | \_\_init\_\_(self, kw)  Use the nobuild key to postpone building of the library, e.g. | source code | |
|  | |  |  | | --- | --- | | \_ensure\_inputs(self, force=False) | source code | |
|  | |  |  | | --- | --- | | \_ensure\_solver(self, pars=None) | source code | |
|  | |  |  | | --- | --- | | \_prepareEventSpecs(self) | source code | |
|  | |  |  | | --- | --- | | compileLib(self, libsources=`[``]`, libdirs=`[``]`)  compileLib generates a python extension DLL with integrator and vector field compiled and linked. | source code | |
|  | |  |  | | --- | --- | | compute(self, trajname, dirn=`'``f``'`, ics=None)  This is an abstract class. | source code | |
|  | |  |  | | --- | --- | | forceLibRefresh(self)  forceLibRefresh should be called after event contents are changed, or alterations are made to the right-hand side of the ODEs. | source code | |
|  | |  |  | | --- | --- | | makeLib(self, libsources=`[``]`, libdirs=`[``]`, include=`[``]`)  makeLib calls makeLibSource and then the compileLib method. | source code | |
|  | |  |  | | --- | --- | | makeLibSource(self, include=`[``]`)  makeLibSource generates the C source for the vector field specification. | source code | |
| **Inherited from `ODEsystem'.ODEsystem`**: `__getstate__`, `__setstate__`, `addMethods`, `checkInitialConditions`, `cleanupMemory`, `haveJacobian`, `haveJacobian_pars`, `haveMass`, `prepDirection`, `set`, `validateICs`  **Inherited from `baseclasses.ctsGen`**: `validateSpec`  **Inherited from `baseclasses.Generator`**: `__copy__`, `__deepcopy__`, `__repr__`, `__str__`, `addEvtPars`, `checkArgs`, `contains`, `get`, `getEventTimes`, `getEvents`, `info`, `query`, `resetEventTimes`, `resetEvents`, `setEventICs`, `showAuxFnSpec`, `showAuxSpec`, `showEventSpec`, `showSpec`  **Inherited from `baseclasses.Generator`** (private): `_addEvents`, `_auxfn_getindex`, `_auxfn_globalindepvar`, `_auxfn_heav`, `_auxfn_if`, `_auxfn_initcond`, `_generate_ixmaps`, `_infostr`, `_kw_process_algparams`, `_kw_process_allvars`, `_kw_process_dispatch`, `_kw_process_events`, `_kw_process_fnspecs`, `_kw_process_ics`, `_kw_process_ignorespecial`, `_kw_process_inputs`, `_kw_process_pars`, `_kw_process_pdomain`, `_kw_process_reuseterms`, `_kw_process_system`, `_kw_process_target`, `_kw_process_tdata`, `_kw_process_tdomain`, `_kw_process_tstep`, `_kw_process_ttype`, `_kw_process_varspecs`, `_kw_process_vfcodeinserts`, `_kw_process_xdomain`, `_kw_process_xtype`, `_makeBoundsEvents`, `_register`, `_set_for_hybrid_DS`  **Inherited from `object`**: `__delattr__`, `__getattribute__`, `__hash__`, `__new__`, `__reduce__`, `__reduce_ex__`, `__setattr__` | |


|  |  |  |  |
| --- | --- | --- | --- |
| |  |  | | --- | --- | | Class Variables | [hide private] | | |
|  | \_paraminfo = `{'atol': 'Absolute error tolerance.', 'beta': 'Th...` |
| **Inherited from `ODEsystem'.ODEsystem`** (private): `_needKeys`, `_optionalKeys`, `_validKeys`  **Inherited from `baseclasses.Generator`** (private): `_querykeys` | |


|  |  |  |  |
| --- | --- | --- | --- |
| |  |  | | --- | --- | | Properties | [hide private] | | |
| **Inherited from `object`**: `__class__` | |


|  |  |  |  |
| --- | --- | --- | --- |
| |  |  | | --- | --- | | Method Details | [hide private] | | |

|  |  |  |
| --- | --- | --- |
| |  |  | | --- | --- | | AuxVars(self, t, xdict, pdict=None, asarray=True) | source code |   asarray is an unused, dummy argument for compatibility with Model.AuxVars  Overrides: ODEsystem'.ODEsystem.AuxVars |

|  |  |  |
| --- | --- | --- |
| |  |  | | --- | --- | | Jacobian(self, t, xdict, pdict=None, asarray=True) | source code |   asarray is an unused, dummy argument for compatibility with Model.Jacobian  Overrides: ODEsystem'.ODEsystem.Jacobian |

|  |  |  |
| --- | --- | --- |
| |  |  | | --- | --- | | JacobianP(self, t, xdict, pdict=None, asarray=True) | source code |   asarray is an unused, dummy argument for compatibility with Model.JacobianP  Overrides: ODEsystem'.ODEsystem.JacobianP |

|  |  |  |
| --- | --- | --- |
| |  |  | | --- | --- | | Rhs(self, t, xdict, pdict=None, asarray=True) | source code |   asarray is an unused, dummy argument for compatibility with Model.Rhs  Overrides: ODEsystem'.ODEsystem.Rhs |

|  |  |  |
| --- | --- | --- |
| |  |  | | --- | --- | | \_\_del\_\_(self)  *(Destructor)* | source code |   Overrides: baseclasses.Generator.\_\_del\_\_ |

|  |  |  |
| --- | --- | --- |
| |  |  | | --- | --- | | \_\_init\_\_(self, kw)  *(Constructor)* | source code |   Use the nobuild key to postpone building of the library, e.g. in order to provide additional build options to makeLibSource and compileLib methods or to make changes to the C code by hand. No build options can be specified otherwise.  Overrides: object.\_\_init\_\_ |

|  |  |  |
| --- | --- | --- |
| |  |  | | --- | --- | | compileLib(self, libsources=`[``]`, libdirs=`[``]`) | source code |  ``` compileLib generates a python extension DLL with integrator and vector field compiled and linked.  libsources list allows additional library sources to be linked. libdirs list allows additional directories to be searched for   precompiled libraries. ``` |

|  |  |  |
| --- | --- | --- |
| |  |  | | --- | --- | | compute(self, trajname, dirn=`'``f``'`, ics=None) | source code |   This is an abstract class.  Overrides: ODEsystem'.ODEsystem.compute *(inherited documentation)* |

|  |  |  |
| --- | --- | --- |
| |  |  | | --- | --- | | forceLibRefresh(self) | source code |   forceLibRefresh should be called after event contents are changed, or alterations are made to the right-hand side of the ODEs.  Currently this function does NOT work! |

|  |  |  |
| --- | --- | --- |
| |  |  | | --- | --- | | makeLib(self, libsources=`[``]`, libdirs=`[``]`, include=`[``]`) | source code |   makeLib calls makeLibSource and then the compileLib method. To postpone compilation of the source to a DLL, call makelibsource() separately. |

|  |  |  |
| --- | --- | --- |
| |  |  | | --- | --- | | makeLibSource(self, include=`[``]`) | source code |   makeLibSource generates the C source for the vector field specification. It should be called only once per vector field. |

  


|  |  |  |  |
| --- | --- | --- | --- |
| |  |  | | --- | --- | | Class Variable Details | [hide private] | | |

|  |  |
| --- | --- |
| \_paraminfo   Value:  |  | | --- | | ``` {'atol': 'Absolute error tolerance.',  'beta': 'The "beta" for stabilized step size control. Larger values f or beta ( <= 0.1 ) make the step size control more stable. Negative in itial value provoke beta=0; default beta=0.04',  'boundsCheckMaxSteps': 'Last step to bounds check if checkBound==1. D efaults to 1000.',  'checkBounds': 'Switch to check variable bounds: 0 = no check, 1 = ch eck up to \'boundsCheckMaxSteps\', 2 = check for every point', ... ``` | |

  


| Home | Trees | Indices | Help | | PyDSTool | | --- | |
| --- | --- | --- | --- | --- | --- |

|  |  |
| --- | --- |
| Generated by Epydoc 3.0.1 on Fri May 4 15:24:06 2012 | http://epydoc.sourceforge.net |
