# Supplementary material for: Hybrid Models and Biological Model Reduction with PyDSTool
Source: PLoS Comput Biol. 2012 Aug 9;8(8):e1002628. doi: 10.1371/journal.pcbi.1002628 (PMC3415397; doi:10.1371/journal.pcbi.1002628)
Supplement: Text S4 — Complete source code for the PyDSTool package (version 0.88.120504). Includes API documentation and help files linking to web pages. This file is identical to the current public release on Sourceforge.net. (ZIP) [file pcbi.1002628.s004.zip › PyDSTool/html/PyDSTool.Generator.EmbeddedSysGen'-pysrc.html]

xml version="1.0" encoding="ascii"?


PyDSTool.Generator.EmbeddedSysGen'


| Home | Trees | Indices | Help | | PyDSTool | | --- | |
| --- | --- | --- | --- | --- | --- |

|  |  |  |  |
| --- | --- | --- | --- |
| Package PyDSTool :: Package Generator :: Module EmbeddedSysGen' | |  | | --- | | [hide private] | | [frames] | no frames] | |

# Source Code for Module PyDSTool.Generator.EmbeddedSysGen'

```
  1  # Embedded dynamical system generator 
  2  from __future__ import division 
  3   
  4  from allimports import * 
  5  from baseclasses import ctsGen, theGenSpecHelper 
  6  from PyDSTool.utils import * 
  7  from PyDSTool.common import * 
  8  from PyDSTool.Interval import uncertain 
  9   
 10  # Other imports 
 11  from numpy import Inf, NaN, isfinite, sometrue, alltrue, array, arange, \ 
 12       transpose, shape 
 13  import math, random 
 14  from copy import copy, deepcopy 
 15  try: 
 16      # use pscyo JIT byte-compiler optimization, if available 
 17      import psyco 
 18      HAVE_PSYCO = True 
 19  except ImportError: 
 20      HAVE_PSYCO = False 
 21   
 22   


23 -class EmbeddedSysGen(ctsGen):


24      """Embedded dynamical system form specifying a trajectory. 
 25   
 26      The embedded system is assumed to be of type Model. 
 27      """ 
 28      # inherit most of these from the embedded system 
 29      _validKeys = ['globalt0', 'xdomain', 'tdata', 'tdomain', 
 30                       'ics', 'pars', 'checklevel', 'pdomain', 'abseps'] 
 31      _needKeys = ctsGen._needKeys + ['specfn', 'system'] 
 32      _optionalKeys = ctsGen._optionalKeys + ['tdomain', 'pars', 'pdomain', 'xdomain', 
 33                                    'ics', 'vars', 'tdata', 'enforcebounds', 
 34                                    'activatedbounds'] 
 35   


36 -    def __init__(self, kw):


37          ctsGen.__init__(self, kw) 
 38          dispatch_list = ['tdomain', 'tdata', 'xtype', 'xdomain', 
 39                           'ics', 'pars', 'pdomain', 'system'] 
 40          if 'varspecs' in kw: 
 41              raise PyDSTool_KeyError('varspecs option invalid for EmbeddedSysGen ' 
 42                                      'class') 
 43          if 'inputs' in kw: 
 44              raise PyDSTool_KeyError('inputs option invalid for EmbeddedSysGen ' 
 45                                      'class') 
 46          try: 
 47              kw['varspecs'] = kw['system'].query('vardomains') 
 48          except (KeyError, AttributeError): 
 49              raise PyDSTool_KeyError("Model-type system must be provided") 
 50          self.funcspec = args(**self._kw_process_dispatch(dispatch_list, kw)) 
 51          self.funcspec.vars = kw['varspecs'].keys() 
 52          self.funcspec.auxvars = [] 
 53          # varspecs not specified by user and must be removed for checkArgs() 
 54          del kw['varspecs'] 
 55          self.indepvartype = float 
 56          try: 
 57              self._embed_spec = kw['specfn'] 
 58          except: 
 59              raise "Must provide a function for the specification of this system" 
 60          else: 
 61              self.foundKeys += 1 
 62          self.eventstruct = EventStruct() 
 63          self.checkArgs(kw) 
 64          assert self.eventstruct.getLowLevelEvents() == [], \ 
 65                 "Events are not supported for EmbeddedSysGen class" 
 66          assert self.eventstruct.getHighLevelEvents() == [], \ 
 67                 "Events are not supported for EmbeddedSysGen class" 
 68          self.indepvariable = Variable(listid, Interval('t_domain', 
 69                                                         self.indepvartype, 
 70                                                self.tdomain, self._abseps), 
 71                               Interval('t', self.indepvartype, self.tdata, 
 72                                        self._abseps), 't') 
 73          self._register(self.indepvariable) 
 74          for x in self.xdomain.keys(): 
 75              # aux vars? 
 76              try: 
 77                  xinterval=Interval(x, self.xtype[x], self.xdomain[x], self._abseps) 
 78              except KeyError, e: 
 79                  raise PyDSTool_KeyError('Mismatch between declared variables ' 
 80                                   'and xspecs: ' + str(e)) 
 81              # placeholder variable so that this class can be 
 82              # copied before it is defined (listid function is a dummy) 
 83              self.variables[x] = Variable(None, self.indepvariable.depdomain, 
 84                                           xinterval, x)

 85          # xdomain and pdomain ignored! 
 86   
 87   


88 -    def compute(self, trajname, ics=None):


89          """ 
 90          """ 
 91          if ics is not None: 
 92              self.set(ics=ics) 
 93          self._solver.set(pars=self.pars, 
 94                           globalt0=self.globalt0, 
 95                           ics=self.initialconditions, 
 96                           checklevel=self.checklevel, 
 97                           abseps=self._abseps) 
 98          self.diagnostics.clearWarnings() 
 99          self.diagnostics.clearErrors() 
100          if not self.defined: 
101              self._register(self.variables) 
102  #        self.validateSpec() 
103          try: 
104              traj = self._embed_spec(self._solver) 
105          except: 
106              print "Error in user-provided embedded system" 
107              raise 
108          self.defined = True 
109          traj.name = trajname 
110          return traj

111   
112   


113 -    def haveJacobian_pars(self):


114          """Report whether generator has an explicit user-specified Jacobian 
115          with respect to pars associated with it.""" 
116          return self._solver.haveJacobian_pars()

117   


118 -    def haveJacobian(self):


119          """Report whether generator has an explicit user-specified Jacobian 
120          associated with it.""" 
121          return self._solver.haveJacobian()

122   
123   


124 -    def set(self, **kw):


125          """Set ExplicitFnGen parameters""" 
126          if remain(kw.keys(), self._validKeys) != []: 
127              raise KeyError("Invalid keys in argument") 
128          if 'globalt0' in kw: 
129              # pass up to generic treatment for this 
130              ctsGen.set(self, globalt0=kw['globalt0']) 
131          if 'checklevel' in kw: 
132              # pass up to generic treatment for this 
133              ctsGen.set(self, checklevel=kw['checklevel']) 
134          if 'abseps' in kw: 
135              # pass up to generic treatment for this 
136              ctsGen.set(self, abseps=kw['abseps']) 
137          # optional keys for this call are 
138          #   ['pars', 'tdomain', 'xdomain', 'pdomain'] 
139          if 'xdomain' in kw: 
140              for k_temp, v in kw['xdomain'].iteritems(): 
141                  k = self._FScompatibleNames(k_temp) 
142                  if k in self.xdomain.keys(): 
143                      if isinstance(v, _seq_types): 
144                          assert len(v) == 2, \ 
145                                 "Invalid size of domain specification for "+k 
146                          if v[0] >= v[1]: 
147                              raise PyDSTool_ValueError('xdomain values must be' 
148                                                        'in order of increasing ' 
149                                                        'size') 
150                      elif isinstance(v, _num_types): 
151                          pass 
152                      else: 
153                          raise PyDSTool_TypeError('Invalid type for xdomain spec' 
154                                                   ' '+k) 
155                      self.xdomain[k] = v 
156                  else: 
157                      raise ValueError('Illegal variable name') 
158                  try: 
159                      self.variables[k].depdomain.set(v) 
160                  except TypeError: 
161                      raise TypeError('xdomain must be a dictionary of variable' 
162                                        ' names -> valid interval 2-tuples or ' 
163                                        'singletons') 
164          if 'pdomain' in kw: 
165              for k_temp, v in kw['pdomain'].iteritems(): 
166                  k = self._FScompatibleNames(k_temp) 
167                  if k in self.pars.keys(): 
168                      if isinstance(v, _seq_types): 
169                          assert len(v) == 2, \ 
170                                 "Invalid size of domain specification for "+k 
171                          if v[0] >= v[1]: 
172                              raise PyDSTool_ValueError('pdomain values must be' 
173                                                        'in order of increasing ' 
174                                                        'size') 
175                          else: 
176                              self.pdomain[k] = copy(v) 
177                      elif isinstance(v, _num_types): 
178                          self.pdomain[k] = [v, v] 
179                      else: 
180                          raise PyDSTool_TypeError('Invalid type for pdomain spec' 
181                                                   ' '+k) 
182                  else: 
183                      raise ValueError('Illegal parameter name') 
184                  try: 
185                      self.parameterDomains[k].depdomain.set(v) 
186                  except TypeError: 
187                      raise TypeError('pdomain must be a dictionary of parameter' 
188                                        ' names -> valid interval 2-tuples or ' 
189                                        'singletons') 
190          if 'tdata' in kw: 
191              self.tdata = kw['tdata'] 
192          if 'tdomain' in kw: 
193              self.tdomain = kw['tdomain'] 
194              self.indepvariable.indepdomain.set(self.tdomain) 
195          if self.tdomain[0] > self.tdata[0]: 
196              if self.indepvariable.indepdomain.contains(self.tdata[0]) == uncertain: 
197                  self.diagnostics.warnings.append((W_UNCERTVAL, 
198                                                    (self.tdata[0],self.tdomain))) 
199              else: 
200                  print 'tdata cannot be specified below smallest '\ 
201                        'value in tdomain\n (possibly due to uncertain bounding).'\ 
202                        ' It has been automatically adjusted from\n ', self.tdata[0], \ 
203                        'to', self.tdomain[0], '(difference of', \ 
204                        self.tdomain[0]-self.tdata[0], ')' 
205              self.tdata[0] = self.tdomain[0] 
206          if self.tdomain[1] < self.tdata[1]: 
207              if self.indepvariable.indepdomain.contains(self.tdata[1]) == uncertain: 
208                  self.diagnostics.warnings.append((W_UNCERTVAL, 
209                                                    (self.tdata[1],self.tdomain))) 
210              else: 
211                  print 'tdata cannot be specified above largest '\ 
212                        'value in tdomain\n (possibly due to uncertain bounding).'\ 
213                        ' It has been automatically adjusted from\n ', \ 
214                        self.tdomain[1], 'to', \ 
215                        self.tdomain[1], '(difference of', \ 
216                        self.tdata[1]-self.tdomain[1], ')' 
217              self.tdata[1] = self.tdomain[1] 
218          self.indepvariable.depdomain.set(self.tdata) 
219          if 'ics' in kw: 
220              for k_temp, v in kw['ics'].iteritems(): 
221                  k = self._FScompatibleNames(k_temp) 
222                  if k in self.xdomain.keys(): 
223                      self._xdatadict[k] = ensurefloat(v) 
224                  else: 
225                      raise ValueError('Illegal variable name') 
226              self.initialconditions.update(self._xdatadict) 
227          if 'pars' in kw: 
228              if not self.pars: 
229                  raise ValueError('No pars were declared for this object' 
230                                     ' at initialization.') 
231              for k_temp, v in kw['pars'].iteritems(): 
232                  k = self._FScompatibleNames(k_temp) 
233                  if k in self.pars: 
234                      cval = self.parameterDomains[k].contains(v) 
235                      if self.checklevel < 3: 
236                          if cval is not notcontained: 
237                              self.pars[k] = ensurefloat(v) 
238                              if cval is uncertain and self.checklevel == 2: 
239                                  print 'Warning: Parameter value at bound' 
240                          else: 
241                              raise PyDSTool_ValueError('Parameter value out of ' 
242                                                        'bounds') 
243                      else: 
244                          if cval is contained: 
245                              self.pars[k] = ensurefloat(v) 
246                          elif cval is uncertain: 
247                              raise PyDSTool_UncertainValueError('Parameter value' 
248                                                                 ' at bound') 
249                          else: 
250                              raise PyDSTool_ValueError('Parameter value out of' 
251                                                        ' bounds') 
252                  else: 
253                      raise PyDSTool_AttributeError('Illegal parameter name')

254   
255   


256 -    def validateSpec(self):


257          ctsGen.validateSpec(self) 
258          try: 
259              for v in self.variables.values(): 
260                  assert isinstance(v, Variable) 
261              assert not self.inputs 
262          except AssertionError: 
263              print 'Invalid system specification' 
264              raise

265   
266   


267 -    def __del__(self):


268          ctsGen.__del__(self)

269   
270   
271   
272  # Register this Generator with the database 
273   
274  symbolMapDict = {} 
275  # in future, provide appropriate mappings for libraries math, 
276  # random, etc. (for now it's left to FuncSpec) 
277  theGenSpecHelper.add(EmbeddedSysGen, symbolMapDict, 'python', None) 
278
```

  


| Home | Trees | Indices | Help | | PyDSTool | | --- | |
| --- | --- | --- | --- | --- | --- |

|  |  |
| --- | --- |
| Generated by Epydoc 3.0.1 on Fri May 4 15:24:24 2012 | http://epydoc.sourceforge.net |
