# Supplementary material for: Hybrid Models and Biological Model Reduction with PyDSTool
Source: PLoS Comput Biol. 2012 Aug 9;8(8):e1002628. doi: 10.1371/journal.pcbi.1002628 (PMC3415397; doi:10.1371/journal.pcbi.1002628)
Supplement: Text S4 — Complete source code for the PyDSTool package (version 0.88.120504). Includes API documentation and help files linking to web pages. This file is identical to the current public release on Sourceforge.net. (ZIP) [file pcbi.1002628.s004.zip › PyDSTool/html/PyDSTool.Generator.Euler_ODEsystem'-module.html]

xml version="1.0" encoding="ascii"?


PyDSTool.Generator.Euler\_ODEsystem'


| Home | Trees | Indices | Help | | PyDSTool | | --- | |
| --- | --- | --- | --- | --- | --- |

|  |  |  |  |
| --- | --- | --- | --- |
| Package PyDSTool :: Package Generator :: Module Euler\_ODEsystem' | |  | | --- | | [hide private] | | [frames] | no frames] | |

# Module Euler\_ODEsystem'

source code

Euler integrator for ODE systems, with no step refinement for
events.


|  |  |  |  |
| --- | --- | --- | --- |
| |  |  | | --- | --- | | Classes | [hide private] | | |
|  | Euler\_ODEsystem  Euler method. |
|  | euler\_solver |


|  |  |  |  |
| --- | --- | --- | --- |
| |  |  | | --- | --- | | Functions | [hide private] | | |
|  | |  |  | | --- | --- | | \_dummy\_userfunc(euler) | source code | |


|  |  |  |  |
| --- | --- | --- | --- |
| |  |  | | --- | --- | | Variables | [hide private] | | |
|  | API = `API_class()` |
|  | Continuous = `Continuous Domain` |
|  | Discrete = `Discrete Domain` |
|  | E\_COMPUTFAIL = `0` |
|  | E\_NONUNIQUETERM = `10` |
|  | HAVE\_PSYCO = `True` |
|  | Inf = `inf` |
|  | LargestInt32 = `2147483647` |
|  | NaN = `nan` |
|  | W\_BISECTLIMIT = `12` |
|  | W\_NONTERMEVENT = `20` |
|  | W\_NONTERMSTATEBD = `21` |
|  | W\_TERMEVENT = `10` |
|  | W\_TERMSTATEBD = `11` |
|  | W\_UNCERTVAL = `0` |
|  | \_1DimplicitSolveMethods = `['newton', 'bisect', 'steffe']` |
|  | \_all\_complex = `(<type 'complex'>, <type 'numpy.complexfloating...` |
|  | \_all\_float = `(<type 'float'>, <type 'numpy.floating'>, <type '...` |
|  | \_all\_int = `(<type 'int'>, <type 'numpy.integer'>, <type 'numpy...` |
|  | \_all\_numpy\_complex = `(<type 'numpy.complex128'>, <type 'numpy....` |
|  | \_all\_numpy\_float = `(<type 'numpy.float64'>, <type 'numpy.float...` |
|  | \_all\_numpy\_int = `(<type 'numpy.int32'>, <type 'numpy.int32'>, ...` |
|  | \_complex\_types = `(<type 'complex'>, <type 'numpy.complexfloati...` |
|  | \_float\_types = `(<type 'float'>, <type 'numpy.floating'>)` |
|  | \_implicitSolveMethods = `['newton', 'bisect', 'steffe', 'fsolve']` |
|  | \_int\_types = `(<type 'int'>, <type 'numpy.integer'>)` |
|  | \_num\_equivtype = `{<type 'float'>: <type 'numpy.float64'>, <typ...` |
|  | \_num\_maxmin = `{<type 'numpy.int32'>: [-2147483648, 2147483647]...` |
|  | \_num\_name2equivtypes = `{'float': (<type 'float'>, <type 'numpy...` |
|  | \_num\_name2type = `{'float': <type 'numpy.float64'>, 'int': <typ...` |
|  | \_num\_type2name = `{<type 'float'>: 'float', <type 'int'>: 'int'...` |
|  | \_num\_types = `(<type 'float'>, <type 'int'>, <type 'numpy.float...` |
|  | \_pytypefromtype = `{<type 'numpy.int32'>: <type 'int'>, <type '...` |
|  | \_real\_types = `(<type 'int'>, <type 'numpy.integer'>, <type 'fl...` |
|  | \_seq\_types = `(<type 'list'>, <type 'tuple'>, <type 'numpy.ndar...` |
|  | contained = `contained` |
|  | e = `2.71828182846` |
|  | errmessages = `{0: 'Computation of trajectory failed', 10: 'Mor...` |
|  | errorfields = `{0: ['t', 'error info'], 10: ['t', 'event list']}` |
|  | isfinite = `<ufunc 'isfinite'>` |
|  | less\_equal = `<ufunc 'less_equal'>` |
|  | notcontained = `notcontained` |
|  | null\_predicate = `null_predicate_class(None)` |
|  | pi = `3.14159265359` |
|  | sign = `<ufunc 'sign'>` |
|  | symbolMapDict = `{}` |
|  | targetLangs = `['c', 'python', 'matlab']` |
|  | theGenSpecHelper = `GenSpecHelper()` |
|  | uncertain = `uncertain` |
|  | warnfields = `{0: ['value', 'interval'], 10: ['t', 'event list'...` |
|  | warnmessages = `{0: 'Uncertain value computed', 10: 'Terminal e...` |


|  |  |  |  |
| --- | --- | --- | --- |
| |  |  | | --- | --- | | Variables Details | [hide private] | | |

|  |  |
| --- | --- |
| \_all\_complex   Value:  |  | | --- | | ``` (<type 'complex'>,  <type 'numpy.complexfloating'>,  <type 'numpy.complex128'>,  <type 'numpy.complex64'>,  <type 'numpy.complex128'>) ``` | |

|  |  |
| --- | --- |
| \_all\_float   Value:  |  | | --- | | ``` (<type 'float'>,  <type 'numpy.floating'>,  <type 'numpy.float64'>,  <type 'numpy.float32'>,  <type 'numpy.float64'>) ``` | |

|  |  |
| --- | --- |
| \_all\_int   Value:  |  | | --- | | ``` (<type 'int'>,  <type 'numpy.integer'>,  <type 'numpy.int32'>,  <type 'numpy.int32'>,  <type 'numpy.int8'>,  <type 'numpy.int16'>,  <type 'numpy.int32'>,  <type 'numpy.int64'>) ``` | |

|  |  |
| --- | --- |
| \_all\_numpy\_complex   Value:  |  | | --- | | ``` (<type 'numpy.complex128'>,  <type 'numpy.complex64'>,  <type 'numpy.complex128'>) ``` | |

|  |  |
| --- | --- |
| \_all\_numpy\_float   Value:  |  | | --- | | ``` (<type 'numpy.float64'>,  <type 'numpy.float32'>,  <type 'numpy.float64'>) ``` | |

|  |  |
| --- | --- |
| \_all\_numpy\_int   Value:  |  | | --- | | ``` (<type 'numpy.int32'>,  <type 'numpy.int32'>,  <type 'numpy.int8'>,  <type 'numpy.int16'>,  <type 'numpy.int32'>,  <type 'numpy.int64'>) ``` | |

|  |  |
| --- | --- |
| \_complex\_types   Value:  |  | | --- | | ``` (<type 'complex'>, <type 'numpy.complexfloating'>) ``` | |

|  |  |
| --- | --- |
| \_num\_equivtype   Value:  |  | | --- | | ``` {<type 'float'>: <type 'numpy.float64'>,  <type 'int'>: <type 'numpy.int32'>,  <type 'numpy.integer'>: <type 'numpy.int32'>,  <type 'numpy.floating'>: <type 'numpy.float64'>,  <type 'numpy.int8'>: <type 'numpy.int32'>,  <type 'numpy.int16'>: <type 'numpy.int32'>,  <type 'numpy.int32'>: <type 'numpy.int32'>,  <type 'numpy.int32'>: <type 'numpy.int32'>, ... ``` | |

|  |  |
| --- | --- |
| \_num\_maxmin   Value:  |  | | --- | | ``` {<type 'numpy.int32'>: [-2147483648, 2147483647],  <type 'numpy.float64'>: [-inf, inf]} ``` | |

|  |  |
| --- | --- |
| \_num\_name2equivtypes   Value:  |  | | --- | | ``` {'float': (<type 'float'>,            <type 'numpy.floating'>,            <type 'numpy.float64'>,            <type 'numpy.float32'>,            <type 'numpy.float64'>),  'int': (<type 'int'>,          <type 'numpy.integer'>,          <type 'numpy.int32'>, ... ``` | |

|  |  |
| --- | --- |
| \_num\_name2type   Value:  |  | | --- | | ``` {'float': <type 'numpy.float64'>, 'int': <type 'numpy.int32'>} ``` | |

|  |  |
| --- | --- |
| \_num\_type2name   Value:  |  | | --- | | ``` {<type 'float'>: 'float',  <type 'int'>: 'int',  <type 'numpy.integer'>: 'int',  <type 'numpy.floating'>: 'float',  <type 'numpy.int8'>: 'int',  <type 'numpy.int16'>: 'int',  <type 'numpy.int32'>: 'int',  <type 'numpy.int32'>: 'int', ... ``` | |

|  |  |
| --- | --- |
| \_num\_types   Value:  |  | | --- | | ``` (<type 'float'>,  <type 'int'>,  <type 'numpy.floating'>,  <type 'numpy.integer'>) ``` | |

|  |  |
| --- | --- |
| \_pytypefromtype   Value:  |  | | --- | | ``` {<type 'numpy.int32'>: <type 'int'>,  <type 'numpy.float64'>: <type 'float'>} ``` | |

|  |  |
| --- | --- |
| \_real\_types   Value:  |  | | --- | | ``` (<type 'int'>,  <type 'numpy.integer'>,  <type 'float'>,  <type 'numpy.floating'>) ``` | |

|  |  |
| --- | --- |
| \_seq\_types   Value:  |  | | --- | | ``` (<type 'list'>, <type 'tuple'>, <type 'numpy.ndarray'>) ``` | |

|  |  |
| --- | --- |
| errmessages   Value:  |  | | --- | | ``` {0: 'Computation of trajectory failed',  10: 'More than one terminal event found'} ``` | |

|  |  |
| --- | --- |
| warnfields   Value:  |  | | --- | | ``` {0: ['value', 'interval'],  10: ['t', 'event list'],  11: ['t', 'var name', 'var value', '\n\tvalue interval'],  12: ['t', 'event list'],  20: ['t', 'event list'],  21: ['t', 'var name', 'var value', '\n\tvalue interval']} ``` | |

|  |  |
| --- | --- |
| warnmessages   Value:  |  | | --- | | ``` {0: 'Uncertain value computed',  10: 'Terminal event(s) found',  11: 'State variable reached bounds (terminal)',  12: 'Bisection limit reached for event',  20: 'Non-terminal event(s) found',  21: 'State or input variable reached bounds (non-terminal)'} ``` | |

  


| Home | Trees | Indices | Help | | PyDSTool | | --- | |
| --- | --- | --- | --- | --- | --- |

|  |  |
| --- | --- |
| Generated by Epydoc 3.0.1 on Fri May 4 15:24:02 2012 | http://epydoc.sourceforge.net |
