# Supplementary material for: Hybrid Models and Biological Model Reduction with PyDSTool
Source: PLoS Comput Biol. 2012 Aug 9;8(8):e1002628. doi: 10.1371/journal.pcbi.1002628 (PMC3415397; doi:10.1371/journal.pcbi.1002628)
Supplement: Text S4 — Complete source code for the PyDSTool package (version 0.88.120504). Includes API documentation and help files linking to web pages. This file is identical to the current public release on Sourceforge.net. (ZIP) [file pcbi.1002628.s004.zip › PyDSTool/html/PyDSTool.Generator.Euler_ODEsystem'-pysrc.html]

xml version="1.0" encoding="ascii"?


PyDSTool.Generator.Euler\_ODEsystem'


| Home | Trees | Indices | Help | | PyDSTool | | --- | |
| --- | --- | --- | --- | --- | --- |

|  |  |  |  |
| --- | --- | --- | --- |
| Package PyDSTool :: Package Generator :: Module Euler\_ODEsystem' | |  | | --- | | [hide private] | | [frames] | no frames] | |

# Source Code for Module PyDSTool.Generator.Euler\_ODEsystem'

```
  1  """Euler integrator for ODE systems, with no step refinement for events.
 
  2  """ 
  3  from __future__ import division 
  4  
 
  5  from allimports import * 
  6  from PyDSTool.Generator import ODEsystem as ODEsystem 
  7  from baseclasses import Generator, theGenSpecHelper, _pollInputs 
  8  from PyDSTool.utils import * 
  9  from PyDSTool.common import * 
 10  
 
 11  # Other imports
 
 12  from numpy import Inf, NaN, isfinite, sometrue, alltrue, sign, all, any, \
 
 13       array, zeros, less_equal, transpose, concatenate, asarray, linspace 
 14  try: 
 15      from numpy import unique 
 16  except ImportError: 
 17      # older version of numpy
 
 18      from numpy import unique1d as unique 
 19  import math, random 
 20  import numpy as np 
 21  from copy import copy, deepcopy 
 22  import os, platform, shutil, sys, gc 
 23  
 
 24  try: 
 25      # use psyco JIT byte-compiler optimization, if available
 
 26      import psyco 
 27      HAVE_PSYCO = True 
 28  except ImportError: 
 29      HAVE_PSYCO = False 
 30  
 
 31  
 


32 -class euler_solver(object):


33 -    def __init__(self, rhs):


34          self.f = rhs 
 35          self.y = None 
 36          self.t = None

 37  
 


38 -    def set_initial_value(self, y0, t0):


39          self.y = y0 
 40          self.t = t0

 41  
 


42 -    def set_f_params(self, extraparams=None):


43          if extraparams is None: 
 44              self.f_params = [] 
 45          else: 
 46              self.f_params = extraparams

 47  
 


48 -    def set_jac_params(self, extraparams=None):


49          """Jacobian is not used""" 
 50          pass

 51  
 


52 -    def integrate(self, dt):


53          """Single step""" 
 54          self.t += dt 
 55          self.y = self.y + dt*self.f(self.t, self.y, self.f_params) 
 56          return 1

 57  
 


58 -    def successful(self):


59          return True

 60  
 


61 -def _dummy_userfunc(euler):


62      pass

 63  
 


64 -class Euler_ODEsystem(ODEsystem):


65      """Euler method. Fixed step.
 
 66  
 
 67      Uses Python target language only for functional specifications.""" 
 68  
 


69 -    def __init__(self, kw):


70          if 'user_func_beforestep' in kw: 
 71              self.ufunc_before = kw['user_func_beforestep'] 
 72              # delete because not covered in ODEsystem
 
 73              del kw['user_func_beforestep'] 
 74          else: 
 75              self.ufunc_before = _dummy_userfunc 
 76          if 'user_func_afterstep' in kw: 
 77              self.ufunc_after = kw['user_func_afterstep'] 
 78              # delete because not covered in ODEsystem
 
 79              del kw['user_func_afterstep'] 
 80          else: 
 81              self.ufunc_after = _dummy_userfunc 
 82          ODEsystem.__init__(self, kw) 
 83          self._paraminfo = {'init_step': 'Fixed step size for time mesh.'} 
 84          self.diagnostics._errorcodes = {1: 'Step OK'} 
 85          self.diagnostics.outputStatsInfo = {'errorStatus': 'Error status on completion.'} 
 86          algparams_def = {'poly_interp': False,
 
 87                           'init_step': 0.01,
 
 88                           'max_pts': 100000
 
 89                           } 
 90          for k, v in algparams_def.iteritems(): 
 91              if k not in self.algparams: 
 92                  self.algparams[k] = v

 93  
 
 94  
 


95 -    def addMethods(self):


96          # override to add _solver function
 
 97          ODEsystem.addMethods(self, usePsyco=HAVE_PSYCO) 
 98          # Jacobian ignored
 
 99          self._solver = euler_solver(getattr(self,self.funcspec.spec[1])) 
100          self._funcreg['_solver'] = ('self', 'euler_solver(getattr(self,' \
 
101                                                    + 'self.funcspec.spec[1]))')

102  
 
103  
 


104 -    def _debug_snapshot(self, solver, dt, inputlist):


105          ivals = [i(solver.t) for i in inputlist] 
106          s = "\n***************\nNew t, x, inputs: " + " ".join([str(s) for s in (solver.t,solver.y,ivals)]) 
107          s += "\ndt="+str(dt)+" f_params="+str(solver.f_params)+" dx/dt=" 
108          s += str(solver.f(solver.t, solver.y, sortedDictValues(self.pars)+ivals)) 
109          return s

110  
 


111 -    def compute(self, trajname, dirn='f', ics=None):


112          continue_integ = ODEsystem.prepDirection(self, dirn) 
113          if self._dircode == -1: 
114              raise NotImplementedError('Backwards integration is not implemented') 
115          if ics is not None: 
116              self.set(ics=ics) 
117          self.validateICs() 
118          self.diagnostics.clearWarnings() 
119          self.diagnostics.clearErrors() 
120          pnames = sortedDictKeys(self.pars) 
121          xnames = self._var_ixmap  # ensures correct order 
122          # Check i.c.'s are well defined (finite)
 
123          self.checkInitialConditions() 
124          haveJac = int(self.haveJacobian()) 
125          indepdom0, indepdom1 = self.indepvariable.depdomain.get() 
126          if continue_integ: 
127              if indepdom0 > self._solver.t: 
128                  print "Previous end time is %f"%self._solver.t 
129                  raise ValueError("Start time not correctly updated for "
 
130                                   "continuing orbit") 
131              x0 = self._solver.y 
132              indepdom0 = self._solver.t 
133              self.indepvariable.depdomain.set((indepdom0,indepdom1)) 
134          else: 
135              x0 = sortedDictValues(self.initialconditions,
 
136                                          self.funcspec.vars) 
137          t0 = indepdom0 
138          dt = self.algparams['init_step'] 
139          # speed up repeated access to solver by making a temp name for it
 
140          solver = self._solver 
141          solver.set_initial_value(x0, t0) 
142          solver.dt = dt 
143          # wrap up each dictionary initial value as a singleton list
 
144          alltData = [t0] 
145          allxDataDict = dict(zip(xnames, map(listid, x0))) 
146          plist = sortedDictValues(self.pars) 
147          extralist = copy(plist) 
148          if self.inputs: 
149              # inputVarList is a list of Variables
 
150              inames = sortedDictKeys(self.inputs) 
151              listend = self.numpars + len(self.inputs) 
152              inputVarList = sortedDictValues(self.inputs) 
153              ilist = _pollInputs(inputVarList, alltData[0]+self.globalt0,
 
154                                 self.checklevel) 
155          else: 
156              ilist = [] 
157              inames = [] 
158              listend = self.numpars 
159              inputVarList = [] 
160          extralist.extend(ilist) 
161          solver.set_f_params(extralist) 
162          if haveJac: 
163              solver.set_jac_params(extralist) 
164          do_poly = self.algparams['poly_interp'] 
165          if do_poly: 
166              rhsfn = getattr(self, self.funcspec.spec[1]) 
167              dx0 = rhsfn(t0, x0, extralist) 
168              alldxDataDict = dict(zip(xnames, map(listid, dx0))) 
169          auxvarsfn = getattr(self,self.funcspec.auxspec[1]) 
170          # Make t mesh if it wasn't given as 'specialtimes'
 
171          if not all(isfinite(self.indepvariable.depdomain.get())): 
172              print "Time domain was: ", self.indepvariable.depdomain.get() 
173              raise ValueError("Ensure time domain is finite") 
174          if dt == indepdom1 - indepdom0: 
175              # single-step integration required
 
176              # special times will not have been set (unless trivially
 
177              # they are [indepdom0, indepdom1])
 
178              tmesh = [indepdom0, indepdom1] 
179          else: 
180              notDone = True 
181              while notDone: 
182                  tmesh = self.indepvariable.depdomain.sample(dt,
 
183                                            strict=True,
 
184                                            avoidendpoints=True) 
185                  notDone = False 
186          eventslist = self.eventstruct.query(['active', 'notvarlinked']) 
187          termevents = self.eventstruct.query(['term'], eventslist) 
188          tmesh.pop(0)  # get rid of first entry for initial condition 
189          xnames = self.funcspec.vars 
190          # storage of all auxiliary variable data
 
191          allaDataDict = {} 
192          anames = self.funcspec.auxvars 
193          avals = auxvarsfn(t0, x0, extralist) 
194          for aix in range(len(anames)): 
195              aname = anames[aix] 
196              try: 
197                  allaDataDict[aname] = [avals[aix]] 
198              except IndexError: 
199                  print "\nEuler generator: There was a problem evaluating " \
 
200                        + "an auxiliary variable" 
201                  print "Debug info: avals (length", len(avals), ") was ", avals 
202                  print "Index out of range was ", aix 
203                  print self.funcspec.auxspec[1] 
204                  print hasattr(self, self.funcspec.auxspec[1]) 
205                  print "Args were:", [t0, x0, extralist] 
206                  raise 
207          # Initialize signs of event detection objects at IC
 
208          self.setEventICs(self.initialconditions, self.globalt0) 
209          if self.inputs: 
210              parsinps = copy(self.pars) 
211              parsinps.update(dict(zip(inames,ilist))) 
212          else: 
213              parsinps = self.pars 
214          if eventslist != []: 
215              dataDict = copy(self.initialconditions) 
216              dataDict.update(dict(zip(anames, avals))) 
217              dataDict['t'] = t0 
218              evsflagged = self.eventstruct.pollHighLevelEvents(None,
 
219                                                              dataDict,
 
220                                                              parsinps,
 
221                                                              eventslist) 
222              if len(evsflagged) > 0: 
223                  raise RuntimeError("Some events flagged at initial condition") 
224              if continue_integ: 
225                  # revert to prevprevsign, since prevsign changed after call
 
226                  self.eventstruct.resetHighLevelEvents(t0, eventslist, 'prev') 
227              elif self._for_hybrid_DS: 
228                  # self._for_hybrid_DS is set internally by HybridModel class
 
229                  # to ensure not to reset events, because they may be about to
 
230                  # flag on first step if previous hybrid state was the same
 
231                  # generator and, for example, two variables are synchronizing
 
232                  # so that their events get very close together.
 
233                  # Just reset the starttimes of these events
 
234                  for evname, ev in eventslist: 
235                      ev.starttime = t0 
236              else: 
237                  # default state is a one-off call to this generator
 
238                  self.eventstruct.resetHighLevelEvents(t0, eventslist, None) 
239                  self.eventstruct.validateEvents(self.funcspec.vars + \
 
240                                              self.funcspec.auxvars + \
 
241                                              self.funcspec.inputs + \
 
242                                              ['t'], eventslist) 
243          evnames = [ev[0] for ev in eventslist] 
244          lastevtime = {}.fromkeys(evnames, None) 
245          # initialize new event info dictionaries
 
246          Evtimes = {} 
247          Evpoints = {} 
248          if continue_integ: 
249              for evname in evnames: 
250                  try: 
251                      # these are in global time, so convert to local time
 
252                      lastevtime[evname] = self.eventstruct.Evtimes[evname][-1] \
 
253                                             - self.globalt0 
254                  except (IndexError, KeyError): 
255                      # IndexError: Evtimes[evname] was None
 
256                      # KeyError: Evtimes does not have key evname
 
257                      pass 
258          for evname in evnames: 
259              Evtimes[evname] = [] 
260              Evpoints[evname] = [] 
261          # temp storage for repeatedly used object attributes (for lookup efficiency)
 
262          depdomains = dict(zip(range(self.dimension),
 
263                          [self.variables[xn].depdomain for xn in xnames])) 
264          # Main integration loop
 
265          num_points = 0 
266          breakwhile = False 
267          while not breakwhile: 
268              try: 
269                  new_t = tmesh.pop(0)  # this destroys tmesh for future use 
270              except IndexError: 
271                  # empty
 
272                  break 
273              # optional user function (not a method)
 
274              self.ufunc_before(self) 
275              try: 
276                  errcode = solver.integrate(dt) 
277              except: 
278                  print "Error calling right hand side function:" 
279                  self.showSpec() 
280                  print "Numerical traceback information (current state, " \
 
281                        + "parameters, etc.)" 
282                  print "in generator dictionary 'traceback'" 
283                  self.traceback = {'vars': dict(zip(xnames,solver.y)),
 
284                                    'pars': dict(zip(pnames,plist)),
 
285                                    'inputs': dict(zip(inames,ilist)),
 
286                                    self.indepvariable.name: new_t} 
287                  raise 
288              avals = auxvarsfn(new_t, solver.y, extralist) 
289              # Uncomment the following assertion for debugging
 
290  #            assert all([isfinite(a) for a in avals]), \
 
291  #               "Some auxiliary variable values not finite"
 
292              if eventslist != []: 
293                  dataDict = dict(zip(xnames,solver.y)) 
294                  dataDict.update(dict(zip(anames,avals))) 
295                  dataDict['t'] = new_t 
296                  if self.inputs: 
297                      parsinps = copy(self.pars) 
298                      parsinps.update(dict(zip(inames,
 
299                                _pollInputs(inputVarList, new_t+self.globalt0,
 
300                                            self.checklevel)))) 
301                  else: 
302                      parsinps = self.pars 
303                  evsflagged = self.eventstruct.pollHighLevelEvents(None,
 
304                                                              dataDict,
 
305                                                              parsinps,
 
306                                                              eventslist) 
307  ##                print new_t, evsflagged
 
308  ##                evsflagged = [ev for ev in evsflagged if solver.t-indepdom0 > ev[1].eventinterval]
 
309                  termevsflagged = filter(lambda e: e in evsflagged, termevents) 
310                  nontermevsflagged = filter(lambda e: e not in termevsflagged,
 
311                                             evsflagged) 
312                  # register any non-terminating events in the warnings
 
313                  # list, unless they are 'precise' in which case flag
 
314                  # them to be resolved after integration completes
 
315                  if len(nontermevsflagged) > 0: 
316                      evnames = [ev[0] for ev in nontermevsflagged] 
317                      precEvts = self.eventstruct.query(['precise'],
 
318                                                            nontermevsflagged) 
319                      # register both precise and non-precise events the same
 
320                      # (Euler currently ignores precise events with its fixed time step)
 
321                      nonprecEvts = self.eventstruct.query(['notprecise'],
 
322                                                           nontermevsflagged) + precEvts 
323                      nonprec_evnames = [e[0] for e in nonprecEvts] + [e[0] for e in precEvts] 
324                      # only record events if they have not been previously
 
325                      # flagged within their event interval
 
326                      if nonprec_evnames != []: 
327                          temp_names = [] 
328                          for evname, ev in nonprecEvts: 
329                              prevevt_time = lastevtime[evname] 
330                              if prevevt_time is None: 
331                                  ignore_ev = False 
332                              else: 
333                                  if solver.t-prevevt_time < ev.eventinterval: 
334                                      ignore_ev = True 
335                                  else: 
336                                      ignore_ev = False 
337                              if not ignore_ev: 
338                                  temp_names.append(evname) 
339                                  lastevtime[evname] = solver.t 
340                          self.diagnostics.warnings.append((W_NONTERMEVENT,
 
341                                       (solver.t, temp_names))) 
342                          for evname in temp_names: 
343                              Evtimes[evname].append(solver.t) 
344                              xv = solver.y 
345                              av = array(avals) 
346                              Evpoints[evname].append(concatenate((xv, av))) 
347                  do_termevs = [] 
348                  if termevsflagged != []: 
349                      # only record events if they have not been previously
 
350                      # flagged within their event interval
 
351                      for e in termevsflagged: 
352                          prevevt_time = lastevtime[e[0]] 
353  ##                        print "Event %s flagged."%e[0]
 
354  ##                        print "  ... last time was ", prevevt_time
 
355  ##                        print "  ... event interval = ", e[1].eventinterval
 
356  ##                        print "  ... t = %f, dt = %f"%(solver.t, dt)
 
357                          if prevevt_time is None: 
358                              ignore_ev = False 
359                          else: 
360  ##                            print "  ... comparison = %f < %f"%(solver.t-dt-prevevt_time, e[1].eventinterval)
 
361                              if solver.t-dt-prevevt_time < e[1].eventinterval: 
362                                  ignore_ev = True 
363  ##                                print "Euler ignore ev"
 
364                              else: 
365                                  ignore_ev = False 
366                          if not ignore_ev: 
367                              do_termevs.append(e) 
368                  if len(do_termevs) > 0: 
369                      # >= 1 active terminal event flagged at this time point
 
370                      evnames = [ev[0] for ev in do_termevs] 
371                      self.diagnostics.warnings.append((W_TERMEVENT, \
 
372                                           (solver.t, evnames))) 
373                      first_found_t = solver.t 
374                      for evname in evnames: 
375                          Evtimes[evname].append(solver.t) 
376                          xv = solver.y 
377                          av = array(avals) 
378                          Evpoints[evname].append(concatenate((xv, av))) 
379                      # break while loop after appending t, x
 
380                      breakwhile = True 
381              # after events have had a chance to be detected at a state boundary
 
382              # we check for any that have not been caught by an event (will be
 
383              # much less accurately determined)
 
384              if not breakwhile: 
385                  # only here if a terminal event hasn't just flagged
 
386                  for xi in xrange(self.dimension): 
387                      if not self.contains(depdomains[xi],
 
388                                       solver.y[xi],
 
389                                       self.checklevel): 
390                          self.diagnostics.warnings.append((W_TERMSTATEBD,
 
391                                      (solver.t,
 
392                                       xnames[xi],solver.y[xi],
 
393                                       depdomains[xi].get()))) 
394                          breakwhile = True 
395                          break  # for loop 
396                  if breakwhile: 
397                      break  # while loop 
398              alltData.append(solver.t) 
399              if do_poly: 
400                  dxvals = rhsfn(solver.t, solver.y, extralist) 
401                  for xi, xname in enumerate(xnames): 
402                      allxDataDict[xname].append(solver.y[xi]) 
403                      alldxDataDict[xname].append(dxvals[xi]) 
404              else: 
405                  for xi, xname in enumerate(xnames): 
406                      allxDataDict[xname].append(solver.y[xi]) 
407              for aix, aname in enumerate(anames): 
408                  allaDataDict[aname].append(avals[aix]) 
409              num_points += 1 
410              if not breakwhile: 
411                  try: 
412                      extralist[self.numpars:listend] = [f(solver.t+self.globalt0,
 
413                                                           self.checklevel) \
 
414                                                    for f in inputVarList] 
415                  except ValueError: 
416                      print 'External input call caused value out of range error:',\
 
417                            't = ', solver.t 
418                      for f in inputVarList: 
419                          if f.diagnostics.hasWarnings(): 
420                              print 'External input variable %s out of range:'%f.name 
421                              print '   t = ', repr(f.diagnostics.warnings[-1][0]), ', ', \
 
422                                    f.name, ' = ', repr(f.diagnostics.warnings[-1][1]) 
423                      raise 
424                  except AssertionError: 
425                      print 'External input call caused t out of range error: t = ', \
 
426                            solver.t 
427                      raise 
428                  solver.set_f_params(extralist) 
429                  breakwhile = not solver.successful() 
430              # optional user function (not a method)
 
431              self.ufunc_after(self) 
432  
 
433          # Check that any terminal events found terminated the code correctly
 
434          if first_found_t is not None: 
435              # ... then terminal events were found.
 
436              try: 
437                  if self.diagnostics.warnings[-1][0] not in [W_TERMEVENT,
 
438                                                              W_TERMSTATEBD]: 
439                      print "t =", solver.t 
440                      print "state =", dict(zip(xnames,solver.y)) 
441                      raise RuntimeError("Event finding code for terminal event "
 
442                                         "failed in Generator " + self.name + \
 
443                                         ": try decreasing eventdelay or "
 
444                                         "eventinterval below eventtol, or the "
 
445                                         "atol and rtol parameters") 
446              except IndexError: 
447                  info(self.diagnostics.outputStats, "Output statistics") 
448                  print "t =", solver.t 
449                  print "x =", solver.y 
450                  raise RuntimeError("Event finding failed in Generator " + \
 
451                                     self.name + ": try decreasing eventdelay "
 
452                                     "or eventinterval below eventtol") 
453          # Package up computed trajectory in Variable variables
 
454          # Add external inputs warnings to self.diagnostics.warnings, if any
 
455          for f in inputVarList: 
456              for winfo in f.diagnostics.warnings: 
457                  self.diagnostics.warnings.append((W_NONTERMSTATEBD,
 
458                                       (winfo[0], f.name, winfo[1],
 
459                                        f.depdomain.get()))) 
460          # check for non-unique terminal event
 
461          termcount = 0 
462          for (w,i) in self.diagnostics.warnings: 
463              if w == W_TERMEVENT or w == W_TERMSTATEBD: 
464                  termcount += 1 
465                  if termcount > 1: 
466                      self.diagnostics.errors.append((E_NONUNIQUETERM,
 
467                                                      (alltData[-1], i[1]))) 
468          # uncomment the following lines for debugging
 
469  #        assert len(alltData) == len(allxDataDict.values()[0]) \
 
470  #             == len(allaDataDict.values()[0]), "Output data size mismatch"
 
471  #        for val_list in allaDataDict.values():
 
472  #            assert all([isfinite(x) for x in val_list])
 
473          # Create variables (self.variables contains no actual data)
 
474          # These versions of the variables are only final if no non-terminal
 
475          # events need to be inserted.
 
476          variables = copyVarDict(self.variables) 
477          for x in xnames: 
478              if len(alltData) > 1: 
479                  if do_poly: 
480                      xvals = array([allxDataDict[x], alldxDataDict[x]]).T 
481                      interp = PiecewisePolynomial(alltData, xvals, 2) 
482                  else: 
483                      xvals = allxDataDict[x] 
484                      interp = interp1d(alltData, xvals) 
485                  variables[x] = Variable(interp, 't', x, x) 
486              else: 
487                  print "Error in Generator:", self.name 
488                  print "t = ", alltData 
489                  print "x = ", allxDataDict 
490                  raise PyDSTool_ValueError("Fewer than 2 data points computed") 
491          for a in anames: 
492              if len(alltData) > 1: 
493                  variables[a] = Variable(interp1d(alltData, allaDataDict[a]),
 
494                                          't', a, a) 
495              else: 
496                  print "Error in Generator:", self.name 
497                  print "t = ", alltData 
498                  print "x = ", allxDataDict 
499                  raise PyDSTool_ValueError("Fewer than 2 data points computed") 
500          self.diagnostics.outputStats = {'last_step': dt,
 
501                              'num_fcns': num_points,
 
502                              'num_steps': num_points,
 
503                              'errorStatus': errcode
 
504                              } 
505          if solver.successful(): 
506              #self.validateSpec()
 
507              for evname, evtlist in Evtimes.iteritems(): 
508                  try: 
509                      self.eventstruct.Evtimes[evname].extend([et+self.globalt0 \
 
510                                              for et in evtlist]) 
511                  except KeyError: 
512                      self.eventstruct.Evtimes[evname] = [et+self.globalt0 \
 
513                                              for et in evtlist] 
514              # build event pointset information (reset previous trajectory's)
 
515              self.trajevents = {} 
516              for (evname, ev) in eventslist: 
517                  evpt = Evpoints[evname] 
518                  if evpt == []: 
519                      self.trajevents[evname] = None 
520                  else: 
521                      evpt = transpose(array(evpt)) 
522                      self.trajevents[evname] = Pointset({'coordnames': xnames+anames,
 
523                                                 'indepvarname': 't',
 
524                                                 'coordarray': evpt,
 
525                                                 'indepvararray': Evtimes[evname],
 
526                                                 'indepvartype': float}) 
527              self.defined = True 
528              return Trajectory(trajname, variables.values(),
 
529                                abseps=self._abseps, globalt0=self.globalt0,
 
530                                checklevel=self.checklevel,
 
531                                FScompatibleNames=self._FScompatibleNames,
 
532                                FScompatibleNamesInv=self._FScompatibleNamesInv,
 
533                                events=self.trajevents,
 
534                                modelNames=self.name,
 
535                                modelEventStructs=self.eventstruct) 
536          else: 
537              try: 
538                  self.diagnostics.errors.append((E_COMPUTFAIL, (solver.t,
 
539                                      self.diagnostics._errorcodes[errcode]))) 
540              except TypeError: 
541                  # e.g. when errcode has been used to return info list
 
542                  print "Error information: ", errcode 
543                  self.diagnostics.errors.append((E_COMPUTFAIL, (solver.t,
 
544                                      self.diagnostics._errorcodes[0]))) 
545              self.defined = False

546  
 
547  
 


548 -    def Rhs(self, t, xdict, pdict=None, asarray=True):


549          """asarray is an unused, dummy argument for compatibility with Model.Rhs""" 
550          # don't need to convert names to FS-compatible as they sort
 
551          # the same
 
552          # also, ensure xdict doesn't contain elements like array([4.1]) instead of 4
 
553          x = [float(val) for val in sortedDictValues(filteredDict(xdict, self.funcspec.vars))] 
554          if pdict is None: 
555              pdict = self.pars 
556          p = sortedDictValues(pdict) 
557          i = _pollInputs(sortedDictValues(self.inputs), t, self.checklevel) 
558          return apply(getattr(self, self.funcspec.spec[1]), [t, x, p+i])

559  
 
560  
 


561 -    def Jacobian(self, t, xdict, pdict=None, asarray=True):


562          """asarray is an unused, dummy argument for compatibility with
 
563          Model.Jacobian""" 
564          if self.haveJacobian(): 
565              # also, ensure xdict doesn't contain elements like array([4.1]) instead of 4
 
566              x = [float(val) for val in sortedDictValues(filteredDict(xdict,
 
567                                                                       self.funcspec.vars))] 
568              if pdict is None: 
569                  pdict = self.pars 
570              p = sortedDictValues(pdict) 
571              i = _pollInputs(sortedDictValues(self.inputs), t, self.checklevel) 
572              return apply(getattr(self, self.funcspec.auxfns["Jacobian"][1]), \
 
573                           [t, x, p+i]) 
574          else: 
575              raise PyDSTool_ExistError("Jacobian not defined")

576  
 
577  
 


578 -    def JacobianP(self, t, xdict, pdict=None, asarray=True):


579          """asarray is an unused, dummy argument for compatibility with
 
580          Model.JacobianP""" 
581          if self.haveJacobian_pars(): 
582              # also, ensure xdict doesn't contain elements like array([4.1]) instead of 4
 
583              x = [float(val) for val in sortedDictValues(filteredDict(xdict,
 
584                                                                       self.funcspec.vars))] 
585              if pdict is None: 
586                  pdict = self.pars 
587              p = sortedDictValues(pdict) 
588              i = _pollInputs(sortedDictValues(self.inputs), t, self.checklevel) 
589              return apply(getattr(self, self.funcspec.auxfns["Jacobian_pars"][1]), \
 
590                          [t, x, p+i]) 
591          else: 
592              raise PyDSTool_ExistError("Jacobian w.r.t. parameters not defined")

593  
 
594  
 


595 -    def AuxVars(self, t, xdict, pdict=None, asarray=True):


596          """asarray is an unused, dummy argument for compatibility with
 
597          Model.AuxVars""" 
598          # also, ensure xdict doesn't contain elements like array([4.1]) instead of 4
 
599          x = [float(val) for val in sortedDictValues(filteredDict(xdict,
 
600                                                                   self.funcspec.vars))] 
601          if pdict is None: 
602              pdict = self.pars 
603          p = sortedDictValues(pdict) 
604          i = _pollInputs(sortedDictValues(self.inputs), t, self.checklevel) 
605          return apply(getattr(self, self.funcspec.auxspec[1]), [t, x, p+i])

606  
 
607  
 


608 -    def __del__(self):


609          ODEsystem.__del__(self)

610  
 
611  
 
612  
 
613  # Register this Generator with the database
 
614  
 
615  symbolMapDict = {} 
616  # in future, provide appropriate mappings for libraries math,
 
617  # random, etc. (for now it's left to FuncSpec)
 
618  theGenSpecHelper.add(Euler_ODEsystem, symbolMapDict, 'python') 
619
```

  


| Home | Trees | Indices | Help | | PyDSTool | | --- | |
| --- | --- | --- | --- | --- | --- |

|  |  |
| --- | --- |
| Generated by Epydoc 3.0.1 on Fri May 4 15:24:12 2012 | http://epydoc.sourceforge.net |
