# Supplementary material for: Hybrid Models and Biological Model Reduction with PyDSTool
Source: PLoS Comput Biol. 2012 Aug 9;8(8):e1002628. doi: 10.1371/journal.pcbi.1002628 (PMC3415397; doi:10.1371/journal.pcbi.1002628)
Supplement: Text S4 — Complete source code for the PyDSTool package (version 0.88.120504). Includes API documentation and help files linking to web pages. This file is identical to the current public release on Sourceforge.net. (ZIP) [file pcbi.1002628.s004.zip › PyDSTool/html/PyDSTool.Generator.Euler_ODEsystem'.Euler_ODEsystem-class.html]

xml version="1.0" encoding="ascii"?


PyDSTool.Generator.Euler\_ODEsystem'.Euler\_ODEsystem


| Home | Trees | Indices | Help | | PyDSTool | | --- | |
| --- | --- | --- | --- | --- | --- |

|  |  |  |  |
| --- | --- | --- | --- |
| Package PyDSTool :: Package Generator :: Module Euler\_ODEsystem' :: Class Euler\_ODEsystem | |  | | --- | | [hide private] | | [frames] | no frames] | |

# Class Euler\_ODEsystem

source code

```
           object --+            
                    |            
baseclasses.Generator --+        
                        |        
       baseclasses.ctsGen --+    
                            |    
         ODEsystem'.ODEsystem --+
                                |
                               Euler_ODEsystem
```

---

Euler method. Fixed step.

Uses Python target language only for functional specifications.


|  |  |  |  |
| --- | --- | --- | --- |
| |  |  | | --- | --- | | Instance Methods | [hide private] | | |
|  | |  |  | | --- | --- | | AuxVars(self, t, xdict, pdict=None, asarray=True)  asarray is an unused, dummy argument for compatibility with Model.AuxVars | source code | |
|  | |  |  | | --- | --- | | Jacobian(self, t, xdict, pdict=None, asarray=True)  asarray is an unused, dummy argument for compatibility with Model.Jacobian | source code | |
|  | |  |  | | --- | --- | | JacobianP(self, t, xdict, pdict=None, asarray=True)  asarray is an unused, dummy argument for compatibility with Model.JacobianP | source code | |
|  | |  |  | | --- | --- | | Rhs(self, t, xdict, pdict=None, asarray=True)  asarray is an unused, dummy argument for compatibility with Model.Rhs | source code | |
|  | |  |  | | --- | --- | | \_\_del\_\_(self) | source code | |
|  | |  |  | | --- | --- | | \_\_init\_\_(self, kw)  x.\_\_init\_\_(...) initializes x; see x.\_\_class\_\_.\_\_doc\_\_ for signature | source code | |
|  | |  |  | | --- | --- | | \_debug\_snapshot(self, solver, dt, inputlist) | source code | |
|  | |  |  | | --- | --- | | addMethods(self)  Add Python-specific functions to this object's methods, accelerating them with psyco, if it is available. | source code | |
|  | |  |  | | --- | --- | | compute(self, trajname, dirn=`'``f``'`, ics=None)  This is an abstract class. | source code | |
| **Inherited from `ODEsystem'.ODEsystem`**: `__getstate__`, `__setstate__`, `checkInitialConditions`, `cleanupMemory`, `haveJacobian`, `haveJacobian_pars`, `haveMass`, `prepDirection`, `set`, `validateICs`  **Inherited from `baseclasses.ctsGen`**: `validateSpec`  **Inherited from `baseclasses.Generator`**: `__copy__`, `__deepcopy__`, `__repr__`, `__str__`, `addEvtPars`, `checkArgs`, `contains`, `get`, `getEventTimes`, `getEvents`, `info`, `query`, `resetEventTimes`, `resetEvents`, `setEventICs`, `showAuxFnSpec`, `showAuxSpec`, `showEventSpec`, `showSpec`  **Inherited from `baseclasses.Generator`** (private): `_addEvents`, `_auxfn_getindex`, `_auxfn_globalindepvar`, `_auxfn_heav`, `_auxfn_if`, `_auxfn_initcond`, `_generate_ixmaps`, `_infostr`, `_kw_process_algparams`, `_kw_process_allvars`, `_kw_process_dispatch`, `_kw_process_events`, `_kw_process_fnspecs`, `_kw_process_ics`, `_kw_process_ignorespecial`, `_kw_process_inputs`, `_kw_process_pars`, `_kw_process_pdomain`, `_kw_process_reuseterms`, `_kw_process_system`, `_kw_process_target`, `_kw_process_tdata`, `_kw_process_tdomain`, `_kw_process_tstep`, `_kw_process_ttype`, `_kw_process_varspecs`, `_kw_process_vfcodeinserts`, `_kw_process_xdomain`, `_kw_process_xtype`, `_makeBoundsEvents`, `_register`, `_set_for_hybrid_DS`  **Inherited from `object`**: `__delattr__`, `__getattribute__`, `__hash__`, `__new__`, `__reduce__`, `__reduce_ex__`, `__setattr__` | |


|  |  |  |  |
| --- | --- | --- | --- |
| |  |  | | --- | --- | | Class Variables | [hide private] | | |
| **Inherited from `ODEsystem'.ODEsystem`** (private): `_needKeys`, `_optionalKeys`, `_validKeys`  **Inherited from `baseclasses.Generator`** (private): `_querykeys` | |


|  |  |  |  |
| --- | --- | --- | --- |
| |  |  | | --- | --- | | Properties | [hide private] | | |
| **Inherited from `object`**: `__class__` | |


|  |  |  |  |
| --- | --- | --- | --- |
| |  |  | | --- | --- | | Method Details | [hide private] | | |

|  |  |  |
| --- | --- | --- |
| |  |  | | --- | --- | | AuxVars(self, t, xdict, pdict=None, asarray=True) | source code |   asarray is an unused, dummy argument for compatibility with Model.AuxVars  Overrides: ODEsystem'.ODEsystem.AuxVars |

|  |  |  |
| --- | --- | --- |
| |  |  | | --- | --- | | Jacobian(self, t, xdict, pdict=None, asarray=True) | source code |   asarray is an unused, dummy argument for compatibility with Model.Jacobian  Overrides: ODEsystem'.ODEsystem.Jacobian |

|  |  |  |
| --- | --- | --- |
| |  |  | | --- | --- | | JacobianP(self, t, xdict, pdict=None, asarray=True) | source code |   asarray is an unused, dummy argument for compatibility with Model.JacobianP  Overrides: ODEsystem'.ODEsystem.JacobianP |

|  |  |  |
| --- | --- | --- |
| |  |  | | --- | --- | | Rhs(self, t, xdict, pdict=None, asarray=True) | source code |   asarray is an unused, dummy argument for compatibility with Model.Rhs  Overrides: ODEsystem'.ODEsystem.Rhs |

|  |  |  |
| --- | --- | --- |
| |  |  | | --- | --- | | \_\_del\_\_(self)  *(Destructor)* | source code |   Overrides: baseclasses.Generator.\_\_del\_\_ |

|  |  |  |
| --- | --- | --- |
| |  |  | | --- | --- | | \_\_init\_\_(self, kw)  *(Constructor)* | source code |   x.\_\_init\_\_(...) initializes x; see x.\_\_class\_\_.\_\_doc\_\_ for signature  Overrides: object.\_\_init\_\_ *(inherited documentation)* |

|  |  |  |
| --- | --- | --- |
| |  |  | | --- | --- | | addMethods(self) | source code |   Add Python-specific functions to this object's methods, accelerating them with psyco, if it is available.  Overrides: ODEsystem'.ODEsystem.addMethods *(inherited documentation)* |

|  |  |  |
| --- | --- | --- |
| |  |  | | --- | --- | | compute(self, trajname, dirn=`'``f``'`, ics=None) | source code |   This is an abstract class.  Overrides: ODEsystem'.ODEsystem.compute *(inherited documentation)* |

  


| Home | Trees | Indices | Help | | PyDSTool | | --- | |
| --- | --- | --- | --- | --- | --- |

|  |  |
| --- | --- |
| Generated by Epydoc 3.0.1 on Fri May 4 15:24:06 2012 | http://epydoc.sourceforge.net |
