# Supplementary material for: Hybrid Models and Biological Model Reduction with PyDSTool
Source: PLoS Comput Biol. 2012 Aug 9;8(8):e1002628. doi: 10.1371/journal.pcbi.1002628 (PMC3415397; doi:10.1371/journal.pcbi.1002628)
Supplement: Text S4 — Complete source code for the PyDSTool package (version 0.88.120504). Includes API documentation and help files linking to web pages. This file is identical to the current public release on Sourceforge.net. (ZIP) [file pcbi.1002628.s004.zip › PyDSTool/html/PyDSTool.Generator.ExplicitFnGen'-pysrc.html]

xml version="1.0" encoding="ascii"?


PyDSTool.Generator.ExplicitFnGen'


| Home | Trees | Indices | Help | | PyDSTool | | --- | |
| --- | --- | --- | --- | --- | --- |

|  |  |  |  |
| --- | --- | --- | --- |
| Package PyDSTool :: Package Generator :: Module ExplicitFnGen' | |  | | --- | | [hide private] | | [frames] | no frames] | |

# Source Code for Module PyDSTool.Generator.ExplicitFnGen'

```
  1  # Explicit function generator
 
  2  from __future__ import division 
  3  
 
  4  from allimports import * 
  5  from baseclasses import ctsGen, theGenSpecHelper, \
 
  6       auxfn_container, _pollInputs 
  7  from PyDSTool.utils import * 
  8  from PyDSTool.common import * 
  9  from PyDSTool.Interval import uncertain 
 10  
 
 11  # Other imports
 
 12  from numpy import Inf, NaN, isfinite, sometrue, alltrue, array, arange, \
 
 13       transpose, shape 
 14  import math, random, types 
 15  from copy import copy, deepcopy 
 16  try: 
 17      # use pscyo JIT byte-compiler optimization, if available
 
 18      import psyco 
 19      HAVE_PSYCO = True 
 20  except ImportError: 
 21      HAVE_PSYCO = False 
 22  
 
 23  
 


24 -class ExplicitFnGen(ctsGen):


25      """Explicit functional form specifying a trajectory.
 
 26  
 
 27      E.g. for an external input. This class allows parametric
 
 28      forms of the function, but with no dependence on x or its
 
 29      own external inputs.""" 
 30      _validKeys = ['globalt0', 'xdomain', 'tdata', 'tdomain',
 
 31                       'ics', 'pars', 'checklevel', 'pdomain', 'abseps'] 
 32      _needKeys = ctsGen._needKeys + ['varspecs'] 
 33      _optionalKeys = ctsGen._optionalKeys + ['tdomain', 'pars', 'pdomain', 'xdomain',
 
 34                                    'xtype', 'ics', 'auxvars', 'vars', 'events',
 
 35                                    'fnspecs', 'tdata', 'enforcebounds',
 
 36                                    'activatedbounds', 'reuseterms'] 
 37  
 


38 -    def __init__(self, kw):


39          ctsGen.__init__(self, kw) 
 40          dispatch_list = ['varspecs', 'tdomain', 'tdata', 'xtype', 'xdomain',
 
 41                           'ics', 'allvars', 'reuseterms', 'pars', 'pdomain',
 
 42                           'fnspecs', 'target'] 
 43          # allow inputs only if it's empty, for compatibility with ModelConstructor
 
 44          # which might put an empty dictionary in for this key
 
 45          if 'inputs' in kw: 
 46              if kw['inputs'] != {}: 
 47                  raise PyDSTool_KeyError('inputs option invalid for ExplicitFnGen '
 
 48                                      'class') 
 49          self.funcspec = ExpFuncSpec(self._kw_process_dispatch(dispatch_list,
 
 50                                                                kw)) 
 51          self.indepvartype = float 
 52          for s in self.funcspec.spec[0]: 
 53              if s.find('x[') > -1: 
 54                  raise ValueError('Variable values cannot depend on '
 
 55                              'other variables in explicit function specs -- '
 
 56                              'in function:\n'+s) 
 57          self._kw_process_events(kw) 
 58          self.checkArgs(kw) 
 59          self.indepvariable = Variable(listid, Interval('t_domain',
 
 60                                                         self.indepvartype,
 
 61                                                self.tdomain, self._abseps),
 
 62                               Interval('t', self.indepvartype, self.tdata,
 
 63                                        self._abseps), 't') 
 64          self._register(self.indepvariable) 
 65          for x in self.funcspec.vars + self.funcspec.auxvars: 
 66              try: 
 67                  xinterval=Interval(x, self.xtype[x], self.xdomain[x], self._abseps) 
 68              except KeyError, e: 
 69                  raise PyDSTool_KeyError('Mismatch between declared variables '
 
 70                                   'and xspecs: ' + str(e)) 
 71              # placeholder variable so that this class can be
 
 72              # copied before it is defined (listid function is a dummy)
 
 73              self.variables[x] = Variable(None, self.indepvariable.depdomain,
 
 74                                           xinterval, x) 
 75          self._generate_ixmaps() 
 76          self.auxfns = auxfn_container(self) 
 77          self.addMethods(usePsyco=HAVE_PSYCO)

 78  
 


79 -    def addMethods(self, usePsyco=False):


80          """Add Python-specific functions to this object's methods,
 
 81          accelerating them with psyco, if it is available.""" 
 82  
 
 83          # Add the auxiliary function specs to this Generator's namespace
 
 84          for auxfnname in self.funcspec._pyauxfns: 
 85              fninfo = self.funcspec._pyauxfns[auxfnname] 
 86              if not hasattr(self, fninfo[1]): 
 87                  # user-defined auxiliary functions
 
 88                  # (built-ins are provided explicitly)
 
 89                  try: 
 90                      exec fninfo[0] 
 91                  except: 
 92                      print 'Error in supplied auxiliary function code' 
 93                  self._funcreg[fninfo[1]] = ('self', fninfo[0]) 
 94                  setattr(self, fninfo[1], types.MethodType(locals()[fninfo[1]],
 
 95                                                             self,
 
 96                                                             self.__class__)) 
 97                  # user auxiliary function interface wrapper
 
 98                  try: 
 99                      uafi_code = self.funcspec._user_auxfn_interface[auxfnname] 
100                      try: 
101                          exec uafi_code 
102                      except: 
103                          print 'Error in auxiliary function wrapper' 
104                          raise 
105                      setattr(self.auxfns, auxfnname,
 
106                              types.MethodType(locals()[auxfnname], self.auxfns,
 
107                                           auxfn_container)) 
108                      self._funcreg[auxfnname] = ('', uafi_code) 
109                  except KeyError: 
110                      # not a user-defined aux fn
 
111                      pass 
112              # bind all the auxfns here
 
113              if HAVE_PSYCO and usePsyco: 
114                  psyco.bind(getattr(self, fninfo[1])) 
115                  try: 
116                      psyco.bind(self.auxfns[auxfnname]) 
117                  except KeyError: 
118                      # not a user-defined aux fn
 
119                      pass 
120          # Add the spec function to this Generator's namespace if
 
121          # target language is python (otherwise integrator exposes it anyway)
 
122          if self.funcspec.targetlang == 'python': 
123              fninfo = self.funcspec.spec 
124              try: 
125                  exec fninfo[0] 
126              except: 
127                  print 'Error in supplied functional specification code' 
128                  raise 
129              self._funcreg[fninfo[1]] = ('self', fninfo[0]) 
130              setattr(self, fninfo[1], types.MethodType(locals()[fninfo[1]],
 
131                                                             self,
 
132                                                             self.__class__)) 
133              if HAVE_PSYCO and usePsyco: 
134                  psyco.bind(getattr(self, fninfo[1])) 
135              # Add the auxiliary spec function (if present) to this
 
136              # Generator's namespace
 
137              if self.funcspec.auxspec != '': 
138                  fninfo = self.funcspec.auxspec 
139                  try: 
140                      exec fninfo[0] 
141                  except: 
142                      print 'Error in supplied auxiliary variable code' 
143                      raise 
144                  self._funcreg[fninfo[1]] = ('self', fninfo[0]) 
145                  setattr(self, fninfo[1], types.MethodType(locals()[fninfo[1]],
 
146                                                             self,
 
147                                                             self.__class__)) 
148                  if HAVE_PSYCO and usePsyco: 
149                      psyco.bind(getattr(self, fninfo[1]))

150  
 
151  
 


152 -    def compute(self, trajname, ics=None):


153          """Attach specification functions to callable interface.""" 
154          # repeat this check (made in __init__) in case events were added since
 
155          assert self.eventstruct.getLowLevelEvents() == [], \
 
156                 "Can only pass high level events to ExplicitFnGen objects" 
157          assert self.eventstruct.query(['highlevel', 'varlinked']) == [], \
 
158                 "Only non-variable linked events are valid for this class" 
159  ##        icdict_local = copy(self.initialconditions)
 
160  ##        t0 = self.indepvariable.depdomain[0]
 
161  ##        icdict_local['t'] = t0
 
162  ##        have_aux = len(self.funcspec.auxvars)>0
 
163  ##        for a in self.funcspec.auxvars:
 
164  ##            # these functions are intended to be methods in their target
 
165  ##            # Variable object, so expect a first argument 'self'
 
166  ##            exec self.funcspec.auxspec[0]
 
167  ##        if have_aux:
 
168  ##            self.initialconditions.update(dict(zip(self.funcspec.auxvars,
 
169  ##                    apply(locals()[self.funcspec.auxspec[1]],
 
170  ##                                  (None, t0, sortedDictValues(icdict_local),
 
171  ##                                       sortedDictValues(self.pars))) )))
 
172          if ics is not None: 
173              self.set(ics=ics) 
174          self.setEventICs(self.initialconditions, self.globalt0) 
175          tempfs = deepcopy(self.funcspec) 
176          tempvars = copyVarDict(self.variables) 
177          # make unique fn for this trajectory: function definition gets executed
 
178          # finally in Variable.addMethods() method
 
179          tempspec = makeUniqueFn(copy(tempfs.spec[0]), 7, self.name) 
180          tempfs.spec = tempspec 
181          for x in self.funcspec.vars: 
182              x_ix = self.funcspec.vars.index(x) 
183              funcname = "_mapspecfn_" + x + "_" + timestamp(7) 
184              funcstr = "def " + funcname + "(self, t):\n\treturn " 
185              if len(self.funcspec.vars) == 1: 
186                  # this clause is unnecessary if [0] is ever dropped
 
187                  # i.e. if spec would return plain scalar in 1D case
 
188                  funcstr += tempfs.spec[1] + "(self, t, [0], " \
 
189                         + repr(sortedDictValues(self.pars)) + ")[0]\n" 
190              else: 
191                  funcstr += tempfs.spec[1] + "(self, t, [0], " \
 
192                         + repr(sortedDictValues(self.pars)) + ")[" \
 
193                         + str(x_ix) + "]\n" 
194              tempvars[x].setOutput((funcname, funcstr), tempfs,
 
195                                          self.globalt0, self._var_namemap,
 
196                                          copy(self.initialconditions)) 
197          if self.funcspec.auxvars != []: 
198              # make unique fn for this trajectory
 
199              tempauxspec = makeUniqueFn(copy(tempfs.auxspec[0]), 7, self.name) 
200              tempfs.auxspec = tempauxspec 
201          for a in self.funcspec.auxvars: 
202              a_ix = self.funcspec.auxvars.index(a) 
203              funcname = "_mapspecfn_" + a + "_" + timestamp(7) 
204              funcstr = "def " + funcname + "(self, t):\n\treturn " 
205              if len(self.funcspec.auxvars) == 1: 
206                  # this clause is unnecessary if [0] is ever dropped
 
207                  # i.e. if auxspec would return plain scalar in 1D case
 
208                  funcstr += tempfs.auxspec[1] + "(self, t, [v(t) " \
 
209                        + "for v in self._refvars], " \
 
210                        + repr(sortedDictValues(self.pars)) \
 
211                        + ")[0]\n" 
212              else: 
213                  funcstr += tempfs.auxspec[1] + "(self, t, [v(t) " \
 
214                        + "for v in self._refvars], " \
 
215                        + repr(sortedDictValues(self.pars)) \
 
216                        + ")[" + str(a_ix) + "]\n" 
217              tempvars[a].setOutput((funcname, funcstr), tempfs,
 
218                                          self.globalt0, self.funcspec.auxvars,
 
219                                          copy(self.initialconditions),
 
220                                          sortedDictValues(tempvars,
 
221                                                           self.funcspec.vars)) 
222          self.diagnostics.clearWarnings() 
223          self.diagnostics.clearErrors() 
224          # Find any events in tdomain, and adjust tdomain in case they
 
225          # are terminal
 
226          eventslist = self.eventstruct.query(['highlevel', 'active',
 
227                                               'notvarlinked']) 
228          termevents = self.eventstruct.query(['term'], eventslist) 
229          Evtimes = {} 
230          Evpoints = {} 
231          for evname, ev in eventslist: 
232              Evtimes[evname] = [] 
233              Evpoints[evname] = [] 
234          if eventslist != []: 
235              if self._for_hybrid_DS: 
236                  # self._for_hybrid_DS is set internally by HybridModel class
 
237                  # to ensure not to reset events, because they may be about to
 
238                  # flag on first step if previous hybrid state was the same
 
239                  # generator and, for example, two variables are synchronizing
 
240                  # so that their events get very close together.
 
241                  # Just reset the starttimes of these events
 
242                  for evname, ev in eventslist: 
243                      ev.starttime = t0 
244              else: 
245                  self.eventstruct.resetHighLevelEvents(self.indepvariable.depdomain[0],
 
246                                                    eventslist) 
247                  self.eventstruct.validateEvents(self.funcspec.vars + \
 
248                                              self.funcspec.auxvars + \
 
249                                              ['t'], eventslist) 
250              for evname, ev in eventslist: 
251                  # select only continuous-valued variables for event detection
 
252                  # (in case of indicator variables used in hybrid systems)
 
253                  evsfound = ev.searchForEvents(self.indepvariable.depdomain.get(),
 
254                                                parDict=self.pars,
 
255                                                vars=copyVarDict(tempvars, only_cts=True),
 
256                                                checklevel=self.checklevel) 
257                  tvals = sortedDictValues(tempvars) 
258                  for evinfo in evsfound: 
259                      Evtimes[evname].append(evinfo[0]) 
260                      Evpoints[evname].append(array([v(evinfo[0]) for v in tvals])) 
261              self.eventstruct.resetHighLevelEvents(self.indepvariable.depdomain[0],
 
262                                                    eventslist) 
263              self.eventstruct.validateEvents(self.funcspec.vars + \
 
264                                              self.funcspec.auxvars + \
 
265                                              ['t'], eventslist) 
266              termevtimes = {} 
267              nontermevtimes = {} 
268              for evname, ev in eventslist: 
269                  numevs = shape(Evtimes[evname])[-1] 
270                  if numevs == 0: 
271                      continue 
272                  if ev.activeFlag: 
273                      if numevs > 1: 
274                          print "Event info:", Evtimes[evname] 
275                      assert numevs <= 1, ("Internal error: more than one "
 
276                                       "terminal event of same type found") 
277                      # For safety, we should assert that this event
 
278                      # also appears in termevents, but we don't
 
279                      if Evtimes[evname][0] in termevtimes.keys(): 
280                          # append event name to this warning
 
281                          warning_ix = termevtimes[Evtimes[evname][0]] 
282                          self.diagnostics.warnings[warning_ix][1][1].append(evname) 
283                      else: 
284                          # make new termevtime entry for the new warning
 
285                          termevtimes[Evtimes[evname][0]] = \
 
286                                     len(self.diagnostics.warnings) 
287                          self.diagnostics.warnings.append((W_TERMEVENT,
 
288                                           (Evtimes[evname][0],
 
289                                           [evname]))) 
290                  else: 
291                      for ev in range(numevs): 
292                          if Evtimes[evname][ev] in nontermevtimes.keys(): 
293                              # append event name to this warning
 
294                              warning_ix = nontermevtimes[Evtimes[evname][ev]] 
295                              self.diagnostics.warnings[warning_ix][1][1].append(evname) 
296                          else: 
297                              # make new nontermevtime entry for the new warning
 
298                              nontermevtimes[Evtimes[evname][ev]] = \
 
299                                                  len(self.diagnostics.warnings) 
300                              self.diagnostics.warnings.append((W_NONTERMEVENT,
 
301                                               (Evtimes[evname][ev],
 
302                                                [evname]))) 
303          termcount = 0 
304          earliest_termtime = self.indepvariable.depdomain[1] 
305          for (w,i) in self.diagnostics.warnings: 
306              if w == W_TERMEVENT or w == W_TERMSTATEBD: 
307                  termcount += 1 
308                  if i[0] < earliest_termtime: 
309                      earliest_termtime = i[0] 
310          # now delete any events found after the earliest terminal event, if any
 
311          if termcount > 0: 
312              warn_temp = [] 
313              for (w,i) in self.diagnostics.warnings: 
314                  if i[0] <= earliest_termtime: 
315                      warn_temp.append((w,i)) 
316              self.diagnostics.warnings = warn_temp 
317          self.indepvariable.depdomain.set([self.indepvariable.depdomain[0],
 
318                                            earliest_termtime]) 
319          for v in tempvars.values(): 
320              v.indepdomain.set(self.indepvariable.depdomain.get()) 
321  ##                print 'Time interval adjusted according to %s: %s' % \
 
322  ##                      (self._warnmessages[w], str(i[0])+", "+ str(i[1]))
 
323          # build event pointset information (reset previous trajectory's)
 
324          self.trajevents = {} 
325          for (evname, ev) in eventslist: 
326              evpt = Evpoints[evname] 
327              if evpt == []: 
328                  self.trajevents[evname] = None 
329              else: 
330                  evpt = transpose(array(evpt)) 
331                  self.trajevents[evname] = Pointset({
 
332                                  'coordnames': sortedDictKeys(tempvars),
 
333                                  'indepvarname': 't',
 
334                                  'coordarray': evpt,
 
335                                  'indepvararray': Evtimes[evname],
 
336                                  'indepvartype': self.indepvartype}) 
337          if not self.defined: 
338              self._register(self.variables) 
339          self.validateSpec() 
340          self.defined = True 
341          return Trajectory(trajname, tempvars.values(),
 
342                            abseps=self._abseps, globalt0=self.globalt0,
 
343                            checklevel=self.checklevel,
 
344                            FScompatibleNames=self._FScompatibleNames,
 
345                            FScompatibleNamesInv=self._FScompatibleNamesInv,
 
346                            events=self.trajevents,
 
347                            modelNames=self.name,
 
348                            modelEventStructs=self.eventstruct)

349  
 
350  
 


351 -    def AuxVars(self, t, xdict, pdict=None, asarray=True):


352          """asarray is an unused, dummy argument for compatibility with
 
353          Model.AuxVars""" 
354          x = sortedDictValues(filteredDict(xdict, self.funcspec.vars)) 
355          if pdict is None: 
356              pdict = self.pars 
357          p = sortedDictValues(pdict) 
358          i = _pollInputs(sortedDictValues(self.inputs), t, self.checklevel) 
359          return apply(getattr(self, self.funcspec.auxspec[1]), [t, x, p+i])

360  
 


361 -    def haveJacobian_pars(self):


362          """Report whether generator has an explicit user-specified Jacobian
 
363          with respect to pars associated with it.""" 
364          return 'Jacobian_pars' in self.funcspec.auxfns

365  
 


366 -    def haveJacobian(self):


367          """Report whether generator has an explicit user-specified Jacobian
 
368          associated with it.""" 
369          return 'Jacobian' in self.funcspec.auxfns

370  
 
371  
 


372 -    def set(self, **kw):


373          """Set ExplicitFnGen parameters""" 
374          if remain(kw.keys(), self._validKeys) != []: 
375              raise KeyError("Invalid keys in argument") 
376          if 'globalt0' in kw: 
377              # pass up to generic treatment for this
 
378              ctsGen.set(self, globalt0=kw['globalt0']) 
379          if 'checklevel' in kw: 
380              # pass up to generic treatment for this
 
381              ctsGen.set(self, checklevel=kw['checklevel']) 
382          if 'abseps' in kw: 
383              # pass up to generic treatment for this
 
384              ctsGen.set(self, abseps=kw['abseps']) 
385          # optional keys for this call are
 
386          #   ['pars', 'tdomain', 'xdomain', 'pdomain']
 
387          if 'xdomain' in kw: 
388              for k_temp, v in kw['xdomain'].iteritems(): 
389                  k = self._FScompatibleNames(k_temp) 
390                  if k in self.funcspec.vars+self.funcspec.auxvars: 
391                      if isinstance(v, _seq_types): 
392                          assert len(v) == 2, \
 
393                                 "Invalid size of domain specification for "+k 
394                          if v[0] >= v[1]: 
395                              raise PyDSTool_ValueError('xdomain values must be'
 
396                                                        'in order of increasing '
 
397                                                        'size') 
398                      elif isinstance(v, _num_types): 
399                          pass 
400                      else: 
401                          raise PyDSTool_TypeError('Invalid type for xdomain spec'
 
402                                                   ' '+k) 
403                      self.xdomain[k] = v 
404                  else: 
405                      raise ValueError('Illegal variable name') 
406                  try: 
407                      self.variables[k].depdomain.set(v) 
408                  except TypeError: 
409                      raise TypeError('xdomain must be a dictionary of variable'
 
410                                        ' names -> valid interval 2-tuples or '
 
411                                        'singletons') 
412                  for ev in self.eventstruct.events.values(): 
413                      ev.xdomain[k] = v 
414          if 'pdomain' in kw: 
415              for k_temp, v in kw['pdomain'].iteritems(): 
416                  k = self._FScompatibleNames(k_temp) 
417                  if k in self.funcspec.pars: 
418                      if isinstance(v, _seq_types): 
419                          assert len(v) == 2, \
 
420                                 "Invalid size of domain specification for "+k 
421                          if v[0] >= v[1]: 
422                              raise PyDSTool_ValueError('pdomain values must be'
 
423                                                        'in order of increasing '
 
424                                                        'size') 
425                          else: 
426                              self.pdomain[k] = copy(v) 
427                      elif isinstance(v, _num_types): 
428                          self.pdomain[k] = [v, v] 
429                      else: 
430                          raise PyDSTool_TypeError('Invalid type for pdomain spec'
 
431                                                   ' '+k) 
432                  else: 
433                      raise ValueError('Illegal parameter name') 
434                  try: 
435                      self.parameterDomains[k].depdomain.set(v) 
436                  except TypeError: 
437                      raise TypeError('pdomain must be a dictionary of parameter'
 
438                                        ' names -> valid interval 2-tuples or '
 
439                                        'singletons') 
440                  for ev in self.eventstruct.events.values(): 
441                      ev.pdomain[k] = v 
442          if 'tdata' in kw: 
443              self.tdata = kw['tdata'] 
444          if 'tdomain' in kw: 
445              self.tdomain = kw['tdomain'] 
446              self.indepvariable.indepdomain.set(self.tdomain) 
447          if self.tdomain[0] > self.tdata[0]: 
448              if self.indepvariable.indepdomain.contains(self.tdata[0]) == uncertain: 
449                  self.diagnostics.warnings.append((W_UNCERTVAL,
 
450                                                    (self.tdata[0],self.tdomain))) 
451              else: 
452                  print 'tdata cannot be specified below smallest '\
 
453                        'value in tdomain\n (possibly due to uncertain bounding).'\
 
454                        ' It has been automatically adjusted from\n ', self.tdata[0], \
 
455                        'to', self.tdomain[0], '(difference of', \
 
456                        self.tdomain[0]-self.tdata[0], ')' 
457              self.tdata[0] = self.tdomain[0] 
458          if self.tdomain[1] < self.tdata[1]: 
459              if self.indepvariable.indepdomain.contains(self.tdata[1]) == uncertain: 
460                  self.diagnostics.warnings.append((W_UNCERTVAL,
 
461                                                    (self.tdata[1],self.tdomain))) 
462              else: 
463                  print 'tdata cannot be specified above largest '\
 
464                        'value in tdomain\n (possibly due to uncertain bounding).'\
 
465                        ' It has been automatically adjusted from\n ', \
 
466                        self.tdomain[1], 'to', \
 
467                        self.tdomain[1], '(difference of', \
 
468                        self.tdata[1]-self.tdomain[1], ')' 
469              self.tdata[1] = self.tdomain[1] 
470          self.indepvariable.depdomain.set(self.tdata) 
471          if 'ics' in kw: 
472              for k_temp, v in kw['ics'].iteritems(): 
473                  k = self._FScompatibleNames(k_temp) 
474                  if k in self.funcspec.vars+self.funcspec.auxvars: 
475                      self._xdatadict[k] = ensurefloat(v) 
476                  else: 
477                      raise ValueError('Illegal variable name') 
478              self.initialconditions.update(self._xdatadict) 
479          if 'pars' in kw: 
480              if not self.pars: 
481                  raise ValueError('No pars were declared for this object'
 
482                                     ' at initialization.') 
483              for k_temp, v in kw['pars'].iteritems(): 
484                  k = self._FScompatibleNames(k_temp) 
485                  if k in self.pars: 
486                      cval = self.parameterDomains[k].contains(v) 
487                      if self.checklevel < 3: 
488                          if cval is not notcontained: 
489                              self.pars[k] = ensurefloat(v) 
490                              if cval is uncertain and self.checklevel == 2: 
491                                  print 'Warning: Parameter value at bound' 
492                          else: 
493                              raise PyDSTool_ValueError('Parameter value out of '
 
494                                                        'bounds') 
495                      else: 
496                          if cval is contained: 
497                              self.pars[k] = ensurefloat(v) 
498                          elif cval is uncertain: 
499                              raise PyDSTool_UncertainValueError('Parameter value'
 
500                                                                 ' at bound') 
501                          else: 
502                              raise PyDSTool_ValueError('Parameter value out of'
 
503                                                        ' bounds') 
504                  else: 
505                      raise PyDSTool_AttributeError('Illegal parameter name')

506  
 
507  
 


508 -    def validateSpec(self):


509          ctsGen.validateSpec(self) 
510          try: 
511              for v in self.variables.values(): 
512                  assert isinstance(v, Variable) 
513              assert not self.inputs 
514          except AssertionError: 
515              print 'Invalid system specification' 
516              raise

517  
 
518  
 


519 -    def __del__(self):


520          ctsGen.__del__(self)

521  
 
522  
 
523  
 
524  # Register this Generator with the database
 
525  
 
526  symbolMapDict = {} 
527  # in future, provide appropriate mappings for libraries math,
 
528  # random, etc. (for now it's left to FuncSpec)
 
529  theGenSpecHelper.add(ExplicitFnGen, symbolMapDict, 'python', 'ExpFuncSpec') 
530
```

  


| Home | Trees | Indices | Help | | PyDSTool | | --- | |
| --- | --- | --- | --- | --- | --- |

|  |  |
| --- | --- |
| Generated by Epydoc 3.0.1 on Fri May 4 15:24:23 2012 | http://epydoc.sourceforge.net |
