# Supplementary material for: Hybrid Models and Biological Model Reduction with PyDSTool
Source: PLoS Comput Biol. 2012 Aug 9;8(8):e1002628. doi: 10.1371/journal.pcbi.1002628 (PMC3415397; doi:10.1371/journal.pcbi.1002628)
Supplement: Text S4 — Complete source code for the PyDSTool package (version 0.88.120504). Includes API documentation and help files linking to web pages. This file is identical to the current public release on Sourceforge.net. (ZIP) [file pcbi.1002628.s004.zip › PyDSTool/html/PyDSTool.Generator.ExplicitFnGen'.ExplicitFnGen-class.html]

xml version="1.0" encoding="ascii"?


PyDSTool.Generator.ExplicitFnGen'.ExplicitFnGen


| Home | Trees | Indices | Help | | PyDSTool | | --- | |
| --- | --- | --- | --- | --- | --- |

|  |  |  |  |
| --- | --- | --- | --- |
| Package PyDSTool :: Package Generator :: Module ExplicitFnGen' :: Class ExplicitFnGen | |  | | --- | | [hide private] | | [frames] | no frames] | |

# Class ExplicitFnGen

source code

```
           object --+        
                    |        
baseclasses.Generator --+    
                        |    
       baseclasses.ctsGen --+
                            |
                           ExplicitFnGen
```

---

Explicit functional form specifying a trajectory.

E.g. for an external input. This class allows parametric forms of the
function, but with no dependence on x or its own external inputs.


|  |  |  |  |
| --- | --- | --- | --- |
| |  |  | | --- | --- | | Instance Methods | [hide private] | | |
|  | |  |  | | --- | --- | | AuxVars(self, t, xdict, pdict=None, asarray=True)  asarray is an unused, dummy argument for compatibility with Model.AuxVars | source code | |
|  | |  |  | | --- | --- | | \_\_del\_\_(self) | source code | |
|  | |  |  | | --- | --- | | \_\_init\_\_(self, kw)  x.\_\_init\_\_(...) initializes x; see x.\_\_class\_\_.\_\_doc\_\_ for signature | source code | |
|  | |  |  | | --- | --- | | addMethods(self, usePsyco=False)  Add Python-specific functions to this object's methods, accelerating them with psyco, if it is available. | source code | |
|  | |  |  | | --- | --- | | compute(self, trajname, ics=None)  Attach specification functions to callable interface. | source code | |
|  | |  |  | | --- | --- | | haveJacobian(self)  Report whether generator has an explicit user-specified Jacobian associated with it. | source code | |
|  | |  |  | | --- | --- | | haveJacobian\_pars(self)  Report whether generator has an explicit user-specified Jacobian with respect to pars associated with it. | source code | |
|  | |  |  | | --- | --- | | set(self, \*\*kw)  Set ExplicitFnGen parameters | source code | |
|  | |  |  | | --- | --- | | validateSpec(self) | source code | |
| **Inherited from `baseclasses.Generator`**: `__copy__`, `__deepcopy__`, `__getstate__`, `__repr__`, `__setstate__`, `__str__`, `addEvtPars`, `checkArgs`, `contains`, `get`, `getEventTimes`, `getEvents`, `info`, `query`, `resetEventTimes`, `resetEvents`, `setEventICs`, `showAuxFnSpec`, `showAuxSpec`, `showEventSpec`, `showSpec`  **Inherited from `baseclasses.Generator`** (private): `_addEvents`, `_auxfn_getindex`, `_auxfn_globalindepvar`, `_auxfn_heav`, `_auxfn_if`, `_auxfn_initcond`, `_generate_ixmaps`, `_infostr`, `_kw_process_algparams`, `_kw_process_allvars`, `_kw_process_dispatch`, `_kw_process_events`, `_kw_process_fnspecs`, `_kw_process_ics`, `_kw_process_ignorespecial`, `_kw_process_inputs`, `_kw_process_pars`, `_kw_process_pdomain`, `_kw_process_reuseterms`, `_kw_process_system`, `_kw_process_target`, `_kw_process_tdata`, `_kw_process_tdomain`, `_kw_process_tstep`, `_kw_process_ttype`, `_kw_process_varspecs`, `_kw_process_vfcodeinserts`, `_kw_process_xdomain`, `_kw_process_xtype`, `_makeBoundsEvents`, `_register`, `_set_for_hybrid_DS`  **Inherited from `object`**: `__delattr__`, `__getattribute__`, `__hash__`, `__new__`, `__reduce__`, `__reduce_ex__`, `__setattr__` | |


|  |  |  |  |
| --- | --- | --- | --- |
| |  |  | | --- | --- | | Class Variables | [hide private] | | |
|  | \_needKeys = `['name', 'varspecs']` |
|  | \_optionalKeys = `['globalt0', 'checklevel', 'model', 'abseps', ...` |
|  | \_validKeys = `['globalt0', 'xdomain', 'tdata', 'tdomain', 'ics'...` |
| **Inherited from `baseclasses.Generator`** (private): `_querykeys` | |


|  |  |  |  |
| --- | --- | --- | --- |
| |  |  | | --- | --- | | Properties | [hide private] | | |
| **Inherited from `object`**: `__class__` | |


|  |  |  |  |
| --- | --- | --- | --- |
| |  |  | | --- | --- | | Method Details | [hide private] | | |

|  |  |  |
| --- | --- | --- |
| |  |  | | --- | --- | | \_\_del\_\_(self)  *(Destructor)* | source code |   Overrides: baseclasses.Generator.\_\_del\_\_ |

|  |  |  |
| --- | --- | --- |
| |  |  | | --- | --- | | \_\_init\_\_(self, kw)  *(Constructor)* | source code |   x.\_\_init\_\_(...) initializes x; see x.\_\_class\_\_.\_\_doc\_\_ for signature  Overrides: object.\_\_init\_\_ *(inherited documentation)* |

|  |  |  |
| --- | --- | --- |
| |  |  | | --- | --- | | haveJacobian(self) | source code |   Report whether generator has an explicit user-specified Jacobian associated with it.  Overrides: baseclasses.Generator.haveJacobian |

|  |  |  |
| --- | --- | --- |
| |  |  | | --- | --- | | haveJacobian\_pars(self) | source code |   Report whether generator has an explicit user-specified Jacobian with respect to pars associated with it.  Overrides: baseclasses.Generator.haveJacobian\_pars |

|  |  |  |
| --- | --- | --- |
| |  |  | | --- | --- | | set(self, \*\*kw) | source code |   Set ExplicitFnGen parameters  Overrides: baseclasses.Generator.set |

|  |  |  |
| --- | --- | --- |
| |  |  | | --- | --- | | validateSpec(self) | source code |   Overrides: baseclasses.Generator.validateSpec |

  


|  |  |  |  |
| --- | --- | --- | --- |
| |  |  | | --- | --- | | Class Variable Details | [hide private] | | |

|  |  |
| --- | --- |
| \_optionalKeys   Value:  |  | | --- | | ``` ['globalt0',  'checklevel',  'model',  'abseps',  'eventPars',  'FScompatibleNames',  'FScompatibleNamesInv',  'tdomain', ... ``` | |

|  |  |
| --- | --- |
| \_validKeys   Value:  |  | | --- | | ``` ['globalt0',  'xdomain',  'tdata',  'tdomain',  'ics',  'pars',  'checklevel',  'pdomain', ... ``` | |

  


| Home | Trees | Indices | Help | | PyDSTool | | --- | |
| --- | --- | --- | --- | --- | --- |

|  |  |
| --- | --- |
| Generated by Epydoc 3.0.1 on Fri May 4 15:24:06 2012 | http://epydoc.sourceforge.net |
