# Supplementary material for: Hybrid Models and Biological Model Reduction with PyDSTool
Source: PLoS Comput Biol. 2012 Aug 9;8(8):e1002628. doi: 10.1371/journal.pcbi.1002628 (PMC3415397; doi:10.1371/journal.pcbi.1002628)
Supplement: Text S4 — Complete source code for the PyDSTool package (version 0.88.120504). Includes API documentation and help files linking to web pages. This file is identical to the current public release on Sourceforge.net. (ZIP) [file pcbi.1002628.s004.zip › PyDSTool/html/PyDSTool.Generator.ExtrapolateTable'-pysrc.html]

xml version="1.0" encoding="ascii"?


PyDSTool.Generator.ExtrapolateTable'


| Home | Trees | Indices | Help | | PyDSTool | | --- | |
| --- | --- | --- | --- | --- | --- |

|  |  |  |  |
| --- | --- | --- | --- |
| Package PyDSTool :: Package Generator :: Module ExtrapolateTable' | |  | | --- | | [hide private] | | [frames] | no frames] | |

# Source Code for Module PyDSTool.Generator.ExtrapolateTable'

```
  1  # Interpolated lookup table with extrapolated end points 
  2  from __future__ import division 
  3   
  4  from allimports import * 
  5  from baseclasses import ctsGen, theGenSpecHelper 
  6  from PyDSTool.utils import * 
  7  from PyDSTool.common import * 
  8   
  9  # Other imports 
 10  from numpy import Inf, NaN, isfinite, sometrue, alltrue, float64, array 
 11  from scipy import polyfit 
 12  import math, random 
 13  from copy import copy, deepcopy 
 14   
 15  # ----------------------------------------------------------------------------- 
 16   


17 -class ExtrapolateTable(ctsGen):


18      """Data lookup table with piecewise linear or piecewise constant interpolation.""" 
 19   


20 -    def __init__(self, kw):


21          try: 
 22              self.tdata = kw['tdata'] 
 23              self._xdatadict = {} 
 24              for k, v in dict(kw['ics']).iteritems(): 
 25                  self._xdatadict[str(k)] = v 
 26              self.foundKeys = 2 
 27              # check for other, invalid keys (but currently just ignored) 
 28          except KeyError: 
 29              raise PyDSTool_KeyError('Keywords missing in argument') 
 30   
 31          if 'npts' in kw: 
 32              self.npts = kw['npts'] 
 33              if self.npts is None: 
 34                  self.npts = -1 
 35              self.foundKeys +=1 
 36          else: 
 37              self.npts = -1 
 38   
 39          tlen = len(self.tdata) 
 40          if self.npts > 1: 
 41              lidx = min(tlen,self.npts) 
 42              if lidx <= 1 or lidx == tlen: 
 43                  tl = self.tdata 
 44                  th = self.tdata 
 45              else: 
 46                  tl = self.tdata[0:lidx] 
 47                  th = self.tdata[-lidx-1:-1] 
 48          else: 
 49              tl = self.tdata 
 50              th = self.tdata 
 51              lidx = -1 
 52          self._lapx = {} 
 53          self._hapx = {} 
 54   
 55          for x in self._xdatadict: 
 56              if lidx > 1: 
 57                  self._lapx[x] = polyfit(tl, self._xdatadict[x][0:lidx], 1) 
 58                  self._hapx[x] = polyfit(th, self._xdatadict[x][-lidx-1:-1], 1) 
 59              else: 
 60                  self._lapx[x] = polyfit(tl, self._xdatadict[x], 1) 
 61                  self._hapx[x] = self._lapx[x] 
 62   
 63          if 'lotime' in kw: 
 64              assert kw['lotime'] < self.tdata[0] 
 65              loT = kw['lotime'] 
 66              for x in self._xdatadict: 
 67                  if tlen == 1: 
 68                      newloval = self._xdatadict[x][0] 
 69                  else: 
 70                      newloval = self._lapx[x][0]*loT + self._xdatadict[x][0] 
 71                      #newloval = (self._xdatadict[x][1] - self._xdatadict[x][0])/(self.tdata[1] - self.tdata[0])*loT 
 72                      #+ self._xdatadict[x][0] 
 73                  if isinstance(self._xdatadict[x], list): 
 74                      self._xdatadict[x] = [newloval] + self._xdatadict[x] 
 75                  else: 
 76                      temp = self._xdatadict[x].tolist() 
 77                      self._xdatadict[x] = array([newloval] + temp) 
 78              self.foundKeys += 1 
 79   
 80          if 'hitime' in kw: 
 81              assert kw['hitime'] > self.tdata[-1] 
 82              hiT = kw['hitime'] 
 83              for x in self._xdatadict: 
 84                  if tlen == 1: 
 85                      newhival = self._xdatadict[x][-1] 
 86                  else: 
 87                      newhival = self._hapx[x][0]*hiT + self._xdatadict[x][-1] 
 88                      #newhival = (self._xdatadict[x][-2] - self._xdatadict[x][-1])/(self.tdata[-2] - self.tdata[-1])*hiT 
 89                      #+ self._xdatadict[x][-1] 
 90                  if isinstance(self._xdatadict[x], list): 
 91                      self._xdatadict[x] = self._xdatadict[x] + [newhival] 
 92                  else: 
 93                      temp = self._xdatadict[x].tolist() 
 94                      self._xdatadict[x] = array(temp + [newhival]) 
 95              self.foundKeys += 1 
 96   
 97          if 'lotime' in kw: 
 98              if isinstance(self.tdata, list): 
 99                  self.tdata = [kw['lotime']] + self.tdata 
100              else: 
101                  temp = self.tdata.tolist() 
102                  self.tdata = [kw['lotime']] + temp 
103   
104          if 'hitime' in kw: 
105              if isinstance(self.tdata, list): 
106                  self.tdata = self.tdata + [kw['hitime']] 
107              else: 
108                  temp = self.tdata.tolist() 
109                  self.tdata = temp + [kw['hitime']] 
110   
111          self.tdata = array(self.tdata) 
112   
113          self.tdomain = extent(self.tdata) 
114          self.xdomain = {} 
115          for x in self._xdatadict: 
116              self.xdomain[x] = extent(self._xdatadict[x]) 
117          ctsGen.__init__(self, kw) 
118          self.funcspec = {} 
119          if 'vars' in kw: 
120              raise PyDSTool_KeyError('vars option invalid for extrapolated table class') 
121          if 'auxvars' in kw: 
122              raise PyDSTool_KeyError('auxvars option invalid for extrapolated table class') 
123          if 'tdomain' in kw: 
124              raise PyDSTool_KeyError('tdomain option invalid for extrapolated table class') 
125          if 'xdomain' in kw: 
126              raise PyDSTool_KeyError('xdomain option invalid for extrapolated table class') 
127          if 'pdomain' in kw: 
128              raise PyDSTool_KeyError('pdomain option invalid for extrapolated table class') 
129          if 'ttype' in kw: 
130              raise PyDSTool_KeyError('ttype option invalid for extrapolated table class') 
131          # hack to allow xtype to run 
132          kw['varspecs'] = {}.fromkeys(self._xdatadict, '') 
133          self._kw_process_dispatch(['varspecs', 'xtype'], kw) 
134          del kw['varspecs'] 
135          self.foundKeys -= 1 
136          if 'method' in kw: 
137              if kw['method']=='linear': 
138                  interp=interp1d 
139              elif kw['method']=='constant': 
140                  interp=interp0d 
141              else: 
142                  raise ValueError("Invalid interpolation method") 
143              self.foundKeys += 1 
144          else: 
145              # default to piecewise linear interpolation 
146              interp=interp1d 
147          for x in self._xdatadict: 
148              self.funcspec[x] = Pointset({'coordarray': self._xdatadict[x], 
149                                           'coordtype': float64, 
150                                           'indepvararray': self.tdata, 
151                                           'indepvartype': float64, 
152                                           'indepvarname': 't', 
153                                           'coordnames': x}) 
154          self._needKeys.extend(['tdata', 'ics']) 
155          self._optionalKeys.extend(['method','lotime','hitime','npts']) 
156          self.indepvartype = float 
157          self.checkArgs(kw) 
158          self.indepvariable = Variable(listid, Interval('t_domain', 
159                                                         self.indepvartype, 
160                                                self.tdomain, self._abseps), 
161                               Interval('t', self.indepvartype, 
162                                        extent(self.tdata), 
163                                        self._abseps), 't') 
164          self._register(self.indepvariable) 
165          for x in self._xdatadict: 
166              self.variables[x] = Variable(interp(copy(self.tdata), 
167                                            self.funcspec[x].toarray()), 't', 
168                                    Interval(x, self.xtype[x], self.xdomain[x], 
169                                             self._abseps), x) 
170          self._register(self.variables) 
171          self.dimension = len(self._xdatadict) 
172          self.validateSpec() 
173          self.defined = True

174   
175   


176 -    def compute(self, trajname):


177          return Trajectory(trajname, [copy(v) for v in self.variables.values()], 
178                            abseps=self._abseps, globalt0=self.globalt0, 
179                            checklevel=self.checklevel, 
180                            FScompatibleNames=self._FScompatibleNames, 
181                            FScompatibleNamesInv=self._FScompatibleNamesInv, 
182                            modelNames=self.name, 
183                            modelEventStructs=self.eventstruct)

184   


185 -    def set(self, **kw):


186          if 'abseps' in kw: 
187              # pass up to generic treatment for this 
188              ctsGen.set(self, abseps=kw['abseps']) 
189              for x in self._xdatadict: 
190                  self.variables[x] = Variable(interp(copy(self.tdata), 
191                                            self.funcspec[x].toarray()), 't', 
192                                    Interval(x, self.xtype[x], self.xdomain[x], 
193                                             self._abseps), x) 
194          if 'checklevel' in kw: 
195              # pass up to generic treatment for this 
196              ctsGen.set(self, checklevel=kw['checklevel']) 
197          if 'globalt0' in kw: 
198              # pass up to generic treatment for this 
199              ctsGen.set(self, globalt0=kw['globalt0'])

200   


201 -    def validateSpec(self):


202          ctsGen.validateSpec(self) 
203          try: 
204              assert isoutputcts(self.indepvariable) 
205              for v in self.variables.values(): 
206                  assert isinstance(v, Variable) 
207              assert not self.inputs 
208          except AssertionError: 
209              print 'Invalid system specification' 
210              raise

211   
212   


213 -    def __del__(self):


214          ctsGen.__del__(self)

215   
216   
217   
218   
219  # Register this Generator with the database 
220   
221  symbolMapDict = {} 
222  # in future, provide appropriate mappings for libraries math, 
223  # random, etc. (for now it's left to FuncSpec) 
224  theGenSpecHelper.add(ExtrapolateTable, symbolMapDict, 'python') 
225
```

  


| Home | Trees | Indices | Help | | PyDSTool | | --- | |
| --- | --- | --- | --- | --- | --- |

|  |  |
| --- | --- |
| Generated by Epydoc 3.0.1 on Fri May 4 15:24:16 2012 | http://epydoc.sourceforge.net |
