# Supplementary material for: Hybrid Models and Biological Model Reduction with PyDSTool
Source: PLoS Comput Biol. 2012 Aug 9;8(8):e1002628. doi: 10.1371/journal.pcbi.1002628 (PMC3415397; doi:10.1371/journal.pcbi.1002628)
Supplement: Text S4 — Complete source code for the PyDSTool package (version 0.88.120504). Includes API documentation and help files linking to web pages. This file is identical to the current public release on Sourceforge.net. (ZIP) [file pcbi.1002628.s004.zip › PyDSTool/html/PyDSTool.Generator.ImplicitFnGen'-pysrc.html]

xml version="1.0" encoding="ascii"?


PyDSTool.Generator.ImplicitFnGen'


| Home | Trees | Indices | Help | | PyDSTool | | --- | |
| --- | --- | --- | --- | --- | --- |

|  |  |  |  |
| --- | --- | --- | --- |
| Package PyDSTool :: Package Generator :: Module ImplicitFnGen' | |  | | --- | | [hide private] | | [frames] | no frames] | |

# Source Code for Module PyDSTool.Generator.ImplicitFnGen'

```
  1  # Implicit function generator
 
  2  from __future__ import division 
  3  
 
  4  from allimports import * 
  5  from baseclasses import ctsGen, theGenSpecHelper 
  6  from PyDSTool.utils import * 
  7  from PyDSTool.common import * 
  8  from PyDSTool.Interval import uncertain 
  9  
 
 10  # Other imports
 
 11  from numpy import Inf, NaN, isfinite, sometrue, alltrue, array, \
 
 12       transpose, shape 
 13  import math, random 
 14  from copy import copy, deepcopy 
 15  try: 
 16      # use pscyo JIT byte-compiler optimization, if available
 
 17      import psyco 
 18      HAVE_PSYCO = True 
 19  except ImportError: 
 20      HAVE_PSYCO = False 
 21  
 
 22  
 


23 -class ImplicitFnGen(ctsGen):


24      """Implicitly defined functional-form trajectory generator.
 
 25      """ 
 26      _validKeys = ['globalt0', 'xdomain', 'tdata', 'tdomain', 'checklevel',
 
 27                     'name', 'ics', 'pars', 'algparams', 'pdomain', 'abseps'] 
 28      _needKeys = ctsGen._needKeys + ['varspecs', 'ics'] 
 29      _optionalKeys = ctsGen._optionalKeys + ['tdomain', 'pars', 'pdomain', 'xdomain',
 
 30                                    'xtype', 'auxvars', 'vars', 'events',
 
 31                                    'algparams', 'fnspecs', 'tdata'] 
 32  
 


33 -    def __init__(self, kw):


34          ctsGen.__init__(self, kw) 
 35          dispatch_list = ['varspecs', 'tdomain', 'tdata', 'xtype', 'xdomain',
 
 36                           'ics', 'allvars', 'pars', 'pdomain', 'fnspecs',
 
 37                           'algparams', 'target'] 
 38          if 'inputs' in kw: 
 39              raise PyDSTool_KeyError("inputs option invalid for ImplicitFnGen "
 
 40                                      "class") 
 41          self.funcspec = ImpFuncSpec(self._kw_process_dispatch(dispatch_list, kw)) 
 42          self.indepvartype = float 
 43          for s in self.funcspec.spec[0]: 
 44              if s.find('x[') > -1: 
 45                  raise ValueError('Variable values cannot depend on '
 
 46                              'other variables in implicit function specs -- '
 
 47                              'in function:\n'+s) 
 48          if 'solvemethod' in self.algparams: 
 49              if self.algparams['solvemethod'] not in _implicitSolveMethods: 
 50                  raise PyDSTool_ValueError('Invalid implicit solver type') 
 51          # Holder and interface for events
 
 52          self.eventstruct = EventStruct() 
 53          if 'events' in kw: 
 54              raise PyDSTool_ValueError('ImplicitFnGen does not presently' \
 
 55                                        ' support events') 
 56  ##            self._addEvents(kw['events'])
 
 57  ##            assert self.eventstruct.getLowLevelEvents() == [], \
 
 58  ##               "Can only pass high level events to ImplicitFnGen objects"
 
 59  ##            assert self.eventstruct.query(['highlevel', 'varlinked']) == [], \
 
 60  ##               "Only non-variable linked events are valid for this class"
 
 61  ##            self.foundKeys += 1
 
 62          self.checkArgs(kw) 
 63          self.newTempVars() 
 64          self._generate_ixmaps()

 65  
 
 66  
 


67 -    def newTempVars(self):


68          self.indepvariable = Variable(listid, Interval('t_domain',
 
 69                                                         self.indepvartype,
 
 70                                          self.tdomain, self._abseps),
 
 71                                        Interval('t', self.indepvartype,
 
 72                                                 self.tdata, self._abseps),
 
 73                                        't') 
 74          if not self.defined: 
 75              self._register(self.indepvariable) 
 76          for x in self.funcspec.vars + self.funcspec.auxvars: 
 77              try: 
 78                  xinterval=Interval(x, self.xtype[x], self.xdomain[x], self._abseps) 
 79              except KeyError, e: 
 80                  raise PyDSTool_KeyError('Mismatch between declared variables'
 
 81                                   ' and xspecs: ' + str(e)) 
 82              # placeholder variable so that this class can be
 
 83              # copied before it is defined (listid function is a dummy)
 
 84              self.variables[x] = Variable(None, self.indepvariable.depdomain,
 
 85                                           xinterval, x)

 86  
 
 87  
 


88 -    def compute(self, trajname, ics=None):


89          """Attach specification functions to callable interface.""" 
 90  
 
 91          assert self.funcspec.targetlang == 'python', \
 
 92                 ('Wrong target language for functional specification. '
 
 93                  'Python needed for this class') 
 94          assert isinstance(self.funcspec, ImpFuncSpec), ('ImplicitFnGen'
 
 95                                      ' requires ImpFuncSpec type to proceed') 
 96          # repeat this check (made in __init__) in case events were added since
 
 97          assert self.eventstruct.getLowLevelEvents() == [], \
 
 98                 "Can only pass high level events to ImplicitFnGen objects" 
 99          assert self.eventstruct.query(['highlevel', 'varlinked']) == [], \
 
100                 "Only non-variable linked events are valid for this class" 
101          if ics is not None: 
102              self.set(ics=ics) 
103          # set some defaults for implicit function
 
104          if 'solvemethod' in self.algparams: 
105              if self.algparams['solvemethod'] not in _implicitSolveMethods: 
106                  raise PyDSTool_ValueError('Invalid implicit solver type') 
107          else: 
108              self.algparams['solvemethod'] = 'fsolve' 
109          if self.algparams['solvemethod'] in _1DimplicitSolveMethods and \
 
110             self.dimension > 1: 
111              raise PyDSTool_TypeError('Inappropriate implicit solver for '
 
112                                       'non-scalar system') 
113          if 'atol' not in self.algparams: 
114              self.algparams['atol'] = 1e-8 
115          if 'maxnumiter' not in self.algparams: 
116              self.algparams['maxnumiter'] = 100 
117          if self.defined: 
118              # reset variables
 
119              self.newTempVars() 
120          self.setEventICs(self.initialconditions, self.globalt0) 
121          tempfs = deepcopy(self.funcspec) 
122          tempvars = copyVarDict(self.variables) 
123          # make unique fn for this trajectory
 
124          tempspec = makeUniqueFn(copy(tempfs.spec[0]), 7, self.name) 
125          tempfs.spec = tempspec 
126          # test supplied code
 
127          try: 
128              exec tempspec[0] in globals() 
129          except: 
130              print 'Error in supplied functional specification code' 
131              raise 
132          # set up implicit function: utils.makeImplicitFunction gets
 
133          # called finally in Variable.addMethods() method.
 
134          tempfs.algparams.update(self.algparams) 
135          if self.haveJacobian(): 
136              tempfs.algparams['jac'] = self.funcspec.auxfns['Jacobian'] 
137          else: 
138              tempfs.algparams['jac'] = None 
139          tempfs.algparams['pars'] = sortedDictValues(self.pars) 
140          if self.dimension == 1 and \
 
141             self.algparams['solvemethod'] in _1DimplicitSolveMethods: 
142              tempfs.algparams['x0'] = sortedDictValues(self.initialconditions,
 
143                                                    self.funcspec.vars)[0] 
144          else: 
145              tempfs.algparams['x0'] = sortedDictValues(self.initialconditions,
 
146                                                    self.funcspec.vars) 
147          tempfs.algparams['impfn_name'] = "impfn_" + timestamp(7) 
148  ##        tempfs.algparams['impfn_name'] = "impfn"
 
149          # create wrapper functions around implicit function, for each variable
 
150          for x in self.funcspec.vars: 
151              x_ix = self.funcspec.vars.index(x) 
152              funcname = "_mapspecfn_" + x + "_" + timestamp(7) 
153              funcstr = "def " + funcname + "(self, t):\n\treturn " \
 
154                      + tempfs.algparams['impfn_name'] + "(self, t)" 
155              if self.dimension > 1: 
156                  funcstr += "[" + str(x_ix) + "]\n" 
157              else: 
158                  funcstr += "\n" 
159              # make output fn of each variable the entry in the output from the
 
160              # same implicit function.
 
161              # initial conditions aren't needed beyond algparams['x0']
 
162              tempvars[x].setOutput((funcname,funcstr), tempfs,
 
163                                    self.globalt0, self._var_namemap) 
164          if self.funcspec.auxvars != []: 
165              # make unique fn for this trajectory
 
166              tempauxspec = makeUniqueFn(copy(tempfs.auxspec[0]), 7, self.name) 
167              tempfs.auxspec = tempauxspec 
168          for a in self.funcspec.auxvars: 
169              a_ix = self.funcspec.auxvars.index(a) 
170              funcname = "_mapspecfn_" + a + "_" + timestamp(7) 
171              funcstr = "def " + funcname + "(self, t):\n\treturn " 
172              if len(self.funcspec.auxvars) == 1: 
173                  # we'll only go through this once!
 
174                  funcstr += tempauxspec[1] + "(self, t, [v(t) " \
 
175                        + "for v in self._refvars], " \
 
176                        + repr(sortedDictValues(self.pars)) \
 
177                        + ")[0]\n" 
178              else: 
179                  funcstr += tempauxspec[1] + "(self, t, [v(t) " \
 
180                        + "for v in self._refvars], " \
 
181                        + repr(sortedDictValues(self.pars)) \
 
182                        + ")[" + str(a_ix) + "]\n" 
183              # initial conditions aren't needed beyond algparams['x0']
 
184              tempvars[a].setOutput((funcname, funcstr), tempfs,
 
185                                          self.globalt0, self.funcspec.auxvars,
 
186                                          None,
 
187                                          sortedDictValues(tempvars,
 
188                                                           self.funcspec.vars)) 
189          self.diagnostics.clearWarnings() 
190          self.diagnostics.clearErrors() 
191          if self.eventstruct.getHighLevelEvents(): 
192              raise PyDSTool_ValueError('ImplicitFnGen does not presently' \
 
193                                        ' support events') 
194  ##        # Find any events in tdomain, and adjust tdomain in case they
 
195  ##        # are terminal
 
196  ##        eventslist = self.eventstruct.query(['highlevel', 'active',
 
197  ##                                             'notvarlinked'])
 
198  ##        termevents = self.eventstruct.query(['term'], eventslist)
 
199  ##        if eventslist != []:
 
200  ##            Evts = []
 
201  ##            for evix in xrange(len(eventslist)):
 
202  ##                (evname, ev) = eventslist[evix]
 
203  ##                evsfound = ev.searchForEvents(self.indepvariable.depdomain.get(),
 
204  ##                                              parDict=self.pars,
 
205  ##                                              vars=tempvars,
 
206  ##                                              checklevel=self.checklevel)
 
207  ##                Evts.append([evinfo[0] for evinfo in evsfound])
 
208  ##        if eventslist != []:
 
209  ##            self.eventstruct.resetHighLevelEvents(self.indepvariable.depdomain[0],
 
210  ##                                                  eventslist)
 
211  ##            self.eventstruct.validateEvents(self.funcspec.vars + \
 
212  ##                                            self.funcspec.auxvars + \
 
213  ##                                            ['t'], eventslist)
 
214  ##            termevtimes = {}
 
215  ##            nontermevtimes = {}
 
216  ##            for evix in xrange(len(eventslist)):
 
217  ##                numevs = shape(Evts[evix])[-1]
 
218  ##                if numevs == 0:
 
219  ##                    continue
 
220  ##                if eventslist[evix][1].activeFlag:
 
221  ##                    if numevs > 1:
 
222  ##                        print "Event info:", Evts[evix]
 
223  ##                    assert numevs <= 1, ("Internal error: more than one "
 
224  ##                                     "terminal event of same type found")
 
225  ##                    # For safety, we should assert that this event
 
226  ##                    # also appears in termevents, but we don't
 
227  ##                    evname = eventslist[evix][0]
 
228  ##                    if Evts[evix][0] in termevtimes.keys():
 
229  ##                        # append event name to this warning
 
230  ##                        warning_ix = termevtimes[Evts[evix][0]]
 
231  ##                        self.diagnostics.warnings[warning_ix][1][1].append(evname)
 
232  ##                    else:
 
233  ##                        # make new termevtime entry for the new warning
 
234  ##                        termevtimes[Evts[evix][0]] = len(self.diagnostics.warnings)
 
235  ##                        self.diagnostics.warnings.append((W_TERMEVENT,
 
236  ##                                         (Evts[evix][0],
 
237  ##                                         [eventslist[evix][0]])))
 
238  ##                else:
 
239  ##                    for ev in range(numevs):
 
240  ##                        if Evts[evix][ev] in nontermevtimes.keys():
 
241  ##                            # append event name to this warning
 
242  ##                            warning_ix = nontermevtimes[Evts[evix][ev]]
 
243  ##                            self.diagnostics.warnings[warning_ix][1][1].append(evname)
 
244  ##                        else:
 
245  ##                            # make new nontermevtime entry for the new warning
 
246  ##                            nontermevtimes[Evts[evix][ev]] = \
 
247  ##                                                        len(self.diagnostics.warnings)
 
248  ##                            self.diagnostics.warnings.append((W_NONTERMEVENT,
 
249  ##                                             (Evts[evix][ev],
 
250  ##                                              [eventslist[evix][0]])))
 
251  ##        termcount = 0
 
252  ##        earliest_termtime = self.indepvariable.depdomain[1]
 
253  ##        for (w,i) in self.diagnostics.warnings:
 
254  ##            if w == W_TERMEVENT or w == W_TERMSTATEBD:
 
255  ##                termcount += 1
 
256  ##                if i[0] < earliest_termtime:
 
257  ##                    earliest_termtime = i[0]
 
258  ##        # now delete any events found after the earliest terminal event, if any
 
259  ##        if termcount > 0:
 
260  ##            warn_temp = []
 
261  ##            for (w,i) in self.diagnostics.warnings:
 
262  ##                if i[0] <= earliest_termtime:
 
263  ##                    warn_temp.append((w,i))
 
264  ##            self.diagnostics.warnings = warn_temp
 
265  ##        self.indepvariable.depdomain.set([self.indepvariable.depdomain[0],
 
266  ##            earliest_termtime])
 
267  ##        for v in tempvars.values():
 
268  ##            v.indepdomain.set(self.indepvariable.depdomain.get())
 
269  ####                print 'Time interval adjusted according to %s: %s' % \
 
270  ####                      (self._warnmessages[w], str(i[0])+", "+ str(i[1]))
 
271          if not self.defined: 
272              self._register(self.variables) 
273          #self.validateSpec()
 
274          self.defined = True 
275          return Trajectory(trajname, tempvars.values(),
 
276                            abseps=self._abseps, globalt0=self.globalt0,
 
277                            checklevel=self.checklevel,
 
278                            FScompatibleNames=self._FScompatibleNames,
 
279                            FScompatibleNamesInv=self._FScompatibleNamesInv,
 
280                            modelNames=self.name,
 
281                            modelEventStructs=self.eventstruct)

282  
 


283 -    def haveJacobian_pars(self):


284          """Report whether generator has an explicit user-specified Jacobian
 
285          with respect to pars associated with it.""" 
286          return 'Jacobian_pars' in self.funcspec.auxfns

287  
 


288 -    def haveJacobian(self):


289          """Report whether generator has an explicit user-specified Jacobian
 
290          associated with it.""" 
291          return 'Jacobian' in self.funcspec.auxfns

292  
 
293  
 


294 -    def set(self, **kw):


295          """Set ImplicitFnGen parameters""" 
296          if remain(kw.keys(), self._validKeys) != []: 
297              raise KeyError("Invalid keys in argument") 
298          if 'globalt0' in kw: 
299              # pass up to generic treatment for this
 
300              ctsGen.set(self, globalt0=kw['globalt0']) 
301          if 'checklevel' in kw: 
302              # pass up to generic treatment for this
 
303              ctsGen.set(self, checklevel=kw['checklevel']) 
304          if 'abseps' in kw: 
305              # pass up to generic treatment for this
 
306              ctsGen.set(self, abseps=kw['abseps']) 
307          # optional keys for this call are ['pars', 'tdomain', 'xdomain', 'pdomain']
 
308          if 'xdomain' in kw: 
309              for k_temp, v in kw['xdomain'].iteritems(): 
310                  k = self._FScompatibleNames(k_temp) 
311                  if k in self.funcspec.vars+self.funcspec.auxvars: 
312                      if isinstance(v, _seq_types): 
313                          assert len(v) == 2, \
 
314                                 "Invalid size of domain specification for "+k 
315                          if v[0] >= v[1]: 
316                              raise PyDSTool_ValueError('xdomain values must be'
 
317                                                        'in order of increasing '
 
318                                                        'size') 
319                      elif isinstance(v, _num_types): 
320                          pass 
321                      else: 
322                          raise PyDSTool_TypeError('Invalid type for xdomain spec'
 
323                                                   ' '+k) 
324                      self.xdomain[k] = v 
325                  else: 
326                      raise ValueError('Illegal variable name') 
327                  try: 
328                      self.variables[k].depdomain.set(v) 
329                  except TypeError: 
330                      raise TypeError('xdomain must be a dictionary of variable'
 
331                                        ' names -> valid interval 2-tuples or '
 
332                                        'singletons') 
333          if 'tdata' in kw: 
334              self.tdata = kw['tdata'] 
335          if 'tdomain' in kw: 
336              self.tdomain = kw['tdomain'] 
337              self.indepvariable.indepdomain.set(self.tdomain) 
338          if self.tdomain[0] > self.tdata[0]: 
339              if self.indepvariable.indepdomain.contains(self.tdata[0]) == uncertain: 
340                  self.diagnostics.warnings.append((W_UNCERTVAL,
 
341                                                    (self.tdata[0],self.tdomain))) 
342              else: 
343                  print 'tdata cannot be specified below smallest '\
 
344                        'value in tdomain\n (possibly due to uncertain bounding).'\
 
345                        ' It has been automatically adjusted from\n ', self.tdata[0], \
 
346                        'to', self.tdomain[0], '(difference of', \
 
347                        self.tdomain[0]-self.tdata[0], ')' 
348              self.tdata[0] = self.tdomain[0] 
349          if self.tdomain[1] < self.tdata[1]: 
350              if self.indepvariable.indepdomain.contains(self.tdata[1]) == uncertain: 
351                  self.diagnostics.warnings.append((W_UNCERTVAL,
 
352                                                    (self.tdata[1],self.tdomain))) 
353              else: 
354                  print 'tdata cannot be specified above largest '\
 
355                        'value in tdomain\n (possibly due to uncertain bounding).'\
 
356                        ' It has been automatically adjusted from\n ', \
 
357                        self.tdomain[1], 'to', \
 
358                        self.tdomain[1], '(difference of', \
 
359                        self.tdata[1]-self.tdomain[1], ')' 
360              self.tdata[1] = self.tdomain[1] 
361          self.indepvariable.depdomain.set(self.tdata) 
362          if 'pdomain' in kw: 
363              for k_temp, v in kw['pdomain'].iteritems(): 
364                  k = self._FScompatibleNames(k_temp) 
365                  if k in self.funcspec.pars: 
366                      if isinstance(v, _seq_types): 
367                          assert len(v) == 2, \
 
368                                 "Invalid size of domain specification for "+k 
369                          if v[0] >= v[1]: 
370                              raise PyDSTool_ValueError('pdomain values must be'
 
371                                                        'in order of increasing '
 
372                                                        'size') 
373                          else: 
374                              self.pdomain[k] = copy(v) 
375                      elif isinstance(v, _num_types): 
376                          self.pdomain[k] = [v, v] 
377                      else: 
378                          raise PyDSTool_TypeError('Invalid type for pdomain spec'
 
379                                                   ' '+k) 
380                  else: 
381                      raise ValueError('Illegal parameter name') 
382                  try: 
383                      self.parameterDomains[k].depdomain.set(v) 
384                  except TypeError: 
385                      raise TypeError('pdomain must be a dictionary of parameter'
 
386                                        ' names -> valid interval 2-tuples or '
 
387                                        'singletons') 
388          if 'ics' in kw: 
389              for k_temp, v in kw['ics'].iteritems(): 
390                  k = self._FScompatibleNames(k_temp) 
391                  if k in self.funcspec.vars+self.funcspec.auxvars: 
392                      self._xdatadict[k] = ensurefloat(v) 
393                  else: 
394                      raise ValueError('Illegal variable name') 
395              self.initialconditions.update(self._xdatadict) 
396          if 'pars' in kw: 
397              if not self.pars: 
398                  raise ValueError('No pars were declared for this object'
 
399                                     ' at initialization.') 
400              for k_temp, v in kw['pars'].iteritems(): 
401                  k = self._FScompatibleNames(k_temp) 
402                  if k in self.pars: 
403                      cval = self.parameterDomains[k].contains(v) 
404                      if self.checklevel < 3: 
405                          if cval is not notcontained: 
406                              self.pars[k] = ensurefloat(v) 
407                              if cval is uncertain and self.checklevel == 2: 
408                                  print 'Warning: Parameter value at bound' 
409                          else: 
410                              raise PyDSTool_ValueError('Parameter value out of bounds') 
411                      else: 
412                          if cval is contained: 
413                              self.pars[k] = ensurefloat(v) 
414                          elif cval is uncertain: 
415                              raise PyDSTool_UncertainValueError('Parameter value at bound') 
416                          else: 
417                              raise PyDSTool_ValueError('Parameter value out of bounds') 
418                  else: 
419                      raise PyDSTool_AttributeError('Illegal parameter name') 
420          if 'algparams' in kw: 
421              for k, v in kw['algparams'].iteritems(): 
422                  self.algparams[k] = v 
423          if 'solvemethod' in self.algparams: 
424              if self.algparams['solvemethod'] not in _implicitSolveMethods: 
425                  raise PyDSTool_ValueError('Invalid implicit solver type')

426  
 
427  
 


428 -    def validateSpec(self):


429          ctsGen.validateSpec(self) 
430          try: 
431              for v in self.variables.values(): 
432                  assert isinstance(v, Variable) 
433              assert not self.inputs 
434          except AssertionError: 
435              print 'Invalid system specification' 
436              raise

437  
 
438  
 


439 -    def __del__(self):


440          ctsGen.__del__(self)

441  
 
442  
 
443  
 
444  
 
445  # Register this Generator with the database
 
446  
 
447  symbolMapDict = {} 
448  # in future, provide appropriate mappings for libraries math,
 
449  # random, etc. (for now it's left to FuncSpec)
 
450  theGenSpecHelper.add(ImplicitFnGen, symbolMapDict, 'python', 'ImpFuncSpec') 
451
```

  


| Home | Trees | Indices | Help | | PyDSTool | | --- | |
| --- | --- | --- | --- | --- | --- |

|  |  |
| --- | --- |
| Generated by Epydoc 3.0.1 on Fri May 4 15:24:25 2012 | http://epydoc.sourceforge.net |
