# Supplementary material for: Hybrid Models and Biological Model Reduction with PyDSTool
Source: PLoS Comput Biol. 2012 Aug 9;8(8):e1002628. doi: 10.1371/journal.pcbi.1002628 (PMC3415397; doi:10.1371/journal.pcbi.1002628)
Supplement: Text S4 — Complete source code for the PyDSTool package (version 0.88.120504). Includes API documentation and help files linking to web pages. This file is identical to the current public release on Sourceforge.net. (ZIP) [file pcbi.1002628.s004.zip › PyDSTool/html/PyDSTool.Generator.ImplicitFnGen'.ImplicitFnGen-class.html]

xml version="1.0" encoding="ascii"?


PyDSTool.Generator.ImplicitFnGen'.ImplicitFnGen


| Home | Trees | Indices | Help | | PyDSTool | | --- | |
| --- | --- | --- | --- | --- | --- |

|  |  |  |  |
| --- | --- | --- | --- |
| Package PyDSTool :: Package Generator :: Module ImplicitFnGen' :: Class ImplicitFnGen | |  | | --- | | [hide private] | | [frames] | no frames] | |

# Class ImplicitFnGen

source code

```
           object --+        
                    |        
baseclasses.Generator --+    
                        |    
       baseclasses.ctsGen --+
                            |
                           ImplicitFnGen
```

---

Implicitly defined functional-form trajectory generator.


|  |  |  |  |
| --- | --- | --- | --- |
| |  |  | | --- | --- | | Instance Methods | [hide private] | | |
|  | |  |  | | --- | --- | | \_\_del\_\_(self) | source code | |
|  | |  |  | | --- | --- | | \_\_init\_\_(self, kw)  x.\_\_init\_\_(...) initializes x; see x.\_\_class\_\_.\_\_doc\_\_ for signature | source code | |
|  | |  |  | | --- | --- | | compute(self, trajname, ics=None)  Attach specification functions to callable interface. | source code | |
|  | |  |  | | --- | --- | | haveJacobian(self)  Report whether generator has an explicit user-specified Jacobian associated with it. | source code | |
|  | |  |  | | --- | --- | | haveJacobian\_pars(self)  Report whether generator has an explicit user-specified Jacobian with respect to pars associated with it. | source code | |
|  | |  |  | | --- | --- | | newTempVars(self) | source code | |
|  | |  |  | | --- | --- | | set(self, \*\*kw)  Set ImplicitFnGen parameters | source code | |
|  | |  |  | | --- | --- | | validateSpec(self) | source code | |
| **Inherited from `baseclasses.Generator`**: `__copy__`, `__deepcopy__`, `__getstate__`, `__repr__`, `__setstate__`, `__str__`, `addEvtPars`, `checkArgs`, `contains`, `get`, `getEventTimes`, `getEvents`, `info`, `query`, `resetEventTimes`, `resetEvents`, `setEventICs`, `showAuxFnSpec`, `showAuxSpec`, `showEventSpec`, `showSpec`  **Inherited from `baseclasses.Generator`** (private): `_addEvents`, `_auxfn_getindex`, `_auxfn_globalindepvar`, `_auxfn_heav`, `_auxfn_if`, `_auxfn_initcond`, `_generate_ixmaps`, `_infostr`, `_kw_process_algparams`, `_kw_process_allvars`, `_kw_process_dispatch`, `_kw_process_events`, `_kw_process_fnspecs`, `_kw_process_ics`, `_kw_process_ignorespecial`, `_kw_process_inputs`, `_kw_process_pars`, `_kw_process_pdomain`, `_kw_process_reuseterms`, `_kw_process_system`, `_kw_process_target`, `_kw_process_tdata`, `_kw_process_tdomain`, `_kw_process_tstep`, `_kw_process_ttype`, `_kw_process_varspecs`, `_kw_process_vfcodeinserts`, `_kw_process_xdomain`, `_kw_process_xtype`, `_makeBoundsEvents`, `_register`, `_set_for_hybrid_DS`  **Inherited from `object`**: `__delattr__`, `__getattribute__`, `__hash__`, `__new__`, `__reduce__`, `__reduce_ex__`, `__setattr__` | |


|  |  |  |  |
| --- | --- | --- | --- |
| |  |  | | --- | --- | | Class Variables | [hide private] | | |
|  | \_needKeys = `['name', 'varspecs', 'ics']` |
|  | \_optionalKeys = `['globalt0', 'checklevel', 'model', 'abseps', ...` |
|  | \_validKeys = `['globalt0', 'xdomain', 'tdata', 'tdomain', 'chec...` |
| **Inherited from `baseclasses.Generator`** (private): `_querykeys` | |


|  |  |  |  |
| --- | --- | --- | --- |
| |  |  | | --- | --- | | Properties | [hide private] | | |
| **Inherited from `object`**: `__class__` | |


|  |  |  |  |
| --- | --- | --- | --- |
| |  |  | | --- | --- | | Method Details | [hide private] | | |

|  |  |  |
| --- | --- | --- |
| |  |  | | --- | --- | | \_\_del\_\_(self)  *(Destructor)* | source code |   Overrides: baseclasses.Generator.\_\_del\_\_ |

|  |  |  |
| --- | --- | --- |
| |  |  | | --- | --- | | \_\_init\_\_(self, kw)  *(Constructor)* | source code |   x.\_\_init\_\_(...) initializes x; see x.\_\_class\_\_.\_\_doc\_\_ for signature  Overrides: object.\_\_init\_\_ *(inherited documentation)* |

|  |  |  |
| --- | --- | --- |
| |  |  | | --- | --- | | haveJacobian(self) | source code |   Report whether generator has an explicit user-specified Jacobian associated with it.  Overrides: baseclasses.Generator.haveJacobian |

|  |  |  |
| --- | --- | --- |
| |  |  | | --- | --- | | haveJacobian\_pars(self) | source code |   Report whether generator has an explicit user-specified Jacobian with respect to pars associated with it.  Overrides: baseclasses.Generator.haveJacobian\_pars |

|  |  |  |
| --- | --- | --- |
| |  |  | | --- | --- | | set(self, \*\*kw) | source code |   Set ImplicitFnGen parameters  Overrides: baseclasses.Generator.set |

|  |  |  |
| --- | --- | --- |
| |  |  | | --- | --- | | validateSpec(self) | source code |   Overrides: baseclasses.Generator.validateSpec |

  


|  |  |  |  |
| --- | --- | --- | --- |
| |  |  | | --- | --- | | Class Variable Details | [hide private] | | |

|  |  |
| --- | --- |
| \_optionalKeys   Value:  |  | | --- | | ``` ['globalt0',  'checklevel',  'model',  'abseps',  'eventPars',  'FScompatibleNames',  'FScompatibleNamesInv',  'tdomain', ... ``` | |

|  |  |
| --- | --- |
| \_validKeys   Value:  |  | | --- | | ``` ['globalt0',  'xdomain',  'tdata',  'tdomain',  'checklevel',  'name',  'ics',  'pars', ... ``` | |

  


| Home | Trees | Indices | Help | | PyDSTool | | --- | |
| --- | --- | --- | --- | --- | --- |

|  |  |
| --- | --- |
| Generated by Epydoc 3.0.1 on Fri May 4 15:24:06 2012 | http://epydoc.sourceforge.net |
