# Supplementary material for: Hybrid Models and Biological Model Reduction with PyDSTool
Source: PLoS Comput Biol. 2012 Aug 9;8(8):e1002628. doi: 10.1371/journal.pcbi.1002628 (PMC3415397; doi:10.1371/journal.pcbi.1002628)
Supplement: Text S4 — Complete source code for the PyDSTool package (version 0.88.120504). Includes API documentation and help files linking to web pages. This file is identical to the current public release on Sourceforge.net. (ZIP) [file pcbi.1002628.s004.zip › PyDSTool/html/PyDSTool.Generator.InterpolateTable'-pysrc.html]

xml version="1.0" encoding="ascii"?


PyDSTool.Generator.InterpolateTable'


| Home | Trees | Indices | Help | | PyDSTool | | --- | |
| --- | --- | --- | --- | --- | --- |

|  |  |  |  |
| --- | --- | --- | --- |
| Package PyDSTool :: Package Generator :: Module InterpolateTable' | |  | | --- | | [hide private] | | [frames] | no frames] | |

# Source Code for Module PyDSTool.Generator.InterpolateTable'

```
  1  # Interpolated lookup table
 
  2  from __future__ import division 
  3  
 
  4  from allimports import * 
  5  from baseclasses import ctsGen, theGenSpecHelper 
  6  from PyDSTool.utils import * 
  7  from PyDSTool.common import * 
  8  
 
  9  # Other imports
 
 10  from numpy import Inf, NaN, isfinite, sometrue, alltrue, float64 
 11  import math, random 
 12  from copy import copy, deepcopy 
 13  
 
 14  # -----------------------------------------------------------------------------
 
 15  
 


16 -class InterpolateTable(ctsGen):


17      """Data lookup table with piecewise linear or piecewise constant interpolation.""" 
 18  
 


19 -    def __init__(self, kw):


20          try: 
 21              self.tdata = kw['tdata'] 
 22              self._xdatadict = {} 
 23              for k, v in dict(kw['ics']).iteritems(): 
 24                  self._xdatadict[str(k)] = v 
 25              self.foundKeys = 2 
 26              # check for other, invalid keys (but currently just ignored)
 
 27          except KeyError: 
 28              raise PyDSTool_KeyError('Keywords missing in argument') 
 29          self.tdomain = extent(self.tdata) 
 30          self.xdomain = {} 
 31          for x in self._xdatadict: 
 32              self.xdomain[x] = extent(self._xdatadict[x]) 
 33          ctsGen.__init__(self, kw) 
 34          self._needKeys.extend(['tdata', 'ics']) 
 35          self._optionalKeys.append('method') 
 36          self.funcspec = {}  # dict, not a FuncSpec instance 
 37          if 'vars' in kw: 
 38              raise PyDSTool_KeyError('vars option invalid for interpolated table class') 
 39          if 'auxvars' in kw: 
 40              raise PyDSTool_KeyError('auxvars option invalid for interpolated table class') 
 41          if 'tdomain' in kw: 
 42              raise PyDSTool_KeyError('tdomain option invalid for interpolated table class') 
 43          if 'xdomain' in kw: 
 44              raise PyDSTool_KeyError('xdomain option invalid for interpolated table class') 
 45          if 'pdomain' in kw: 
 46              raise PyDSTool_KeyError('pdomain option invalid for interpolated table class') 
 47          if 'ttype' in kw: 
 48              raise PyDSTool_KeyError('ttype option invalid for interpolated table class') 
 49          # hack to allow xtype to run
 
 50          kw['varspecs'] = {}.fromkeys(self._xdatadict, '') 
 51          self._kw_process_dispatch(['varspecs', 'xtype'], kw) 
 52          del kw['varspecs'] 
 53          self.foundKeys -= 1 
 54          if 'method' in kw: 
 55              if kw['method']=='linear': 
 56                  interp=interp1d 
 57              elif kw['method']=='constant': 
 58                  interp=interp0d 
 59              else: 
 60                  raise ValueError("Invalid interpolation method") 
 61              self.foundKeys += 1 
 62          else: 
 63              # default to piecewise linear interpolation
 
 64              interp=interp1d 
 65          self.indepvartype = float 
 66          for x in self._xdatadict: 
 67              self.funcspec[x] = Pointset({'coordarray': self._xdatadict[x],
 
 68                                           'coordtype': self.xtype[x],
 
 69                                           'indepvararray': self.tdata,
 
 70                                           'indepvartype': self.indepvartype,
 
 71                                           'indepvarname': 't',
 
 72                                           'coordnames': x}) 
 73          self.checkArgs(kw) 
 74          self.indepvariable = Variable(listid, Interval('t_domain',
 
 75                                                         self.indepvartype,
 
 76                                                self.tdomain, self._abseps),
 
 77                               Interval('t', self.indepvartype,
 
 78                                        extent(self.tdata),
 
 79                                        self._abseps), 't') 
 80          self._register(self.indepvariable) 
 81          for x in self._xdatadict: 
 82              self.variables[x] = Variable(interp(copy(self.tdata),
 
 83                                            self.funcspec[x].toarray()), 't',
 
 84                                    Interval(x, self.xtype[x], self.xdomain[x],
 
 85                                             self._abseps), x) 
 86          self._register(self.variables) 
 87          self.dimension = len(self._xdatadict) 
 88          self.validateSpec() 
 89          self.defined = True

 90  
 
 91  
 


92 -    def compute(self, trajname):


93          return Trajectory(trajname, [copy(v) for v in self.variables.values()],
 
 94                            abseps=self._abseps, globalt0=self.globalt0,
 
 95                            checklevel=self.checklevel,
 
 96                            FScompatibleNames=self._FScompatibleNames,
 
 97                            FScompatibleNamesInv=self._FScompatibleNamesInv,
 
 98                            modelNames=self.name,
 
 99                            modelEventStructs=self.eventstruct)

100  
 
101  
 


102 -    def set(self, **kw):


103          if 'abseps' in kw: 
104              # pass up to generic treatment for this
 
105              ctsGen.set(self, abseps=kw['abseps']) 
106              for x in self._xdatadict: 
107                  self.variables[x] = Variable(interp(copy(self.tdata),
 
108                                            self.funcspec[x].toarray()), 't',
 
109                                    Interval(x, self.xtype[x], self.xdomain[x],
 
110                                             self._abseps), x) 
111          if 'checklevel' in kw: 
112              # pass up to generic treatment for this
 
113              ctsGen.set(self, checklevel=kw['checklevel']) 
114          if 'globalt0' in kw: 
115              # pass up to generic treatment for this
 
116              ctsGen.set(self, globalt0=kw['globalt0'])

117  
 


118 -    def validateSpec(self):


119          ctsGen.validateSpec(self) 
120          try: 
121              assert isoutputcts(self.indepvariable) 
122              for v in self.variables.values(): 
123                  assert isinstance(v, Variable) 
124              assert not self.inputs 
125          except AssertionError: 
126              print 'Invalid system specification' 
127              raise

128  
 
129  
 


130 -    def __del__(self):


131          ctsGen.__del__(self)

132  
 
133  
 
134  
 
135  
 
136  # Register this Generator with the database
 
137  
 
138  symbolMapDict = {} 
139  # in future, provide appropriate mappings for libraries math,
 
140  # random, etc. (for now it's left to FuncSpec)
 
141  theGenSpecHelper.add(InterpolateTable, symbolMapDict, 'python') 
142
```

  


| Home | Trees | Indices | Help | | PyDSTool | | --- | |
| --- | --- | --- | --- | --- | --- |

|  |  |
| --- | --- |
| Generated by Epydoc 3.0.1 on Fri May 4 15:24:11 2012 | http://epydoc.sourceforge.net |
