# Supplementary material for: Hybrid Models and Biological Model Reduction with PyDSTool
Source: PLoS Comput Biol. 2012 Aug 9;8(8):e1002628. doi: 10.1371/journal.pcbi.1002628 (PMC3415397; doi:10.1371/journal.pcbi.1002628)
Supplement: Text S4 — Complete source code for the PyDSTool package (version 0.88.120504). Includes API documentation and help files linking to web pages. This file is identical to the current public release on Sourceforge.net. (ZIP) [file pcbi.1002628.s004.zip › PyDSTool/html/PyDSTool.Generator.LookupTable'-pysrc.html]

xml version="1.0" encoding="ascii"?


PyDSTool.Generator.LookupTable'


| Home | Trees | Indices | Help | | PyDSTool | | --- | |
| --- | --- | --- | --- | --- | --- |

|  |  |  |  |
| --- | --- | --- | --- |
| Package PyDSTool :: Package Generator :: Module LookupTable' | |  | | --- | | [hide private] | | [frames] | no frames] | |

# Source Code for Module PyDSTool.Generator.LookupTable'

```
  1  # Lookup table
 
  2  from __future__ import division 
  3  
 
  4  from allimports import * 
  5  from baseclasses import discGen, theGenSpecHelper 
  6  from PyDSTool.utils import * 
  7  from PyDSTool.common import * 
  8  
 
  9  # Other imports
 
 10  from numpy import Inf, NaN, isfinite, sometrue, alltrue 
 11  import math, random 
 12  from copy import copy, deepcopy 
 13  
 
 14  # -----------------------------------------------------------------------------
 
 15  
 


16 -class LookupTable(discGen):


17      """Lookup table trajectory with no interpolation.
 
 18  
 
 19      Independent and dependent variables may be integers or floats.""" 
 20  
 


21 -    def __init__(self, kw):


22          try: 
 23              self.tdata = kw['tdata'] 
 24              self._xdatadict = {} 
 25              for k, v in dict(kw['ics']).iteritems(): 
 26                  self._xdatadict[str(k)] = v 
 27              self.foundKeys = 2 
 28              # check for other, invalid keys (but currently just ignored)
 
 29          except KeyError: 
 30              raise PyDSTool_KeyError('Invalid keyword passed') 
 31          self.tdomain = self.tdata 
 32          discGen.__init__(self, kw) 
 33          self._needKeys.extend(['tdata', 'ics']) 
 34          # return values unused (side effects only)
 
 35          # hack to allow xtype to run
 
 36          kw['varspecs'] = {}.fromkeys(self._xdatadict, '') 
 37          self._kw_process_dispatch(['varspecs', 'ttype', 'xtype'], kw) 
 38          del kw['varspecs'] 
 39          self.foundKeys -= 1 
 40          self.funcspec = {} 
 41          if 'vars' in kw: 
 42              raise PyDSTool_KeyError("vars option invalid for lookup table "
 
 43                                      "class") 
 44          if 'auxvars' in kw: 
 45              raise PyDSTool_KeyError("auxvars option invalid for lookup table "
 
 46                                      "class") 
 47          for x in self._xdatadict: 
 48              self.funcspec[x] = Pointset({'coordarray': self._xdatadict[x],
 
 49                                           'coordtype': self.xtype[x],
 
 50                                           'indepvararray': self.tdata,
 
 51                                           'indepvartype': self.indepvartype,
 
 52                                           'indepvarname': 't',
 
 53                                           'coordnames': x}) 
 54  
 
 55          self.checkArgs(kw) 
 56          self.indepvariable = Variable(listid, {'t_domain': self.tdomain},
 
 57                               {'t': self.tdata}, 't') 
 58          self._register(self.indepvariable) 
 59          for x in self._xdatadict: 
 60              self.variables[x] = Variable(self.funcspec[x],
 
 61                                           {'t': copy(self.indepvariable.depdomain)},
 
 62                                           {x: self.funcspec[x].toarray()}, x) 
 63          self._register(self.variables) 
 64          self.dimension = len(self._xdatadict) 
 65          self.validateSpec() 
 66          self.defined = True

 67  
 
 68  
 


69 -    def compute(self, trajname):


70          if self.defined: 
 71              #self.validateSpec()
 
 72              self.diagnostics.clearWarnings() 
 73              self.diagnostics.clearErrors() 
 74          return Trajectory(trajname, [copy(v) for v in self.variables.values()],
 
 75                            abseps=self._abseps, globalt0=self.globalt0,
 
 76                            checklevel=self.checklevel,
 
 77                            FScompatibleNames=self._FScompatibleNames,
 
 78                            FScompatibleNamesInv=self._FScompatibleNamesInv,
 
 79                            modelNames=self.name,
 
 80                            modelEventStructs=self.eventstruct)

 81  
 


82 -    def validateSpec(self):


83          discGen.validateSpec(self) 
 84          try: 
 85              assert isoutputdiscrete(self.indepvariable) 
 86              for v in self.variables.values(): 
 87                  assert isinstance(v, Variable) 
 88              assert not self.inputs 
 89          except AssertionError: 
 90              print 'Invalid system specification' 
 91              raise

 92  
 
 93  
 


94 -    def __del__(self):


95          discGen.__del__(self)

 96  
 
 97  
 
 98  
 
 99  
 
100  # Register this Generator with the database
 
101  
 
102  symbolMapDict = {} 
103  # in future, provide appropriate mappings for libraries math,
 
104  # random, etc. (for now it's left to FuncSpec)
 
105  theGenSpecHelper.add(LookupTable, symbolMapDict, 'python') 
106
```

  


| Home | Trees | Indices | Help | | PyDSTool | | --- | |
| --- | --- | --- | --- | --- | --- |

|  |  |
| --- | --- |
| Generated by Epydoc 3.0.1 on Fri May 4 15:24:24 2012 | http://epydoc.sourceforge.net |
