# Supplementary material for: Hybrid Models and Biological Model Reduction with PyDSTool
Source: PLoS Comput Biol. 2012 Aug 9;8(8):e1002628. doi: 10.1371/journal.pcbi.1002628 (PMC3415397; doi:10.1371/journal.pcbi.1002628)
Supplement: Text S4 — Complete source code for the PyDSTool package (version 0.88.120504). Includes API documentation and help files linking to web pages. This file is identical to the current public release on Sourceforge.net. (ZIP) [file pcbi.1002628.s004.zip › PyDSTool/html/PyDSTool.Generator.MapSystem'-pysrc.html]

xml version="1.0" encoding="ascii"?


PyDSTool.Generator.MapSystem'


| Home | Trees | Indices | Help | | PyDSTool | | --- | |
| --- | --- | --- | --- | --- | --- |

|  |  |  |  |
| --- | --- | --- | --- |
| Package PyDSTool :: Package Generator :: Module MapSystem' | |  | | --- | | [hide private] | | [frames] | no frames] | |

# Source Code for Module PyDSTool.Generator.MapSystem'

```
  1  # MapSystem
 
  2  # For the MapSystem class, maps have an explicit "time" step, generating
 
  3  #  values for x at a specifc "time". Purely abstract time (i.e., iteration
 
  4  #  steps) is represented using integers. (We could make LookupTable a
 
  5  #  0-param sequence of maps with explicit time range?)
 
  6  from __future__ import division 
  7  
 
  8  from allimports import * 
  9  from baseclasses import Generator, discGen, theGenSpecHelper, \
 
 10       auxfn_container, _pollInputs 
 11  from PyDSTool.utils import * 
 12  from PyDSTool.common import * 
 13  from PyDSTool.Variable import Variable 
 14  from PyDSTool.Trajectory import Trajectory 
 15  from PyDSTool.Points import Pointset 
 16  from PyDSTool.Interval import uncertain 
 17  
 
 18  # Other imports
 
 19  from numpy import Inf, NaN, isfinite, sometrue, alltrue, array, transpose, \
 
 20       concatenate 
 21  import math, random, types 
 22  from copy import copy, deepcopy 
 23  try: 
 24      import psyco 
 25      HAVE_PSYCO = True 
 26  except ImportError: 
 27      HAVE_PSYCO = False 
 28  
 
 29  
 


30 -class MapSystem(discGen):


31      """Discrete dynamical systems, as maps (difference equations).
 
 32  
 
 33      """ 
 34      _validKeys = ['globalt0', 'xdomain', 'tdata', 'tdomain', 'checklevel',
 
 35                       'ics', 'pars', 'inputs', 'pdomain', 'abseps'] 
 36      _needKeys = discGen._needKeys + ['varspecs'] 
 37      _optionalKeys = discGen._optionalKeys + ['tdomain', 'xdomain', 'inputs', 'tdata',
 
 38                            'ics', 'events', 'system', 'ignorespecial',
 
 39                            'auxvars', 'vars', 'fnspecs', 'ttype', 'xtype',
 
 40                            'tstep', 'checklevel', 'pars', 'pdomain',
 
 41                            'vfcodeinsert_start', 'vfcodeinsert_end',
 
 42                            'enforcebounds', 'activatedbounds', 'reuseterms'] 
 43  
 


44 -    def __init__(self, kw):


45          discGen.__init__(self, kw) 
 46          self.diagnostics._errmessages[E_COMPUTFAIL] = 'Computation of trajectory failed' 
 47          # user auxiliary function interface
 
 48          self.auxfns = auxfn_container(self) 
 49          dispatch_list = ['varspecs', 'tdomain', 'ttype', 'tdata', 'tstep',
 
 50                            'inputs', 'ics', 'allvars', 'xtype', 'pars',
 
 51                            'xdomain', 'reuseterms', 'algparams', 'pdomain',
 
 52                            'system', 'fnspecs', 'vfcodeinserts', 'ignorespecial'] 
 53          # process keys and build func spec
 
 54          self.funcspec = RHSfuncSpec(self._kw_process_dispatch(dispatch_list,
 
 55                                                                kw)) 
 56          self._kw_process_events(kw) 
 57          self.checkArgs(kw) 
 58          tindepdomain = Interval('t_domain', self.indepvartype, self.tdomain,
 
 59                                  self._abseps) 
 60          tdepdomain = Interval('t', self.indepvartype, self.tdata, self._abseps) 
 61          self.indepvariable = Variable(listid, tindepdomain, tdepdomain, 't') 
 62          self._register(self.indepvariable) 
 63          for xname in self.funcspec.vars + self.funcspec.auxvars: 
 64              # Add a temporary dependent variable domain, for validation testing
 
 65              # during integration
 
 66              self.variables[xname] = Variable(indepdomain=tdepdomain,
 
 67                                           depdomain=Interval(xname,
 
 68                                                            self.xtype[xname],
 
 69                                                            self.xdomain[xname],
 
 70                                                            self._abseps)) 
 71          self._register(self.variables) 
 72          self._generate_ixmaps() 
 73          # Introduce any python-specified code to the local namespace
 
 74          self.addMethods() 
 75          # all registration completed
 
 76          self.validateSpec()

 77  
 
 78  
 


79 -    def addMethods(self):


80          # Add the auxiliary function specs to this Generator's namespace
 
 81          for auxfnname in self.funcspec._pyauxfns: 
 82              fninfo = self.funcspec._pyauxfns[auxfnname] 
 83              if not hasattr(self, fninfo[1]): 
 84                  # user-defined auxiliary functions
 
 85                  # (built-ins are provided explicitly)
 
 86                  if self._solver: 
 87                      fnstr = fninfo[0].replace(self._solver.name, 'ds._solver') 
 88  ##                    self._funcreg[self._solver.name] = self._solver
 
 89                  else: 
 90                      fnstr = fninfo[0] 
 91                  try: 
 92                      exec fnstr 
 93                  except: 
 94                      print 'Error in supplied auxiliary function code' 
 95                  self._funcreg[fninfo[1]] = ('self', fnstr) 
 96                  setattr(self, fninfo[1], types.MethodType(locals()[fninfo[1]],
 
 97                                                             self,
 
 98                                                             self.__class__)) 
 99                  # user auxiliary function interface wrapper
 
100                  try: 
101                      uafi_code = self.funcspec._user_auxfn_interface[auxfnname] 
102                      try: 
103                          exec uafi_code 
104                      except: 
105                          print 'Error in auxiliary function wrapper' 
106                          raise 
107                      setattr(self.auxfns, auxfnname,
 
108                              types.MethodType(locals()[auxfnname], self.auxfns,
 
109                                           auxfn_container)) 
110                      self._funcreg[auxfnname] = ('', uafi_code) 
111                  except KeyError: 
112                      # not a user-defined aux fn
 
113                      pass 
114              # bind all the auxfns here
 
115              if HAVE_PSYCO: 
116                  psyco.bind(getattr(self, fninfo[1])) 
117                  try: 
118                      psyco.bind(self.auxfns[auxfnname]) 
119                  except KeyError: 
120                      # not a user-defined aux fn
 
121                      pass 
122          if self.funcspec.targetlang == 'python': 
123              # Add the spec function to this Generator's namespace
 
124              fninfo = self.funcspec.spec 
125              if self._solver: 
126                  fnstr = fninfo[0].replace(self._solver.name, 'ds._solver') 
127              else: 
128                  fnstr = fninfo[0] 
129              try: 
130                  exec fnstr 
131              except: 
132                  print 'Error in supplied functional specification code' 
133                  raise 
134              self._funcreg[fninfo[1]] = ('self', fnstr) 
135              setattr(self, fninfo[1], types.MethodType(locals()[fninfo[1]],
 
136                                                                 self,
 
137                                                                 self.__class__)) 
138              if HAVE_PSYCO and not self._solver: 
139                  psyco.bind(getattr(self, fninfo[1])) 
140              # Add the auxiliary spec function (if present) to this
 
141              # Generator's namespace
 
142              if self.funcspec.auxspec != '': 
143                  fninfo = self.funcspec.auxspec 
144                  if self._solver: 
145                      fnstr = fninfo[0].replace(self._solver.name, 'ds._solver') 
146                  else: 
147                      fnstr = fninfo[0] 
148                  try: 
149                      exec fnstr 
150                  except: 
151                      print 'Error in supplied auxiliary variable code' 
152                      raise 
153                  self._funcreg[fninfo[1]] = ('self', fnstr) 
154                  setattr(self, fninfo[1], types.MethodType(locals()[fninfo[1]],
 
155                                                                 self,
 
156                                                                 self.__class__)) 
157                  if HAVE_PSYCO and not self._solver: 
158                      psyco.bind(getattr(self, fninfo[1]))

159  
 
160  
 
161      # Method for pickling protocol (setstate same as default)
 


162 -    def __getstate__(self):


163          d = copy(self.__dict__) 
164          for fname, finfo in self._funcreg.iteritems(): 
165              try: 
166                  del d[fname] 
167              except KeyError: 
168                  pass 
169          # delete user auxiliary function interface
 
170          try: 
171              del d['auxfns'] 
172          except KeyError: 
173              pass 
174          return d

175  
 
176  
 


177 -    def __setstate__(self, state):


178          self.__dict__.update(state) 
179          if self._funcreg != {}: 
180              self.auxfns = auxfn_container(self) 
181              self.addMethods()

182  
 
183  
 


184 -    def haveJacobian_pars(self):


185          """Report whether generator has an explicit user-specified Jacobian
 
186          with respect to pars associated with it.""" 
187          return 'Jacobian_pars' in self.funcspec.auxfns

188  
 
189  
 


190 -    def haveJacobian(self):


191          """Report whether map system has an explicit user-specified Jacobian
 
192          associated with it.""" 
193          return 'Jacobian' in self.funcspec.auxfns

194  
 
195  
 


196 -    def checkInitialConditions(self, checkauxvars=False):


197          for xname, val in self.initialconditions.iteritems(): 
198              if xname not in self.funcspec.vars and not checkauxvars: 
199                  # auxvars do not need initial conditions unless
 
200                  # explicitly requested (e.g. for user call to RHS
 
201                  # function of C code vector field before trajectory
 
202                  # has been computed)
 
203                  continue 
204              try: 
205                  if not isfinite(val): 
206                      raise ValueError("Initial condition for "+xname+" has been "
 
207                                      "incorrectly initialized") 
208              except TypeError: 
209                  print "Found: ", val 
210                  print "of type: ", type(val) 
211                  raise TypeError("Invalid type for %s`s initial"%xname \
 
212                                  + "condition value") 
213              if not self.contains(self.variables[xname].depdomain,
 
214                                   val, self.checklevel): 
215                  print "Bounds: ", self.variables[xname].depdomain.get() 
216                  print "Variable value: ", val 
217                  raise ValueError("Initial condition for "+xname+" has been "
 
218                                     "set outside of prescribed bounds")

219  
 
220  
 


221 -    def set(self, **kw):


222          """Set map system parameters""" 
223          if remain(kw.keys(), self._validKeys) != []: 
224              raise KeyError("Invalid keys in argument") 
225          if 'globalt0' in kw: 
226              # pass up to generic treatment for this
 
227              discGen.set(self, globalt0=kw['globalt0']) 
228          if 'checklevel' in kw: 
229              # pass up to generic treatment for this
 
230              discGen.set(self, checklevel=kw['checklevel']) 
231          if 'abseps' in kw: 
232              # pass up to generic treatment for this
 
233              discGen.set(self, abseps=kw['abseps']) 
234          # optional keys for this call are ['pars', 'tdomain', 'ics',
 
235          #   'algparams', 'tdata', 'xdomain', 'inputs', 'pdomain']
 
236          if 'ics' in kw: 
237              for k_temp, v in kw['ics'].iteritems(): 
238                  k = self._FScompatibleNames(k_temp) 
239                  if k in self.funcspec.vars+self.funcspec.auxvars: 
240                      self._xdatadict[k] = ensurefloat(v) 
241                  else: 
242                      raise ValueError('Illegal variable name, %s'%k) 
243              self.initialconditions.update(self._xdatadict) 
244          tchange = False 
245          if 'tdata' in kw: 
246              self.tdata = kw['tdata'] 
247              tchange = True 
248          if 'tdomain' in kw: 
249              self.tdomain = kw['tdomain'] 
250              self.indepvariable.indepdomain.set(self.tdomain) 
251              tchange = True 
252          if tchange: 
253              if self.tdomain[0] > self.tdata[0]: 
254                  if self.indepvariable.indepdomain.contains(self.tdata[0]) == uncertain: 
255                      self.diagnostics.warnings.append((W_UNCERTVAL,
 
256                                                        (self.tdata[0],self.tdomain))) 
257                  else: 
258                      print 'tdata cannot be specified below smallest '\
 
259                            'value in tdomain\n (possibly due to uncertain bounding).'\
 
260                            ' It has been automatically adjusted from\n ', \
 
261                            self.tdata[0], 'to', self.tdomain[0], '(difference of', \
 
262                            self.tdomain[0]-self.tdata[0], ')' 
263                  self.tdata[0] = self.tdomain[0] 
264              if self.tdomain[1] < self.tdata[1]: 
265                  if self.indepvariable.indepdomain.contains(self.tdata[1]) == uncertain: 
266                      self.diagnostics.warnings.append((W_UNCERTVAL,
 
267                                                        (self.tdata[1],self.tdomain))) 
268                  else: 
269                      print 'tdata cannot be specified above largest '\
 
270                            'value in tdomain\n (possibly due to uncertain bounding).'\
 
271                            ' It has been automatically adjusted from\n ', \
 
272                            self.tdomain[1], 'to', \
 
273                            self.tdomain[1], '(difference of', \
 
274                            self.tdata[1]-self.tdomain[1], ')' 
275                  self.tdata[1] = self.tdomain[1] 
276              self.indepvariable.depdomain.set(self.tdata) 
277          if 'xdomain' in kw: 
278              for k_temp, v in kw['xdomain'].iteritems(): 
279                  k = self._FScompatibleNames(k_temp) 
280                  if k in self.funcspec.vars+self.funcspec.auxvars: 
281                      if isinstance(v, _seq_types): 
282                          assert len(v) == 2, \
 
283                                 "Invalid size of domain specification for "+k 
284                          if v[0] >= v[1]: 
285                              raise PyDSTool_ValueError('xdomain values must be'
 
286                                                        'in order of increasing '
 
287                                                        'size') 
288                          else: 
289                              self.xdomain[k] = copy(v) 
290                      elif isinstance(v, _num_types): 
291                          self.xdomain[k] = [v, v] 
292                      else: 
293                          raise PyDSTool_TypeError('Invalid type for xdomain spec'
 
294                                                   ' '+k) 
295                      self.xdomain[k] = v 
296                  else: 
297                      raise ValueError('Illegal variable name') 
298                  try: 
299                      self.variables[k].depdomain.set(v) 
300                  except TypeError: 
301                      raise TypeError('xdomain must be a dictionary of variable'
 
302                                        ' names -> valid interval 2-tuples or '
 
303                                        'singletons') 
304                  try: 
305                      evs = self.eventstruct.events.values() 
306                  except AttributeError: 
307                      evs = [] 
308                  for ev in evs: 
309                      ev.xdomain[k] = v 
310          if 'pdomain' in kw: 
311              for k_temp, v in kw['pdomain'].iteritems(): 
312                  k = self._FScompatibleNames(k_temp) 
313                  if k in self.funcspec.pars: 
314                      if isinstance(v, _seq_types): 
315                          assert len(v) == 2, \
 
316                                 "Invalid size of domain specification for "+k 
317                          if v[0] >= v[1]: 
318                              raise PyDSTool_ValueError('pdomain values must be'
 
319                                                        'in order of increasing '
 
320                                                        'size') 
321                          else: 
322                              self.pdomain[k] = copy(v) 
323                      elif isinstance(v, _num_types): 
324                          self.pdomain[k] = [v, v] 
325                      else: 
326                          raise PyDSTool_TypeError('Invalid type for pdomain spec'
 
327                                                   ' '+k) 
328                  else: 
329                      raise ValueError('Illegal parameter name') 
330                  try: 
331                      self.parameterDomains[k].depdomain.set(v) 
332                  except TypeError: 
333                      raise TypeError('xdomain must be a dictionary of parameter'
 
334                                        ' names -> valid interval 2-tuples or '
 
335                                        'singletons') 
336                  try: 
337                      evs = self.eventstruct.events.values() 
338                  except AttributeError: 
339                      evs = [] 
340                  for ev in evs: 
341                      ev.pdomain[k] = self.pdomain[k] 
342          if 'pars' in kw: 
343              assert self.numpars > 0, ('No pars were declared for this '
 
344                                        'model') 
345              for k_temp, v in kw['pars'].iteritems(): 
346                  k = self._FScompatibleNames(k_temp) 
347                  if k in self.pars: 
348                      cval = self.parameterDomains[k].contains(v) 
349                      if self.checklevel < 3: 
350                          if cval is not notcontained: 
351                              self.pars[k] = ensurefloat(v) 
352                              if cval is uncertain and self.checklevel == 2: 
353                                  print 'Warning: Parameter value at bound' 
354                          else: 
355                              raise PyDSTool_ValueError('Parameter value out of bounds') 
356                      else: 
357                          if cval is contained: 
358                              self.pars[k] = ensurefloat(v) 
359                          elif cval is uncertain: 
360                              raise PyDSTool_UncertainValueError('Parameter value at bound') 
361                          else: 
362                              raise PyDSTool_ValueError('Parameter value out of bounds') 
363                  else: 
364                      raise PyDSTool_ValueError('Illegal parameter name') 
365              # pass on parameter changes to embedded system, if present
 
366              if self._solver: 
367                  try: 
368                      shared_pars = intersect(kw['pars'].keys(), self._solver.pars) 
369                  except AttributeError: 
370                      # no pars for this kind of solver
 
371                      pass 
372                  else: 
373                      if shared_pars != []: 
374                          self._solver.set(pars=filteredDict(kw['pars'], shared_pars)) 
375          if 'inputs' in kw: 
376              assert self.inputs, ('Cannot provide inputs after '
 
377                                               'initialization without them') 
378              inputs = copy(kw['inputs']) 
379              _inputs = {} 
380              if isinstance(inputs, Trajectory): 
381                  # extract the variables
 
382                  _inputs = self._FScompatibleNames(inputs.variables) 
383              elif isinstance(inputs, Variable): 
384                  _inputs = {self._FScompatibleNames(inputs.name): inputs} 
385              elif isinstance(inputs, Pointset): 
386                  # turn into Variables
 
387                  for n in inputs.coordnames: 
388                      x_array = inputs[n] 
389                      nFS = self._FScompatibleNames(n) 
390                      _inputs[nFS] = \
 
391                             Variable(interp1d(inputs.indepvararray,
 
392                                                         x_array), 't',
 
393                                           Interval(nFS, float, extent(x_array),
 
394                                                    abseps=self._abseps),
 
395                                           name=n)  # keep original name here 
396              elif isinstance(inputs, dict): 
397                  _inputs = self._FScompatibleNames(inputs) 
398                  # ensure values are Variables
 
399                  for v in _inputs.values(): 
400                      if not isinstance(v, Variable): 
401                          raise TypeError("Invalid specification of inputs") 
402              else: 
403                  raise TypeError("Invalid specification of inputs") 
404              if _inputs: 
405                  for i in _inputs: 
406                      assert i in self.inputs, 'Incorrect input name provided' 
407                      self.inputs[i] = _inputs[i] 
408                  # re-calc inputs ixmaps
 
409                  self._generate_ixmaps('inputs') 
410              self._extInputsChanged = True 
411          if 'inputs_t0' in kw: 
412              assert self.inputs, ('Cannot provide inputs after '
 
413                                  'initialization without them') 
414              inputs_t0 = self._FScompatibleNames(kw['inputs_t0']) 
415              for iname, t0 in inputs_t0.items(): 
416                  self.inputs[iname]._internal_t_offset = t0 
417              self._extInputsChanged = True

418  
 
419  
 


420 -    def compute(self, trajname, ics=None):


421          assert self.funcspec.targetlang == 'python', \
 
422                 ('Wrong target language for functional specification. '
 
423                  'Python needed for this class') 
424          assert isinstance(self.funcspec, RHSfuncSpec), ('Map system '
 
425                                      'requires RHSfuncSpec type to proceed') 
426          self.diagnostics.clearWarnings() 
427          self.diagnostics.clearErrors() 
428          if ics is not None: 
429              self.set(ics=ics) 
430          xnames = self._var_ixmap  # ensures correct order 
431          # wrap up each dictionary initial value as a singleton list
 
432          alltData = [self.indepvariable.depdomain[0]] 
433          allxDataDict = dict(zip(xnames, map(listid,
 
434                                     sortedDictValues(self.initialconditions,
 
435                                                      self.funcspec.vars)))) 
436          rhsfn = getattr(self,self.funcspec.spec[1]) 
437          # Check i.c.'s are well defined (finite)
 
438          self.checkInitialConditions() 
439          self.setEventICs(self.initialconditions, self.globalt0) 
440          ic = sortedDictValues(self.initialconditions, self.funcspec.vars) 
441          plist = sortedDictValues(self.pars) 
442          extralist = copy(plist) 
443          ilist = [] 
444          if self.inputs: 
445              # inputVarList is a list of Variables
 
446              listend = self.numpars + len(self.inputs) 
447              inputVarList = sortedDictValues(self.inputs) 
448              try: 
449                  for f in inputVarList: 
450                      f.diagnostics.clearWarnings() 
451                      ilist.append(f(alltData[0], self.checklevel)) 
452              except AssertionError: 
453                  print 'External input call has t out of range: t = ', \
 
454                      self.indepvariable.depdomain[0] 
455                  print 'Maybe checklevel is 3 and initial time is not', \
 
456                              'completely inside valid time interval' 
457                  raise 
458              except ValueError: 
459                  print 'External input call has value out of range: t = ', \
 
460                        self.indepvariable.depdomain[0] 
461                  for f in inputVarList: 
462                      if f.diagnostics.hasWarnings(): 
463                          print 'External input %s out of range:' % f.name 
464                          print '   t = ', repr(f.diagnostics.warnings[-1][0]), ', ', \
 
465                                f.name, ' = ', repr(f.diagnostics.warnings[-1][1]) 
466                  raise 
467          else: 
468              listend = self.numpars 
469              inputVarList = [] 
470          extralist.extend(ilist) 
471          precevents = self.eventstruct.query(['precise']) 
472          if precevents != []: 
473              raise PyDSTool_ValueError('precise events are not valid for map systems') 
474          eventslist = self.eventstruct.query(['highlevel', 'active',
 
475                                               'notvarlinked']) 
476          termevents = self.eventstruct.query(['term'], eventslist) 
477                  # initialize event info dictionaries
 
478          Evtimes = {} 
479          Evpoints = {} 
480          for (evname, ev) in eventslist: 
481              Evtimes[evname] = [] 
482              Evpoints[evname] = [] 
483          if eventslist != []: 
484              if self._for_hybrid_DS: 
485                  # self._for_hybrid_DS is set internally by HybridModel class
 
486                  # to ensure not to reset events, because they may be about to
 
487                  # flag on first step if previous hybrid state was the same
 
488                  # generator and, for example, two variables are synchronizing
 
489                  # so that their events get very close together.
 
490                  # Just reset the starttimes of these events
 
491                  for evname, ev in eventslist: 
492                      ev.starttime = self.indepvariable.depdomain[0] 
493              else: 
494                  self.eventstruct.resetHighLevelEvents(self.indepvariable.depdomain[0],
 
495                                                    eventslist) 
496              self.eventstruct.validateEvents(self.funcspec.vars + \
 
497                                              self.funcspec.auxvars + \
 
498                                              ['t'], eventslist) 
499  
 
500          # per-iteration storage of variable data (initial values are irrelevant)
 
501          xDataDict = {} 
502          # storage of all auxiliary variable data
 
503          allaDataDict = {} 
504          anames = self.funcspec.auxvars 
505          avals = apply(getattr(self,self.funcspec.auxspec[1]),
 
506                        [self.indepvariable.depdomain[0],
 
507                         sortedDictValues(self.initialconditions,
 
508                                          self.funcspec.vars),
 
509                         extralist]) 
510          for aix in range(len(anames)): 
511              aname = anames[aix] 
512              allaDataDict[aname] = [avals[aix]] 
513          # temp storage of first time at which terminal events found
 
514          # (this is used for keeping the correct end point of new mesh)
 
515          first_found_t = None 
516          tmesh = self.indepvariable.depdomain.sample(self.tstep,
 
517                                          strict=False,
 
518                                          avoidendpoints=self.checklevel>2) 
519          # Main loop
 
520          breakwhile = False 
521          success = False 
522          x = ic 
523          notdone = True 
524          # did i=0 for initial condition already
 
525          i = 1 
526          while notdone: 
527              t = tmesh[i] 
528              ## COMPUTE NEXT STATE y from x
 
529              try: 
530                  y = rhsfn(t, x, extralist) 
531              except: 
532                  print "Error in calling right hand side function:" 
533                  self.showSpec() 
534                  raise 
535              for xi in xrange(self.dimension): 
536                  xDataDict[xnames[xi]] = y[xi] 
537                  if not self.contains(self.variables[xnames[xi]].depdomain,
 
538                                   y[xi], self.checklevel): 
539                      self.diagnostics.warnings.append((W_TERMSTATEBD,
 
540                                      (t, xnames[xi], y[xi],
 
541                                       self.variables[xnames[xi]].depdomain))) 
542                      breakwhile = True 
543                      break  # for loop 
544              if breakwhile: 
545                  notdone = False 
546                  continue 
547              avals = apply(getattr(self,self.funcspec.auxspec[1]), [t,
 
548                              sortedDictValues(xDataDict),
 
549                              extralist]) 
550              if eventslist != []: 
551                  dataDict = copy(xDataDict) 
552                  dataDict['t'] = t 
553                  evsflagged = self.eventstruct.pollHighLevelEvents(None,
 
554                                                              dataDict,
 
555                                                              self.pars,
 
556                                                              eventslist) 
557                  termevsflagged = filter(lambda e: e in evsflagged, termevents) 
558                  nontermevsflagged = filter(lambda e: e not in termevsflagged,
 
559                                             evsflagged) 
560                  # register any non-terminating events in the warnings list
 
561                  if len(nontermevsflagged) > 0: 
562                      evnames = [ev[0] for ev in nontermevsflagged] 
563                      self.diagnostics.warnings.append((W_NONTERMEVENT,
 
564                                   (t, evnames))) 
565                      for evname in evnames: 
566                          Evtimes[evname].append(t) 
567                          xv = y 
568                          av = array(avals) 
569                          Evpoints[evname].append(concatenate((xv, av))) 
570                  if termevsflagged != []: 
571                      # active terminal event flagged at this time point
 
572                      # register the event in the warnings
 
573                      evnames = [ev[0] for ev in termevsflagged] 
574                      self.diagnostics.warnings.append((W_TERMEVENT, \
 
575                                               (t, evnames))) 
576                      for evname in evnames: 
577                          Evtimes[evname].append(t) 
578                          xv = y 
579                          av = array(avals) 
580                          Evpoints[evname].append(concatenate((xv, av))) 
581                      notdone = False 
582                      # ?? if continue here then won't add the event point to the
 
583                      # trajectory values being constructed!
 
584                      #continue
 
585              alltData.append(t) 
586              for xi in range(self.dimension): 
587                  allxDataDict[xnames[xi]].append(y[xi]) 
588              for aix in range(len(anames)): 
589                  aname = anames[aix] 
590                  allaDataDict[aname].append(avals[aix]) 
591              try: 
592                  extralist[self.numpars:listend] = [apply(f,
 
593                                                  [t, self.checklevel]) \
 
594                                                for f in inputVarList] 
595              except ValueError: 
596                  print 'External input call caused value out of range error:', \
 
597                        't = ', t 
598                  for f in inputVarList: 
599                      if f.hasWarnings(): 
600                          print 'External input variable %s out of range:' % f.name 
601                          print '   t = ', repr(f.diagnostics.warnings[-1][0]), ', ', \
 
602                                f.name, ' = ', repr(f.diagnostics.warnings[-1][1]) 
603                  raise 
604              except AssertionError: 
605                  print 'External input call caused t out of range error: t = ', t 
606                  raise 
607              if i >= len(tmesh) - 1: 
608                  notdone = False 
609              else: 
610                  i += 1 
611                  x = y 
612          # update success flag
 
613          success = not notdone 
614          # Check that any terminal events found terminated the code correctly
 
615          if first_found_t is not None: 
616              assert self.diagnostics.warnings[-1][0] == W_TERMEVENT, ("Event finding code "
 
617                                          "for terminal event failed") 
618          # Package up computed trajectory in Variable variables
 
619          # Add external inputs warnings to self.dignostics.warnings, if any
 
620          for f in inputVarList: 
621              for winfo in f.diagnostics.warnings: 
622                  self.diagnostics.warnings.append((W_NONTERMSTATEBD,
 
623                                       (winfo[0], f.name, winfo[1],
 
624                                        f.depdomain))) 
625          # check for non-unique terminal event
 
626          termcount = 0 
627          for (w,i) in self.diagnostics.warnings: 
628              if w == W_TERMEVENT or w == W_TERMSTATEBD: 
629                  termcount += 1 
630                  if termcount > 1: 
631                      self.diagnostics.errors.append((E_NONUNIQUETERM,
 
632                                                      (alltData[-1], i[1]))) 
633  ##                print 'Time interval adjusted according to %s: %s' % \
 
634  ##                      (self._warnmessages[w], str(i[0])+", "+ str(i[1]))
 
635          # Create variables (self.variables contains no actual data)
 
636          variables = copyVarDict(self.variables) 
637          # build event pointset information (reset previous trajectory's)
 
638          self.trajevents = {} 
639          for (evname,  ev) in eventslist: 
640              evpt = Evpoints[evname] 
641              if evpt == []: 
642                  self.trajevents[evname] = None 
643              else: 
644                  evpt = transpose(array(evpt)) 
645                  self.trajevents[evname] = Pointset({'coordnames': xnames+anames,
 
646                                  'indepvarname': 't',
 
647                                  'coordarray': evpt,
 
648                                  'indepvararray': Evtimes[evname],
 
649                                  'indepvartype': self.variables[xnames[0]].indepvartype}) 
650          for x in xnames: 
651              if len(alltData) > 1: 
652                  variables[x] = Variable(Pointset({'coordnames': [x],
 
653                                 'coordarray': allxDataDict[x],
 
654                                 'coordtype': self.variables[x].coordtype,
 
655                                 'indepvarname': 't',
 
656                                 'indepvararray': alltData,
 
657                                 'indepvartype': self.variables[x].indepvartype}), 't', x, x) 
658              else: 
659                  raise PyDSTool_ValueError("Fewer than 2 data points computed") 
660          for a in anames: 
661              if len(alltData) > 1: 
662                  variables[a] = Variable(Pointset({'coordnames': [a],
 
663                                 'coordarray': allaDataDict[a],
 
664                                 'coordtype': self.variables[a].coordtype,
 
665                                 'indepvarname': 't',
 
666                                 'indepvararray': alltData,
 
667                                 'indepvartype': self.variables[a].indepvartype}), 't', a, a) 
668              else: 
669                  raise PyDSTool_ValueError("Fewer than 2 data points computed") 
670  
 
671          if success: 
672              #self.validateSpec()
 
673              self.defined = True 
674              return Trajectory(trajname, variables.values(),
 
675                                abseps=self._abseps, globalt0=self.globalt0,
 
676                                checklevel=self.checklevel,
 
677                                FScompatibleNames=self._FScompatibleNames,
 
678                                FScompatibleNamesInv=self._FScompatibleNamesInv,
 
679                                events=self.trajevents,
 
680                                modelNames=self.name,
 
681                                modelEventStructs=self.eventstruct) 
682          else: 
683              print 'Trajectory computation failed' 
684              self.diagnostics.errors.append((E_COMPUTFAIL,
 
685                                              (t, self._errorcodes[errcode]))) 
686              self.defined = False

687  
 


688 -    def Rhs(self, t, xdict, pdict=None, asarray=True):


689          """asarray is an unused, dummy argument for compatibility with Model.Rhs""" 
690          # don't need to convert names to FS-compatible as they sort
 
691          # the same
 
692          x = sortedDictValues(filteredDict(xdict, self.funcspec.vars)) 
693          if pdict is None: 
694              pdict = self.pars 
695          p = sortedDictValues(pdict) 
696          i = _pollInputs(sortedDictValues(self.inputs), t, self.checklevel) 
697          return apply(getattr(self,self.funcspec.spec[1]), [t, x, p+i])

698  
 
699  
 


700 -    def Jacobian(self, t, xdict, pdict=None, asarray=True):


701          """asarray is an unused, dummy argument for compatibility with
 
702          Model.Jacobian""" 
703          if self.haveJacobian(): 
704              x = sortedDictValues(filteredDict(xdict, self.funcspec.vars)) 
705              if pdict is None: 
706                  pdict = self.pars 
707              p = sortedDictValues(pdict) 
708              i = _pollInputs(sortedDictValues(self.inputs), t, self.checklevel) 
709              return apply(getattr(self,self.funcspec.auxfns["Jacobian"][1]), \
 
710                           [t, x, p+i]) 
711          else: 
712              raise PyDSTool_ExistError("Jacobian not defined")

713  
 
714  
 


715 -    def JacobianP(self, t, xdict, pdict=None, asarray=True):


716          """asarray is an unused, dummy argument for compatibility with
 
717          Model.JacobianP""" 
718          if self.haveJacobian_pars(): 
719              x = sortedDictValues(filteredDict(xdict, self.funcspec.vars)) 
720              if pdict is None: 
721                  pdict = self.pars 
722              p = sortedDictValues(pdict) 
723              i = _pollInputs(sortedDictValues(self.inputs), t, self.checklevel) 
724              return apply(getattr(self,self.funcspec.auxfns["Jacobian_pars"][1]), \
 
725                          [t, x, p+i]) 
726          else: 
727              raise PyDSTool_ExistError("Jacobian w.r.t. parameters not defined")

728  
 
729  
 


730 -    def AuxVars(self, t, xdict, pdict=None, asarray=True):


731          """asarray is an unused, dummy argument for compatibility with
 
732          Model.AuxVars""" 
733          x = sortedDictValues(filteredDict(xdict, self.funcspec.vars)) 
734          if pdict is None: 
735              pdict = self.pars 
736          p = sortedDictValues(pdict) 
737          i = _pollInputs(sortedDictValues(self.inputs), t, self.checklevel) 
738          return apply(getattr(self,self.funcspec.auxspec[1]), [t, x, p+i])

739  
 
740  
 


741 -    def __del__(self):


742          discGen.__del__(self)

743  
 
744  
 
745  
 
746  # Register this Generator with the database
 
747  
 
748  symbolMapDict = {} 
749  # in future, provide appropriate mappings for libraries math,
 
750  # random, etc. (for now it's left to FuncSpec)
 
751  theGenSpecHelper.add(MapSystem, symbolMapDict, 'python') 
752
```

  


| Home | Trees | Indices | Help | | PyDSTool | | --- | |
| --- | --- | --- | --- | --- | --- |

|  |  |
| --- | --- |
| Generated by Epydoc 3.0.1 on Fri May 4 15:24:16 2012 | http://epydoc.sourceforge.net |
