# Supplementary material for: Hybrid Models and Biological Model Reduction with PyDSTool
Source: PLoS Comput Biol. 2012 Aug 9;8(8):e1002628. doi: 10.1371/journal.pcbi.1002628 (PMC3415397; doi:10.1371/journal.pcbi.1002628)
Supplement: Text S4 — Complete source code for the PyDSTool package (version 0.88.120504). Includes API documentation and help files linking to web pages. This file is identical to the current public release on Sourceforge.net. (ZIP) [file pcbi.1002628.s004.zip › PyDSTool/html/PyDSTool.Generator.messagecodes-module.html]

xml version="1.0" encoding="ascii"?


PyDSTool.Generator.messagecodes


| Home | Trees | Indices | Help | | PyDSTool | | --- | |
| --- | --- | --- | --- | --- | --- |

|  |  |  |  |
| --- | --- | --- | --- |
| Package PyDSTool :: Package Generator :: Module messagecodes | |  | | --- | | [hide private] | | [frames] | no frames] | |

# Module messagecodes

source code

Message code definitions for Generators


|  |  |  |  |
| --- | --- | --- | --- |
| |  |  | | --- | --- | | Variables | [hide private] | | |
|  | W\_UNCERTVAL = `0` |
|  | W\_TERMEVENT = `10` |
|  | W\_TERMSTATEBD = `11` |
|  | W\_BISECTLIMIT = `12` |
|  | W\_NONTERMEVENT = `20` |
|  | W\_NONTERMSTATEBD = `21` |
|  | E\_COMPUTFAIL = `0` |
|  | E\_NONUNIQUETERM = `10` |
|  | errmessages = `{0: 'Computation of trajectory failed', 10: 'Mor...` |
|  | errorfields = `{0: ['t', 'error info'], 10: ['t', 'event list']}` |
|  | warnmessages = `{0: 'Uncertain value computed', 10: 'Terminal e...` |
|  | warnfields = `{0: ['value', 'interval'], 10: ['t', 'event list'...` |


|  |  |  |  |
| --- | --- | --- | --- |
| |  |  | | --- | --- | | Variables Details | [hide private] | | |

|  |  |
| --- | --- |
| errmessages   Value:  |  | | --- | | ``` {0: 'Computation of trajectory failed',  10: 'More than one terminal event found'} ``` | |

|  |  |
| --- | --- |
| warnmessages   Value:  |  | | --- | | ``` {0: 'Uncertain value computed',  10: 'Terminal event(s) found',  11: 'State variable reached bounds (terminal)',  12: 'Bisection limit reached for event',  20: 'Non-terminal event(s) found',  21: 'State or input variable reached bounds (non-terminal)'} ``` | |

|  |  |
| --- | --- |
| warnfields   Value:  |  | | --- | | ``` {0: ['value', 'interval'],  10: ['t', 'event list'],  11: ['t', 'var name', 'var value', '\n\tvalue interval'],  12: ['t', 'event list'],  20: ['t', 'event list'],  21: ['t', 'var name', 'var value', '\n\tvalue interval']} ``` | |

  


| Home | Trees | Indices | Help | | PyDSTool | | --- | |
| --- | --- | --- | --- | --- | --- |

|  |  |
| --- | --- |
| Generated by Epydoc 3.0.1 on Fri May 4 15:24:03 2012 | http://epydoc.sourceforge.net |
