# Supplementary material for: Hybrid Models and Biological Model Reduction with PyDSTool
Source: PLoS Comput Biol. 2012 Aug 9;8(8):e1002628. doi: 10.1371/journal.pcbi.1002628 (PMC3415397; doi:10.1371/journal.pcbi.1002628)
Supplement: Text S4 — Complete source code for the PyDSTool package (version 0.88.120504). Includes API documentation and help files linking to web pages. This file is identical to the current public release on Sourceforge.net. (ZIP) [file pcbi.1002628.s004.zip › PyDSTool/html/PyDSTool.Generator.messagecodes-pysrc.html]

xml version="1.0" encoding="ascii"?


PyDSTool.Generator.messagecodes


| Home | Trees | Indices | Help | | PyDSTool | | --- | |
| --- | --- | --- | --- | --- | --- |

|  |  |  |  |
| --- | --- | --- | --- |
| Package PyDSTool :: Package Generator :: Module messagecodes | |  | | --- | | [hide private] | | [frames] | no frames] | |

# Source Code for Module PyDSTool.Generator.messagecodes

```
 1  """Message code definitions for Generators
 
 2  """ 
 3  
 
 4  ## Warning message codes:
 
 5  # terminals must have 1 in tens place, non-terminals with 2
 
 6  # additional types from 00-09 (models make use of this format)
 
 7  W_UNCERTVAL = 00 
 8  W_TERMEVENT = 10 
 9  W_TERMSTATEBD = 11 
10  W_BISECTLIMIT = 12 
11  W_NONTERMEVENT = 20 
12  W_NONTERMSTATEBD = 21 
13  
 
14  ## Error message codes
 
15  # computation errors have 0 in tens place, event errors have 1 in tens place
 
16  E_COMPUTFAIL    = 00 
17  E_NONUNIQUETERM = 10 
18  
 
19  errmessages = {E_NONUNIQUETERM: 'More than one terminal event found',
 
20                 E_COMPUTFAIL: 'Computation of trajectory failed'} 
21  
 
22  errorfields = {E_NONUNIQUETERM: ['t', 'event list'],
 
23                 E_COMPUTFAIL: ['t', 'error info']} 
24  
 
25  warnmessages = {W_UNCERTVAL: 'Uncertain value computed',
 
26                W_TERMEVENT: 'Terminal event(s) found',
 
27                W_NONTERMEVENT: 'Non-terminal event(s) found',
 
28                W_TERMSTATEBD: 'State variable reached bounds (terminal)',
 
29                W_BISECTLIMIT: 'Bisection limit reached for event',
 
30                W_NONTERMSTATEBD: 'State or input variable reached ' + \
 
31                                'bounds (non-terminal)'} 
32  
 
33  warnfields = {W_UNCERTVAL: ['value', 'interval'],
 
34                  W_TERMEVENT: ['t', 'event list'],
 
35                  W_BISECTLIMIT: ['t', 'event list'],
 
36                  W_NONTERMEVENT: ['t', 'event list'],
 
37                  W_TERMSTATEBD: ['t', 'var name', 'var value',
 
38                                  '\n\tvalue interval'],
 
39                  W_NONTERMSTATEBD: ['t', 'var name', 'var value',
 
40                                     '\n\tvalue interval']} 
41
```

  


| Home | Trees | Indices | Help | | PyDSTool | | --- | |
| --- | --- | --- | --- | --- | --- |

|  |  |
| --- | --- |
| Generated by Epydoc 3.0.1 on Fri May 4 15:24:23 2012 | http://epydoc.sourceforge.net |
